# Apical Localization of RNA Polymerases Modulates Transcription Dynamics and Supercoiling Domains Revealed by Cryo-ET

**DOI:** 10.1016/j.molcel.2026.04.013

**Published:** 2026-05-08

**Authors:** Meng Zhang, Cristhian Cañari-Chumpitaz, Jianfang Liu, Bibiana Onoa, Sinead de Cleir, Enze Cheng, Katherinne I. Requejo, Carlos Bustamante

**Affiliations:** 1 California Institute for Quantitative Biosciences, University of California, Berkeley, USA; 2 The Molecular Foundry, Lawrence Berkeley National Laboratory, Berkeley, USA; 3 Department of Chemistry, University of California Berkeley, Berkeley, CA, USA; 4 Department of Biology, Stanford University, Stanford, CA USA; 5 Innovative Genomics Institute, University of California Berkeley, Berkeley, CA, USA; 6 Department of Molecular and Cell Biology, University of California, Berkeley, USA; 7 Howard Hughes Medical Institute, University of California, Berkeley, USA; 8 Department of Physics, University of California, Berkeley, USA; 9 Department of Chemistry, University of California, Berkeley, USA; 10 Molecular Biophysics and Integrative Bioimaging Division, Lawrence Berkeley National Laboratory, USA; 11 Kavli Energy Nanoscience Institute, University of California, Berkeley, USA; 12 Jason Choy laboratory of single molecule biophysics, University of California, Berkeley, USA

## Abstract

Protein interactions with canonical B-form DNA are well-characterized, yet the effect on these interactions of torsionally constrained DNA—ubiquitous in cells—remains underexplored. Using cryo-electron tomography (cryo-ET), we 3D-reconstructed entire negatively supercoiled DNA substrates bound to active RNA polymerase (RNAP), revealing diverse DNA supercoiling conformations and their interplay with transcription. RNAP preferentially localizes at plectoneme apices in a swiveled, pause-prone state. RNAP, along with other DNA-melting proteins like dCas9, can act as torsional roadblocks that segregate “twin-supercoiling domains” during active transcription, independent of external DNA/RNAP tethering. Co-transcribing RNAPs further intensify this domain separation: tandem oriented RNAPs relieve negative supercoiling more effectively than opposing ones, promote greater RNAP accumulation and enhanced elongation, both in vitro and in vivo. Topoisomerase I relieves torsional stress and facilitates RNAP escape from apical stalls, thereby supporting apical transcription regulation. Together, these findings support a load-and-release mechanism at plectoneme apices that may underlie supercoiling-dependent transcriptional bursting.

## eTOC Blurb

Using cryo-ET, Zhang et al. reveal how RNA polymerase interacts with supercoiled DNA in three dimensions. They show that apical localization shapes transcription dynamics and supercoiling domains, providing a structural basis for transcriptional bursting and coordination between multiple polymerases.

## Graphical Abstract

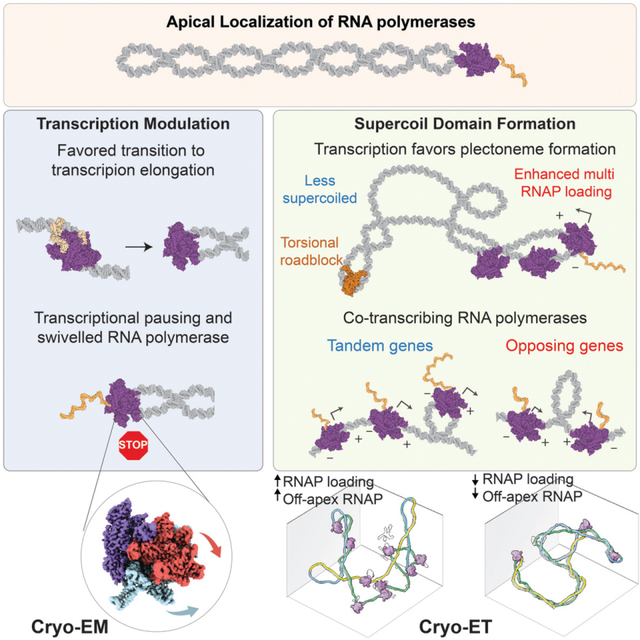

## INTRODUCTION

The influence of DNA supercoiling on both prokaryotic and eukaryotic DNA transactions^1^—including transcription, replication, and chromatin segregation,^2,3^ underscores the role of deviations from canonical B-form DNA in cellular metabolism. Prokaryotic genomes inherently accumulate negative torsional stress with an average supercoiling density of ~ −0.06. Accordingly, the interplay between DNA supercoiling and the activities of DNA-binding proteins, such as the collective transcriptional behavior of RNA polymerases (RNAPs) on supercoiled templates and torsional regulation by DNA gyrase and topoisomerase, remain an area of active research.^4–8^

The need to visualize DNA supercoiling dynamics has spurred the development of diverse biophysical approaches. In vitro single-molecule fluorescence microscopy enables real-time tracking of DNA plectoneme formation.^9^ Atomic Force Microscopy (AFM) allowed the detection of kinks in supercoiled DNA.^10^ However, these approaches typically require partial confinement or surface immobilization of DNA. Cryo-electron tomography (cryo-ET) has revisited this topic using small minicircles in a more native state,^11–13^ but the ~300 bp minicircles were not of sufficient length to capture the complexity of the plectoneme structures.^14^ Structural studies of transcription have largely focused on RNA polymerase (RNAP) states and cofactor-mediated regulation on linear templates,^15–19^ often overlooking template supercoiling. The “twin-supercoiling domain model”,^20^ that describes overwinding (positive supercoils) ahead of the RNAP and transcription-induced DNA unwinding (negative supercoils) at its wake, still lacks detailed 3D structural characterization despite strong biochemical support.^21–24^ Consequently, questions persist such as: What are the structures naturally adopted by supercoiled DNA in 3D? What is the spatial relationship between a transcribing RNAP and its supercoiled substrate? And how do supercoiling and transcription mutually affect each other?

In this cryo-ET study, we directly visualized individual plasmids (~2 kbp) in their native negatively supercoiled state, allowing precise quantification of 3D conformational dynamics. Using this plasmid, we stalled and initiated *E.coli* RNAP to systematically probe the interplay between supercoiling and transcription. We determined the orientation of RNAPs on their templates and discovered that their preferred apical binding on negatively supercoiled plectonemes persists during active transcription. RNAPs were found swiveled in this apical configuration, functionally facilitating initiation but hindering elongation—a previously unrecognized mode. Interestingly, we discover that this apical binding also applies to dCas9, turning it into a “soft” torsional barrier that hinders free DNA rotation. The simultaneous apical binding of dCas9 and RNAP on opposite apices of a plasmid leads to the structural visualization of supercoiling-organized transcription domains, inducing non-plectonemic DNA loops and promoting multi-RNAP slow co-transcriptional events. This apical-torsional regulatory mechanism provides a direct structural basis for understanding and experimentally probing how co-transcribing RNAPs impose mutual torsional constraints under tandem and opposing gene contexts, yielding contrasting in vivo transcriptional outcomes. Additionally, introducing *E.coli* Topoisomerase I (TopI) in the presence of RNAP only partially releases the torsion within the plasmid, which, however, is sufficient to disrupt RNAP's apical positioning and increase transcription elongation activity. Therefore, we propose an 'on-off' switch model of apical constraint that plays a critical role in linking torsional stress to transcription regulation.

## RESULTS

### Cryo-ET per-particle analysis enables full 3D DNA tracing of negatively supercoiled plasmids

**Figure 1:**
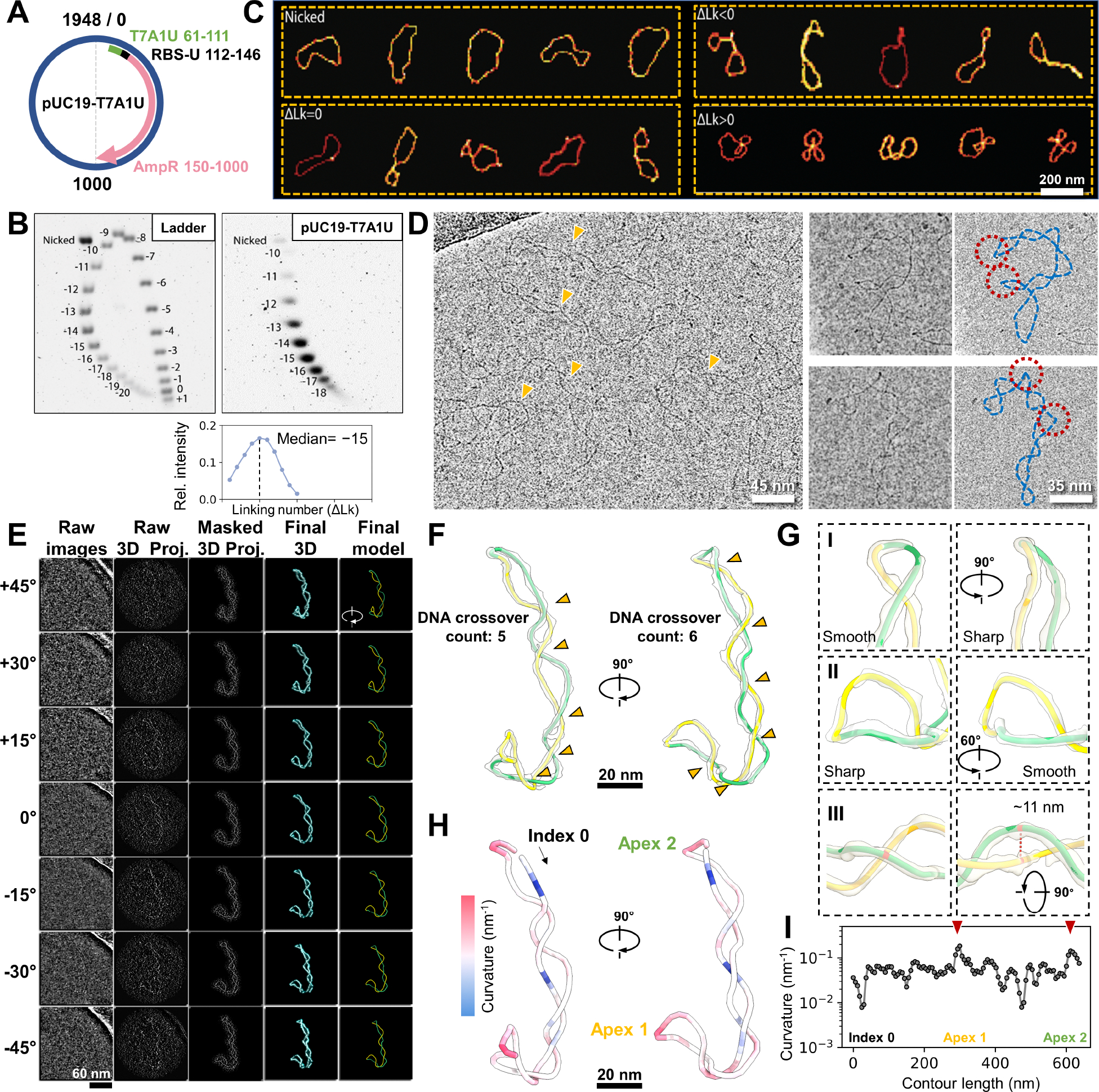
3D reconstruction of an individual negative-supercoiled (–sc) DNA plasmid (**A**) pUC19 plasmid construct features a T7A1 promoter (green), a U-less stalling site (black), and a transcriptional region (pink). (**B**) 2D gel electrophoresis of the –sc pUC19-T7A1U plasmid and ΔLk quantification. (**C**) AFM images of pUC19 plasmids showing ΔLk variants. (**D**) Cryo-EM images of –sc plasmid (orange arrowhead) highlighting sharp DNA kinks (red circle). (**E**) Cryo-ET per-particle 3D reconstruction steps of a representative –sc plasmid. (**F**) Zoom-in view of the final map and model from E, showing two intertwined DNA segments (yellow and green) with the DNA crossing marked by arrowheads in the view. (**G**) Different 3D views influence 2D assessments of DNA curvature (panels I, II) and spacing (panel III). (**H**) Color-coded map of the plasmid in F, with high-curvature regions in red and low-curvature regions in blue. (**I**) Tracing of the DNA curvature in H along the circular DNA plasmid.

To explore RNAP's interactions with its naturally occurring substrate, we examined the structure and dynamics of a ~2 kbp modified pUC19 -T7A1U circular plasmid (Figure 1A) isolated from early stationary-phase *E. coli* cells. 2D gel analysis revealed that the two DNA strands of the plasmid loop over each other 15 times fewer (ΔLk= −15) compared to their relaxed state (Lk_0_ =186), presenting a physiologically relevant supercoiling density (σ=ΔLk/Lk_0_) of −0.08 (Figure 1B). The size of pUC19 permits multiple plectoneme formation, as observed by AFM, leading to significant conformational deviation among ΔLk variants. (Figure 1C). To capture the native topological diversity of DNA free from surface constraints, we used cryo-electron tomography (cryo-ET)^25–27^ to resolve the 3D conformation of large plasmid molecules.

We initiated cryo-EM imaging under low-salt conditions (5 mM KCl) to capture loosely coiled plasmids,^14^ revealing predominantly long plectonemes (Figure 1D, **orange arrows**) and occasional sharp kinks (Figure 1D, **red circles**) resembling those seen by high-resolution AFM.^10^ Following a deep learning-based segmentation,^28^ local “per-particle” 3D reconstruction^26^, and IsoNet missing wedge correction^29^ (Video S1 and Supplementary Data 1), we obtained 3D maps of individual plasmids (Figure 1E) at resolution of ~30–40 Å (Supplementary Data, Particle gallery #006). Using a DNA detection algorithm with manual curation (Supplementary Data 2), we traced full 3D plasmid contours, enabling precise supercoiling quantification. For the example in Figure 1E, spatial quantification^30^ of right-handed crossings in 3D gave a writhe (Wr) of −6.2. Accurate structural analysis is difficult to achieve from 2D projections alone due to projection artifacts—e.g., DNA crossing number varies (Figure 1F, **orange arrows**), smooth curves may appear as sharp kinks, and true bends can be obscured in certain views. (Figure 1G, **panels I-II**). To overcome these limitations, we measured 3D curvature (reciprocal of osculating sphere radius; Figure 1H) and inter-helix spacing in plectonemes (Figure 1G, **panel III**). The curvature distribution showed two peaks (Figure 1I, **red arrows**), corresponding to plectoneme apices.

We then compared plasmids under low salt (5 mM K^+^; Figure S1A) and high salt (40 mM K^+^, 5 mM Mg^2+^, near-physiological ionic strength; Figure S1B) conditions, (see full dataset in Supplementary Data Particle Gallery). Under higher salt, plasmids exhibited increased elongation, indicated by a larger radius of gyration (Rg), and greater DNA intertwining with mean Wr shifting from −5.7 to −10.2; *P* <0.0001 (Figure S1C–D). This result aligns with theoretical predictions that electrostatic screening favors DNA crossing,^14^ promoting the conversion of negative supercoiling into writhe at the expense of twist (ΔTw). As a result, plectoneme width decreased with high salt from 13.2 to 8.4 nm; *P* <0.001 (Figure S1E), accompanied by increased mean curvature, particularly at distal apices (from 0.19 to 0.23 nm^−1^; *P* <0.001, Figure S1F–G). Given that some proteins recognize highly curved DNA region via indirect readout^31^, we assessed apex number distributions under both conditions and observed comparable results: ~60% of plasmids remaining unbranched (two apices; Figure S1H). Clearly, generation of new apices is energetically unfavorable. These analyses provide a structural framework for understanding supercoiling dynamics (see below).

### RNAP apical binding favors promoter escape and affects the configuration of the negatively supercoiled template

**Figure 2:**
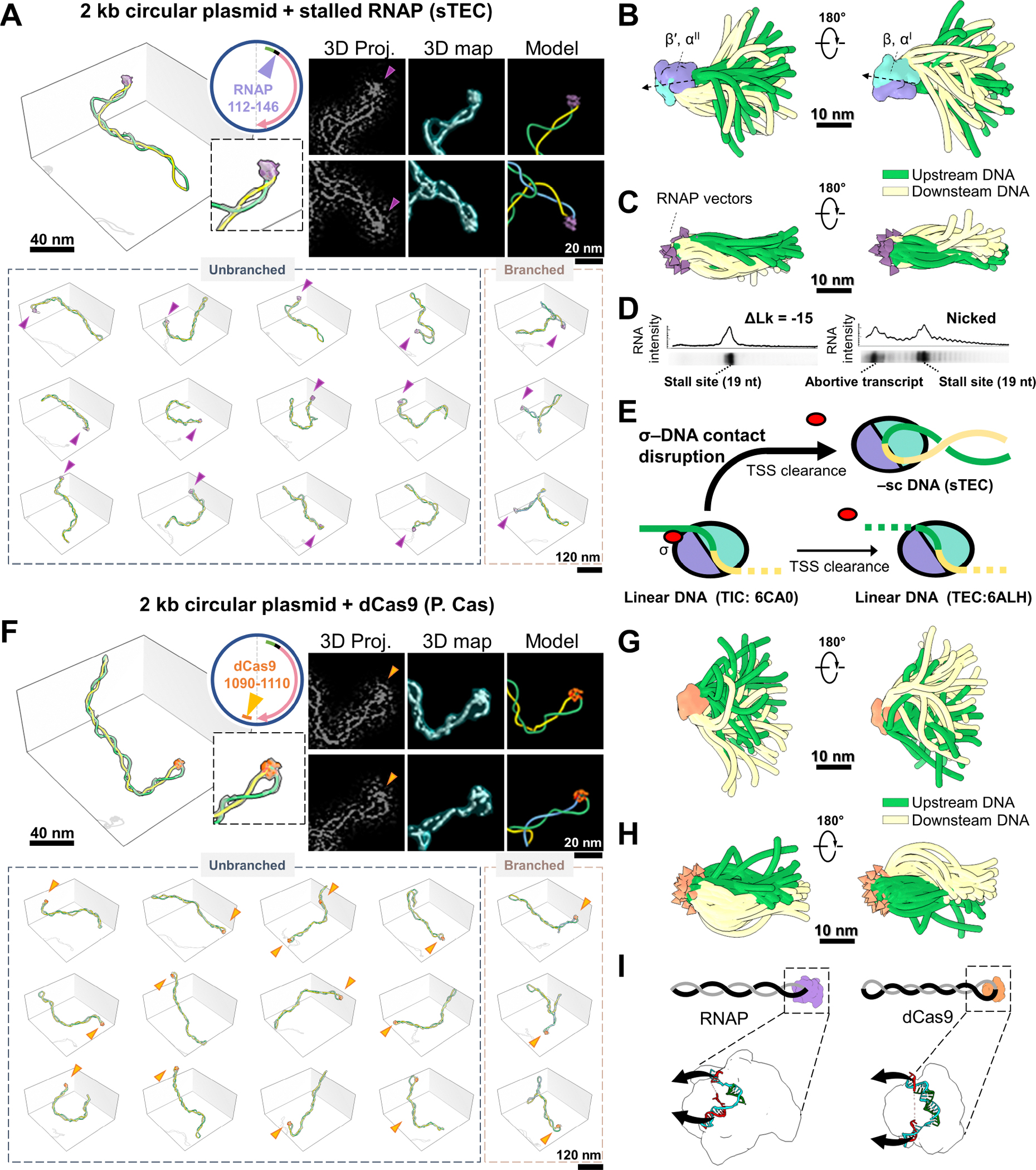
Conformational dynamics and regulatory effect of apically bound proteins on DNA supercoiling (**A**) Cryo-ET (3D maps and models) of the –sc plasmids bound with stalled RNAP (sTEC), with plasmid construct design in circle and plectoneme apex modeling steps on top right. RNAPs in sTECs are marked by purple arrowheads in the bottom particle collection (**B**) The superimposition of all bound RNAPs reveals apical DNA dynamics. The dashed vector indicates RNAP orientation. (**C**) The superimposition of all apical DNA segments shows the RNAP orientation dynamics (purple arrowheads). (**D**) Electrophoresis gel shows reduced abortive transcript levels on –sc DNA compared to nicked DNA. (**E**) Schematic highlighting DNA geometry conversion in TEC on –sc DNA (top), and in TIC (bottom left) and TEC (bottom right) on linear DNA templates. (**F**) Cryo-ET of dCas9 (orange) bound to –sc plasmids. (**G and H**) Superimposition of all bound dCas9s and all apical DNA segments, respectively. (**I**) Schematic highlights dCas9's smaller pocket curvature compared to that of RNAP, reshaping bound plasmids with enlarged distal ends and tightly intertwined body.

An early study in the 1990s revealed unexpected apical localization of RNAP on supercoiled DNA,^32^ a finding recently revisited by single-molecule experiments.^9^ To gain structural insights, we incubated RNAP with pUC19 -T7A1U plasmids (bearing a single T7A1-driven gene; Figure 2A, **top circle**) at a 3:1 ratio under high-salt conditions and obtained stalled transcription elongation complexes (sTECs) via UTP starvation, halting RNAP 19 nt downstream of the transcription start site (TSS). 3D reconstruction revealed that ~90% of RNAPs localized at plectoneme apices (Figure 2A, **lower panels**). Sub-tomogram averaging (STA) resolved RNAP orientation at the apex.^28^ Averaging all RNAP particles (bound and unbound) yielded a 14 Å map with blurred DNA entry/exit sites (Figure S2A–B, **top**), whereas averaging only plasmid-bound RNAPs (17 Å resolution) revealed a DNA density protrusion indicating the downstream direction (Figure S2A, **bottom, black arrow**). This feature is absent in RNAP structures resolved with linear DNA templates (4YLN, 6CA0, 6JBQ, 6N60,^33–36^ which show only upstream DNA bound to the σ factor (Figure S2C). Mapping STA-determined RNAP orientations onto their respective plasmid models (Figure 2A, **top right**) recovered DNA information otherwise averaged out. Superimposing all RNAPs at apical regions highlighted DNA's conformational flexibility (Figure 2B), while superimposing DNA at the same region revealed relatively restricted RNAP apical localization (Figure 2C).

Negative DNA supercoiling is believed to facilitate transcription initiation by enhancing open complex formation through DNA unwinding.^37,38^ However, its impact on other initiation events, such as promoter search and escape, remains unclear. Bulk transcription assays on nicked and negatively supercoiled (–sc) templates revealed more efficient promoter escape on –sc DNA, evidenced by a reduced rate of abortive initiation before RNAP reached the 19 nt stall site (Figure 2D). Structural superposition of the apically stalled TECs (sTEC) in –sc plasmids onto transcription initiation complexes (TICs) assembled on linear DNA (such as 6CA0) revealed a significant deviation in the consensus upstream DNA trajectory—approximately 170° bend in the former (Figure S2C). This reorientation of the upstream DNA in –sc complexes would disrupt most DNA-σ-factor interaction, if present (Figure 2E). As weakening of DNA-σ contacts is a necessary step for RNAP release from the promoter and transition into elongation^39^, this apical configuration on –sc DNA provides a possible explanation for the increased promotor clearance observed in the bulk assay (Figure 2E).

Having characterized RNAP’s apical binding, we asked whether this feature is unique to RNAP or shared by other DNA-binding proteins. We replaced RNAP with catalytically inactive dCas9 guided by an sgRNA targeting the plasmid's transcription termination site (TTS) (Figure 2F, **top circle**). We chose dCas9 for its RNAP-like DNA binding (i.e. forming a bubble via DNA-RNA hybrid) and its visibility (~10 nm) in cryo-ET. Notably, dCas9 also exhibited apical binding on –sc plasmids (Figure 2F, **bottom**) with STA analysis revealing the consensus entry-exit DNA orientation (Figure S2D, **arrows**; 17Å resolution, Figure S2E). While both proteins share apical binding, quantitative 3D analysis reveals structural differences between dCas9- and RNAP-plasmid complexes (P.Cas vs. sTEC), with the former displaying more elongated, tightly wound plectonemes, as indicated by increased Rg and a more negative Wr (−9.5 to −11.8; *P* <0.0001, Figure S2F, **top**). Enlarged distal loops were also observed at dCas9-bound apices, showing reduced curvature (from 0.25 to 0.22 nm^−1^; *P* <0.05) and increased DNA spacing (from 7.7 to 10.3 nm; *P* <0.05) compared to RNAP (Figure S2F, **bottom**). Mapping dCas9 orientation highlighted these distinctions (Figure 2G–H).

Why do two DNA-binding proteins with similar mechanisms reshape plectonemes differently? Structural comparison of RNAP^40^ and dCas9^41^ binding pockets revealed that, despite its larger size (15 vs. 10 nm), RNAP features a more acute binding pocket with a sharper curvature than dCas9 (Figure 2I), favoring DNA melting over negative writhe. Coarse-grained molecular dynamics (CGMD) simulations using oxDNA^42^ further support this conclusion, showing that tighter apex bending with strand separations of 4.8 and 6.4 nm (Figure S3A) yield sharper apices and reduced negative writhe compared to looser apex bending (8 nm separation) (Figure S3B, **left and middle**). A positive correlation (r =0.51) was observed between apex curvature and writhe number, based on data pooled from all trajectories (Figure S3B, **right**). Taken together, these findings suggest that apex localization is not unique to RNAP, but reflects a general structural adaptation of supercoiled DNA to the geometry of bound proteins that minimizes the energy of the overall configuration. Significantly, we found that DNA sequence at the promoter and dCas9 binding site played a minor effect on proteins apical localization^43^ (Figure S3C).

### Transcription induces branched supercoiling while preserving RNAP's apical localization

RNAP translocation along DNA requires rotation of either the enzyme or the template maintain register at the active site. When both are rotationally constrained—e.g., by RNAP tethering^44,45^ or DNA confinement within dense genome architecture^46–48^ —twin supercoiling domains can form.^20^ However, such external constraints might not always apply. We therefore asked whether intrinsic constraints arising from RNAP apical localization on a negatively supercoiled (–sc) template^48–50^ are sufficient to sustain transcription and generate topological domains.

**Figure 3:**
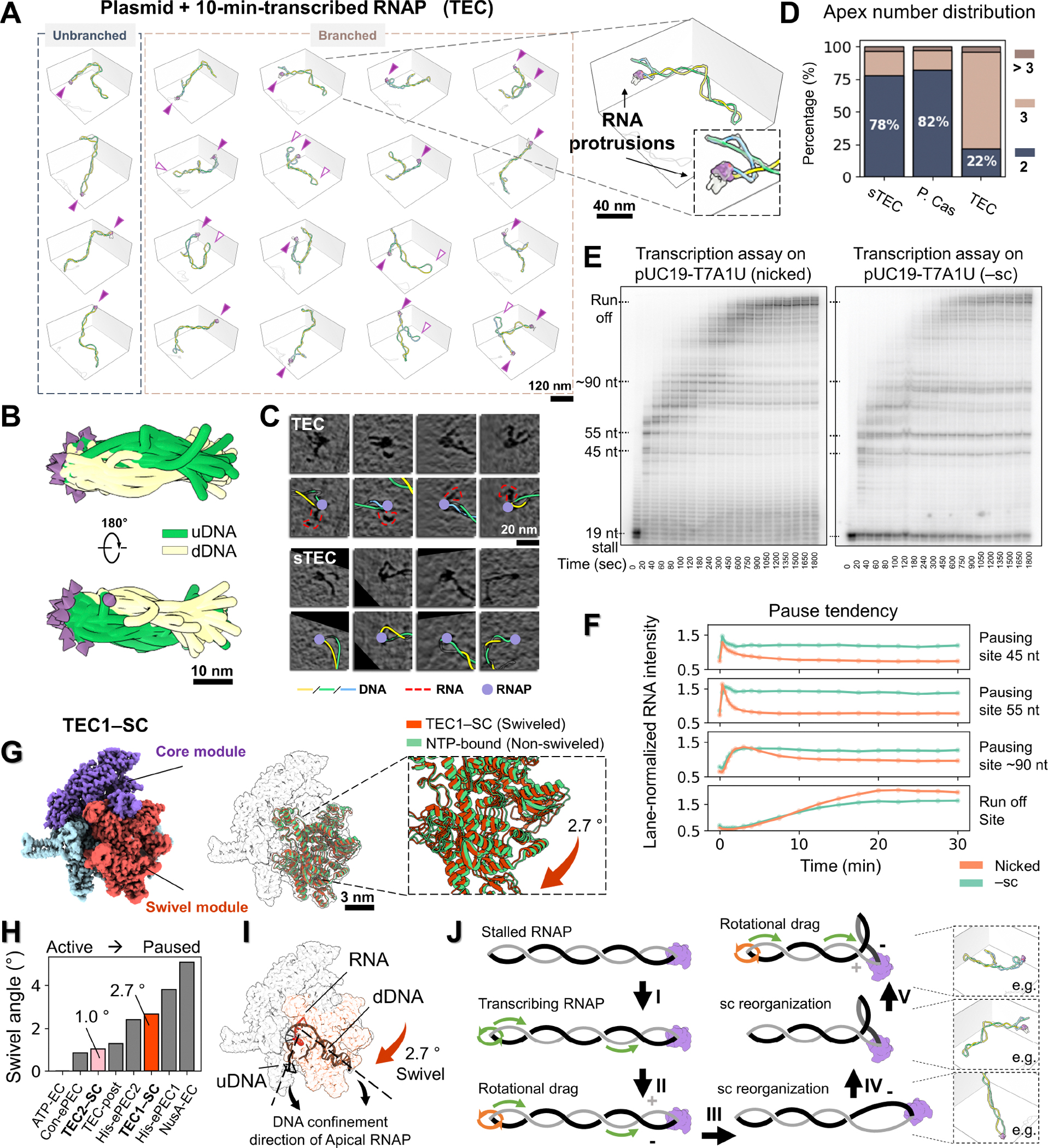
Interplay between apically bound RNAP and –sc DNA during non-equilibrium transcription (**A**) Cryo-ET of TEC on –sc templates after 10 minutes of transcription, with RNAP and large DNA loops indicated by solid and hollow purple arrowheads, respectively. (**B**) Alignment and superimposition of all apical DNA segments. (**C**) Z-dimensional slices (10 nm-thickness) of sub-tomograms comparing transcribed (top) and stalled (bottom) RNAPs, overlaid with models. (**D**) Plasmid apex number distribution; N=84. (**E-F**) Single-round *in vitro* transcription of RNAP transcription on nicked and –sc templates over time, with quantification of their pause release kinetics, respectively. (**G**) Single-particle cryo-EM reconstruction of the major RNAP class, TEC1–SC on –sc DNA, overlaid with the non-swiveled 6RH3 model. (**H**) Comparison of TEC1–SC swivel angle to reported RNAP structures from active transcription to various paused states. (**I**) TEC1–SC active-site DNA orientation and subunit swivel direction. (**J**) Schematic illustration of transcription-induced new plectoneme formation on –sc plasmid. DNA translational and rotational motion is indicated by green arrows, with orange color denoting drags.

To test this, we resumed transcription in stalled TECs (sTECs) by adding NTPs (100 μM each) and imaged samples after 10 min. Remarkably, 3D reconstructions show that RNAP remains apically localized in TECs (Figure 3A–B, **purple arrows**), with nascent RNA density intermittently observed around RNAP in both 3D maps (Figure 3A, **right**) and 2D slices (Figure 3C). Due to cryo-ET resolution limits and RNA heterogeneity^51^, only the direction of RNA protrusion relative to RNAP was annotated (Figure S3D, **arrows**). Quantitative analysis shows that actively transcribing plasmids predominantly adopt multi-branched conformations (>78%, Figure 3D) with reduced spatial extension and smaller radius of gyration (Figure S3E, **top, red arrow**). Blunt apices also emerge in TECs (hollow purple arrows, Figure 3A and S3E) but are rare in sTECs. While total writhe is similar (−9.5 vs. −9.8) (Figure S3E, **top**), branch-level analysis reveals a broader writhe density (branch Wr/branch length, Figure S3F, **I-III**) distribution with a slight increase in TECs (−0.13 vs. −0.15 nm^−1^; *P* <0.05). Branching preferentially occurs proximal to RNAP, consistent with localized torsional stress (Figure S3G). Together, these results indicate that intrinsic –sc constraints, coupled with active DNA translocation, generate torsional imbalance that promotes plectoneme branching.

Torsional stress in constrained templates is known to slow elongation and increase backtracking.^21^ Given that the persistent apical constraint of RNAP on plasmids arises from the natural steady-state degree of –sc, single-round transcription assays on –sc DNA revealed slower elongation kinetics (Figure 3E) and increased pausing compared to nicked DNA (Figure 3F). These observations raise the possibility that apically localized RNAP adopts a distinct conformational state. To test this hypothesis, we performed single-particle analysis (SPA) of TECs on –sc templates. 3D classification yielded three classes, with Class 1 and Class 2 (TEC1–SC and TEC2–SC) comprising 34% and 40% of particles (Figure S4A–C) and resolved at ~2.9 Å (Figure S4D–E). Both structures adopt a post-translocated state, featuring unfolded trigger helices and absence of incoming NTPs (Figure S4F). Minor Class 3 (26%) exhibited faint DNA density and was excluded from further analysis. Swivel angle analysis following Kang et al.^52^ found that TEC1–SC exhibits significant swiveling (~2.7°; Figure 3G), comparable to the swivel state displayed by the His-elemental paused elongation complex (His-ePEC, Figure 3H). Interestingly, we notice that the TEC1–SC DNA orientation (Figure 3I, **dashed line**) and the DNA confinement geometry in apically located RNAP (Figure 3I, **black arrow**) align with the swivel module orientation (Figure 3I, **red arrow**). As downstream DNA contacts this module, these results suggest that constrained DNA bending drives swiveling, thereby slowing elongation.

Building on these findings, we propose a model for transcription on intrinsically – sc DNA (Figure 3J): The persistent apical positioning of RNAP causes slower transcription and requires DNA rotation (I), but this rotation propagates slowly across the plasmid,^53,54^ generating transient (+) torsional stress ahead of and (−) stress behind the enzyme (II). The (+) stress is rapidly absorbed by the –sc background, preventing cancellation via diffusion (III). Under high salt, which favors writhe over twist (Figure S1D), the additional (−) stress promotes the formation of new plectoneme branches (IV-V).

### Dual apical protein binding promotes the formation of topological domains

The results above highlighted RNAP's role in generating torsional stress during non-equilibrium transcription of supercoiled DNA. Torsional stress during transcription can also accumulate due to the presence of torsional roadblocks, such as DNA-binding proteins and transcription factors,^55,56^ leading to the formation of topological domains that can affect RNAP molecules distant kilobases away.^57^ Nonetheless, the mechanism through which small DNA-binding proteins impose rotational constraints and favor the generation of topological domains remains elusive.

**Figure 4:**
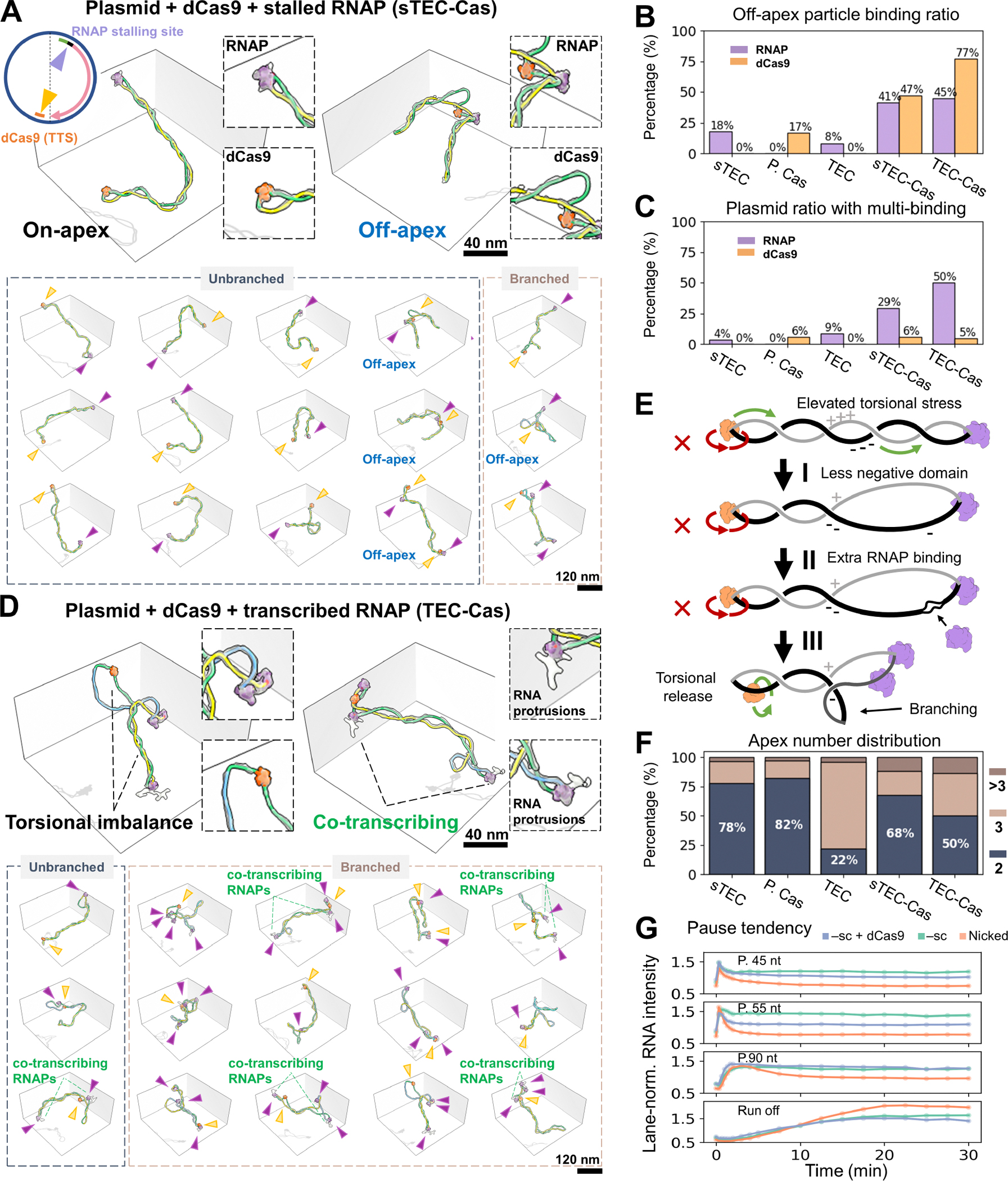
Plectoneme apical confinement promotes topological domains and multi-RNAP transcription (**A**) Cryo-ET of –sc plasmids simultaneously bound by stalled RNAP (purple) and dCas9 (orange). Representative particles show RNAP and dCas9 co-localized at the apex (top left) or off-apex (top right), both consistent with the opposing-position construct design (circular plot). (**B**) Off-apex particle ratios for RNAP and dCas9 across conditions; N=301 (**C**) Ratio of plasmids bound by multiple RNAPs or dCas9; N=324. (**D**) Cryo-ET of –sc plasmid resuming transcription for 10 minutes in the presence of torsional roadblock dCas9 (**E**) Schematics illustrating RNAP transcription-induced topological domains and supercoiling rearrangement in the presence of dCas9. (**F**) Quantification of plasmids’ apex number; N=162. (**G**) Assessment of RNAP pause release in the presence of dCas9 during transcription via electrophoresis. The RNA band intensities were quantified at three strong pausing sites and run-off site.

To gain insight into this process, we employed dCas9 again but in this case for its capability of delaying DNA rotation, as reported in magnetic tweezers assays.^58^ To confirm that dCas9 can serve as a programmable torsional roadblock, we performed DNA relaxation experiments using topoisomerase I (TopI) in the absence and presence of dCas9 bound to the transcriptional termination site (TTS) (Figure 4A, **top circle**). TopI relaxes torsion in –sc DNA by rotating the helix until equilibrium (ΔLk = 0) is achieved. Our results demonstrate that dCas9 delays the relaxation kinetics of pUC19 plasmids, and topoisomers with ΔLk from −3 to −1 still persist after a 30-minute reaction (Figure S5A). We repeated the experiment using human Topoisomerase IB (hTopIB), which relaxes supercoiling via a direct DNA rotation mechanism rather than strand passage (as in TopI), and similarly observed that dCas9 delays DNA relaxation (Figure S5B). Because dCas9 primarily delays, rather than halts, –sc relaxation, we term it a “soft” torsional block. To understand this mechanism, we performed CGMD simulations to investigate how a –sc plasmid relaxes after nicking, with or without an apical constraint. Results reveal that imposing an apical constraint on one of the two apices slows supercoiling torsional relaxation dynamics when the nick is positioned between the apices (Video S2 and Figure S5C, **Model II-III vs. I**). However, when the nick is placed directly on the apex, relaxation becomes fastest, even with the opposite apex constrained (Figure S5C, **Model IV**). Based on the observed relaxation speeds (IV > I > II and III), and given that model IV is the only scenario where torsional propagation encounters no apices (with the nick created at the very end), it suggests that the apex, particularly the confined apex (mimicking the effect of dCas9 binding), hinders free DNA rotation.

To experimentally test the role of torsional roadblocks on transcription, we first initiated transcription on pUC19 plasmids with dCas9 bound at the TTS (Figure 4A, **top circle**). Imaging of stalled TECs (sTEC-Cas) harboring a ~20 nt transcript revealed that RNAP and dCas9 predominantly localize to opposite plectoneme ends (Figure 4A **top left, and bottom columns 1–3**). We note that on unbranched plasmids, their apical localization is mutually reinforcing, as both favor and induce bending at their target sites (Figure 2A,F). Even under this mild transcription and stalling scenario, sTEC-Cas exhibited noticeably less negative writhe domains compared to the dCas9-bound-only plasmid (P.Cas), resembling instead those of active TECs (Figure S5D, **red arrow**). Notably, mild transcription conditions also led to a significant increase in non-apical binding cases (Figure 4A, **blue label** and Figure S5E) for both RNAP and dCas9 (from 18% to 41% and from 17% to 47%, respectively, Figure 4B). Given that the increased off-apex dCas9 ratio was not due to additional dCas9 binding events (both 6% for P.Cas and sTEC-Cas; Figure 4C), it suggests that transcription-induced accumulation of (+) torsional stress between the front of RNAP and dCas9, and (−) stress between dCas9 and the back of RNAP, promotes partial escape of the dCas9 from the apices as the system seeks to minimize its energy.

### Torsional block enhances cooperative RNAP transcription

As shown above, mild (+) and (−) torsional stresses differentially accumulate in each half of the plasmid, delineated by RNAP and dCas9, resulting in more frequent off-apical dCas9 events, suggesting the “soft” nature of dCas9 when acting as a torsional block. Accordingly, we anticipated increased off-apex localization of dCas9 after 10 minutes of active RNAP transcription with the full set of NTPs at 100 μM (TEC-Cas; Figure 4D). Indeed, the off-apex ratios of both RNAP and dCas9 increased, with dCas9 reaching 77% (Figure 4B). Meanwhile, we observed the coexistence of large, non-plectonemic DNA loops and tightly wound DNA regions within the same TEC-Cas particle (Figure 4D, **top left**). Quantitative analysis confirmed that, although overall plasmid morphology remained similar (Figure S6A, **top**), TEC-Cas exhibited a broader writhe density distribution across plectonemic branches, with two peaks above and one slightly below the mean of the plasmid-only control (Figure S6B, **red arrows**). This shift toward less negative writhe was accompanied by blunter apices, characterized by reduced apex curvature (from 2.5 to 2.3 nm^−1^; *P* < 0.05) and increased apical-DNA spacing (from 8.5 to 11.1 nm; *P* < 0.001, Figure S6A, **bottom**). Notably, less-negative domains were more prominent, which seems contrary to the expected symmetry of (+) and (−) torsion generated by RNAP (Figure 4E, **I**). This asymmetry can be rationalized by the marked increase in multi-RNAP binding (from 29% to 50%; Figure 4C) observed in TEC-Cas. Accordingly, as each bound RNAP unwinds ~10 bp of DNA, they absorb excess negative supercoiling (Figure 4E, **II**). Moreover, the increase in the proportion of multi-branch form plasmids in TEC-Cas (from 32% to 50%, Figure 4F), providing an additional mechanism to buffer excess negative supercoiling (Figure 4F, **III**).

Since structural studies provide only static snapshots of domain separation, we employed CGMD to better visualize how torsional domains dynamically emerge as a consequence of apical constraint in the absence of external tethering. We treated the domains as harmonic (linear torsional springs)^59^ to mimic the (+) and (−) torsion generated by RNAP. The simulations compare a free versus constrained apex in managing torsional stress (Video S3). Notably, the constrained apex successfully recapitulated the formation of large loops and branching structures observed experimentally.

Leveraging dCas9’s gRNA-directed DNA binding as a fiducial marker, we tentatively mapped RNAP positions and orientations on the plasmid construct (Figure S6C). Some RNAPs were found outside the transcriptional region or oriented upstream of the TSS (brown and blue arrows), which we interpret as non-specific binding enhanced by transcription-induced –sc domain. In contrast, most RNAPs were located within the transcriptional region and oriented downstream (pink arrows), with some plasmids displaying multiple transcribing RNAPs. The direct visualization of two RNAPs on the same template, both exhibiting nascent RNA protrusions (Figure 4D, **green labels**), supports this interpretation. These findings suggest that dCas9-induced topological imbalance in TECs promotes cooperative RNAP transcription, potentially enhancing transcriptional kinetics. To corroborate this hypothesis and link structure to function, we performed a bulk transcription assay in the presence of dCas9 (Figure S6D). The results show that while pause frequency remains unchanged, pause escape is faster in the TEC-Cas—particularly at the 45 nt and 55 nt pause sites (Figure 4G, **blue vs. green curve**). This enhanced pause escape suggests that the accumulation of (+) torsion ahead of RNAP, induced by the dCas9 torsional block, relieves the enzyme’s apical constraint on a –sc template, thereby facilitating elongation—though not to the level observed with nicked templates (Figure 4G, **orange curve**). Together, these results suggest that, in the context of –sc, twin-domain formation facilitates both RNAP initiation and elongation, with a stronger effect on the former.

### TopI removes the apical constraint of RNAP facilitating DNA transcription

**Figure 5:**
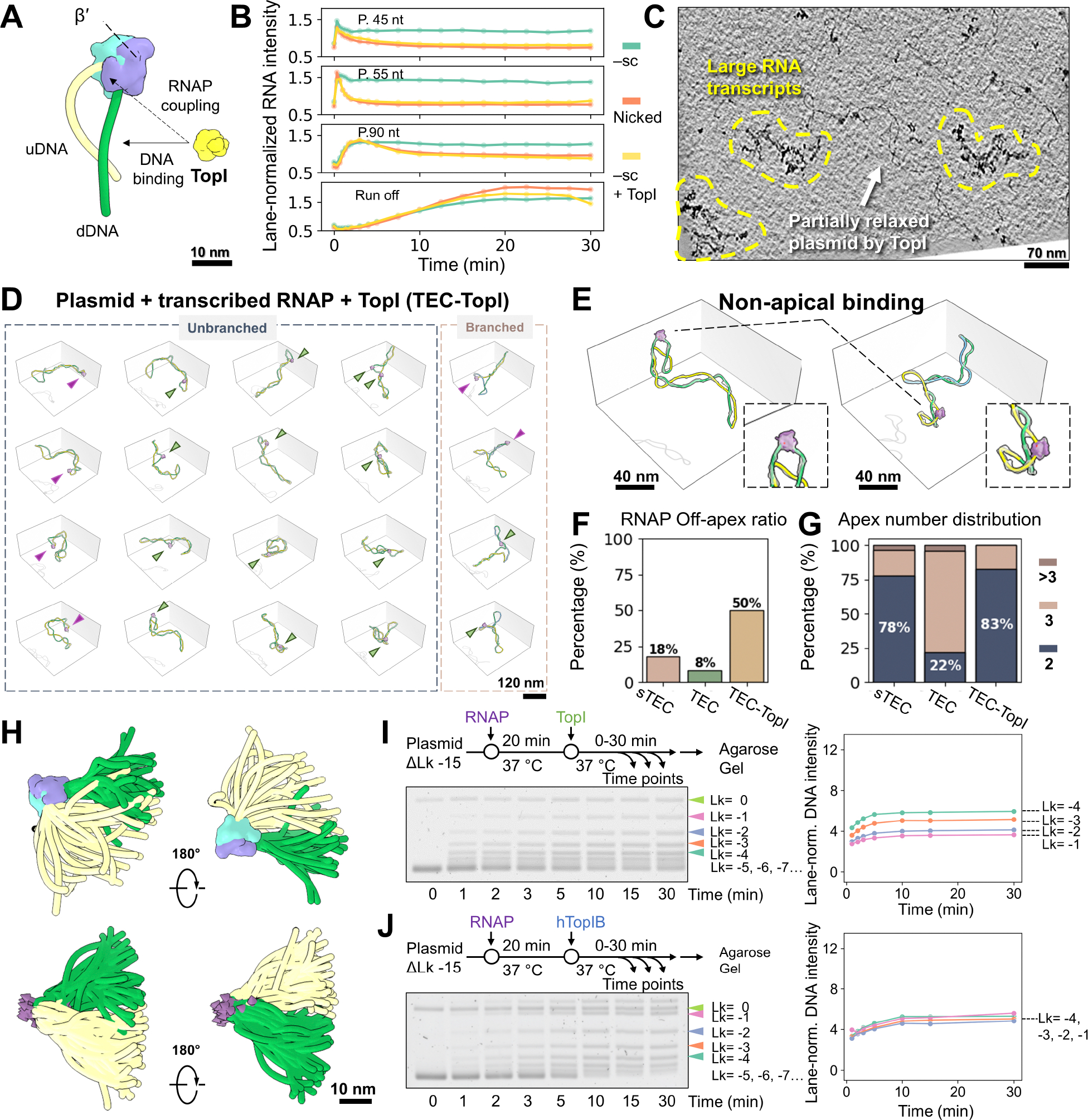
TopI releases RNAP from apical constraint during transcription (**A**) Schematic showing TopI bound to DNA or coupled with RNAP. (**B**) Assessment of RNAP pause release in the presence of TopI from single-round *in vitro* transcription assay. (**C**) Cryo-ET z-dimensional slice (50 nm-thickness) of sample after 10 minutes of transcription in the presence of TopI. (**D**) Cryo-ET of the plasmid particles in the presence of RNAP and TopI after 10 minutes of of transcription (TEC-TopI). Apical and non-apical RNAP binding are indicated by purple and green arrowheads, respectively. (**E**) Representative TEC–TopI complexes showing RNAPs escape from the apices. (**F-G**) Quantification of the non-apical RNAP ratio (N=90) and plasmid’s apex number (N=85), respectively. (**H**) Superimposition of all bound RNAPs (top panel) and all apical DNA segments (bottom panel). (**I-J**) DNA supercoiling relaxation assays mediated by TopI and hTopIB in the presence of RNAP, respectively, with ΔLk quantification shown on the right.

Although the presence of a torsional roadblock like dCas9 promotes transcription by multiple RNAPs, it could not fully replicate the elongation dynamics seen on nicked circular plasmids. Accordingly, we sought to determine if TopI^60,61^ (Figure 5A) could alleviate the apical constraints on RNAP by relaxing the DNA plectoneme and thereby relieve RNAP from its pauses.

Bulk transcription assays on –sc plasmid with TopI confirmed markedly improved transcription (Figure 5B, **yellow vs. green, and** Figure S6E), exhibiting kinetics similar to those of nicked templates without torsional stress (Figure 5B, **orange**). Based on these results, we conducted a cryo-ET imaging study of RNAP transcription in the presence of TopI (RNAP:TopI:plasmid=3:1:1). 10 minutes after the re-start of the sTEC, TEC-TopI sample revealed the presence of large RNA transcripts (Figure 5C). Moreover, TopI activity alone is sufficient to displace 50% of RNAPs from their apical localization, even in the absence of a torsional block (Figure 5D, green arrows, and Figure 5E), yielding the highest off-apex ratio across all tested conditions (Figure 5F), and without inducing plasmid branching (Figure 5G). Quantitative analysis showed that blunt apices predominated in TopI-relaxed plasmids, in contrast to unrelaxed ones, with a marked reduction in curvature (from 0.25 to 0.18 nm^−1^; *P* <0.0001) and an increase in DNA spacing (from 7.3 to 15.2 nm; *P* <0.0001). (Figure S6F). We attribute this prominent apex structural change to the relaxation of the plectoneme (mean Wr =−6.4; Figure S6F). Analysis of RNAP orientations in TEC-TopI particles reveals highly dynamic entry–exit DNA (Figure 5H), in contrast to the apically constrained configuration observed in TECs (Figure 3B). These results suggest that not only torsional constraint, but also a high degree of –sc, is required for RNAP apical localization and the longer pauses observed during single-round transcription.

Interestingly, while TopI facilitates RNAP transcription, RNAP also modulates TopI activity. Previous bulk assays showed that TopI alone (1:1 with plasmid) rapidly relaxed supercoils (ΔLk from −15 to −1) within minutes (Figure S5A). However, the addition of RNAP (plasmid:RNAP:TopI = 1:3:1) notably slowed relaxation kinetics, with topoisomer bands clustering at ΔLk < −4 even after 30 minutes (Figure 5I). 2D gel further resolved these bands, revealing accumulation of topoisomers at ΔLk = −8 (Figure S7A). Given the reported coupling between RNAP and TopI^8^ (Figure 5A, **dashed arrow**), which could potentially impair TopI function, we performed a control experiment with excess TopI (plasmid:RNAP:TopI = 1:3:9; Figure S7B). The relaxation kinetics were similar to those observed at a 1:3:1 ratio, indicating that the slower relaxation is not due to TopI inactivation upon interaction with RNAP, but rather reflects RNAP’s torsional block function—similar to the dCas9-mediated TopI downregulation (Figure S5A). Moreover, hTopIB, known not to interact with RNAP, showed a similarly slowed relaxation pattern on RNAP-bound plasmids, further supporting the above interpretation (Figure 5J). Interestingly, the torsional block effect of RNAP appears to be more pronounced than that of dCas9 (dCas9-topoisomers clustered at ΔLk = −1 to −3), likely due to its larger size and its ability to induce sharper kinks when it melts the DNA duplex. Indeed, supporting the requirement of DNA melting for DNA-binding protein to act as a roadblock, use of RNAP core—which binds DNA but cannot form stable transcription bubbles—resulted in faster relaxation than the holo-enzyme and ended in fully relaxed plasmids. (Figure S7C). Consistently, dead EcoRI mutant (dEcoRI), which bends but does not melt DNA, failed to act as a torsional barrier and permitted rapid, full relaxation (Figure S7D). These results suggest that both DNA bending and duplex melting are required for a protein to function effectively as a torsional block.

### Tandem promoters facilitate supercoiling domain formation and favors transcription over opposing promoters

**Figure 6:**
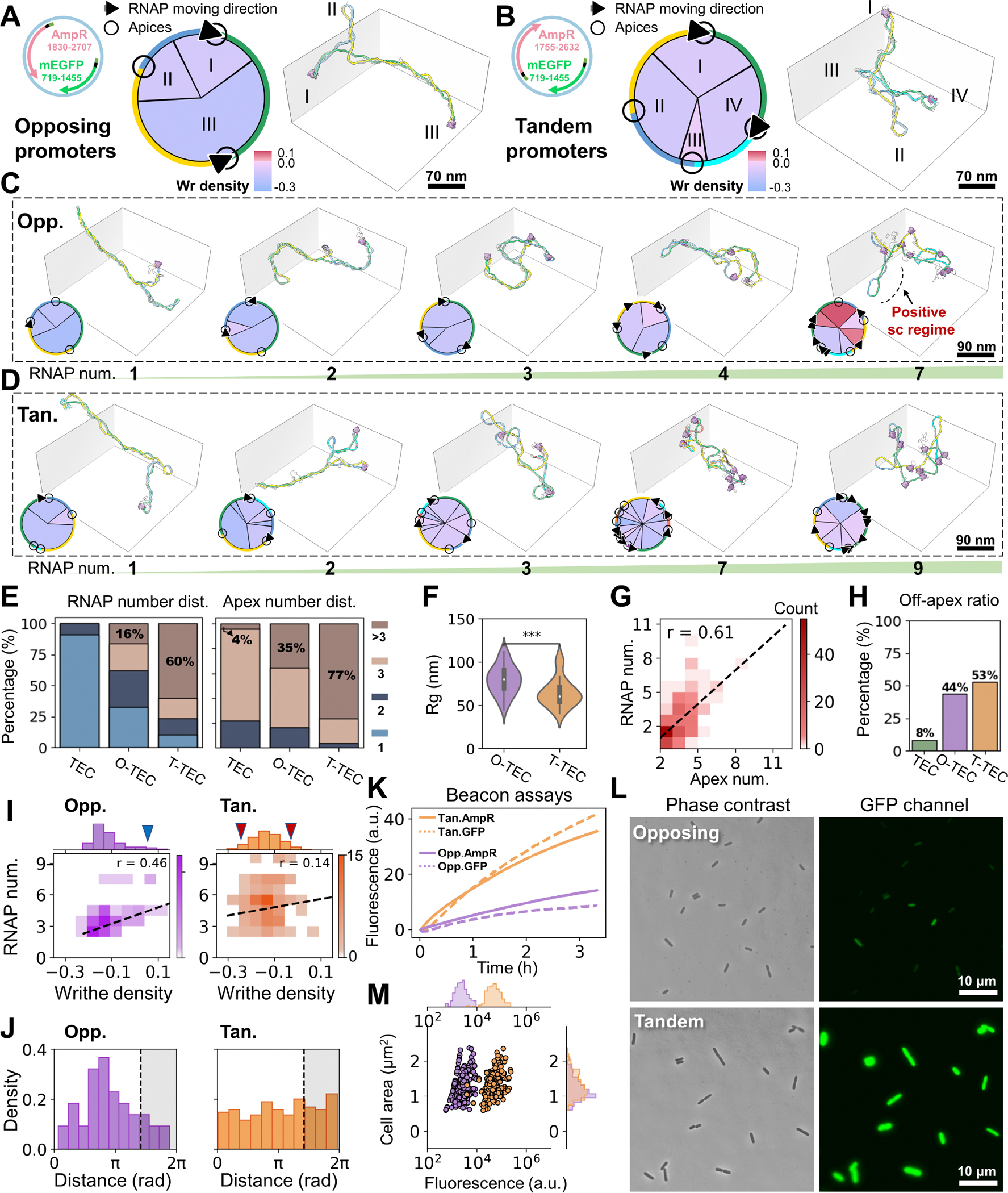
Tandem promoters enhance topological domain formation and transcription compared to opposing promoters. (**A**) Opposing dual-T7A1U promoter construct (left) driving transcription of AmpR and mEGFP. Circular plasmid layout (middle) with rim color-matched to the 3D model (right). Arrowheads indicate RNAP transcription direction; circles mark apical sites. Inner sectors represent individual plectonemes (I–III), color-coded by writhe density (blue–pink–red). (**B**) Tandem promoter construct, identical to A, but with AmpR flipped. (**C-D**) Representative TECs from opposing and tandem constructs, ordered by RNAP count. (**E**) Plasmid distribution by bound RNAP number and apex count; N=90. (**F-H**) Quantification of plasmid Rg (N=67), correlation between RNAP number and apex count (N=206), and ratio of RNAP located off-apex; N=232. (**I**) Plectoneme writhe density distribution vs. bound RNAP number; N=230 (**J**) Distance distribution of opposing RNAPs (left) and tandem RNAPs (right) on the circular plasmid layout. Shaded regions indicate less likely RNAP separations, attributed to the non-specifically bound RNAP population; N=212 (**K**) Quantification of *in vitro* gene expression by molecular beacon assay. (**L-M**) Imaging and quantification of in vivo GFP gene expression in *E. coli* MG1655 carrying either plasmid of tandem or opposing constructs; N=529.

We learned from the above experiments that the restricted rotational freedom of RNAP at apices confers dual functions: (1) generation of torsional strain during transcription, a prerequisite for the twin-domain model, and (2) serve as a torsional roadblock for a second transcribing RNAP as is also observed with dCas9. This dual function parallels the cellular scenario where multiple RNAPs co-transcribe the same genomic DNA, enabling transcriptional coupling between neighboring genes through the formation of topological domains.^62^ To gain insight into this system, we designed dual-promoter circular pUC19 plasmids (2.8 kb) containing two identical T7A1 promoters, each with a U-less stalling sequence, driving transcription of AmpR and mEGFP, either in opposite (topologically mixed convergent and divergent; Figure 6A, **left**) or tandem (i.e., same-direction; Figure 6B, **left**) orientations. After UTP starvation–induced stalling, TECs were imaged after 10 minutes of transcription. To facilitate visualization of these more complex structures, we projected plasmids onto a circular layout (Figure 6A–B, **middle**), with the rim color-coded to match the 3D molecular model (Figure 6A–B, **right**). Arrowheads indicate the transcription direction of bound RNAPs, and circles mark apical sites. Inner circular sectors represent individual plectonemes (e.g., I, II, and III), color-coded by its writhe density (blue–pink–red, −0.3 to 0.1) (Figure 6A, **middle**).

Significantly, as RNAP loading increased, plasmids underwent major reconfiguration from elongated plectonemes to expanded, globular structures (Figure 6C, opposing, and Figure 6D, **tandem**). Under identical reaction conditions, we observed a higher number of RNAPs in tandem TECs (T-TECs) compared to opposing TECs (O-TECs). T-TEC particles exhibited up to 10 bound RNAPs and 9 apices, with 60% of plasmids bound by more than 3 RNAPs and 77% forming more than 3 branches (Figure 6E). In contrast, multi-RNAP loading and multi-apices were down to 16% and 35% in O-TECs, and dropped further to 0% and 4%, respectively in single-promoter TECs, respectively. As a result, the highly branched T-TECs exhibited reduced plectonemic extension, with a significantly smaller Rg compared to O-TECs (64 nm vs. 79 nm; *P* <0.001, Figure 6F). Statistical analysis confirmed a positive correlation between RNAP occupancy and plasmid branching (*r* = 0.61, Figure 6G), suggesting that RNAP number potentially reflects the generation of domain separation. Notably, the higher number of RNAPs was not necessarily localized at apices. In both O-TECs and T-TECs, increased multi-RNAP binding led to elevated off-apex RNAP localization, reaching 44% for O-TECs and 54% for T-TECs (Figure 6H), in contrast to off-apex localization of single TECs (8%), but comparable to levels observed in TEC-Cas and TEC-TopI (45% and 50%, respectively).

The overall phenomena of multi-RNAP binding and multi-branching are not fundamentally distinct from those observed in TEC-Cas, but rather represent more extreme cases, especially in T-TECs. This result supports the continued validity of the TEC-Cas model (Figure 4E), with the RNAP version of “dCas9” reinforcing domain separation through its dual functions. In this context, we compare O-TECs and T-TECs in their supercoiling domain formation. Visually, as more RNAPs were loaded, a shift toward non-plectonemic DNA loops (lower negative writhe density; pink circle segments, Figure S7E) was more frequently observed in T-TECs than in O-TECs. This trend was further confirmed as increased number of RNAPs led to a broader writhe density distribution of T-TECs, displaying enriched hypo and hyper –sc domains compared to O-TECs (Figure 6I, **red arrows**). In contrast, a subset of O-TECs exhibited plectoneme writhe densities extending into the far-positive regime (Figure 6I, **blue arrow**). Examination of these particles revealed, for the first time, TECs with long positive plectonemes (Figure 6C and S7E, **red label**), supporting that opposing RNAPs, transcribing toward each other mutually act as torsional blocks that accumulate positive supercoiling. One T-TEC also showed short positive supercoiling (Figure S7E), but likely due to DNA entanglement between plectonemes. Nevertheless, this observation also suggests the possibility of mutual torsional interference between tandem RNAPs due to differential translocation speeds, consistent with the on–off apex model observed in TEC-Cas particles.

Interestingly, we observed a higher correlation between the writhe density and RNAP number in the O-TECs than T-TECs (r =0.46 vs. r =0,14, Figure 6I). We interpret it as the accumulation of torsional stress in the opposing geometry leading to higher changes in writhe compared to the cancellation of torsional stress when RNAP transcribes in tandem (i.e., further multi-RNAP loading has limited impact on torsional accumulation). Furthermore, by identifying all possible RNAP pairs on the plasmid and measuring their angular separation in polar plots, we found that opposing RNAPs were more likely to be separated by 2.7 radians (~1.2 kbp) (Figure 6J), in contrast to the uniform distribution observed for tandem RNAPs—further supporting a model of transcriptional hindrance in opposing configurations.

To confirm the functional consequences of the observed structural differences between tandem and opposing gene arrangements, we measured gene expression both in vitro and in vivo. RNA molecular beacon assays^63^ revealed comparable in vitro transcript levels of AmpR and mEGFP within the same construct (Figure 6K), as expected since both genes are driven by identical promoters (T7A1). However, higher RNA levels were observed with the tandem arrangement compared to the opposing one for both genes (Figure 6K). We also compared in vivo gene expression of mEGFP by measuring fluorescence emission in live *E. coli* cells harboring either construct, grown in M9GluCAAT medium. Consistent with the in vitro results, tandem constructs showed nearly a ten-fold higher fluorescence than their opposing counterparts (Figure 6L–M). These results are highly consistent with our cryo-ET observations: the tandem gene arrangement promotes increased transcriptional activity, including higher RNAP loading, increased numbers of off-apex RNAPs less prone to pausing, and lower accumulated torsional stress due to the cancellation of neighboring RNAPs.

## DISCUSSION

We demonstrate that cryo-ET, combined with modern image processing, enables detailed 3D visualization of highly dynamic DNA/protein complexes previously underexplored. By reconstructing the full DNA backbone of plasmids extracted from cells and bearing natural –sc densities, we precisely quantify plectoneme conformational dynamics. This marks a major advance in understanding DNA supercoiling, previously limited to surface-level AFM,^10,64^ negative-stain electron microscopy,^65^ or cryo-ET imaging of ~300 bp minicircles.^12^ By directly imaging individual ~2–3 kb plasmids, we quantify writhe and show that under near-physiological ionic strength (40 mM KCl, 5 mM MgCl_2_), torsional stress is stored primarily as writhe (Wr ≈ −10 for ΔLk ~ −15; Figure S1B, S1D). We find that lowering the ionic strength reduces Wr to −5, increases DNA strand spacing of plectonemic DNA and lowers apex curvature. Previously, such 3D conformational changes were accessible only through coarse-grained Brownian dynamics simulations.^66,67^ Our findings provide a basis for refining theoretical models to better reflect DNA’s typical topological state in cells.

Our full DNA tracing in 3D reveals that DNA-melting proteins like RNAP and dCas9 preferentially localize at plectoneme apices when bound to –sc DNA (Figures 2A, 2F). We find that this apical localization, first noted by ten Heggeler-Bordier et al.^32^ and revisited by Janissen et al.,^9^ is sequence-independent and persists as RNAP translocates along DNA (Figure 3A). Direct observation of RNA transcripts shows that this geometry helps segregate nascent RNA from the DNA body (Figure 3A–B), potentially aiding co-transcriptional events such as RNA folding, RNA processing, ribosome binding, while preventing RNA-DNA hybrid formation. Notably, RNAP localizes apically only at σ ≈ −0.08 (ΔLk = −15); relaxation by TopI to σ ≈ −0.04 (ΔLk = −8) reduces this localization (Figure 5F), suggesting a possible role of –sc on gene expression.

Beyond stabilizing open complex formation,^37^ we show that –sc promotes transcription by facilitating promoter escape and reducing abortive initiation (Figure 2D). The ~170° bending induced on the upstream DNA in the apically positioned TECs disrupts the majority of σ-factor:DNA contacts, a requirement for promoter clearance, offering an additional layer of regulation and helping rationalize the supercoiling sensitivity of certain bacterial promoters.^68^ Negative supercoiling also enhances RNAP binding following the formation of hyper-negative domains (Figure S6B), likely accelerating promoter search and triggering transcriptional cascades.

However, supercoiling can also impede transcription by promoting RNAP pausing (Figure 3F), in line with single-molecule studies.^21^ In contrast to those studies, which used surface fixed DNA, our work shows that under no additional torsional barrier other than the inherit-torsional stress of plectonemic DNA, the apical localization of RNAP can induce a swiveled state in RNAP with increased propensity to pausing (Figure 3G). While nascent RNA structures, transcription factors, and DNA sequence are known to promote pausing by stabilizing the swiveled state, our results reveal that DNA topology, through apical localization, can similarly regulate this conformational transition. This supercoiling-induced pausing is reversed by TopI (Figure 5B) and also by the action of co-transcribing RNAPs in tandem, which reduces apical localization (Figure 6H), restores elongation, and promote a more “fluid” transcriptional mode.^24^

**Figure 7.**
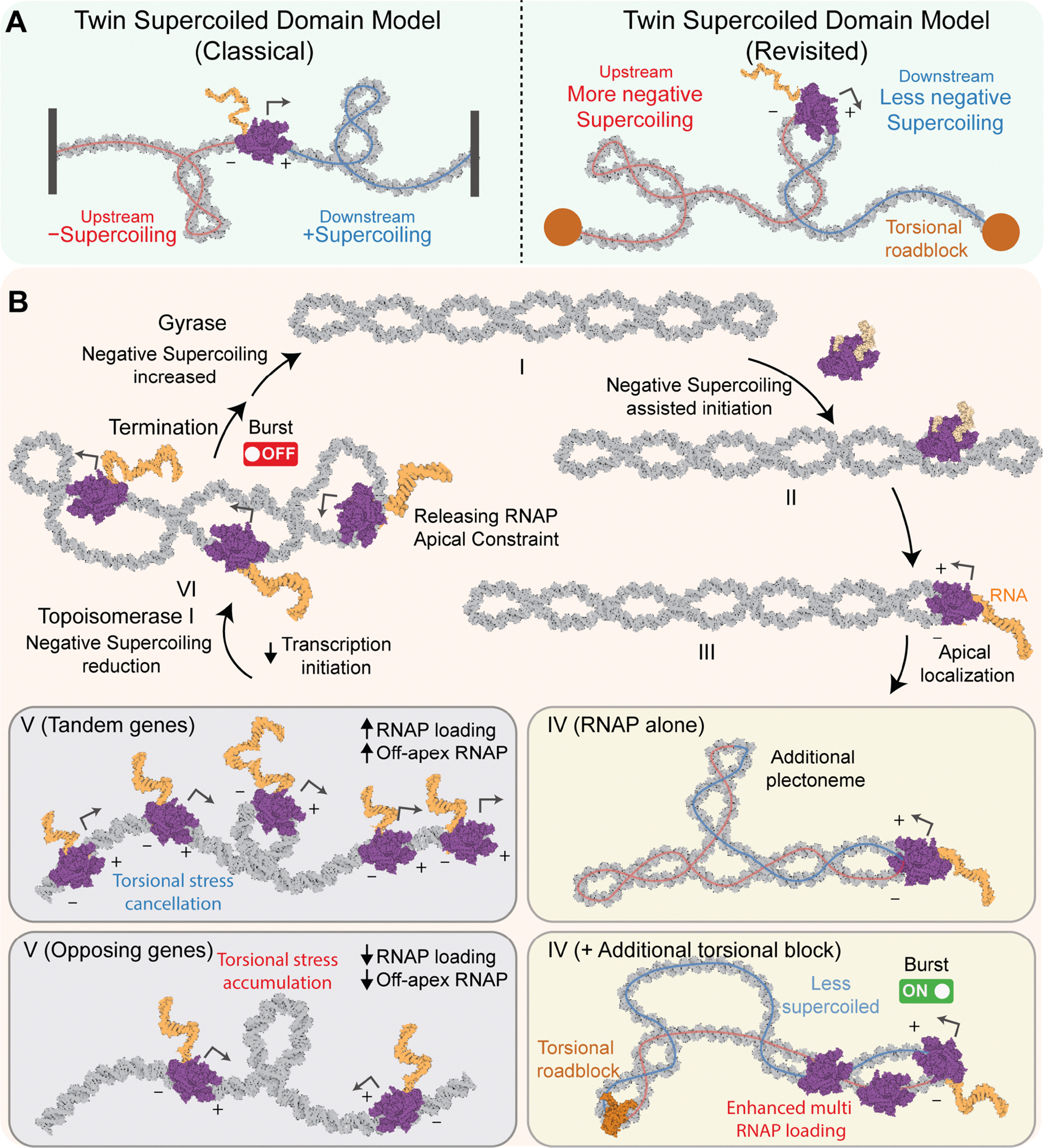
Revised twin-supercoiled domain model and its connection to transcriptional bursting. (**A**) Comparison of classical twin-supercoiled domain and a revised model considering the apical localization of RNAP in –sc DNA. (**B**) Summary model connecting transcriptional induced torsional stress and transcriptional bursting in –sc DNA. (See Discussion for further description of the model).

The twin-supercoiled domain model has long explained transcription-induced supercoiling in prokaryotes and eukaryotes,^5,24,69,70^ but direct visualization of the resulting supercoils (Figure 7A, **left**) has remained elusive. Early models suggested that long RNA transcripts or anchoring to cellular structures were required to prevent RNAP rotation and allow torsional stress to build. Here, we show direct structural evidence that rotationally constrained, apically localized RNAP—with even short (~20 nt) transcripts—can induce new plectoneme formation. This suggests long transcripts are not necessary to constrain RNAP, aligning with recent RNase-treatment studies.^9^ A single apically positioned RNAP can generate plectoneme branching, as observed in our experiments and predicted by other simulation work,^71^ though the latter required unrealistically high elongation rates and forces. Incorporating our 3D model of the apical RNAP configuration may help reconcile this discrepancy. Under physiological conditions with initial negative supercoiling, positive supercoil formation was rare, contrary to textbook models. Instead, we observed writhe density changes upstream and downstream of RNAP (Figure 7A, **right**), especially when torsional barriers such as neighboring RNAPs were present. This role of RNAP and DNA-melting proteins like dCas9 may extend to similar enzymes in both domains of life. DNA-bending proteins like dEcoRI did not show this effect, but other factors such as nucleoid-associated proteins^72^ or nucleosomes—which store ~1.7 DNA turns—may also act as torsional barriers.^71,73^ Together, these observations define a revised twin-supercoil model that updates a canonical textbook framework under physiologically relevant, tether-free negative supercoiling.

Negative supercoiling promotes RNAP initiation but hinders elongation, supporting a pulse-like transcription model reminiscent of in vivo “transcriptional bursting”.^74,75^ Single-molecule studies have linked supercoiling to this behavior.^22^ Based on our findings, we propose an updated model for –sc DNA (Figure 7B, **I–III**). Initially (Figure 7B, **I–III**), physiological DNA stores negative torsion that facilitates transcription initiation, promoter escape, and apical RNAP localization. In the absence of additional torsional roadblocks besides an apically located RNAP, transcription-induced supercoiling may lead to the formation of additional plectonemes (Figure 7B, **IV, RNAP alone**). In contrast, additional torsional blocks segment topological domains—reducing the negative writhe in front of the RNAP while increasing it behind, leading to enhanced RNAP binding and transcriptional burst initiation (Figure 7B, **IV +additional torsional block**). When RNAPs bind in tandem, they can cancel each other’s torsional stress, promoting transcriptional synergy (Figure 7B, **V, Tandem**); however, opposing RNAPs may stall due to accumulated stress (Figure 7B, **V, Opposing**). To complete transcription, topoisomerase relieves –sc, displacing RNAPs from the apex and facilitating elongation (Figure 7B, **VI**). The requirement for topoisomerase in active transcription is supported by the direct interaction between *E. coli* TopI and RNAP^60^ and by the observed recruitment of topoisomerase at transcription start sites.^8,61^ The resulting relief of negative torsion reduces the likelihood of new initiation, effectively acting as an OFF switch. The cycle can restart via ATP-dependent DNA gyrase activity, which restores negative supercoiling.

## LIMITATIONS OF THE STUDY

Due to the limited plasmid size and circular topology under the imaging conditions, orientation classes are not entirely independent. Consequently, while the system allows systematic classification across all major gene orientations (tandem, convergent, and divergent), it cannot fully separate convergent and divergent configurations —a coupling that nonetheless also occur naturally in vivo.

## RESOURCE AVAILABILITY

### Lead Contact

Further information and requests for resources and reagents should be directed to and will be fulfilled by the Lead Contact, Carlos Bustamante (carlosb@berkeley.edu).

### Materials Availability

All unique reagents generated in this study are available from the lead contact with a completed Materials Transfer Agreement.

### Data and Code Availability

- Cryo-ET maps of ~2 kb negatively supercoiled pUC19-T7A1U plasmids under multiple conditions were deposited in the EMDB, including low salt (EMD-47843), high salt without additional proteins (EMD-47844), stalled RNA polymerase (EMD-47847), dCas9 (EMD-47849), active transcription with RNA polymerase (EMD-47850), stalled RNA polymerase with dCas9 (EMD-47851), transcribed RNA polymerase with dCas9 (EMD-47853), and transcribed RNA polymerase with Topoisomerase I (EMD-47855). Cryo-ET maps of ~2.8 kb negatively supercoiled pUC19-T7A1U plasmids with opposing and tandem promoters were deposited under EMD-71622 and EMD-71618, respectively. Single-particle analysis maps of TEC1–SC and TEC2–SC were deposited under EMD-71675 and EMD-71676, with corresponding atomic models available in the PDB (9PIP and 9PIQ).The individual particle 3D reconstructed maps, models, original gel images, fluorescence images, the script calculate the TECs swivel angle, and data used to generate the statistical analysis presented in the figures have been deposited on GitHub Zenodo at (https://doi.org/10.5281/zenodo.16423113).
- This paper does not report original code.
- Any additional information required to reanalyze the data reported in this paper is available from the lead contact upon request.

## STAR Methods

### EXPERIMENTAL MODEL AND STUDY PARTICIPANT DETAILS

All experiments in this study were performed using purified biomolecular components and plasmid DNA derived from *Escherichia coli* (DH5α, BL21(DE3) pLysS, Rosetta(DE3) pLysS MG1655, and NEB Stable competent cells). No human participants or vertebrate animals were involved. Bacterial strains were cultured in standard LB medium, and plasmid DNA was extracted using a QIAGEN Maxiprep kit in accordance with the manufacturer’s instructions.

### METHOD DETAILS

#### Oligonucleotides and RNA

All DNA oligonucleotides listed below were obtained from Integrated DNA Technologies (IDT). Except for PCR primers, all oligonucleotides were purified in-house using denaturing urea-polyacrylamide gels (PAGE) prepared from SequaGel UreaGel 29:1 Concentrate (National Diagnostics).

#### Preparation of single guide RNAs (sgRNAs)

sgRNA specific oligonucleotides and scaffold oligonucleotides (CBD03 and CBD04) were ordered from IDT and purified in-house by polyacrylamide-gel electrophoresis (PAGE). In a 30 μL reaction, oligonucleotides (20 μL, 50 μM) were annealed and extended by T4 DNA polymerase in CutSmart 1X buffer to produce the dsDNA template. After heat inactivation, 10 μL of this reaction (~33.3 μM dsDNA template) were used for a 20 μL in vitro transcription reaction using HiScribe T7 In vitro Transcription Kit (New England Biolabs). In vitro transcription reactions were performed at 37°C for 6 hours, after which Dnase I was added to degrade the DNA template. sgRNAs were purified by urea PAGE. Bands corresponding to the correct RNA size were cut out as gel slices, eluted overnight at room temperature in 2X PK buffer (200 mM Tris-HCl, pH 7.5, 25 mM EDTA, pH 8.0, 300 mM NaCl and 2% SDS (w/v)), phenol-chloroform extracted and precipitated with 2X volume of 200-proof 100% ethanol (Koptec). Then, samples were air dried and suspended in UltraPure DNase/RNase-free distilled water (Invitrogen, Thermo Fisher Scientific).

#### Cloning of Single promoter plasmid pUC19-T7A1U, pUC19-T7A1-AmpR-mEGFP-Tandem and pUC19-T7A1-AmpR-mEGFP-Opposing constructs

Single promoter plasmid pUC19-T7A1-U was generated using around-the-horn PCR from template plasmid pUC19 using primers (CBD01/CBD02) with extensions containing the T7A1 promoter sequence as well mutations in the initially transcribed region to form a U-less cassette. These primers also contained EcoRI recognition sites for subsequent digestion and circularization by ligation. PCR products were digested with EcoRI and DpnI and were further purified by agarose-gel extraction. Purified PCR products were ligated using T4 DNA Ligase and transformed into DH5α cells. The tandem construct (pUC19-T7A1-AmpR-mEGFP-Tandem) was generated using Gibson Assembly of three PCR fragment assembly reaction: fragment 1 (PCR with template pUC19-T7A1-U using primers CBD05/06, fragment (PCR with template pUC19-T7A1-U using primers CBD07/08), and fragment 3 (PCR from plasmid containing mEGFP using primers CBD09/10). Each PCR product was purified by gel-extraction. Gibson Assembly was carried using NEB Gibson Assembly Master Mix (NEB, #E2611L) following manufacturer instructions and then assembly reaction transformed into DH5α cells. The opposing construct (pUC19-T7A1-AmpR-mEGFP-Opposing) was obtained using Golden Gate Assembly from two PCR fragments: fragment 1 (PCR with template pUC19-T7A1-AmpR-mEGFP-Tandem using primers CBD11/12) and fragment 2 (PCR with template pUC19-T7A1-U using primers CBD13/14). The PCR fragments were treated with BsaI (NEB) and BsmbI (NEB), respectively, and then ligated using T4 DNA ligase (NEB). Given the instability of this plasmid in DH5α cells, NEB^®^ Stable Competent *E. coli* cells were used for transformation instead. In all cases, plasmids from positive clones were extracted using QIAGEN miniprep kit and their sequences were confirmed by DNA sequencing.

For large scale production of plasmids, plasmids were retransformed into DH5α or NEB Stable competent cells, accordingly. Cells were grown in 1 L of LB media and extracted using QIAGEN Maxiprep kit following manufacturer instructions. For bulk biochemical as well as cryo-ET structural studies, supercoiled plasmid pUC19-T7A1-U was further purified using low-melting agarose (SeaPlaque Agarose, Lonza) from 1% agarose gel (1X TAE with 1X SYBR Safe DNA Staining). Under these electrophoretic conditions nicked DNA was well separated from supercoiled DNA. The band corresponding to supercoiled DNA was excised and extracted using β-Agarase (New England Biolabs), followed by phenol-chloroform extraction and ethanol precipitation. To desalt and concentrate the samples, the purified plasmids were further purified with Monarch PCR & DNA Cleanup Kit (New England Biolabs) using manufacturer instructions. The isolated purified plasmids were stored at −20 °C until further use.

#### Generation of different DNA topologies

To generate different DNA topologies (nicked, ΔLk=0, ΔLk < 0, and ΔLk>0) for AFM, pUC19-T7A1-U plasmids were treated with different enzymes to obtain the desired topology and they were further purified by agarose gel extraction using the β-Agarase described above. To generate nicked topology, plasmids (20 μg) were incubated in a 100 μL reaction with nicking endonuclease Nt.BspQI (20 units) in NEB 3.1 Buffer for 6 hours at 50 °C. For ΔLk = 0 topology, nicked plasmids (20 μg) were re-ligated by incubation in a 50 μL mixture with T4 DNA ligase (800 units) in T4 DNA ligase 1X Buffer at room temperature. For ΔLk < 0 topology, we used isolated plasmids with their native supercoiling degree as extracted from the stationary phase cells. For ΔLk > 0 topology, plasmids (600 fmol) were incubated in a 20 μL reaction with reverse gyrase TopR2 (0.1 μM), and ATP (1 mM) in Reverse Gyrase 1X Buffer (50 mM Tris–HCl pH 8.0, 20 mM MgCl_2_, 100 mM NaCl, 0.5 mM DTT, and 0.5 mM EDTA) for 1 hour at 75 °C. Once plasmid topologies were generated, plasmids were purified using the Monarch PCR DNA Cleanup Kit.

#### Expression and purification of *Streptococcus pyogenes* catalytically inactive dCas9 and nickase Cas9 D10A (SpCas9 D10A)

Purification of SpCas9 D10A was performed using a reported protocol.^90^ Briefly, plasmid MJ825 (Addgene, #39315) or MJ841 (Addgene, #39318) was transformed into BL21(DE3) cells. One liter of Terrific Broth culture containing 50 μg/mL kanamycin was grown at 37°C. Upon reaching OD 0.6, cells were induced with IPTG to a final concentration of 0.5 mM at 20°C and were grown overnight (~16 hours). Cells were harvested by spinning at 5000 rpm for 10 minutes and the cell pellet was stored at −80 °C. Cells were resuspended in 50 mL Lysis Buffer (50 mM Tris-HCl pH 7.5, NaCl 500 mM, 5% (v/v) glycerol, 1 mM DTT, supplemented with 4 tablets of mini-EDTA free protease inhibitor) and lysed using a sonicator. The lysate was centrifuged for 30 minutes at 4°C at 20,000 g. The clarified supernatant was passed through a HisTrap 5 mL column, washed with buffer NiA (50 mM Tris-HCl pH 7.5, NaCl 500 mM, 5% (v/v) glycerol, 1 mM DTT, 10 mM imidazole) and eluted on a linear gradient with buffer NiB (50 mM Tris-HCl pH 7.5, NaCl 500 mM, 5% (v/v) glycerol, 1 mM DTT, 300 mM imidazole). Positive fractions containing SpCas9 D10A (verified by SDS-PAGE) were combined, and 2 mL of TEV protease were added and dialyzed overnight on Dialysis buffer (50 mM Tris-HCl pH 7.5, NaCl 500 mM, 5% (v/v) glycerol, 1 mM DTT). The samples were further purified on HiTrap Heparin column using a linear gradient of KCl from 200 mM to 1000 mM (50 mM Tris-HCl pH 7.5, 5% (v/v) glycerol, 1 mM DTT), followed by size-exclusion chromatography on a Superdex S300 column using Gel Filtration buffer (20 mM Tris-HCl pH 7.5, KCl 200 mM, 5% (v/v) glycerol, 1 mM DTT).

#### Expression and purification of *S*. solfataricus reverse gyrase (TopR2)

The gene fragment expressing the reverse gyrase (TopR2) was obtained by PCR from *Sulfolobus solfataricus* genomic DNA (ATCC, 35092D-5). This fragment was inserted into an expression plasmid with His6 N-terminal tag with a TEV protease site (2Bc-T, Addgene #37236) using ligation independent cloning. Protein expression was carried with this plasmid (2Bc-T-TopR2) following a reported protocol.^91^ Briefly, plasmid 2Bc-T-TopR2 was transformed into Rosetta (DE3) cells. One liter of 2xYT culture containing 100 μg/mL ampicillin, 34 μg/mL chloramphenicol, and 1% glucose was grown at 37°C. Upon reaching OD 0.5, cells were induced with IPTG to a final concentration of 1 mM at 37°C and were grown for 6 hours. Cells were harvested by spinning at 5000 rpm for 10 min and the cell pellet was stored at −80°C. The pellets of induced cells were resuspended in Lysis buffer (40 mM Tris–HCl pH 8.0, 100 mM NaCl, 1 mM DTT, 0.1 mM EDTA). After sonication on ice, the sample was centrifuged at 30,000 g for 30 minutes. The resulting supernatant underwent heat treatment at 75°C for 13 minutes and was clarified by centrifugation at 30,000 g for 20 minutes. Polyethylenimine was then added to achieve a final concentration of 0.3%, and the mixture was stirred for 1 hour before being centrifuged at 40,000 g for 30 minutes. The supernatant was adjusted to 70% (NH_4_)_2_SO_4_ saturation, stirred for 30 minutes at 4°C, and centrifuged at 20,000 g for 30 minutes. The proteins were dissolved in buffer B (Lysis Buffer with 200 mM NaCl and 10% (v/v) ethylene glycol). After dialysis against buffer B, the proteins were loaded onto a 5 mL HiTrap Heparin HP column pre-equilibrated with buffer B using an FPLC AKTA system (GE Healthcare). The column was washed with 50 mL of buffer B, and bound proteins were eluted with a NaCl gradient ranging from 0.2 to 1.2 M NaCl in buffer B.

#### SpCas9 D10A nicking assay

To test the activity of the sgRNA and nickase dCas9, a nicking assay was developed to monitor activity by electrophoretic mobility of DNA in agarose gel. sgRNA (90 fmol) was incubated at room temperature for 5 min with SpCas9 D10A (90 fmol) in Cas9 Reaction Buffer (20 mM HEPES pH 6.5, 100 mM NaCl, 5 mM MgCl_2_, 0.1 mM EDTA). Supercoiled plasmid (9 fmol) was added, and the reaction was kept at 37°C for one hour. The samples were treated with Proteinase K and then loaded into an agarose gel (1% agarose, 1X TAE 2.5 μg/mL chloroquine).

#### DNA topoisomerase relaxation assay

The DNA relaxation activity of *E. coli* DNA topoisomerase I on supercoiled pUC19-T7A1U was tested under different reaction conditions. In all reaction conditions, a total reaction volume of ~40 μL containing ~200 ng (~ 0.157 pmol) of negatively supercoiled plasmid was used and relaxation was carried out in TB40 buffer (20 mM Tris pH 8.0, 40 mM KCl, 5 mM MgCl_2_, 0.02 mg/mL BSA, and 1 mM DTT). An incubation at 37°C for 20 minutes was done for all conditions prior to the addition of *E. coli* Topoisomerase I (New England Biolabs, M0301S, 0.720 pmol). (1) For Topoisomerase I only condition, topoisomerase was added after an incubation of 37°C for 20 minutes. (2) To test the effect of a stalled transcription complex, *E.coli* RNA Polymerase Holoenzyme (New England Biolabs, M0551S, 1.700 pmol) was supplemented to the previous reaction condition with 10 μM of ATP, GTP, CTP (Thermo Scientific, R0481) and 50 μM of GpA dinucleotide (TriLink Biotechnologies) and incubated during 20 min at 37°C. (3) To test for the effect of a dCas9: sgRNA complex, a sgRNA:dCas9 complex at a concentration of 1.55 μM was first formed by incubating 15.5 pmoles of sgRNA and 15.5 pmoles of dCas9 in TB40 buffer (10 μL) at 25°C for 10 minutes. Afterward, the sgRNA: dCas9 complex was incubated with the supercoiled plasmids for 20 minutes at 37 °C. (4) To test the effect of *E.coli* RNAP without DNA-melting, *E.coli* RNAP core (NEB, 1.7 pmol) was supplemented to the plasmid-only condition and incubated at 37°C incubation prior to the addition of topoisomerase. (5) To test the effect DNA binding protein that does not melt DNA, dEcoRI (EcoRI Q111, 2 pmol, a gift from ModrichLab) was supplemented to the plasmid-only condition and incubated at the 37°C incubation prior to the addition of topoisomerase.

In each assay, after 20 minutes of incubation, *E. coli* Topoisomerase I (NEB, 0.72 pmol) or human topoisomerase (hTop1B, 4U,Topogen) was added to a total reaction volume of 40 μL and time-points were taken at 0, 1, 2, 3, 5, 10, 15, and 30 minutes and quenched in a 100 μL of a Stop Buffer containing 8 U Proteinase K (NEB), 4 μL Glycogen (5 mg/mL, Invitrogen), and 86 μL of 2X PK buffer (Tris 200 mM pH 7.5, EDTA 25 mM, NaCl 300 mM, SDS 2%). The timepoints were extracted with phenol (pH > 7.5) and phenol/chloroform/isoamyl alcohol (25:24:1 Mixture, pH 6.7/8.0, FisherBioReagents^™^) and then precipitated with 70% ethanol and redissolved in EB buffer. The time points were electrophoresed using a 1.5% (w/v) agarose gel that was run at 80V for 3 h with 1X TAE buffer (40 mM Tris-acetate, pH 8.1, 2 mM EDTA) in ice and post-stained in a solution of SYBR Safe in water before being visualized using Typhoon imager.

#### 2D agarose gel electrophoresis

##### DNA topoisomer ladder generation

Plasmid pUC19-T7A1U was nicked with Nt.BspQI (New England Biolabs). Nicked pUC19-T7A1U plasmids (400 ng) were religated in 1X T4 DNA ligase buffer with increasing amounts of ethidium bromide (0, 40, 80, 120, 160, 200, 400, and 800 ng) using 100 U of T4 DNA ligase (New England Biolabs) in 40 μL reactions. The reaction proceeded overnight at room temperature and was quenched with Proteinase K (NEB), followed by extraction using 2-butanol, phenol-chloroform extraction, and ethanol precipitation. A mixture of the different ligation reactions was made to generate the 2D topoisomer ladder.

##### Separation of DNA topoisomers in 2D agarose gel

A large 2% agarose slab was cast in 1X TAE containing 2.5 μg/mL chloroquine. 100 ng of plasmid was loaded on a narrow lane in the 2D gel and electrophoresis was carried out with the following conditions: 1) For the first dimension: 40V (22 hours) in 1X TAE 2.5 μg/mL chloroquine and 2) For the second dimension: 60V (8 hours) in 1X TAE 25 μg/mL chloroquine. The gel was then stained using SYBR Safe before imaging on Typhoon FLA 9500.

#### *In vitro* Transcription assay

To test the effect of DNA supercoiling, soft-torsional block by dCas9 and Topoisomerase I in single-round transcription elongation, we performed experiments using radiolabeled RNA. Stalled elongation complexes labeled with radiolabeled nascent RNA were formed by assembling *E.coli* RNA polymerase Holoenzyme (10 pmol, New England Biolabs) in pUC19-T7A1U (1 pmol) TB40 buffer (20 mM Tris pH 8.0, 40 mM KCl, 5 mM MgCl_2_, 0.02 mg/mL BSA, and 1 mM DTT) in the presence of 10 μM of ATP, GTP, CTP (Thermo Scientific, R0481) and 50 μM of GpA dinucleotide (TriLink Biotechnologies) and α-P32-ATP (Perkin Elmer). The reactions were incubated for 20 min at 37 °C and then placed on ice. The excess of radiolabeled nucleotides was removed using a size-exclusion column Microspin G-25 (GE Healthcare). The stalled complexes were reinitiated by adding NTPs at 10 μM and reaction allowed to proceed at room temperature. Time points were quenched using 2X RNA formamide loading dye and taken at 0, 20, 40, 60, 80, 100, 120, 180, 240, 300, 450, 600, 750, 900, 1050, 1200, 1350, 1500, 1650, 1800 s. Quenched samples were ran in 10% denaturing urea gel 19:1 acrylamide/bisacrylamide, the resolved RNA were imaged on Typhoon FLA 9500 and the bands were quantified with ImageQuant TL 8.2.

The reactions were performed using nicked and supercoiled DNA templates of single promoter pUC19-T7A1U. For conditions where dCas9 was used, dCas9:sgRNA targeting TTS (CBR01) were incubated at 10 min after addition of *E. coli* RNAP Holoenzyme. When Topoisomerase I was used, *E. coli* Topoisomerase I was added 10 min after addition of *E coli* RNAP and allowed to relax DNA before isolating stalled elongation complexes. The abortive initiation measurements in Figure 2D were based on autoradiographed RNA intensities at time point 0 for both nicked and –sc pUC19-T7A1U templates. As can be seen from these experiments, more efficient loading and stalling of RNAP was observed on –sc DNA templates than nicked after removal of the excess radiolabeled nucleotides (i.e.: higher absolute amount was observed at 19 nt stalled site for the –sc condition than the nicked condition). To account for this, gel intensities were scaled accordingly in Figure 3E, and lane-normalization was used for quantification accordingly (See below in Gel quantification).

#### Real-time transcription assays using a molecular beacon

To measure RNA synthesis in real time for both AmpR and mEGFP gene from the opposing and tandem constructs, we adapted a fluorescence-based methodology using molecular beacons that possess a 2′-*O*-methylribonucleotide backbone.^63^ Molecular beacons CBMB01 and CBMB02 targeting 3’ RNA ends of AmpR and mEGFP transcripts were used in separate reactions. Briefly, each reaction was performed in TB40 buffer (20 mM Tris pH 8.0, 40 mM KCl, 5 mM MgCl_2_, 0.02 mg/mL BSA, and 1 mM DTT) in the presence of 1 mM of ATP, GTP, CTP, UTP (Thermo Scientific, R0481), 50 μM of GpA dinucleotide (TriLink Biotechnologies), 8 nM of plasmid, 85 nM of *E.coli* RNAP Holoenzyme (NEB), and 500 nM of molecular beacon. To ensure that all of the transcription reactions started simultaneously, the reaction mixtures were prepared on an ice-cold metal block. Then, all transcription reactions were incubated simultaneously in an iQ5 Thermal Cycler for 2 hours at 37°C. During the reaction, the fluorescence intensity FAM of the molecular beacon was recorded every 20 s.

#### Measurement of GFP fluorescence emission in *E. coli* MG1655 with tandem and opposing constructs

The following experiments were performed using similar experimental conditions as reported by Kim et al.^24^ Briefly, plasmids for the tandem and opposing constructs (pUC19-T7A1-AmpR-mEGFP-Tandem and pUC19-T7A1-AmpR-mEGFP-Tandem) were transformed into *E.coli* MG1655 cells using standard electroporation protocols. Positive clones were selected by their growth on an ampicillin antibiotic plate and by their fluorescence emission in a trans-illuminator under blue light. Three colonies from each construct were selected, mixed and a ~ 5 mL inoculate grown in M9GluCAAT media containing M9 minimal medium (6 g/L Na_2_HPO_4_, 3 g/L KH_2_PO_4_, 0.5 g/L NaCl, 1 g/L NH_4_Cl, 2 mM MgSO_4_, 0.1 mM CaCl_2_) supplemented with 0.2% glucose, 0.1% casamino acids (Difco Laboratories),1 mg/L thiamine and ampicillin 50 μg/mL at 37 °C. The overnight inoculate was then diluted 10,000 fold and then grown to exponential phase in M9GluCAAT with ampicillin 50 μg/mL to an optical density OD_600_=0.2. Then, ~0.5 μL of these cells were spotted into an agarose pad made with 1% agar and M9GluCAAT covered with a no. 1.5 coverslip, then imaged in the microscope at room temperature. Imaging by phase contrast and fluorescence microscopy was performed on a Nikon Eclipse Ti microscope equipped with either a 1.40 NA phase-contrast oil objective, a Hamamatsu Orca-Flash4.0 V2 CMOS camera, a Sola Light Engine (Lumencor) and pE-4000 (CoolLED) light source. For GFP excitation/emission the ET470/40x Chroma dichroic was used. The microscopes were controlled by the Nikon Elements software.

#### Atomic Force Microscopy

##### Slow scan Atomic Force Microscopy (AFM)

DNA samples of different topologies of pUC19-T7A1U (1–2 nM) were diluted in 10 mM HEPES pH 7.0 and 5 mM MgCl_2_ prior to sample deposition. Two microliters of DNA were deposited on freshly cleaved mica and incubated at room temperature for 2 or 3 minutes. Mica was rinsed with 50 μL of water five times and dried under N2 gas flow. AFM measurements were performed with a Multimode AFM Nanoscope 8 (Bruker Co.). The samples were imaged in tapping mode; the silicon cantilevers (Nanosensors) were excited at their resonance frequency (280–350 kHz) with free amplitudes of 2–10 nm. The image amplitude (set point As) and free amplitude (A0) ratio (As/A0) was kept at 0.8. All samples were imaged at room temperature in air, at a relative humidity of 30%. Raw static AFM images acquired in air were flattened and leveled using Gwyddion 2.5. A mask of the entire DNA molecule using thresholding was obtained and only pixels corresponding to DNA regions shown in Figure 1C.

#### Cryo-ET sample preparation for transcriptional modulation of DNA supercoiling

##### Preparation of circular plasmid under low salt and high salt conditions

For the preliminary investigation into the conformational dynamics of DNA supercoiling in response to varying ionic strength, the above extracted and purified pUC19-T7A1U plasmid was diluted to a concentration of 100 nM under two distinct buffer conditions: low salt and high salt buffer (20 mM Tris-Cl, 0.5 mM DTT, and 2% Trehalose; 20 mM Tris-Cl, 40 mM KCl, 5 mM MgCl_2_, 0.5 mM DTT, and 2% Trehalose, respectively) preceding the application onto the EM grids.

##### Formation of RNAP-pUC19-T7A1U stalled complex

To investigate the localization of RNAP on the plasmid, a 10 μL incubation reaction was prepared to induce RNAP stalling 19 nucleotides downstream from the TSS of the T7A1 promoter. This was achieved by employing UTP starvation in a system composed of 60 nM pUC19-T7A1U plasmid, 180 nM RNAP (3:1 ratio to the plasmid), 50 μM GpA dinucleotide primer (facilitating RNAP initiation), and 10 μM rNTP mix (excluding UTP). The reaction buffer consisted of 20 mM Tris-Cl, 40 mM KCl, 5 mM MgCl_2_, 0.5 mM DTT, and 2% Trehalose. This reaction was then allowed to incubate for 20 minutes at 37°C to create the RNAP stall complex before application onto the TEM grids.

##### Formation of dCas9-pUC19-T7A1U complex

Following the same incubation protocol as described above, the RNAP in the system was replaced by dCas9, an alternative DNA-binding protein. To do so, the dCas9 protein and its guiding RNA (sgRNA) were first assembled in a separate vial at a 1:1 ratio, reaching a concentration of 500 nM. This assembly was achieved by incubating the dCas9-sgRNA mixture in a buffer containing 20 mM Tris-Cl, 40 mM KCl, 5 mM MgCl_2_, and 0.5 mM DTT for 10 minutes at room temperature. Subsequently, the incubated dCas9-sgRNA complex was introduced into the plasmid, yielding a system containing 60 nM pUC19-T7A1U, 120 nM dCas9-sgRNA (2:1 ratio to the plasmid), a GpA dinucleotide primer (50 μM) for initiation, and an rNTP mix (10 μM, excluding UTP). The reaction buffer composition remained consistent with 20 mM Tris-Cl, 40 mM KCl, 5 mM MgCl_2_, 0.5 mM DTT, and 2% Trehalose. This reaction mixture was further incubated for 20 minutes at 37°C before being applied to the TEM grids for further analysis.

##### Formation of transcribed (none-equilibrium) RNAP-pUC19-T7A1U complex

To study the activation of RNAP transcription on the negatively supercoiled plasmid, the RNAP stalled complex was first prepared as described above. At the end of its 20-minute incubation at 37°C, rNTP (including UTP) was added to the solution, adjusting the concentration of each nucleotide to reach 100 μM. The transcription reaction continued for 10 minutes at room temperature before being transferred onto ice prior to application onto the TEM grids. For single-particle analysis, the transcription elongation complex concentration (both RNAP and plasmid) was scaled up 4-fold without changing the buffer conditions.

##### Formation of stalled and transcribed RNAP-dCas9-pUC19-T7A1U complexes

To study active RNAP transcription on the negatively supercoiled plasmid in the presence of dCas9 torsional block, the previous dCas9-pUC19-T7A1U complex was first prepared. Subsequently, RNAP was introduced into the incubated solution at a 3:1:1 ratio to dCas9 and plasmid, with concentrations of 180 nM, 60 nM, and 60 nM, respectively, maintained under the same physiological salt condition. In this sequence, the addition of rNTP (10 μM final concentration, excluding UTP) allowed the accumulation of mild torsion as RNAP initiated transcription from the TSS to the U-less stalling site, spanning 19 nucleotides. To induce additional torsional stress in the system, rNTP (including UTP) was added to the stalled RNAP-dCas9-pUC19-T7A1U complex at 100 μM final concentration. The sample was subjected to a 10 minutes incubation at room temperature for DNA transcription before being transferred to the EM grids.

##### Formation of transcribed RNAP-topI-pUC19-T7A1U complexes

To relieve RNAP from its apical constraint, topoisomerase I was introduced into the transcription system. The stalled RNAP-pUC19-T7A1U complex was initially prepared following the same established protocol. After a 20-minute incubation at 37°C, topoisomerase I was added to the solution at a ratio of 3:1:1 with respect to RNAP and plasmid at concentrations of 180 nM, 60 nM, and 60 nM, respectively, under the same physiological salt condition. Subsequently, rNTP (including UTP) was added to the solution mixture, reaching a final concentration of 100 μM, and the transcription persisted for 10 minutes at room temperature before application onto the EM grids.

##### Formation of transcribed RNAP-pUC19-T7A1U dual-promoter complexes

To study RNAP transcription activation on negatively supercoiled plasmids containing dual promoters (i.e., opposing and tandem constructs), RNAP-stalled complexes were prepared using the same procedure as described above for the single-promoter pUC19-T7A1U template, with the exception that the RNAP concentration was doubled to 360 nM (corresponding to a 6:1 molar ratio of RNAP to plasmid) to accommodate dual-promoter initiation. Following a 20-minute incubation at 37 °C, rNTPs (including UTP) were added to a final concentration of 100 μM for each nucleotide. The transcription reaction was allowed to proceed for 10 minutes at room temperature and then placed on ice prior to application onto TEM grids.

#### TEM specimen preparation

3 μL of a sample after the above incubation was deposited onto a glow-discharged (PELCO easiGlow^™^ Glow Discharge Cleaning System) 200 mesh Quantifoil gold grid (hole size ranging from 1 micron to 2 microns, Electron Microscopy Sciences) for 30 seconds. Following a 20-second on-grid incubation, the grid was plunge-frozen in liquid ethane at ~95% humidity and 15°C using a Leica EM GP rapid-plunging device (Leica, Buffalo Grove, IL, USA) after controlled blotting with filter paper (3–5 s). The resulting flash-frozen grids were then transferred into liquid nitrogen for storage.

#### TEM data acquisition

The Cryo-EM data were acquired using a Titan Krios (FEI) transmission electron microscope operating at 300 kV high tension and equipped with a Gatan energy filter. Imaging was performed with a Gatan K3 Summit direct electron detection camera, employing a magnification of x53,000 (where each pixel of the micrographs corresponds to 1.67 Å in specimens) in super-resolution and correlated double sampling (CDS) mode. Tilt series of the samples were captured from −51° to +51° at 3° step or −55° to +55° at 5° step increments using a dose symmetry scheme. Data collection was automated using SerialEM software^78^ to track the specimen and maintain a defocus of ~2.5 μm. The total dose for the tilt image series ranged from ~110–150 e^−^/Å^2^. At each tilt angle, a total of 8 frames were collected, with an exposure time of 0.25 s per frame. Single-particle movie data were collected using a Gatan K3 direct electron detector at a nominal magnification of ×81,000, corresponding to a pixel size of 1.05 Å. Each exposure lasted 7.39 seconds and was recorded over 50 frames, with a total electron dose of 50 e^−^/Å^2^. Micrographs were acquired in non-CDS and super-resolution modes, with a defocus range of 0.6–1.6 μm, using SerialEM.

#### Cryo-ET tilt series processing and 3D reconstruction

The beam-induced motion of cryo-EM frames was corrected using MotionCor2.^79^ To improve the contrast of low-dose cryo-ET tilt series images, a deep learning-based denoising method (NOISE2NOISE^92^) was implemented, involving the division of motion-corrected movie frames into even and odd halves for network training and subsequent particle resolution estimation (Figure S8A). Predictions for even and odd frames for a single tilt were averaged together. The defocus value of the cryo-ET tilt series was determined using GCTF.^93^ A carbon area perpendicular to the tilt axis was included during data collection to assist in contrast transfer function (CTF) detection and later tilt series alignment. The tilt series were initially aligned using IMOD with patch tracking function^77^ and subsequently imported into e2tomo software^28^ for 3D reconstruction (Figure S8B). Approximately 100 subtomogram patches containing DNA features were cropped for manual annotation and subsequently submitted for training. This convolutional neural network (CNN) was then applied across all tomograms for DNA identification (Figure S8C).

#### Individual particle 3D reconstruction refinement and modeling

After the deep learning-based DNA annotation, individual plasmid particles were manually labeled in the tomogram (Figure S8C, **right**). In brief, the DNA-annotated tomograms were binned by 4 (6.68 Apix) and divided into isolated small surface pieces within Chimera.^80^ Density segments from a single plasmid particle were then manually selected and grouped, followed by low-pass filtering to a 6 nm resolution to serve as the particle-shape mask. The criteria for selecting plasmid particles from the annotated tomogram included: 1) the particle should display supercoiling, manifesting as a helical structure (cyan color) without nicking (red color) (Figure S8C, **right**), 2) plasmid particles should be isolated and not significantly entangled with others; and 3) particles not partially attached to the carbon area. The center of mass of the particle shape mask was calculated (Figure S8D, **left**), and this information was used to crop 1400×1400 pixel local tilt series within the raw large micrograph before denoising. This cropped tilt series, with a plasmid particle in the center, was binned by 7 (a larger crop area and bin=8 were used for the later opposing and tandem construct plasmid particles for their larger size) and then submitted for local 3D reconstruct with EMAN2.^28^ To minimize artifacts caused by the limited tilt angle range, the 3D reconstructed maps were missing-wedge compensated using IsoNet software^29^ (Figure S8E), employing the previously created low-resolution mask. The output map was then low-pass filtered to 2 nm, serving as the final map for the modeling.

Plasmid modeling was facilitated through the following steps: from the 3D reconstructed maps of the plasmid particle, a spine line representing the particle's superhelical axis was initially computed (Figure S9, **first row, left**). This involved applying a strong Gaussian kernel to the map, followed by skeletonization using the 'lee' method from the scikit-image package. Along the determined spine line, a sampling cylinder with dimensions of 60 nm diameter and 15 nm height was generated, utilizing a 5 nm step. The DNA density within the cylinder was divided into two regions, and the weight centers for each region were recorded (Figure S9, **first row, right**). Subsequently, the tracing centers were interconnected based on rotation and distance to the previous centers, with a subsequent round of manual correction undertaken to address misconnection cases, particularly in low-quality areas of the EM map (Figure S9, **second row, left**). The resulting threaded points underwent final processing through smoothing and interpolation with a 2 nm spatial interval, yielding the final model for the 3D map of the supercoiled DNA (Figure S9, **second row, right**).

#### Sub-tomogram averaging of RNAP and dCas9

The sub-tomogram averaging of RNAP and dCas9 particles was carried out utilizing the EMAN2 e2tomo. Briefly, the CTF-corrected and imod-aligned raw tilt series were imported into the software, followed by e2tomo 3D reconstruction, yielding a 4×-binned tomogram (equivalent to 6.68 Å with an unbinned pixel size of 1.67 Å). Subsequently, approximately 100 particles for each protein type were manually selected, and ab-initio maps were reconstructed for the subsequent round of reference-based boxing. Employing a template matching threshold value (vthr) of 7.5, a total of 1781 and 875 particles were extracted for RNAP and dCas9, respectively. Particle cropping, alignment, and averaging procedures were executed using the e2spt_refine_new.py script. No symmetry was applied during particle alignment. Following 8 rounds of alignment (iters=p,p,p,t,p,p,t,r), resolutions of 14.1 Å and 17.6 Å were determined for RNAP and dCas9, respectively (0.143 cut-off), employing Fourier Shell Correlation (FSC) with odd and even particles from the masked, final average. By mapping the orientation-determined particles back to their tomogram using e2spt_mapptclstotomo.py, DNA-bound particles were selectively chosen if their center of mass was within a distance of <1 nm to any points of the plasmid model. A subset of 232 RNAP and 116 dCas9 particles was then subjected to another round refinement (iters=p,p,p,t,p), resulting in resolutions of 17.7 Å and 18.9 Å, respectively, evaluated under the same standards.

#### Single-particle analysis of TECs on negatively supercoiled DNA template

Single-particle 3D reconstruction was performed using cryoSPARC.^83^ The detailed data processing workflow for TECs on negatively supercoiled DNA templates is shown in Figure S4A. A total of 10,476 micrographs underwent patch motion correction, patch CTF estimation, and particle picking, yielding 2,063,152 particles. Initial 2D classification and ab initio reconstruction revealed moderate orientation bias of RNAP particles. To preserve rare views, particle cleanup was performed via three rounds of heterogeneous refinement against 6 models—5 representing noisy junk references and one low-pass-filtered good model from the initial 3D reconstruction—instead of standard 2D classification. This cleanup yielded 611,747 particles (binned 3, in a 108-pixel box), which were used for homogeneous refinement, achieving a 6.5 Å reconstruction. Subsequent 3D classification at 15 Å resolution separated two major classes: TIC (186,816 particles, with σ-factor bound) and TEC (424,931 particles, without σ). The TEC particles were re-boxed (unbinned, in a 324-pixel box) and subjected to ab initio reconstruction, non-uniform refinement, and orientation rebalancing using cryoSPARC’s default settings. Further 3D classification at 10 Å resolution of TEC particles yielded three classes: Class I (TEC1–SC) and Class II (TEC2–SC), both showing DNA in the RNAP active site and a downstream DNA density protrusion, and Class III, which showed only faint DNA density and was not analyzed further. Non-uniform and local refinement of TEC1–SC (46,743 particles) and TEC2–SC (54,869 particles) yielded final structures at 2.9 Å resolution. Maps were post-processed with DeepEMhancer.^86^ Initial models were built by fitting domains from known TEC structures (e.g., PDB: 6ALH) into the density maps using ChimeraX.^81^ Models were iteratively refined using phenix.real_space_refine,^88^ manually adjusted in ISOLDE,^87^ and validated with MolProbity^89^ (Table S1). The swivel angle calculation for TEC1–SC and TEC2–SC followed the definition of the core and swivel modules as described by Kang et al.,^52^ and using PDB structure 6RH3 as the reference. Additional structures (1:6RH3, 2:8EG8,4:6ALH, 5:8EHI, 7:8EHF, and 8:7PYK) were also included in the measurement as control. Measurements were performed in ChimeraX, and the ChimeraX script is available in the Zenodo repository.

#### Plectoneme apical site sequence-based prediction

Prediction of DNA plectoneme formation loci along the pUC19-T7A1U sequence was performed using a computational model developed by Kim et al.^43^ To account for the circular topology of the plasmid while matching the required linear DNA input format, the pUC19-T7A1U sequence was duplicated three times and joined head-to-tail to simulate continuity; only the central region corresponding to the original plasmid was plotted to eliminate edge artifacts. To provide a reference for interpreting the plectoneme formation scores, a control sequence consisting of polyA–Widom 601–polyA was used. This construct includes the well-characterized Widom 601 nucleosome positioning element, flanked by polyadenine tracts, and is known for its high intrinsic bendability, which provides a comparative baseline for sequence-driven plectoneme localization.

#### Construction of superimposed models and mapping RNAP position on plasmid

To align all models from the same experimental condition, orientation-determined particles within the same group were back-mapped to their respective tomograms. Concurrently, the plasmid model was also back-mapped to the tomogram, so that it allows the determination of the relative orientation between the plasmid and plasmid-bound particles (RNAP and dCas9). Given the current resolution limitations of cryo-ET in resolving DNA sequence information, the position of dCas9 was utilized as a marker to infer the TSS, assuming specificity in dCas9 binding as per the construct design. The entire length of the plasmid trace was normalized to 1950 base pairs, with the point nearest to the dCas9 particle designated as 1090 bp. As none of the particles are symmetrical, the upstream and downstream positions of the bound RNAP were determined relative to the plasmid.

#### Coarse-grained simulation of DNA supercoiling relaxation

Coarse-grained molecular dynamics (CGMD) simulations were conducted using the GPU-accelerated, sequence-dependent oxDNA2 model.^42,94^ The simulation environment was set up under the canonical NVT ensemble (constant number of particles, volume, and temperature). To enhance computational efficiency, the 525-bp minicircle DNA system were employed. The in-silico plasmid model was generated using TacoxDNA,^85^ with a DNA twist deficit of −5 and a writhe of 0, yielding a supercoiling density of −0.1. The circular DNA plasmid was energy minimized and relaxed using parameters tailored from oxDNA example simulations (at dna.physics.ox.ac.uk). These parameters included the following (thermostat = john, interaction_type = DNA2, newtonian_steps = 103, diff_coeff = 2.5, salt_concentration = 0.5, T = 300K, dt = 0.005, verlet_skin = 0.05, rcut = 2.0).

To study the impact of varying levels of plectoneme apex constraint on DNA supercoiling structural dynamics, simulations were initiated by introducing a small bulge on the minicircle DNA (Figure S3A, **left**), which facilitated apex formation at the target site during relaxation. The DNA base pairs at the two distal ends of the bulge were subjected to different levels of constraint, implemented as a mutual force trap between the two red sites (Figure S3A, **dashed line box**), with spring stiffness set to 1 and spring equilibrium distances (r0) set to 8.0 nm, 6.4 nm (80%), and 4.8 nm (60%). A fully unconstrained condition was also included. For each condition, the plasmid was first energy minimized for 2 × 10^6^ steps, followed by relaxation for 3 × 10^7^ steps (equivalent to 300 μs), with a frame sampling interval of 5 × 10^4^ steps, yielding 600 frames. The final 500 frame structures after plasmid relaxation and apices formation were used to compute the global writhe distribution and plectoneme apex curvature statistics.

To study the effect of plectoneme apex constraint on DNA supercoiling torsional relaxation, simulations were initiated by relaxing the minicircle for 3×10^6^ steps (30 μs) until the formation of plectonemes and the appearance of apices. The final frame structure was edited to create a nicked plasmid by introducing a single-strand break at base pair index 940 for models I and II, 828 for model III, and 564 for model IV (Figure S5C). This nicked configuration was designed to mimic the activity of Topoisomerase I (TopI). Four DNA supercoiling torsional relaxation simulations were conducted: For model I, the nicked plasmid was allowed to relax without any external constraints for 3×10^7^ steps (equivalent to 300 μs). For the model II, III, and IV, a mutual force trap was applied to constrain the sharp apex region, covering approximately 20 base pairs. These traps, functioning as springs, were formed between base pairs at indices 798, 251 on one side and 817 and 232 on the other. The spring stiffness was set to 1, and r0 was defined as the initial distance between the mass centers of each pair of base pairs in the initial structure divided by a factor of 2. The second batch of simulations were also run 3×10^7^ steps. All simulations were repeated two times using the same relaxation parameters mentioned above. Movies were generated using the last run of each simulation at a rate of 1×10^5^ steps per frame.

The equilibrated final frame structure prior to the nick was also utilized to simulate the formation of DNA twin domains. In brief, two rotating harmonic traps were applied to the base pairs at indices 107 and 942, as well as indices 366 and 683, positioned within the intermediate region of the plectoneme. This approach segments plectoneme two sections: the first section contains an apex with constraints, while the second section features a free apex. This setup allows for a comparison of the effects of (+) torsional propagation on one strand, (−) torsional propagation on the other, and their mutual cancellation when the rotating trap is applied. The center of rotation was defined as the center of mass of the selected base pair, with the rotation axis determined as the unit vector extending from the center of the base pair to the center of mass of another base pair located 10 base pairs upstream. The stiffness of the trap was set to 10 in oxDNA, with a rotation rate of 1×10^5^. The confined apex was configured as described earlier. The simulation was run for 3×10^6^ steps and with one repeat. Movies were created with frames captured every 1×10^4^ steps.

### QUANTIFICATION AND STATISTICAL ANALYSIS

#### Gel quantification

The gel images were initially adjusted using the GIMP cage transformation function to correct bending along the horizontal direction, facilitating subsequent auto-processing steps. The quantification of gel intensity was facilitated by using the Python script gel_lane_finder script,^95^ which enable the automated annotation of gel lanes. The two-dimensional band was averaged in the horizontal direction, resulting in a one-dimensional array representing the pixel-wise intensity along the annotated gel lane. To standardize the intensity values, which may be influenced by variations in sample loading for each lane, the sum of pixel intensity in the one-dimensional array within each lane was employed to normalize the intensity at each pixel point. Specific representative pausing positions were traced across the temporal evolution of the transcription. For supercoiling relaxation experiments, the presence of a small fraction of pre-existing nicked species in the negatively supercoiled DNA template (i.e., the top band at time zero, ΔLk = 0) may affect the interpretation of the results. Therefore, the ΔLk = 0 lane was excluded from the analysis. This gel quantification workflow was applied to Figures 3F, 4G, 5A, 5I–J, S5A–B, and S7B–D.

#### Evaluation of the cryo-ET 3D reconstruction resolution

The resolution for the individual particle reconstructions were estimated by two methods. 1) Data-to-Data based analysis: the Fourier Shell Correlation (FSC) was calculated between two independently reconstructed 3D maps, in which each map was based on one-half of the tilt-series (split by even and odd frames for each tilt). The frequencies at which the FSC curve first falls to values of 0.143 were used to represent the reconstruction resolution. 2) Data-to-Model based analysis: the FSC curve between the final 3D reconstruction and the density map converted from the corresponding fitting model was calculated. The frequencies at which the FSC curve fell below 0.5 was used to estimate the resolution. The density map of the fitting model was generated by pdb2mrc in EMAN software.^96^ FSC evaluation results are provided for each particle in Table S2.

#### Quantification and statistical analysis of supercoiled DNA plasmid morphology

The quantification of the supercoiled DNA plasmid's morphology involved measurements of radius of gyration, global writhe number, and plectoneme branch writhe density. To quantify these characteristics, the eigenvalues of the gyration tensor were calculated and designated as r1, r2, and r3, with the relationship r1 > r2 > r3. The radius of gyration (R) was determined by the formula R = r1^2^ + r2^2^ + r3^2^. The calculation of the writhe number was facilitated by the E-CAM polymer_data_analysis module, which implements a double integral computation method proposed by Klenin & Langowski.^97^ Violin plots were generated to represent data distributions with a kernel density estimation. The centered white dot indicates the median, the thick black bar represents the interquartile range (IQR; 25%–75% percentiles), and the thin black lines (whiskers) extend to 1.5× IQR. Statistical significance was assessed using a Mann–Whitney test, with p < 0.05 considered significant. Statistical details for each experiment are provided in Figures 6F, S1C, S1D, S2B, S2F, S3E, S6A, and S6F.

#### Quantification and statistical analysis of the curvature and apexes along the supercoiled DNA

To determine the curvature of the plasmid along its DNA trajectory, a loop iterated from index 1 to N-1 for each point on the DNA plasmid model. The curvature (κ) at each point (i) was computed with its two neighboring points (i-1 and i+1) using the formula κ = 1/R = 4S/fgh, where R is the radius of the circle circumscribing a triangle formed by the three points. Here, S represents the area of the triangle, and f, g, and h are the side lengths opposite the vertices of the triangle. The curvature at each point along the DNA trajectory was color-coded using a gradient, with points exhibiting the largest curvature values near the distal ends of each plectoneme branch utilized as the apexes of the plasmid. The same violin plot representation and statistical analysis were used as for the quantification of supercoiled DNA plasmid morphology. Statistical details for each experiment are provided in Figures S1G, S2B, S2F, S3E, S6A, and S6F.

#### Quantification and statistical analysis of the distance between DNA along the plasmid's helical axis

To calculate the distance between DNA along the supercoiling helical axis, the points on the DNA plasmid model were sequentially sorted and segmented into different color groups using the apical points on the plasmid (Figure S1E). For each point (i) in a group, its nearest neighboring point in another color group was determined, and the center of mass as well as the distance between the two points were calculated. This calculation was repeated from point 1 to N for each segment, resulting in a list of centers and distances that were used to represent writhe helix axis and the distances between the DNA along the supercoiling helix (Figure S3F, **II**). Given the difficulty of defining the start and end site for a circular plasmid, the apexes were utilized as the zero index to align the calculated distance list. The same violin plot representation and statistical analysis were used as for the quantification of supercoiled DNA plasmid morphology. Statistical details for each experiment are provided in Figures S2F, S3E, S6A, and S6F.

#### Quantification and statistical analysis of the local writhe density of the plasmid

Utilizing the previously calculated writhe helical axis trace of the model (Figure S3F, **II**), the branches of the plasmid can be determined and split at the junction (Figure S3F, **I**). Each helical axis branch was color-coded, and these color codes were used to segment the points on the plasmid model based on their vicinity (Figure S3F, **III**). These branch segmentations were treated as smaller closed plasmids and subjected to writhe number calculation using the previously described method.^97^ In the statistical analysis, to account for the greater contribution of longer plectonemes to writhe density, long DNA plectonemes were segmented into 100 nm units. Plectonemes shorter than 100 nm were left unsegmented and included as individual units. The same violin plot representation and statistical analysis were used as for the quantification of supercoiled DNA plasmid morphology. Statistical details for each experiment are provided in Figures S3F, S5D, and S6B.

## Supplementary Material

1 Document S1. Figures S1–S9 and Tables S1 and Data S1.

2 Table S2. Cryo-ET per-particle data collection and evaluation, related to STAR methods.

3 Video S1. 3D reconstruction workflow of a representative negatively supercoiled 2 kbp plasmid particle, related to Figure 1.

4 Video S2. Coarse-grained MD simulation of negatively supercoiled plasmid relaxation with and without apical constraint, related to Figure 4.
(A) Left: time evolution of a nicked plasmid without apical constraint. (B) Right: the same system with apical constraint applied.

5 Video S3. Coarse-grained MD simulation of negative DNA supercoiling in managing positive and negative torsion with and without apical constraints, related to Figure 4.
(A) Left: time evolution of an apically constrained plasmid under continuous DNA rotation, leading to the formation of a low-supercoiling domain. (B) Right: a replicate of A that instead develops a new plectoneme at the end.

## Figures and Tables

**Key Resources Table:** 

REAGENT or RESOURCE	SOURCE	IDENTIFIER
Bacterial and virus strains		
BL21(DE3) pLysS *E. coli* competent cells	Macrolab, QB3-Berkeley	N/A
DH5α E. coli competent cells	Macrolab, QB3-Berkeley	N/A
NEB^®^ Stable *E. coli* competent cells	New England Biolabs	C3040H
MG1655	Coli Genetic Stock Center at Yale	Cat#CGSC6300
MG1655/pUC19-T7A1U-AmpR-mEGFP-Tandem	This study	N/A
MG1655/pUC19-T7A1U-AmpR-mEGFP-Opposing	This study	N/A
Rosetta(DE3) pLysS	Macrolab, QB3-Berkeley	N/A
Chemicals, peptides, and recombinant proteins		
β-Agarase I	New England Biolabs	Cat#M0392S
Ampicillin	Sigma-Aldrich	Cat#A0166; CAS: 69-53-4
ATP, [α^32^P]	Perkin Elmer	Cat#BLU003H250UC
Bovine serum albumin (BSA)	New England Biolabs	Cat#B9000
BsaI-HF^®^v2	New England Biolabs	Cat#R3733
BsmBI-v2	New England Biolabs	Cat#R0739
Calcium chloride dihydrate	Sigma-Aldrich	Cat#C5080; CAS: 10035-04-8
Chloramphenicol	Sigma-Aldrich	Cat#C0378; CAS: 56-75-7
Chloroform	Sigma-Aldrich	Cat#C2432; CAS: 67-66-3
Chloroquine diphosphate salt	Sigma-Aldrich	Cat#C6628; CAS: 50-63-5
cOmpleteTM, EDTA-free protease inhibitor cocktail	Roche	Cat#11873580001
Dithiothreitol (DTT)	Sigma-Aldrich	Cat#43815; CAS: 3483-12-3
EcoRI-HF^®^	New England Biolabs	Cat#R3101S
dEcoRI (EcoRI R111Q)	Wright, D. et al^76^	N/A
*E. coli* RNA Polymerase, Core Enzyme	New England Biolabs	Cat#M0550S
*E. coli* RNA Polymerase, Holoenzyme	New England Biolabs	Cat#M0551S
*E. coli* topoisomerase I	New England Biolabs	Cat#M0301S
Ethanol, 200-proof	Koptec	Cat#V1016; CAS: 64-17-5
Ethylenediaminetetraacetic acid, EDTA	Sigma-Aldrich	Cat#E6758; CAS: 60-00-4
Formamide (Deionized)	Sigma-Aldrich	Cat#F9037; CAS: 75-12-7
Glycerol	Sigma-Aldrich	Cat#G5516; CAS: 56-81-5
Glycogen (5 mg/ml)	Invitrogen	Cat#AM9510
GpA RNA Dinucleotid (5’-3’)	TriLink Biotechnologies	Cat#O-31009-05
HEPES	Sigma-Aldrich	Cat# H4034; CAS: 7365-45-9
Human Topoisomerase IB	Topogen	TG2005HRC3
Imidazole CAS: 367-93-1	Sigma-Aldrich	Cat#I5513; CAS: 288-32-4
Isopropyl b-D-1-thiogalactopyranoside, IPTG	Thermo Fisher Scientific	Cat#50-490-794; CAS: 367-93-1
Kanamycin sulfate	Sigma-Aldrich	Cat#60615; CAS: 70560-51-9
LB broth, Miller	Fisher Bioreagents	Cat#BP1426-2
Magnesium chloride, MgCl2	Thermo Fisher Scientific	Cat# BP214; CAS: 7786-30-3
Magnesium sulfate, MgSO4	Sigma-Aldrich	Cat#M7506; CAS: 7487-88-9
Nt.BspQI	New England Biolabs	Cat#R0644S
NTP Set, 100 mM Solution	Thermo-Scientific	Cat#R0481
Phenol:Chloroform:Isoamyl Alcohol 25:24:1	Fisher Scientific	Cat#BP1752I-100
Phusion High-Fidelity DNA Polymerase	New England Biolabs	Cat#M0530S
Potassium chloride, KCl	Sigma-Aldrich	Cat#P9541; CAS: 7447-40-7
Potassium phosphate dibasic, K_2_HPO4	Sigma-Aldrich	Cat#60353; CAS: 7758-11-4
Potassium phosphate monobasic, KH_2_PO4	Sigma-Aldrich	Cat#P9791; CAS: 7778-77-0
Proteinase K	New England Biolabs	Cat#P8107S
SeaPlaque^®^ Agarose	Lonza	Cat#50100
SequaGel UreaGel 29:1 Concentrate	National Diagnostics	Cat#EC-828
Sodium chloride, NaCl	Sigma-Aldrich	Cat#S9888; CAS: 7647-14-5
Sodium dodecyl sulfate, SDS	Sigma-Aldrich	Cat#L5750; CAS: 151-21-3
Sodium phosphate dibasic, Na_2_HPO4	Sigma-Aldrich	Cat#S3264; CAS: 7558-79-4
*S. solfataricus* reverse gyrase (TopR2)	This study	N/A
SYBR^™^ Safe DNA Gel Stain	Invitrogen	Cat#S33102
Terrific Broth	Thermo Fisher Scientific	Cat#BP2468500
TEV Protease	Macrolab, QB3-Berkeley	N/A
TopVision Agarose	Thermo Fisher Scientific	Cat#R0491
T4 DNA Ligase	New England Biolabs	Cat#M0202
D-(+)-Trehalose dihydrate	Sigma-Aldrich	Cat#T9531
Tris Base	Genesee Scientific	Cat#18-146; CAS: 77-86-1
Urea	Sigma-Aldrich	Cat#U1250; CAS: 57-13-6
Critical commercial assays		
HiScribe^®^ T7 High Yield RNA Synthesis Kit	New England Biolabs	Cat# E2040L
Monarch^®^ Spin PCR & DNA Cleanup Kit (5 μg)	New England Biolabs	Cat# T1130S
QIAprep Spin Miniprep Kit	QIAGEN	Cat# 27104
QIAGEN Plasmid Maxi Kit	QIAGEN	Cat# 12162
Deposited data		
Cryo-ET maps of P. LS particles	Electron Microscopy Data Bank (EMDB)	EMD-47843
Cryo-ET maps of P. HS particles	Electron Microscopy Data Bank (EMDB)	EMD-47844
Cryo-ET maps of sTEC particles	Electron Microscopy Data Bank (EMDB)	EMD-47850
Cryo-ET maps of P. Cas particles	Electron Microscopy Data Bank (EMDB)	EMD-47849
Cryo-ET maps of TEC particles	Electron Microscopy Data Bank (EMDB)	EMD-47850
Cryo-ET maps of sTEC-Cas particles	Electron Microscopy Data Bank (EMDB)	EMD-47851
Cryo-ET maps of TEC-Cas particles	Electron Microscopy Data Bank (EMDB)	EMD-47853
Cryo-ET maps of TEC-TopI particles	Electron Microscopy Data Bank (EMDB)	EMD-47855
Cryo-ET maps of Opp-TEC particles	Electron Microscopy Data Bank (EMDB)	EMD-71622
Cryo-ET maps of Tan-TEC particles	Electron Microscopy Data Bank (EMDB)	EMD-71618
SPA maps of TEC1–SC	Electron Microscopy Data Bank (EMDB)	EMD-71675
SPA maps of TEC2–SC	Electron Microscopy Data Bank (EMDB)	EMD-71676
PDB of TEC1–SC	Protein Data Bank (PDB)	9PIP
PDB of TEC2–SC	Protein Data Bank (PDB)	9PIQ
Cryo-ET raw tilt-series data, 3D reconstructions, and structural models	This paper; Zenodo Data	https://doi.org/10.5281/zenodo.16423113
oxDNA DNA supercoiling simulations	This paper; Zenodo Data	https://doi.org/10.5281/zenodo.16423113
Script for swivel angle calculation	This paper; Zenodo Data	https://doi.org/10.5281/zenodo.16423113
Unprocessed fluorescence and gel image	This paper; Zenodo Data	https://doi.org/10.5281/zenodo.16423113
Oligonucleotides		
CBD01: AATACTAGAATTCTTATCAAAAAGAGTATTGACTTAAAGTCTAACCTATAGGATACTTACAGCCGAAAAAAGCAACAAAAAAATTGAAAAAGGAAGAGTATGAGTATTCAAC	IDT	N/A
CBD02: AATACTAGAATTCCTGCATTAATGAATCGGCC	IDT	N/A
CBD03: AAAAGCACCGACTCGGTGCCACTTTTTCAAGTTGATAACGGACTAGCCTTATTTTAACTTGCTATTTCTAGCTCTAAAAC	IDT	N/A
CBD04: TTCTAATACGACTCACTATAGTGGTCATGAGATTATCAAAAGTTTTAGAGCTAGAAATAG	IDT	N/A
CBD05 GCGCCCAATACGCAAACCG	IDT	N/A
CBD06 CCAGGAACCGTAAAAAGGCCG	IDT	N/A
CBD07 GGCCTTTTTACGGTTCCTGGTGGCCGATTCATTAATGCAGGAATTCTTATC	IDT	N/A
CBD08 TCCTCGCCCTTGCTCACCATACTCTTCCTTTTTCAATTTTTTTGTTGCTTTTTTCG	IDT	N/A
CBD09: ATGGTGAGCAAGGGCGAGGAG	IDT	N/A
CBD10: GCGGTTTGCGTATTGGGCGCGATTAAAACGAAAGGCCCAGTCTTTCGA	IDT	N/A
CBD11: CCACGTCTCATTGAGATCCTTTTTTTCTGCGCGTAATCT	IDT	N/A
CBD12: CCACGTCTCAACGCGCGGGGAGAGGCG	IDT	N/A
CBD13: CCACGTCTCAGCGTGAAGATCCTTTGATCTTTTCTACGGGGTCTG	IDT	N/A
CBD14: CCACGTCTCATCAATGGCCGATTCATTAATGCAGGAATTCTTATC	IDT	N/A
CBR01 (sgRNA targeting TTS): GUGGUCAUGAGAUUAUCAAAAGUUUUAGAGCUAGAAAUAGCAAGUUAAAAUAAGGCUAGUCCGUUAUCAACUUGAAAAAGUGGCACCGAGUCGGUGCUUUU	This study	N/A
CBMB01 (Molecular Beacon for AmpR): /56-FAM/mCmGmCmUmAmUmCmUmAmAmAmGmUmAmUmAmUmAmUmGmAmGmUmAmAmAmGmCmG/3BHQ_1/	IDT	N/A
CBMB02 (Molecular Beacon for mEGFP): /56-FAM/mCmGmCmUmAmGmCmGmCmCmUmCmAmGmCmUmAmUmCmAmUmGmUmCmGmAmGmCmG/3BHQ_1/	IDT	N/A
Recombinant DNA		
pET His6 LIC cloning vector (2Bc-T)	Addgene	37236
p2Bc-T-TopR2	This study	N/A
pMJ825	Addgene	#39315
pMJ841	Addgene	#39318
pUC19	New England Biolabs	N3041S
pUC19-T7A1U	This study	N/A
pUC19-T7A1U-AmpR-mEGFP-Tandem	This study	N/A
pUC19-T7A1U-AmpR-mEGFP-Opposing	This study	N/A
*Sulfolobus solfataricus* genomic DNA	ATCC	35092D-5
Software and algorithms		
IMOD	Kremer et al.^77^	https://bio3d.colorado.edu/imod/
Serial EM	Schorb et al.^78^	https://bio3d.colorado.edu/SerialEM/
MotionCor2	Zheng et al.^79^	https://msg.ucsf.edu/software
UCSF Chimera	Pettersen et al.^80^	https://www.cgl.ucsf.edu/chimera/
ChimeraX	Meng et al.^81^	
EMAN2	Chen et al.^28^	https://blake.bcm.edu/emanwiki/EMAN2
CARE	Weigert et al.^82^	https://csbdeep.bioimagecomputing.com/tools/care/
DNA plectoneme site prediction	Kim et al.^43^	https://github.com/elifesciences-publications/Plectoneme_analysis
Cryo-SPARC	Punjani et al.^83^	https://cryosparc.com/
3D skeletonizing	Lee et al.^84^	https://scikit-image.org/docs/0.13.x/auto_examples/edges/plot_skeleton.html
IsoNet	Liu et al.^29^	https://github.com/IsoNet-cryoET/IsoNet
oxDNA	Suma et al.^85^	https://dna.physics.ox.ac.uk/index.php/Main_Page
DeepEMhancer	Sanchez-Garcia et al.^86^	https://github.com/rsanchezgarc/deepEMhancer
ISOLDE	Croll et al.^87^	https://tristanic.github.io/isolde/
PHENIX	Liebschner et al.^88^	https://phenix-online.org/documentation/overviews/cryo-em_index.html
MolProbity	Chen et al.^89^	https://www.phenix-online.org/documentation/reference/molprobity_tool.html
The E-CAM Software Library	Eastman et al.^30^	https://e-cam.readthedocs.io/en/latest/
Other		
Amicon Ultra-0.5 Centrifugal Filter Unit, 3K MWCO	Thermo Fisher Scientific	Cat#UFC500324
Amicon Ultra-0.5 Centrifugal Filter Unit, 10K MWCO	Thermo Fisher Scientific	Cat#UFC501024
Amicon Ultra-15 Centrifugal Filter Unit, 10K MWCO	Thermo Fisher Scientific	Cat#UFC901024
Amicon Ultra-15 Centrifugal Filter Unit, 30K MWCO	Thermo Fisher Scientific	Cat#UFC903008
HiPrepTM Sephacryl S300 16/60	GE Healthcare	Cat#17-1167-01
HisTrap HP	GE Healthcare	Cat#17-5248-02
HiTrap Heparin HP	GE Healthcare	Cat#17-0407-01
HiTrap Q HP	GE Healthcare	Cat#17-1154-01
UltraPure DNase/RNase-Free Distilled Water	Thermo Fisher Scientific	Cat#10977-023
Quantifoil R 2/1 200 mesh Au	Electron Microscopy Sciences	Cat#661-200-AU
Quantifoil R 3.5/1 200 mesh Au	Electron Microscopy Sciences	Cat#660-200-AU

## Highlights:

DNA-melting proteins (RNAP, dCas9) preferentially localize to apices on –sc. DNA

Apical RNAP promotes initiation but slows elongation by torsional regulation

RNAP and dCas9 act as paired torsional roadblocks segregating supercoiling domains

Supercoiling domains couple RNAPs to enable cooperative transcription
