# Supplementary material for: Apical Localization of RNA Polymerases Modulates Transcription Dynamics and Supercoiling Domains Revealed by Cryo-ET
Source: Mol Cell. Author manuscript; Available in PMC 2026 Jul 8. (PMC13344188; doi:10.1016/j.molcel.2026.04.013)
Supplement: 1 — Document S1. Figures S1–S9 and Tables S1 and Data S1. [file NIHMS2174472-supplement-1.pdf]

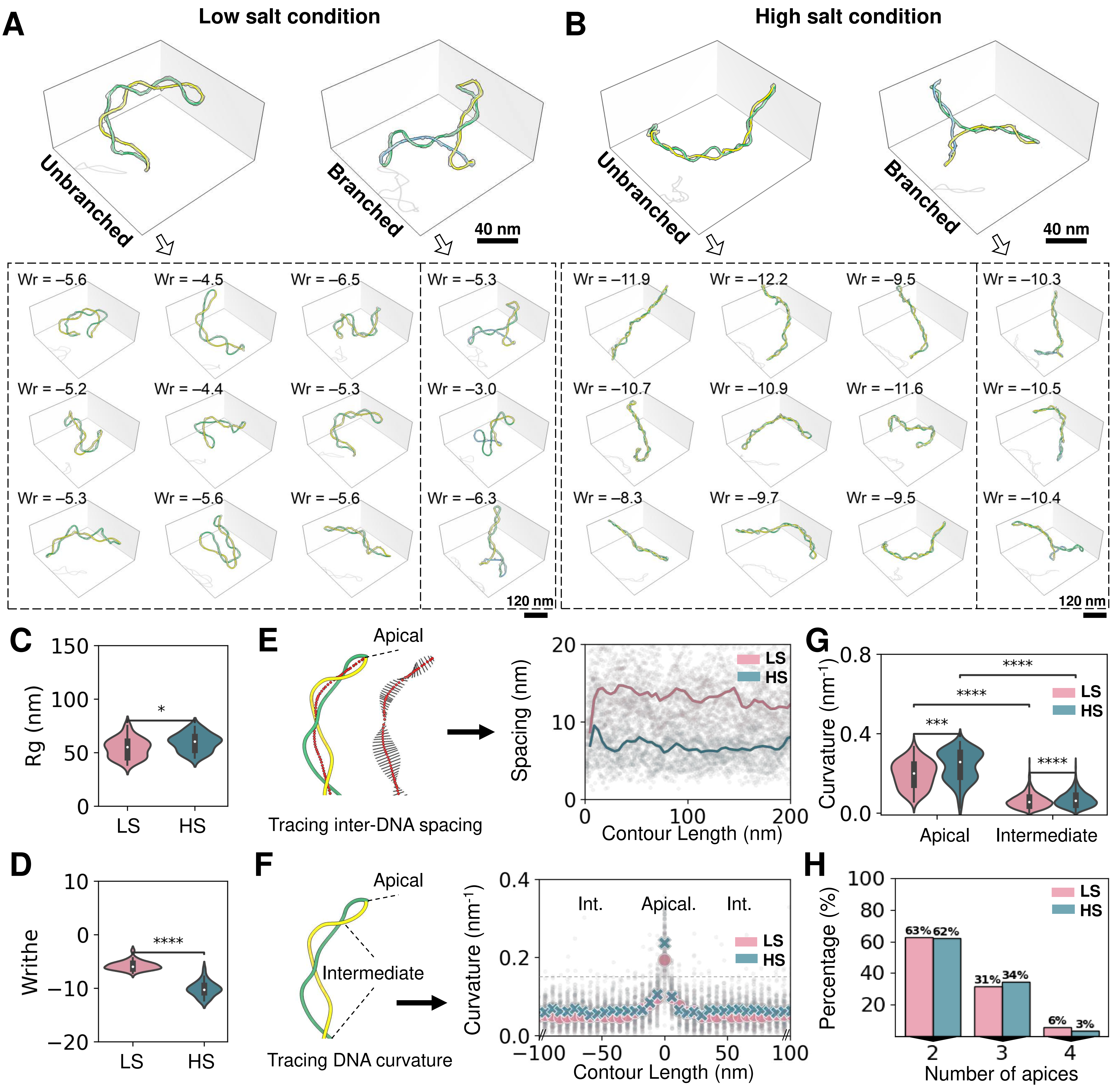

Figure S1: Structural analysis of –sc plasmid under low salt and high salt conditions, related to Figure 1
(**A-B**) Representative cryo-ET map and model of the –sc plasmid (ΔLk centered on -15) under low salt (LS) and high salt (HS) conditions, respectively. Two exemplary particles (unbranched and branch) are displayed on the top panel, with their collection presented on the bottom, featuring writhe numbers. (**C-D**) Statistical analysis of plasmid radius of gyration (Rg) and writhe number using violin plots, respectively; N=64. (**E**) Schematic of inter-DNA spacing quantification (black bars) along the plectoneme axis (red dots). The inter-DNA spacing of the plectoneme was plotted from the plasmid apex toward the intermediate region; N=158 (**F**) Schematic illustrating the apical and intermediate regions of a plectoneme, accompanied by DNA curvature quantification. (**G**) Statistical analysis of DNA curvature near the apical and intermediate regions of the plectoneme under LS and HS conditions; N=158 (**H**) Distribution of the number of apices of the plasmid in LS and HS conditions; N=64. Statistics are calculated using a Mann-Whitney test, where *p < 0.05, **p < 0.01, ***p < 0.001, ****p < 0.0001; and ns, not significant.

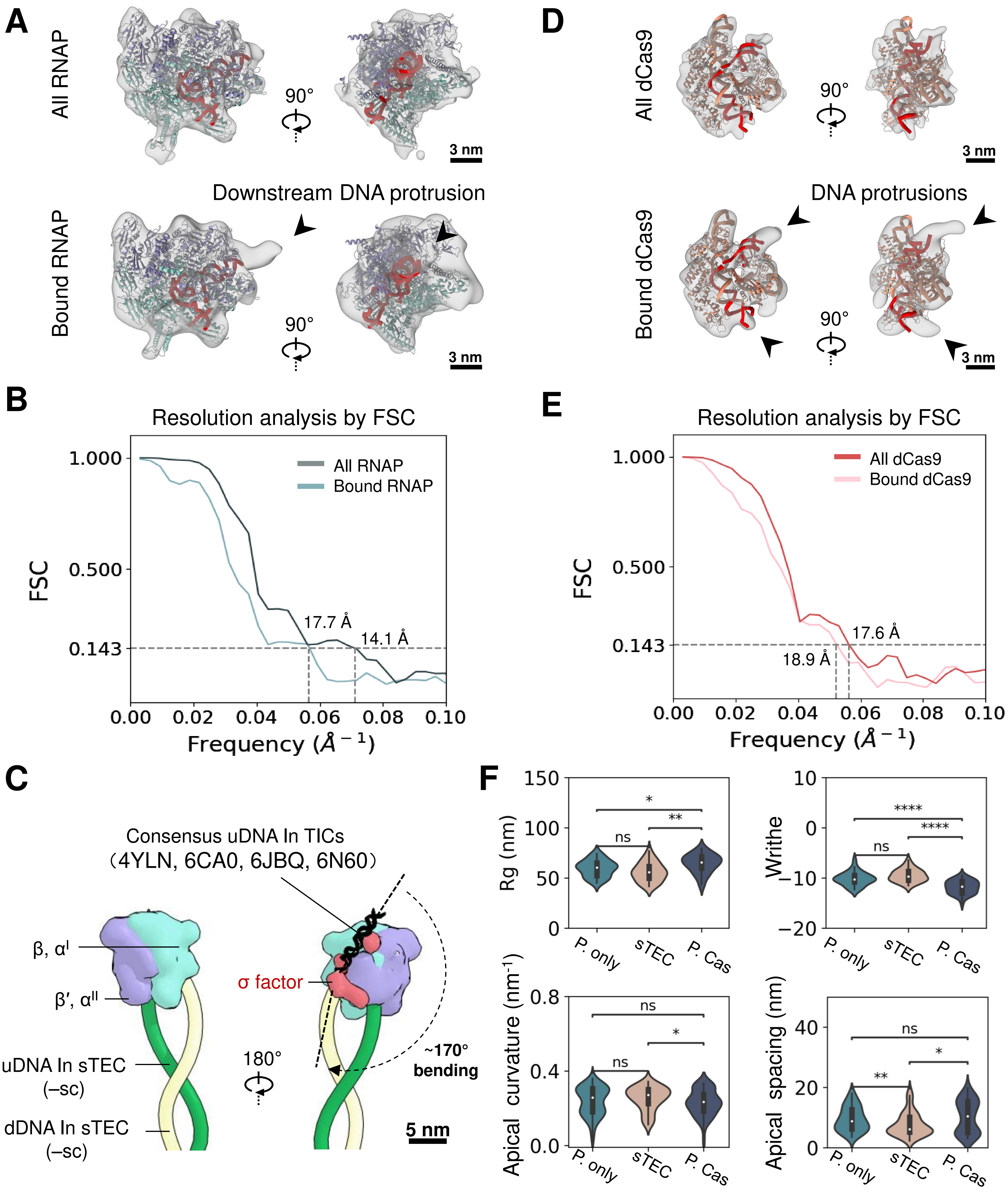

Figure S2: Sub-tomogram averaging analysis of apically bound RNAP and dCas9, related to Figure 2

(**A-B**) Two orthogonal views of the sub-tomogram averaged RNAP map docked with PDB structure 6ALH. Averaging all RNAPs selected from the tomogram (N=1781) yielded a 14.1 Å resolution map (top), while using only plasmid-bound RNAPs after inspection (N=232) resulted in a 17.7 Å resolution map (bottom). (**C**) Superimposition of consensus TIC upstream DNA (black helix) and the TIC σ-factor (red) from PDBs 4YLN, 6CA0, 6JBQ, and 6N60 onto the apically stalled TEC on –sc DNA reveals a ~170° upstream DNA bend. (**D-E**) Sub-tomogram averaged dCas9 map docked with PDB structure 6O0X. Averaging all dCas9 particles selected from the tomogram (N=875) yielded a 17.6 Å resolution map (top), while the plasmid-bound subset (N=116) achieved a resolution of 18.9 Å (bottom). Resolutions were estimated by measuring the Fourier shell correlation (FSC) between two independently determined half-maps at 0.143. (**F**) Statistical analysis of global plasmid Rg and writhe number (N=90), along with local DNA curvature and inter-DNA spacing at apical sites (N=207), for plasmid only, plasmid with stalled RNAP, and plasmid with dCas9, respectively. Statistics are calculated using a Mann-Whitney test, where $^*p < 0.05$, $^{**}p < 0.01$, $^{***}p < 0.001$, $^{****}p < 0.0001$; and ns, not significant.

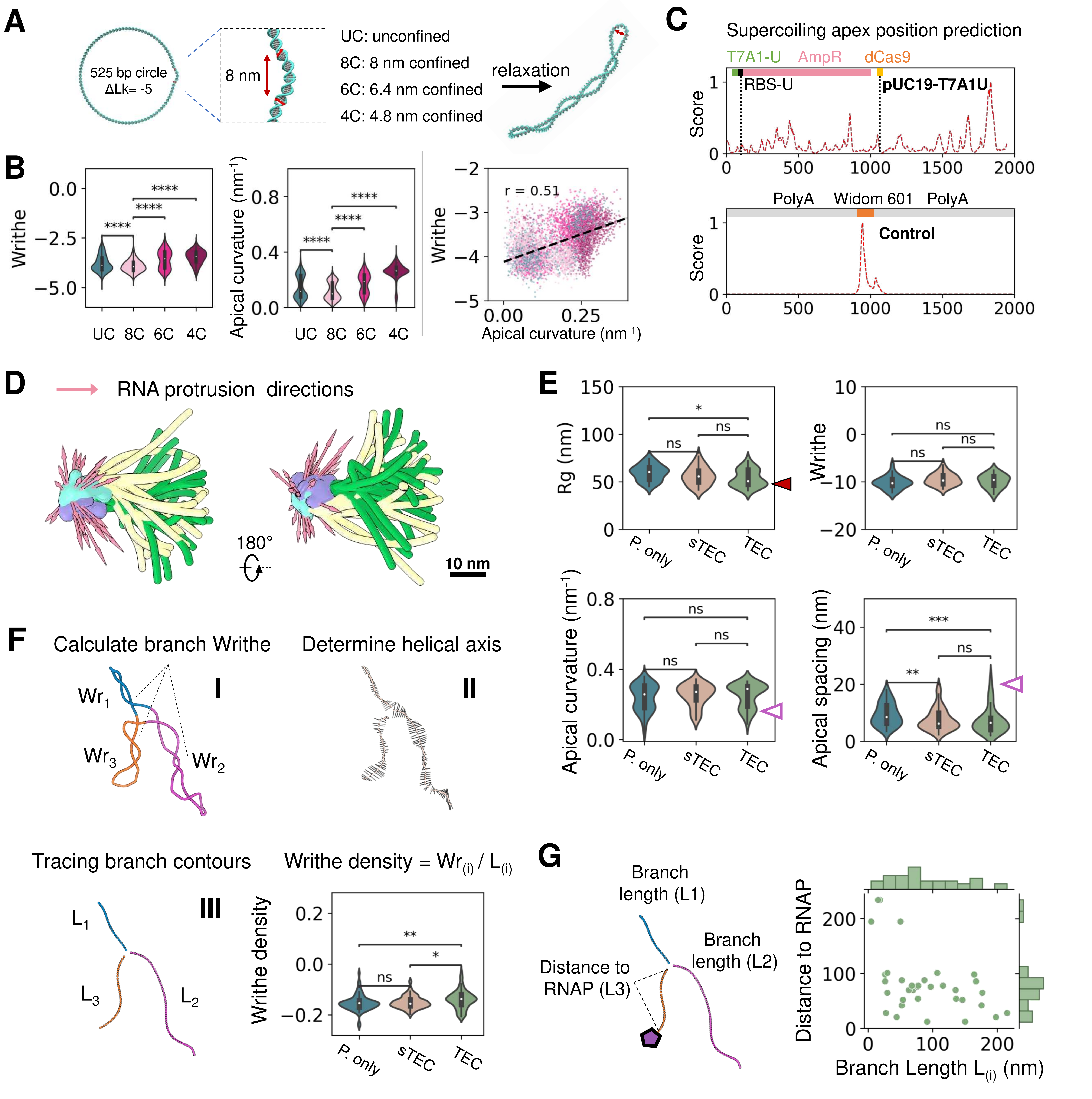

Figure S3: Quantification of plasmid geometry upon RNAP/dCas9 binding, related to Figure 3
(**A**) Generation of –sc DNA minicircles with a target site subjected to varying levels of constraint, defined by spring distances of 8, 6.4, and 4.8 nm, to modulate DNA relaxation. (**B**) Quantification of relaxed plasmid writhe, apical DNA curvature, and their correlation following MD simulation (sampled from frame 100 onward after relaxation; three replicates). (**C**) Predicted DNA plectoneme formation loci along the pUC19-T7A1U sequence. A control was generated using a polyA–Widom601–polyA sequence. (**D**) Overlay of apically bound RNAPs in TECs with RNA protrusion directions indicated by pink arrows. (**E**) Statistical analysis of global plasmid Rg and writhe number (N=79), along with local DNA curvature and inter-DNA spacing at apical sites (N=192) for sTECs and TECs. (**F**) Plectoneme branches were segmented (I), and their superhelical axes (II) and contour lengths (III) were determined. Writhe density was calculated as writhe number divided by segment contour length. Bottom right: writhe density distribution; N=187. (**G**) Plot of plectoneme branch length versus distance from the branch to the apical RNAP; N=38. Statistics are calculated using a Mann-Whitney test, where *p < 0.05, **p < 0.01, ***p < 0.001, ****p < 0.0001; and ns, not significant.

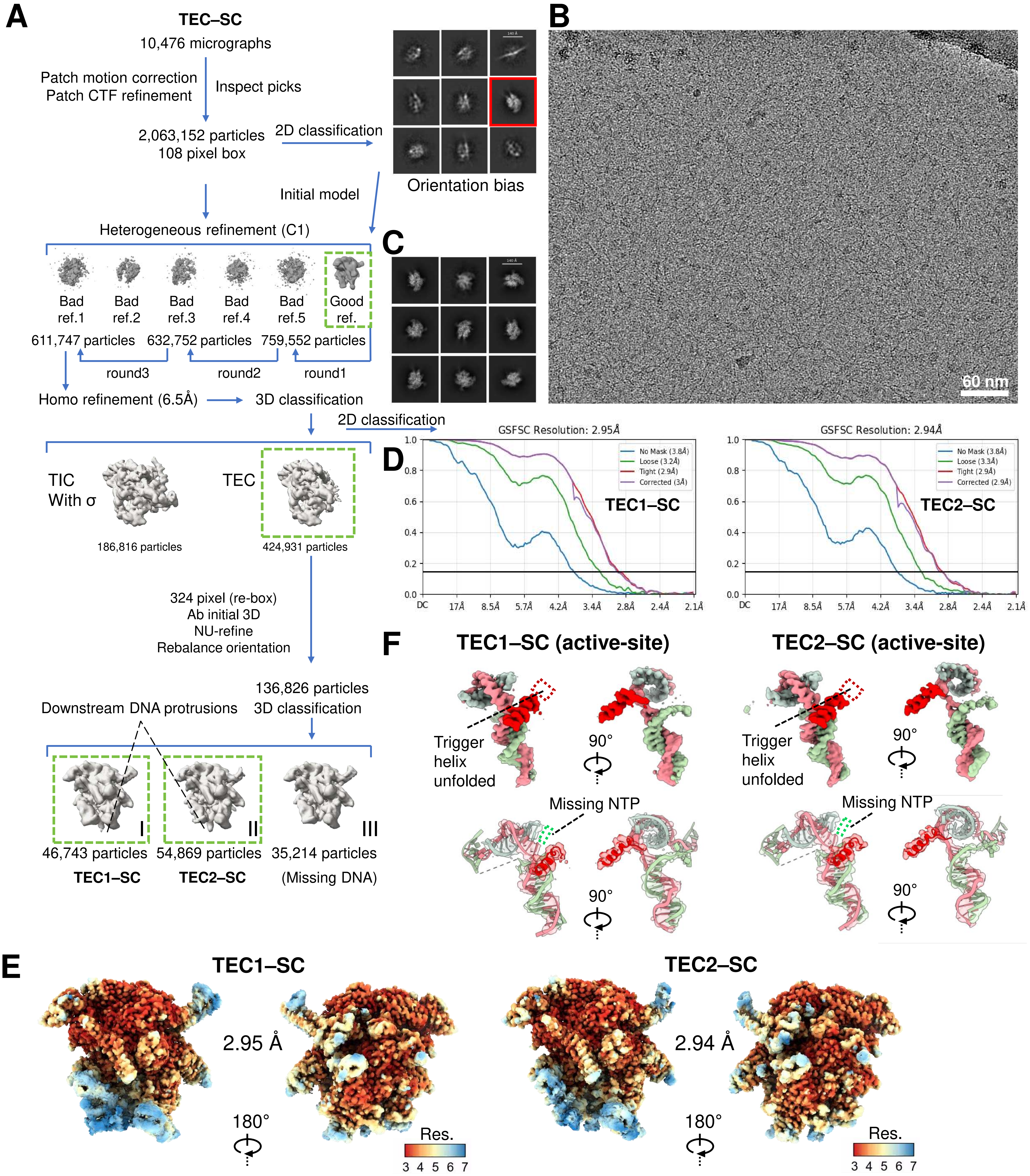

Figure S4: Cryo-EM analysis of the transcription elongation complex (TEC) on a –sc plasmid template, related to Figure 3

(**A**) Workflow of single-particle analysis and 3D reconstruction of TECs. Three final maps were obtained: the first (TEC1–SC, global resolution of 2.95 Å from 46,743 particles) and the second (TEC2–SC, global resolution of 2.94 Å from 54,869 particles) show particles with downstream DNA protrusion. The third class was not further analyzed due to the absence of DNA density, indicating likely unbound RNAPs. (**B**) Representative reference-free 2D class averages of TEC particles, displaying various TECs orientations. (**C**) Representative cryo-EM micrograph of TEC particles assembled on negatively supercoiled pUC19-T7A1U plasmid in vitreous ice. (**D**) Fourier shell correlation (FSC) curves between independently reconstructed even–odd maps. Resolution was estimated at the 0.143 FSC cutoff. (**E**) Cryo-EM density maps of TEC1–SC (left) and TEC2–SC (right), color-coded by local resolution, ranging from 3 Å (red) to yellow to 7 Å (blue). (**F**) Examination of RNAP active sites shows that both TEC1–SC and TEC2–SC are in a post-translocated state, with the trigger helix unfolded and the NTP absent.

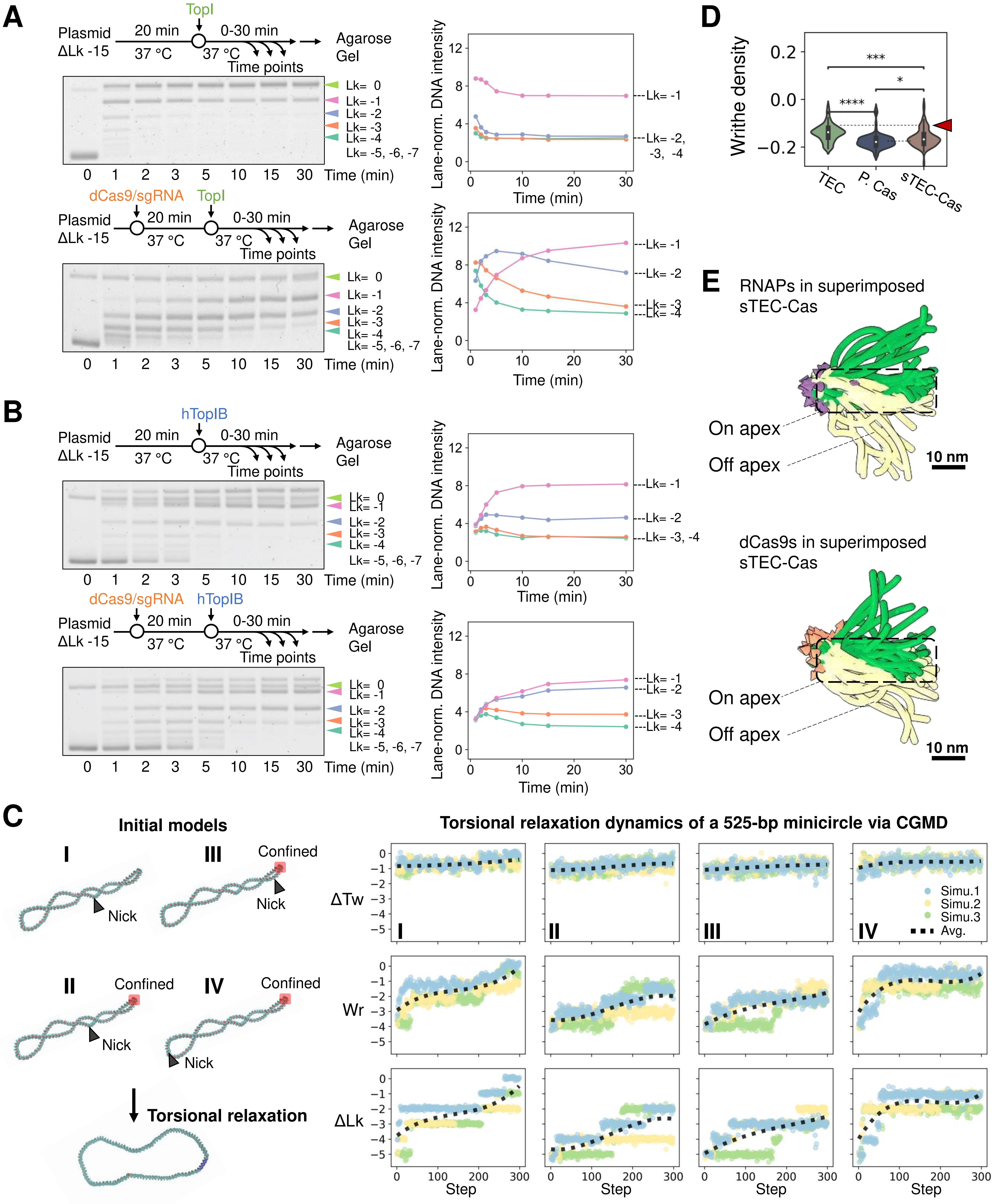

Figure S5: Apical binding of dCas9 hinders DNA rotation, related to Figure 4
(**A**) DNA supercoiling relaxation assay in the absence (top) and presence (bottom) of a dCas9. Right panel: quantification of ΔLk changes over time following TopI addition. (**B**) Control experiment of A, with TopI replaced by hTopIB for DNA supercoiling relaxation. (**C**) Coarse-grained MD simulations of 525-bp –sc (ΔLk = –5) minicircle plasmid relaxation dynamics, initiated with different nick locations. Simulations were conducted without apical confinement (Model I) and with apical restriction (Models II–IV). Topological parameters (ΔTw, Wr, and ΔLk) are shown in rows 1–3 for each model. Each simulation was performed in triplicate, with averages indicated by black dashed lines. (**D**) Statistical analysis of plectoneme branch writhe density distribution; N=226. (**E**) Superimposition of apical DNA segments illustrating the dynamics of stalled RNAP and dCas9 when co-present on the plasmid.

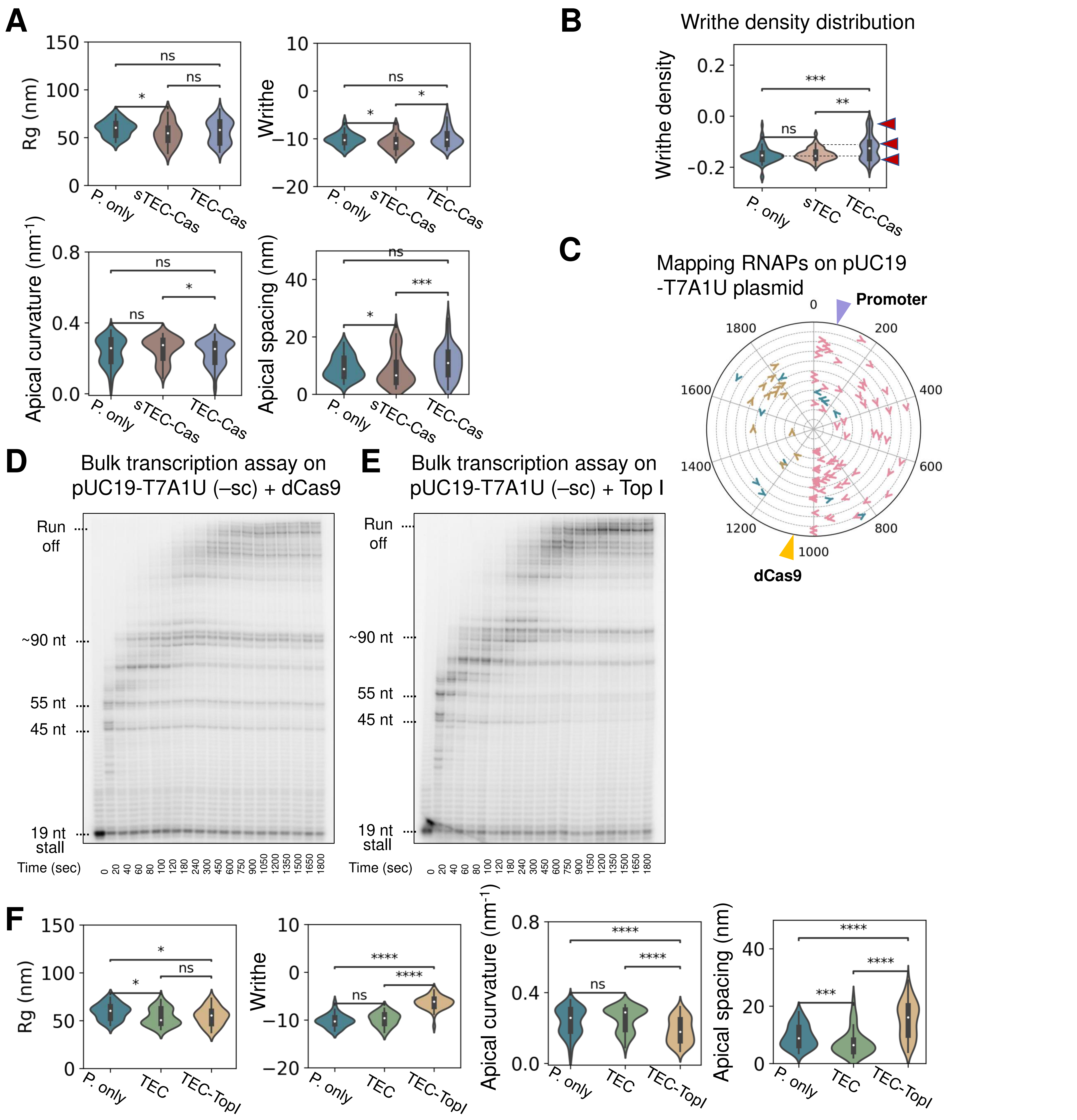

Figure S6: Quantification of plasmid morphology with co-presence of RNAP and dCas9, and of RNAP and TopI, related to Figures 4 and 5

(**A**) Statistical analysis of global plasmid Rg and writhe number (N=107), along with local DNA curvature and inter-DNA spacing at apical sites (N=270) for sTEC-Cas and TEC-Cas particles. (**B**) Statistical comparison of the plectoneme writhe density distribution; N=198. (**C**) Mapping all bound RNAPs on pUC19-T7A1U templates using dCas9 as fiducial. For visualization, different plasmids and their associated RNAPs are displayed at distinct radii in the polar plot. RNAP on the transcriptional region, oriented downstream, is marked in pink; RNAP on the non-transcriptional region or pointing upstream, is colored in tan and blue, respectively; N=89 (**D–E**) Electrophoresis assay of RNAP transcription on –sc pUC19-T7A1U templates in the presence of dCas9 and in the presence of TopI over time, respectively. (**F**) Same statistical measurements as in (A), performed on TEC-TopI particles (90 particles and 212 apices total) . All statistics are calculated using a Mann-Whitney test, where $^*p < 0.05$, $^{**}p < 0.01$, $^{***}p < 0.001$, $^{****}p < 0.0001$; and ns, not significant.

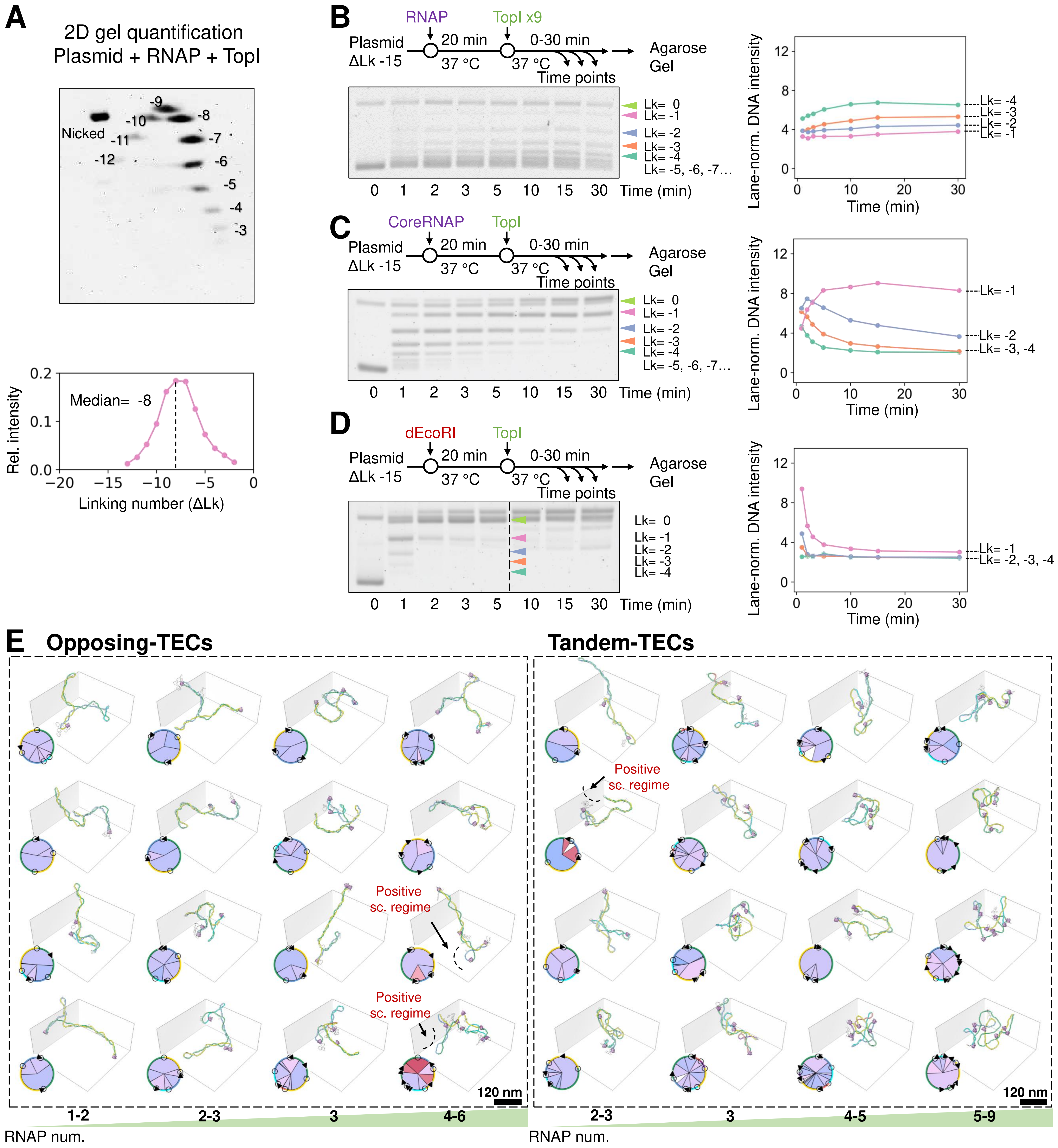

Figure S7: Top I and RNAP coupling slows supercoiling relaxation, and particle collections of dual-promoter TECs, related to Figures 5 and 6

(**A**) 2D electrophoresis gel of –sc pUC19-T7A1U plasmid in the presence of RNAP and TopI, with quantification of the dominant ΔLk value. (**B**) 1D gel-based DNA supercoiling relaxation assay of plasmid in the presence of RNAP and a large excess of TopI. (**C**) DNA supercoiling relaxation assay of plasmid in the presence of RNAP core (lacking σ and unable to form stable transcription bubbles). (**D**) DNA supercoiling relaxation assay of plasmid in the presence of dEcoRI. 1D gel-based linking number quantification is shown in the right panel for B-D. (**E**) Particle collections of opposing-promoter TECs and tandem-promoter TECs, ordered by the number of bound RNAPs. Each dual-promoter TEC is also shown with its corresponding circular plasmid layout, annotated in red to indicate plectonemes extending into the positive supercoiling regime.

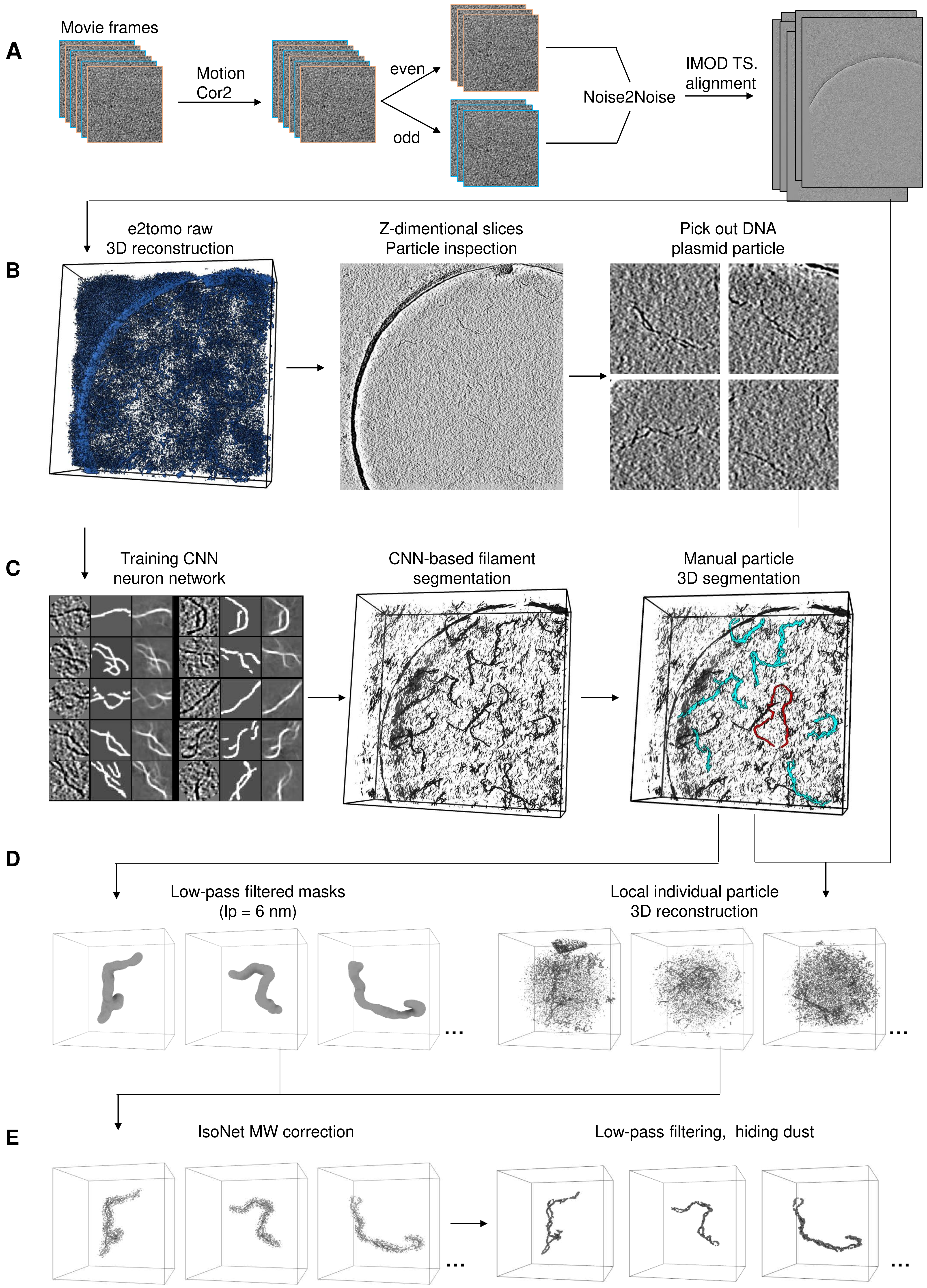

Figure S8: Cryo-ET 3D reconstruction workflow, related to Figure 1 and STAR Methods

(**A**) The motion-corrected tilt series were split into even and odd sets and subjected to deep learning-based image denoising (N2N method). (**B**) The denoised tilt series were initially patch tracking aligned in imod and submitted for full tomogram reconstruction in e2tomo. (**C**) DNA segments selected from the large 3D map were used to train a CNN network for annotating all DNA elements in the tomogram. (**D**) A mask of the target particle was created to calculate its center of mass, which was then used to extract raw tilts from the initial low-contrast micrograph for localized 3D reconstruction of individual particles. (**E**) The map was processed with IsoNet for missing wedge correction, followed by low-pass filtering to 2 nm and dust removal, resulting in final map for modeling.

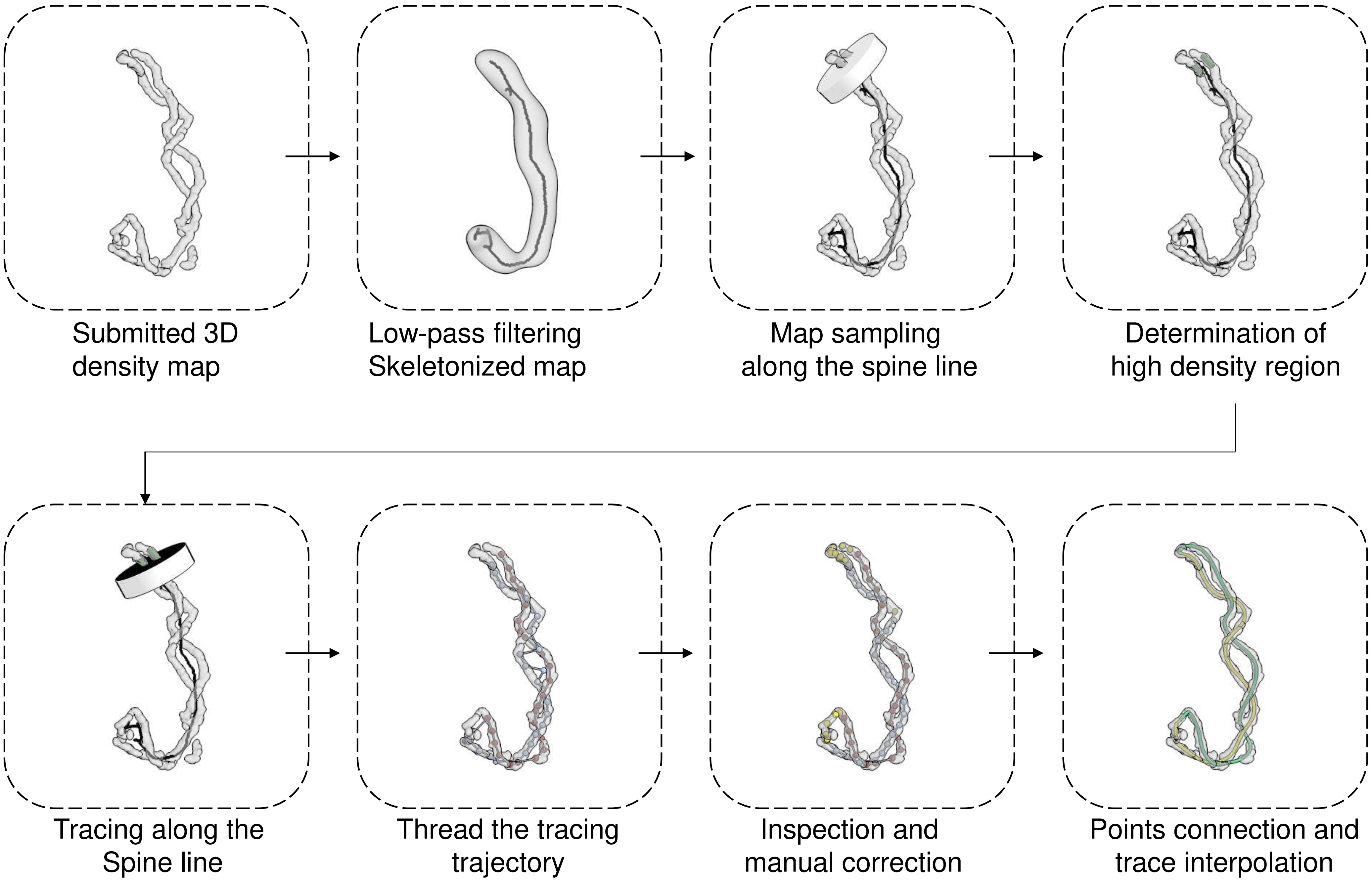

Figure S9: DNA tracing within cryo-ET maps for plasmid particle modeling, related to Figure 1 and STAR Methods
DNA model fitting workflow, see also STAR methods section: Individual particle 3D reconstruction, refinement, and modeling.

	TEC1-SC.	TEC2-SC.
EMDB: Map	EMD-71675	EMD-71676
PDB: Model	9PIP	9PIQ
Microscope	Thermo Fisher Krios G3i	Thermo Fisher Krios G3i
Detector	Gatan K3	Gatan K3
Voltage (kV)	300	300
Magnification	81,000	81,000
Electron exposure(e^-/Å^2)	50	50
Defocus range (μm)	−0.6 to −1.6	−0.6 to −1.6
Pixel size (Å)	1.05	1.05
Images	10476	10476
Initial particles	2,063,152	2,063,152
Final particles	46,743	54,869
Symmetry	C1	C1
Box size (pix)	324	324
Resolution (Å) (FSC = 0.143)	2.95	2.94
Map sharpening B factor (Å^2)	--	--
Initial model used (PDB code)	6ALH	6ALH
Model resolution (Å)	4.4	4.4
FSC threshold	0.5	0.5
Model composition		
Non-hydrogen atoms	26008	26008
Protein residues	3230	3230
Ligands	--	--
B factors (Å^2)		
Protein	50.7	49.0
Ligand	--	--
R.m.s. deviations		
Bond lengths(Å)	0.0064	0.006
Bond angles (°)	1.17	1.13
Validation		
MolProbity score	1.64	1.71
Clashscore	5.45	5.73
Poor rotamers(%)	0.04	0.00
Ramachandran plot		
Favored (%)	94.95	94.12
Allowed (%)	5.05	5.88
Disallowed (%)	0.00	0.00

Table S1: Cryo-EM single-particle data collection and refinement statistics, related to Figure 3

Data S1. Gallery of reconstructed individual plasmid particles in this study, relate to Figure 1, 2, 3, 4, 5 and 6

Apical Localization of RNA Polymerases Modulates Transcription Dynamics and Supercoiling Domains Revealed by Cryo-ET

Meng Zhang[1,2,7*], Cristhian Cañari-Chumpitaz[1,3,4,7*], Jianfang Liu[2], Bibiana Onoa[1,5], Sinead de Cleir[6], Enze Cheng[8], Katherinne I. Requejo[1,7], Carlos Bustamante[1,6,7,8,9,10,11,12**]

[1] California Institute for Quantitative Biosciences, University of California, Berkeley, USA
[2] The Molecular Foundry, Lawrence Berkeley National Laboratory, Berkeley, USA
[3] Department of Chemistry, University of California Berkeley, Berkeley, CA, USA
[4] Department of Biology, Stanford University, Stanford, CA USA
[5] Innovative Genomics Institute, University of California Berkeley, Berkeley, CA, USA
[6] Department of Molecular and Cell Biology, University of California, Berkeley, USA
[7] Howard Hughes Medical Institute, University of California, Berkeley, USA
[8] Department of Physics, University of California, Berkeley, USA
[9] Department of Chemistry, University of California, Berkeley, USA
[10] Molecular Biophysics and Integrative Bioimaging Division, Lawrence Berkeley National Laboratory, USA
[11] Kavli Energy Nanoscience Institute, University of California, Berkeley, USA
[12]Jason Choy laboratory of single molecule biophysics, University of California, Berkeley, USA
* These authors contributed equally
** Correspondence should be addressed to: C. B. (carlosb@berkeley.edu)

Particle gallery of plasmid in low salt condition (P. LS) 2
Particle gallery of plasmid in high salt condition (P. HS) 19
Particle gallery of stalled transcription elongate complexes (sTEC) 34
Particle gallery of plasmid particles in the presence of dCas9 (P. Cas) 48
Particle gallery of transcription elongate complexes (TEC) 64
Particle gallery of sTECs in the presence of dCas9 (sTEC-Cas9) 76
Particle gallery of TECs in the presence of dCas9 (TEC-Cas9) 93
Particle gallery of TECs in the presence of TopI (TEC-TopI) 114
Particle gallery of TECs containing two opposing promoters (Opp-TEC) 134
Particle gallery of TECs containing two tandem promoters (Tan-TEC) 152

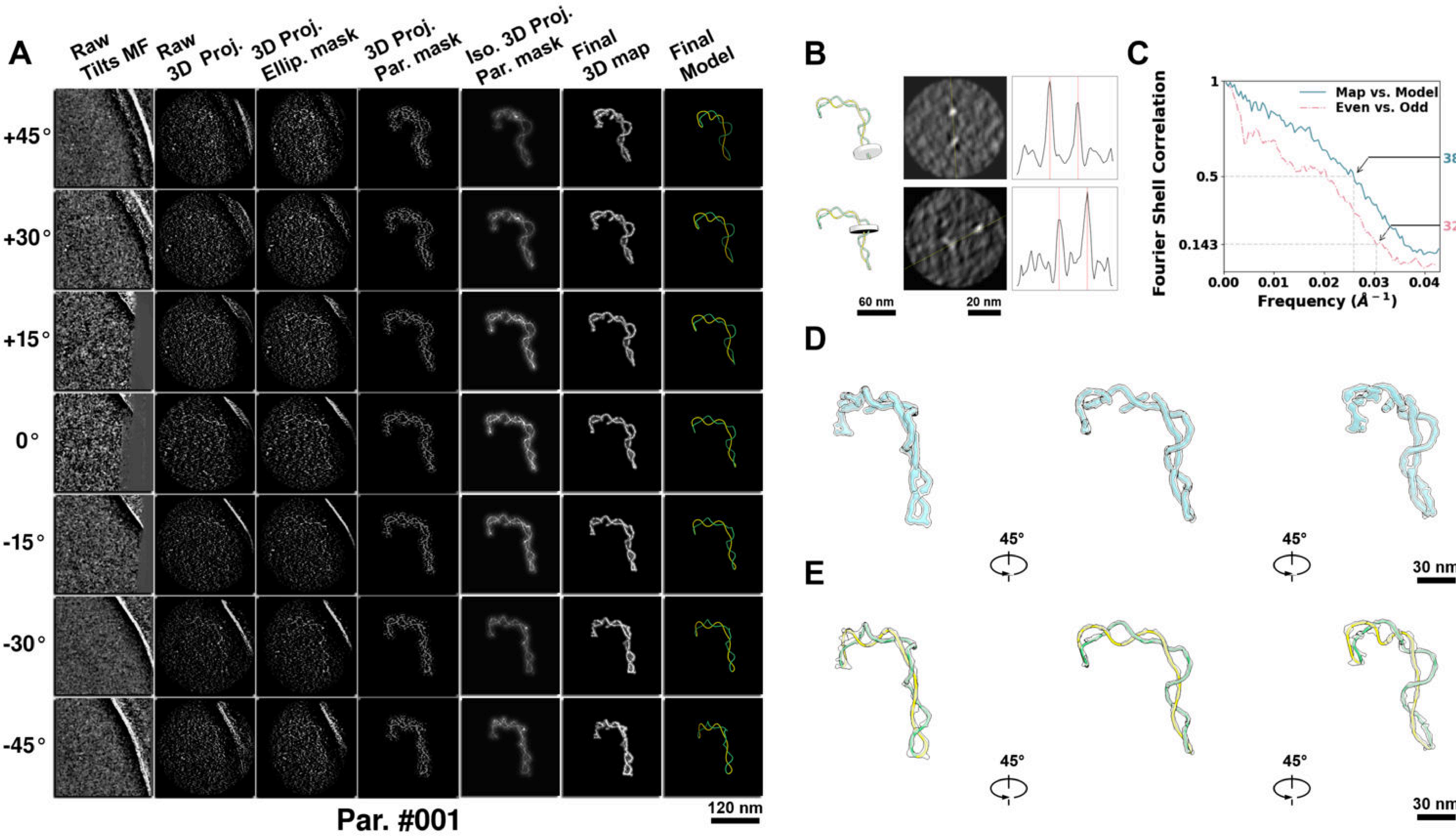

Supplementary Particle Figure 1. Cryo-ET 3D reconstruction of an individual P.LS particle.
(A) 3D reconstruction of the plasmid particle (index no. 1). The first column shows seven representative tilt images from +45° to -45° in step of 15°. The second, third, and fourth columns show 3D projections of the particle with spherical, ellipsoidal (thinner along the z-dimension), and particle-shaped masks, respectively. The fifth column displays the 3D projections of the enhanced and IsoNet missing-wedge-corrected particle. The sixth and seventh columns present the final 3D map and the flexibly fitted model, respectively. (B) Two cross-sectional views (12 nm thickness) of the plasmid density map along its plectoneme axis are shown in the left-middle panel. The intensity profile along the line crossing the two high-density DNA spots is displayed in the right panel. (C) Resolution assessment of the final 3D map using Fourier shell correlation (FSC). Two criteria are shown: FSC between two half-maps reconstructed from even and odd frames (evaluated at 0.143) and FSC between the final 3D map and the fitted model (evaluated at 0.5). (D) Zoomed-in views of the final 3D density map from panel A, displayed at two contour levels. (E) Superimposition of the high-contour level map from panel D onto its fitted model.

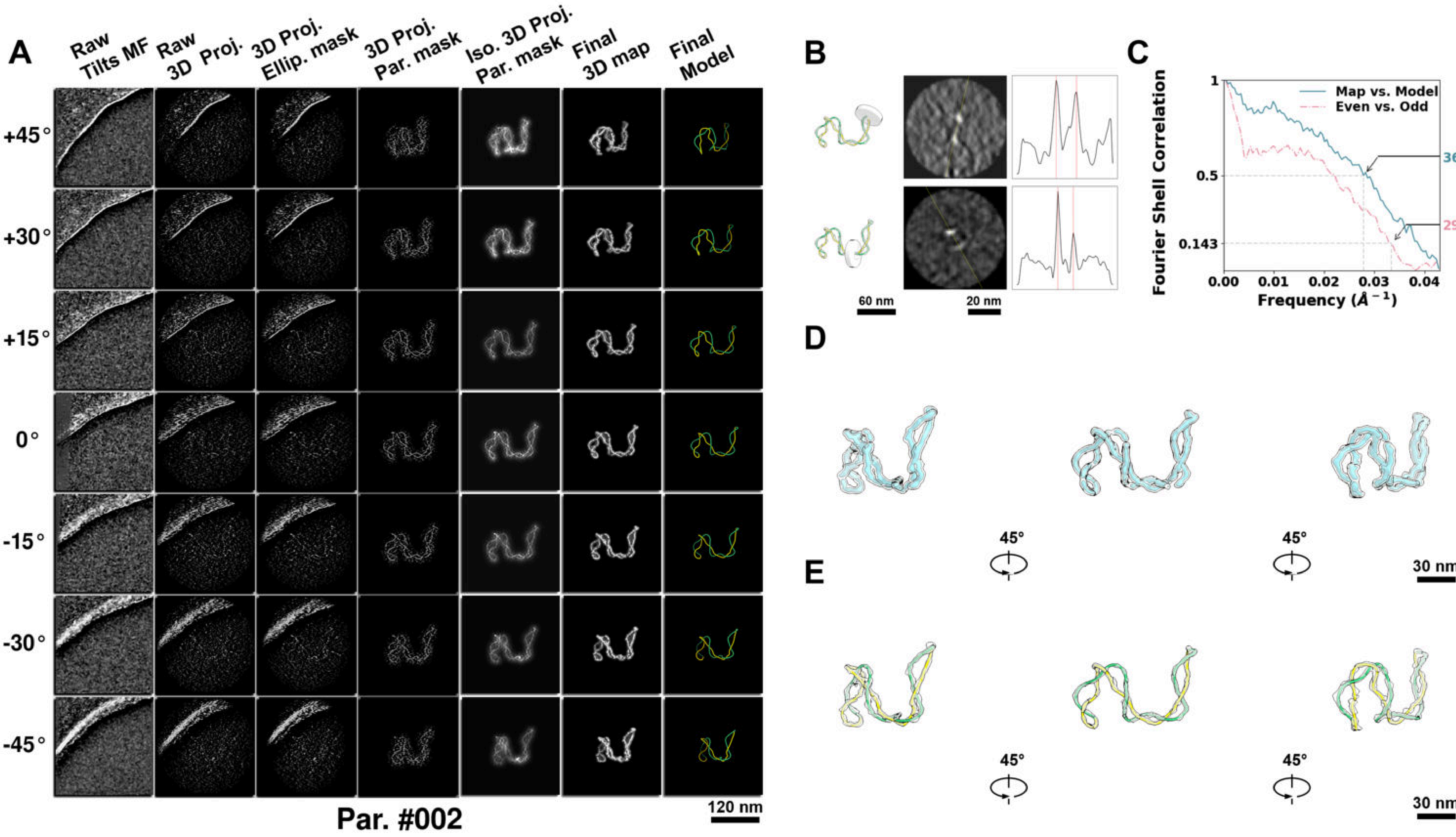

Supplementary Particle Figure 2. Cryo-ET 3D reconstruction of an individual P.LS particle.
(A) 3D reconstruction of the plasmid particle (index no. 2). The first column shows seven representative tilt images from +45° to -45° in step of 15°. The second, third, and fourth columns show 3D projections of the particle with spherical, ellipsoidal (thinner along the z-dimension), and particle-shaped masks, respectively. The fifth column displays the 3D projections of the enhanced and IsoNet missing-wedge-corrected particle. The sixth and seventh columns present the final 3D map and the flexibly fitted model, respectively. (B) Two cross-sectional views (12 nm thickness) of the plasmid density map along its plectoneme axis are shown in the left-middle panel. The intensity profile along the line crossing the two high-density DNA spots is displayed in the right panel. (C) Resolution assessment of the final 3D map using Fourier shell correlation (FSC). Two criteria are shown: FSC between two half-maps reconstructed from even and odd frames (evaluated at 0.143) and FSC between the final 3D map and the fitted model (evaluated at 0.5). (D) Zoomed-in views of the final 3D density map from panel A, displayed at two contour levels. (E) Superimposition of the high-contour level map from panel D onto its fitted model.

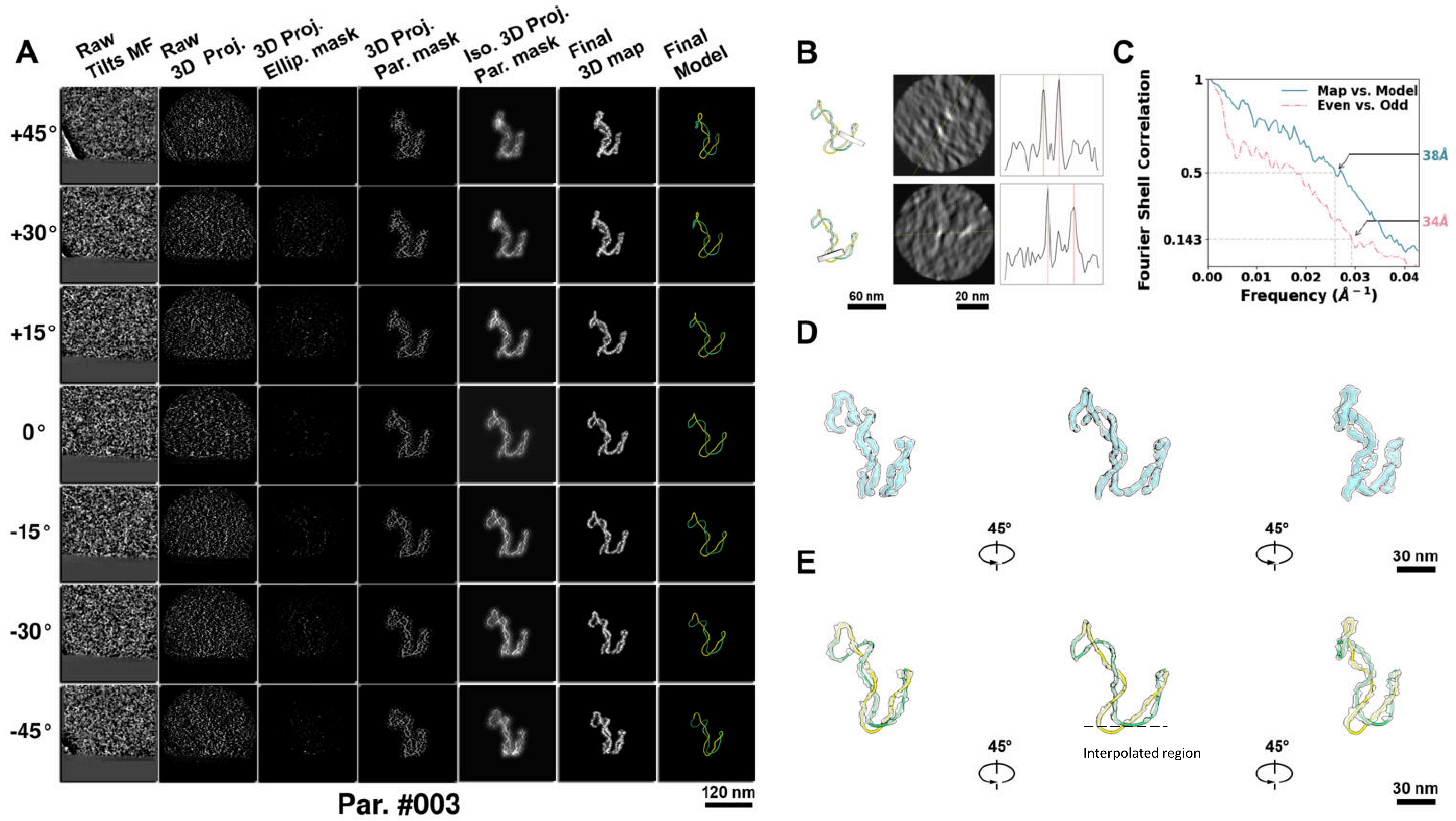

Supplementary Particle Figure 3. Cryo-ET 3D reconstruction of an individual P.LS particle.
(A) 3D reconstruction of the plasmid particle (index no. 3). The first column shows seven representative tilt images from +45° to -45° in step of 15°. The second, third, and fourth columns show 3D projections of the particle with spherical, ellipsoidal (thinner along the z-dimension), and particle-shaped masks, respectively. The fifth column displays the 3D projections of the enhanced and IsoNet missing-wedge-corrected particle. The sixth and seventh columns present the final 3D map and the flexibly fitted model, respectively. (B) Two cross-sectional views (12 nm thickness) of the plasmid density map along its plectoneme axis are shown in the left-middle panel. The intensity profile along the line crossing the two high-density DNA spots is displayed in the right panel. (C) Resolution assessment of the final 3D map using Fourier shell correlation (FSC). Two criteria are shown: FSC between two half-maps reconstructed from even and odd frames (evaluated at 0.143) and FSC between the final 3D map and the fitted model (evaluated at 0.5). (D) Zoomed-in views of the final 3D density map from panel A, displayed at two contour levels. (E) Superimposition of the high-contour level map from panel D onto its fitted model.

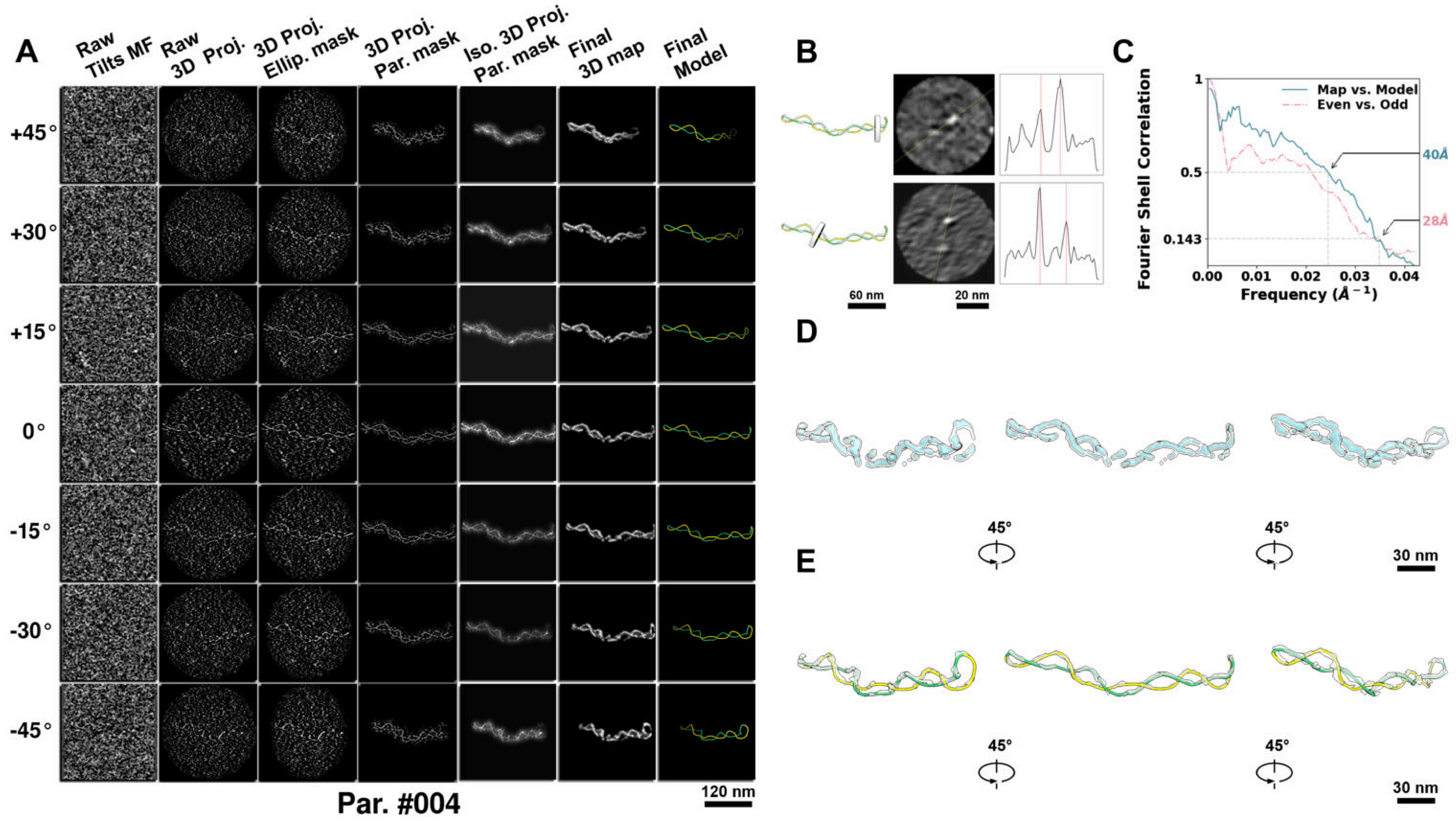

Supplementary Particle Figure 4. Cryo-ET 3D reconstruction of an individual P.LS particle.
(A) 3D reconstruction of the plasmid particle (index no. 4). The first column shows seven representative tilt images from +45° to -45° in step of 15°. The second, third, and fourth columns show 3D projections of the particle with spherical, ellipsoidal (thinner along the z-dimension), and particle-shaped masks, respectively. The fifth column displays the 3D projections of the enhanced and IsoNet missing-wedge-corrected particle. The sixth and seventh columns present the final 3D map and the flexibly fitted model, respectively. (B) Two cross-sectional views (12 nm thickness) of the plasmid density map along its plectoneme axis are shown in the left-middle panel. The intensity profile along the line crossing the two high-density DNA spots is displayed in the right panel. (C) Resolution assessment of the final 3D map using Fourier shell correlation (FSC). Two criteria are shown: FSC between two half-maps reconstructed from even and odd frames (evaluated at 0.143) and FSC between the final 3D map and the fitted model (evaluated at 0.5). (D) Zoomed-in views of the final 3D density map from panel A, displayed at two contour levels. (E) Superimposition of the high-contour level map from panel D onto its fitted model.

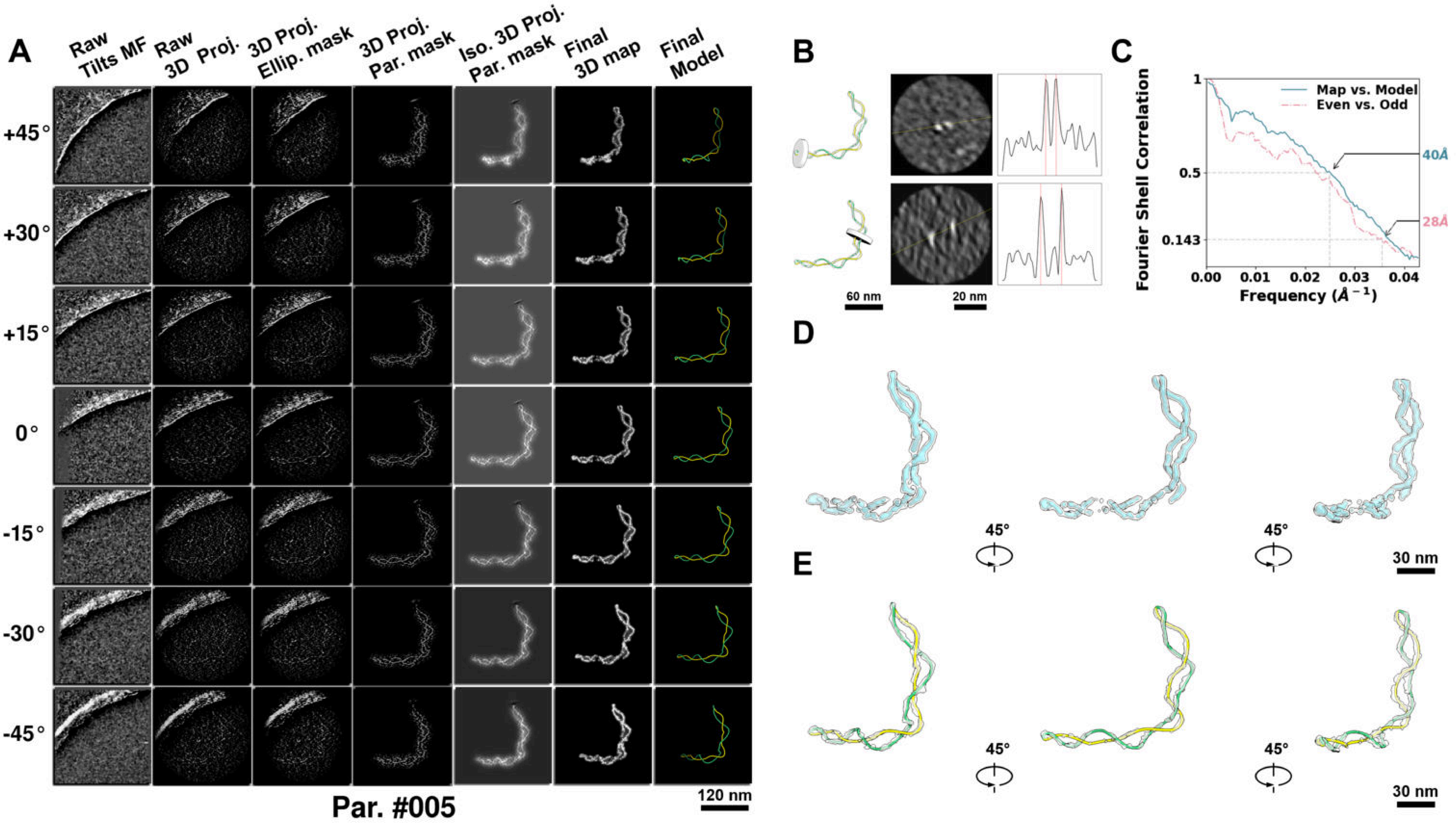

Supplementary Particle Figure 5. Cryo-ET 3D reconstruction of an individual P.LS particle.
(A) 3D reconstruction of the plasmid particle (index no. 5). The first column shows seven representative tilt images from +45° to -45° in step of 15°. The second, third, and fourth columns show 3D projections of the particle with spherical, ellipsoidal (thinner along the z-dimension), and particle-shaped masks, respectively. The fifth column displays the 3D projections of the enhanced and IsoNet missing-wedge-corrected particle. The sixth and seventh columns present the final 3D map and the flexibly fitted model, respectively. (B) Two cross-sectional views (12 nm thickness) of the plasmid density map along its plectoneme axis are shown in the left-middle panel. The intensity profile along the line crossing the two high-density DNA spots is displayed in the right panel. (C) Resolution assessment of the final 3D map using Fourier shell correlation (FSC). Two criteria are shown: FSC between two half-maps reconstructed from even and odd frames (evaluated at 0.143) and FSC between the final 3D map and the fitted model (evaluated at 0.5). (D) Zoomed-in views of the final 3D density map from panel A, displayed at two contour levels. (E) Superimposition of the high-contour level map from panel D onto its fitted model.

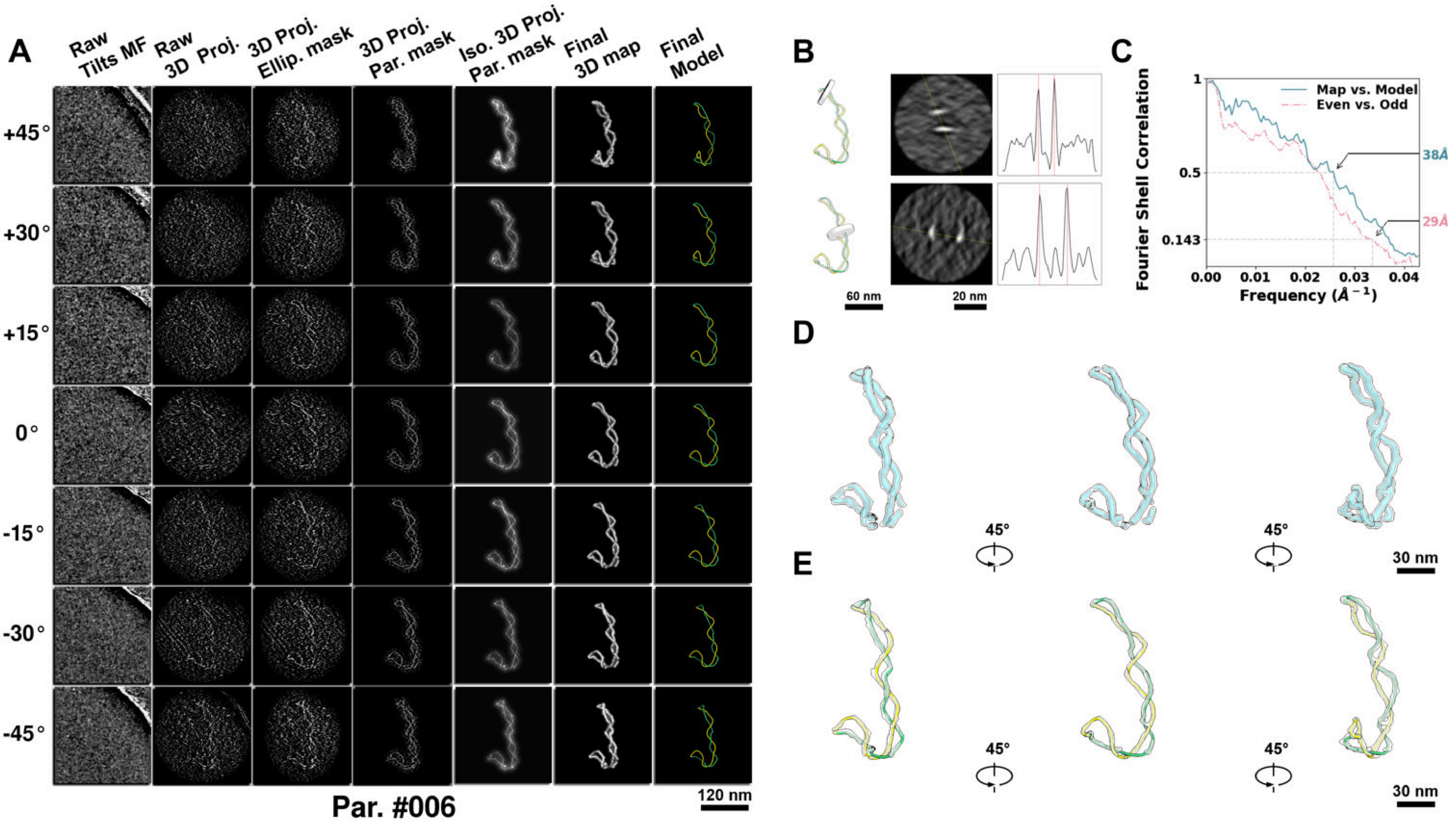

Supplementary Particle Figure 6. Cryo-ET 3D reconstruction of an individual P.LS particle.
(A) 3D reconstruction of the plasmid particle (index no. 6). The first column shows seven representative tilt images from +45° to -45° in step of 15°. The second, third, and fourth columns show 3D projections of the particle with spherical, ellipsoidal (thinner along the z-dimension), and particle-shaped masks, respectively. The fifth column displays the 3D projections of the enhanced and IsoNet missing-wedge-corrected particle. The sixth and seventh columns present the final 3D map and the flexibly fitted model, respectively. (B) Two cross-sectional views (12 nm thickness) of the plasmid density map along its plectoneme axis are shown in the left-middle panel. The intensity profile along the line crossing the two high-density DNA spots is displayed in the right panel. (C) Resolution assessment of the final 3D map using Fourier shell correlation (FSC). Two criteria are shown: FSC between two half-maps reconstructed from even and odd frames (evaluated at 0.143) and FSC between the final 3D map and the fitted model (evaluated at 0.5). (D) Zoomed-in views of the final 3D density map from panel A, displayed at two contour levels. (E) Superimposition of the high-contour level map from panel D onto its fitted model.

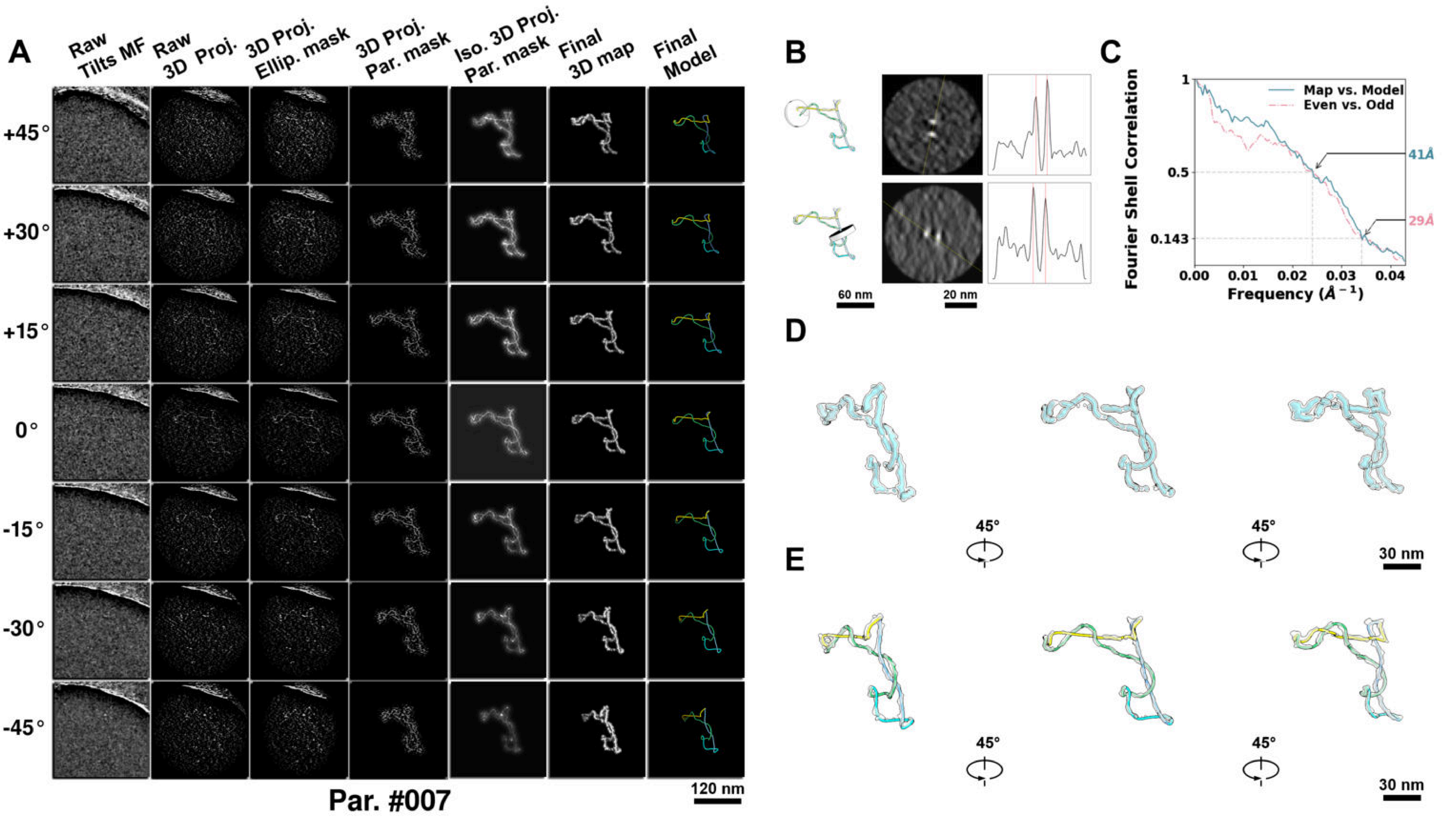

Supplementary Particle Figure 7. Cryo-ET 3D reconstruction of an individual P.LS particle.
(A) 3D reconstruction of the plasmid particle (index no. 7). The first column shows seven representative tilt images from +45° to -45° in step of 15°. The second, third, and fourth columns show 3D projections of the particle with spherical, ellipsoidal (thinner along the z-dimension), and particle-shaped masks, respectively. The fifth column displays the 3D projections of the enhanced and IsoNet missing-wedge-corrected particle. The sixth and seventh columns present the final 3D map and the flexibly fitted model, respectively. (B) Two cross-sectional views (12 nm thickness) of the plasmid density map along its plectoneme axis are shown in the left-middle panel. The intensity profile along the line crossing the two high-density DNA spots is displayed in the right panel. (C) Resolution assessment of the final 3D map using Fourier shell correlation (FSC). Two criteria are shown: FSC between two half-maps reconstructed from even and odd frames (evaluated at 0.143) and FSC between the final 3D map and the fitted model (evaluated at 0.5). (D) Zoomed-in views of the final 3D density map from panel A, displayed at two contour levels. (E) Superimposition of the high-contour level map from panel D onto its fitted model.

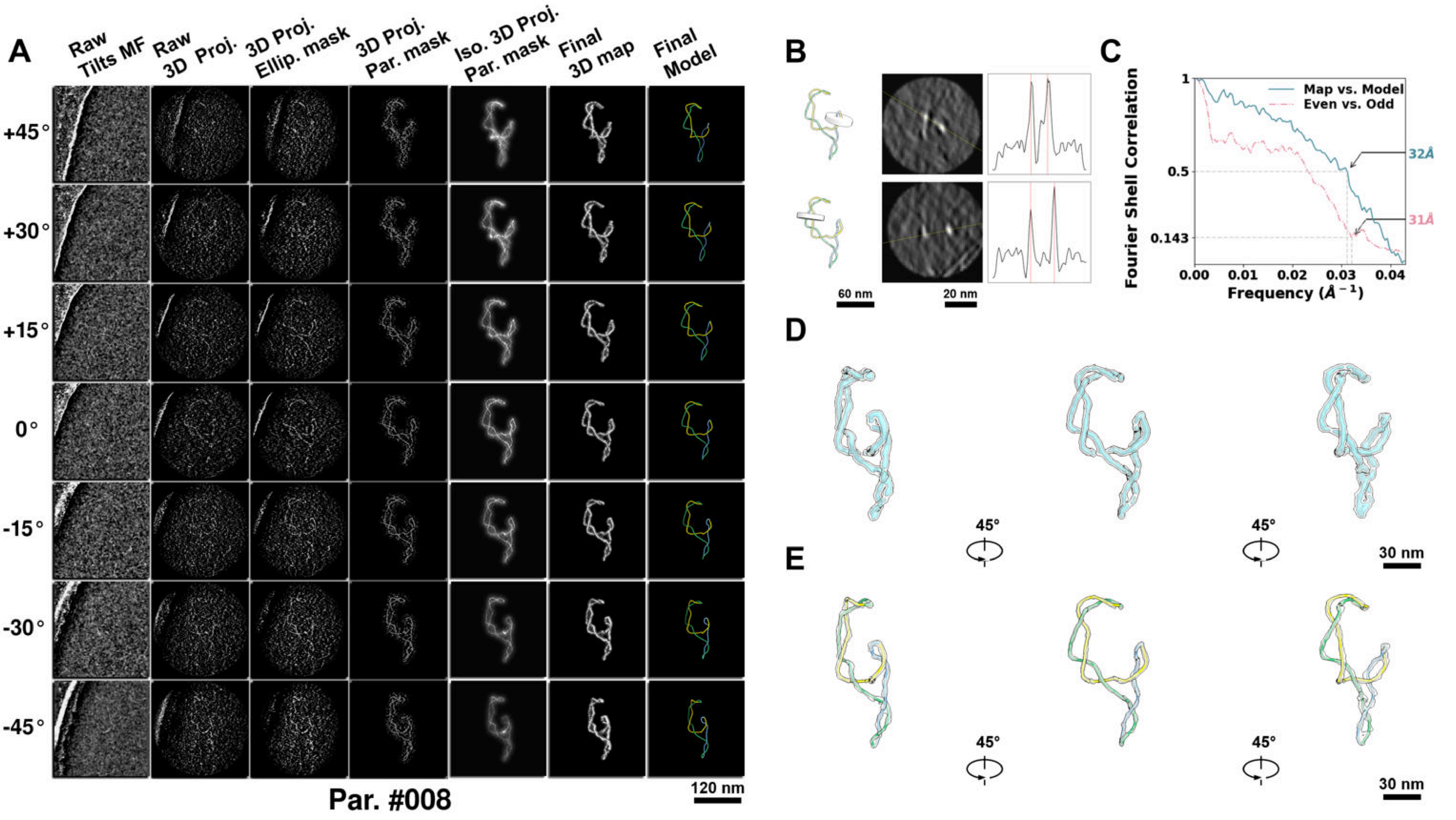

Supplementary Particle Figure 8. Cryo-ET 3D reconstruction of an individual P.LS particle.
(A) 3D reconstruction of the plasmid particle (index no. 8). The first column shows seven representative tilt images from +45° to -45° in step of 15°. The second, third, and fourth columns show 3D projections of the particle with spherical, ellipsoidal (thinner along the z-dimension), and particle-shaped masks, respectively. The fifth column displays the 3D projections of the enhanced and IsoNet missing-wedge-corrected particle. The sixth and seventh columns present the final 3D map and the flexibly fitted model, respectively. (B) Two cross-sectional views (12 nm thickness) of the plasmid density map along its plectoneme axis are shown in the left-middle panel. The intensity profile along the line crossing the two high-density DNA spots is displayed in the right panel. (C) Resolution assessment of the final 3D map using Fourier shell correlation (FSC). Two criteria are shown: FSC between two half-maps reconstructed from even and odd frames (evaluated at 0.143) and FSC between the final 3D map and the fitted model (evaluated at 0.5). (D) Zoomed-in views of the final 3D density map from panel A, displayed at two contour levels. (E) Superimposition of the high-contour level map from panel D onto its fitted model.

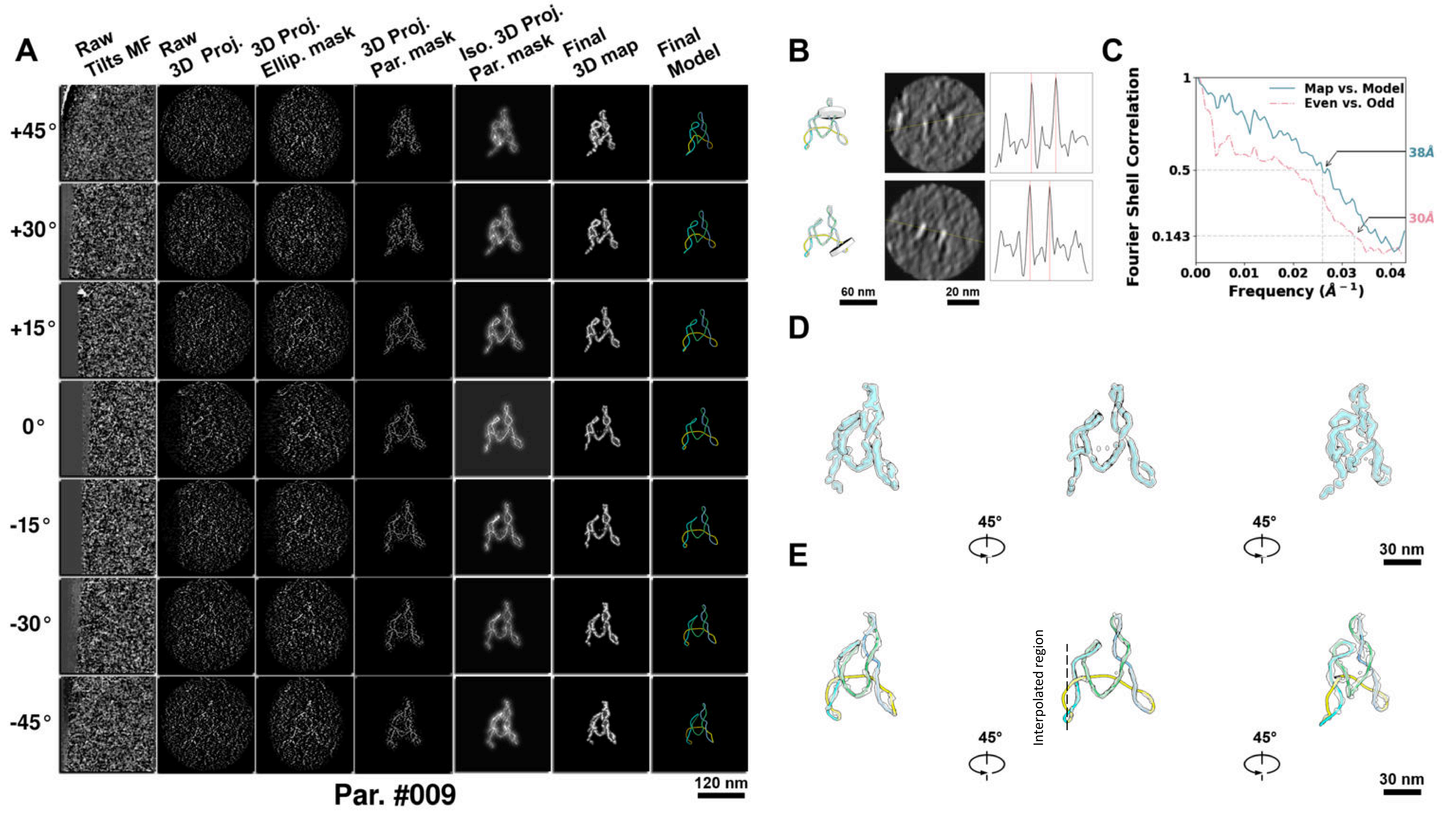

Supplementary Particle Figure 9. Cryo-ET 3D reconstruction of an individual P.LS particle.
(A) 3D reconstruction of the plasmid particle (index no. 9). The first column shows seven representative tilt images from +45° to -45° in step of 15°. The second, third, and fourth columns show 3D projections of the particle with spherical, ellipsoidal (thinner along the z-dimension), and particle-shaped masks, respectively. The fifth column displays the 3D projections of the enhanced and IsoNet missing-wedge-corrected particle. The sixth and seventh columns present the final 3D map and the flexibly fitted model, respectively. (B) Two cross-sectional views (12 nm thickness) of the plasmid density map along its plectoneme axis are shown in the left-middle panel. The intensity profile along the line crossing the two high-density DNA spots is displayed in the right panel. (C) Resolution assessment of the final 3D map using Fourier shell correlation (FSC). Two criteria are shown: FSC between two half-maps reconstructed from even and odd frames (evaluated at 0.143) and FSC between the final 3D map and the fitted model (evaluated at 0.5). (D) Zoomed-in views of the final 3D density map from panel A, displayed at two contour levels. (E) Superimposition of the high-contour level map from panel D onto its fitted model.

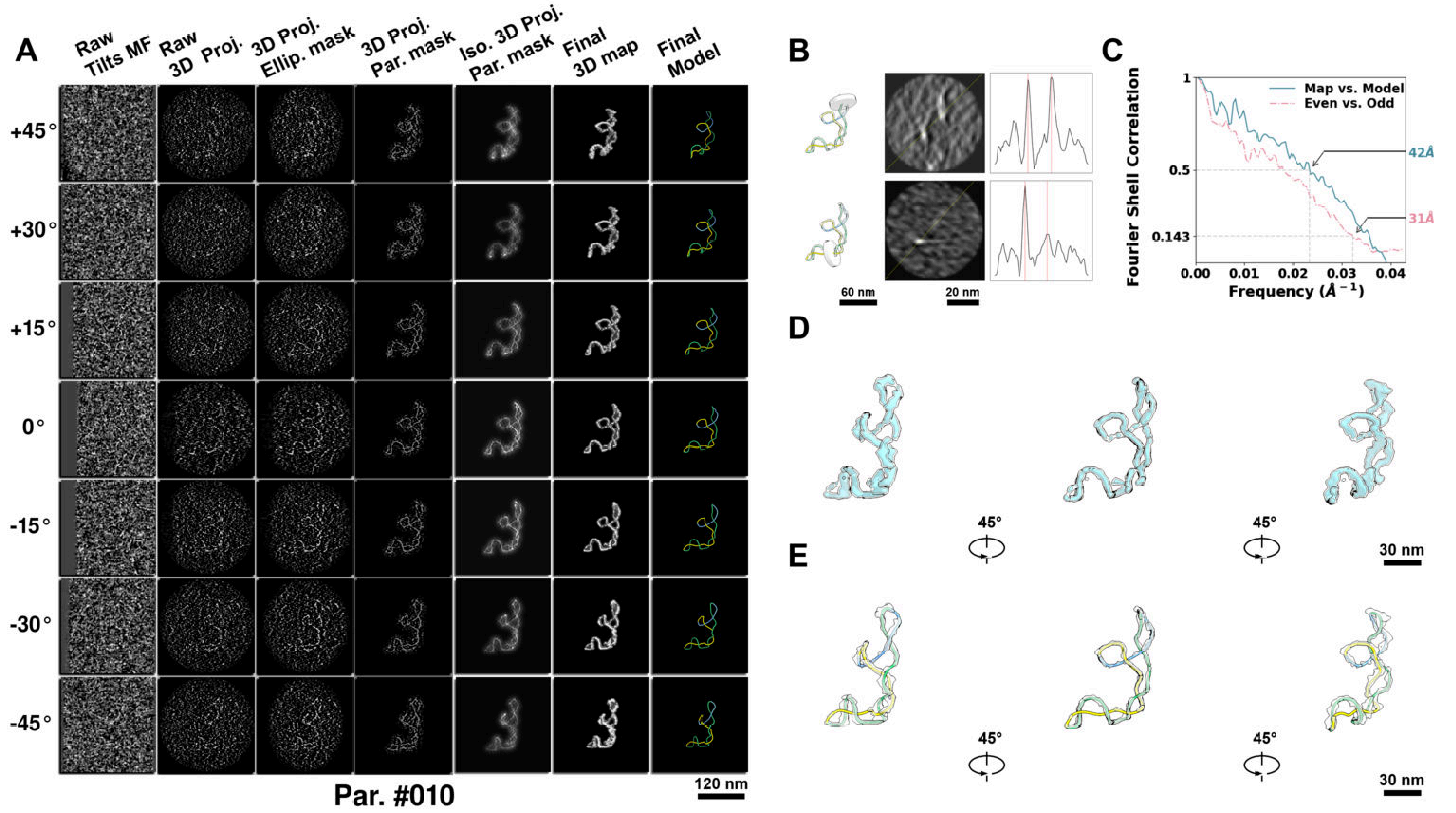

Supplementary Particle Figure 10. Cryo-ET 3D reconstruction of an individual P.LS particle.
(A) 3D reconstruction of the plasmid particle (index no. 10). The first column shows seven representative tilt images from +45° to -45° in step of 15°. The second, third, and fourth columns show 3D projections of the particle with spherical, ellipsoidal (thinner along the z-dimension), and particle-shaped masks, respectively. The fifth column displays the 3D projections of the enhanced and IsoNet missing-wedge-corrected particle. The sixth and seventh columns present the final 3D map and the flexibly fitted model, respectively. (B) Two cross-sectional views (12 nm thickness) of the plasmid density map along its plectoneme axis are shown in the left-middle panel. The intensity profile along the line crossing the two high-density DNA spots is displayed in the right panel. (C) Resolution assessment of the final 3D map using Fourier shell correlation (FSC). Two criteria are shown: FSC between two half-maps reconstructed from even and odd frames (evaluated at 0.143) and FSC between the final 3D map and the fitted model (evaluated at 0.5). (D) Zoomed-in views of the final 3D density map from panel A, displayed at two contour levels. (E) Superimposition of the high-contour level map from panel D onto its fitted model.

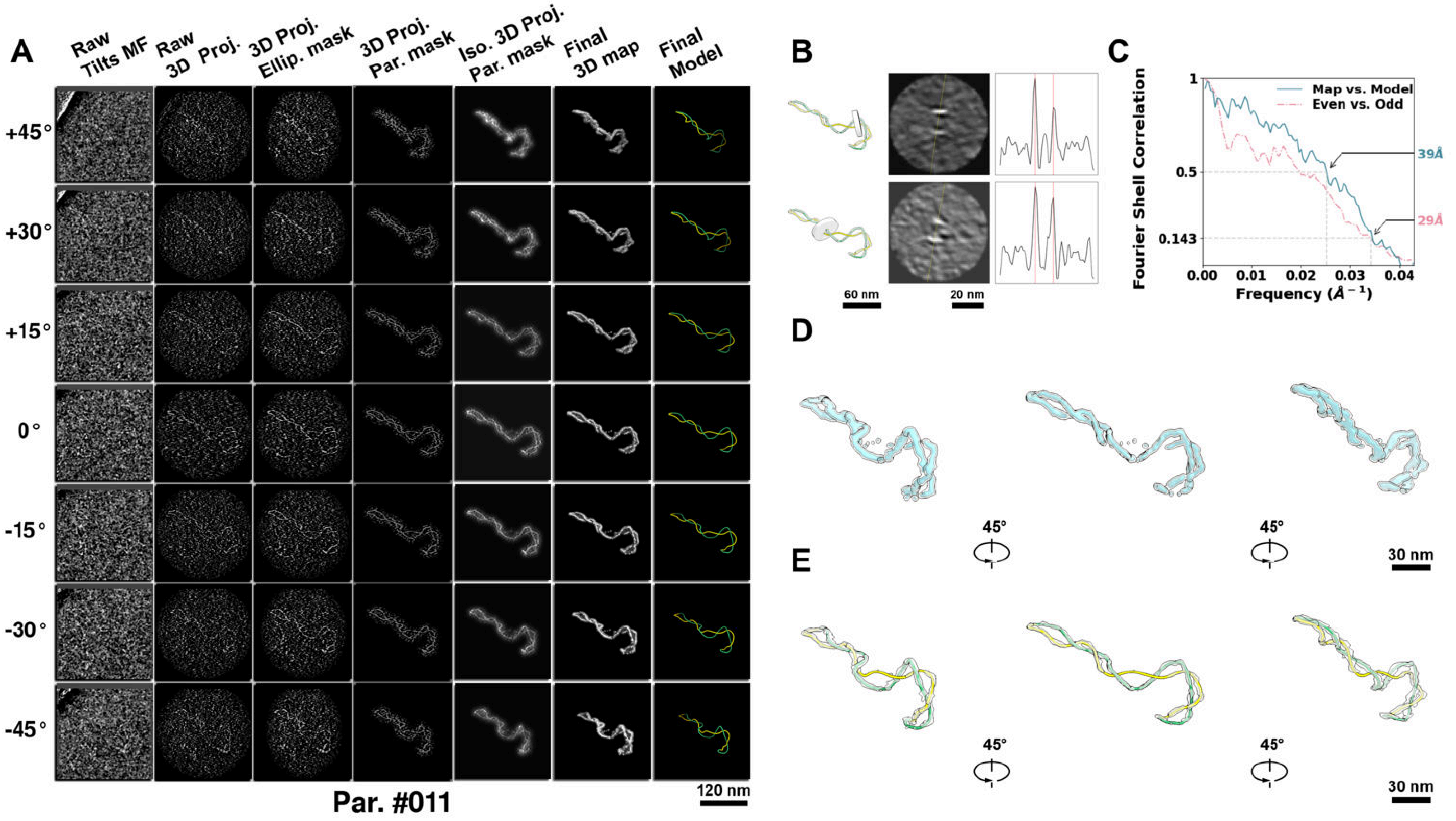

Supplementary Particle Figure 11. Cryo-ET 3D reconstruction of an individual P.LS particle.
(A) 3D reconstruction of the plasmid particle (index no. 11). The first column shows seven representative tilt images from +45° to -45° in step of 15°. The second, third, and fourth columns show 3D projections of the particle with spherical, ellipsoidal (thinner along the z-dimension), and particle-shaped masks, respectively. The fifth column displays the 3D projections of the enhanced and IsoNet missing-wedge-corrected particle. The sixth and seventh columns present the final 3D map and the flexibly fitted model, respectively. (B) Two cross-sectional views (12 nm thickness) of the plasmid density map along its plectoneme axis are shown in the left-middle panel. The intensity profile along the line crossing the two high-density DNA spots is displayed in the right panel. (C) Resolution assessment of the final 3D map using Fourier shell correlation (FSC). Two criteria are shown: FSC between two half-maps reconstructed from even and odd frames (evaluated at 0.143) and FSC between the final 3D map and the fitted model (evaluated at 0.5). (D) Zoomed-in views of the final 3D density map from panel A, displayed at two contour levels. (E) Superimposition of the high-contour level map from panel D onto its fitted model.

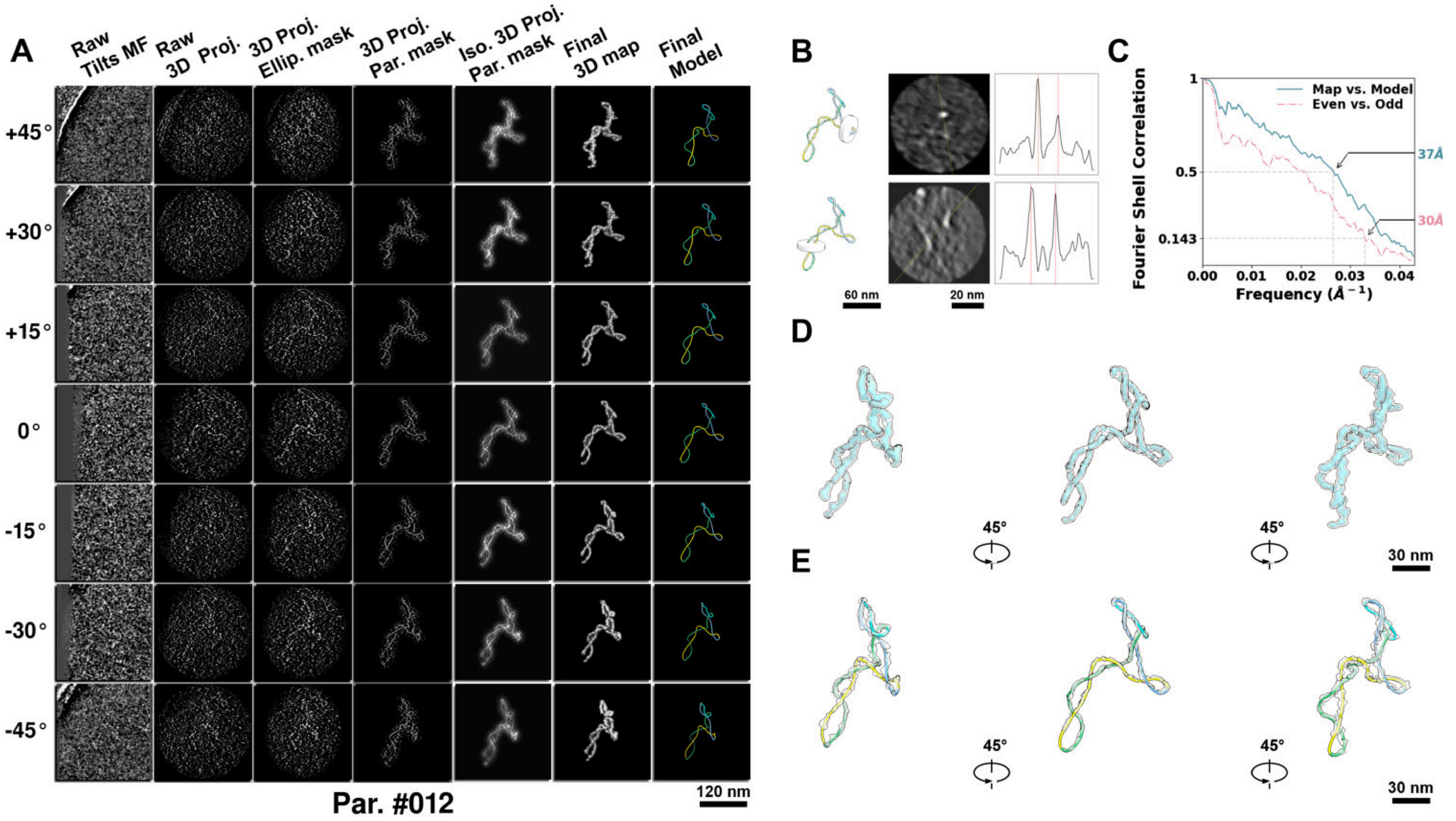

Supplementary Particle Figure 12. Cryo-ET 3D reconstruction of an individual P.LS particle.
(A) 3D reconstruction of the plasmid particle (index no. 12). The first column shows seven representative tilt images from +45° to -45° in step of 15°. The second, third, and fourth columns show 3D projections of the particle with spherical, ellipsoidal (thinner along the z-dimension), and particle-shaped masks, respectively. The fifth column displays the 3D projections of the enhanced and IsoNet missing-wedge-corrected particle. The sixth and seventh columns present the final 3D map and the flexibly fitted model, respectively. (B) Two cross-sectional views (12 nm thickness) of the plasmid density map along its plectoneme axis are shown in the left-middle panel. The intensity profile along the line crossing the two high-density DNA spots is displayed in the right panel. (C) Resolution assessment of the final 3D map using Fourier shell correlation (FSC). Two criteria are shown: FSC between two half-maps reconstructed from even and odd frames (evaluated at 0.143) and FSC between the final 3D map and the fitted model (evaluated at 0.5). (D) Zoomed-in views of the final 3D density map from panel A, displayed at two contour levels. (E) Superimposition of the high-contour level map from panel D onto its fitted model.

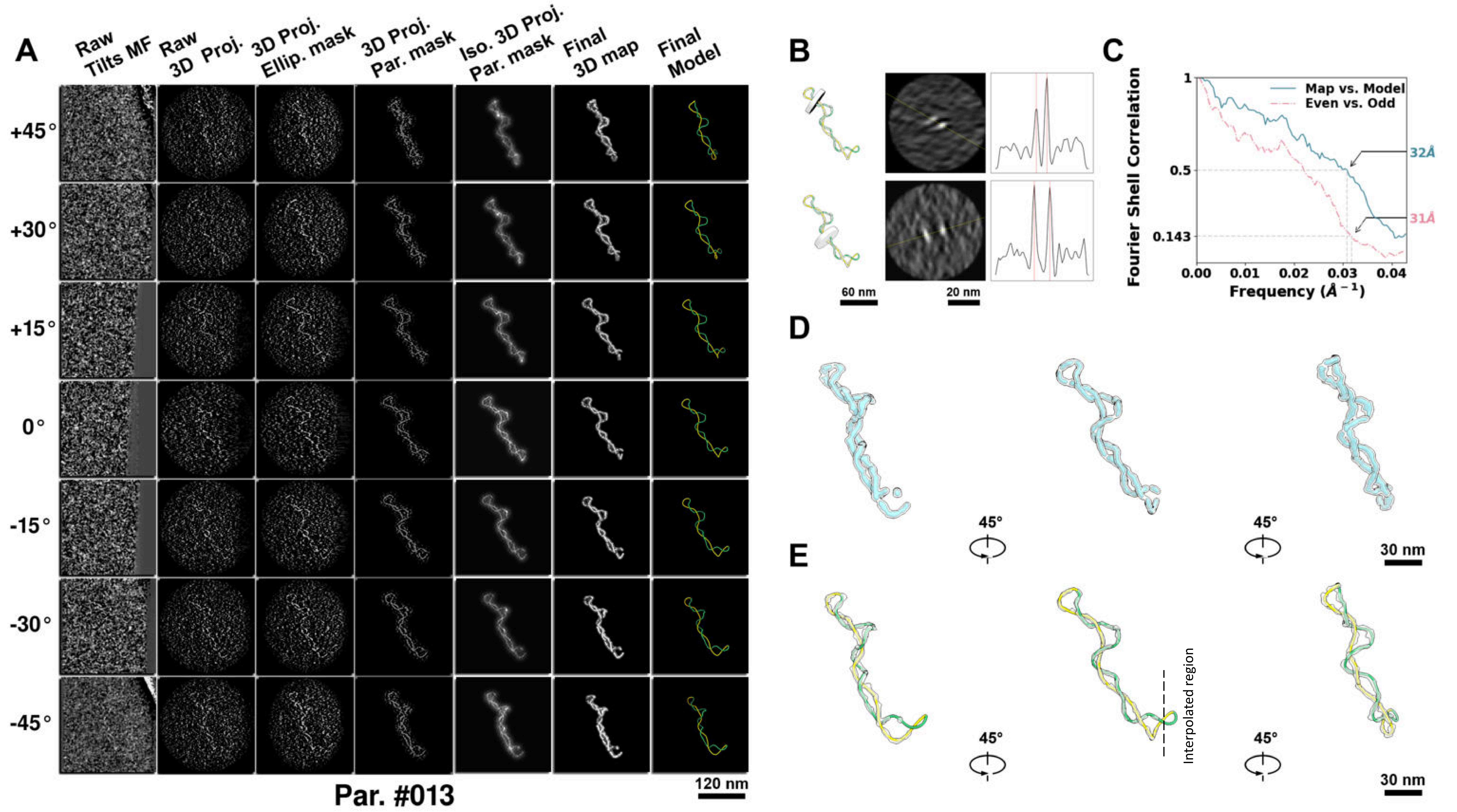

Supplementary Particle Figure 13. Cryo-ET 3D reconstruction of an individual P.LS particle.
(A) 3D reconstruction of the plasmid particle (index no. 13). The first column shows seven representative tilt images from +45° to -45° in step of 15°. The second, third, and fourth columns show 3D projections of the particle with spherical, ellipsoidal (thinner along the z-dimension), and particle-shaped masks, respectively. The fifth column displays the 3D projections of the enhanced and IsoNet missing-wedge-corrected particle. The sixth and seventh columns present the final 3D map and the flexibly fitted model, respectively. (B) Two cross-sectional views (12 nm thickness) of the plasmid density map along its plectoneme axis are shown in the left-middle panel. The intensity profile along the line crossing the two high-density DNA spots is displayed in the right panel. (C) Resolution assessment of the final 3D map using Fourier shell correlation (FSC). Two criteria are shown: FSC between two half-maps reconstructed from even and odd frames (evaluated at 0.143) and FSC between the final 3D map and the fitted model (evaluated at 0.5). (D) Zoomed-in views of the final 3D density map from panel A, displayed at two contour levels. (E) Superimposition of the high-contour level map from panel D onto its fitted model.

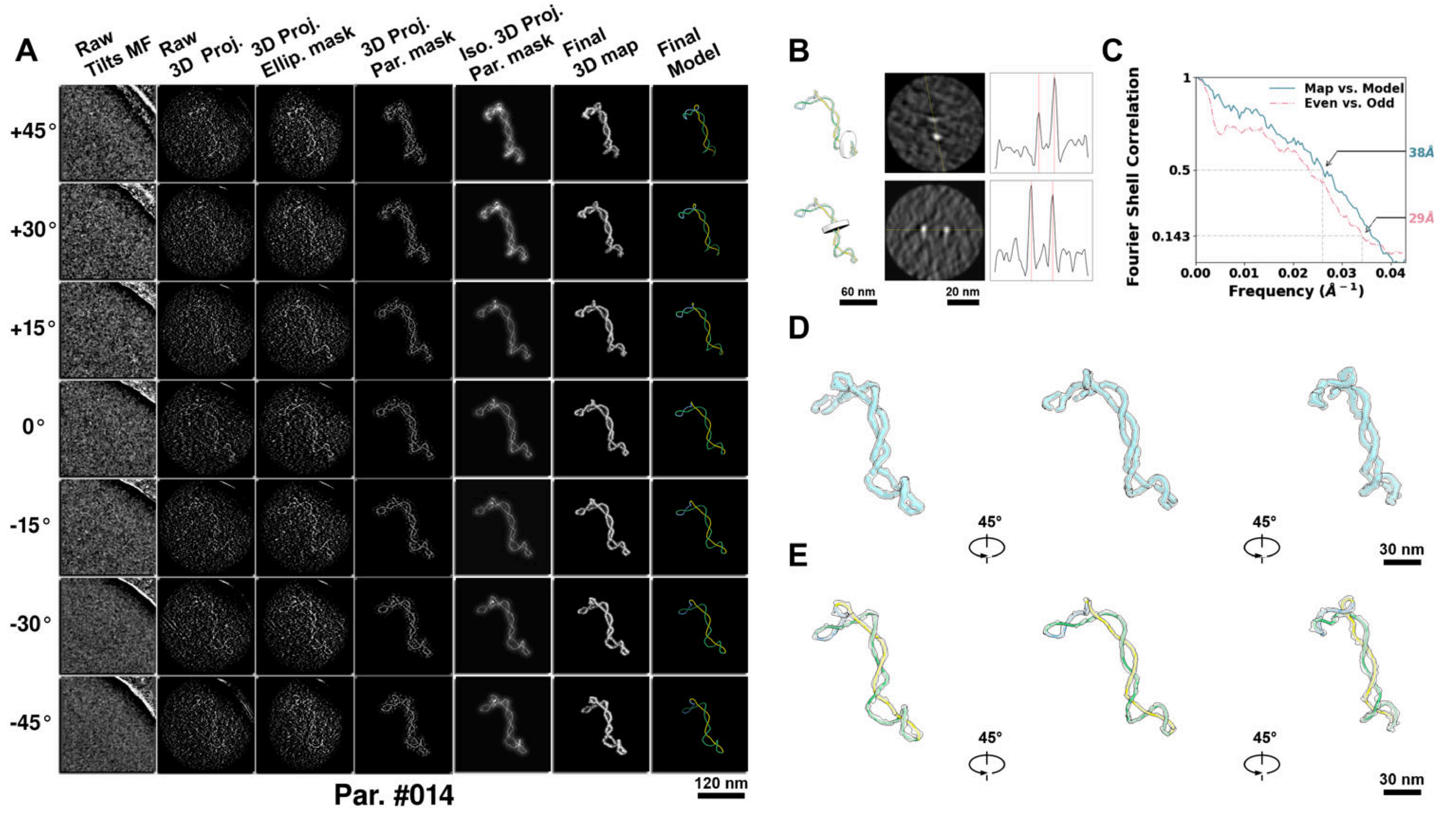

Supplementary Particle Figure 14. Cryo-ET 3D reconstruction of an individual P.LS particle.
(A) 3D reconstruction of the plasmid particle (index no. 14). The first column shows seven representative tilt images from +45° to -45° in step of 15°. The second, third, and fourth columns show 3D projections of the particle with spherical, ellipsoidal (thinner along the z-dimension), and particle-shaped masks, respectively. The fifth column displays the 3D projections of the enhanced and IsoNet missing-wedge-corrected particle. The sixth and seventh columns present the final 3D map and the flexibly fitted model, respectively. (B) Two cross-sectional views (12 nm thickness) of the plasmid density map along its plectoneme axis are shown in the left-middle panel. The intensity profile along the line crossing the two high-density DNA spots is displayed in the right panel. (C) Resolution assessment of the final 3D map using Fourier shell correlation (FSC). Two criteria are shown: FSC between two half-maps reconstructed from even and odd frames (evaluated at 0.143) and FSC between the final 3D map and the fitted model (evaluated at 0.5). (D) Zoomed-in views of the final 3D density map from panel A, displayed at two contour levels. (E) Superimposition of the high-contour level map from panel D onto its fitted model.

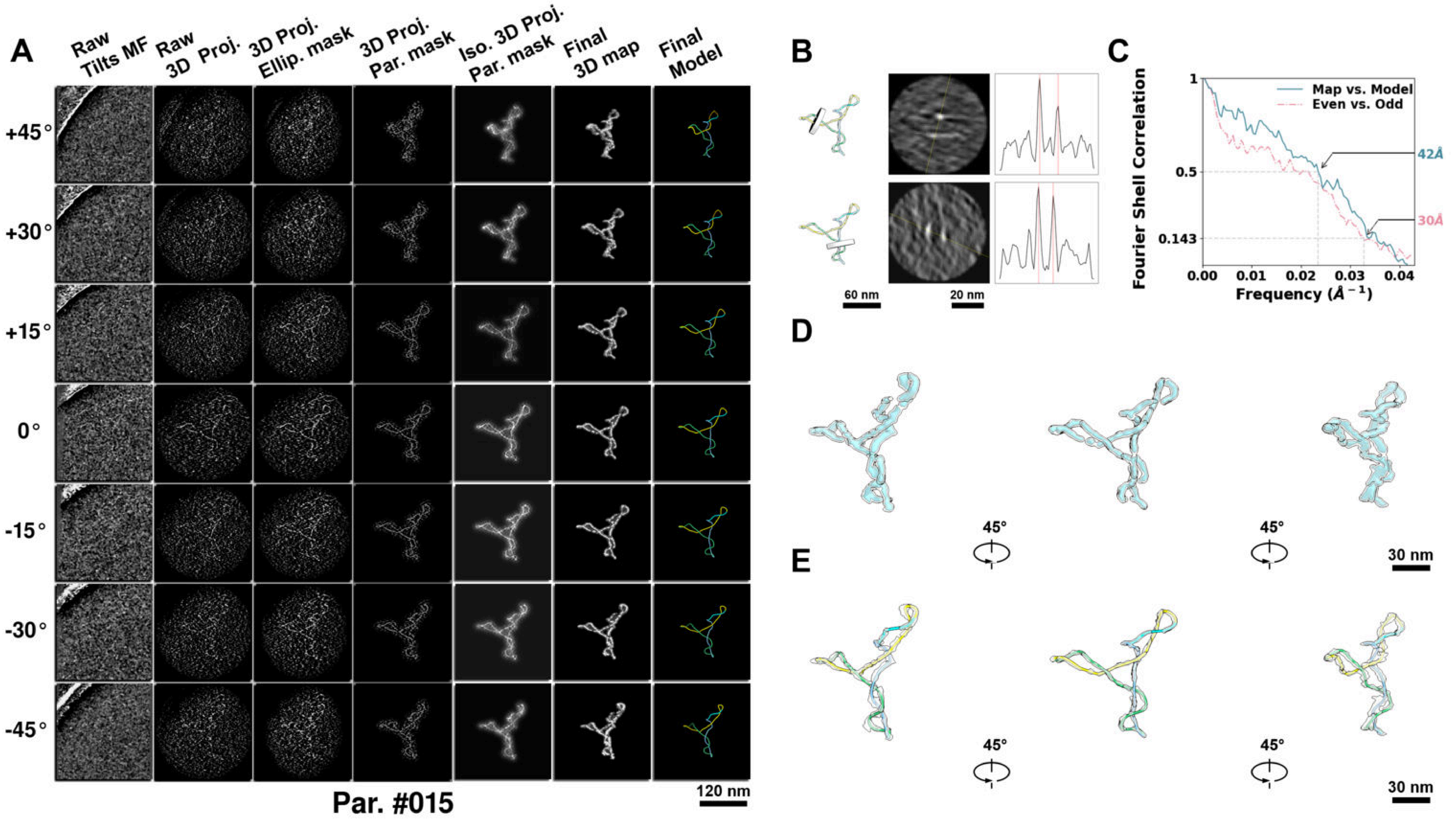

Supplementary Particle Figure 15. Cryo-ET 3D reconstruction of an individual P.LS particle.
(A) 3D reconstruction of the plasmid particle (index no. 15). The first column shows seven representative tilt images from +45° to -45° in step of 15°. The second, third, and fourth columns show 3D projections of the particle with spherical, ellipsoidal (thinner along the z-dimension), and particle-shaped masks, respectively. The fifth column displays the 3D projections of the enhanced and IsoNet missing-wedge-corrected particle. The sixth and seventh columns present the final 3D map and the flexibly fitted model, respectively. (B) Two cross-sectional views (12 nm thickness) of the plasmid density map along its plectoneme axis are shown in the left-middle panel. The intensity profile along the line crossing the two high-density DNA spots is displayed in the right panel. (C) Resolution assessment of the final 3D map using Fourier shell correlation (FSC). Two criteria are shown: FSC between two half-maps reconstructed from even and odd frames (evaluated at 0.143) and FSC between the final 3D map and the fitted model (evaluated at 0.5). (D) Zoomed-in views of the final 3D density map from panel A, displayed at two contour levels. (E) Superimposition of the high-contour level map from panel D onto its fitted model.

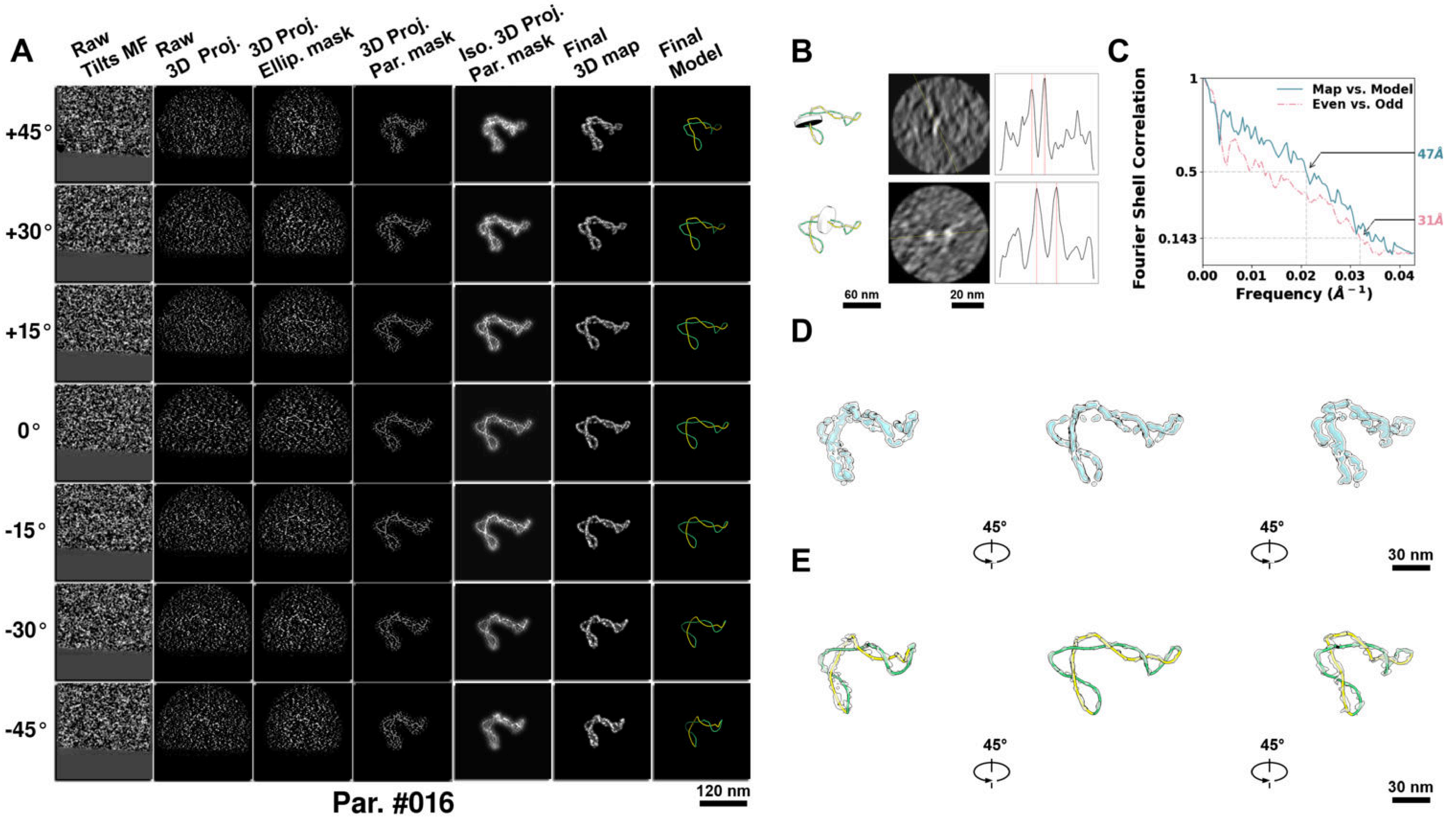

Supplementary Particle Figure 16. Cryo-ET 3D reconstruction of an individual P.LS particle.
(A) 3D reconstruction of the plasmid particle (index no. 16). The first column shows seven representative tilt images from +45° to -45° in step of 15°. The second, third, and fourth columns show 3D projections of the particle with spherical, ellipsoidal (thinner along the z-dimension), and particle-shaped masks, respectively. The fifth column displays the 3D projections of the enhanced and IsoNet missing-wedge-corrected particle. The sixth and seventh columns present the final 3D map and the flexibly fitted model, respectively. (B) Two cross-sectional views (12 nm thickness) of the plasmid density map along its plectoneme axis are shown in the left-middle panel. The intensity profile along the line crossing the two high-density DNA spots is displayed in the right panel. (C) Resolution assessment of the final 3D map using Fourier shell correlation (FSC). Two criteria are shown: FSC between two half-maps reconstructed from even and odd frames (evaluated at 0.143) and FSC between the final 3D map and the fitted model (evaluated at 0.5). (D) Zoomed-in views of the final 3D density map from panel A, displayed at two contour levels. (E) Superimposition of the high-contour level map from panel D onto its fitted model.

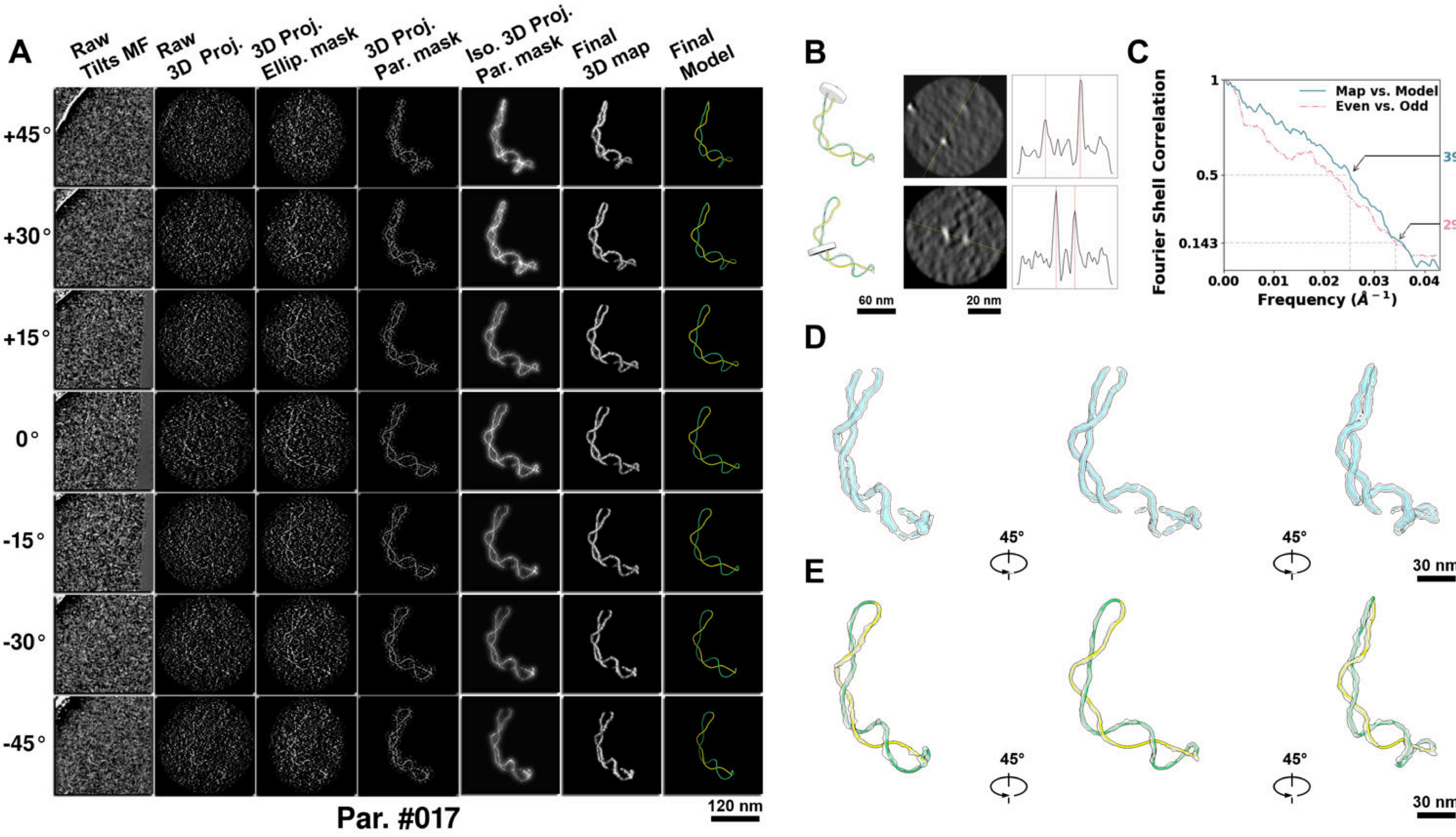

Supplementary Particle Figure 17. Cryo-ET 3D reconstruction of an individual P.LS particle.
(A) 3D reconstruction of the plasmid particle (index no. 17). The first column shows seven representative tilt images from +45° to -45° in step of 15°. The second, third, and fourth columns show 3D projections of the particle with spherical, ellipsoidal (thinner along the z-dimension), and particle-shaped masks, respectively. The fifth column displays the 3D projections of the enhanced and IsoNet missing-wedge-corrected particle. The sixth and seventh columns present the final 3D map and the flexibly fitted model, respectively. (B) Two cross-sectional views (12 nm thickness) of the plasmid density map along its plectoneme axis are shown in the left-middle panel. The intensity profile along the line crossing the two high-density DNA spots is displayed in the right panel. (C) Resolution assessment of the final 3D map using Fourier shell correlation (FSC). Two criteria are shown: FSC between two half-maps reconstructed from even and odd frames (evaluated at 0.143) and FSC between the final 3D map and the fitted model (evaluated at 0.5). (D) Zoomed-in views of the final 3D density map from panel A, displayed at two contour levels. (E) Superimposition of the high-contour level map from panel D onto its fitted model.

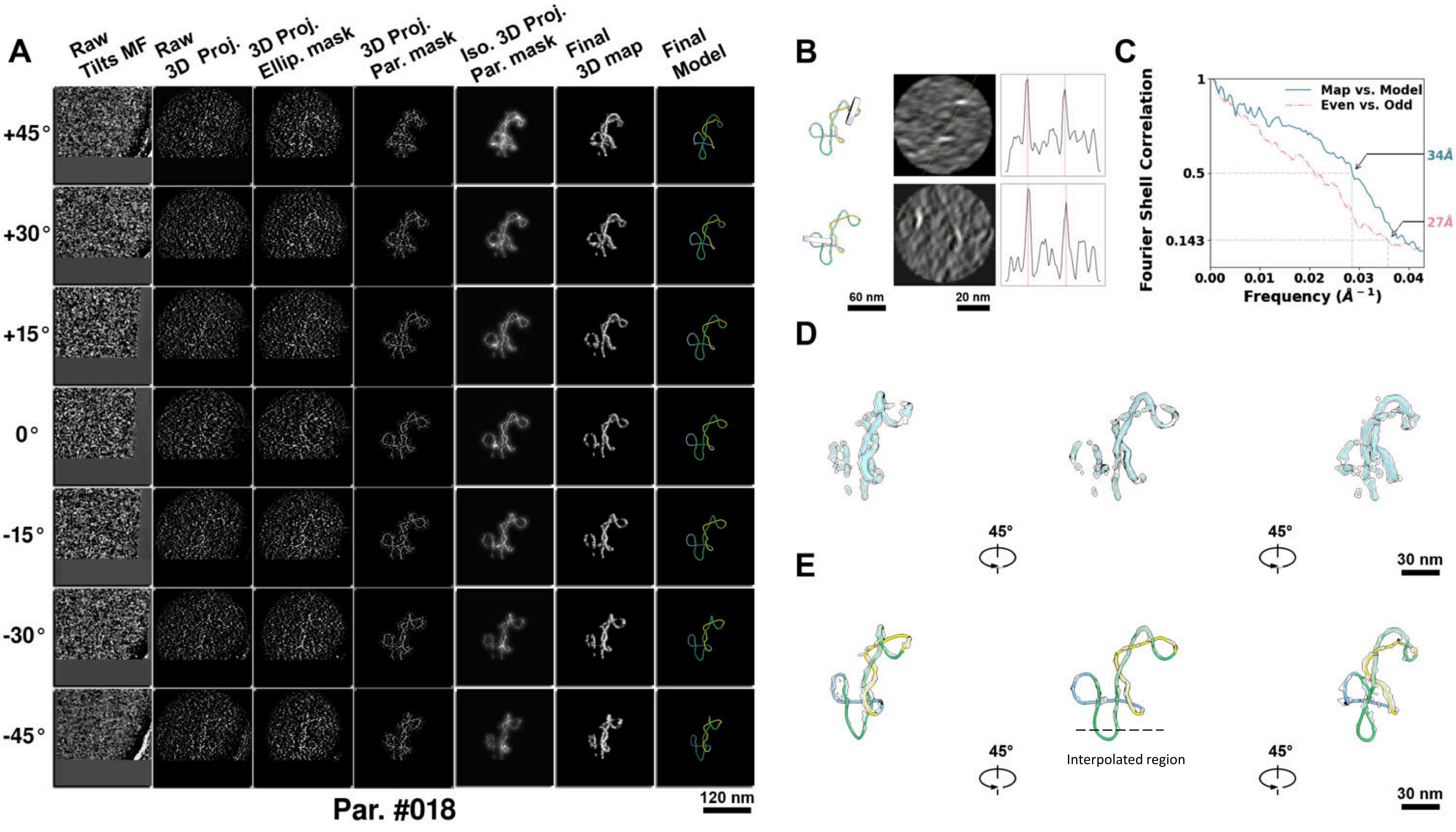

Supplementary Particle Figure 18. Cryo-ET 3D reconstruction of an individual P.LS particle.
(A) 3D reconstruction of the plasmid particle (index no. 18). The first column shows seven representative tilt images from +45° to -45° in step of 15°. The second, third, and fourth columns show 3D projections of the particle with spherical, ellipsoidal (thinner along the z-dimension), and particle-shaped masks, respectively. The fifth column displays the 3D projections of the enhanced and IsoNet missing-wedge-corrected particle. The sixth and seventh columns present the final 3D map and the flexibly fitted model, respectively. (B) Two cross-sectional views (12 nm thickness) of the plasmid density map along its plectoneme axis are shown in the left-middle panel. The intensity profile along the line crossing the two high-density DNA spots is displayed in the right panel. (C) Resolution assessment of the final 3D map using Fourier shell correlation (FSC). Two criteria are shown: FSC between two half-maps reconstructed from even and odd frames (evaluated at 0.143) and FSC between the final 3D map and the fitted model (evaluated at 0.5). (D) Zoomed-in views of the final 3D density map from panel A, displayed at two contour levels. (E) Superimposition of the high-contour level map from panel D onto its fitted model.

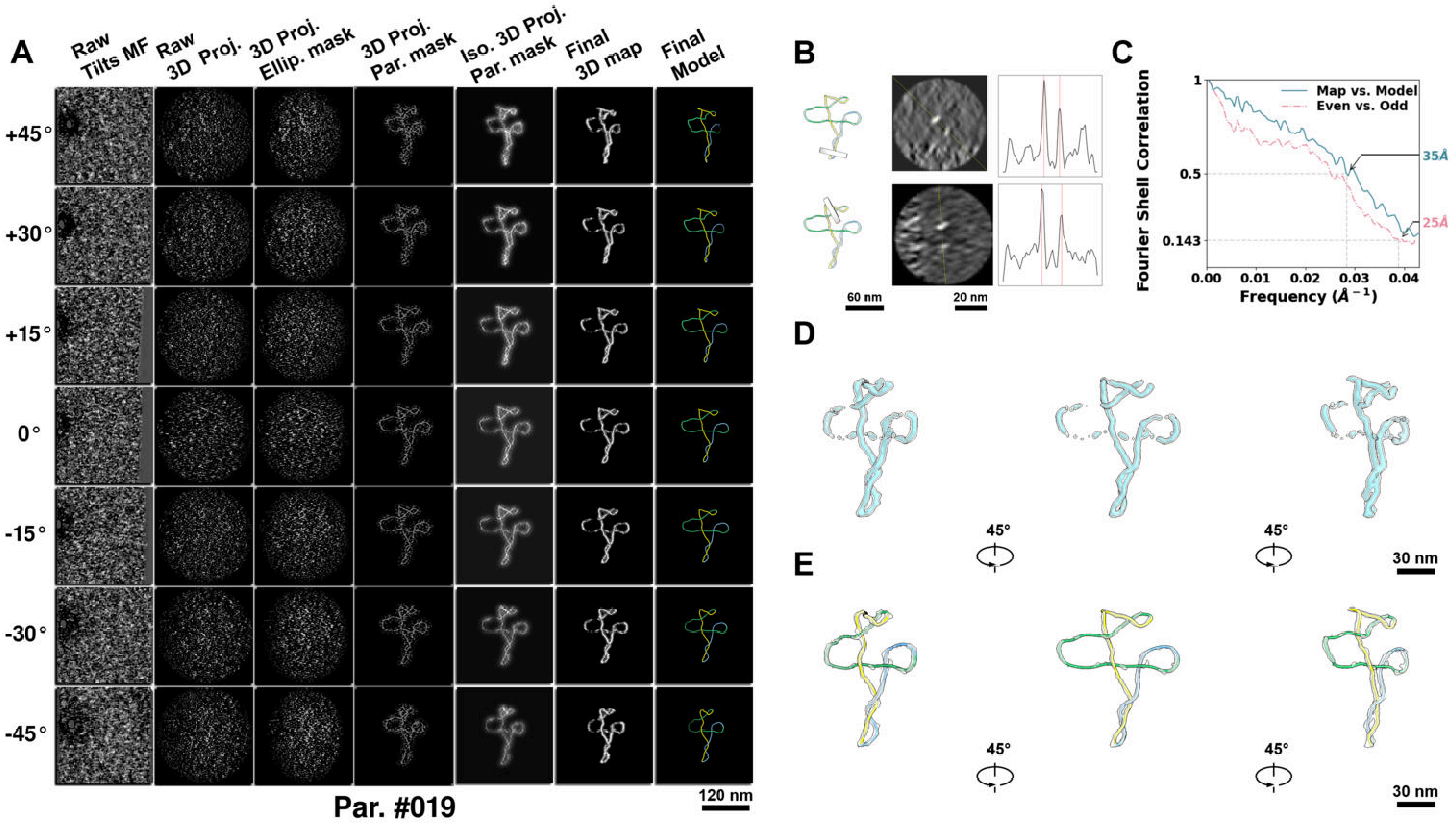

Supplementary Particle Figure 19. Cryo-ET 3D reconstruction of an individual P.LS particle.
(A) 3D reconstruction of the plasmid particle (index no. 19). The first column shows seven representative tilt images from +45° to -45° in step of 15°. The second, third, and fourth columns show 3D projections of the particle with spherical, ellipsoidal (thinner along the z-dimension), and particle-shaped masks, respectively. The fifth column displays the 3D projections of the enhanced and IsoNet missing-wedge-corrected particle. The sixth and seventh columns present the final 3D map and the flexibly fitted model, respectively. (B) Two cross-sectional views (12 nm thickness) of the plasmid density map along its plectoneme axis are shown in the left-middle panel. The intensity profile along the line crossing the two high-density DNA spots is displayed in the right panel. (C) Resolution assessment of the final 3D map using Fourier shell correlation (FSC). Two criteria are shown: FSC between two half-maps reconstructed from even and odd frames (evaluated at 0.143) and FSC between the final 3D map and the fitted model (evaluated at 0.5). (D) Zoomed-in views of the final 3D density map from panel A, displayed at two contour levels. (E) Superimposition of the high-contour level map from panel D onto its fitted model.

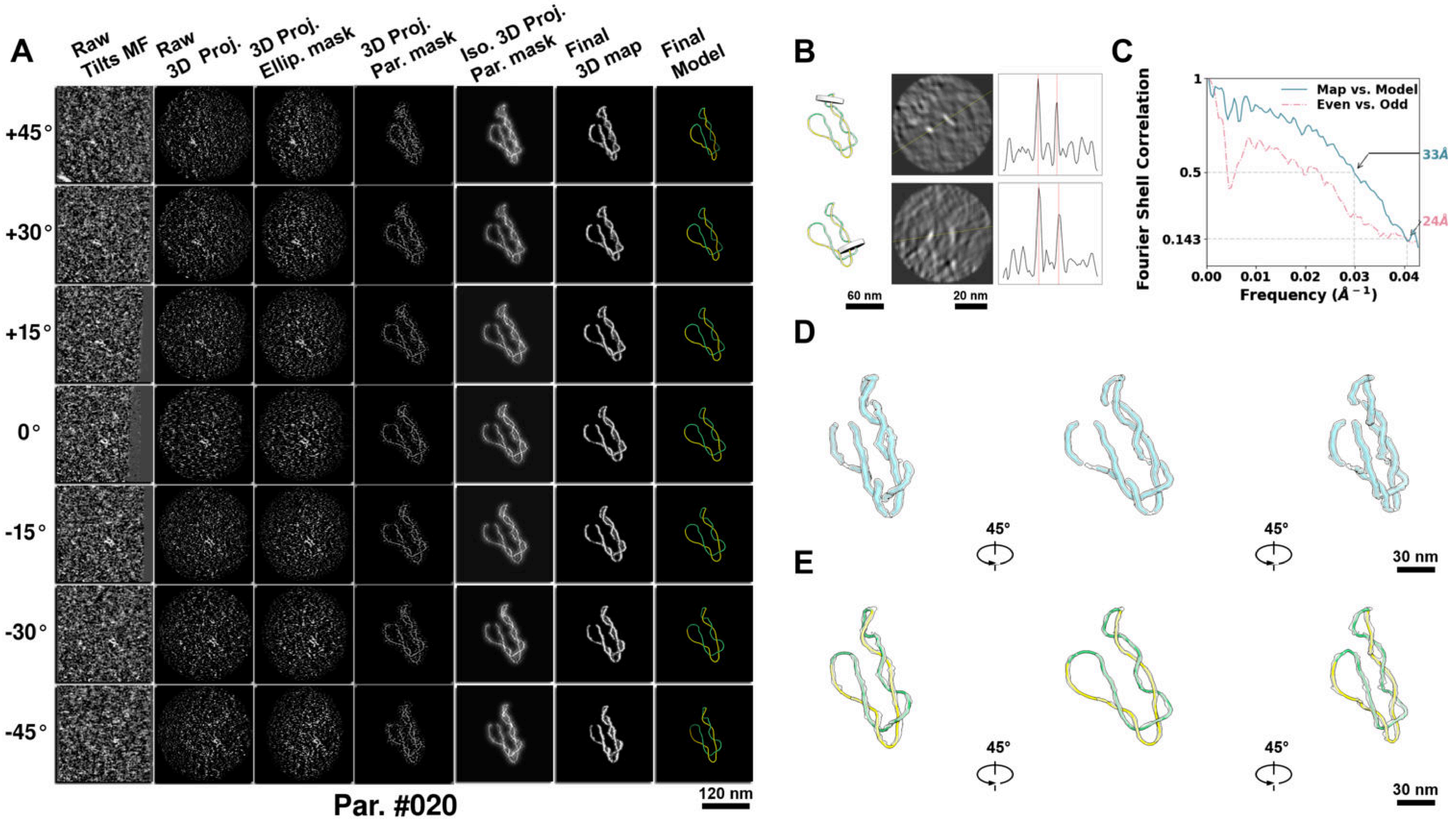

Supplementary Particle Figure 20. Cryo-ET 3D reconstruction of an individual P.LS particle.
(A) 3D reconstruction of the plasmid particle (index no. 20). The first column shows seven representative tilt images from +45° to -45° in step of 15°. The second, third, and fourth columns show 3D projections of the particle with spherical, ellipsoidal (thinner along the z-dimension), and particle-shaped masks, respectively. The fifth column displays the 3D projections of the enhanced and IsoNet missing-wedge-corrected particle. The sixth and seventh columns present the final 3D map and the flexibly fitted model, respectively. (B) Two cross-sectional views (12 nm thickness) of the plasmid density map along its plectoneme axis are shown in the left-middle panel. The intensity profile along the line crossing the two high-density DNA spots is displayed in the right panel. (C) Resolution assessment of the final 3D map using Fourier shell correlation (FSC). Two criteria are shown: FSC between two half-maps reconstructed from even and odd frames (evaluated at 0.143) and FSC between the final 3D map and the fitted model (evaluated at 0.5). (D) Zoomed-in views of the final 3D density map from panel A, displayed at two contour levels. (E) Superimposition of the high-contour level map from panel D onto its fitted model.

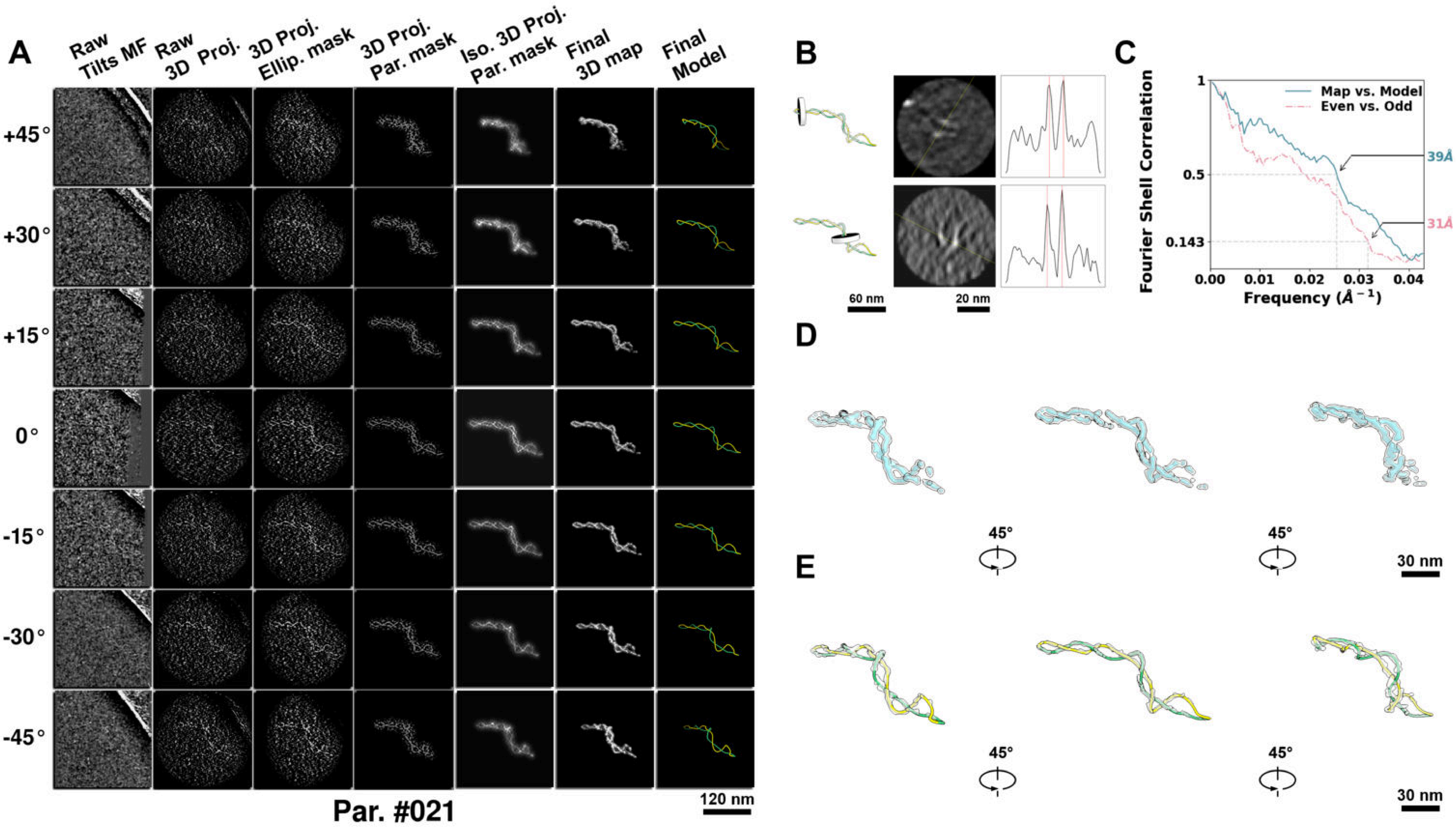

Supplementary Particle Figure 21. Cryo-ET 3D reconstruction of an individual P.LS particle.
(A) 3D reconstruction of the plasmid particle (index no. 21). The first column shows seven representative tilt images from +45° to -45° in step of 15°. The second, third, and fourth columns show 3D projections of the particle with spherical, ellipsoidal (thinner along the z-dimension), and particle-shaped masks, respectively. The fifth column displays the 3D projections of the enhanced and IsoNet missing-wedge-corrected particle. The sixth and seventh columns present the final 3D map and the flexibly fitted model, respectively. (B) Two cross-sectional views (12 nm thickness) of the plasmid density map along its plectoneme axis are shown in the left-middle panel. The intensity profile along the line crossing the two high-density DNA spots is displayed in the right panel. (C) Resolution assessment of the final 3D map using Fourier shell correlation (FSC). Two criteria are shown: FSC between two half-maps reconstructed from even and odd frames (evaluated at 0.143) and FSC between the final 3D map and the fitted model (evaluated at 0.5). (D) Zoomed-in views of the final 3D density map from panel A, displayed at two contour levels. (E) Superimposition of the high-contour level map from panel D onto its fitted model.

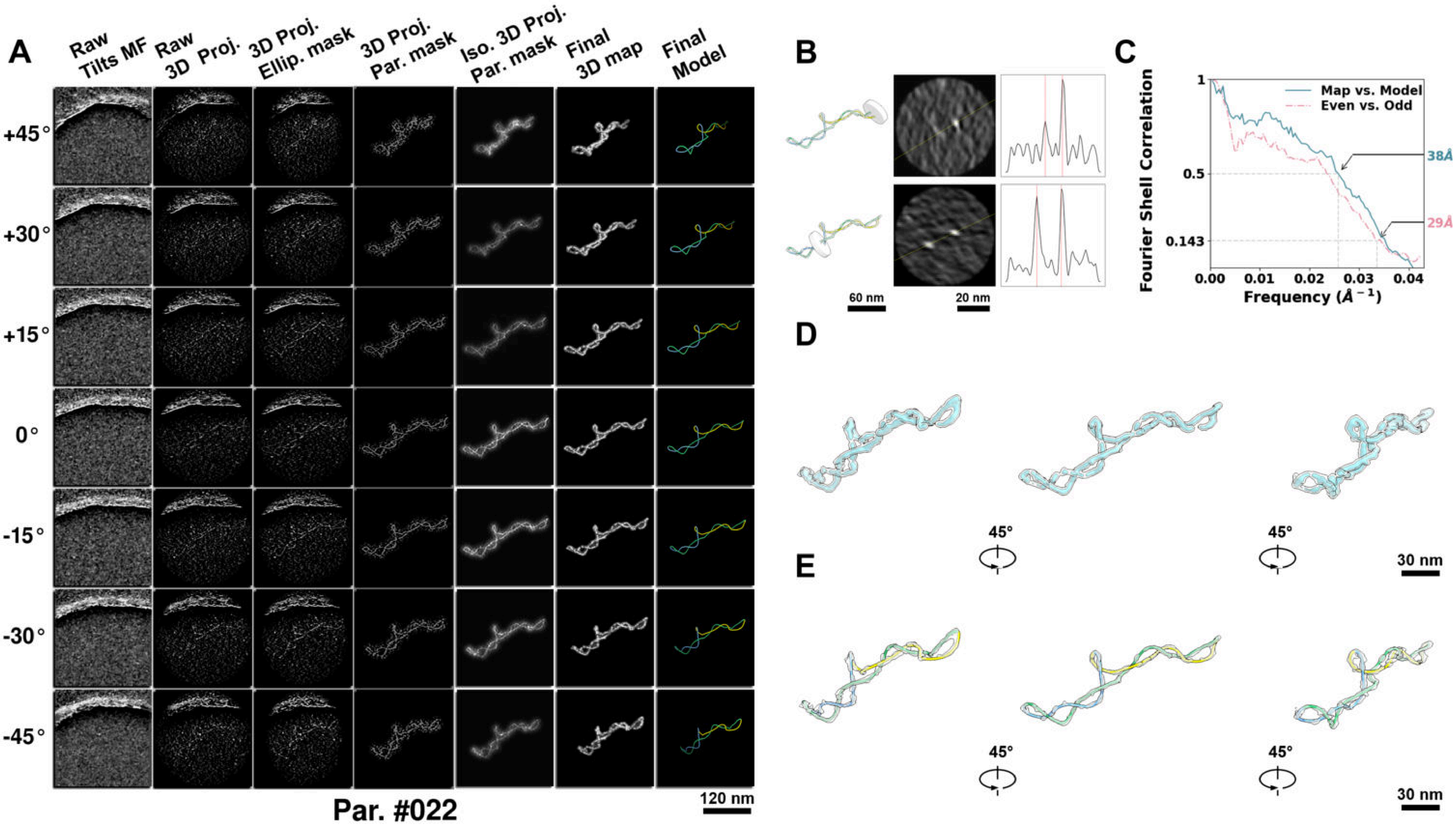

Supplementary Particle Figure 22. Cryo-ET 3D reconstruction of an individual P.LS particle.
(A) 3D reconstruction of the plasmid particle (index no. 22). The first column shows seven representative tilt images from +45° to -45° in step of 15°. The second, third, and fourth columns show 3D projections of the particle with spherical, ellipsoidal (thinner along the z-dimension), and particle-shaped masks, respectively. The fifth column displays the 3D projections of the enhanced and IsoNet missing-wedge-corrected particle. The sixth and seventh columns present the final 3D map and the flexibly fitted model, respectively. (B) Two cross-sectional views (12 nm thickness) of the plasmid density map along its plectoneme axis are shown in the left-middle panel. The intensity profile along the line crossing the two high-density DNA spots is displayed in the right panel. (C) Resolution assessment of the final 3D map using Fourier shell correlation (FSC). Two criteria are shown: FSC between two half-maps reconstructed from even and odd frames (evaluated at 0.143) and FSC between the final 3D map and the fitted model (evaluated at 0.5). (D) Zoomed-in views of the final 3D density map from panel A, displayed at two contour levels. (E) Superimposition of the high-contour level map from panel D onto its fitted model.

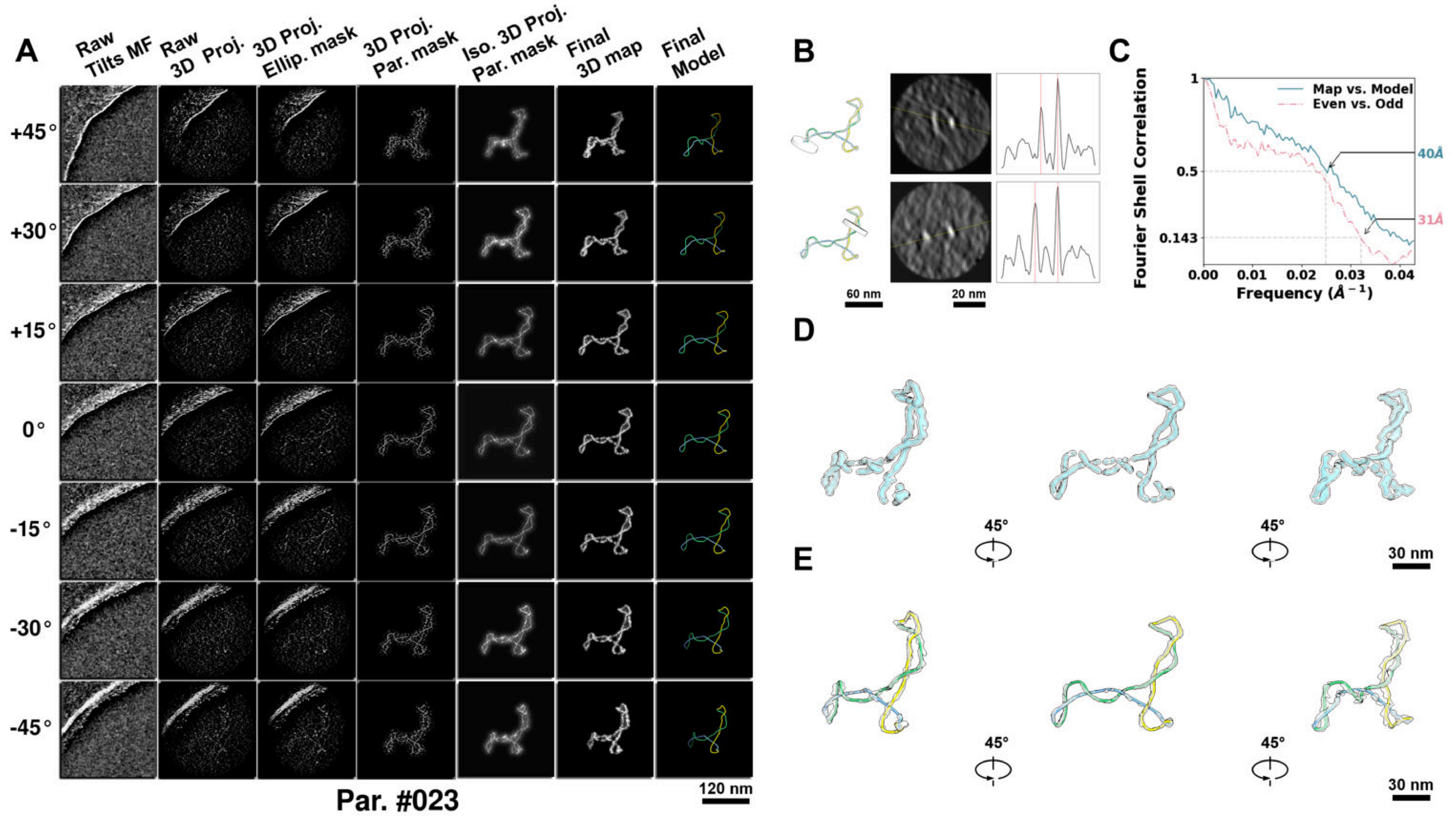

Supplementary Particle Figure 23. Cryo-ET 3D reconstruction of an individual P.LS particle.
(A) 3D reconstruction of the plasmid particle (index no. 23). The first column shows seven representative tilt images from +45° to -45° in step of 15°. The second, third, and fourth columns show 3D projections of the particle with spherical, ellipsoidal (thinner along the z-dimension), and particle-shaped masks, respectively. The fifth column displays the 3D projections of the enhanced and IsoNet missing-wedge-corrected particle. The sixth and seventh columns present the final 3D map and the flexibly fitted model, respectively. (B) Two cross-sectional views (12 nm thickness) of the plasmid density map along its plectoneme axis are shown in the left-middle panel. The intensity profile along the line crossing the two high-density DNA spots is displayed in the right panel. (C) Resolution assessment of the final 3D map using Fourier shell correlation (FSC). Two criteria are shown: FSC between two half-maps reconstructed from even and odd frames (evaluated at 0.143) and FSC between the final 3D map and the fitted model (evaluated at 0.5). (D) Zoomed-in views of the final 3D density map from panel A, displayed at two contour levels. (E) Superimposition of the high-contour level map from panel D onto its fitted model.

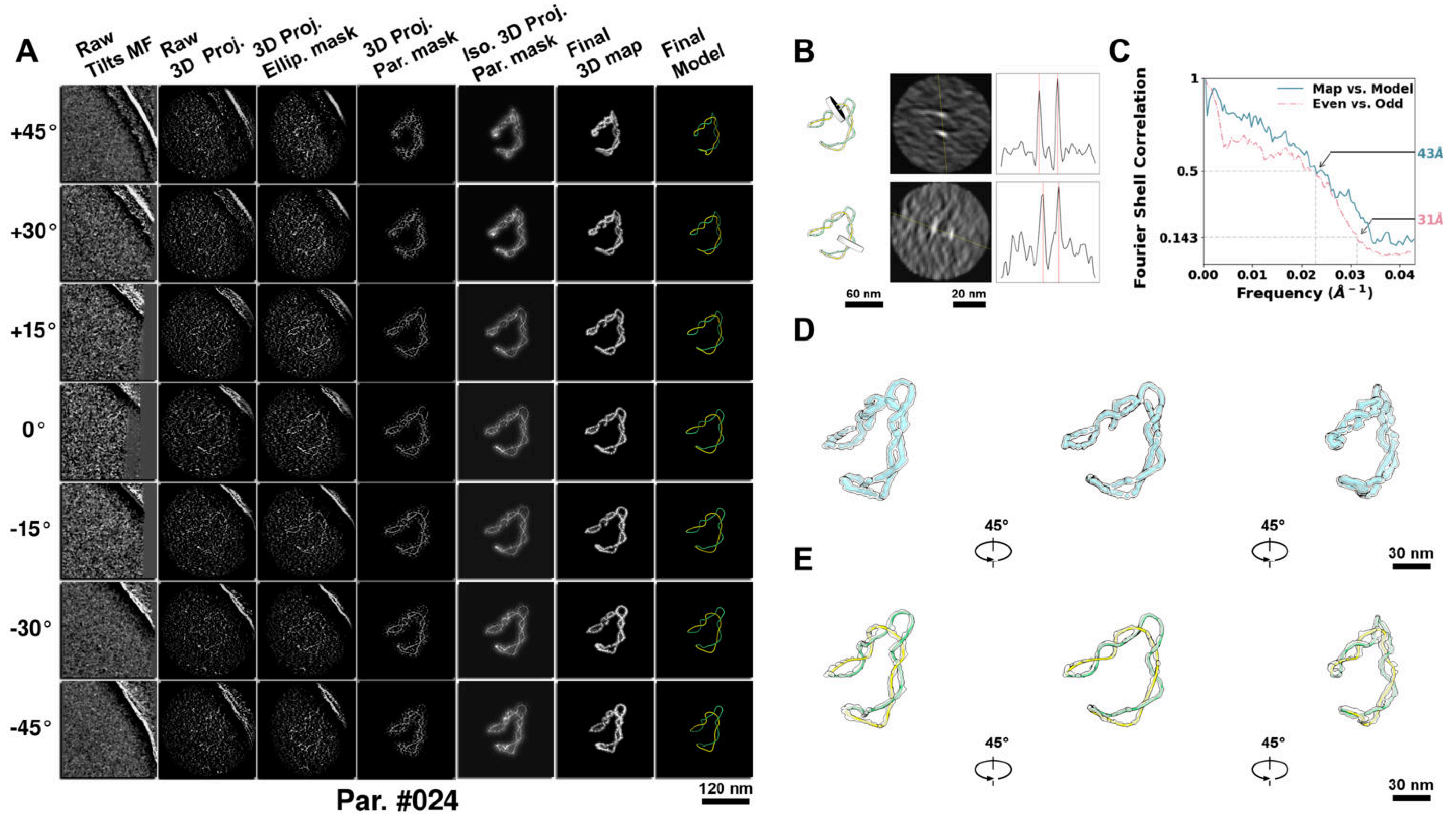

Supplementary Particle Figure 24. Cryo-ET 3D reconstruction of an individual P.LS particle.
(A) 3D reconstruction of the plasmid particle (index no. 24). The first column shows seven representative tilt images from +45° to -45° in step of 15°. The second, third, and fourth columns show 3D projections of the particle with spherical, ellipsoidal (thinner along the z-dimension), and particle-shaped masks, respectively. The fifth column displays the 3D projections of the enhanced and IsoNet missing-wedge-corrected particle. The sixth and seventh columns present the final 3D map and the flexibly fitted model, respectively. (B) Two cross-sectional views (12 nm thickness) of the plasmid density map along its plectoneme axis are shown in the left-middle panel. The intensity profile along the line crossing the two high-density DNA spots is displayed in the right panel. (C) Resolution assessment of the final 3D map using Fourier shell correlation (FSC). Two criteria are shown: FSC between two half-maps reconstructed from even and odd frames (evaluated at 0.143) and FSC between the final 3D map and the fitted model (evaluated at 0.5). (D) Zoomed-in views of the final 3D density map from panel A, displayed at two contour levels. (E) Superimposition of the high-contour level map from panel D onto its fitted model.

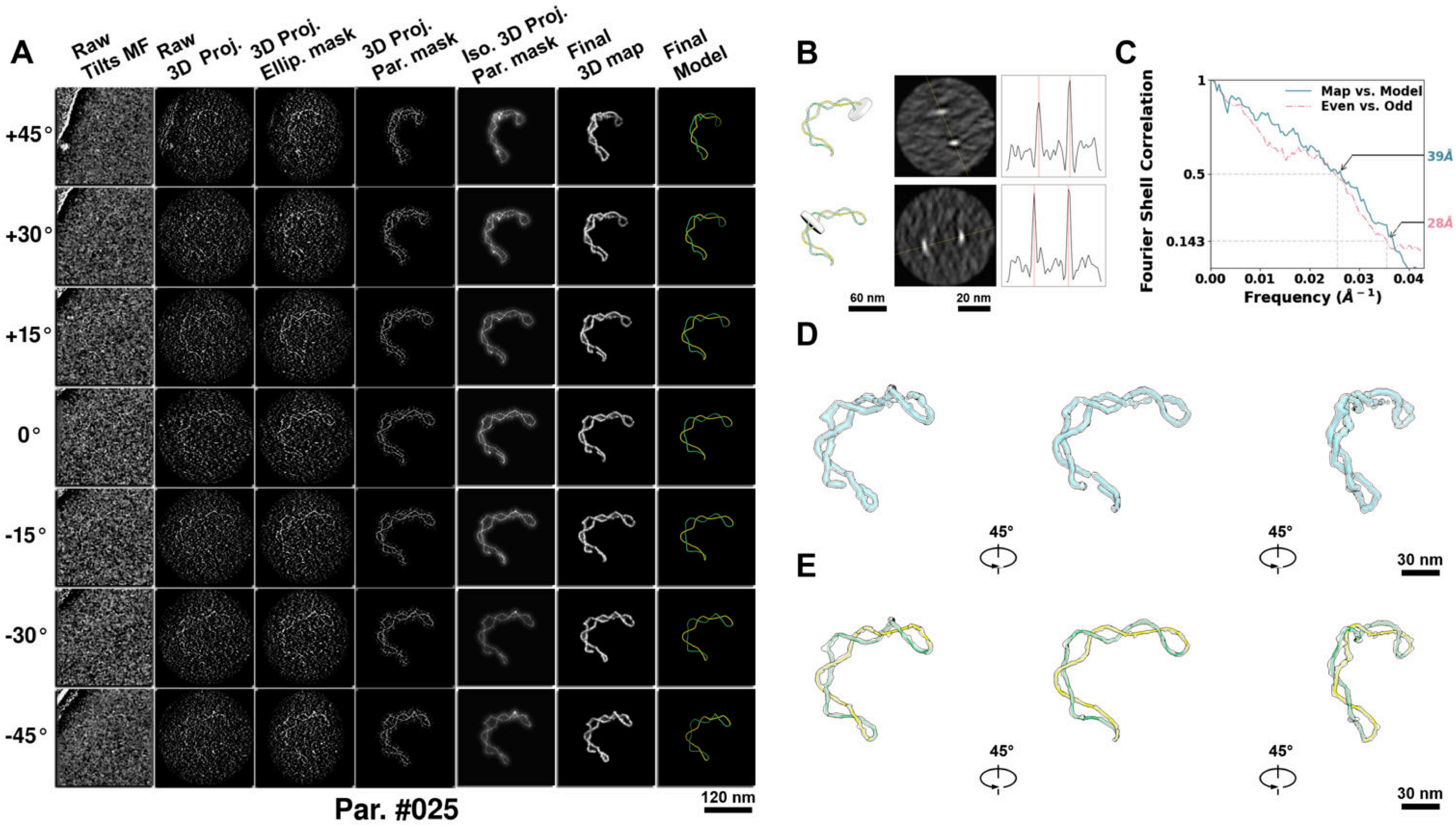

Supplementary Particle Figure 25. Cryo-ET 3D reconstruction of an individual P.LS particle.
(A) 3D reconstruction of the plasmid particle (index no. 25). The first column shows seven representative tilt images from +45° to -45° in step of 15°. The second, third, and fourth columns show 3D projections of the particle with spherical, ellipsoidal (thinner along the z-dimension), and particle-shaped masks, respectively. The fifth column displays the 3D projections of the enhanced and IsoNet missing-wedge-corrected particle. The sixth and seventh columns present the final 3D map and the flexibly fitted model, respectively. (B) Two cross-sectional views (12 nm thickness) of the plasmid density map along its plectoneme axis are shown in the left-middle panel. The intensity profile along the line crossing the two high-density DNA spots is displayed in the right panel. (C) Resolution assessment of the final 3D map using Fourier shell correlation (FSC). Two criteria are shown: FSC between two half-maps reconstructed from even and odd frames (evaluated at 0.143) and FSC between the final 3D map and the fitted model (evaluated at 0.5). (D) Zoomed-in views of the final 3D density map from panel A, displayed at two contour levels. (E) Superimposition of the high-contour level map from panel D onto its fitted model.

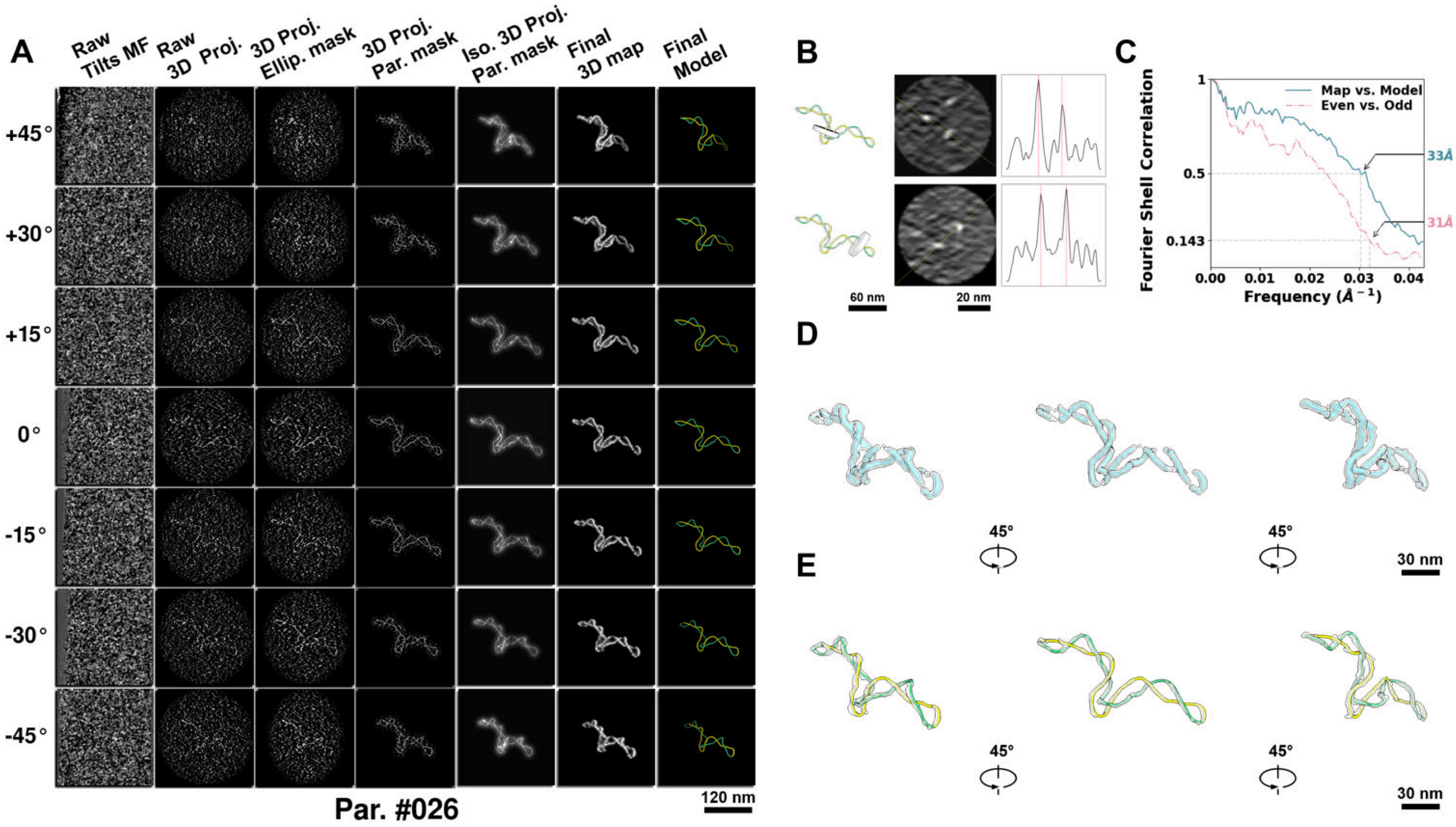

Supplementary Particle Figure 26. Cryo-ET 3D reconstruction of an individual P.LS particle.
(A) 3D reconstruction of the plasmid particle (index no. 26). The first column shows seven representative tilt images from +45° to -45° in step of 15°. The second, third, and fourth columns show 3D projections of the particle with spherical, ellipsoidal (thinner along the z-dimension), and particle-shaped masks, respectively. The fifth column displays the 3D projections of the enhanced and IsoNet missing-wedge-corrected particle. The sixth and seventh columns present the final 3D map and the flexibly fitted model, respectively. (B) Two cross-sectional views (12 nm thickness) of the plasmid density map along its plectoneme axis are shown in the left-middle panel. The intensity profile along the line crossing the two high-density DNA spots is displayed in the right panel. (C) Resolution assessment of the final 3D map using Fourier shell correlation (FSC). Two criteria are shown: FSC between two half-maps reconstructed from even and odd frames (evaluated at 0.143) and FSC between the final 3D map and the fitted model (evaluated at 0.5). (D) Zoomed-in views of the final 3D density map from panel A, displayed at two contour levels. (E) Superimposition of the high-contour level map from panel D onto its fitted model.

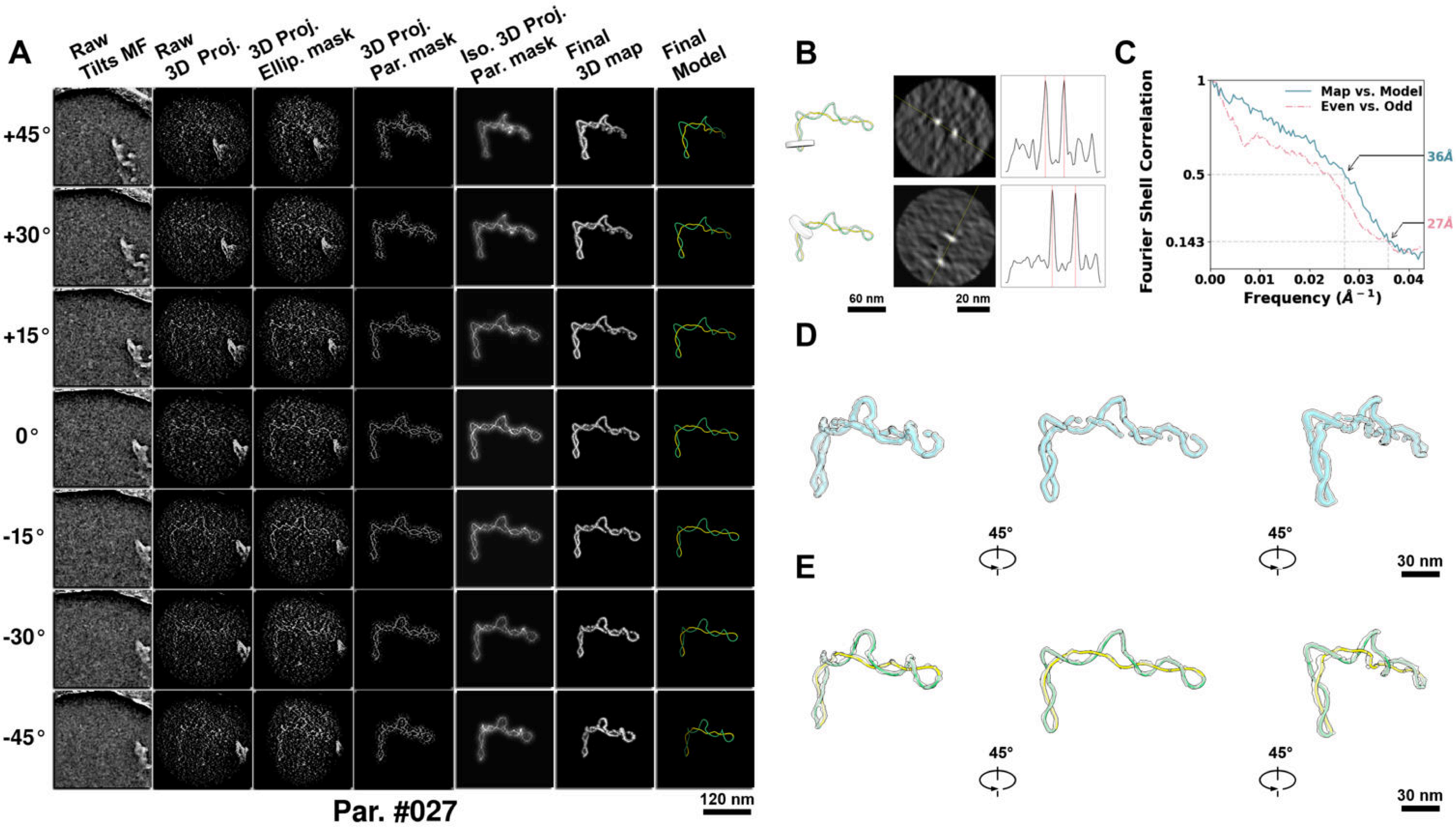

Supplementary Particle Figure 27. Cryo-ET 3D reconstruction of an individual P.LS particle.
(A) 3D reconstruction of the plasmid particle (index no. 27). The first column shows seven representative tilt images from +45° to -45° in step of 15°. The second, third, and fourth columns show 3D projections of the particle with spherical, ellipsoidal (thinner along the z-dimension), and particle-shaped masks, respectively. The fifth column displays the 3D projections of the enhanced and IsoNet missing-wedge-corrected particle. The sixth and seventh columns present the final 3D map and the flexibly fitted model, respectively. (B) Two cross-sectional views (12 nm thickness) of the plasmid density map along its plectoneme axis are shown in the left-middle panel. The intensity profile along the line crossing the two high-density DNA spots is displayed in the right panel. (C) Resolution assessment of the final 3D map using Fourier shell correlation (FSC). Two criteria are shown: FSC between two half-maps reconstructed from even and odd frames (evaluated at 0.143) and FSC between the final 3D map and the fitted model (evaluated at 0.5). (D) Zoomed-in views of the final 3D density map from panel A, displayed at two contour levels. (E) Superimposition of the high-contour level map from panel D onto its fitted model.

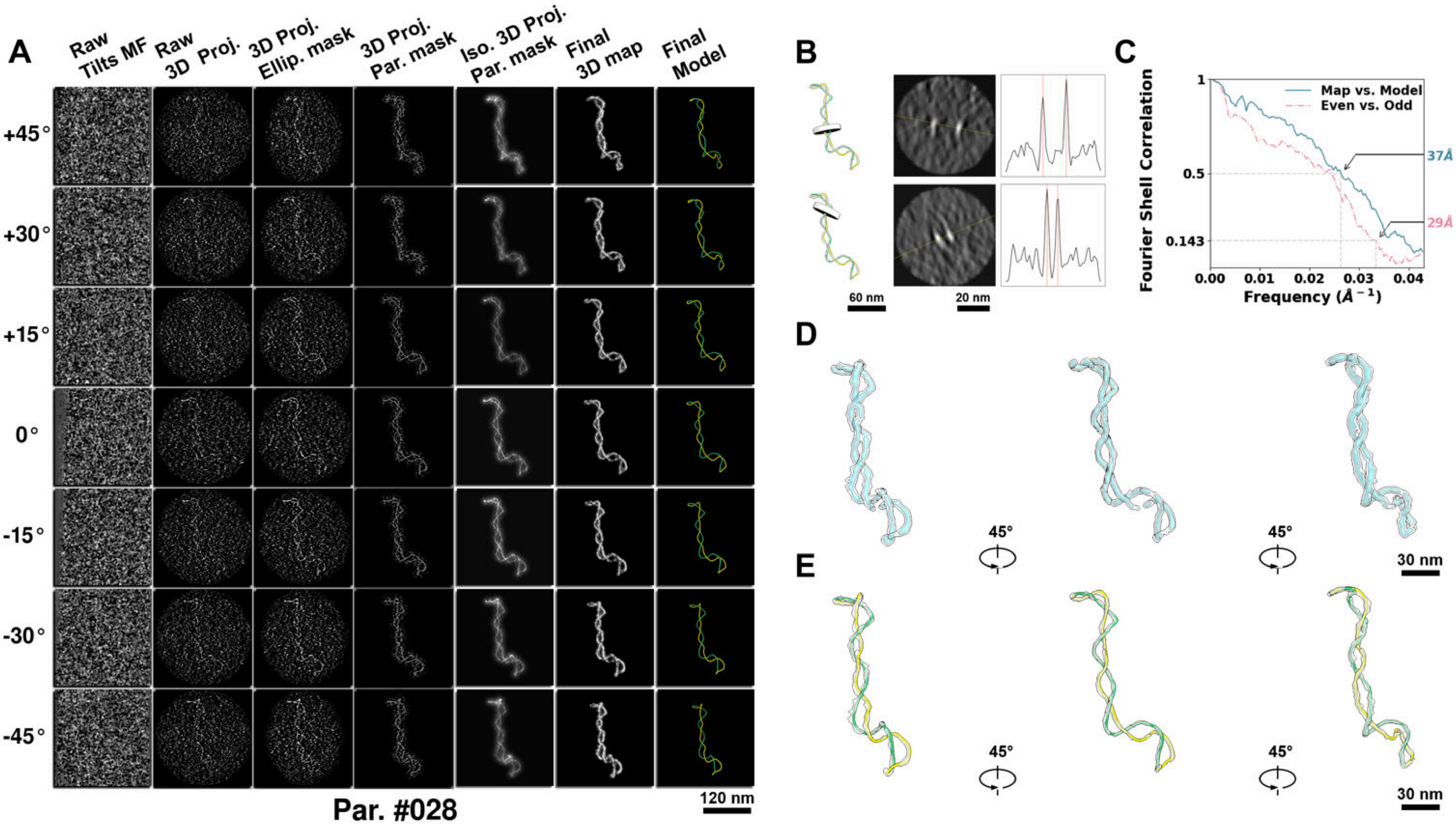

Supplementary Particle Figure 28. Cryo-ET 3D reconstruction of an individual P.LS particle.
(A) 3D reconstruction of the plasmid particle (index no. 28). The first column shows seven representative tilt images from +45° to -45° in step of 15°. The second, third, and fourth columns show 3D projections of the particle with spherical, ellipsoidal (thinner along the z-dimension), and particle-shaped masks, respectively. The fifth column displays the 3D projections of the enhanced and IsoNet missing-wedge-corrected particle. The sixth and seventh columns present the final 3D map and the flexibly fitted model, respectively. (B) Two cross-sectional views (12 nm thickness) of the plasmid density map along its plectoneme axis are shown in the left-middle panel. The intensity profile along the line crossing the two high-density DNA spots is displayed in the right panel. (C) Resolution assessment of the final 3D map using Fourier shell correlation (FSC). Two criteria are shown: FSC between two half-maps reconstructed from even and odd frames (evaluated at 0.143) and FSC between the final 3D map and the fitted model (evaluated at 0.5). (D) Zoomed-in views of the final 3D density map from panel A, displayed at two contour levels. (E) Superimposition of the high-contour level map from panel D onto its fitted model.

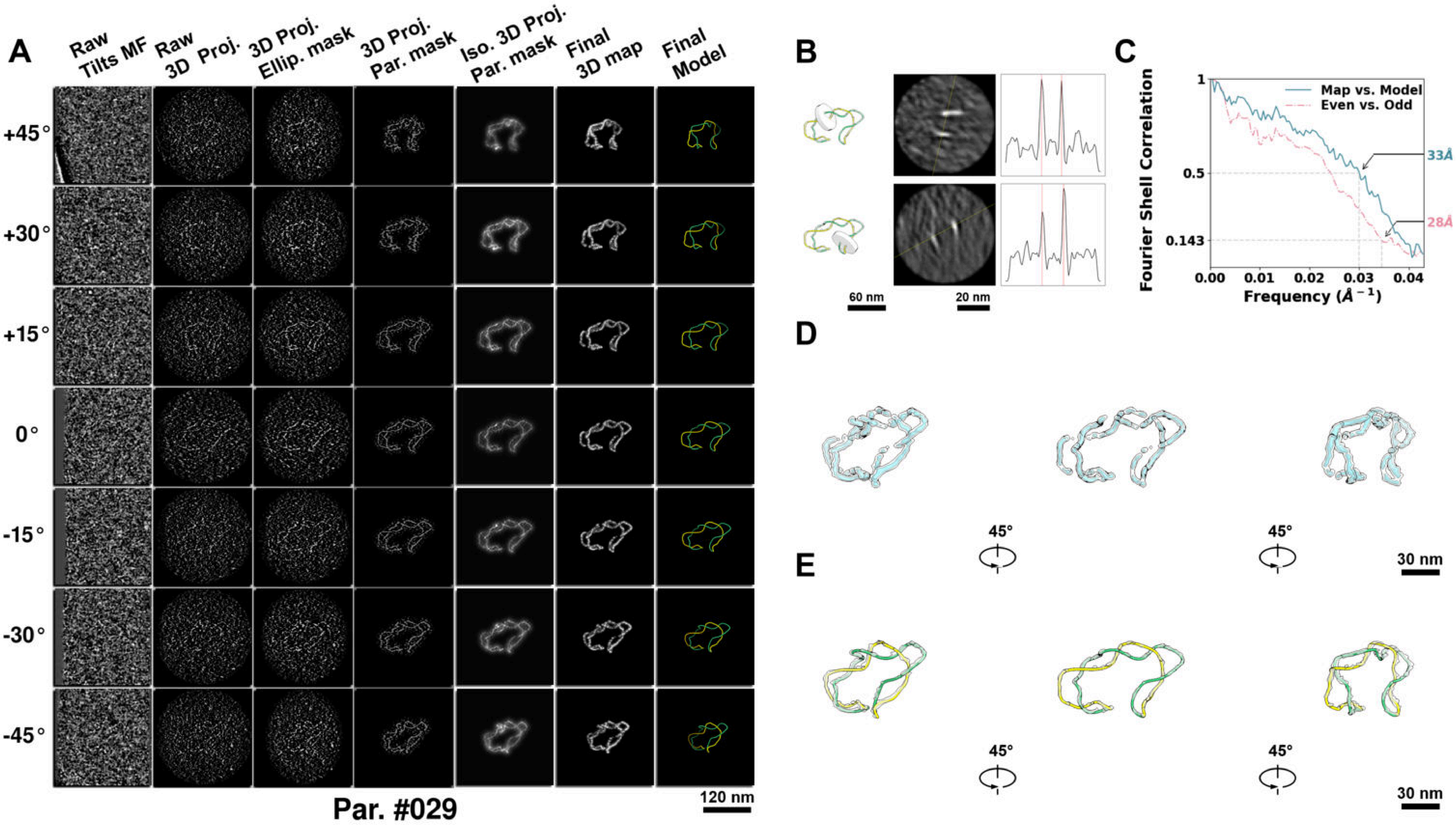

Supplementary Particle Figure 29. Cryo-ET 3D reconstruction of an individual P.LS particle.
(A) 3D reconstruction of the plasmid particle (index no. 29). The first column shows seven representative tilt images from +45° to -45° in step of 15°. The second, third, and fourth columns show 3D projections of the particle with spherical, ellipsoidal (thinner along the z-dimension), and particle-shaped masks, respectively. The fifth column displays the 3D projections of the enhanced and IsoNet missing-wedge-corrected particle. The sixth and seventh columns present the final 3D map and the flexibly fitted model, respectively. (B) Two cross-sectional views (12 nm thickness) of the plasmid density map along its plectoneme axis are shown in the left-middle panel. The intensity profile along the line crossing the two high-density DNA spots is displayed in the right panel. (C) Resolution assessment of the final 3D map using Fourier shell correlation (FSC). Two criteria are shown: FSC between two half-maps reconstructed from even and odd frames (evaluated at 0.143) and FSC between the final 3D map and the fitted model (evaluated at 0.5). (D) Zoomed-in views of the final 3D density map from panel A, displayed at two contour levels. (E) Superimposition of the high-contour level map from panel D onto its fitted model.

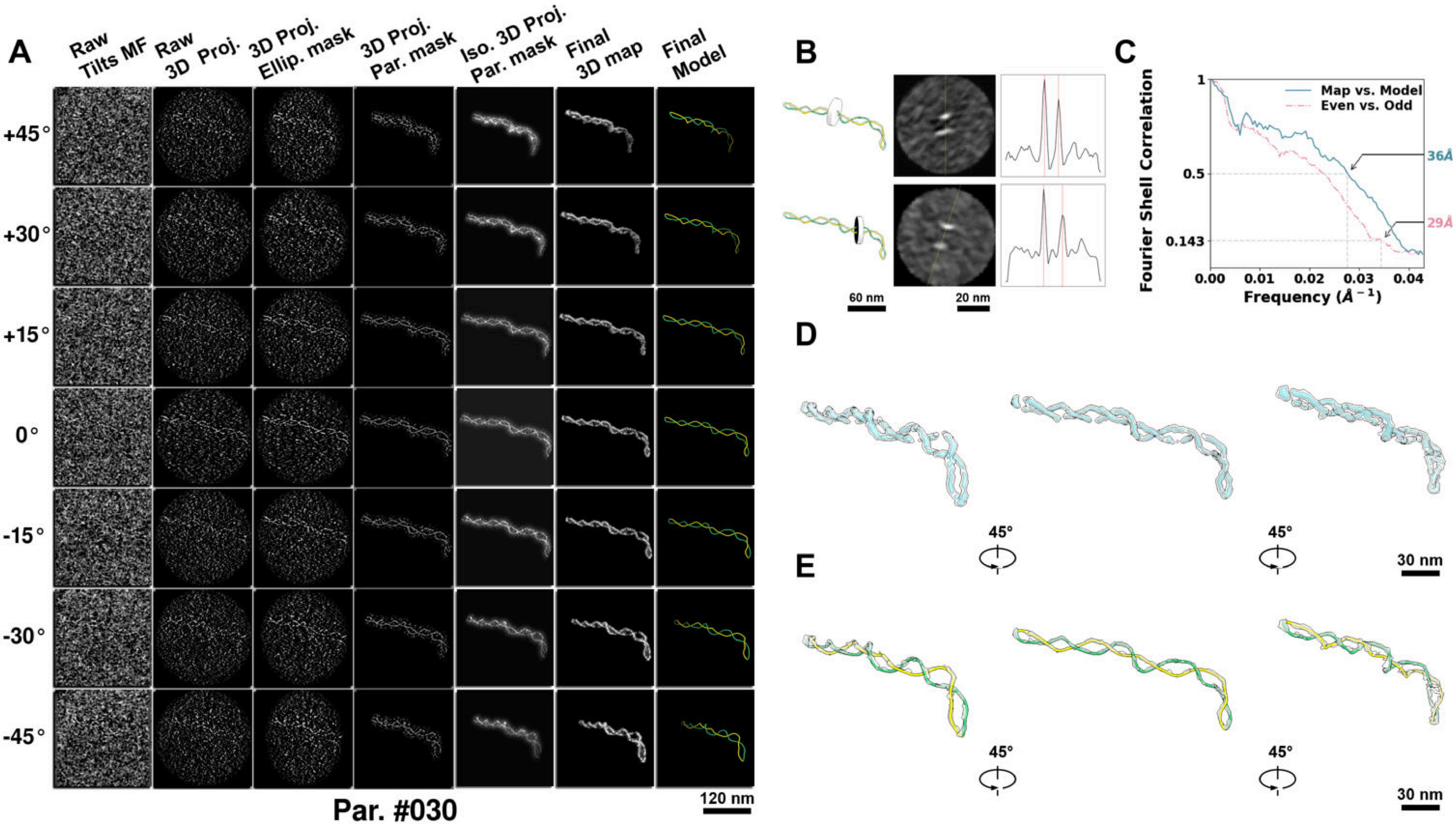

Supplementary Particle Figure 30. Cryo-ET 3D reconstruction of an individual P.LS particle.
(A) 3D reconstruction of the plasmid particle (index no. 30). The first column shows seven representative tilt images from +45° to -45° in step of 15°. The second, third, and fourth columns show 3D projections of the particle with spherical, ellipsoidal (thinner along the z-dimension), and particle-shaped masks, respectively. The fifth column displays the 3D projections of the enhanced and IsoNet missing-wedge-corrected particle. The sixth and seventh columns present the final 3D map and the flexibly fitted model, respectively. (B) Two cross-sectional views (12 nm thickness) of the plasmid density map along its plectoneme axis are shown in the left-middle panel. The intensity profile along the line crossing the two high-density DNA spots is displayed in the right panel. (C) Resolution assessment of the final 3D map using Fourier shell correlation (FSC). Two criteria are shown: FSC between two half-maps reconstructed from even and odd frames (evaluated at 0.143) and FSC between the final 3D map and the fitted model (evaluated at 0.5). (D) Zoomed-in views of the final 3D density map from panel A, displayed at two contour levels. (E) Superimposition of the high-contour level map from panel D onto its fitted model.

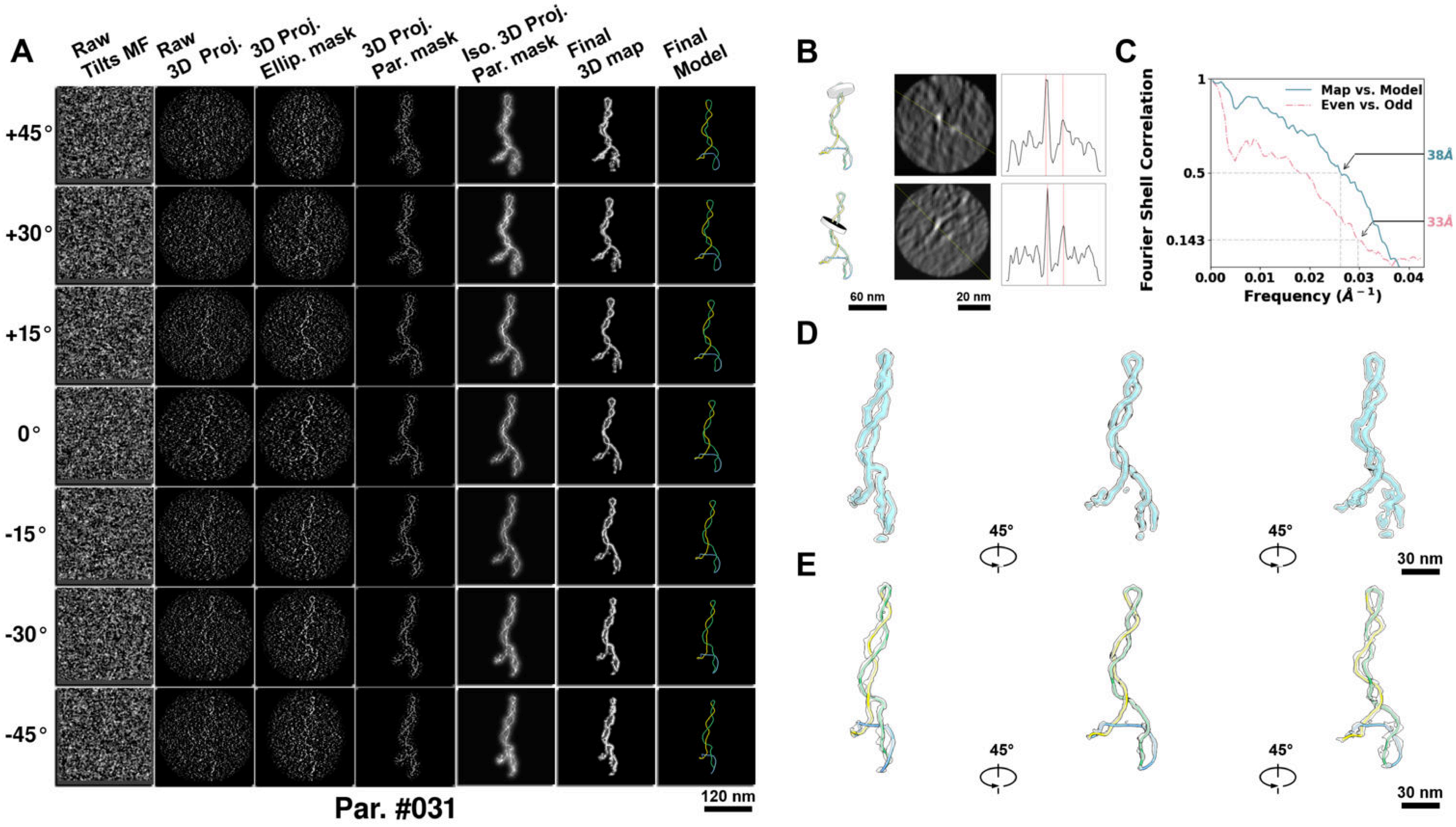

Supplementary Particle Figure 31. Cryo-ET 3D reconstruction of an individual P.LS particle.
(A) 3D reconstruction of the plasmid particle (index no. 31). The first column shows seven representative tilt images from +45° to -45° in step of 15°. The second, third, and fourth columns show 3D projections of the particle with spherical, ellipsoidal (thinner along the z-dimension), and particle-shaped masks, respectively. The fifth column displays the 3D projections of the enhanced and IsoNet missing-wedge-corrected particle. The sixth and seventh columns present the final 3D map and the flexibly fitted model, respectively. (B) Two cross-sectional views (12 nm thickness) of the plasmid density map along its plectoneme axis are shown in the left-middle panel. The intensity profile along the line crossing the two high-density DNA spots is displayed in the right panel. (C) Resolution assessment of the final 3D map using Fourier shell correlation (FSC). Two criteria are shown: FSC between two half-maps reconstructed from even and odd frames (evaluated at 0.143) and FSC between the final 3D map and the fitted model (evaluated at 0.5). (D) Zoomed-in views of the final 3D density map from panel A, displayed at two contour levels. (E) Superimposition of the high-contour level map from panel D onto its fitted model.

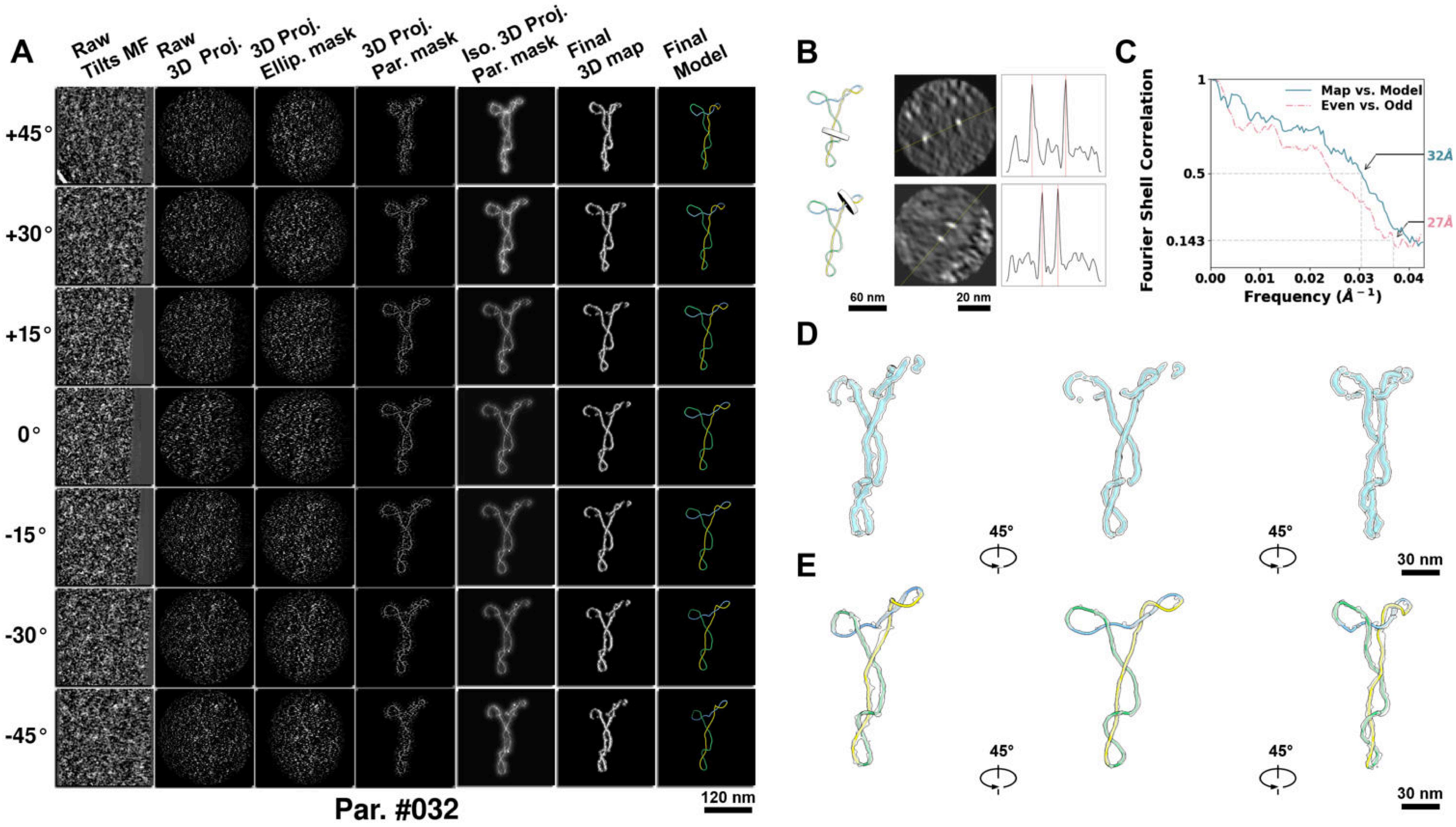

Supplementary Particle Figure 32. Cryo-ET 3D reconstruction of an individual P.LS particle.
(A) 3D reconstruction of the plasmid particle (index no. 32). The first column shows seven representative tilt images from +45° to -45° in step of 15°. The second, third, and fourth columns show 3D projections of the particle with spherical, ellipsoidal (thinner along the z-dimension), and particle-shaped masks, respectively. The fifth column displays the 3D projections of the enhanced and IsoNet missing-wedge-corrected particle. The sixth and seventh columns present the final 3D map and the flexibly fitted model, respectively. (B) Two cross-sectional views (12 nm thickness) of the plasmid density map along its plectoneme axis are shown in the left-middle panel. The intensity profile along the line crossing the two high-density DNA spots is displayed in the right panel. (C) Resolution assessment of the final 3D map using Fourier shell correlation (FSC). Two criteria are shown: FSC between two half-maps reconstructed from even and odd frames (evaluated at 0.143) and FSC between the final 3D map and the fitted model (evaluated at 0.5). (D) Zoomed-in views of the final 3D density map from panel A, displayed at two contour levels. (E) Superimposition of the high-contour level map from panel D onto its fitted model.

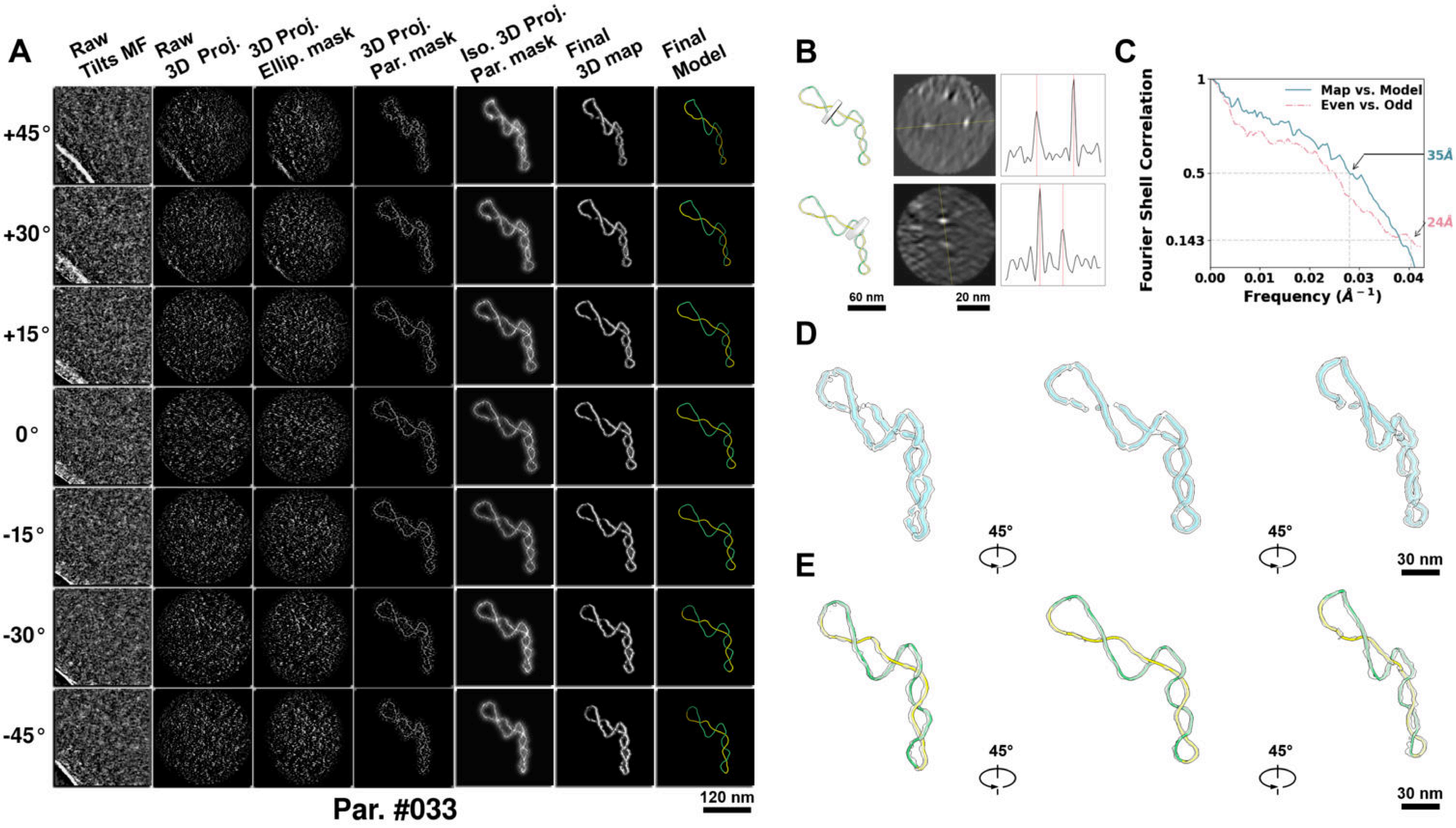

Supplementary Particle Figure 33. Cryo-ET 3D reconstruction of an individual P.LS particle.
(A) 3D reconstruction of the plasmid particle (index no. 33). The first column shows seven representative tilt images from +45° to -45° in step of 15°. The second, third, and fourth columns show 3D projections of the particle with spherical, ellipsoidal (thinner along the z-dimension), and particle-shaped masks, respectively. The fifth column displays the 3D projections of the enhanced and IsoNet missing-wedge-corrected particle. The sixth and seventh columns present the final 3D map and the flexibly fitted model, respectively. (B) Two cross-sectional views (12 nm thickness) of the plasmid density map along its plectoneme axis are shown in the left-middle panel. The intensity profile along the line crossing the two high-density DNA spots is displayed in the right panel. (C) Resolution assessment of the final 3D map using Fourier shell correlation (FSC). Two criteria are shown: FSC between two half-maps reconstructed from even and odd frames (evaluated at 0.143) and FSC between the final 3D map and the fitted model (evaluated at 0.5). (D) Zoomed-in views of the final 3D density map from panel A, displayed at two contour levels. (E) Superimposition of the high-contour level map from panel D onto its fitted model.

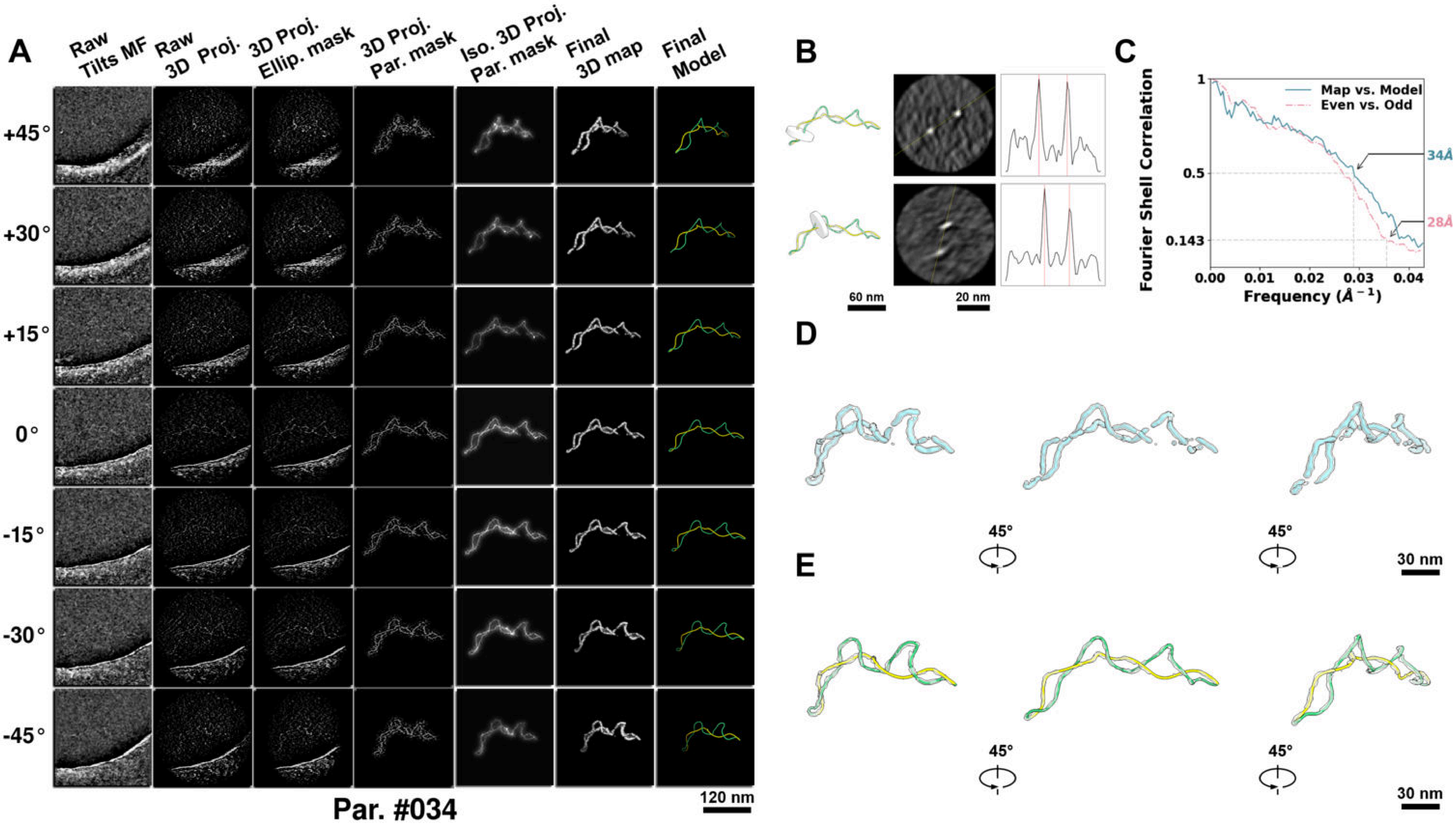

Supplementary Particle Figure 34. Cryo-ET 3D reconstruction of an individual P.LS particle.
(A) 3D reconstruction of the plasmid particle (index no. 34). The first column shows seven representative tilt images from +45° to -45° in step of 15°. The second, third, and fourth columns show 3D projections of the particle with spherical, ellipsoidal (thinner along the z-dimension), and particle-shaped masks, respectively. The fifth column displays the 3D projections of the enhanced and IsoNet missing-wedge-corrected particle. The sixth and seventh columns present the final 3D map and the flexibly fitted model, respectively. (B) Two cross-sectional views (12 nm thickness) of the plasmid density map along its plectoneme axis are shown in the left-middle panel. The intensity profile along the line crossing the two high-density DNA spots is displayed in the right panel. (C) Resolution assessment of the final 3D map using Fourier shell correlation (FSC). Two criteria are shown: FSC between two half-maps reconstructed from even and odd frames (evaluated at 0.143) and FSC between the final 3D map and the fitted model (evaluated at 0.5). (D) Zoomed-in views of the final 3D density map from panel A, displayed at two contour levels. (E) Superimposition of the high-contour level map from panel D onto its fitted model.

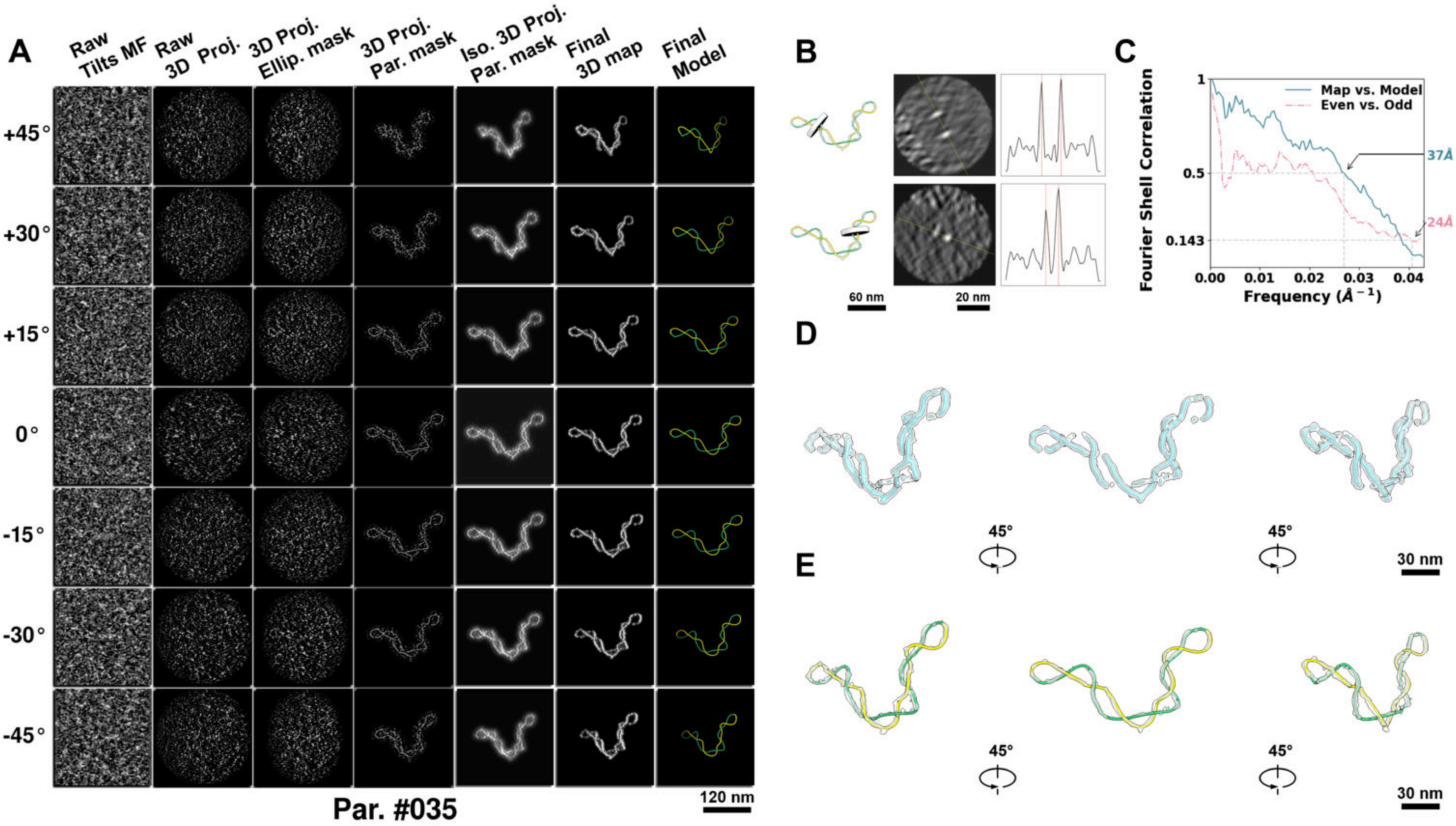

Supplementary Particle Figure 35. Cryo-ET 3D reconstruction of an individual P.LS particle.
(A) 3D reconstruction of the plasmid particle (index no. 35). The first column shows seven representative tilt images from +45° to -45° in step of 15°. The second, third, and fourth columns show 3D projections of the particle with spherical, ellipsoidal (thinner along the z-dimension), and particle-shaped masks, respectively. The fifth column displays the 3D projections of the enhanced and IsoNet missing-wedge-corrected particle. The sixth and seventh columns present the final 3D map and the flexibly fitted model, respectively. (B) Two cross-sectional views (12 nm thickness) of the plasmid density map along its plectoneme axis are shown in the left-middle panel. The intensity profile along the line crossing the two high-density DNA spots is displayed in the right panel. (C) Resolution assessment of the final 3D map using Fourier shell correlation (FSC). Two criteria are shown: FSC between two half-maps reconstructed from even and odd frames (evaluated at 0.143) and FSC between the final 3D map and the fitted model (evaluated at 0.5). (D) Zoomed-in views of the final 3D density map from panel A, displayed at two contour levels. (E) Superimposition of the high-contour level map from panel D onto its fitted model.

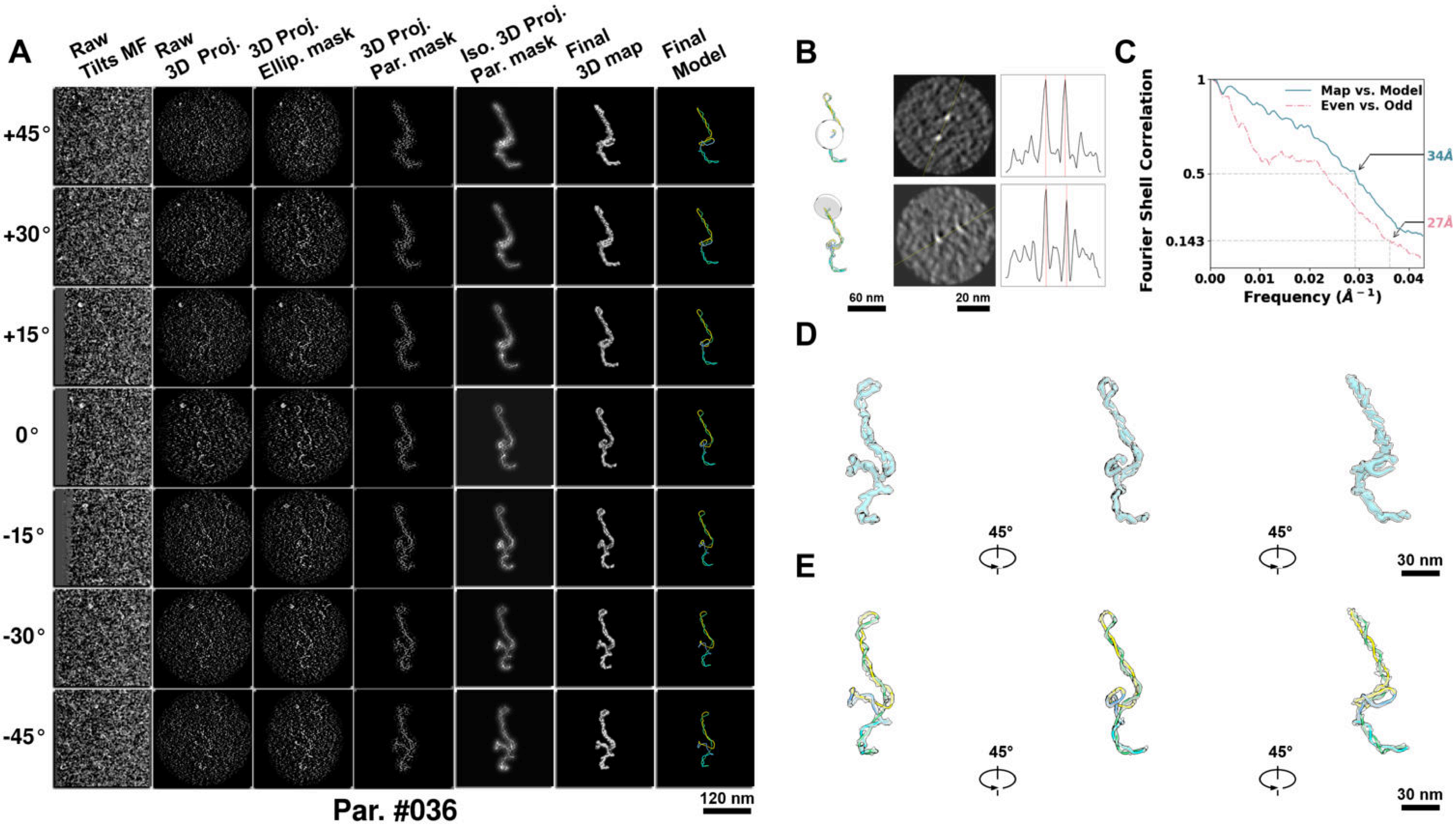

Supplementary Particle Figure 36. Cryo-ET 3D reconstruction of an individual P.HS particle.
(A) 3D reconstruction of the plasmid particle (index no. 36). The first column shows seven representative tilt images from +45° to -45° in step of 15°. The second, third, and fourth columns show 3D projections of the particle with spherical, ellipsoidal (thinner along the z-dimension), and particle-shaped masks, respectively. The fifth column displays the 3D projections of the enhanced and IsoNet missing-wedge-corrected particle. The sixth and seventh columns present the final 3D map and the flexibly fitted model, respectively. (B) Two cross-sectional views (12 nm thickness) of the plasmid density map along its plectoneme axis are shown in the left-middle panel. The intensity profile along the line crossing the two high-density DNA spots is displayed in the right panel. (C) Resolution assessment of the final 3D map using Fourier shell correlation (FSC). Two criteria are shown: FSC between two half-maps reconstructed from even and odd frames (evaluated at 0.143) and FSC between the final 3D map and the fitted model (evaluated at 0.5). (D) Zoomed-in views of the final 3D density map from panel A, displayed at two contour levels. (E) Superimposition of the high-contour level map from panel D onto its fitted model.

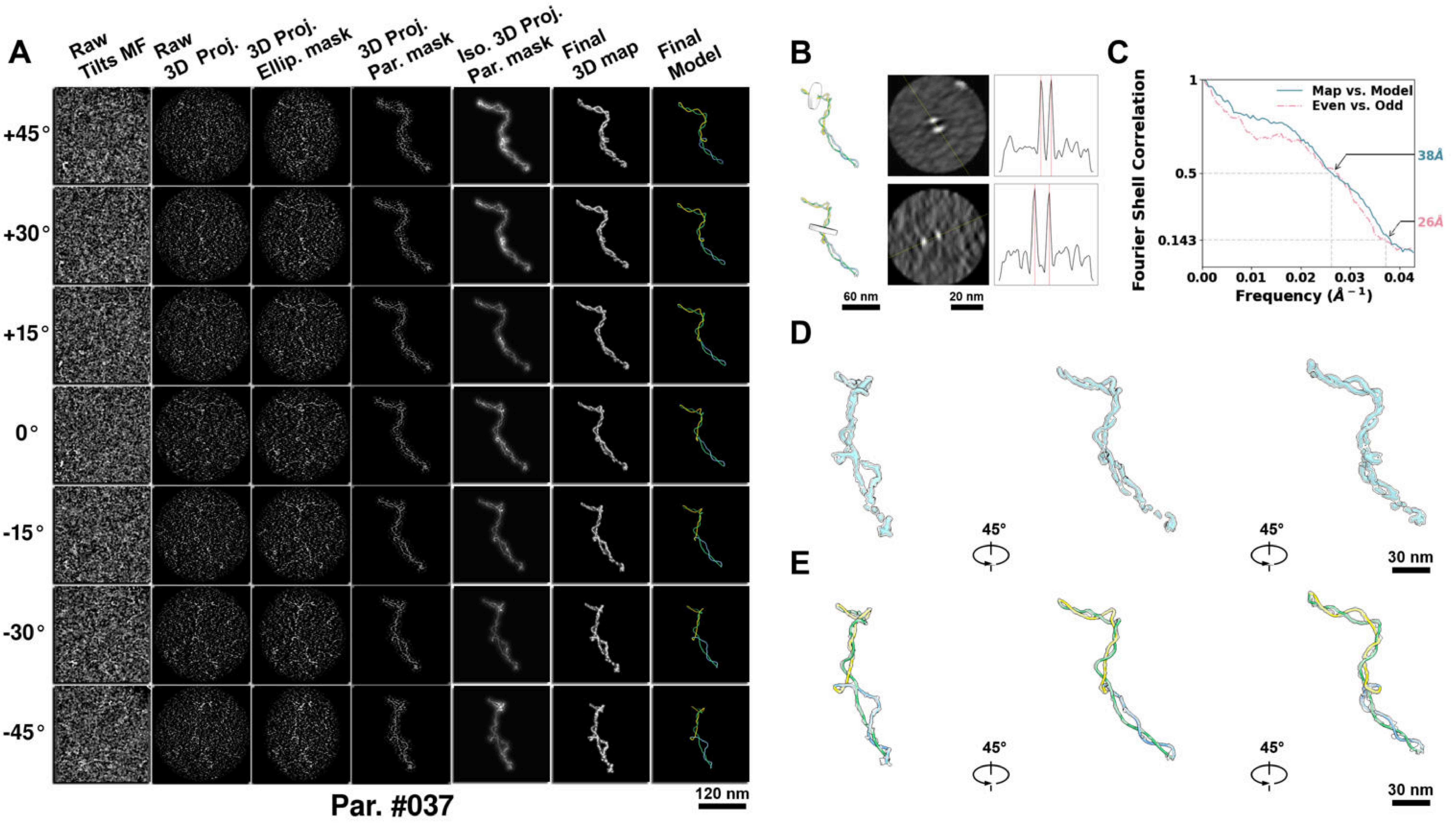

Supplementary Particle Figure 37. Cryo-ET 3D reconstruction of an individual P.HS particle.
(A) 3D reconstruction of the plasmid particle (index no. 37). The first column shows seven representative tilt images from +45° to -45° in step of 15°. The second, third, and fourth columns show 3D projections of the particle with spherical, ellipsoidal (thinner along the z-dimension), and particle-shaped masks, respectively. The fifth column displays the 3D projections of the enhanced and IsoNet missing-wedge-corrected particle. The sixth and seventh columns present the final 3D map and the flexibly fitted model, respectively. (B) Two cross-sectional views (12 nm thickness) of the plasmid density map along its plectoneme axis are shown in the left-middle panel. The intensity profile along the line crossing the two high-density DNA spots is displayed in the right panel. (C) Resolution assessment of the final 3D map using Fourier shell correlation (FSC). Two criteria are shown: FSC between two half-maps reconstructed from even and odd frames (evaluated at 0.143) and FSC between the final 3D map and the fitted model (evaluated at 0.5). (D) Zoomed-in views of the final 3D density map from panel A, displayed at two contour levels. (E) Superimposition of the high-contour level map from panel D onto its fitted model.

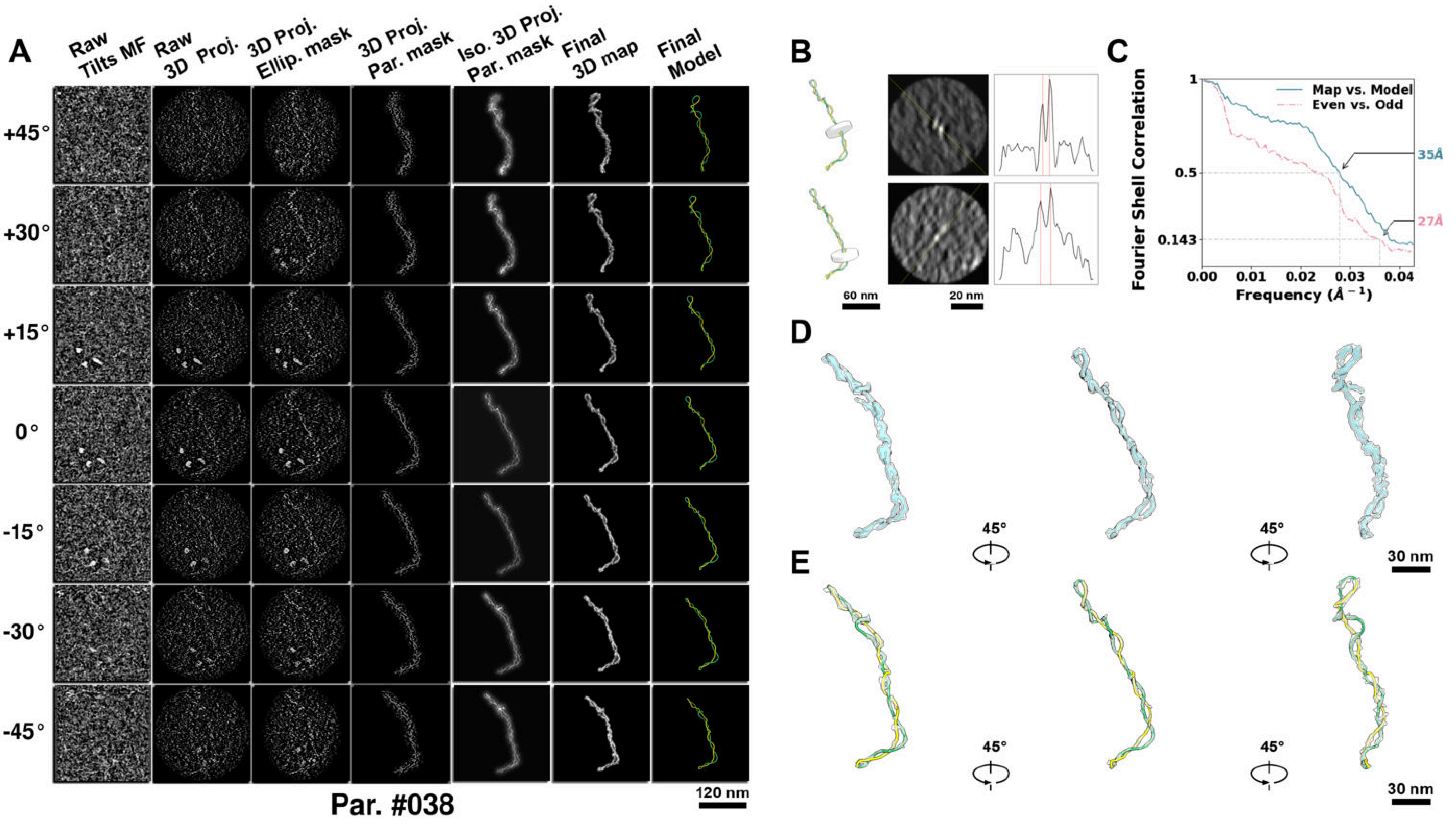

Supplementary Particle Figure 38. Cryo-ET 3D reconstruction of an individual P.HS particle.
(A) 3D reconstruction of the plasmid particle (index no. 38). The first column shows seven representative tilt images from +45° to -45° in step of 15°. The second, third, and fourth columns show 3D projections of the particle with spherical, ellipsoidal (thinner along the z-dimension), and particle-shaped masks, respectively. The fifth column displays the 3D projections of the enhanced and IsoNet missing-wedge-corrected particle. The sixth and seventh columns present the final 3D map and the flexibly fitted model, respectively. (B) Two cross-sectional views (12 nm thickness) of the plasmid density map along its plectoneme axis are shown in the left-middle panel. The intensity profile along the line crossing the two high-density DNA spots is displayed in the right panel. (C) Resolution assessment of the final 3D map using Fourier shell correlation (FSC). Two criteria are shown: FSC between two half-maps reconstructed from even and odd frames (evaluated at 0.143) and FSC between the final 3D map and the fitted model (evaluated at 0.5). (D) Zoomed-in views of the final 3D density map from panel A, displayed at two contour levels. (E) Superimposition of the high-contour level map from panel D onto its fitted model.

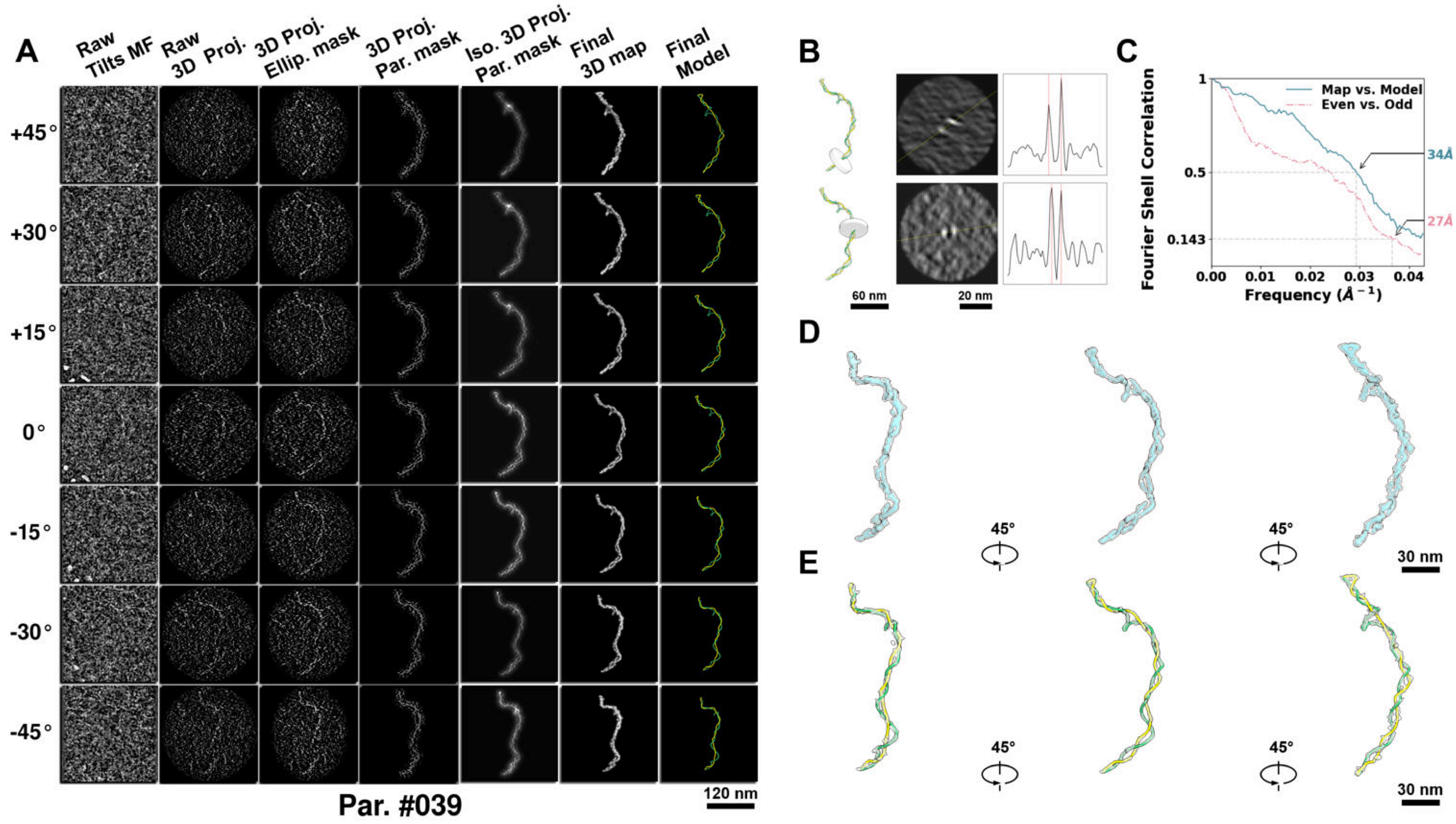

Supplementary Particle Figure 39. Cryo-ET 3D reconstruction of an individual P.HS particle.
(A) 3D reconstruction of the plasmid particle (index no. 39). The first column shows seven representative tilt images from +45° to -45° in step of 15°. The second, third, and fourth columns show 3D projections of the particle with spherical, ellipsoidal (thinner along the z-dimension), and particle-shaped masks, respectively. The fifth column displays the 3D projections of the enhanced and IsoNet missing-wedge-corrected particle. The sixth and seventh columns present the final 3D map and the flexibly fitted model, respectively. (B) Two cross-sectional views (12 nm thickness) of the plasmid density map along its plectoneme axis are shown in the left-middle panel. The intensity profile along the line crossing the two high-density DNA spots is displayed in the right panel. (C) Resolution assessment of the final 3D map using Fourier shell correlation (FSC). Two criteria are shown: FSC between two half-maps reconstructed from even and odd frames (evaluated at 0.143) and FSC between the final 3D map and the fitted model (evaluated at 0.5). (D) Zoomed-in views of the final 3D density map from panel A, displayed at two contour levels. (E) Superimposition of the high-contour level map from panel D onto its fitted model.

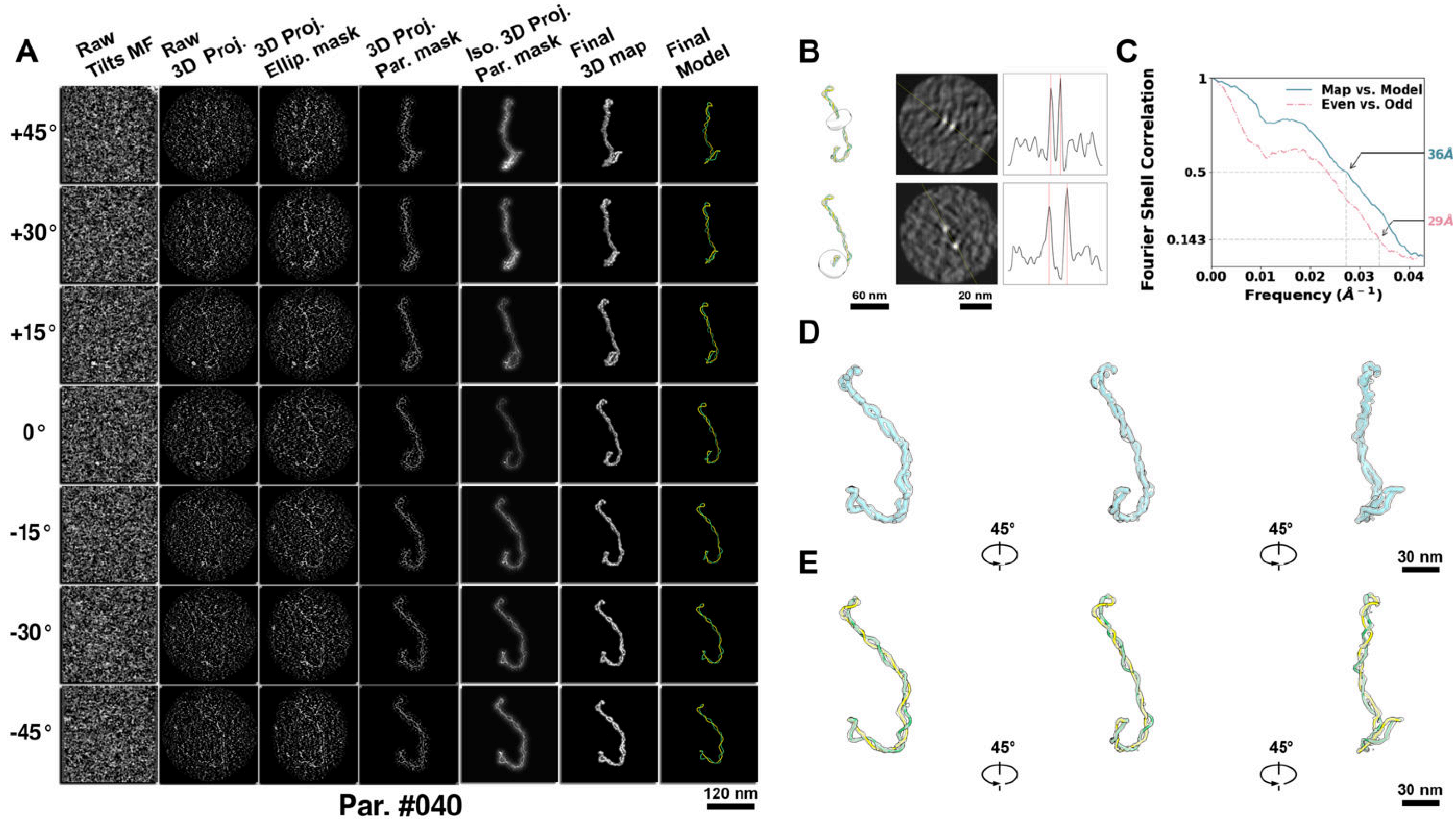

Supplementary Particle Figure 40. Cryo-ET 3D reconstruction of an individual P.HS particle.
(A) 3D reconstruction of the plasmid particle (index no. 40). The first column shows seven representative tilt images from +45° to -45° in step of 15°. The second, third, and fourth columns show 3D projections of the particle with spherical, ellipsoidal (thinner along the z-dimension), and particle-shaped masks, respectively. The fifth column displays the 3D projections of the enhanced and IsoNet missing-wedge-corrected particle. The sixth and seventh columns present the final 3D map and the flexibly fitted model, respectively. (B) Two cross-sectional views (12 nm thickness) of the plasmid density map along its plectoneme axis are shown in the left-middle panel. The intensity profile along the line crossing the two high-density DNA spots is displayed in the right panel. (C) Resolution assessment of the final 3D map using Fourier shell correlation (FSC). Two criteria are shown: FSC between two half-maps reconstructed from even and odd frames (evaluated at 0.143) and FSC between the final 3D map and the fitted model (evaluated at 0.5). (D) Zoomed-in views of the final 3D density map from panel A, displayed at two contour levels. (E) Superimposition of the high-contour level map from panel D onto its fitted model.

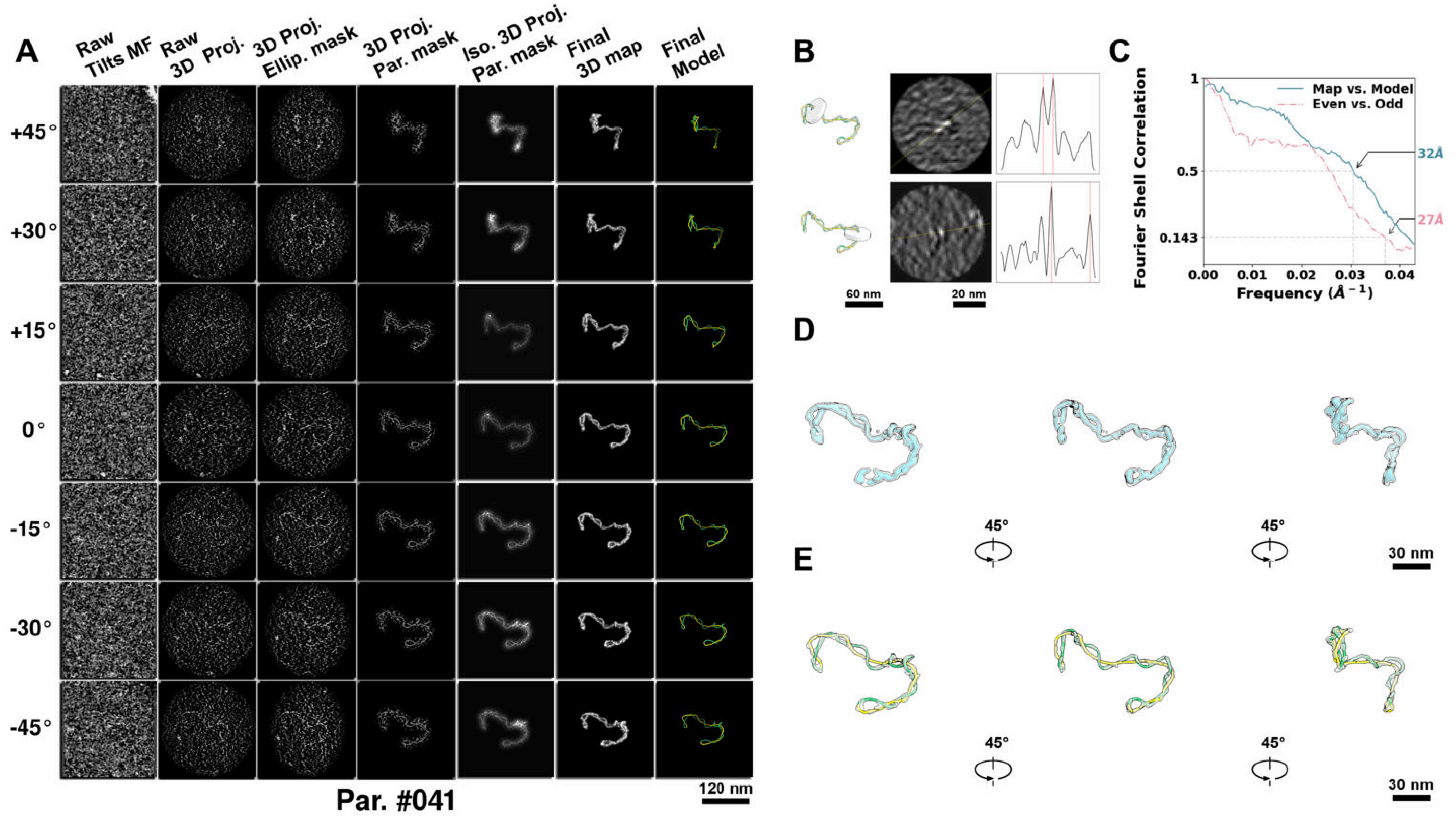

Supplementary Particle Figure 41. Cryo-ET 3D reconstruction of an individual P.HS particle.
(A) 3D reconstruction of the plasmid particle (index no. 41). The first column shows seven representative tilt images from +45° to -45° in step of 15°. The second, third, and fourth columns show 3D projections of the particle with spherical, ellipsoidal (thinner along the z-dimension), and particle-shaped masks, respectively. The fifth column displays the 3D projections of the enhanced and IsoNet missing-wedge-corrected particle. The sixth and seventh columns present the final 3D map and the flexibly fitted model, respectively. (B) Two cross-sectional views (12 nm thickness) of the plasmid density map along its plectoneme axis are shown in the left-middle panel. The intensity profile along the line crossing the two high-density DNA spots is displayed in the right panel. (C) Resolution assessment of the final 3D map using Fourier shell correlation (FSC). Two criteria are shown: FSC between two half-maps reconstructed from even and odd frames (evaluated at 0.143) and FSC between the final 3D map and the fitted model (evaluated at 0.5). (D) Zoomed-in views of the final 3D density map from panel A, displayed at two contour levels. (E) Superimposition of the high-contour level map from panel D onto its fitted model.

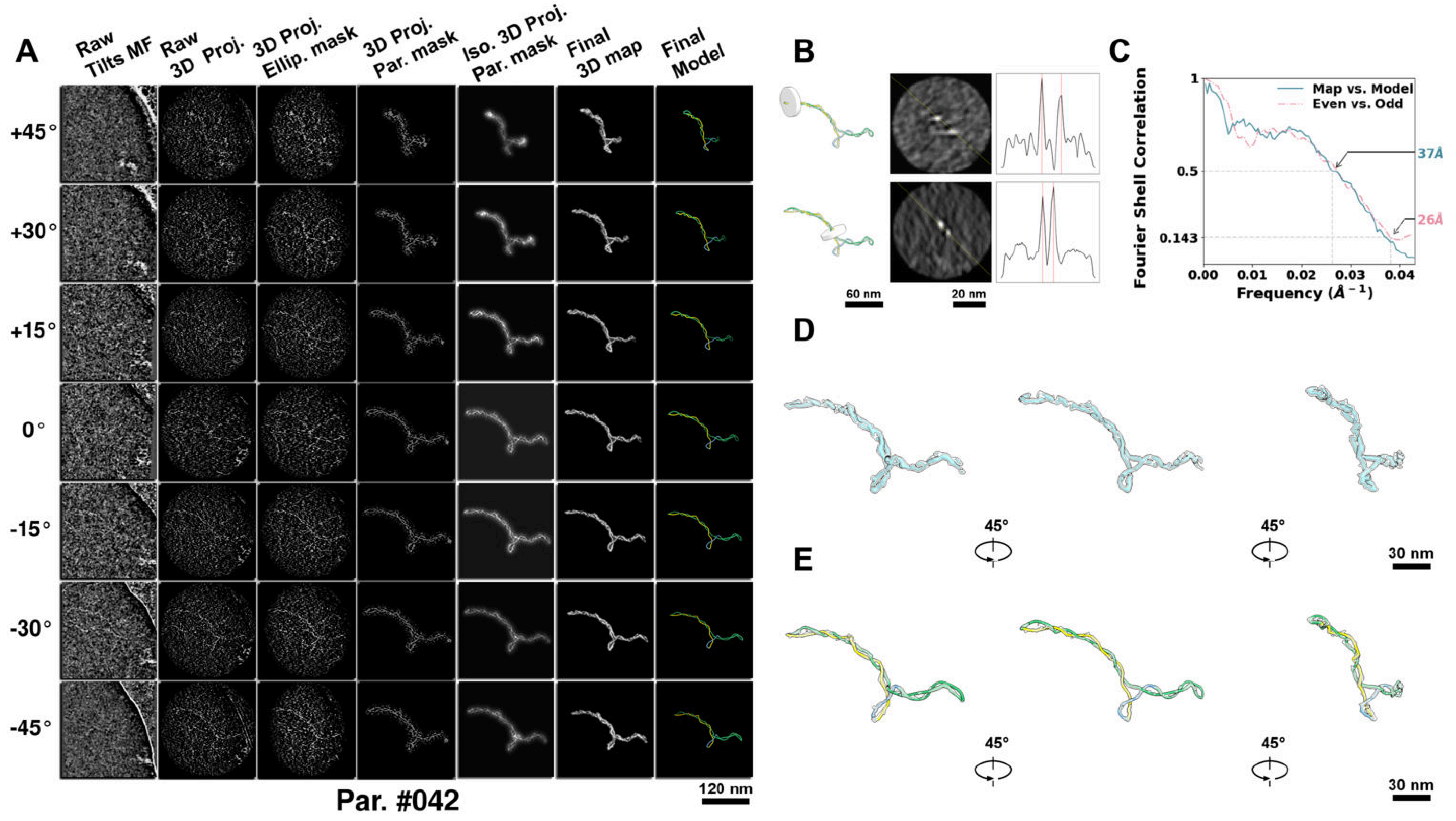

Supplementary Particle Figure 42. Cryo-ET 3D reconstruction of an individual P.HS particle.
(A) 3D reconstruction of the plasmid particle (index no. 42). The first column shows seven representative tilt images from +45° to -45° in step of 15°. The second, third, and fourth columns show 3D projections of the particle with spherical, ellipsoidal (thinner along the z-dimension), and particle-shaped masks, respectively. The fifth column displays the 3D projections of the enhanced and IsoNet missing-wedge-corrected particle. The sixth and seventh columns present the final 3D map and the flexibly fitted model, respectively. (B) Two cross-sectional views (12 nm thickness) of the plasmid density map along its plectoneme axis are shown in the left-middle panel. The intensity profile along the line crossing the two high-density DNA spots is displayed in the right panel. (C) Resolution assessment of the final 3D map using Fourier shell correlation (FSC). Two criteria are shown: FSC between two half-maps reconstructed from even and odd frames (evaluated at 0.143) and FSC between the final 3D map and the fitted model (evaluated at 0.5). (D) Zoomed-in views of the final 3D density map from panel A, displayed at two contour levels. (E) Superimposition of the high-contour level map from panel D onto its fitted model.

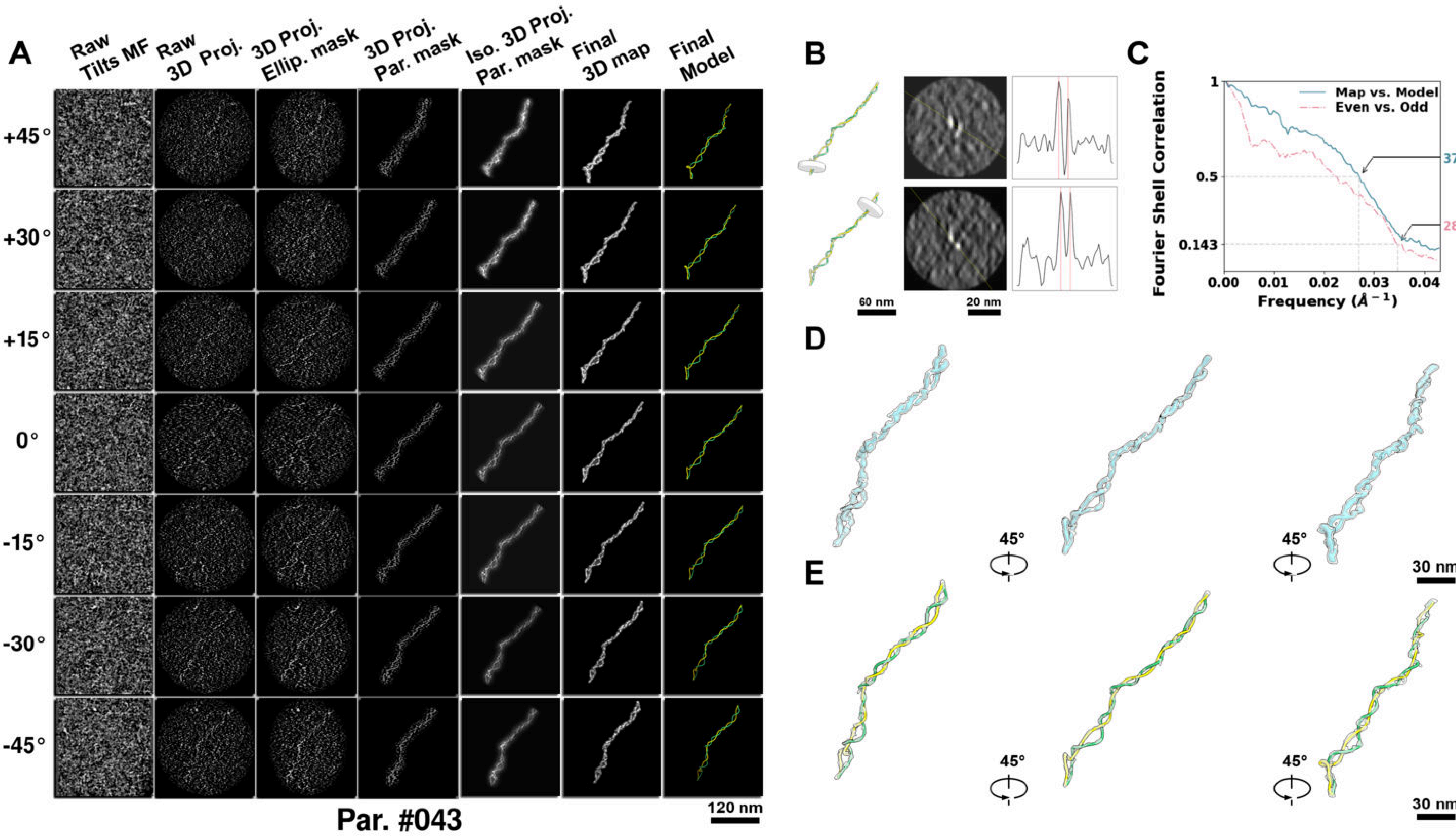

Supplementary Particle Figure 43. Cryo-ET 3D reconstruction of an individual P.HS particle.
(A) 3D reconstruction of the plasmid particle (index no. 43). The first column shows seven representative tilt images from +45° to -45° in step of 15°. The second, third, and fourth columns show 3D projections of the particle with spherical, ellipsoidal (thinner along the z-dimension), and particle-shaped masks, respectively. The fifth column displays the 3D projections of the enhanced and IsoNet missing-wedge-corrected particle. The sixth and seventh columns present the final 3D map and the flexibly fitted model, respectively. (B) Two cross-sectional views (12 nm thickness) of the plasmid density map along its plectoneme axis are shown in the left-middle panel. The intensity profile along the line crossing the two high-density DNA spots is displayed in the right panel. (C) Resolution assessment of the final 3D map using Fourier shell correlation (FSC). Two criteria are shown: FSC between two half-maps reconstructed from even and odd frames (evaluated at 0.143) and FSC between the final 3D map and the fitted model (evaluated at 0.5). (D) Zoomed-in views of the final 3D density map from panel A, displayed at two contour levels. (E) Superimposition of the high-contour level map from panel D onto its fitted model.

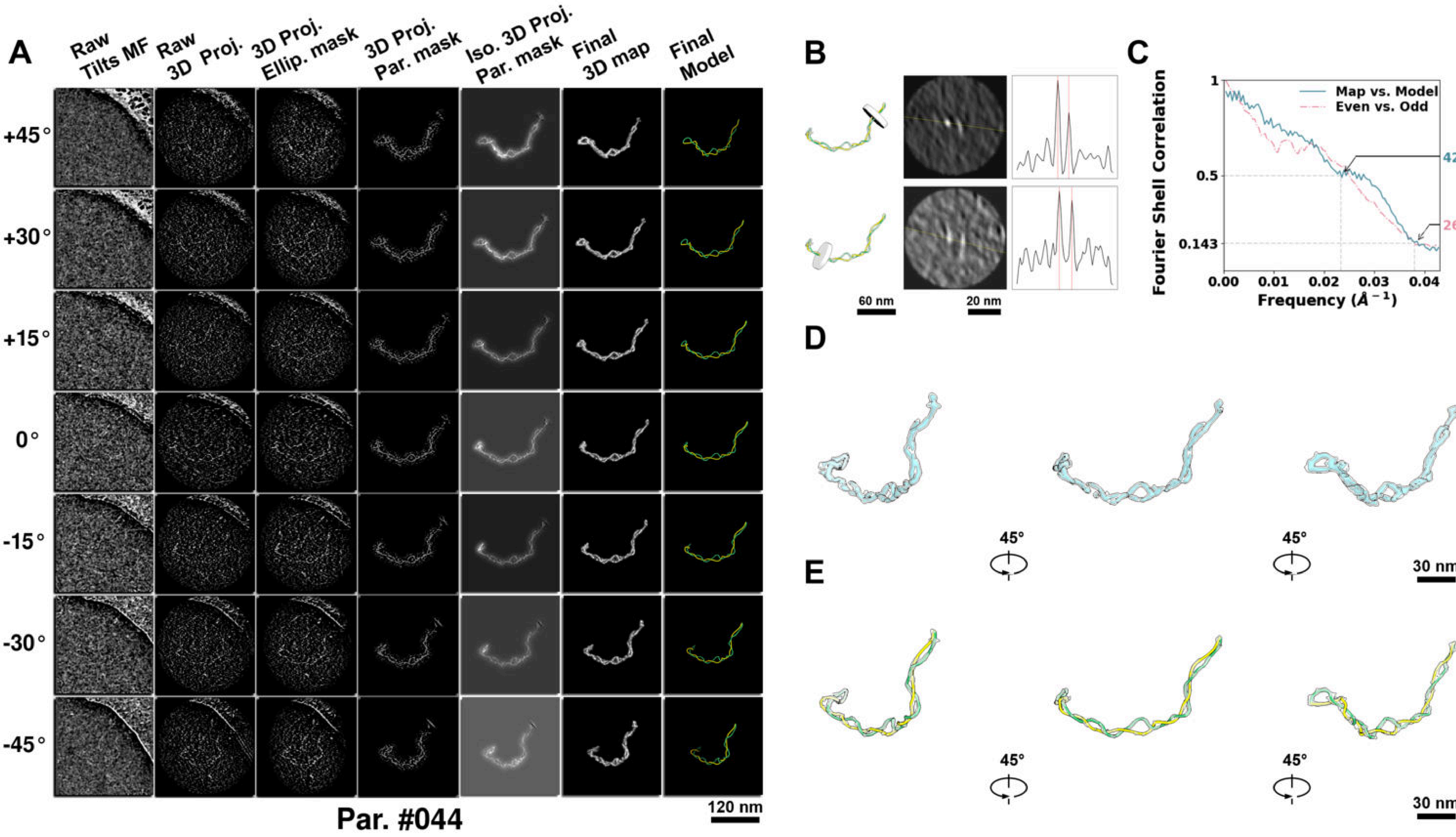

Supplementary Particle Figure 44. Cryo-ET 3D reconstruction of an individual P.HS particle.
(A) 3D reconstruction of the plasmid particle (index no. 44). The first column shows seven representative tilt images from +45° to -45° in step of 15°. The second, third, and fourth columns show 3D projections of the particle with spherical, ellipsoidal (thinner along the z-dimension), and particle-shaped masks, respectively. The fifth column displays the 3D projections of the enhanced and IsoNet missing-wedge-corrected particle. The sixth and seventh columns present the final 3D map and the flexibly fitted model, respectively. (B) Two cross-sectional views (12 nm thickness) of the plasmid density map along its plectoneme axis are shown in the left-middle panel. The intensity profile along the line crossing the two high-density DNA spots is displayed in the right panel. (C) Resolution assessment of the final 3D map using Fourier shell correlation (FSC). Two criteria are shown: FSC between two half-maps reconstructed from even and odd frames (evaluated at 0.143) and FSC between the final 3D map and the fitted model (evaluated at 0.5). (D) Zoomed-in views of the final 3D density map from panel A, displayed at two contour levels. (E) Superimposition of the high-contour level map from panel D onto its fitted model.

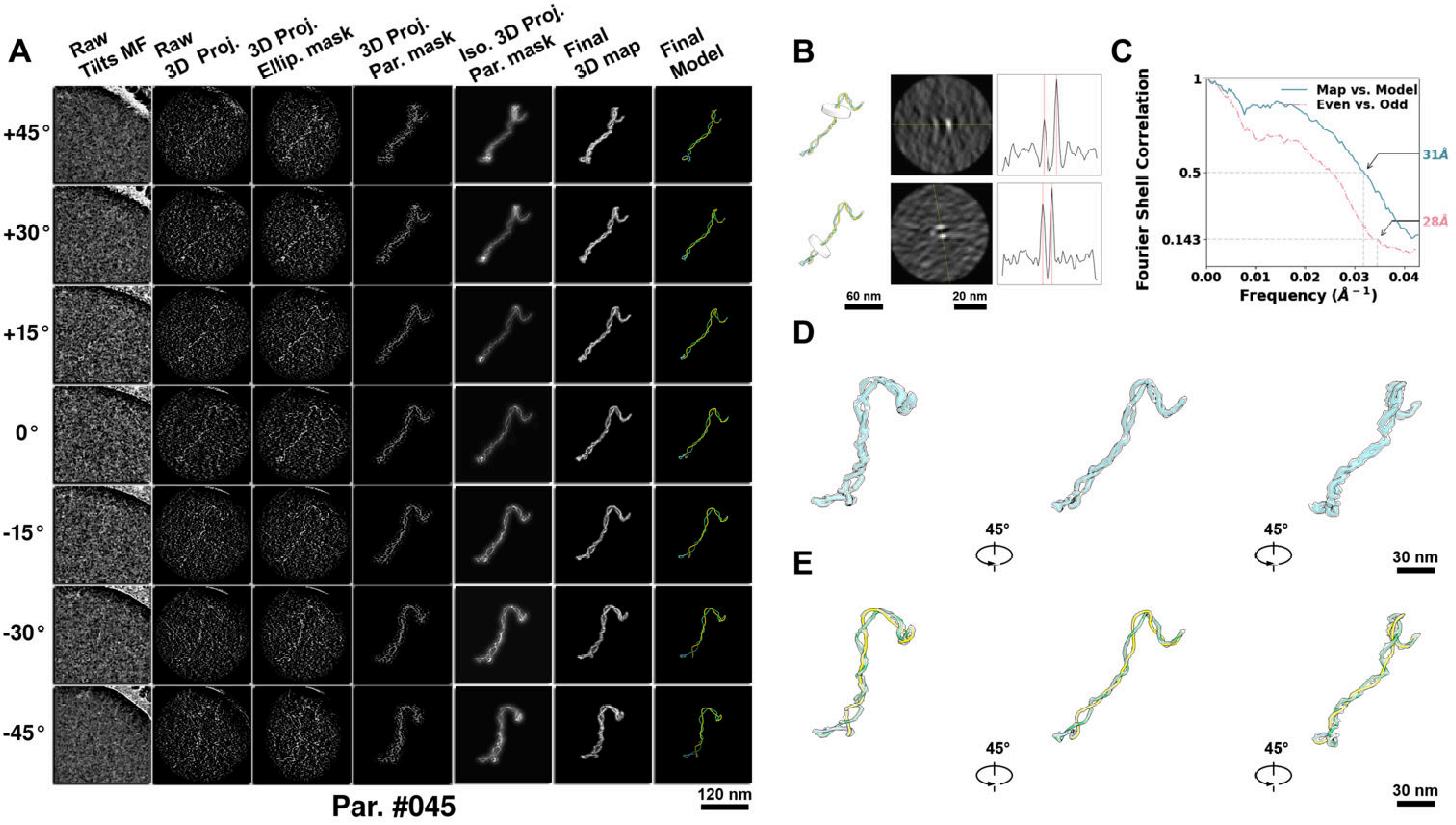

Supplementary Particle Figure 45. Cryo-ET 3D reconstruction of an individual P.HS particle.
(A) 3D reconstruction of the plasmid particle (index no. 45). The first column shows seven representative tilt images from +45° to -45° in step of 15°. The second, third, and fourth columns show 3D projections of the particle with spherical, ellipsoidal (thinner along the z-dimension), and particle-shaped masks, respectively. The fifth column displays the 3D projections of the enhanced and IsoNet missing-wedge-corrected particle. The sixth and seventh columns present the final 3D map and the flexibly fitted model, respectively. (B) Two cross-sectional views (12 nm thickness) of the plasmid density map along its plectoneme axis are shown in the left-middle panel. The intensity profile along the line crossing the two high-density DNA spots is displayed in the right panel. (C) Resolution assessment of the final 3D map using Fourier shell correlation (FSC). Two criteria are shown: FSC between two half-maps reconstructed from even and odd frames (evaluated at 0.143) and FSC between the final 3D map and the fitted model (evaluated at 0.5). (D) Zoomed-in views of the final 3D density map from panel A, displayed at two contour levels. (E) Superimposition of the high-contour level map from panel D onto its fitted model.

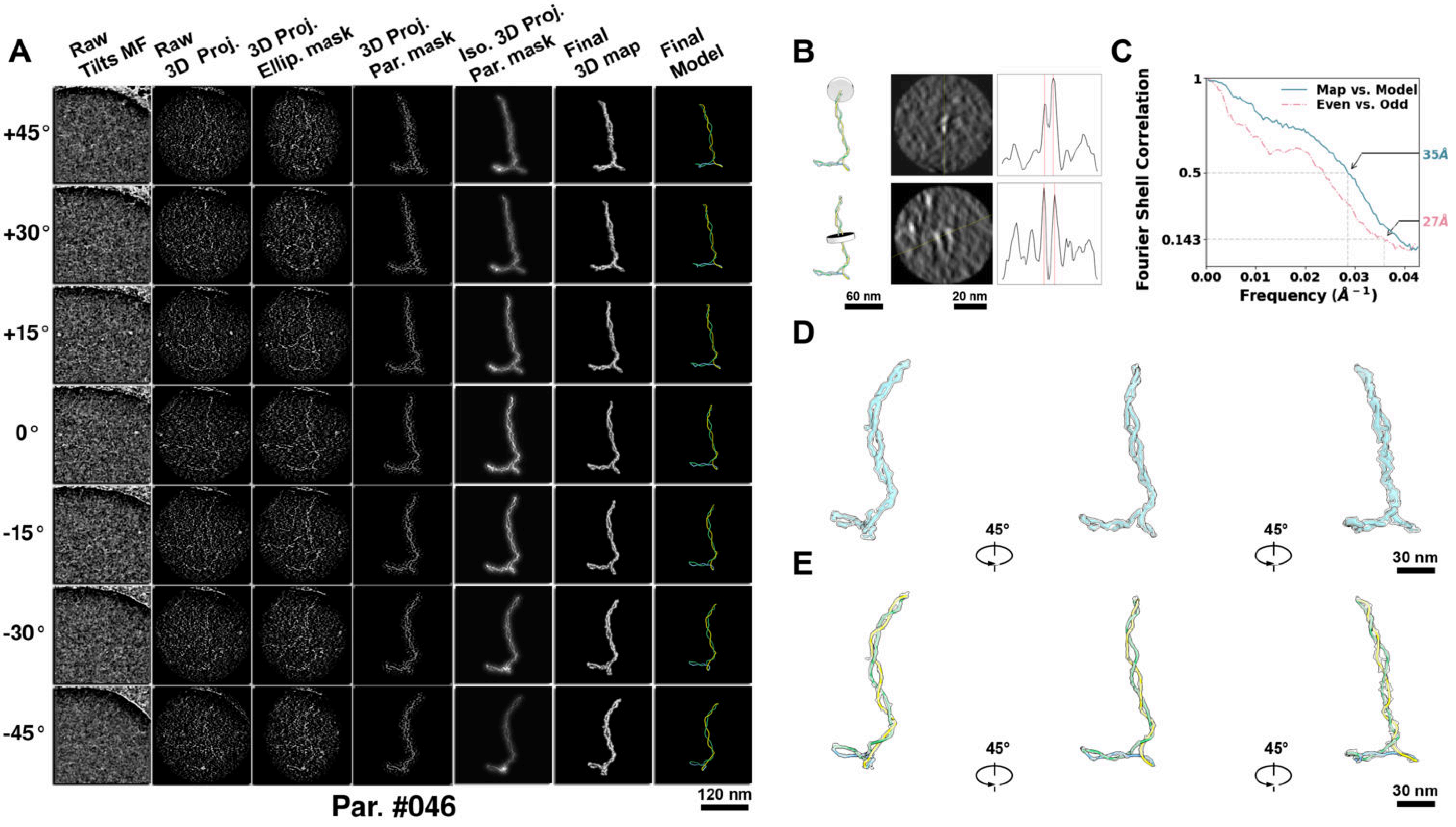

Supplementary Particle Figure 46. Cryo-ET 3D reconstruction of an individual P.HS particle.
(A) 3D reconstruction of the plasmid particle (index no. 46). The first column shows seven representative tilt images from +45° to -45° in step of 15°. The second, third, and fourth columns show 3D projections of the particle with spherical, ellipsoidal (thinner along the z-dimension), and particle-shaped masks, respectively. The fifth column displays the 3D projections of the enhanced and IsoNet missing-wedge-corrected particle. The sixth and seventh columns present the final 3D map and the flexibly fitted model, respectively. (B) Two cross-sectional views (12 nm thickness) of the plasmid density map along its plectoneme axis are shown in the left-middle panel. The intensity profile along the line crossing the two high-density DNA spots is displayed in the right panel. (C) Resolution assessment of the final 3D map using Fourier shell correlation (FSC). Two criteria are shown: FSC between two half-maps reconstructed from even and odd frames (evaluated at 0.143) and FSC between the final 3D map and the fitted model (evaluated at 0.5). (D) Zoomed-in views of the final 3D density map from panel A, displayed at two contour levels. (E) Superimposition of the high-contour level map from panel D onto its fitted model.

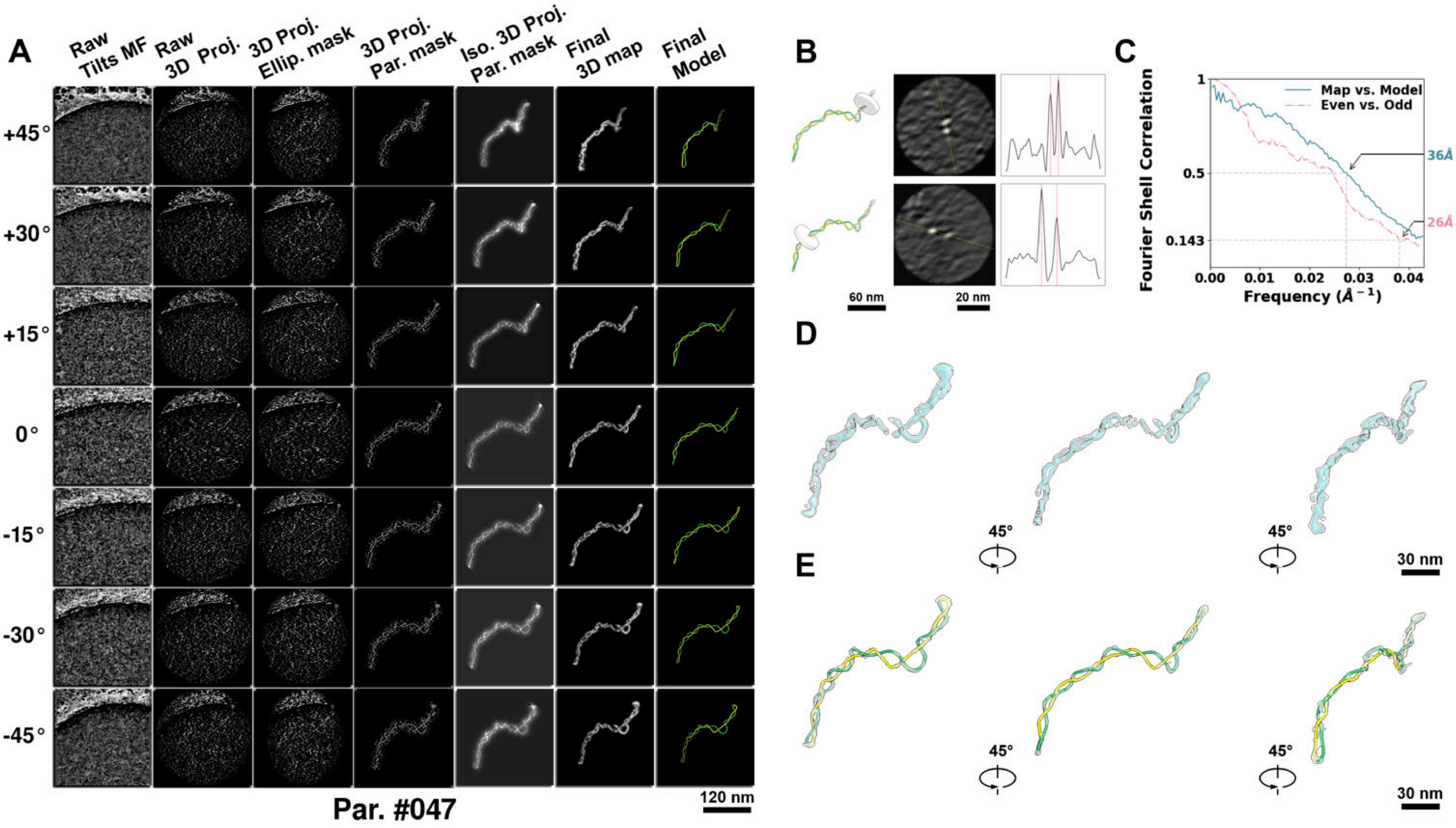

Supplementary Particle Figure 47. Cryo-ET 3D reconstruction of an individual P.HS particle.
(A) 3D reconstruction of the plasmid particle (index no. 47). The first column shows seven representative tilt images from +45° to -45° in step of 15°. The second, third, and fourth columns show 3D projections of the particle with spherical, ellipsoidal (thinner along the z-dimension), and particle-shaped masks, respectively. The fifth column displays the 3D projections of the enhanced and IsoNet missing-wedge-corrected particle. The sixth and seventh columns present the final 3D map and the flexibly fitted model, respectively. (B) Two cross-sectional views (12 nm thickness) of the plasmid density map along its plectoneme axis are shown in the left-middle panel. The intensity profile along the line crossing the two high-density DNA spots is displayed in the right panel. (C) Resolution assessment of the final 3D map using Fourier shell correlation (FSC). Two criteria are shown: FSC between two half-maps reconstructed from even and odd frames (evaluated at 0.143) and FSC between the final 3D map and the fitted model (evaluated at 0.5). (D) Zoomed-in views of the final 3D density map from panel A, displayed at two contour levels. (E) Superimposition of the high-contour level map from panel D onto its fitted model.

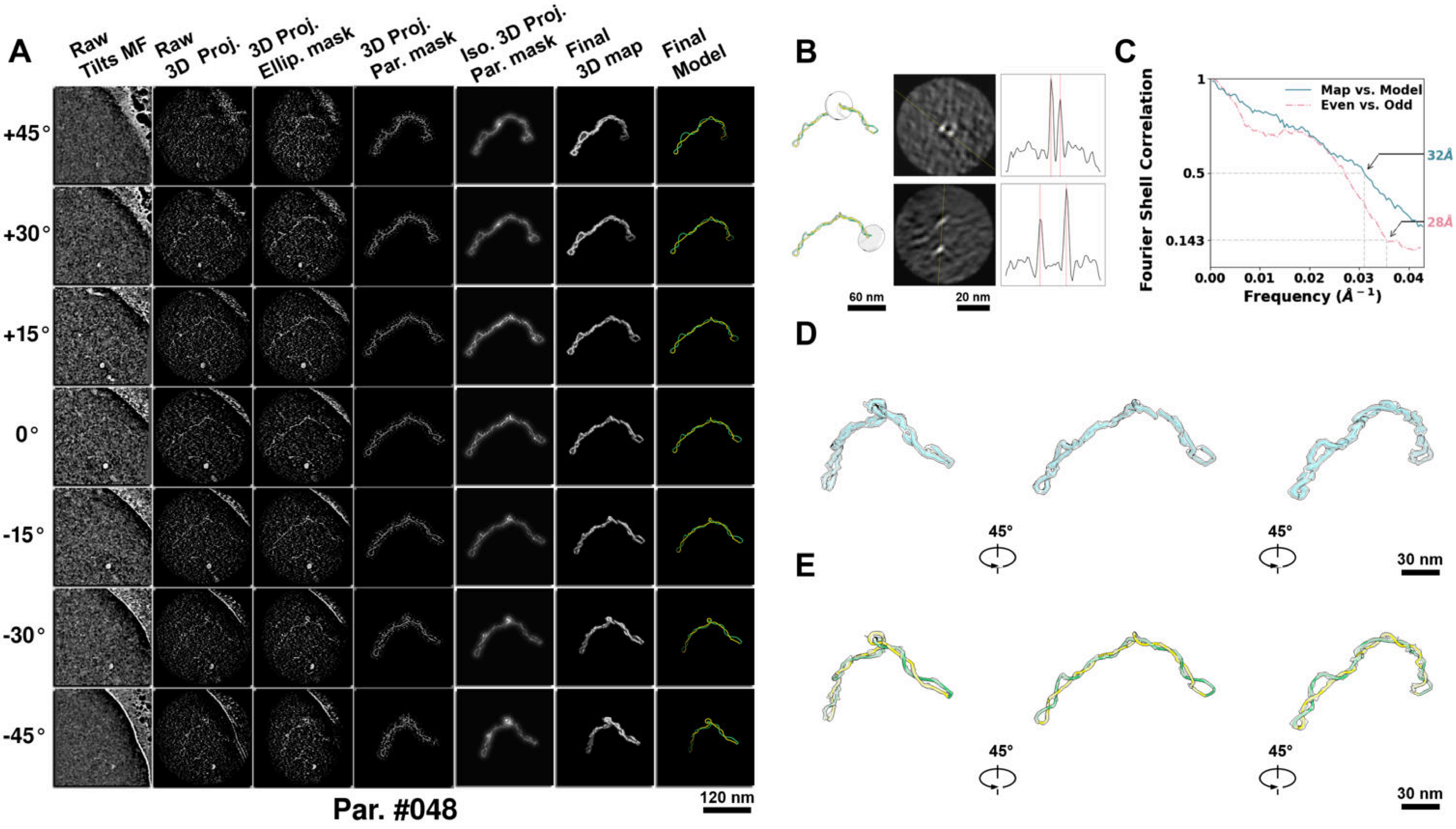

Supplementary Particle Figure 48. Cryo-ET 3D reconstruction of an individual P.HS particle.
(A) 3D reconstruction of the plasmid particle (index no. 48). The first column shows seven representative tilt images from +45° to -45° in step of 15°. The second, third, and fourth columns show 3D projections of the particle with spherical, ellipsoidal (thinner along the z-dimension), and particle-shaped masks, respectively. The fifth column displays the 3D projections of the enhanced and IsoNet missing-wedge-corrected particle. The sixth and seventh columns present the final 3D map and the flexibly fitted model, respectively. (B) Two cross-sectional views (12 nm thickness) of the plasmid density map along its plectoneme axis are shown in the left-middle panel. The intensity profile along the line crossing the two high-density DNA spots is displayed in the right panel. (C) Resolution assessment of the final 3D map using Fourier shell correlation (FSC). Two criteria are shown: FSC between two half-maps reconstructed from even and odd frames (evaluated at 0.143) and FSC between the final 3D map and the fitted model (evaluated at 0.5). (D) Zoomed-in views of the final 3D density map from panel A, displayed at two contour levels. (E) Superimposition of the high-contour level map from panel D onto its fitted model.

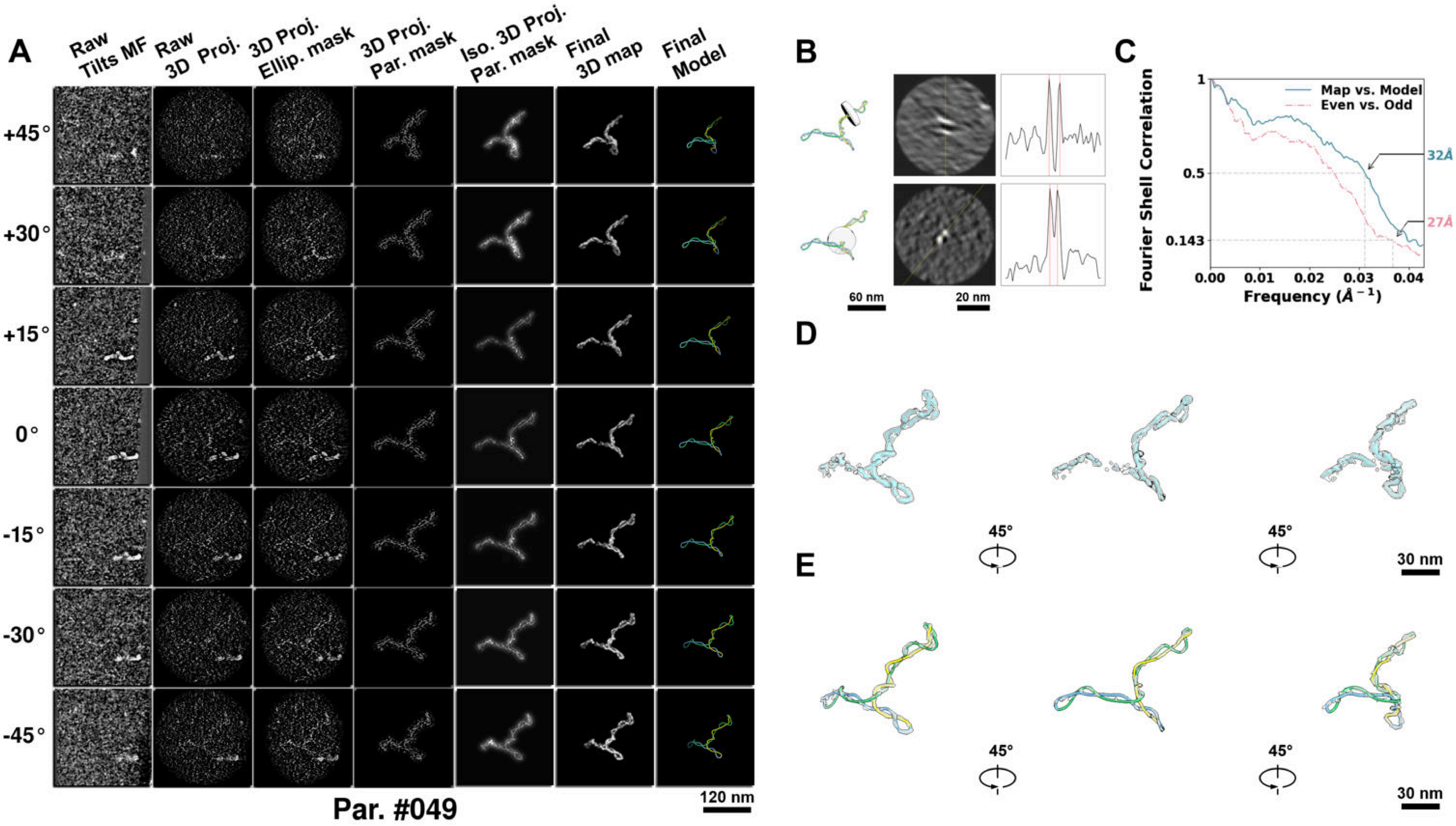

Supplementary Particle Figure 49. Cryo-ET 3D reconstruction of an individual P.HS particle.
(A) 3D reconstruction of the plasmid particle (index no. 49). The first column shows seven representative tilt images from +45° to -45° in step of 15°. The second, third, and fourth columns show 3D projections of the particle with spherical, ellipsoidal (thinner along the z-dimension), and particle-shaped masks, respectively. The fifth column displays the 3D projections of the enhanced and IsoNet missing-wedge-corrected particle. The sixth and seventh columns present the final 3D map and the flexibly fitted model, respectively. (B) Two cross-sectional views (12 nm thickness) of the plasmid density map along its plectoneme axis are shown in the left-middle panel. The intensity profile along the line crossing the two high-density DNA spots is displayed in the right panel. (C) Resolution assessment of the final 3D map using Fourier shell correlation (FSC). Two criteria are shown: FSC between two half-maps reconstructed from even and odd frames (evaluated at 0.143) and FSC between the final 3D map and the fitted model (evaluated at 0.5). (D) Zoomed-in views of the final 3D density map from panel A, displayed at two contour levels. (E) Superimposition of the high-contour level map from panel D onto its fitted model.

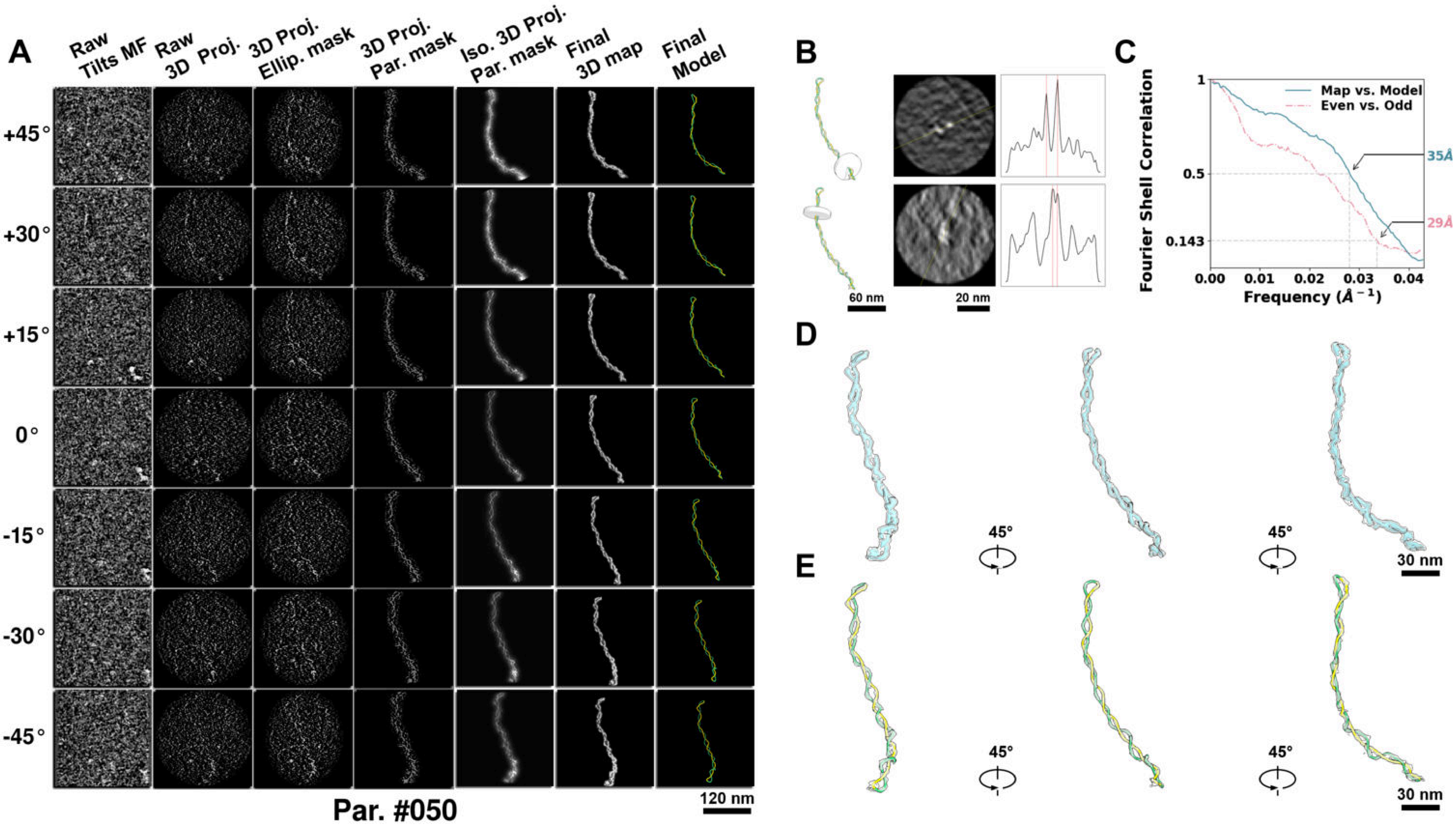

Supplementary Particle Figure 50. Cryo-ET 3D reconstruction of an individual P.HS particle.
(A) 3D reconstruction of the plasmid particle (index no. 50). The first column shows seven representative tilt images from +45° to -45° in step of 15°. The second, third, and fourth columns show 3D projections of the particle with spherical, ellipsoidal (thinner along the z-dimension), and particle-shaped masks, respectively. The fifth column displays the 3D projections of the enhanced and IsoNet missing-wedge-corrected particle. The sixth and seventh columns present the final 3D map and the flexibly fitted model, respectively. (B) Two cross-sectional views (12 nm thickness) of the plasmid density map along its plectoneme axis are shown in the left-middle panel. The intensity profile along the line crossing the two high-density DNA spots is displayed in the right panel. (C) Resolution assessment of the final 3D map using Fourier shell correlation (FSC). Two criteria are shown: FSC between two half-maps reconstructed from even and odd frames (evaluated at 0.143) and FSC between the final 3D map and the fitted model (evaluated at 0.5). (D) Zoomed-in views of the final 3D density map from panel A, displayed at two contour levels. (E) Superimposition of the high-contour level map from panel D onto its fitted model.

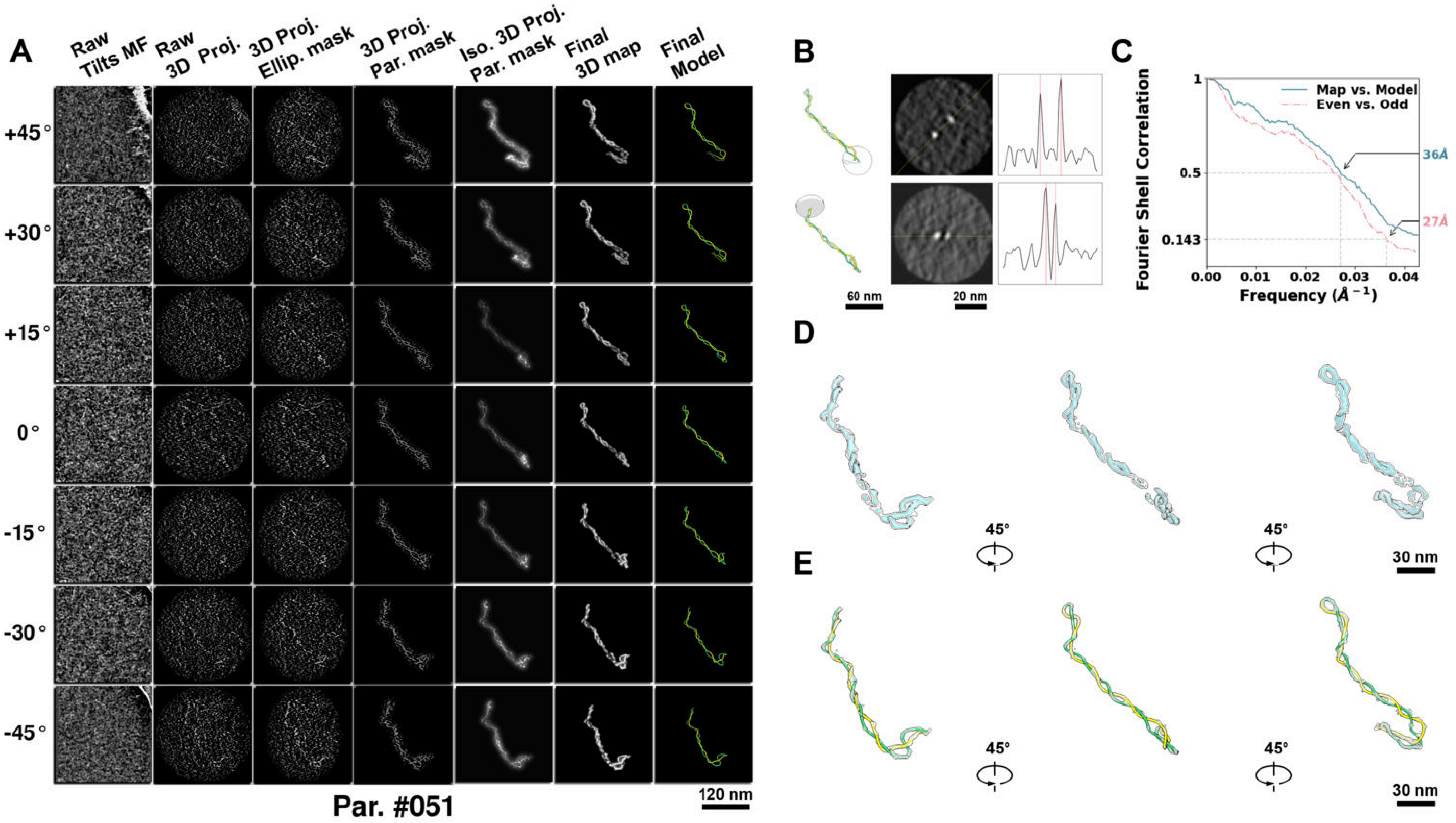

Supplementary Particle Figure 51. Cryo-ET 3D reconstruction of an individual P.HS particle.
(A) 3D reconstruction of the plasmid particle (index no. 51). The first column shows seven representative tilt images from +45° to -45° in step of 15°. The second, third, and fourth columns show 3D projections of the particle with spherical, ellipsoidal (thinner along the z-dimension), and particle-shaped masks, respectively. The fifth column displays the 3D projections of the enhanced and IsoNet missing-wedge-corrected particle. The sixth and seventh columns present the final 3D map and the flexibly fitted model, respectively. (B) Two cross-sectional views (12 nm thickness) of the plasmid density map along its plectoneme axis are shown in the left-middle panel. The intensity profile along the line crossing the two high-density DNA spots is displayed in the right panel. (C) Resolution assessment of the final 3D map using Fourier shell correlation (FSC). Two criteria are shown: FSC between two half-maps reconstructed from even and odd frames (evaluated at 0.143) and FSC between the final 3D map and the fitted model (evaluated at 0.5). (D) Zoomed-in views of the final 3D density map from panel A, displayed at two contour levels. (E) Superimposition of the high-contour level map from panel D onto its fitted model.

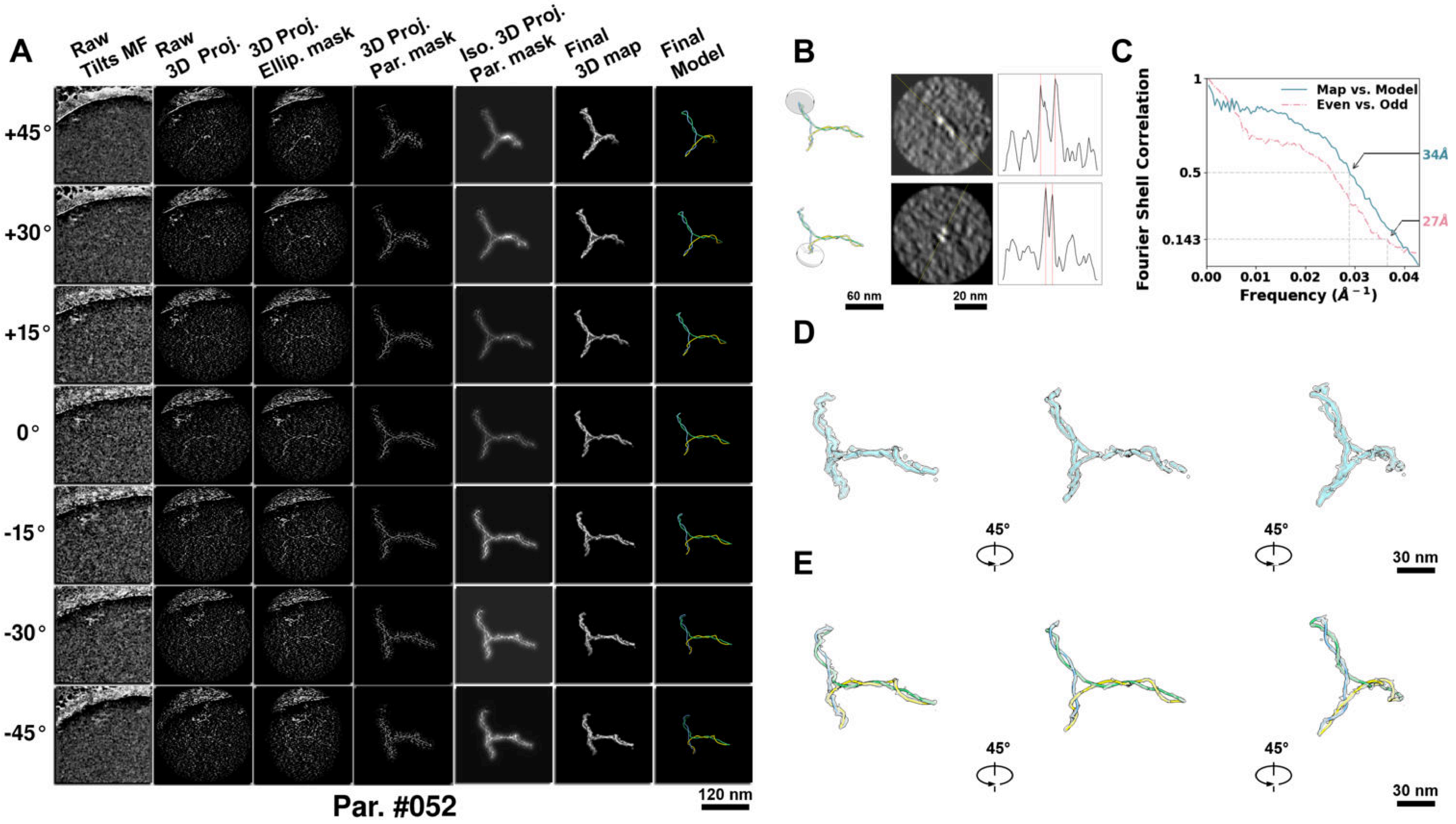

Supplementary Particle Figure 52. Cryo-ET 3D reconstruction of an individual P.HS particle.
(A) 3D reconstruction of the plasmid particle (index no. 52). The first column shows seven representative tilt images from +45° to -45° in step of 15°. The second, third, and fourth columns show 3D projections of the particle with spherical, ellipsoidal (thinner along the z-dimension), and particle-shaped masks, respectively. The fifth column displays the 3D projections of the enhanced and IsoNet missing-wedge-corrected particle. The sixth and seventh columns present the final 3D map and the flexibly fitted model, respectively. (B) Two cross-sectional views (12 nm thickness) of the plasmid density map along its plectoneme axis are shown in the left-middle panel. The intensity profile along the line crossing the two high-density DNA spots is displayed in the right panel. (C) Resolution assessment of the final 3D map using Fourier shell correlation (FSC). Two criteria are shown: FSC between two half-maps reconstructed from even and odd frames (evaluated at 0.143) and FSC between the final 3D map and the fitted model (evaluated at 0.5). (D) Zoomed-in views of the final 3D density map from panel A, displayed at two contour levels. (E) Superimposition of the high-contour level map from panel D onto its fitted model.

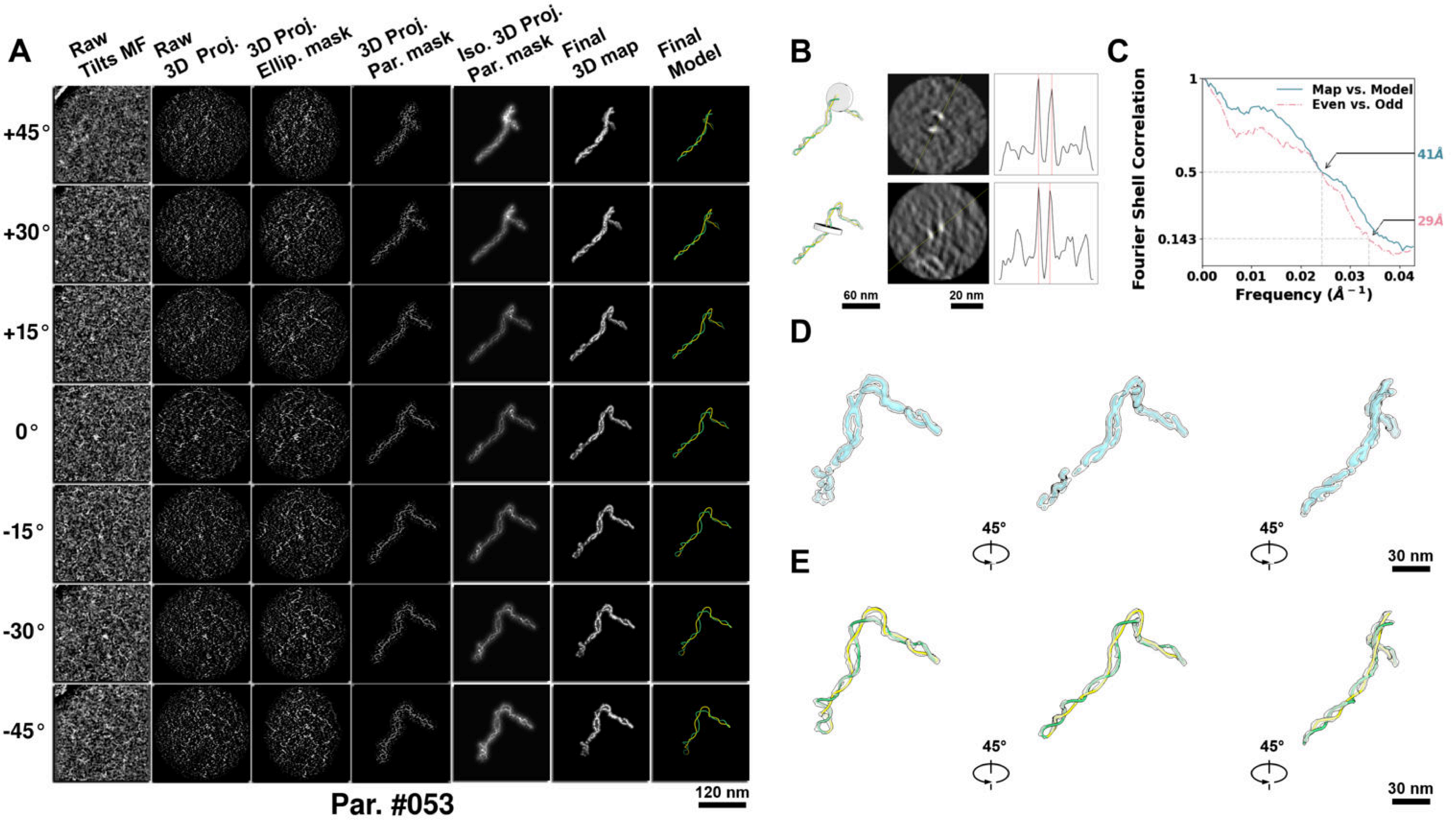

Supplementary Particle Figure 53. Cryo-ET 3D reconstruction of an individual P.HS particle.
(A) 3D reconstruction of the plasmid particle (index no. 53). The first column shows seven representative tilt images from +45° to -45° in step of 15°. The second, third, and fourth columns show 3D projections of the particle with spherical, ellipsoidal (thinner along the z-dimension), and particle-shaped masks, respectively. The fifth column displays the 3D projections of the enhanced and IsoNet missing-wedge-corrected particle. The sixth and seventh columns present the final 3D map and the flexibly fitted model, respectively. (B) Two cross-sectional views (12 nm thickness) of the plasmid density map along its plectoneme axis are shown in the left-middle panel. The intensity profile along the line crossing the two high-density DNA spots is displayed in the right panel. (C) Resolution assessment of the final 3D map using Fourier shell correlation (FSC). Two criteria are shown: FSC between two half-maps reconstructed from even and odd frames (evaluated at 0.143) and FSC between the final 3D map and the fitted model (evaluated at 0.5). (D) Zoomed-in views of the final 3D density map from panel A, displayed at two contour levels. (E) Superimposition of the high-contour level map from panel D onto its fitted model.

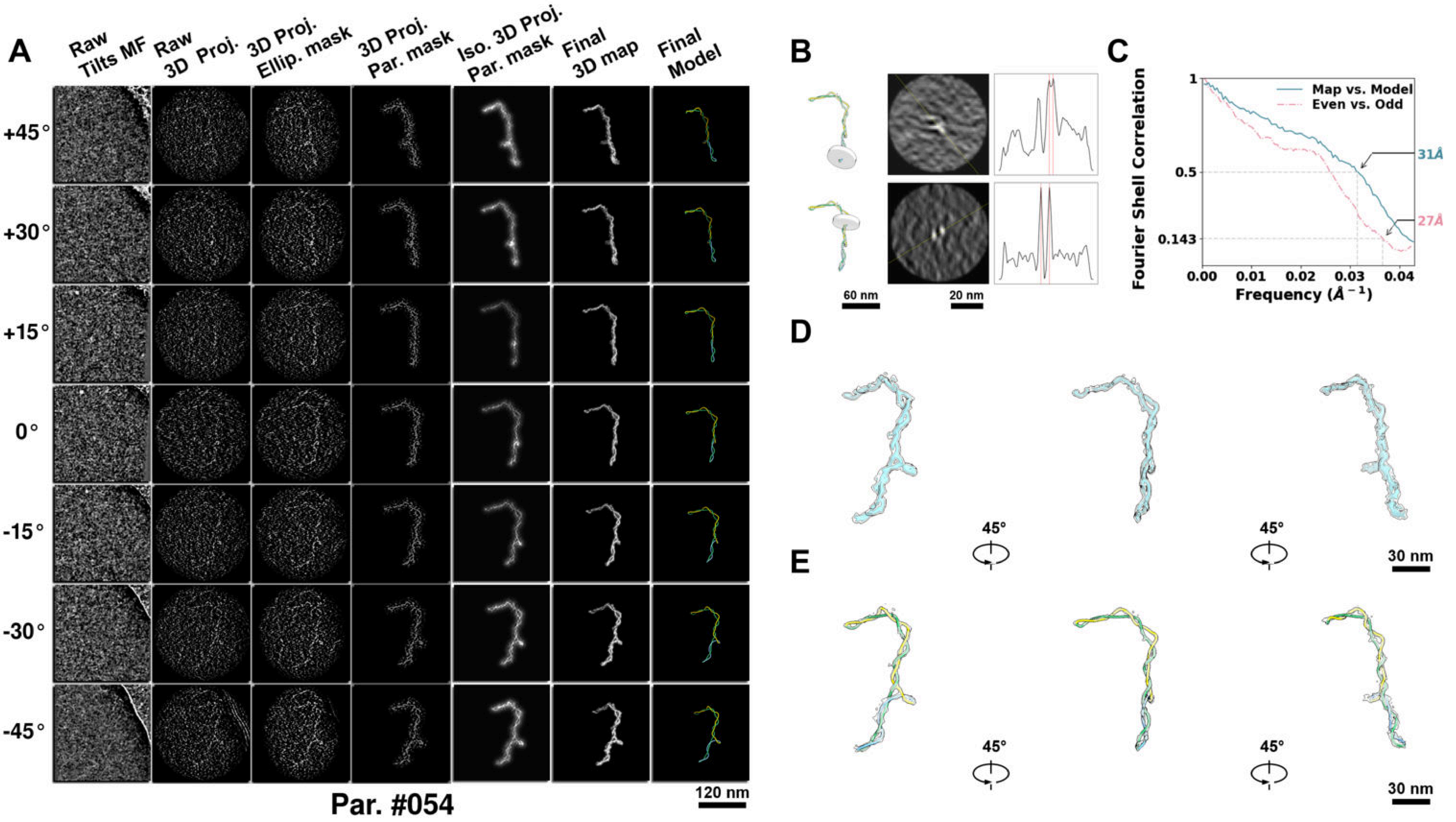

Supplementary Particle Figure 54. Cryo-ET 3D reconstruction of an individual P.HS particle.
(A) 3D reconstruction of the plasmid particle (index no. 54). The first column shows seven representative tilt images from +45° to -45° in step of 15°. The second, third, and fourth columns show 3D projections of the particle with spherical, ellipsoidal (thinner along the z-dimension), and particle-shaped masks, respectively. The fifth column displays the 3D projections of the enhanced and IsoNet missing-wedge-corrected particle. The sixth and seventh columns present the final 3D map and the flexibly fitted model, respectively. (B) Two cross-sectional views (12 nm thickness) of the plasmid density map along its plectoneme axis are shown in the left-middle panel. The intensity profile along the line crossing the two high-density DNA spots is displayed in the right panel. (C) Resolution assessment of the final 3D map using Fourier shell correlation (FSC). Two criteria are shown: FSC between two half-maps reconstructed from even and odd frames (evaluated at 0.143) and FSC between the final 3D map and the fitted model (evaluated at 0.5). (D) Zoomed-in views of the final 3D density map from panel A, displayed at two contour levels. (E) Superimposition of the high-contour level map from panel D onto its fitted model.

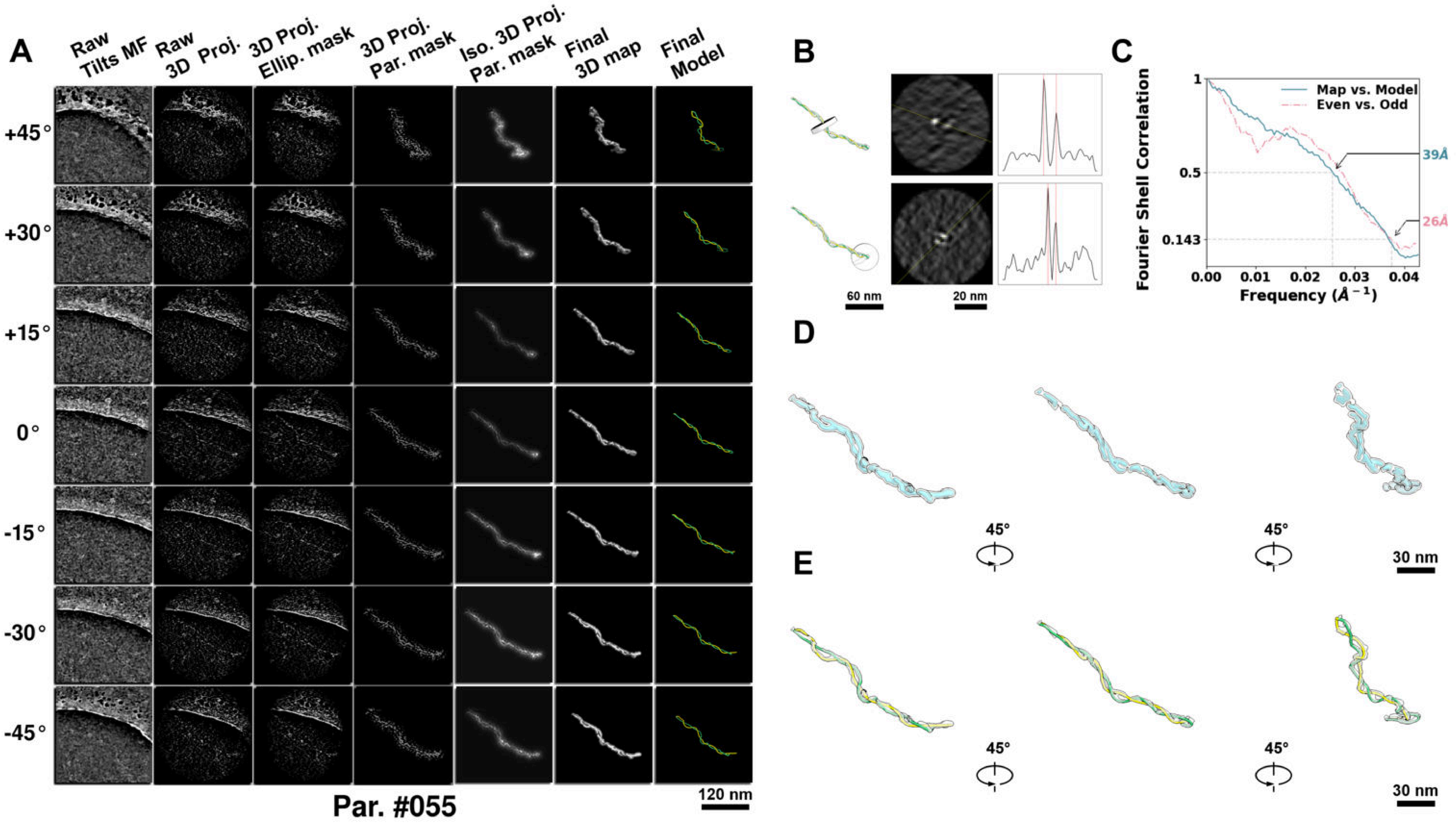

Supplementary Particle Figure 55. Cryo-ET 3D reconstruction of an individual P.HS particle.
(A) 3D reconstruction of the plasmid particle (index no. 55). The first column shows seven representative tilt images from +45° to -45° in step of 15°. The second, third, and fourth columns show 3D projections of the particle with spherical, ellipsoidal (thinner along the z-dimension), and particle-shaped masks, respectively. The fifth column displays the 3D projections of the enhanced and IsoNet missing-wedge-corrected particle. The sixth and seventh columns present the final 3D map and the flexibly fitted model, respectively. (B) Two cross-sectional views (12 nm thickness) of the plasmid density map along its plectoneme axis are shown in the left-middle panel. The intensity profile along the line crossing the two high-density DNA spots is displayed in the right panel. (C) Resolution assessment of the final 3D map using Fourier shell correlation (FSC). Two criteria are shown: FSC between two half-maps reconstructed from even and odd frames (evaluated at 0.143) and FSC between the final 3D map and the fitted model (evaluated at 0.5). (D) Zoomed-in views of the final 3D density map from panel A, displayed at two contour levels. (E) Superimposition of the high-contour level map from panel D onto its fitted model.

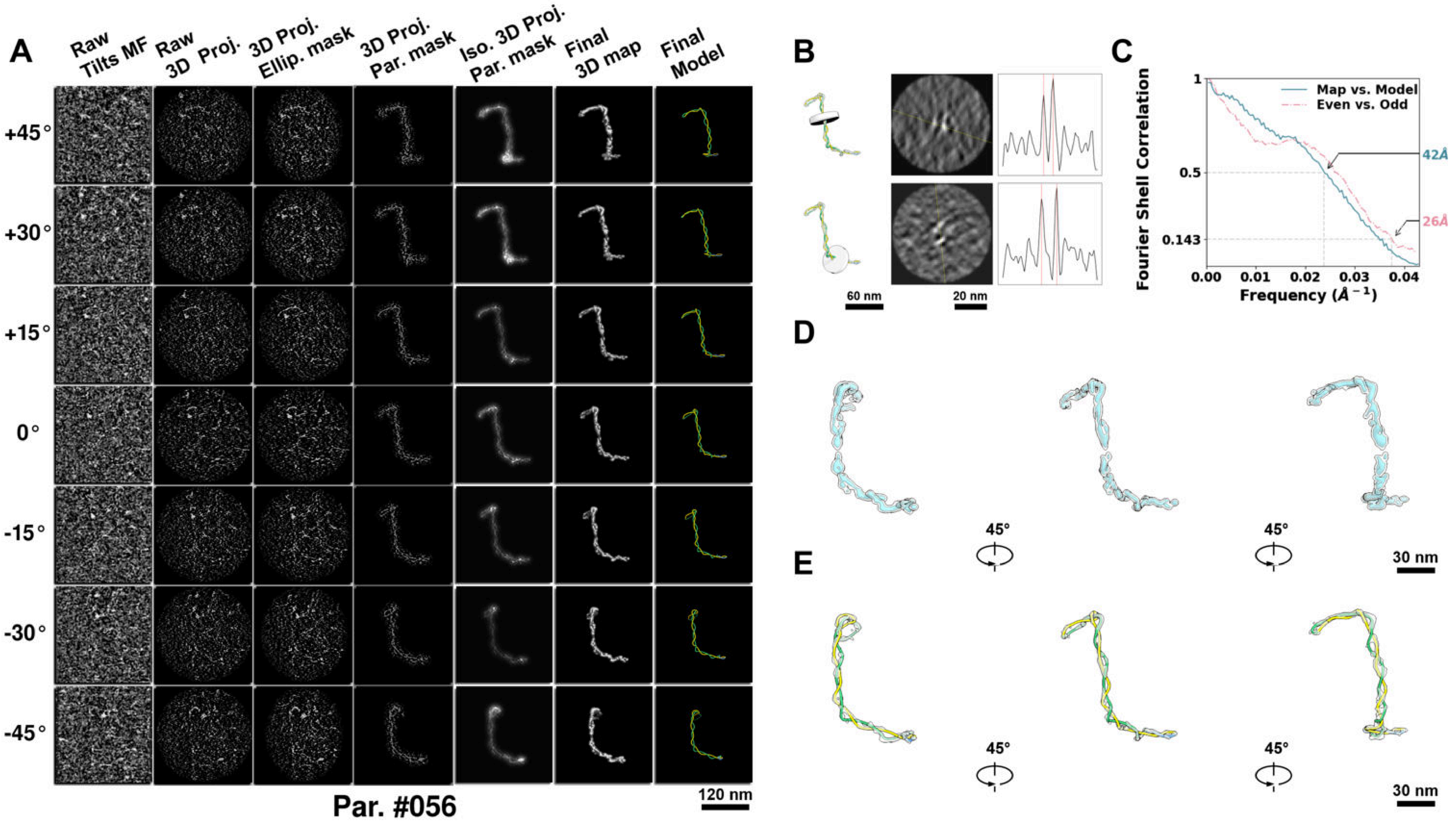

Supplementary Particle Figure 56. Cryo-ET 3D reconstruction of an individual P.HS particle.
(A) 3D reconstruction of the plasmid particle (index no. 56). The first column shows seven representative tilt images from +45° to -45° in step of 15°. The second, third, and fourth columns show 3D projections of the particle with spherical, ellipsoidal (thinner along the z-dimension), and particle-shaped masks, respectively. The fifth column displays the 3D projections of the enhanced and IsoNet missing-wedge-corrected particle. The sixth and seventh columns present the final 3D map and the flexibly fitted model, respectively. (B) Two cross-sectional views (12 nm thickness) of the plasmid density map along its plectoneme axis are shown in the left-middle panel. The intensity profile along the line crossing the two high-density DNA spots is displayed in the right panel. (C) Resolution assessment of the final 3D map using Fourier shell correlation (FSC). Two criteria are shown: FSC between two half-maps reconstructed from even and odd frames (evaluated at 0.143) and FSC between the final 3D map and the fitted model (evaluated at 0.5). (D) Zoomed-in views of the final 3D density map from panel A, displayed at two contour levels. (E) Superimposition of the high-contour level map from panel D onto its fitted model.

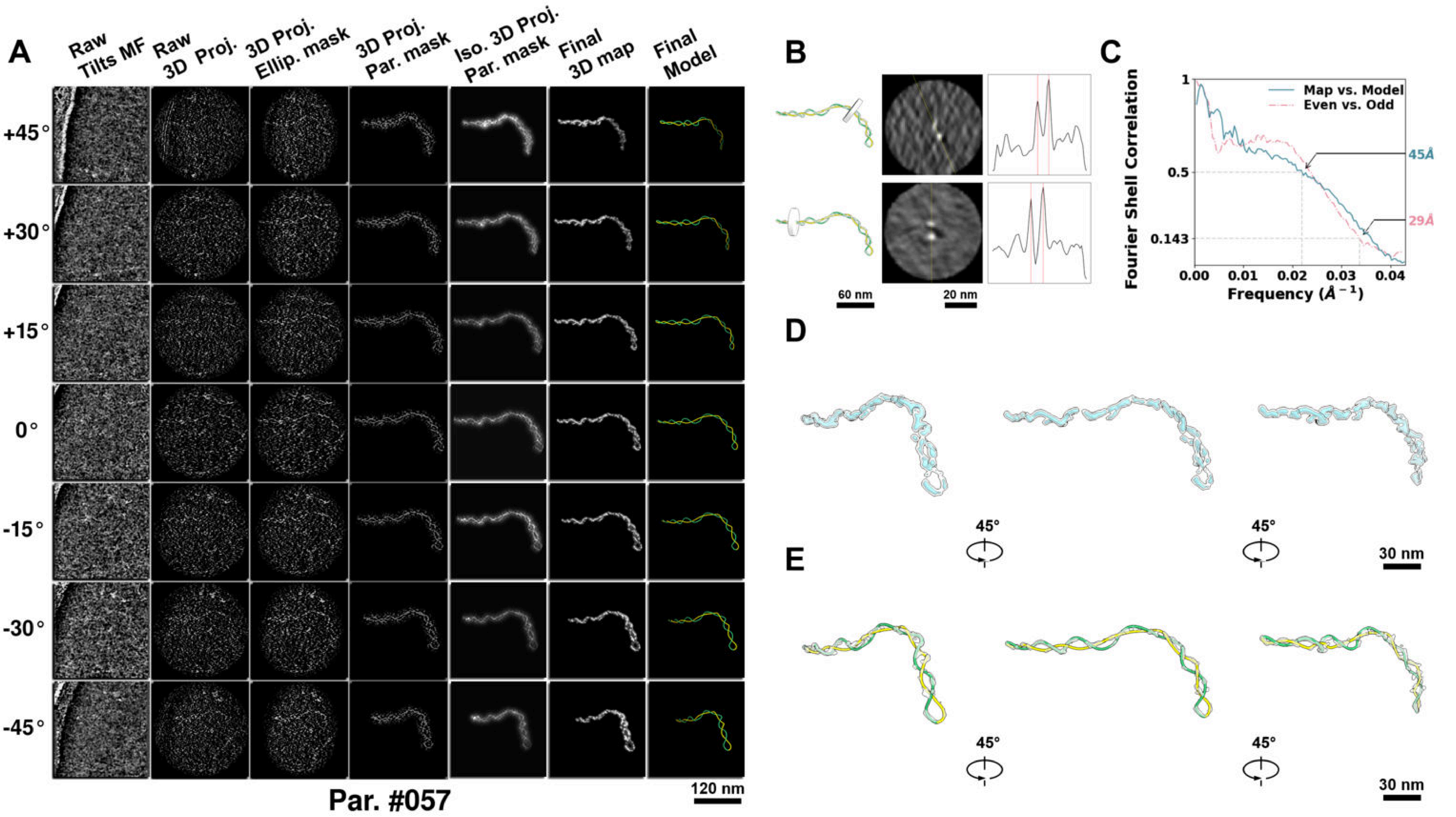

Supplementary Particle Figure 57. Cryo-ET 3D reconstruction of an individual P.HS particle.
(A) 3D reconstruction of the plasmid particle (index no. 57). The first column shows seven representative tilt images from +45° to -45° in step of 15°. The second, third, and fourth columns show 3D projections of the particle with spherical, ellipsoidal (thinner along the z-dimension), and particle-shaped masks, respectively. The fifth column displays the 3D projections of the enhanced and IsoNet missing-wedge-corrected particle. The sixth and seventh columns present the final 3D map and the flexibly fitted model, respectively. (B) Two cross-sectional views (12 nm thickness) of the plasmid density map along its plectoneme axis are shown in the left-middle panel. The intensity profile along the line crossing the two high-density DNA spots is displayed in the right panel. (C) Resolution assessment of the final 3D map using Fourier shell correlation (FSC). Two criteria are shown: FSC between two half-maps reconstructed from even and odd frames (evaluated at 0.143) and FSC between the final 3D map and the fitted model (evaluated at 0.5). (D) Zoomed-in views of the final 3D density map from panel A, displayed at two contour levels. (E) Superimposition of the high-contour level map from panel D onto its fitted model.

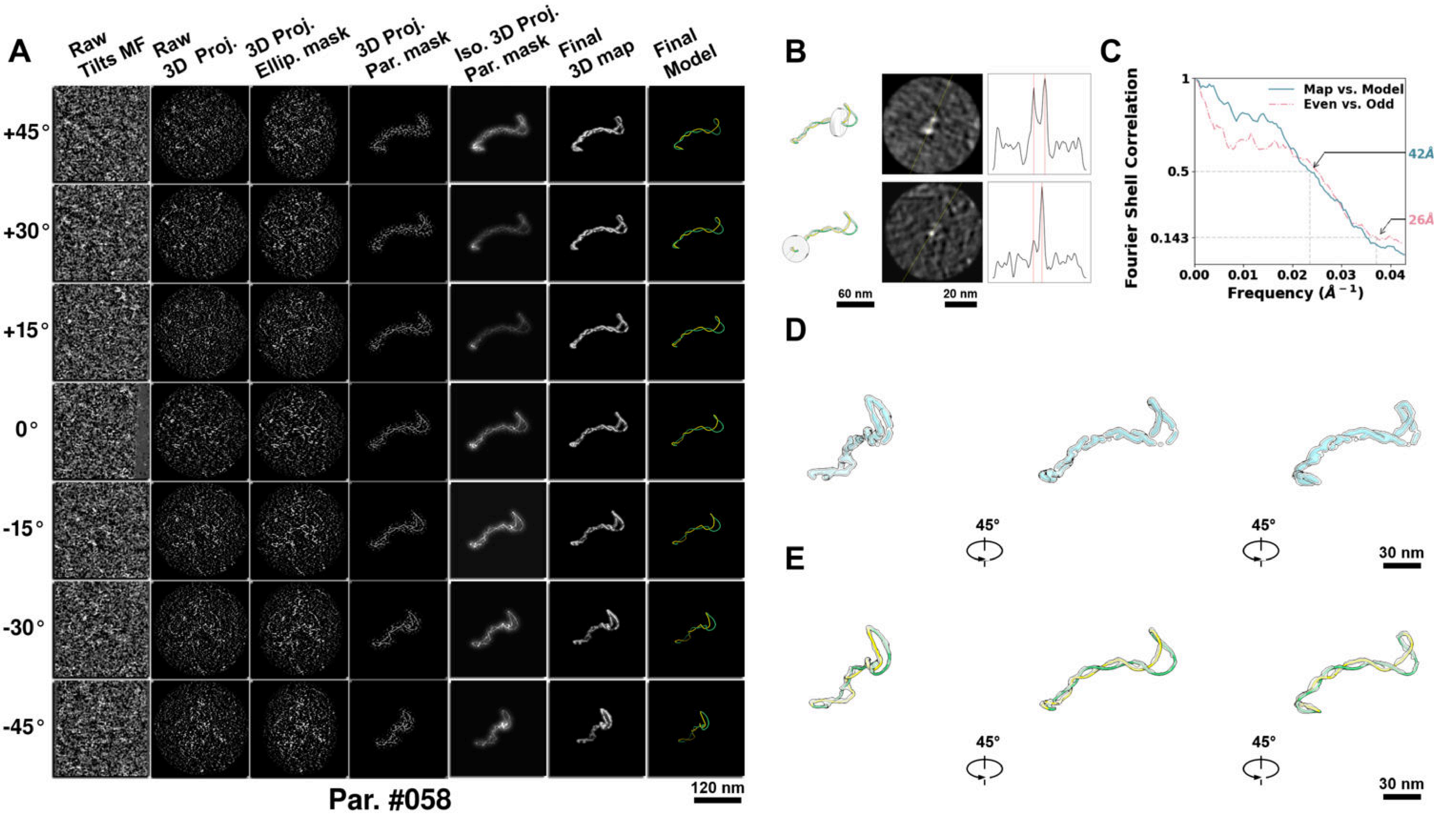

Supplementary Particle Figure 58. Cryo-ET 3D reconstruction of an individual P.HS particle.
(A) 3D reconstruction of the plasmid particle (index no. 58). The first column shows seven representative tilt images from +45° to -45° in step of 15°. The second, third, and fourth columns show 3D projections of the particle with spherical, ellipsoidal (thinner along the z-dimension), and particle-shaped masks, respectively. The fifth column displays the 3D projections of the enhanced and IsoNet missing-wedge-corrected particle. The sixth and seventh columns present the final 3D map and the flexibly fitted model, respectively. (B) Two cross-sectional views (12 nm thickness) of the plasmid density map along its plectoneme axis are shown in the left-middle panel. The intensity profile along the line crossing the two high-density DNA spots is displayed in the right panel. (C) Resolution assessment of the final 3D map using Fourier shell correlation (FSC). Two criteria are shown: FSC between two half-maps reconstructed from even and odd frames (evaluated at 0.143) and FSC between the final 3D map and the fitted model (evaluated at 0.5). (D) Zoomed-in views of the final 3D density map from panel A, displayed at two contour levels. (E) Superimposition of the high-contour level map from panel D onto its fitted model.

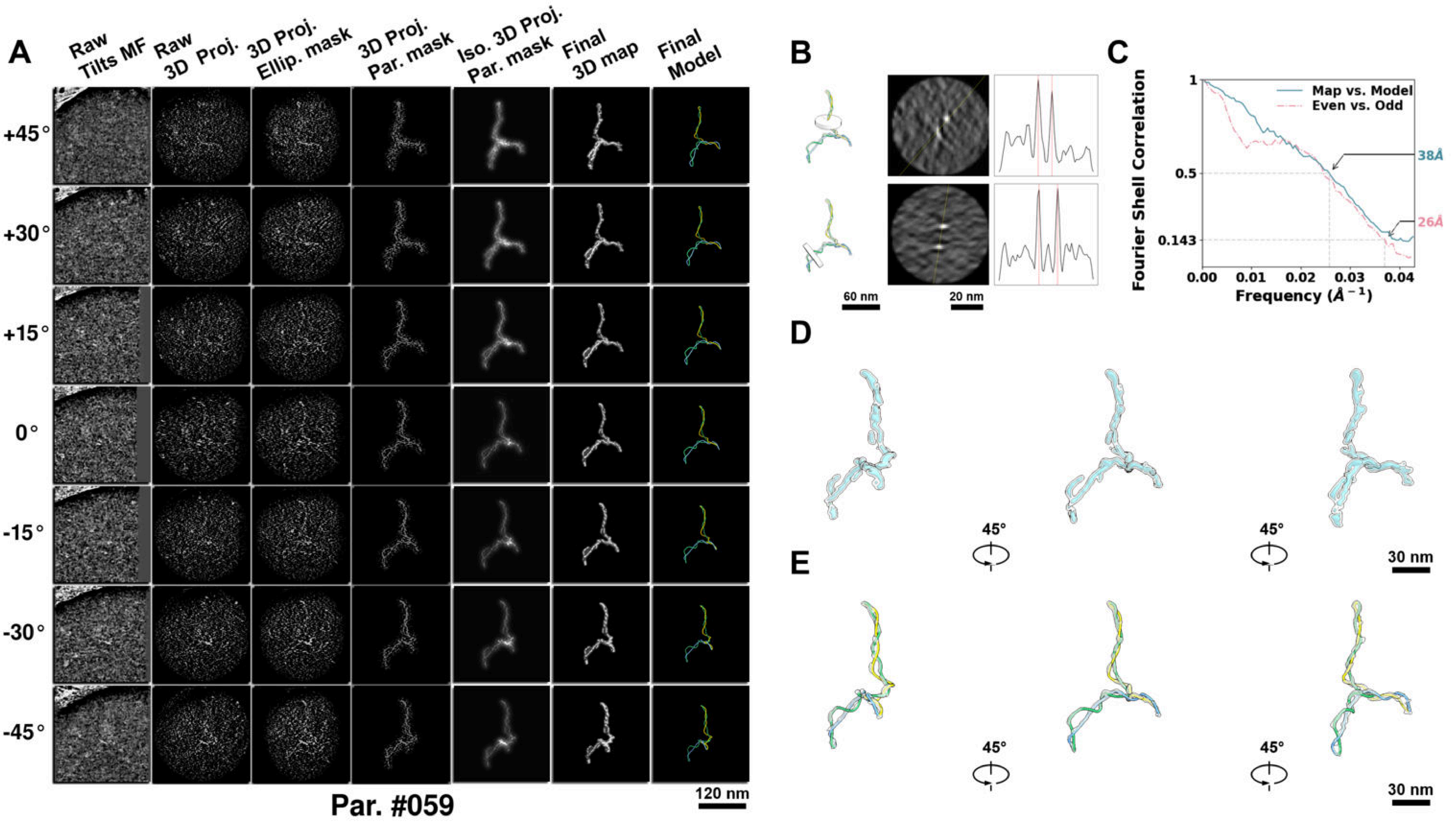

Supplementary Particle Figure 59. Cryo-ET 3D reconstruction of an individual P.HS particle.
(A) 3D reconstruction of the plasmid particle (index no. 59). The first column shows seven representative tilt images from +45° to -45° in step of 15°. The second, third, and fourth columns show 3D projections of the particle with spherical, ellipsoidal (thinner along the z-dimension), and particle-shaped masks, respectively. The fifth column displays the 3D projections of the enhanced and IsoNet missing-wedge-corrected particle. The sixth and seventh columns present the final 3D map and the flexibly fitted model, respectively. (B) Two cross-sectional views (12 nm thickness) of the plasmid density map along its plectoneme axis are shown in the left-middle panel. The intensity profile along the line crossing the two high-density DNA spots is displayed in the right panel. (C) Resolution assessment of the final 3D map using Fourier shell correlation (FSC). Two criteria are shown: FSC between two half-maps reconstructed from even and odd frames (evaluated at 0.143) and FSC between the final 3D map and the fitted model (evaluated at 0.5). (D) Zoomed-in views of the final 3D density map from panel A, displayed at two contour levels. (E) Superimposition of the high-contour level map from panel D onto its fitted model.

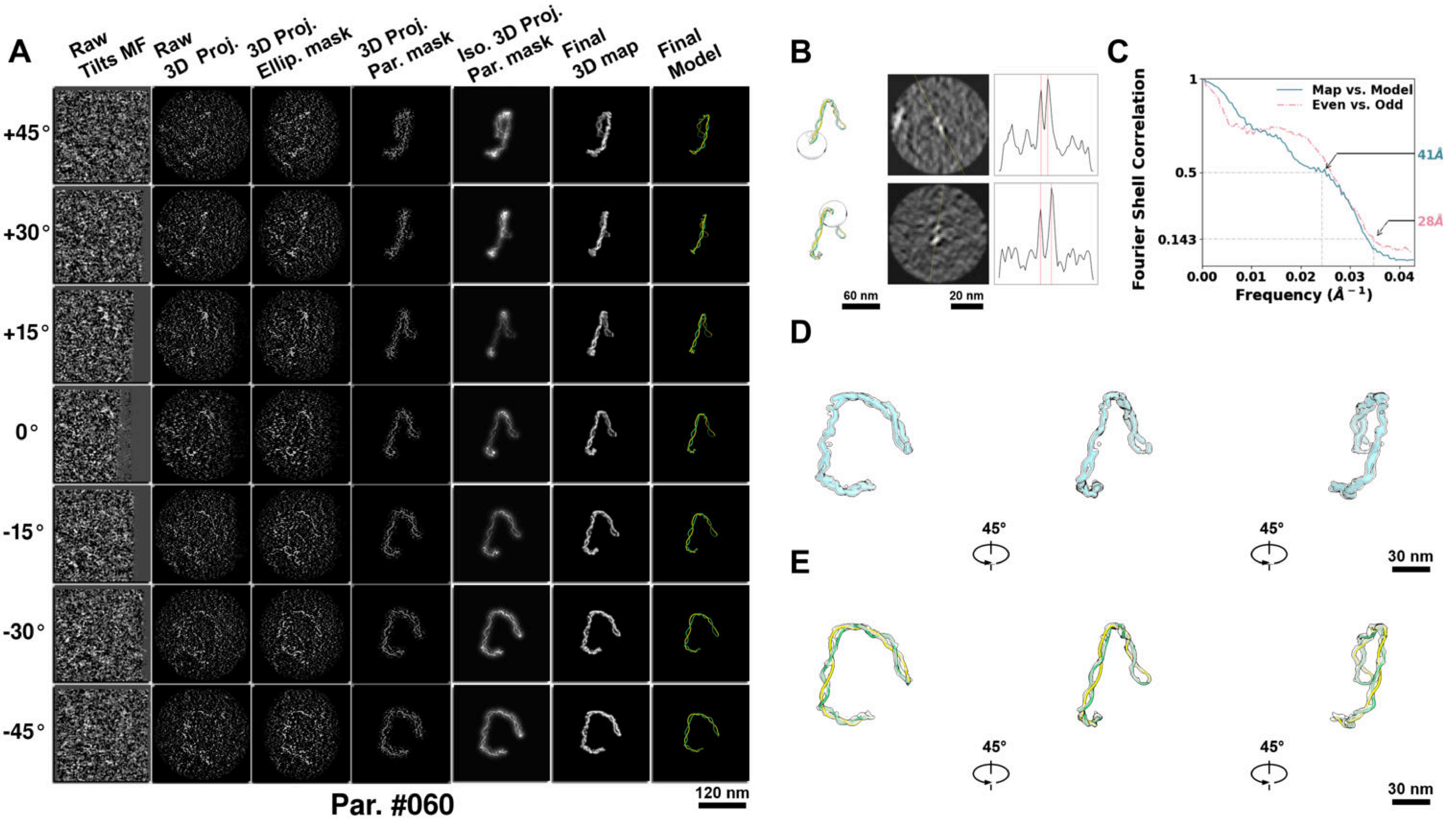

Supplementary Particle Figure 60. Cryo-ET 3D reconstruction of an individual P.HS particle.
(A) 3D reconstruction of the plasmid particle (index no. 60). The first column shows seven representative tilt images from +45° to -45° in step of 15°. The second, third, and fourth columns show 3D projections of the particle with spherical, ellipsoidal (thinner along the z-dimension), and particle-shaped masks, respectively. The fifth column displays the 3D projections of the enhanced and IsoNet missing-wedge-corrected particle. The sixth and seventh columns present the final 3D map and the flexibly fitted model, respectively. (B) Two cross-sectional views (12 nm thickness) of the plasmid density map along its plectoneme axis are shown in the left-middle panel. The intensity profile along the line crossing the two high-density DNA spots is displayed in the right panel. (C) Resolution assessment of the final 3D map using Fourier shell correlation (FSC). Two criteria are shown: FSC between two half-maps reconstructed from even and odd frames (evaluated at 0.143) and FSC between the final 3D map and the fitted model (evaluated at 0.5). (D) Zoomed-in views of the final 3D density map from panel A, displayed at two contour levels. (E) Superimposition of the high-contour level map from panel D onto its fitted model.

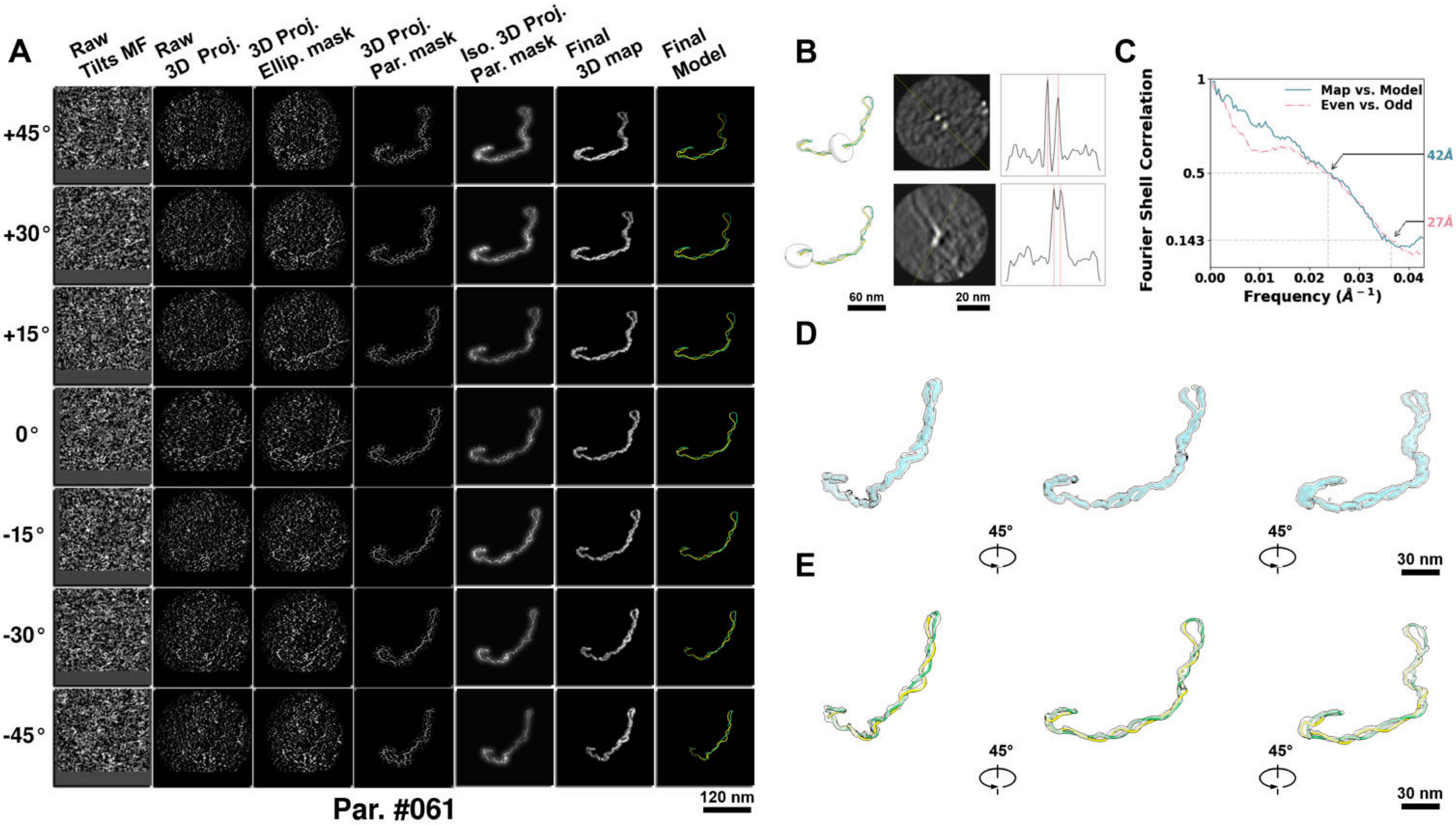

Supplementary Particle Figure 61. Cryo-ET 3D reconstruction of an individual P.HS particle.
(A) 3D reconstruction of the plasmid particle (index no. 61). The first column shows seven representative tilt images from +45° to -45° in step of 15°. The second, third, and fourth columns show 3D projections of the particle with spherical, ellipsoidal (thinner along the z-dimension), and particle-shaped masks, respectively. The fifth column displays the 3D projections of the enhanced and IsoNet missing-wedge-corrected particle. The sixth and seventh columns present the final 3D map and the flexibly fitted model, respectively. (B) Two cross-sectional views (12 nm thickness) of the plasmid density map along its plectoneme axis are shown in the left-middle panel. The intensity profile along the line crossing the two high-density DNA spots is displayed in the right panel. (C) Resolution assessment of the final 3D map using Fourier shell correlation (FSC). Two criteria are shown: FSC between two half-maps reconstructed from even and odd frames (evaluated at 0.143) and FSC between the final 3D map and the fitted model (evaluated at 0.5). (D) Zoomed-in views of the final 3D density map from panel A, displayed at two contour levels. (E) Superimposition of the high-contour level map from panel D onto its fitted model.

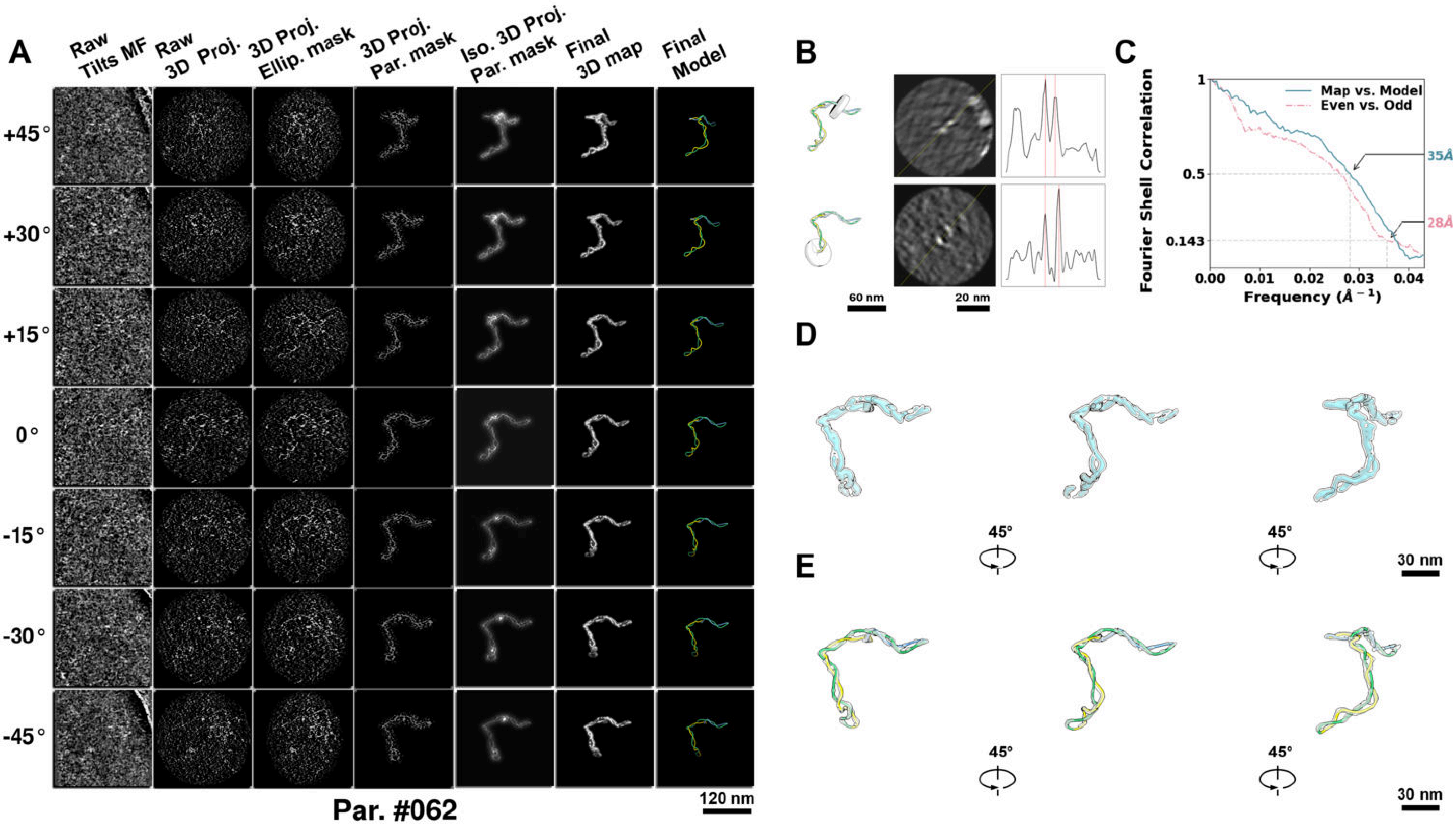

Supplementary Particle Figure 62. Cryo-ET 3D reconstruction of an individual P.HS particle.
(A) 3D reconstruction of the plasmid particle (index no. 62). The first column shows seven representative tilt images from +45° to -45° in step of 15°. The second, third, and fourth columns show 3D projections of the particle with spherical, ellipsoidal (thinner along the z-dimension), and particle-shaped masks, respectively. The fifth column displays the 3D projections of the enhanced and IsoNet missing-wedge-corrected particle. The sixth and seventh columns present the final 3D map and the flexibly fitted model, respectively. (B) Two cross-sectional views (12 nm thickness) of the plasmid density map along its plectoneme axis are shown in the left-middle panel. The intensity profile along the line crossing the two high-density DNA spots is displayed in the right panel. (C) Resolution assessment of the final 3D map using Fourier shell correlation (FSC). Two criteria are shown: FSC between two half-maps reconstructed from even and odd frames (evaluated at 0.143) and FSC between the final 3D map and the fitted model (evaluated at 0.5). (D) Zoomed-in views of the final 3D density map from panel A, displayed at two contour levels. (E) Superimposition of the high-contour level map from panel D onto its fitted model.

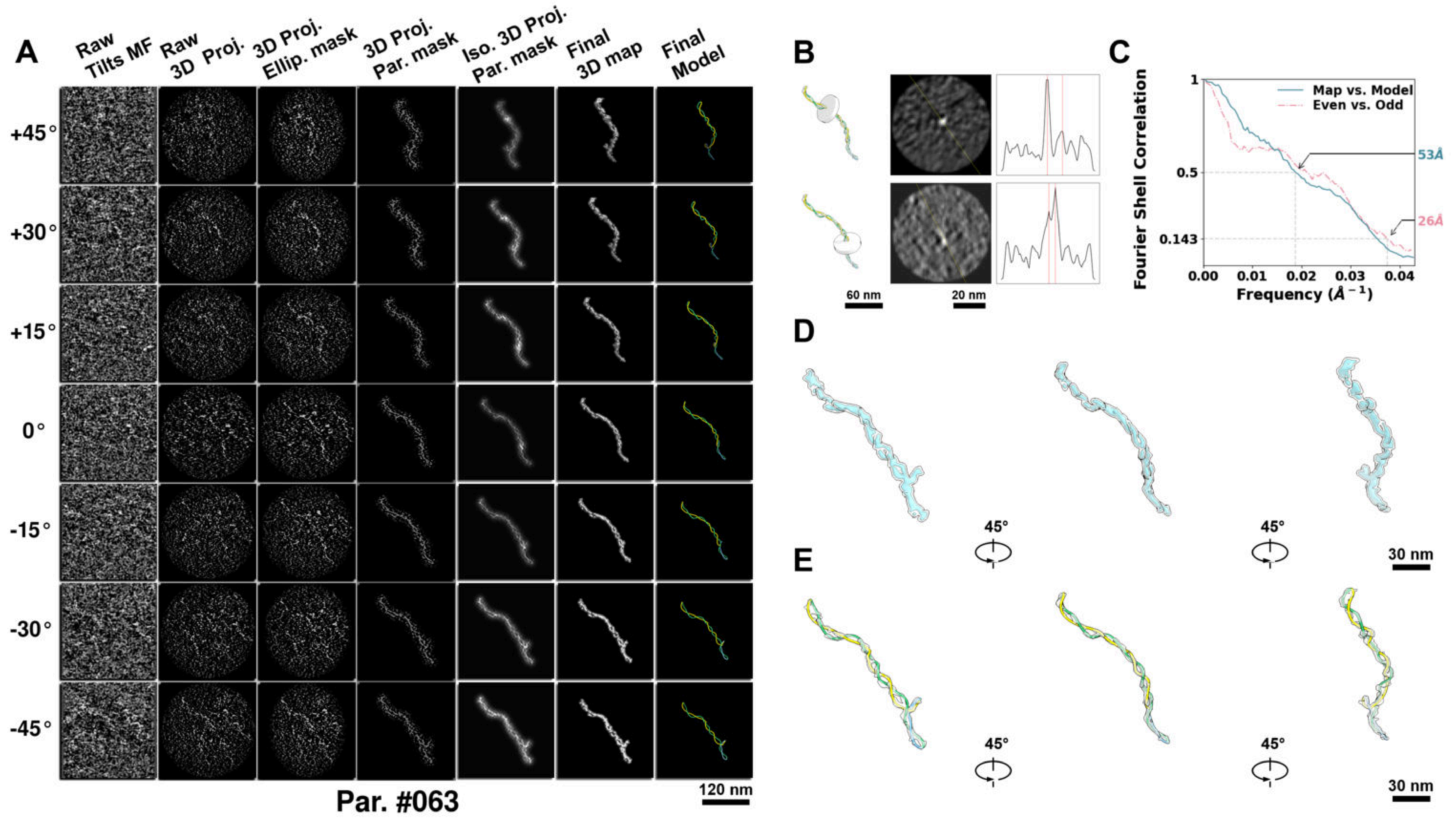

Supplementary Particle Figure 63. Cryo-ET 3D reconstruction of an individual P.HS particle.
(A) 3D reconstruction of the plasmid particle (index no. 63). The first column shows seven representative tilt images from +45° to -45° in step of 15°. The second, third, and fourth columns show 3D projections of the particle with spherical, ellipsoidal (thinner along the z-dimension), and particle-shaped masks, respectively. The fifth column displays the 3D projections of the enhanced and IsoNet missing-wedge-corrected particle. The sixth and seventh columns present the final 3D map and the flexibly fitted model, respectively. (B) Two cross-sectional views (12 nm thickness) of the plasmid density map along its plectoneme axis are shown in the left-middle panel. The intensity profile along the line crossing the two high-density DNA spots is displayed in the right panel. (C) Resolution assessment of the final 3D map using Fourier shell correlation (FSC). Two criteria are shown: FSC between two half-maps reconstructed from even and odd frames (evaluated at 0.143) and FSC between the final 3D map and the fitted model (evaluated at 0.5). (D) Zoomed-in views of the final 3D density map from panel A, displayed at two contour levels. (E) Superimposition of the high-contour level map from panel D onto its fitted model.

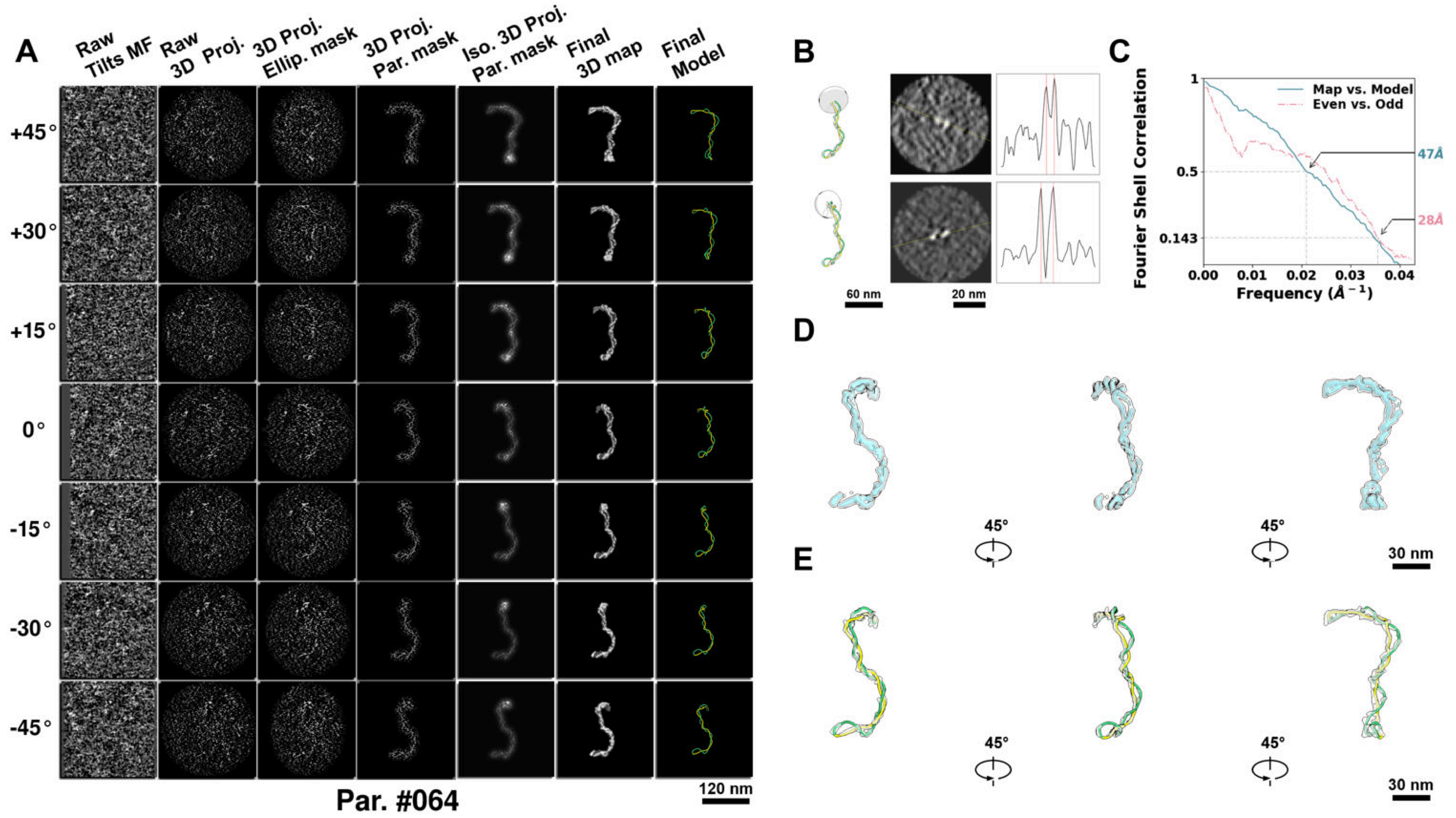

Supplementary Particle Figure 64. Cryo-ET 3D reconstruction of an individual P.HS particle.
(A) 3D reconstruction of the plasmid particle (index no. 64). The first column shows seven representative tilt images from +45° to -45° in step of 15°. The second, third, and fourth columns show 3D projections of the particle with spherical, ellipsoidal (thinner along the z-dimension), and particle-shaped masks, respectively. The fifth column displays the 3D projections of the enhanced and IsoNet missing-wedge-corrected particle. The sixth and seventh columns present the final 3D map and the flexibly fitted model, respectively. (B) Two cross-sectional views (12 nm thickness) of the plasmid density map along its plectoneme axis are shown in the left-middle panel. The intensity profile along the line crossing the two high-density DNA spots is displayed in the right panel. (C) Resolution assessment of the final 3D map using Fourier shell correlation (FSC). Two criteria are shown: FSC between two half-maps reconstructed from even and odd frames (evaluated at 0.143) and FSC between the final 3D map and the fitted model (evaluated at 0.5). (D) Zoomed-in views of the final 3D density map from panel A, displayed at two contour levels. (E) Superimposition of the high-contour level map from panel D onto its fitted model.

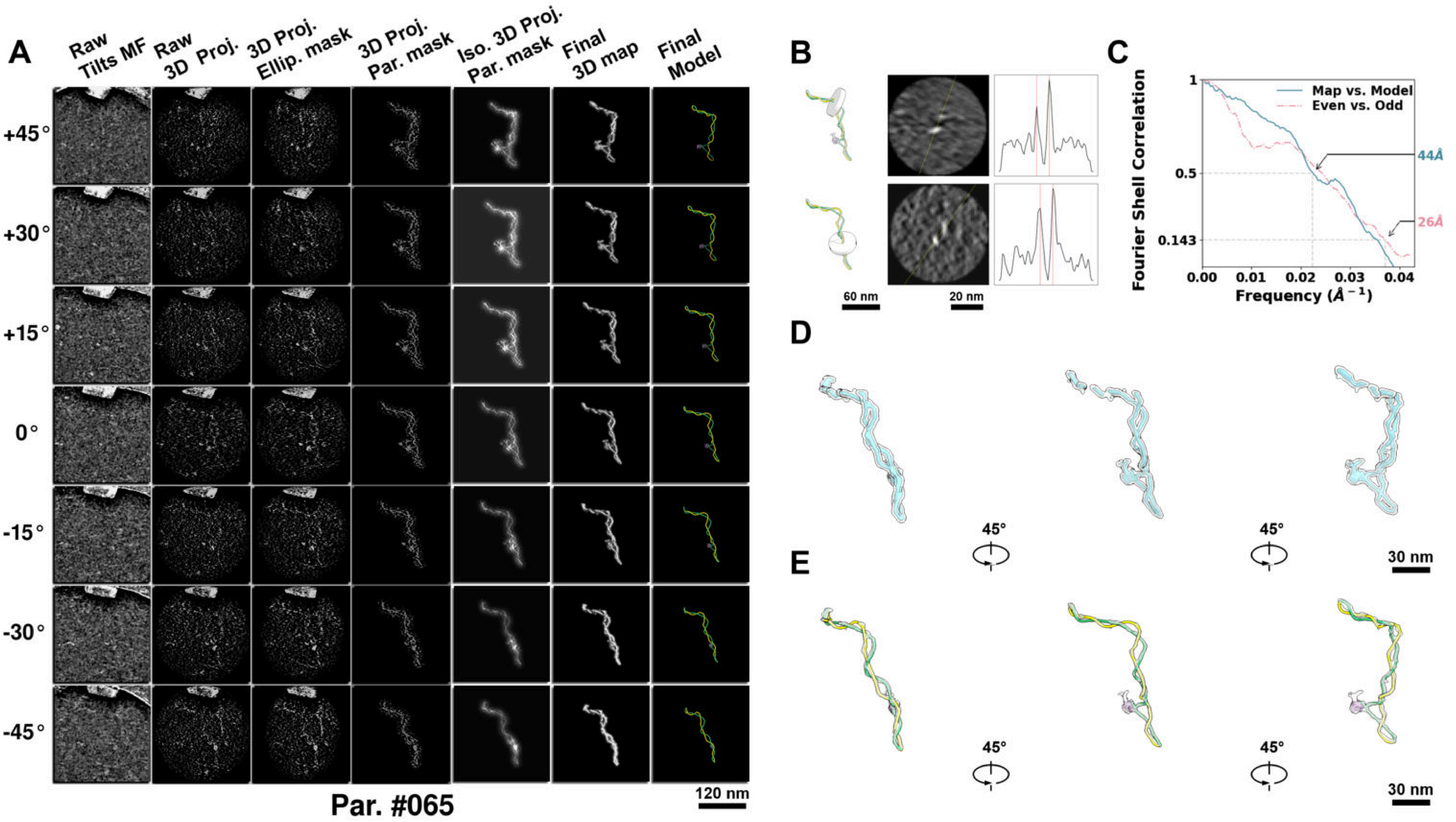

Supplementary Particle Figure 65. Cryo-ET 3D reconstruction of an individual sTEC particle.
(A) 3D reconstruction of the plasmid particle (index no. 65). The first column shows seven representative tilt images from +45° to -45° in step of 15°. The second, third, and fourth columns show 3D projections of the particle with spherical, ellipsoidal (thinner along the z-dimension), and particle-shaped masks, respectively. The fifth column displays the 3D projections of the enhanced and IsoNet missing-wedge-corrected particle. The sixth and seventh columns present the final 3D map and the flexibly fitted model, respectively. (B) Two cross-sectional views (12 nm thickness) of the plasmid density map along its plectoneme axis are shown in the left-middle panel. The intensity profile along the line crossing the two high-density DNA spots is displayed in the right panel. (C) Resolution assessment of the final 3D map using Fourier shell correlation (FSC). Two criteria are shown: FSC between two half-maps reconstructed from even and odd frames (evaluated at 0.143) and FSC between the final 3D map and the fitted model (evaluated at 0.5). (D) Zoomed-in views of the final 3D density map from panel A, displayed at two contour levels. (E) Superimposition of the high-contour level map from panel D onto its fitted model.

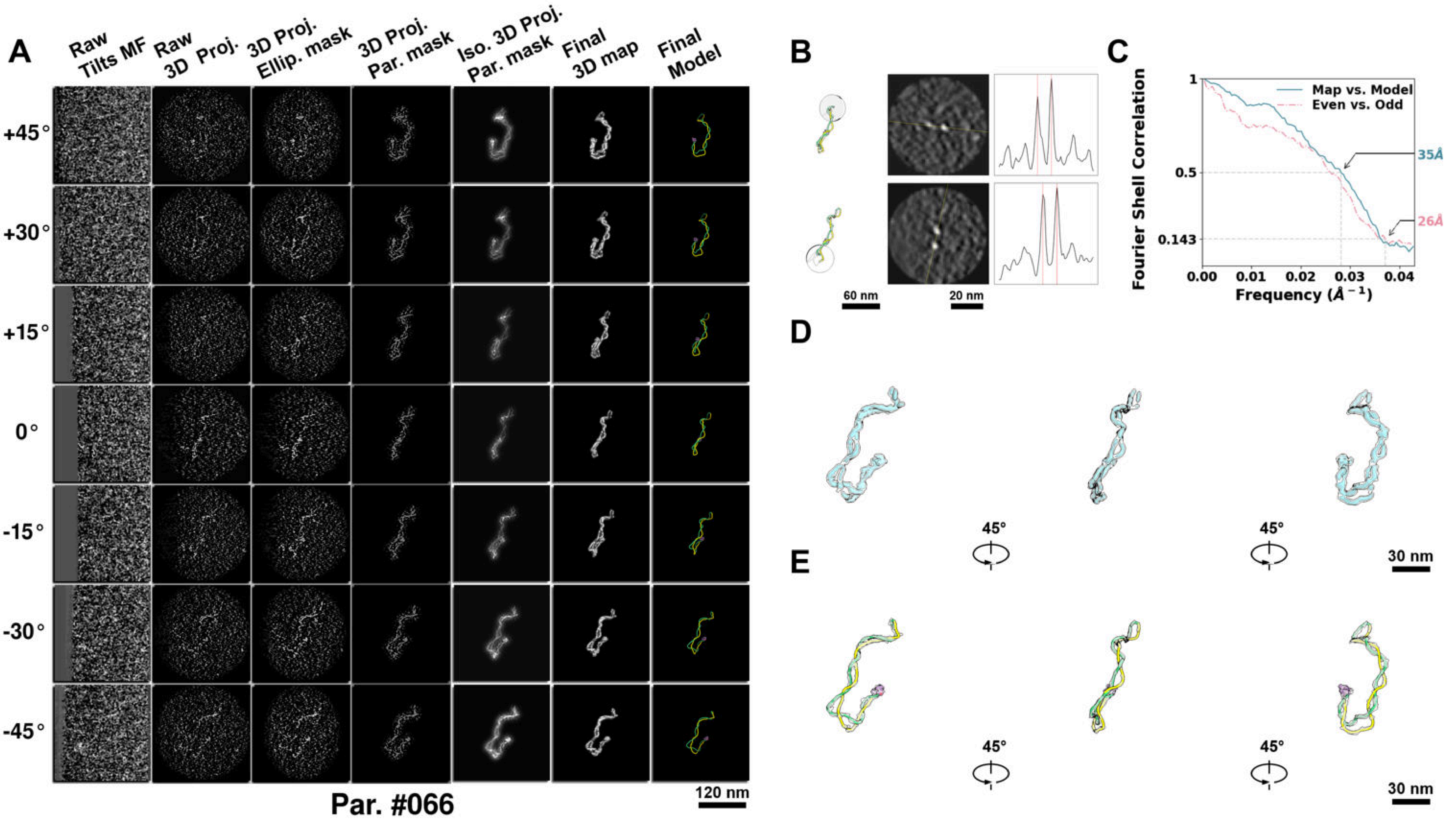

Supplementary Particle Figure 66. Cryo-ET 3D reconstruction of an individual sTEC particle.
(A) 3D reconstruction of the plasmid particle (index no. 66). The first column shows seven representative tilt images from +45° to -45° in step of 15°. The second, third, and fourth columns show 3D projections of the particle with spherical, ellipsoidal (thinner along the z-dimension), and particle-shaped masks, respectively. The fifth column displays the 3D projections of the enhanced and IsoNet missing-wedge-corrected particle. The sixth and seventh columns present the final 3D map and the flexibly fitted model, respectively. (B) Two cross-sectional views (12 nm thickness) of the plasmid density map along its plectoneme axis are shown in the left-middle panel. The intensity profile along the line crossing the two high-density DNA spots is displayed in the right panel. (C) Resolution assessment of the final 3D map using Fourier shell correlation (FSC). Two criteria are shown: FSC between two half-maps reconstructed from even and odd frames (evaluated at 0.143) and FSC between the final 3D map and the fitted model (evaluated at 0.5). (D) Zoomed-in views of the final 3D density map from panel A, displayed at two contour levels. (E) Superimposition of the high-contour level map from panel D onto its fitted model.

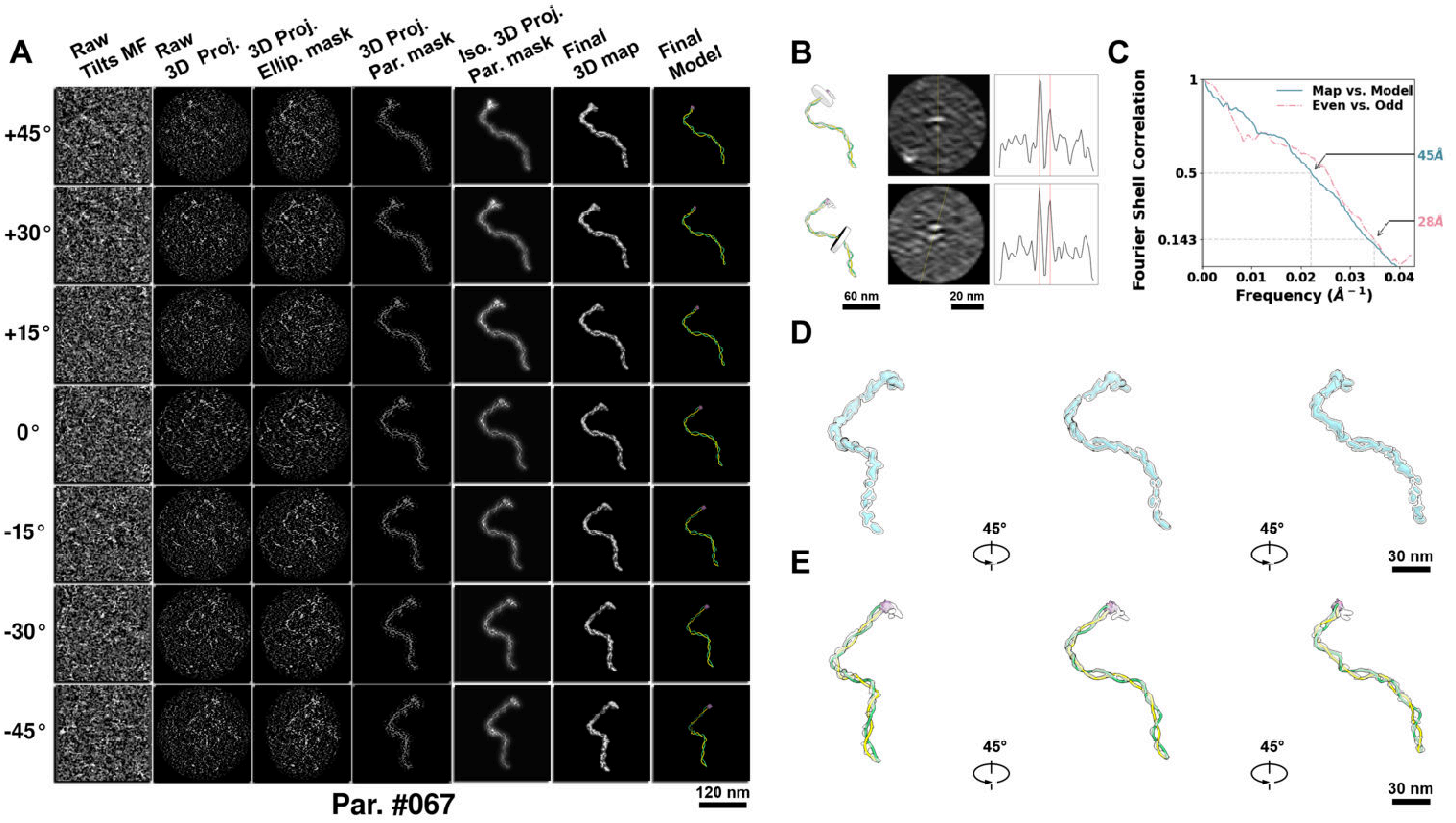

Supplementary Particle Figure 67. Cryo-ET 3D reconstruction of an individual sTEC particle.
(A) 3D reconstruction of the plasmid particle (index no. 67). The first column shows seven representative tilt images from +45° to -45° in step of 15°. The second, third, and fourth columns show 3D projections of the particle with spherical, ellipsoidal (thinner along the z-dimension), and particle-shaped masks, respectively. The fifth column displays the 3D projections of the enhanced and IsoNet missing-wedge-corrected particle. The sixth and seventh columns present the final 3D map and the flexibly fitted model, respectively. (B) Two cross-sectional views (12 nm thickness) of the plasmid density map along its plectoneme axis are shown in the left-middle panel. The intensity profile along the line crossing the two high-density DNA spots is displayed in the right panel. (C) Resolution assessment of the final 3D map using Fourier shell correlation (FSC). Two criteria are shown: FSC between two half-maps reconstructed from even and odd frames (evaluated at 0.143) and FSC between the final 3D map and the fitted model (evaluated at 0.5). (D) Zoomed-in views of the final 3D density map from panel A, displayed at two contour levels. (E) Superimposition of the high-contour level map from panel D onto its fitted model.

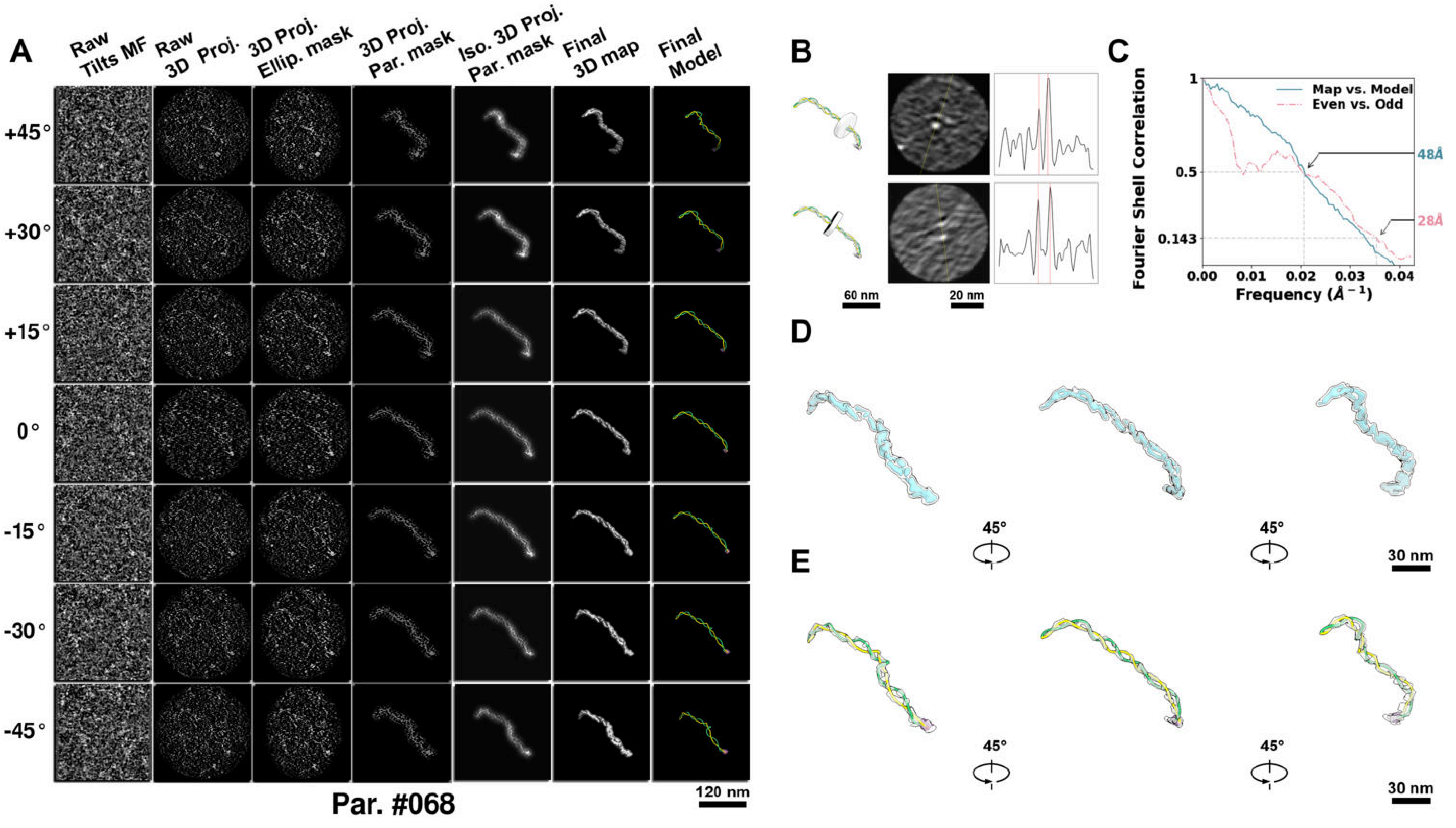

Supplementary Particle Figure 68. Cryo-ET 3D reconstruction of an individual sTEC particle.
(A) 3D reconstruction of the plasmid particle (index no. 68). The first column shows seven representative tilt images from +45° to -45° in step of 15°. The second, third, and fourth columns show 3D projections of the particle with spherical, ellipsoidal (thinner along the z-dimension), and particle-shaped masks, respectively. The fifth column displays the 3D projections of the enhanced and IsoNet missing-wedge-corrected particle. The sixth and seventh columns present the final 3D map and the flexibly fitted model, respectively. (B) Two cross-sectional views (12 nm thickness) of the plasmid density map along its plectoneme axis are shown in the left-middle panel. The intensity profile along the line crossing the two high-density DNA spots is displayed in the right panel. (C) Resolution assessment of the final 3D map using Fourier shell correlation (FSC). Two criteria are shown: FSC between two half-maps reconstructed from even and odd frames (evaluated at 0.143) and FSC between the final 3D map and the fitted model (evaluated at 0.5). (D) Zoomed-in views of the final 3D density map from panel A, displayed at two contour levels. (E) Superimposition of the high-contour level map from panel D onto its fitted model.

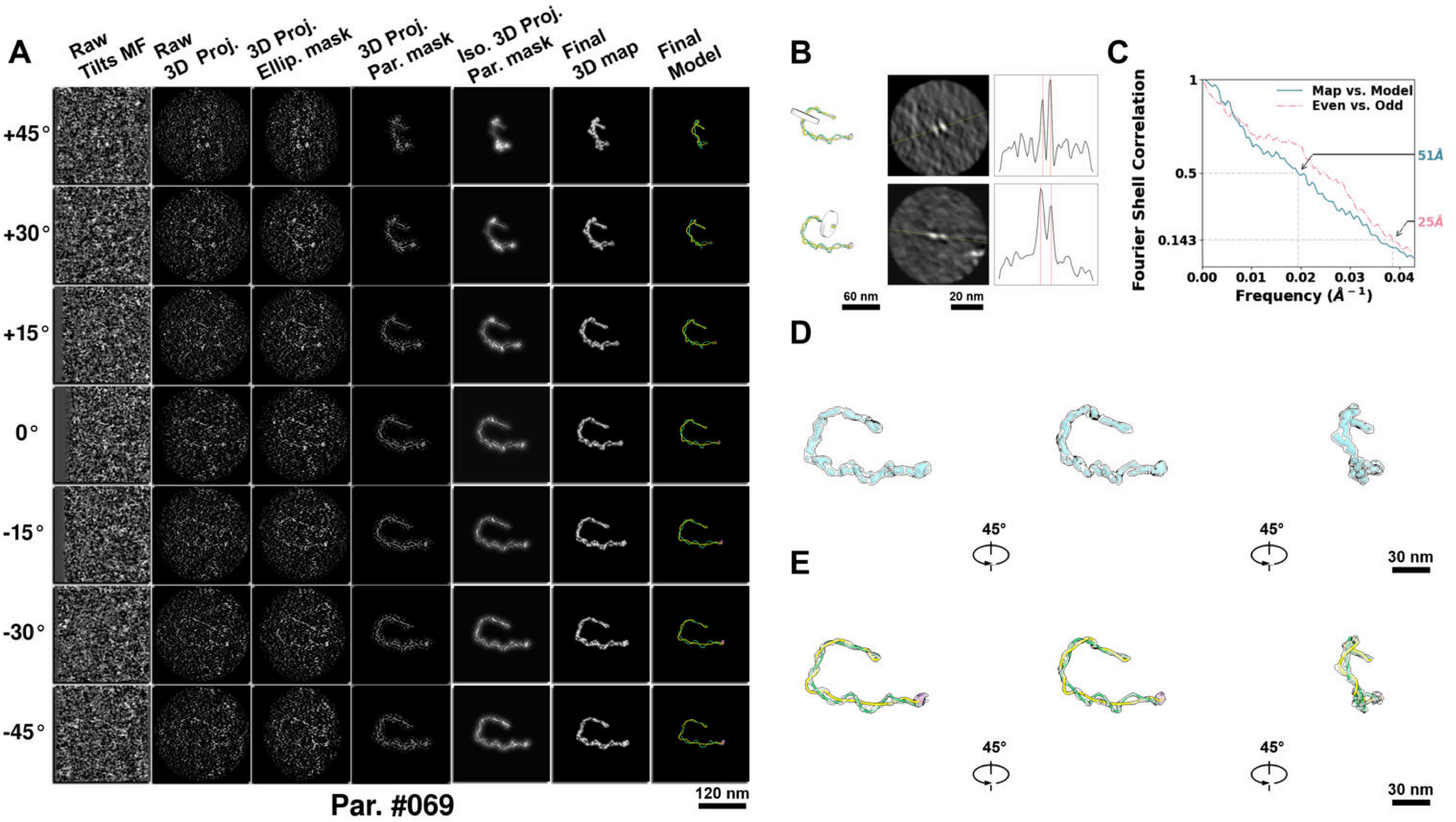

Supplementary Particle Figure 69. Cryo-ET 3D reconstruction of an individual sTEC particle.
(A) 3D reconstruction of the plasmid particle (index no. 69). The first column shows seven representative tilt images from +45° to -45° in step of 15°. The second, third, and fourth columns show 3D projections of the particle with spherical, ellipsoidal (thinner along the z-dimension), and particle-shaped masks, respectively. The fifth column displays the 3D projections of the enhanced and IsoNet missing-wedge-corrected particle. The sixth and seventh columns present the final 3D map and the flexibly fitted model, respectively. (B) Two cross-sectional views (12 nm thickness) of the plasmid density map along its plectoneme axis are shown in the left-middle panel. The intensity profile along the line crossing the two high-density DNA spots is displayed in the right panel. (C) Resolution assessment of the final 3D map using Fourier shell correlation (FSC). Two criteria are shown: FSC between two half-maps reconstructed from even and odd frames (evaluated at 0.143) and FSC between the final 3D map and the fitted model (evaluated at 0.5). (D) Zoomed-in views of the final 3D density map from panel A, displayed at two contour levels. (E) Superimposition of the high-contour level map from panel D onto its fitted model.

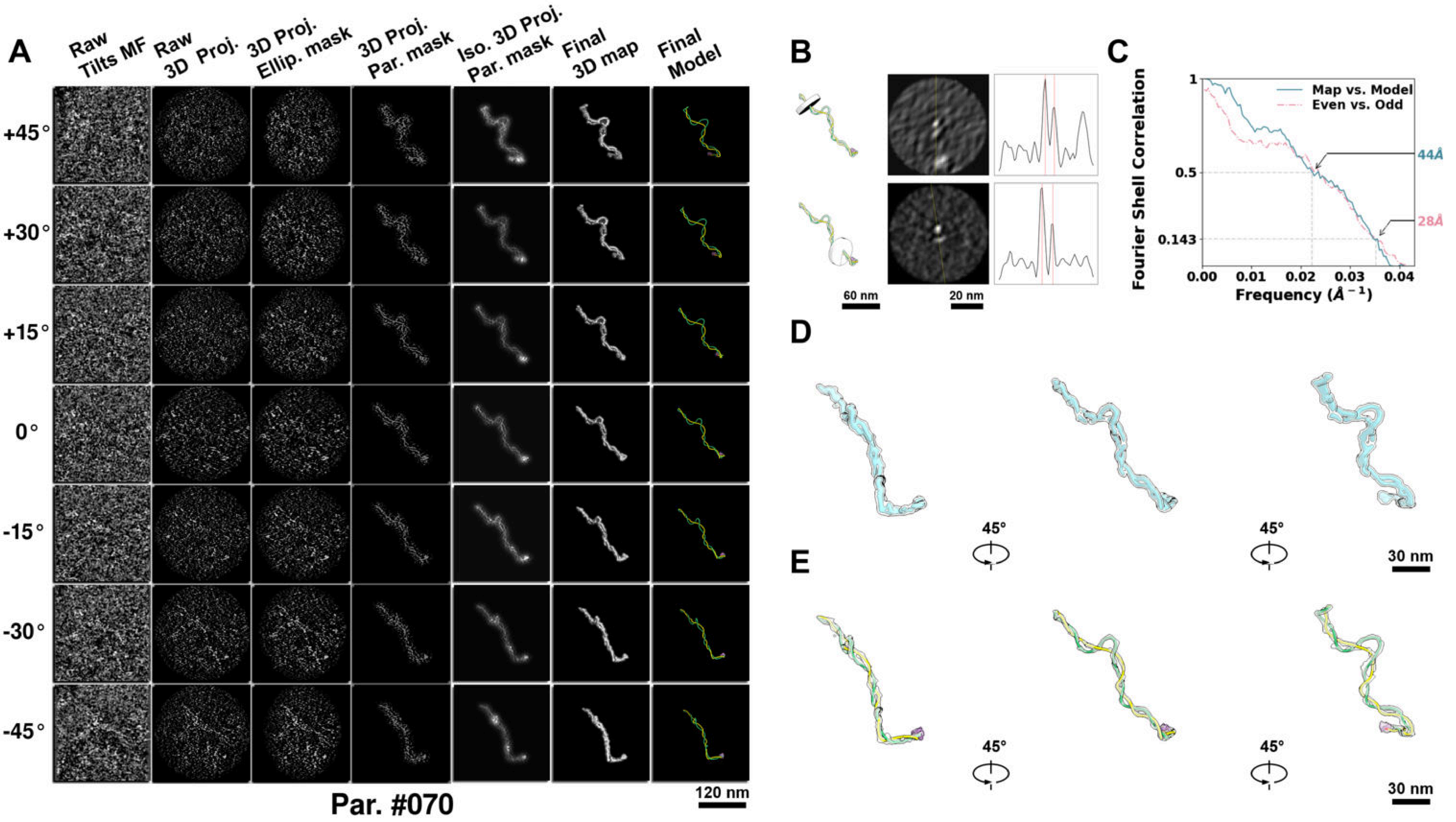

Supplementary Particle Figure 70. Cryo-ET 3D reconstruction of an individual sTEC particle.
(A) 3D reconstruction of the plasmid particle (index no. 70). The first column shows seven representative tilt images from +45° to -45° in step of 15°. The second, third, and fourth columns show 3D projections of the particle with spherical, ellipsoidal (thinner along the z-dimension), and particle-shaped masks, respectively. The fifth column displays the 3D projections of the enhanced and IsoNet missing-wedge-corrected particle. The sixth and seventh columns present the final 3D map and the flexibly fitted model, respectively. (B) Two cross-sectional views (12 nm thickness) of the plasmid density map along its plectoneme axis are shown in the left-middle panel. The intensity profile along the line crossing the two high-density DNA spots is displayed in the right panel. (C) Resolution assessment of the final 3D map using Fourier shell correlation (FSC). Two criteria are shown: FSC between two half-maps reconstructed from even and odd frames (evaluated at 0.143) and FSC between the final 3D map and the fitted model (evaluated at 0.5). (D) Zoomed-in views of the final 3D density map from panel A, displayed at two contour levels. (E) Superimposition of the high-contour level map from panel D onto its fitted model.

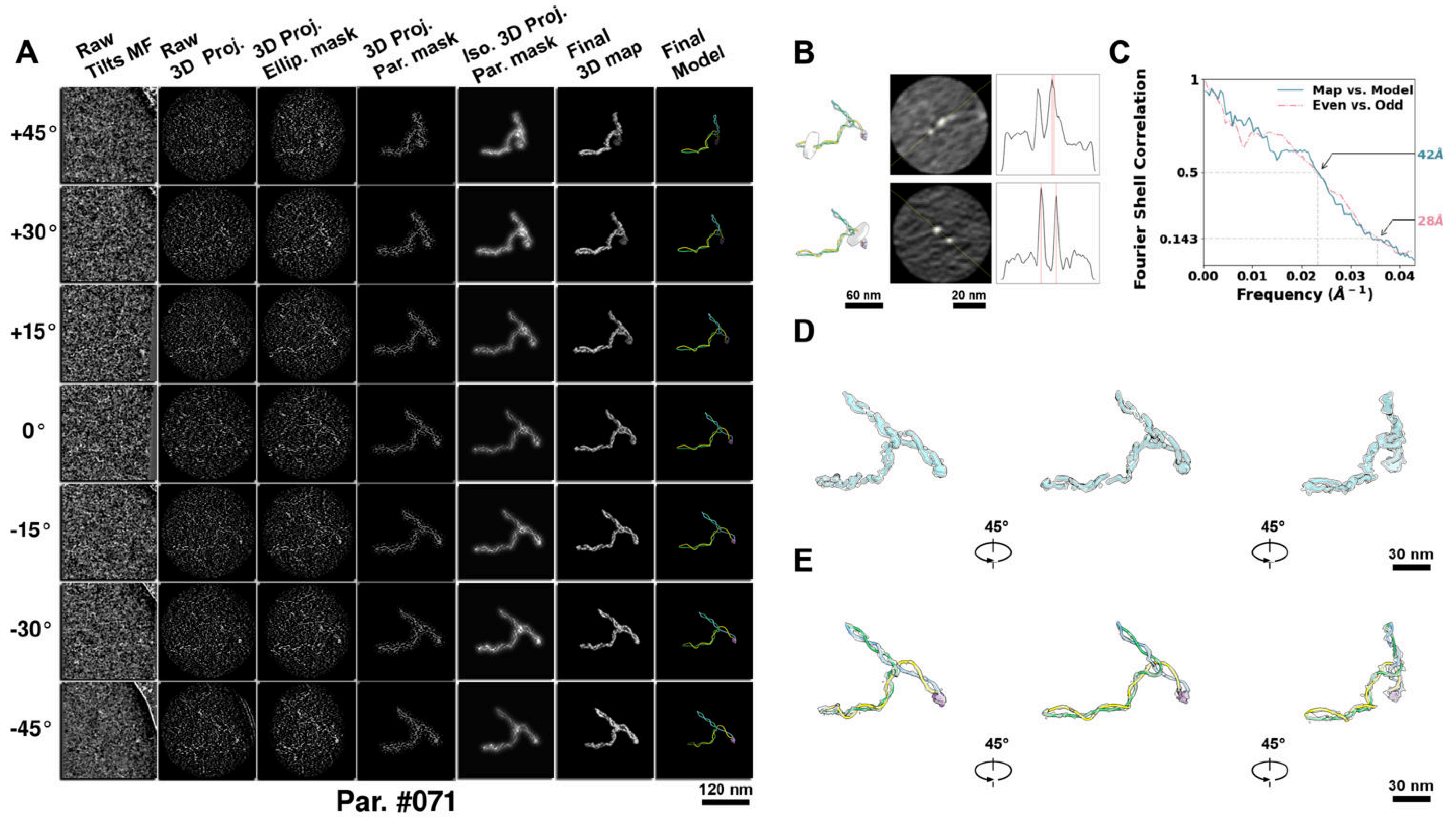

Supplementary Particle Figure 71. Cryo-ET 3D reconstruction of an individual sTEC particle.
(A) 3D reconstruction of the plasmid particle (index no. 71). The first column shows seven representative tilt images from +45° to -45° in step of 15°. The second, third, and fourth columns show 3D projections of the particle with spherical, ellipsoidal (thinner along the z-dimension), and particle-shaped masks, respectively. The fifth column displays the 3D projections of the enhanced and IsoNet missing-wedge-corrected particle. The sixth and seventh columns present the final 3D map and the flexibly fitted model, respectively. (B) Two cross-sectional views (12 nm thickness) of the plasmid density map along its plectoneme axis are shown in the left-middle panel. The intensity profile along the line crossing the two high-density DNA spots is displayed in the right panel. (C) Resolution assessment of the final 3D map using Fourier shell correlation (FSC). Two criteria are shown: FSC between two half-maps reconstructed from even and odd frames (evaluated at 0.143) and FSC between the final 3D map and the fitted model (evaluated at 0.5). (D) Zoomed-in views of the final 3D density map from panel A, displayed at two contour levels. (E) Superimposition of the high-contour level map from panel D onto its fitted model.

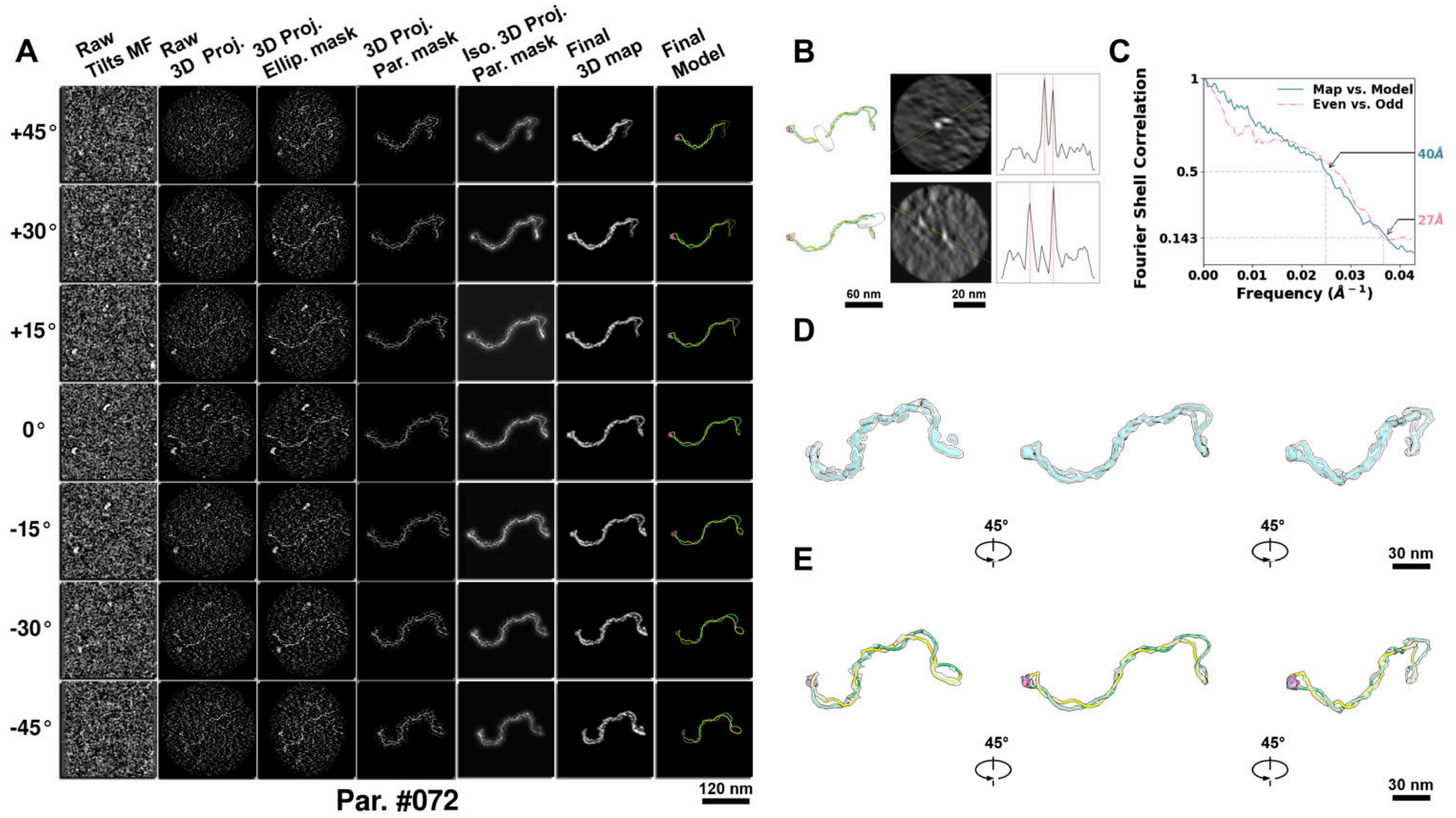

Supplementary Particle Figure 72. Cryo-ET 3D reconstruction of an individual sTEC particle.
(A) 3D reconstruction of the plasmid particle (index no. 72). The first column shows seven representative tilt images from +45° to -45° in step of 15°. The second, third, and fourth columns show 3D projections of the particle with spherical, ellipsoidal (thinner along the z-dimension), and particle-shaped masks, respectively. The fifth column displays the 3D projections of the enhanced and IsoNet missing-wedge-corrected particle. The sixth and seventh columns present the final 3D map and the flexibly fitted model, respectively. (B) Two cross-sectional views (12 nm thickness) of the plasmid density map along its plectoneme axis are shown in the left-middle panel. The intensity profile along the line crossing the two high-density DNA spots is displayed in the right panel. (C) Resolution assessment of the final 3D map using Fourier shell correlation (FSC). Two criteria are shown: FSC between two half-maps reconstructed from even and odd frames (evaluated at 0.143) and FSC between the final 3D map and the fitted model (evaluated at 0.5). (D) Zoomed-in views of the final 3D density map from panel A, displayed at two contour levels. (E) Superimposition of the high-contour level map from panel D onto its fitted model.

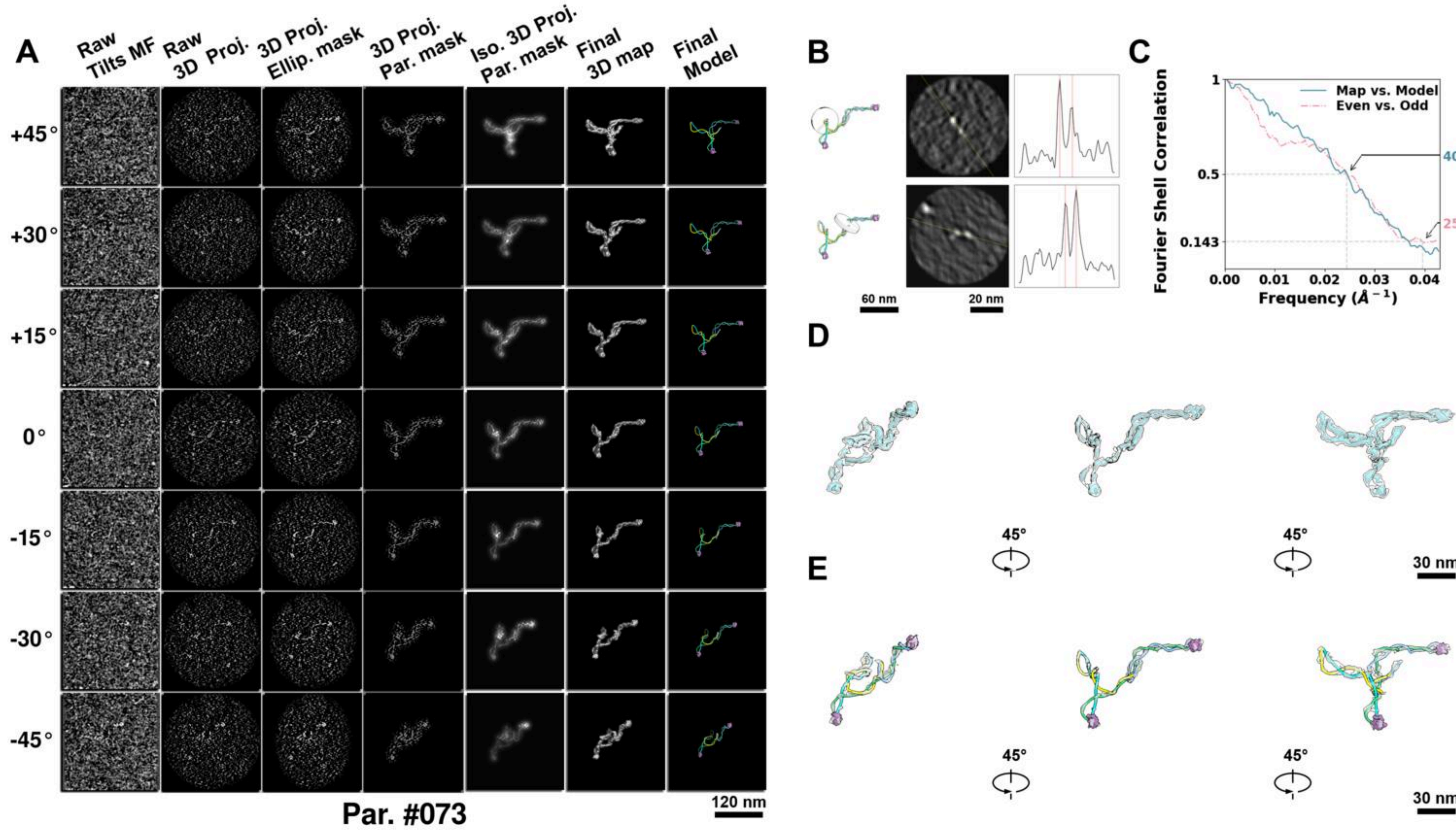

Supplementary Particle Figure 73. Cryo-ET 3D reconstruction of an individual sTEC particle.
(A) 3D reconstruction of the plasmid particle (index no. 73). The first column shows seven representative tilt images from +45° to -45° in step of 15°. The second, third, and fourth columns show 3D projections of the particle with spherical, ellipsoidal (thinner along the z-dimension), and particle-shaped masks, respectively. The fifth column displays the 3D projections of the enhanced and IsoNet missing-wedge-corrected particle. The sixth and seventh columns present the final 3D map and the flexibly fitted model, respectively. (B) Two cross-sectional views (12 nm thickness) of the plasmid density map along its plectoneme axis are shown in the left-middle panel. The intensity profile along the line crossing the two high-density DNA spots is displayed in the right panel. (C) Resolution assessment of the final 3D map using Fourier shell correlation (FSC). Two criteria are shown: FSC between two half-maps reconstructed from even and odd frames (evaluated at 0.143) and FSC between the final 3D map and the fitted model (evaluated at 0.5). (D) Zoomed-in views of the final 3D density map from panel A, displayed at two contour levels. (E) Superimposition of the high-contour level map from panel D onto its fitted model.

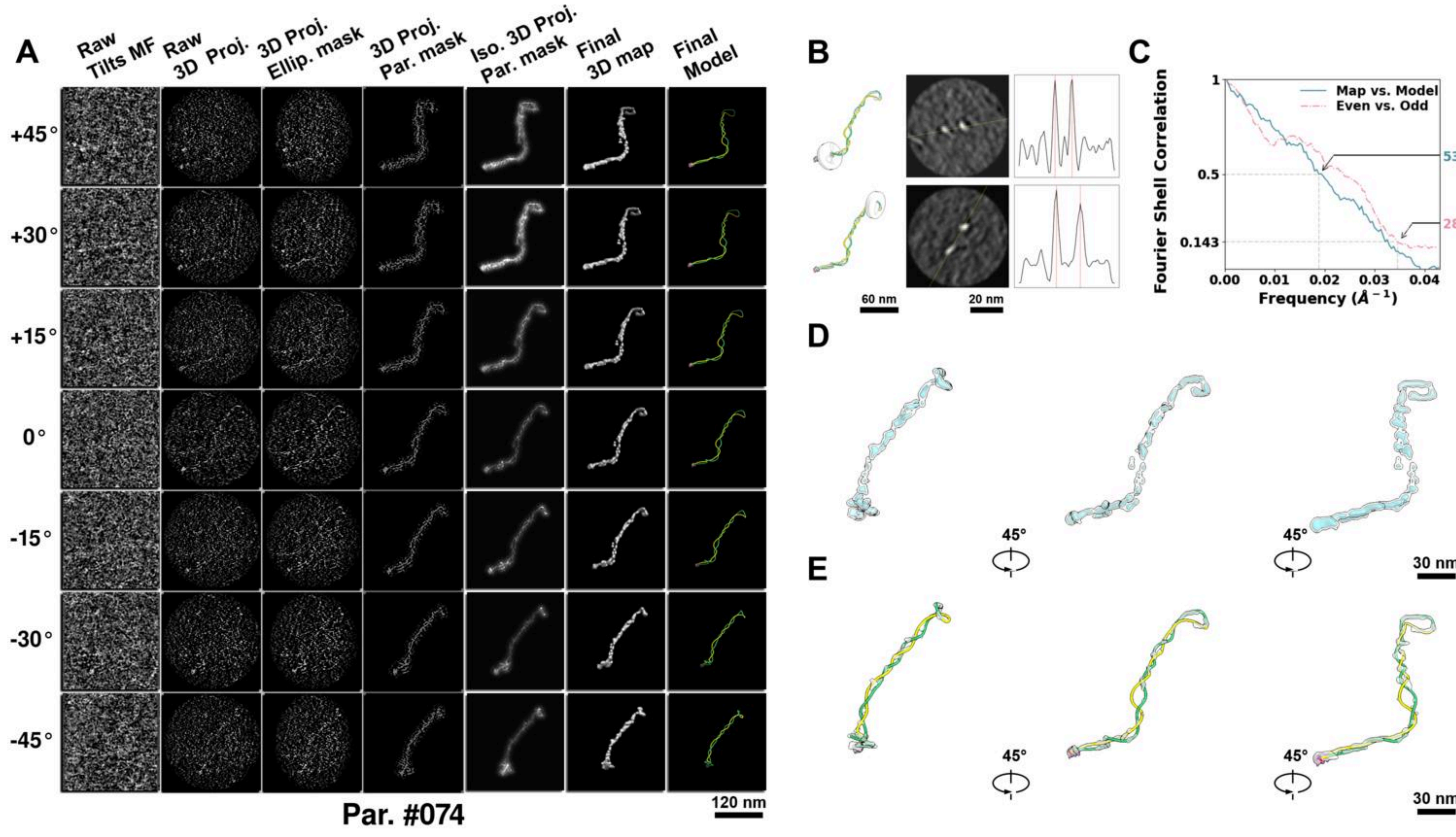

Supplementary Particle Figure 74. Cryo-ET 3D reconstruction of an individual sTEC particle.
(A) 3D reconstruction of the plasmid particle (index no. 74). The first column shows seven representative tilt images from +45° to -45° in step of 15°. The second, third, and fourth columns show 3D projections of the particle with spherical, ellipsoidal (thinner along the z-dimension), and particle-shaped masks, respectively. The fifth column displays the 3D projections of the enhanced and IsoNet missing-wedge-corrected particle. The sixth and seventh columns present the final 3D map and the flexibly fitted model, respectively. (B) Two cross-sectional views (12 nm thickness) of the plasmid density map along its plectoneme axis are shown in the left-middle panel. The intensity profile along the line crossing the two high-density DNA spots is displayed in the right panel. (C) Resolution assessment of the final 3D map using Fourier shell correlation (FSC). Two criteria are shown: FSC between two half-maps reconstructed from even and odd frames (evaluated at 0.143) and FSC between the final 3D map and the fitted model (evaluated at 0.5). (D) Zoomed-in views of the final 3D density map from panel A, displayed at two contour levels. (E) Superimposition of the high-contour level map from panel D onto its fitted model.

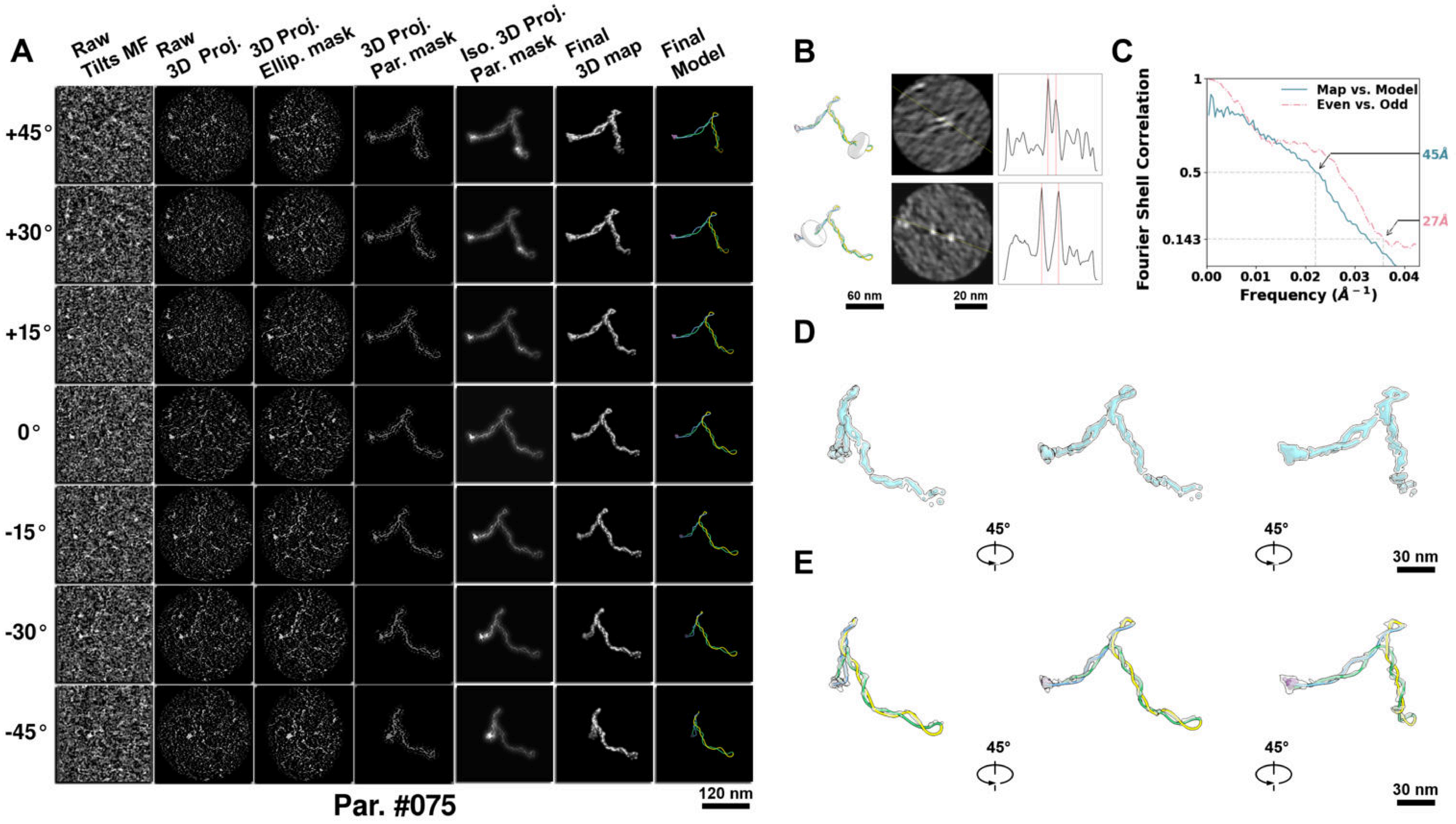

Supplementary Particle Figure 75. Cryo-ET 3D reconstruction of an individual sTEC particle.
(A) 3D reconstruction of the plasmid particle (index no. 75). The first column shows seven representative tilt images from +45° to -45° in step of 15°. The second, third, and fourth columns show 3D projections of the particle with spherical, ellipsoidal (thinner along the z-dimension), and particle-shaped masks, respectively. The fifth column displays the 3D projections of the enhanced and IsoNet missing-wedge-corrected particle. The sixth and seventh columns present the final 3D map and the flexibly fitted model, respectively. (B) Two cross-sectional views (12 nm thickness) of the plasmid density map along its plectoneme axis are shown in the left-middle panel. The intensity profile along the line crossing the two high-density DNA spots is displayed in the right panel. (C) Resolution assessment of the final 3D map using Fourier shell correlation (FSC). Two criteria are shown: FSC between two half-maps reconstructed from even and odd frames (evaluated at 0.143) and FSC between the final 3D map and the fitted model (evaluated at 0.5). (D) Zoomed-in views of the final 3D density map from panel A, displayed at two contour levels. (E) Superimposition of the high-contour level map from panel D onto its fitted model.

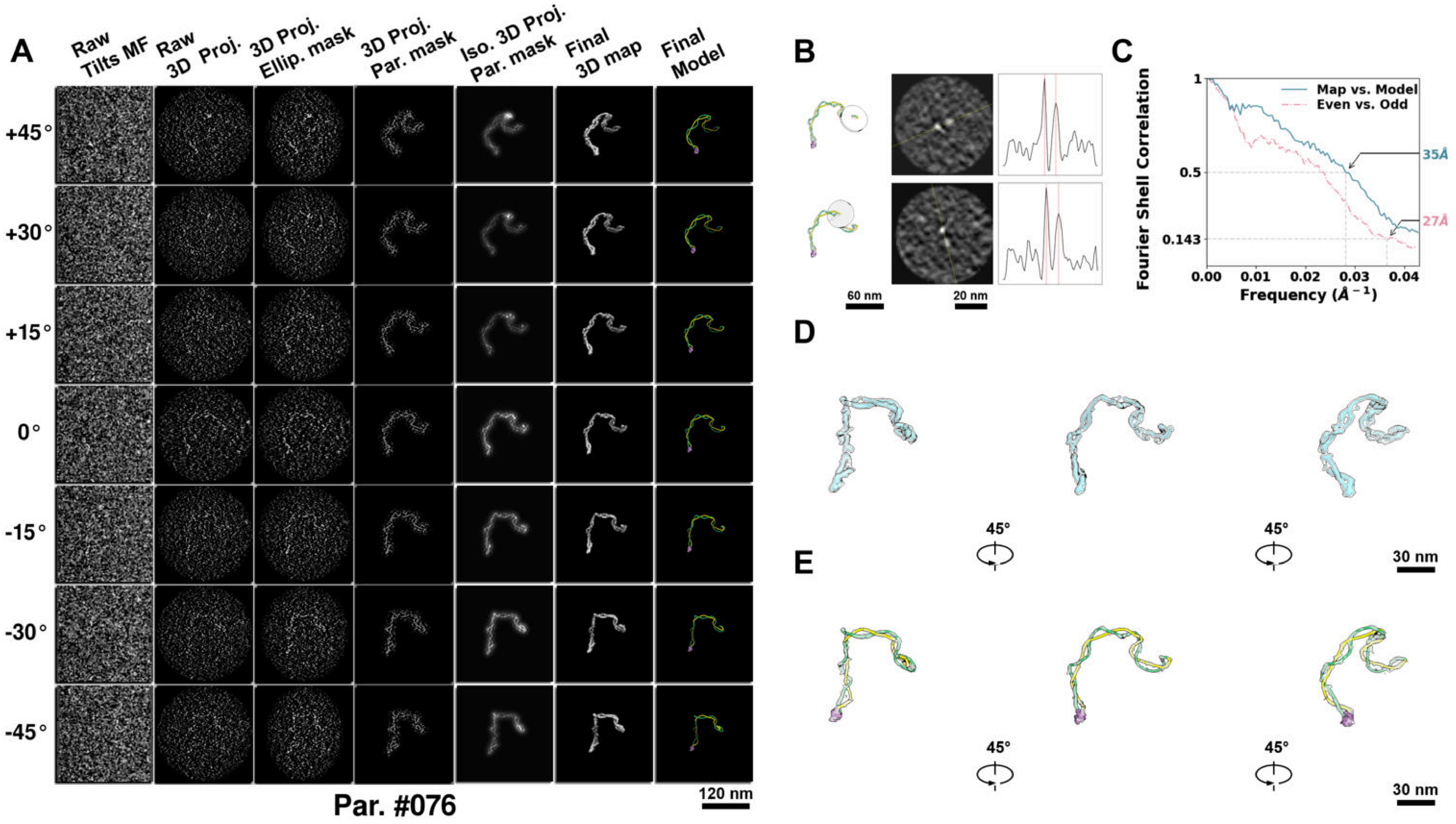

Supplementary Particle Figure 76. Cryo-ET 3D reconstruction of an individual sTEC particle.
(A) 3D reconstruction of the plasmid particle (index no. 76). The first column shows seven representative tilt images from +45° to -45° in step of 15°. The second, third, and fourth columns show 3D projections of the particle with spherical, ellipsoidal (thinner along the z-dimension), and particle-shaped masks, respectively. The fifth column displays the 3D projections of the enhanced and IsoNet missing-wedge-corrected particle. The sixth and seventh columns present the final 3D map and the flexibly fitted model, respectively. (B) Two cross-sectional views (12 nm thickness) of the plasmid density map along its plectoneme axis are shown in the left-middle panel. The intensity profile along the line crossing the two high-density DNA spots is displayed in the right panel. (C) Resolution assessment of the final 3D map using Fourier shell correlation (FSC). Two criteria are shown: FSC between two half-maps reconstructed from even and odd frames (evaluated at 0.143) and FSC between the final 3D map and the fitted model (evaluated at 0.5). (D) Zoomed-in views of the final 3D density map from panel A, displayed at two contour levels. (E) Superimposition of the high-contour level map from panel D onto its fitted model.

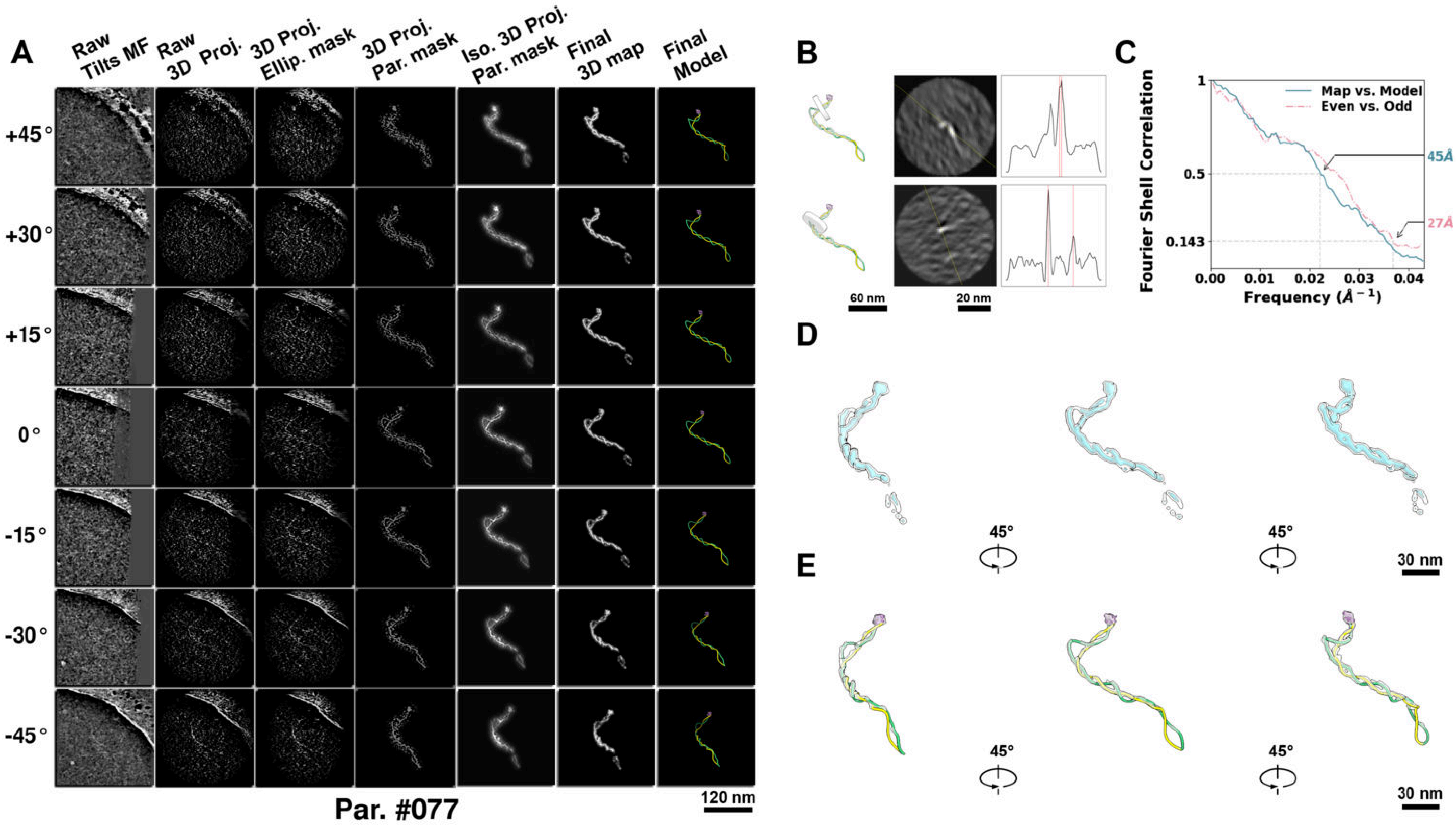

Supplementary Particle Figure 77. Cryo-ET 3D reconstruction of an individual sTEC particle.
(A) 3D reconstruction of the plasmid particle (index no. 77). The first column shows seven representative tilt images from +45° to -45° in step of 15°. The second, third, and fourth columns show 3D projections of the particle with spherical, ellipsoidal (thinner along the z-dimension), and particle-shaped masks, respectively. The fifth column displays the 3D projections of the enhanced and IsoNet missing-wedge-corrected particle. The sixth and seventh columns present the final 3D map and the flexibly fitted model, respectively. (B) Two cross-sectional views (12 nm thickness) of the plasmid density map along its plectoneme axis are shown in the left-middle panel. The intensity profile along the line crossing the two high-density DNA spots is displayed in the right panel. (C) Resolution assessment of the final 3D map using Fourier shell correlation (FSC). Two criteria are shown: FSC between two half-maps reconstructed from even and odd frames (evaluated at 0.143) and FSC between the final 3D map and the fitted model (evaluated at 0.5). (D) Zoomed-in views of the final 3D density map from panel A, displayed at two contour levels. (E) Superimposition of the high-contour level map from panel D onto its fitted model.

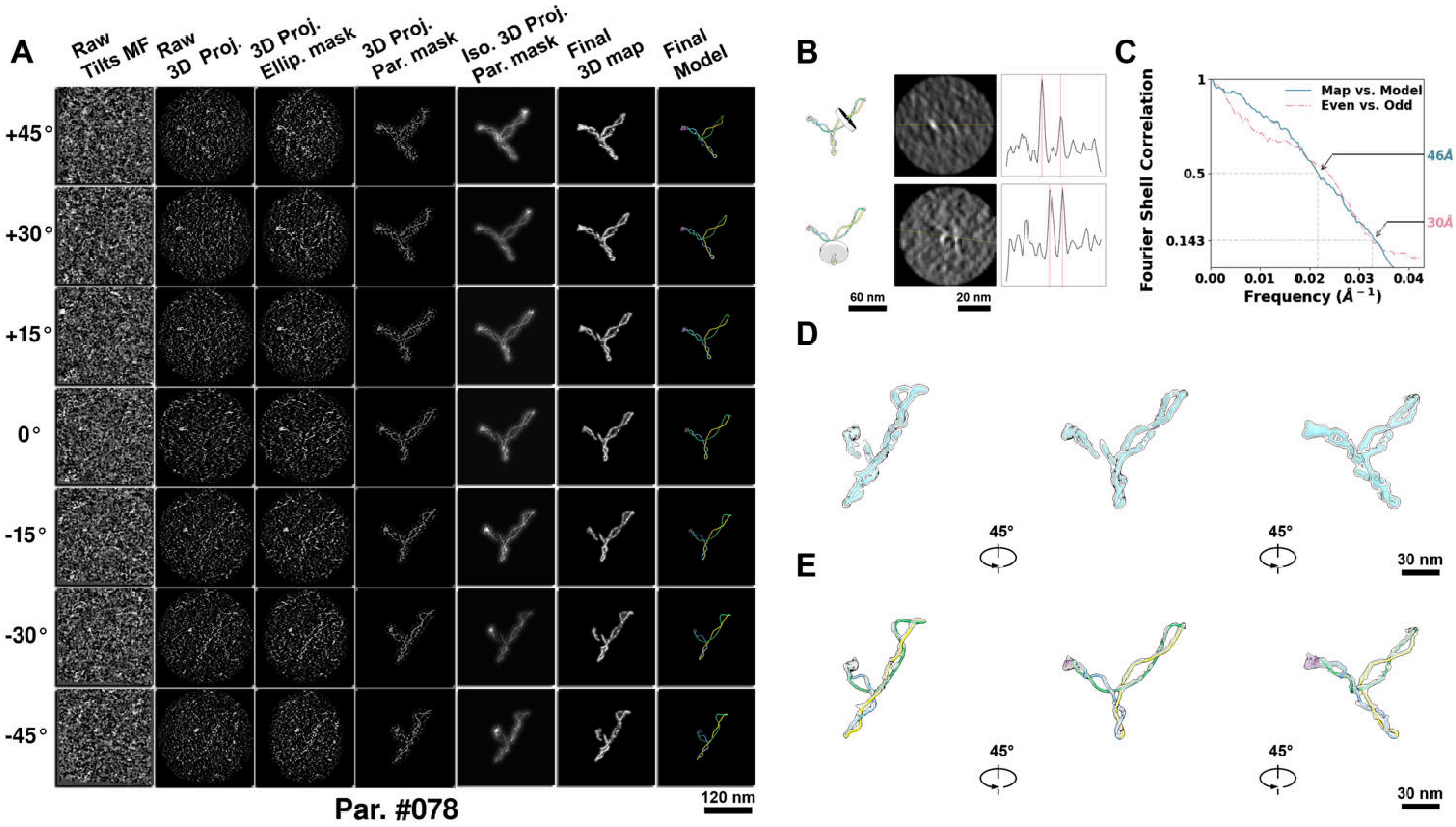

Supplementary Particle Figure 78. Cryo-ET 3D reconstruction of an individual sTEC particle.
(A) 3D reconstruction of the plasmid particle (index no. 78). The first column shows seven representative tilt images from +45° to -45° in step of 15°. The second, third, and fourth columns show 3D projections of the particle with spherical, ellipsoidal (thinner along the z-dimension), and particle-shaped masks, respectively. The fifth column displays the 3D projections of the enhanced and IsoNet missing-wedge-corrected particle. The sixth and seventh columns present the final 3D map and the flexibly fitted model, respectively. (B) Two cross-sectional views (12 nm thickness) of the plasmid density map along its plectoneme axis are shown in the left-middle panel. The intensity profile along the line crossing the two high-density DNA spots is displayed in the right panel. (C) Resolution assessment of the final 3D map using Fourier shell correlation (FSC). Two criteria are shown: FSC between two half-maps reconstructed from even and odd frames (evaluated at 0.143) and FSC between the final 3D map and the fitted model (evaluated at 0.5). (D) Zoomed-in views of the final 3D density map from panel A, displayed at two contour levels. (E) Superimposition of the high-contour level map from panel D onto its fitted model.

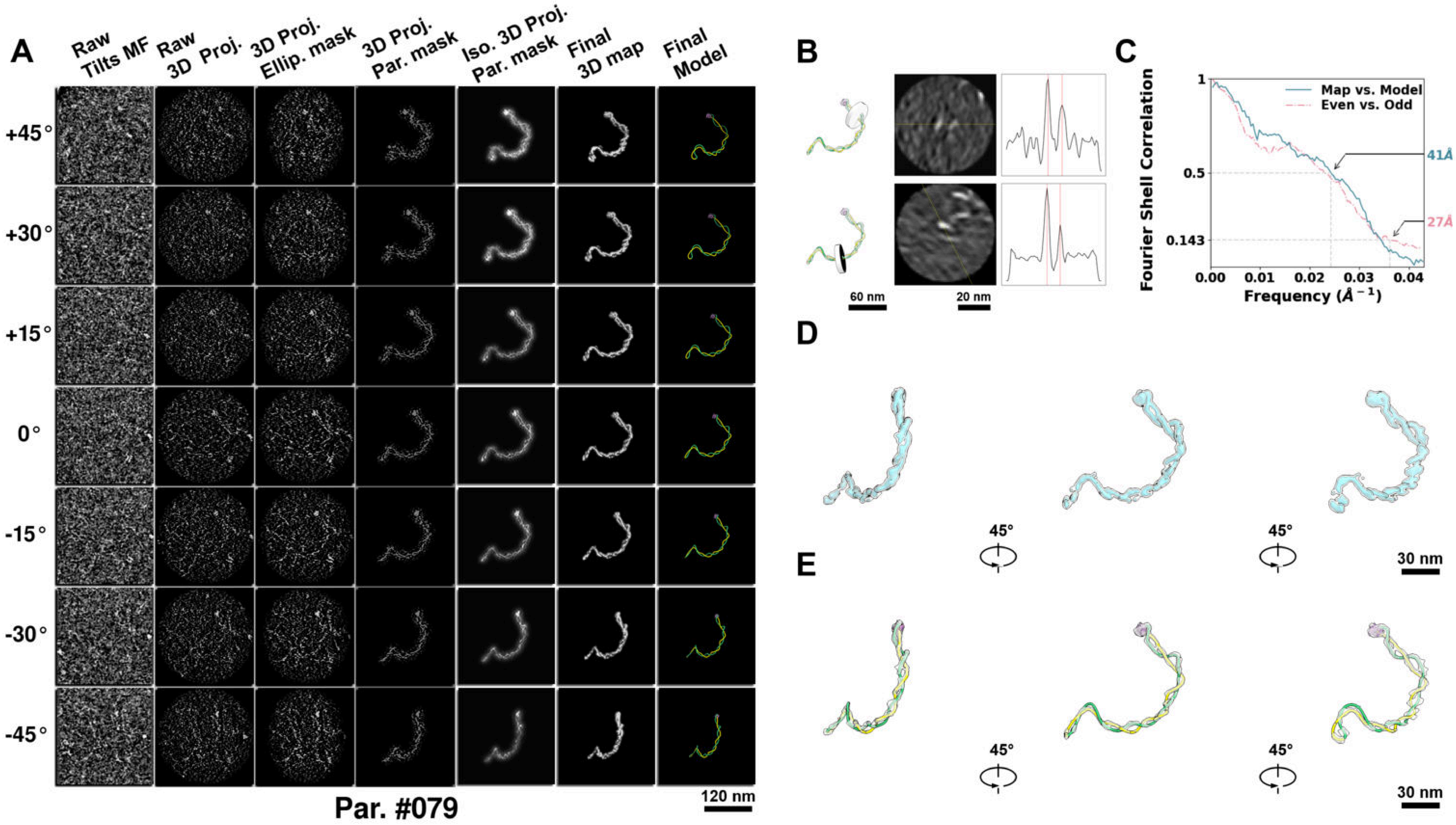

Supplementary Particle Figure 79. Cryo-ET 3D reconstruction of an individual sTEC particle.
(A) 3D reconstruction of the plasmid particle (index no. 79). The first column shows seven representative tilt images from +45° to -45° in step of 15°. The second, third, and fourth columns show 3D projections of the particle with spherical, ellipsoidal (thinner along the z-dimension), and particle-shaped masks, respectively. The fifth column displays the 3D projections of the enhanced and IsoNet missing-wedge-corrected particle. The sixth and seventh columns present the final 3D map and the flexibly fitted model, respectively. (B) Two cross-sectional views (12 nm thickness) of the plasmid density map along its plectoneme axis are shown in the left-middle panel. The intensity profile along the line crossing the two high-density DNA spots is displayed in the right panel. (C) Resolution assessment of the final 3D map using Fourier shell correlation (FSC). Two criteria are shown: FSC between two half-maps reconstructed from even and odd frames (evaluated at 0.143) and FSC between the final 3D map and the fitted model (evaluated at 0.5). (D) Zoomed-in views of the final 3D density map from panel A, displayed at two contour levels. (E) Superimposition of the high-contour level map from panel D onto its fitted model.

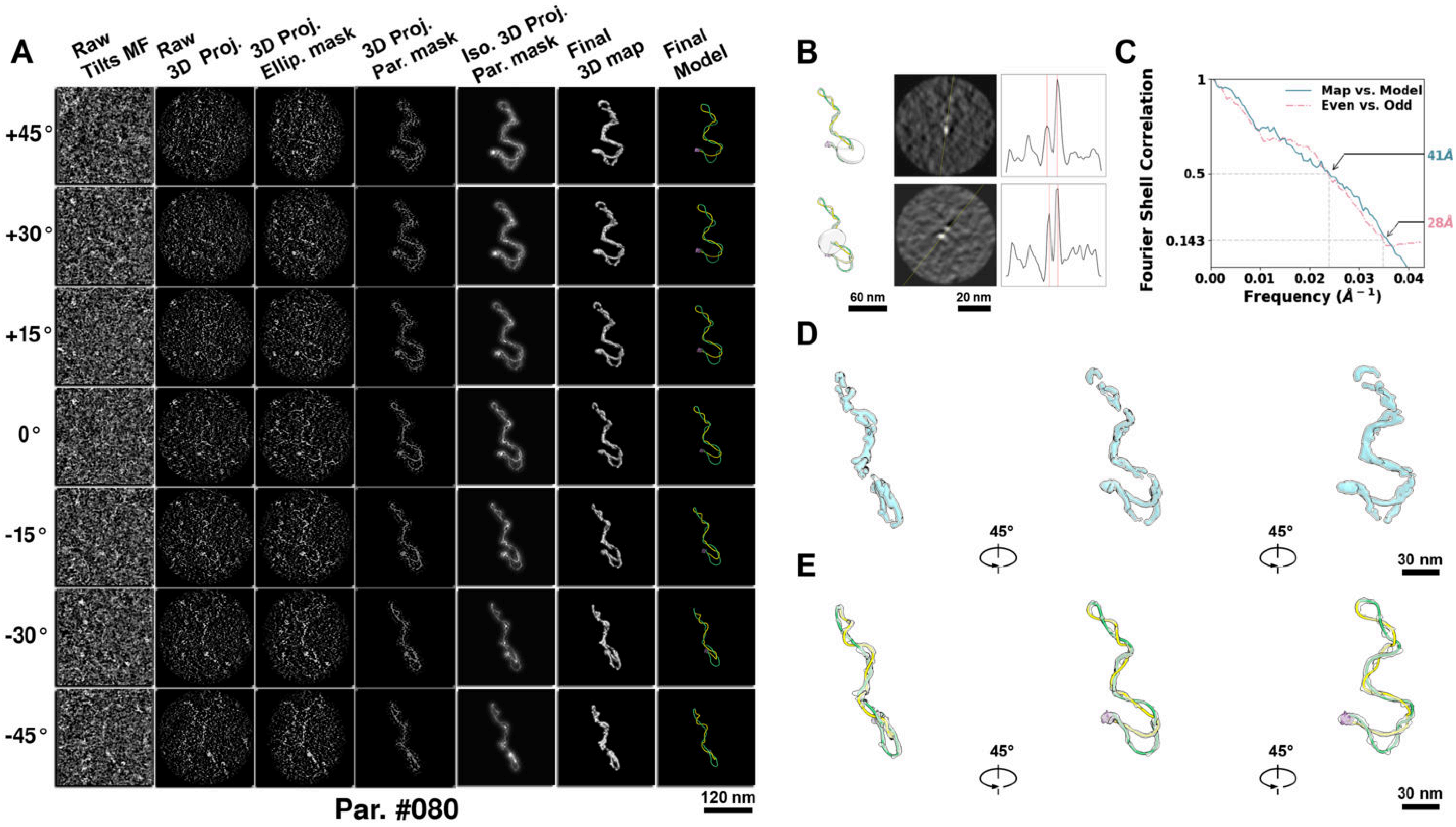

Supplementary Particle Figure 80. Cryo-ET 3D reconstruction of an individual sTEC particle.
(A) 3D reconstruction of the plasmid particle (index no. 80). The first column shows seven representative tilt images from +45° to -45° in step of 15°. The second, third, and fourth columns show 3D projections of the particle with spherical, ellipsoidal (thinner along the z-dimension), and particle-shaped masks, respectively. The fifth column displays the 3D projections of the enhanced and IsoNet missing-wedge-corrected particle. The sixth and seventh columns present the final 3D map and the flexibly fitted model, respectively. (B) Two cross-sectional views (12 nm thickness) of the plasmid density map along its plectoneme axis are shown in the left-middle panel. The intensity profile along the line crossing the two high-density DNA spots is displayed in the right panel. (C) Resolution assessment of the final 3D map using Fourier shell correlation (FSC). Two criteria are shown: FSC between two half-maps reconstructed from even and odd frames (evaluated at 0.143) and FSC between the final 3D map and the fitted model (evaluated at 0.5). (D) Zoomed-in views of the final 3D density map from panel A, displayed at two contour levels. (E) Superimposition of the high-contour level map from panel D onto its fitted model.

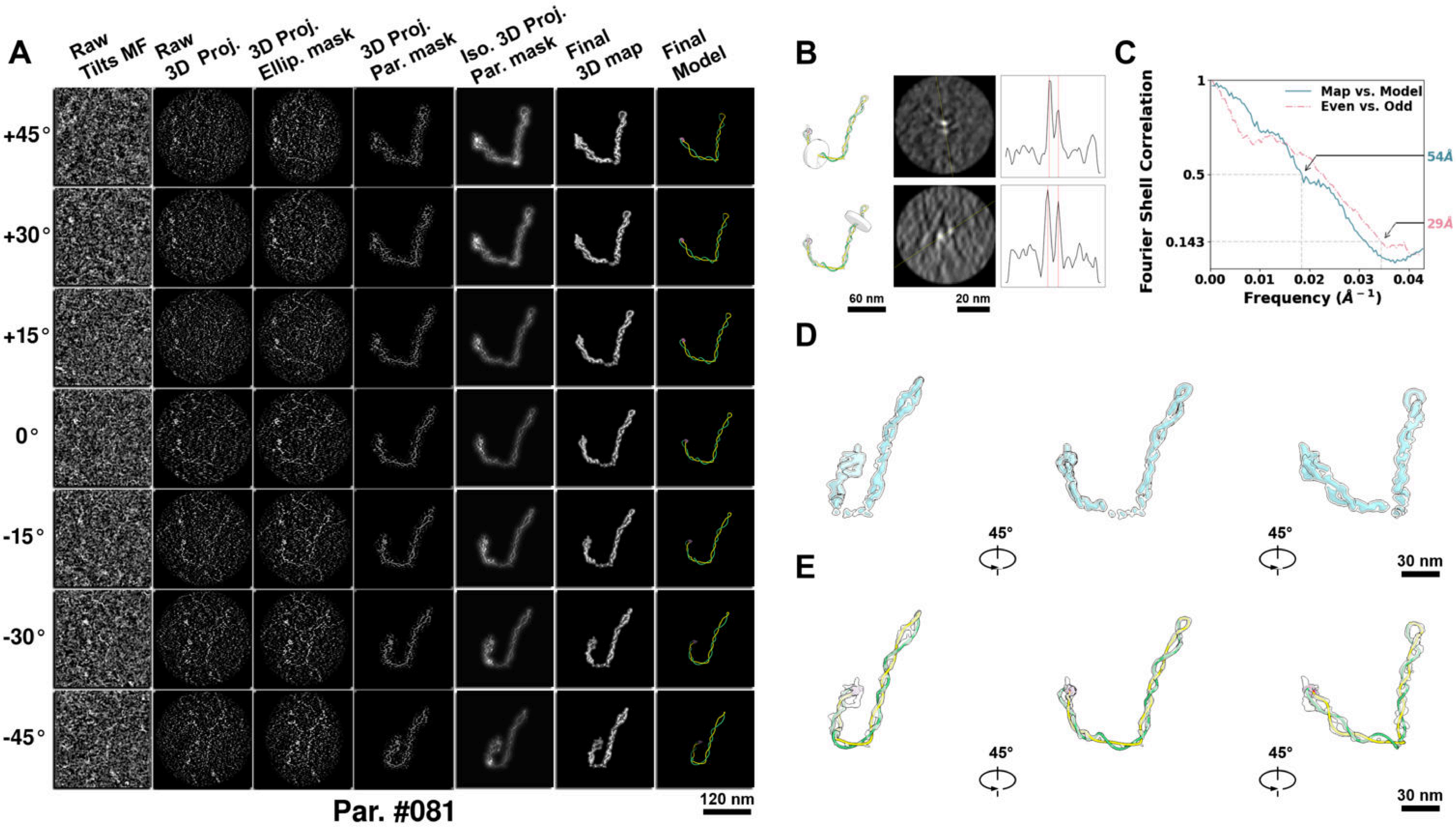

Supplementary Particle Figure 81. Cryo-ET 3D reconstruction of an individual sTEC particle.
(A) 3D reconstruction of the plasmid particle (index no. 81). The first column shows seven representative tilt images from +45° to -45° in step of 15°. The second, third, and fourth columns show 3D projections of the particle with spherical, ellipsoidal (thinner along the z-dimension), and particle-shaped masks, respectively. The fifth column displays the 3D projections of the enhanced and IsoNet missing-wedge-corrected particle. The sixth and seventh columns present the final 3D map and the flexibly fitted model, respectively. (B) Two cross-sectional views (12 nm thickness) of the plasmid density map along its plectoneme axis are shown in the left-middle panel. The intensity profile along the line crossing the two high-density DNA spots is displayed in the right panel. (C) Resolution assessment of the final 3D map using Fourier shell correlation (FSC). Two criteria are shown: FSC between two half-maps reconstructed from even and odd frames (evaluated at 0.143) and FSC between the final 3D map and the fitted model (evaluated at 0.5). (D) Zoomed-in views of the final 3D density map from panel A, displayed at two contour levels. (E) Superimposition of the high-contour level map from panel D onto its fitted model.

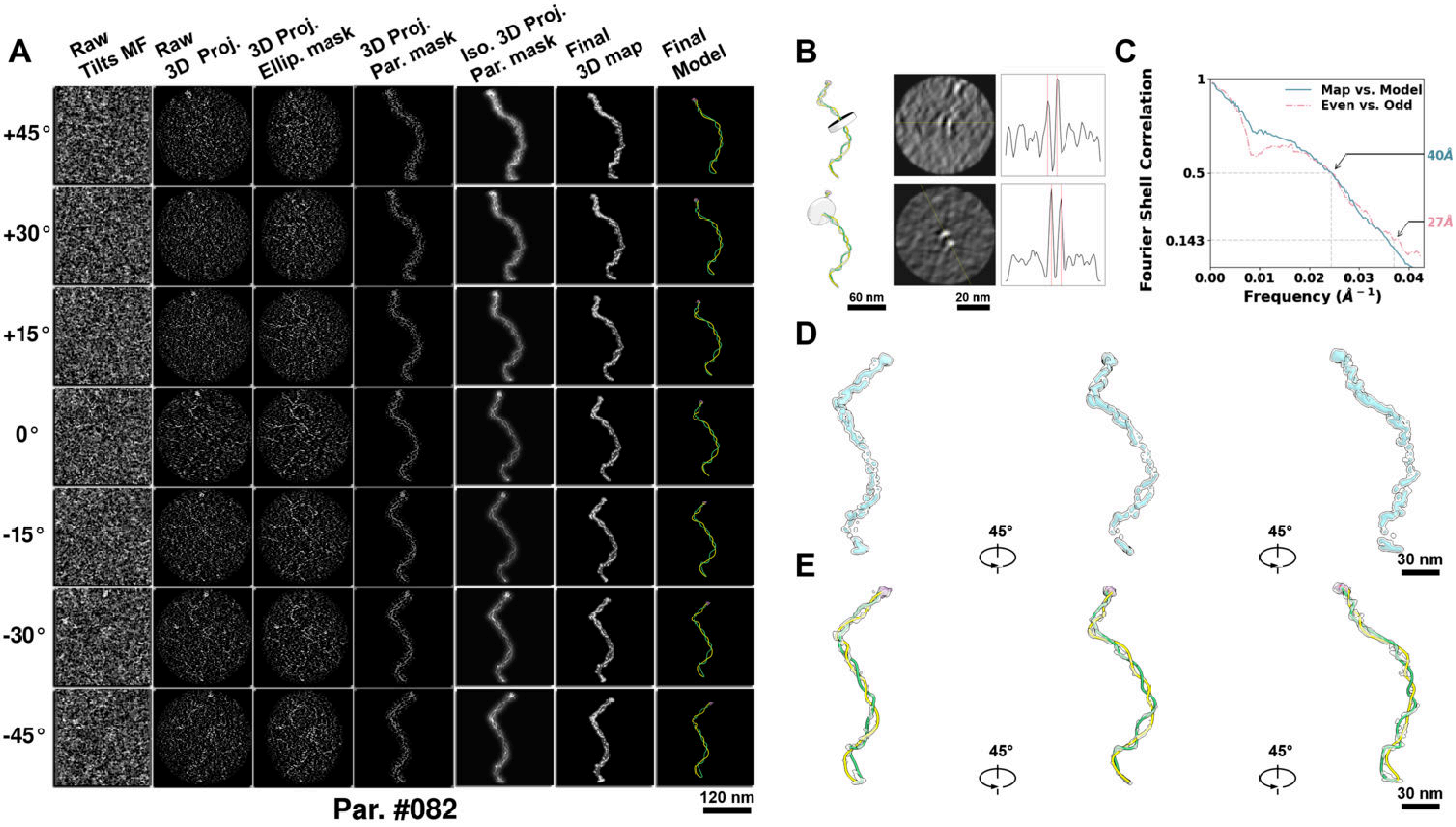

Supplementary Particle Figure 82. Cryo-ET 3D reconstruction of an individual sTEC particle.
(A) 3D reconstruction of the plasmid particle (index no. 82). The first column shows seven representative tilt images from +45° to -45° in step of 15°. The second, third, and fourth columns show 3D projections of the particle with spherical, ellipsoidal (thinner along the z-dimension), and particle-shaped masks, respectively. The fifth column displays the 3D projections of the enhanced and IsoNet missing-wedge-corrected particle. The sixth and seventh columns present the final 3D map and the flexibly fitted model, respectively. (B) Two cross-sectional views (12 nm thickness) of the plasmid density map along its plectoneme axis are shown in the left-middle panel. The intensity profile along the line crossing the two high-density DNA spots is displayed in the right panel. (C) Resolution assessment of the final 3D map using Fourier shell correlation (FSC). Two criteria are shown: FSC between two half-maps reconstructed from even and odd frames (evaluated at 0.143) and FSC between the final 3D map and the fitted model (evaluated at 0.5). (D) Zoomed-in views of the final 3D density map from panel A, displayed at two contour levels. (E) Superimposition of the high-contour level map from panel D onto its fitted model.

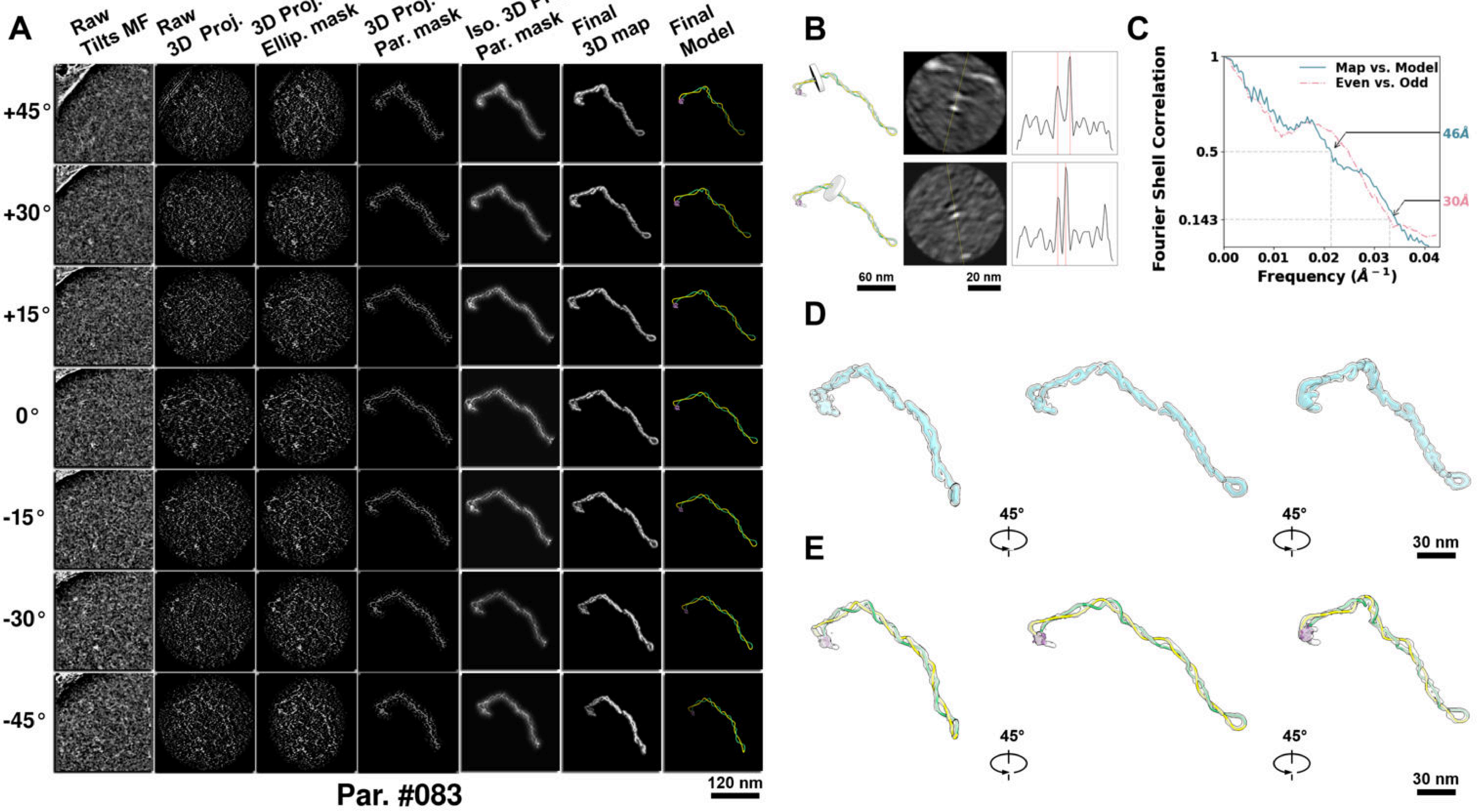

Supplementary Particle Figure 83. Cryo-ET 3D reconstruction of an individual sTEC particle.
(A) 3D reconstruction of the plasmid particle (index no. 83). The first column shows seven representative tilt images from +45° to -45° in step of 15°. The second, third, and fourth columns show 3D projections of the particle with spherical, ellipsoidal (thinner along the z-dimension), and particle-shaped masks, respectively. The fifth column displays the 3D projections of the enhanced and IsoNet missing-wedge-corrected particle. The sixth and seventh columns present the final 3D map and the flexibly fitted model, respectively. (B) Two cross-sectional views (12 nm thickness) of the plasmid density map along its plectoneme axis are shown in the left-middle panel. The intensity profile along the line crossing the two high-density DNA spots is displayed in the right panel. (C) Resolution assessment of the final 3D map using Fourier shell correlation (FSC). Two criteria are shown: FSC between two half-maps reconstructed from even and odd frames (evaluated at 0.143) and FSC between the final 3D map and the fitted model (evaluated at 0.5). (D) Zoomed-in views of the final 3D density map from panel A, displayed at two contour levels. (E) Superimposition of the high-contour level map from panel D onto its fitted model.

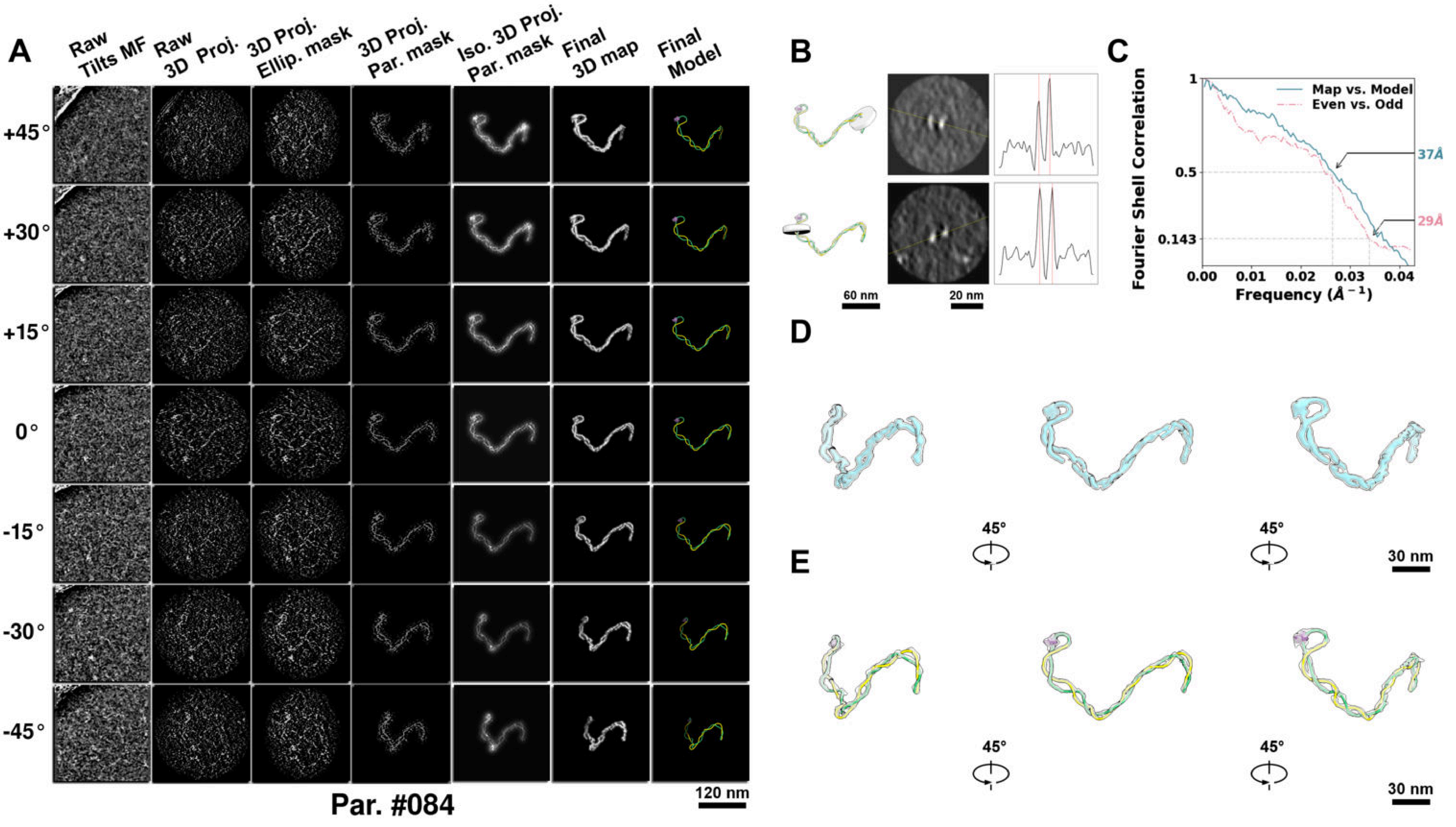

Supplementary Particle Figure 84. Cryo-ET 3D reconstruction of an individual sTEC particle.
(A) 3D reconstruction of the plasmid particle (index no. 84). The first column shows seven representative tilt images from +45° to -45° in step of 15°. The second, third, and fourth columns show 3D projections of the particle with spherical, ellipsoidal (thinner along the z-dimension), and particle-shaped masks, respectively. The fifth column displays the 3D projections of the enhanced and IsoNet missing-wedge-corrected particle. The sixth and seventh columns present the final 3D map and the flexibly fitted model, respectively. (B) Two cross-sectional views (12 nm thickness) of the plasmid density map along its plectoneme axis are shown in the left-middle panel. The intensity profile along the line crossing the two high-density DNA spots is displayed in the right panel. (C) Resolution assessment of the final 3D map using Fourier shell correlation (FSC). Two criteria are shown: FSC between two half-maps reconstructed from even and odd frames (evaluated at 0.143) and FSC between the final 3D map and the fitted model (evaluated at 0.5). (D) Zoomed-in views of the final 3D density map from panel A, displayed at two contour levels. (E) Superimposition of the high-contour level map from panel D onto its fitted model.

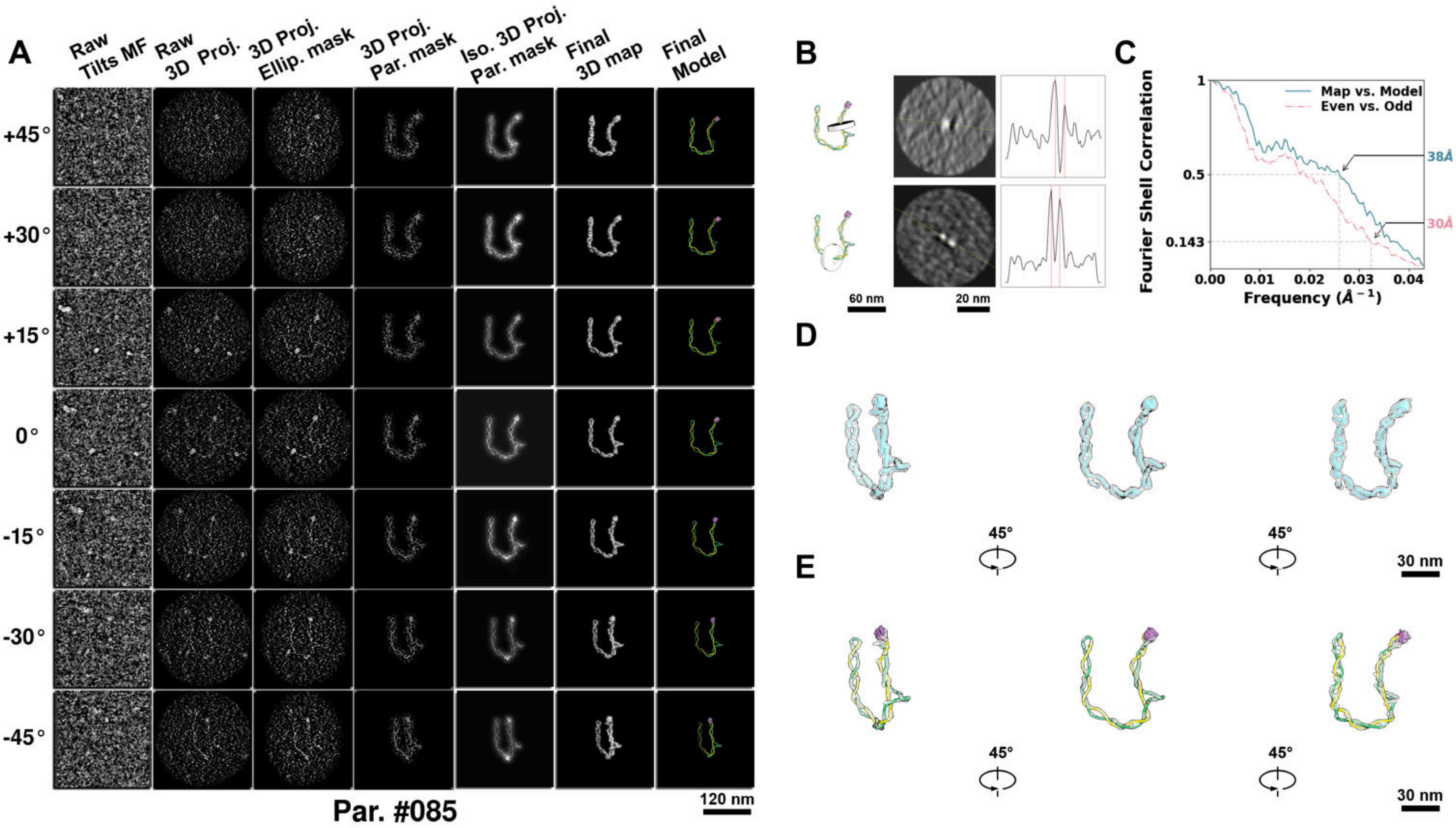

Supplementary Particle Figure 85. Cryo-ET 3D reconstruction of an individual sTEC particle.
(A) 3D reconstruction of the plasmid particle (index no. 85). The first column shows seven representative tilt images from +45° to -45° in step of 15°. The second, third, and fourth columns show 3D projections of the particle with spherical, ellipsoidal (thinner along the z-dimension), and particle-shaped masks, respectively. The fifth column displays the 3D projections of the enhanced and IsoNet missing-wedge-corrected particle. The sixth and seventh columns present the final 3D map and the flexibly fitted model, respectively. (B) Two cross-sectional views (12 nm thickness) of the plasmid density map along its plectoneme axis are shown in the left-middle panel. The intensity profile along the line crossing the two high-density DNA spots is displayed in the right panel. (C) Resolution assessment of the final 3D map using Fourier shell correlation (FSC). Two criteria are shown: FSC between two half-maps reconstructed from even and odd frames (evaluated at 0.143) and FSC between the final 3D map and the fitted model (evaluated at 0.5). (D) Zoomed-in views of the final 3D density map from panel A, displayed at two contour levels. (E) Superimposition of the high-contour level map from panel D onto its fitted model.

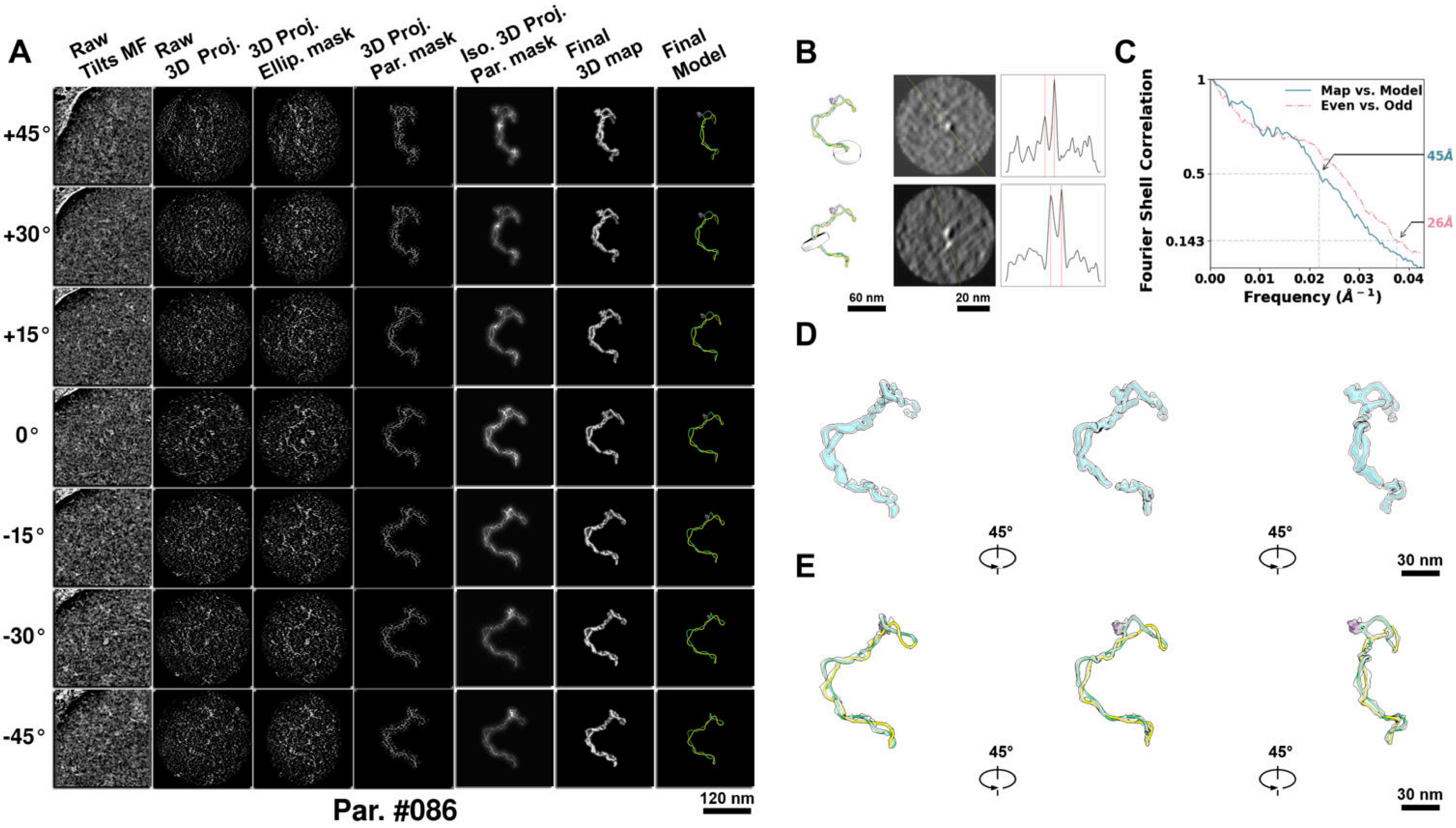

Supplementary Particle Figure 86. Cryo-ET 3D reconstruction of an individual sTEC particle.
(A) 3D reconstruction of the plasmid particle (index no. 86). The first column shows seven representative tilt images from +45° to -45° in step of 15°. The second, third, and fourth columns show 3D projections of the particle with spherical, ellipsoidal (thinner along the z-dimension), and particle-shaped masks, respectively. The fifth column displays the 3D projections of the enhanced and IsoNet missing-wedge-corrected particle. The sixth and seventh columns present the final 3D map and the flexibly fitted model, respectively. (B) Two cross-sectional views (12 nm thickness) of the plasmid density map along its plectoneme axis are shown in the left-middle panel. The intensity profile along the line crossing the two high-density DNA spots is displayed in the right panel. (C) Resolution assessment of the final 3D map using Fourier shell correlation (FSC). Two criteria are shown: FSC between two half-maps reconstructed from even and odd frames (evaluated at 0.143) and FSC between the final 3D map and the fitted model (evaluated at 0.5). (D) Zoomed-in views of the final 3D density map from panel A, displayed at two contour levels. (E) Superimposition of the high-contour level map from panel D onto its fitted model.

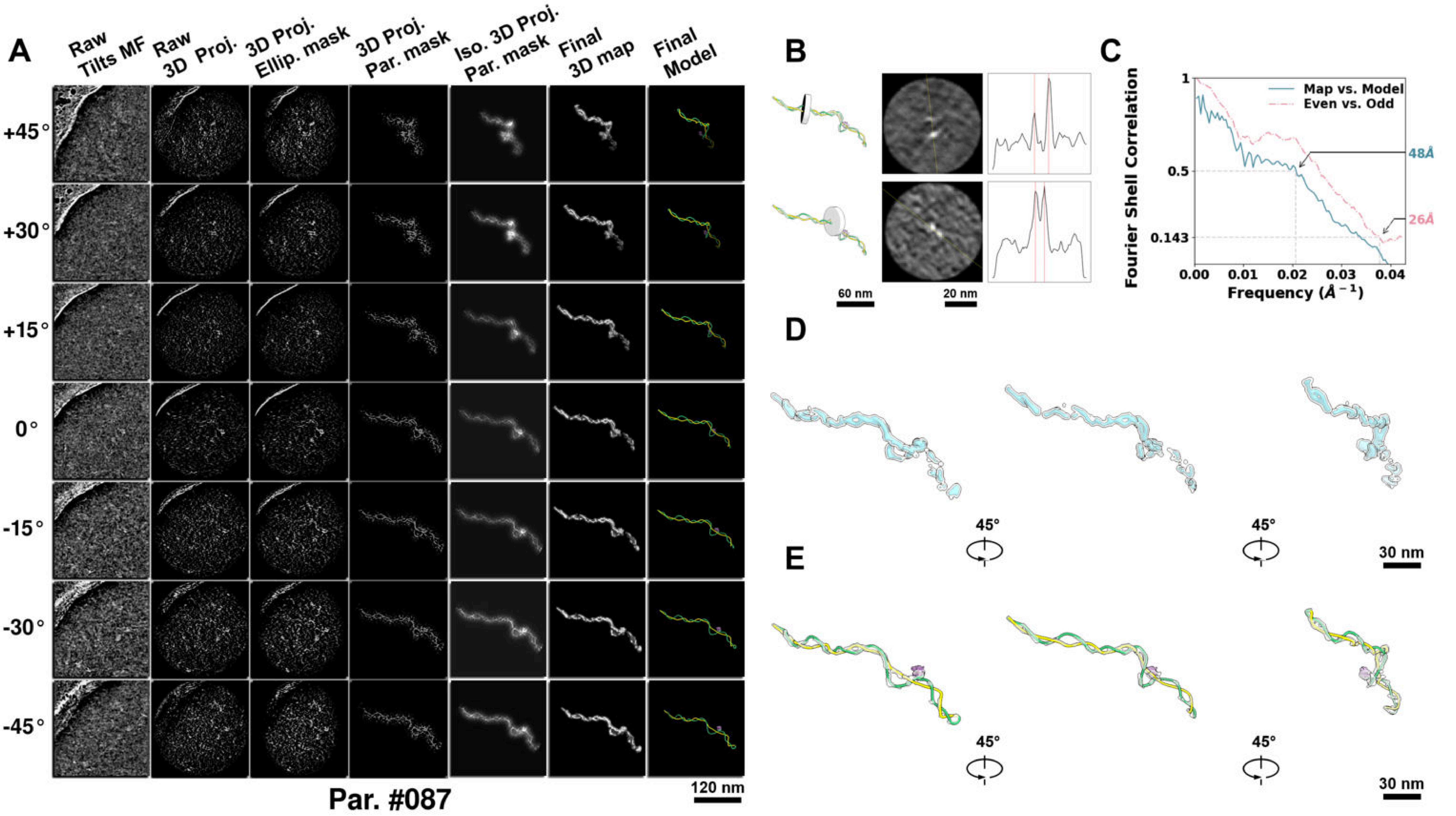

Supplementary Particle Figure 87. Cryo-ET 3D reconstruction of an individual sTEC particle.
(A) 3D reconstruction of the plasmid particle (index no. 87). The first column shows seven representative tilt images from +45° to -45° in step of 15°. The second, third, and fourth columns show 3D projections of the particle with spherical, ellipsoidal (thinner along the z-dimension), and particle-shaped masks, respectively. The fifth column displays the 3D projections of the enhanced and IsoNet missing-wedge-corrected particle. The sixth and seventh columns present the final 3D map and the flexibly fitted model, respectively. (B) Two cross-sectional views (12 nm thickness) of the plasmid density map along its plectoneme axis are shown in the left-middle panel. The intensity profile along the line crossing the two high-density DNA spots is displayed in the right panel. (C) Resolution assessment of the final 3D map using Fourier shell correlation (FSC). Two criteria are shown: FSC between two half-maps reconstructed from even and odd frames (evaluated at 0.143) and FSC between the final 3D map and the fitted model (evaluated at 0.5). (D) Zoomed-in views of the final 3D density map from panel A, displayed at two contour levels. (E) Superimposition of the high-contour level map from panel D onto its fitted model.

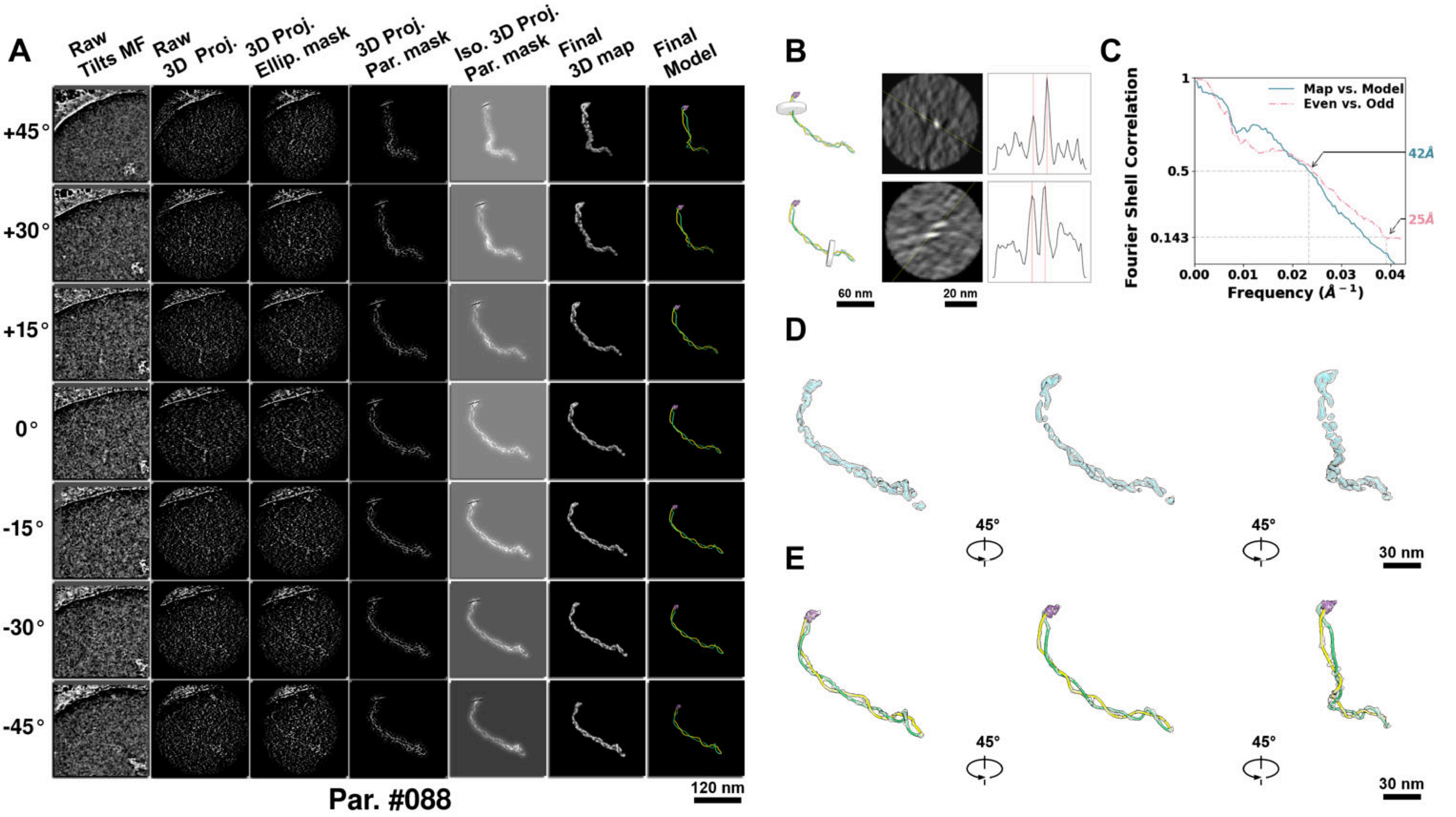

Supplementary Particle Figure 88. Cryo-ET 3D reconstruction of an individual sTEC particle.
(A) 3D reconstruction of the plasmid particle (index no. 88). The first column shows seven representative tilt images from +45° to -45° in step of 15°. The second, third, and fourth columns show 3D projections of the particle with spherical, ellipsoidal (thinner along the z-dimension), and particle-shaped masks, respectively. The fifth column displays the 3D projections of the enhanced and IsoNet missing-wedge-corrected particle. The sixth and seventh columns present the final 3D map and the flexibly fitted model, respectively. (B) Two cross-sectional views (12 nm thickness) of the plasmid density map along its plectoneme axis are shown in the left-middle panel. The intensity profile along the line crossing the two high-density DNA spots is displayed in the right panel. (C) Resolution assessment of the final 3D map using Fourier shell correlation (FSC). Two criteria are shown: FSC between two half-maps reconstructed from even and odd frames (evaluated at 0.143) and FSC between the final 3D map and the fitted model (evaluated at 0.5). (D) Zoomed-in views of the final 3D density map from panel A, displayed at two contour levels. (E) Superimposition of the high-contour level map from panel D onto its fitted model.

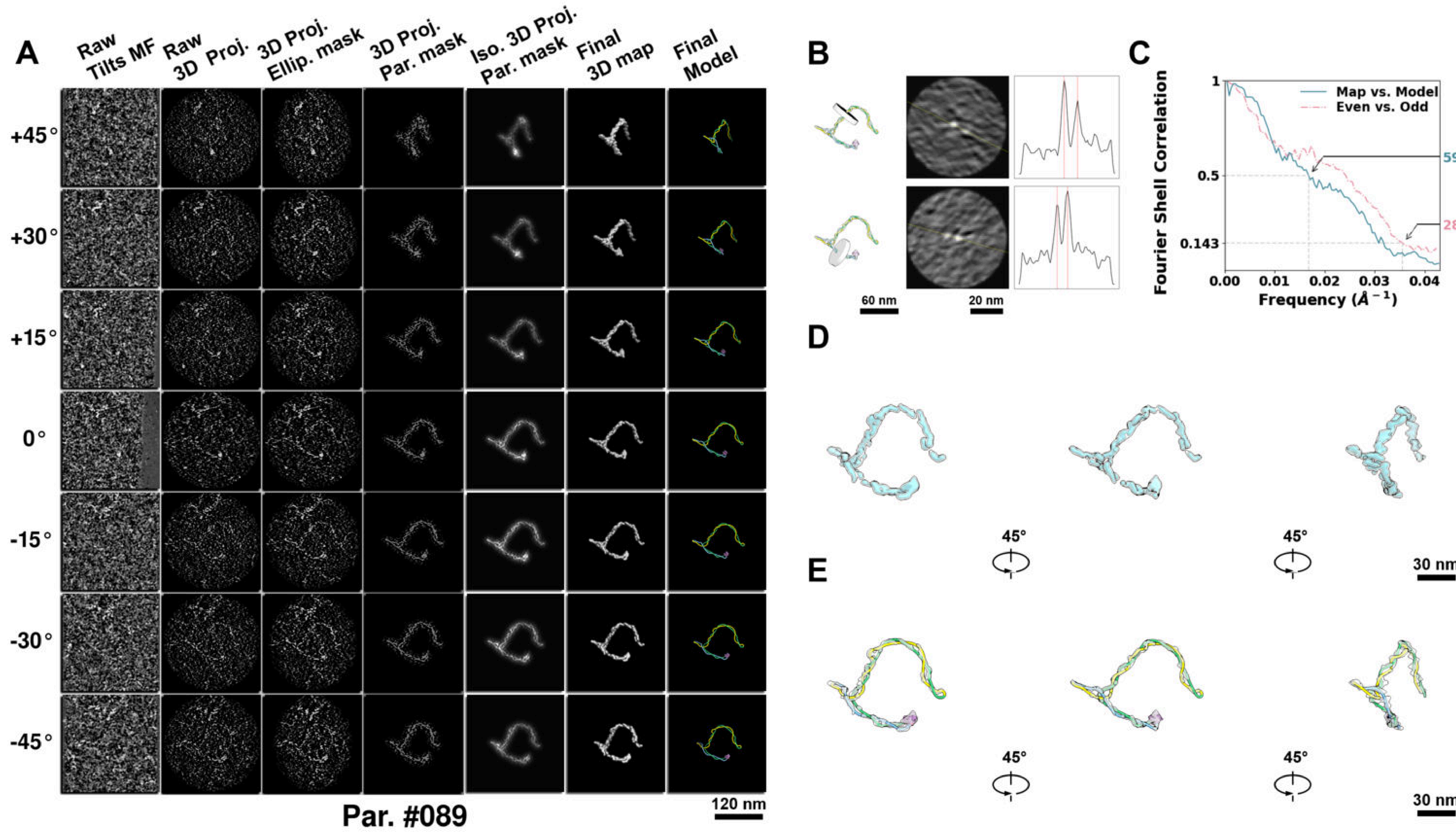

Supplementary Particle Figure 89. Cryo-ET 3D reconstruction of an individual sTEC particle.
(A) 3D reconstruction of the plasmid particle (index no. 89). The first column shows seven representative tilt images from +45° to -45° in step of 15°. The second, third, and fourth columns show 3D projections of the particle with spherical, ellipsoidal (thinner along the z-dimension), and particle-shaped masks, respectively. The fifth column displays the 3D projections of the enhanced and IsoNet missing-wedge-corrected particle. The sixth and seventh columns present the final 3D map and the flexibly fitted model, respectively. (B) Two cross-sectional views (12 nm thickness) of the plasmid density map along its plectoneme axis are shown in the left-middle panel. The intensity profile along the line crossing the two high-density DNA spots is displayed in the right panel. (C) Resolution assessment of the final 3D map using Fourier shell correlation (FSC). Two criteria are shown: FSC between two half-maps reconstructed from even and odd frames (evaluated at 0.143) and FSC between the final 3D map and the fitted model (evaluated at 0.5). (D) Zoomed-in views of the final 3D density map from panel A, displayed at two contour levels. (E) Superimposition of the high-contour level map from panel D onto its fitted model.

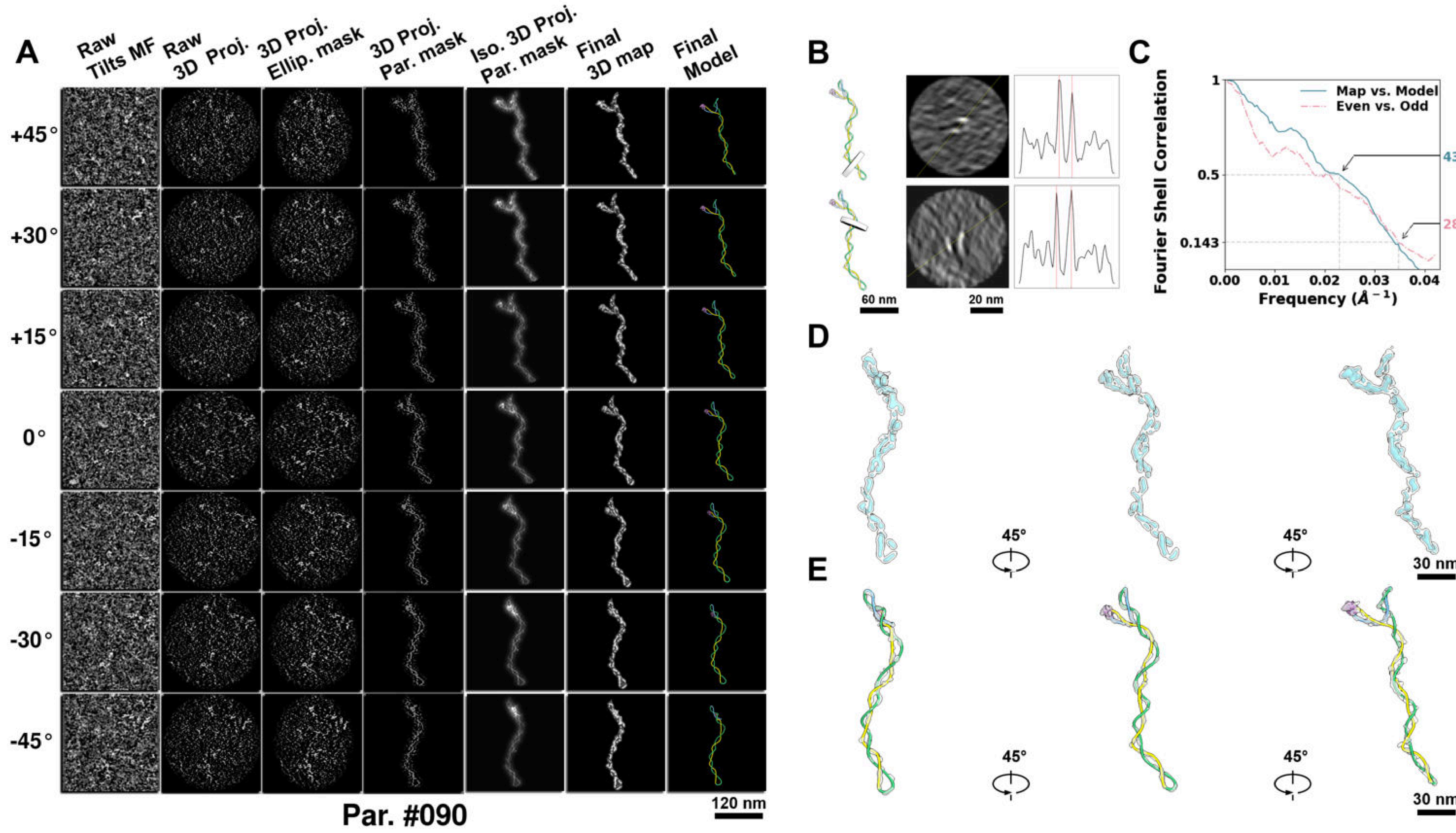

Supplementary Particle Figure 90. Cryo-ET 3D reconstruction of an individual sTEC particle.
(A) 3D reconstruction of the plasmid particle (index no. 90). The first column shows seven representative tilt images from +45° to -45° in step of 15°. The second, third, and fourth columns show 3D projections of the particle with spherical, ellipsoidal (thinner along the z-dimension), and particle-shaped masks, respectively. The fifth column displays the 3D projections of the enhanced and IsoNet missing-wedge-corrected particle. The sixth and seventh columns present the final 3D map and the flexibly fitted model, respectively. (B) Two cross-sectional views (12 nm thickness) of the plasmid density map along its plectoneme axis are shown in the left-middle panel. The intensity profile along the line crossing the two high-density DNA spots is displayed in the right panel. (C) Resolution assessment of the final 3D map using Fourier shell correlation (FSC). Two criteria are shown: FSC between two half-maps reconstructed from even and odd frames (evaluated at 0.143) and FSC between the final 3D map and the fitted model (evaluated at 0.5). (D) Zoomed-in views of the final 3D density map from panel A, displayed at two contour levels. (E) Superimposition of the high-contour level map from panel D onto its fitted model.

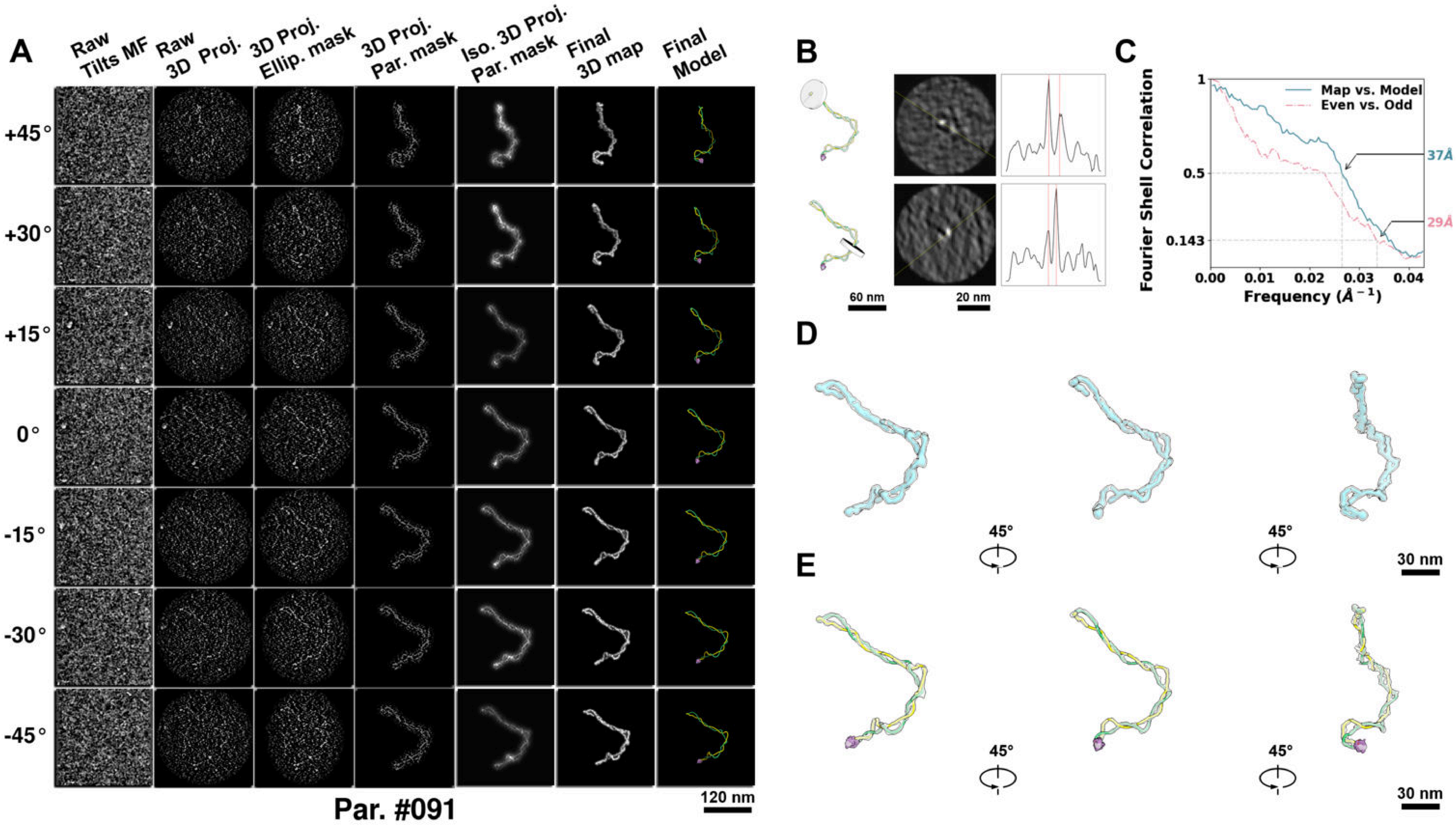

Supplementary Particle Figure 91. Cryo-ET 3D reconstruction of an individual sTEC particle.
(A) 3D reconstruction of the plasmid particle (index no. 91). The first column shows seven representative tilt images from +45° to -45° in step of 15°. The second, third, and fourth columns show 3D projections of the particle with spherical, ellipsoidal (thinner along the z-dimension), and particle-shaped masks, respectively. The fifth column displays the 3D projections of the enhanced and IsoNet missing-wedge-corrected particle. The sixth and seventh columns present the final 3D map and the flexibly fitted model, respectively. (B) Two cross-sectional views (12 nm thickness) of the plasmid density map along its plectoneme axis are shown in the left-middle panel. The intensity profile along the line crossing the two high-density DNA spots is displayed in the right panel. (C) Resolution assessment of the final 3D map using Fourier shell correlation (FSC). Two criteria are shown: FSC between two half-maps reconstructed from even and odd frames (evaluated at 0.143) and FSC between the final 3D map and the fitted model (evaluated at 0.5). (D) Zoomed-in views of the final 3D density map from panel A, displayed at two contour levels. (E) Superimposition of the high-contour level map from panel D onto its fitted model.

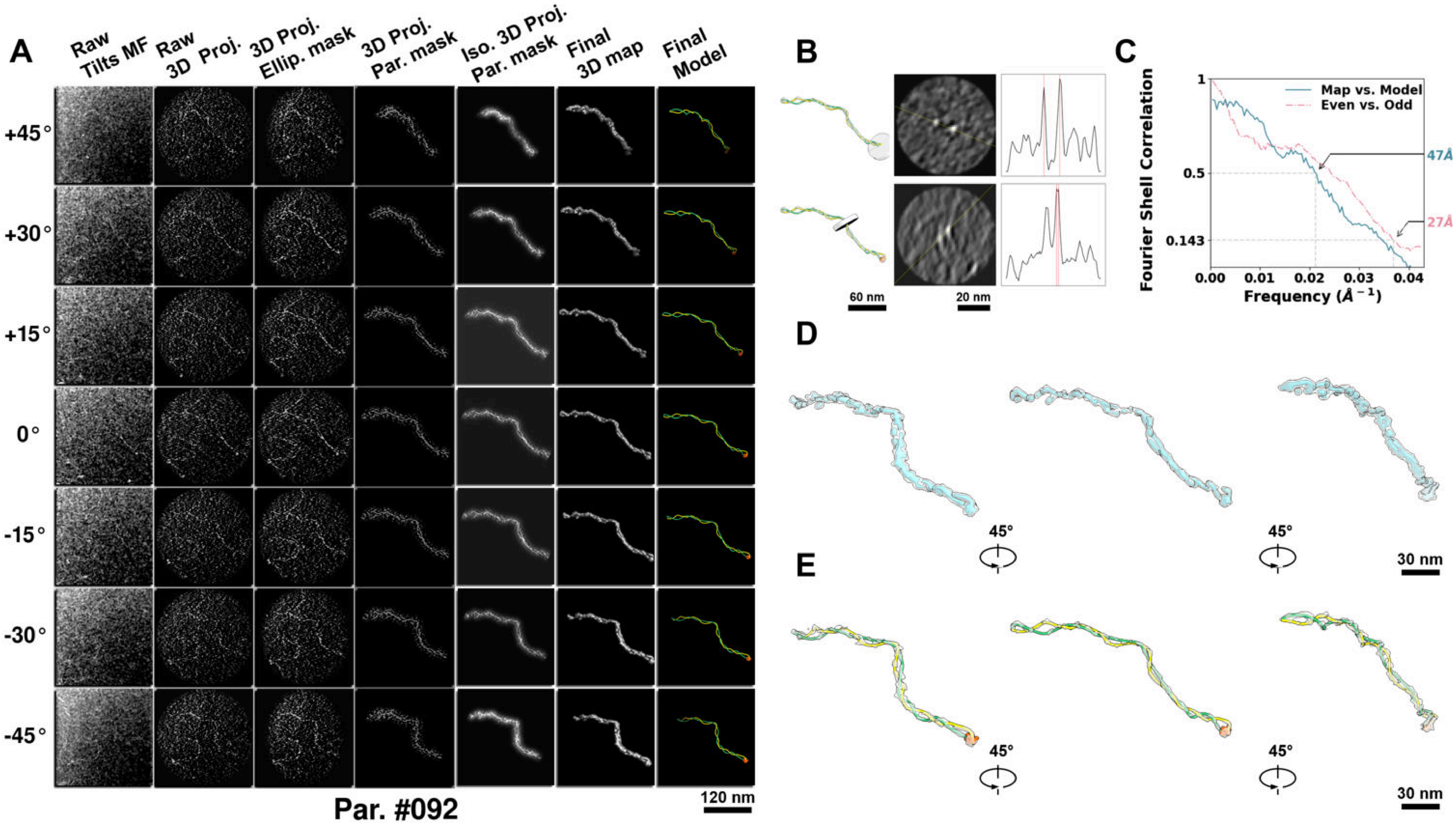

Supplementary Particle Figure 92. Cryo-ET 3D reconstruction of an individual P.Cas particle.
(A) 3D reconstruction of the plasmid particle (index no. 92). The first column shows seven representative tilt images from +45° to -45° in step of 15°. The second, third, and fourth columns show 3D projections of the particle with spherical, ellipsoidal (thinner along the z-dimension), and particle-shaped masks, respectively. The fifth column displays the 3D projections of the enhanced and IsoNet missing-wedge-corrected particle. The sixth and seventh columns present the final 3D map and the flexibly fitted model, respectively. (B) Two cross-sectional views (12 nm thickness) of the plasmid density map along its plectoneme axis are shown in the left-middle panel. The intensity profile along the line crossing the two high-density DNA spots is displayed in the right panel. (C) Resolution assessment of the final 3D map using Fourier shell correlation (FSC). Two criteria are shown: FSC between two half-maps reconstructed from even and odd frames (evaluated at 0.143) and FSC between the final 3D map and the fitted model (evaluated at 0.5). (D) Zoomed-in views of the final 3D density map from panel A, displayed at two contour levels. (E) Superimposition of the high-contour level map from panel D onto its fitted model.

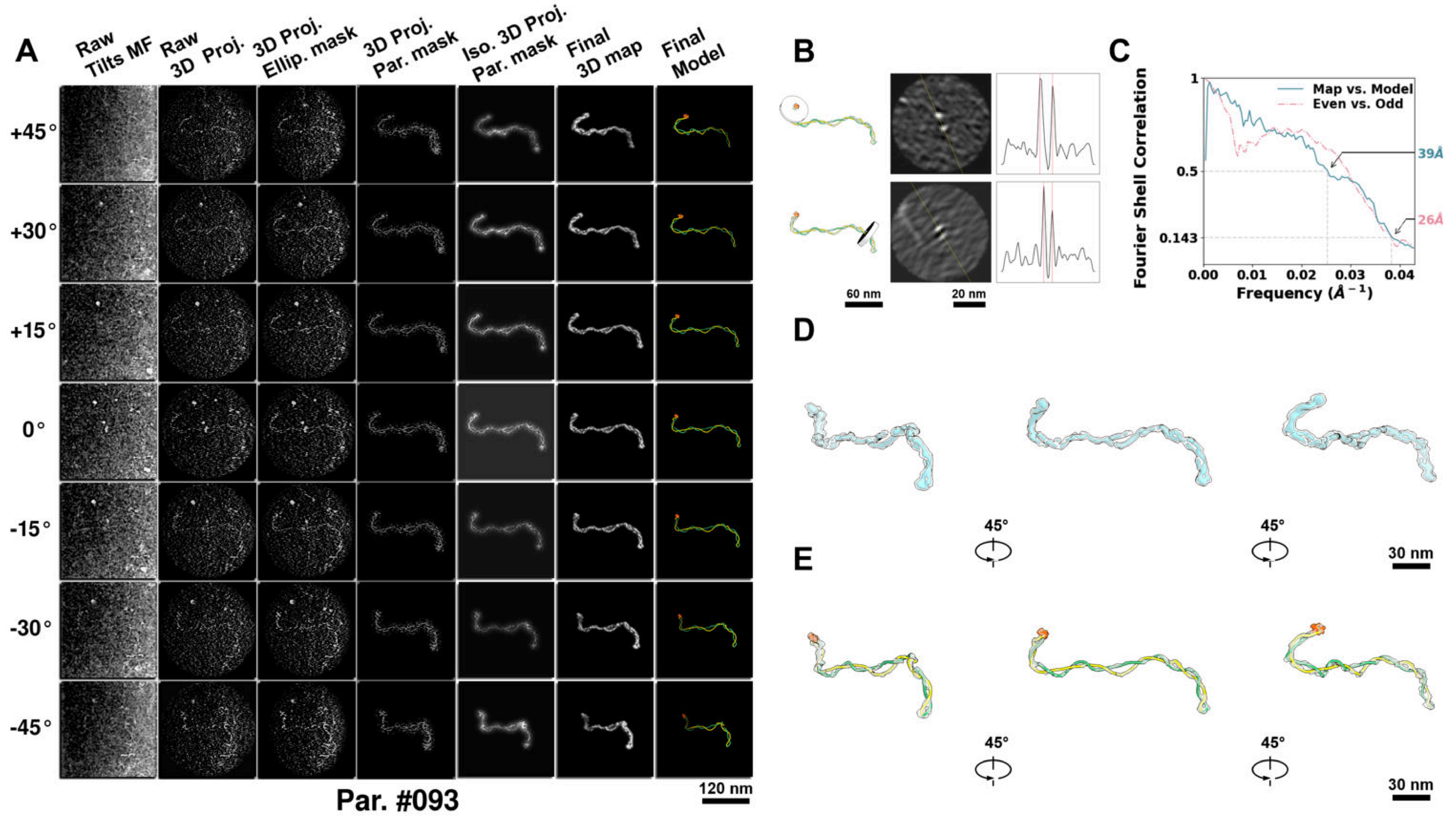

Supplementary Particle Figure 93. Cryo-ET 3D reconstruction of an individual P.Cas particle.
(A) 3D reconstruction of the plasmid particle (index no. 93). The first column shows seven representative tilt images from +45° to -45° in step of 15°. The second, third, and fourth columns show 3D projections of the particle with spherical, ellipsoidal (thinner along the z-dimension), and particle-shaped masks, respectively. The fifth column displays the 3D projections of the enhanced and IsoNet missing-wedge-corrected particle. The sixth and seventh columns present the final 3D map and the flexibly fitted model, respectively. (B) Two cross-sectional views (12 nm thickness) of the plasmid density map along its plectoneme axis are shown in the left-middle panel. The intensity profile along the line crossing the two high-density DNA spots is displayed in the right panel. (C) Resolution assessment of the final 3D map using Fourier shell correlation (FSC). Two criteria are shown: FSC between two half-maps reconstructed from even and odd frames (evaluated at 0.143) and FSC between the final 3D map and the fitted model (evaluated at 0.5). (D) Zoomed-in views of the final 3D density map from panel A, displayed at two contour levels. (E) Superimposition of the high-contour level map from panel D onto its fitted model.

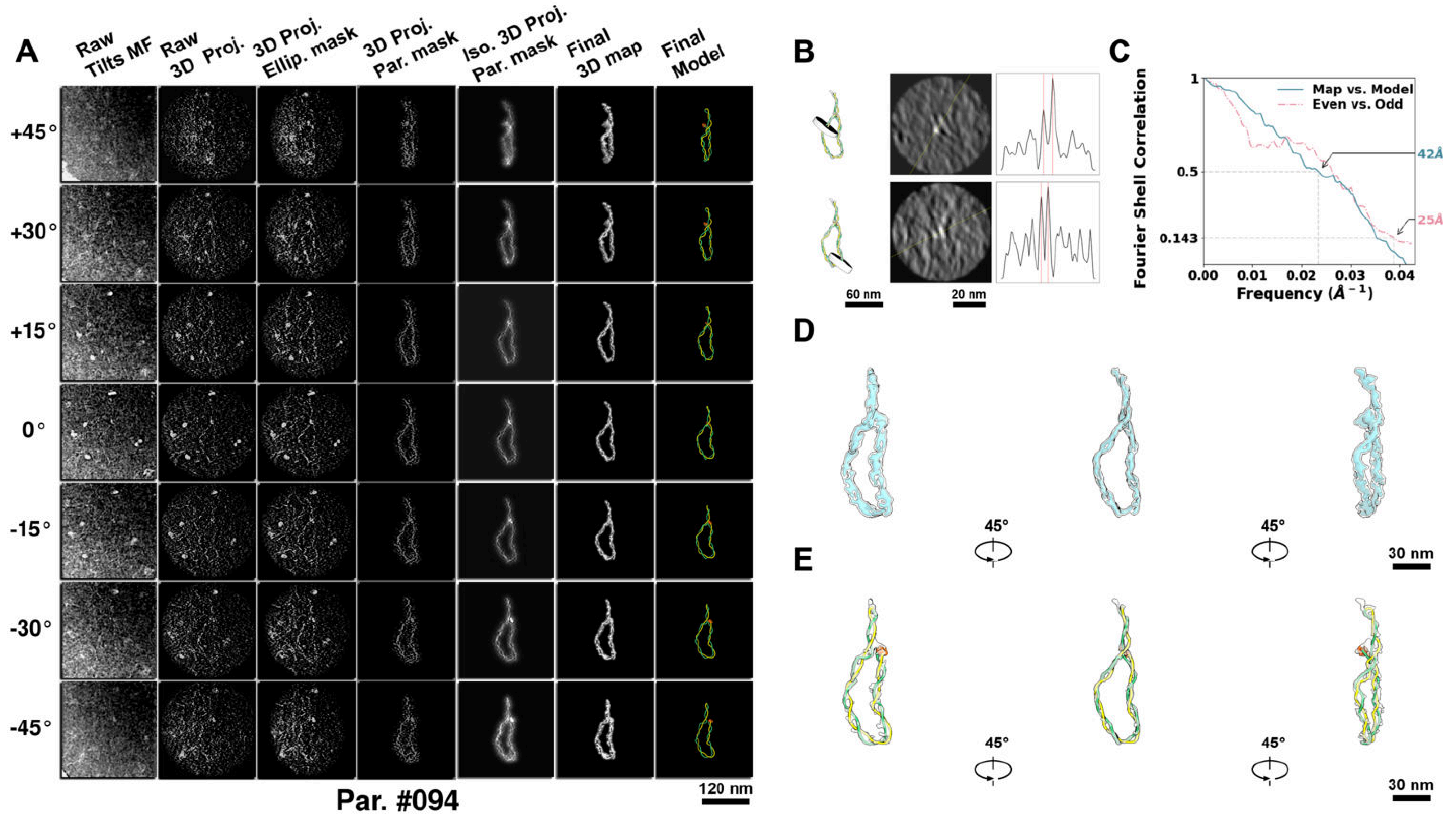

Supplementary Particle Figure 94. Cryo-ET 3D reconstruction of an individual P.Cas particle.
(A) 3D reconstruction of the plasmid particle (index no. 94). The first column shows seven representative tilt images from +45° to -45° in step of 15°. The second, third, and fourth columns show 3D projections of the particle with spherical, ellipsoidal (thinner along the z-dimension), and particle-shaped masks, respectively. The fifth column displays the 3D projections of the enhanced and IsoNet missing-wedge-corrected particle. The sixth and seventh columns present the final 3D map and the flexibly fitted model, respectively. (B) Two cross-sectional views (12 nm thickness) of the plasmid density map along its plectoneme axis are shown in the left-middle panel. The intensity profile along the line crossing the two high-density DNA spots is displayed in the right panel. (C) Resolution assessment of the final 3D map using Fourier shell correlation (FSC). Two criteria are shown: FSC between two half-maps reconstructed from even and odd frames (evaluated at 0.143) and FSC between the final 3D map and the fitted model (evaluated at 0.5). (D) Zoomed-in views of the final 3D density map from panel A, displayed at two contour levels. (E) Superimposition of the high-contour level map from panel D onto its fitted model.

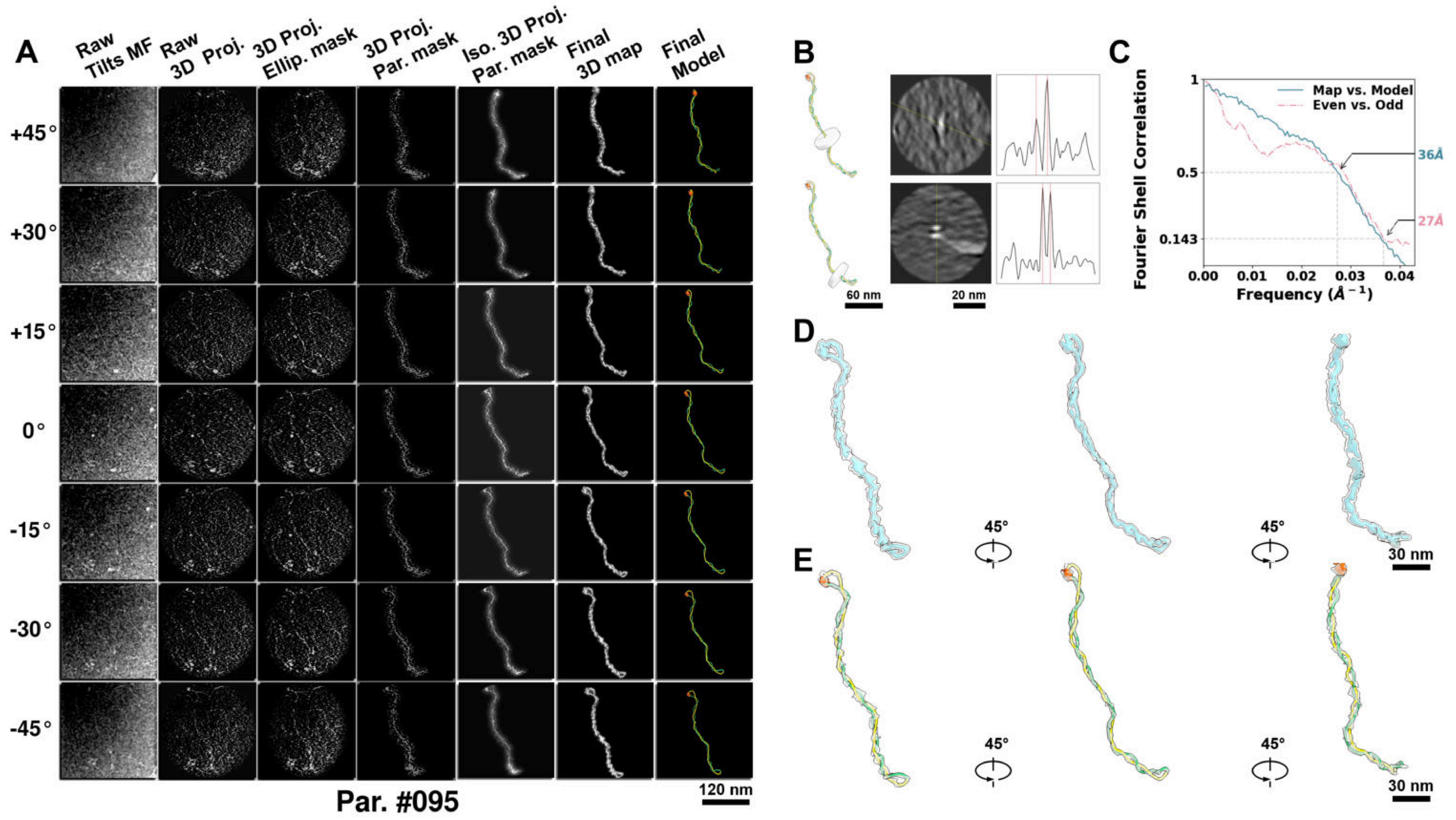

Supplementary Particle Figure 95. Cryo-ET 3D reconstruction of an individual P.Cas particle.
(A) 3D reconstruction of the plasmid particle (index no. 95). The first column shows seven representative tilt images from +45° to -45° in step of 15°. The second, third, and fourth columns show 3D projections of the particle with spherical, ellipsoidal (thinner along the z-dimension), and particle-shaped masks, respectively. The fifth column displays the 3D projections of the enhanced and IsoNet missing-wedge-corrected particle. The sixth and seventh columns present the final 3D map and the flexibly fitted model, respectively. (B) Two cross-sectional views (12 nm thickness) of the plasmid density map along its plectoneme axis are shown in the left-middle panel. The intensity profile along the line crossing the two high-density DNA spots is displayed in the right panel. (C) Resolution assessment of the final 3D map using Fourier shell correlation (FSC). Two criteria are shown: FSC between two half-maps reconstructed from even and odd frames (evaluated at 0.143) and FSC between the final 3D map and the fitted model (evaluated at 0.5). (D) Zoomed-in views of the final 3D density map from panel A, displayed at two contour levels. (E) Superimposition of the high-contour level map from panel D onto its fitted model.

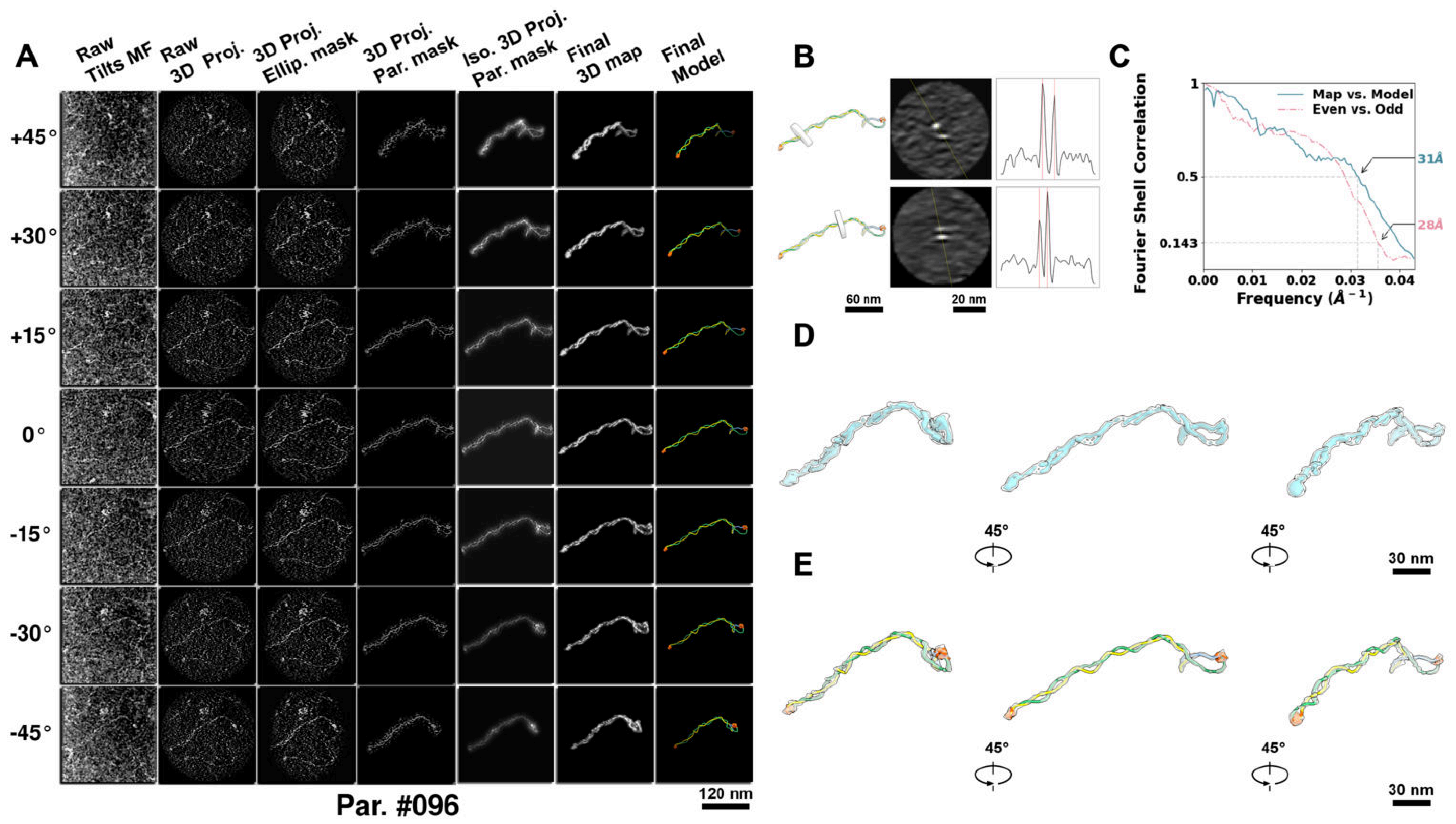

Supplementary Particle Figure 96. Cryo-ET 3D reconstruction of an individual P.Cas particle.
(A) 3D reconstruction of the plasmid particle (index no. 96). The first column shows seven representative tilt images from +45° to -45° in step of 15°. The second, third, and fourth columns show 3D projections of the particle with spherical, ellipsoidal (thinner along the z-dimension), and particle-shaped masks, respectively. The fifth column displays the 3D projections of the enhanced and IsoNet missing-wedge-corrected particle. The sixth and seventh columns present the final 3D map and the flexibly fitted model, respectively. (B) Two cross-sectional views (12 nm thickness) of the plasmid density map along its plectoneme axis are shown in the left-middle panel. The intensity profile along the line crossing the two high-density DNA spots is displayed in the right panel. (C) Resolution assessment of the final 3D map using Fourier shell correlation (FSC). Two criteria are shown: FSC between two half-maps reconstructed from even and odd frames (evaluated at 0.143) and FSC between the final 3D map and the fitted model (evaluated at 0.5). (D) Zoomed-in views of the final 3D density map from panel A, displayed at two contour levels. (E) Superimposition of the high-contour level map from panel D onto its fitted model.

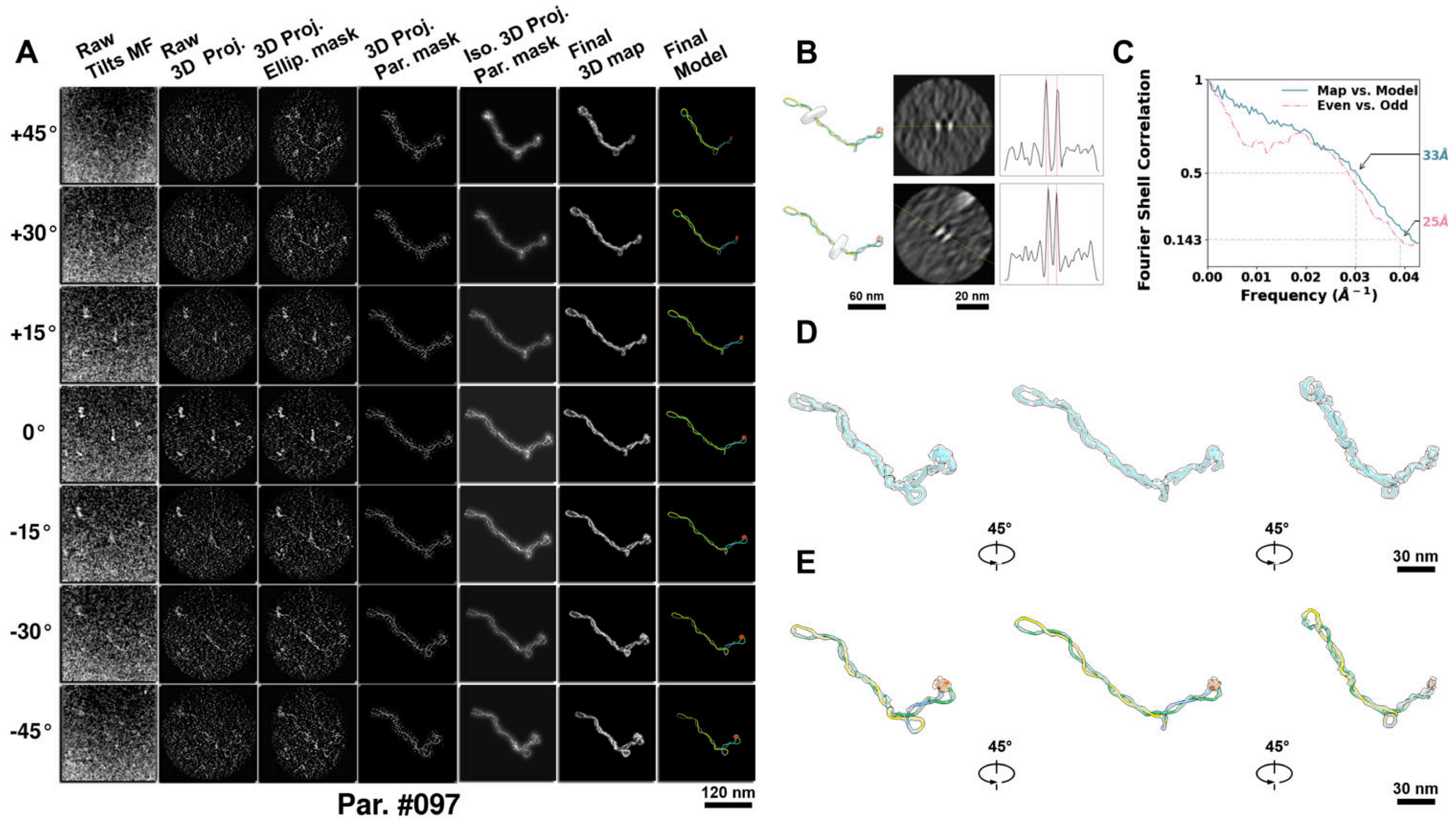

Supplementary Particle Figure 97. Cryo-ET 3D reconstruction of an individual P.Cas particle.
(A) 3D reconstruction of the plasmid particle (index no. 97). The first column shows seven representative tilt images from +45° to -45° in step of 15°. The second, third, and fourth columns show 3D projections of the particle with spherical, ellipsoidal (thinner along the z-dimension), and particle-shaped masks, respectively. The fifth column displays the 3D projections of the enhanced and IsoNet missing-wedge-corrected particle. The sixth and seventh columns present the final 3D map and the flexibly fitted model, respectively. (B) Two cross-sectional views (12 nm thickness) of the plasmid density map along its plectoneme axis are shown in the left-middle panel. The intensity profile along the line crossing the two high-density DNA spots is displayed in the right panel. (C) Resolution assessment of the final 3D map using Fourier shell correlation (FSC). Two criteria are shown: FSC between two half-maps reconstructed from even and odd frames (evaluated at 0.143) and FSC between the final 3D map and the fitted model (evaluated at 0.5). (D) Zoomed-in views of the final 3D density map from panel A, displayed at two contour levels. (E) Superimposition of the high-contour level map from panel D onto its fitted model.

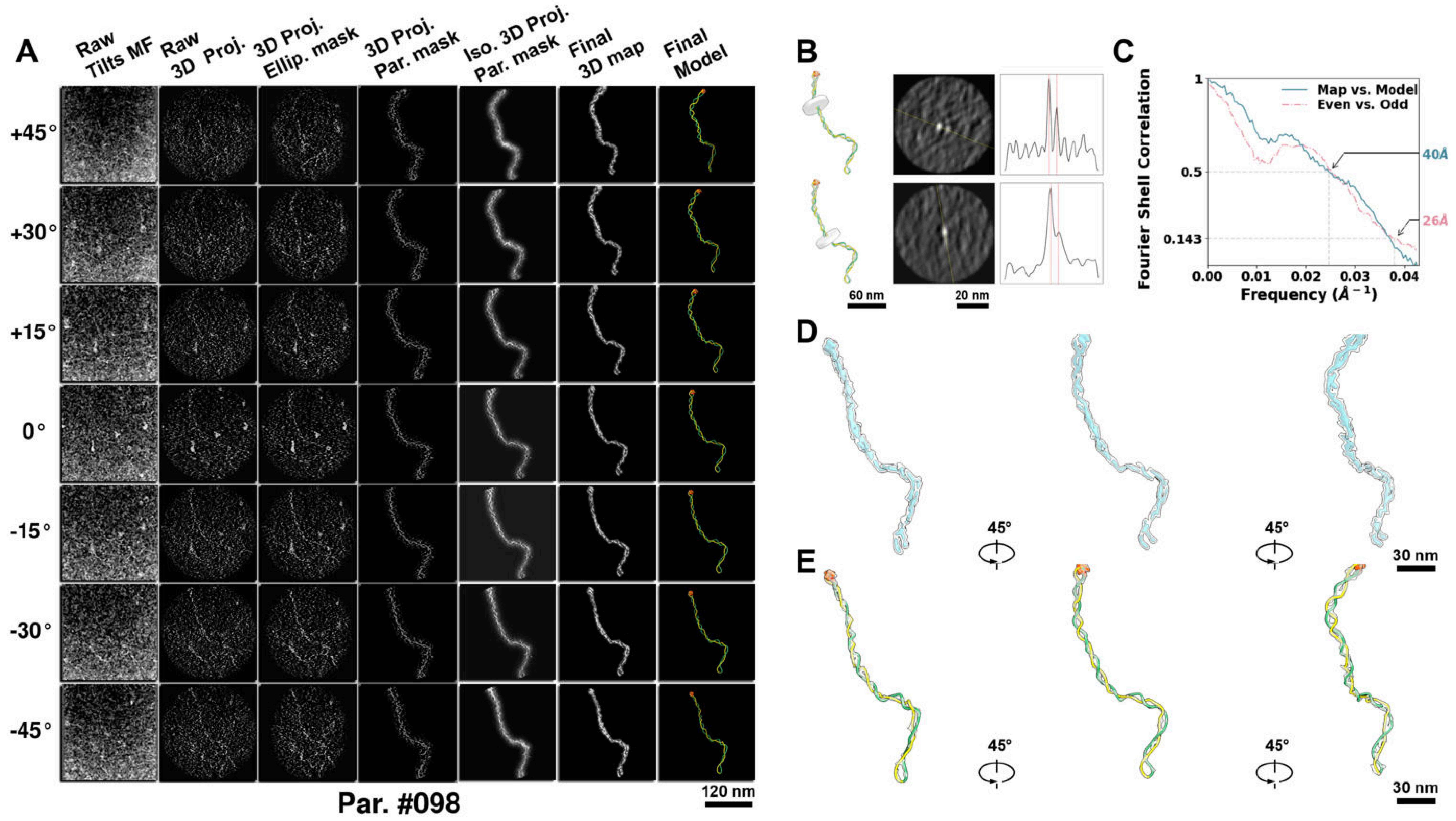

Supplementary Particle Figure 98. Cryo-ET 3D reconstruction of an individual P.Cas particle.
(A) 3D reconstruction of the plasmid particle (index no. 98). The first column shows seven representative tilt images from +45° to -45° in step of 15°. The second, third, and fourth columns show 3D projections of the particle with spherical, ellipsoidal (thinner along the z-dimension), and particle-shaped masks, respectively. The fifth column displays the 3D projections of the enhanced and IsoNet missing-wedge-corrected particle. The sixth and seventh columns present the final 3D map and the flexibly fitted model, respectively. (B) Two cross-sectional views (12 nm thickness) of the plasmid density map along its plectoneme axis are shown in the left-middle panel. The intensity profile along the line crossing the two high-density DNA spots is displayed in the right panel. (C) Resolution assessment of the final 3D map using Fourier shell correlation (FSC). Two criteria are shown: FSC between two half-maps reconstructed from even and odd frames (evaluated at 0.143) and FSC between the final 3D map and the fitted model (evaluated at 0.5). (D) Zoomed-in views of the final 3D density map from panel A, displayed at two contour levels. (E) Superimposition of the high-contour level map from panel D onto its fitted model.

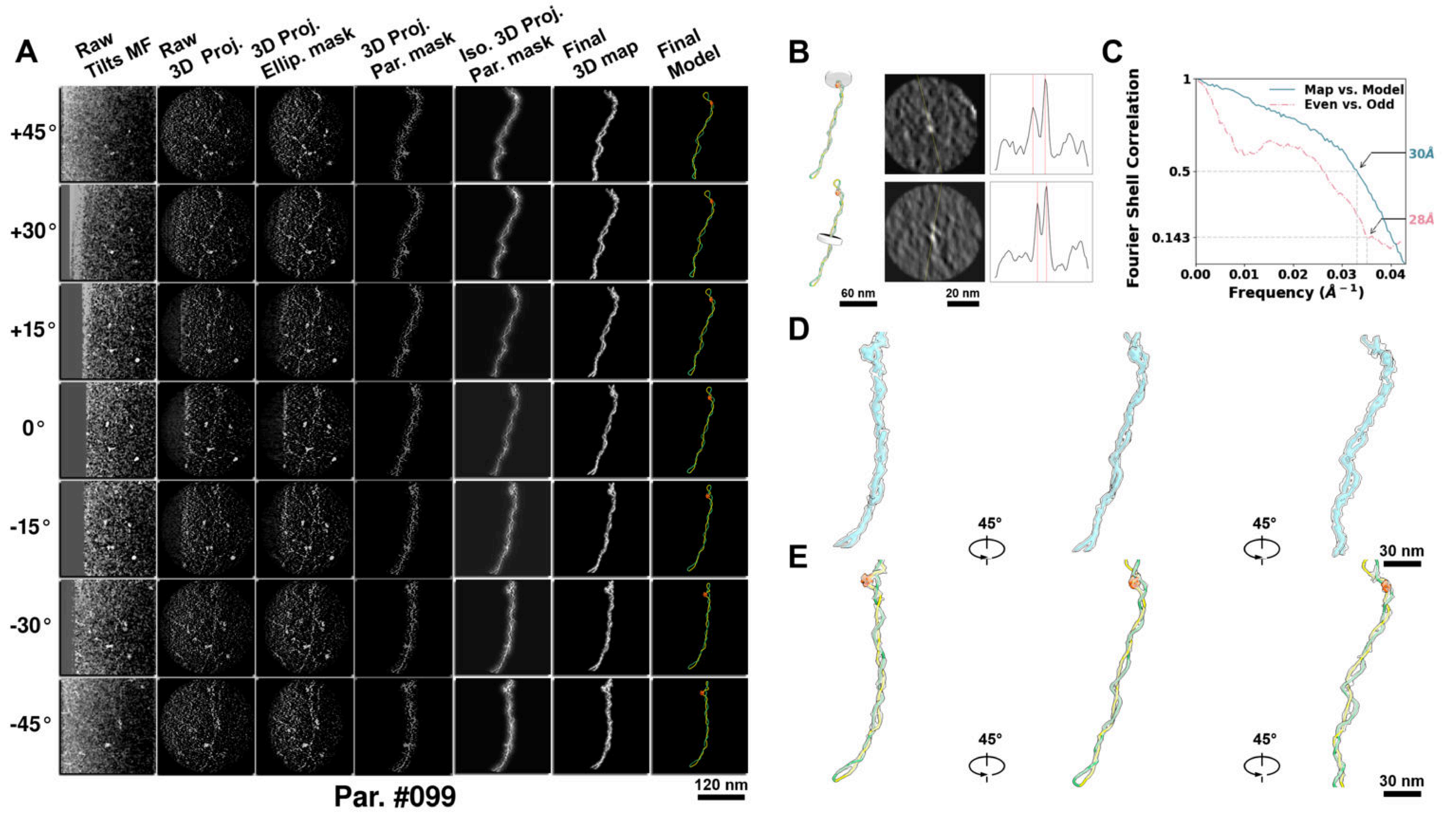

Supplementary Particle Figure 99. Cryo-ET 3D reconstruction of an individual P.Cas particle.
(A) 3D reconstruction of the plasmid particle (index no. 99). The first column shows seven representative tilt images from +45° to -45° in step of 15°. The second, third, and fourth columns show 3D projections of the particle with spherical, ellipsoidal (thinner along the z-dimension), and particle-shaped masks, respectively. The fifth column displays the 3D projections of the enhanced and IsoNet missing-wedge-corrected particle. The sixth and seventh columns present the final 3D map and the flexibly fitted model, respectively. (B) Two cross-sectional views (12 nm thickness) of the plasmid density map along its plectoneme axis are shown in the left-middle panel. The intensity profile along the line crossing the two high-density DNA spots is displayed in the right panel. (C) Resolution assessment of the final 3D map using Fourier shell correlation (FSC). Two criteria are shown: FSC between two half-maps reconstructed from even and odd frames (evaluated at 0.143) and FSC between the final 3D map and the fitted model (evaluated at 0.5). (D) Zoomed-in views of the final 3D density map from panel A, displayed at two contour levels. (E) Superimposition of the high-contour level map from panel D onto its fitted model.

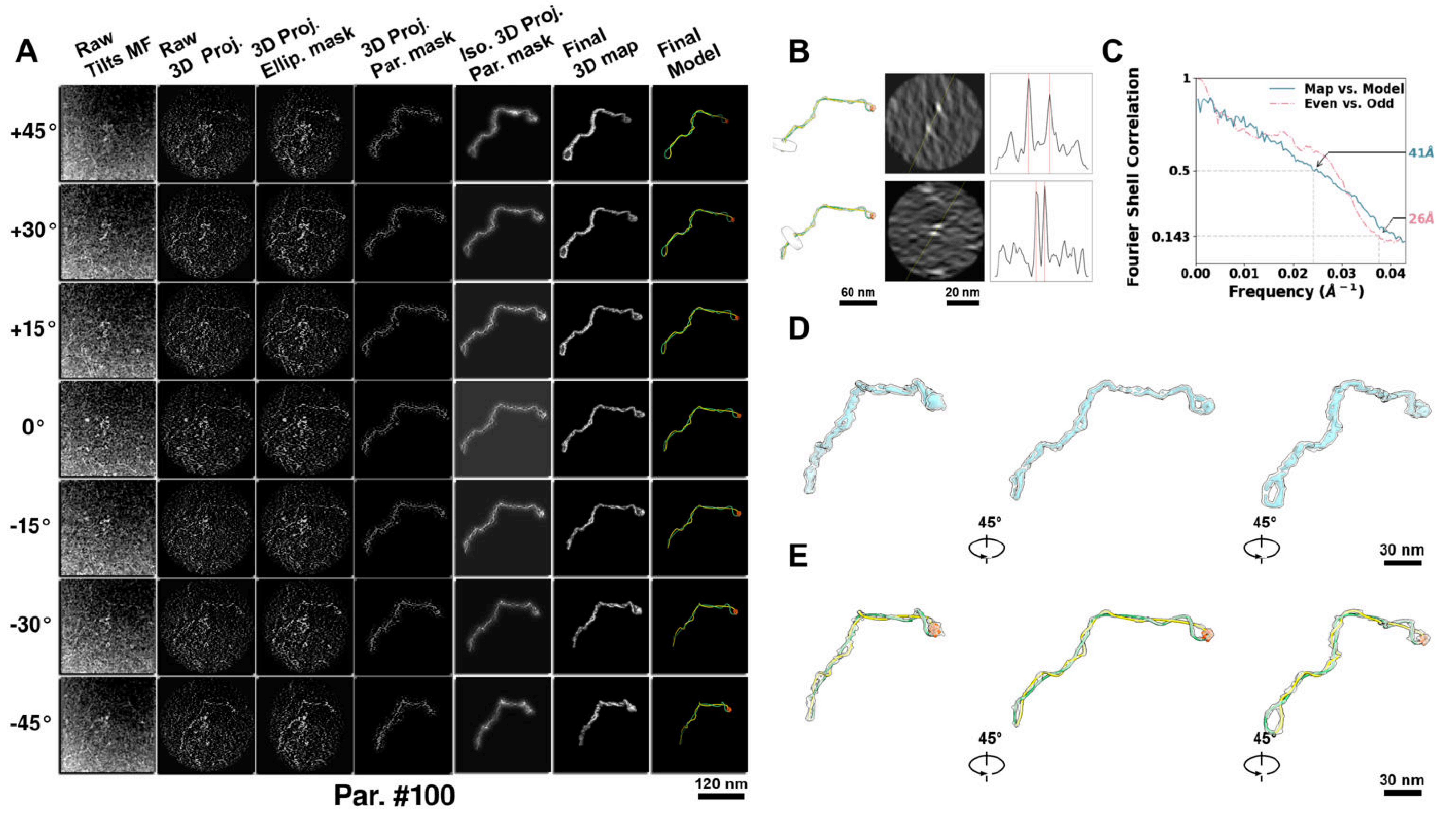

Supplementary Particle Figure 100. Cryo-ET 3D reconstruction of an individual P.Cas particle.
(A) 3D reconstruction of the plasmid particle (index no. 100). The first column shows seven representative tilt images from +45° to -45° in step of 15°. The second, third, and fourth columns show 3D projections of the particle with spherical, ellipsoidal (thinner along the z-dimension), and particle-shaped masks, respectively. The fifth column displays the 3D projections of the enhanced and IsoNet missing-wedge-corrected particle. The sixth and seventh columns present the final 3D map and the flexibly fitted model, respectively. (B) Two cross-sectional views (12 nm thickness) of the plasmid density map along its plectoneme axis are shown in the left-middle panel. The intensity profile along the line crossing the two high-density DNA spots is displayed in the right panel. (C) Resolution assessment of the final 3D map using Fourier shell correlation (FSC). Two criteria are shown: FSC between two half-maps reconstructed from even and odd frames (evaluated at 0.143) and FSC between the final 3D map and the fitted model (evaluated at 0.5). (D) Zoomed-in views of the final 3D density map from panel A, displayed at two contour levels. (E) Superimposition of the high-contour level map from panel D onto its fitted model.

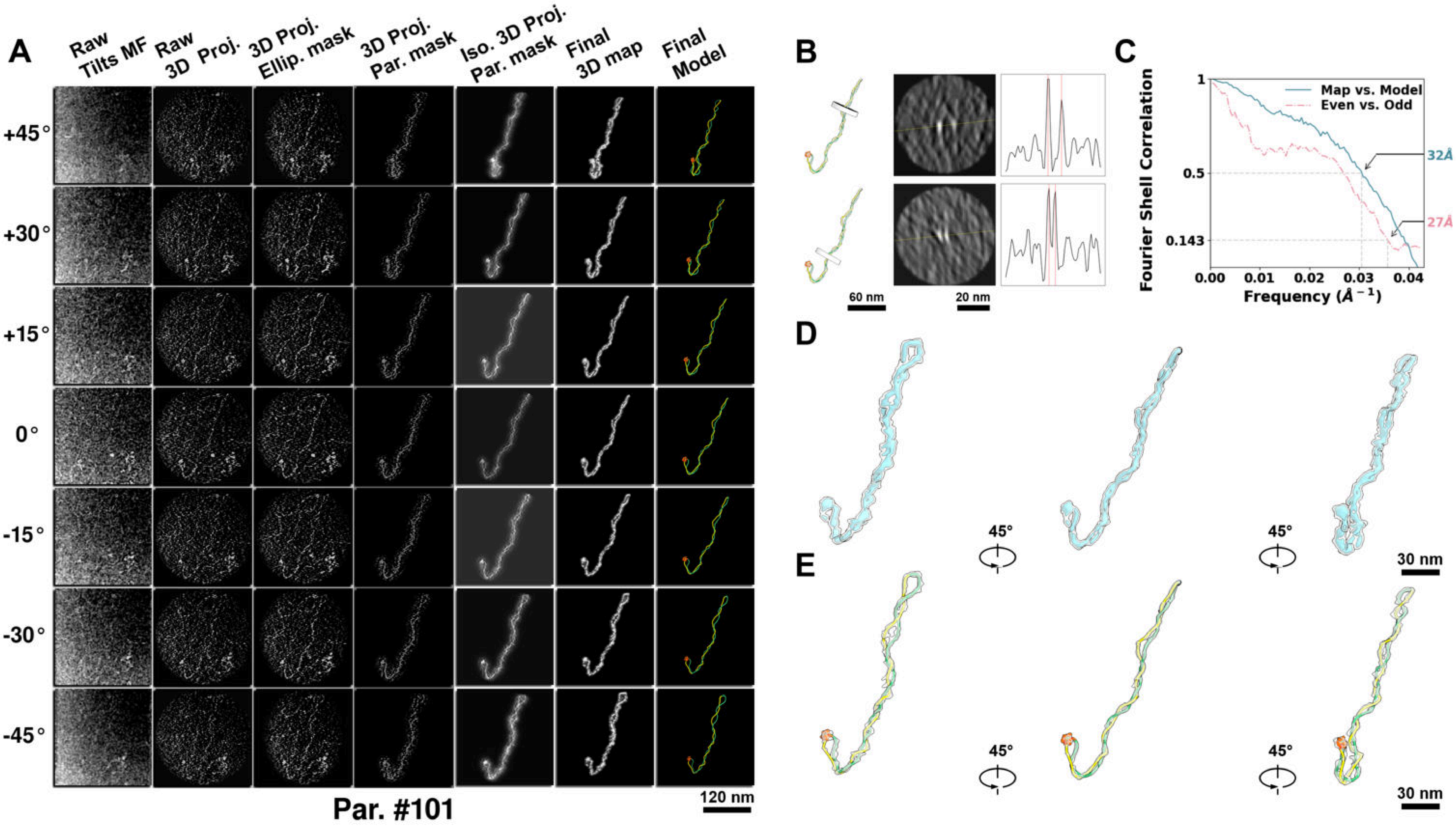

Supplementary Particle Figure 101. Cryo-ET 3D reconstruction of an individual P.Cas particle.
(A) 3D reconstruction of the plasmid particle (index no. 101). The first column shows seven representative tilt images from +45° to -45° in step of 15°. The second, third, and fourth columns show 3D projections of the particle with spherical, ellipsoidal (thinner along the z-dimension), and particle-shaped masks, respectively. The fifth column displays the 3D projections of the enhanced and IsoNet missing-wedge-corrected particle. The sixth and seventh columns present the final 3D map and the flexibly fitted model, respectively. (B) Two cross-sectional views (12 nm thickness) of the plasmid density map along its plectoneme axis are shown in the left-middle panel. The intensity profile along the line crossing the two high-density DNA spots is displayed in the right panel. (C) Resolution assessment of the final 3D map using Fourier shell correlation (FSC). Two criteria are shown: FSC between two half-maps reconstructed from even and odd frames (evaluated at 0.143) and FSC between the final 3D map and the fitted model (evaluated at 0.5). (D) Zoomed-in views of the final 3D density map from panel A, displayed at two contour levels. (E) Superimposition of the high-contour level map from panel D onto its fitted model.

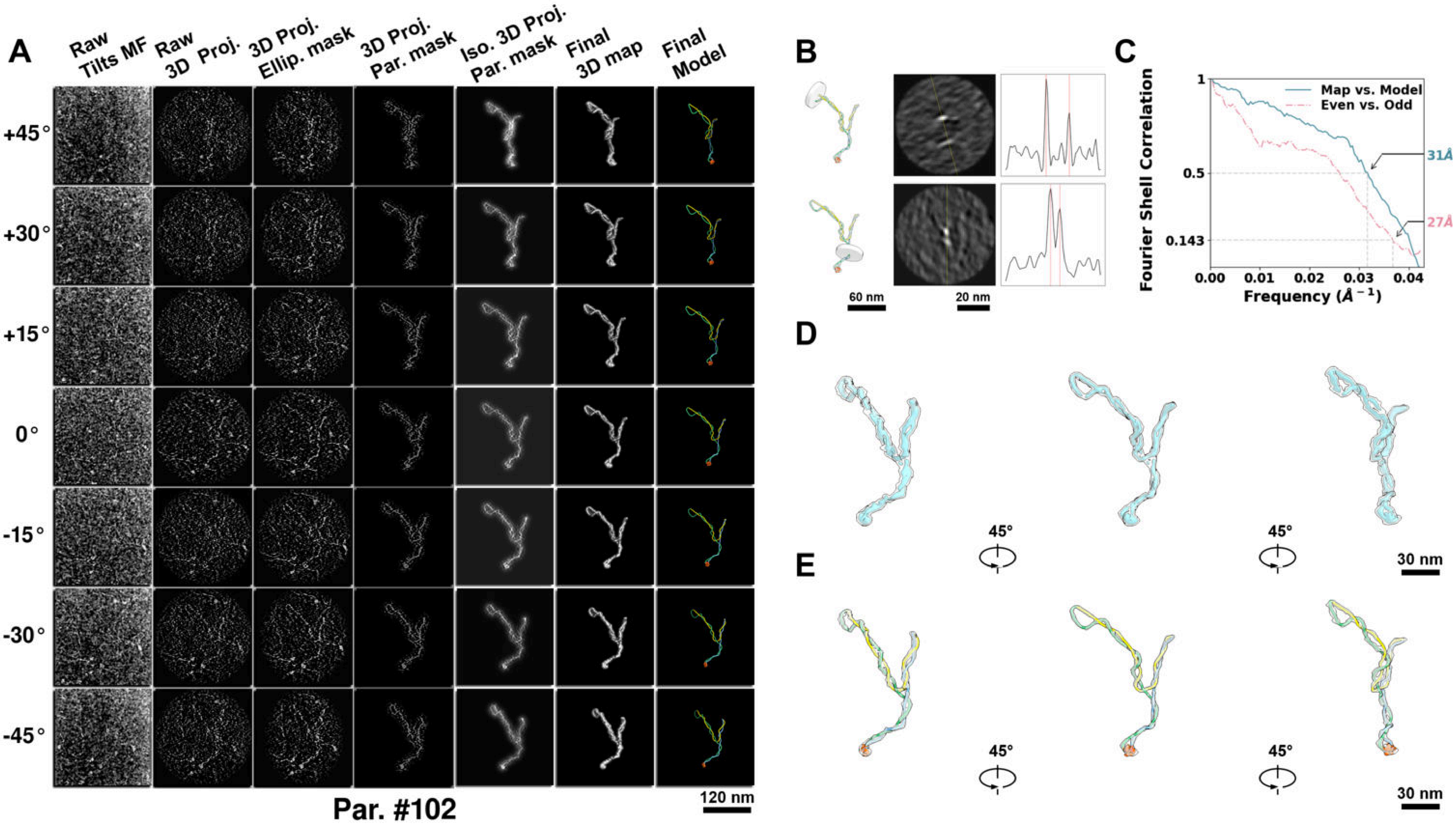

Supplementary Particle Figure 102. Cryo-ET 3D reconstruction of an individual P.Cas particle.
(A) 3D reconstruction of the plasmid particle (index no. 102). The first column shows seven representative tilt images from +45° to -45° in step of 15°. The second, third, and fourth columns show 3D projections of the particle with spherical, ellipsoidal (thinner along the z-dimension), and particle-shaped masks, respectively. The fifth column displays the 3D projections of the enhanced and IsoNet missing-wedge-corrected particle. The sixth and seventh columns present the final 3D map and the flexibly fitted model, respectively. (B) Two cross-sectional views (12 nm thickness) of the plasmid density map along its plectoneme axis are shown in the left-middle panel. The intensity profile along the line crossing the two high-density DNA spots is displayed in the right panel. (C) Resolution assessment of the final 3D map using Fourier shell correlation (FSC). Two criteria are shown: FSC between two half-maps reconstructed from even and odd frames (evaluated at 0.143) and FSC between the final 3D map and the fitted model (evaluated at 0.5). (D) Zoomed-in views of the final 3D density map from panel A, displayed at two contour levels. (E) Superimposition of the high-contour level map from panel D onto its fitted model.

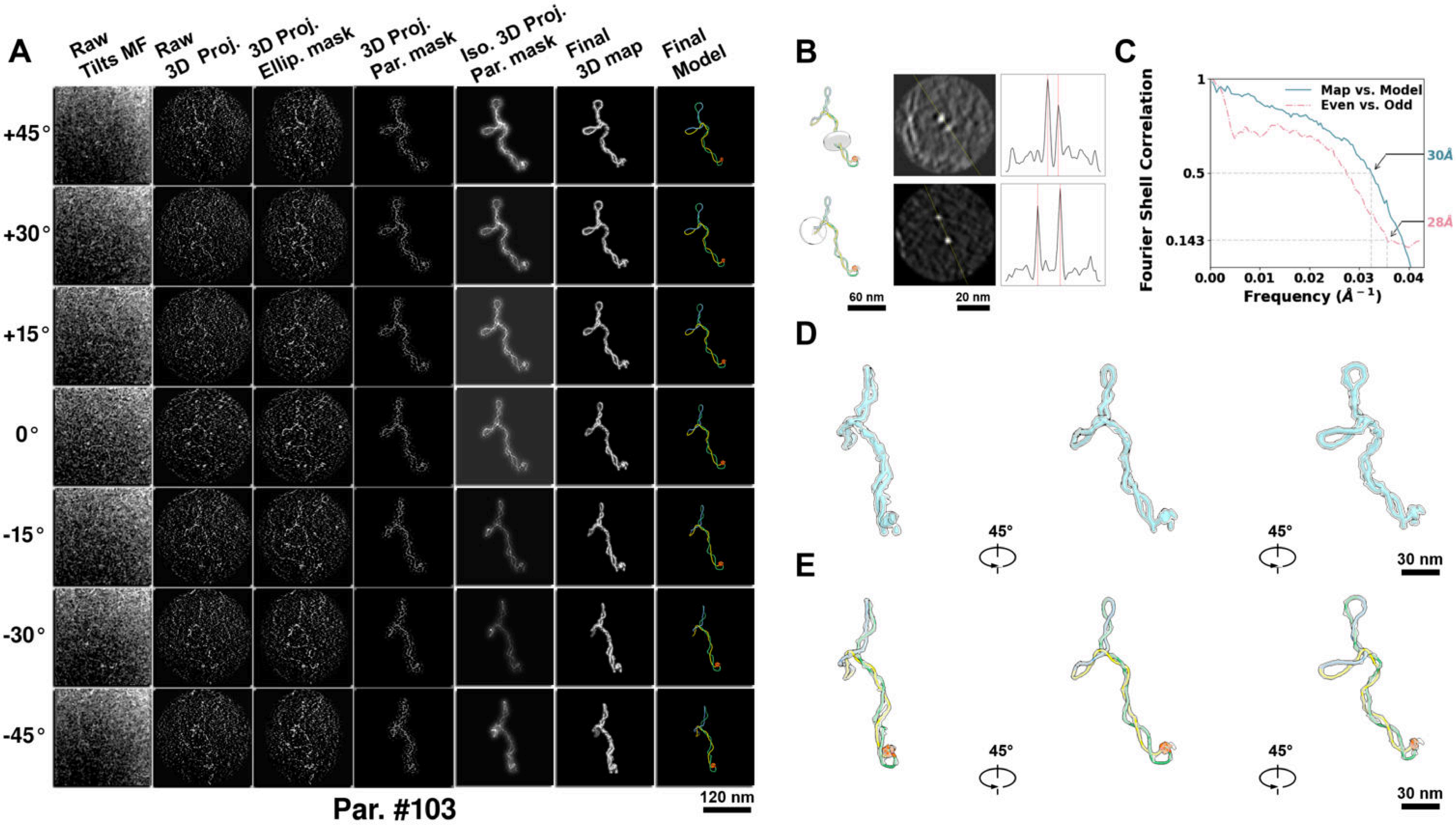

Supplementary Particle Figure 103. Cryo-ET 3D reconstruction of an individual P.Cas particle.
(A) 3D reconstruction of the plasmid particle (index no. 103). The first column shows seven representative tilt images from +45° to -45° in step of 15°. The second, third, and fourth columns show 3D projections of the particle with spherical, ellipsoidal (thinner along the z-dimension), and particle-shaped masks, respectively. The fifth column displays the 3D projections of the enhanced and IsoNet missing-wedge-corrected particle. The sixth and seventh columns present the final 3D map and the flexibly fitted model, respectively. (B) Two cross-sectional views (12 nm thickness) of the plasmid density map along its plectoneme axis are shown in the left-middle panel. The intensity profile along the line crossing the two high-density DNA spots is displayed in the right panel. (C) Resolution assessment of the final 3D map using Fourier shell correlation (FSC). Two criteria are shown: FSC between two half-maps reconstructed from even and odd frames (evaluated at 0.143) and FSC between the final 3D map and the fitted model (evaluated at 0.5). (D) Zoomed-in views of the final 3D density map from panel A, displayed at two contour levels. (E) Superimposition of the high-contour level map from panel D onto its fitted model.

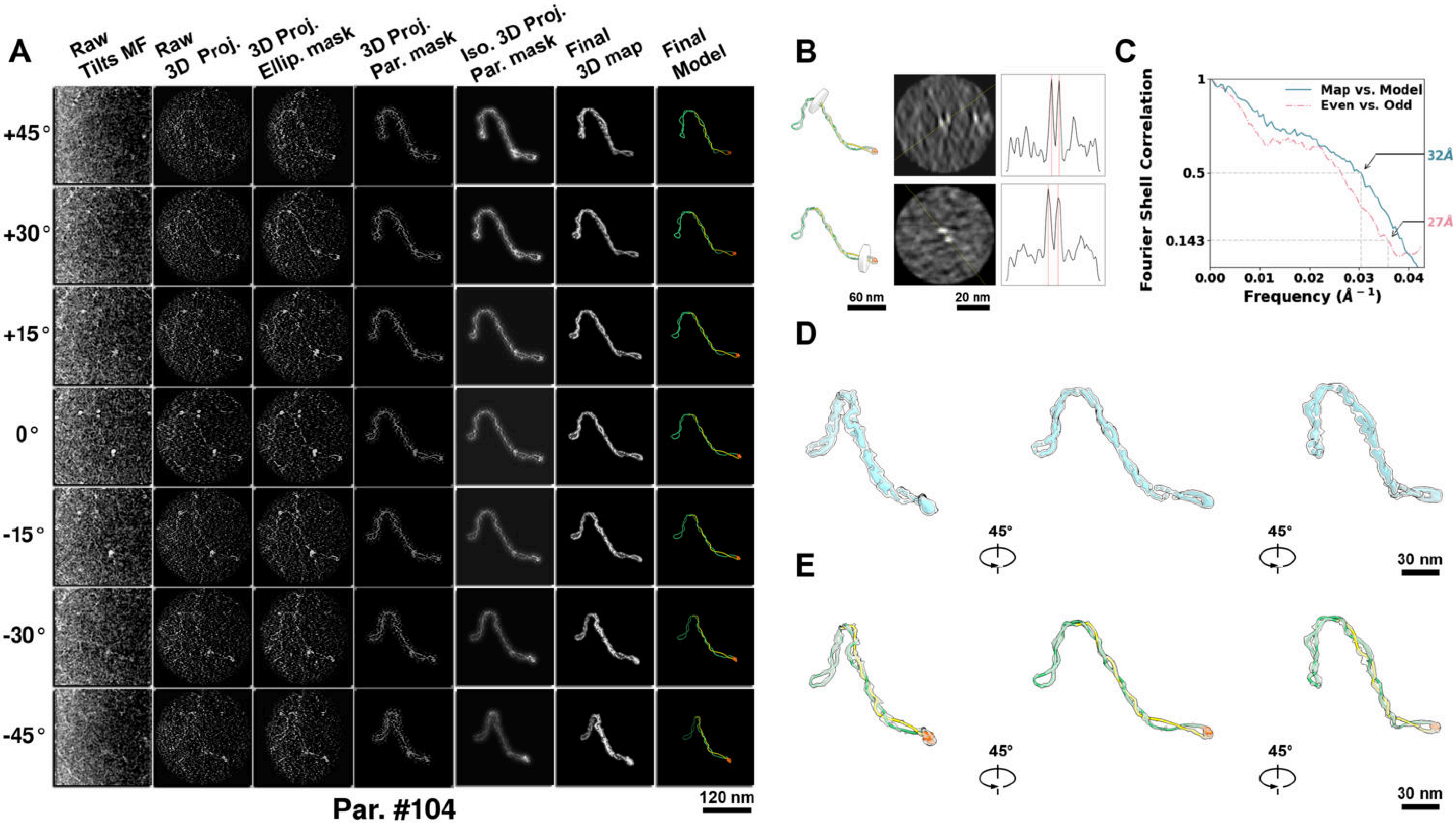

Supplementary Particle Figure 104. Cryo-ET 3D reconstruction of an individual P.Cas particle.
(A) 3D reconstruction of the plasmid particle (index no. 104). The first column shows seven representative tilt images from +45° to -45° in step of 15°. The second, third, and fourth columns show 3D projections of the particle with spherical, ellipsoidal (thinner along the z-dimension), and particle-shaped masks, respectively. The fifth column displays the 3D projections of the enhanced and IsoNet missing-wedge-corrected particle. The sixth and seventh columns present the final 3D map and the flexibly fitted model, respectively. (B) Two cross-sectional views (12 nm thickness) of the plasmid density map along its plectoneme axis are shown in the left-middle panel. The intensity profile along the line crossing the two high-density DNA spots is displayed in the right panel. (C) Resolution assessment of the final 3D map using Fourier shell correlation (FSC). Two criteria are shown: FSC between two half-maps reconstructed from even and odd frames (evaluated at 0.143) and FSC between the final 3D map and the fitted model (evaluated at 0.5). (D) Zoomed-in views of the final 3D density map from panel A, displayed at two contour levels. (E) Superimposition of the high-contour level map from panel D onto its fitted model.

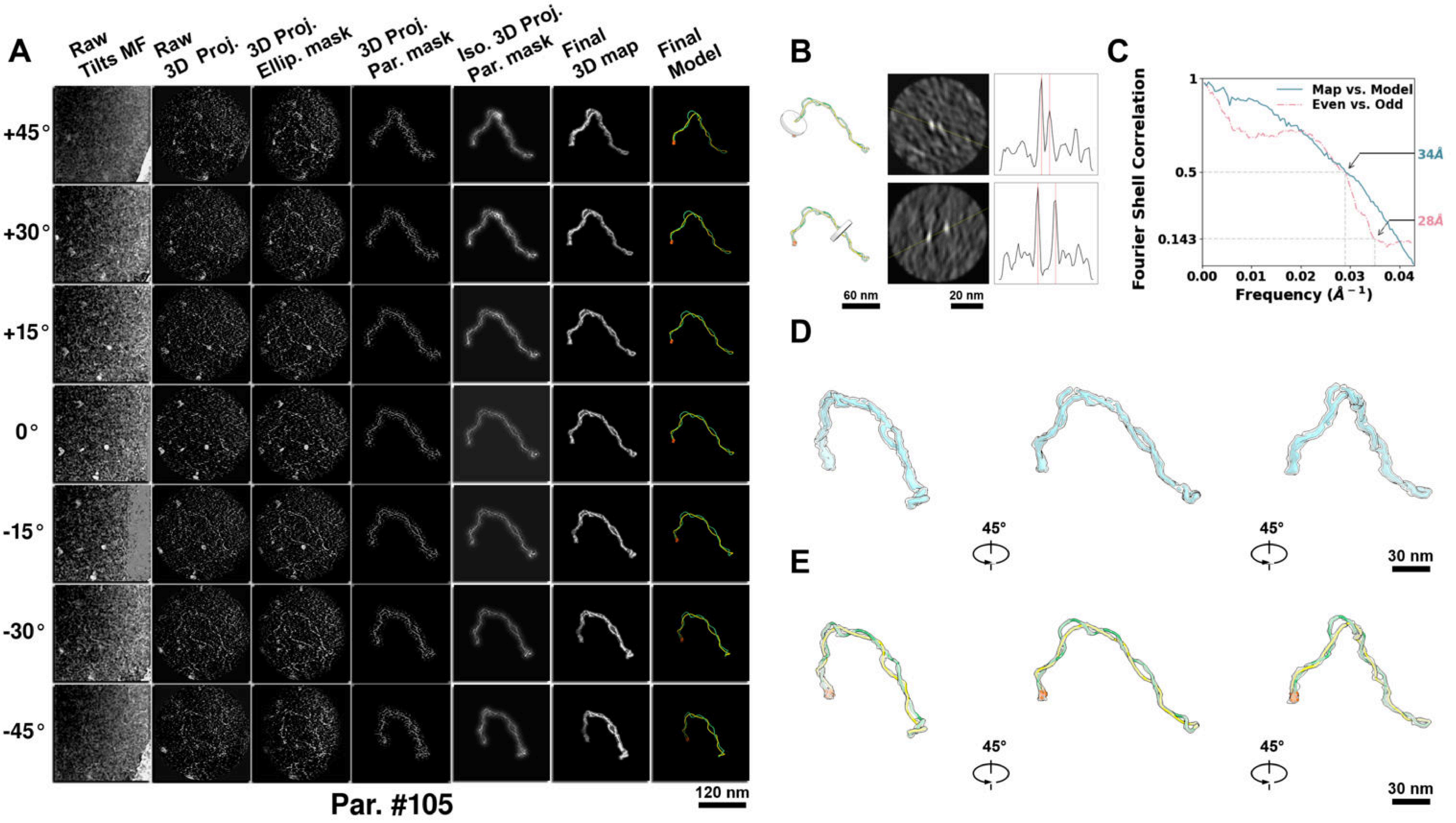

Supplementary Particle Figure 105. Cryo-ET 3D reconstruction of an individual P.Cas particle.
(A) 3D reconstruction of the plasmid particle (index no. 105). The first column shows seven representative tilt images from +45° to -45° in step of 15°. The second, third, and fourth columns show 3D projections of the particle with spherical, ellipsoidal (thinner along the z-dimension), and particle-shaped masks, respectively. The fifth column displays the 3D projections of the enhanced and IsoNet missing-wedge-corrected particle. The sixth and seventh columns present the final 3D map and the flexibly fitted model, respectively. (B) Two cross-sectional views (12 nm thickness) of the plasmid density map along its plectoneme axis are shown in the left-middle panel. The intensity profile along the line crossing the two high-density DNA spots is displayed in the right panel. (C) Resolution assessment of the final 3D map using Fourier shell correlation (FSC). Two criteria are shown: FSC between two half-maps reconstructed from even and odd frames (evaluated at 0.143) and FSC between the final 3D map and the fitted model (evaluated at 0.5). (D) Zoomed-in views of the final 3D density map from panel A, displayed at two contour levels. (E) Superimposition of the high-contour level map from panel D onto its fitted model.

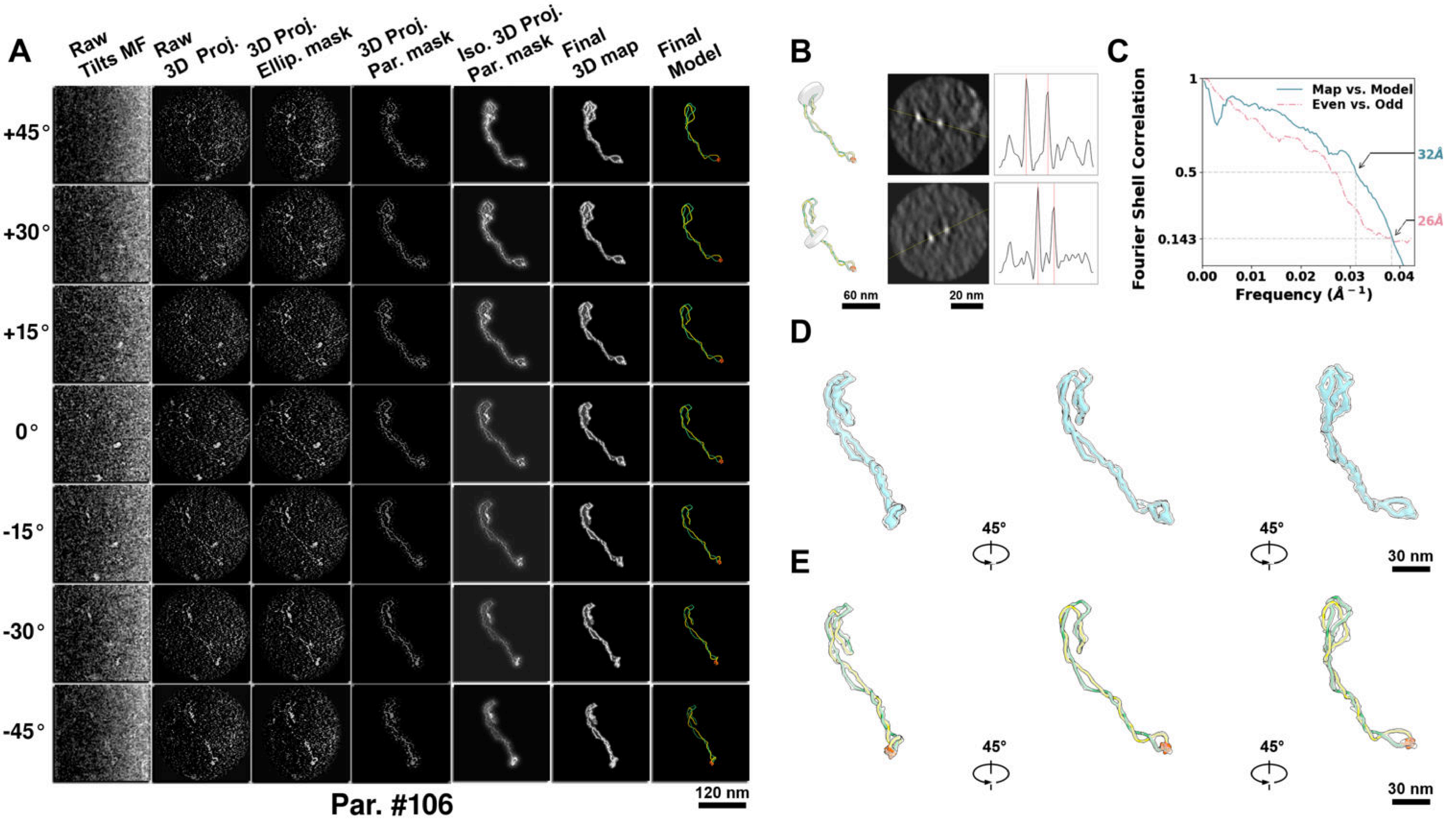

Supplementary Particle Figure 106. Cryo-ET 3D reconstruction of an individual P.Cas particle.
(A) 3D reconstruction of the plasmid particle (index no. 106). The first column shows seven representative tilt images from +45° to -45° in step of 15°. The second, third, and fourth columns show 3D projections of the particle with spherical, ellipsoidal (thinner along the z-dimension), and particle-shaped masks, respectively. The fifth column displays the 3D projections of the enhanced and IsoNet missing-wedge-corrected particle. The sixth and seventh columns present the final 3D map and the flexibly fitted model, respectively. (B) Two cross-sectional views (12 nm thickness) of the plasmid density map along its plectoneme axis are shown in the left-middle panel. The intensity profile along the line crossing the two high-density DNA spots is displayed in the right panel. (C) Resolution assessment of the final 3D map using Fourier shell correlation (FSC). Two criteria are shown: FSC between two half-maps reconstructed from even and odd frames (evaluated at 0.143) and FSC between the final 3D map and the fitted model (evaluated at 0.5). (D) Zoomed-in views of the final 3D density map from panel A, displayed at two contour levels. (E) Superimposition of the high-contour level map from panel D onto its fitted model.

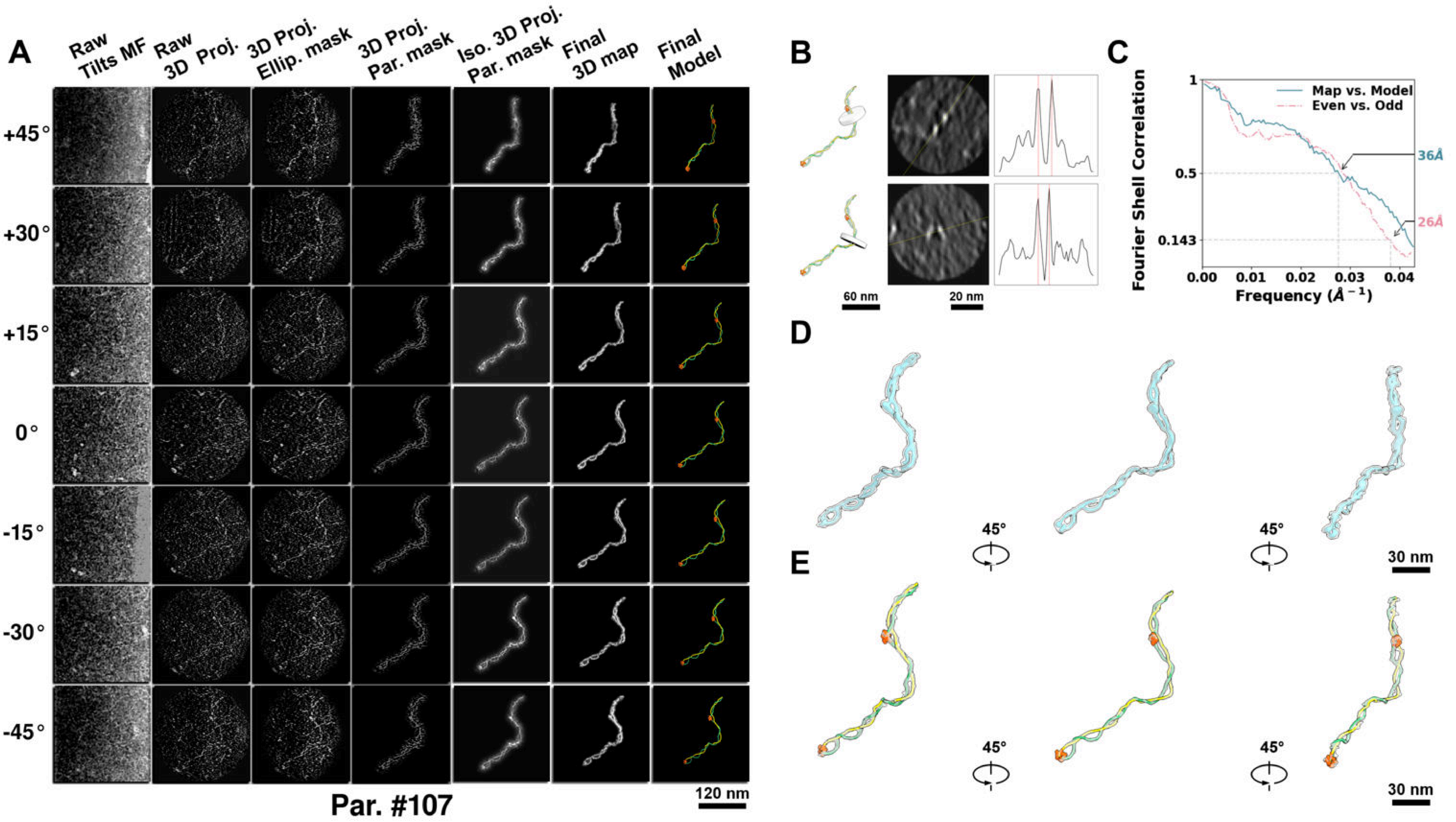

Supplementary Particle Figure 107. Cryo-ET 3D reconstruction of an individual P.Cas particle.
(A) 3D reconstruction of the plasmid particle (index no. 107). The first column shows seven representative tilt images from +45° to -45° in step of 15°. The second, third, and fourth columns show 3D projections of the particle with spherical, ellipsoidal (thinner along the z-dimension), and particle-shaped masks, respectively. The fifth column displays the 3D projections of the enhanced and IsoNet missing-wedge-corrected particle. The sixth and seventh columns present the final 3D map and the flexibly fitted model, respectively. (B) Two cross-sectional views (12 nm thickness) of the plasmid density map along its plectoneme axis are shown in the left-middle panel. The intensity profile along the line crossing the two high-density DNA spots is displayed in the right panel. (C) Resolution assessment of the final 3D map using Fourier shell correlation (FSC). Two criteria are shown: FSC between two half-maps reconstructed from even and odd frames (evaluated at 0.143) and FSC between the final 3D map and the fitted model (evaluated at 0.5). (D) Zoomed-in views of the final 3D density map from panel A, displayed at two contour levels. (E) Superimposition of the high-contour level map from panel D onto its fitted model.

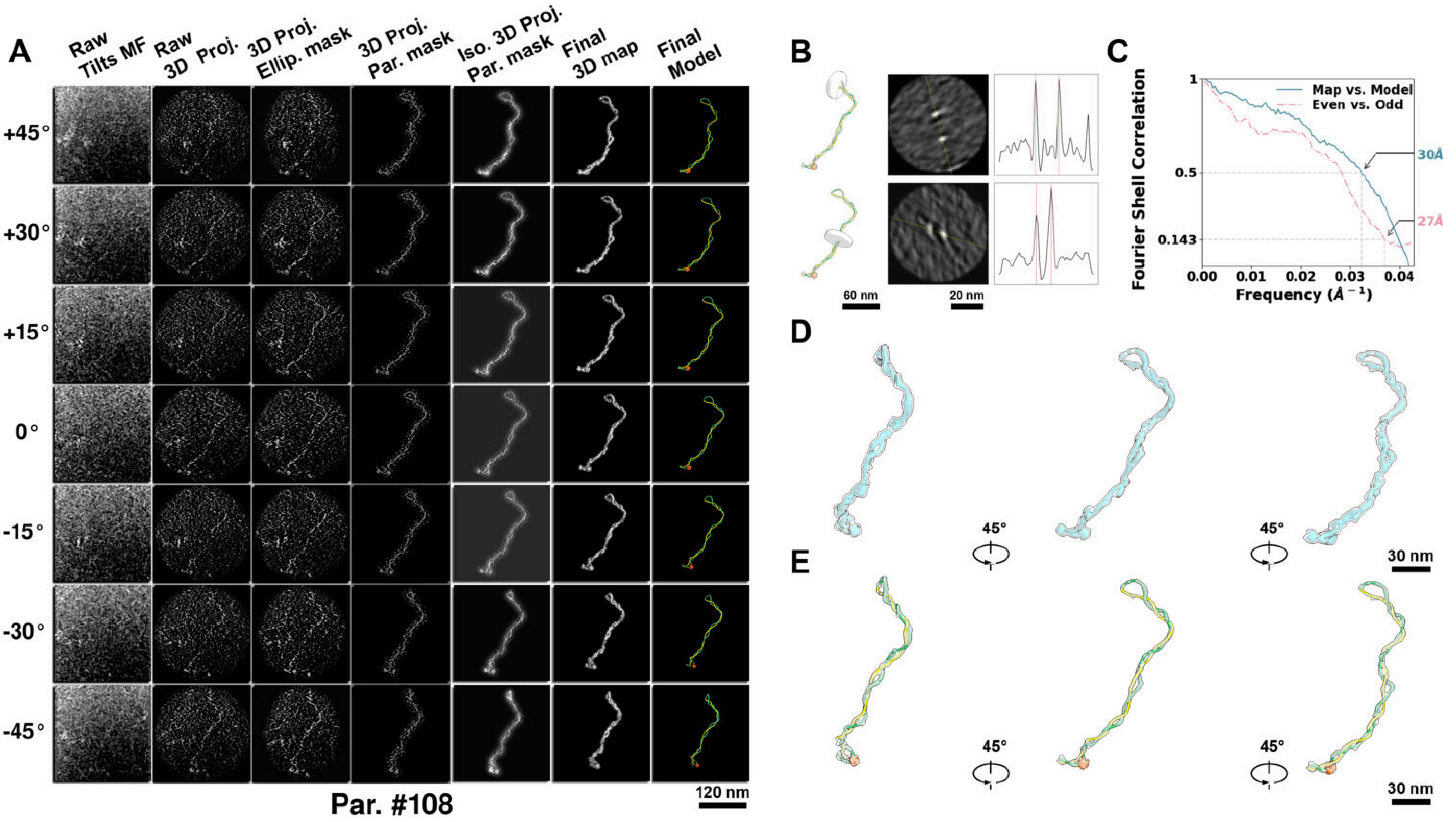

Supplementary Particle Figure 108. Cryo-ET 3D reconstruction of an individual P.Cas particle.
(A) 3D reconstruction of the plasmid particle (index no. 108). The first column shows seven representative tilt images from +45° to -45° in step of 15°. The second, third, and fourth columns show 3D projections of the particle with spherical, ellipsoidal (thinner along the z-dimension), and particle-shaped masks, respectively. The fifth column displays the 3D projections of the enhanced and IsoNet missing-wedge-corrected particle. The sixth and seventh columns present the final 3D map and the flexibly fitted model, respectively. (B) Two cross-sectional views (12 nm thickness) of the plasmid density map along its plectoneme axis are shown in the left-middle panel. The intensity profile along the line crossing the two high-density DNA spots is displayed in the right panel. (C) Resolution assessment of the final 3D map using Fourier shell correlation (FSC). Two criteria are shown: FSC between two half-maps reconstructed from even and odd frames (evaluated at 0.143) and FSC between the final 3D map and the fitted model (evaluated at 0.5). (D) Zoomed-in views of the final 3D density map from panel A, displayed at two contour levels. (E) Superimposition of the high-contour level map from panel D onto its fitted model.

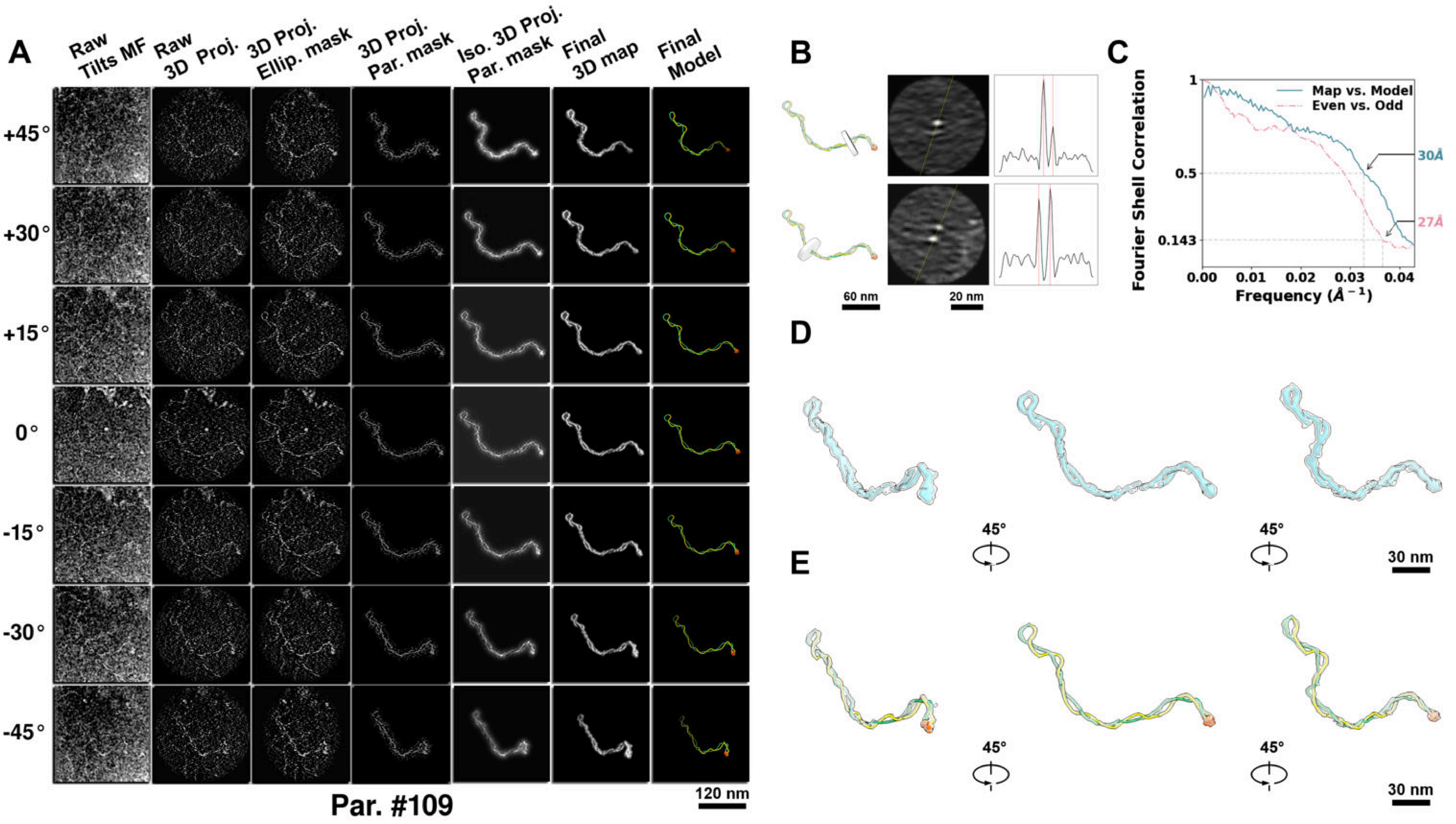

Supplementary Particle Figure 109. Cryo-ET 3D reconstruction of an individual P.Cas particle.
(A) 3D reconstruction of the plasmid particle (index no. 109). The first column shows seven representative tilt images from +45° to -45° in step of 15°. The second, third, and fourth columns show 3D projections of the particle with spherical, ellipsoidal (thinner along the z-dimension), and particle-shaped masks, respectively. The fifth column displays the 3D projections of the enhanced and IsoNet missing-wedge-corrected particle. The sixth and seventh columns present the final 3D map and the flexibly fitted model, respectively. (B) Two cross-sectional views (12 nm thickness) of the plasmid density map along its plectoneme axis are shown in the left-middle panel. The intensity profile along the line crossing the two high-density DNA spots is displayed in the right panel. (C) Resolution assessment of the final 3D map using Fourier shell correlation (FSC). Two criteria are shown: FSC between two half-maps reconstructed from even and odd frames (evaluated at 0.143) and FSC between the final 3D map and the fitted model (evaluated at 0.5). (D) Zoomed-in views of the final 3D density map from panel A, displayed at two contour levels. (E) Superimposition of the high-contour level map from panel D onto its fitted model.

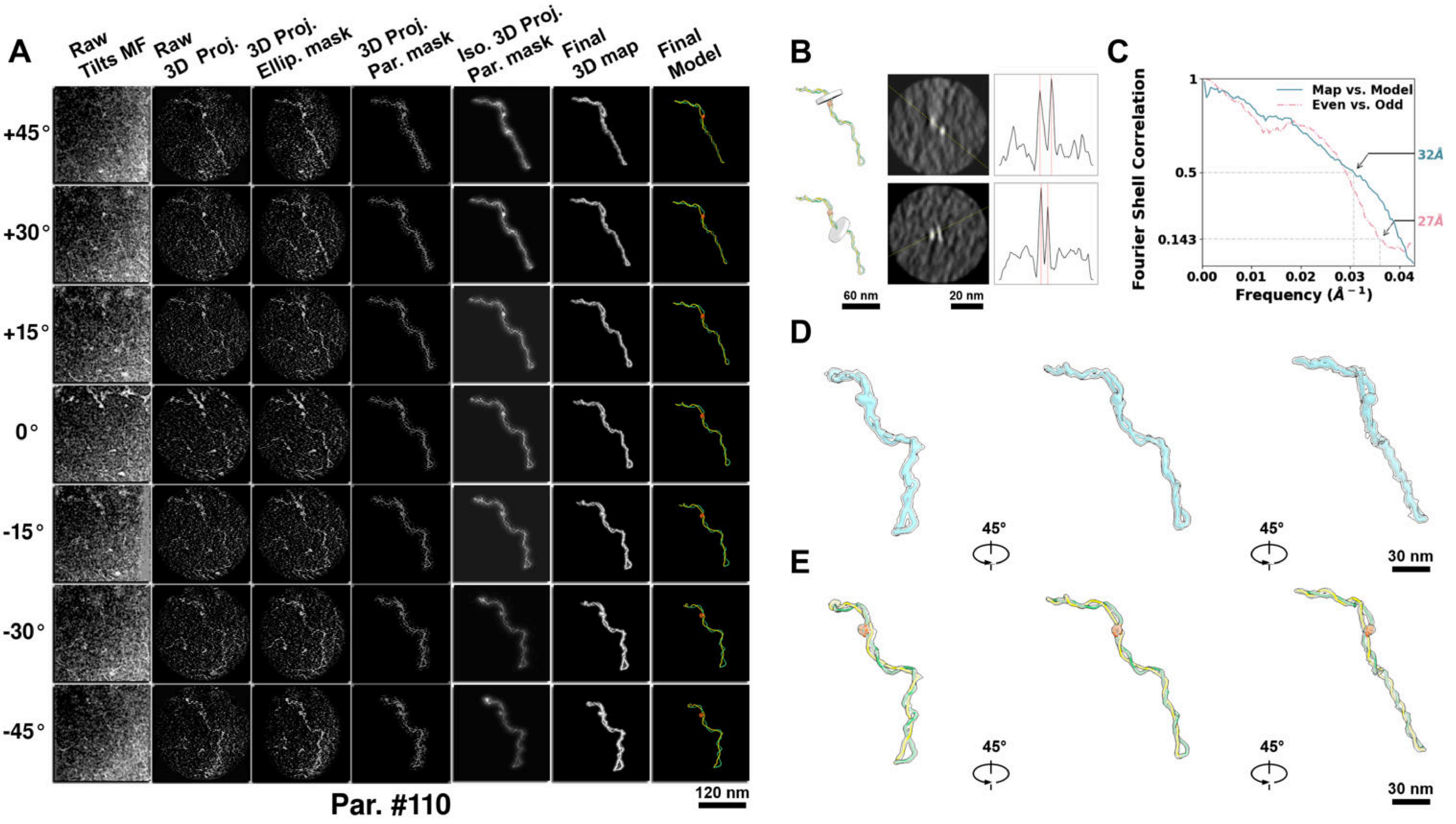

Supplementary Particle Figure 110. Cryo-ET 3D reconstruction of an individual P.Cas particle.
(A) 3D reconstruction of the plasmid particle (index no. 110). The first column shows seven representative tilt images from +45° to -45° in step of 15°. The second, third, and fourth columns show 3D projections of the particle with spherical, ellipsoidal (thinner along the z-dimension), and particle-shaped masks, respectively. The fifth column displays the 3D projections of the enhanced and IsoNet missing-wedge-corrected particle. The sixth and seventh columns present the final 3D map and the flexibly fitted model, respectively. (B) Two cross-sectional views (12 nm thickness) of the plasmid density map along its plectoneme axis are shown in the left-middle panel. The intensity profile along the line crossing the two high-density DNA spots is displayed in the right panel. (C) Resolution assessment of the final 3D map using Fourier shell correlation (FSC). Two criteria are shown: FSC between two half-maps reconstructed from even and odd frames (evaluated at 0.143) and FSC between the final 3D map and the fitted model (evaluated at 0.5). (D) Zoomed-in views of the final 3D density map from panel A, displayed at two contour levels. (E) Superimposition of the high-contour level map from panel D onto its fitted model.

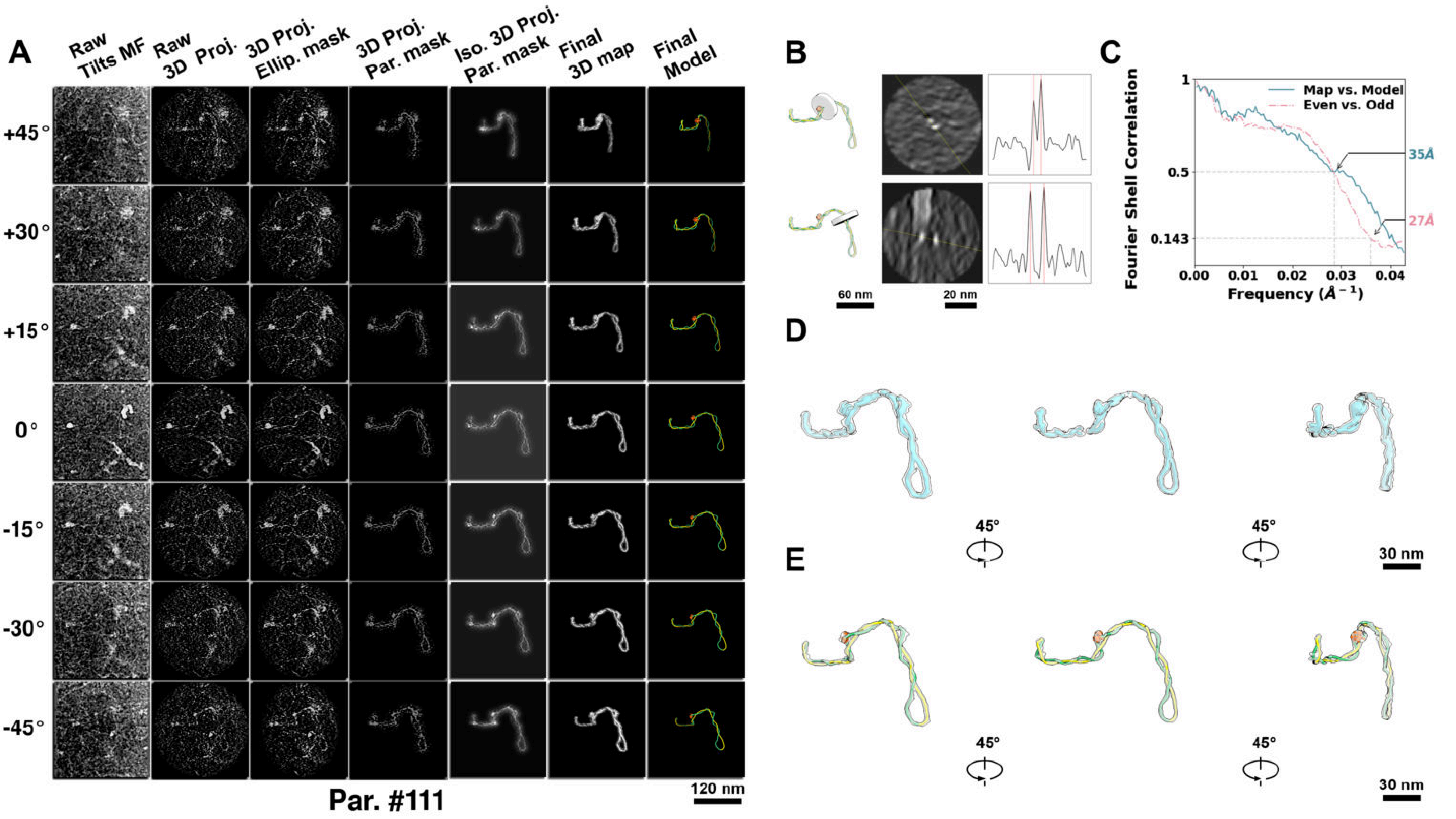

Supplementary Particle Figure 111. Cryo-ET 3D reconstruction of an individual P.Cas particle.
(A) 3D reconstruction of the plasmid particle (index no. 111). The first column shows seven representative tilt images from +45° to -45° in step of 15°. The second, third, and fourth columns show 3D projections of the particle with spherical, ellipsoidal (thinner along the z-dimension), and particle-shaped masks, respectively. The fifth column displays the 3D projections of the enhanced and IsoNet missing-wedge-corrected particle. The sixth and seventh columns present the final 3D map and the flexibly fitted model, respectively. (B) Two cross-sectional views (12 nm thickness) of the plasmid density map along its plectoneme axis are shown in the left-middle panel. The intensity profile along the line crossing the two high-density DNA spots is displayed in the right panel. (C) Resolution assessment of the final 3D map using Fourier shell correlation (FSC). Two criteria are shown: FSC between two half-maps reconstructed from even and odd frames (evaluated at 0.143) and FSC between the final 3D map and the fitted model (evaluated at 0.5). (D) Zoomed-in views of the final 3D density map from panel A, displayed at two contour levels. (E) Superimposition of the high-contour level map from panel D onto its fitted model.

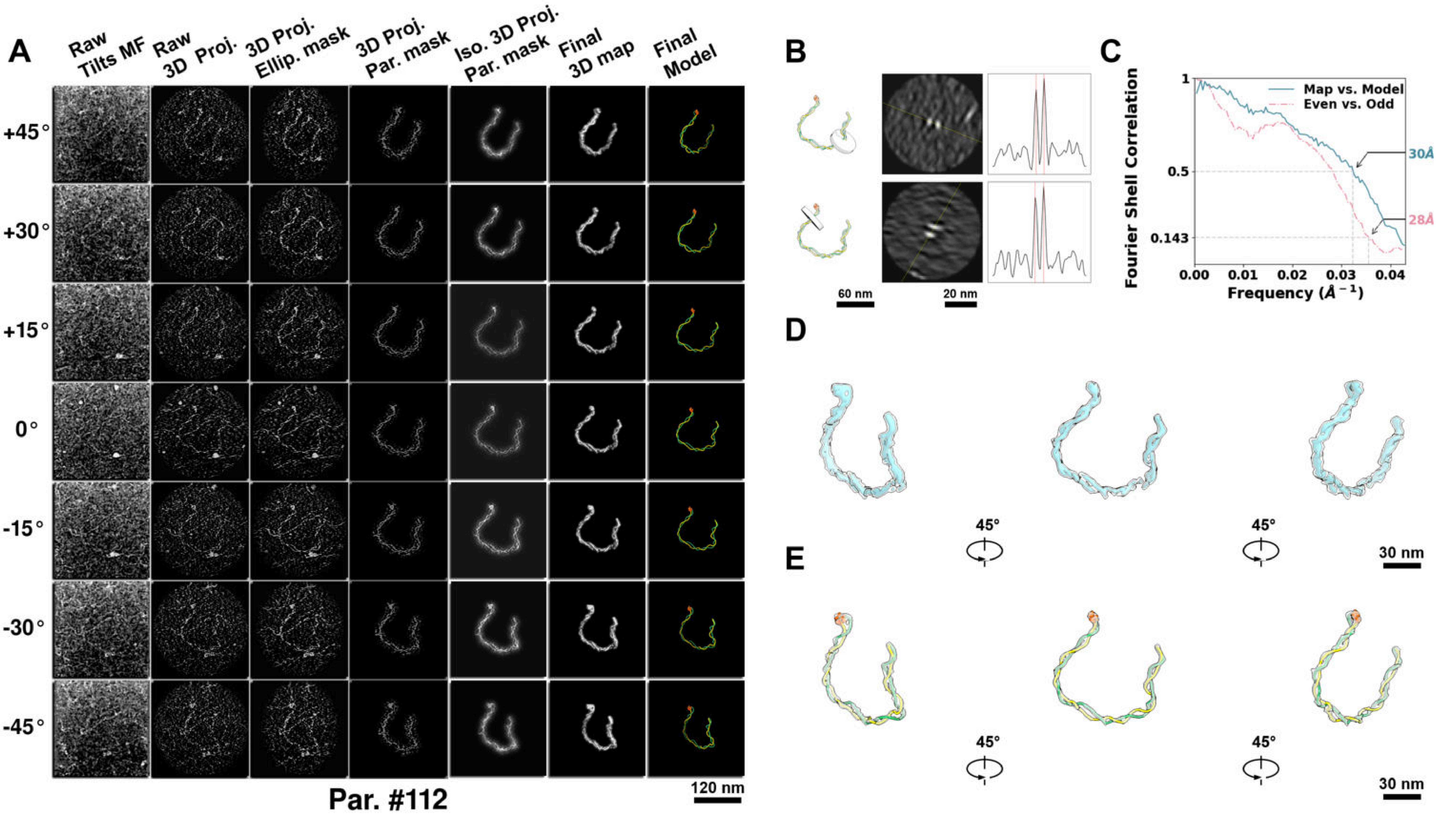

Supplementary Particle Figure 112. Cryo-ET 3D reconstruction of an individual P.Cas particle.
(A) 3D reconstruction of the plasmid particle (index no. 112). The first column shows seven representative tilt images from +45° to -45° in step of 15°. The second, third, and fourth columns show 3D projections of the particle with spherical, ellipsoidal (thinner along the z-dimension), and particle-shaped masks, respectively. The fifth column displays the 3D projections of the enhanced and IsoNet missing-wedge-corrected particle. The sixth and seventh columns present the final 3D map and the flexibly fitted model, respectively. (B) Two cross-sectional views (12 nm thickness) of the plasmid density map along its plectoneme axis are shown in the left-middle panel. The intensity profile along the line crossing the two high-density DNA spots is displayed in the right panel. (C) Resolution assessment of the final 3D map using Fourier shell correlation (FSC). Two criteria are shown: FSC between two half-maps reconstructed from even and odd frames (evaluated at 0.143) and FSC between the final 3D map and the fitted model (evaluated at 0.5). (D) Zoomed-in views of the final 3D density map from panel A, displayed at two contour levels. (E) Superimposition of the high-contour level map from panel D onto its fitted model.

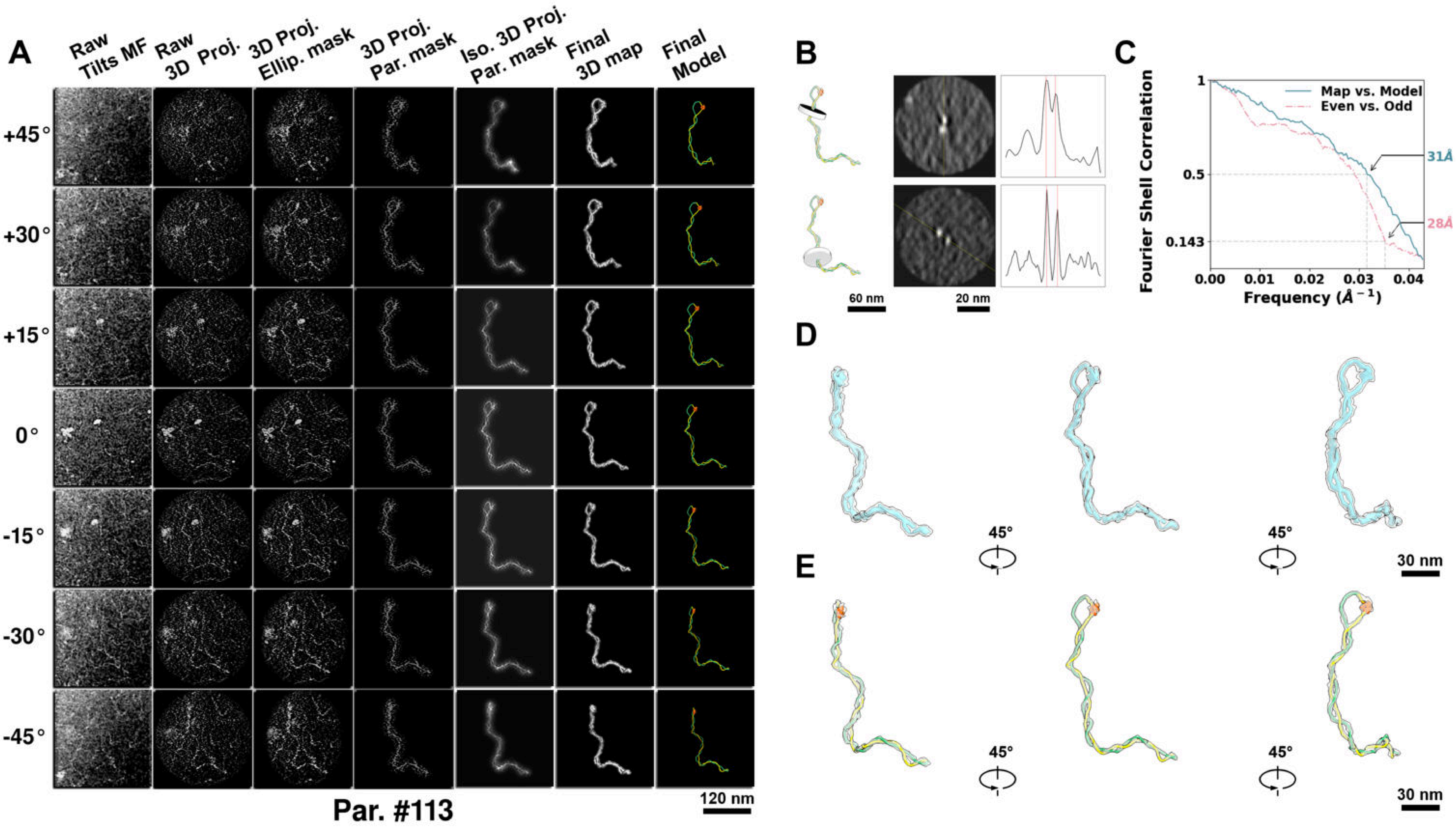

Supplementary Particle Figure 113. Cryo-ET 3D reconstruction of an individual P.Cas particle.
(A) 3D reconstruction of the plasmid particle (index no. 113). The first column shows seven representative tilt images from +45° to -45° in step of 15°. The second, third, and fourth columns show 3D projections of the particle with spherical, ellipsoidal (thinner along the z-dimension), and particle-shaped masks, respectively. The fifth column displays the 3D projections of the enhanced and IsoNet missing-wedge-corrected particle. The sixth and seventh columns present the final 3D map and the flexibly fitted model, respectively. (B) Two cross-sectional views (12 nm thickness) of the plasmid density map along its plectoneme axis are shown in the left-middle panel. The intensity profile along the line crossing the two high-density DNA spots is displayed in the right panel. (C) Resolution assessment of the final 3D map using Fourier shell correlation (FSC). Two criteria are shown: FSC between two half-maps reconstructed from even and odd frames (evaluated at 0.143) and FSC between the final 3D map and the fitted model (evaluated at 0.5). (D) Zoomed-in views of the final 3D density map from panel A, displayed at two contour levels. (E) Superimposition of the high-contour level map from panel D onto its fitted model.

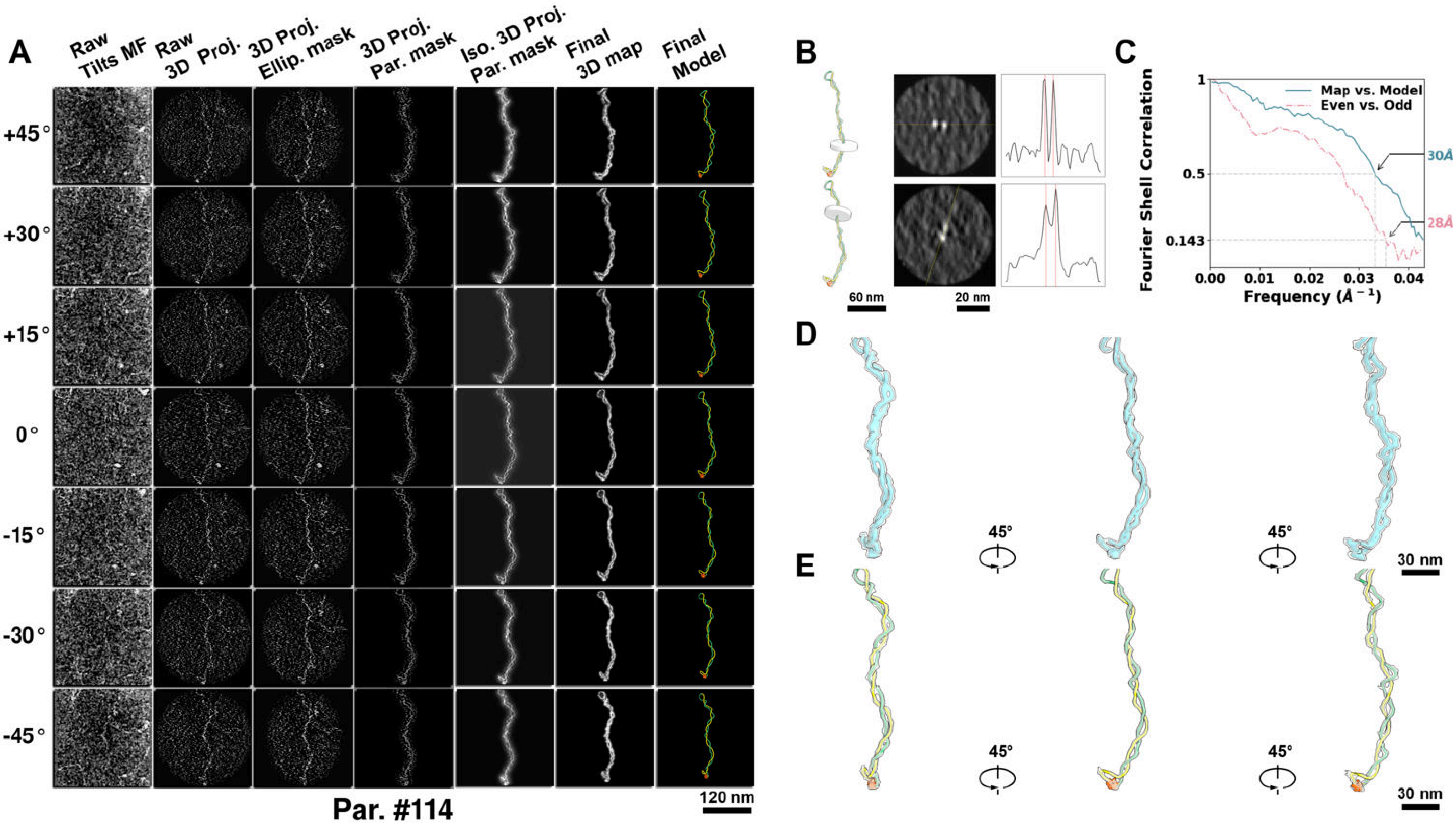

Supplementary Particle Figure 114. Cryo-ET 3D reconstruction of an individual P.Cas particle.
(A) 3D reconstruction of the plasmid particle (index no. 114). The first column shows seven representative tilt images from +45° to -45° in step of 15°. The second, third, and fourth columns show 3D projections of the particle with spherical, ellipsoidal (thinner along the z-dimension), and particle-shaped masks, respectively. The fifth column displays the 3D projections of the enhanced and IsoNet missing-wedge-corrected particle. The sixth and seventh columns present the final 3D map and the flexibly fitted model, respectively. (B) Two cross-sectional views (12 nm thickness) of the plasmid density map along its plectoneme axis are shown in the left-middle panel. The intensity profile along the line crossing the two high-density DNA spots is displayed in the right panel. (C) Resolution assessment of the final 3D map using Fourier shell correlation (FSC). Two criteria are shown: FSC between two half-maps reconstructed from even and odd frames (evaluated at 0.143) and FSC between the final 3D map and the fitted model (evaluated at 0.5). (D) Zoomed-in views of the final 3D density map from panel A, displayed at two contour levels. (E) Superimposition of the high-contour level map from panel D onto its fitted model.

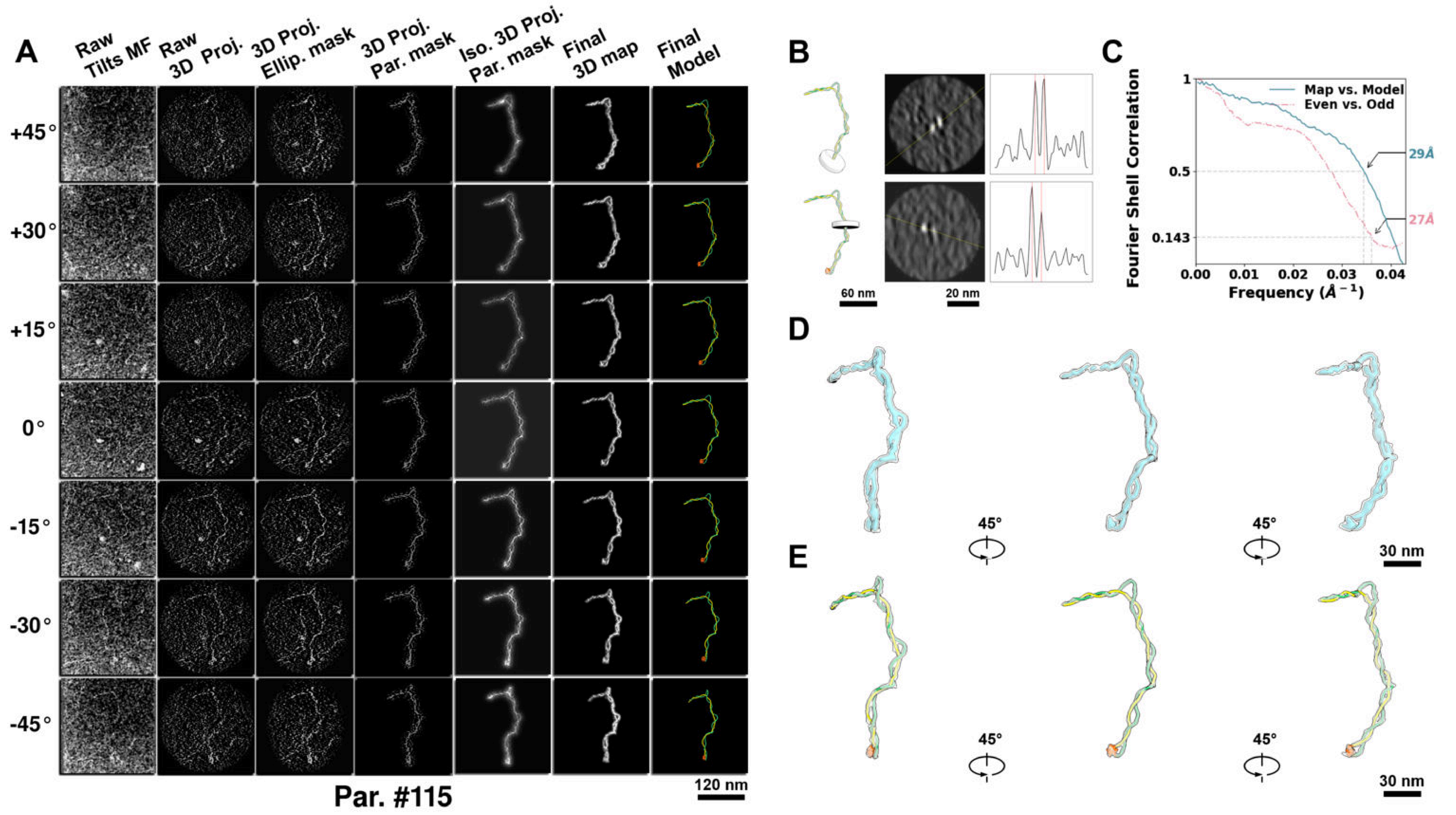

Supplementary Particle Figure 115. Cryo-ET 3D reconstruction of an individual P.Cas particle.
(A) 3D reconstruction of the plasmid particle (index no. 115). The first column shows seven representative tilt images from +45° to -45° in step of 15°. The second, third, and fourth columns show 3D projections of the particle with spherical, ellipsoidal (thinner along the z-dimension), and particle-shaped masks, respectively. The fifth column displays the 3D projections of the enhanced and IsoNet missing-wedge-corrected particle. The sixth and seventh columns present the final 3D map and the flexibly fitted model, respectively. (B) Two cross-sectional views (12 nm thickness) of the plasmid density map along its plectoneme axis are shown in the left-middle panel. The intensity profile along the line crossing the two high-density DNA spots is displayed in the right panel. (C) Resolution assessment of the final 3D map using Fourier shell correlation (FSC). Two criteria are shown: FSC between two half-maps reconstructed from even and odd frames (evaluated at 0.143) and FSC between the final 3D map and the fitted model (evaluated at 0.5). (D) Zoomed-in views of the final 3D density map from panel A, displayed at two contour levels. (E) Superimposition of the high-contour level map from panel D onto its fitted model.

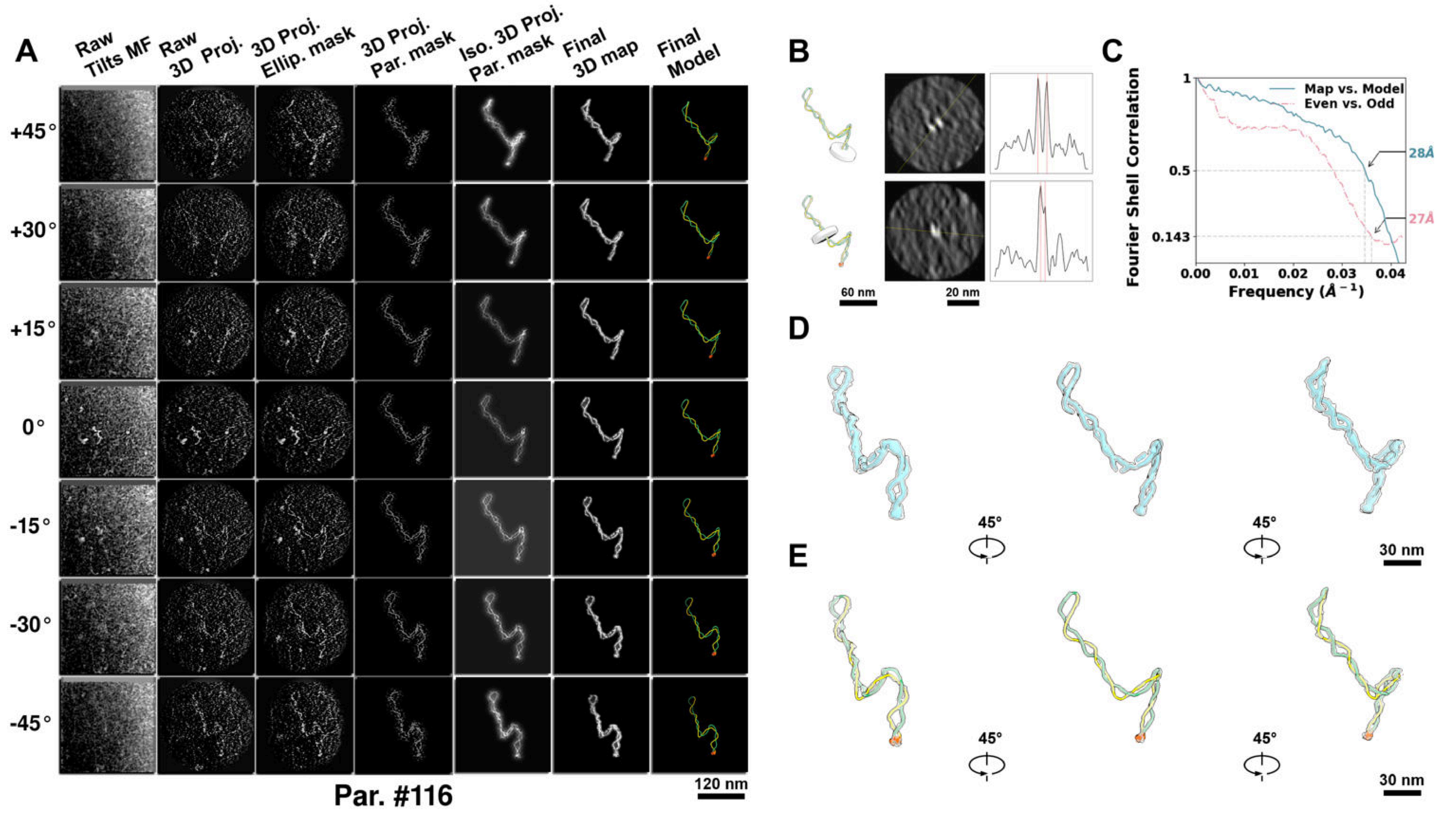

Supplementary Particle Figure 116. Cryo-ET 3D reconstruction of an individual P.Cas particle.
(A) 3D reconstruction of the plasmid particle (index no. 116). The first column shows seven representative tilt images from +45° to -45° in step of 15°. The second, third, and fourth columns show 3D projections of the particle with spherical, ellipsoidal (thinner along the z-dimension), and particle-shaped masks, respectively. The fifth column displays the 3D projections of the enhanced and IsoNet missing-wedge-corrected particle. The sixth and seventh columns present the final 3D map and the flexibly fitted model, respectively. (B) Two cross-sectional views (12 nm thickness) of the plasmid density map along its plectoneme axis are shown in the left-middle panel. The intensity profile along the line crossing the two high-density DNA spots is displayed in the right panel. (C) Resolution assessment of the final 3D map using Fourier shell correlation (FSC). Two criteria are shown: FSC between two half-maps reconstructed from even and odd frames (evaluated at 0.143) and FSC between the final 3D map and the fitted model (evaluated at 0.5). (D) Zoomed-in views of the final 3D density map from panel A, displayed at two contour levels. (E) Superimposition of the high-contour level map from panel D onto its fitted model.

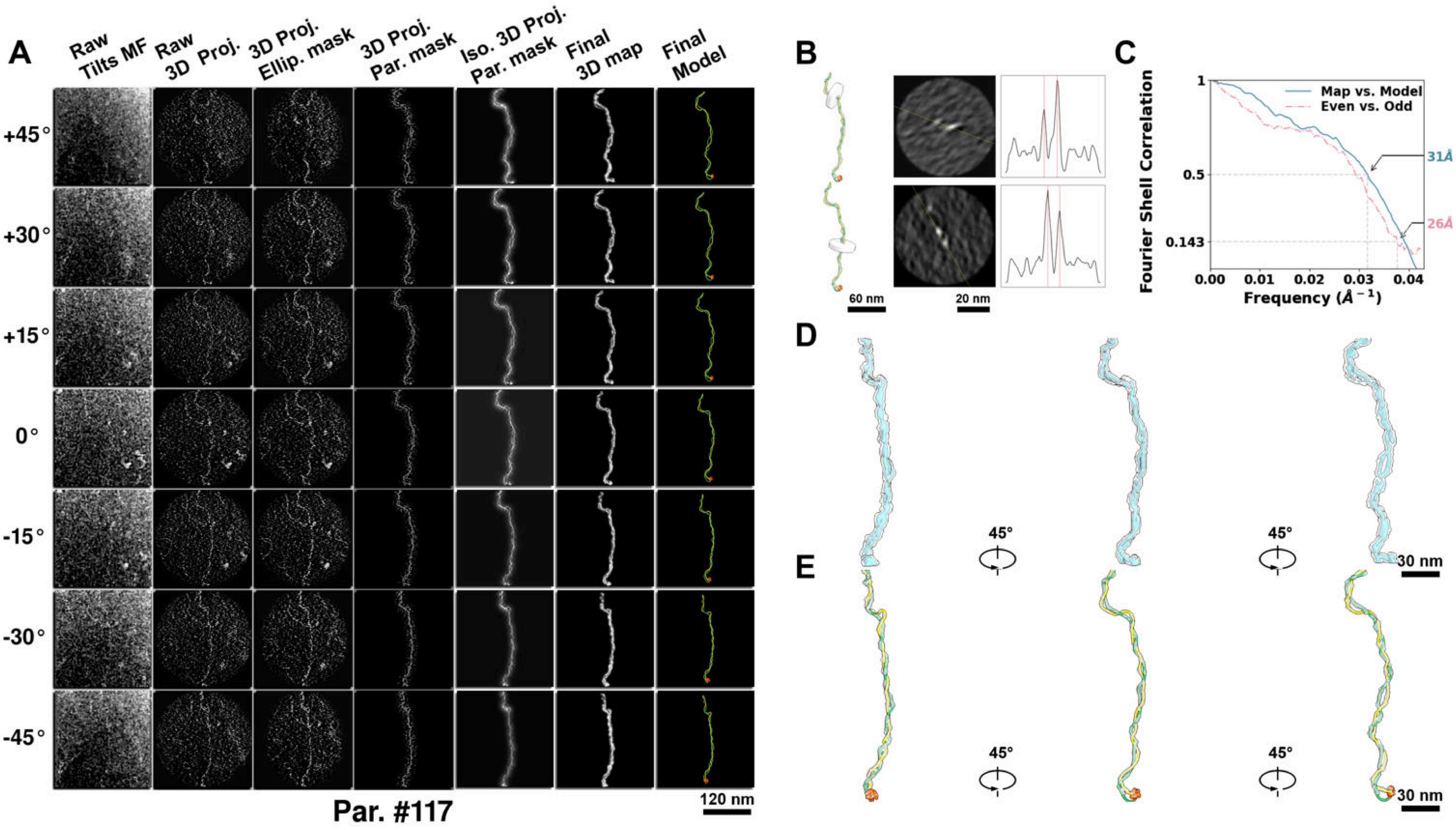

Supplementary Particle Figure 117. Cryo-ET 3D reconstruction of an individual P.Cas particle.
(A) 3D reconstruction of the plasmid particle (index no. 117). The first column shows seven representative tilt images from +45° to -45° in step of 15°. The second, third, and fourth columns show 3D projections of the particle with spherical, ellipsoidal (thinner along the z-dimension), and particle-shaped masks, respectively. The fifth column displays the 3D projections of the enhanced and IsoNet missing-wedge-corrected particle. The sixth and seventh columns present the final 3D map and the flexibly fitted model, respectively. (B) Two cross-sectional views (12 nm thickness) of the plasmid density map along its plectoneme axis are shown in the left-middle panel. The intensity profile along the line crossing the two high-density DNA spots is displayed in the right panel. (C) Resolution assessment of the final 3D map using Fourier shell correlation (FSC). Two criteria are shown: FSC between two half-maps reconstructed from even and odd frames (evaluated at 0.143) and FSC between the final 3D map and the fitted model (evaluated at 0.5). (D) Zoomed-in views of the final 3D density map from panel A, displayed at two contour levels. (E) Superimposition of the high-contour level map from panel D onto its fitted model.

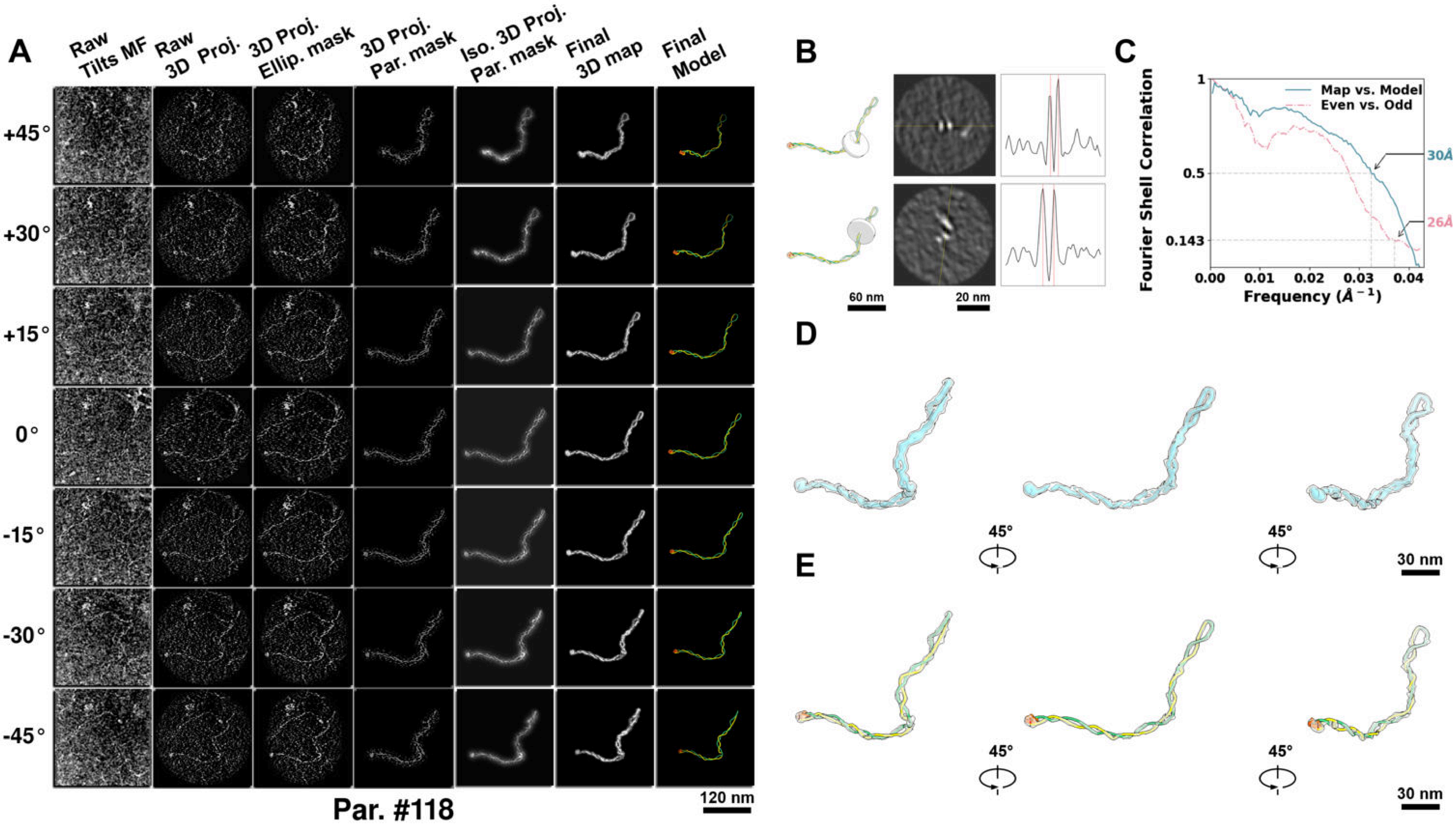

Supplementary Particle Figure 118. Cryo-ET 3D reconstruction of an individual P.Cas particle.
(A) 3D reconstruction of the plasmid particle (index no. 118). The first column shows seven representative tilt images from +45° to -45° in step of 15°. The second, third, and fourth columns show 3D projections of the particle with spherical, ellipsoidal (thinner along the z-dimension), and particle-shaped masks, respectively. The fifth column displays the 3D projections of the enhanced and IsoNet missing-wedge-corrected particle. The sixth and seventh columns present the final 3D map and the flexibly fitted model, respectively. (B) Two cross-sectional views (12 nm thickness) of the plasmid density map along its plectoneme axis are shown in the left-middle panel. The intensity profile along the line crossing the two high-density DNA spots is displayed in the right panel. (C) Resolution assessment of the final 3D map using Fourier shell correlation (FSC). Two criteria are shown: FSC between two half-maps reconstructed from even and odd frames (evaluated at 0.143) and FSC between the final 3D map and the fitted model (evaluated at 0.5). (D) Zoomed-in views of the final 3D density map from panel A, displayed at two contour levels. (E) Superimposition of the high-contour level map from panel D onto its fitted model.

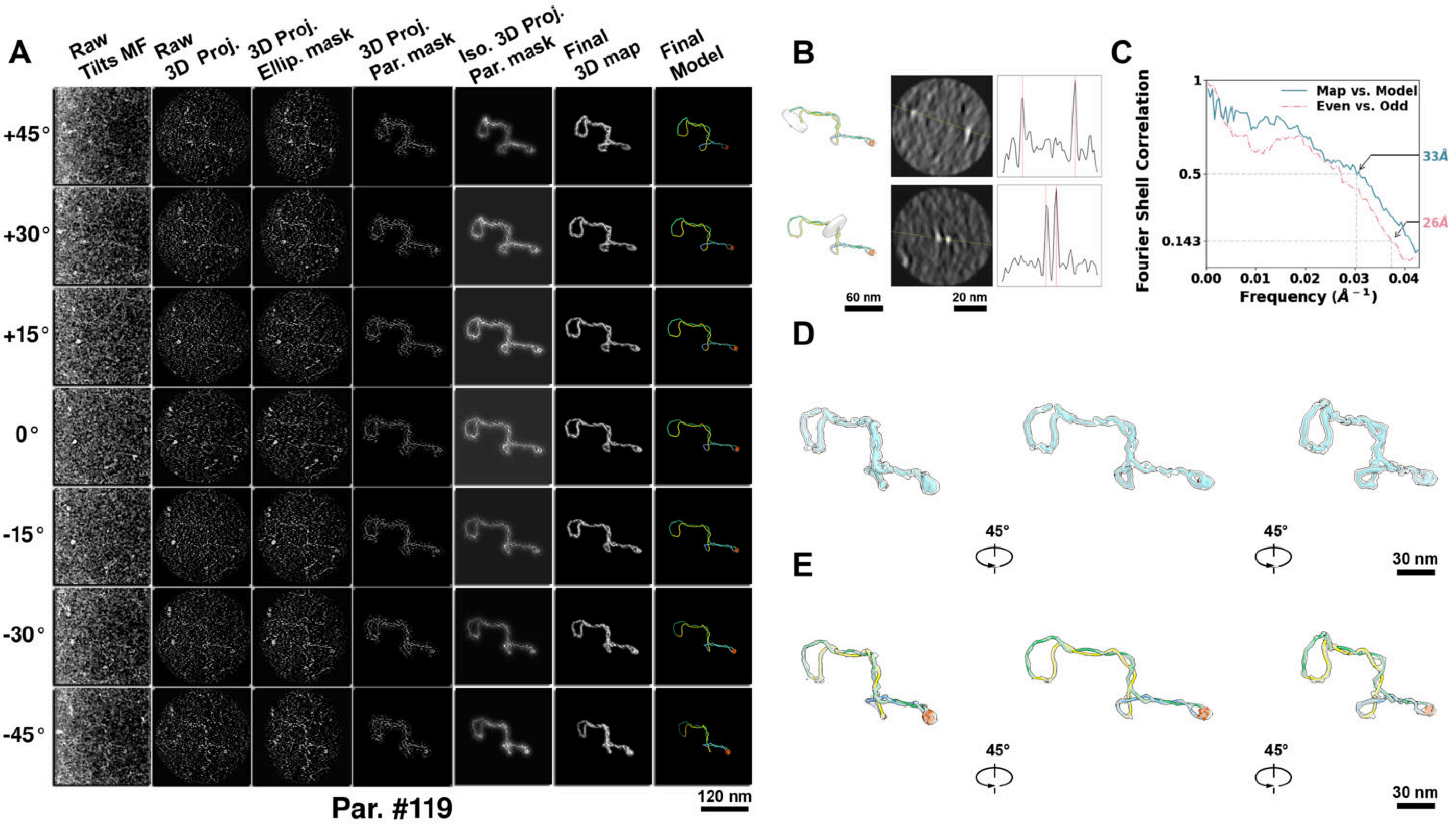

Supplementary Particle Figure 119. Cryo-ET 3D reconstruction of an individual P.Cas particle.
(A) 3D reconstruction of the plasmid particle (index no. 119). The first column shows seven representative tilt images from +45° to -45° in step of 15°. The second, third, and fourth columns show 3D projections of the particle with spherical, ellipsoidal (thinner along the z-dimension), and particle-shaped masks, respectively. The fifth column displays the 3D projections of the enhanced and IsoNet missing-wedge-corrected particle. The sixth and seventh columns present the final 3D map and the flexibly fitted model, respectively. (B) Two cross-sectional views (12 nm thickness) of the plasmid density map along its plectoneme axis are shown in the left-middle panel. The intensity profile along the line crossing the two high-density DNA spots is displayed in the right panel. (C) Resolution assessment of the final 3D map using Fourier shell correlation (FSC). Two criteria are shown: FSC between two half-maps reconstructed from even and odd frames (evaluated at 0.143) and FSC between the final 3D map and the fitted model (evaluated at 0.5). (D) Zoomed-in views of the final 3D density map from panel A, displayed at two contour levels. (E) Superimposition of the high-contour level map from panel D onto its fitted model.

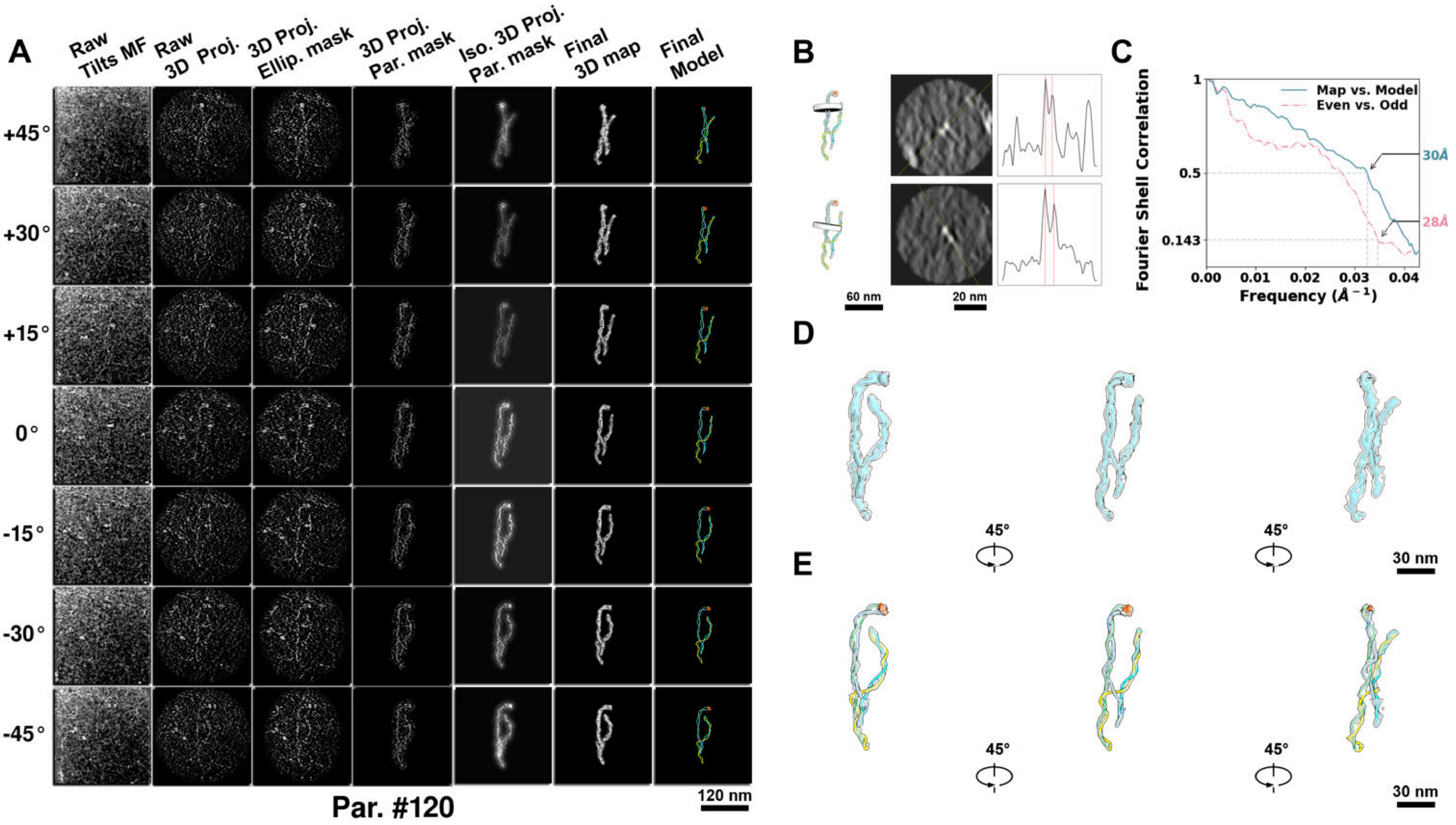

Supplementary Particle Figure 120. Cryo-ET 3D reconstruction of an individual P.Cas particle.
(A) 3D reconstruction of the plasmid particle (index no. 120). The first column shows seven representative tilt images from +45° to -45° in step of 15°. The second, third, and fourth columns show 3D projections of the particle with spherical, ellipsoidal (thinner along the z-dimension), and particle-shaped masks, respectively. The fifth column displays the 3D projections of the enhanced and IsoNet missing-wedge-corrected particle. The sixth and seventh columns present the final 3D map and the flexibly fitted model, respectively. (B) Two cross-sectional views (12 nm thickness) of the plasmid density map along its plectoneme axis are shown in the left-middle panel. The intensity profile along the line crossing the two high-density DNA spots is displayed in the right panel. (C) Resolution assessment of the final 3D map using Fourier shell correlation (FSC). Two criteria are shown: FSC between two half-maps reconstructed from even and odd frames (evaluated at 0.143) and FSC between the final 3D map and the fitted model (evaluated at 0.5). (D) Zoomed-in views of the final 3D density map from panel A, displayed at two contour levels. (E) Superimposition of the high-contour level map from panel D onto its fitted model.

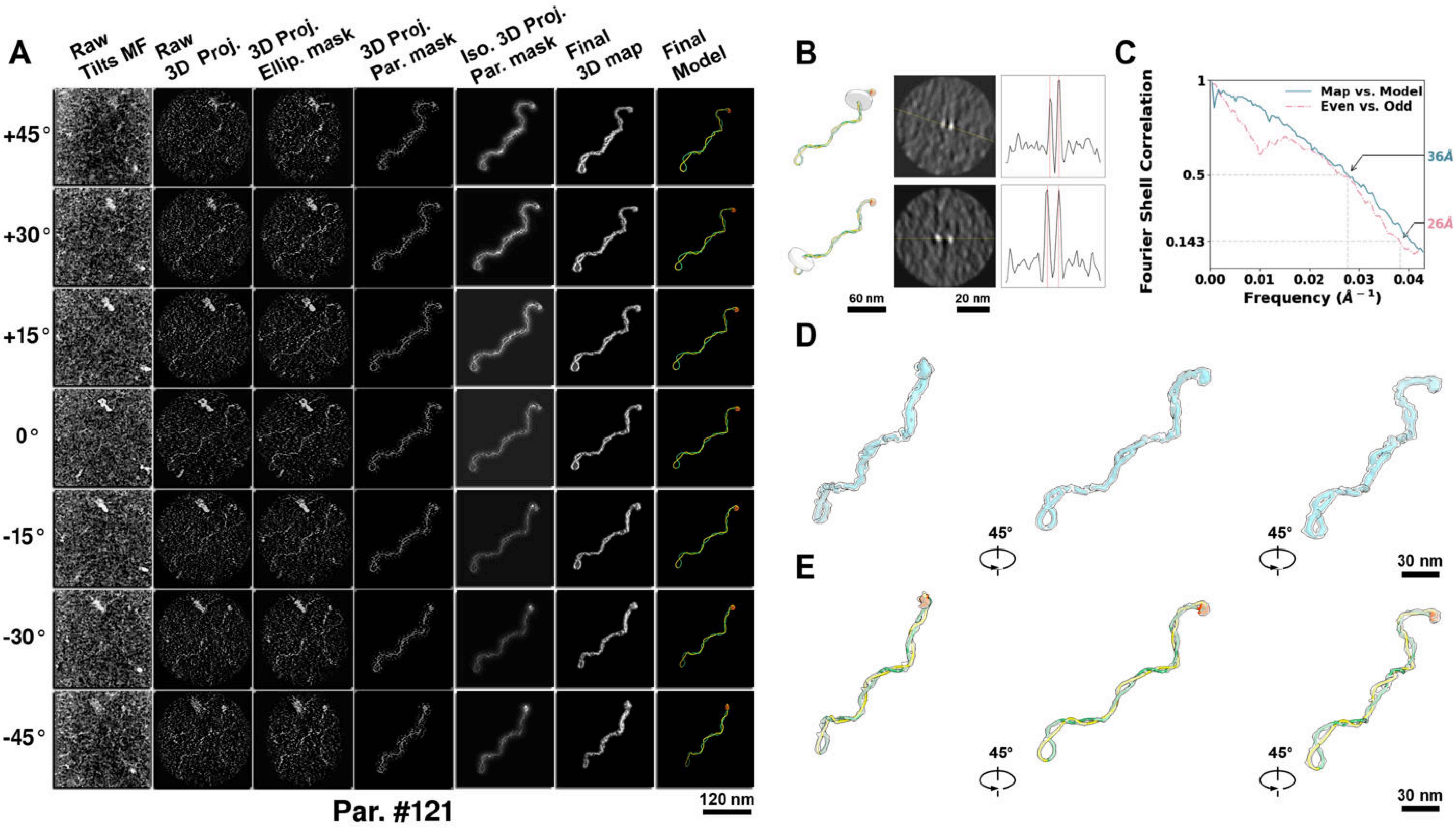

Supplementary Particle Figure 121. Cryo-ET 3D reconstruction of an individual P.Cas particle.
(A) 3D reconstruction of the plasmid particle (index no. 121). The first column shows seven representative tilt images from +45° to -45° in step of 15°. The second, third, and fourth columns show 3D projections of the particle with spherical, ellipsoidal (thinner along the z-dimension), and particle-shaped masks, respectively. The fifth column displays the 3D projections of the enhanced and IsoNet missing-wedge-corrected particle. The sixth and seventh columns present the final 3D map and the flexibly fitted model, respectively. (B) Two cross-sectional views (12 nm thickness) of the plasmid density map along its plectoneme axis are shown in the left-middle panel. The intensity profile along the line crossing the two high-density DNA spots is displayed in the right panel. (C) Resolution assessment of the final 3D map using Fourier shell correlation (FSC). Two criteria are shown: FSC between two half-maps reconstructed from even and odd frames (evaluated at 0.143) and FSC between the final 3D map and the fitted model (evaluated at 0.5). (D) Zoomed-in views of the final 3D density map from panel A, displayed at two contour levels. (E) Superimposition of the high-contour level map from panel D onto its fitted model.

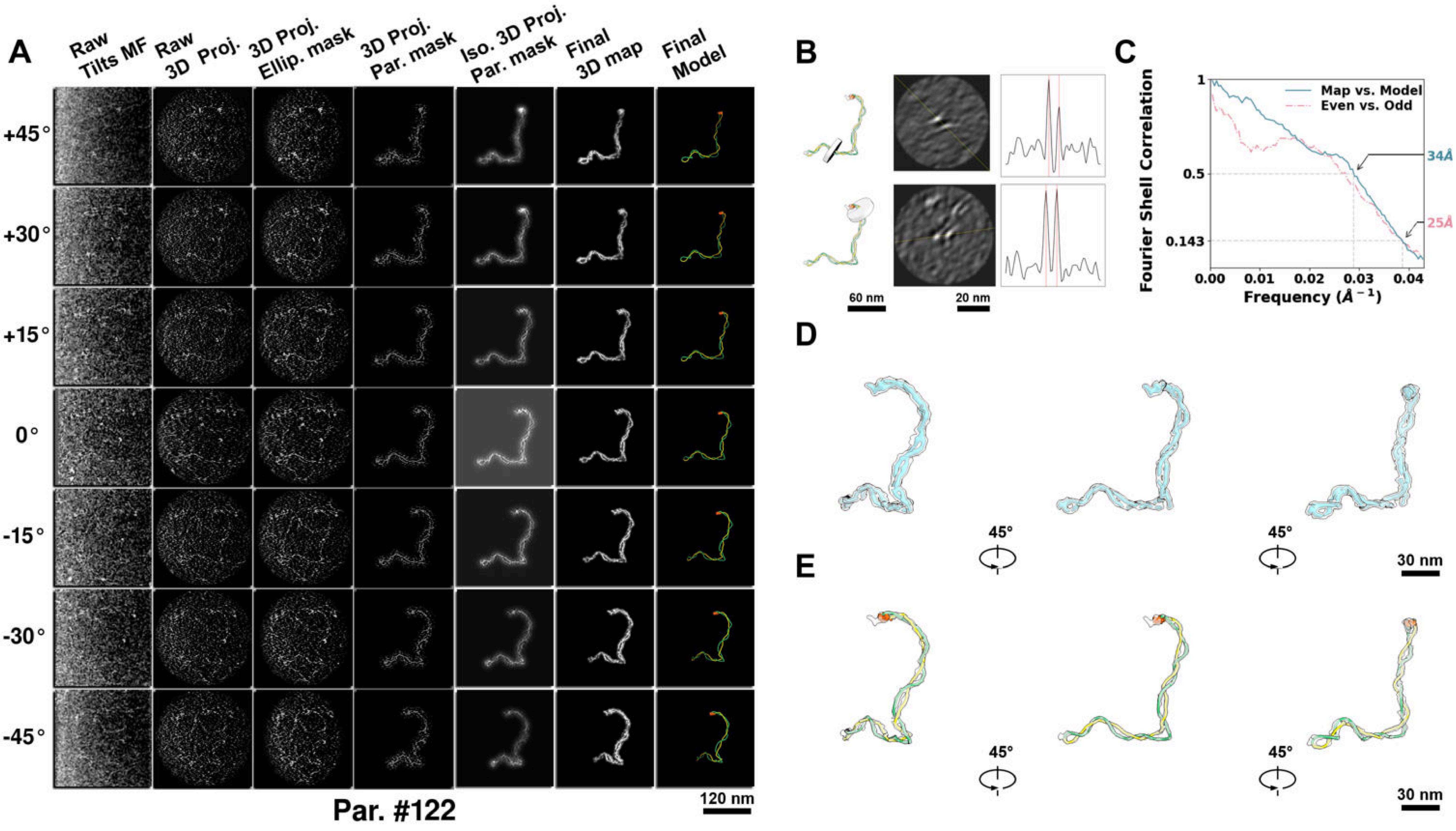

Supplementary Particle Figure 122. Cryo-ET 3D reconstruction of an individual P.Cas particle.
(A) 3D reconstruction of the plasmid particle (index no. 122). The first column shows seven representative tilt images from +45° to -45° in step of 15°. The second, third, and fourth columns show 3D projections of the particle with spherical, ellipsoidal (thinner along the z-dimension), and particle-shaped masks, respectively. The fifth column displays the 3D projections of the enhanced and IsoNet missing-wedge-corrected particle. The sixth and seventh columns present the final 3D map and the flexibly fitted model, respectively. (B) Two cross-sectional views (12 nm thickness) of the plasmid density map along its plectoneme axis are shown in the left-middle panel. The intensity profile along the line crossing the two high-density DNA spots is displayed in the right panel. (C) Resolution assessment of the final 3D map using Fourier shell correlation (FSC). Two criteria are shown: FSC between two half-maps reconstructed from even and odd frames (evaluated at 0.143) and FSC between the final 3D map and the fitted model (evaluated at 0.5). (D) Zoomed-in views of the final 3D density map from panel A, displayed at two contour levels. (E) Superimposition of the high-contour level map from panel D onto its fitted model.

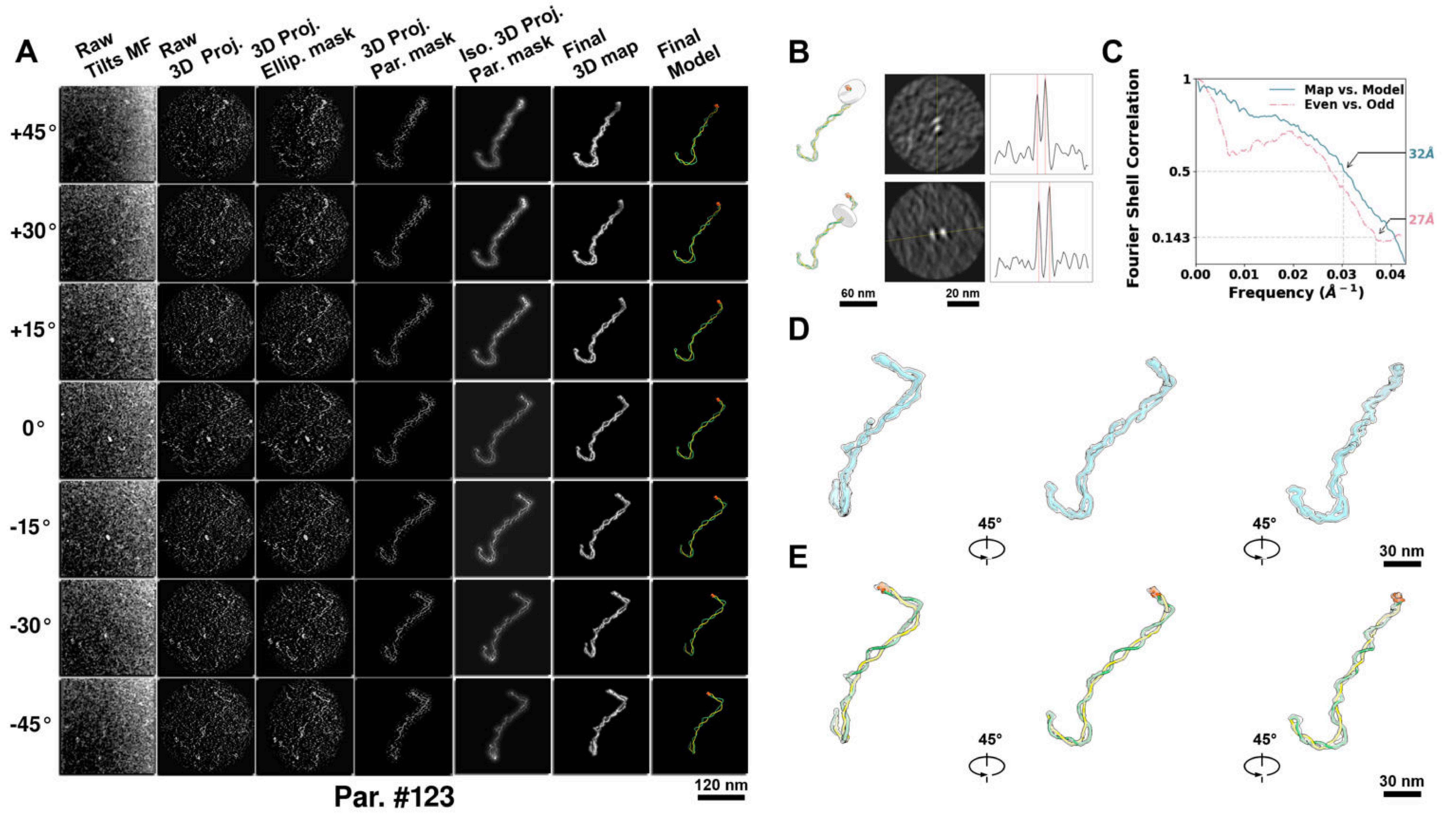

Supplementary Particle Figure 123. Cryo-ET 3D reconstruction of an individual P.Cas particle.
(A) 3D reconstruction of the plasmid particle (index no. 123). The first column shows seven representative tilt images from +45° to -45° in step of 15°. The second, third, and fourth columns show 3D projections of the particle with spherical, ellipsoidal (thinner along the z-dimension), and particle-shaped masks, respectively. The fifth column displays the 3D projections of the enhanced and IsoNet missing-wedge-corrected particle. The sixth and seventh columns present the final 3D map and the flexibly fitted model, respectively. (B) Two cross-sectional views (12 nm thickness) of the plasmid density map along its plectoneme axis are shown in the left-middle panel. The intensity profile along the line crossing the two high-density DNA spots is displayed in the right panel. (C) Resolution assessment of the final 3D map using Fourier shell correlation (FSC). Two criteria are shown: FSC between two half-maps reconstructed from even and odd frames (evaluated at 0.143) and FSC between the final 3D map and the fitted model (evaluated at 0.5). (D) Zoomed-in views of the final 3D density map from panel A, displayed at two contour levels. (E) Superimposition of the high-contour level map from panel D onto its fitted model.

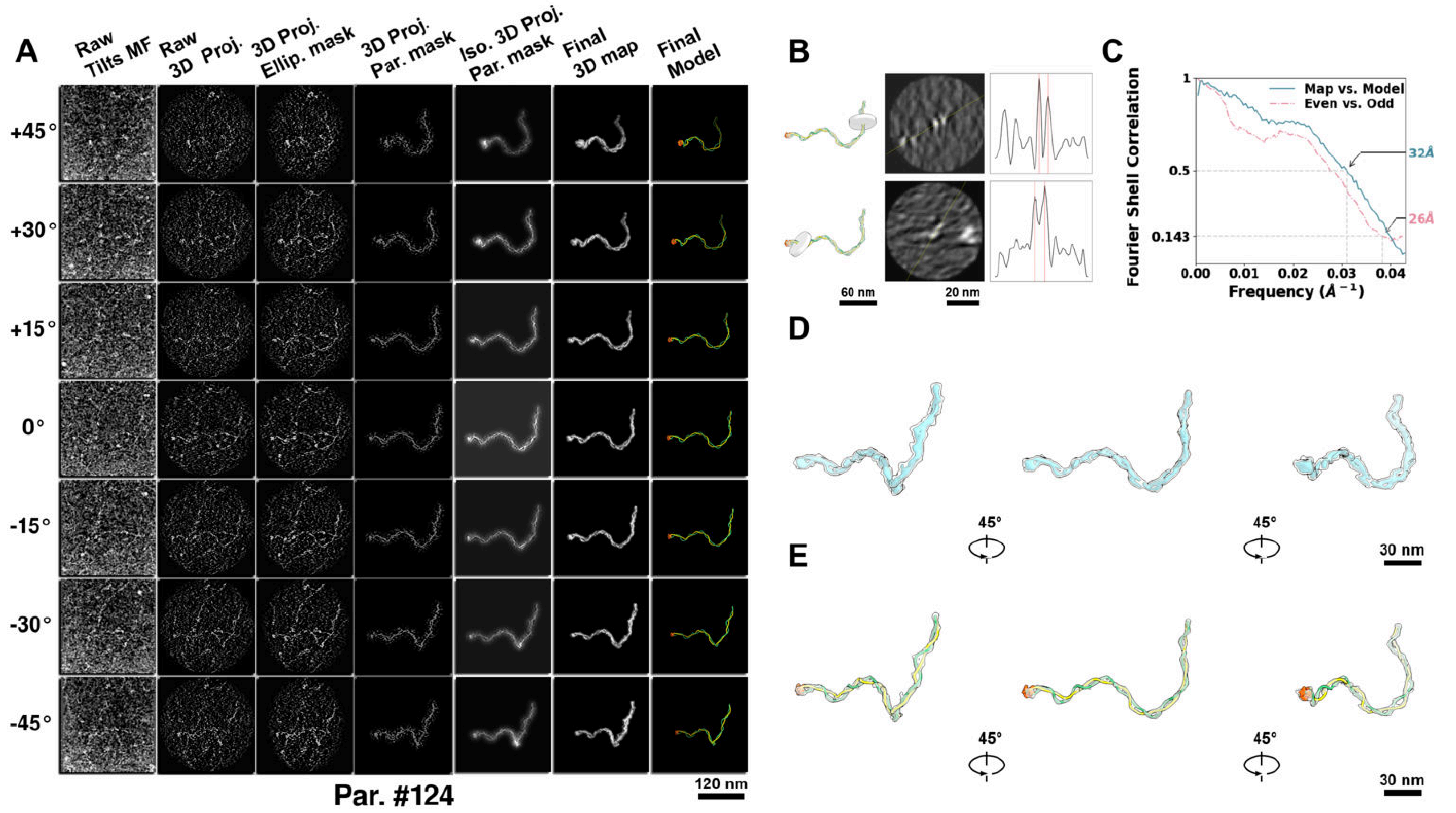

Supplementary Particle Figure 124. Cryo-ET 3D reconstruction of an individual P.Cas particle.
(A) 3D reconstruction of the plasmid particle (index no. 124). The first column shows seven representative tilt images from +45° to -45° in step of 15°. The second, third, and fourth columns show 3D projections of the particle with spherical, ellipsoidal (thinner along the z-dimension), and particle-shaped masks, respectively. The fifth column displays the 3D projections of the enhanced and IsoNet missing-wedge-corrected particle. The sixth and seventh columns present the final 3D map and the flexibly fitted model, respectively. (B) Two cross-sectional views (12 nm thickness) of the plasmid density map along its plectoneme axis are shown in the left-middle panel. The intensity profile along the line crossing the two high-density DNA spots is displayed in the right panel. (C) Resolution assessment of the final 3D map using Fourier shell correlation (FSC). Two criteria are shown: FSC between two half-maps reconstructed from even and odd frames (evaluated at 0.143) and FSC between the final 3D map and the fitted model (evaluated at 0.5). (D) Zoomed-in views of the final 3D density map from panel A, displayed at two contour levels. (E) Superimposition of the high-contour level map from panel D onto its fitted model.

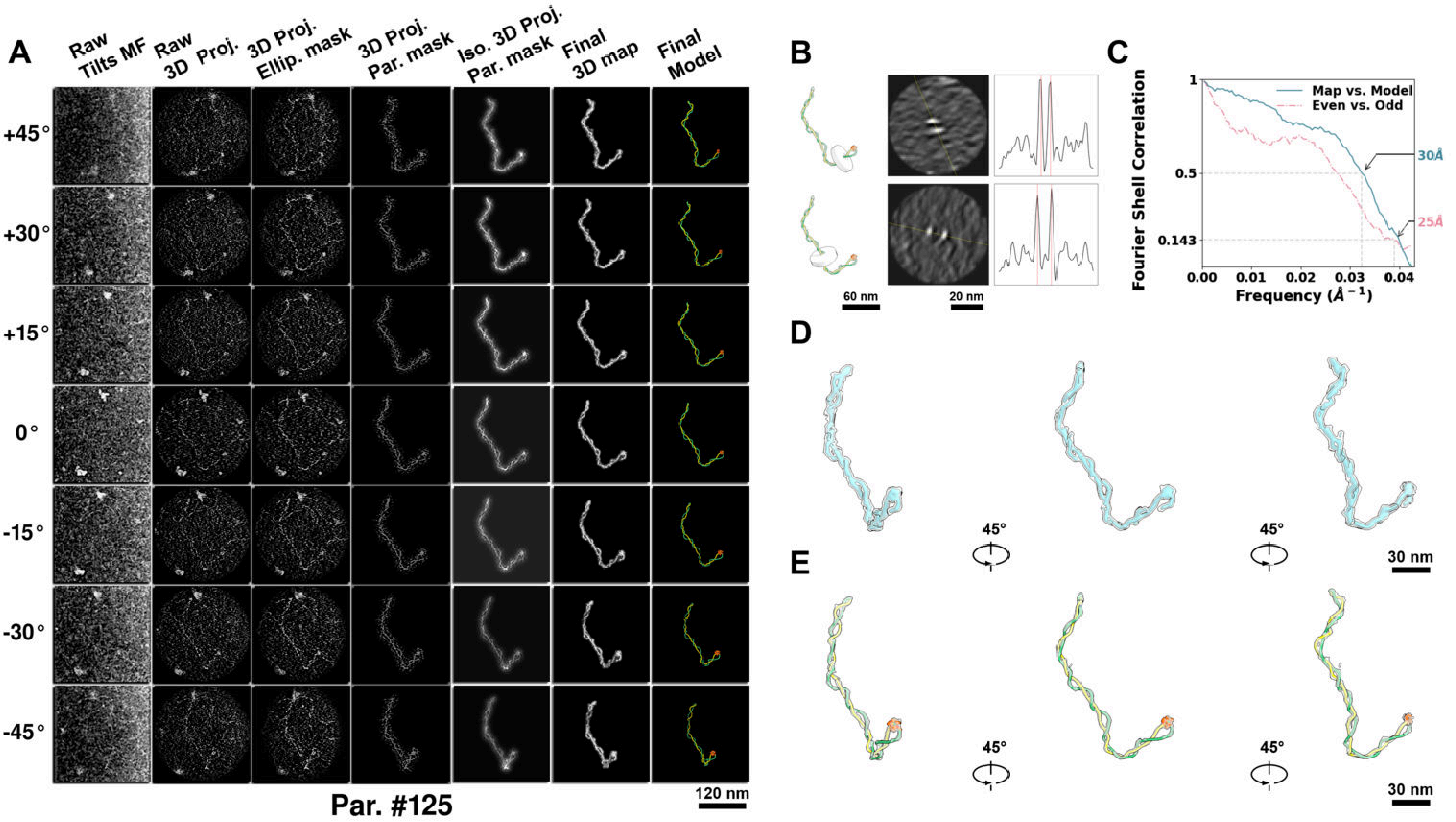

Supplementary Particle Figure 125. Cryo-ET 3D reconstruction of an individual P.Cas particle.
(A) 3D reconstruction of the plasmid particle (index no. 125). The first column shows seven representative tilt images from +45° to -45° in step of 15°. The second, third, and fourth columns show 3D projections of the particle with spherical, ellipsoidal (thinner along the z-dimension), and particle-shaped masks, respectively. The fifth column displays the 3D projections of the enhanced and IsoNet missing-wedge-corrected particle. The sixth and seventh columns present the final 3D map and the flexibly fitted model, respectively. (B) Two cross-sectional views (12 nm thickness) of the plasmid density map along its plectoneme axis are shown in the left-middle panel. The intensity profile along the line crossing the two high-density DNA spots is displayed in the right panel. (C) Resolution assessment of the final 3D map using Fourier shell correlation (FSC). Two criteria are shown: FSC between two half-maps reconstructed from even and odd frames (evaluated at 0.143) and FSC between the final 3D map and the fitted model (evaluated at 0.5). (D) Zoomed-in views of the final 3D density map from panel A, displayed at two contour levels. (E) Superimposition of the high-contour level map from panel D onto its fitted model.

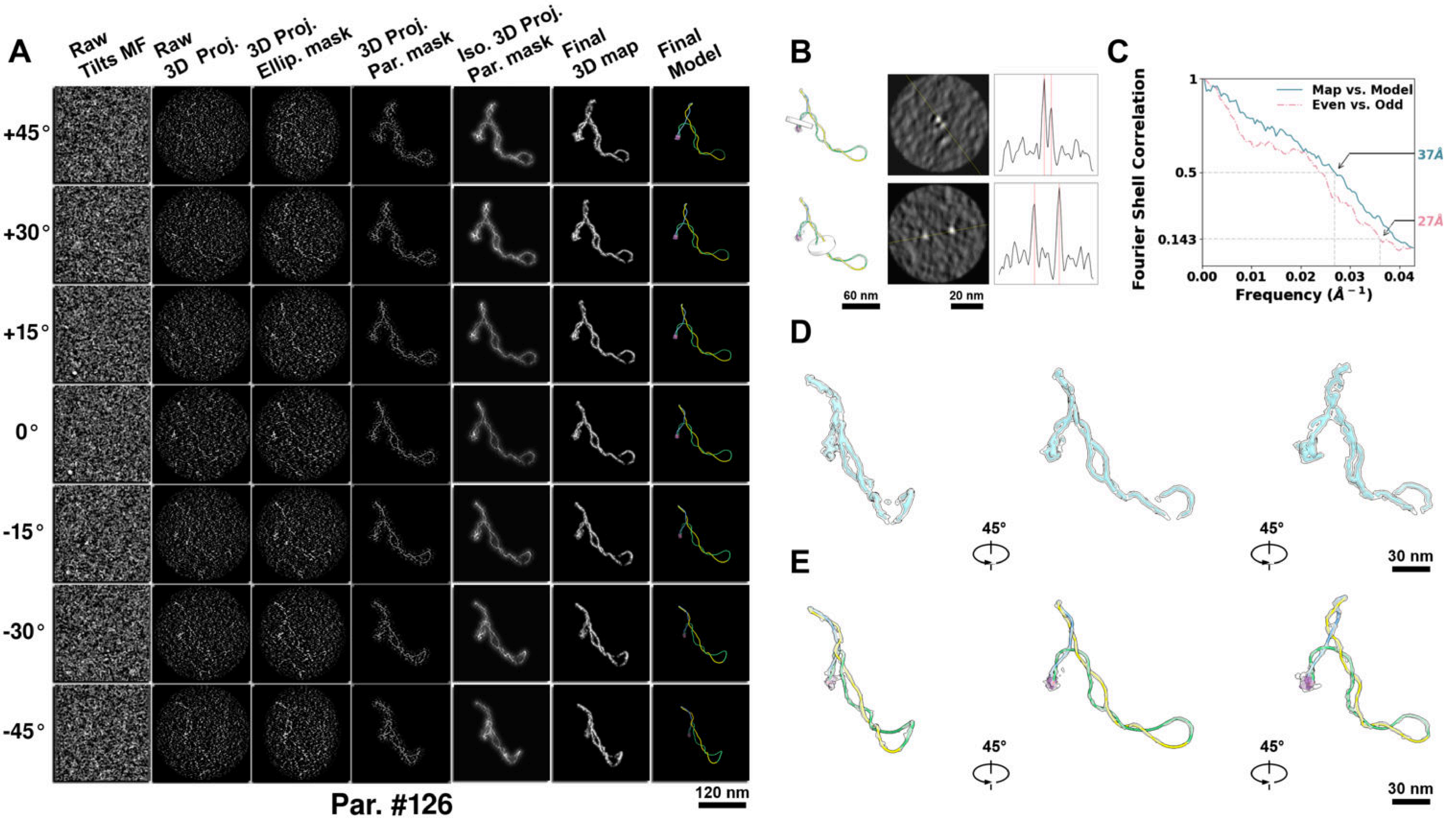

Supplementary Particle Figure 126. Cryo-ET 3D reconstruction of an individual TEC particle.
(A) 3D reconstruction of the plasmid particle (index no. 126). The first column shows seven representative tilt images from +45° to -45° in step of 15°. The second, third, and fourth columns show 3D projections of the particle with spherical, ellipsoidal (thinner along the z-dimension), and particle-shaped masks, respectively. The fifth column displays the 3D projections of the enhanced and IsoNet missing-wedge-corrected particle. The sixth and seventh columns present the final 3D map and the flexibly fitted model, respectively. (B) Two cross-sectional views (12 nm thickness) of the plasmid density map along its plectoneme axis are shown in the left-middle panel. The intensity profile along the line crossing the two high-density DNA spots is displayed in the right panel. (C) Resolution assessment of the final 3D map using Fourier shell correlation (FSC). Two criteria are shown: FSC between two half-maps reconstructed from even and odd frames (evaluated at 0.143) and FSC between the final 3D map and the fitted model (evaluated at 0.5). (D) Zoomed-in views of the final 3D density map from panel A, displayed at two contour levels. (E) Superimposition of the high-contour level map from panel D onto its fitted model.

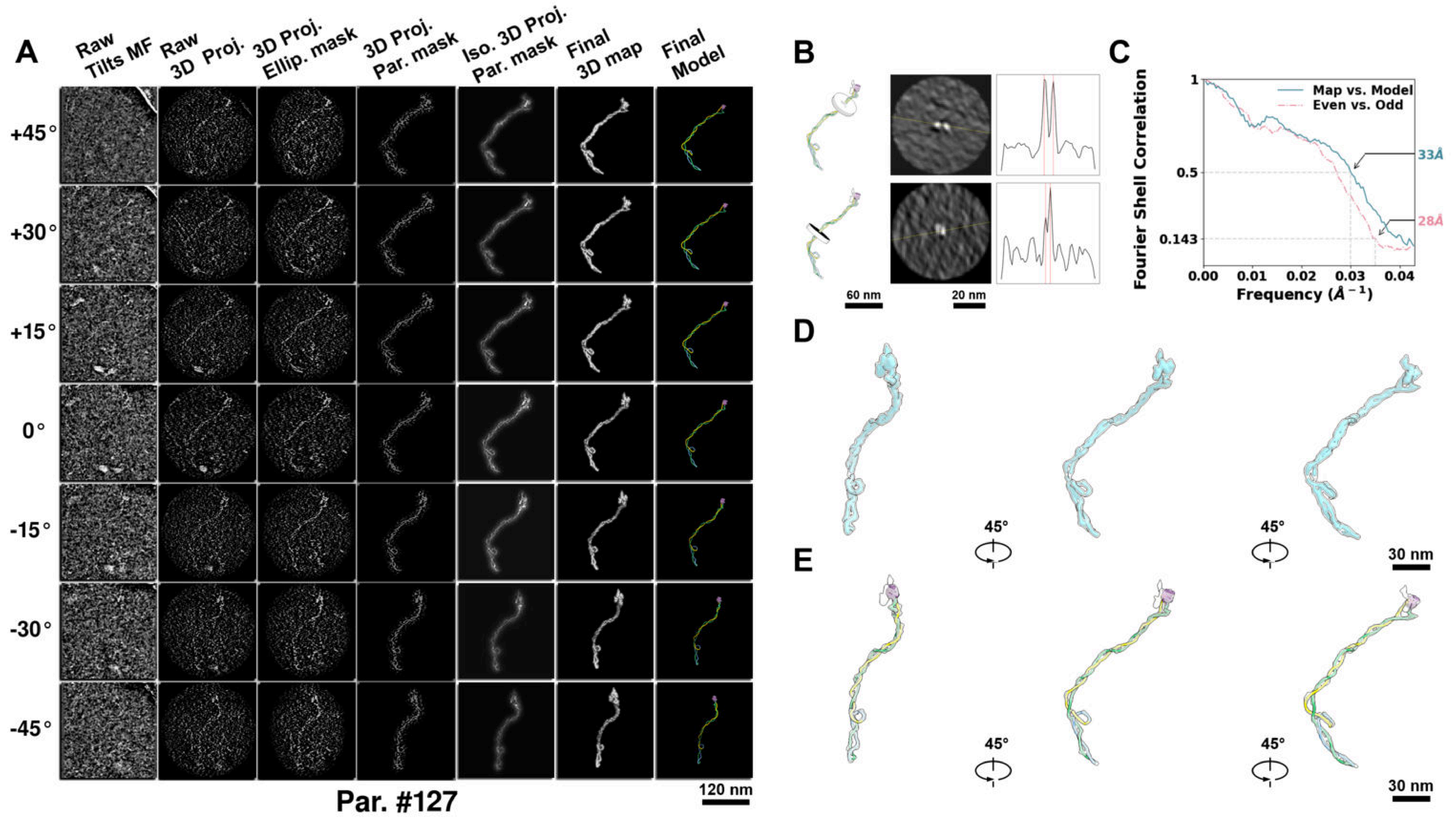

Supplementary Particle Figure 127. Cryo-ET 3D reconstruction of an individual TEC particle.
(A) 3D reconstruction of the plasmid particle (index no. 127). The first column shows seven representative tilt images from +45° to -45° in step of 15°. The second, third, and fourth columns show 3D projections of the particle with spherical, ellipsoidal (thinner along the z-dimension), and particle-shaped masks, respectively. The fifth column displays the 3D projections of the enhanced and IsoNet missing-wedge-corrected particle. The sixth and seventh columns present the final 3D map and the flexibly fitted model, respectively. (B) Two cross-sectional views (12 nm thickness) of the plasmid density map along its plectoneme axis are shown in the left-middle panel. The intensity profile along the line crossing the two high-density DNA spots is displayed in the right panel. (C) Resolution assessment of the final 3D map using Fourier shell correlation (FSC). Two criteria are shown: FSC between two half-maps reconstructed from even and odd frames (evaluated at 0.143) and FSC between the final 3D map and the fitted model (evaluated at 0.5). (D) Zoomed-in views of the final 3D density map from panel A, displayed at two contour levels. (E) Superimposition of the high-contour level map from panel D onto its fitted model.

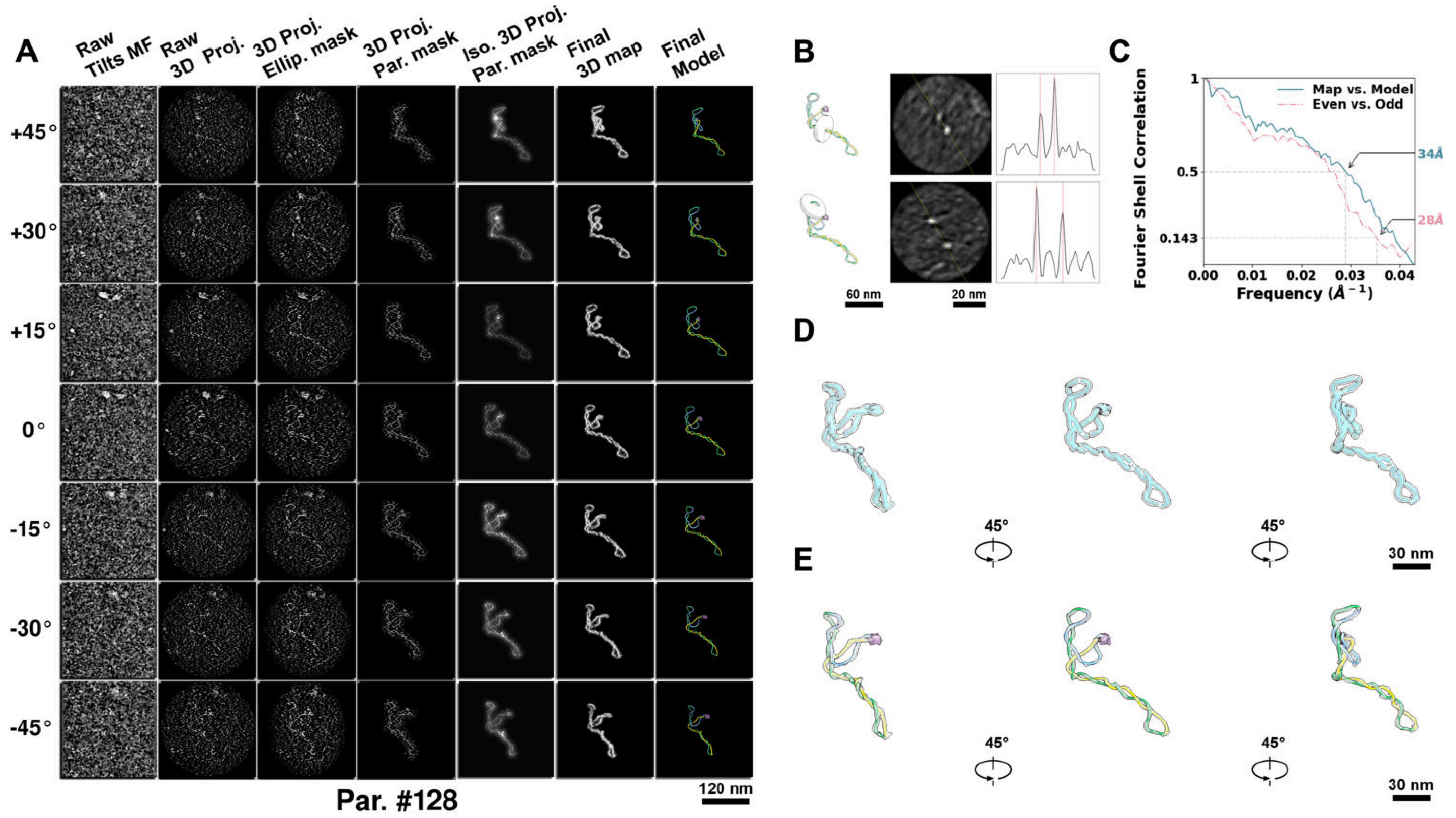

Supplementary Particle Figure 128. Cryo-ET 3D reconstruction of an individual TEC particle.
(A) 3D reconstruction of the plasmid particle (index no. 128). The first column shows seven representative tilt images from +45° to -45° in step of 15°. The second, third, and fourth columns show 3D projections of the particle with spherical, ellipsoidal (thinner along the z-dimension), and particle-shaped masks, respectively. The fifth column displays the 3D projections of the enhanced and IsoNet missing-wedge-corrected particle. The sixth and seventh columns present the final 3D map and the flexibly fitted model, respectively. (B) Two cross-sectional views (12 nm thickness) of the plasmid density map along its plectoneme axis are shown in the left-middle panel. The intensity profile along the line crossing the two high-density DNA spots is displayed in the right panel. (C) Resolution assessment of the final 3D map using Fourier shell correlation (FSC). Two criteria are shown: FSC between two half-maps reconstructed from even and odd frames (evaluated at 0.143) and FSC between the final 3D map and the fitted model (evaluated at 0.5). (D) Zoomed-in views of the final 3D density map from panel A, displayed at two contour levels. (E) Superimposition of the high-contour level map from panel D onto its fitted model.

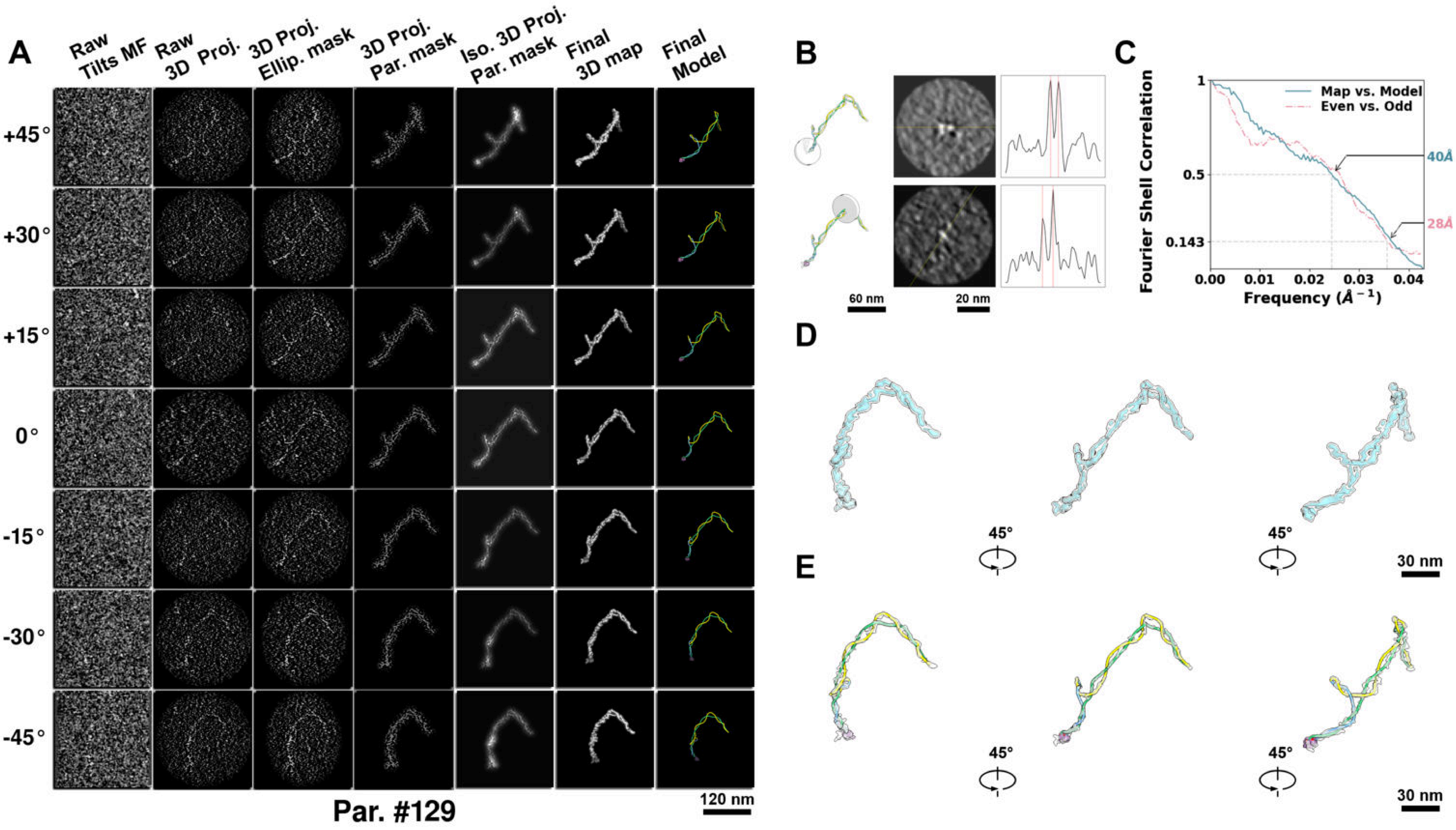

Supplementary Particle Figure 129. Cryo-ET 3D reconstruction of an individual TEC particle.
(A) 3D reconstruction of the plasmid particle (index no. 129). The first column shows seven representative tilt images from +45° to -45° in step of 15°. The second, third, and fourth columns show 3D projections of the particle with spherical, ellipsoidal (thinner along the z-dimension), and particle-shaped masks, respectively. The fifth column displays the 3D projections of the enhanced and IsoNet missing-wedge-corrected particle. The sixth and seventh columns present the final 3D map and the flexibly fitted model, respectively. (B) Two cross-sectional views (12 nm thickness) of the plasmid density map along its plectoneme axis are shown in the left-middle panel. The intensity profile along the line crossing the two high-density DNA spots is displayed in the right panel. (C) Resolution assessment of the final 3D map using Fourier shell correlation (FSC). Two criteria are shown: FSC between two half-maps reconstructed from even and odd frames (evaluated at 0.143) and FSC between the final 3D map and the fitted model (evaluated at 0.5). (D) Zoomed-in views of the final 3D density map from panel A, displayed at two contour levels. (E) Superimposition of the high-contour level map from panel D onto its fitted model.

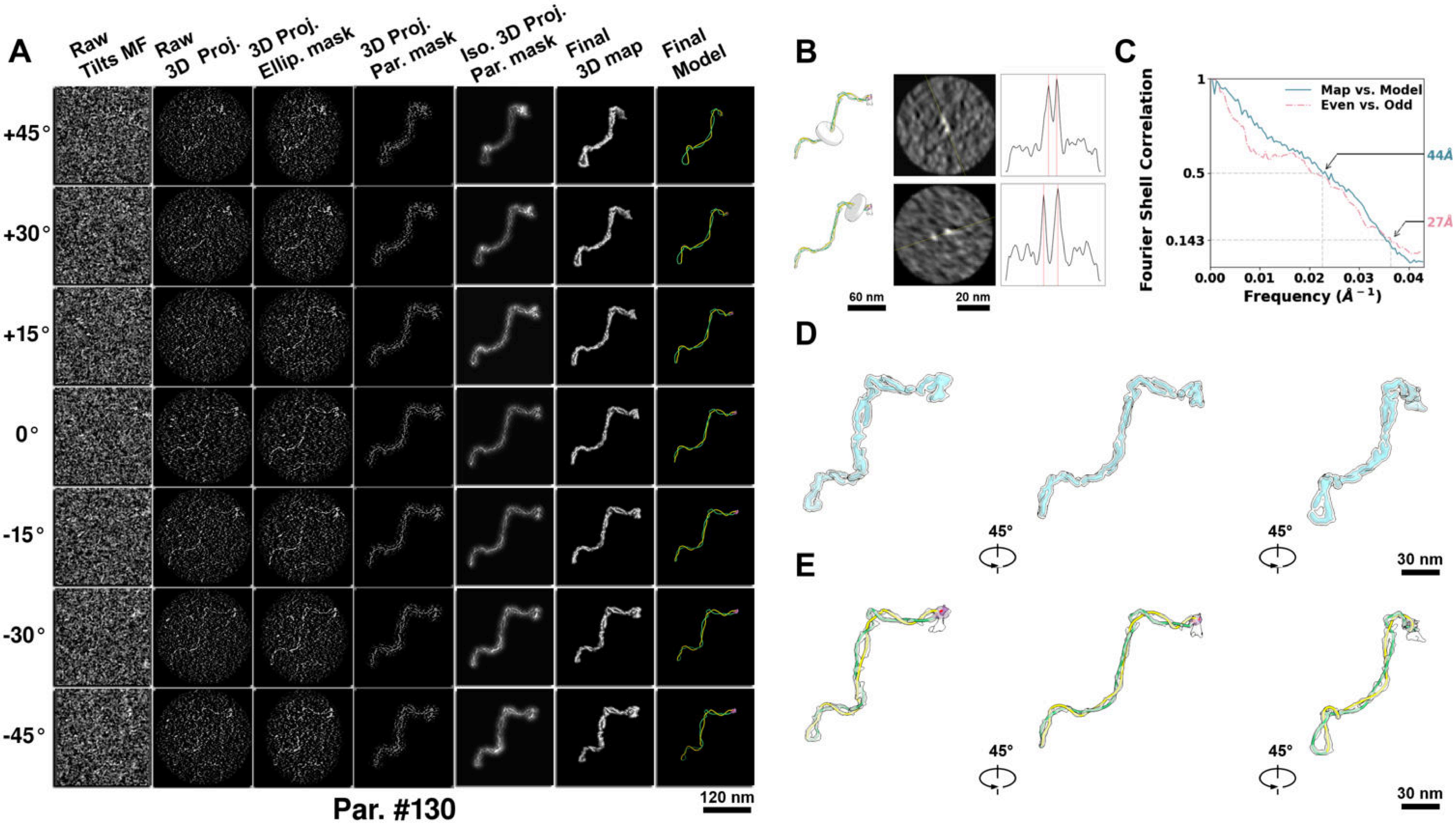

Supplementary Particle Figure 130. Cryo-ET 3D reconstruction of an individual TEC particle.
(A) 3D reconstruction of the plasmid particle (index no. 130). The first column shows seven representative tilt images from +45° to -45° in step of 15°. The second, third, and fourth columns show 3D projections of the particle with spherical, ellipsoidal (thinner along the z-dimension), and particle-shaped masks, respectively. The fifth column displays the 3D projections of the enhanced and IsoNet missing-wedge-corrected particle. The sixth and seventh columns present the final 3D map and the flexibly fitted model, respectively. (B) Two cross-sectional views (12 nm thickness) of the plasmid density map along its plectoneme axis are shown in the left-middle panel. The intensity profile along the line crossing the two high-density DNA spots is displayed in the right panel. (C) Resolution assessment of the final 3D map using Fourier shell correlation (FSC). Two criteria are shown: FSC between two half-maps reconstructed from even and odd frames (evaluated at 0.143) and FSC between the final 3D map and the fitted model (evaluated at 0.5). (D) Zoomed-in views of the final 3D density map from panel A, displayed at two contour levels. (E) Superimposition of the high-contour level map from panel D onto its fitted model.

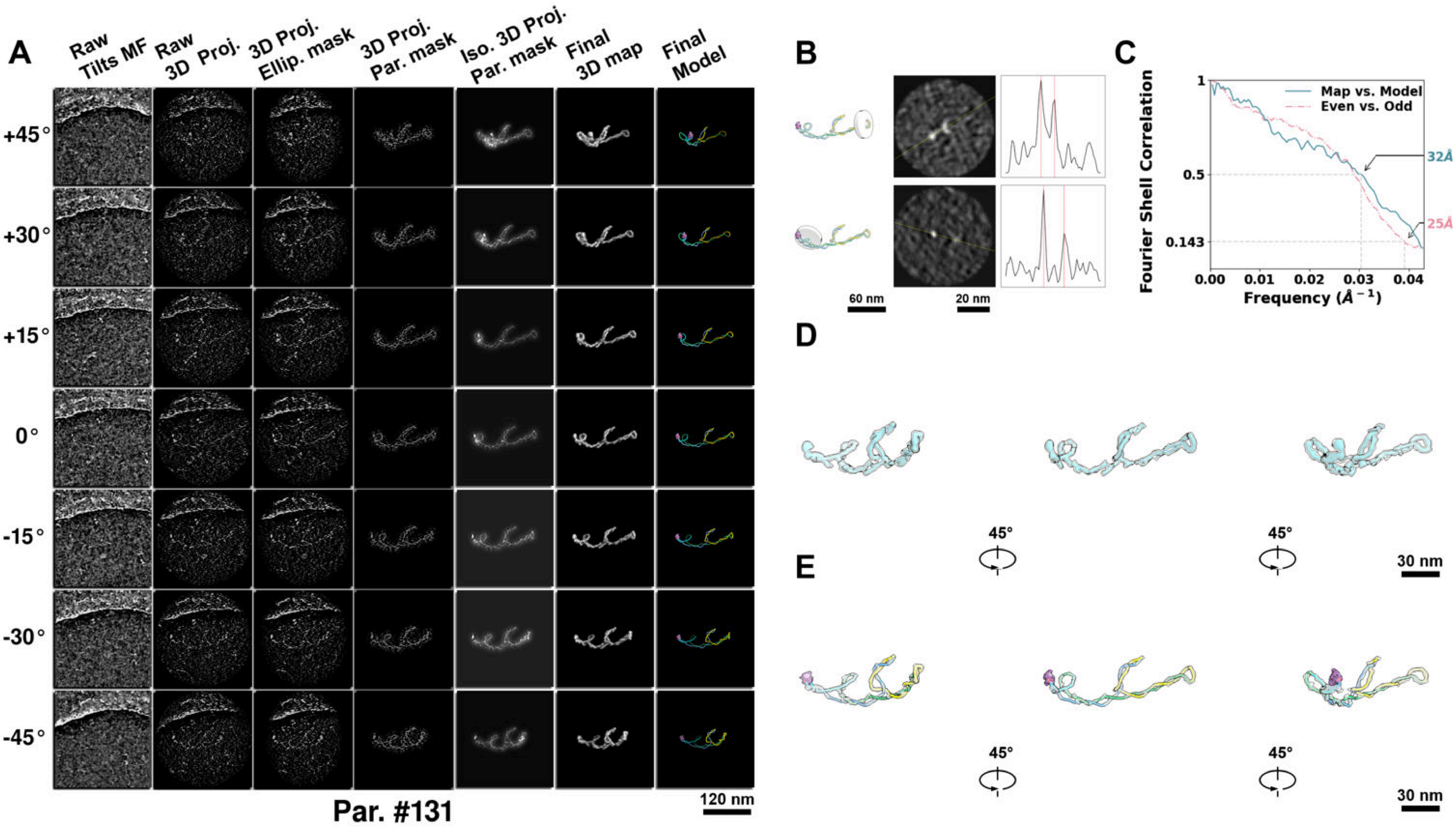

Supplementary Particle Figure 131. Cryo-ET 3D reconstruction of an individual TEC particle.
(A) 3D reconstruction of the plasmid particle (index no. 131). The first column shows seven representative tilt images from +45° to -45° in step of 15°. The second, third, and fourth columns show 3D projections of the particle with spherical, ellipsoidal (thinner along the z-dimension), and particle-shaped masks, respectively. The fifth column displays the 3D projections of the enhanced and IsoNet missing-wedge-corrected particle. The sixth and seventh columns present the final 3D map and the flexibly fitted model, respectively. (B) Two cross-sectional views (12 nm thickness) of the plasmid density map along its plectoneme axis are shown in the left-middle panel. The intensity profile along the line crossing the two high-density DNA spots is displayed in the right panel. (C) Resolution assessment of the final 3D map using Fourier shell correlation (FSC). Two criteria are shown: FSC between two half-maps reconstructed from even and odd frames (evaluated at 0.143) and FSC between the final 3D map and the fitted model (evaluated at 0.5). (D) Zoomed-in views of the final 3D density map from panel A, displayed at two contour levels. (E) Superimposition of the high-contour level map from panel D onto its fitted model.

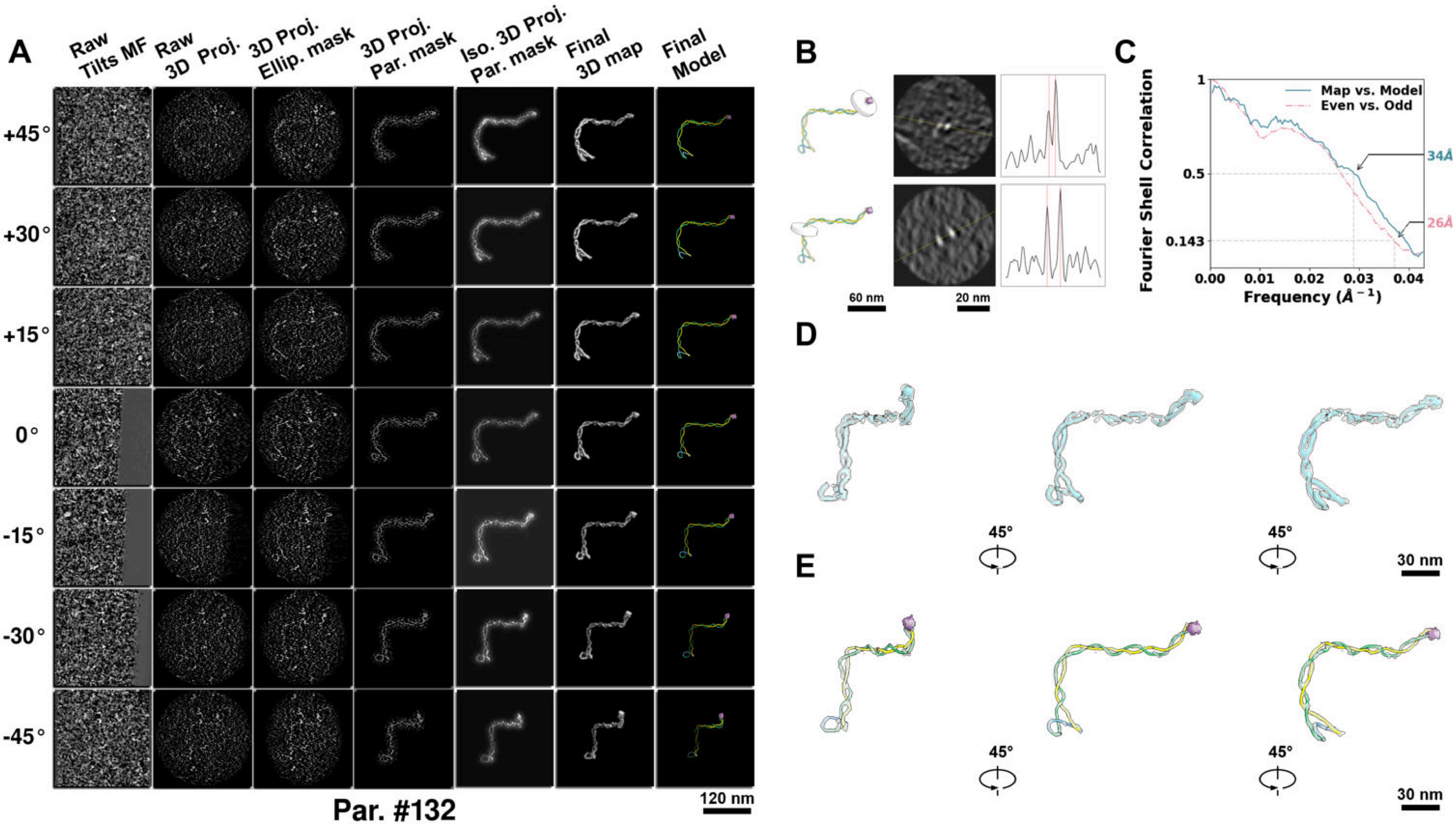

Supplementary Particle Figure 132. Cryo-ET 3D reconstruction of an individual TEC particle.
(A) 3D reconstruction of the plasmid particle (index no. 132). The first column shows seven representative tilt images from +45° to -45° in step of 15°. The second, third, and fourth columns show 3D projections of the particle with spherical, ellipsoidal (thinner along the z-dimension), and particle-shaped masks, respectively. The fifth column displays the 3D projections of the enhanced and IsoNet missing-wedge-corrected particle. The sixth and seventh columns present the final 3D map and the flexibly fitted model, respectively. (B) Two cross-sectional views (12 nm thickness) of the plasmid density map along its plectoneme axis are shown in the left-middle panel. The intensity profile along the line crossing the two high-density DNA spots is displayed in the right panel. (C) Resolution assessment of the final 3D map using Fourier shell correlation (FSC). Two criteria are shown: FSC between two half-maps reconstructed from even and odd frames (evaluated at 0.143) and FSC between the final 3D map and the fitted model (evaluated at 0.5). (D) Zoomed-in views of the final 3D density map from panel A, displayed at two contour levels. (E) Superimposition of the high-contour level map from panel D onto its fitted model.

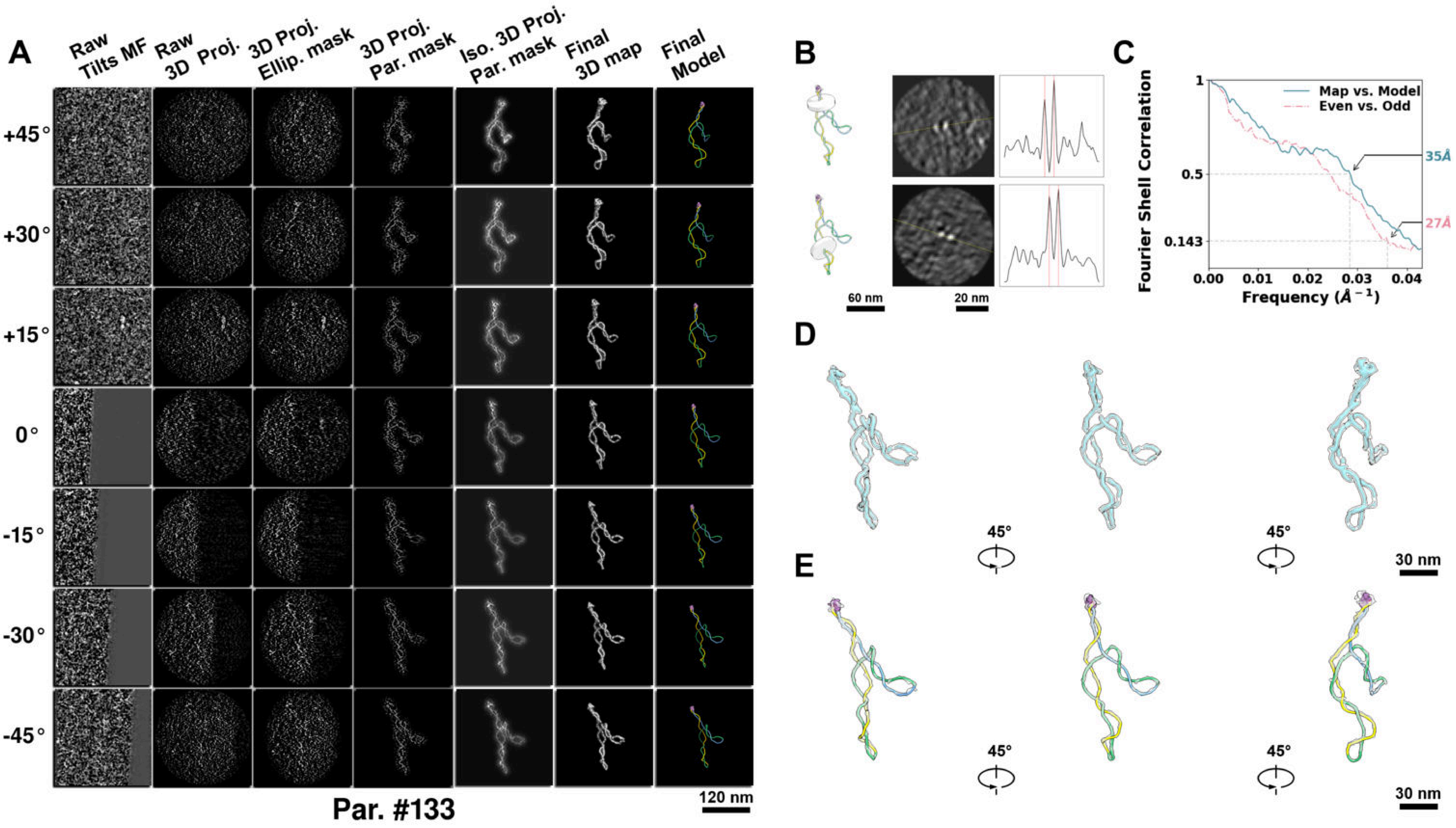

Supplementary Particle Figure 133. Cryo-ET 3D reconstruction of an individual TEC particle.
(A) 3D reconstruction of the plasmid particle (index no. 133). The first column shows seven representative tilt images from +45° to -45° in step of 15°. The second, third, and fourth columns show 3D projections of the particle with spherical, ellipsoidal (thinner along the z-dimension), and particle-shaped masks, respectively. The fifth column displays the 3D projections of the enhanced and IsoNet missing-wedge-corrected particle. The sixth and seventh columns present the final 3D map and the flexibly fitted model, respectively. (B) Two cross-sectional views (12 nm thickness) of the plasmid density map along its plectoneme axis are shown in the left-middle panel. The intensity profile along the line crossing the two high-density DNA spots is displayed in the right panel. (C) Resolution assessment of the final 3D map using Fourier shell correlation (FSC). Two criteria are shown: FSC between two half-maps reconstructed from even and odd frames (evaluated at 0.143) and FSC between the final 3D map and the fitted model (evaluated at 0.5). (D) Zoomed-in views of the final 3D density map from panel A, displayed at two contour levels. (E) Superimposition of the high-contour level map from panel D onto its fitted model.

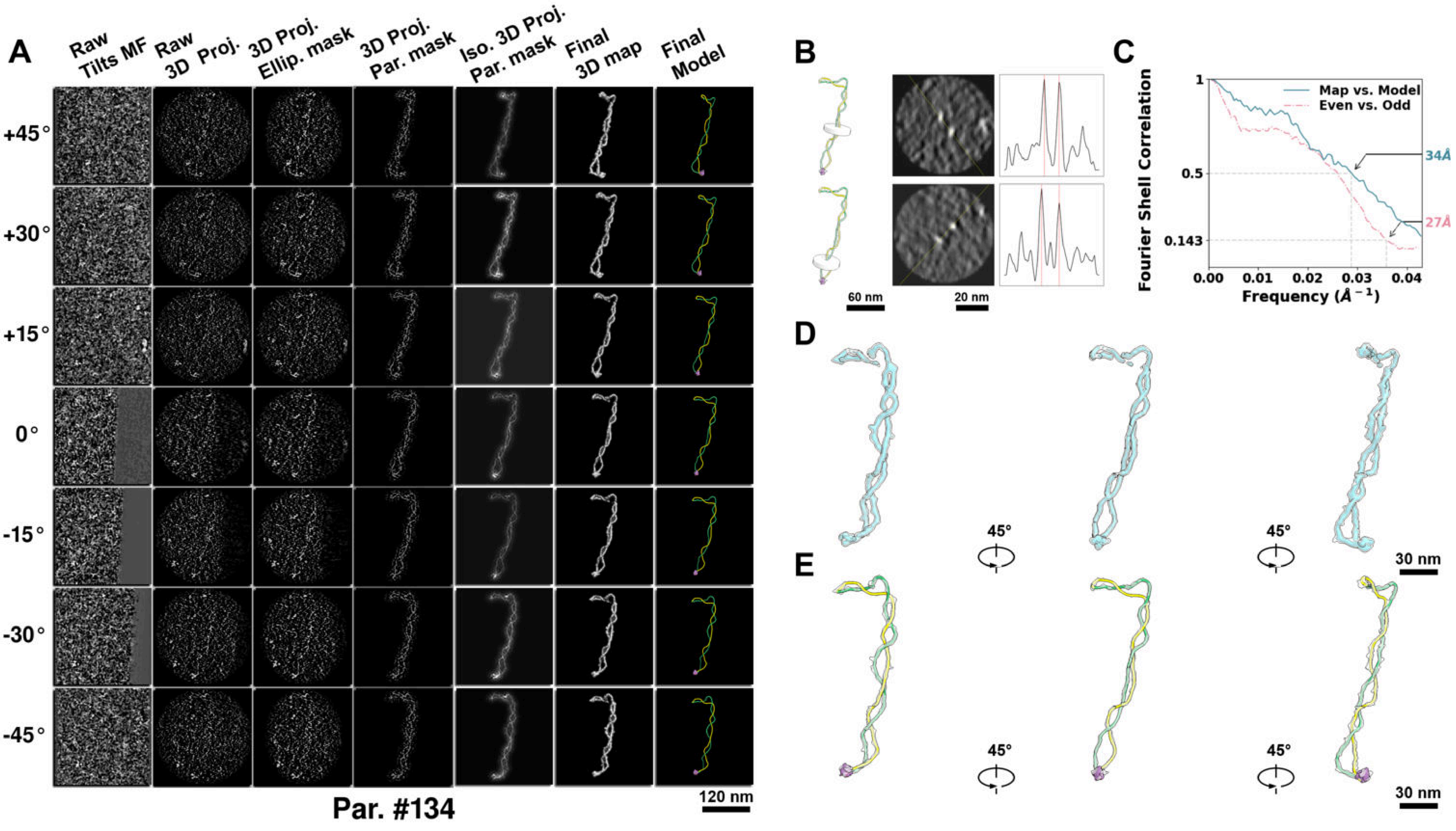

Supplementary Particle Figure 134. Cryo-ET 3D reconstruction of an individual TEC particle.
(A) 3D reconstruction of the plasmid particle (index no. 134). The first column shows seven representative tilt images from +45° to -45° in step of 15°. The second, third, and fourth columns show 3D projections of the particle with spherical, ellipsoidal (thinner along the z-dimension), and particle-shaped masks, respectively. The fifth column displays the 3D projections of the enhanced and IsoNet missing-wedge-corrected particle. The sixth and seventh columns present the final 3D map and the flexibly fitted model, respectively. (B) Two cross-sectional views (12 nm thickness) of the plasmid density map along its plectoneme axis are shown in the left-middle panel. The intensity profile along the line crossing the two high-density DNA spots is displayed in the right panel. (C) Resolution assessment of the final 3D map using Fourier shell correlation (FSC). Two criteria are shown: FSC between two half-maps reconstructed from even and odd frames (evaluated at 0.143) and FSC between the final 3D map and the fitted model (evaluated at 0.5). (D) Zoomed-in views of the final 3D density map from panel A, displayed at two contour levels. (E) Superimposition of the high-contour level map from panel D onto its fitted model.

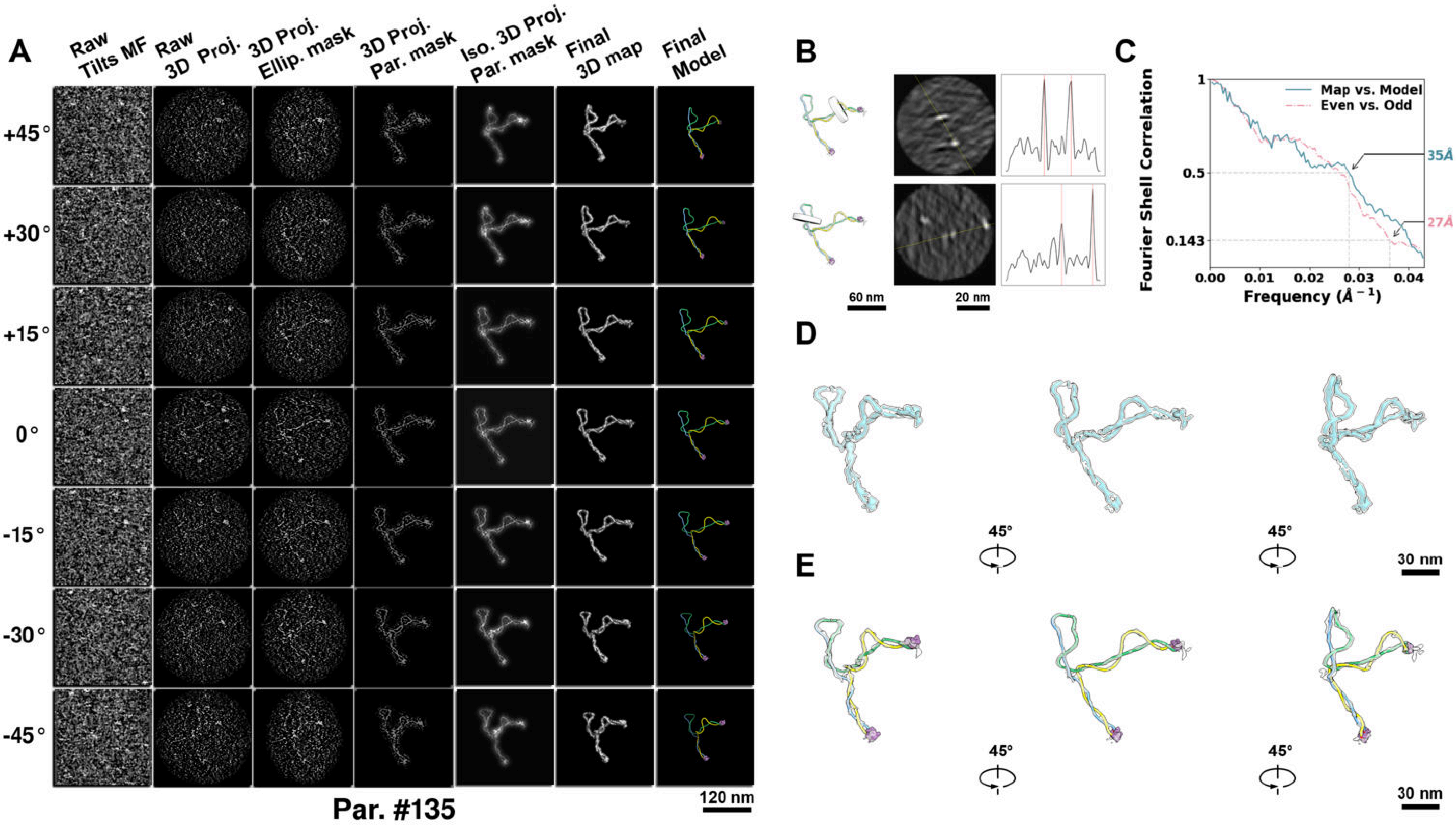

Supplementary Particle Figure 135. Cryo-ET 3D reconstruction of an individual TEC particle.
(A) 3D reconstruction of the plasmid particle (index no. 135). The first column shows seven representative tilt images from +45° to -45° in step of 15°. The second, third, and fourth columns show 3D projections of the particle with spherical, ellipsoidal (thinner along the z-dimension), and particle-shaped masks, respectively. The fifth column displays the 3D projections of the enhanced and IsoNet missing-wedge-corrected particle. The sixth and seventh columns present the final 3D map and the flexibly fitted model, respectively. (B) Two cross-sectional views (12 nm thickness) of the plasmid density map along its plectoneme axis are shown in the left-middle panel. The intensity profile along the line crossing the two high-density DNA spots is displayed in the right panel. (C) Resolution assessment of the final 3D map using Fourier shell correlation (FSC). Two criteria are shown: FSC between two half-maps reconstructed from even and odd frames (evaluated at 0.143) and FSC between the final 3D map and the fitted model (evaluated at 0.5). (D) Zoomed-in views of the final 3D density map from panel A, displayed at two contour levels. (E) Superimposition of the high-contour level map from panel D onto its fitted model.

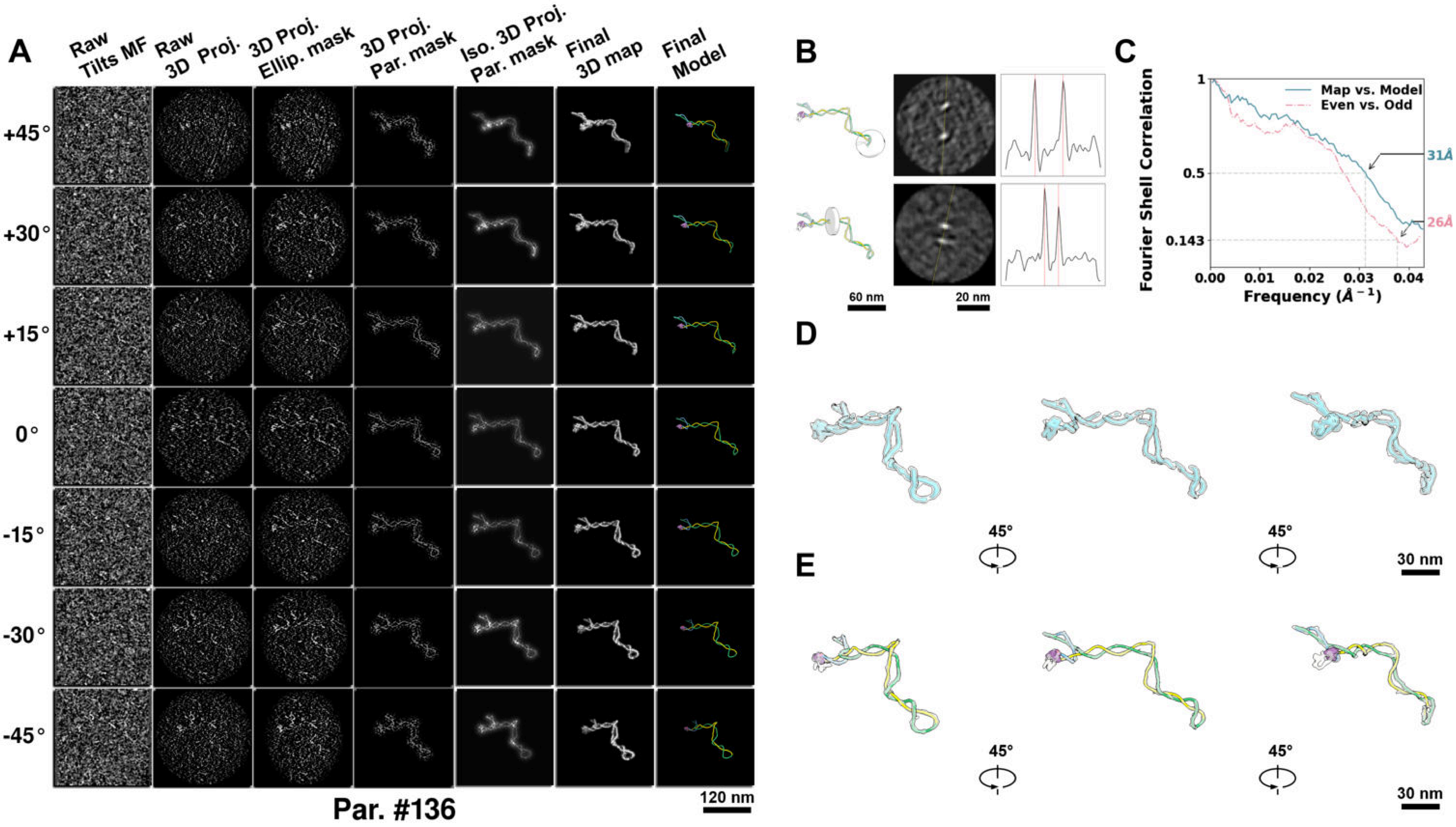

Supplementary Particle Figure 136. Cryo-ET 3D reconstruction of an individual TEC particle.
(A) 3D reconstruction of the plasmid particle (index no. 136). The first column shows seven representative tilt images from +45° to -45° in step of 15°. The second, third, and fourth columns show 3D projections of the particle with spherical, ellipsoidal (thinner along the z-dimension), and particle-shaped masks, respectively. The fifth column displays the 3D projections of the enhanced and IsoNet missing-wedge-corrected particle. The sixth and seventh columns present the final 3D map and the flexibly fitted model, respectively. (B) Two cross-sectional views (12 nm thickness) of the plasmid density map along its plectoneme axis are shown in the left-middle panel. The intensity profile along the line crossing the two high-density DNA spots is displayed in the right panel. (C) Resolution assessment of the final 3D map using Fourier shell correlation (FSC). Two criteria are shown: FSC between two half-maps reconstructed from even and odd frames (evaluated at 0.143) and FSC between the final 3D map and the fitted model (evaluated at 0.5). (D) Zoomed-in views of the final 3D density map from panel A, displayed at two contour levels. (E) Superimposition of the high-contour level map from panel D onto its fitted model.

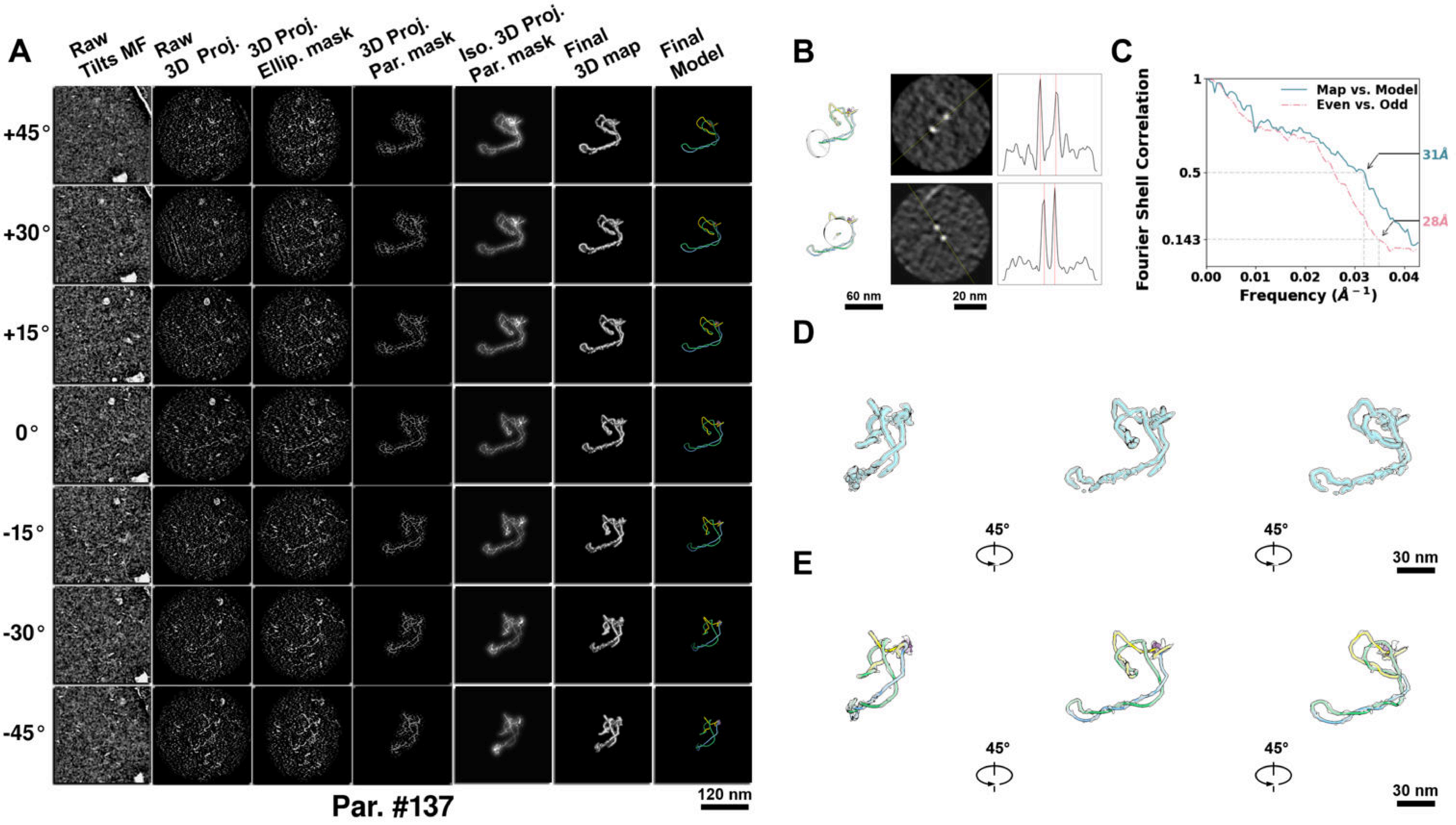

Supplementary Particle Figure 137. Cryo-ET 3D reconstruction of an individual TEC particle.
(A) 3D reconstruction of the plasmid particle (index no. 137). The first column shows seven representative tilt images from +45° to -45° in step of 15°. The second, third, and fourth columns show 3D projections of the particle with spherical, ellipsoidal (thinner along the z-dimension), and particle-shaped masks, respectively. The fifth column displays the 3D projections of the enhanced and IsoNet missing-wedge-corrected particle. The sixth and seventh columns present the final 3D map and the flexibly fitted model, respectively. (B) Two cross-sectional views (12 nm thickness) of the plasmid density map along its plectoneme axis are shown in the left-middle panel. The intensity profile along the line crossing the two high-density DNA spots is displayed in the right panel. (C) Resolution assessment of the final 3D map using Fourier shell correlation (FSC). Two criteria are shown: FSC between two half-maps reconstructed from even and odd frames (evaluated at 0.143) and FSC between the final 3D map and the fitted model (evaluated at 0.5). (D) Zoomed-in views of the final 3D density map from panel A, displayed at two contour levels. (E) Superimposition of the high-contour level map from panel D onto its fitted model.

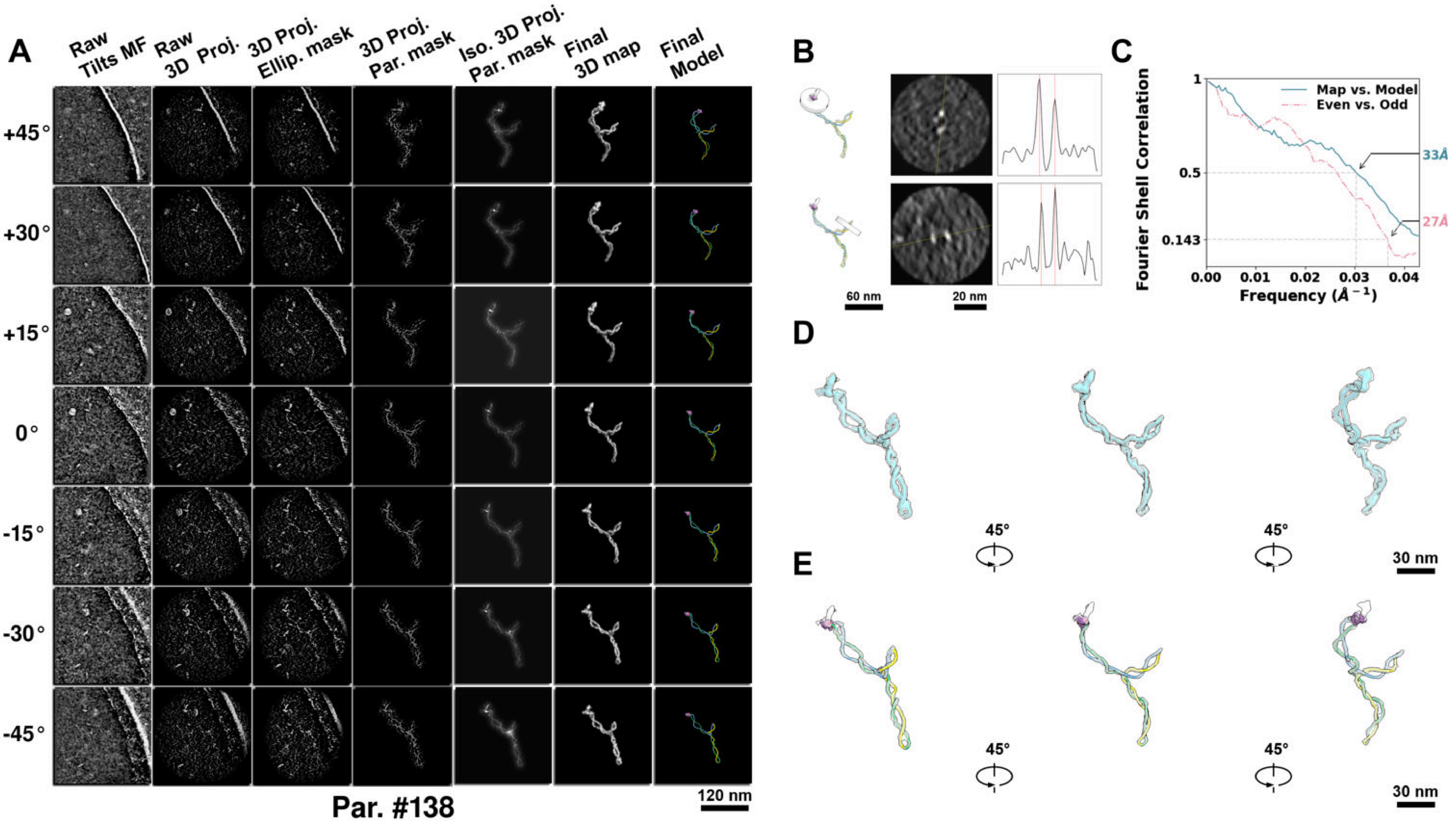

Supplementary Particle Figure 138. Cryo-ET 3D reconstruction of an individual TEC particle.
(A) 3D reconstruction of the plasmid particle (index no. 138). The first column shows seven representative tilt images from +45° to -45° in step of 15°. The second, third, and fourth columns show 3D projections of the particle with spherical, ellipsoidal (thinner along the z-dimension), and particle-shaped masks, respectively. The fifth column displays the 3D projections of the enhanced and IsoNet missing-wedge-corrected particle. The sixth and seventh columns present the final 3D map and the flexibly fitted model, respectively. (B) Two cross-sectional views (12 nm thickness) of the plasmid density map along its plectoneme axis are shown in the left-middle panel. The intensity profile along the line crossing the two high-density DNA spots is displayed in the right panel. (C) Resolution assessment of the final 3D map using Fourier shell correlation (FSC). Two criteria are shown: FSC between two half-maps reconstructed from even and odd frames (evaluated at 0.143) and FSC between the final 3D map and the fitted model (evaluated at 0.5). (D) Zoomed-in views of the final 3D density map from panel A, displayed at two contour levels. (E) Superimposition of the high-contour level map from panel D onto its fitted model.

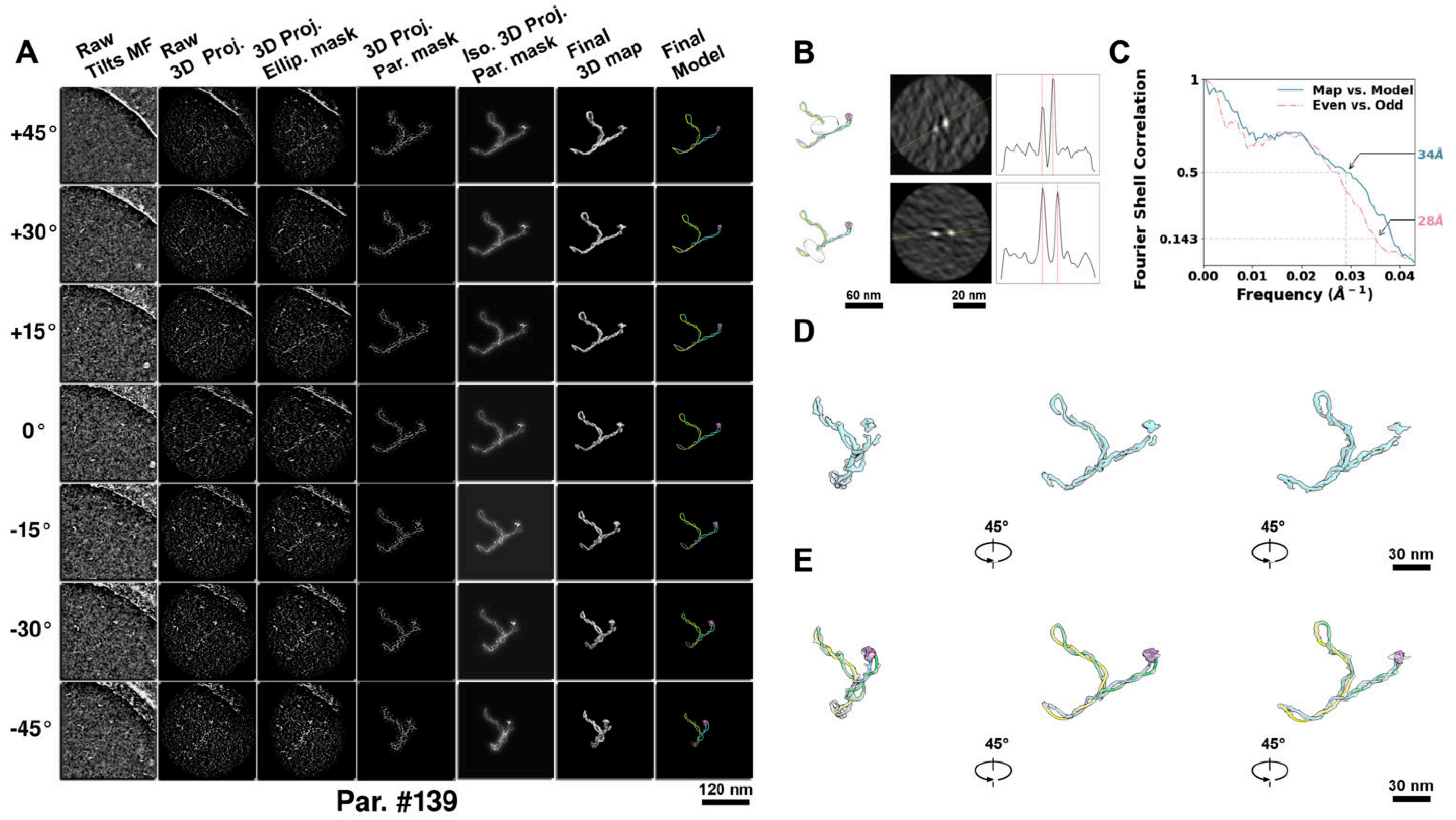

Supplementary Particle Figure 139. Cryo-ET 3D reconstruction of an individual TEC particle.
(A) 3D reconstruction of the plasmid particle (index no. 139). The first column shows seven representative tilt images from +45° to -45° in step of 15°. The second, third, and fourth columns show 3D projections of the particle with spherical, ellipsoidal (thinner along the z-dimension), and particle-shaped masks, respectively. The fifth column displays the 3D projections of the enhanced and IsoNet missing-wedge-corrected particle. The sixth and seventh columns present the final 3D map and the flexibly fitted model, respectively. (B) Two cross-sectional views (12 nm thickness) of the plasmid density map along its plectoneme axis are shown in the left-middle panel. The intensity profile along the line crossing the two high-density DNA spots is displayed in the right panel. (C) Resolution assessment of the final 3D map using Fourier shell correlation (FSC). Two criteria are shown: FSC between two half-maps reconstructed from even and odd frames (evaluated at 0.143) and FSC between the final 3D map and the fitted model (evaluated at 0.5). (D) Zoomed-in views of the final 3D density map from panel A, displayed at two contour levels. (E) Superimposition of the high-contour level map from panel D onto its fitted model.

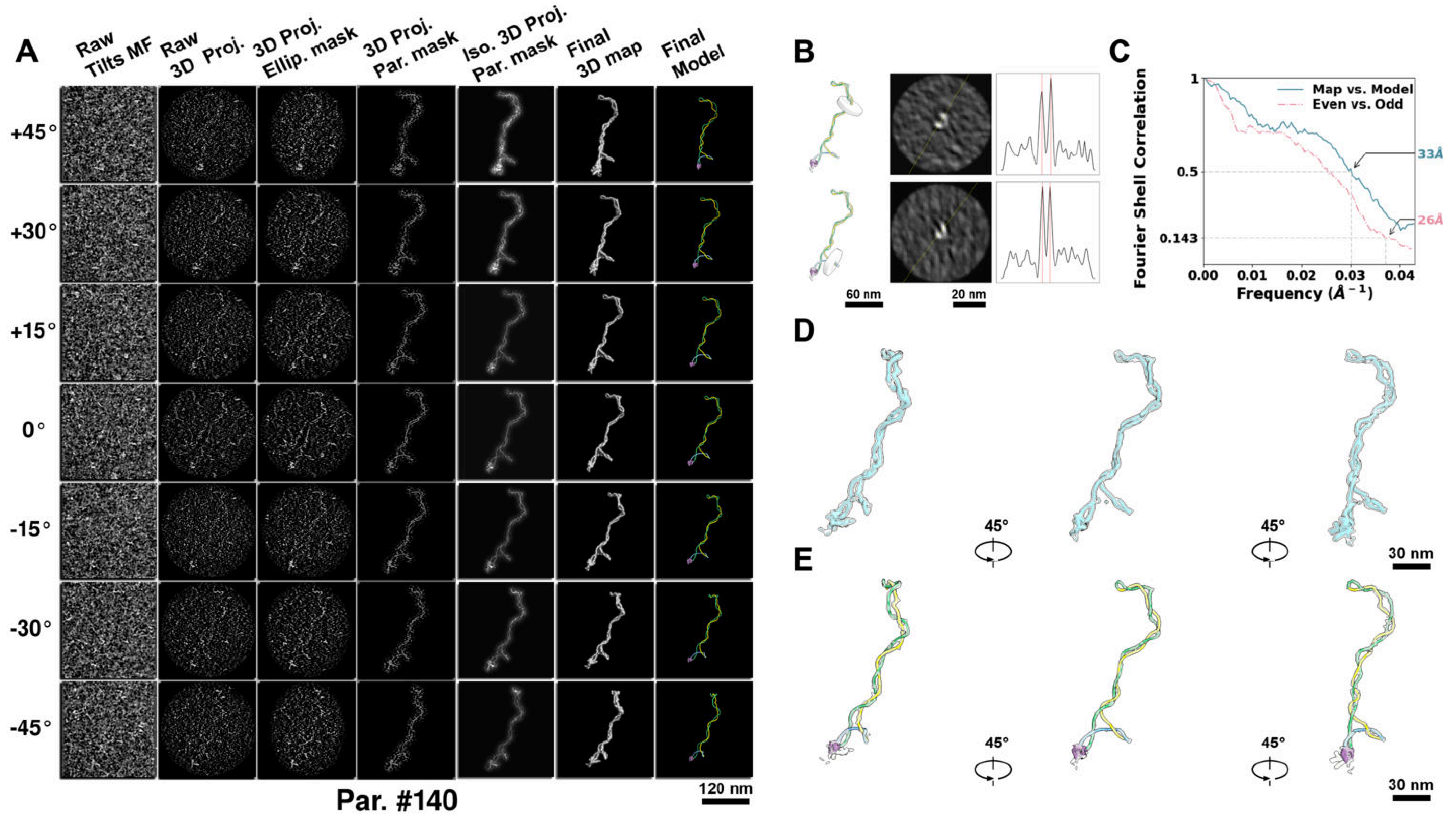

Supplementary Particle Figure 140. Cryo-ET 3D reconstruction of an individual TEC particle.
(A) 3D reconstruction of the plasmid particle (index no. 140). The first column shows seven representative tilt images from +45° to -45° in step of 15°. The second, third, and fourth columns show 3D projections of the particle with spherical, ellipsoidal (thinner along the z-dimension), and particle-shaped masks, respectively. The fifth column displays the 3D projections of the enhanced and IsoNet missing-wedge-corrected particle. The sixth and seventh columns present the final 3D map and the flexibly fitted model, respectively. (B) Two cross-sectional views (12 nm thickness) of the plasmid density map along its plectoneme axis are shown in the left-middle panel. The intensity profile along the line crossing the two high-density DNA spots is displayed in the right panel. (C) Resolution assessment of the final 3D map using Fourier shell correlation (FSC). Two criteria are shown: FSC between two half-maps reconstructed from even and odd frames (evaluated at 0.143) and FSC between the final 3D map and the fitted model (evaluated at 0.5). (D) Zoomed-in views of the final 3D density map from panel A, displayed at two contour levels. (E) Superimposition of the high-contour level map from panel D onto its fitted model.

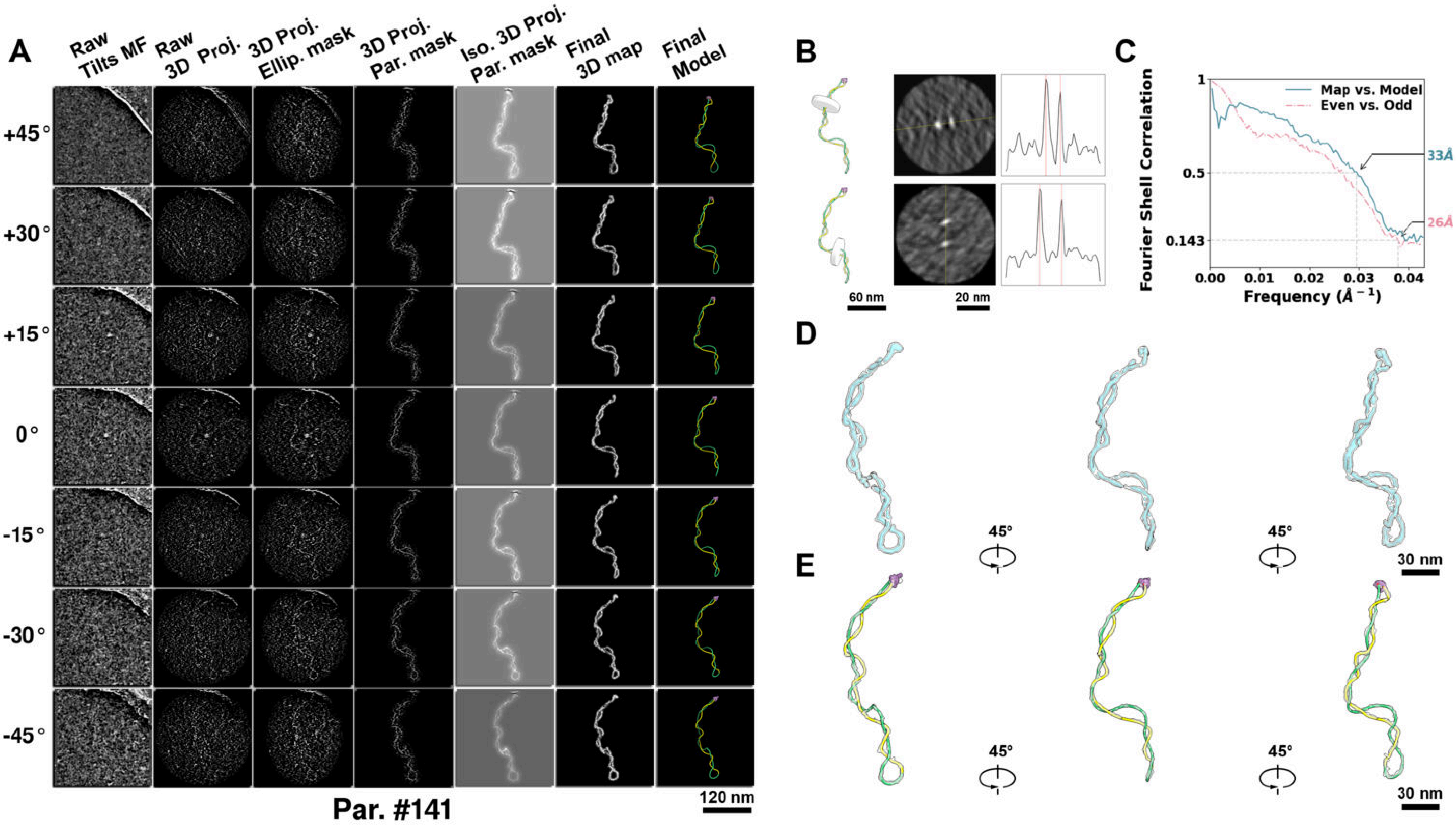

Supplementary Particle Figure 141. Cryo-ET 3D reconstruction of an individual TEC particle.
(A) 3D reconstruction of the plasmid particle (index no. 141). The first column shows seven representative tilt images from +45° to -45° in step of 15°. The second, third, and fourth columns show 3D projections of the particle with spherical, ellipsoidal (thinner along the z-dimension), and particle-shaped masks, respectively. The fifth column displays the 3D projections of the enhanced and IsoNet missing-wedge-corrected particle. The sixth and seventh columns present the final 3D map and the flexibly fitted model, respectively. (B) Two cross-sectional views (12 nm thickness) of the plasmid density map along its plectoneme axis are shown in the left-middle panel. The intensity profile along the line crossing the two high-density DNA spots is displayed in the right panel. (C) Resolution assessment of the final 3D map using Fourier shell correlation (FSC). Two criteria are shown: FSC between two half-maps reconstructed from even and odd frames (evaluated at 0.143) and FSC between the final 3D map and the fitted model (evaluated at 0.5). (D) Zoomed-in views of the final 3D density map from panel A, displayed at two contour levels. (E) Superimposition of the high-contour level map from panel D onto its fitted model.

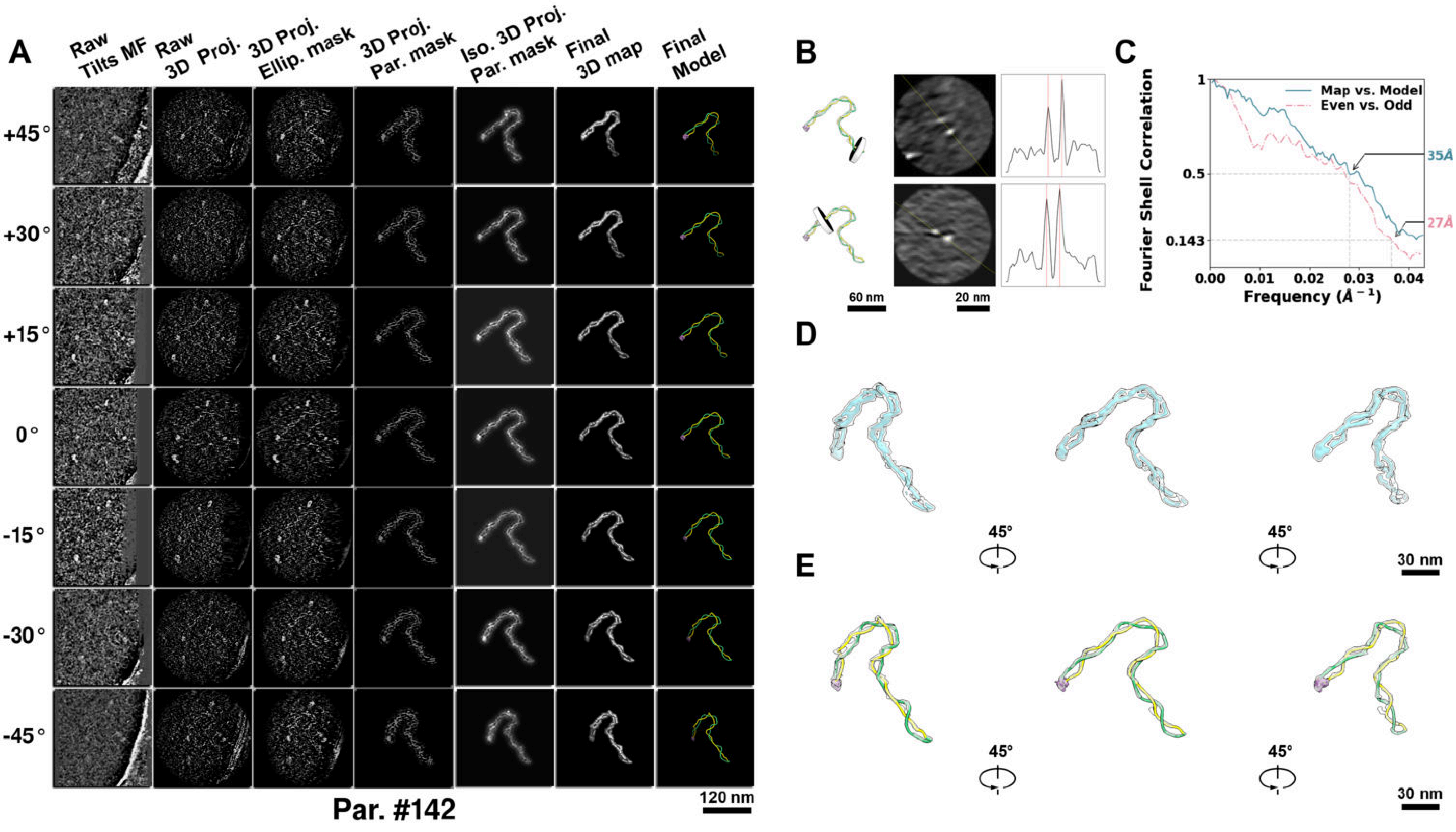

Supplementary Particle Figure 142. Cryo-ET 3D reconstruction of an individual TEC particle.
(A) 3D reconstruction of the plasmid particle (index no. 142). The first column shows seven representative tilt images from +45° to -45° in step of 15°. The second, third, and fourth columns show 3D projections of the particle with spherical, ellipsoidal (thinner along the z-dimension), and particle-shaped masks, respectively. The fifth column displays the 3D projections of the enhanced and IsoNet missing-wedge-corrected particle. The sixth and seventh columns present the final 3D map and the flexibly fitted model, respectively. (B) Two cross-sectional views (12 nm thickness) of the plasmid density map along its plectoneme axis are shown in the left-middle panel. The intensity profile along the line crossing the two high-density DNA spots is displayed in the right panel. (C) Resolution assessment of the final 3D map using Fourier shell correlation (FSC). Two criteria are shown: FSC between two half-maps reconstructed from even and odd frames (evaluated at 0.143) and FSC between the final 3D map and the fitted model (evaluated at 0.5). (D) Zoomed-in views of the final 3D density map from panel A, displayed at two contour levels. (E) Superimposition of the high-contour level map from panel D onto its fitted model.

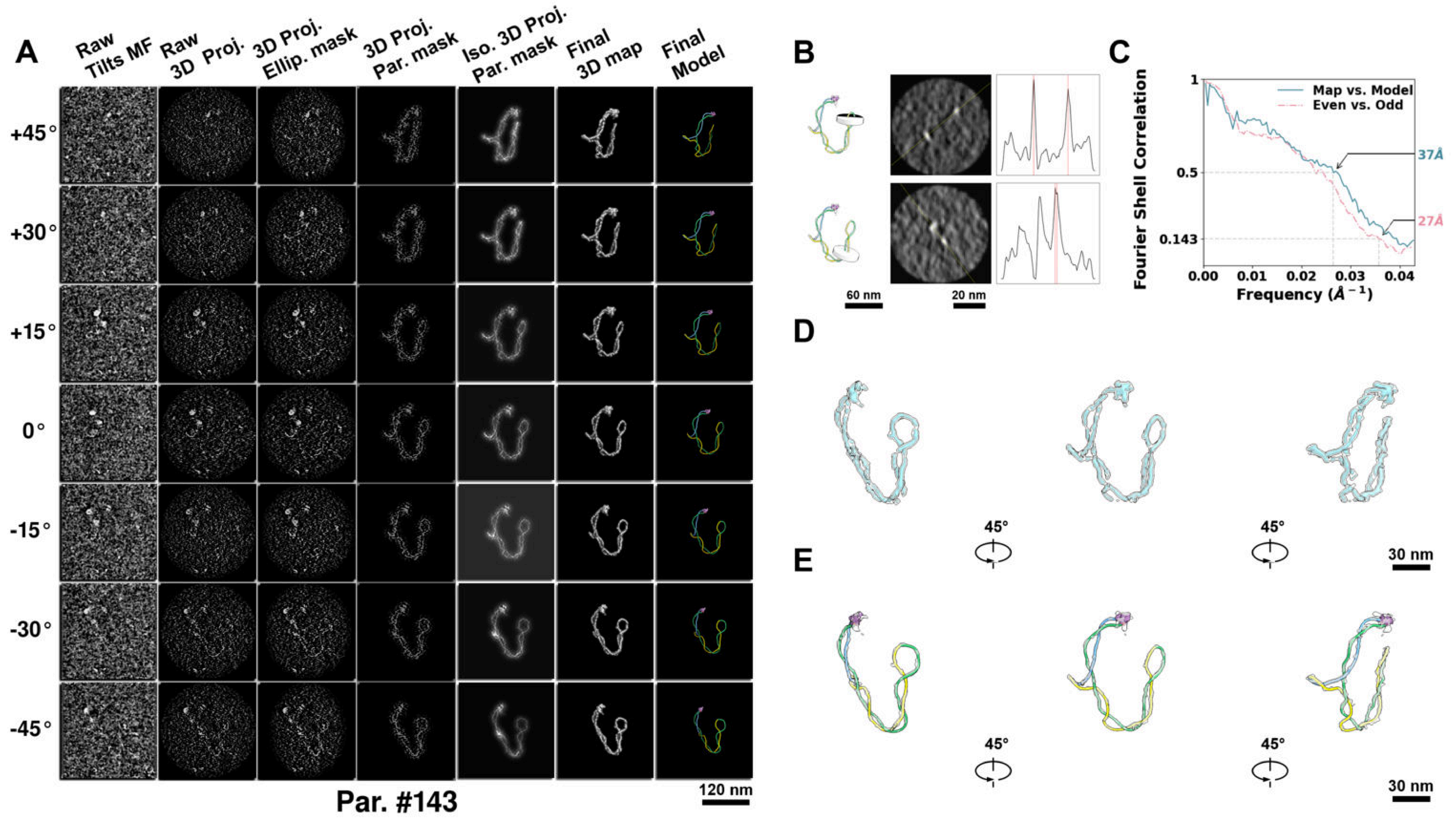

Supplementary Particle Figure 143. Cryo-ET 3D reconstruction of an individual TEC particle.
(A) 3D reconstruction of the plasmid particle (index no. 143). The first column shows seven representative tilt images from +45° to -45° in step of 15°. The second, third, and fourth columns show 3D projections of the particle with spherical, ellipsoidal (thinner along the z-dimension), and particle-shaped masks, respectively. The fifth column displays the 3D projections of the enhanced and IsoNet missing-wedge-corrected particle. The sixth and seventh columns present the final 3D map and the flexibly fitted model, respectively. (B) Two cross-sectional views (12 nm thickness) of the plasmid density map along its plectoneme axis are shown in the left-middle panel. The intensity profile along the line crossing the two high-density DNA spots is displayed in the right panel. (C) Resolution assessment of the final 3D map using Fourier shell correlation (FSC). Two criteria are shown: FSC between two half-maps reconstructed from even and odd frames (evaluated at 0.143) and FSC between the final 3D map and the fitted model (evaluated at 0.5). (D) Zoomed-in views of the final 3D density map from panel A, displayed at two contour levels. (E) Superimposition of the high-contour level map from panel D onto its fitted model.

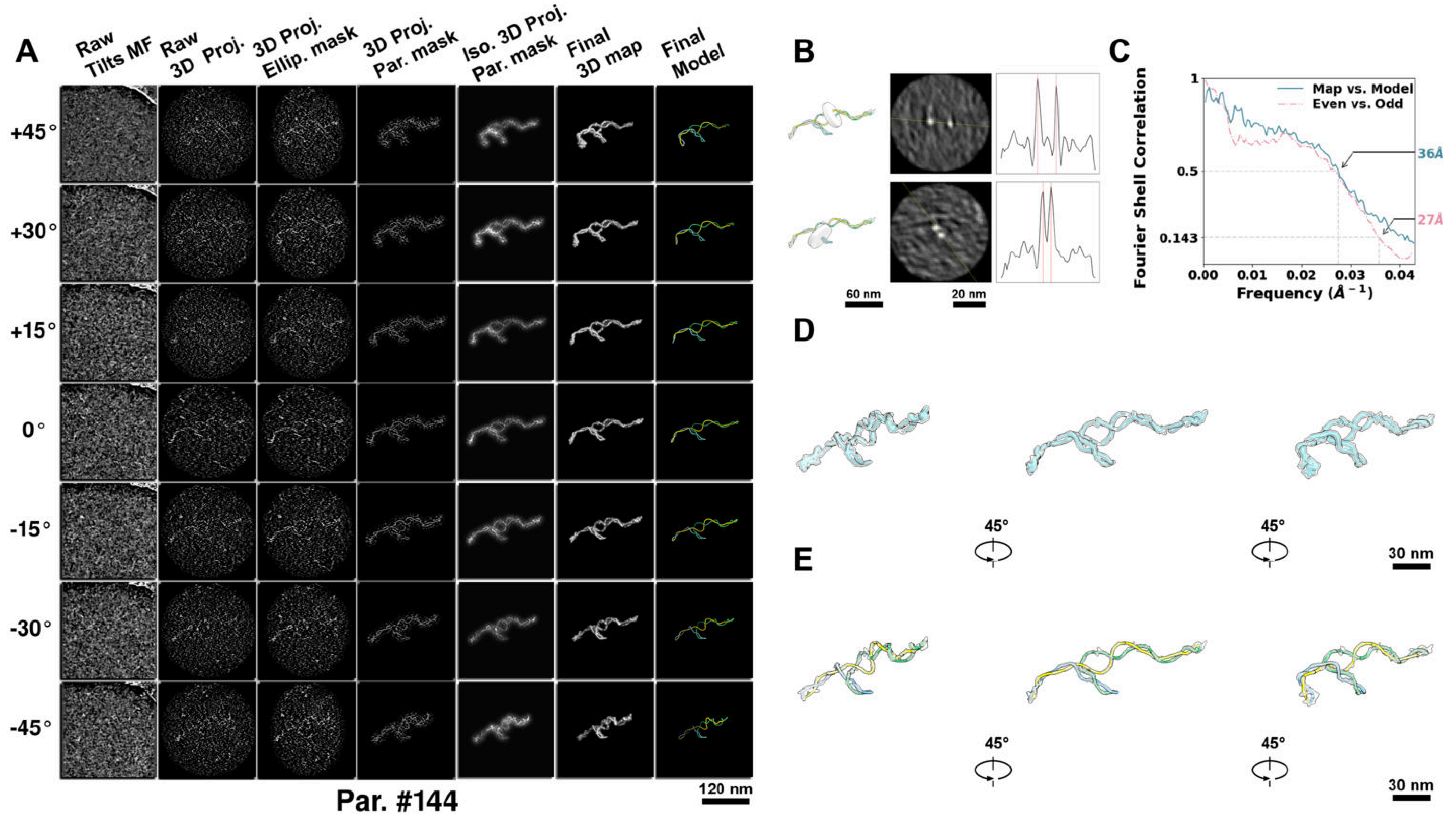

Supplementary Particle Figure 144. Cryo-ET 3D reconstruction of an individual TEC particle.
(A) 3D reconstruction of the plasmid particle (index no. 144). The first column shows seven representative tilt images from +45° to -45° in step of 15°. The second, third, and fourth columns show 3D projections of the particle with spherical, ellipsoidal (thinner along the z-dimension), and particle-shaped masks, respectively. The fifth column displays the 3D projections of the enhanced and IsoNet missing-wedge-corrected particle. The sixth and seventh columns present the final 3D map and the flexibly fitted model, respectively. (B) Two cross-sectional views (12 nm thickness) of the plasmid density map along its plectoneme axis are shown in the left-middle panel. The intensity profile along the line crossing the two high-density DNA spots is displayed in the right panel. (C) Resolution assessment of the final 3D map using Fourier shell correlation (FSC). Two criteria are shown: FSC between two half-maps reconstructed from even and odd frames (evaluated at 0.143) and FSC between the final 3D map and the fitted model (evaluated at 0.5). (D) Zoomed-in views of the final 3D density map from panel A, displayed at two contour levels. (E) Superimposition of the high-contour level map from panel D onto its fitted model.

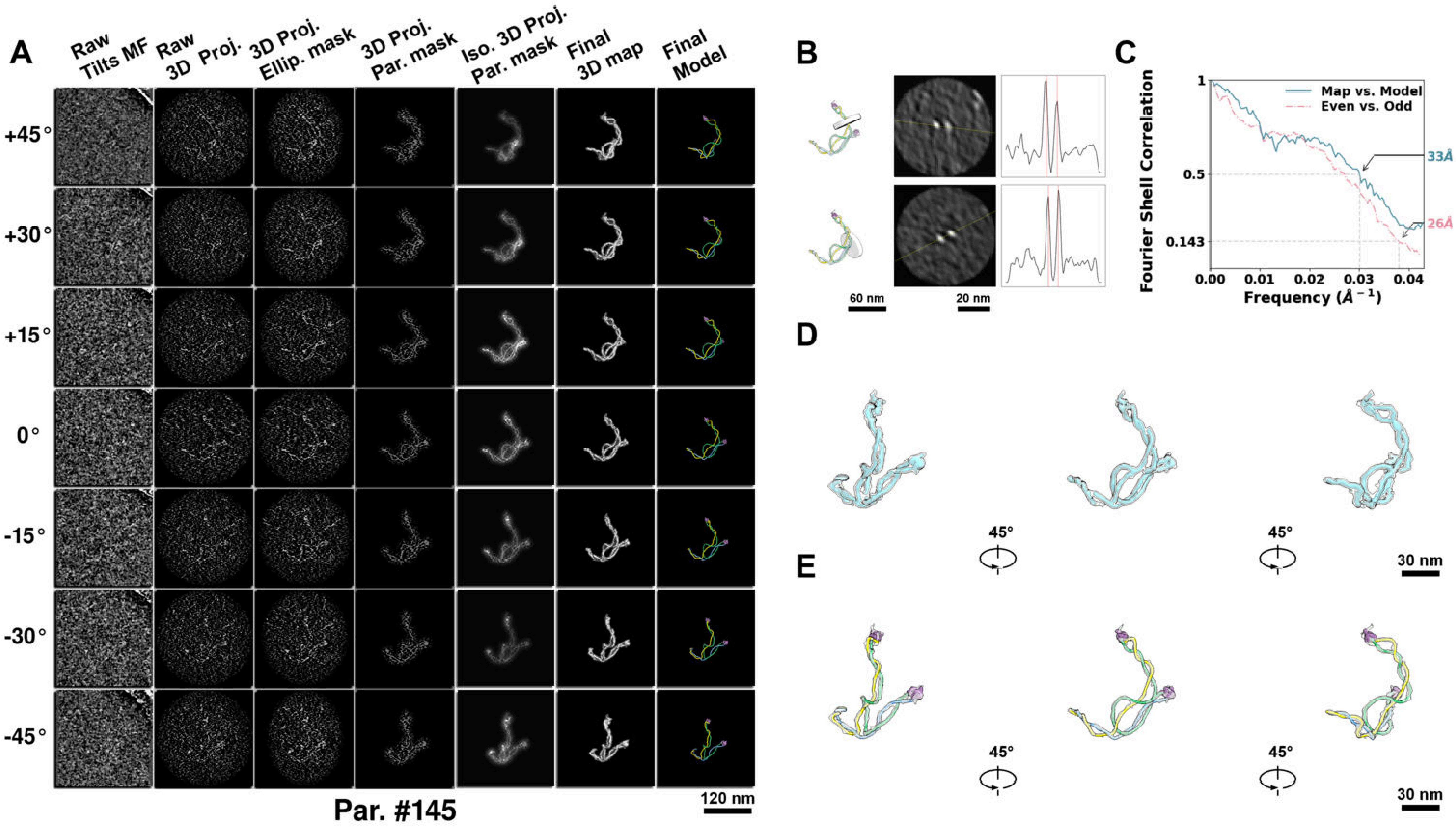

Supplementary Particle Figure 145. Cryo-ET 3D reconstruction of an individual TEC particle.
(A) 3D reconstruction of the plasmid particle (index no. 145). The first column shows seven representative tilt images from +45° to -45° in step of 15°. The second, third, and fourth columns show 3D projections of the particle with spherical, ellipsoidal (thinner along the z-dimension), and particle-shaped masks, respectively. The fifth column displays the 3D projections of the enhanced and IsoNet missing-wedge-corrected particle. The sixth and seventh columns present the final 3D map and the flexibly fitted model, respectively. (B) Two cross-sectional views (12 nm thickness) of the plasmid density map along its plectoneme axis are shown in the left-middle panel. The intensity profile along the line crossing the two high-density DNA spots is displayed in the right panel. (C) Resolution assessment of the final 3D map using Fourier shell correlation (FSC). Two criteria are shown: FSC between two half-maps reconstructed from even and odd frames (evaluated at 0.143) and FSC between the final 3D map and the fitted model (evaluated at 0.5). (D) Zoomed-in views of the final 3D density map from panel A, displayed at two contour levels. (E) Superimposition of the high-contour level map from panel D onto its fitted model.

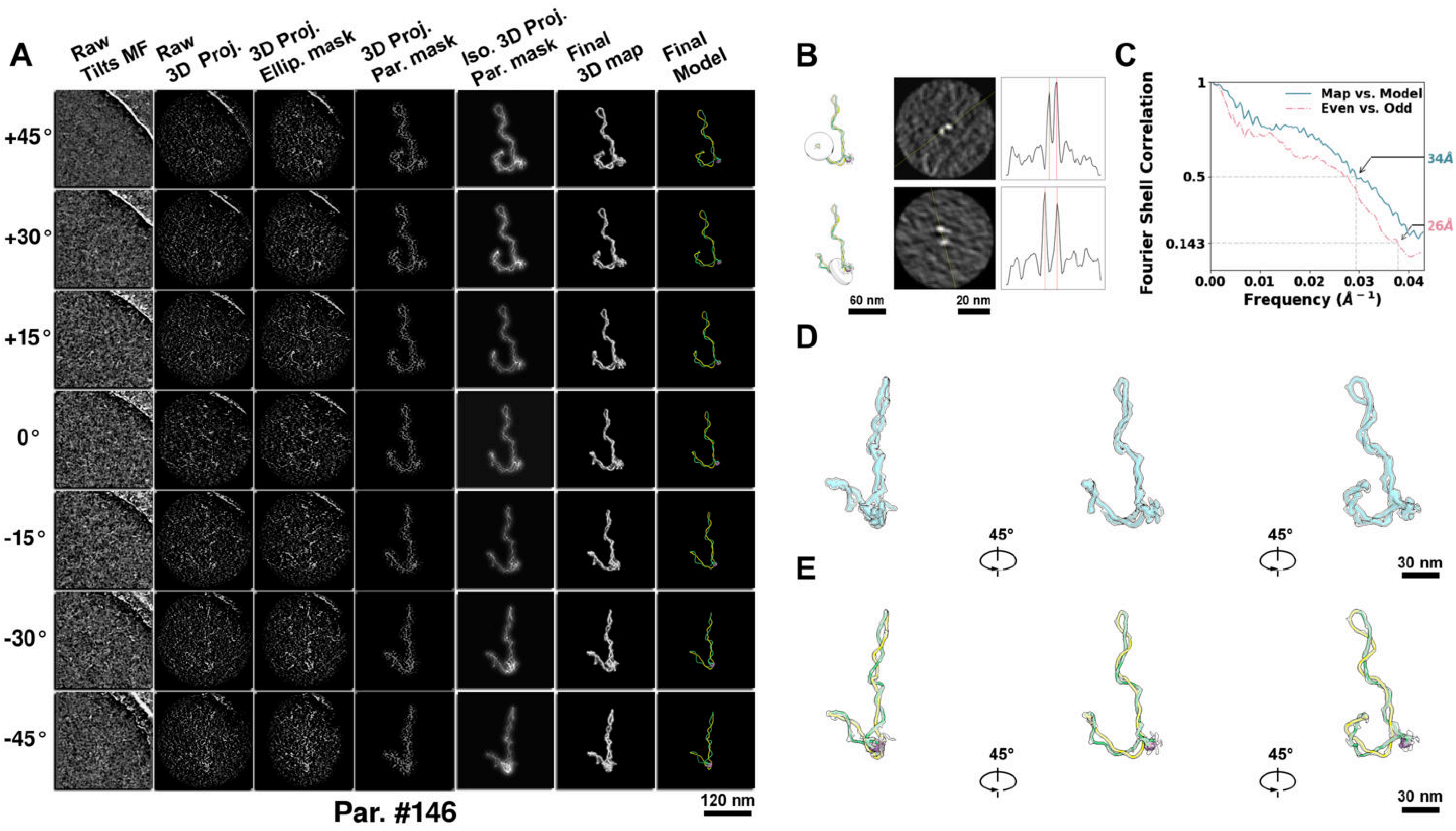

Supplementary Particle Figure 146. Cryo-ET 3D reconstruction of an individual TEC particle.
(A) 3D reconstruction of the plasmid particle (index no. 146). The first column shows seven representative tilt images from +45° to -45° in step of 15°. The second, third, and fourth columns show 3D projections of the particle with spherical, ellipsoidal (thinner along the z-dimension), and particle-shaped masks, respectively. The fifth column displays the 3D projections of the enhanced and IsoNet missing-wedge-corrected particle. The sixth and seventh columns present the final 3D map and the flexibly fitted model, respectively. (B) Two cross-sectional views (12 nm thickness) of the plasmid density map along its plectoneme axis are shown in the left-middle panel. The intensity profile along the line crossing the two high-density DNA spots is displayed in the right panel. (C) Resolution assessment of the final 3D map using Fourier shell correlation (FSC). Two criteria are shown: FSC between two half-maps reconstructed from even and odd frames (evaluated at 0.143) and FSC between the final 3D map and the fitted model (evaluated at 0.5). (D) Zoomed-in views of the final 3D density map from panel A, displayed at two contour levels. (E) Superimposition of the high-contour level map from panel D onto its fitted model.

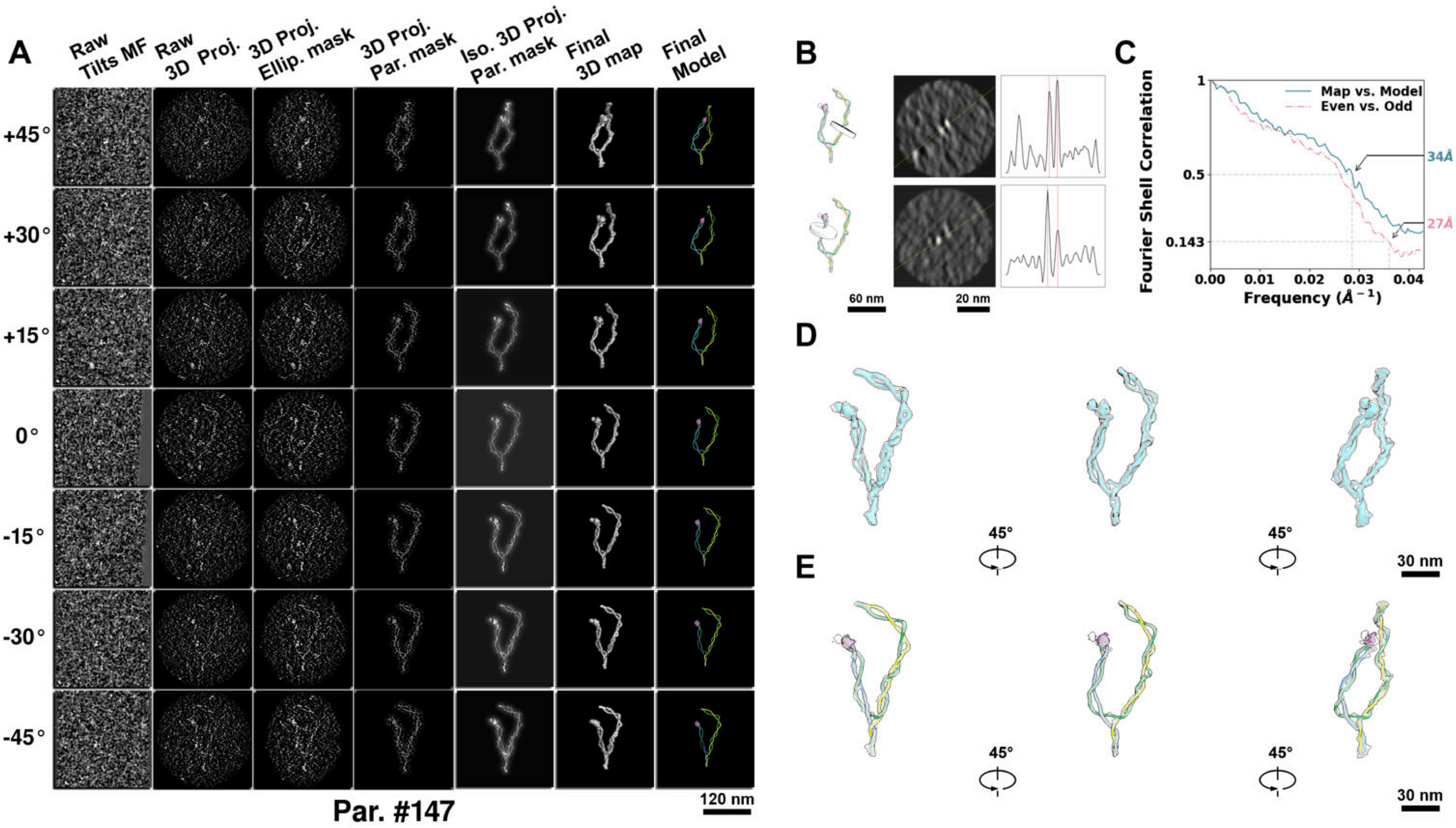

Supplementary Particle Figure 147. Cryo-ET 3D reconstruction of an individual TEC particle.
(A) 3D reconstruction of the plasmid particle (index no. 147). The first column shows seven representative tilt images from +45° to -45° in step of 15°. The second, third, and fourth columns show 3D projections of the particle with spherical, ellipsoidal (thinner along the z-dimension), and particle-shaped masks, respectively. The fifth column displays the 3D projections of the enhanced and IsoNet missing-wedge-corrected particle. The sixth and seventh columns present the final 3D map and the flexibly fitted model, respectively. (B) Two cross-sectional views (12 nm thickness) of the plasmid density map along its plectoneme axis are shown in the left-middle panel. The intensity profile along the line crossing the two high-density DNA spots is displayed in the right panel. (C) Resolution assessment of the final 3D map using Fourier shell correlation (FSC). Two criteria are shown: FSC between two half-maps reconstructed from even and odd frames (evaluated at 0.143) and FSC between the final 3D map and the fitted model (evaluated at 0.5). (D) Zoomed-in views of the final 3D density map from panel A, displayed at two contour levels. (E) Superimposition of the high-contour level map from panel D onto its fitted model.

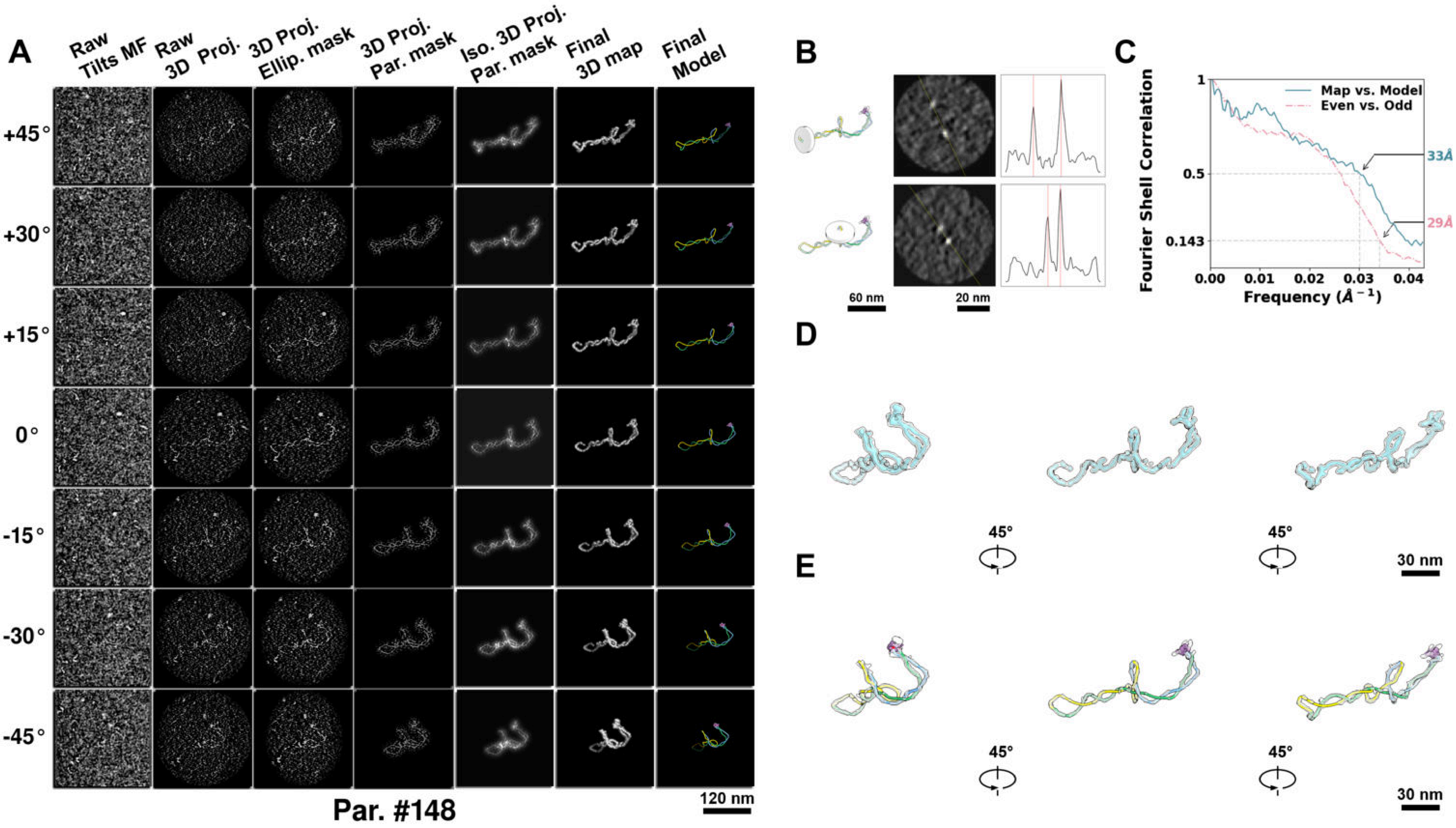

Supplementary Particle Figure 148. Cryo-ET 3D reconstruction of an individual TEC particle.
(A) 3D reconstruction of the plasmid particle (index no. 148). The first column shows seven representative tilt images from +45° to -45° in step of 15°. The second, third, and fourth columns show 3D projections of the particle with spherical, ellipsoidal (thinner along the z-dimension), and particle-shaped masks, respectively. The fifth column displays the 3D projections of the enhanced and IsoNet missing-wedge-corrected particle. The sixth and seventh columns present the final 3D map and the flexibly fitted model, respectively. (B) Two cross-sectional views (12 nm thickness) of the plasmid density map along its plectoneme axis are shown in the left-middle panel. The intensity profile along the line crossing the two high-density DNA spots is displayed in the right panel. (C) Resolution assessment of the final 3D map using Fourier shell correlation (FSC). Two criteria are shown: FSC between two half-maps reconstructed from even and odd frames (evaluated at 0.143) and FSC between the final 3D map and the fitted model (evaluated at 0.5). (D) Zoomed-in views of the final 3D density map from panel A, displayed at two contour levels. (E) Superimposition of the high-contour level map from panel D onto its fitted model.

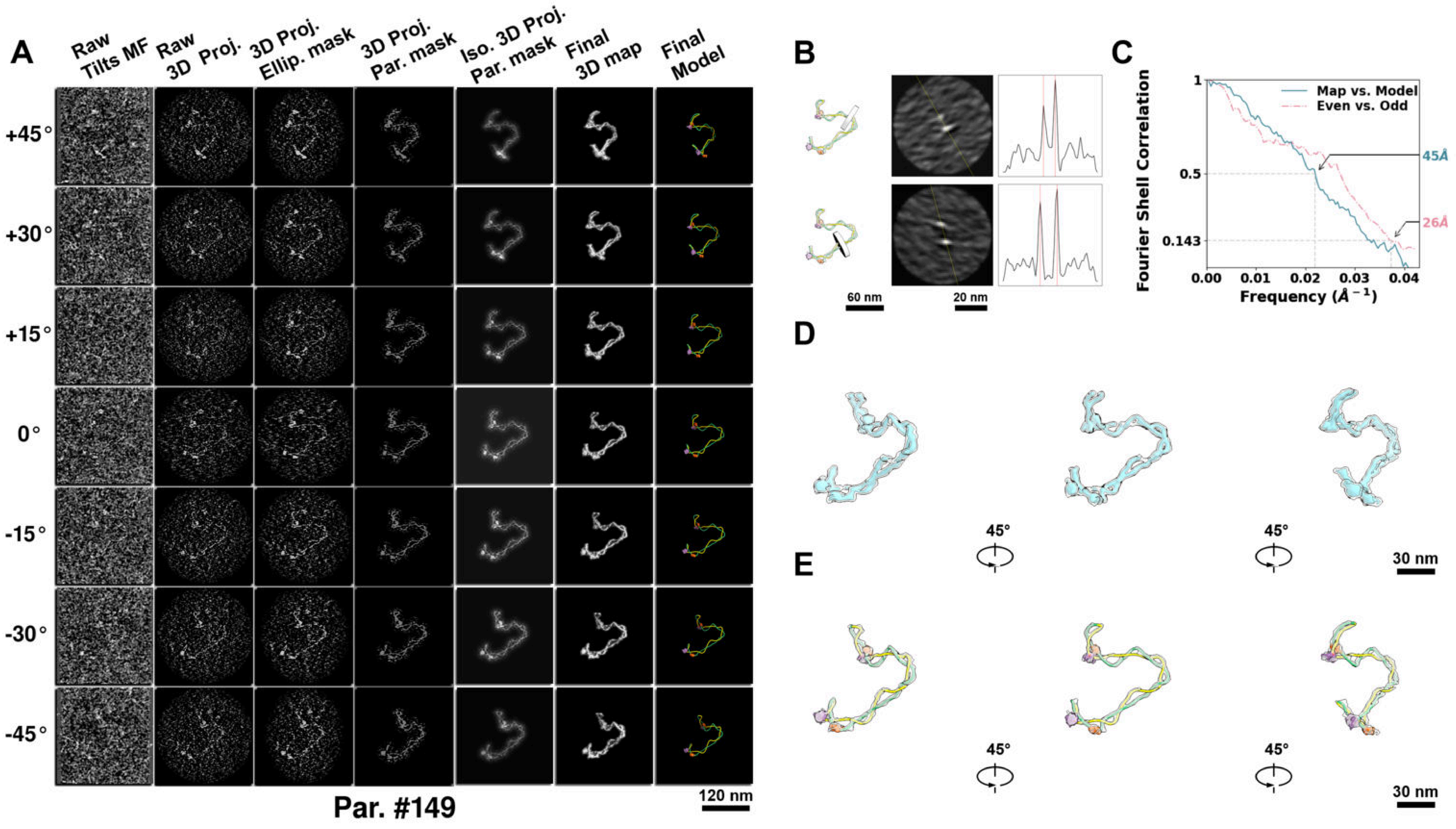

Supplementary Particle Figure 149. Cryo-ET 3D reconstruction of an individual sTEC-Cas particle.
(A) 3D reconstruction of the plasmid particle (index no. 149). The first column shows seven representative tilt images from +45° to -45° in step of 15°. The second, third, and fourth columns show 3D projections of the particle with spherical, ellipsoidal (thinner along the z-dimension), and particle-shaped masks, respectively. The fifth column displays the 3D projections of the enhanced and IsoNet missing-wedge-corrected particle. The sixth and seventh columns present the final 3D map and the flexibly fitted model, respectively. (B) Two cross-sectional views (12 nm thickness) of the plasmid density map along its plectoneme axis are shown in the left-middle panel. The intensity profile along the line crossing the two high-density DNA spots is displayed in the right panel. (C) Resolution assessment of the final 3D map using Fourier shell correlation (FSC). Two criteria are shown: FSC between two half-maps reconstructed from even and odd frames (evaluated at 0.143) and FSC between the final 3D map and the fitted model (evaluated at 0.5). (D) Zoomed-in views of the final 3D density map from panel A, displayed at two contour levels. (E) Superimposition of the high-contour level map from panel D onto its fitted model.

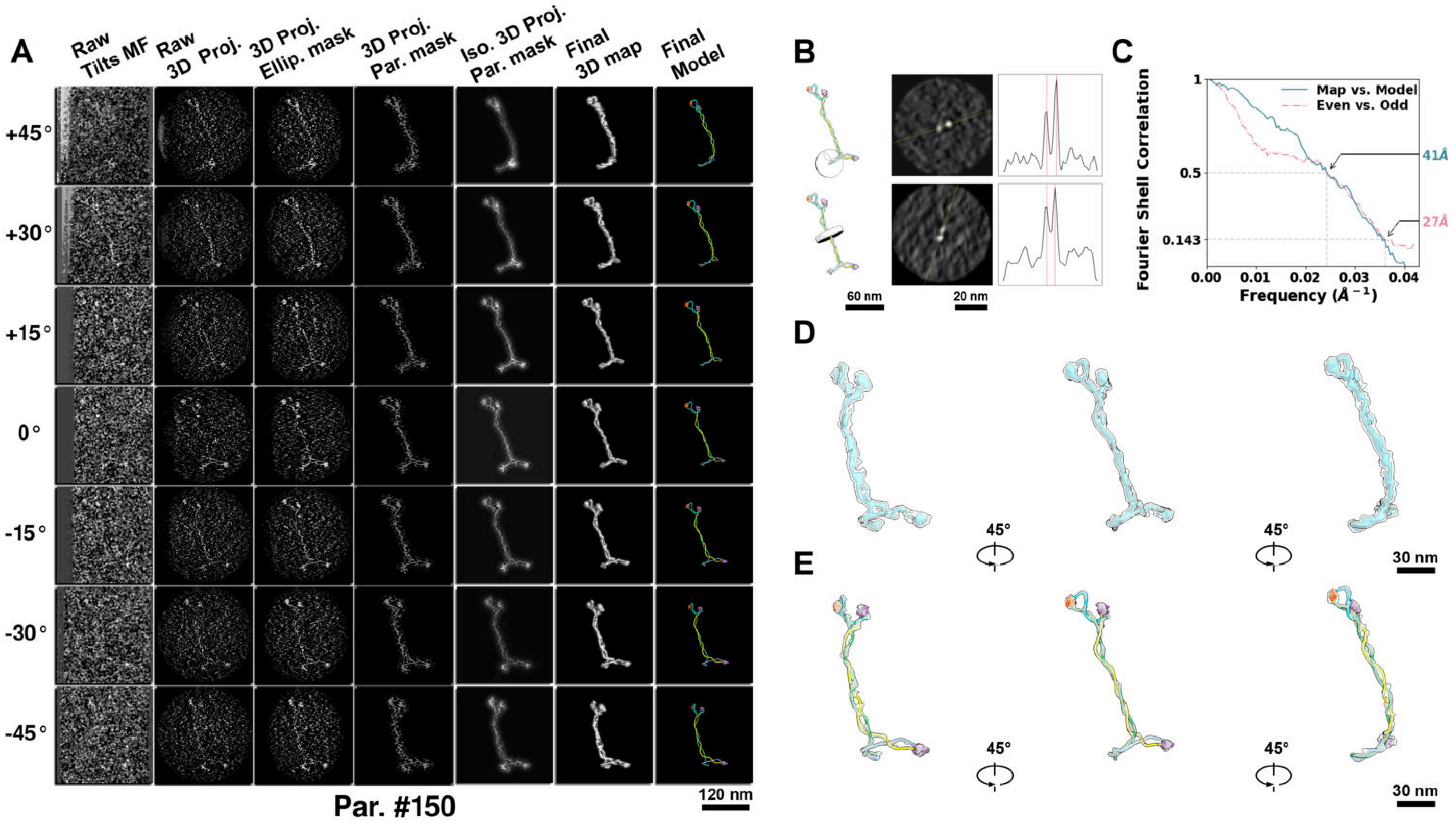

Supplementary Particle Figure 150. Cryo-ET 3D reconstruction of an individual sTEC-Cas particle.
(A) 3D reconstruction of the plasmid particle (index no. 150). The first column shows seven representative tilt images from +45° to -45° in step of 15°. The second, third, and fourth columns show 3D projections of the particle with spherical, ellipsoidal (thinner along the z-dimension), and particle-shaped masks, respectively. The fifth column displays the 3D projections of the enhanced and IsoNet missing-wedge-corrected particle. The sixth and seventh columns present the final 3D map and the flexibly fitted model, respectively. (B) Two cross-sectional views (12 nm thickness) of the plasmid density map along its plectoneme axis are shown in the left-middle panel. The intensity profile along the line crossing the two high-density DNA spots is displayed in the right panel. (C) Resolution assessment of the final 3D map using Fourier shell correlation (FSC). Two criteria are shown: FSC between two half-maps reconstructed from even and odd frames (evaluated at 0.143) and FSC between the final 3D map and the fitted model (evaluated at 0.5). (D) Zoomed-in views of the final 3D density map from panel A, displayed at two contour levels. (E) Superimposition of the high-contour level map from panel D onto its fitted model.

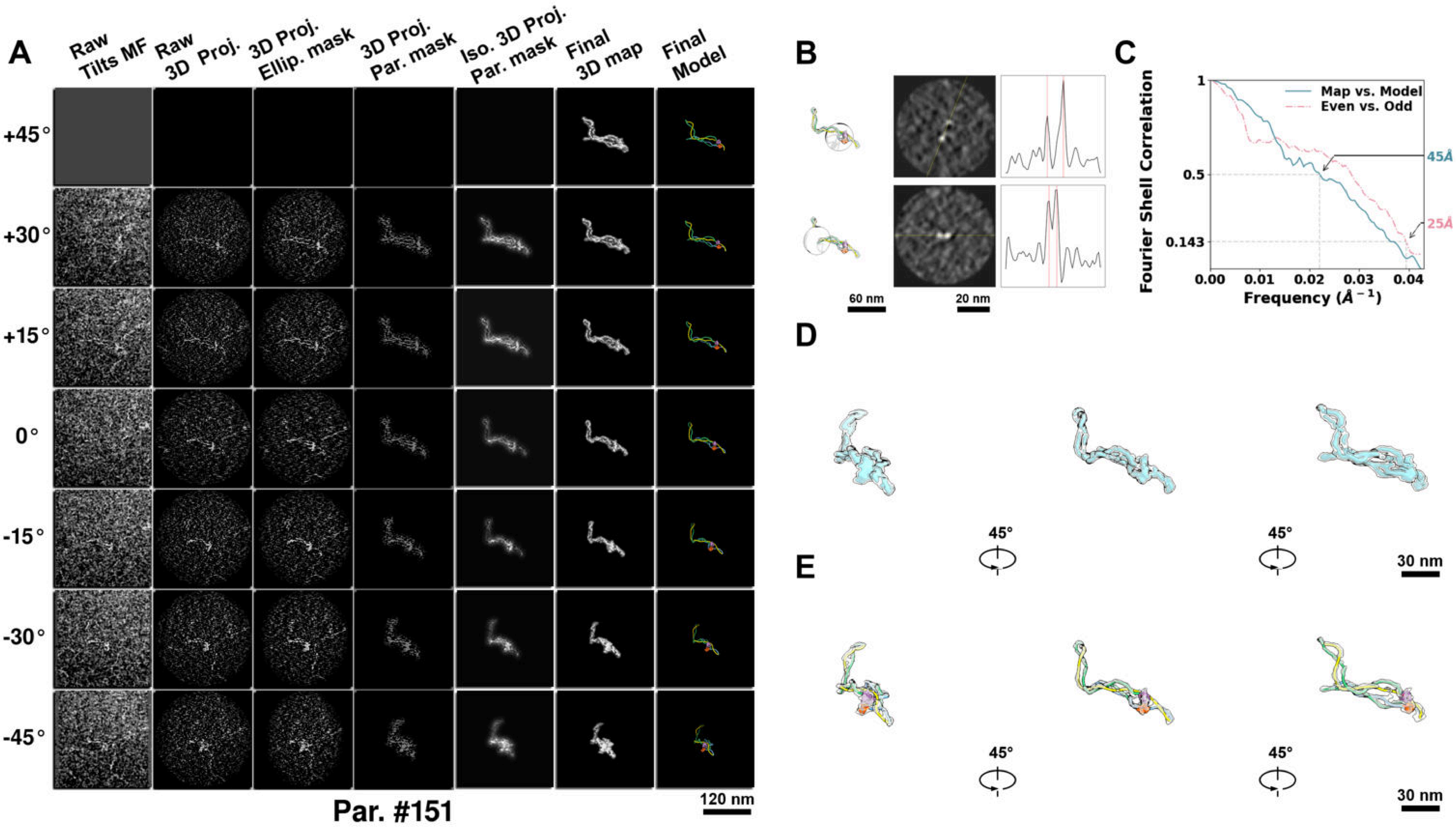

Supplementary Particle Figure 151. Cryo-ET 3D reconstruction of an individual sTEC-Cas particle.
(A) 3D reconstruction of the plasmid particle (index no. 151). The first column shows seven representative tilt images from +45° to -45° in step of 15°. The second, third, and fourth columns show 3D projections of the particle with spherical, ellipsoidal (thinner along the z-dimension), and particle-shaped masks, respectively. The fifth column displays the 3D projections of the enhanced and IsoNet missing-wedge-corrected particle. The sixth and seventh columns present the final 3D map and the flexibly fitted model, respectively. (B) Two cross-sectional views (12 nm thickness) of the plasmid density map along its plectoneme axis are shown in the left-middle panel. The intensity profile along the line crossing the two high-density DNA spots is displayed in the right panel. (C) Resolution assessment of the final 3D map using Fourier shell correlation (FSC). Two criteria are shown: FSC between two half-maps reconstructed from even and odd frames (evaluated at 0.143) and FSC between the final 3D map and the fitted model (evaluated at 0.5). (D) Zoomed-in views of the final 3D density map from panel A, displayed at two contour levels. (E) Superimposition of the high-contour level map from panel D onto its fitted model.

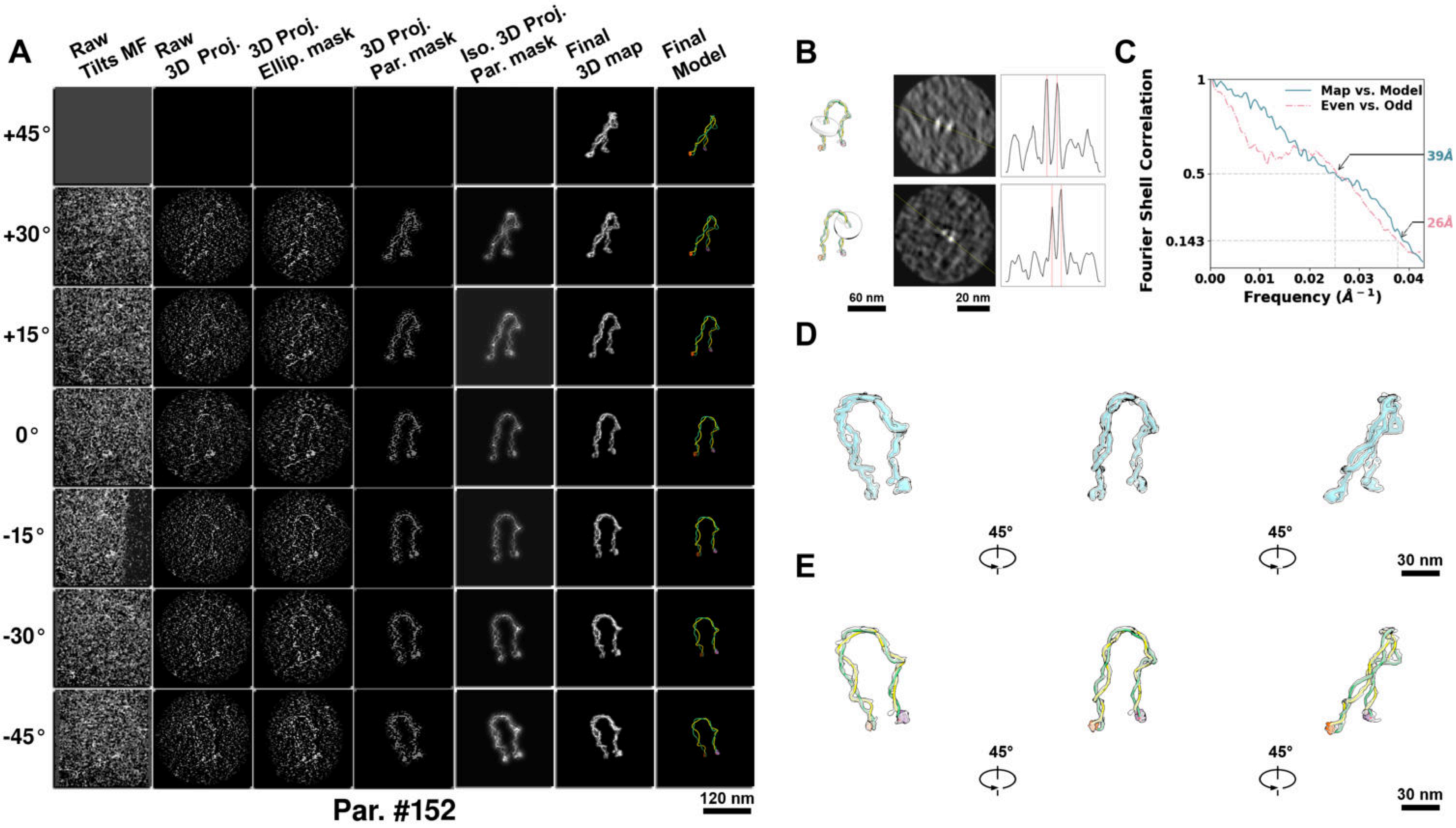

Supplementary Particle Figure 152. Cryo-ET 3D reconstruction of an individual sTEC-Cas particle.
(A) 3D reconstruction of the plasmid particle (index no. 152). The first column shows seven representative tilt images from +45° to -45° in step of 15°. The second, third, and fourth columns show 3D projections of the particle with spherical, ellipsoidal (thinner along the z-dimension), and particle-shaped masks, respectively. The fifth column displays the 3D projections of the enhanced and IsoNet missing-wedge-corrected particle. The sixth and seventh columns present the final 3D map and the flexibly fitted model, respectively. (B) Two cross-sectional views (12 nm thickness) of the plasmid density map along its plectoneme axis are shown in the left-middle panel. The intensity profile along the line crossing the two high-density DNA spots is displayed in the right panel. (C) Resolution assessment of the final 3D map using Fourier shell correlation (FSC). Two criteria are shown: FSC between two half-maps reconstructed from even and odd frames (evaluated at 0.143) and FSC between the final 3D map and the fitted model (evaluated at 0.5). (D) Zoomed-in views of the final 3D density map from panel A, displayed at two contour levels. (E) Superimposition of the high-contour level map from panel D onto its fitted model.

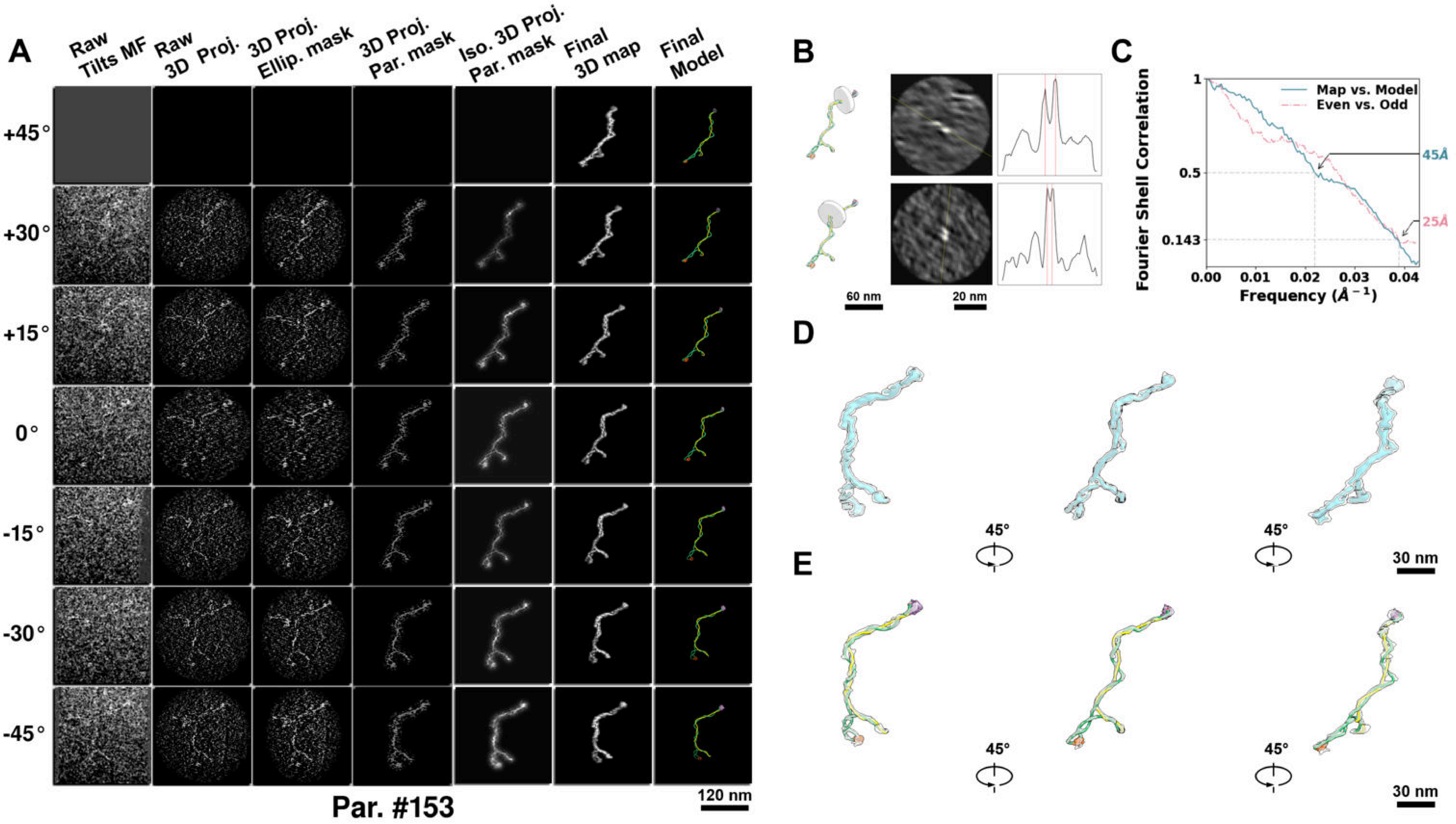

Supplementary Particle Figure 153. Cryo-ET 3D reconstruction of an individual sTEC-Cas particle.
(A) 3D reconstruction of the plasmid particle (index no. 153). The first column shows seven representative tilt images from +45° to -45° in step of 15°. The second, third, and fourth columns show 3D projections of the particle with spherical, ellipsoidal (thinner along the z-dimension), and particle-shaped masks, respectively. The fifth column displays the 3D projections of the enhanced and IsoNet missing-wedge-corrected particle. The sixth and seventh columns present the final 3D map and the flexibly fitted model, respectively. (B) Two cross-sectional views (12 nm thickness) of the plasmid density map along its plectoneme axis are shown in the left-middle panel. The intensity profile along the line crossing the two high-density DNA spots is displayed in the right panel. (C) Resolution assessment of the final 3D map using Fourier shell correlation (FSC). Two criteria are shown: FSC between two half-maps reconstructed from even and odd frames (evaluated at 0.143) and FSC between the final 3D map and the fitted model (evaluated at 0.5). (D) Zoomed-in views of the final 3D density map from panel A, displayed at two contour levels. (E) Superimposition of the high-contour level map from panel D onto its fitted model.

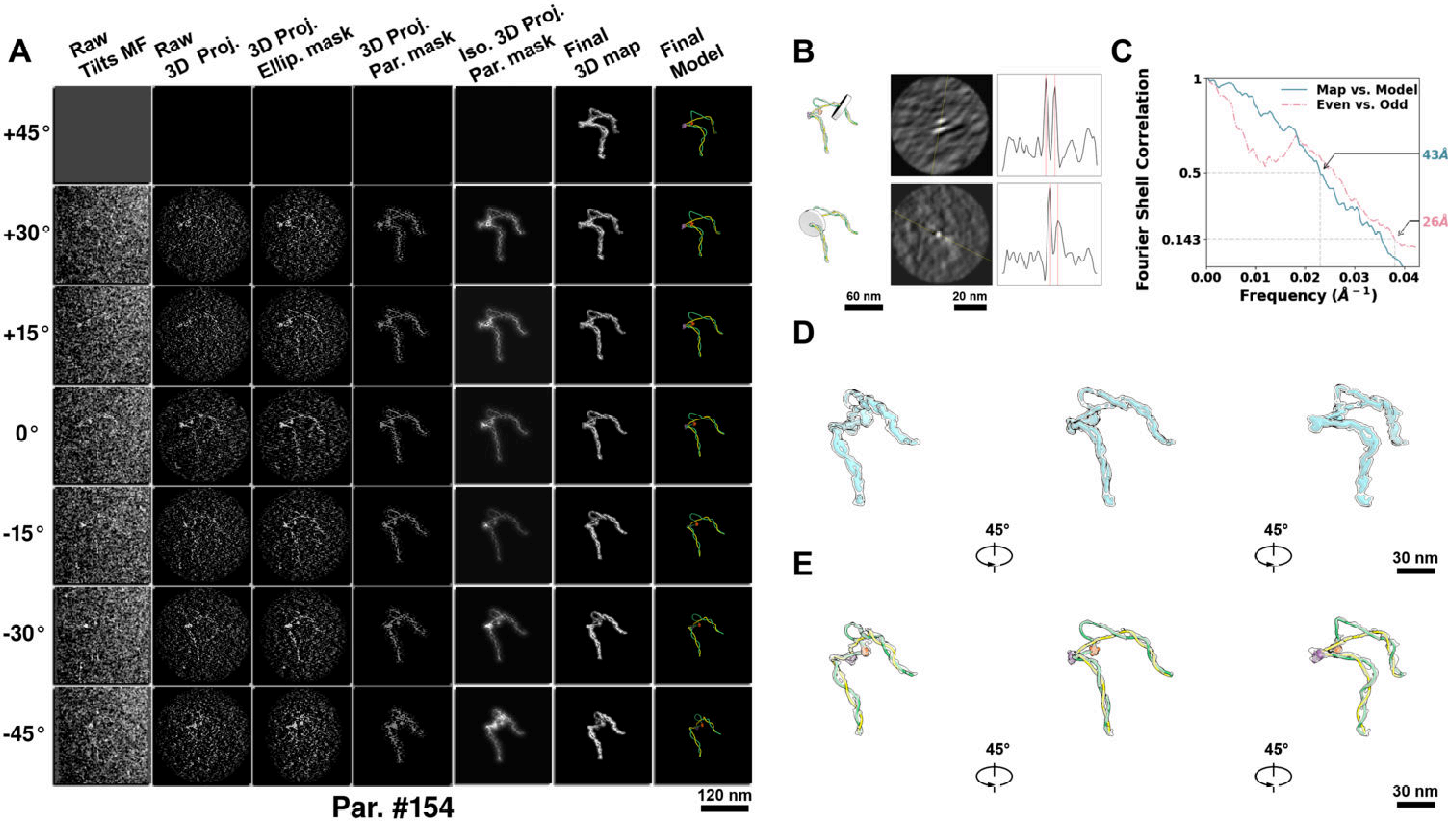

Supplementary Particle Figure 154. Cryo-ET 3D reconstruction of an individual sTEC-Cas particle.
(A) 3D reconstruction of the plasmid particle (index no. 154). The first column shows seven representative tilt images from +45° to -45° in step of 15°. The second, third, and fourth columns show 3D projections of the particle with spherical, ellipsoidal (thinner along the z-dimension), and particle-shaped masks, respectively. The fifth column displays the 3D projections of the enhanced and IsoNet missing-wedge-corrected particle. The sixth and seventh columns present the final 3D map and the flexibly fitted model, respectively. (B) Two cross-sectional views (12 nm thickness) of the plasmid density map along its plectoneme axis are shown in the left-middle panel. The intensity profile along the line crossing the two high-density DNA spots is displayed in the right panel. (C) Resolution assessment of the final 3D map using Fourier shell correlation (FSC). Two criteria are shown: FSC between two half-maps reconstructed from even and odd frames (evaluated at 0.143) and FSC between the final 3D map and the fitted model (evaluated at 0.5). (D) Zoomed-in views of the final 3D density map from panel A, displayed at two contour levels. (E) Superimposition of the high-contour level map from panel D onto its fitted model.

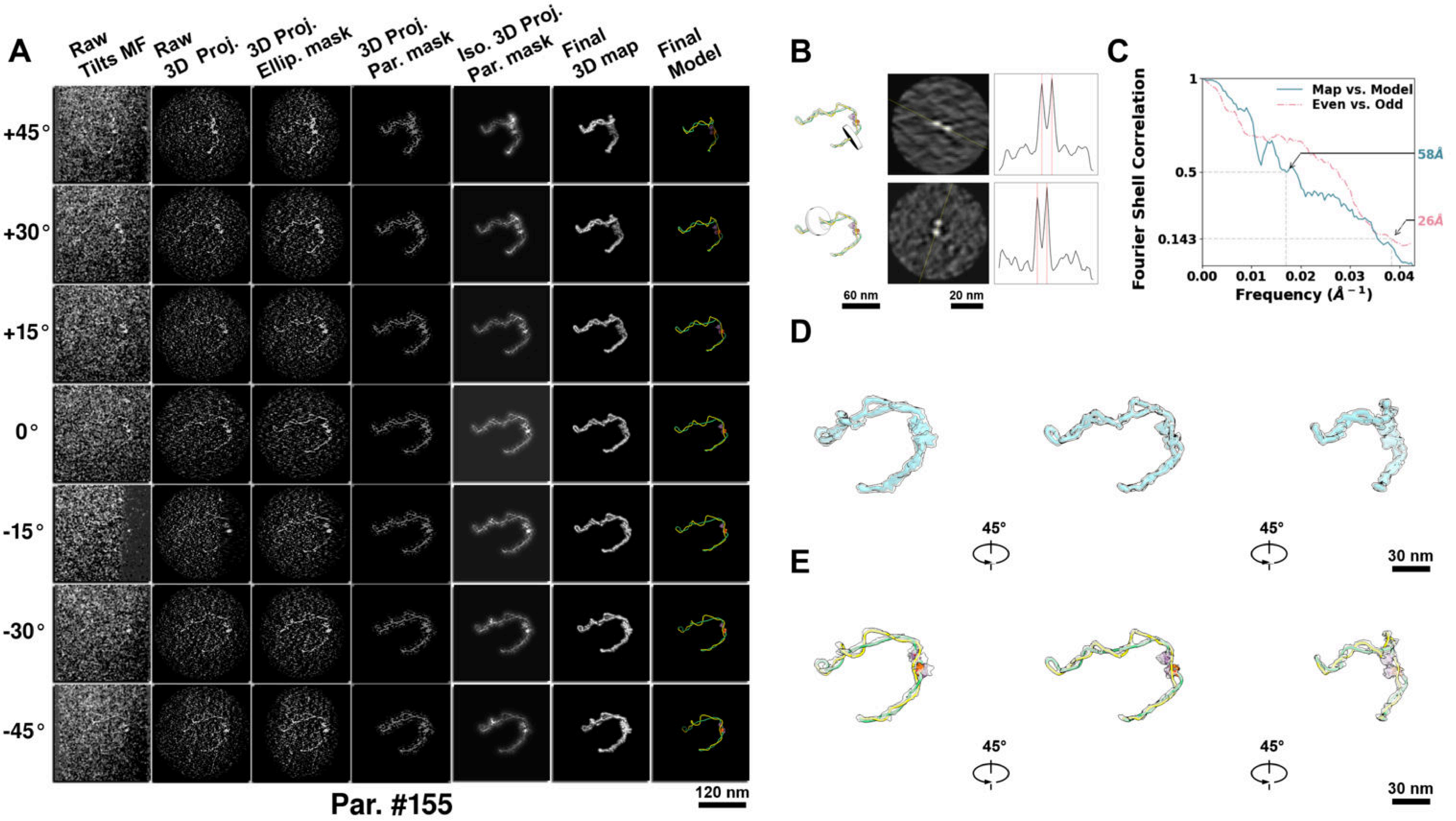

Supplementary Particle Figure 155. Cryo-ET 3D reconstruction of an individual sTEC-Cas particle.
(A) 3D reconstruction of the plasmid particle (index no. 155). The first column shows seven representative tilt images from +45° to -45° in step of 15°. The second, third, and fourth columns show 3D projections of the particle with spherical, ellipsoidal (thinner along the z-dimension), and particle-shaped masks, respectively. The fifth column displays the 3D projections of the enhanced and IsoNet missing-wedge-corrected particle. The sixth and seventh columns present the final 3D map and the flexibly fitted model, respectively. (B) Two cross-sectional views (12 nm thickness) of the plasmid density map along its plectoneme axis are shown in the left-middle panel. The intensity profile along the line crossing the two high-density DNA spots is displayed in the right panel. (C) Resolution assessment of the final 3D map using Fourier shell correlation (FSC). Two criteria are shown: FSC between two half-maps reconstructed from even and odd frames (evaluated at 0.143) and FSC between the final 3D map and the fitted model (evaluated at 0.5). (D) Zoomed-in views of the final 3D density map from panel A, displayed at two contour levels. (E) Superimposition of the high-contour level map from panel D onto its fitted model.

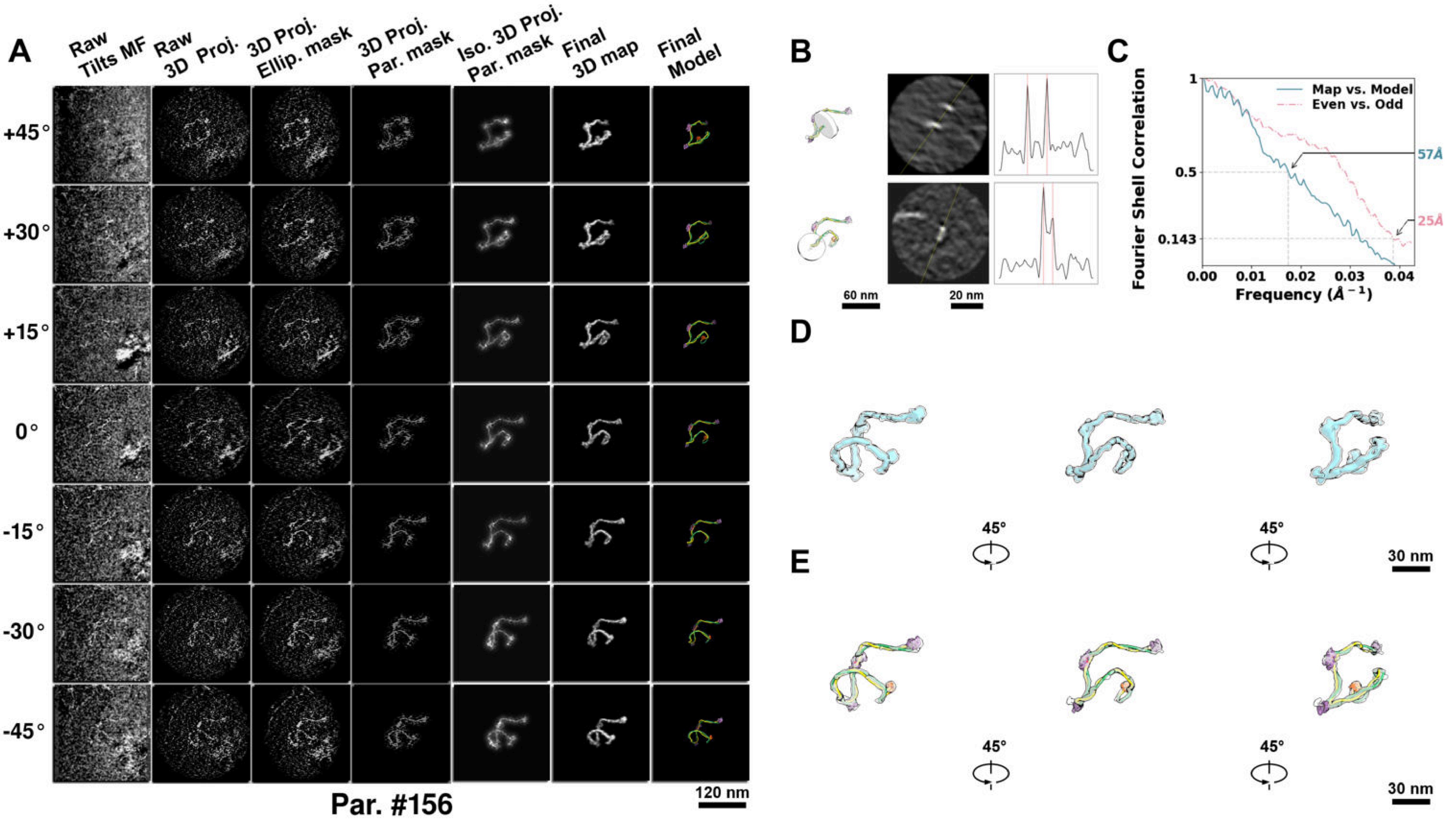

Supplementary Particle Figure 156. Cryo-ET 3D reconstruction of an individual sTEC-Cas particle.
(A) 3D reconstruction of the plasmid particle (index no. 156). The first column shows seven representative tilt images from +45° to -45° in step of 15°. The second, third, and fourth columns show 3D projections of the particle with spherical, ellipsoidal (thinner along the z-dimension), and particle-shaped masks, respectively. The fifth column displays the 3D projections of the enhanced and IsoNet missing-wedge-corrected particle. The sixth and seventh columns present the final 3D map and the flexibly fitted model, respectively. (B) Two cross-sectional views (12 nm thickness) of the plasmid density map along its plectoneme axis are shown in the left-middle panel. The intensity profile along the line crossing the two high-density DNA spots is displayed in the right panel. (C) Resolution assessment of the final 3D map using Fourier shell correlation (FSC). Two criteria are shown: FSC between two half-maps reconstructed from even and odd frames (evaluated at 0.143) and FSC between the final 3D map and the fitted model (evaluated at 0.5). (D) Zoomed-in views of the final 3D density map from panel A, displayed at two contour levels. (E) Superimposition of the high-contour level map from panel D onto its fitted model.

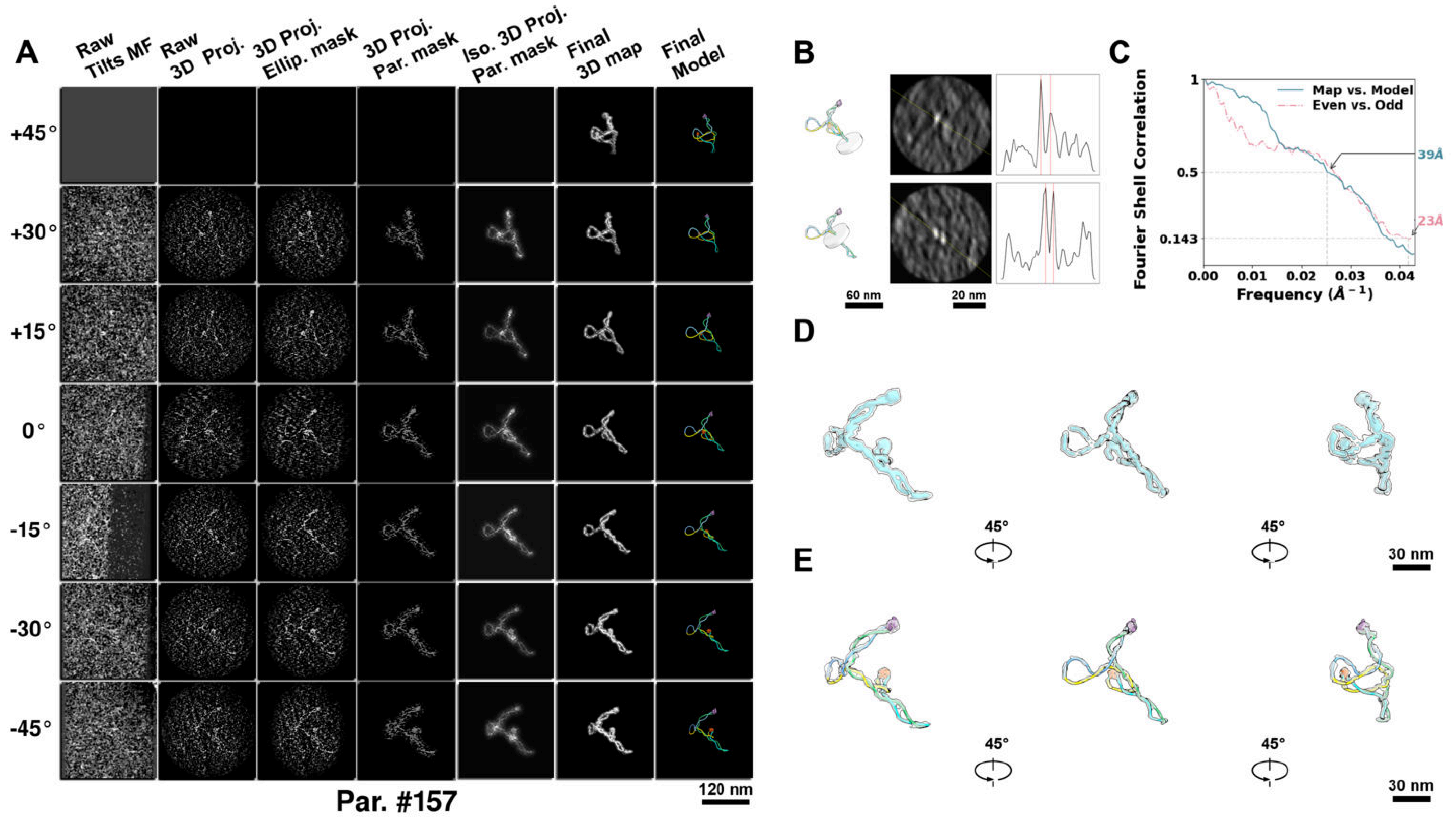

Supplementary Particle Figure 157. Cryo-ET 3D reconstruction of an individual sTEC-Cas particle.
(A) 3D reconstruction of the plasmid particle (index no. 157). The first column shows seven representative tilt images from +45° to -45° in step of 15°. The second, third, and fourth columns show 3D projections of the particle with spherical, ellipsoidal (thinner along the z-dimension), and particle-shaped masks, respectively. The fifth column displays the 3D projections of the enhanced and IsoNet missing-wedge-corrected particle. The sixth and seventh columns present the final 3D map and the flexibly fitted model, respectively. (B) Two cross-sectional views (12 nm thickness) of the plasmid density map along its plectoneme axis are shown in the left-middle panel. The intensity profile along the line crossing the two high-density DNA spots is displayed in the right panel. (C) Resolution assessment of the final 3D map using Fourier shell correlation (FSC). Two criteria are shown: FSC between two half-maps reconstructed from even and odd frames (evaluated at 0.143) and FSC between the final 3D map and the fitted model (evaluated at 0.5). (D) Zoomed-in views of the final 3D density map from panel A, displayed at two contour levels. (E) Superimposition of the high-contour level map from panel D onto its fitted model.

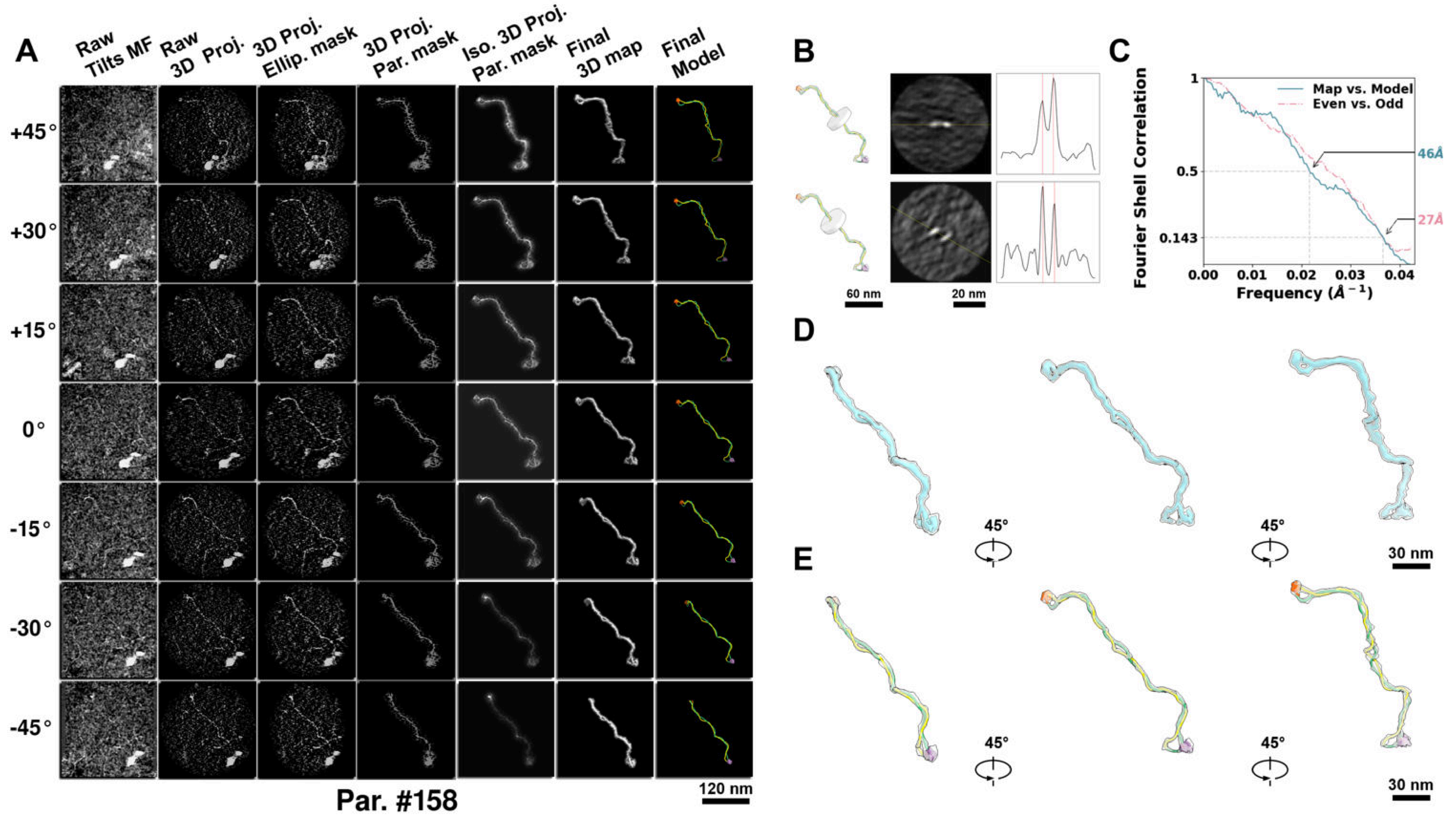

Supplementary Particle Figure 158. Cryo-ET 3D reconstruction of an individual sTEC-Cas particle.
(A) 3D reconstruction of the plasmid particle (index no. 158). The first column shows seven representative tilt images from +45° to -45° in step of 15°. The second, third, and fourth columns show 3D projections of the particle with spherical, ellipsoidal (thinner along the z-dimension), and particle-shaped masks, respectively. The fifth column displays the 3D projections of the enhanced and IsoNet missing-wedge-corrected particle. The sixth and seventh columns present the final 3D map and the flexibly fitted model, respectively. (B) Two cross-sectional views (12 nm thickness) of the plasmid density map along its plectoneme axis are shown in the left-middle panel. The intensity profile along the line crossing the two high-density DNA spots is displayed in the right panel. (C) Resolution assessment of the final 3D map using Fourier shell correlation (FSC). Two criteria are shown: FSC between two half-maps reconstructed from even and odd frames (evaluated at 0.143) and FSC between the final 3D map and the fitted model (evaluated at 0.5). (D) Zoomed-in views of the final 3D density map from panel A, displayed at two contour levels. (E) Superimposition of the high-contour level map from panel D onto its fitted model.

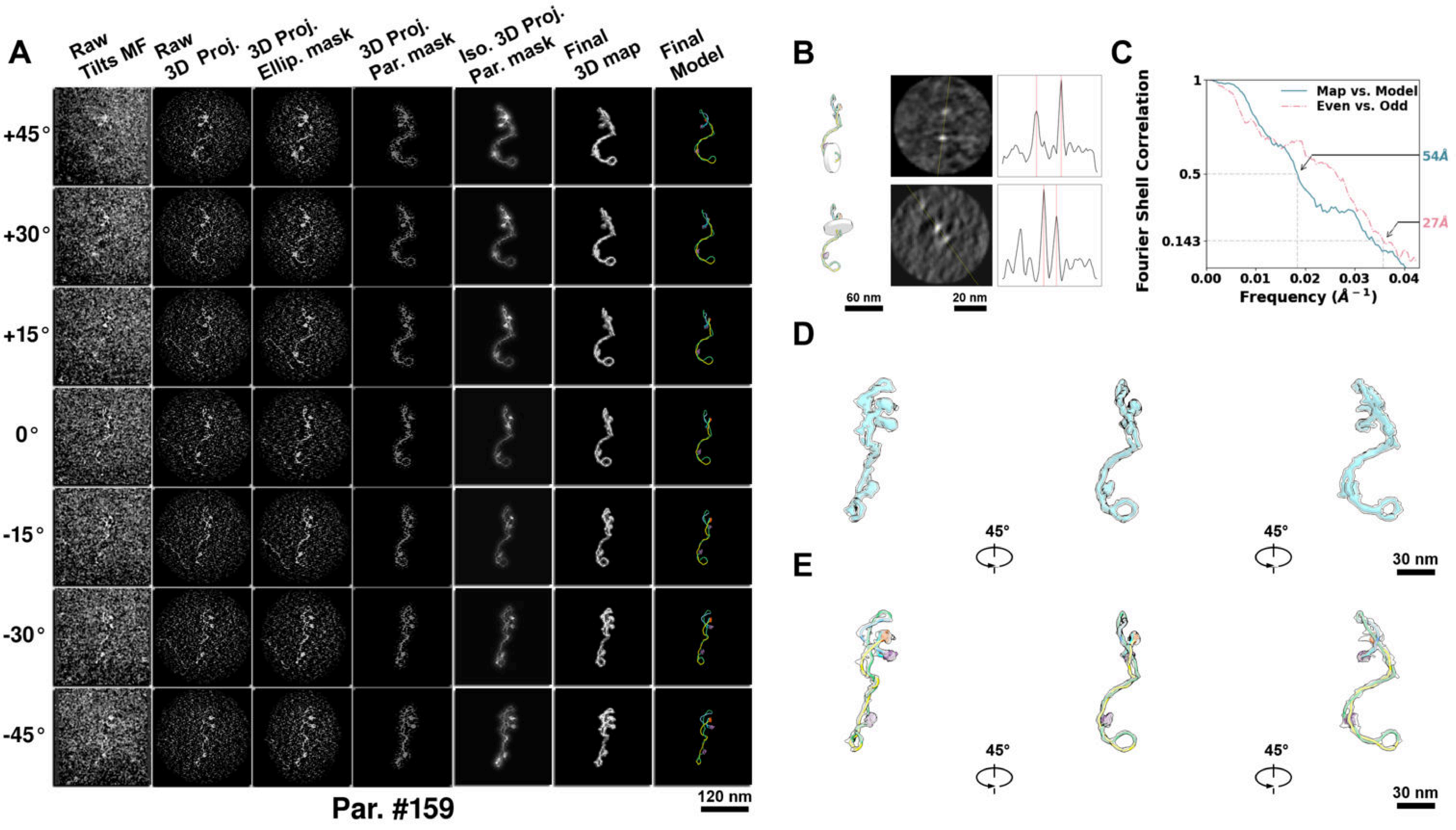

Supplementary Particle Figure 159. Cryo-ET 3D reconstruction of an individual sTEC-Cas particle.
(A) 3D reconstruction of the plasmid particle (index no. 159). The first column shows seven representative tilt images from +45° to -45° in step of 15°. The second, third, and fourth columns show 3D projections of the particle with spherical, ellipsoidal (thinner along the z-dimension), and particle-shaped masks, respectively. The fifth column displays the 3D projections of the enhanced and IsoNet missing-wedge-corrected particle. The sixth and seventh columns present the final 3D map and the flexibly fitted model, respectively. (B) Two cross-sectional views (12 nm thickness) of the plasmid density map along its plectoneme axis are shown in the left-middle panel. The intensity profile along the line crossing the two high-density DNA spots is displayed in the right panel. (C) Resolution assessment of the final 3D map using Fourier shell correlation (FSC). Two criteria are shown: FSC between two half-maps reconstructed from even and odd frames (evaluated at 0.143) and FSC between the final 3D map and the fitted model (evaluated at 0.5). (D) Zoomed-in views of the final 3D density map from panel A, displayed at two contour levels. (E) Superimposition of the high-contour level map from panel D onto its fitted model.

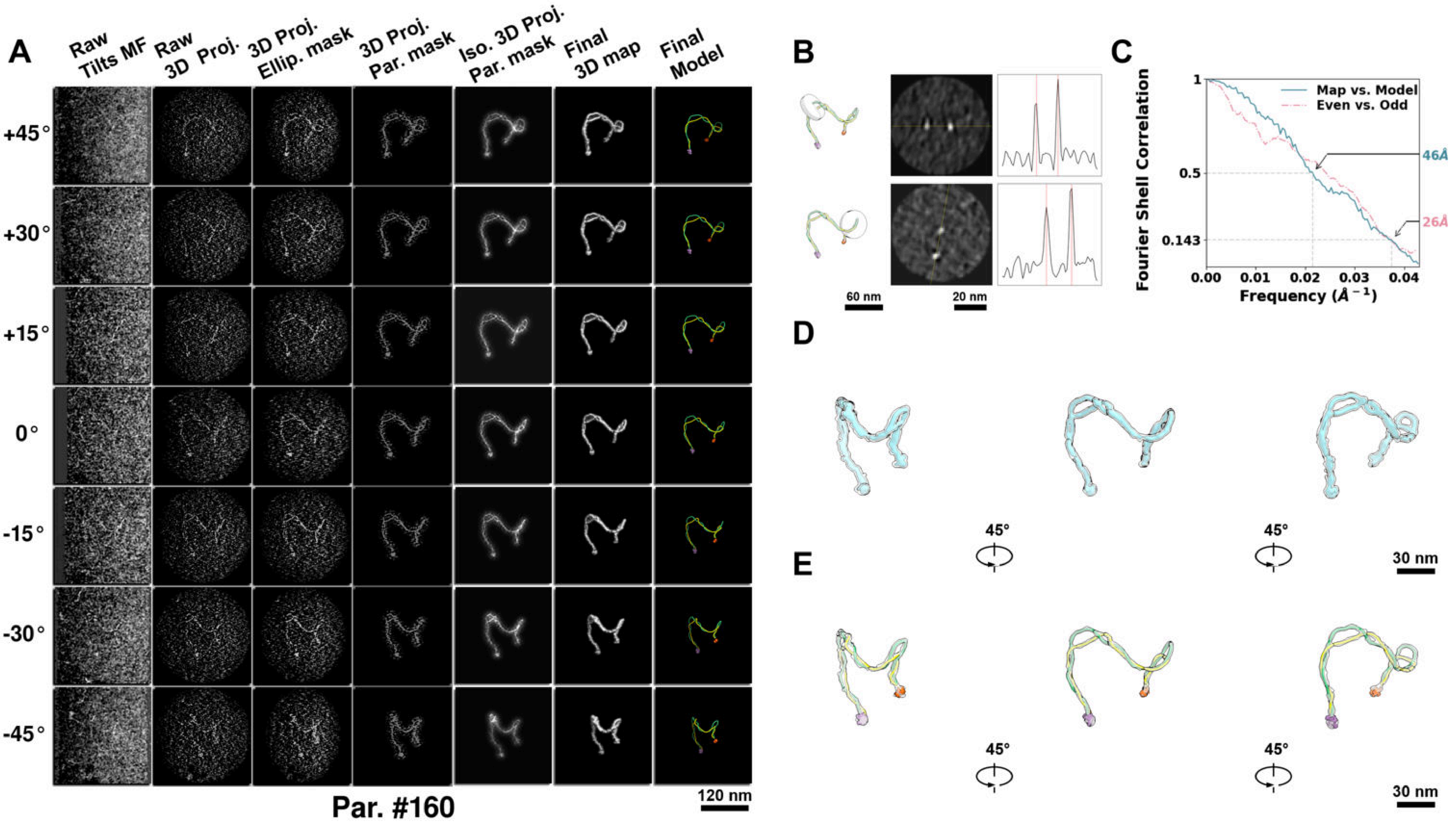

Supplementary Particle Figure 160. Cryo-ET 3D reconstruction of an individual sTEC-Cas particle.
(A) 3D reconstruction of the plasmid particle (index no. 160). The first column shows seven representative tilt images from +45° to -45° in step of 15°. The second, third, and fourth columns show 3D projections of the particle with spherical, ellipsoidal (thinner along the z-dimension), and particle-shaped masks, respectively. The fifth column displays the 3D projections of the enhanced and IsoNet missing-wedge-corrected particle. The sixth and seventh columns present the final 3D map and the flexibly fitted model, respectively. (B) Two cross-sectional views (12 nm thickness) of the plasmid density map along its plectoneme axis are shown in the left-middle panel. The intensity profile along the line crossing the two high-density DNA spots is displayed in the right panel. (C) Resolution assessment of the final 3D map using Fourier shell correlation (FSC). Two criteria are shown: FSC between two half-maps reconstructed from even and odd frames (evaluated at 0.143) and FSC between the final 3D map and the fitted model (evaluated at 0.5). (D) Zoomed-in views of the final 3D density map from panel A, displayed at two contour levels. (E) Superimposition of the high-contour level map from panel D onto its fitted model.

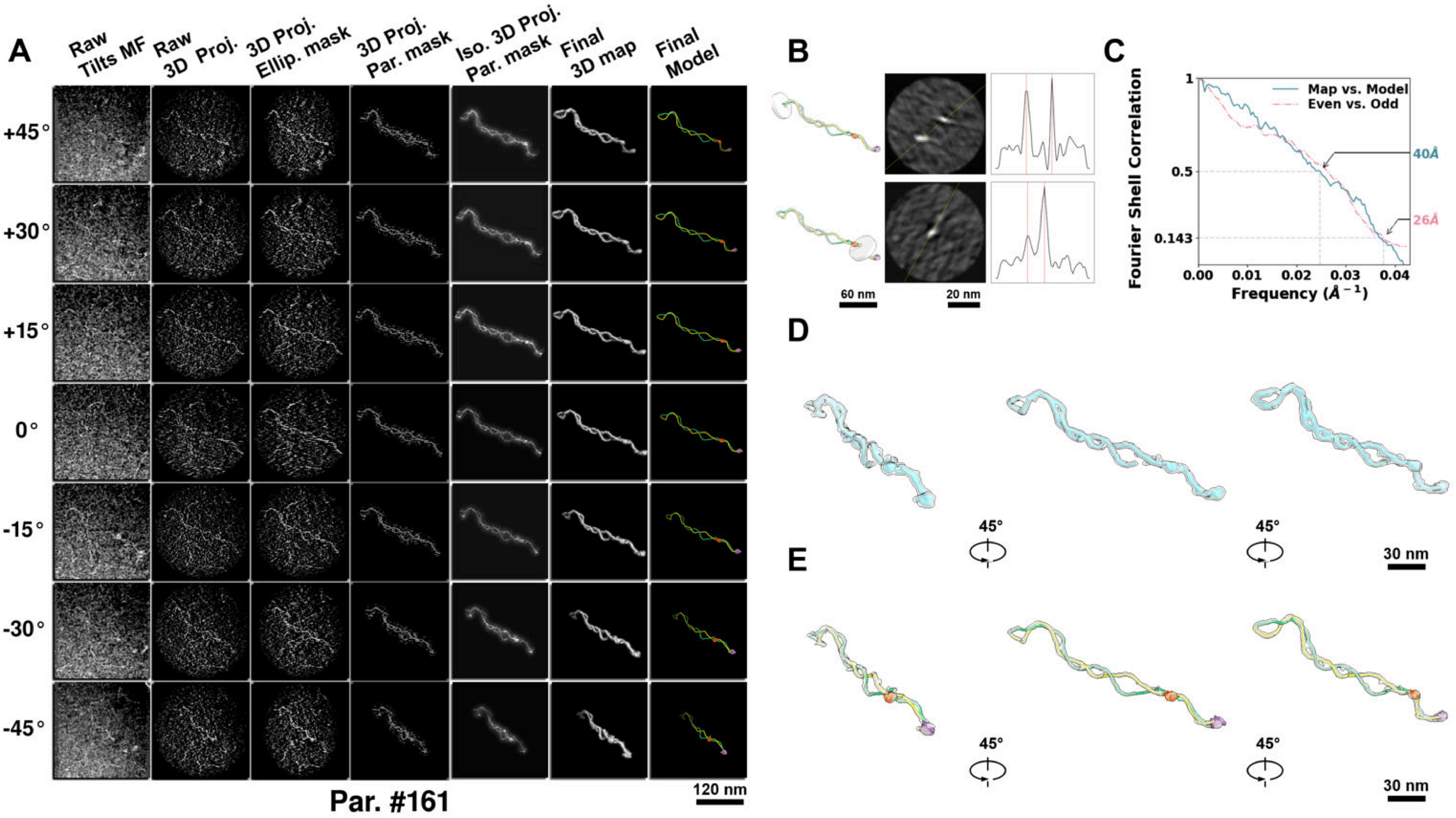

Supplementary Particle Figure 161. Cryo-ET 3D reconstruction of an individual sTEC-Cas particle.
(A) 3D reconstruction of the plasmid particle (index no. 161). The first column shows seven representative tilt images from +45° to -45° in step of 15°. The second, third, and fourth columns show 3D projections of the particle with spherical, ellipsoidal (thinner along the z-dimension), and particle-shaped masks, respectively. The fifth column displays the 3D projections of the enhanced and IsoNet missing-wedge-corrected particle. The sixth and seventh columns present the final 3D map and the flexibly fitted model, respectively. (B) Two cross-sectional views (12 nm thickness) of the plasmid density map along its plectoneme axis are shown in the left-middle panel. The intensity profile along the line crossing the two high-density DNA spots is displayed in the right panel. (C) Resolution assessment of the final 3D map using Fourier shell correlation (FSC). Two criteria are shown: FSC between two half-maps reconstructed from even and odd frames (evaluated at 0.143) and FSC between the final 3D map and the fitted model (evaluated at 0.5). (D) Zoomed-in views of the final 3D density map from panel A, displayed at two contour levels. (E) Superimposition of the high-contour level map from panel D onto its fitted model.

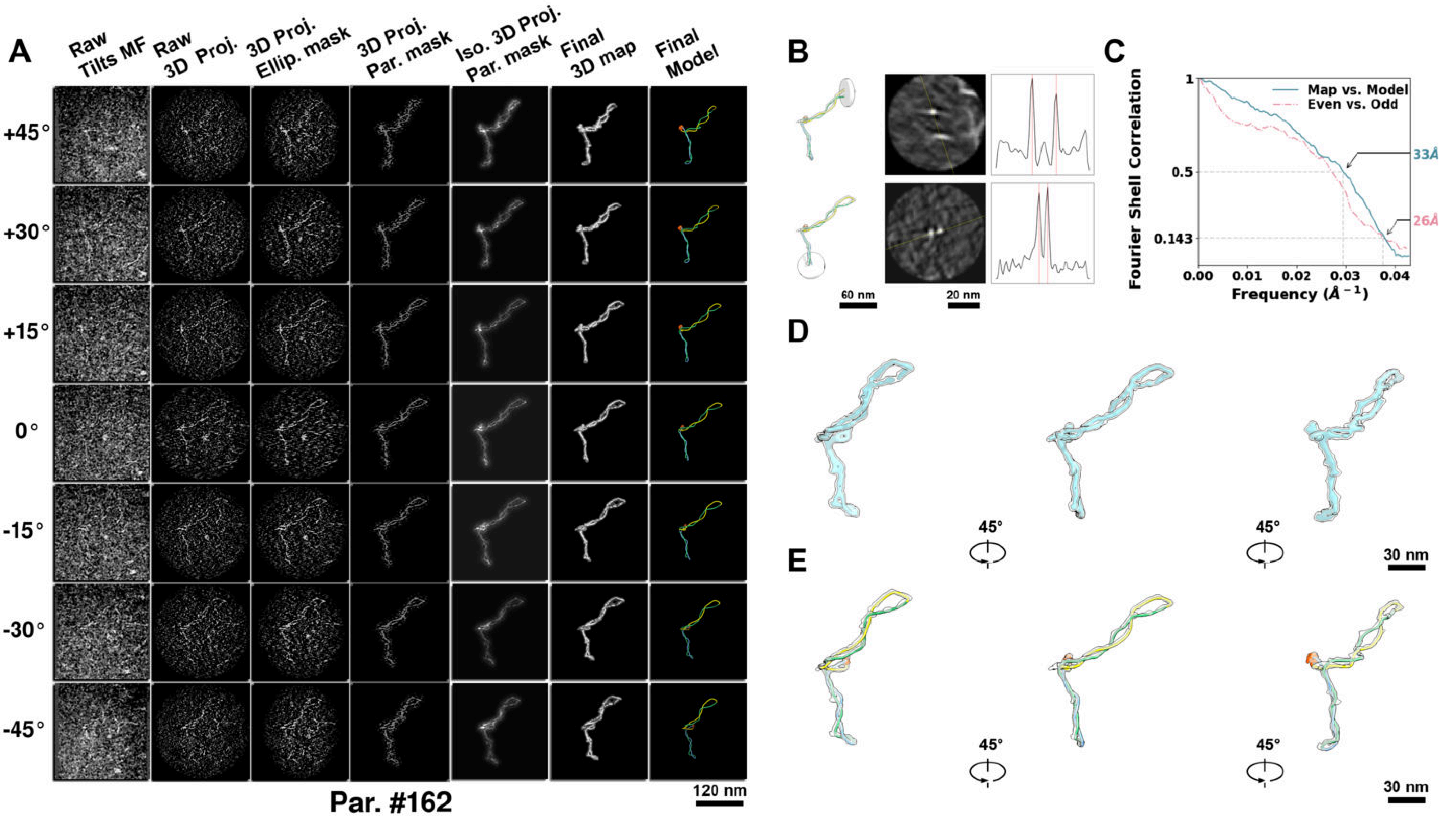

Supplementary Particle Figure 162. Cryo-ET 3D reconstruction of an individual sTEC-Cas particle.
(A) 3D reconstruction of the plasmid particle (index no. 162). The first column shows seven representative tilt images from +45° to -45° in step of 15°. The second, third, and fourth columns show 3D projections of the particle with spherical, ellipsoidal (thinner along the z-dimension), and particle-shaped masks, respectively. The fifth column displays the 3D projections of the enhanced and IsoNet missing-wedge-corrected particle. The sixth and seventh columns present the final 3D map and the flexibly fitted model, respectively. (B) Two cross-sectional views (12 nm thickness) of the plasmid density map along its plectoneme axis are shown in the left-middle panel. The intensity profile along the line crossing the two high-density DNA spots is displayed in the right panel. (C) Resolution assessment of the final 3D map using Fourier shell correlation (FSC). Two criteria are shown: FSC between two half-maps reconstructed from even and odd frames (evaluated at 0.143) and FSC between the final 3D map and the fitted model (evaluated at 0.5). (D) Zoomed-in views of the final 3D density map from panel A, displayed at two contour levels. (E) Superimposition of the high-contour level map from panel D onto its fitted model.

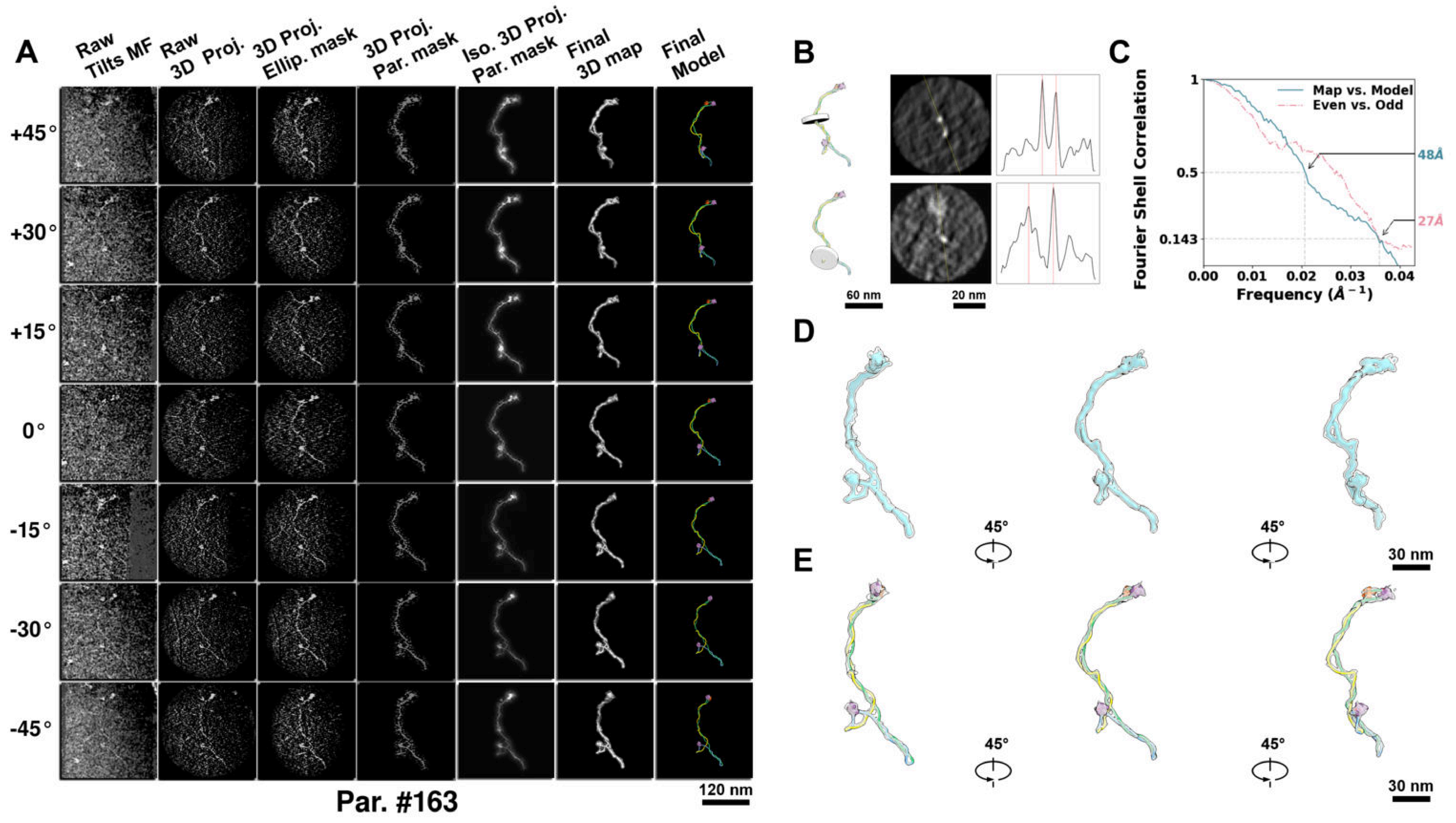

Supplementary Particle Figure 163. Cryo-ET 3D reconstruction of an individual sTEC-Cas particle.
(A) 3D reconstruction of the plasmid particle (index no. 163). The first column shows seven representative tilt images from +45° to -45° in step of 15°. The second, third, and fourth columns show 3D projections of the particle with spherical, ellipsoidal (thinner along the z-dimension), and particle-shaped masks, respectively. The fifth column displays the 3D projections of the enhanced and IsoNet missing-wedge-corrected particle. The sixth and seventh columns present the final 3D map and the flexibly fitted model, respectively. (B) Two cross-sectional views (12 nm thickness) of the plasmid density map along its plectoneme axis are shown in the left-middle panel. The intensity profile along the line crossing the two high-density DNA spots is displayed in the right panel. (C) Resolution assessment of the final 3D map using Fourier shell correlation (FSC). Two criteria are shown: FSC between two half-maps reconstructed from even and odd frames (evaluated at 0.143) and FSC between the final 3D map and the fitted model (evaluated at 0.5). (D) Zoomed-in views of the final 3D density map from panel A, displayed at two contour levels. (E) Superimposition of the high-contour level map from panel D onto its fitted model.

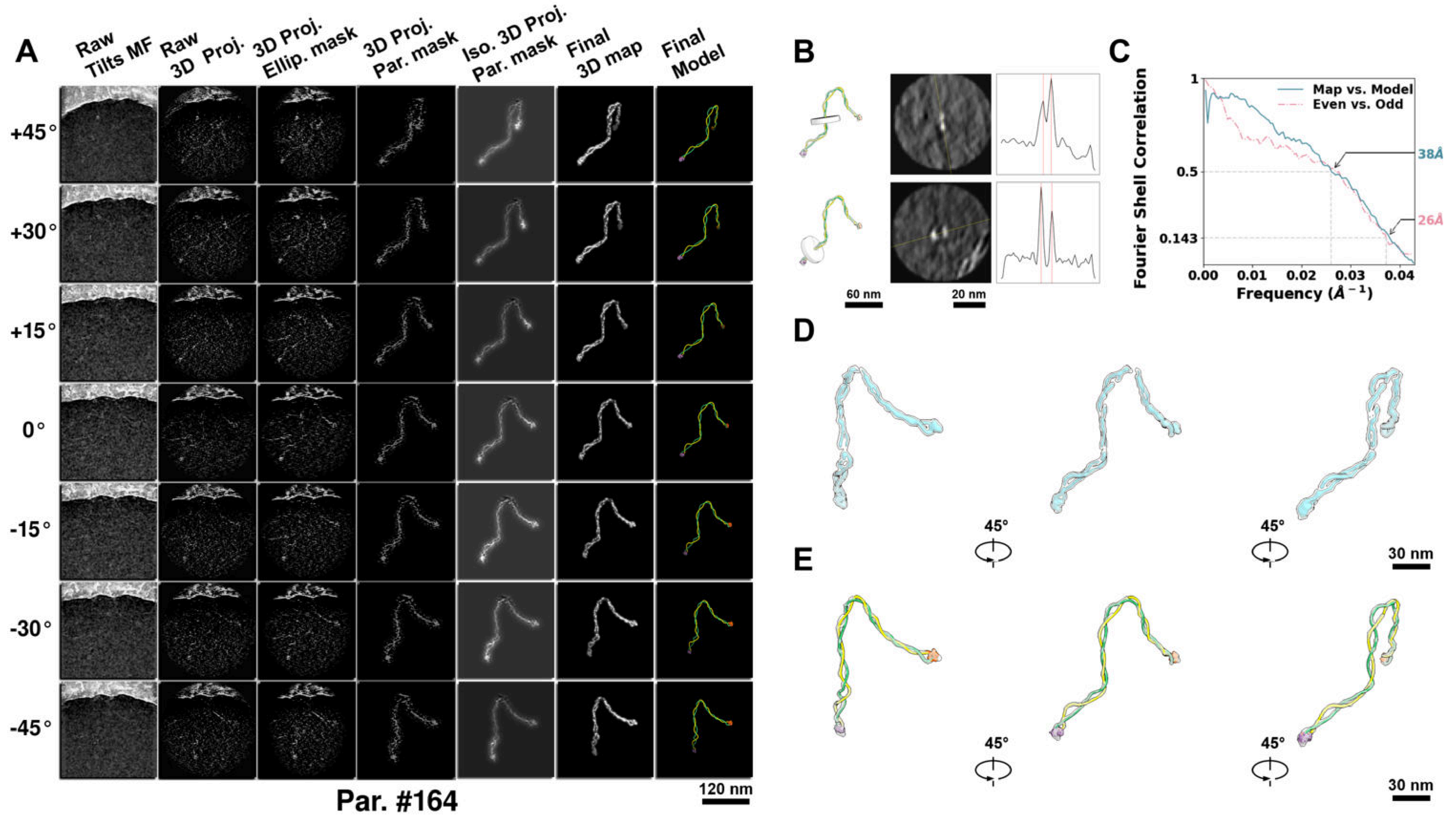

Supplementary Particle Figure 164. Cryo-ET 3D reconstruction of an individual sTEC-Cas particle.
(A) 3D reconstruction of the plasmid particle (index no. 164). The first column shows seven representative tilt images from +45° to -45° in step of 15°. The second, third, and fourth columns show 3D projections of the particle with spherical, ellipsoidal (thinner along the z-dimension), and particle-shaped masks, respectively. The fifth column displays the 3D projections of the enhanced and IsoNet missing-wedge-corrected particle. The sixth and seventh columns present the final 3D map and the flexibly fitted model, respectively. (B) Two cross-sectional views (12 nm thickness) of the plasmid density map along its plectoneme axis are shown in the left-middle panel. The intensity profile along the line crossing the two high-density DNA spots is displayed in the right panel. (C) Resolution assessment of the final 3D map using Fourier shell correlation (FSC). Two criteria are shown: FSC between two half-maps reconstructed from even and odd frames (evaluated at 0.143) and FSC between the final 3D map and the fitted model (evaluated at 0.5). (D) Zoomed-in views of the final 3D density map from panel A, displayed at two contour levels. (E) Superimposition of the high-contour level map from panel D onto its fitted model.

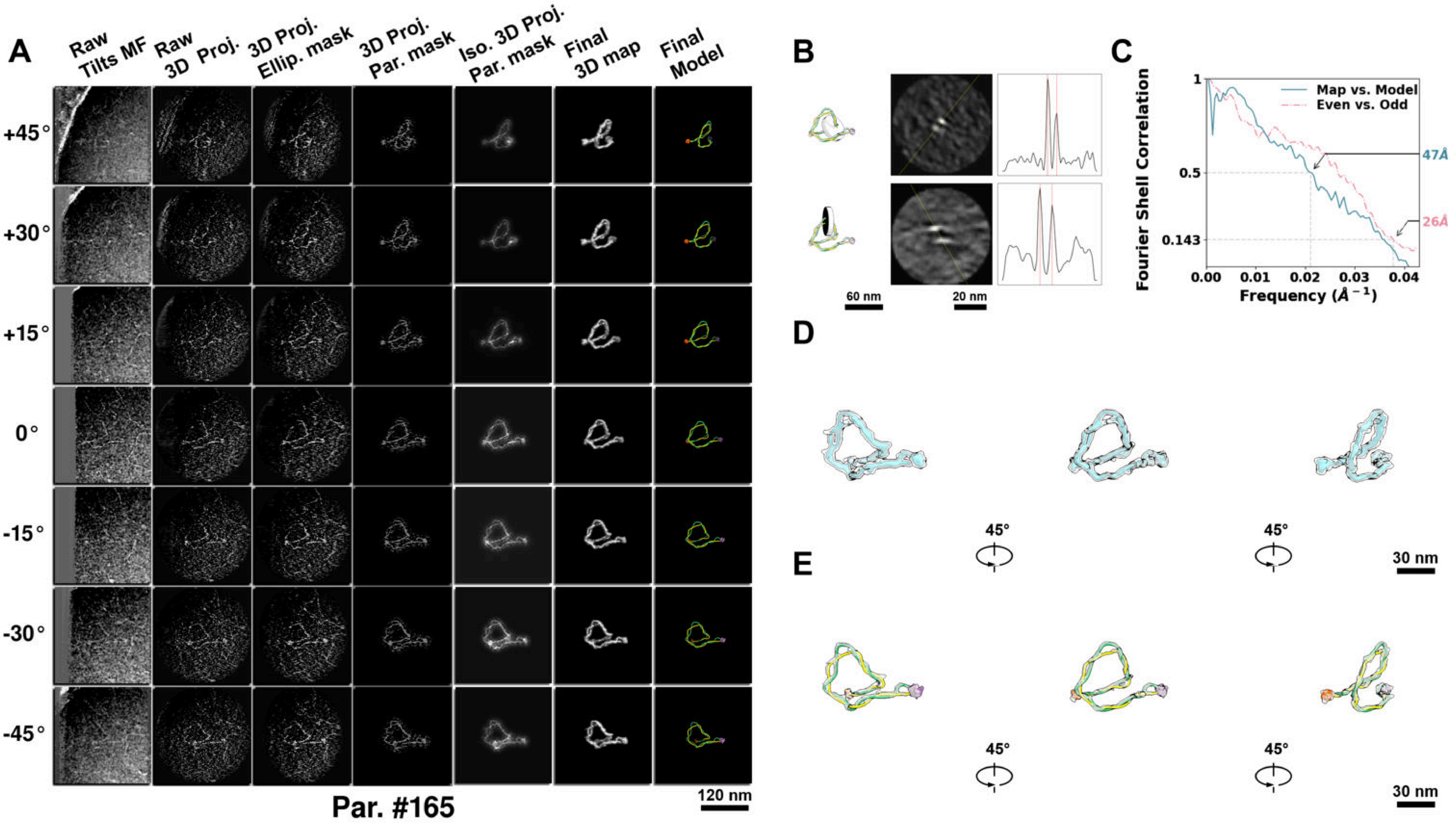

Supplementary Particle Figure 165. Cryo-ET 3D reconstruction of an individual sTEC-Cas particle.
(A) 3D reconstruction of the plasmid particle (index no. 165). The first column shows seven representative tilt images from +45° to -45° in step of 15°. The second, third, and fourth columns show 3D projections of the particle with spherical, ellipsoidal (thinner along the z-dimension), and particle-shaped masks, respectively. The fifth column displays the 3D projections of the enhanced and IsoNet missing-wedge-corrected particle. The sixth and seventh columns present the final 3D map and the flexibly fitted model, respectively. (B) Two cross-sectional views (12 nm thickness) of the plasmid density map along its plectoneme axis are shown in the left-middle panel. The intensity profile along the line crossing the two high-density DNA spots is displayed in the right panel. (C) Resolution assessment of the final 3D map using Fourier shell correlation (FSC). Two criteria are shown: FSC between two half-maps reconstructed from even and odd frames (evaluated at 0.143) and FSC between the final 3D map and the fitted model (evaluated at 0.5). (D) Zoomed-in views of the final 3D density map from panel A, displayed at two contour levels. (E) Superimposition of the high-contour level map from panel D onto its fitted model.

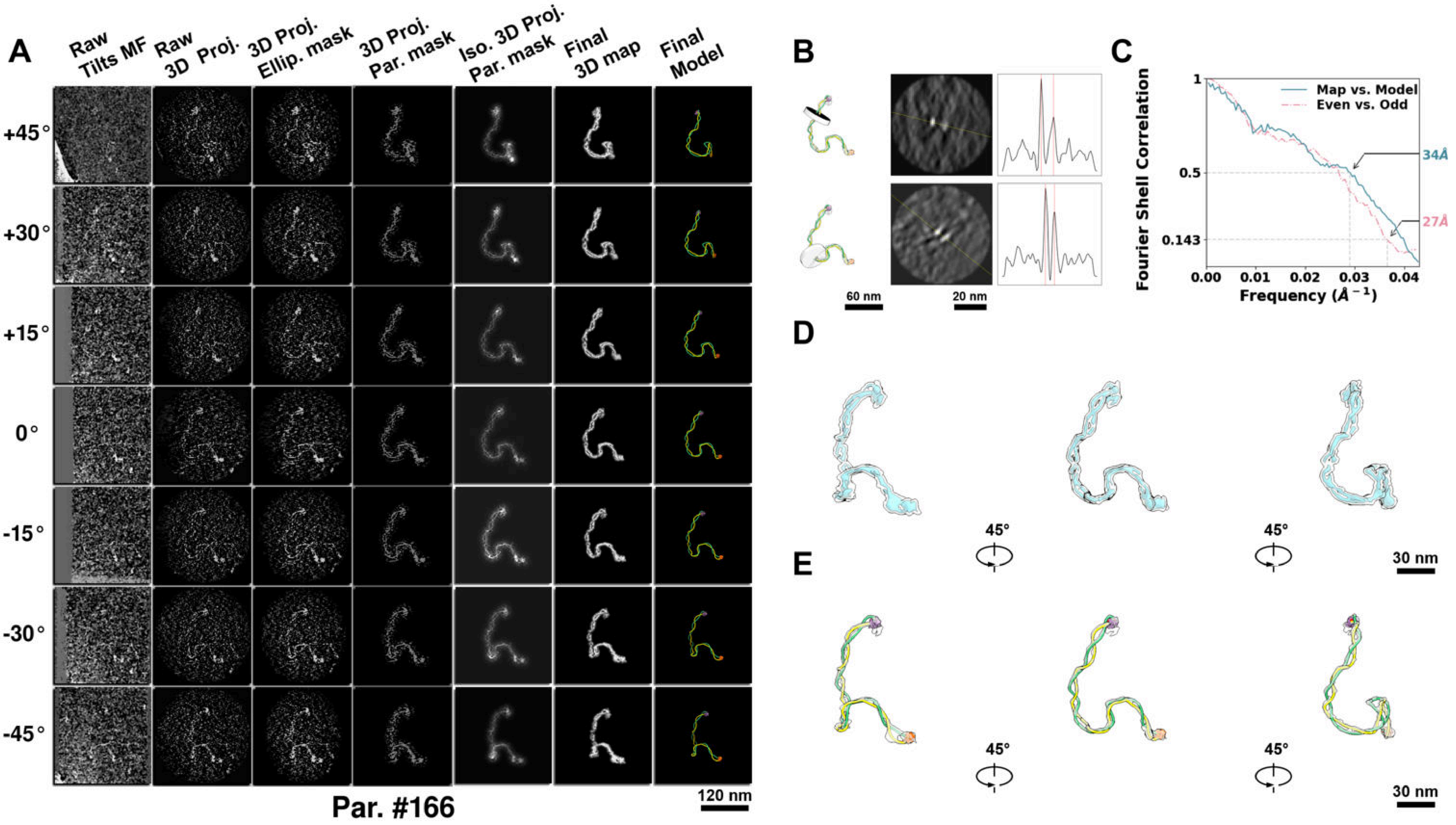

Supplementary Particle Figure 166. Cryo-ET 3D reconstruction of an individual sTEC-Cas particle.
(A) 3D reconstruction of the plasmid particle (index no. 166). The first column shows seven representative tilt images from +45° to -45° in step of 15°. The second, third, and fourth columns show 3D projections of the particle with spherical, ellipsoidal (thinner along the z-dimension), and particle-shaped masks, respectively. The fifth column displays the 3D projections of the enhanced and IsoNet missing-wedge-corrected particle. The sixth and seventh columns present the final 3D map and the flexibly fitted model, respectively. (B) Two cross-sectional views (12 nm thickness) of the plasmid density map along its plectoneme axis are shown in the left-middle panel. The intensity profile along the line crossing the two high-density DNA spots is displayed in the right panel. (C) Resolution assessment of the final 3D map using Fourier shell correlation (FSC). Two criteria are shown: FSC between two half-maps reconstructed from even and odd frames (evaluated at 0.143) and FSC between the final 3D map and the fitted model (evaluated at 0.5). (D) Zoomed-in views of the final 3D density map from panel A, displayed at two contour levels. (E) Superimposition of the high-contour level map from panel D onto its fitted model.

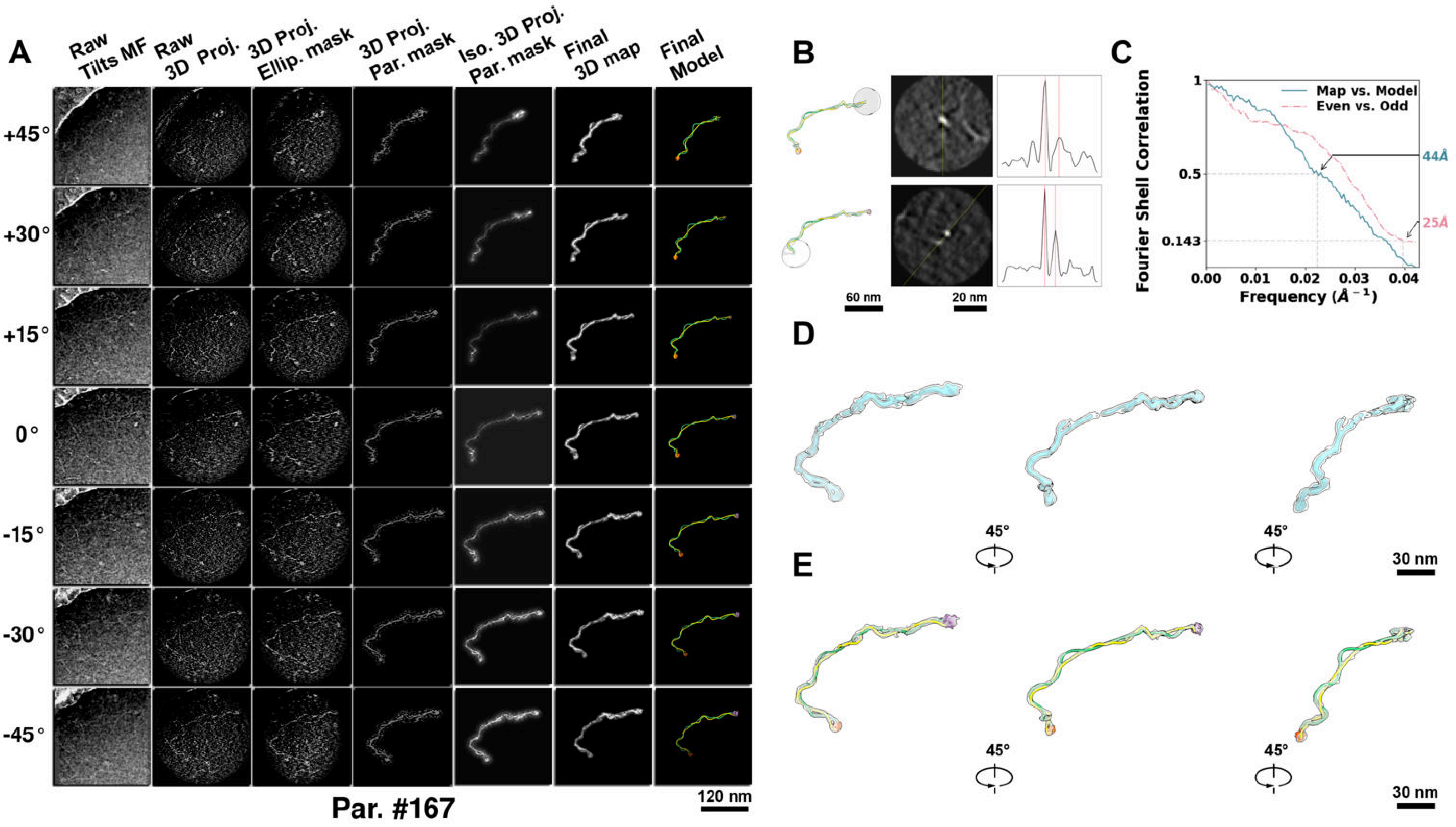

Supplementary Particle Figure 167. Cryo-ET 3D reconstruction of an individual sTEC-Cas particle.
(A) 3D reconstruction of the plasmid particle (index no. 167). The first column shows seven representative tilt images from +45° to -45° in step of 15°. The second, third, and fourth columns show 3D projections of the particle with spherical, ellipsoidal (thinner along the z-dimension), and particle-shaped masks, respectively. The fifth column displays the 3D projections of the enhanced and IsoNet missing-wedge-corrected particle. The sixth and seventh columns present the final 3D map and the flexibly fitted model, respectively. (B) Two cross-sectional views (12 nm thickness) of the plasmid density map along its plectoneme axis are shown in the left-middle panel. The intensity profile along the line crossing the two high-density DNA spots is displayed in the right panel. (C) Resolution assessment of the final 3D map using Fourier shell correlation (FSC). Two criteria are shown: FSC between two half-maps reconstructed from even and odd frames (evaluated at 0.143) and FSC between the final 3D map and the fitted model (evaluated at 0.5). (D) Zoomed-in views of the final 3D density map from panel A, displayed at two contour levels. (E) Superimposition of the high-contour level map from panel D onto its fitted model.

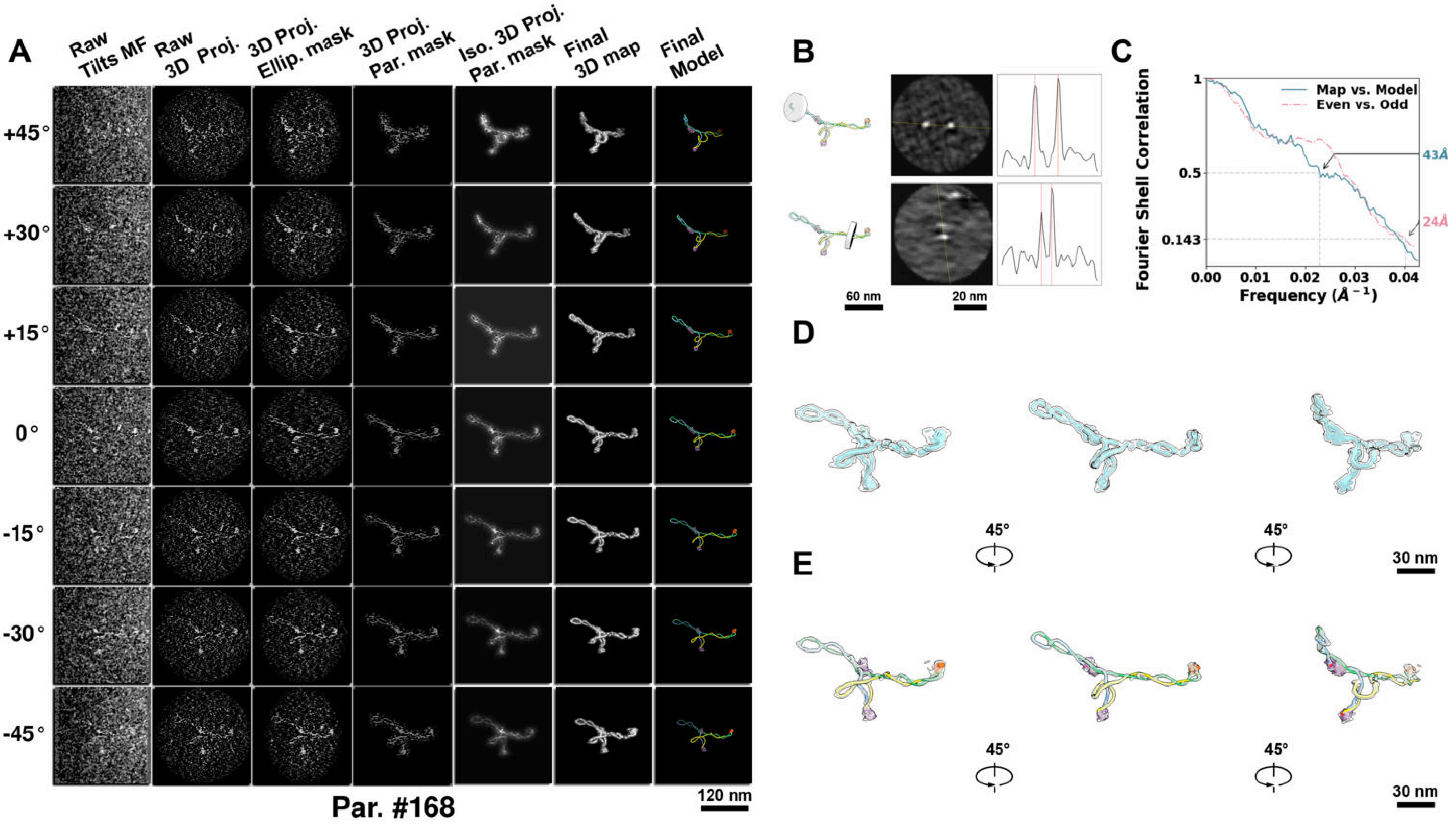

Supplementary Particle Figure 168. Cryo-ET 3D reconstruction of an individual sTEC-Cas particle.
(A) 3D reconstruction of the plasmid particle (index no. 168). The first column shows seven representative tilt images from +45° to -45° in step of 15°. The second, third, and fourth columns show 3D projections of the particle with spherical, ellipsoidal (thinner along the z-dimension), and particle-shaped masks, respectively. The fifth column displays the 3D projections of the enhanced and IsoNet missing-wedge-corrected particle. The sixth and seventh columns present the final 3D map and the flexibly fitted model, respectively. (B) Two cross-sectional views (12 nm thickness) of the plasmid density map along its plectoneme axis are shown in the left-middle panel. The intensity profile along the line crossing the two high-density DNA spots is displayed in the right panel. (C) Resolution assessment of the final 3D map using Fourier shell correlation (FSC). Two criteria are shown: FSC between two half-maps reconstructed from even and odd frames (evaluated at 0.143) and FSC between the final 3D map and the fitted model (evaluated at 0.5). (D) Zoomed-in views of the final 3D density map from panel A, displayed at two contour levels. (E) Superimposition of the high-contour level map from panel D onto its fitted model.

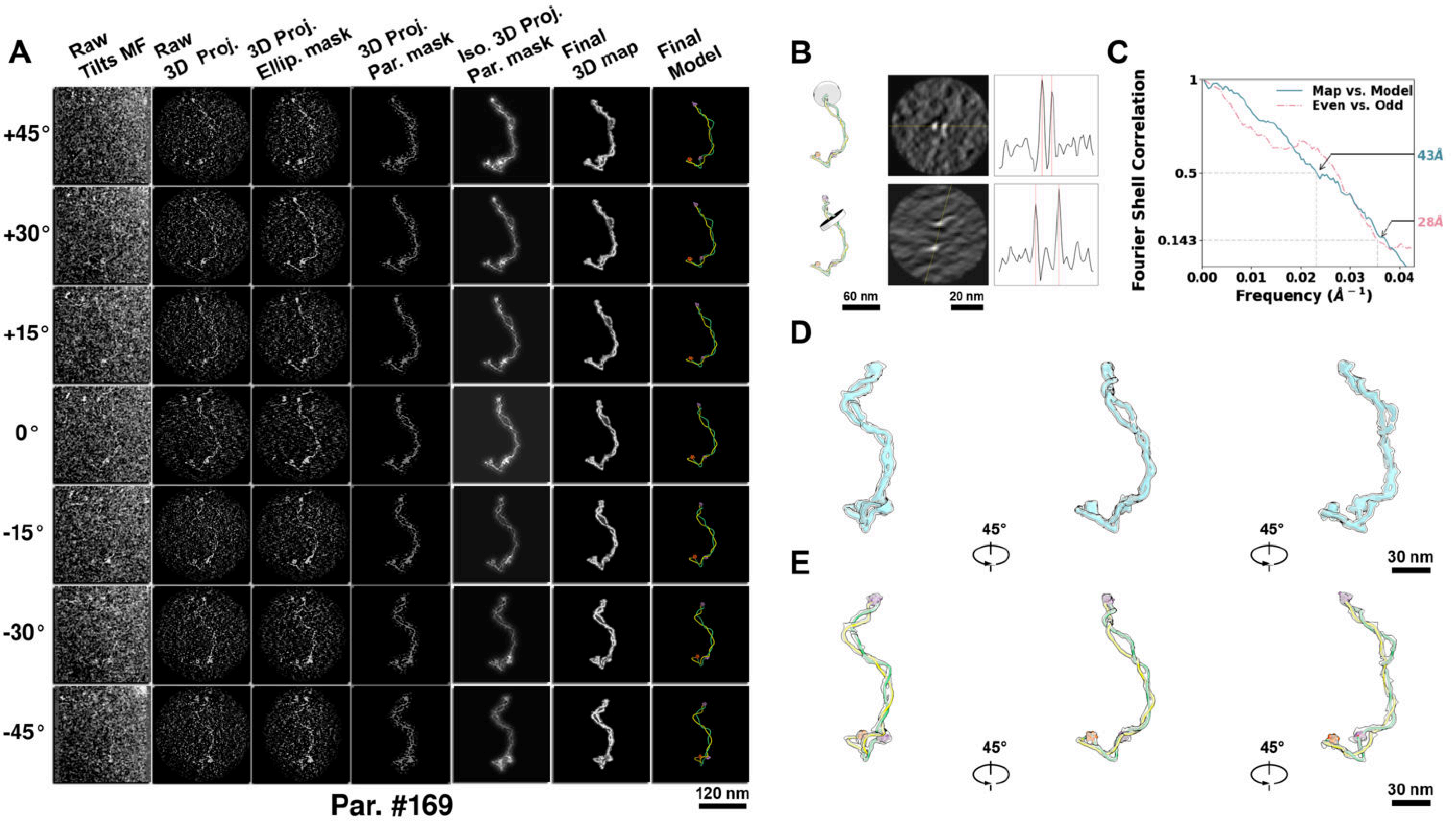

Supplementary Particle Figure 169. Cryo-ET 3D reconstruction of an individual sTEC-Cas particle.
(A) 3D reconstruction of the plasmid particle (index no. 169). The first column shows seven representative tilt images from +45° to -45° in step of 15°. The second, third, and fourth columns show 3D projections of the particle with spherical, ellipsoidal (thinner along the z-dimension), and particle-shaped masks, respectively. The fifth column displays the 3D projections of the enhanced and IsoNet missing-wedge-corrected particle. The sixth and seventh columns present the final 3D map and the flexibly fitted model, respectively. (B) Two cross-sectional views (12 nm thickness) of the plasmid density map along its plectoneme axis are shown in the left-middle panel. The intensity profile along the line crossing the two high-density DNA spots is displayed in the right panel. (C) Resolution assessment of the final 3D map using Fourier shell correlation (FSC). Two criteria are shown: FSC between two half-maps reconstructed from even and odd frames (evaluated at 0.143) and FSC between the final 3D map and the fitted model (evaluated at 0.5). (D) Zoomed-in views of the final 3D density map from panel A, displayed at two contour levels. (E) Superimposition of the high-contour level map from panel D onto its fitted model.

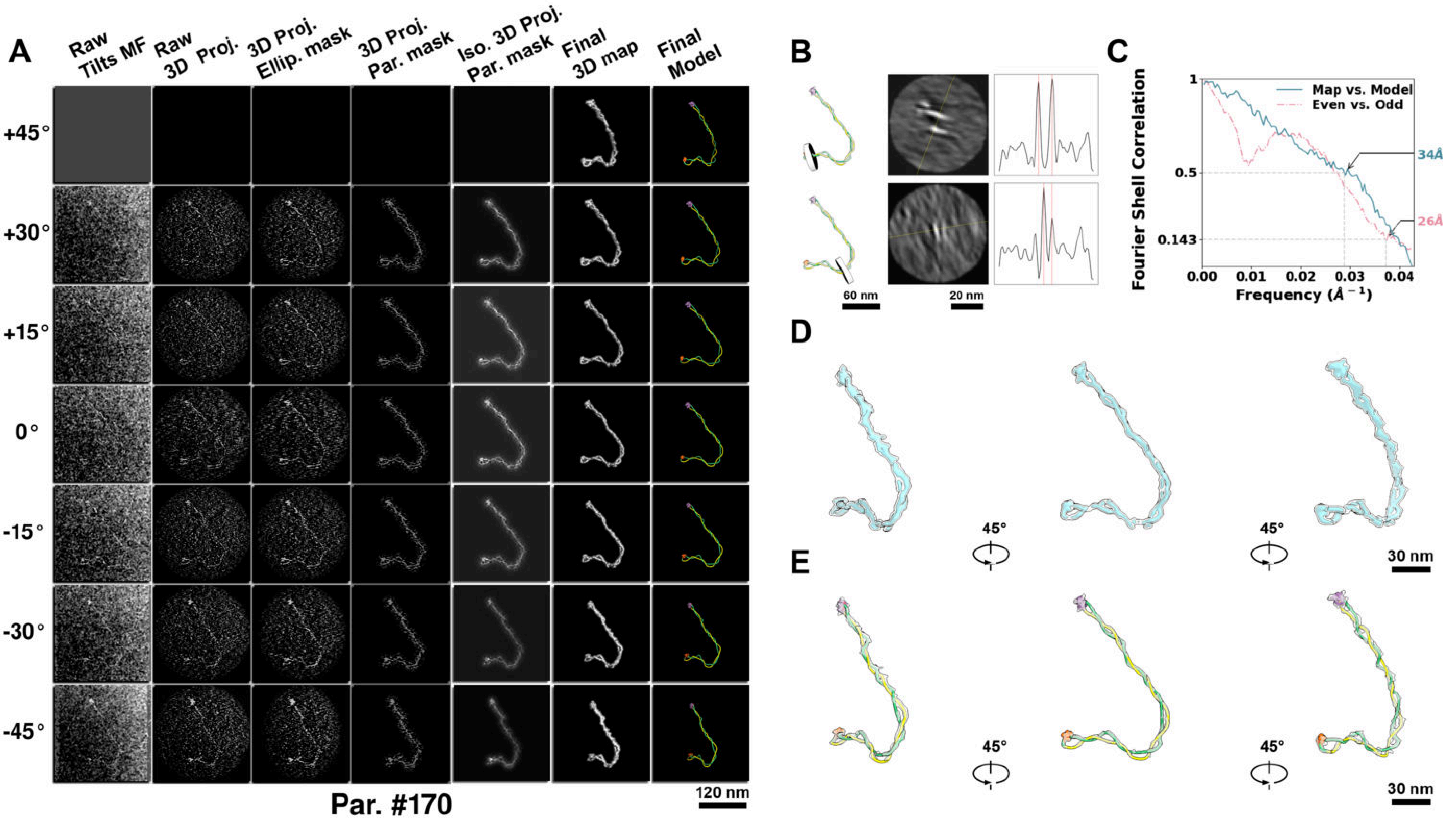

Supplementary Particle Figure 170. Cryo-ET 3D reconstruction of an individual sTEC-Cas particle.
(A) 3D reconstruction of the plasmid particle (index no. 170). The first column shows seven representative tilt images from +45° to -45° in step of 15°. The second, third, and fourth columns show 3D projections of the particle with spherical, ellipsoidal (thinner along the z-dimension), and particle-shaped masks, respectively. The fifth column displays the 3D projections of the enhanced and IsoNet missing-wedge-corrected particle. The sixth and seventh columns present the final 3D map and the flexibly fitted model, respectively. (B) Two cross-sectional views (12 nm thickness) of the plasmid density map along its plectoneme axis are shown in the left-middle panel. The intensity profile along the line crossing the two high-density DNA spots is displayed in the right panel. (C) Resolution assessment of the final 3D map using Fourier shell correlation (FSC). Two criteria are shown: FSC between two half-maps reconstructed from even and odd frames (evaluated at 0.143) and FSC between the final 3D map and the fitted model (evaluated at 0.5). (D) Zoomed-in views of the final 3D density map from panel A, displayed at two contour levels. (E) Superimposition of the high-contour level map from panel D onto its fitted model.

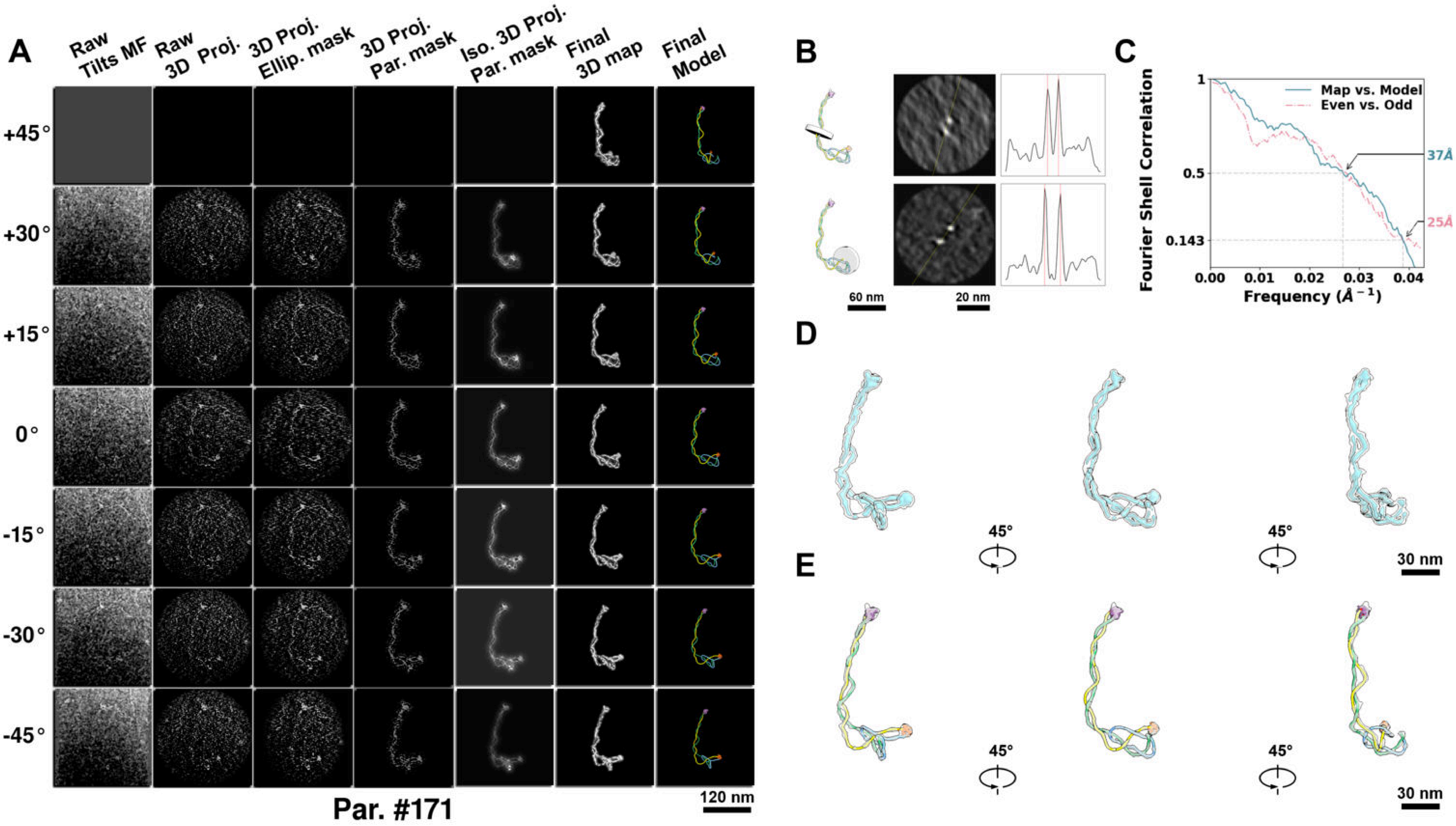

Supplementary Particle Figure 171. Cryo-ET 3D reconstruction of an individual sTEC-Cas particle.
(A) 3D reconstruction of the plasmid particle (index no. 171). The first column shows seven representative tilt images from +45° to -45° in step of 15°. The second, third, and fourth columns show 3D projections of the particle with spherical, ellipsoidal (thinner along the z-dimension), and particle-shaped masks, respectively. The fifth column displays the 3D projections of the enhanced and IsoNet missing-wedge-corrected particle. The sixth and seventh columns present the final 3D map and the flexibly fitted model, respectively. (B) Two cross-sectional views (12 nm thickness) of the plasmid density map along its plectoneme axis are shown in the left-middle panel. The intensity profile along the line crossing the two high-density DNA spots is displayed in the right panel. (C) Resolution assessment of the final 3D map using Fourier shell correlation (FSC). Two criteria are shown: FSC between two half-maps reconstructed from even and odd frames (evaluated at 0.143) and FSC between the final 3D map and the fitted model (evaluated at 0.5). (D) Zoomed-in views of the final 3D density map from panel A, displayed at two contour levels. (E) Superimposition of the high-contour level map from panel D onto its fitted model.

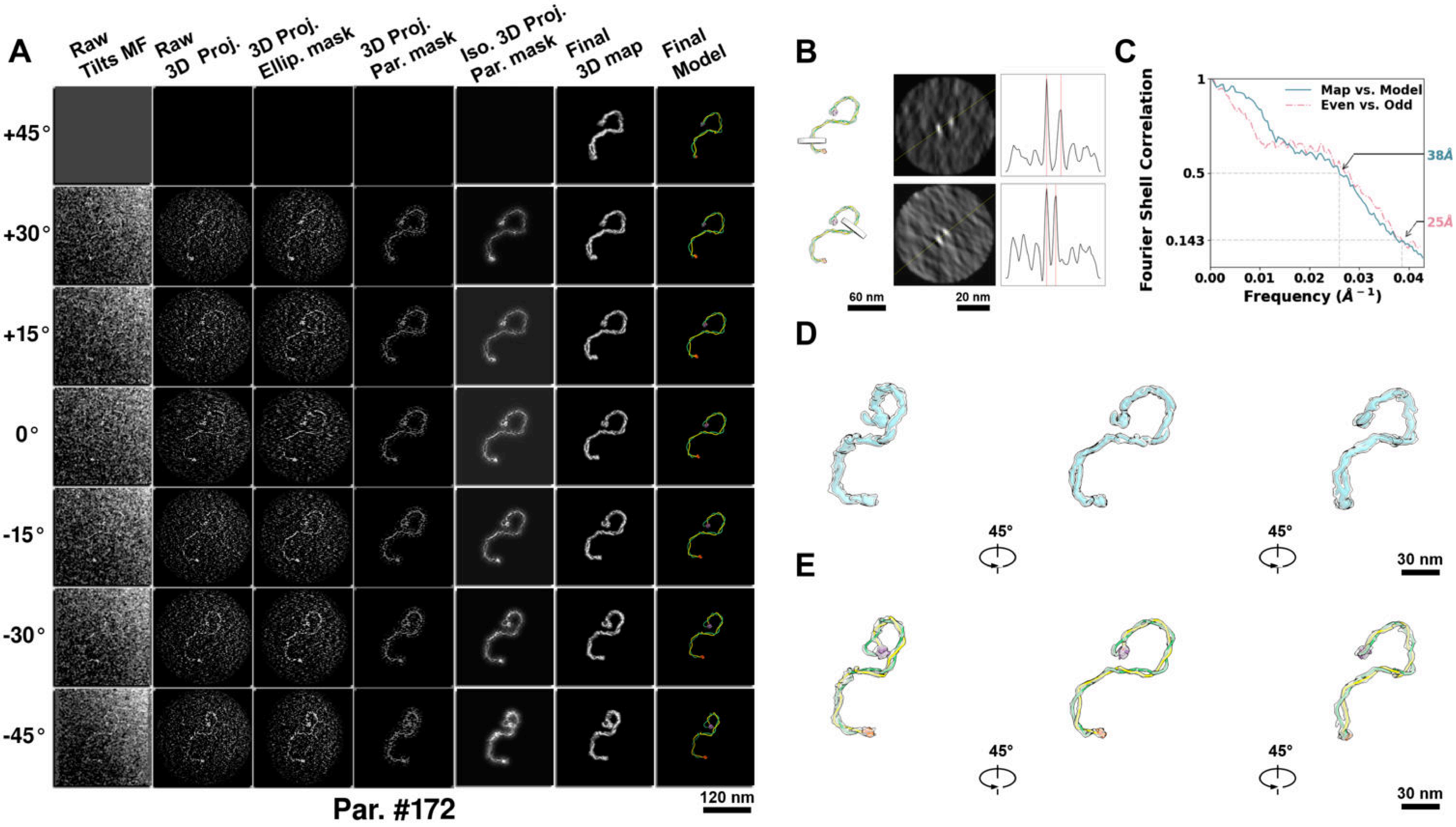

Supplementary Particle Figure 172. Cryo-ET 3D reconstruction of an individual sTEC-Cas particle.
(A) 3D reconstruction of the plasmid particle (index no. 172). The first column shows seven representative tilt images from +45° to -45° in step of 15°. The second, third, and fourth columns show 3D projections of the particle with spherical, ellipsoidal (thinner along the z-dimension), and particle-shaped masks, respectively. The fifth column displays the 3D projections of the enhanced and IsoNet missing-wedge-corrected particle. The sixth and seventh columns present the final 3D map and the flexibly fitted model, respectively. (B) Two cross-sectional views (12 nm thickness) of the plasmid density map along its plectoneme axis are shown in the left-middle panel. The intensity profile along the line crossing the two high-density DNA spots is displayed in the right panel. (C) Resolution assessment of the final 3D map using Fourier shell correlation (FSC). Two criteria are shown: FSC between two half-maps reconstructed from even and odd frames (evaluated at 0.143) and FSC between the final 3D map and the fitted model (evaluated at 0.5). (D) Zoomed-in views of the final 3D density map from panel A, displayed at two contour levels. (E) Superimposition of the high-contour level map from panel D onto its fitted model.

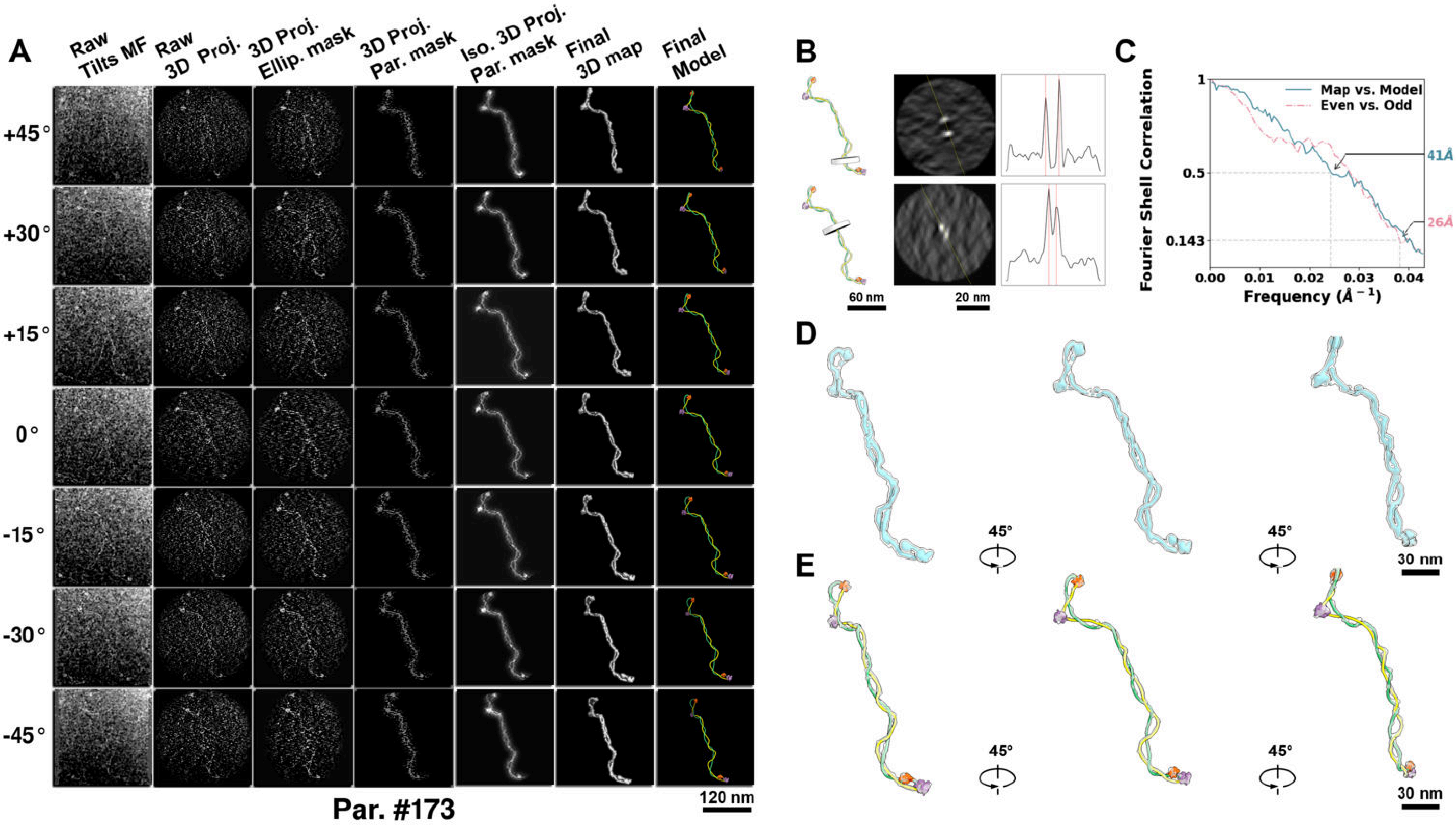

Supplementary Particle Figure 173. Cryo-ET 3D reconstruction of an individual sTEC-Cas particle.
(A) 3D reconstruction of the plasmid particle (index no. 173). The first column shows seven representative tilt images from +45° to -45° in step of 15°. The second, third, and fourth columns show 3D projections of the particle with spherical, ellipsoidal (thinner along the z-dimension), and particle-shaped masks, respectively. The fifth column displays the 3D projections of the enhanced and IsoNet missing-wedge-corrected particle. The sixth and seventh columns present the final 3D map and the flexibly fitted model, respectively. (B) Two cross-sectional views (12 nm thickness) of the plasmid density map along its plectoneme axis are shown in the left-middle panel. The intensity profile along the line crossing the two high-density DNA spots is displayed in the right panel. (C) Resolution assessment of the final 3D map using Fourier shell correlation (FSC). Two criteria are shown: FSC between two half-maps reconstructed from even and odd frames (evaluated at 0.143) and FSC between the final 3D map and the fitted model (evaluated at 0.5). (D) Zoomed-in views of the final 3D density map from panel A, displayed at two contour levels. (E) Superimposition of the high-contour level map from panel D onto its fitted model.

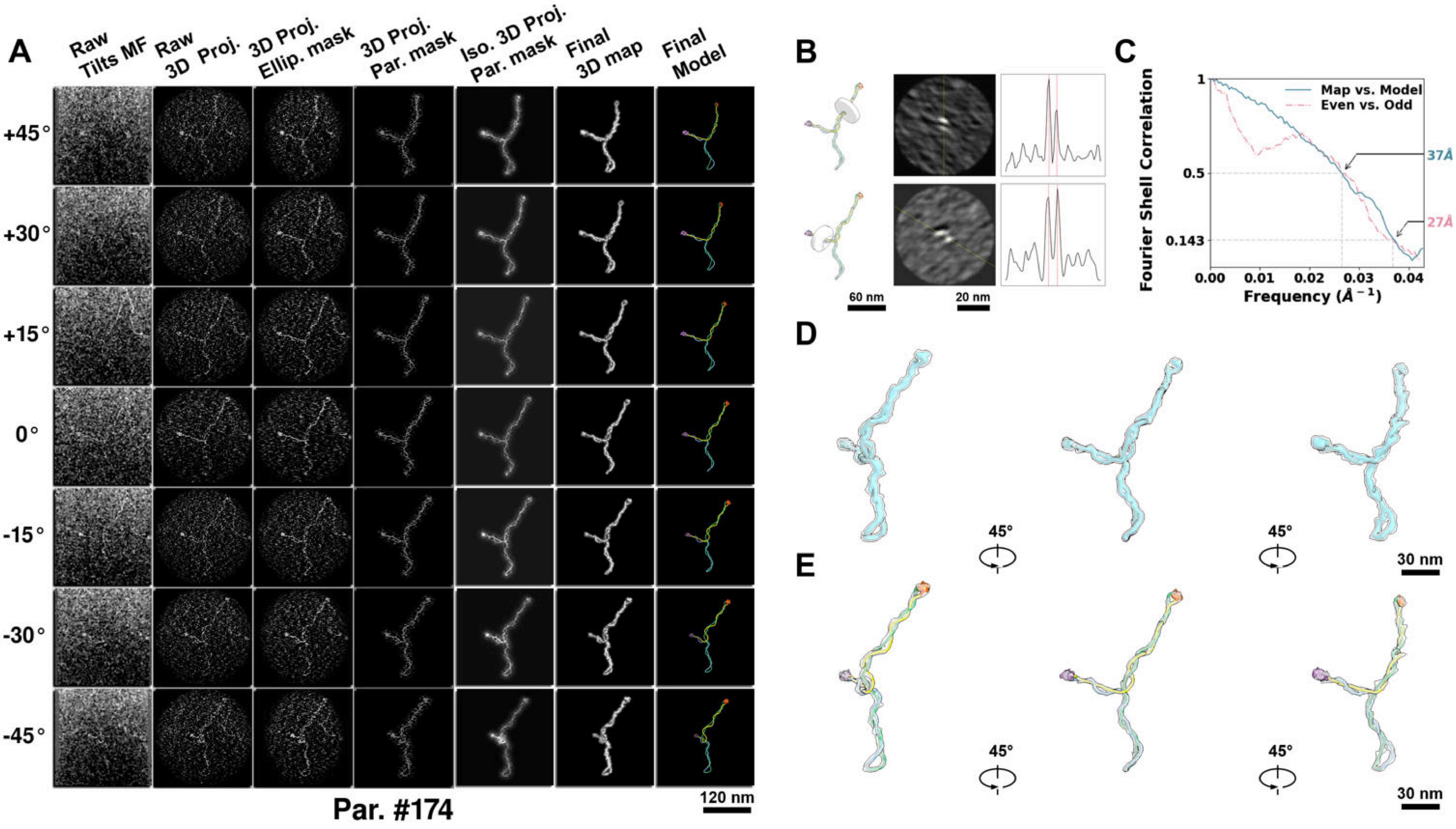

Supplementary Particle Figure 174. Cryo-ET 3D reconstruction of an individual sTEC-Cas particle.
(A) 3D reconstruction of the plasmid particle (index no. 174). The first column shows seven representative tilt images from +45° to -45° in step of 15°. The second, third, and fourth columns show 3D projections of the particle with spherical, ellipsoidal (thinner along the z-dimension), and particle-shaped masks, respectively. The fifth column displays the 3D projections of the enhanced and IsoNet missing-wedge-corrected particle. The sixth and seventh columns present the final 3D map and the flexibly fitted model, respectively. (B) Two cross-sectional views (12 nm thickness) of the plasmid density map along its plectoneme axis are shown in the left-middle panel. The intensity profile along the line crossing the two high-density DNA spots is displayed in the right panel. (C) Resolution assessment of the final 3D map using Fourier shell correlation (FSC). Two criteria are shown: FSC between two half-maps reconstructed from even and odd frames (evaluated at 0.143) and FSC between the final 3D map and the fitted model (evaluated at 0.5). (D) Zoomed-in views of the final 3D density map from panel A, displayed at two contour levels. (E) Superimposition of the high-contour level map from panel D onto its fitted model.

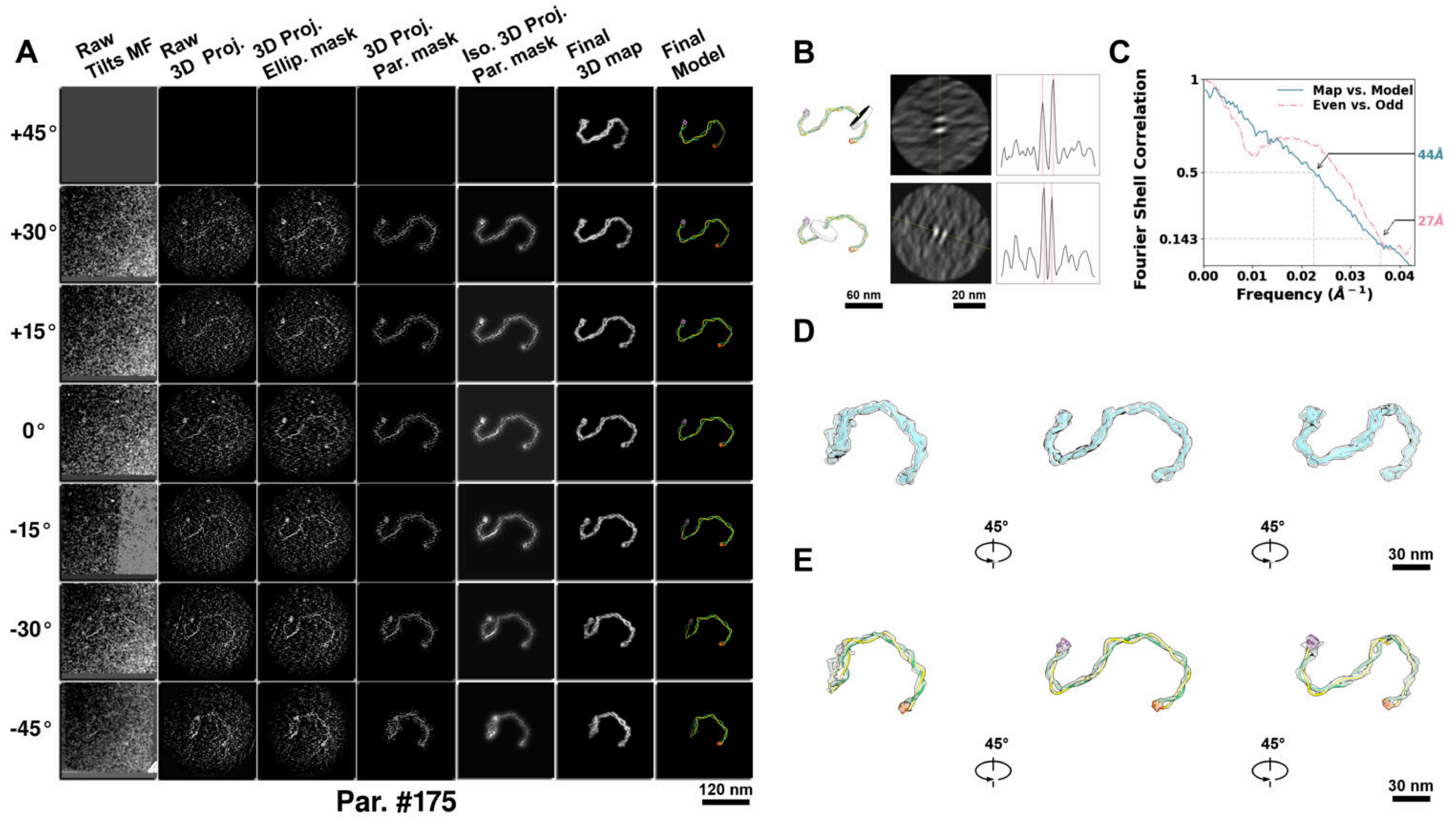

Supplementary Particle Figure 175. Cryo-ET 3D reconstruction of an individual sTEC-Cas particle.
(A) 3D reconstruction of the plasmid particle (index no. 175). The first column shows seven representative tilt images from +45° to -45° in step of 15°. The second, third, and fourth columns show 3D projections of the particle with spherical, ellipsoidal (thinner along the z-dimension), and particle-shaped masks, respectively. The fifth column displays the 3D projections of the enhanced and IsoNet missing-wedge-corrected particle. The sixth and seventh columns present the final 3D map and the flexibly fitted model, respectively. (B) Two cross-sectional views (12 nm thickness) of the plasmid density map along its plectoneme axis are shown in the left-middle panel. The intensity profile along the line crossing the two high-density DNA spots is displayed in the right panel. (C) Resolution assessment of the final 3D map using Fourier shell correlation (FSC). Two criteria are shown: FSC between two half-maps reconstructed from even and odd frames (evaluated at 0.143) and FSC between the final 3D map and the fitted model (evaluated at 0.5). (D) Zoomed-in views of the final 3D density map from panel A, displayed at two contour levels. (E) Superimposition of the high-contour level map from panel D onto its fitted model.

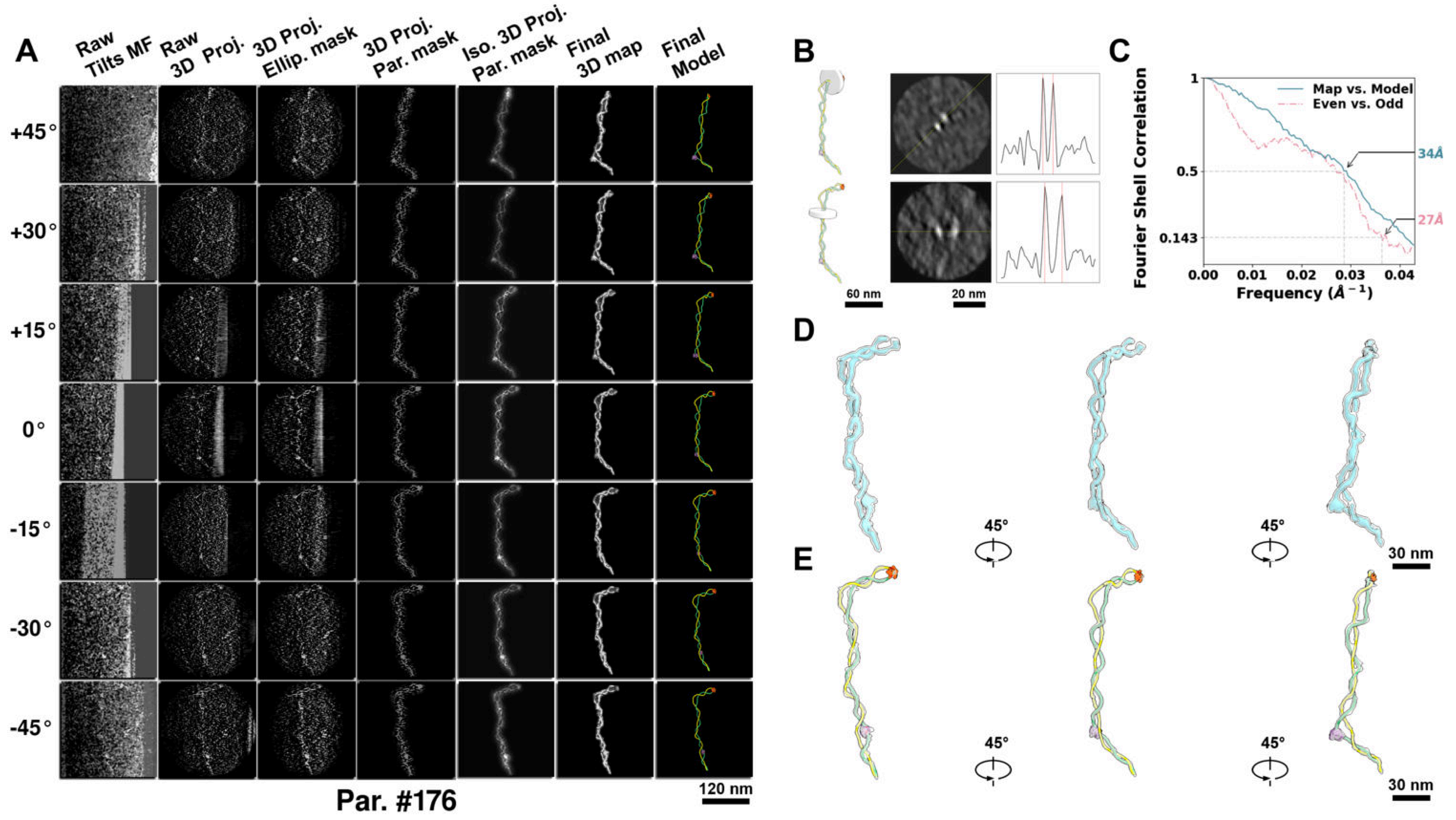

Supplementary Particle Figure 176. Cryo-ET 3D reconstruction of an individual sTEC-Cas particle.
(A) 3D reconstruction of the plasmid particle (index no. 176). The first column shows seven representative tilt images from +45° to -45° in step of 15°. The second, third, and fourth columns show 3D projections of the particle with spherical, ellipsoidal (thinner along the z-dimension), and particle-shaped masks, respectively. The fifth column displays the 3D projections of the enhanced and IsoNet missing-wedge-corrected particle. The sixth and seventh columns present the final 3D map and the flexibly fitted model, respectively. (B) Two cross-sectional views (12 nm thickness) of the plasmid density map along its plectoneme axis are shown in the left-middle panel. The intensity profile along the line crossing the two high-density DNA spots is displayed in the right panel. (C) Resolution assessment of the final 3D map using Fourier shell correlation (FSC). Two criteria are shown: FSC between two half-maps reconstructed from even and odd frames (evaluated at 0.143) and FSC between the final 3D map and the fitted model (evaluated at 0.5). (D) Zoomed-in views of the final 3D density map from panel A, displayed at two contour levels. (E) Superimposition of the high-contour level map from panel D onto its fitted model.

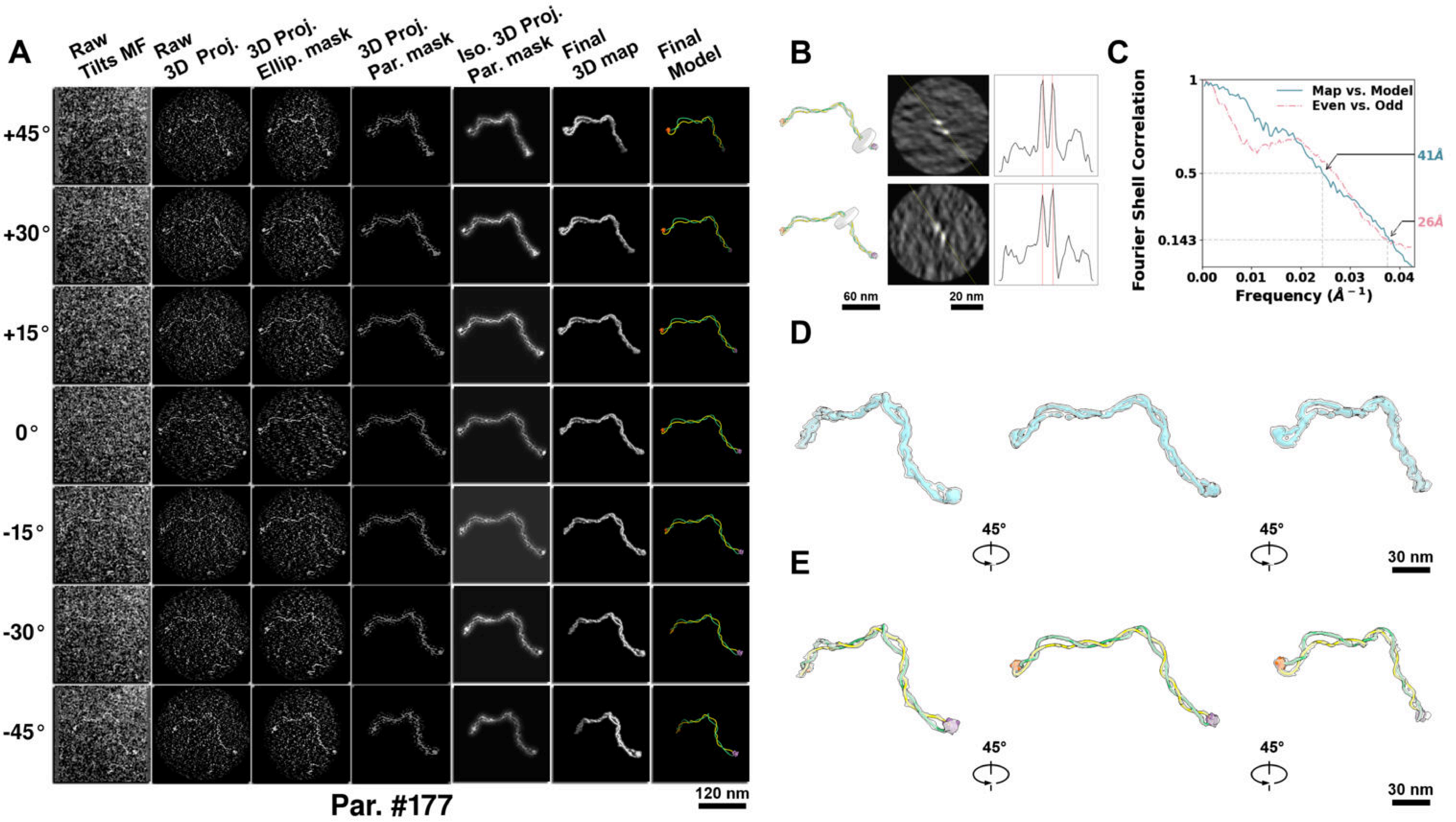

Supplementary Particle Figure 177. Cryo-ET 3D reconstruction of an individual sTEC-Cas particle.
(A) 3D reconstruction of the plasmid particle (index no. 177). The first column shows seven representative tilt images from +45° to -45° in step of 15°. The second, third, and fourth columns show 3D projections of the particle with spherical, ellipsoidal (thinner along the z-dimension), and particle-shaped masks, respectively. The fifth column displays the 3D projections of the enhanced and IsoNet missing-wedge-corrected particle. The sixth and seventh columns present the final 3D map and the flexibly fitted model, respectively. (B) Two cross-sectional views (12 nm thickness) of the plasmid density map along its plectoneme axis are shown in the left-middle panel. The intensity profile along the line crossing the two high-density DNA spots is displayed in the right panel. (C) Resolution assessment of the final 3D map using Fourier shell correlation (FSC). Two criteria are shown: FSC between two half-maps reconstructed from even and odd frames (evaluated at 0.143) and FSC between the final 3D map and the fitted model (evaluated at 0.5). (D) Zoomed-in views of the final 3D density map from panel A, displayed at two contour levels. (E) Superimposition of the high-contour level map from panel D onto its fitted model.

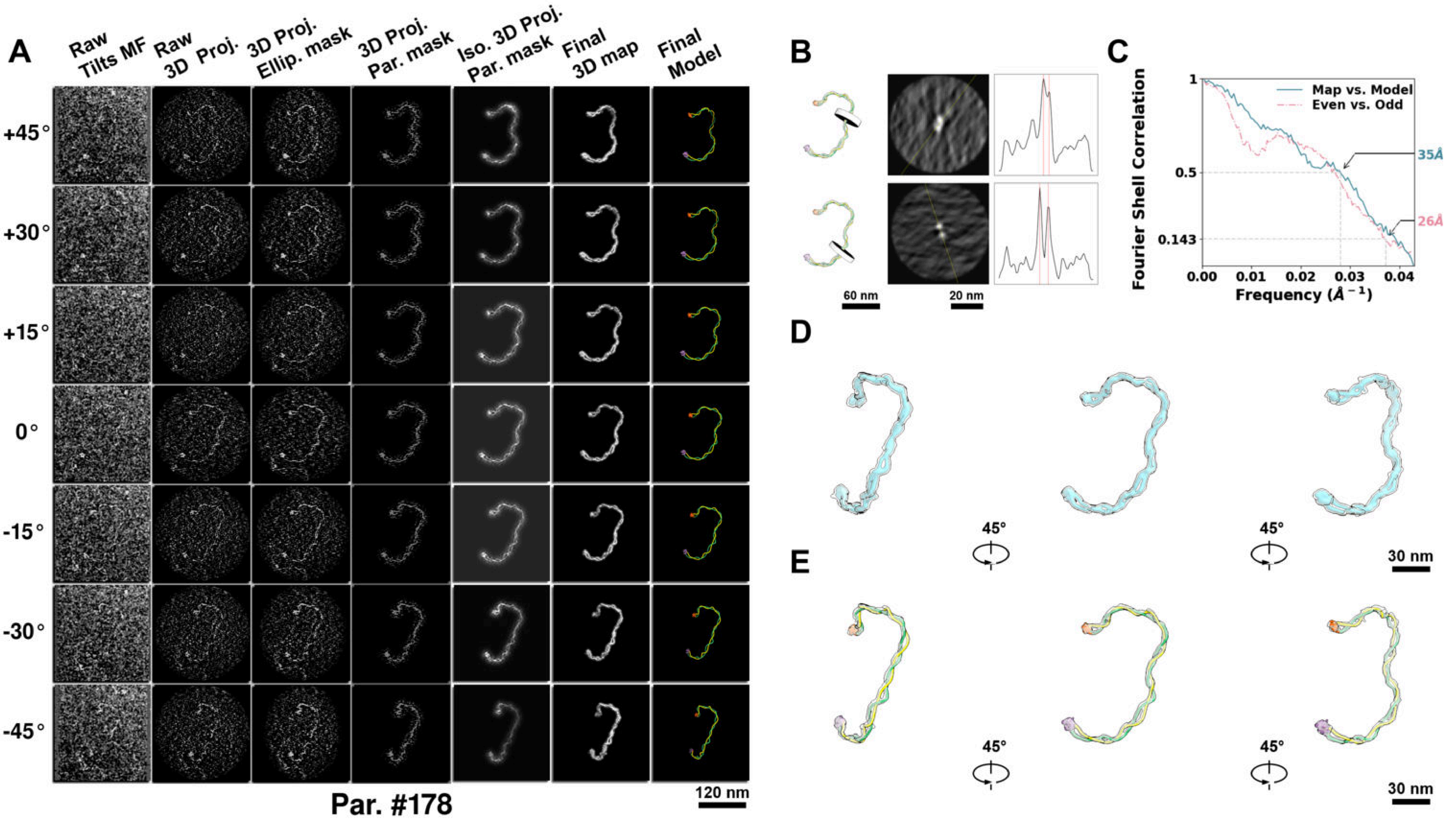

Supplementary Particle Figure 178. Cryo-ET 3D reconstruction of an individual sTEC-Cas particle.
(A) 3D reconstruction of the plasmid particle (index no. 178). The first column shows seven representative tilt images from +45° to -45° in step of 15°. The second, third, and fourth columns show 3D projections of the particle with spherical, ellipsoidal (thinner along the z-dimension), and particle-shaped masks, respectively. The fifth column displays the 3D projections of the enhanced and IsoNet missing-wedge-corrected particle. The sixth and seventh columns present the final 3D map and the flexibly fitted model, respectively. (B) Two cross-sectional views (12 nm thickness) of the plasmid density map along its plectoneme axis are shown in the left-middle panel. The intensity profile along the line crossing the two high-density DNA spots is displayed in the right panel. (C) Resolution assessment of the final 3D map using Fourier shell correlation (FSC). Two criteria are shown: FSC between two half-maps reconstructed from even and odd frames (evaluated at 0.143) and FSC between the final 3D map and the fitted model (evaluated at 0.5). (D) Zoomed-in views of the final 3D density map from panel A, displayed at two contour levels. (E) Superimposition of the high-contour level map from panel D onto its fitted model.

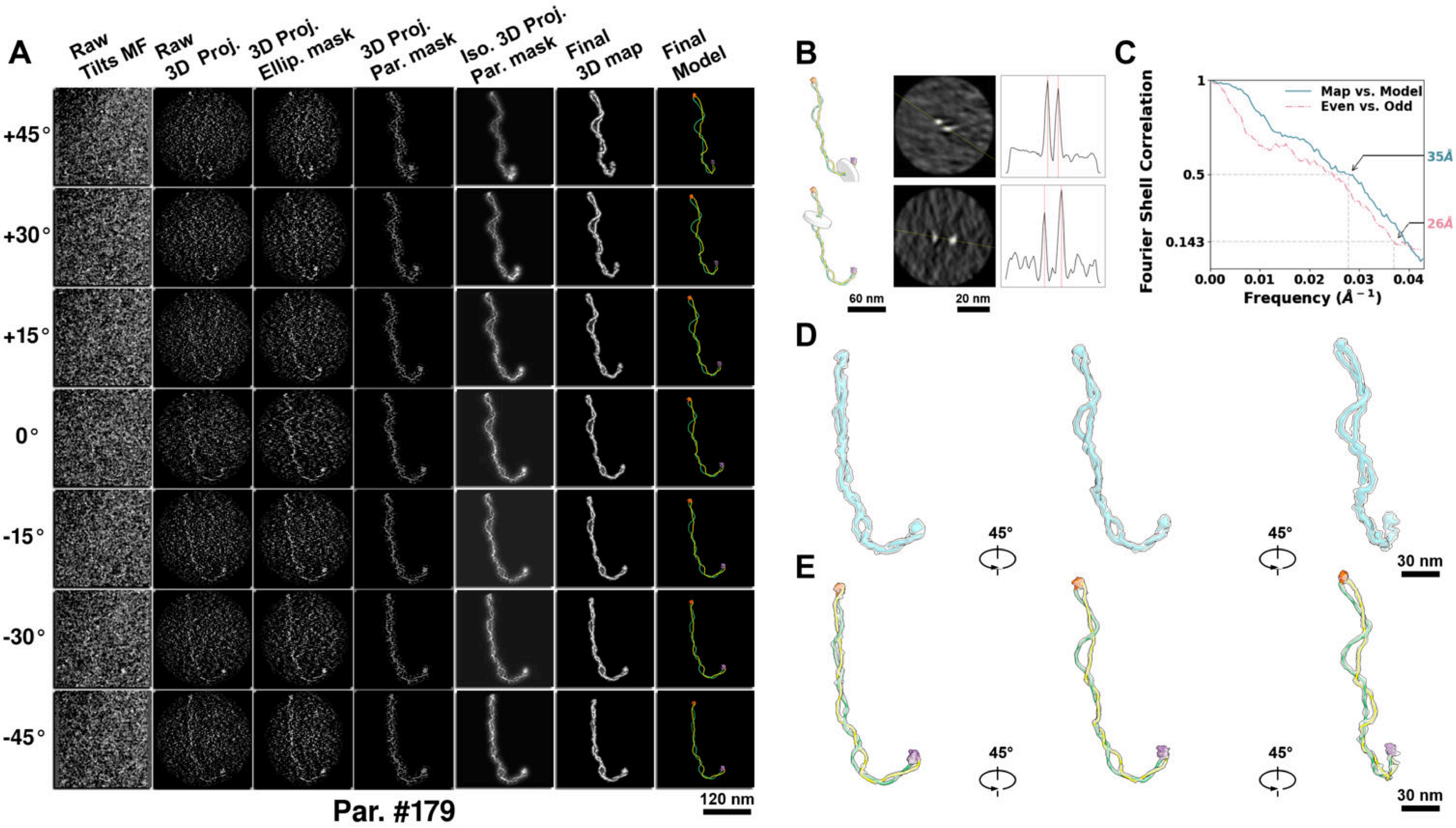

Supplementary Particle Figure 179. Cryo-ET 3D reconstruction of an individual sTEC-Cas particle.
(A) 3D reconstruction of the plasmid particle (index no. 179). The first column shows seven representative tilt images from +45° to -45° in step of 15°. The second, third, and fourth columns show 3D projections of the particle with spherical, ellipsoidal (thinner along the z-dimension), and particle-shaped masks, respectively. The fifth column displays the 3D projections of the enhanced and IsoNet missing-wedge-corrected particle. The sixth and seventh columns present the final 3D map and the flexibly fitted model, respectively. (B) Two cross-sectional views (12 nm thickness) of the plasmid density map along its plectoneme axis are shown in the left-middle panel. The intensity profile along the line crossing the two high-density DNA spots is displayed in the right panel. (C) Resolution assessment of the final 3D map using Fourier shell correlation (FSC). Two criteria are shown: FSC between two half-maps reconstructed from even and odd frames (evaluated at 0.143) and FSC between the final 3D map and the fitted model (evaluated at 0.5). (D) Zoomed-in views of the final 3D density map from panel A, displayed at two contour levels. (E) Superimposition of the high-contour level map from panel D onto its fitted model.

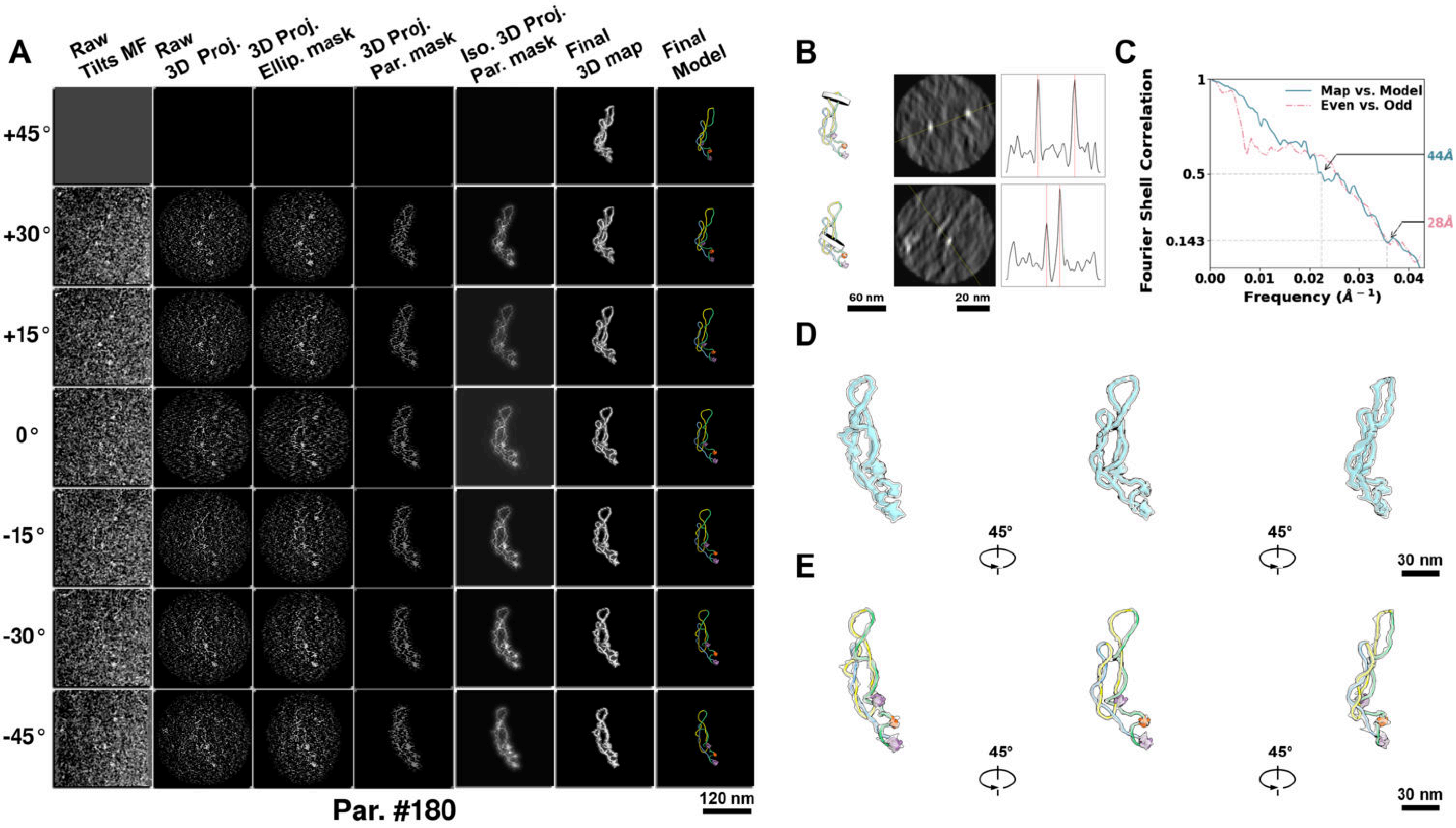

Supplementary Particle Figure 180. Cryo-ET 3D reconstruction of an individual sTEC-Cas particle.
(A) 3D reconstruction of the plasmid particle (index no. 180). The first column shows seven representative tilt images from +45° to -45° in step of 15°. The second, third, and fourth columns show 3D projections of the particle with spherical, ellipsoidal (thinner along the z-dimension), and particle-shaped masks, respectively. The fifth column displays the 3D projections of the enhanced and IsoNet missing-wedge-corrected particle. The sixth and seventh columns present the final 3D map and the flexibly fitted model, respectively. (B) Two cross-sectional views (12 nm thickness) of the plasmid density map along its plectoneme axis are shown in the left-middle panel. The intensity profile along the line crossing the two high-density DNA spots is displayed in the right panel. (C) Resolution assessment of the final 3D map using Fourier shell correlation (FSC). Two criteria are shown: FSC between two half-maps reconstructed from even and odd frames (evaluated at 0.143) and FSC between the final 3D map and the fitted model (evaluated at 0.5). (D) Zoomed-in views of the final 3D density map from panel A, displayed at two contour levels. (E) Superimposition of the high-contour level map from panel D onto its fitted model.

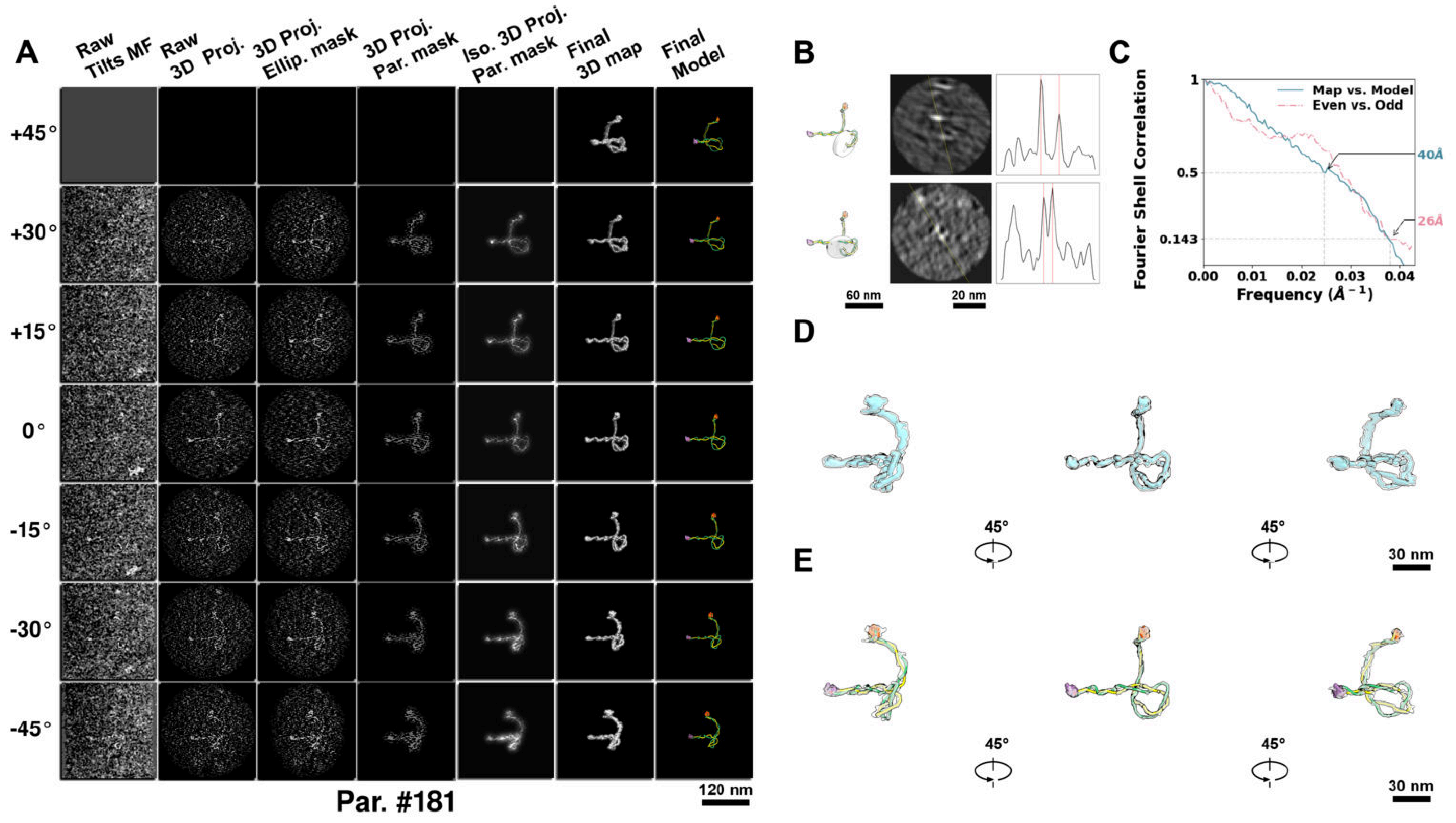

Supplementary Particle Figure 181. Cryo-ET 3D reconstruction of an individual sTEC-Cas particle.
(A) 3D reconstruction of the plasmid particle (index no. 181). The first column shows seven representative tilt images from +45° to -45° in step of 15°. The second, third, and fourth columns show 3D projections of the particle with spherical, ellipsoidal (thinner along the z-dimension), and particle-shaped masks, respectively. The fifth column displays the 3D projections of the enhanced and IsoNet missing-wedge-corrected particle. The sixth and seventh columns present the final 3D map and the flexibly fitted model, respectively. (B) Two cross-sectional views (12 nm thickness) of the plasmid density map along its plectoneme axis are shown in the left-middle panel. The intensity profile along the line crossing the two high-density DNA spots is displayed in the right panel. (C) Resolution assessment of the final 3D map using Fourier shell correlation (FSC). Two criteria are shown: FSC between two half-maps reconstructed from even and odd frames (evaluated at 0.143) and FSC between the final 3D map and the fitted model (evaluated at 0.5). (D) Zoomed-in views of the final 3D density map from panel A, displayed at two contour levels. (E) Superimposition of the high-contour level map from panel D onto its fitted model.

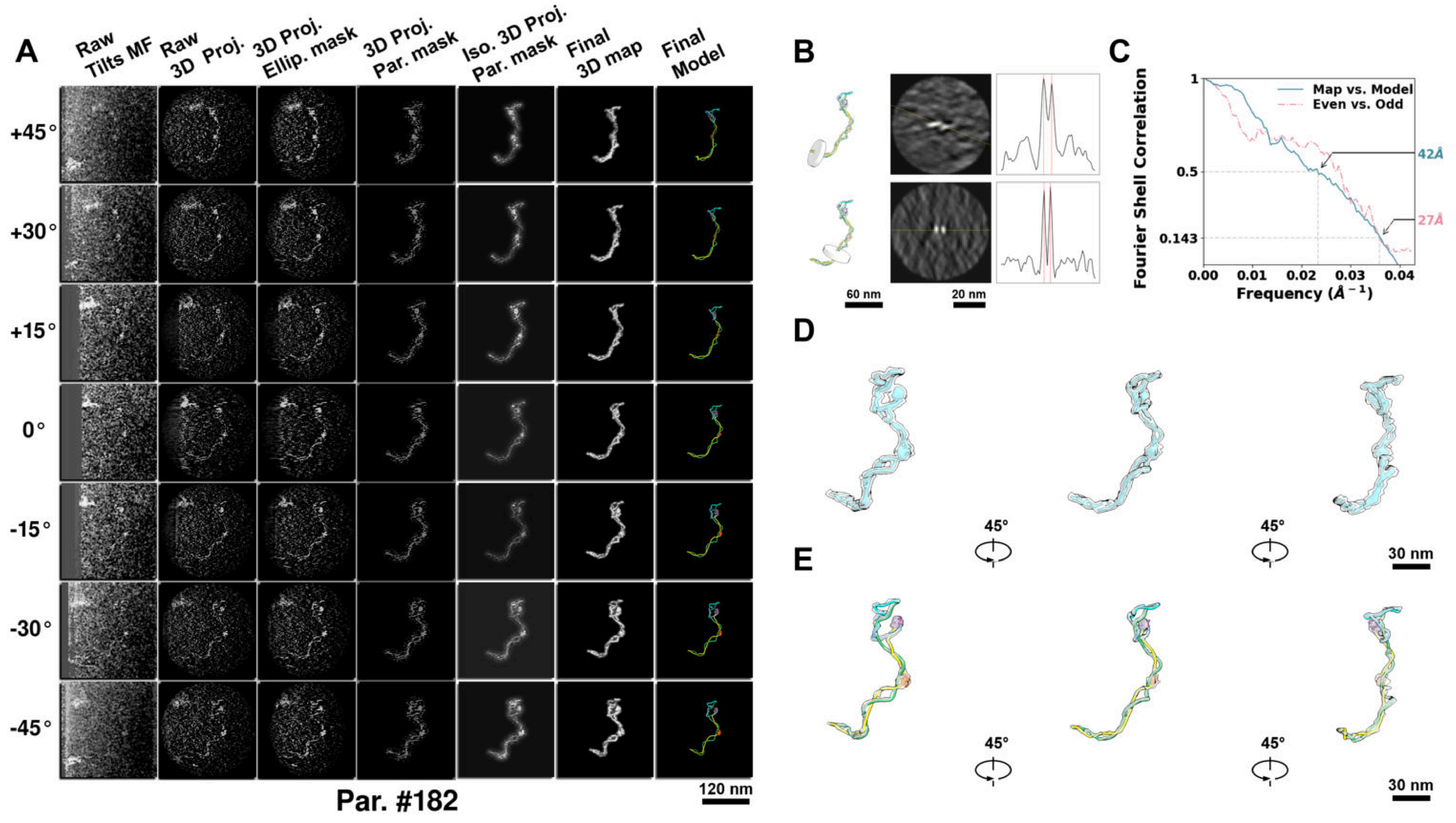

Supplementary Particle Figure 182. Cryo-ET 3D reconstruction of an individual sTEC-Cas particle.
(A) 3D reconstruction of the plasmid particle (index no. 182). The first column shows seven representative tilt images from +45° to -45° in step of 15°. The second, third, and fourth columns show 3D projections of the particle with spherical, ellipsoidal (thinner along the z-dimension), and particle-shaped masks, respectively. The fifth column displays the 3D projections of the enhanced and IsoNet missing-wedge-corrected particle. The sixth and seventh columns present the final 3D map and the flexibly fitted model, respectively. (B) Two cross-sectional views (12 nm thickness) of the plasmid density map along its plectoneme axis are shown in the left-middle panel. The intensity profile along the line crossing the two high-density DNA spots is displayed in the right panel. (C) Resolution assessment of the final 3D map using Fourier shell correlation (FSC). Two criteria are shown: FSC between two half-maps reconstructed from even and odd frames (evaluated at 0.143) and FSC between the final 3D map and the fitted model (evaluated at 0.5). (D) Zoomed-in views of the final 3D density map from panel A, displayed at two contour levels. (E) Superimposition of the high-contour level map from panel D onto its fitted model.

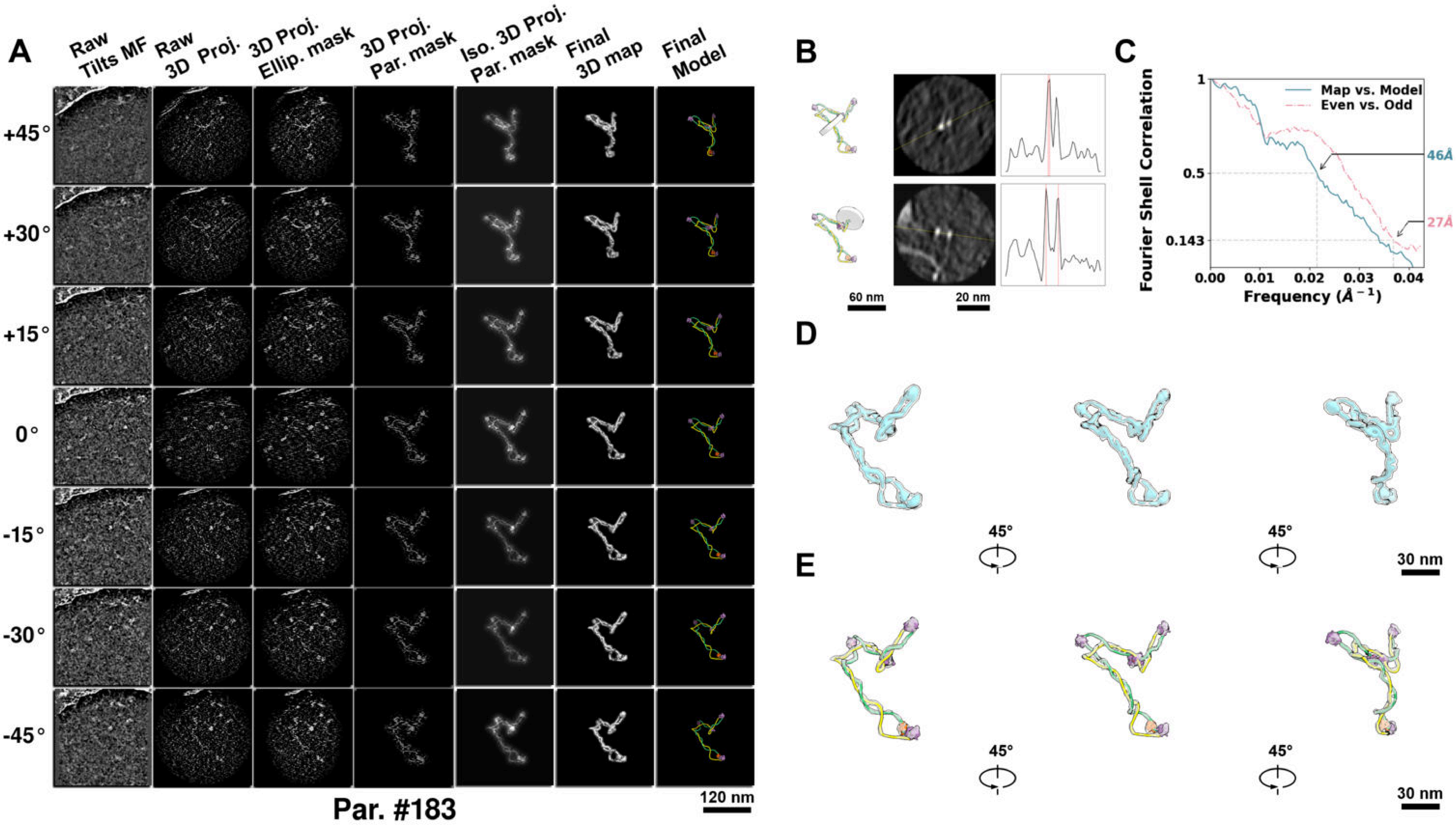

Supplementary Particle Figure 183. Cryo-ET 3D reconstruction of an individual TEC-Cas particle.
(A) 3D reconstruction of the plasmid particle (index no. 183). The first column shows seven representative tilt images from +45° to -45° in step of 15°. The second, third, and fourth columns show 3D projections of the particle with spherical, ellipsoidal (thinner along the z-dimension), and particle-shaped masks, respectively. The fifth column displays the 3D projections of the enhanced and IsoNet missing-wedge-corrected particle. The sixth and seventh columns present the final 3D map and the flexibly fitted model, respectively. (B) Two cross-sectional views (12 nm thickness) of the plasmid density map along its plectoneme axis are shown in the left-middle panel. The intensity profile along the line crossing the two high-density DNA spots is displayed in the right panel. (C) Resolution assessment of the final 3D map using Fourier shell correlation (FSC). Two criteria are shown: FSC between two half-maps reconstructed from even and odd frames (evaluated at 0.143) and FSC between the final 3D map and the fitted model (evaluated at 0.5). (D) Zoomed-in views of the final 3D density map from panel A, displayed at two contour levels. (E) Superimposition of the high-contour level map from panel D onto its fitted model.

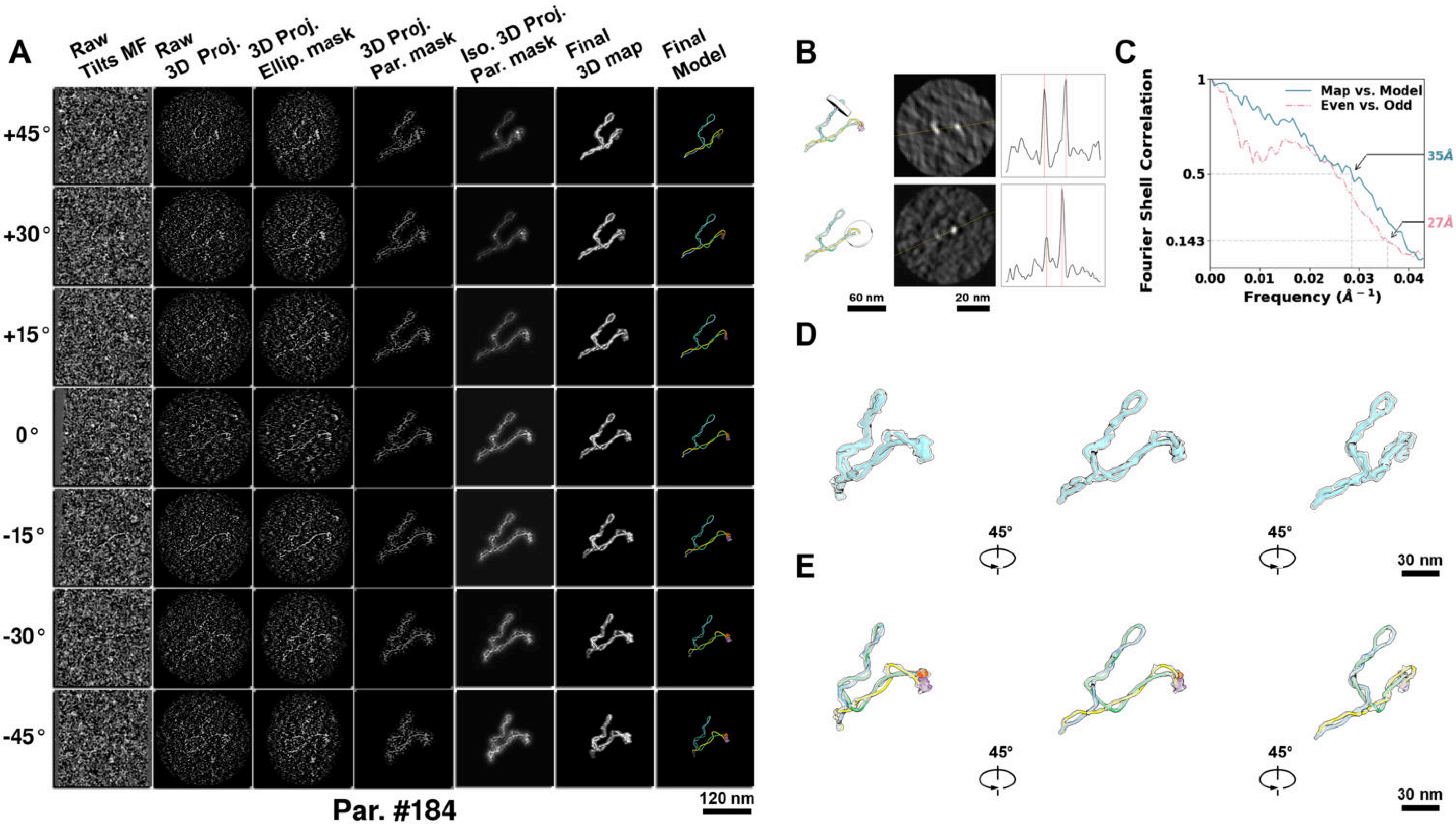

Supplementary Particle Figure 184. Cryo-ET 3D reconstruction of an individual TEC-Cas particle.
(A) 3D reconstruction of the plasmid particle (index no. 184). The first column shows seven representative tilt images from +45° to -45° in step of 15°. The second, third, and fourth columns show 3D projections of the particle with spherical, ellipsoidal (thinner along the z-dimension), and particle-shaped masks, respectively. The fifth column displays the 3D projections of the enhanced and IsoNet missing-wedge-corrected particle. The sixth and seventh columns present the final 3D map and the flexibly fitted model, respectively. (B) Two cross-sectional views (12 nm thickness) of the plasmid density map along its plectoneme axis are shown in the left-middle panel. The intensity profile along the line crossing the two high-density DNA spots is displayed in the right panel. (C) Resolution assessment of the final 3D map using Fourier shell correlation (FSC). Two criteria are shown: FSC between two half-maps reconstructed from even and odd frames (evaluated at 0.143) and FSC between the final 3D map and the fitted model (evaluated at 0.5). (D) Zoomed-in views of the final 3D density map from panel A, displayed at two contour levels. (E) Superimposition of the high-contour level map from panel D onto its fitted model.

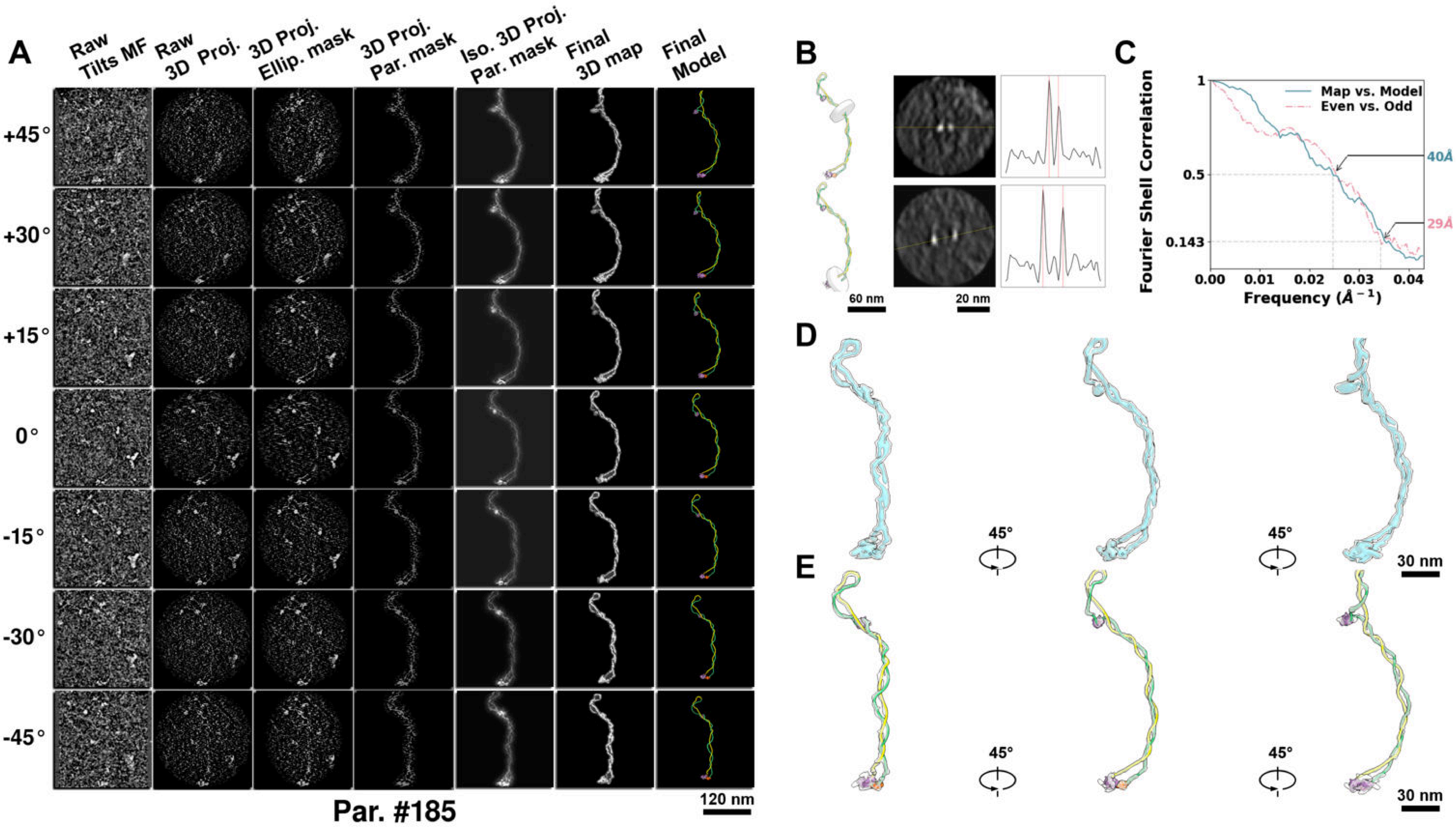

Supplementary Particle Figure 185. Cryo-ET 3D reconstruction of an individual TEC-Cas particle.
(A) 3D reconstruction of the plasmid particle (index no. 185). The first column shows seven representative tilt images from +45° to -45° in step of 15°. The second, third, and fourth columns show 3D projections of the particle with spherical, ellipsoidal (thinner along the z-dimension), and particle-shaped masks, respectively. The fifth column displays the 3D projections of the enhanced and IsoNet missing-wedge-corrected particle. The sixth and seventh columns present the final 3D map and the flexibly fitted model, respectively. (B) Two cross-sectional views (12 nm thickness) of the plasmid density map along its plectoneme axis are shown in the left-middle panel. The intensity profile along the line crossing the two high-density DNA spots is displayed in the right panel. (C) Resolution assessment of the final 3D map using Fourier shell correlation (FSC). Two criteria are shown: FSC between two half-maps reconstructed from even and odd frames (evaluated at 0.143) and FSC between the final 3D map and the fitted model (evaluated at 0.5). (D) Zoomed-in views of the final 3D density map from panel A, displayed at two contour levels. (E) Superimposition of the high-contour level map from panel D onto its fitted model.

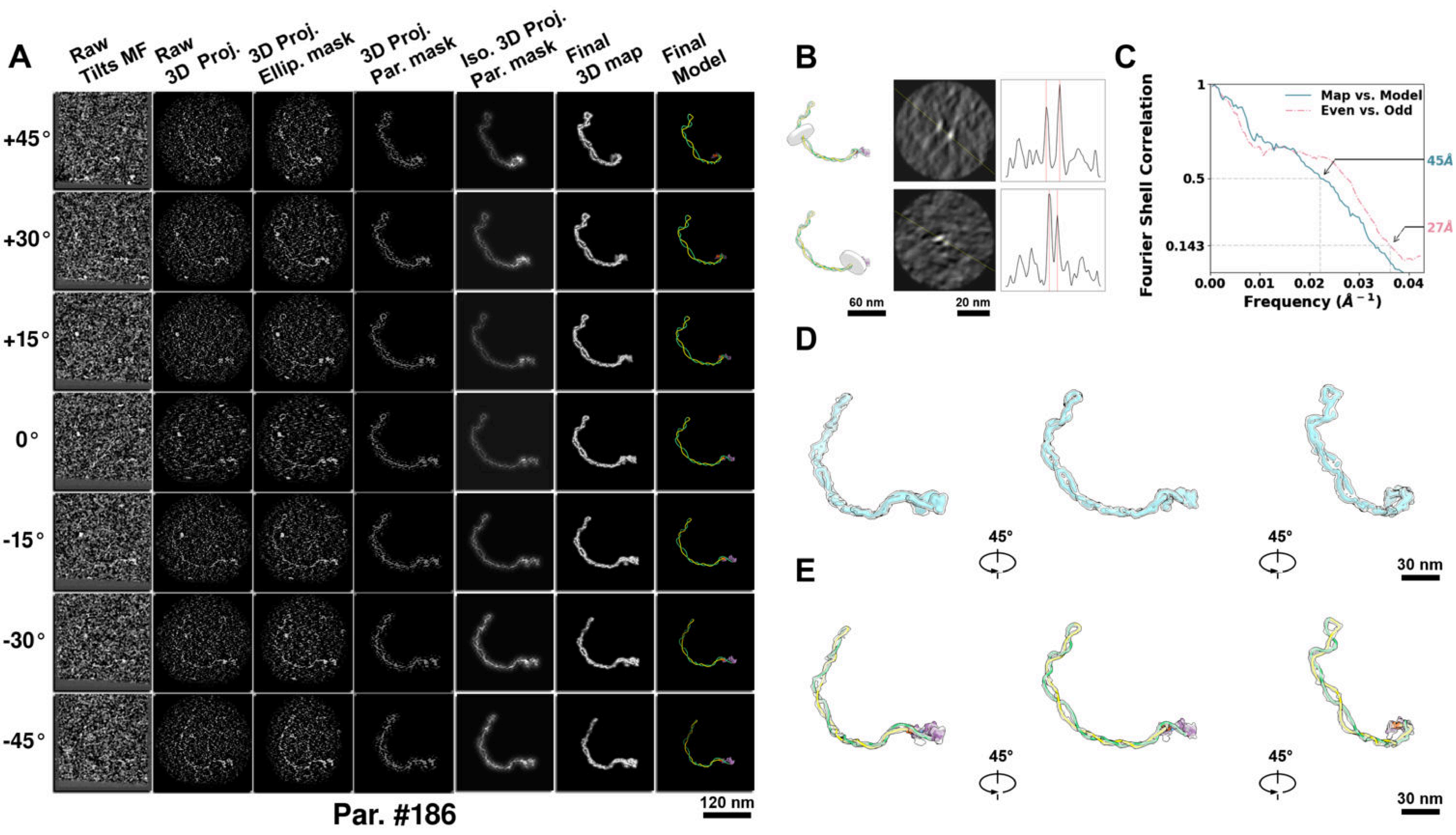

Supplementary Particle Figure 186. Cryo-ET 3D reconstruction of an individual TEC-Cas particle.
(A) 3D reconstruction of the plasmid particle (index no. 186). The first column shows seven representative tilt images from +45° to -45° in step of 15°. The second, third, and fourth columns show 3D projections of the particle with spherical, ellipsoidal (thinner along the z-dimension), and particle-shaped masks, respectively. The fifth column displays the 3D projections of the enhanced and IsoNet missing-wedge-corrected particle. The sixth and seventh columns present the final 3D map and the flexibly fitted model, respectively. (B) Two cross-sectional views (12 nm thickness) of the plasmid density map along its plectoneme axis are shown in the left-middle panel. The intensity profile along the line crossing the two high-density DNA spots is displayed in the right panel. (C) Resolution assessment of the final 3D map using Fourier shell correlation (FSC). Two criteria are shown: FSC between two half-maps reconstructed from even and odd frames (evaluated at 0.143) and FSC between the final 3D map and the fitted model (evaluated at 0.5). (D) Zoomed-in views of the final 3D density map from panel A, displayed at two contour levels. (E) Superimposition of the high-contour level map from panel D onto its fitted model.

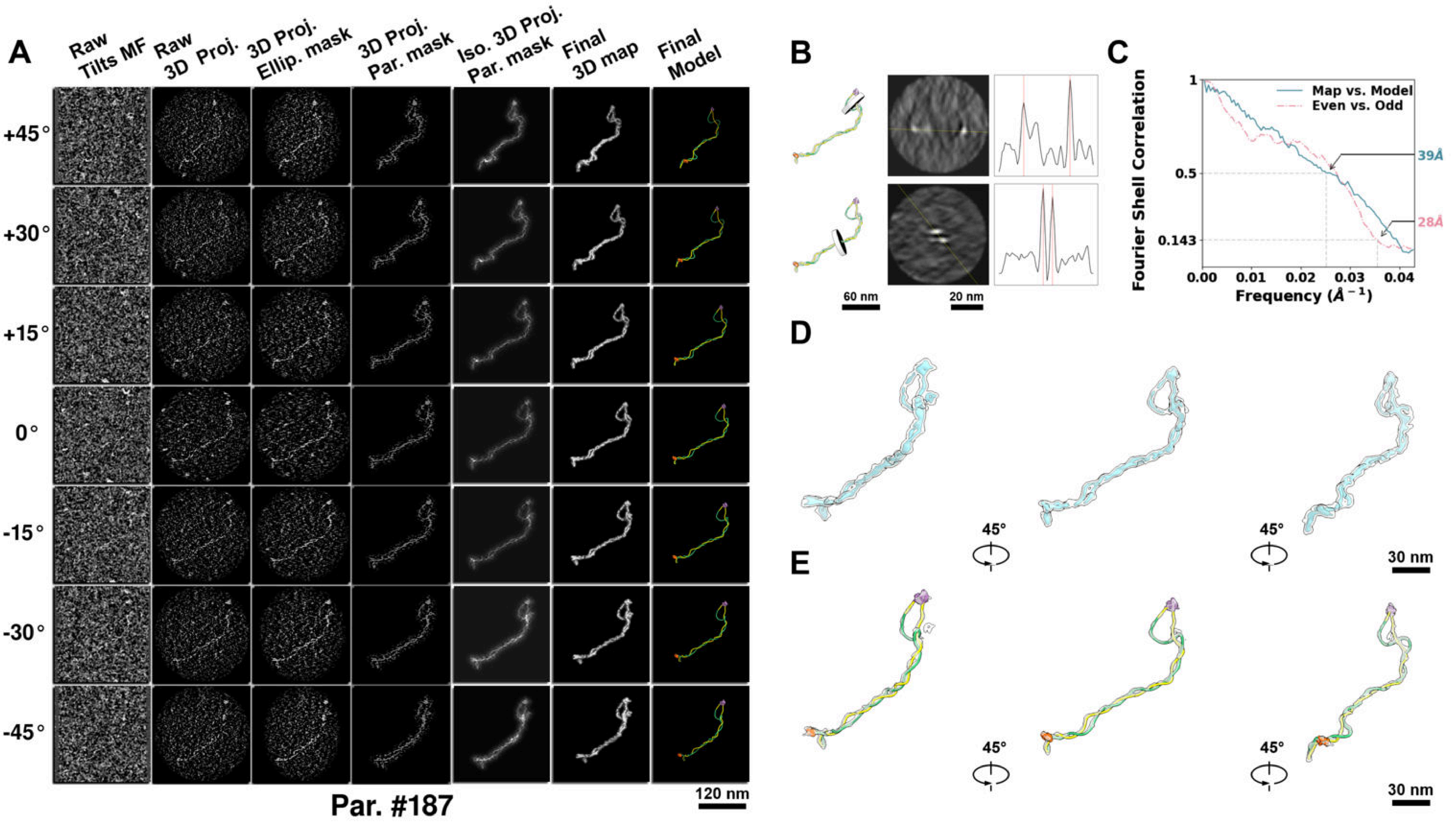

Supplementary Particle Figure 187. Cryo-ET 3D reconstruction of an individual TEC-Cas particle.
(A) 3D reconstruction of the plasmid particle (index no. 187). The first column shows seven representative tilt images from +45° to -45° in step of 15°. The second, third, and fourth columns show 3D projections of the particle with spherical, ellipsoidal (thinner along the z-dimension), and particle-shaped masks, respectively. The fifth column displays the 3D projections of the enhanced and IsoNet missing-wedge-corrected particle. The sixth and seventh columns present the final 3D map and the flexibly fitted model, respectively. (B) Two cross-sectional views (12 nm thickness) of the plasmid density map along its plectoneme axis are shown in the left-middle panel. The intensity profile along the line crossing the two high-density DNA spots is displayed in the right panel. (C) Resolution assessment of the final 3D map using Fourier shell correlation (FSC). Two criteria are shown: FSC between two half-maps reconstructed from even and odd frames (evaluated at 0.143) and FSC between the final 3D map and the fitted model (evaluated at 0.5). (D) Zoomed-in views of the final 3D density map from panel A, displayed at two contour levels. (E) Superimposition of the high-contour level map from panel D onto its fitted model.

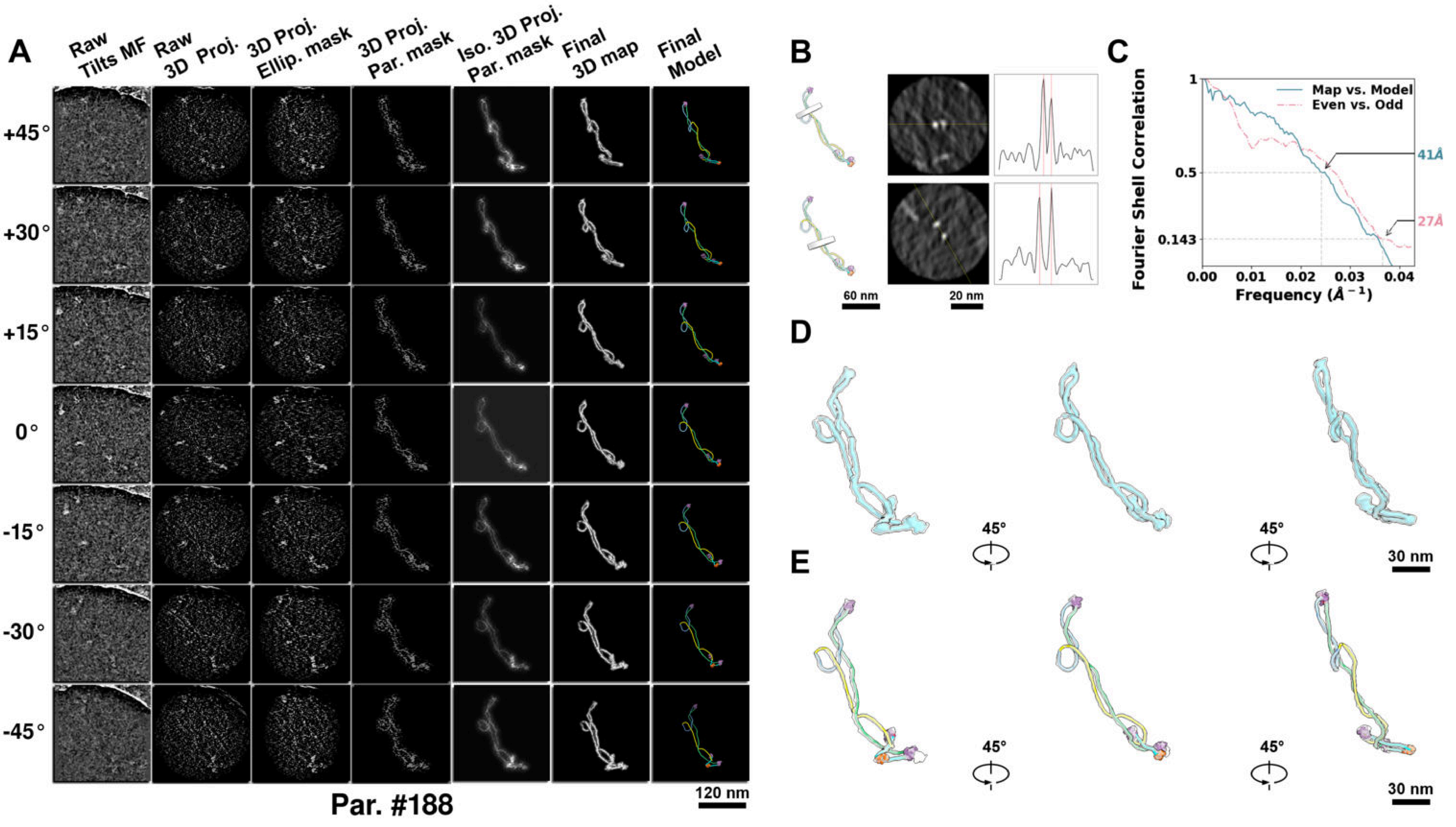

Supplementary Particle Figure 188. Cryo-ET 3D reconstruction of an individual TEC-Cas particle.
(A) 3D reconstruction of the plasmid particle (index no. 188). The first column shows seven representative tilt images from +45° to -45° in step of 15°. The second, third, and fourth columns show 3D projections of the particle with spherical, ellipsoidal (thinner along the z-dimension), and particle-shaped masks, respectively. The fifth column displays the 3D projections of the enhanced and IsoNet missing-wedge-corrected particle. The sixth and seventh columns present the final 3D map and the flexibly fitted model, respectively. (B) Two cross-sectional views (12 nm thickness) of the plasmid density map along its plectoneme axis are shown in the left-middle panel. The intensity profile along the line crossing the two high-density DNA spots is displayed in the right panel. (C) Resolution assessment of the final 3D map using Fourier shell correlation (FSC). Two criteria are shown: FSC between two half-maps reconstructed from even and odd frames (evaluated at 0.143) and FSC between the final 3D map and the fitted model (evaluated at 0.5). (D) Zoomed-in views of the final 3D density map from panel A, displayed at two contour levels. (E) Superimposition of the high-contour level map from panel D onto its fitted model.

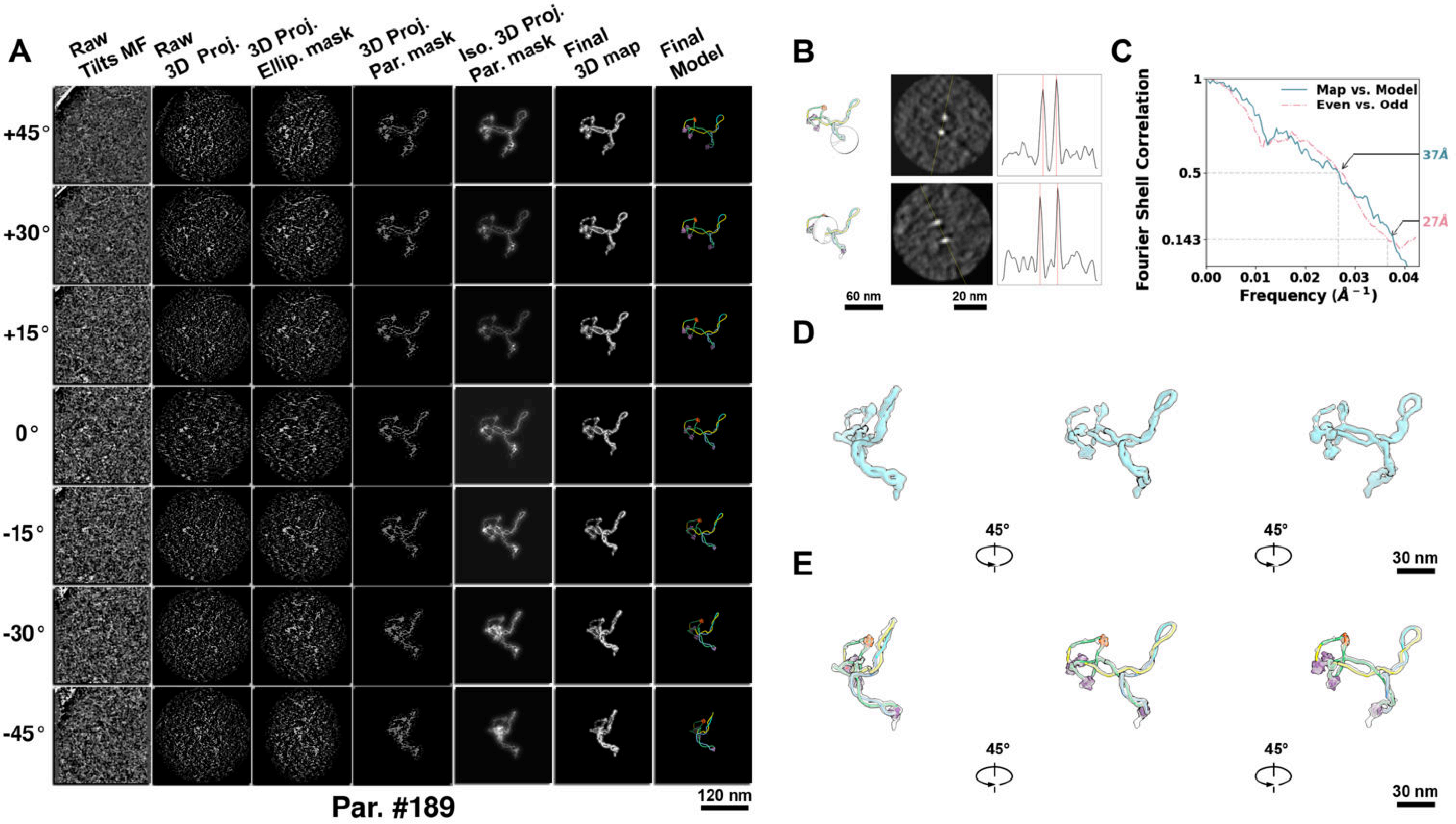

Supplementary Particle Figure 189. Cryo-ET 3D reconstruction of an individual TEC-Cas particle.
(A) 3D reconstruction of the plasmid particle (index no. 189). The first column shows seven representative tilt images from +45° to -45° in step of 15°. The second, third, and fourth columns show 3D projections of the particle with spherical, ellipsoidal (thinner along the z-dimension), and particle-shaped masks, respectively. The fifth column displays the 3D projections of the enhanced and IsoNet missing-wedge-corrected particle. The sixth and seventh columns present the final 3D map and the flexibly fitted model, respectively. (B) Two cross-sectional views (12 nm thickness) of the plasmid density map along its plectoneme axis are shown in the left-middle panel. The intensity profile along the line crossing the two high-density DNA spots is displayed in the right panel. (C) Resolution assessment of the final 3D map using Fourier shell correlation (FSC). Two criteria are shown: FSC between two half-maps reconstructed from even and odd frames (evaluated at 0.143) and FSC between the final 3D map and the fitted model (evaluated at 0.5). (D) Zoomed-in views of the final 3D density map from panel A, displayed at two contour levels. (E) Superimposition of the high-contour level map from panel D onto its fitted model.

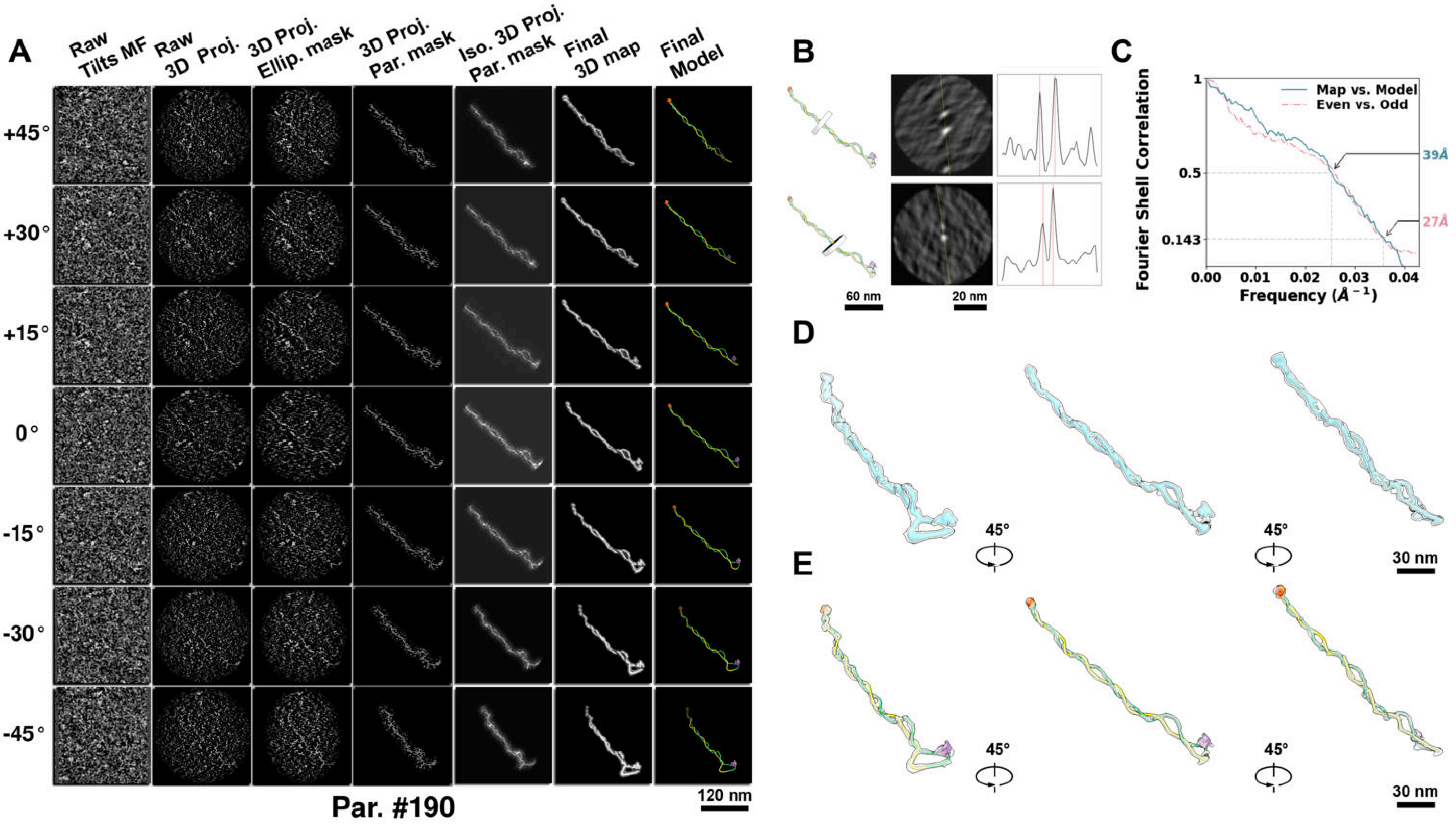

Supplementary Particle Figure 190. Cryo-ET 3D reconstruction of an individual TEC-Cas particle.
(A) 3D reconstruction of the plasmid particle (index no. 190). The first column shows seven representative tilt images from +45° to -45° in step of 15°. The second, third, and fourth columns show 3D projections of the particle with spherical, ellipsoidal (thinner along the z-dimension), and particle-shaped masks, respectively. The fifth column displays the 3D projections of the enhanced and IsoNet missing-wedge-corrected particle. The sixth and seventh columns present the final 3D map and the flexibly fitted model, respectively. (B) Two cross-sectional views (12 nm thickness) of the plasmid density map along its plectoneme axis are shown in the left-middle panel. The intensity profile along the line crossing the two high-density DNA spots is displayed in the right panel. (C) Resolution assessment of the final 3D map using Fourier shell correlation (FSC). Two criteria are shown: FSC between two half-maps reconstructed from even and odd frames (evaluated at 0.143) and FSC between the final 3D map and the fitted model (evaluated at 0.5). (D) Zoomed-in views of the final 3D density map from panel A, displayed at two contour levels. (E) Superimposition of the high-contour level map from panel D onto its fitted model.

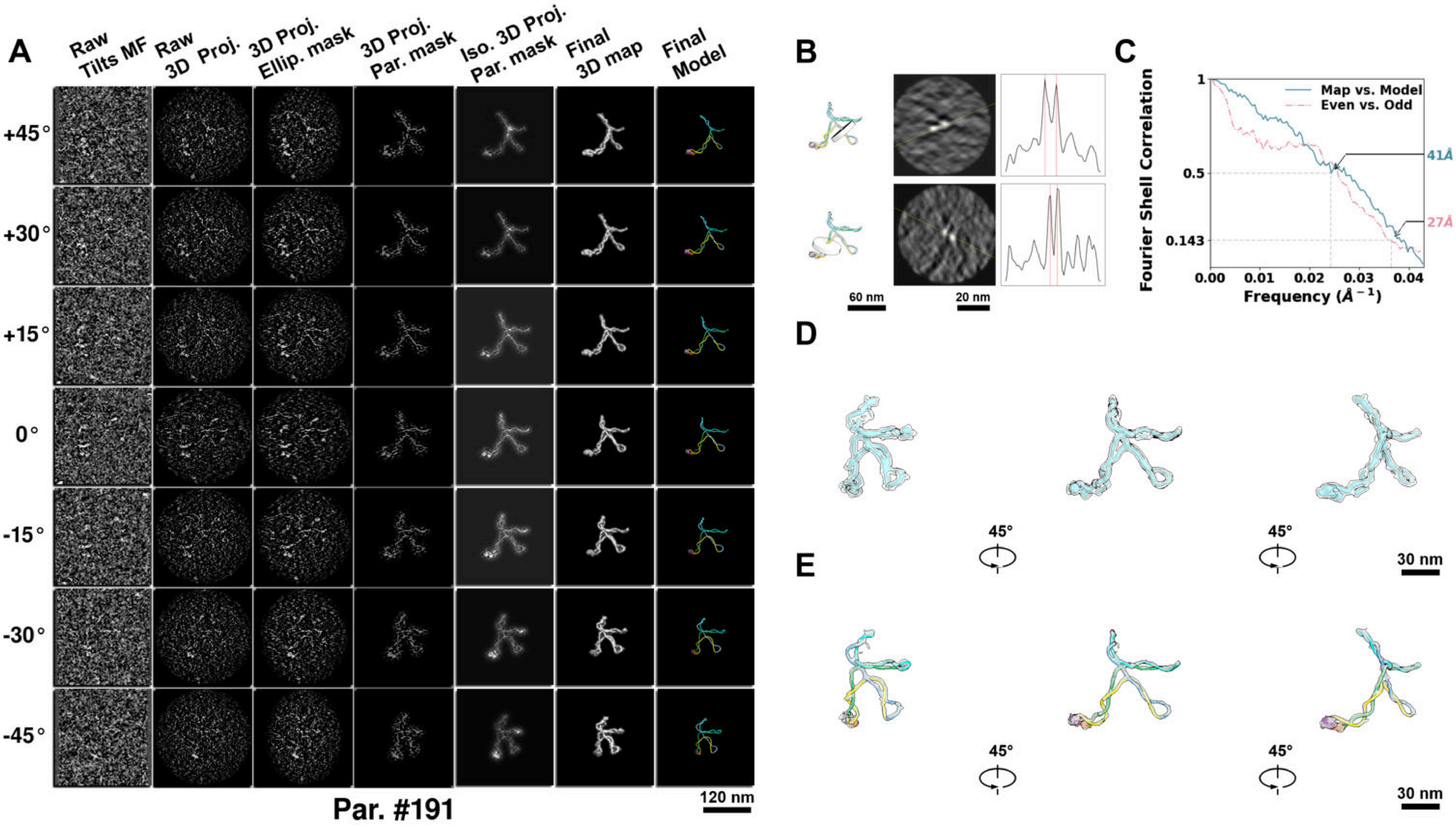

Supplementary Particle Figure 191. Cryo-ET 3D reconstruction of an individual TEC-Cas particle.
(A) 3D reconstruction of the plasmid particle (index no. 191). The first column shows seven representative tilt images from +45° to -45° in step of 15°. The second, third, and fourth columns show 3D projections of the particle with spherical, ellipsoidal (thinner along the z-dimension), and particle-shaped masks, respectively. The fifth column displays the 3D projections of the enhanced and IsoNet missing-wedge-corrected particle. The sixth and seventh columns present the final 3D map and the flexibly fitted model, respectively. (B) Two cross-sectional views (12 nm thickness) of the plasmid density map along its plectoneme axis are shown in the left-middle panel. The intensity profile along the line crossing the two high-density DNA spots is displayed in the right panel. (C) Resolution assessment of the final 3D map using Fourier shell correlation (FSC). Two criteria are shown: FSC between two half-maps reconstructed from even and odd frames (evaluated at 0.143) and FSC between the final 3D map and the fitted model (evaluated at 0.5). (D) Zoomed-in views of the final 3D density map from panel A, displayed at two contour levels. (E) Superimposition of the high-contour level map from panel D onto its fitted model.

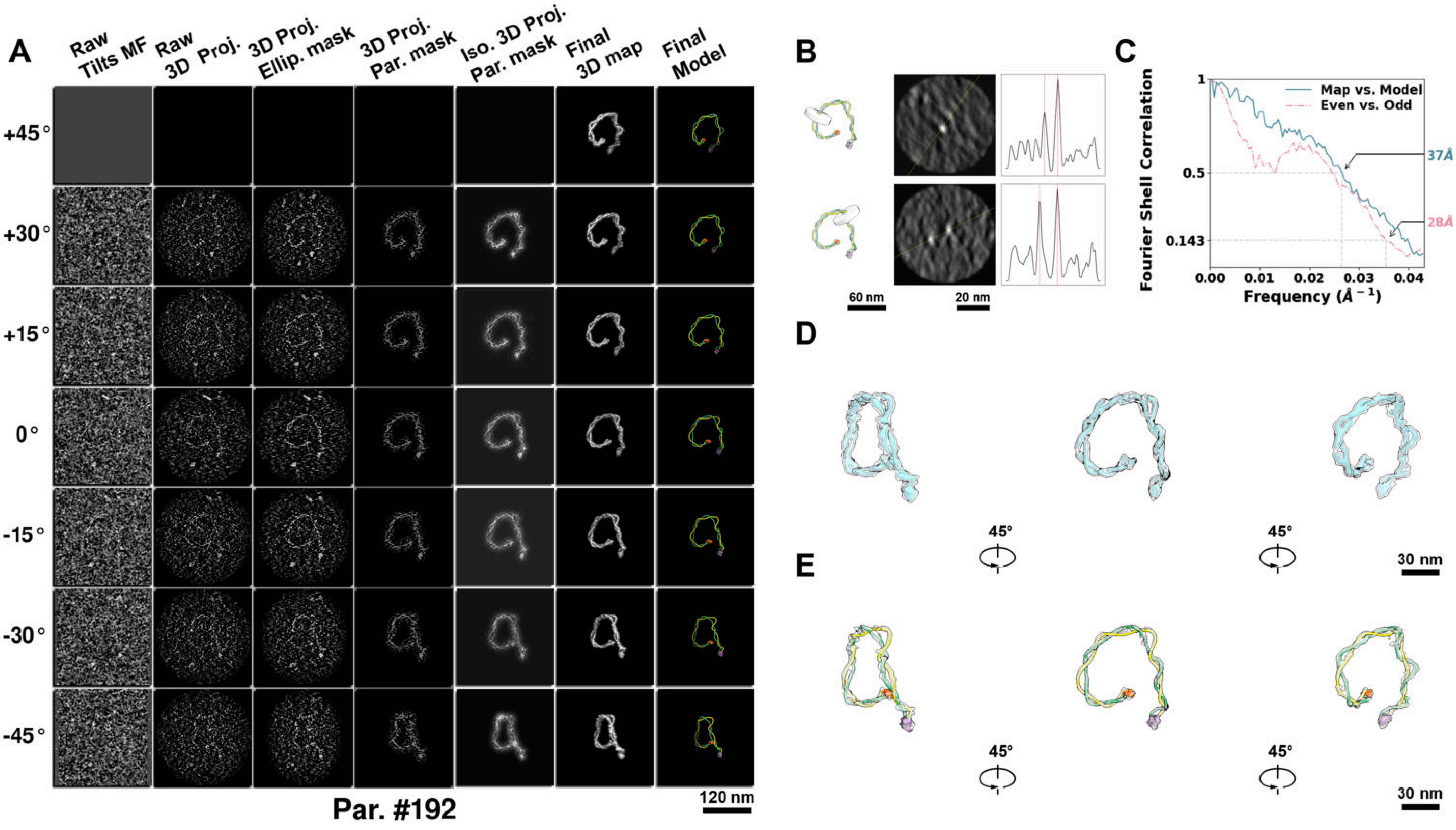

Supplementary Particle Figure 192. Cryo-ET 3D reconstruction of an individual TEC-Cas particle.
(A) 3D reconstruction of the plasmid particle (index no. 192). The first column shows seven representative tilt images from +45° to -45° in step of 15°. The second, third, and fourth columns show 3D projections of the particle with spherical, ellipsoidal (thinner along the z-dimension), and particle-shaped masks, respectively. The fifth column displays the 3D projections of the enhanced and IsoNet missing-wedge-corrected particle. The sixth and seventh columns present the final 3D map and the flexibly fitted model, respectively. (B) Two cross-sectional views (12 nm thickness) of the plasmid density map along its plectoneme axis are shown in the left-middle panel. The intensity profile along the line crossing the two high-density DNA spots is displayed in the right panel. (C) Resolution assessment of the final 3D map using Fourier shell correlation (FSC). Two criteria are shown: FSC between two half-maps reconstructed from even and odd frames (evaluated at 0.143) and FSC between the final 3D map and the fitted model (evaluated at 0.5). (D) Zoomed-in views of the final 3D density map from panel A, displayed at two contour levels. (E) Superimposition of the high-contour level map from panel D onto its fitted model.

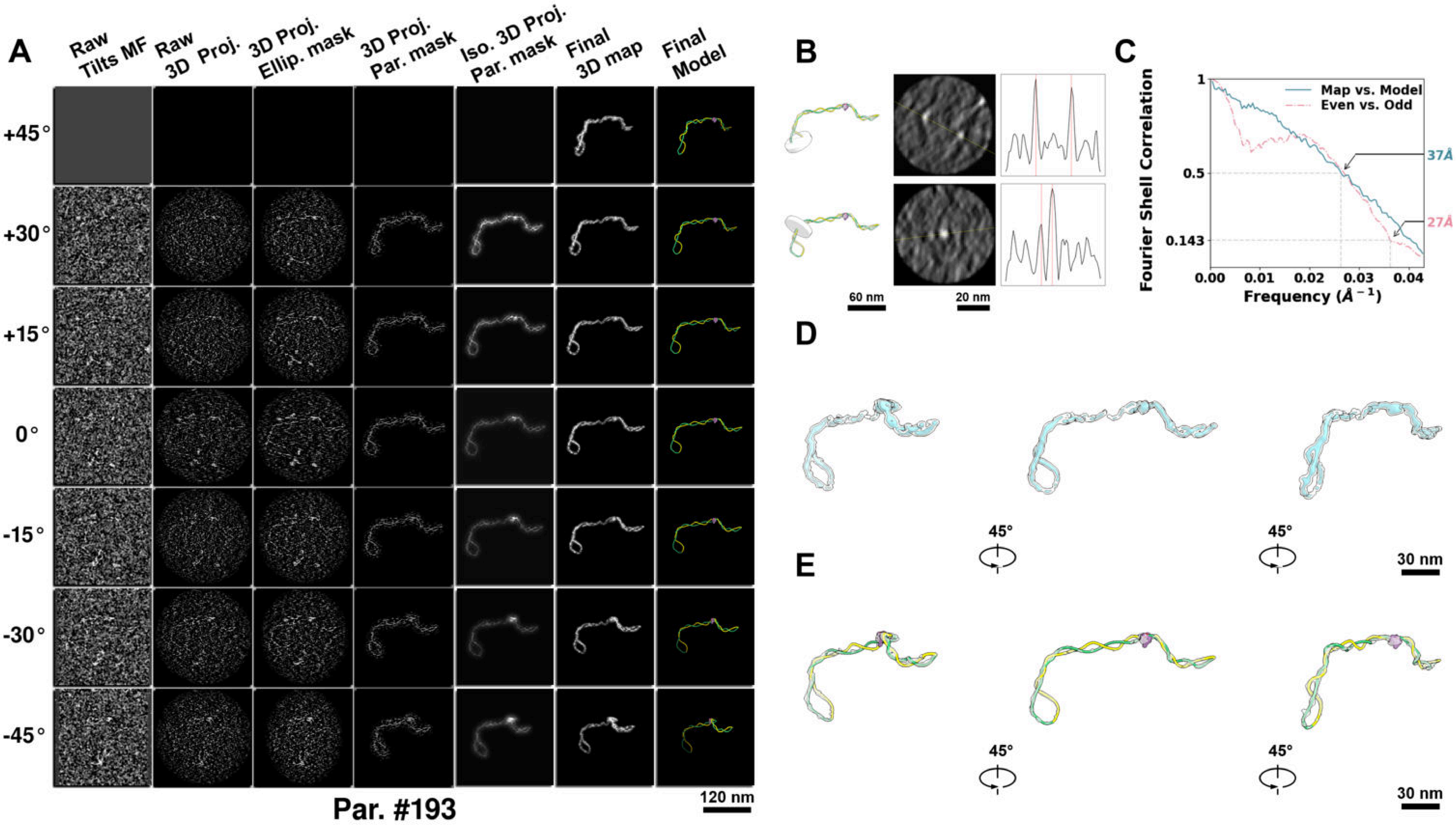

Supplementary Particle Figure 193. Cryo-ET 3D reconstruction of an individual TEC-Cas particle.
(A) 3D reconstruction of the plasmid particle (index no. 193). The first column shows seven representative tilt images from +45° to -45° in step of 15°. The second, third, and fourth columns show 3D projections of the particle with spherical, ellipsoidal (thinner along the z-dimension), and particle-shaped masks, respectively. The fifth column displays the 3D projections of the enhanced and IsoNet missing-wedge-corrected particle. The sixth and seventh columns present the final 3D map and the flexibly fitted model, respectively. (B) Two cross-sectional views (12 nm thickness) of the plasmid density map along its plectoneme axis are shown in the left-middle panel. The intensity profile along the line crossing the two high-density DNA spots is displayed in the right panel. (C) Resolution assessment of the final 3D map using Fourier shell correlation (FSC). Two criteria are shown: FSC between two half-maps reconstructed from even and odd frames (evaluated at 0.143) and FSC between the final 3D map and the fitted model (evaluated at 0.5). (D) Zoomed-in views of the final 3D density map from panel A, displayed at two contour levels. (E) Superimposition of the high-contour level map from panel D onto its fitted model.

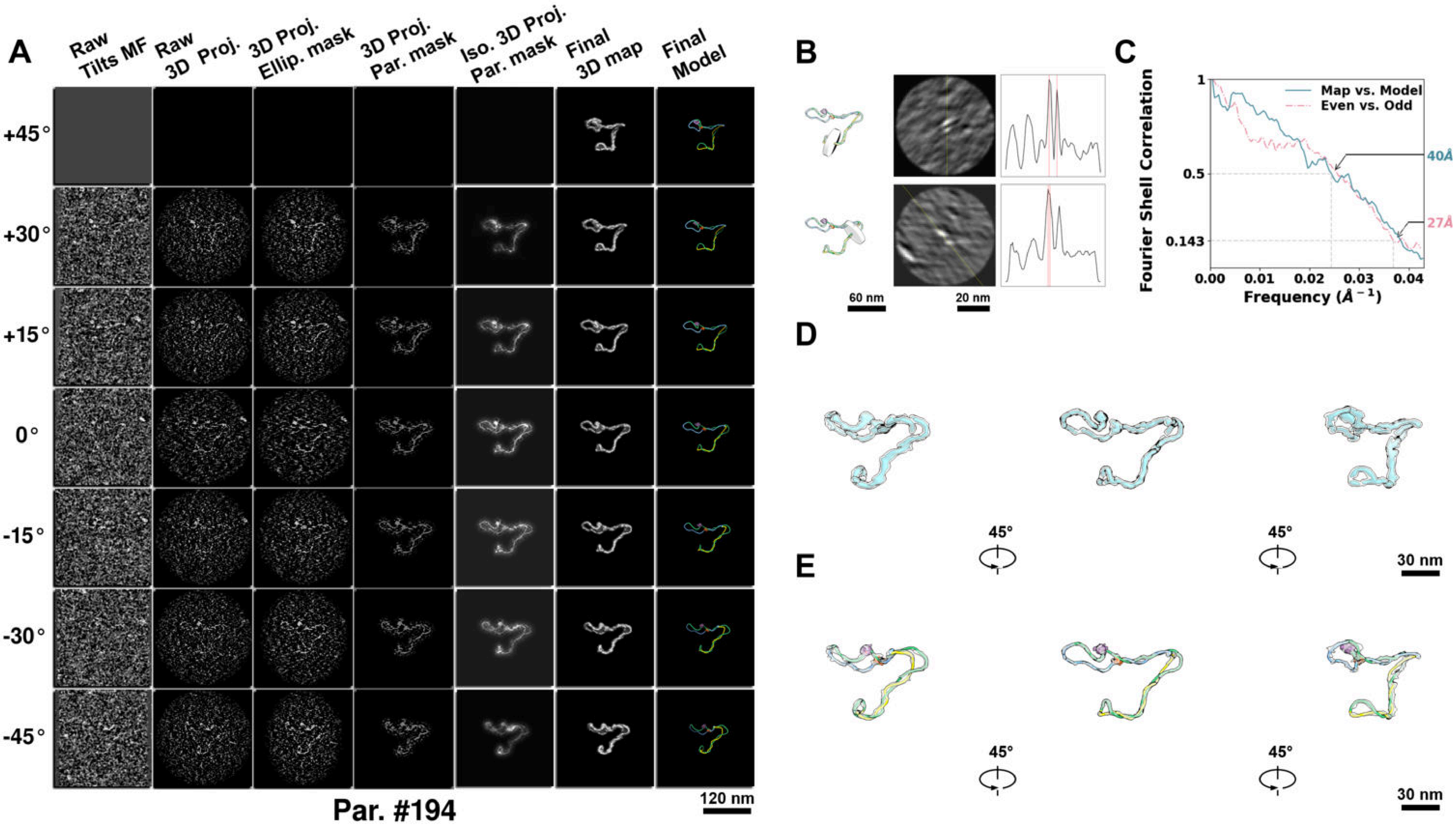

Supplementary Particle Figure 194. Cryo-ET 3D reconstruction of an individual TEC-Cas particle.
(A) 3D reconstruction of the plasmid particle (index no. 194). The first column shows seven representative tilt images from +45° to -45° in step of 15°. The second, third, and fourth columns show 3D projections of the particle with spherical, ellipsoidal (thinner along the z-dimension), and particle-shaped masks, respectively. The fifth column displays the 3D projections of the enhanced and IsoNet missing-wedge-corrected particle. The sixth and seventh columns present the final 3D map and the flexibly fitted model, respectively. (B) Two cross-sectional views (12 nm thickness) of the plasmid density map along its plectoneme axis are shown in the left-middle panel. The intensity profile along the line crossing the two high-density DNA spots is displayed in the right panel. (C) Resolution assessment of the final 3D map using Fourier shell correlation (FSC). Two criteria are shown: FSC between two half-maps reconstructed from even and odd frames (evaluated at 0.143) and FSC between the final 3D map and the fitted model (evaluated at 0.5). (D) Zoomed-in views of the final 3D density map from panel A, displayed at two contour levels. (E) Superimposition of the high-contour level map from panel D onto its fitted model.

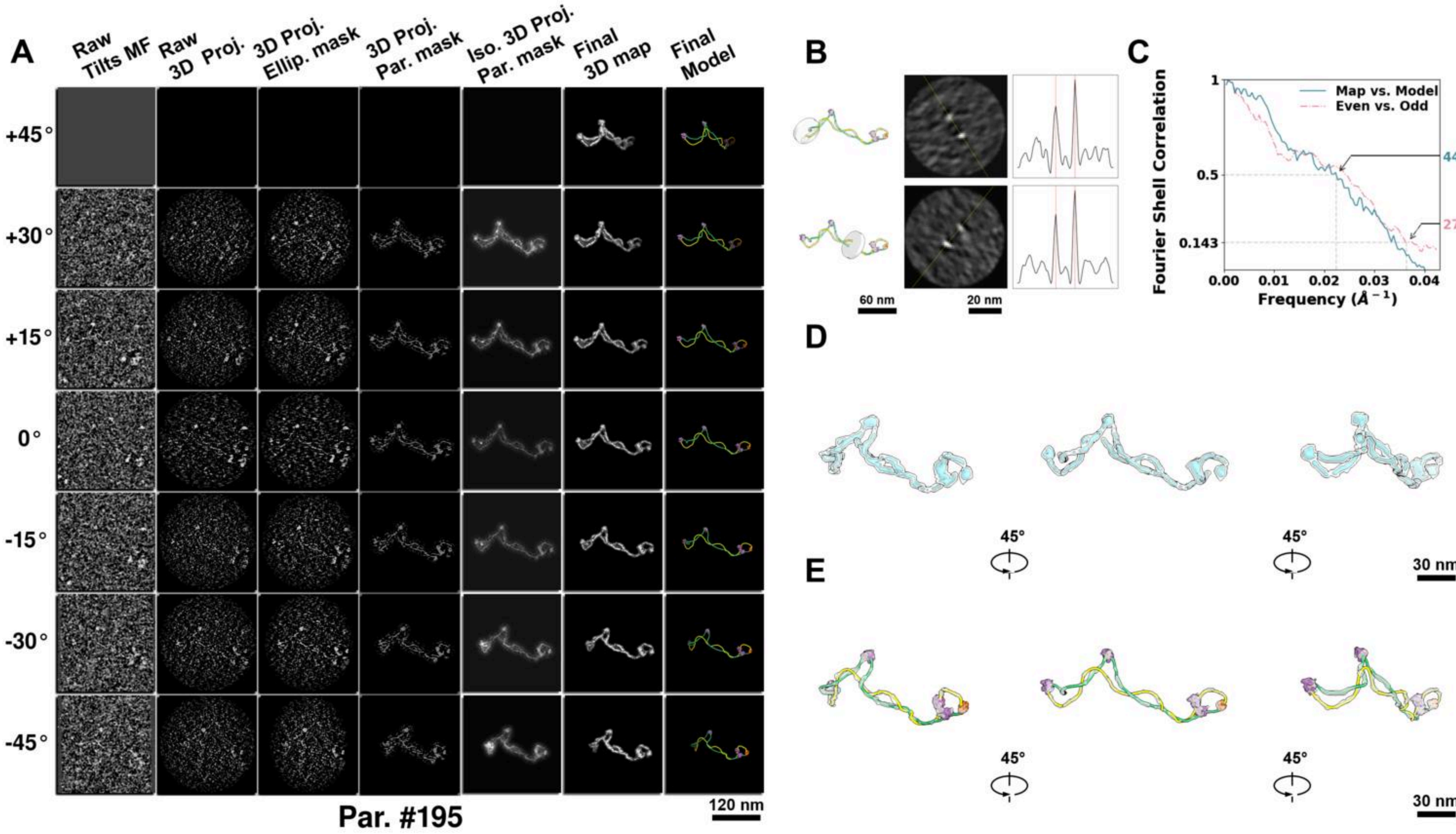

Supplementary Particle Figure 195. Cryo-ET 3D reconstruction of an individual TEC-Cas particle.
(A) 3D reconstruction of the plasmid particle (index no. 195). The first column shows seven representative tilt images from +45° to -45° in step of 15°. The second, third, and fourth columns show 3D projections of the particle with spherical, ellipsoidal (thinner along the z-dimension), and particle-shaped masks, respectively. The fifth column displays the 3D projections of the enhanced and IsoNet missing-wedge-corrected particle. The sixth and seventh columns present the final 3D map and the flexibly fitted model, respectively. (B) Two cross-sectional views (12 nm thickness) of the plasmid density map along its plectoneme axis are shown in the left-middle panel. The intensity profile along the line crossing the two high-density DNA spots is displayed in the right panel. (C) Resolution assessment of the final 3D map using Fourier shell correlation (FSC). Two criteria are shown: FSC between two half-maps reconstructed from even and odd frames (evaluated at 0.143) and FSC between the final 3D map and the fitted model (evaluated at 0.5). (D) Zoomed-in views of the final 3D density map from panel A, displayed at two contour levels. (E) Superimposition of the high-contour level map from panel D onto its fitted model.

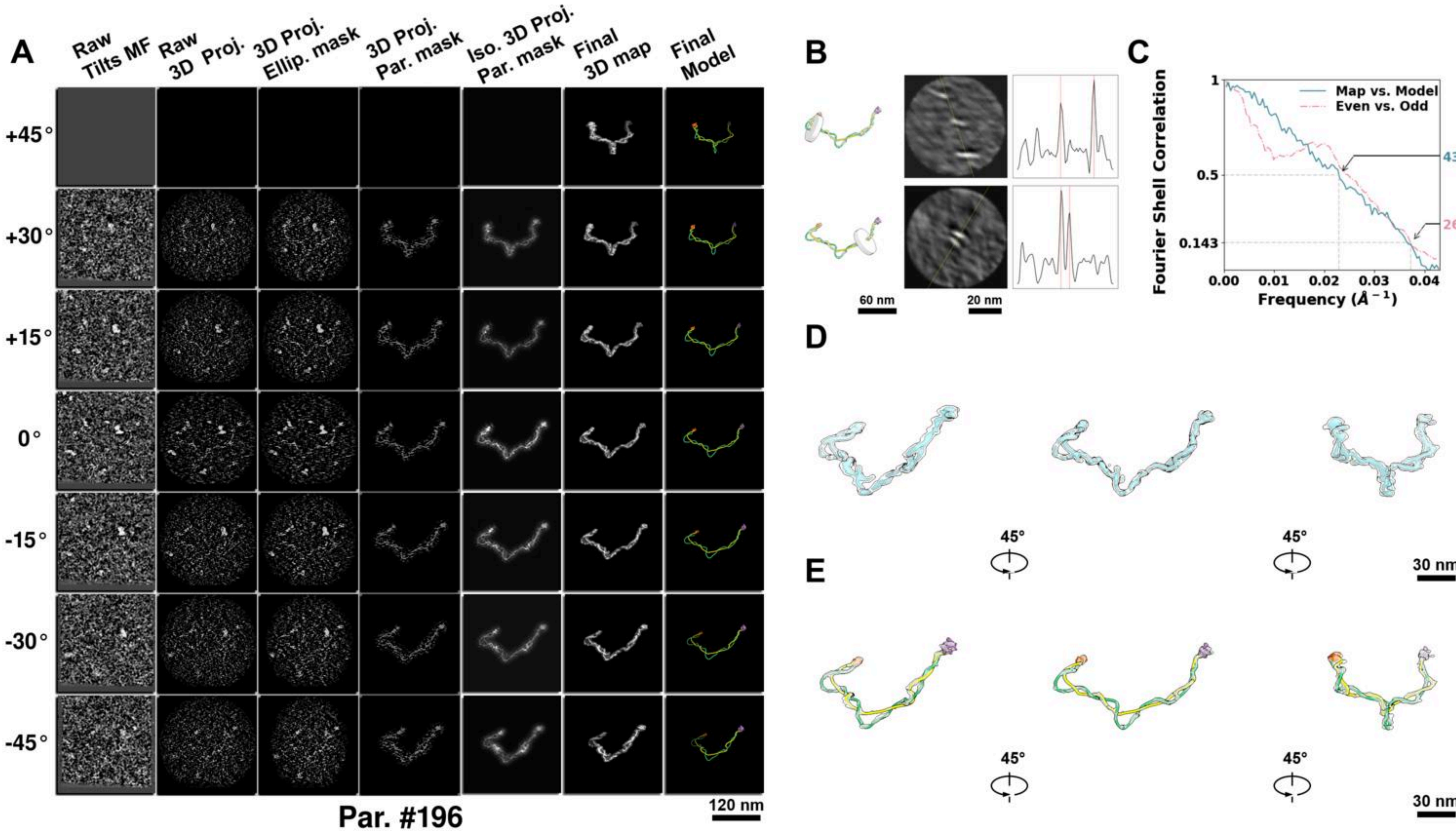

Supplementary Particle Figure 196. Cryo-ET 3D reconstruction of an individual TEC-Cas particle.
(A) 3D reconstruction of the plasmid particle (index no. 196). The first column shows seven representative tilt images from +45° to -45° in step of 15°. The second, third, and fourth columns show 3D projections of the particle with spherical, ellipsoidal (thinner along the z-dimension), and particle-shaped masks, respectively. The fifth column displays the 3D projections of the enhanced and IsoNet missing-wedge-corrected particle. The sixth and seventh columns present the final 3D map and the flexibly fitted model, respectively. (B) Two cross-sectional views (12 nm thickness) of the plasmid density map along its plectoneme axis are shown in the left-middle panel. The intensity profile along the line crossing the two high-density DNA spots is displayed in the right panel. (C) Resolution assessment of the final 3D map using Fourier shell correlation (FSC). Two criteria are shown: FSC between two half-maps reconstructed from even and odd frames (evaluated at 0.143) and FSC between the final 3D map and the fitted model (evaluated at 0.5). (D) Zoomed-in views of the final 3D density map from panel A, displayed at two contour levels. (E) Superimposition of the high-contour level map from panel D onto its fitted model.

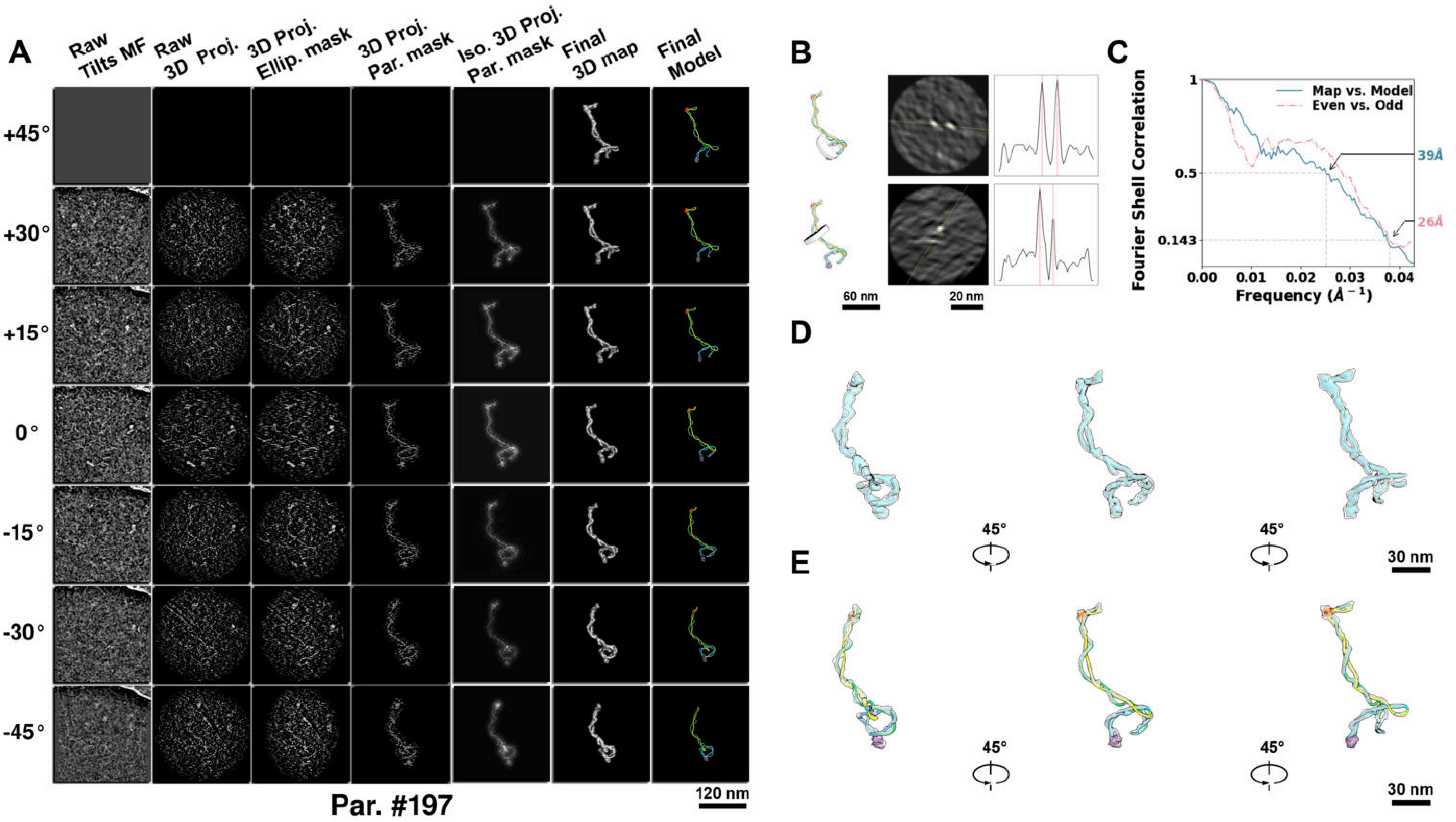

Supplementary Particle Figure 197. Cryo-ET 3D reconstruction of an individual TEC-Cas particle.
(A) 3D reconstruction of the plasmid particle (index no. 197). The first column shows seven representative tilt images from +45° to -45° in step of 15°. The second, third, and fourth columns show 3D projections of the particle with spherical, ellipsoidal (thinner along the z-dimension), and particle-shaped masks, respectively. The fifth column displays the 3D projections of the enhanced and IsoNet missing-wedge-corrected particle. The sixth and seventh columns present the final 3D map and the flexibly fitted model, respectively. (B) Two cross-sectional views (12 nm thickness) of the plasmid density map along its plectoneme axis are shown in the left-middle panel. The intensity profile along the line crossing the two high-density DNA spots is displayed in the right panel. (C) Resolution assessment of the final 3D map using Fourier shell correlation (FSC). Two criteria are shown: FSC between two half-maps reconstructed from even and odd frames (evaluated at 0.143) and FSC between the final 3D map and the fitted model (evaluated at 0.5). (D) Zoomed-in views of the final 3D density map from panel A, displayed at two contour levels. (E) Superimposition of the high-contour level map from panel D onto its fitted model.

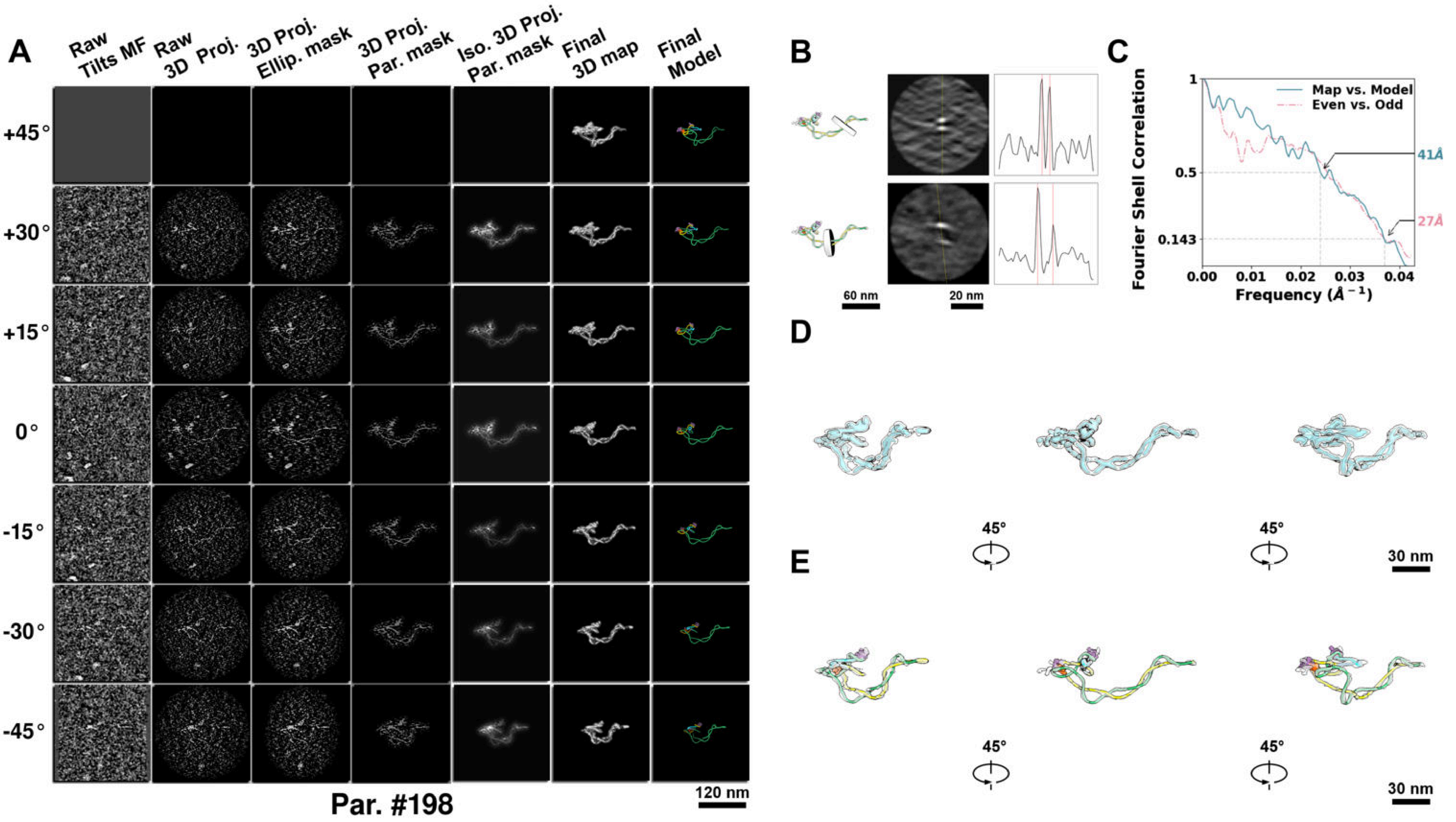

Supplementary Particle Figure 198. Cryo-ET 3D reconstruction of an individual TEC-Cas particle.
(A) 3D reconstruction of the plasmid particle (index no. 198). The first column shows seven representative tilt images from +45° to -45° in step of 15°. The second, third, and fourth columns show 3D projections of the particle with spherical, ellipsoidal (thinner along the z-dimension), and particle-shaped masks, respectively. The fifth column displays the 3D projections of the enhanced and IsoNet missing-wedge-corrected particle. The sixth and seventh columns present the final 3D map and the flexibly fitted model, respectively. (B) Two cross-sectional views (12 nm thickness) of the plasmid density map along its plectoneme axis are shown in the left-middle panel. The intensity profile along the line crossing the two high-density DNA spots is displayed in the right panel. (C) Resolution assessment of the final 3D map using Fourier shell correlation (FSC). Two criteria are shown: FSC between two half-maps reconstructed from even and odd frames (evaluated at 0.143) and FSC between the final 3D map and the fitted model (evaluated at 0.5). (D) Zoomed-in views of the final 3D density map from panel A, displayed at two contour levels. (E) Superimposition of the high-contour level map from panel D onto its fitted model.

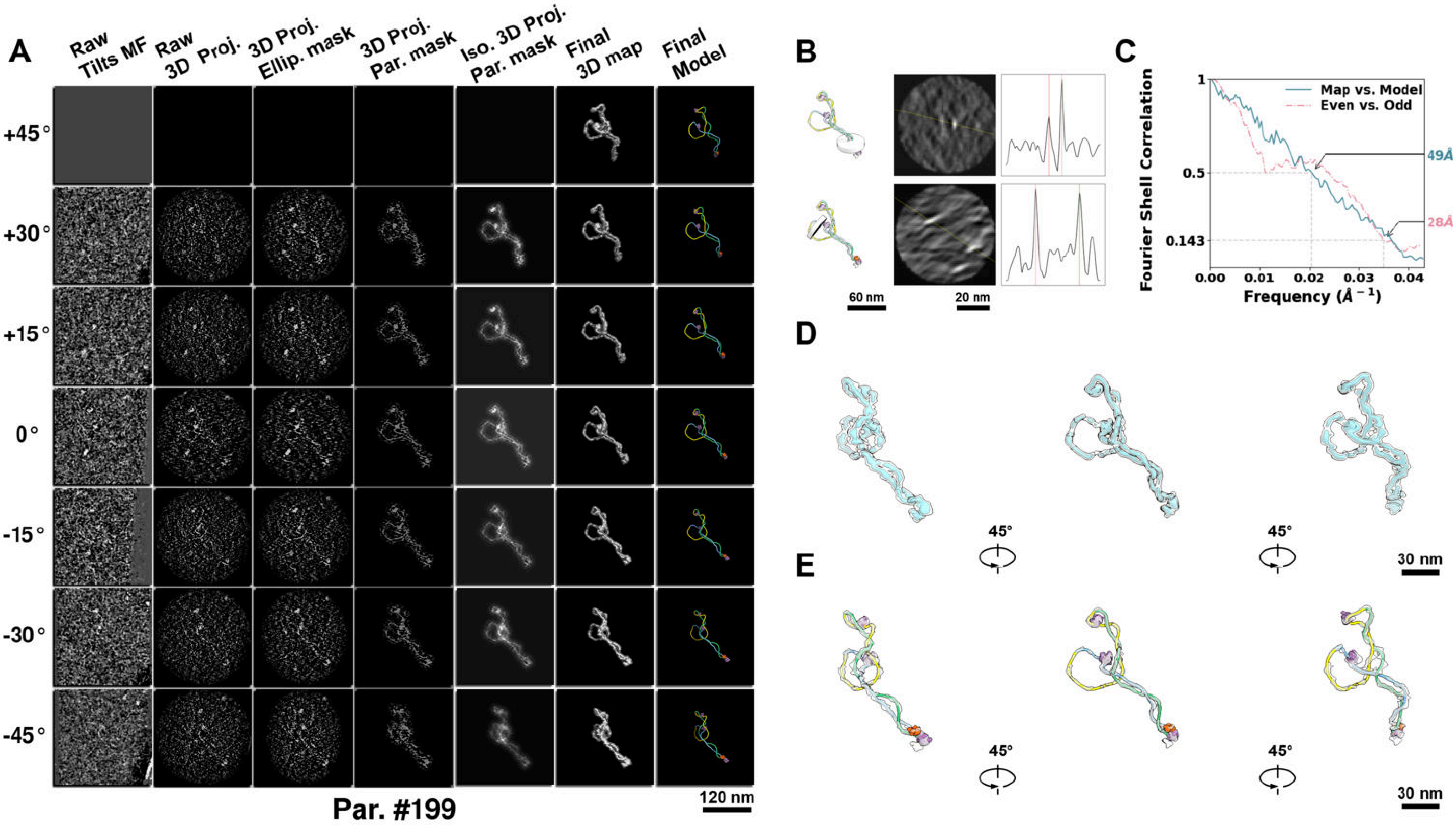

Supplementary Particle Figure 199. Cryo-ET 3D reconstruction of an individual TEC-Cas particle.
(A) 3D reconstruction of the plasmid particle (index no. 199). The first column shows seven representative tilt images from +45° to -45° in step of 15°. The second, third, and fourth columns show 3D projections of the particle with spherical, ellipsoidal (thinner along the z-dimension), and particle-shaped masks, respectively. The fifth column displays the 3D projections of the enhanced and IsoNet missing-wedge-corrected particle. The sixth and seventh columns present the final 3D map and the flexibly fitted model, respectively. (B) Two cross-sectional views (12 nm thickness) of the plasmid density map along its plectoneme axis are shown in the left-middle panel. The intensity profile along the line crossing the two high-density DNA spots is displayed in the right panel. (C) Resolution assessment of the final 3D map using Fourier shell correlation (FSC). Two criteria are shown: FSC between two half-maps reconstructed from even and odd frames (evaluated at 0.143) and FSC between the final 3D map and the fitted model (evaluated at 0.5). (D) Zoomed-in views of the final 3D density map from panel A, displayed at two contour levels. (E) Superimposition of the high-contour level map from panel D onto its fitted model.

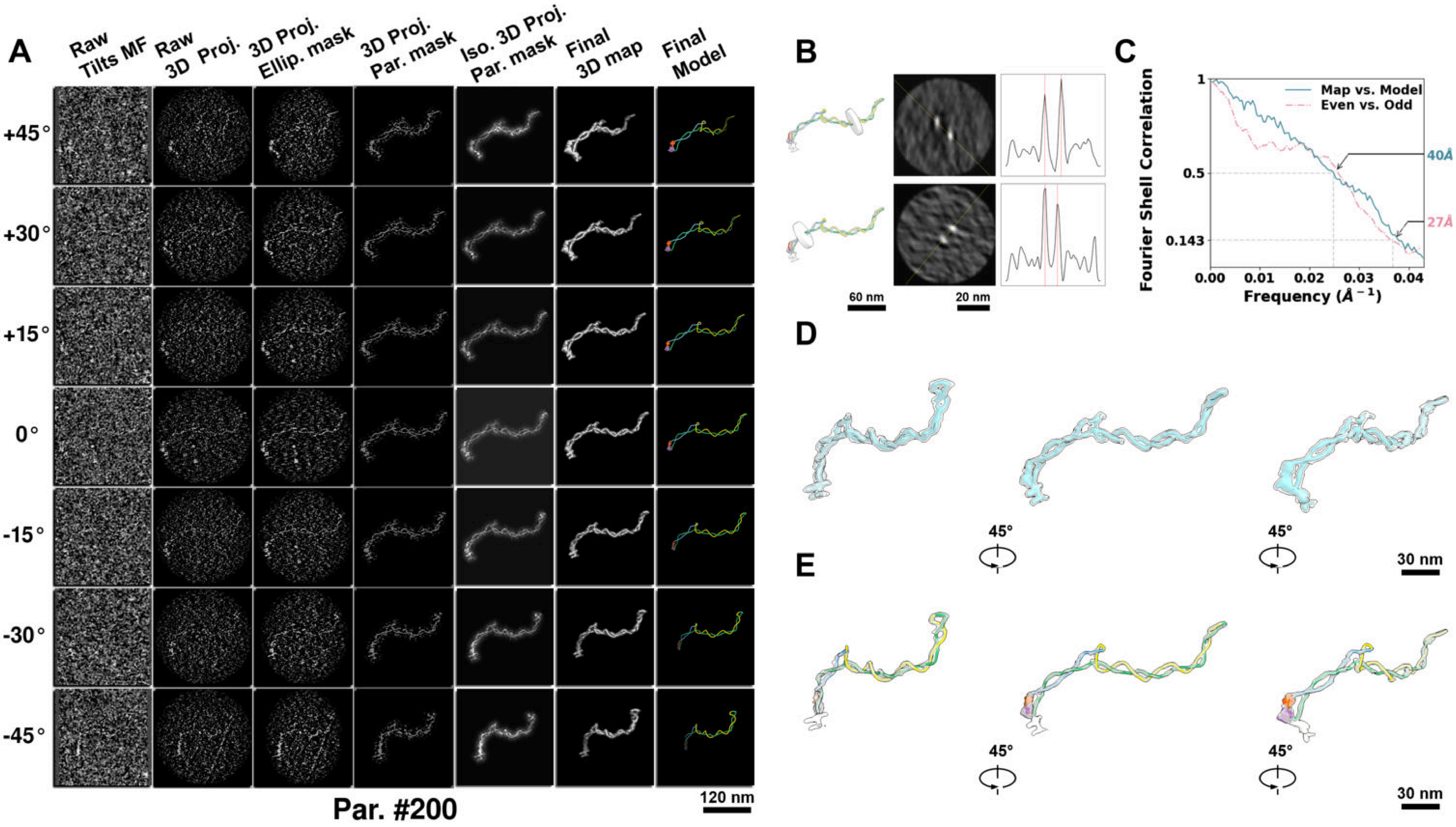

Supplementary Particle Figure 200. Cryo-ET 3D reconstruction of an individual TEC-Cas particle.
(A) 3D reconstruction of the plasmid particle (index no. 200). The first column shows seven representative tilt images from +45° to -45° in step of 15°. The second, third, and fourth columns show 3D projections of the particle with spherical, ellipsoidal (thinner along the z-dimension), and particle-shaped masks, respectively. The fifth column displays the 3D projections of the enhanced and IsoNet missing-wedge-corrected particle. The sixth and seventh columns present the final 3D map and the flexibly fitted model, respectively. (B) Two cross-sectional views (12 nm thickness) of the plasmid density map along its plectoneme axis are shown in the left-middle panel. The intensity profile along the line crossing the two high-density DNA spots is displayed in the right panel. (C) Resolution assessment of the final 3D map using Fourier shell correlation (FSC). Two criteria are shown: FSC between two half-maps reconstructed from even and odd frames (evaluated at 0.143) and FSC between the final 3D map and the fitted model (evaluated at 0.5). (D) Zoomed-in views of the final 3D density map from panel A, displayed at two contour levels. (E) Superimposition of the high-contour level map from panel D onto its fitted model.

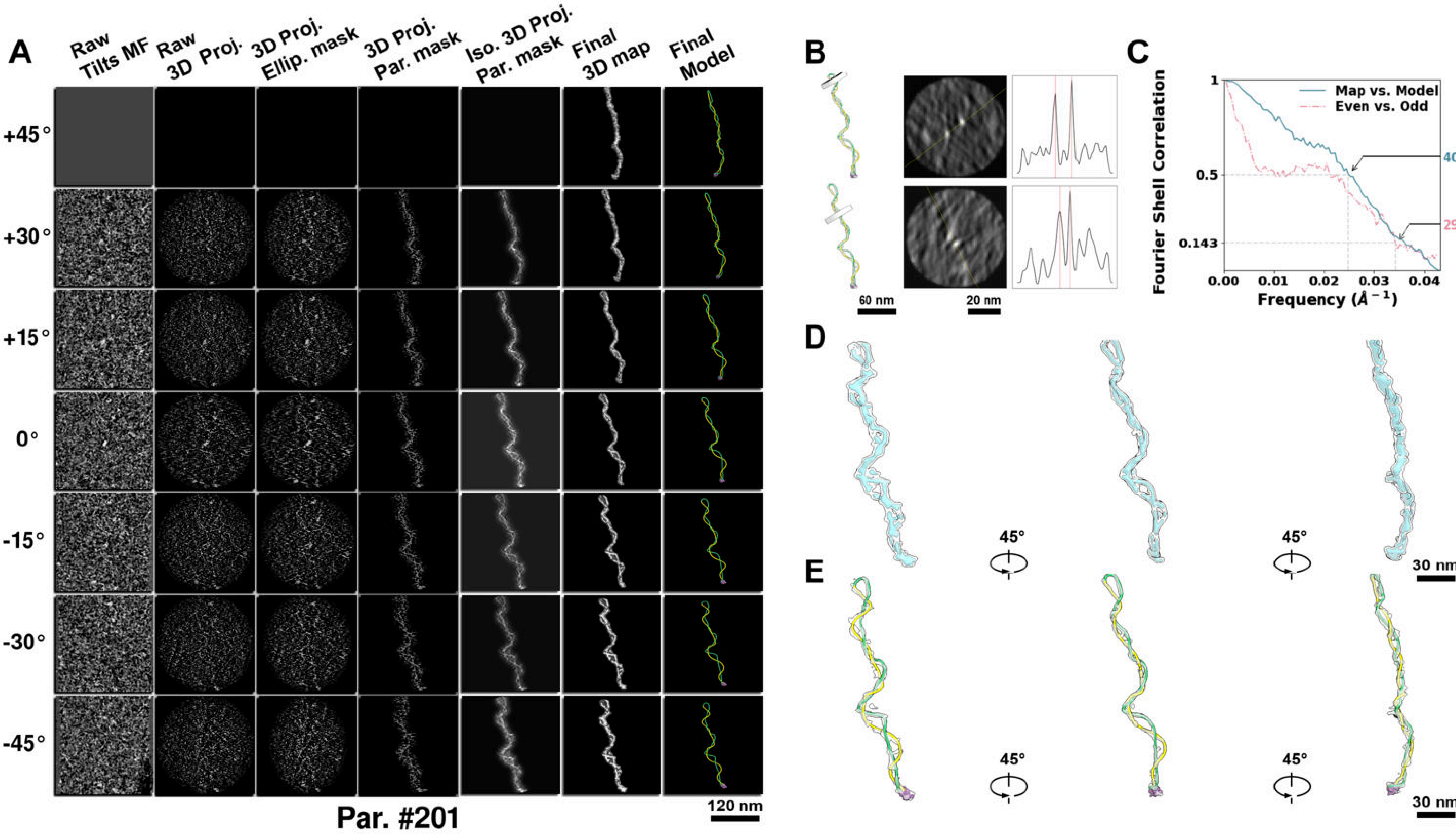

Supplementary Particle Figure 201. Cryo-ET 3D reconstruction of an individual TEC-Cas particle.
(A) 3D reconstruction of the plasmid particle (index no. 201). The first column shows seven representative tilt images from +45° to -45° in step of 15°. The second, third, and fourth columns show 3D projections of the particle with spherical, ellipsoidal (thinner along the z-dimension), and particle-shaped masks, respectively. The fifth column displays the 3D projections of the enhanced and IsoNet missing-wedge-corrected particle. The sixth and seventh columns present the final 3D map and the flexibly fitted model, respectively. (B) Two cross-sectional views (12 nm thickness) of the plasmid density map along its plectoneme axis are shown in the left-middle panel. The intensity profile along the line crossing the two high-density DNA spots is displayed in the right panel. (C) Resolution assessment of the final 3D map using Fourier shell correlation (FSC). Two criteria are shown: FSC between two half-maps reconstructed from even and odd frames (evaluated at 0.143) and FSC between the final 3D map and the fitted model (evaluated at 0.5). (D) Zoomed-in views of the final 3D density map from panel A, displayed at two contour levels. (E) Superimposition of the high-contour level map from panel D onto its fitted model.

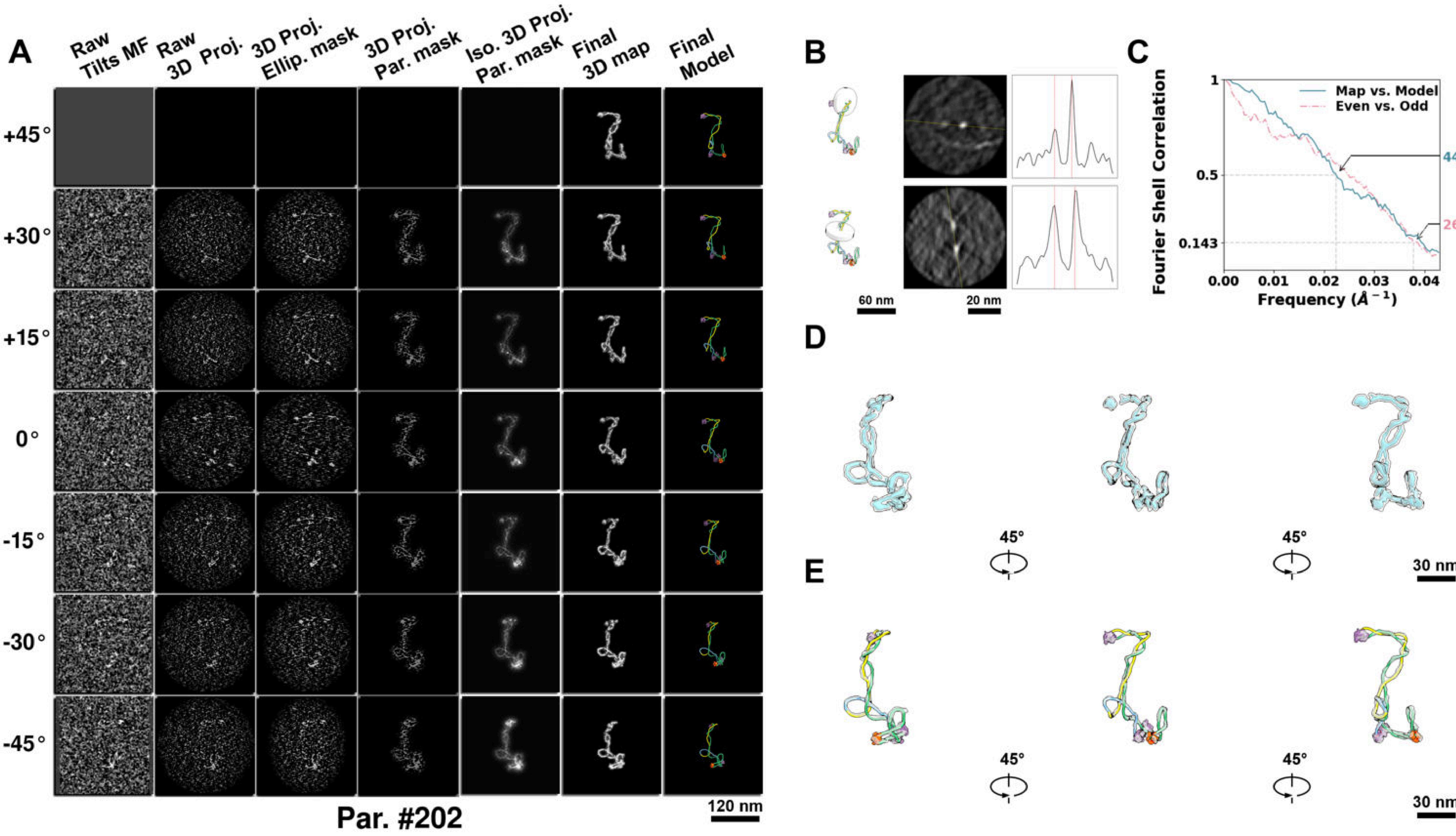

Supplementary Particle Figure 202. Cryo-ET 3D reconstruction of an individual TEC-Cas particle.
(A) 3D reconstruction of the plasmid particle (index no. 202). The first column shows seven representative tilt images from +45° to -45° in step of 15°. The second, third, and fourth columns show 3D projections of the particle with spherical, ellipsoidal (thinner along the z-dimension), and particle-shaped masks, respectively. The fifth column displays the 3D projections of the enhanced and IsoNet missing-wedge-corrected particle. The sixth and seventh columns present the final 3D map and the flexibly fitted model, respectively. (B) Two cross-sectional views (12 nm thickness) of the plasmid density map along its plectoneme axis are shown in the left-middle panel. The intensity profile along the line crossing the two high-density DNA spots is displayed in the right panel. (C) Resolution assessment of the final 3D map using Fourier shell correlation (FSC). Two criteria are shown: FSC between two half-maps reconstructed from even and odd frames (evaluated at 0.143) and FSC between the final 3D map and the fitted model (evaluated at 0.5). (D) Zoomed-in views of the final 3D density map from panel A, displayed at two contour levels. (E) Superimposition of the high-contour level map from panel D onto its fitted model.

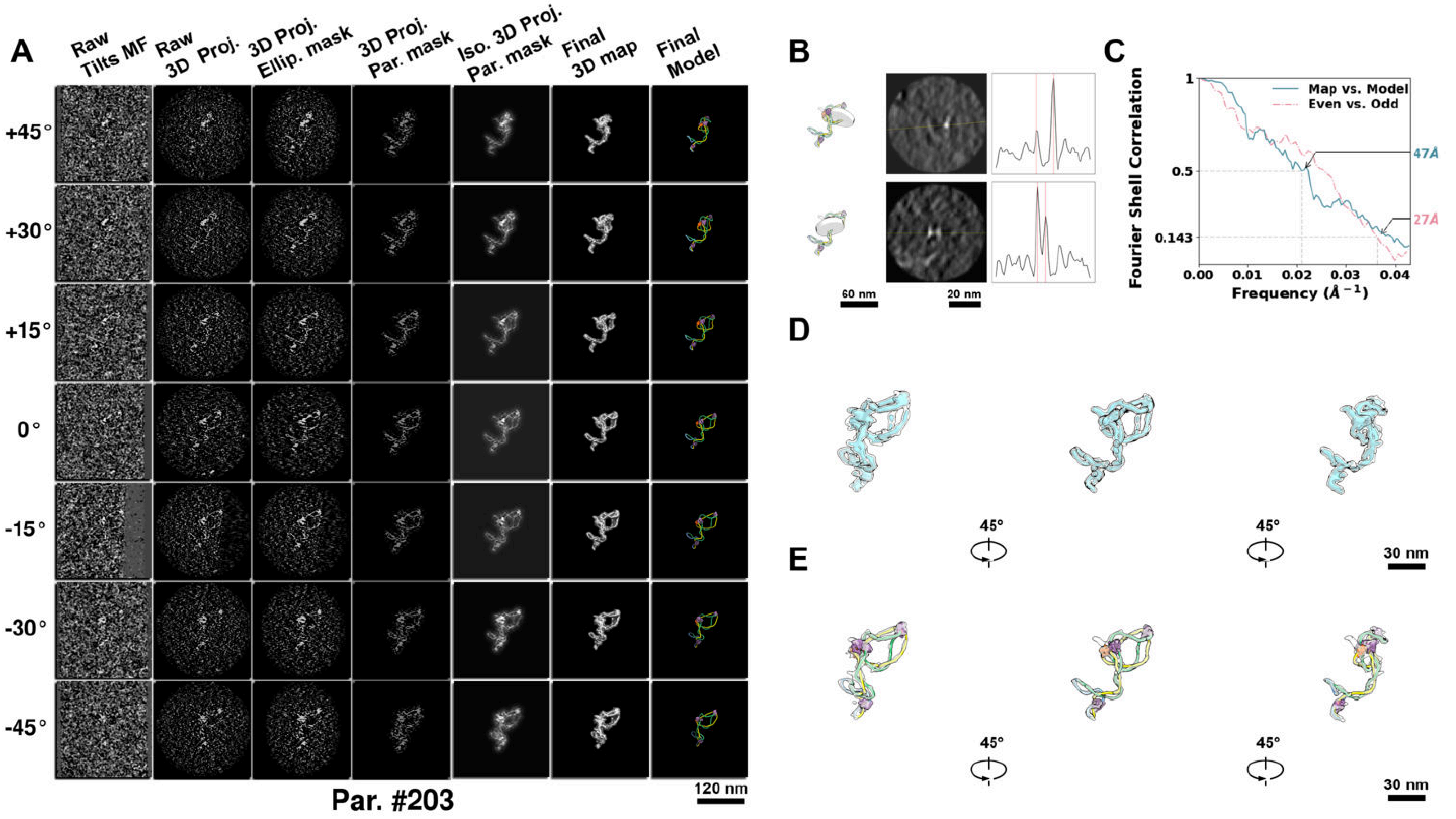

Supplementary Particle Figure 203. Cryo-ET 3D reconstruction of an individual TEC-Cas particle.
(A) 3D reconstruction of the plasmid particle (index no. 203). The first column shows seven representative tilt images from +45° to -45° in step of 15°. The second, third, and fourth columns show 3D projections of the particle with spherical, ellipsoidal (thinner along the z-dimension), and particle-shaped masks, respectively. The fifth column displays the 3D projections of the enhanced and IsoNet missing-wedge-corrected particle. The sixth and seventh columns present the final 3D map and the flexibly fitted model, respectively. (B) Two cross-sectional views (12 nm thickness) of the plasmid density map along its plectoneme axis are shown in the left-middle panel. The intensity profile along the line crossing the two high-density DNA spots is displayed in the right panel. (C) Resolution assessment of the final 3D map using Fourier shell correlation (FSC). Two criteria are shown: FSC between two half-maps reconstructed from even and odd frames (evaluated at 0.143) and FSC between the final 3D map and the fitted model (evaluated at 0.5). (D) Zoomed-in views of the final 3D density map from panel A, displayed at two contour levels. (E) Superimposition of the high-contour level map from panel D onto its fitted model.

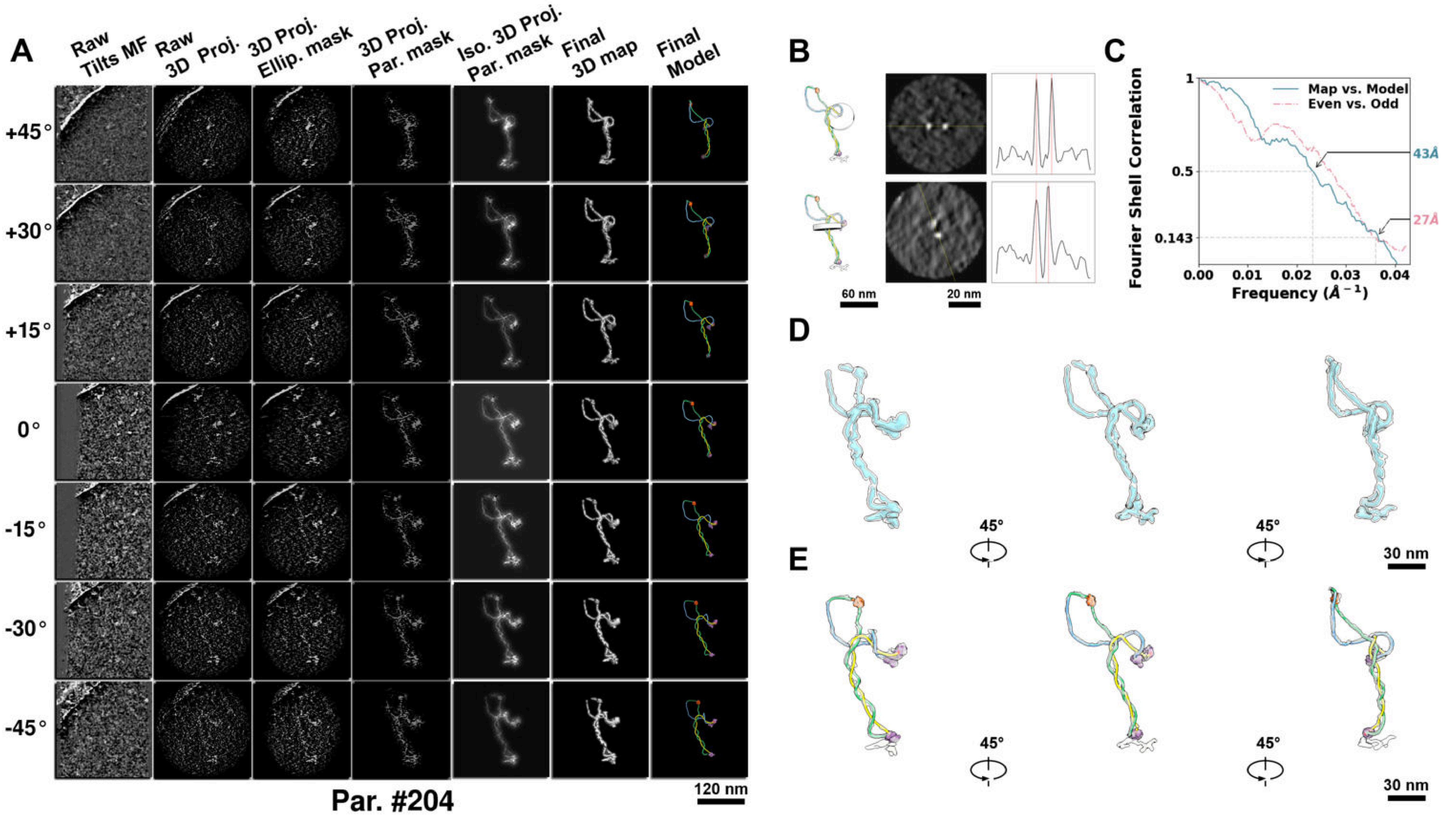

Supplementary Particle Figure 204. Cryo-ET 3D reconstruction of an individual TEC-Cas particle.
(A) 3D reconstruction of the plasmid particle (index no. 204). The first column shows seven representative tilt images from +45° to -45° in step of 15°. The second, third, and fourth columns show 3D projections of the particle with spherical, ellipsoidal (thinner along the z-dimension), and particle-shaped masks, respectively. The fifth column displays the 3D projections of the enhanced and IsoNet missing-wedge-corrected particle. The sixth and seventh columns present the final 3D map and the flexibly fitted model, respectively. (B) Two cross-sectional views (12 nm thickness) of the plasmid density map along its plectoneme axis are shown in the left-middle panel. The intensity profile along the line crossing the two high-density DNA spots is displayed in the right panel. (C) Resolution assessment of the final 3D map using Fourier shell correlation (FSC). Two criteria are shown: FSC between two half-maps reconstructed from even and odd frames (evaluated at 0.143) and FSC between the final 3D map and the fitted model (evaluated at 0.5). (D) Zoomed-in views of the final 3D density map from panel A, displayed at two contour levels. (E) Superimposition of the high-contour level map from panel D onto its fitted model.

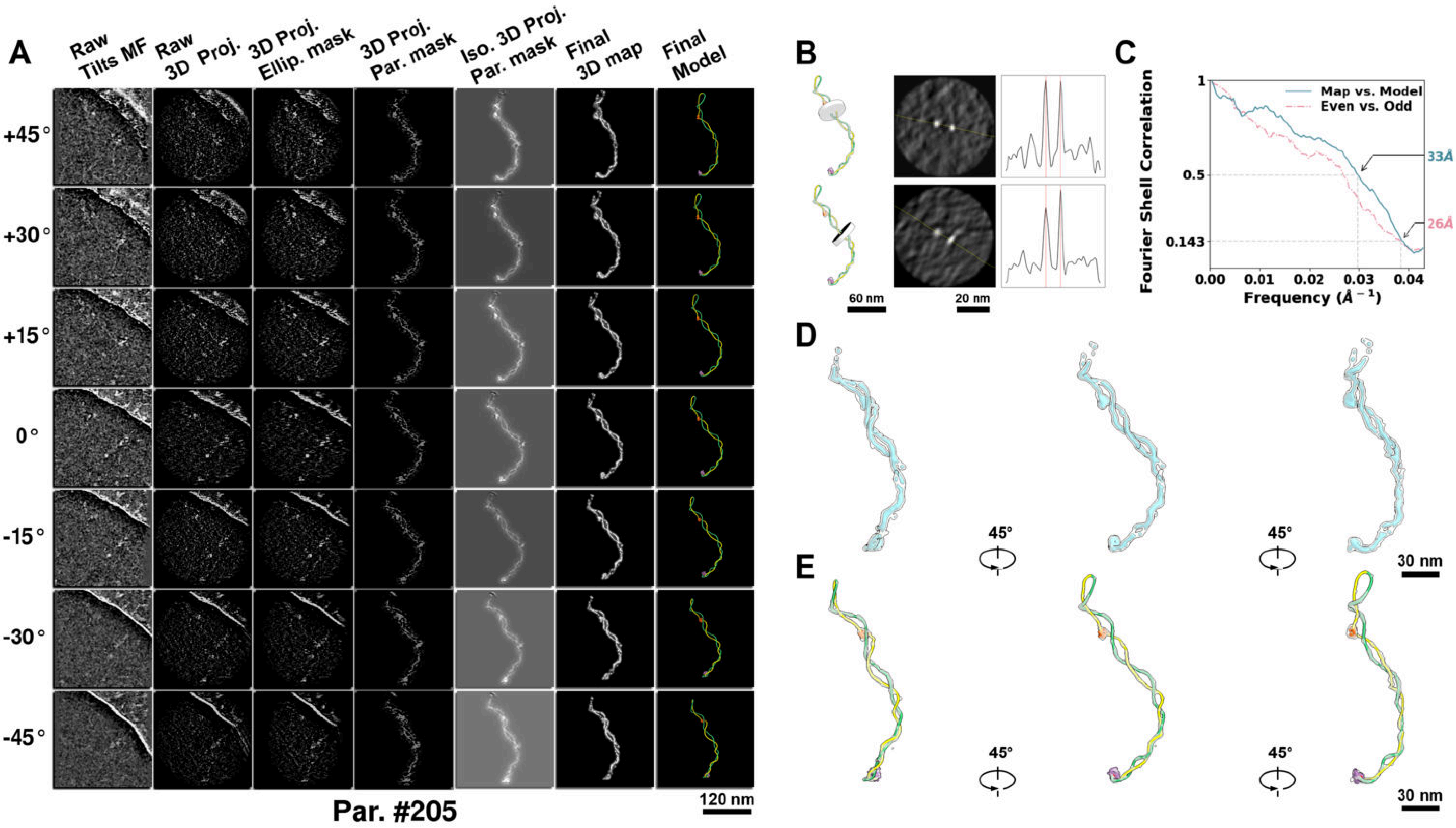

Supplementary Particle Figure 205. Cryo-ET 3D reconstruction of an individual TEC-Cas particle.
(A) 3D reconstruction of the plasmid particle (index no. 205). The first column shows seven representative tilt images from +45° to -45° in step of 15°. The second, third, and fourth columns show 3D projections of the particle with spherical, ellipsoidal (thinner along the z-dimension), and particle-shaped masks, respectively. The fifth column displays the 3D projections of the enhanced and IsoNet missing-wedge-corrected particle. The sixth and seventh columns present the final 3D map and the flexibly fitted model, respectively. (B) Two cross-sectional views (12 nm thickness) of the plasmid density map along its plectoneme axis are shown in the left-middle panel. The intensity profile along the line crossing the two high-density DNA spots is displayed in the right panel. (C) Resolution assessment of the final 3D map using Fourier shell correlation (FSC). Two criteria are shown: FSC between two half-maps reconstructed from even and odd frames (evaluated at 0.143) and FSC between the final 3D map and the fitted model (evaluated at 0.5). (D) Zoomed-in views of the final 3D density map from panel A, displayed at two contour levels. (E) Superimposition of the high-contour level map from panel D onto its fitted model.

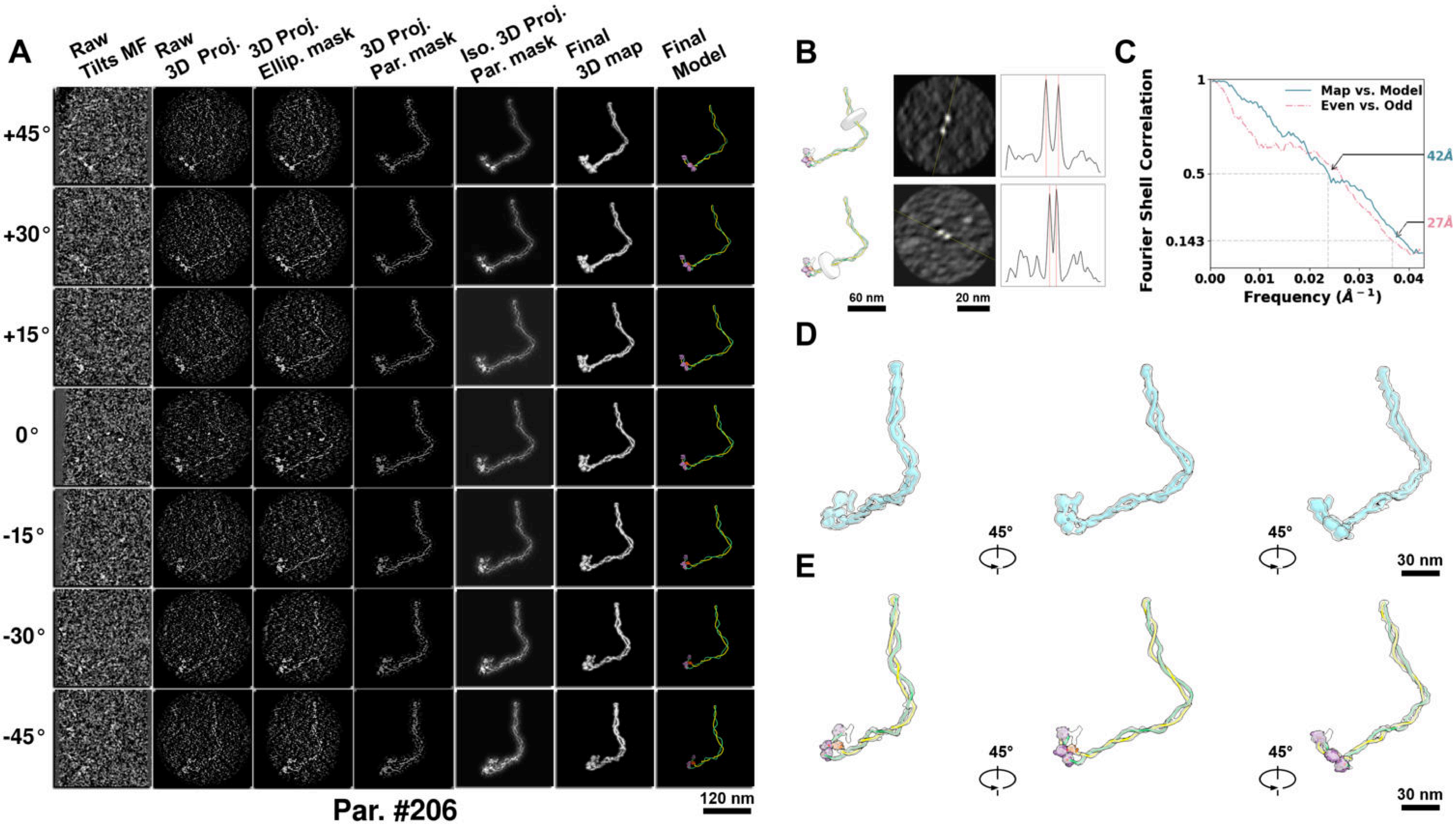

Supplementary Particle Figure 206. Cryo-ET 3D reconstruction of an individual TEC-Cas particle.
(A) 3D reconstruction of the plasmid particle (index no. 206). The first column shows seven representative tilt images from +45° to -45° in step of 15°. The second, third, and fourth columns show 3D projections of the particle with spherical, ellipsoidal (thinner along the z-dimension), and particle-shaped masks, respectively. The fifth column displays the 3D projections of the enhanced and IsoNet missing-wedge-corrected particle. The sixth and seventh columns present the final 3D map and the flexibly fitted model, respectively. (B) Two cross-sectional views (12 nm thickness) of the plasmid density map along its plectoneme axis are shown in the left-middle panel. The intensity profile along the line crossing the two high-density DNA spots is displayed in the right panel. (C) Resolution assessment of the final 3D map using Fourier shell correlation (FSC). Two criteria are shown: FSC between two half-maps reconstructed from even and odd frames (evaluated at 0.143) and FSC between the final 3D map and the fitted model (evaluated at 0.5). (D) Zoomed-in views of the final 3D density map from panel A, displayed at two contour levels. (E) Superimposition of the high-contour level map from panel D onto its fitted model.

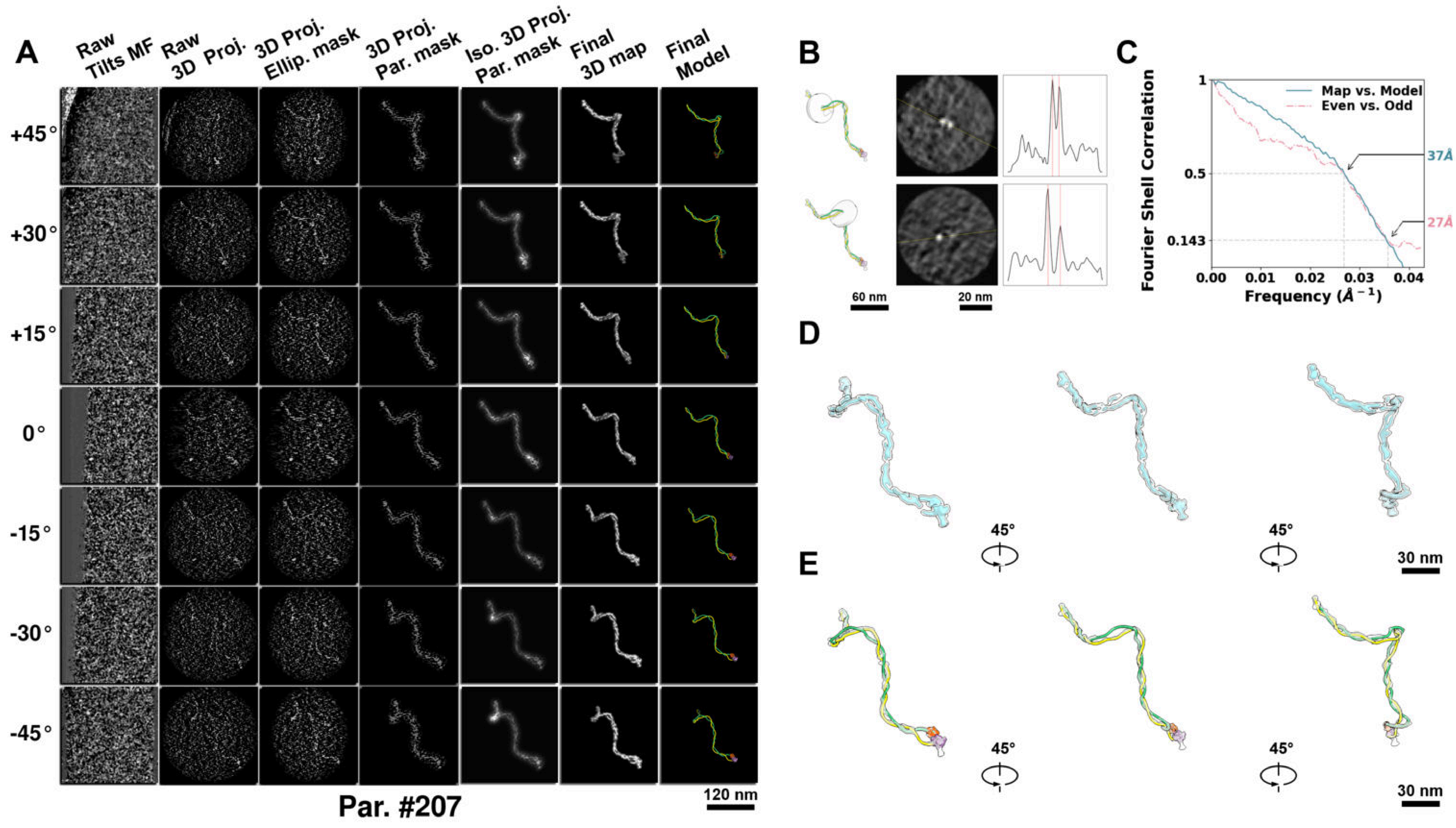

Supplementary Particle Figure 207. Cryo-ET 3D reconstruction of an individual TEC-Cas particle.
(A) 3D reconstruction of the plasmid particle (index no. 207). The first column shows seven representative tilt images from +45° to -45° in step of 15°. The second, third, and fourth columns show 3D projections of the particle with spherical, ellipsoidal (thinner along the z-dimension), and particle-shaped masks, respectively. The fifth column displays the 3D projections of the enhanced and IsoNet missing-wedge-corrected particle. The sixth and seventh columns present the final 3D map and the flexibly fitted model, respectively. (B) Two cross-sectional views (12 nm thickness) of the plasmid density map along its plectoneme axis are shown in the left-middle panel. The intensity profile along the line crossing the two high-density DNA spots is displayed in the right panel. (C) Resolution assessment of the final 3D map using Fourier shell correlation (FSC). Two criteria are shown: FSC between two half-maps reconstructed from even and odd frames (evaluated at 0.143) and FSC between the final 3D map and the fitted model (evaluated at 0.5). (D) Zoomed-in views of the final 3D density map from panel A, displayed at two contour levels. (E) Superimposition of the high-contour level map from panel D onto its fitted model.

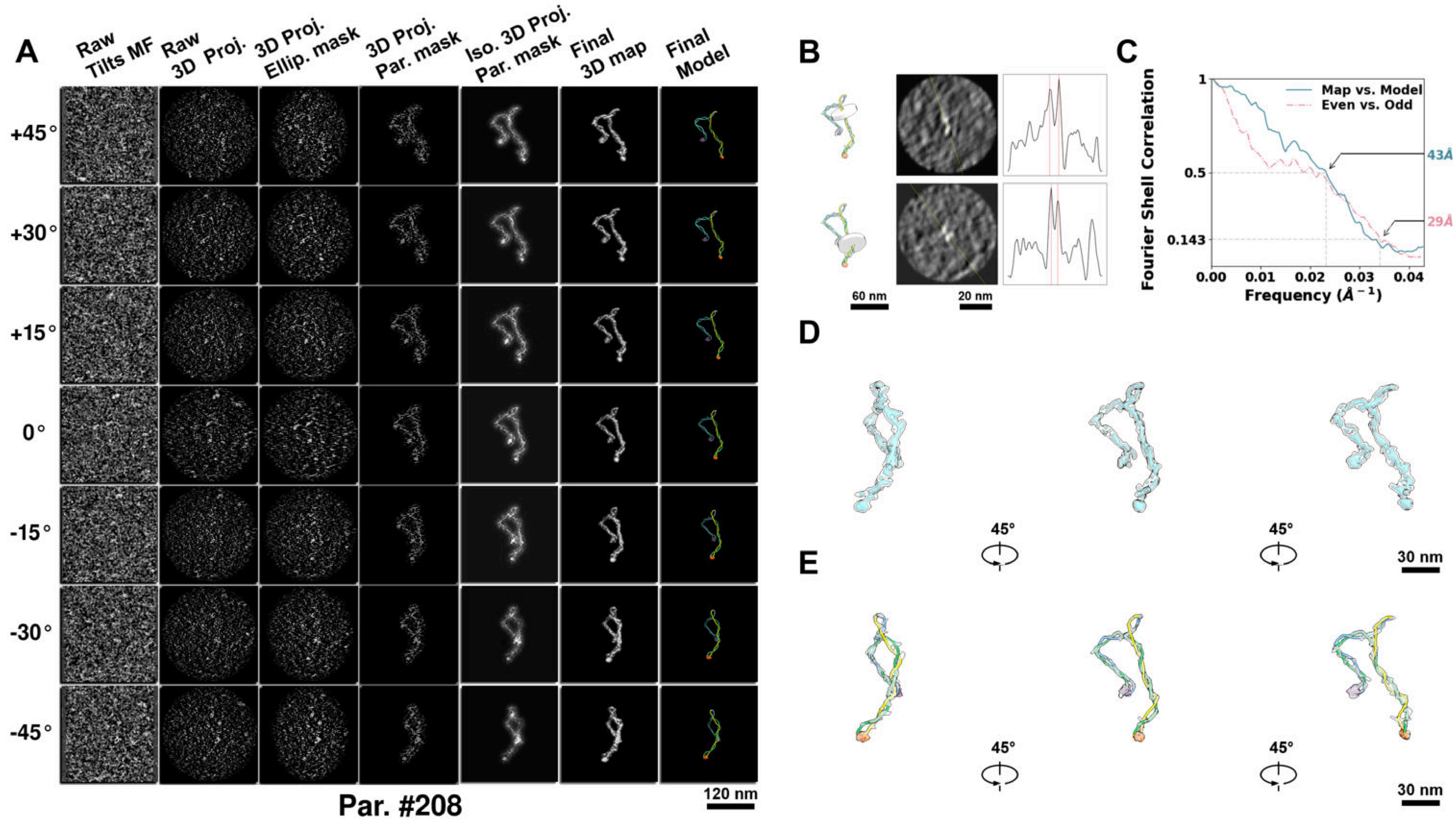

Supplementary Particle Figure 208. Cryo-ET 3D reconstruction of an individual TEC-Cas particle.
(A) 3D reconstruction of the plasmid particle (index no. 208). The first column shows seven representative tilt images from +45° to -45° in step of 15°. The second, third, and fourth columns show 3D projections of the particle with spherical, ellipsoidal (thinner along the z-dimension), and particle-shaped masks, respectively. The fifth column displays the 3D projections of the enhanced and IsoNet missing-wedge-corrected particle. The sixth and seventh columns present the final 3D map and the flexibly fitted model, respectively. (B) Two cross-sectional views (12 nm thickness) of the plasmid density map along its plectoneme axis are shown in the left-middle panel. The intensity profile along the line crossing the two high-density DNA spots is displayed in the right panel. (C) Resolution assessment of the final 3D map using Fourier shell correlation (FSC). Two criteria are shown: FSC between two half-maps reconstructed from even and odd frames (evaluated at 0.143) and FSC between the final 3D map and the fitted model (evaluated at 0.5). (D) Zoomed-in views of the final 3D density map from panel A, displayed at two contour levels. (E) Superimposition of the high-contour level map from panel D onto its fitted model.

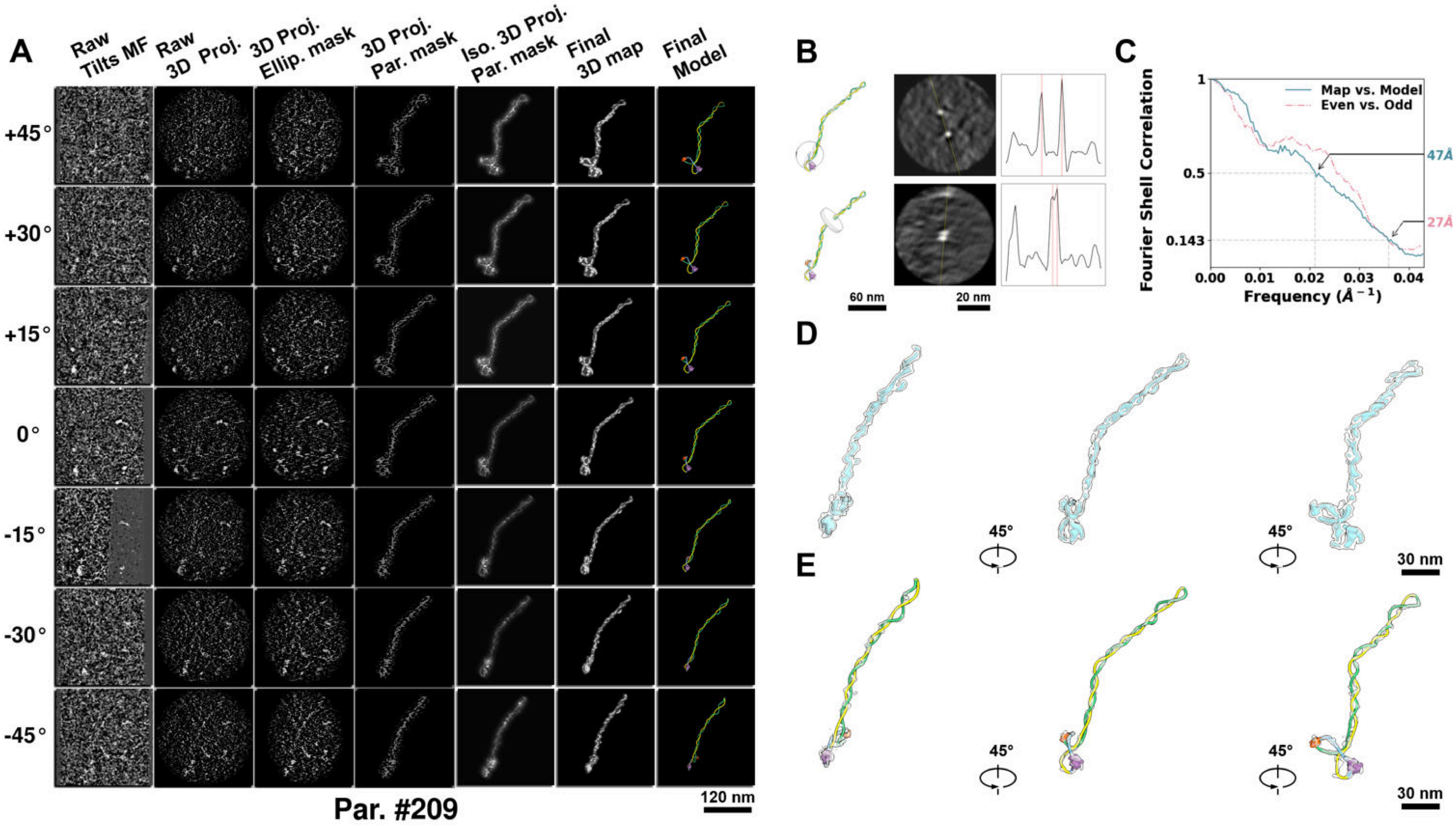

Supplementary Particle Figure 209. Cryo-ET 3D reconstruction of an individual TEC-Cas particle.
(A) 3D reconstruction of the plasmid particle (index no. 209). The first column shows seven representative tilt images from +45° to -45° in step of 15°. The second, third, and fourth columns show 3D projections of the particle with spherical, ellipsoidal (thinner along the z-dimension), and particle-shaped masks, respectively. The fifth column displays the 3D projections of the enhanced and IsoNet missing-wedge-corrected particle. The sixth and seventh columns present the final 3D map and the flexibly fitted model, respectively. (B) Two cross-sectional views (12 nm thickness) of the plasmid density map along its plectoneme axis are shown in the left-middle panel. The intensity profile along the line crossing the two high-density DNA spots is displayed in the right panel. (C) Resolution assessment of the final 3D map using Fourier shell correlation (FSC). Two criteria are shown: FSC between two half-maps reconstructed from even and odd frames (evaluated at 0.143) and FSC between the final 3D map and the fitted model (evaluated at 0.5). (D) Zoomed-in views of the final 3D density map from panel A, displayed at two contour levels. (E) Superimposition of the high-contour level map from panel D onto its fitted model.

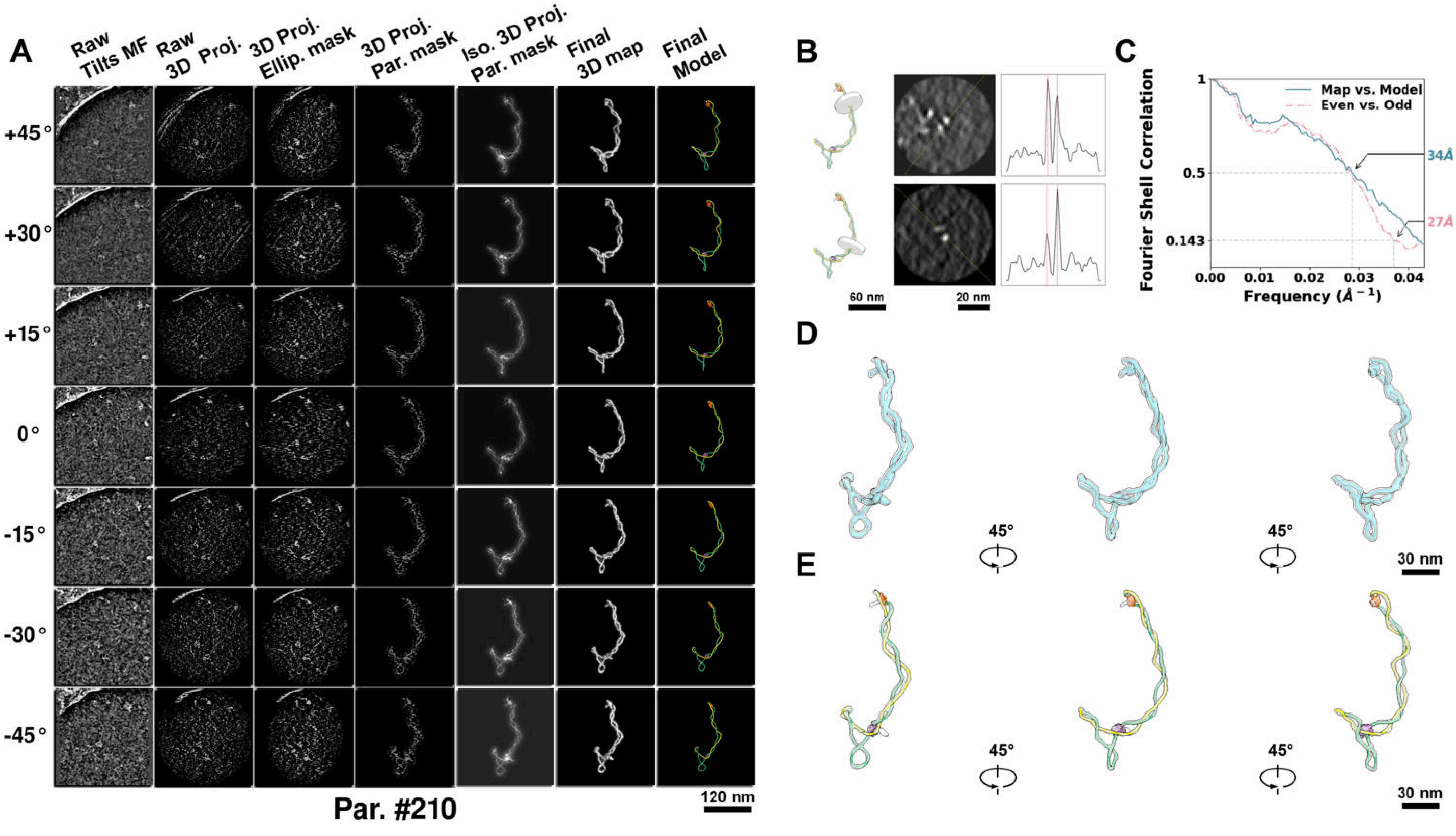

Supplementary Particle Figure 210. Cryo-ET 3D reconstruction of an individual TEC-Cas particle.
(A) 3D reconstruction of the plasmid particle (index no. 210). The first column shows seven representative tilt images from +45° to -45° in step of 15°. The second, third, and fourth columns show 3D projections of the particle with spherical, ellipsoidal (thinner along the z-dimension), and particle-shaped masks, respectively. The fifth column displays the 3D projections of the enhanced and IsoNet missing-wedge-corrected particle. The sixth and seventh columns present the final 3D map and the flexibly fitted model, respectively. (B) Two cross-sectional views (12 nm thickness) of the plasmid density map along its plectoneme axis are shown in the left-middle panel. The intensity profile along the line crossing the two high-density DNA spots is displayed in the right panel. (C) Resolution assessment of the final 3D map using Fourier shell correlation (FSC). Two criteria are shown: FSC between two half-maps reconstructed from even and odd frames (evaluated at 0.143) and FSC between the final 3D map and the fitted model (evaluated at 0.5). (D) Zoomed-in views of the final 3D density map from panel A, displayed at two contour levels. (E) Superimposition of the high-contour level map from panel D onto its fitted model.

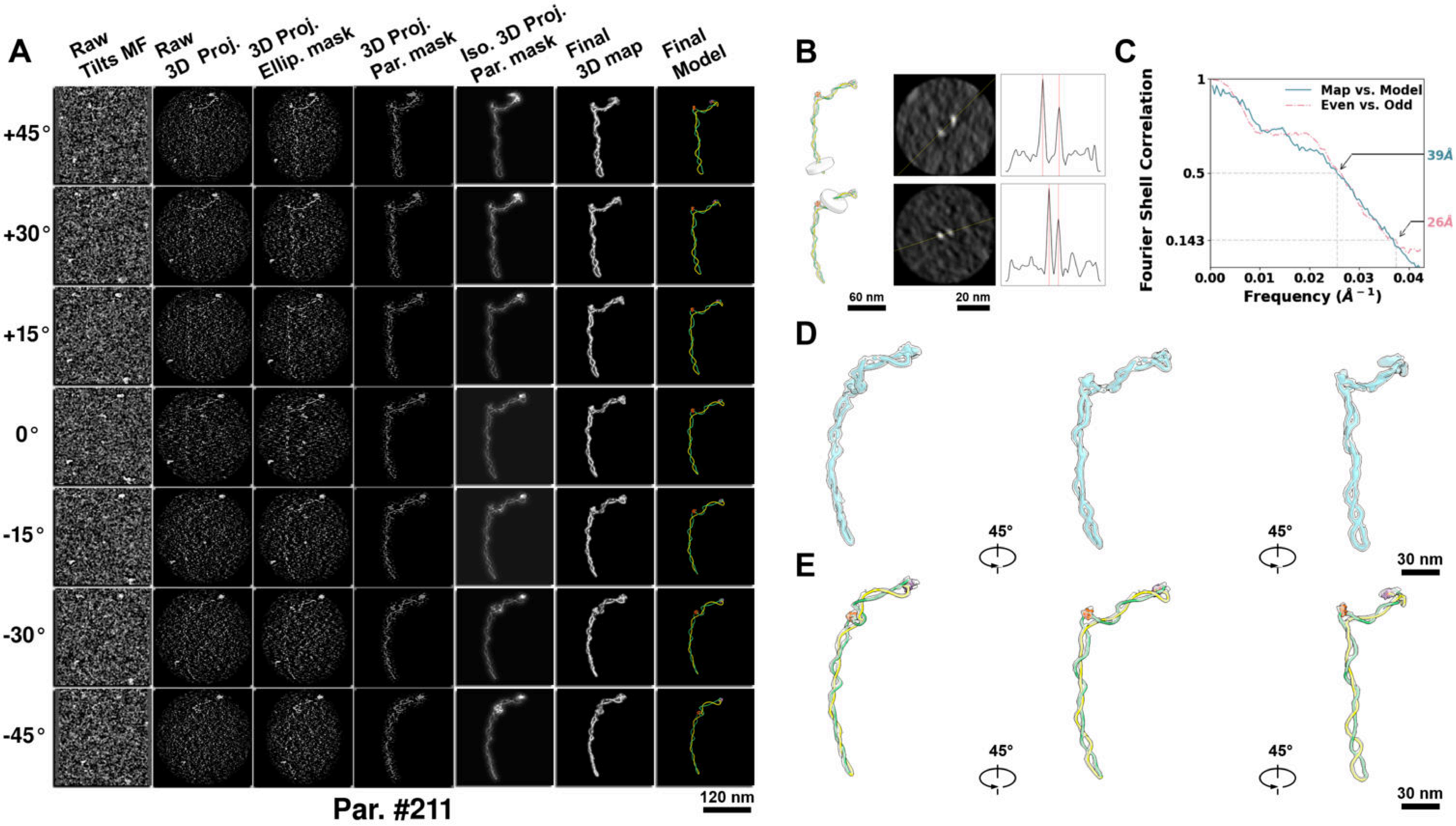

Supplementary Particle Figure 211. Cryo-ET 3D reconstruction of an individual TEC-Cas particle.
(A) 3D reconstruction of the plasmid particle (index no. 211). The first column shows seven representative tilt images from +45° to -45° in step of 15°. The second, third, and fourth columns show 3D projections of the particle with spherical, ellipsoidal (thinner along the z-dimension), and particle-shaped masks, respectively. The fifth column displays the 3D projections of the enhanced and IsoNet missing-wedge-corrected particle. The sixth and seventh columns present the final 3D map and the flexibly fitted model, respectively. (B) Two cross-sectional views (12 nm thickness) of the plasmid density map along its plectoneme axis are shown in the left-middle panel. The intensity profile along the line crossing the two high-density DNA spots is displayed in the right panel. (C) Resolution assessment of the final 3D map using Fourier shell correlation (FSC). Two criteria are shown: FSC between two half-maps reconstructed from even and odd frames (evaluated at 0.143) and FSC between the final 3D map and the fitted model (evaluated at 0.5). (D) Zoomed-in views of the final 3D density map from panel A, displayed at two contour levels. (E) Superimposition of the high-contour level map from panel D onto its fitted model.

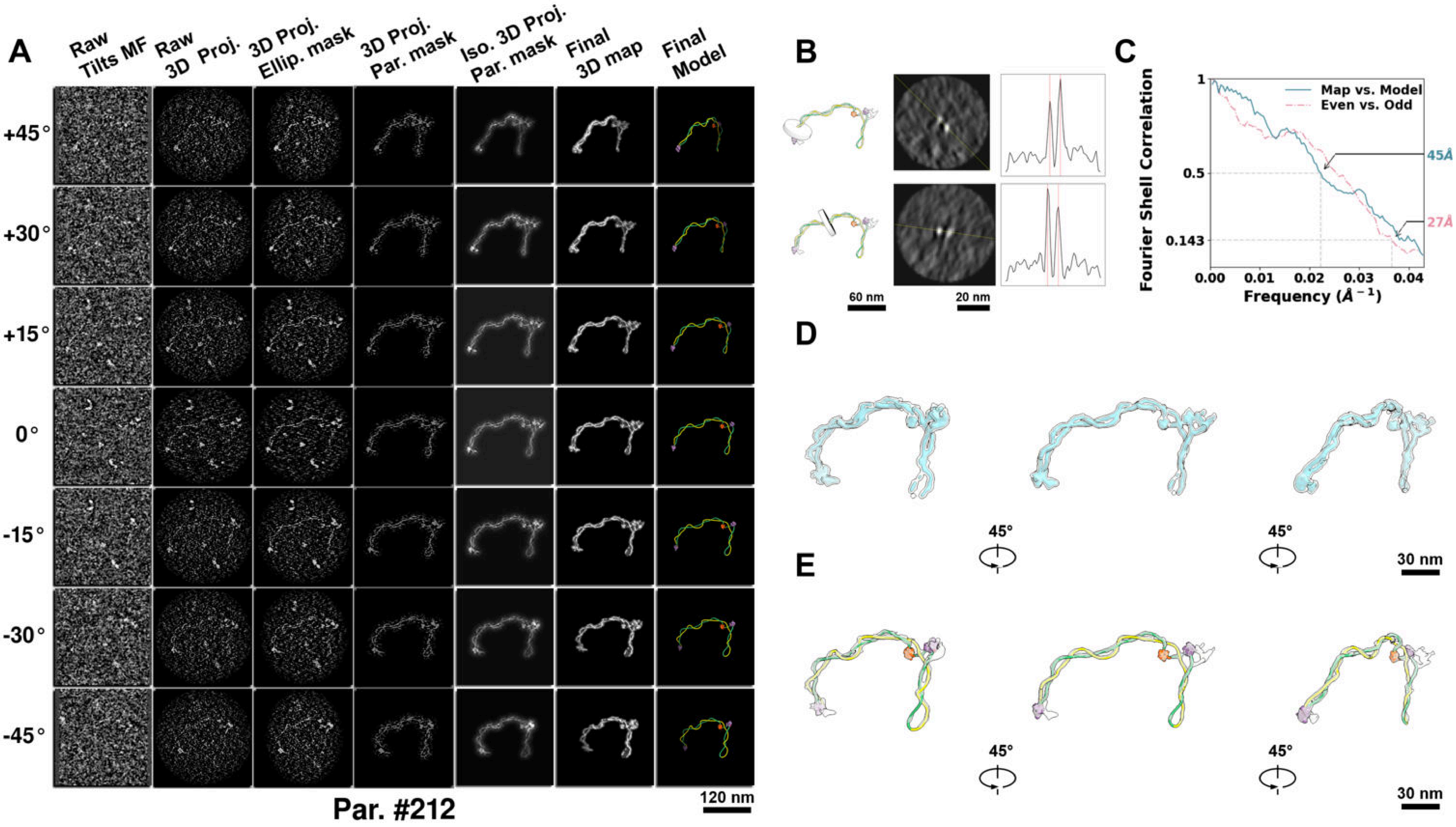

Supplementary Particle Figure 212. Cryo-ET 3D reconstruction of an individual TEC-Cas particle.
(A) 3D reconstruction of the plasmid particle (index no. 212). The first column shows seven representative tilt images from +45° to -45° in step of 15°. The second, third, and fourth columns show 3D projections of the particle with spherical, ellipsoidal (thinner along the z-dimension), and particle-shaped masks, respectively. The fifth column displays the 3D projections of the enhanced and IsoNet missing-wedge-corrected particle. The sixth and seventh columns present the final 3D map and the flexibly fitted model, respectively. (B) Two cross-sectional views (12 nm thickness) of the plasmid density map along its plectoneme axis are shown in the left-middle panel. The intensity profile along the line crossing the two high-density DNA spots is displayed in the right panel. (C) Resolution assessment of the final 3D map using Fourier shell correlation (FSC). Two criteria are shown: FSC between two half-maps reconstructed from even and odd frames (evaluated at 0.143) and FSC between the final 3D map and the fitted model (evaluated at 0.5). (D) Zoomed-in views of the final 3D density map from panel A, displayed at two contour levels. (E) Superimposition of the high-contour level map from panel D onto its fitted model.

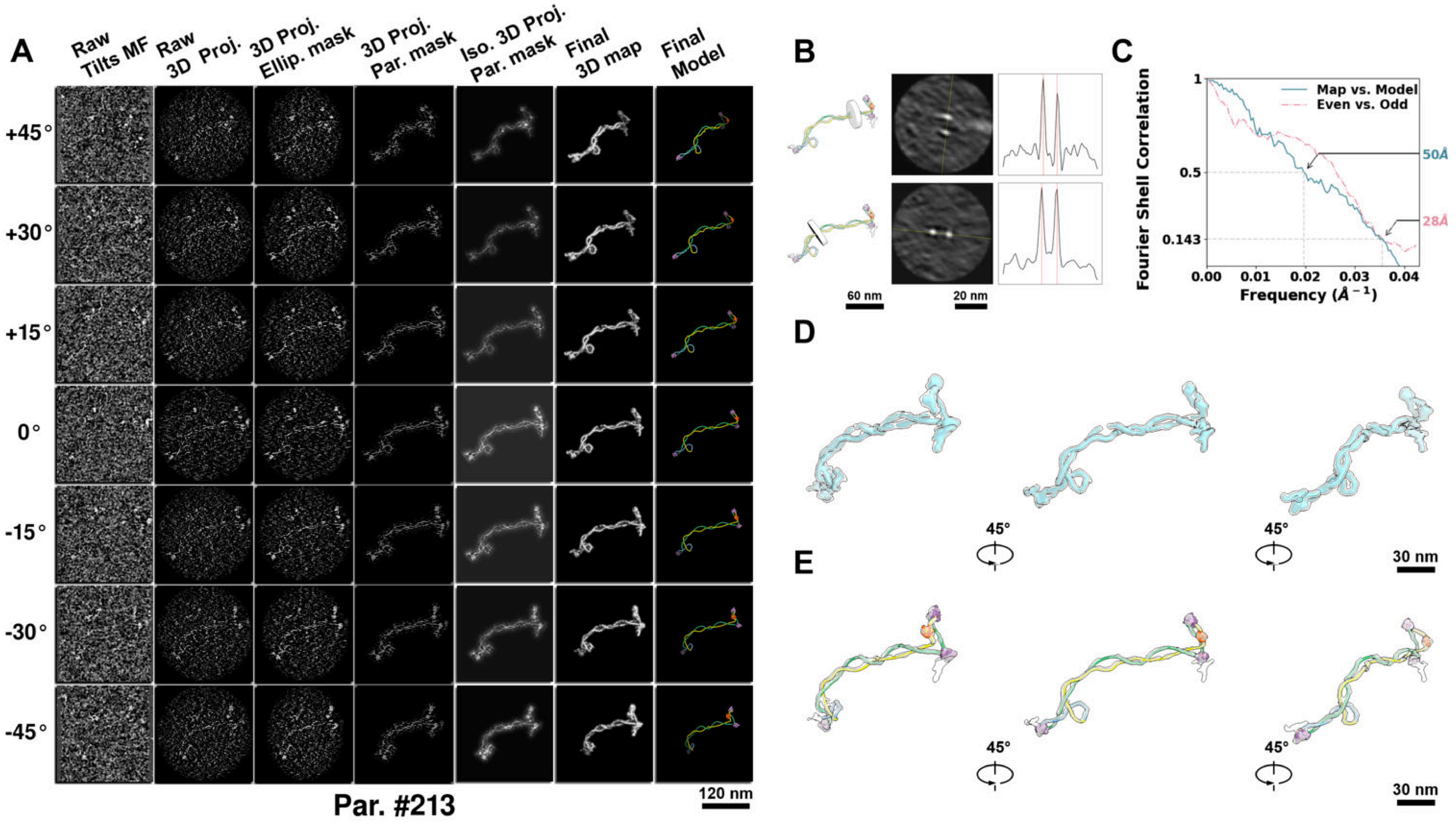

Supplementary Particle Figure 213. Cryo-ET 3D reconstruction of an individual TEC-Cas particle.
(A) 3D reconstruction of the plasmid particle (index no. 213). The first column shows seven representative tilt images from +45° to -45° in step of 15°. The second, third, and fourth columns show 3D projections of the particle with spherical, ellipsoidal (thinner along the z-dimension), and particle-shaped masks, respectively. The fifth column displays the 3D projections of the enhanced and IsoNet missing-wedge-corrected particle. The sixth and seventh columns present the final 3D map and the flexibly fitted model, respectively. (B) Two cross-sectional views (12 nm thickness) of the plasmid density map along its plectoneme axis are shown in the left-middle panel. The intensity profile along the line crossing the two high-density DNA spots is displayed in the right panel. (C) Resolution assessment of the final 3D map using Fourier shell correlation (FSC). Two criteria are shown: FSC between two half-maps reconstructed from even and odd frames (evaluated at 0.143) and FSC between the final 3D map and the fitted model (evaluated at 0.5). (D) Zoomed-in views of the final 3D density map from panel A, displayed at two contour levels. (E) Superimposition of the high-contour level map from panel D onto its fitted model.

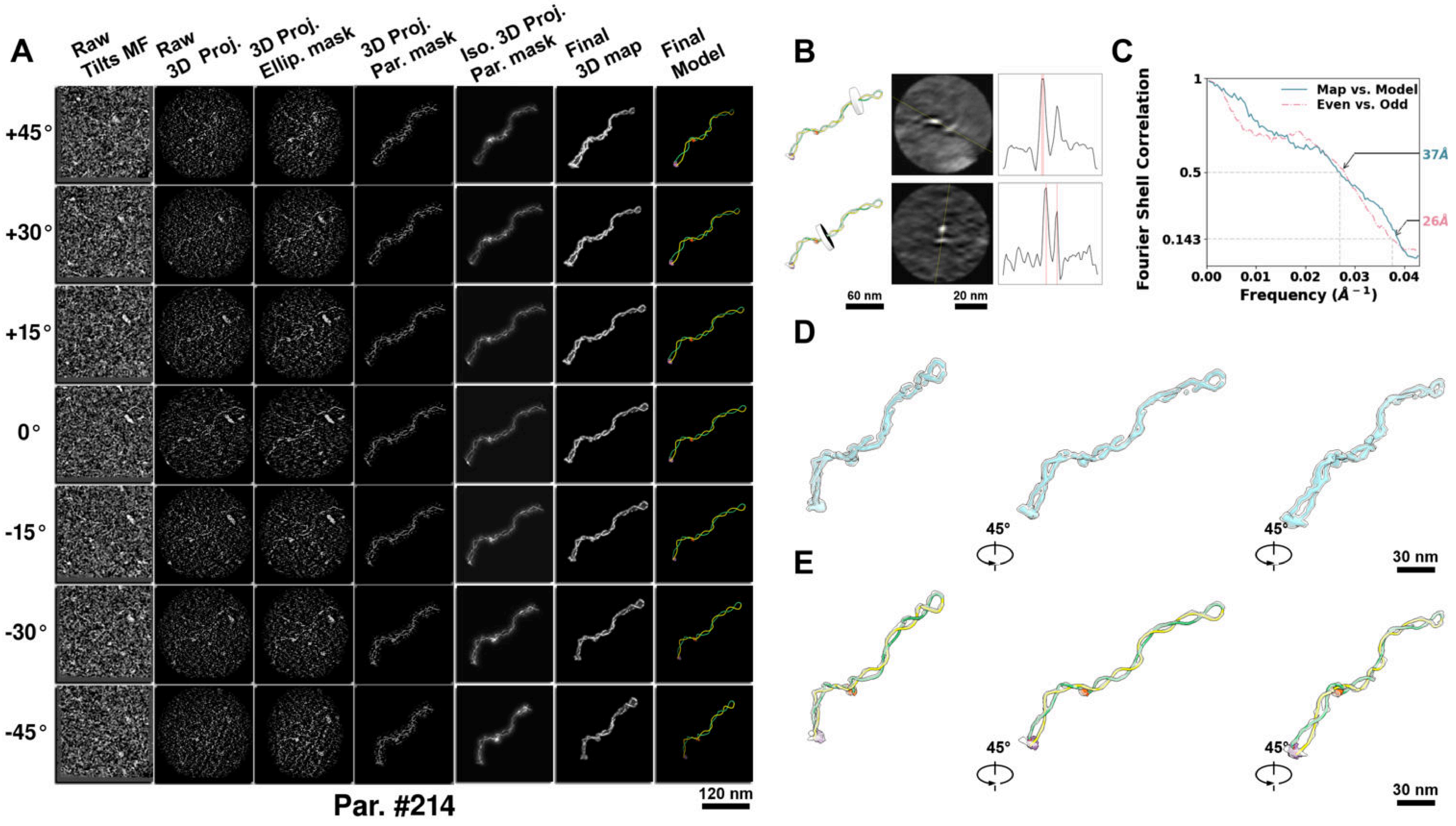

Supplementary Particle Figure 214. Cryo-ET 3D reconstruction of an individual TEC-Cas particle.
(A) 3D reconstruction of the plasmid particle (index no. 214). The first column shows seven representative tilt images from +45° to -45° in step of 15°. The second, third, and fourth columns show 3D projections of the particle with spherical, ellipsoidal (thinner along the z-dimension), and particle-shaped masks, respectively. The fifth column displays the 3D projections of the enhanced and IsoNet missing-wedge-corrected particle. The sixth and seventh columns present the final 3D map and the flexibly fitted model, respectively. (B) Two cross-sectional views (12 nm thickness) of the plasmid density map along its plectoneme axis are shown in the left-middle panel. The intensity profile along the line crossing the two high-density DNA spots is displayed in the right panel. (C) Resolution assessment of the final 3D map using Fourier shell correlation (FSC). Two criteria are shown: FSC between two half-maps reconstructed from even and odd frames (evaluated at 0.143) and FSC between the final 3D map and the fitted model (evaluated at 0.5). (D) Zoomed-in views of the final 3D density map from panel A, displayed at two contour levels. (E) Superimposition of the high-contour level map from panel D onto its fitted model.

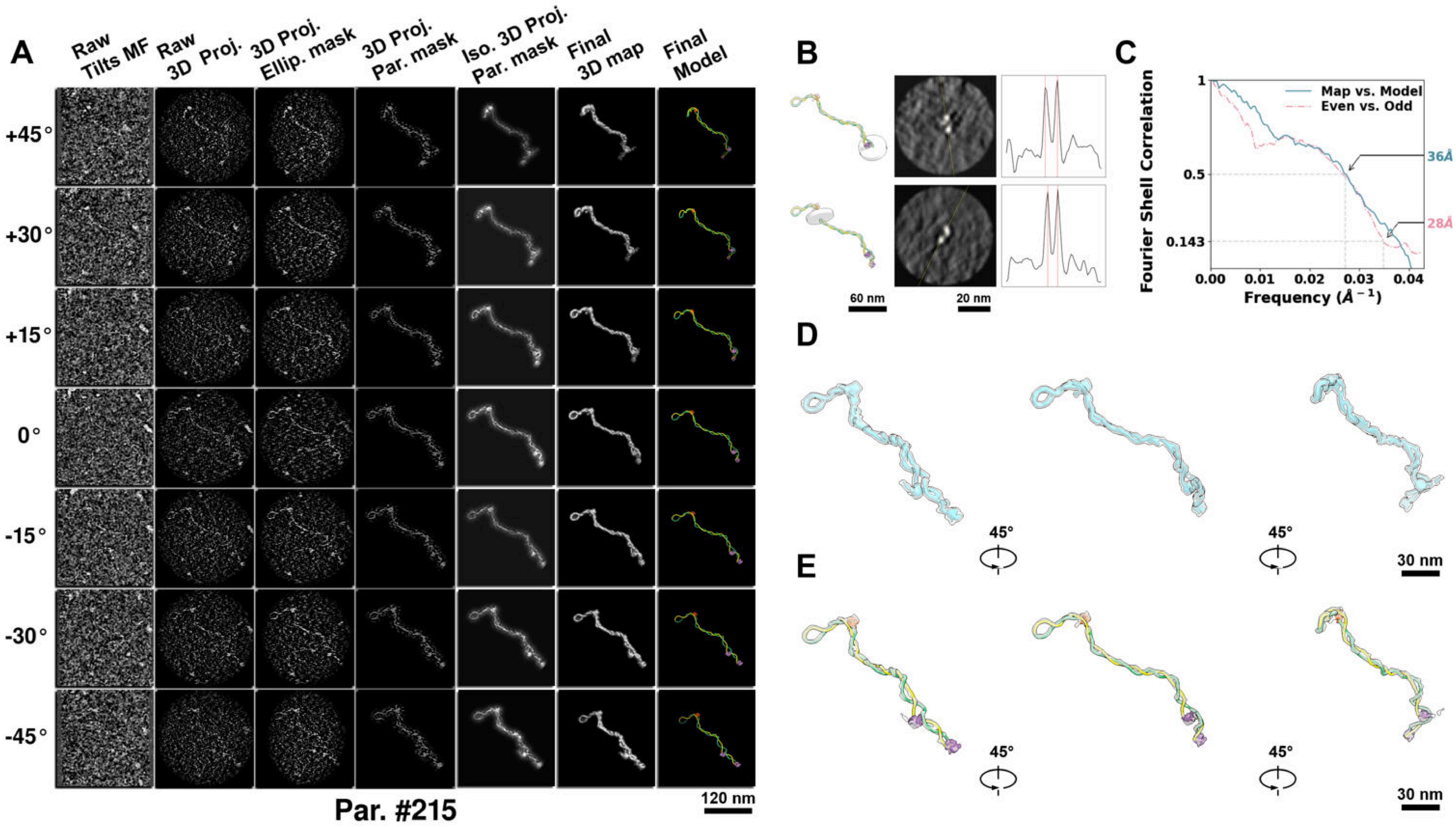

Supplementary Particle Figure 215. Cryo-ET 3D reconstruction of an individual TEC-Cas particle.
(A) 3D reconstruction of the plasmid particle (index no. 215). The first column shows seven representative tilt images from +45° to -45° in step of 15°. The second, third, and fourth columns show 3D projections of the particle with spherical, ellipsoidal (thinner along the z-dimension), and particle-shaped masks, respectively. The fifth column displays the 3D projections of the enhanced and IsoNet missing-wedge-corrected particle. The sixth and seventh columns present the final 3D map and the flexibly fitted model, respectively. (B) Two cross-sectional views (12 nm thickness) of the plasmid density map along its plectoneme axis are shown in the left-middle panel. The intensity profile along the line crossing the two high-density DNA spots is displayed in the right panel. (C) Resolution assessment of the final 3D map using Fourier shell correlation (FSC). Two criteria are shown: FSC between two half-maps reconstructed from even and odd frames (evaluated at 0.143) and FSC between the final 3D map and the fitted model (evaluated at 0.5). (D) Zoomed-in views of the final 3D density map from panel A, displayed at two contour levels. (E) Superimposition of the high-contour level map from panel D onto its fitted model.

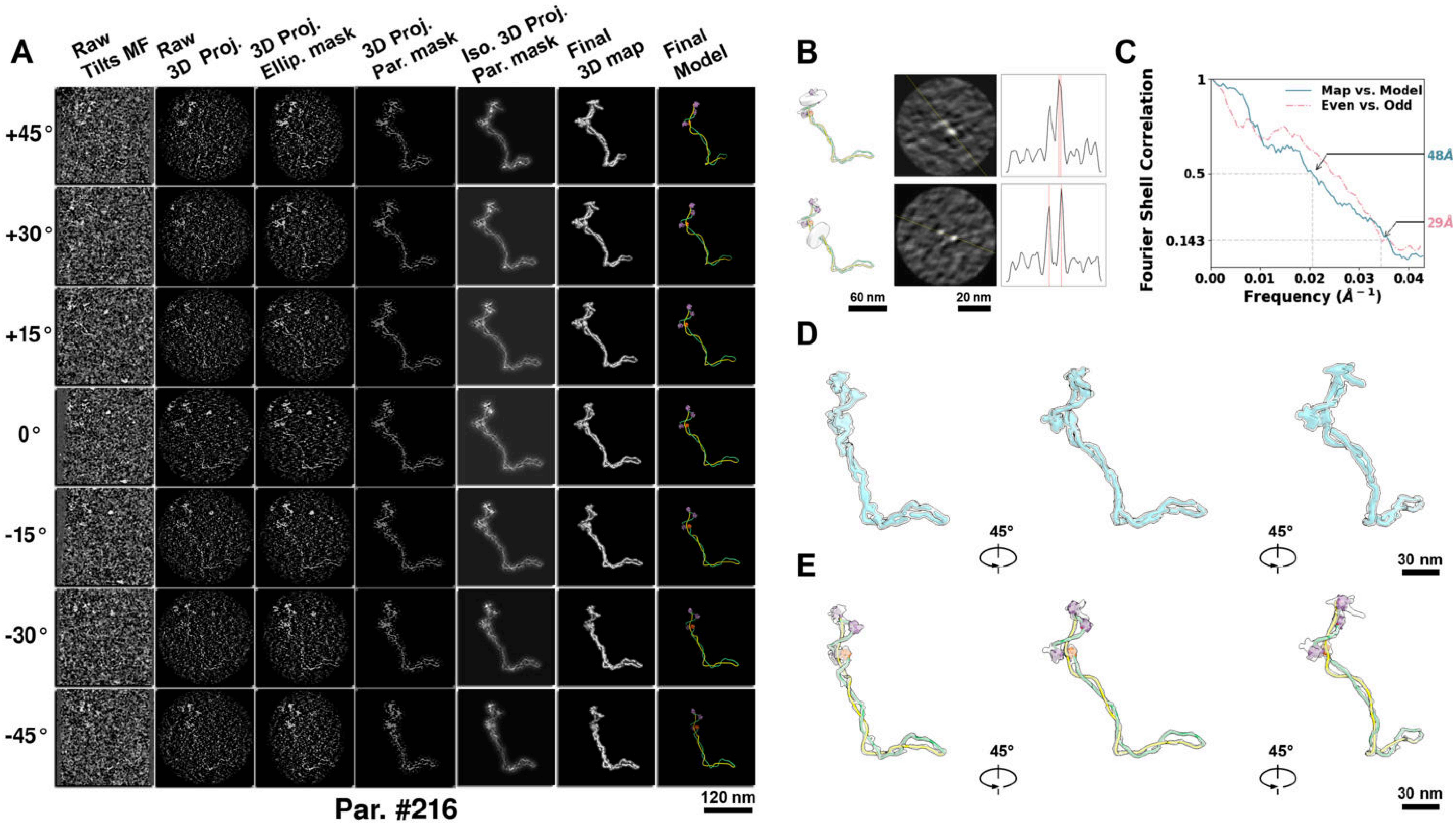

Supplementary Particle Figure 216. Cryo-ET 3D reconstruction of an individual TEC-Cas particle.
(A) 3D reconstruction of the plasmid particle (index no. 216). The first column shows seven representative tilt images from +45° to -45° in step of 15°. The second, third, and fourth columns show 3D projections of the particle with spherical, ellipsoidal (thinner along the z-dimension), and particle-shaped masks, respectively. The fifth column displays the 3D projections of the enhanced and IsoNet missing-wedge-corrected particle. The sixth and seventh columns present the final 3D map and the flexibly fitted model, respectively. (B) Two cross-sectional views (12 nm thickness) of the plasmid density map along its plectoneme axis are shown in the left-middle panel. The intensity profile along the line crossing the two high-density DNA spots is displayed in the right panel. (C) Resolution assessment of the final 3D map using Fourier shell correlation (FSC). Two criteria are shown: FSC between two half-maps reconstructed from even and odd frames (evaluated at 0.143) and FSC between the final 3D map and the fitted model (evaluated at 0.5). (D) Zoomed-in views of the final 3D density map from panel A, displayed at two contour levels. (E) Superimposition of the high-contour level map from panel D onto its fitted model.

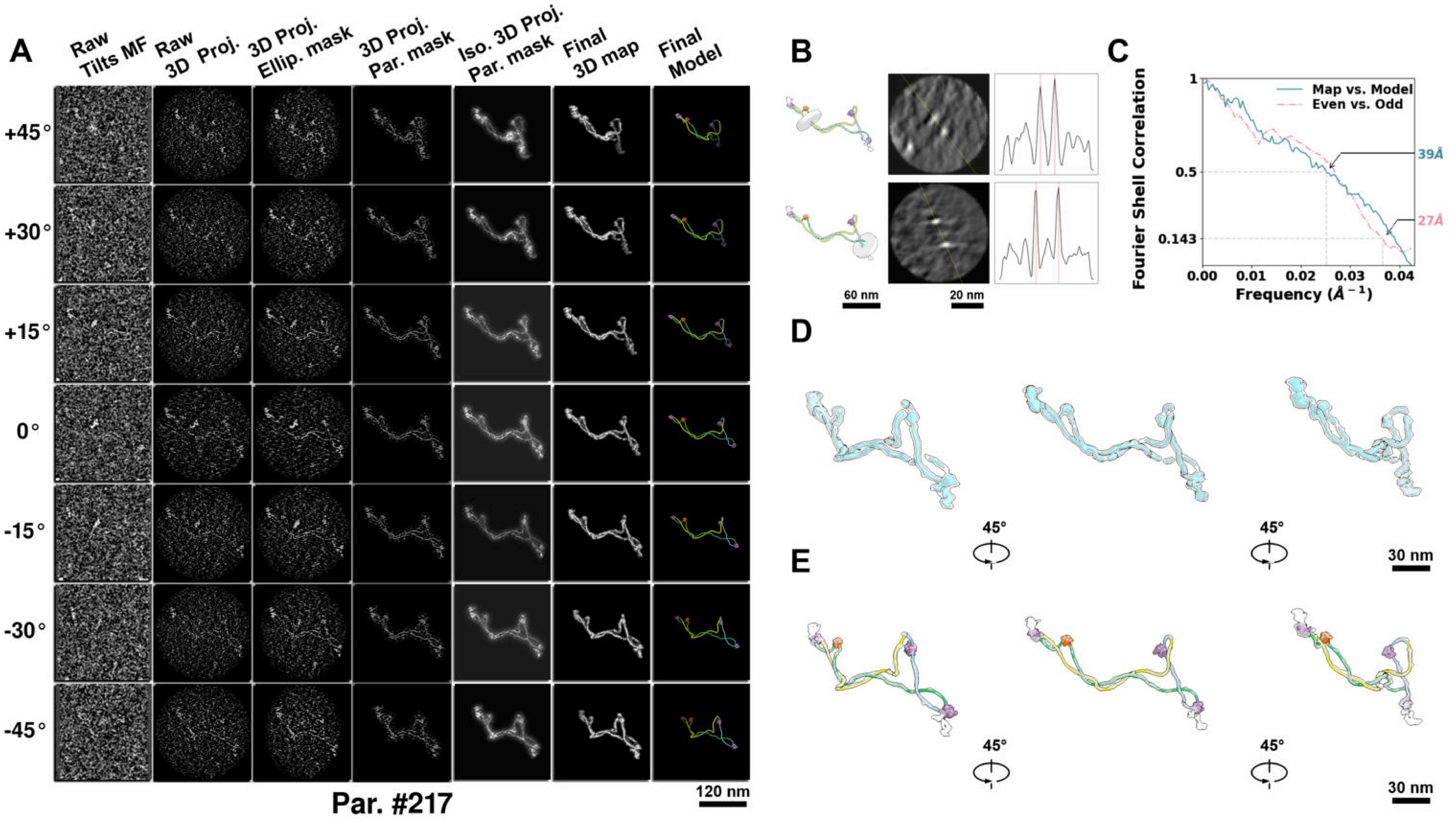

Supplementary Particle Figure 217. Cryo-ET 3D reconstruction of an individual TEC-Cas particle.
(A) 3D reconstruction of the plasmid particle (index no. 217). The first column shows seven representative tilt images from +45° to -45° in step of 15°. The second, third, and fourth columns show 3D projections of the particle with spherical, ellipsoidal (thinner along the z-dimension), and particle-shaped masks, respectively. The fifth column displays the 3D projections of the enhanced and IsoNet missing-wedge-corrected particle. The sixth and seventh columns present the final 3D map and the flexibly fitted model, respectively. (B) Two cross-sectional views (12 nm thickness) of the plasmid density map along its plectoneme axis are shown in the left-middle panel. The intensity profile along the line crossing the two high-density DNA spots is displayed in the right panel. (C) Resolution assessment of the final 3D map using Fourier shell correlation (FSC). Two criteria are shown: FSC between two half-maps reconstructed from even and odd frames (evaluated at 0.143) and FSC between the final 3D map and the fitted model (evaluated at 0.5). (D) Zoomed-in views of the final 3D density map from panel A, displayed at two contour levels. (E) Superimposition of the high-contour level map from panel D onto its fitted model.

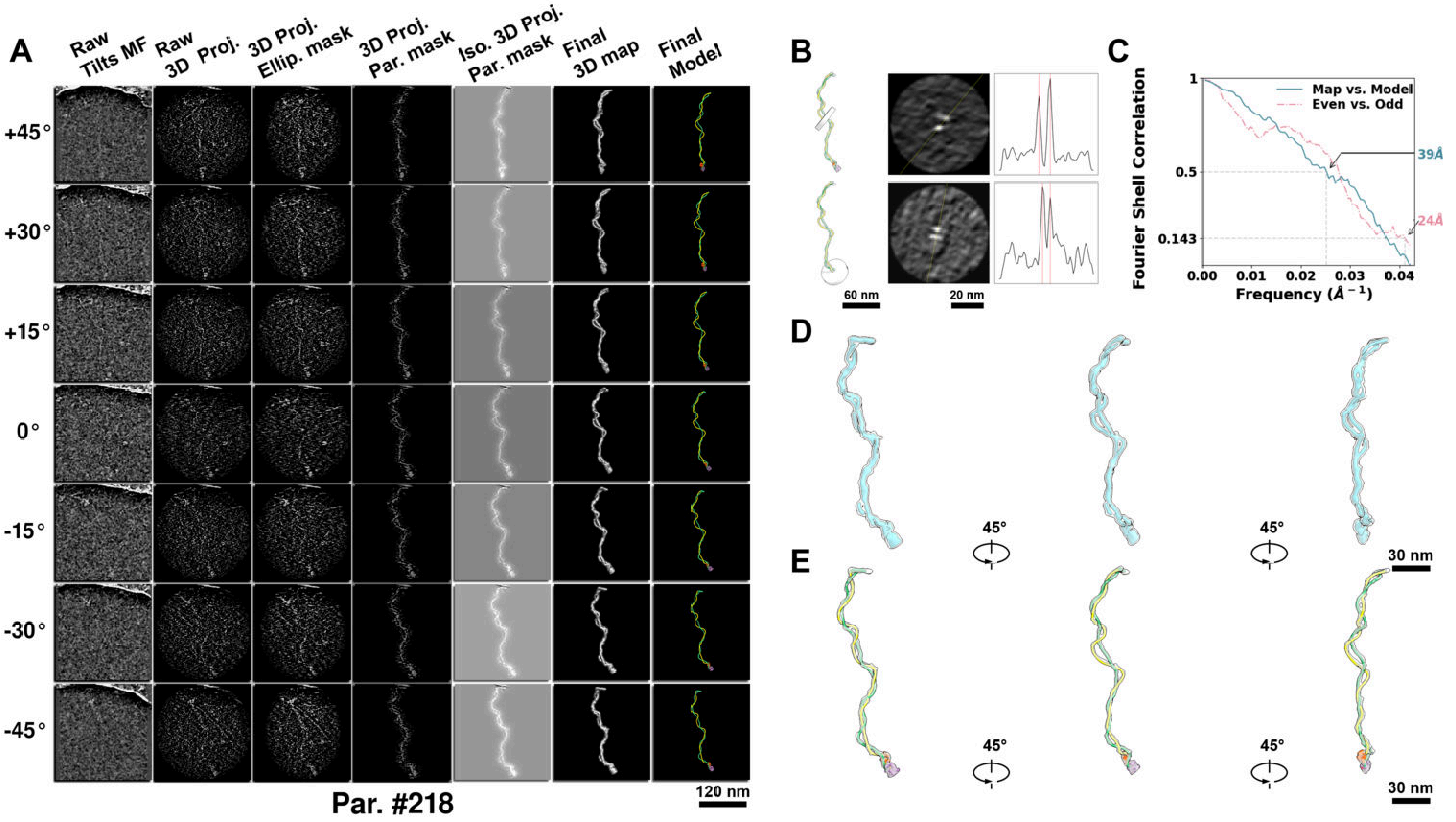

Supplementary Particle Figure 218. Cryo-ET 3D reconstruction of an individual TEC-Cas particle.
(A) 3D reconstruction of the plasmid particle (index no. 218). The first column shows seven representative tilt images from +45° to -45° in step of 15°. The second, third, and fourth columns show 3D projections of the particle with spherical, ellipsoidal (thinner along the z-dimension), and particle-shaped masks, respectively. The fifth column displays the 3D projections of the enhanced and IsoNet missing-wedge-corrected particle. The sixth and seventh columns present the final 3D map and the flexibly fitted model, respectively. (B) Two cross-sectional views (12 nm thickness) of the plasmid density map along its plectoneme axis are shown in the left-middle panel. The intensity profile along the line crossing the two high-density DNA spots is displayed in the right panel. (C) Resolution assessment of the final 3D map using Fourier shell correlation (FSC). Two criteria are shown: FSC between two half-maps reconstructed from even and odd frames (evaluated at 0.143) and FSC between the final 3D map and the fitted model (evaluated at 0.5). (D) Zoomed-in views of the final 3D density map from panel A, displayed at two contour levels. (E) Superimposition of the high-contour level map from panel D onto its fitted model.

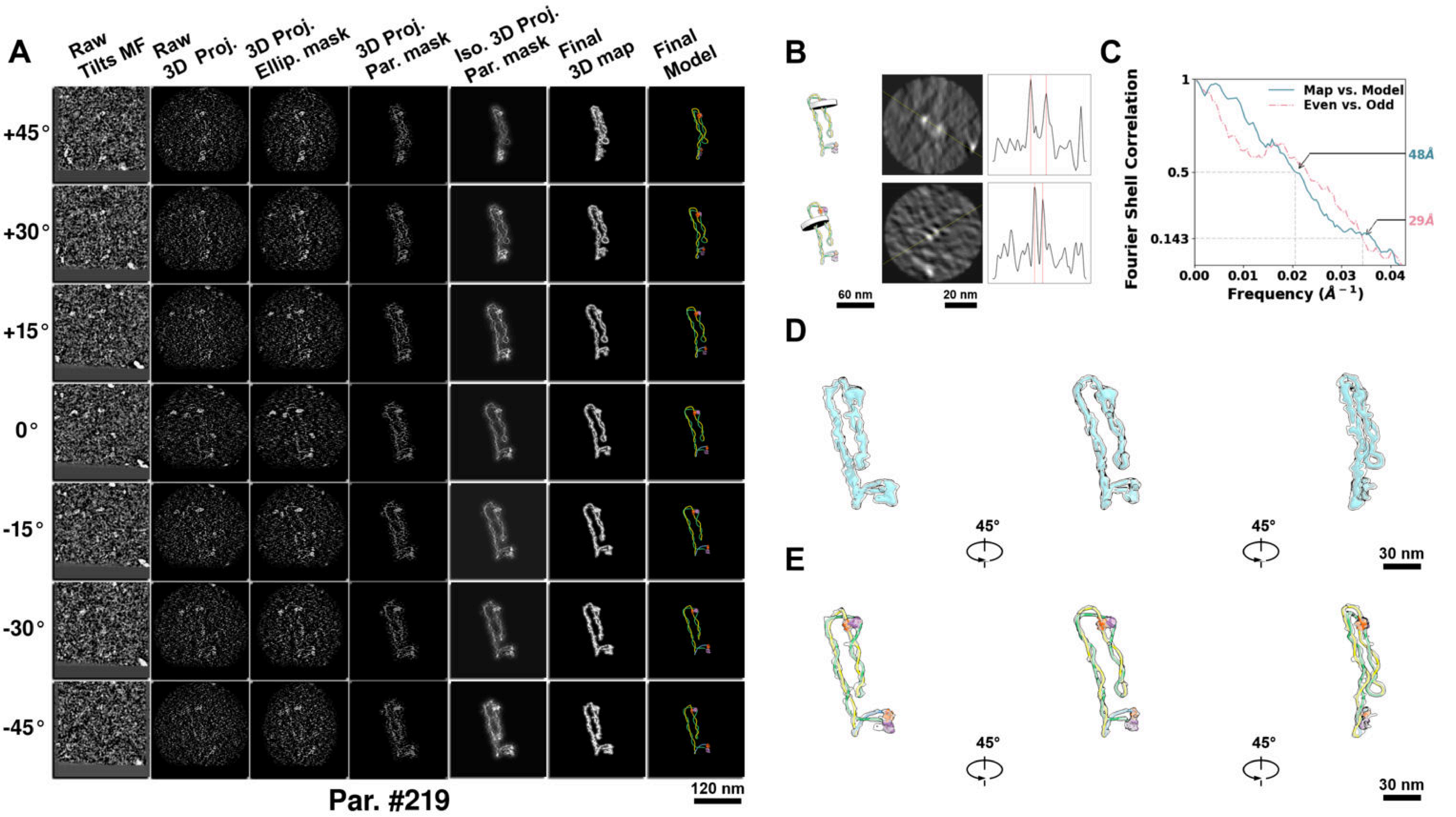

Supplementary Particle Figure 219. Cryo-ET 3D reconstruction of an individual TEC-Cas particle.
(A) 3D reconstruction of the plasmid particle (index no. 219). The first column shows seven representative tilt images from +45° to -45° in step of 15°. The second, third, and fourth columns show 3D projections of the particle with spherical, ellipsoidal (thinner along the z-dimension), and particle-shaped masks, respectively. The fifth column displays the 3D projections of the enhanced and IsoNet missing-wedge-corrected particle. The sixth and seventh columns present the final 3D map and the flexibly fitted model, respectively. (B) Two cross-sectional views (12 nm thickness) of the plasmid density map along its plectoneme axis are shown in the left-middle panel. The intensity profile along the line crossing the two high-density DNA spots is displayed in the right panel. (C) Resolution assessment of the final 3D map using Fourier shell correlation (FSC). Two criteria are shown: FSC between two half-maps reconstructed from even and odd frames (evaluated at 0.143) and FSC between the final 3D map and the fitted model (evaluated at 0.5). (D) Zoomed-in views of the final 3D density map from panel A, displayed at two contour levels. (E) Superimposition of the high-contour level map from panel D onto its fitted model.

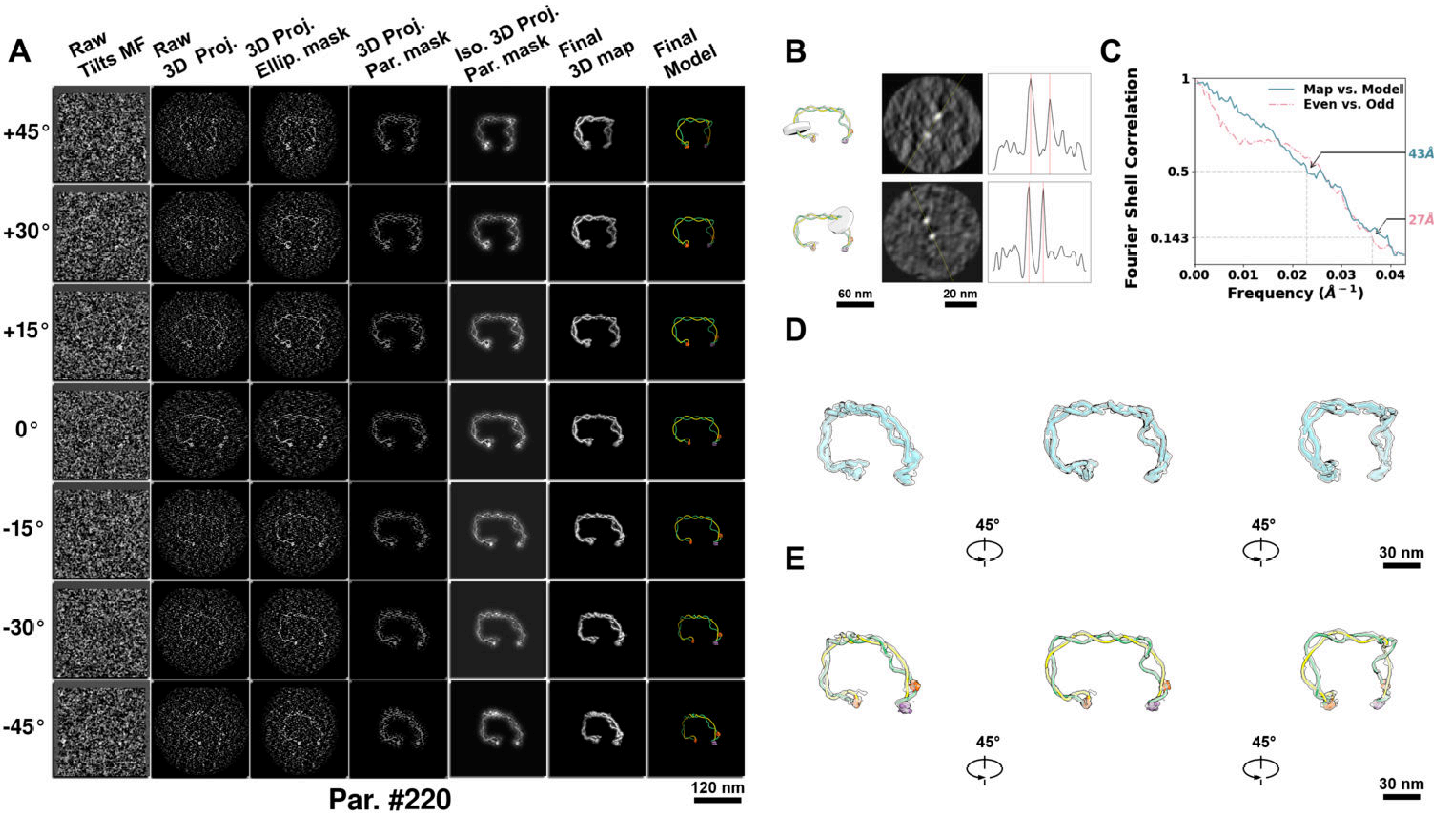

Supplementary Particle Figure 220. Cryo-ET 3D reconstruction of an individual TEC-Cas particle.
(A) 3D reconstruction of the plasmid particle (index no. 220). The first column shows seven representative tilt images from +45° to -45° in step of 15°. The second, third, and fourth columns show 3D projections of the particle with spherical, ellipsoidal (thinner along the z-dimension), and particle-shaped masks, respectively. The fifth column displays the 3D projections of the enhanced and IsoNet missing-wedge-corrected particle. The sixth and seventh columns present the final 3D map and the flexibly fitted model, respectively. (B) Two cross-sectional views (12 nm thickness) of the plasmid density map along its plectoneme axis are shown in the left-middle panel. The intensity profile along the line crossing the two high-density DNA spots is displayed in the right panel. (C) Resolution assessment of the final 3D map using Fourier shell correlation (FSC). Two criteria are shown: FSC between two half-maps reconstructed from even and odd frames (evaluated at 0.143) and FSC between the final 3D map and the fitted model (evaluated at 0.5). (D) Zoomed-in views of the final 3D density map from panel A, displayed at two contour levels. (E) Superimposition of the high-contour level map from panel D onto its fitted model.

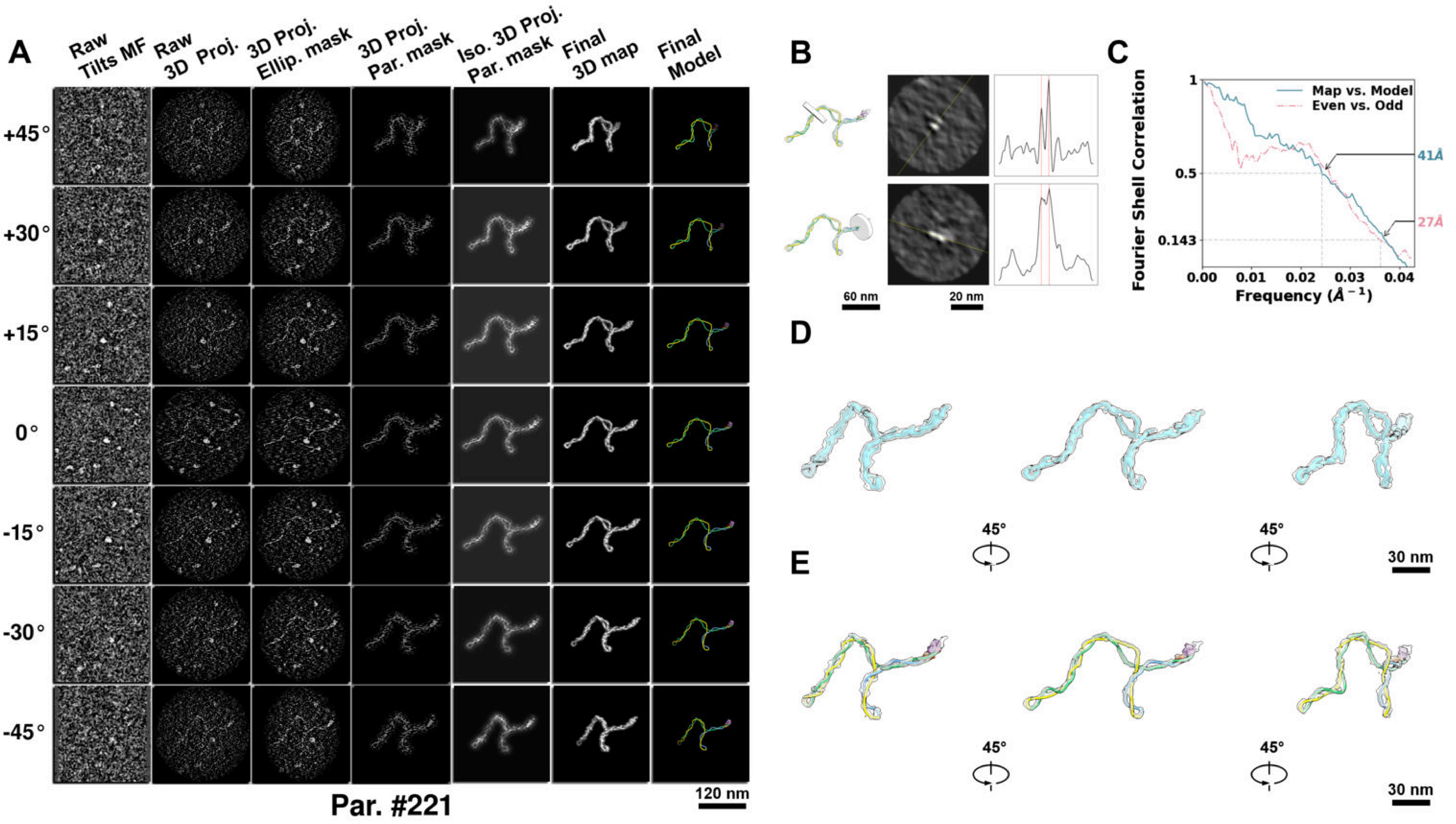

Supplementary Particle Figure 221. Cryo-ET 3D reconstruction of an individual TEC-Cas particle.
(A) 3D reconstruction of the plasmid particle (index no. 221). The first column shows seven representative tilt images from +45° to -45° in step of 15°. The second, third, and fourth columns show 3D projections of the particle with spherical, ellipsoidal (thinner along the z-dimension), and particle-shaped masks, respectively. The fifth column displays the 3D projections of the enhanced and IsoNet missing-wedge-corrected particle. The sixth and seventh columns present the final 3D map and the flexibly fitted model, respectively. (B) Two cross-sectional views (12 nm thickness) of the plasmid density map along its plectoneme axis are shown in the left-middle panel. The intensity profile along the line crossing the two high-density DNA spots is displayed in the right panel. (C) Resolution assessment of the final 3D map using Fourier shell correlation (FSC). Two criteria are shown: FSC between two half-maps reconstructed from even and odd frames (evaluated at 0.143) and FSC between the final 3D map and the fitted model (evaluated at 0.5). (D) Zoomed-in views of the final 3D density map from panel A, displayed at two contour levels. (E) Superimposition of the high-contour level map from panel D onto its fitted model.

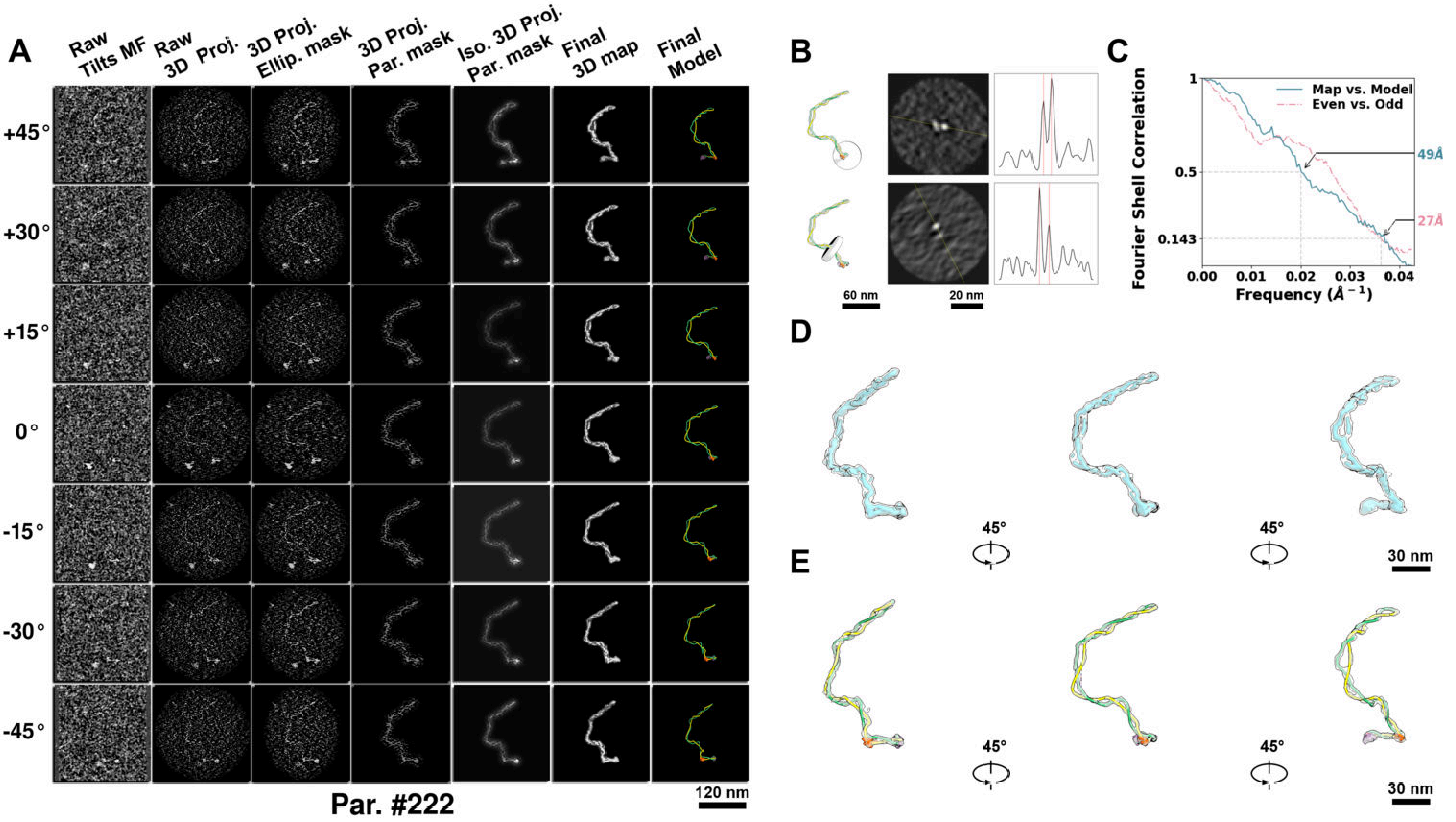

Supplementary Particle Figure 222. Cryo-ET 3D reconstruction of an individual TEC-Cas particle.
(A) 3D reconstruction of the plasmid particle (index no. 222). The first column shows seven representative tilt images from +45° to -45° in step of 15°. The second, third, and fourth columns show 3D projections of the particle with spherical, ellipsoidal (thinner along the z-dimension), and particle-shaped masks, respectively. The fifth column displays the 3D projections of the enhanced and IsoNet missing-wedge-corrected particle. The sixth and seventh columns present the final 3D map and the flexibly fitted model, respectively. (B) Two cross-sectional views (12 nm thickness) of the plasmid density map along its plectoneme axis are shown in the left-middle panel. The intensity profile along the line crossing the two high-density DNA spots is displayed in the right panel. (C) Resolution assessment of the final 3D map using Fourier shell correlation (FSC). Two criteria are shown: FSC between two half-maps reconstructed from even and odd frames (evaluated at 0.143) and FSC between the final 3D map and the fitted model (evaluated at 0.5). (D) Zoomed-in views of the final 3D density map from panel A, displayed at two contour levels. (E) Superimposition of the high-contour level map from panel D onto its fitted model.

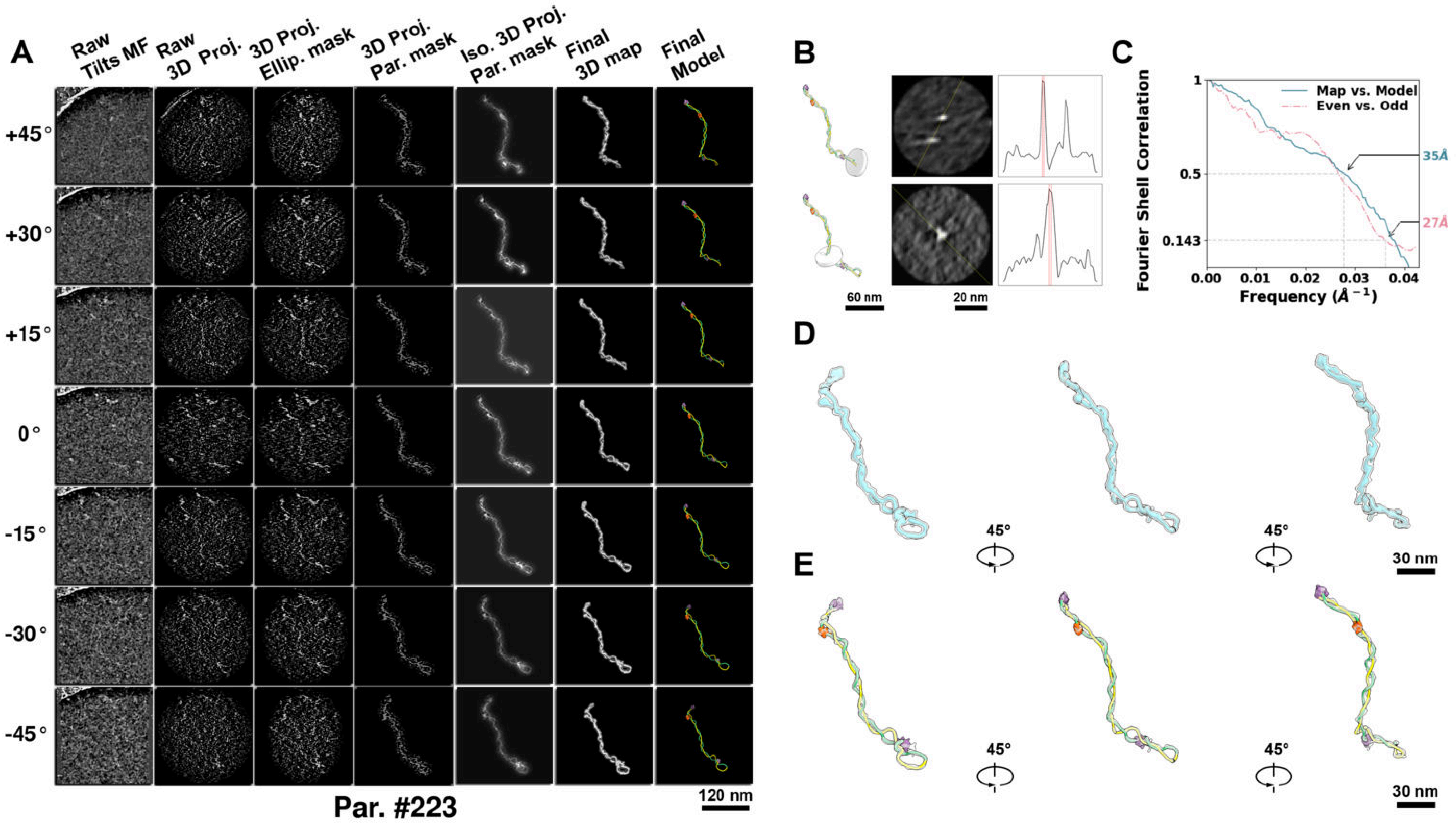

Supplementary Particle Figure 223. Cryo-ET 3D reconstruction of an individual TEC-Cas particle.
(A) 3D reconstruction of the plasmid particle (index no. 223). The first column shows seven representative tilt images from +45° to -45° in step of 15°. The second, third, and fourth columns show 3D projections of the particle with spherical, ellipsoidal (thinner along the z-dimension), and particle-shaped masks, respectively. The fifth column displays the 3D projections of the enhanced and IsoNet missing-wedge-corrected particle. The sixth and seventh columns present the final 3D map and the flexibly fitted model, respectively. (B) Two cross-sectional views (12 nm thickness) of the plasmid density map along its plectoneme axis are shown in the left-middle panel. The intensity profile along the line crossing the two high-density DNA spots is displayed in the right panel. (C) Resolution assessment of the final 3D map using Fourier shell correlation (FSC). Two criteria are shown: FSC between two half-maps reconstructed from even and odd frames (evaluated at 0.143) and FSC between the final 3D map and the fitted model (evaluated at 0.5). (D) Zoomed-in views of the final 3D density map from panel A, displayed at two contour levels. (E) Superimposition of the high-contour level map from panel D onto its fitted model.

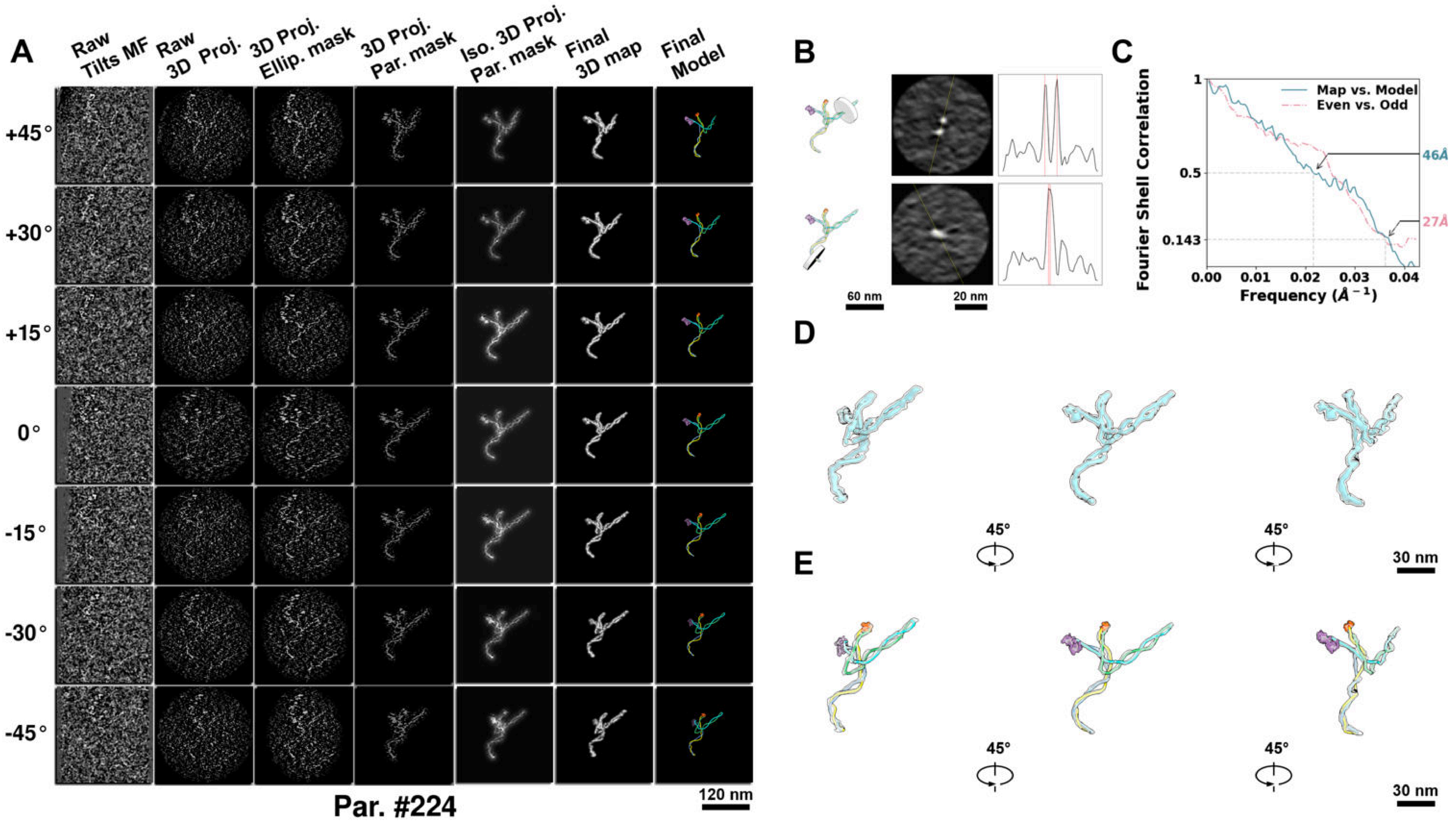

Supplementary Particle Figure 224. Cryo-ET 3D reconstruction of an individual TEC-Cas particle.
(A) 3D reconstruction of the plasmid particle (index no. 224). The first column shows seven representative tilt images from +45° to -45° in step of 15°. The second, third, and fourth columns show 3D projections of the particle with spherical, ellipsoidal (thinner along the z-dimension), and particle-shaped masks, respectively. The fifth column displays the 3D projections of the enhanced and IsoNet missing-wedge-corrected particle. The sixth and seventh columns present the final 3D map and the flexibly fitted model, respectively. (B) Two cross-sectional views (12 nm thickness) of the plasmid density map along its plectoneme axis are shown in the left-middle panel. The intensity profile along the line crossing the two high-density DNA spots is displayed in the right panel. (C) Resolution assessment of the final 3D map using Fourier shell correlation (FSC). Two criteria are shown: FSC between two half-maps reconstructed from even and odd frames (evaluated at 0.143) and FSC between the final 3D map and the fitted model (evaluated at 0.5). (D) Zoomed-in views of the final 3D density map from panel A, displayed at two contour levels. (E) Superimposition of the high-contour level map from panel D onto its fitted model.

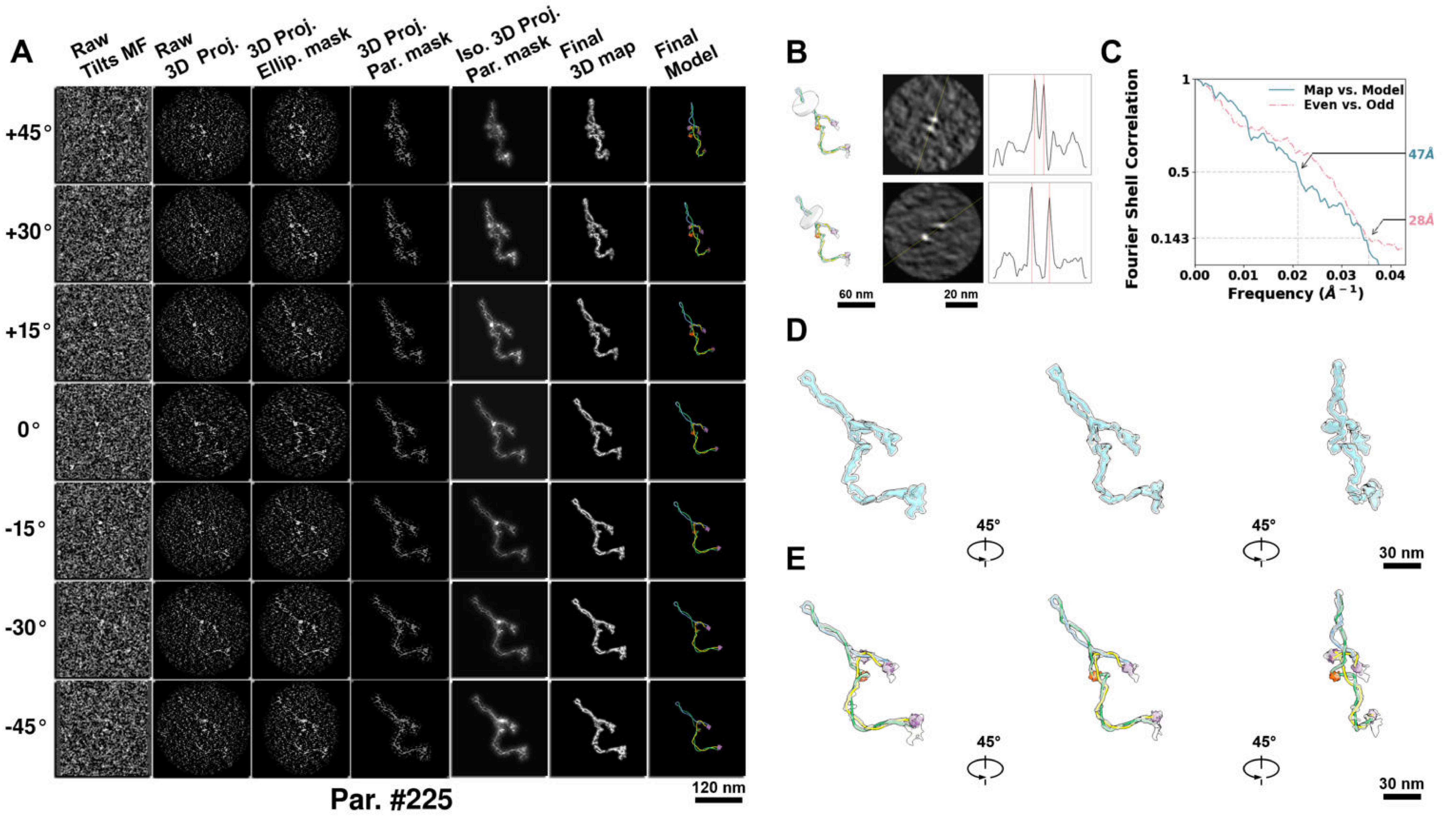

Supplementary Particle Figure 225. Cryo-ET 3D reconstruction of an individual TEC-Cas particle.
(A) 3D reconstruction of the plasmid particle (index no. 225). The first column shows seven representative tilt images from +45° to -45° in step of 15°. The second, third, and fourth columns show 3D projections of the particle with spherical, ellipsoidal (thinner along the z-dimension), and particle-shaped masks, respectively. The fifth column displays the 3D projections of the enhanced and IsoNet missing-wedge-corrected particle. The sixth and seventh columns present the final 3D map and the flexibly fitted model, respectively. (B) Two cross-sectional views (12 nm thickness) of the plasmid density map along its plectoneme axis are shown in the left-middle panel. The intensity profile along the line crossing the two high-density DNA spots is displayed in the right panel. (C) Resolution assessment of the final 3D map using Fourier shell correlation (FSC). Two criteria are shown: FSC between two half-maps reconstructed from even and odd frames (evaluated at 0.143) and FSC between the final 3D map and the fitted model (evaluated at 0.5). (D) Zoomed-in views of the final 3D density map from panel A, displayed at two contour levels. (E) Superimposition of the high-contour level map from panel D onto its fitted model.

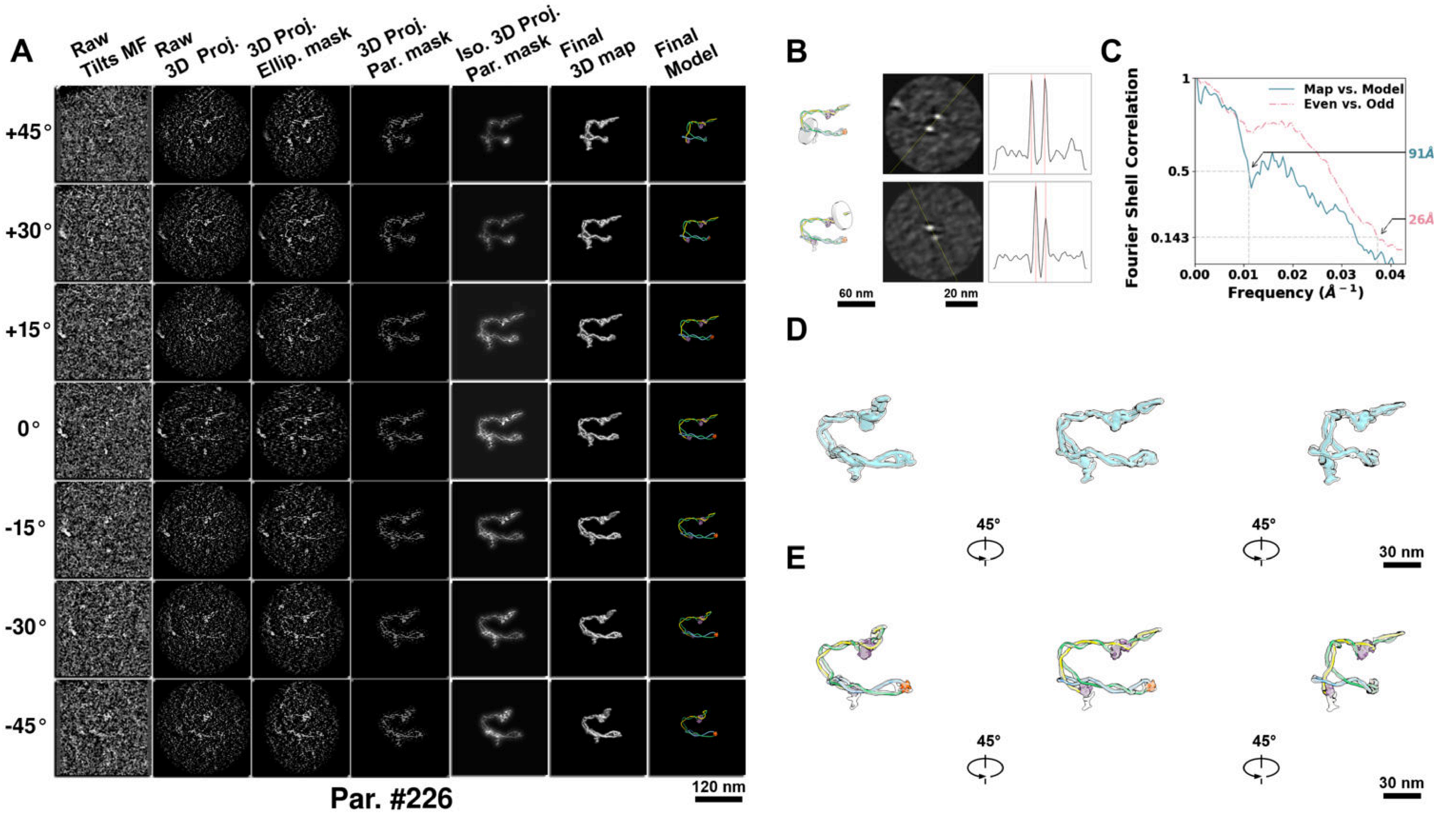

Supplementary Particle Figure 226. Cryo-ET 3D reconstruction of an individual TEC-Cas particle.
(A) 3D reconstruction of the plasmid particle (index no. 226). The first column shows seven representative tilt images from +45° to -45° in step of 15°. The second, third, and fourth columns show 3D projections of the particle with spherical, ellipsoidal (thinner along the z-dimension), and particle-shaped masks, respectively. The fifth column displays the 3D projections of the enhanced and IsoNet missing-wedge-corrected particle. The sixth and seventh columns present the final 3D map and the flexibly fitted model, respectively. (B) Two cross-sectional views (12 nm thickness) of the plasmid density map along its plectoneme axis are shown in the left-middle panel. The intensity profile along the line crossing the two high-density DNA spots is displayed in the right panel. (C) Resolution assessment of the final 3D map using Fourier shell correlation (FSC). Two criteria are shown: FSC between two half-maps reconstructed from even and odd frames (evaluated at 0.143) and FSC between the final 3D map and the fitted model (evaluated at 0.5). (D) Zoomed-in views of the final 3D density map from panel A, displayed at two contour levels. (E) Superimposition of the high-contour level map from panel D onto its fitted model.

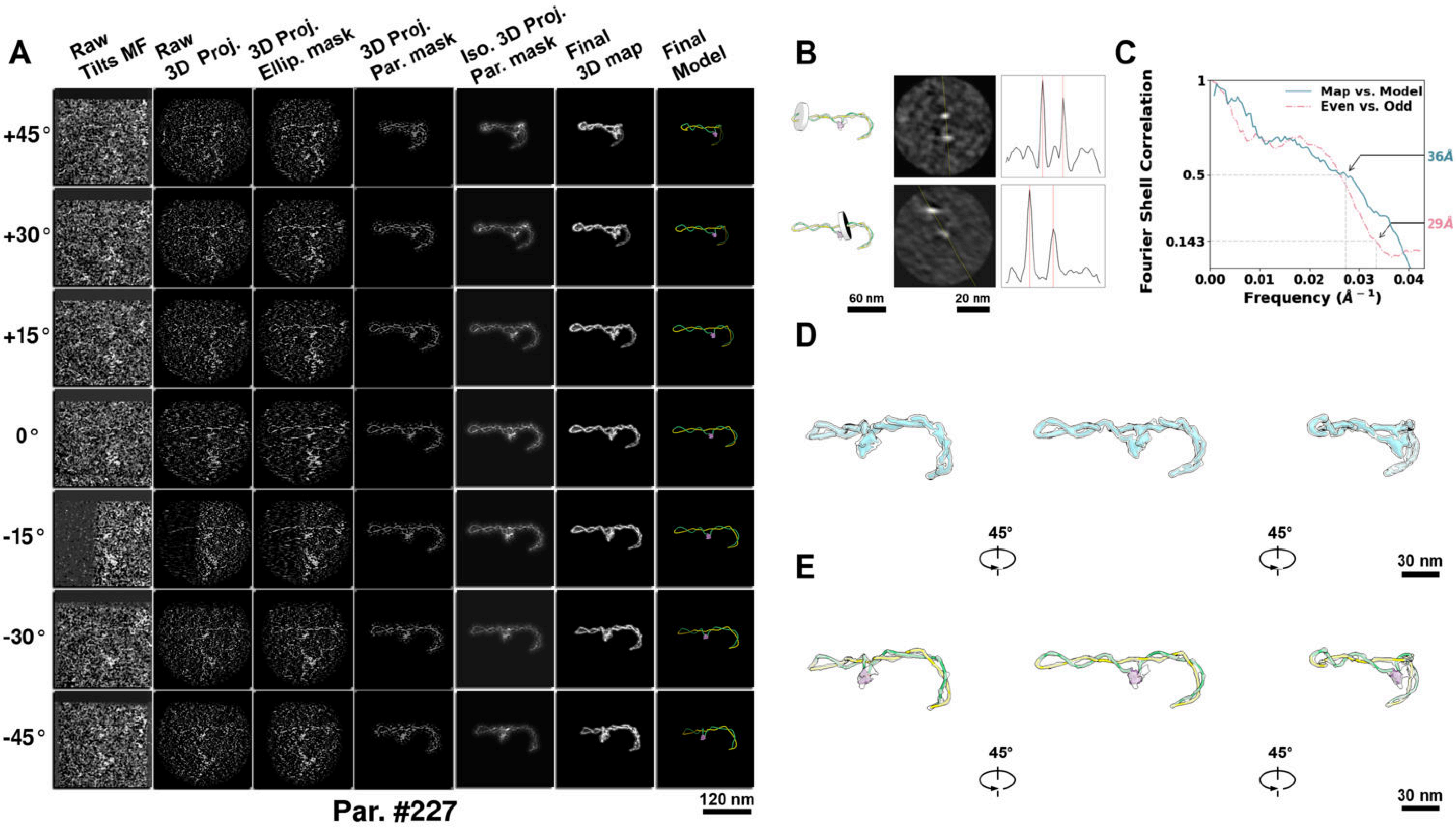

Supplementary Particle Figure 227. Cryo-ET 3D reconstruction of an individual TEC-TopI particle.
(A) 3D reconstruction of the plasmid particle (index no. 227). The first column shows seven representative tilt images from +45° to -45° in step of 15°. The second, third, and fourth columns show 3D projections of the particle with spherical, ellipsoidal (thinner along the z-dimension), and particle-shaped masks, respectively. The fifth column displays the 3D projections of the enhanced and IsoNet missing-wedge-corrected particle. The sixth and seventh columns present the final 3D map and the flexibly fitted model, respectively. (B) Two cross-sectional views (12 nm thickness) of the plasmid density map along its plectoneme axis are shown in the left-middle panel. The intensity profile along the line crossing the two high-density DNA spots is displayed in the right panel. (C) Resolution assessment of the final 3D map using Fourier shell correlation (FSC). Two criteria are shown: FSC between two half-maps reconstructed from even and odd frames (evaluated at 0.143) and FSC between the final 3D map and the fitted model (evaluated at 0.5). (D) Zoomed-in views of the final 3D density map from panel A, displayed at two contour levels. (E) Superimposition of the high-contour level map from panel D onto its fitted model.

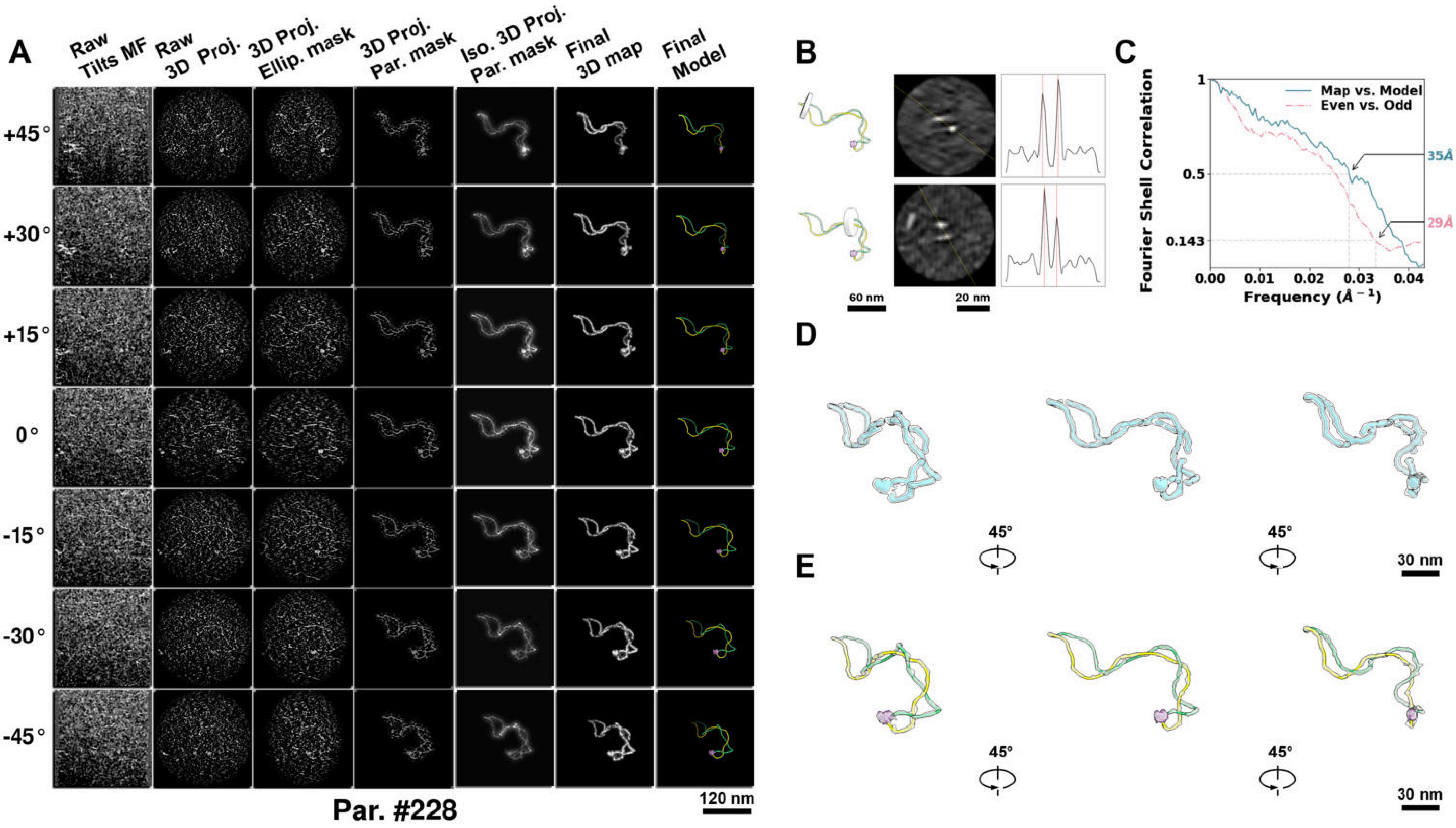

Supplementary Particle Figure 228. Cryo-ET 3D reconstruction of an individual TEC-TopI particle.
(A) 3D reconstruction of the plasmid particle (index no. 228). The first column shows seven representative tilt images from +45° to -45° in step of 15°. The second, third, and fourth columns show 3D projections of the particle with spherical, ellipsoidal (thinner along the z-dimension), and particle-shaped masks, respectively. The fifth column displays the 3D projections of the enhanced and IsoNet missing-wedge-corrected particle. The sixth and seventh columns present the final 3D map and the flexibly fitted model, respectively. (B) Two cross-sectional views (12 nm thickness) of the plasmid density map along its plectoneme axis are shown in the left-middle panel. The intensity profile along the line crossing the two high-density DNA spots is displayed in the right panel. (C) Resolution assessment of the final 3D map using Fourier shell correlation (FSC). Two criteria are shown: FSC between two half-maps reconstructed from even and odd frames (evaluated at 0.143) and FSC between the final 3D map and the fitted model (evaluated at 0.5). (D) Zoomed-in views of the final 3D density map from panel A, displayed at two contour levels. (E) Superimposition of the high-contour level map from panel D onto its fitted model.

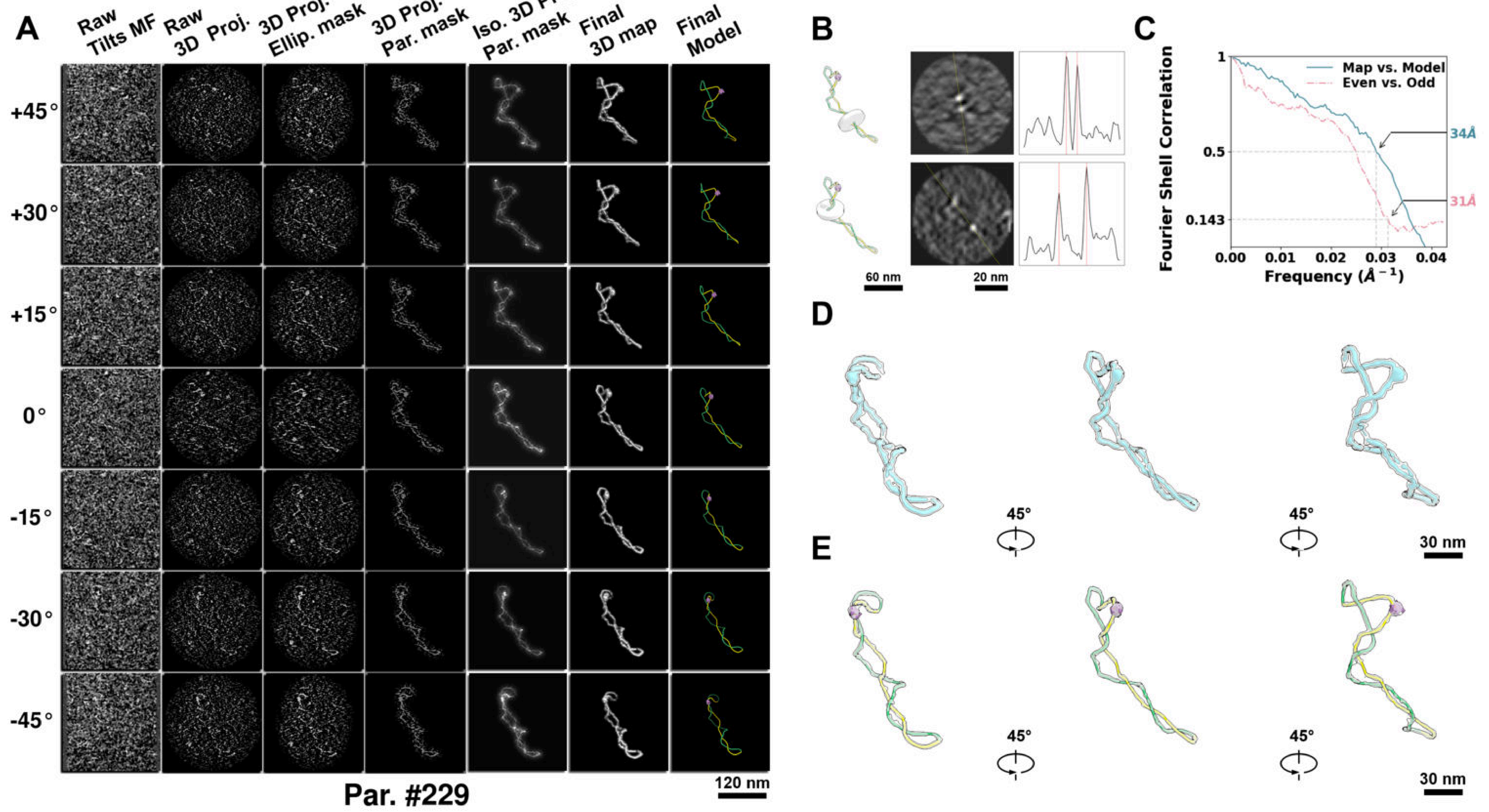

Supplementary Particle Figure 229. Cryo-ET 3D reconstruction of an individual TEC-TopI particle.
(A) 3D reconstruction of the plasmid particle (index no. 229). The first column shows seven representative tilt images from +45° to -45° in step of 15°. The second, third, and fourth columns show 3D projections of the particle with spherical, ellipsoidal (thinner along the z-dimension), and particle-shaped masks, respectively. The fifth column displays the 3D projections of the enhanced and IsoNet missing-wedge-corrected particle. The sixth and seventh columns present the final 3D map and the flexibly fitted model, respectively. (B) Two cross-sectional views (12 nm thickness) of the plasmid density map along its plectoneme axis are shown in the left-middle panel. The intensity profile along the line crossing the two high-density DNA spots is displayed in the right panel. (C) Resolution assessment of the final 3D map using Fourier shell correlation (FSC). Two criteria are shown: FSC between two half-maps reconstructed from even and odd frames (evaluated at 0.143) and FSC between the final 3D map and the fitted model (evaluated at 0.5). (D) Zoomed-in views of the final 3D density map from panel A, displayed at two contour levels. (E) Superimposition of the high-contour level map from panel D onto its fitted model.

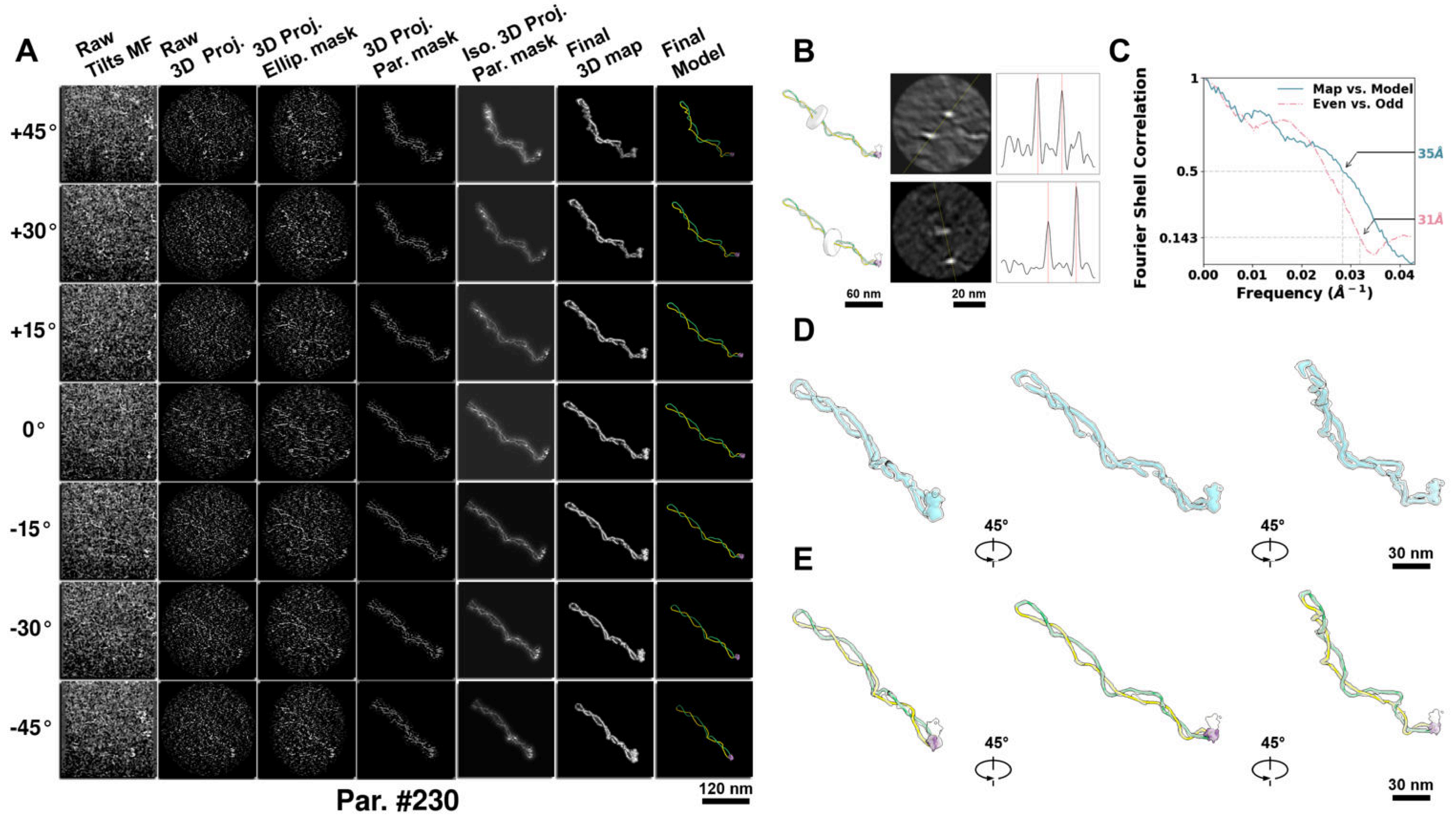

Supplementary Particle Figure 230. Cryo-ET 3D reconstruction of an individual TEC-TopI particle.
(A) 3D reconstruction of the plasmid particle (index no. 230). The first column shows seven representative tilt images from +45° to -45° in step of 15°. The second, third, and fourth columns show 3D projections of the particle with spherical, ellipsoidal (thinner along the z-dimension), and particle-shaped masks, respectively. The fifth column displays the 3D projections of the enhanced and IsoNet missing-wedge-corrected particle. The sixth and seventh columns present the final 3D map and the flexibly fitted model, respectively. (B) Two cross-sectional views (12 nm thickness) of the plasmid density map along its plectoneme axis are shown in the left-middle panel. The intensity profile along the line crossing the two high-density DNA spots is displayed in the right panel. (C) Resolution assessment of the final 3D map using Fourier shell correlation (FSC). Two criteria are shown: FSC between two half-maps reconstructed from even and odd frames (evaluated at 0.143) and FSC between the final 3D map and the fitted model (evaluated at 0.5). (D) Zoomed-in views of the final 3D density map from panel A, displayed at two contour levels. (E) Superimposition of the high-contour level map from panel D onto its fitted model.

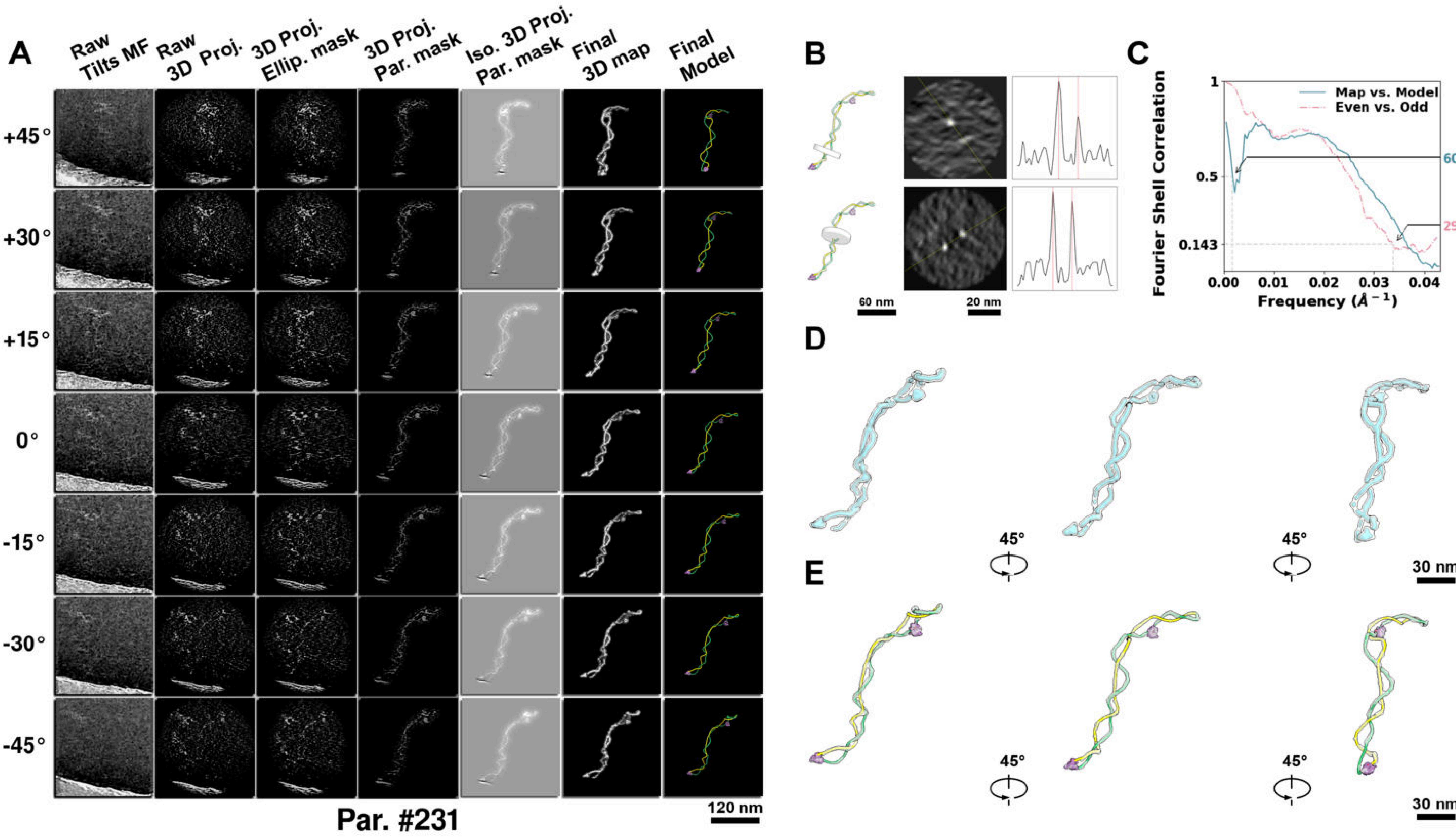

Supplementary Particle Figure 231. Cryo-ET 3D reconstruction of an individual TEC-TopI particle.
(A) 3D reconstruction of the plasmid particle (index no. 231). The first column shows seven representative tilt images from +45° to -45° in step of 15°. The second, third, and fourth columns show 3D projections of the particle with spherical, ellipsoidal (thinner along the z-dimension), and particle-shaped masks, respectively. The fifth column displays the 3D projections of the enhanced and IsoNet missing-wedge-corrected particle. The sixth and seventh columns present the final 3D map and the flexibly fitted model, respectively. (B) Two cross-sectional views (12 nm thickness) of the plasmid density map along its plectoneme axis are shown in the left-middle panel. The intensity profile along the line crossing the two high-density DNA spots is displayed in the right panel. (C) Resolution assessment of the final 3D map using Fourier shell correlation (FSC). Two criteria are shown: FSC between two half-maps reconstructed from even and odd frames (evaluated at 0.143) and FSC between the final 3D map and the fitted model (evaluated at 0.5). (D) Zoomed-in views of the final 3D density map from panel A, displayed at two contour levels. (E) Superimposition of the high-contour level map from panel D onto its fitted model.

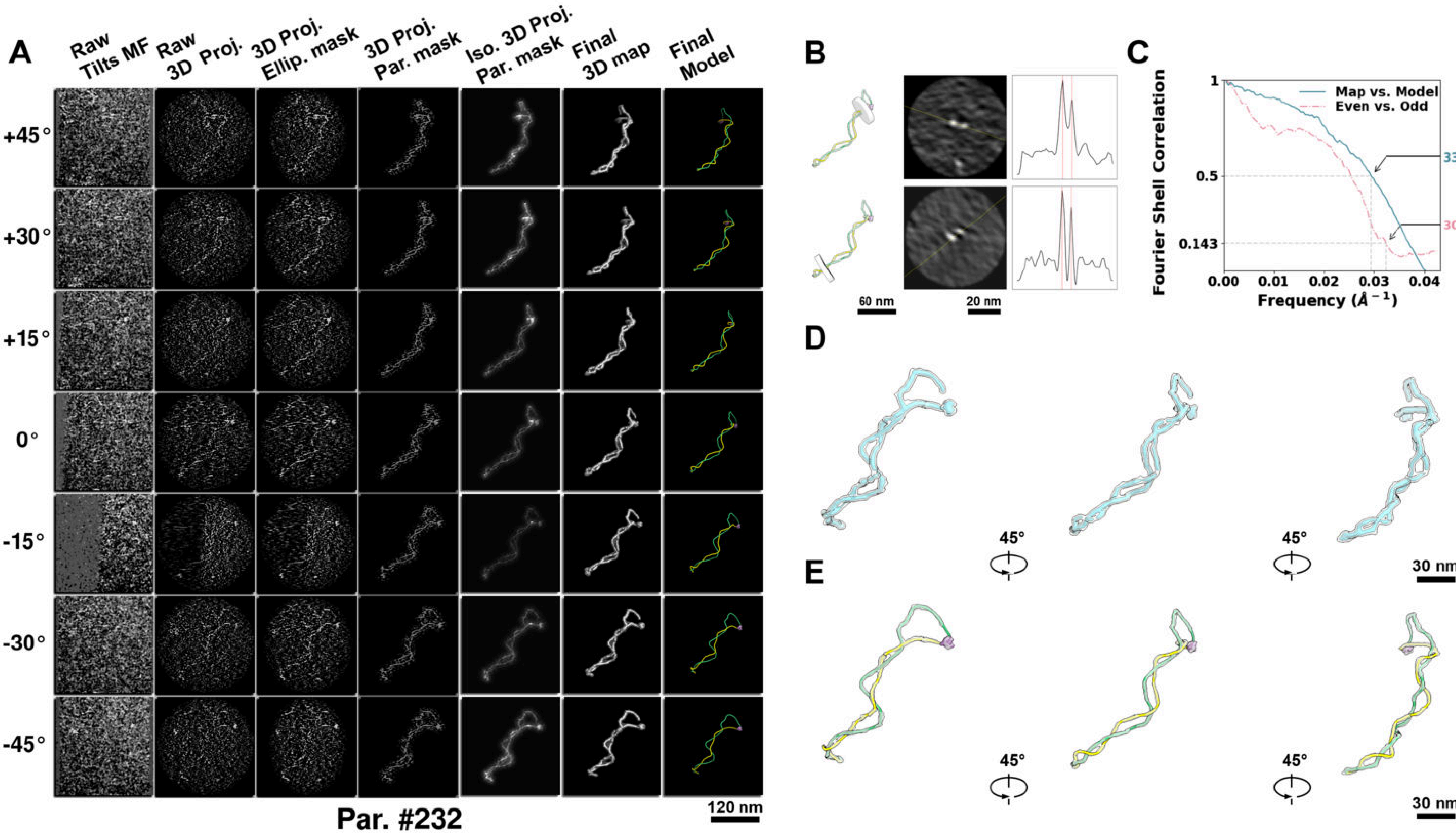

Supplementary Particle Figure 232. Cryo-ET 3D reconstruction of an individual TEC-TopI particle.
(A) 3D reconstruction of the plasmid particle (index no. 232). The first column shows seven representative tilt images from +45° to -45° in step of 15°. The second, third, and fourth columns show 3D projections of the particle with spherical, ellipsoidal (thinner along the z-dimension), and particle-shaped masks, respectively. The fifth column displays the 3D projections of the enhanced and IsoNet missing-wedge-corrected particle. The sixth and seventh columns present the final 3D map and the flexibly fitted model, respectively. (B) Two cross-sectional views (12 nm thickness) of the plasmid density map along its plectoneme axis are shown in the left-middle panel. The intensity profile along the line crossing the two high-density DNA spots is displayed in the right panel. (C) Resolution assessment of the final 3D map using Fourier shell correlation (FSC). Two criteria are shown: FSC between two half-maps reconstructed from even and odd frames (evaluated at 0.143) and FSC between the final 3D map and the fitted model (evaluated at 0.5). (D) Zoomed-in views of the final 3D density map from panel A, displayed at two contour levels. (E) Superimposition of the high-contour level map from panel D onto its fitted model.

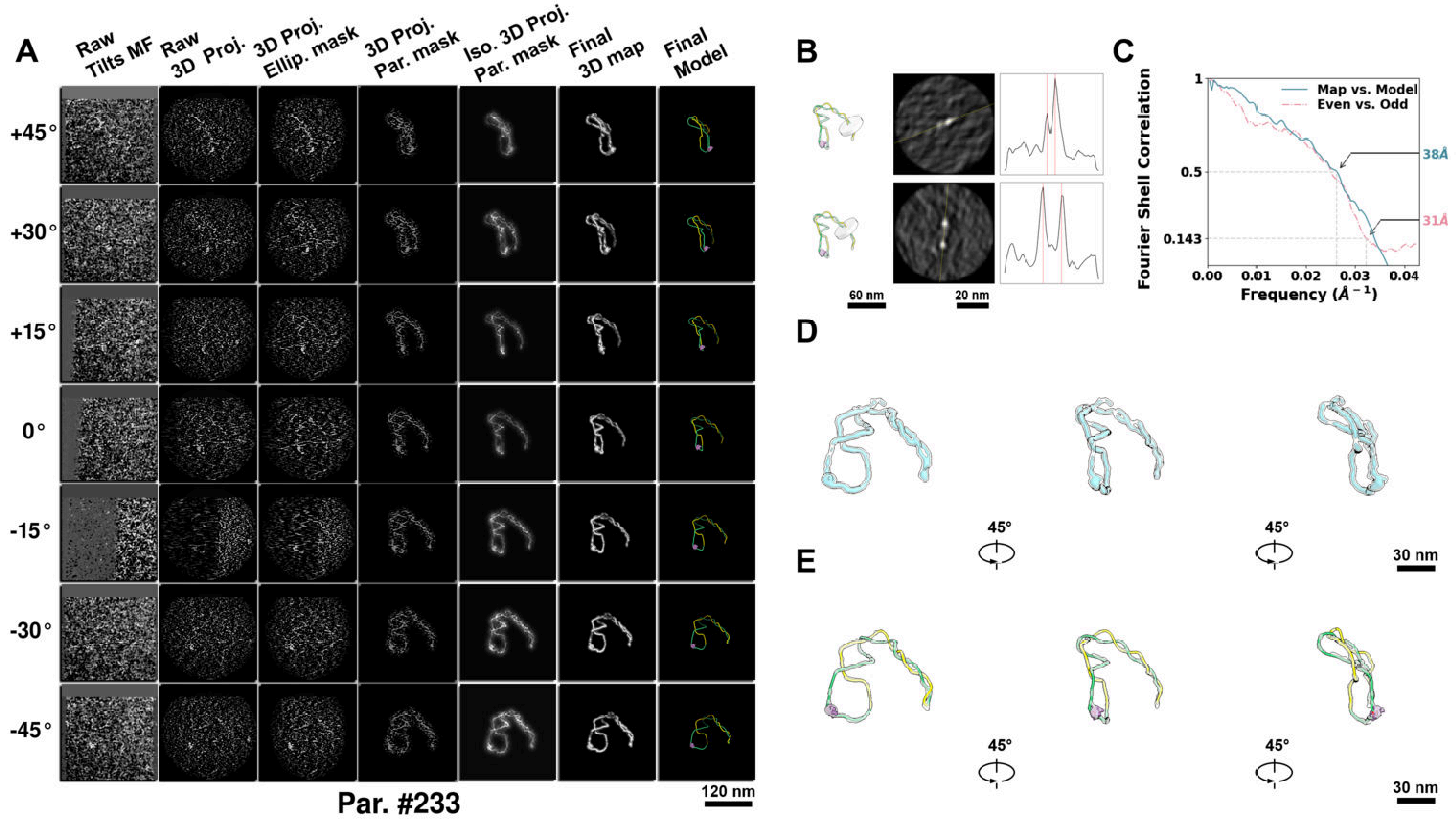

Supplementary Particle Figure 233. Cryo-ET 3D reconstruction of an individual TEC-TopI particle.
(A) 3D reconstruction of the plasmid particle (index no. 233). The first column shows seven representative tilt images from +45° to -45° in step of 15°. The second, third, and fourth columns show 3D projections of the particle with spherical, ellipsoidal (thinner along the z-dimension), and particle-shaped masks, respectively. The fifth column displays the 3D projections of the enhanced and IsoNet missing-wedge-corrected particle. The sixth and seventh columns present the final 3D map and the flexibly fitted model, respectively. (B) Two cross-sectional views (12 nm thickness) of the plasmid density map along its plectoneme axis are shown in the left-middle panel. The intensity profile along the line crossing the two high-density DNA spots is displayed in the right panel. (C) Resolution assessment of the final 3D map using Fourier shell correlation (FSC). Two criteria are shown: FSC between two half-maps reconstructed from even and odd frames (evaluated at 0.143) and FSC between the final 3D map and the fitted model (evaluated at 0.5). (D) Zoomed-in views of the final 3D density map from panel A, displayed at two contour levels. (E) Superimposition of the high-contour level map from panel D onto its fitted model.

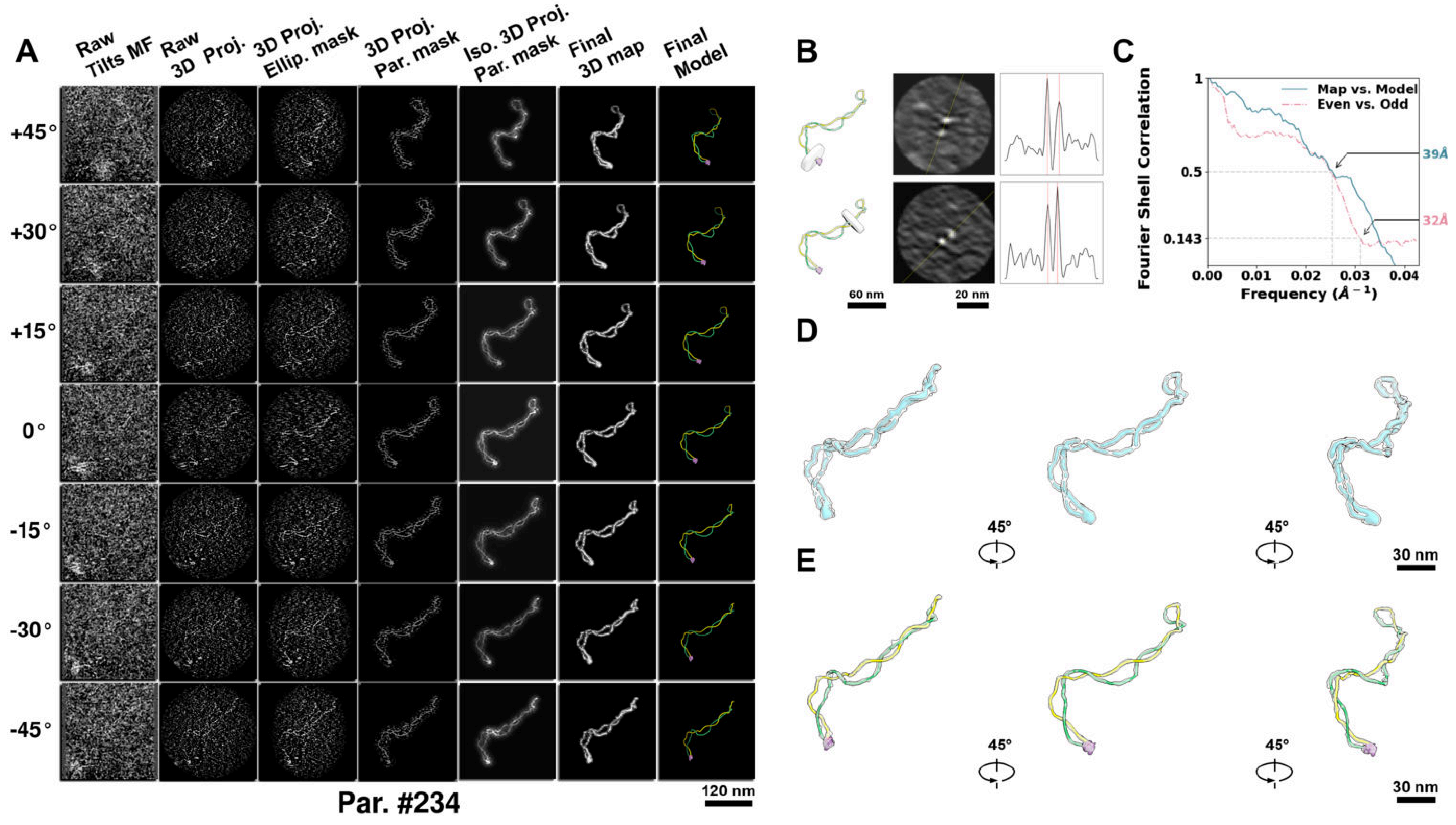

Supplementary Particle Figure 234. Cryo-ET 3D reconstruction of an individual TEC-TopI particle.
(A) 3D reconstruction of the plasmid particle (index no. 234). The first column shows seven representative tilt images from +45° to -45° in step of 15°. The second, third, and fourth columns show 3D projections of the particle with spherical, ellipsoidal (thinner along the z-dimension), and particle-shaped masks, respectively. The fifth column displays the 3D projections of the enhanced and IsoNet missing-wedge-corrected particle. The sixth and seventh columns present the final 3D map and the flexibly fitted model, respectively. (B) Two cross-sectional views (12 nm thickness) of the plasmid density map along its plectoneme axis are shown in the left-middle panel. The intensity profile along the line crossing the two high-density DNA spots is displayed in the right panel. (C) Resolution assessment of the final 3D map using Fourier shell correlation (FSC). Two criteria are shown: FSC between two half-maps reconstructed from even and odd frames (evaluated at 0.143) and FSC between the final 3D map and the fitted model (evaluated at 0.5). (D) Zoomed-in views of the final 3D density map from panel A, displayed at two contour levels. (E) Superimposition of the high-contour level map from panel D onto its fitted model.

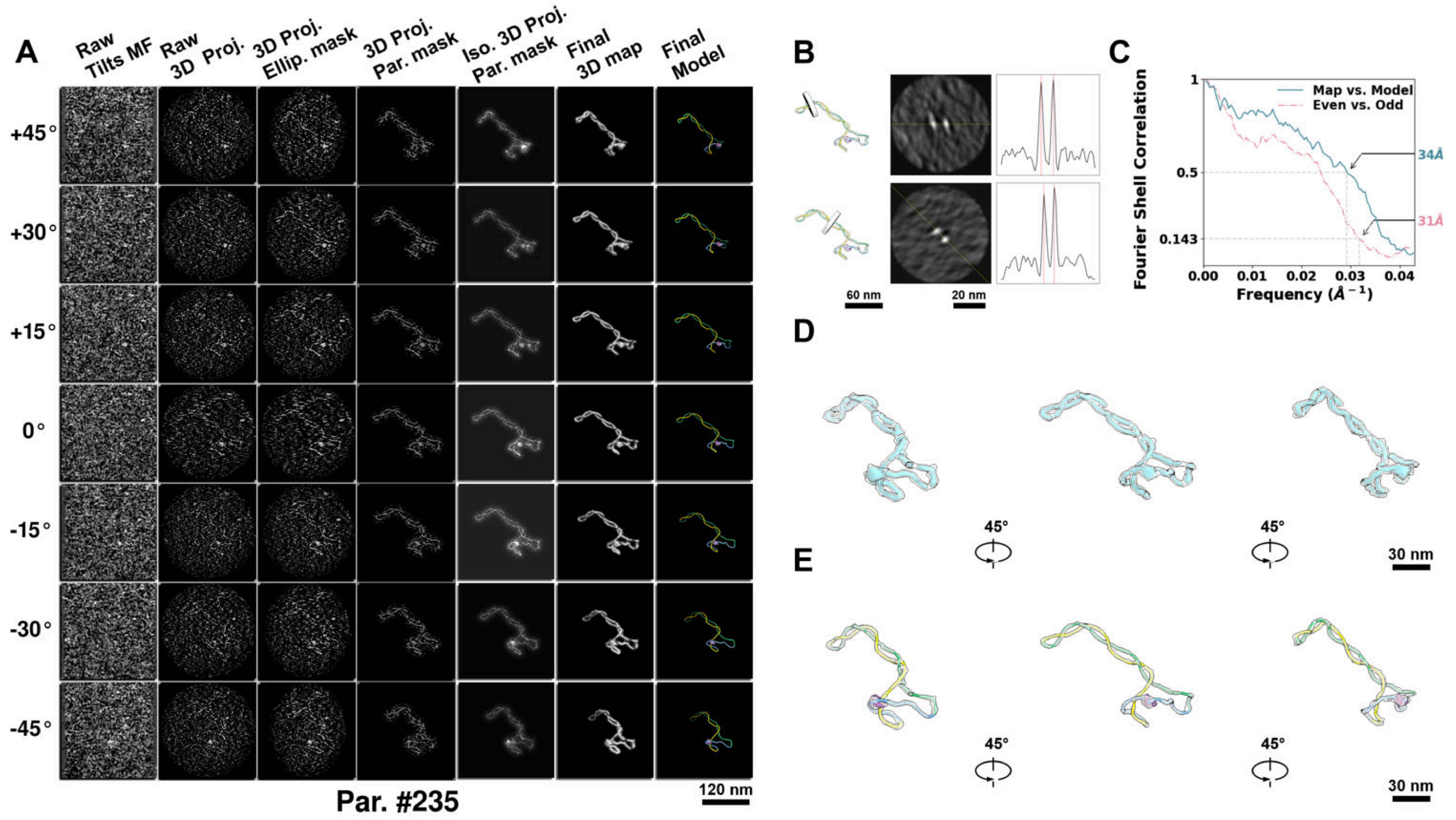

Supplementary Particle Figure 235. Cryo-ET 3D reconstruction of an individual TEC-TopI particle.
(A) 3D reconstruction of the plasmid particle (index no. 235). The first column shows seven representative tilt images from +45° to -45° in step of 15°. The second, third, and fourth columns show 3D projections of the particle with spherical, ellipsoidal (thinner along the z-dimension), and particle-shaped masks, respectively. The fifth column displays the 3D projections of the enhanced and IsoNet missing-wedge-corrected particle. The sixth and seventh columns present the final 3D map and the flexibly fitted model, respectively. (B) Two cross-sectional views (12 nm thickness) of the plasmid density map along its plectoneme axis are shown in the left-middle panel. The intensity profile along the line crossing the two high-density DNA spots is displayed in the right panel. (C) Resolution assessment of the final 3D map using Fourier shell correlation (FSC). Two criteria are shown: FSC between two half-maps reconstructed from even and odd frames (evaluated at 0.143) and FSC between the final 3D map and the fitted model (evaluated at 0.5). (D) Zoomed-in views of the final 3D density map from panel A, displayed at two contour levels. (E) Superimposition of the high-contour level map from panel D onto its fitted model.

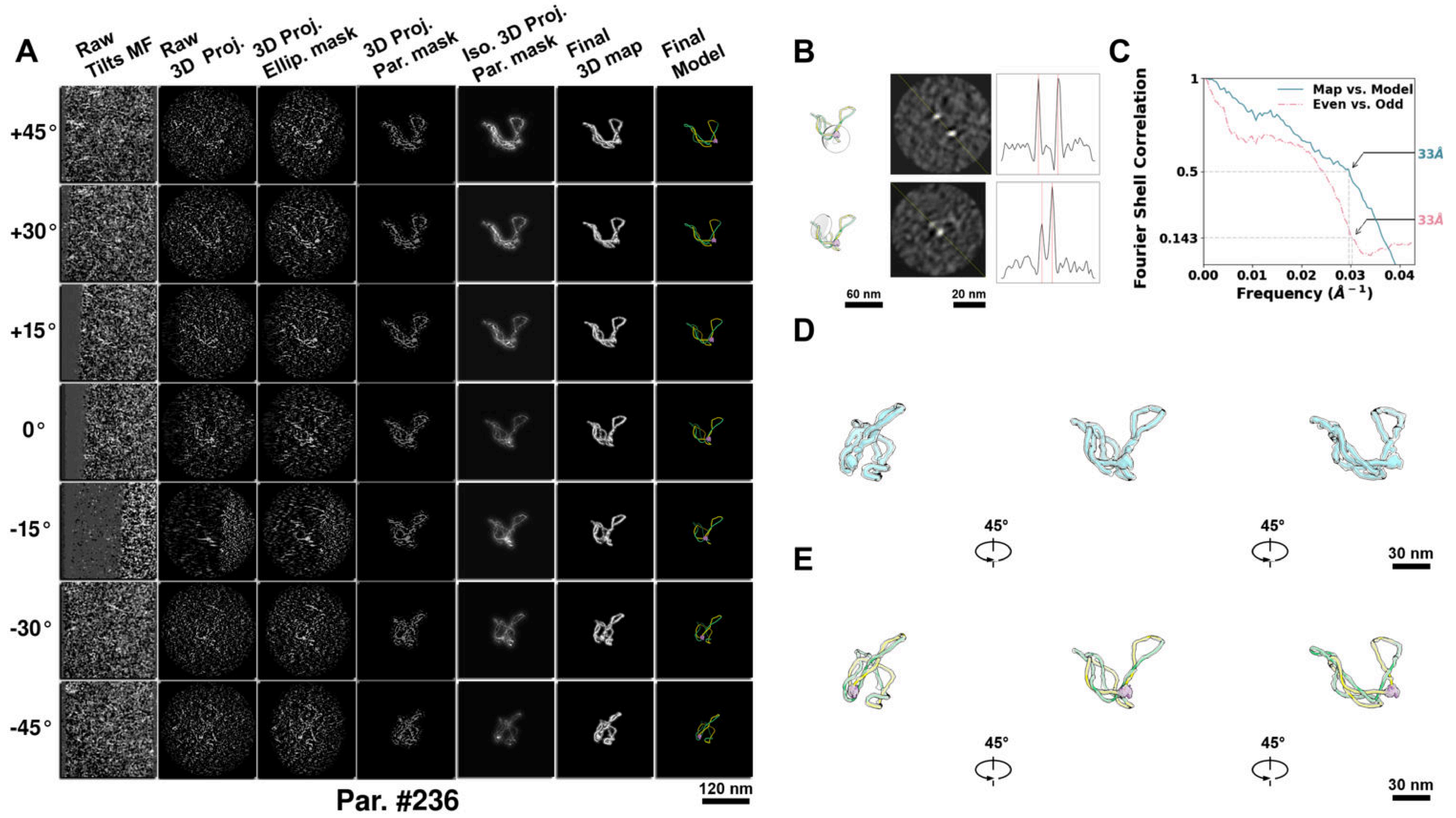

Supplementary Particle Figure 236. Cryo-ET 3D reconstruction of an individual TEC-TopI particle.
(A) 3D reconstruction of the plasmid particle (index no. 236). The first column shows seven representative tilt images from +45° to -45° in step of 15°. The second, third, and fourth columns show 3D projections of the particle with spherical, ellipsoidal (thinner along the z-dimension), and particle-shaped masks, respectively. The fifth column displays the 3D projections of the enhanced and IsoNet missing-wedge-corrected particle. The sixth and seventh columns present the final 3D map and the flexibly fitted model, respectively. (B) Two cross-sectional views (12 nm thickness) of the plasmid density map along its plectoneme axis are shown in the left-middle panel. The intensity profile along the line crossing the two high-density DNA spots is displayed in the right panel. (C) Resolution assessment of the final 3D map using Fourier shell correlation (FSC). Two criteria are shown: FSC between two half-maps reconstructed from even and odd frames (evaluated at 0.143) and FSC between the final 3D map and the fitted model (evaluated at 0.5). (D) Zoomed-in views of the final 3D density map from panel A, displayed at two contour levels. (E) Superimposition of the high-contour level map from panel D onto its fitted model.

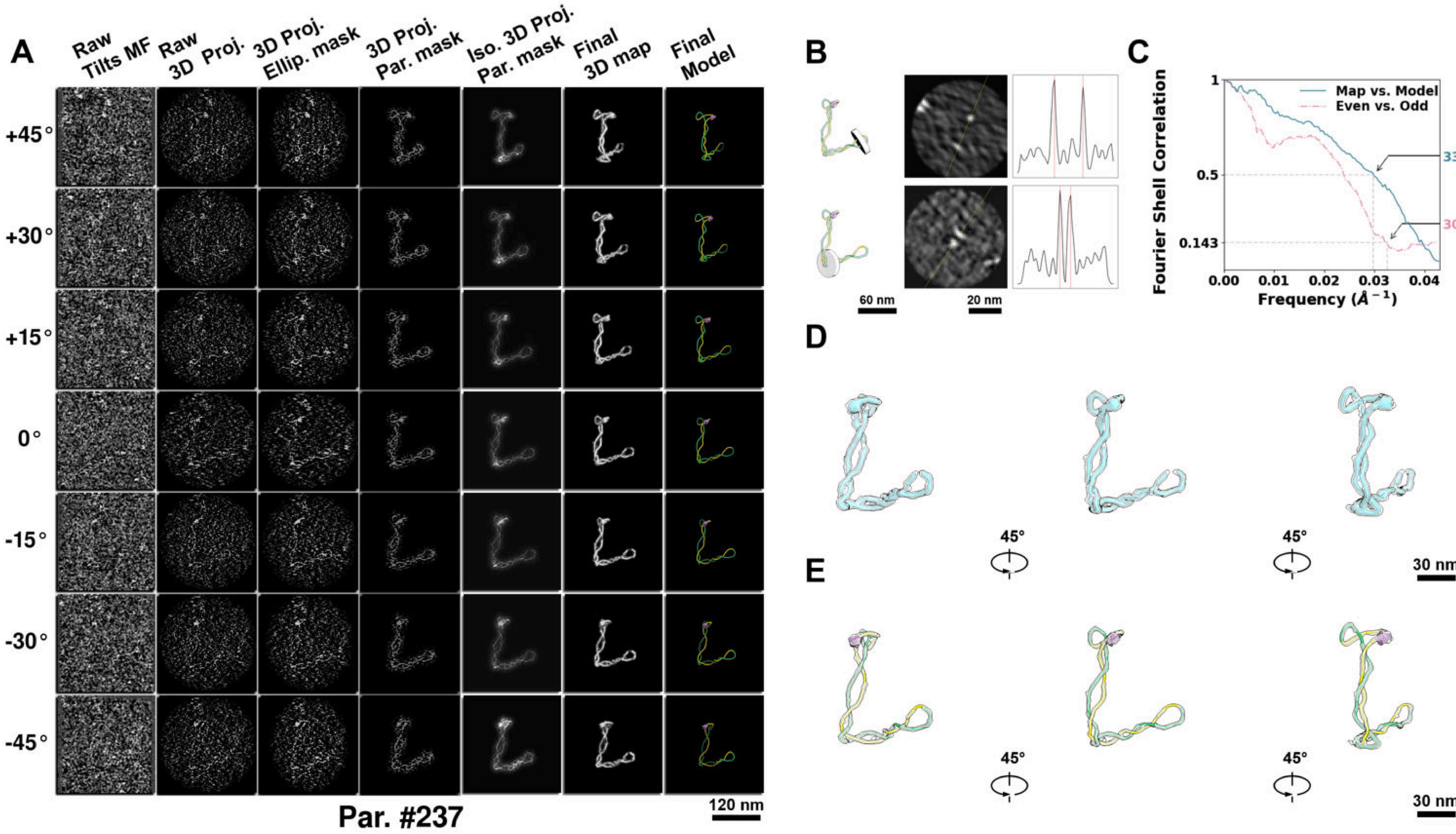

Supplementary Particle Figure 237. Cryo-ET 3D reconstruction of an individual TEC-TopI particle.
(A) 3D reconstruction of the plasmid particle (index no. 237). The first column shows seven representative tilt images from +45° to -45° in step of 15°. The second, third, and fourth columns show 3D projections of the particle with spherical, ellipsoidal (thinner along the z-dimension), and particle-shaped masks, respectively. The fifth column displays the 3D projections of the enhanced and IsoNet missing-wedge-corrected particle. The sixth and seventh columns present the final 3D map and the flexibly fitted model, respectively. (B) Two cross-sectional views (12 nm thickness) of the plasmid density map along its plectoneme axis are shown in the left-middle panel. The intensity profile along the line crossing the two high-density DNA spots is displayed in the right panel. (C) Resolution assessment of the final 3D map using Fourier shell correlation (FSC). Two criteria are shown: FSC between two half-maps reconstructed from even and odd frames (evaluated at 0.143) and FSC between the final 3D map and the fitted model (evaluated at 0.5). (D) Zoomed-in views of the final 3D density map from panel A, displayed at two contour levels. (E) Superimposition of the high-contour level map from panel D onto its fitted model.

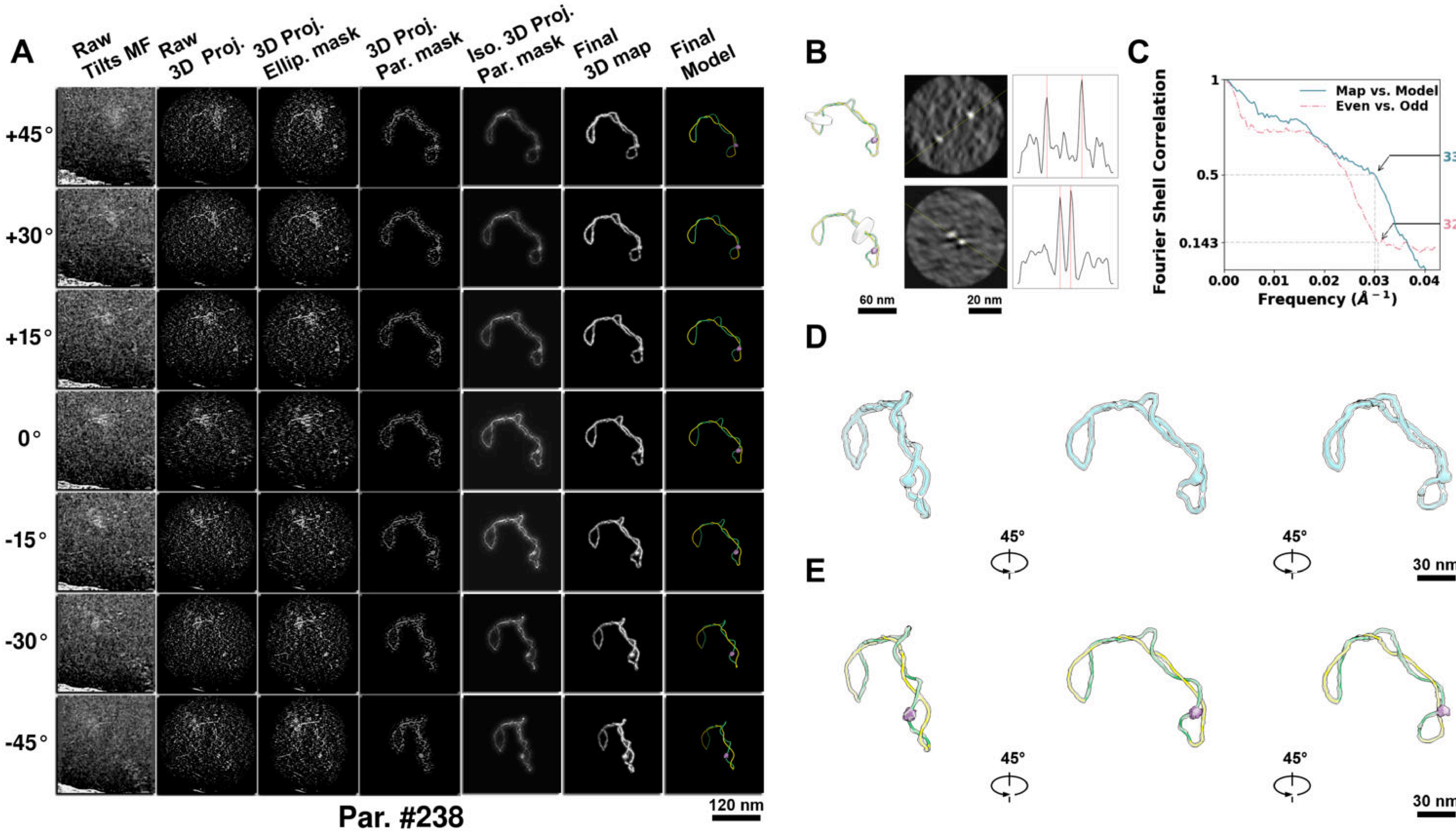

Supplementary Particle Figure 238. Cryo-ET 3D reconstruction of an individual TEC-TopI particle.
(A) 3D reconstruction of the plasmid particle (index no. 238). The first column shows seven representative tilt images from +45° to -45° in step of 15°. The second, third, and fourth columns show 3D projections of the particle with spherical, ellipsoidal (thinner along the z-dimension), and particle-shaped masks, respectively. The fifth column displays the 3D projections of the enhanced and IsoNet missing-wedge-corrected particle. The sixth and seventh columns present the final 3D map and the flexibly fitted model, respectively. (B) Two cross-sectional views (12 nm thickness) of the plasmid density map along its plectoneme axis are shown in the left-middle panel. The intensity profile along the line crossing the two high-density DNA spots is displayed in the right panel. (C) Resolution assessment of the final 3D map using Fourier shell correlation (FSC). Two criteria are shown: FSC between two half-maps reconstructed from even and odd frames (evaluated at 0.143) and FSC between the final 3D map and the fitted model (evaluated at 0.5). (D) Zoomed-in views of the final 3D density map from panel A, displayed at two contour levels. (E) Superimposition of the high-contour level map from panel D onto its fitted model.

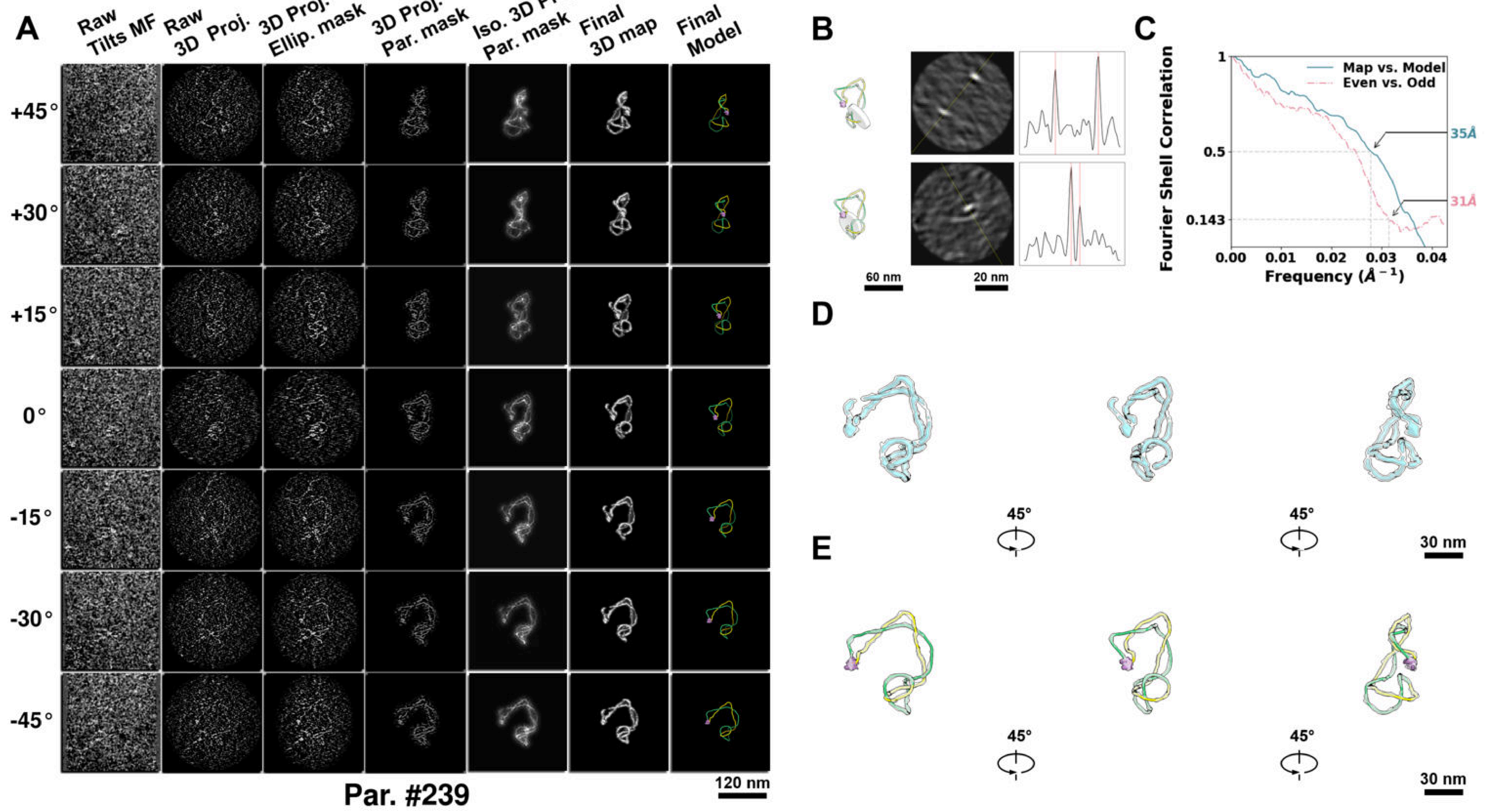

Supplementary Particle Figure 239. Cryo-ET 3D reconstruction of an individual TEC-TopI particle.
(A) 3D reconstruction of the plasmid particle (index no. 239). The first column shows seven representative tilt images from +45° to -45° in step of 15°. The second, third, and fourth columns show 3D projections of the particle with spherical, ellipsoidal (thinner along the z-dimension), and particle-shaped masks, respectively. The fifth column displays the 3D projections of the enhanced and IsoNet missing-wedge-corrected particle. The sixth and seventh columns present the final 3D map and the flexibly fitted model, respectively. (B) Two cross-sectional views (12 nm thickness) of the plasmid density map along its plectoneme axis are shown in the left-middle panel. The intensity profile along the line crossing the two high-density DNA spots is displayed in the right panel. (C) Resolution assessment of the final 3D map using Fourier shell correlation (FSC). Two criteria are shown: FSC between two half-maps reconstructed from even and odd frames (evaluated at 0.143) and FSC between the final 3D map and the fitted model (evaluated at 0.5). (D) Zoomed-in views of the final 3D density map from panel A, displayed at two contour levels. (E) Superimposition of the high-contour level map from panel D onto its fitted model.

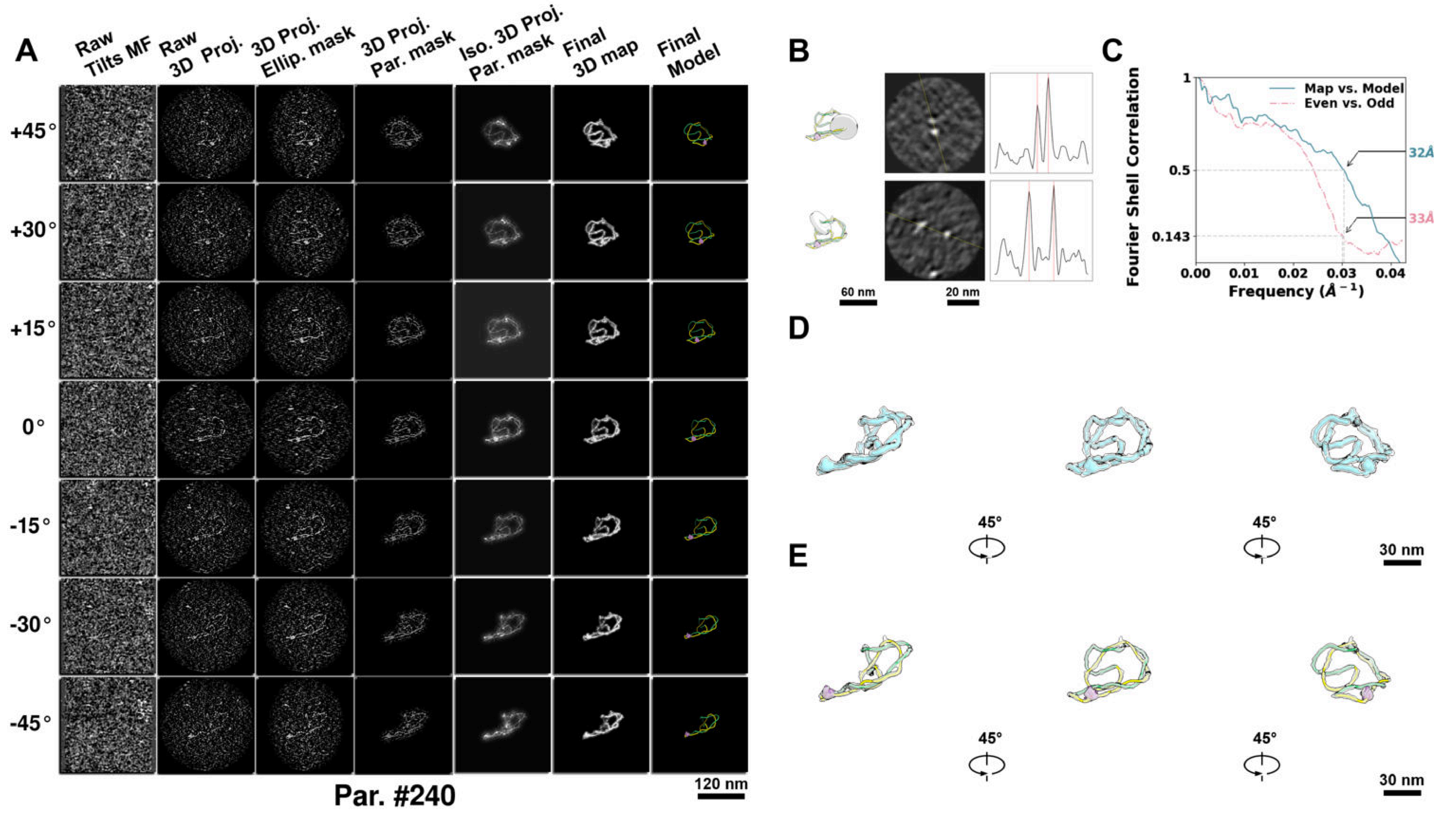

Supplementary Particle Figure 240. Cryo-ET 3D reconstruction of an individual TEC-TopI particle.
(A) 3D reconstruction of the plasmid particle (index no. 240). The first column shows seven representative tilt images from +45° to -45° in step of 15°. The second, third, and fourth columns show 3D projections of the particle with spherical, ellipsoidal (thinner along the z-dimension), and particle-shaped masks, respectively. The fifth column displays the 3D projections of the enhanced and IsoNet missing-wedge-corrected particle. The sixth and seventh columns present the final 3D map and the flexibly fitted model, respectively. (B) Two cross-sectional views (12 nm thickness) of the plasmid density map along its plectoneme axis are shown in the left-middle panel. The intensity profile along the line crossing the two high-density DNA spots is displayed in the right panel. (C) Resolution assessment of the final 3D map using Fourier shell correlation (FSC). Two criteria are shown: FSC between two half-maps reconstructed from even and odd frames (evaluated at 0.143) and FSC between the final 3D map and the fitted model (evaluated at 0.5). (D) Zoomed-in views of the final 3D density map from panel A, displayed at two contour levels. (E) Superimposition of the high-contour level map from panel D onto its fitted model.

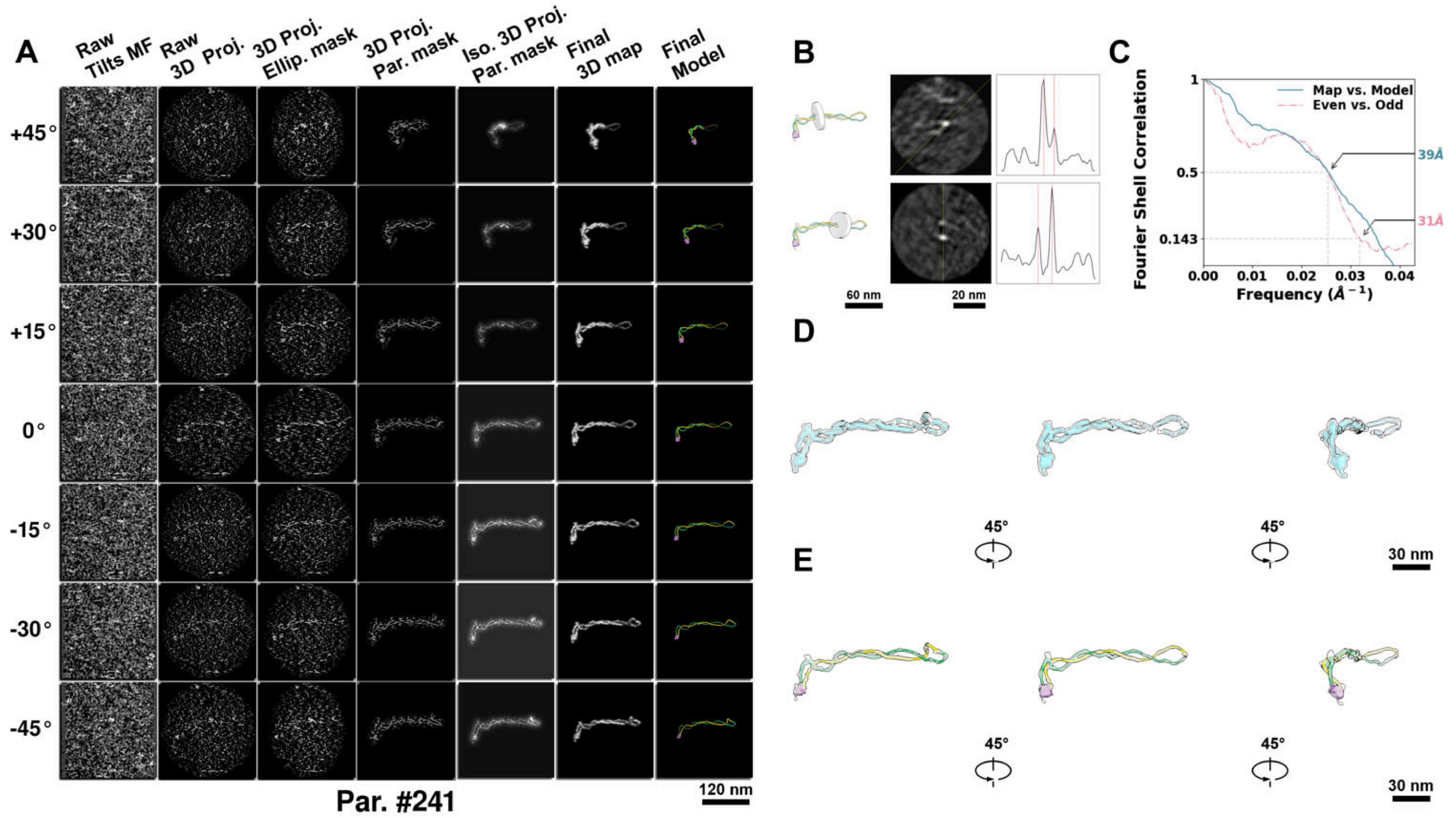

Supplementary Particle Figure 241. Cryo-ET 3D reconstruction of an individual TEC-TopI particle.
(A) 3D reconstruction of the plasmid particle (index no. 241). The first column shows seven representative tilt images from +45° to -45° in step of 15°. The second, third, and fourth columns show 3D projections of the particle with spherical, ellipsoidal (thinner along the z-dimension), and particle-shaped masks, respectively. The fifth column displays the 3D projections of the enhanced and IsoNet missing-wedge-corrected particle. The sixth and seventh columns present the final 3D map and the flexibly fitted model, respectively. (B) Two cross-sectional views (12 nm thickness) of the plasmid density map along its plectoneme axis are shown in the left-middle panel. The intensity profile along the line crossing the two high-density DNA spots is displayed in the right panel. (C) Resolution assessment of the final 3D map using Fourier shell correlation (FSC). Two criteria are shown: FSC between two half-maps reconstructed from even and odd frames (evaluated at 0.143) and FSC between the final 3D map and the fitted model (evaluated at 0.5). (D) Zoomed-in views of the final 3D density map from panel A, displayed at two contour levels. (E) Superimposition of the high-contour level map from panel D onto its fitted model.

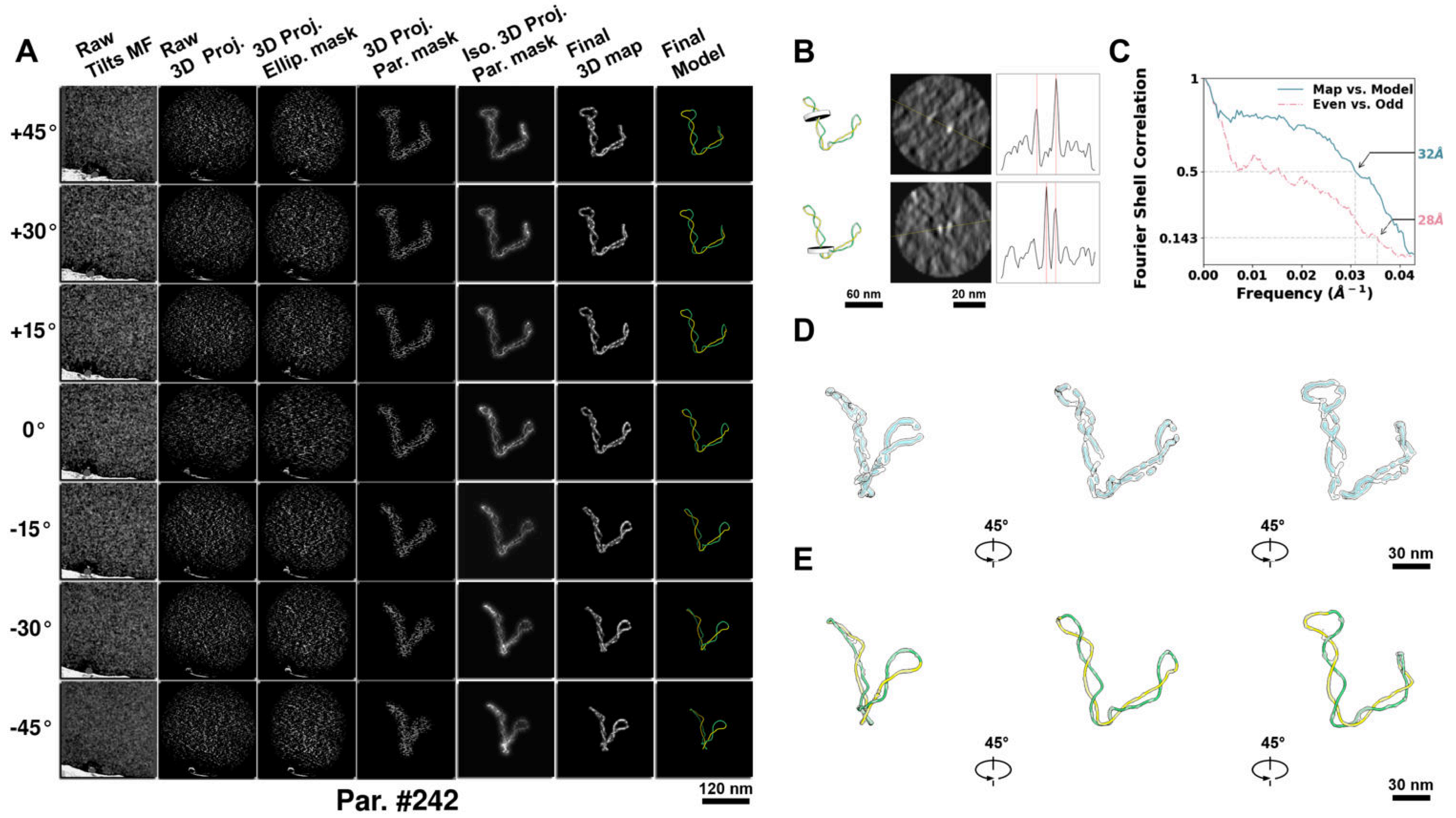

Supplementary Particle Figure 242. Cryo-ET 3D reconstruction of an individual TEC-TopI particle.
(A) 3D reconstruction of the plasmid particle (index no. 242). The first column shows seven representative tilt images from +45° to -45° in step of 15°. The second, third, and fourth columns show 3D projections of the particle with spherical, ellipsoidal (thinner along the z-dimension), and particle-shaped masks, respectively. The fifth column displays the 3D projections of the enhanced and IsoNet missing-wedge-corrected particle. The sixth and seventh columns present the final 3D map and the flexibly fitted model, respectively. (B) Two cross-sectional views (12 nm thickness) of the plasmid density map along its plectoneme axis are shown in the left-middle panel. The intensity profile along the line crossing the two high-density DNA spots is displayed in the right panel. (C) Resolution assessment of the final 3D map using Fourier shell correlation (FSC). Two criteria are shown: FSC between two half-maps reconstructed from even and odd frames (evaluated at 0.143) and FSC between the final 3D map and the fitted model (evaluated at 0.5). (D) Zoomed-in views of the final 3D density map from panel A, displayed at two contour levels. (E) Superimposition of the high-contour level map from panel D onto its fitted model.

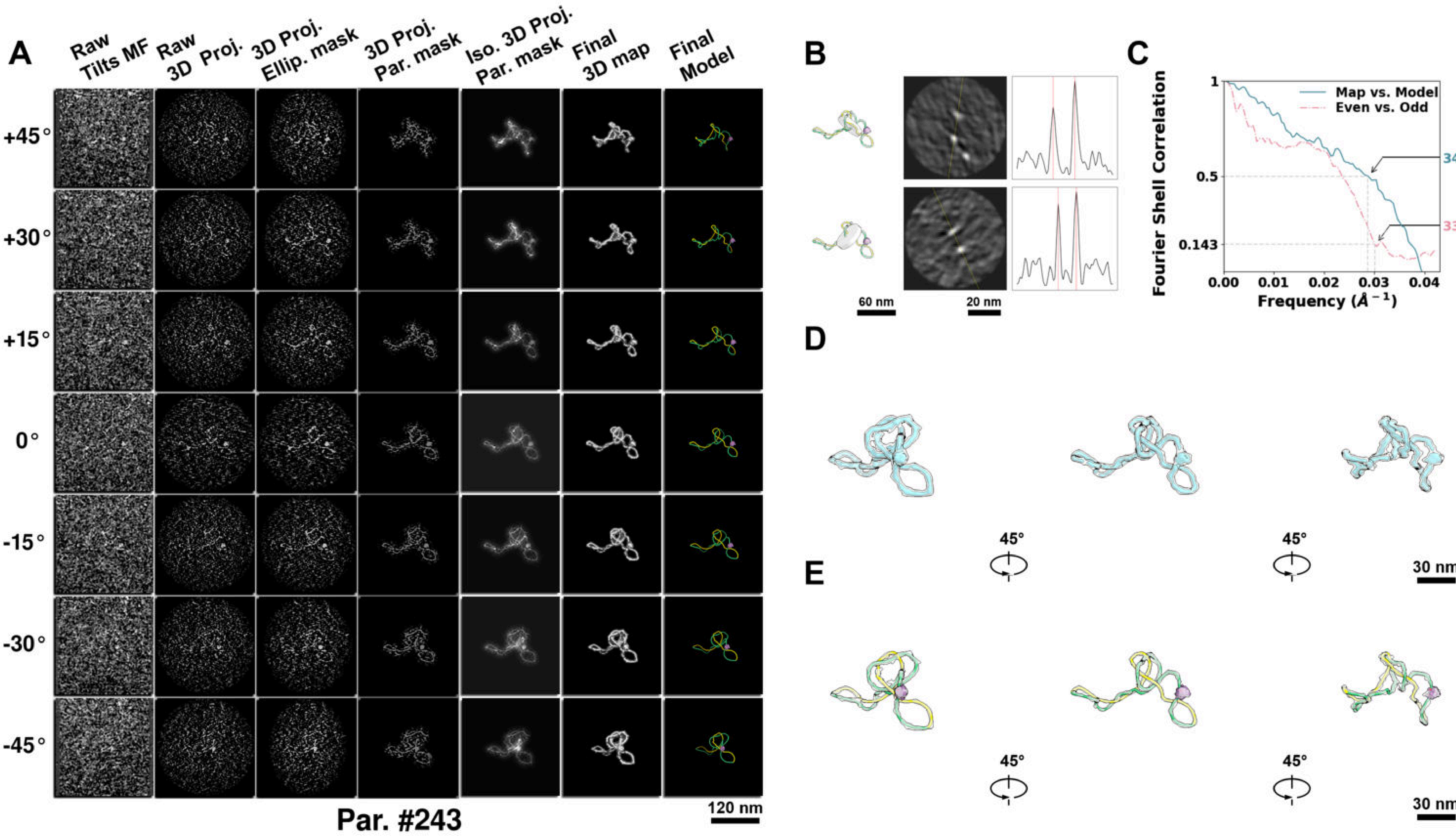

Supplementary Particle Figure 243. Cryo-ET 3D reconstruction of an individual TEC-TopI particle.
(A) 3D reconstruction of the plasmid particle (index no. 243). The first column shows seven representative tilt images from +45° to -45° in step of 15°. The second, third, and fourth columns show 3D projections of the particle with spherical, ellipsoidal (thinner along the z-dimension), and particle-shaped masks, respectively. The fifth column displays the 3D projections of the enhanced and IsoNet missing-wedge-corrected particle. The sixth and seventh columns present the final 3D map and the flexibly fitted model, respectively. (B) Two cross-sectional views (12 nm thickness) of the plasmid density map along its plectoneme axis are shown in the left-middle panel. The intensity profile along the line crossing the two high-density DNA spots is displayed in the right panel. (C) Resolution assessment of the final 3D map using Fourier shell correlation (FSC). Two criteria are shown: FSC between two half-maps reconstructed from even and odd frames (evaluated at 0.143) and FSC between the final 3D map and the fitted model (evaluated at 0.5). (D) Zoomed-in views of the final 3D density map from panel A, displayed at two contour levels. (E) Superimposition of the high-contour level map from panel D onto its fitted model.

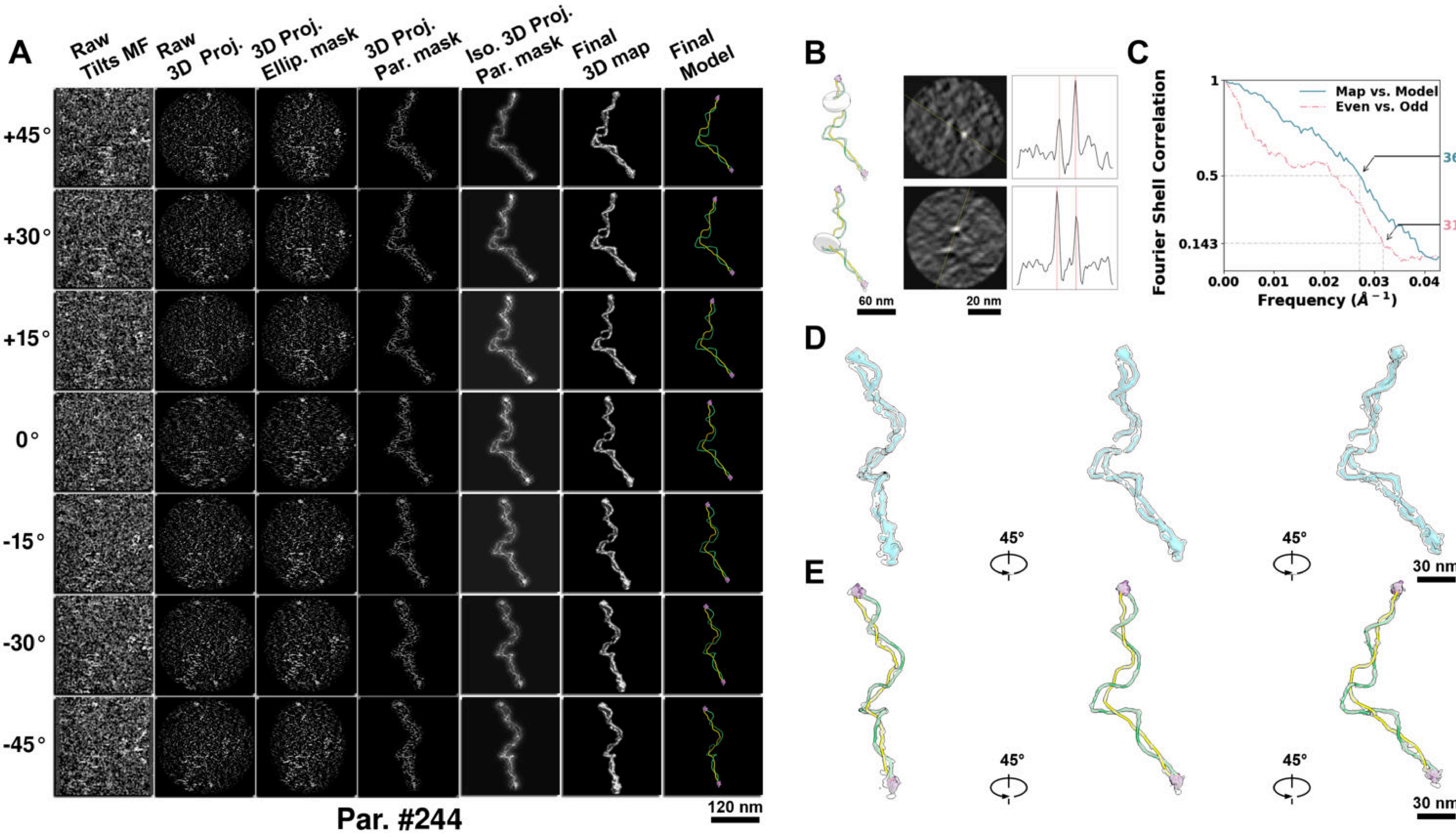

Supplementary Particle Figure 244. Cryo-ET 3D reconstruction of an individual TEC-TopI particle.
(A) 3D reconstruction of the plasmid particle (index no. 244). The first column shows seven representative tilt images from +45° to -45° in step of 15°. The second, third, and fourth columns show 3D projections of the particle with spherical, ellipsoidal (thinner along the z-dimension), and particle-shaped masks, respectively. The fifth column displays the 3D projections of the enhanced and IsoNet missing-wedge-corrected particle. The sixth and seventh columns present the final 3D map and the flexibly fitted model, respectively. (B) Two cross-sectional views (12 nm thickness) of the plasmid density map along its plectoneme axis are shown in the left-middle panel. The intensity profile along the line crossing the two high-density DNA spots is displayed in the right panel. (C) Resolution assessment of the final 3D map using Fourier shell correlation (FSC). Two criteria are shown: FSC between two half-maps reconstructed from even and odd frames (evaluated at 0.143) and FSC between the final 3D map and the fitted model (evaluated at 0.5). (D) Zoomed-in views of the final 3D density map from panel A, displayed at two contour levels. (E) Superimposition of the high-contour level map from panel D onto its fitted model.

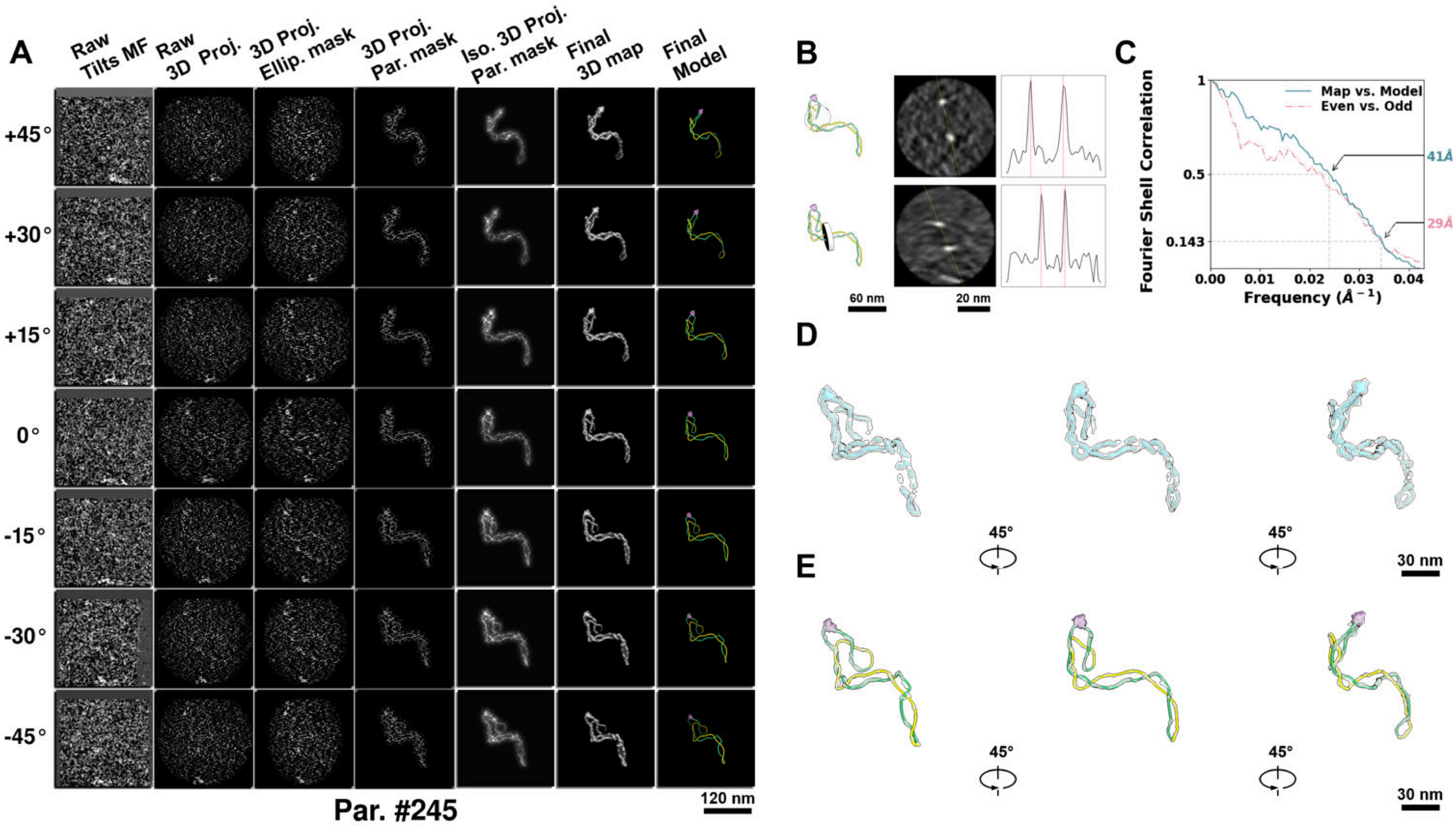

Supplementary Particle Figure 245. Cryo-ET 3D reconstruction of an individual TEC-TopI particle.
(A) 3D reconstruction of the plasmid particle (index no. 245). The first column shows seven representative tilt images from +45° to -45° in step of 15°. The second, third, and fourth columns show 3D projections of the particle with spherical, ellipsoidal (thinner along the z-dimension), and particle-shaped masks, respectively. The fifth column displays the 3D projections of the enhanced and IsoNet missing-wedge-corrected particle. The sixth and seventh columns present the final 3D map and the flexibly fitted model, respectively. (B) Two cross-sectional views (12 nm thickness) of the plasmid density map along its plectoneme axis are shown in the left-middle panel. The intensity profile along the line crossing the two high-density DNA spots is displayed in the right panel. (C) Resolution assessment of the final 3D map using Fourier shell correlation (FSC). Two criteria are shown: FSC between two half-maps reconstructed from even and odd frames (evaluated at 0.143) and FSC between the final 3D map and the fitted model (evaluated at 0.5). (D) Zoomed-in views of the final 3D density map from panel A, displayed at two contour levels. (E) Superimposition of the high-contour level map from panel D onto its fitted model.

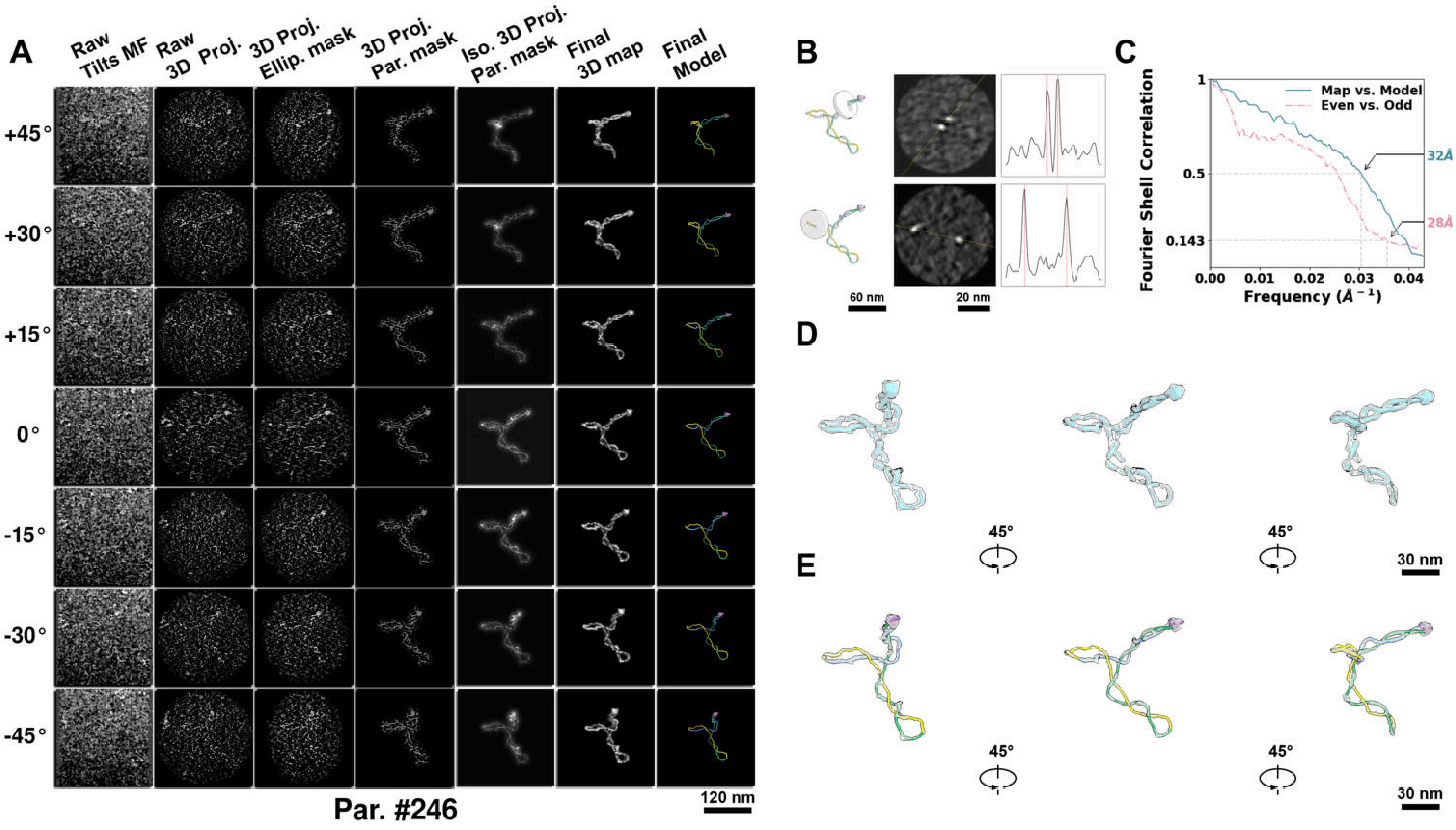

Supplementary Particle Figure 246. Cryo-ET 3D reconstruction of an individual TEC-TopI particle.
(A) 3D reconstruction of the plasmid particle (index no. 246). The first column shows seven representative tilt images from +45° to -45° in step of 15°. The second, third, and fourth columns show 3D projections of the particle with spherical, ellipsoidal (thinner along the z-dimension), and particle-shaped masks, respectively. The fifth column displays the 3D projections of the enhanced and IsoNet missing-wedge-corrected particle. The sixth and seventh columns present the final 3D map and the flexibly fitted model, respectively. (B) Two cross-sectional views (12 nm thickness) of the plasmid density map along its plectoneme axis are shown in the left-middle panel. The intensity profile along the line crossing the two high-density DNA spots is displayed in the right panel. (C) Resolution assessment of the final 3D map using Fourier shell correlation (FSC). Two criteria are shown: FSC between two half-maps reconstructed from even and odd frames (evaluated at 0.143) and FSC between the final 3D map and the fitted model (evaluated at 0.5). (D) Zoomed-in views of the final 3D density map from panel A, displayed at two contour levels. (E) Superimposition of the high-contour level map from panel D onto its fitted model.

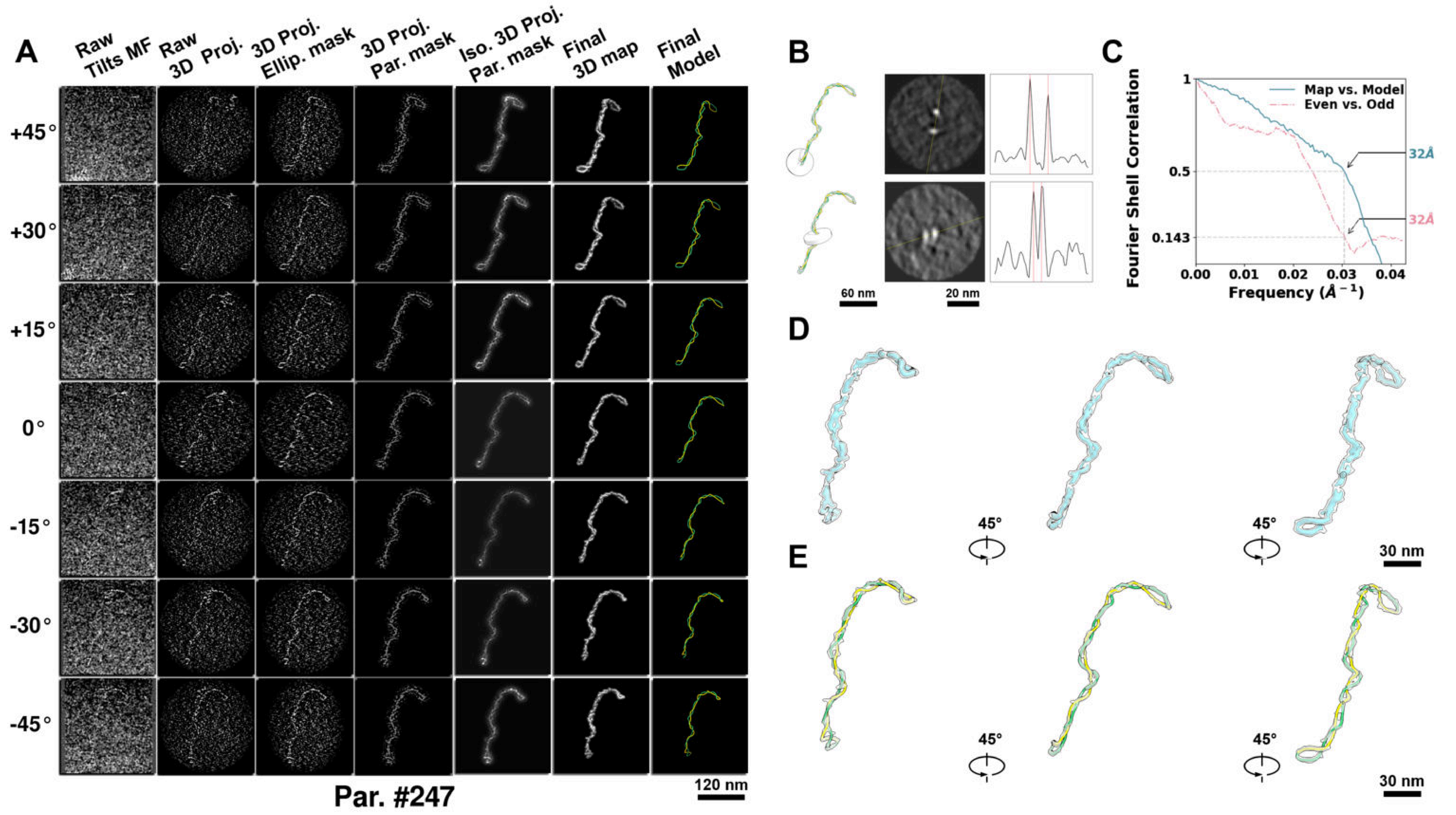

Supplementary Particle Figure 247. Cryo-ET 3D reconstruction of an individual TEC-TopI particle.
(A) 3D reconstruction of the plasmid particle (index no. 247). The first column shows seven representative tilt images from +45° to -45° in step of 15°. The second, third, and fourth columns show 3D projections of the particle with spherical, ellipsoidal (thinner along the z-dimension), and particle-shaped masks, respectively. The fifth column displays the 3D projections of the enhanced and IsoNet missing-wedge-corrected particle. The sixth and seventh columns present the final 3D map and the flexibly fitted model, respectively. (B) Two cross-sectional views (12 nm thickness) of the plasmid density map along its plectoneme axis are shown in the left-middle panel. The intensity profile along the line crossing the two high-density DNA spots is displayed in the right panel. (C) Resolution assessment of the final 3D map using Fourier shell correlation (FSC). Two criteria are shown: FSC between two half-maps reconstructed from even and odd frames (evaluated at 0.143) and FSC between the final 3D map and the fitted model (evaluated at 0.5). (D) Zoomed-in views of the final 3D density map from panel A, displayed at two contour levels. (E) Superimposition of the high-contour level map from panel D onto its fitted model.

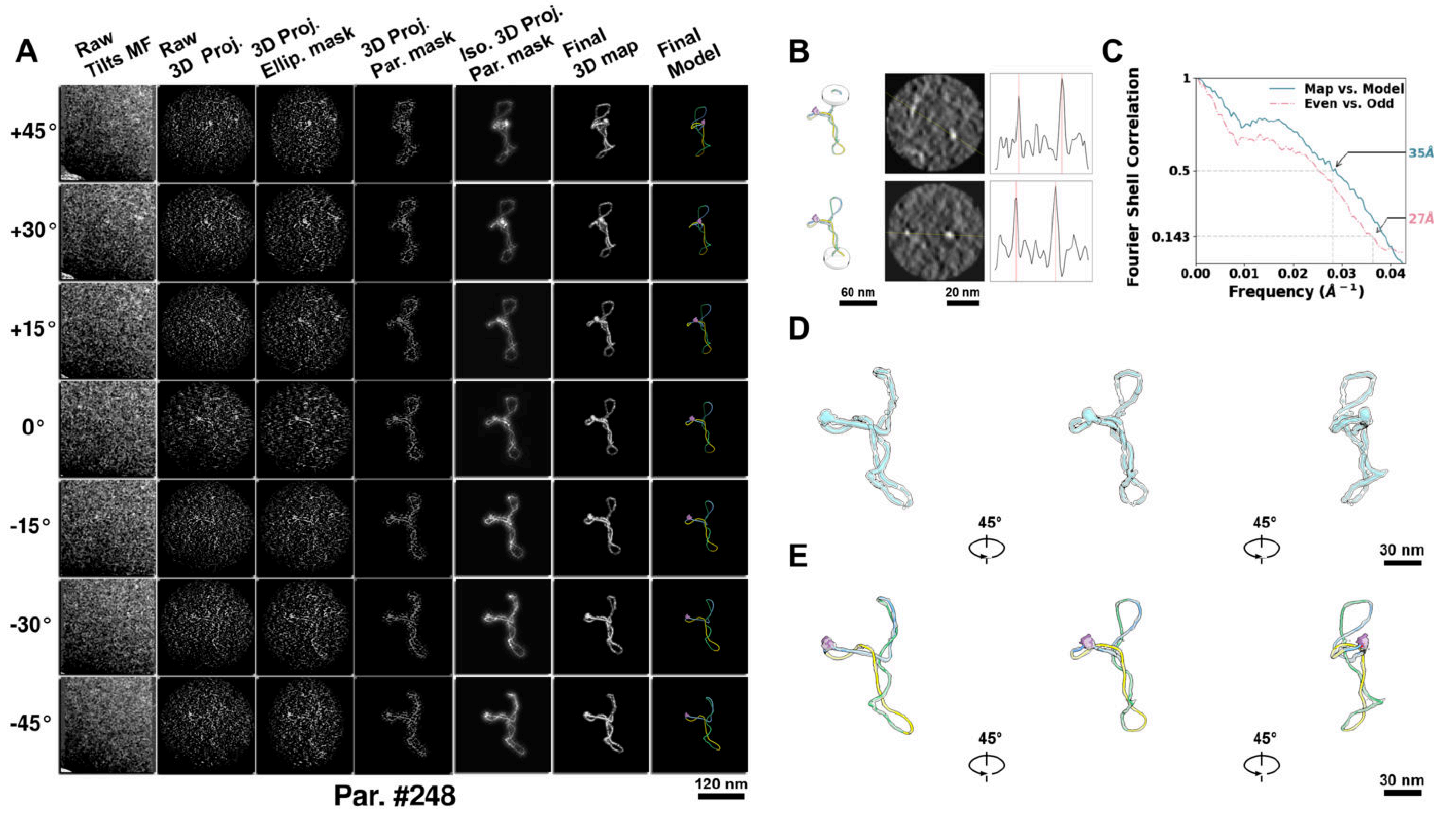

Supplementary Particle Figure 248. Cryo-ET 3D reconstruction of an individual TEC-TopI particle.
(A) 3D reconstruction of the plasmid particle (index no. 248). The first column shows seven representative tilt images from +45° to -45° in step of 15°. The second, third, and fourth columns show 3D projections of the particle with spherical, ellipsoidal (thinner along the z-dimension), and particle-shaped masks, respectively. The fifth column displays the 3D projections of the enhanced and IsoNet missing-wedge-corrected particle. The sixth and seventh columns present the final 3D map and the flexibly fitted model, respectively. (B) Two cross-sectional views (12 nm thickness) of the plasmid density map along its plectoneme axis are shown in the left-middle panel. The intensity profile along the line crossing the two high-density DNA spots is displayed in the right panel. (C) Resolution assessment of the final 3D map using Fourier shell correlation (FSC). Two criteria are shown: FSC between two half-maps reconstructed from even and odd frames (evaluated at 0.143) and FSC between the final 3D map and the fitted model (evaluated at 0.5). (D) Zoomed-in views of the final 3D density map from panel A, displayed at two contour levels. (E) Superimposition of the high-contour level map from panel D onto its fitted model.

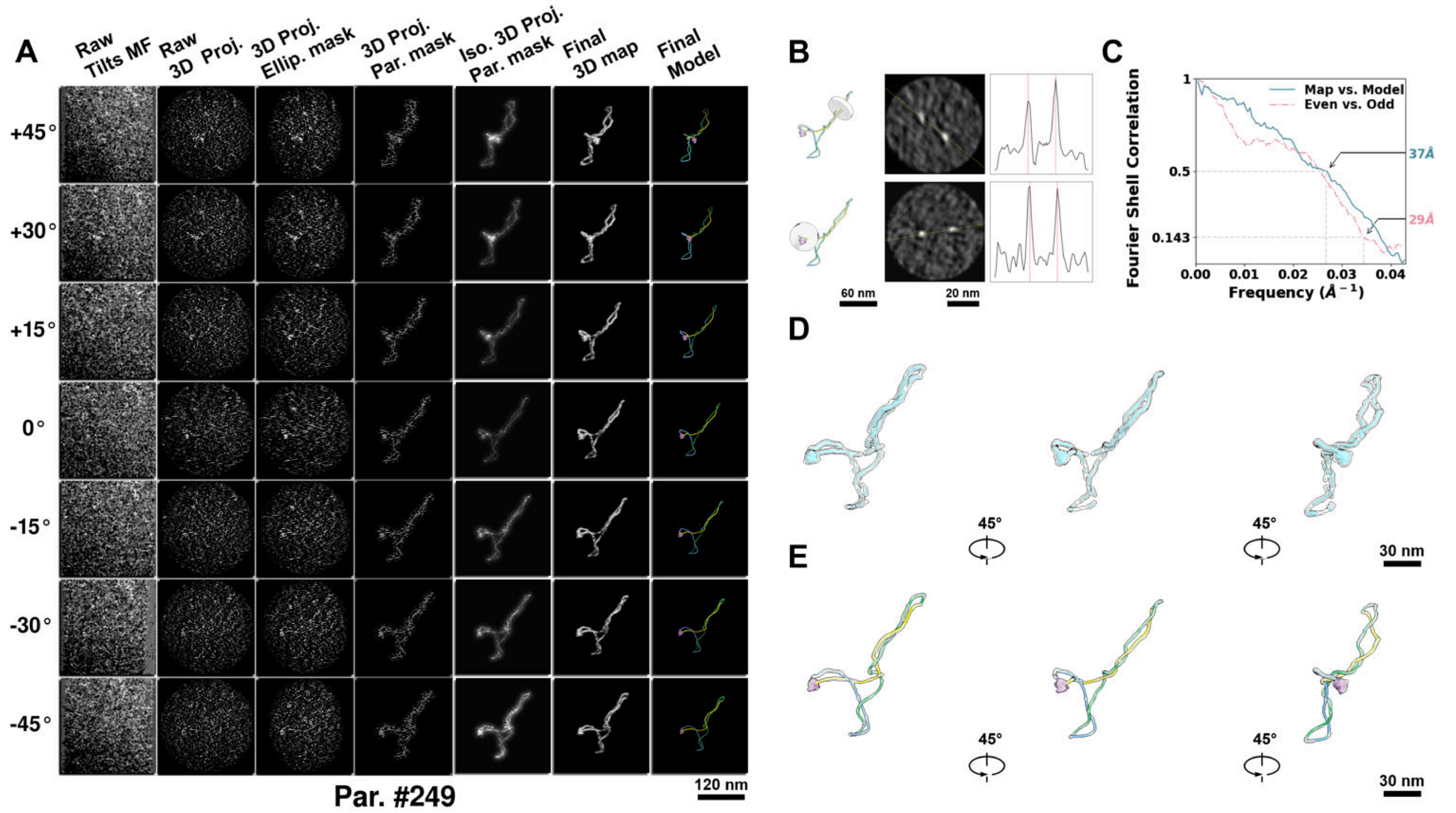

Supplementary Particle Figure 249. Cryo-ET 3D reconstruction of an individual TEC-TopI particle.
(A) 3D reconstruction of the plasmid particle (index no. 249). The first column shows seven representative tilt images from +45° to -45° in step of 15°. The second, third, and fourth columns show 3D projections of the particle with spherical, ellipsoidal (thinner along the z-dimension), and particle-shaped masks, respectively. The fifth column displays the 3D projections of the enhanced and IsoNet missing-wedge-corrected particle. The sixth and seventh columns present the final 3D map and the flexibly fitted model, respectively. (B) Two cross-sectional views (12 nm thickness) of the plasmid density map along its plectoneme axis are shown in the left-middle panel. The intensity profile along the line crossing the two high-density DNA spots is displayed in the right panel. (C) Resolution assessment of the final 3D map using Fourier shell correlation (FSC). Two criteria are shown: FSC between two half-maps reconstructed from even and odd frames (evaluated at 0.143) and FSC between the final 3D map and the fitted model (evaluated at 0.5). (D) Zoomed-in views of the final 3D density map from panel A, displayed at two contour levels. (E) Superimposition of the high-contour level map from panel D onto its fitted model.

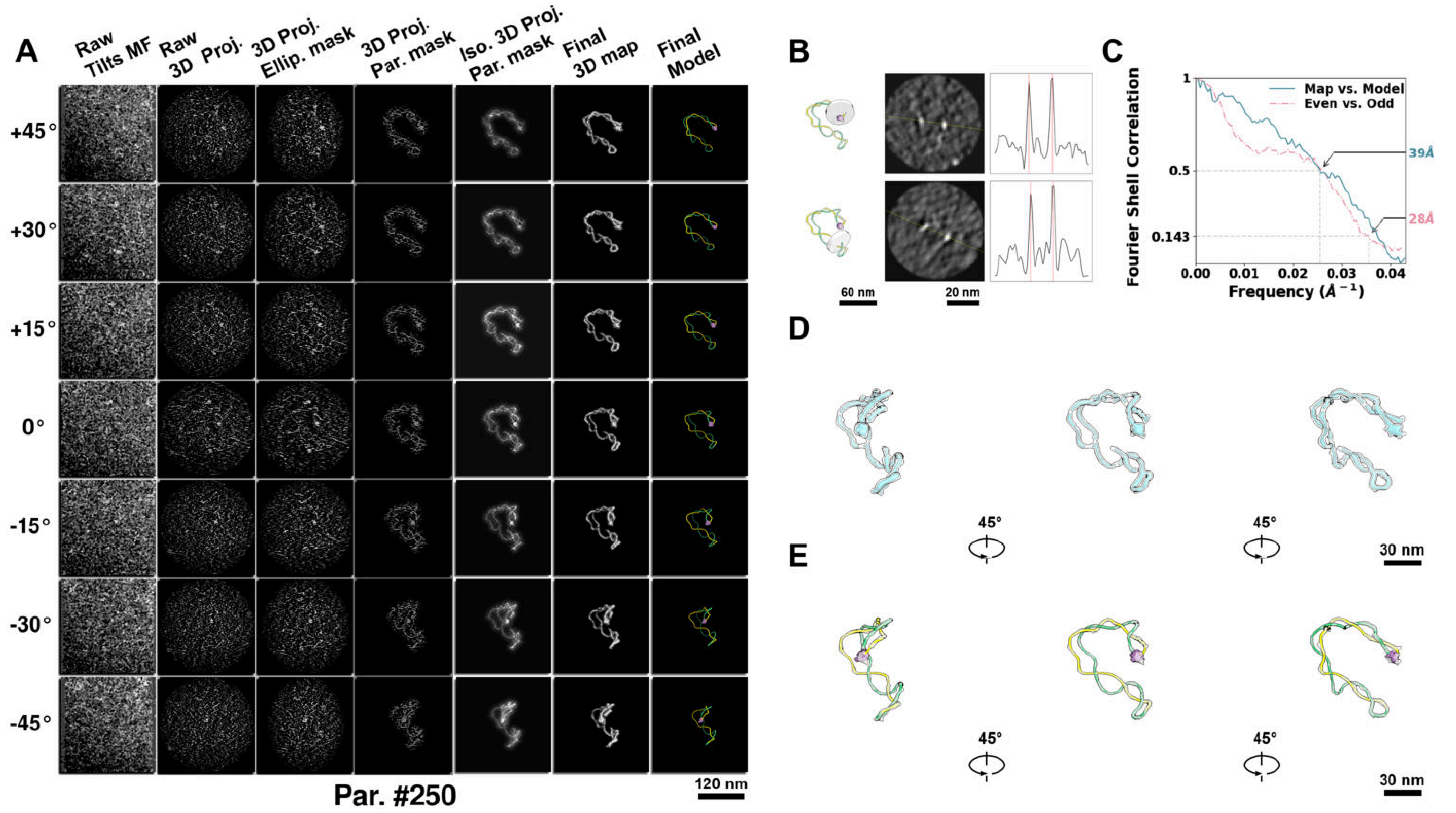

Supplementary Particle Figure 250. Cryo-ET 3D reconstruction of an individual TEC-TopI particle.
(A) 3D reconstruction of the plasmid particle (index no. 250). The first column shows seven representative tilt images from +45° to -45° in step of 15°. The second, third, and fourth columns show 3D projections of the particle with spherical, ellipsoidal (thinner along the z-dimension), and particle-shaped masks, respectively. The fifth column displays the 3D projections of the enhanced and IsoNet missing-wedge-corrected particle. The sixth and seventh columns present the final 3D map and the flexibly fitted model, respectively. (B) Two cross-sectional views (12 nm thickness) of the plasmid density map along its plectoneme axis are shown in the left-middle panel. The intensity profile along the line crossing the two high-density DNA spots is displayed in the right panel. (C) Resolution assessment of the final 3D map using Fourier shell correlation (FSC). Two criteria are shown: FSC between two half-maps reconstructed from even and odd frames (evaluated at 0.143) and FSC between the final 3D map and the fitted model (evaluated at 0.5). (D) Zoomed-in views of the final 3D density map from panel A, displayed at two contour levels. (E) Superimposition of the high-contour level map from panel D onto its fitted model.

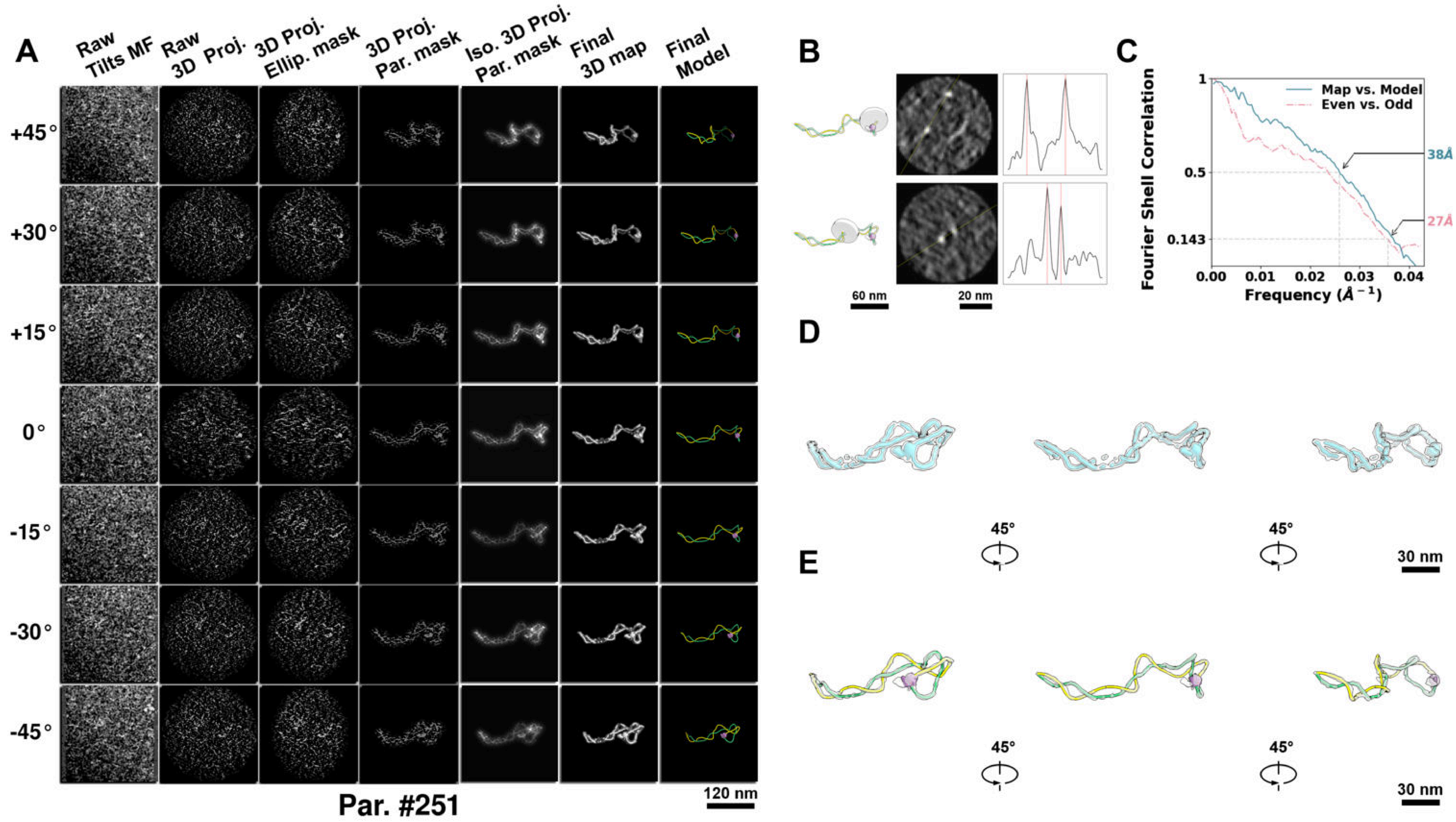

Supplementary Particle Figure 251. Cryo-ET 3D reconstruction of an individual TEC-TopI particle.
(A) 3D reconstruction of the plasmid particle (index no. 251). The first column shows seven representative tilt images from +45° to -45° in step of 15°. The second, third, and fourth columns show 3D projections of the particle with spherical, ellipsoidal (thinner along the z-dimension), and particle-shaped masks, respectively. The fifth column displays the 3D projections of the enhanced and IsoNet missing-wedge-corrected particle. The sixth and seventh columns present the final 3D map and the flexibly fitted model, respectively. (B) Two cross-sectional views (12 nm thickness) of the plasmid density map along its plectoneme axis are shown in the left-middle panel. The intensity profile along the line crossing the two high-density DNA spots is displayed in the right panel. (C) Resolution assessment of the final 3D map using Fourier shell correlation (FSC). Two criteria are shown: FSC between two half-maps reconstructed from even and odd frames (evaluated at 0.143) and FSC between the final 3D map and the fitted model (evaluated at 0.5). (D) Zoomed-in views of the final 3D density map from panel A, displayed at two contour levels. (E) Superimposition of the high-contour level map from panel D onto its fitted model.

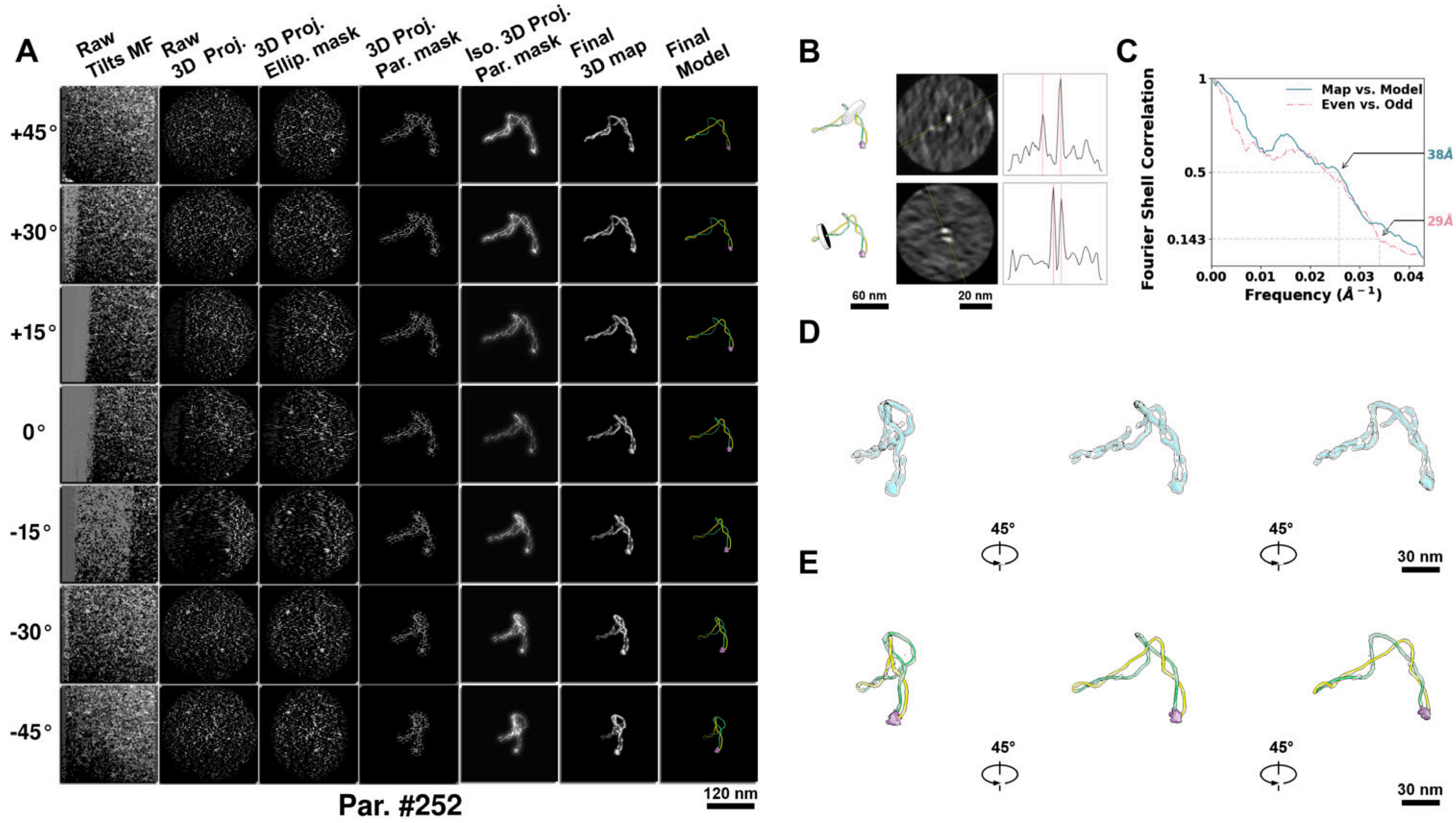

Supplementary Particle Figure 252. Cryo-ET 3D reconstruction of an individual TEC-TopI particle.
(A) 3D reconstruction of the plasmid particle (index no. 252). The first column shows seven representative tilt images from +45° to -45° in step of 15°. The second, third, and fourth columns show 3D projections of the particle with spherical, ellipsoidal (thinner along the z-dimension), and particle-shaped masks, respectively. The fifth column displays the 3D projections of the enhanced and IsoNet missing-wedge-corrected particle. The sixth and seventh columns present the final 3D map and the flexibly fitted model, respectively. (B) Two cross-sectional views (12 nm thickness) of the plasmid density map along its plectoneme axis are shown in the left-middle panel. The intensity profile along the line crossing the two high-density DNA spots is displayed in the right panel. (C) Resolution assessment of the final 3D map using Fourier shell correlation (FSC). Two criteria are shown: FSC between two half-maps reconstructed from even and odd frames (evaluated at 0.143) and FSC between the final 3D map and the fitted model (evaluated at 0.5). (D) Zoomed-in views of the final 3D density map from panel A, displayed at two contour levels. (E) Superimposition of the high-contour level map from panel D onto its fitted model.

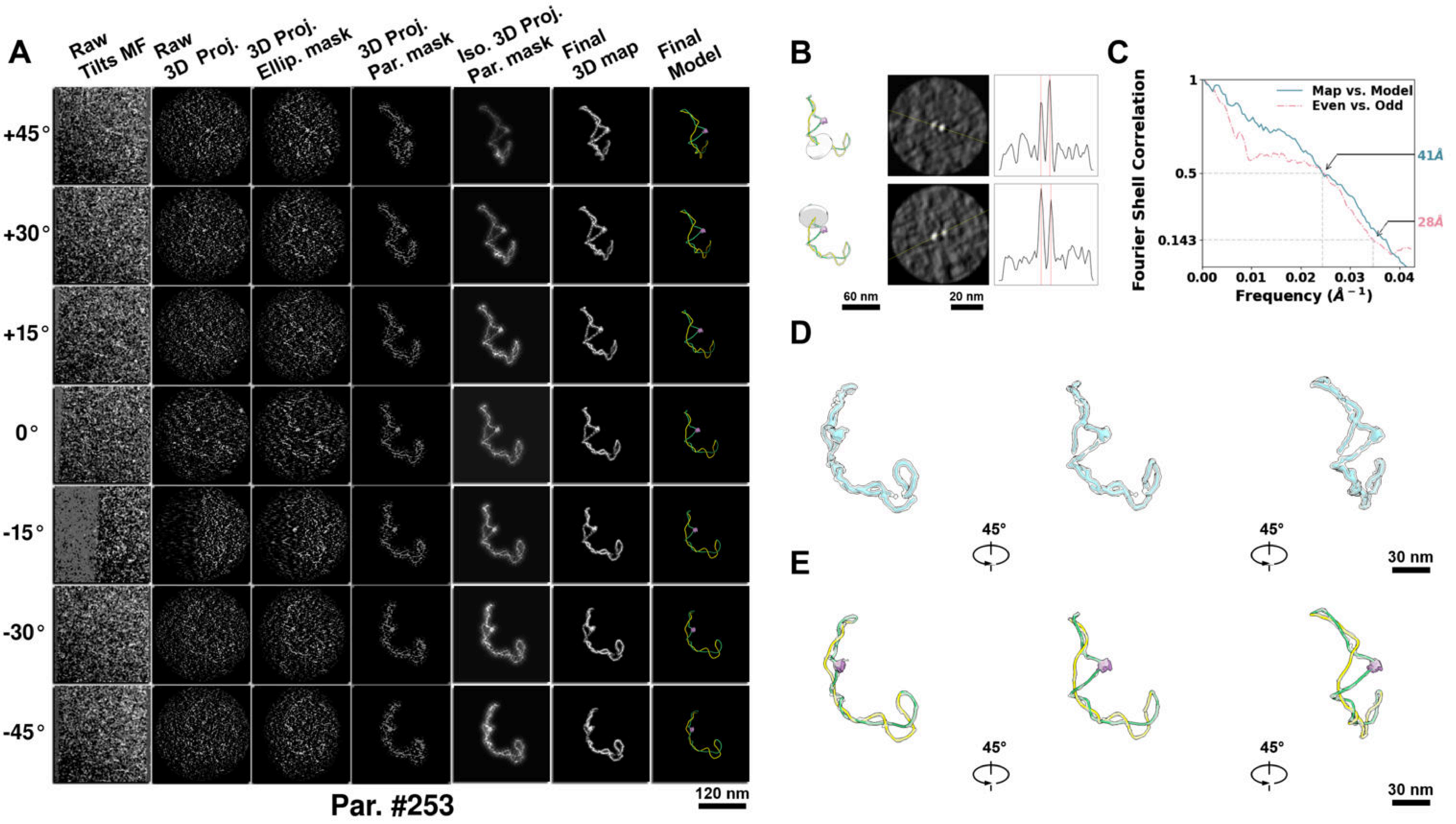

Supplementary Particle Figure 253. Cryo-ET 3D reconstruction of an individual TEC-TopI particle.
(A) 3D reconstruction of the plasmid particle (index no. 253). The first column shows seven representative tilt images from +45° to -45° in step of 15°. The second, third, and fourth columns show 3D projections of the particle with spherical, ellipsoidal (thinner along the z-dimension), and particle-shaped masks, respectively. The fifth column displays the 3D projections of the enhanced and IsoNet missing-wedge-corrected particle. The sixth and seventh columns present the final 3D map and the flexibly fitted model, respectively. (B) Two cross-sectional views (12 nm thickness) of the plasmid density map along its plectoneme axis are shown in the left-middle panel. The intensity profile along the line crossing the two high-density DNA spots is displayed in the right panel. (C) Resolution assessment of the final 3D map using Fourier shell correlation (FSC). Two criteria are shown: FSC between two half-maps reconstructed from even and odd frames (evaluated at 0.143) and FSC between the final 3D map and the fitted model (evaluated at 0.5). (D) Zoomed-in views of the final 3D density map from panel A, displayed at two contour levels. (E) Superimposition of the high-contour level map from panel D onto its fitted model.

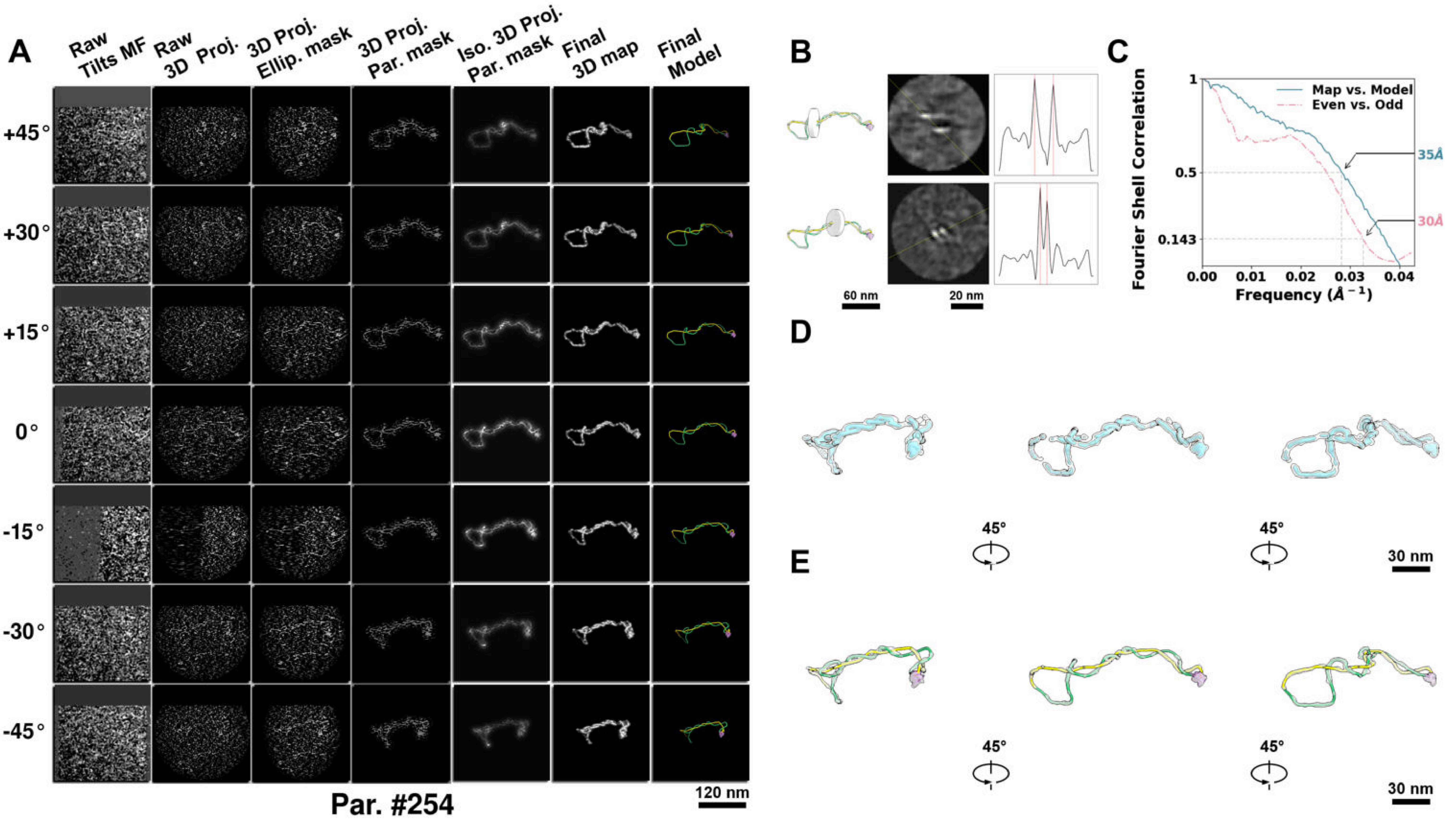

Supplementary Particle Figure 254. Cryo-ET 3D reconstruction of an individual TEC-TopI particle.
(A) 3D reconstruction of the plasmid particle (index no. 254). The first column shows seven representative tilt images from +45° to -45° in step of 15°. The second, third, and fourth columns show 3D projections of the particle with spherical, ellipsoidal (thinner along the z-dimension), and particle-shaped masks, respectively. The fifth column displays the 3D projections of the enhanced and IsoNet missing-wedge-corrected particle. The sixth and seventh columns present the final 3D map and the flexibly fitted model, respectively. (B) Two cross-sectional views (12 nm thickness) of the plasmid density map along its plectoneme axis are shown in the left-middle panel. The intensity profile along the line crossing the two high-density DNA spots is displayed in the right panel. (C) Resolution assessment of the final 3D map using Fourier shell correlation (FSC). Two criteria are shown: FSC between two half-maps reconstructed from even and odd frames (evaluated at 0.143) and FSC between the final 3D map and the fitted model (evaluated at 0.5). (D) Zoomed-in views of the final 3D density map from panel A, displayed at two contour levels. (E) Superimposition of the high-contour level map from panel D onto its fitted model.

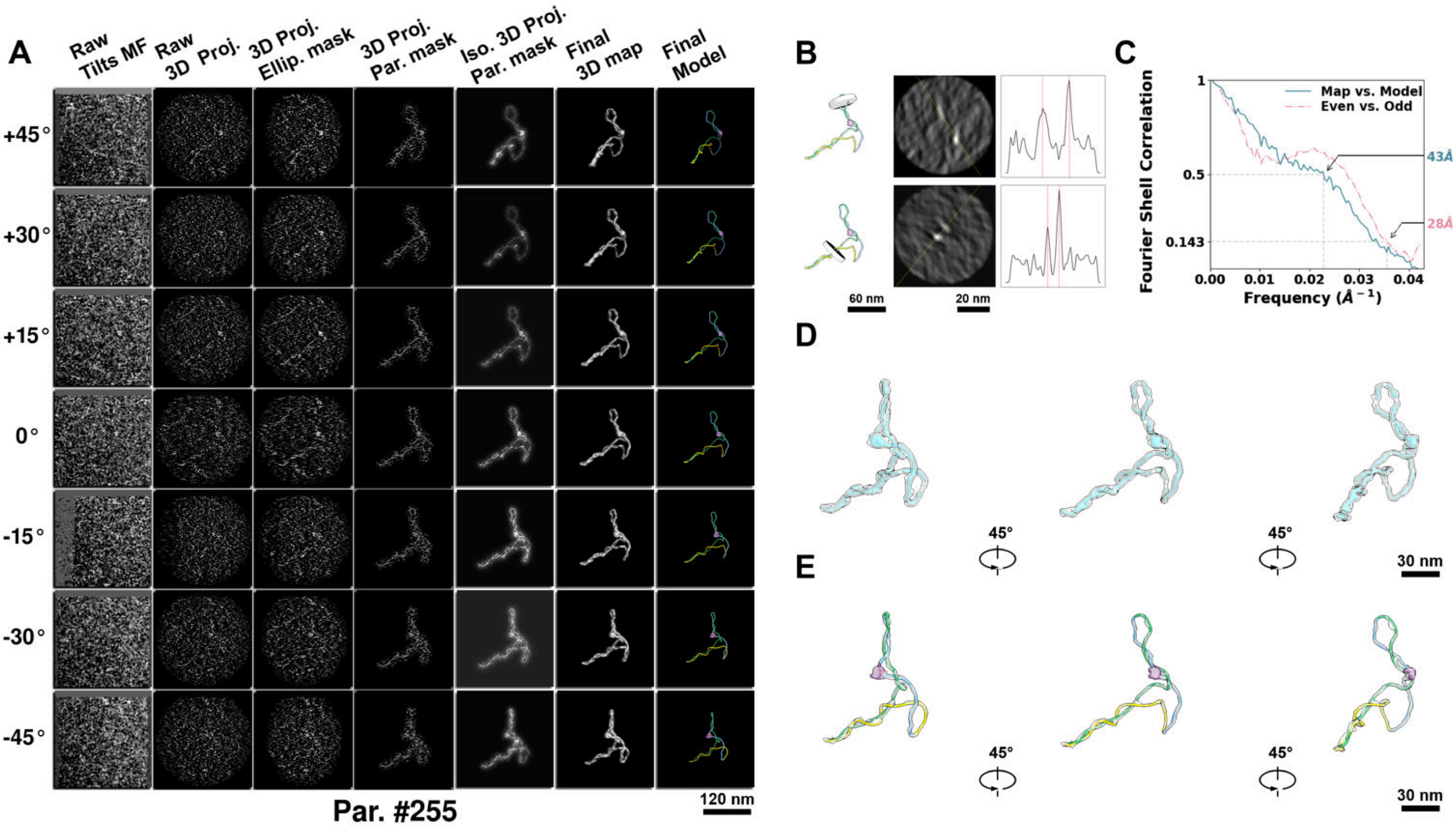

Supplementary Particle Figure 255. Cryo-ET 3D reconstruction of an individual TEC-TopI particle.
(A) 3D reconstruction of the plasmid particle (index no. 255). The first column shows seven representative tilt images from +45° to -45° in step of 15°. The second, third, and fourth columns show 3D projections of the particle with spherical, ellipsoidal (thinner along the z-dimension), and particle-shaped masks, respectively. The fifth column displays the 3D projections of the enhanced and IsoNet missing-wedge-corrected particle. The sixth and seventh columns present the final 3D map and the flexibly fitted model, respectively. (B) Two cross-sectional views (12 nm thickness) of the plasmid density map along its plectoneme axis are shown in the left-middle panel. The intensity profile along the line crossing the two high-density DNA spots is displayed in the right panel. (C) Resolution assessment of the final 3D map using Fourier shell correlation (FSC). Two criteria are shown: FSC between two half-maps reconstructed from even and odd frames (evaluated at 0.143) and FSC between the final 3D map and the fitted model (evaluated at 0.5). (D) Zoomed-in views of the final 3D density map from panel A, displayed at two contour levels. (E) Superimposition of the high-contour level map from panel D onto its fitted model.

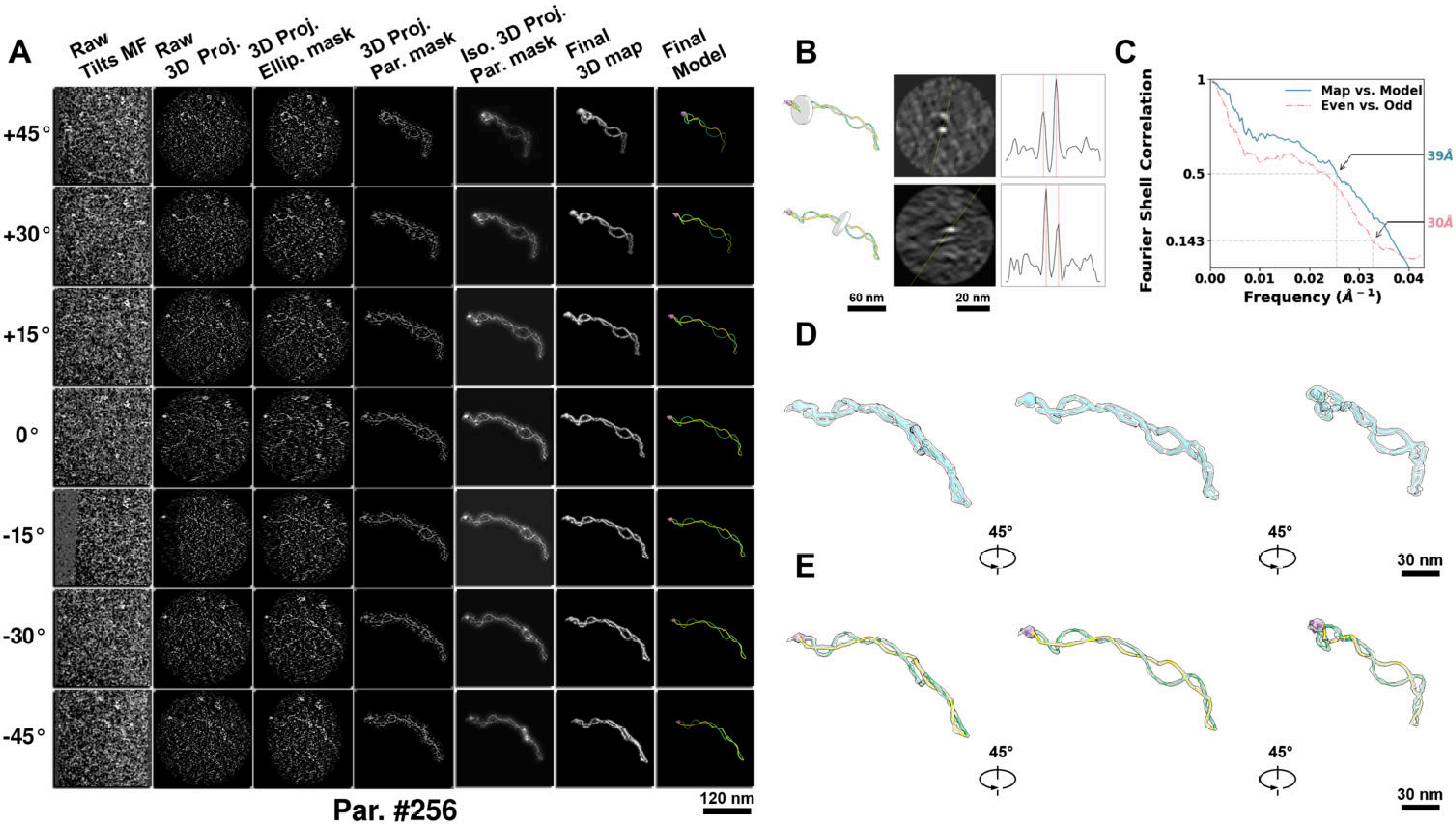

Supplementary Particle Figure 256. Cryo-ET 3D reconstruction of an individual TEC-TopI particle.
(A) 3D reconstruction of the plasmid particle (index no. 256). The first column shows seven representative tilt images from +45° to -45° in step of 15°. The second, third, and fourth columns show 3D projections of the particle with spherical, ellipsoidal (thinner along the z-dimension), and particle-shaped masks, respectively. The fifth column displays the 3D projections of the enhanced and IsoNet missing-wedge-corrected particle. The sixth and seventh columns present the final 3D map and the flexibly fitted model, respectively. (B) Two cross-sectional views (12 nm thickness) of the plasmid density map along its plectoneme axis are shown in the left-middle panel. The intensity profile along the line crossing the two high-density DNA spots is displayed in the right panel. (C) Resolution assessment of the final 3D map using Fourier shell correlation (FSC). Two criteria are shown: FSC between two half-maps reconstructed from even and odd frames (evaluated at 0.143) and FSC between the final 3D map and the fitted model (evaluated at 0.5). (D) Zoomed-in views of the final 3D density map from panel A, displayed at two contour levels. (E) Superimposition of the high-contour level map from panel D onto its fitted model.

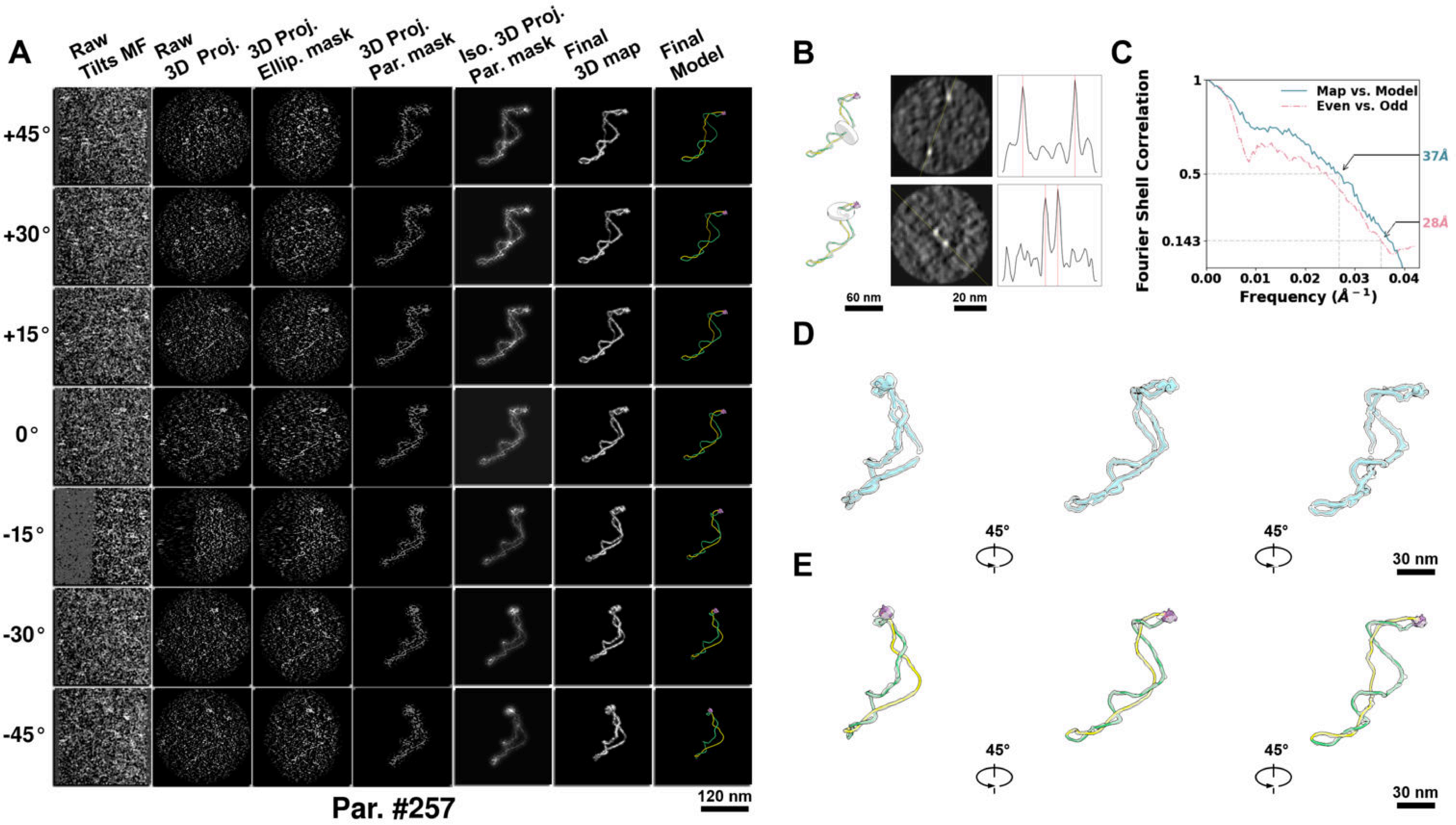

Supplementary Particle Figure 257. Cryo-ET 3D reconstruction of an individual TEC-TopI particle.
(A) 3D reconstruction of the plasmid particle (index no. 257). The first column shows seven representative tilt images from +45° to -45° in step of 15°. The second, third, and fourth columns show 3D projections of the particle with spherical, ellipsoidal (thinner along the z-dimension), and particle-shaped masks, respectively. The fifth column displays the 3D projections of the enhanced and IsoNet missing-wedge-corrected particle. The sixth and seventh columns present the final 3D map and the flexibly fitted model, respectively. (B) Two cross-sectional views (12 nm thickness) of the plasmid density map along its plectoneme axis are shown in the left-middle panel. The intensity profile along the line crossing the two high-density DNA spots is displayed in the right panel. (C) Resolution assessment of the final 3D map using Fourier shell correlation (FSC). Two criteria are shown: FSC between two half-maps reconstructed from even and odd frames (evaluated at 0.143) and FSC between the final 3D map and the fitted model (evaluated at 0.5). (D) Zoomed-in views of the final 3D density map from panel A, displayed at two contour levels. (E) Superimposition of the high-contour level map from panel D onto its fitted model.

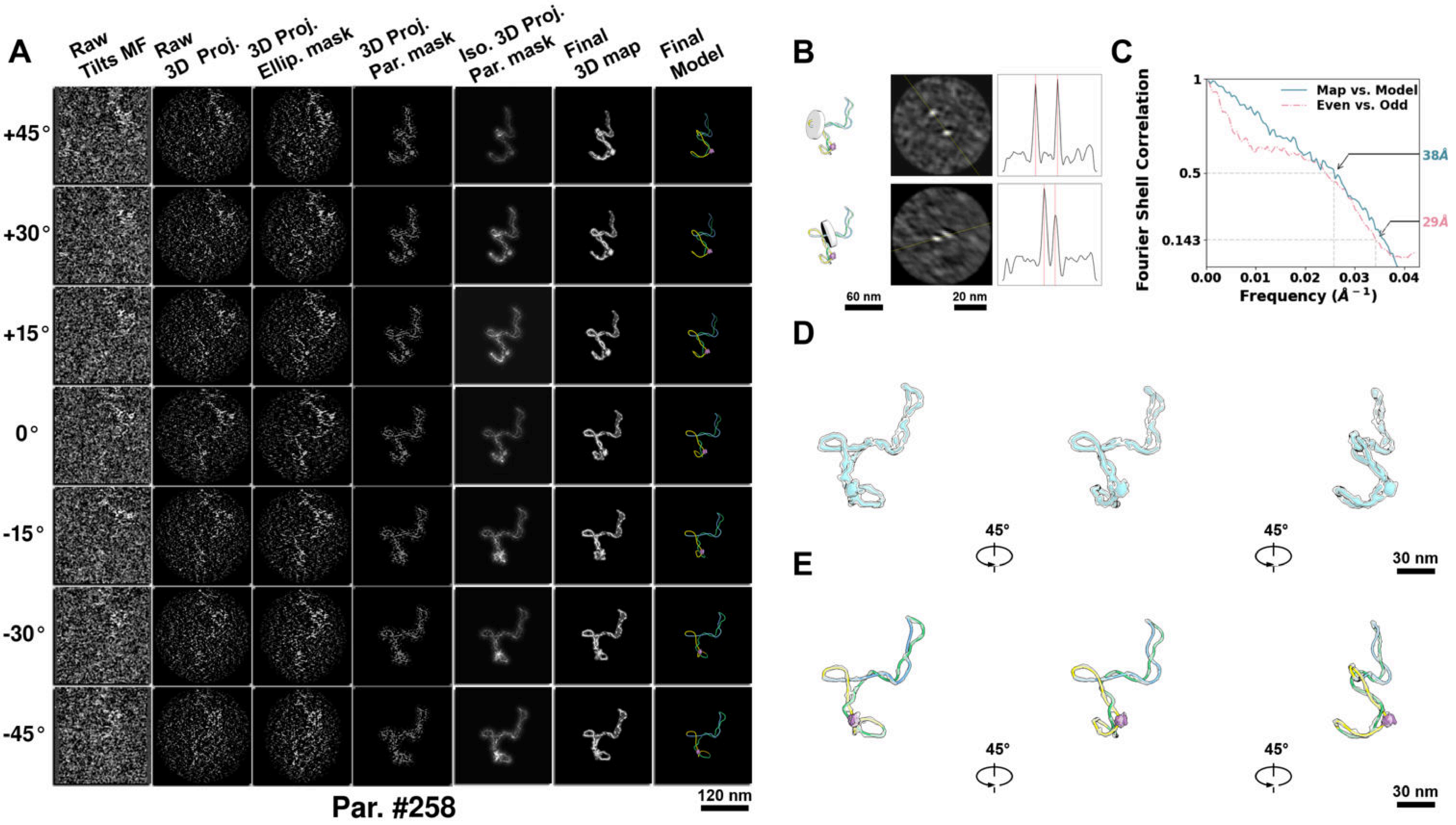

Supplementary Particle Figure 258. Cryo-ET 3D reconstruction of an individual TEC-TopI particle.
(A) 3D reconstruction of the plasmid particle (index no. 258). The first column shows seven representative tilt images from +45° to -45° in step of 15°. The second, third, and fourth columns show 3D projections of the particle with spherical, ellipsoidal (thinner along the z-dimension), and particle-shaped masks, respectively. The fifth column displays the 3D projections of the enhanced and IsoNet missing-wedge-corrected particle. The sixth and seventh columns present the final 3D map and the flexibly fitted model, respectively. (B) Two cross-sectional views (12 nm thickness) of the plasmid density map along its plectoneme axis are shown in the left-middle panel. The intensity profile along the line crossing the two high-density DNA spots is displayed in the right panel. (C) Resolution assessment of the final 3D map using Fourier shell correlation (FSC). Two criteria are shown: FSC between two half-maps reconstructed from even and odd frames (evaluated at 0.143) and FSC between the final 3D map and the fitted model (evaluated at 0.5). (D) Zoomed-in views of the final 3D density map from panel A, displayed at two contour levels. (E) Superimposition of the high-contour level map from panel D onto its fitted model.

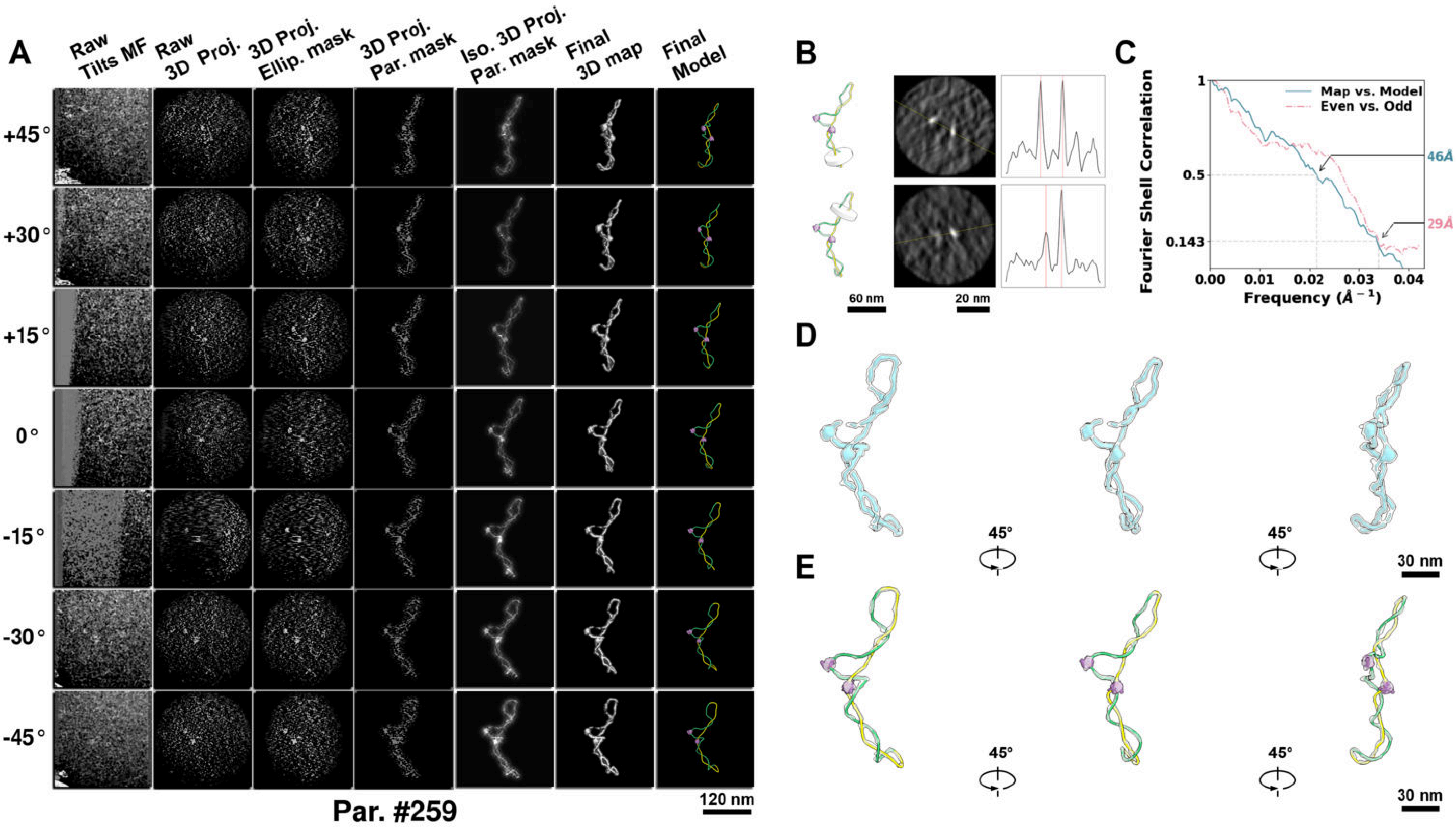

Supplementary Particle Figure 259. Cryo-ET 3D reconstruction of an individual TEC-TopI particle.
(A) 3D reconstruction of the plasmid particle (index no. 259). The first column shows seven representative tilt images from +45° to -45° in step of 15°. The second, third, and fourth columns show 3D projections of the particle with spherical, ellipsoidal (thinner along the z-dimension), and particle-shaped masks, respectively. The fifth column displays the 3D projections of the enhanced and IsoNet missing-wedge-corrected particle. The sixth and seventh columns present the final 3D map and the flexibly fitted model, respectively. (B) Two cross-sectional views (12 nm thickness) of the plasmid density map along its plectoneme axis are shown in the left-middle panel. The intensity profile along the line crossing the two high-density DNA spots is displayed in the right panel. (C) Resolution assessment of the final 3D map using Fourier shell correlation (FSC). Two criteria are shown: FSC between two half-maps reconstructed from even and odd frames (evaluated at 0.143) and FSC between the final 3D map and the fitted model (evaluated at 0.5). (D) Zoomed-in views of the final 3D density map from panel A, displayed at two contour levels. (E) Superimposition of the high-contour level map from panel D onto its fitted model.

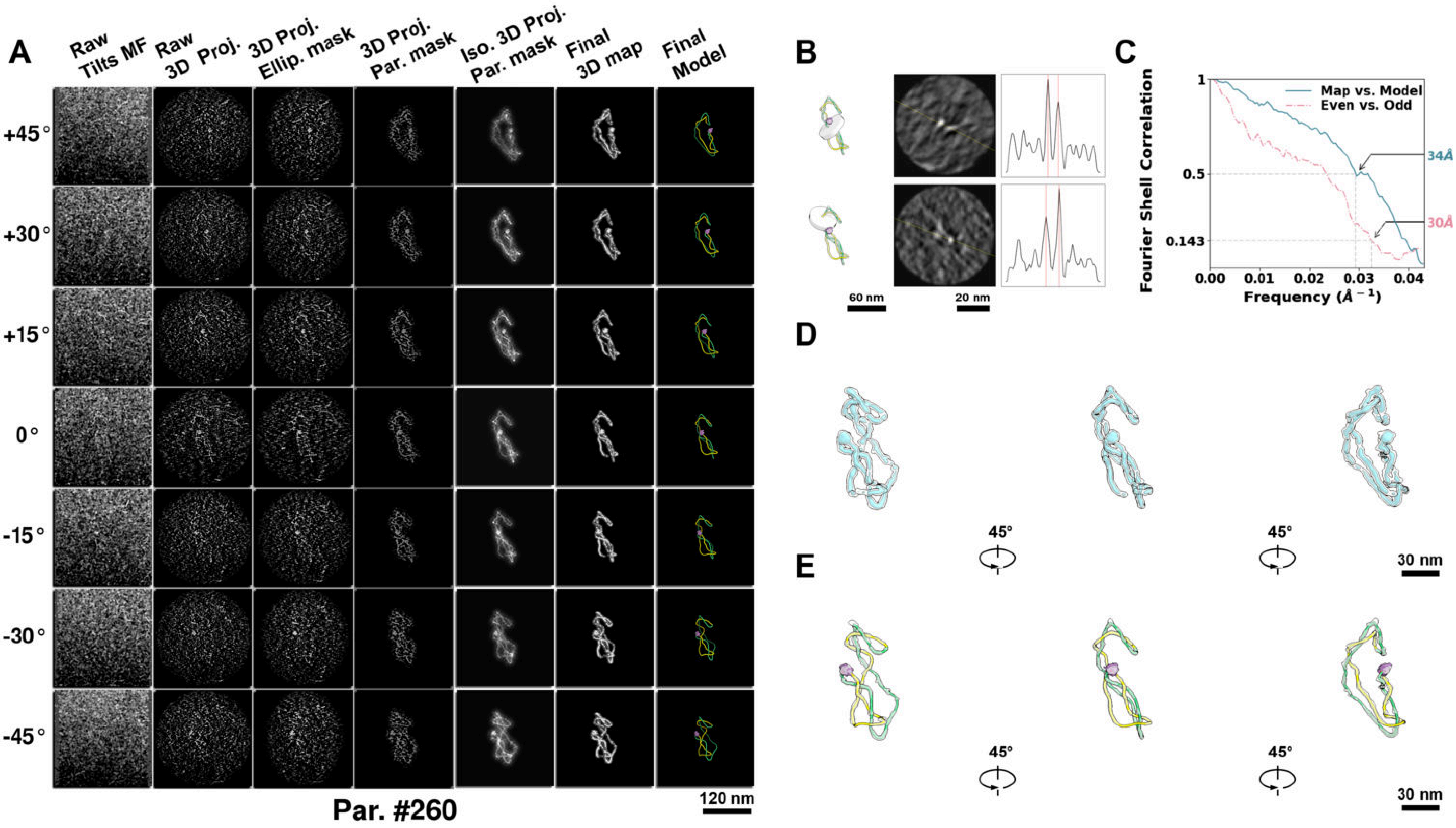

Supplementary Particle Figure 260. Cryo-ET 3D reconstruction of an individual TEC-TopI particle.
(A) 3D reconstruction of the plasmid particle (index no. 260). The first column shows seven representative tilt images from +45° to -45° in step of 15°. The second, third, and fourth columns show 3D projections of the particle with spherical, ellipsoidal (thinner along the z-dimension), and particle-shaped masks, respectively. The fifth column displays the 3D projections of the enhanced and IsoNet missing-wedge-corrected particle. The sixth and seventh columns present the final 3D map and the flexibly fitted model, respectively. (B) Two cross-sectional views (12 nm thickness) of the plasmid density map along its plectoneme axis are shown in the left-middle panel. The intensity profile along the line crossing the two high-density DNA spots is displayed in the right panel. (C) Resolution assessment of the final 3D map using Fourier shell correlation (FSC). Two criteria are shown: FSC between two half-maps reconstructed from even and odd frames (evaluated at 0.143) and FSC between the final 3D map and the fitted model (evaluated at 0.5). (D) Zoomed-in views of the final 3D density map from panel A, displayed at two contour levels. (E) Superimposition of the high-contour level map from panel D onto its fitted model.

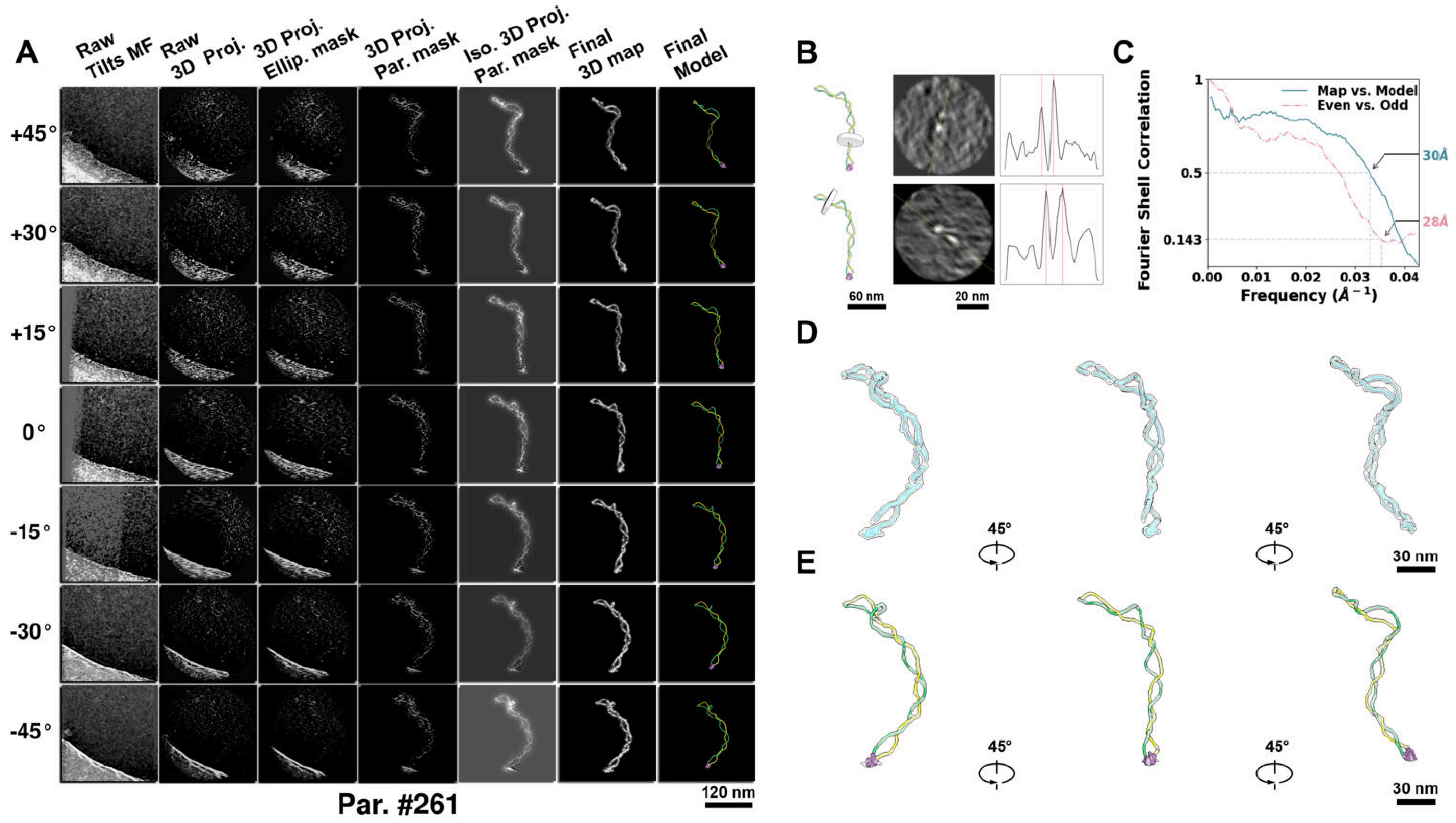

Supplementary Particle Figure 261. Cryo-ET 3D reconstruction of an individual TEC-TopI particle.
(A) 3D reconstruction of the plasmid particle (index no. 261). The first column shows seven representative tilt images from +45° to -45° in step of 15°. The second, third, and fourth columns show 3D projections of the particle with spherical, ellipsoidal (thinner along the z-dimension), and particle-shaped masks, respectively. The fifth column displays the 3D projections of the enhanced and IsoNet missing-wedge-corrected particle. The sixth and seventh columns present the final 3D map and the flexibly fitted model, respectively. (B) Two cross-sectional views (12 nm thickness) of the plasmid density map along its plectoneme axis are shown in the left-middle panel. The intensity profile along the line crossing the two high-density DNA spots is displayed in the right panel. (C) Resolution assessment of the final 3D map using Fourier shell correlation (FSC). Two criteria are shown: FSC between two half-maps reconstructed from even and odd frames (evaluated at 0.143) and FSC between the final 3D map and the fitted model (evaluated at 0.5). (D) Zoomed-in views of the final 3D density map from panel A, displayed at two contour levels. (E) Superimposition of the high-contour level map from panel D onto its fitted model.

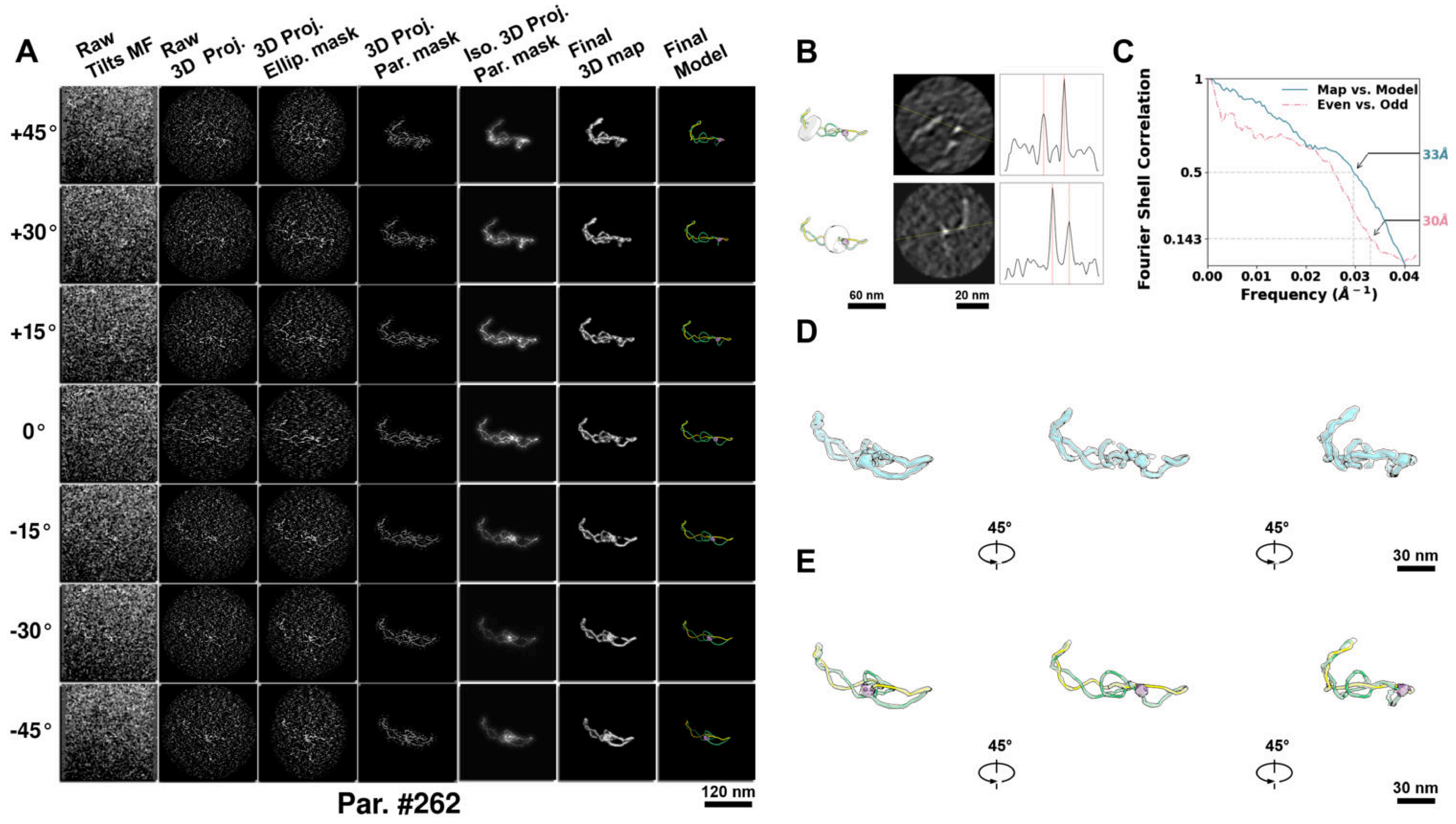

Supplementary Particle Figure 262. Cryo-ET 3D reconstruction of an individual TEC-TopI particle.
(A) 3D reconstruction of the plasmid particle (index no. 262). The first column shows seven representative tilt images from +45° to -45° in step of 15°. The second, third, and fourth columns show 3D projections of the particle with spherical, ellipsoidal (thinner along the z-dimension), and particle-shaped masks, respectively. The fifth column displays the 3D projections of the enhanced and IsoNet missing-wedge-corrected particle. The sixth and seventh columns present the final 3D map and the flexibly fitted model, respectively. (B) Two cross-sectional views (12 nm thickness) of the plasmid density map along its plectoneme axis are shown in the left-middle panel. The intensity profile along the line crossing the two high-density DNA spots is displayed in the right panel. (C) Resolution assessment of the final 3D map using Fourier shell correlation (FSC). Two criteria are shown: FSC between two half-maps reconstructed from even and odd frames (evaluated at 0.143) and FSC between the final 3D map and the fitted model (evaluated at 0.5). (D) Zoomed-in views of the final 3D density map from panel A, displayed at two contour levels. (E) Superimposition of the high-contour level map from panel D onto its fitted model.

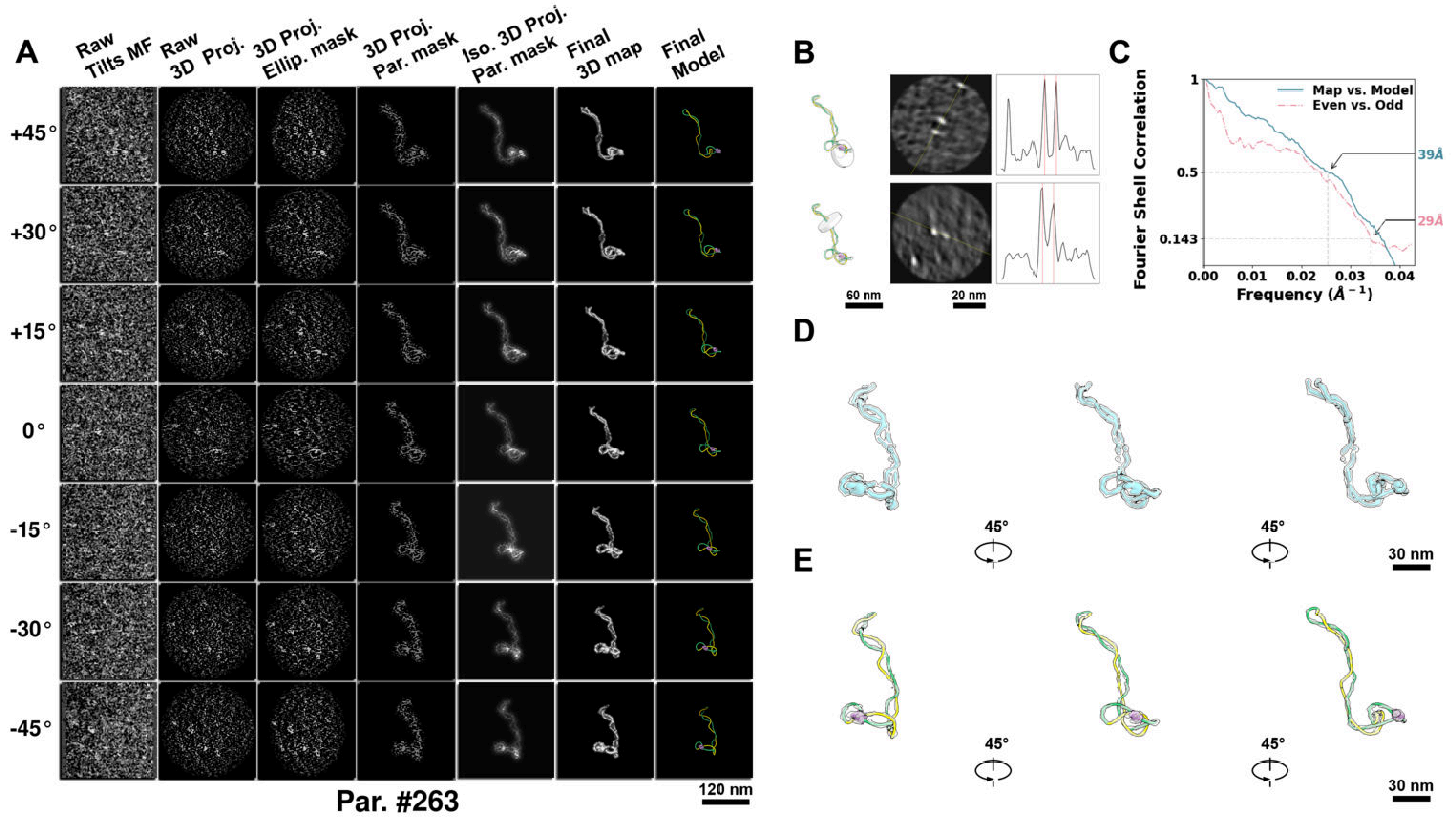

Supplementary Particle Figure 263. Cryo-ET 3D reconstruction of an individual TEC-TopI particle.
(A) 3D reconstruction of the plasmid particle (index no. 263). The first column shows seven representative tilt images from +45° to -45° in step of 15°. The second, third, and fourth columns show 3D projections of the particle with spherical, ellipsoidal (thinner along the z-dimension), and particle-shaped masks, respectively. The fifth column displays the 3D projections of the enhanced and IsoNet missing-wedge-corrected particle. The sixth and seventh columns present the final 3D map and the flexibly fitted model, respectively. (B) Two cross-sectional views (12 nm thickness) of the plasmid density map along its plectoneme axis are shown in the left-middle panel. The intensity profile along the line crossing the two high-density DNA spots is displayed in the right panel. (C) Resolution assessment of the final 3D map using Fourier shell correlation (FSC). Two criteria are shown: FSC between two half-maps reconstructed from even and odd frames (evaluated at 0.143) and FSC between the final 3D map and the fitted model (evaluated at 0.5). (D) Zoomed-in views of the final 3D density map from panel A, displayed at two contour levels. (E) Superimposition of the high-contour level map from panel D onto its fitted model.

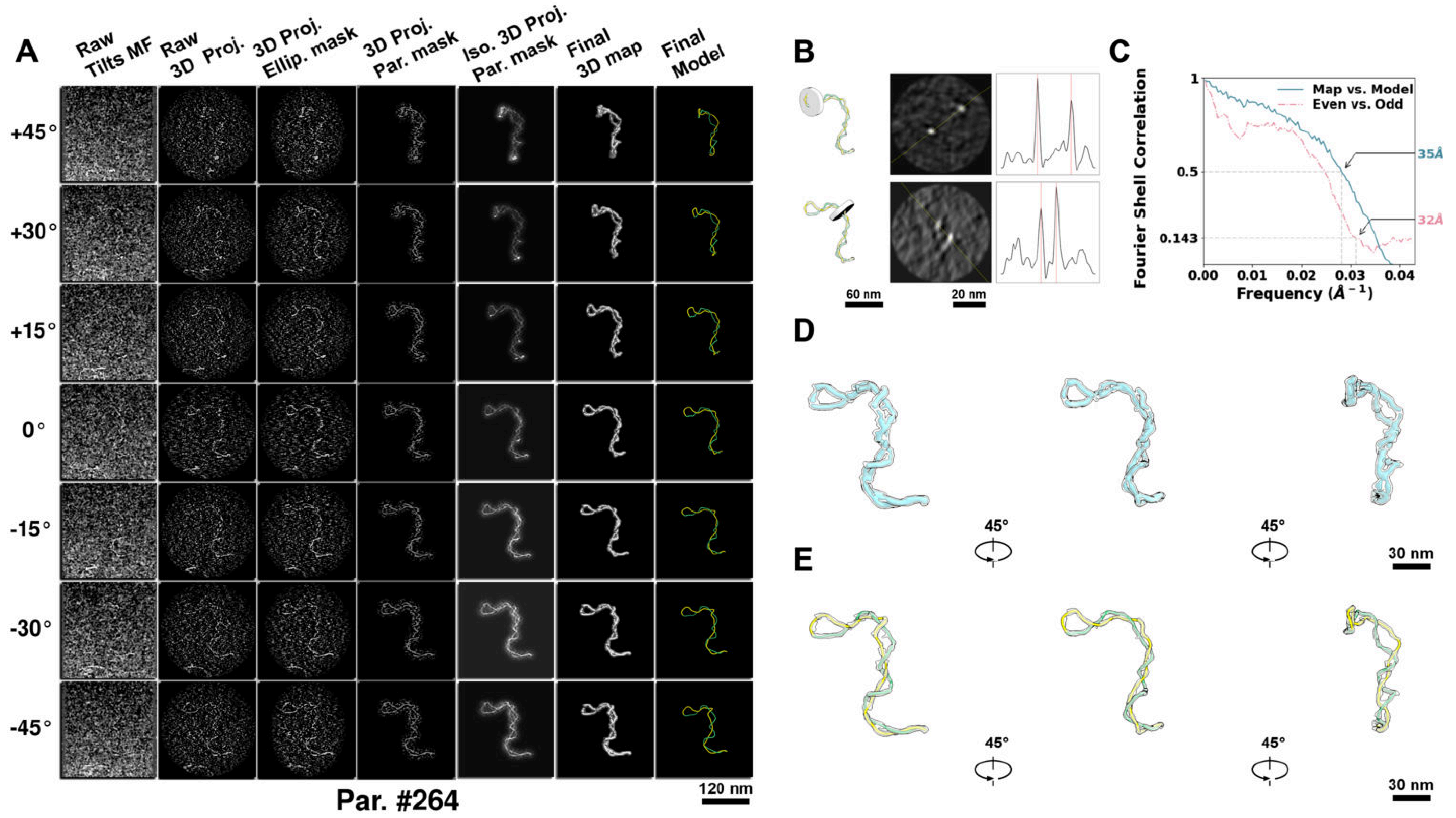

Supplementary Particle Figure 264. Cryo-ET 3D reconstruction of an individual TEC-TopI particle.
(A) 3D reconstruction of the plasmid particle (index no. 264). The first column shows seven representative tilt images from +45° to -45° in step of 15°. The second, third, and fourth columns show 3D projections of the particle with spherical, ellipsoidal (thinner along the z-dimension), and particle-shaped masks, respectively. The fifth column displays the 3D projections of the enhanced and IsoNet missing-wedge-corrected particle. The sixth and seventh columns present the final 3D map and the flexibly fitted model, respectively. (B) Two cross-sectional views (12 nm thickness) of the plasmid density map along its plectoneme axis are shown in the left-middle panel. The intensity profile along the line crossing the two high-density DNA spots is displayed in the right panel. (C) Resolution assessment of the final 3D map using Fourier shell correlation (FSC). Two criteria are shown: FSC between two half-maps reconstructed from even and odd frames (evaluated at 0.143) and FSC between the final 3D map and the fitted model (evaluated at 0.5). (D) Zoomed-in views of the final 3D density map from panel A, displayed at two contour levels. (E) Superimposition of the high-contour level map from panel D onto its fitted model.

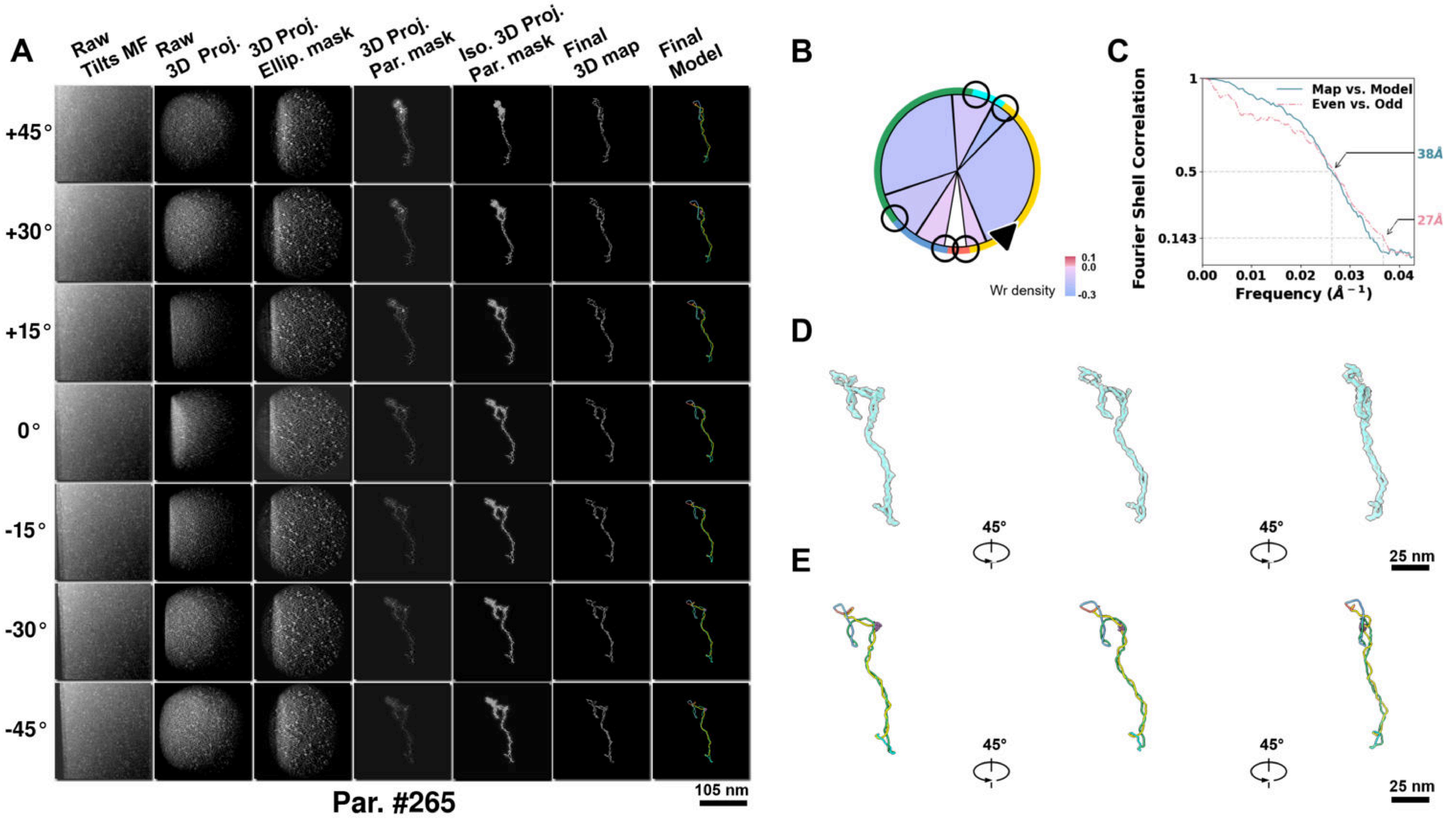

Supplementary Particle Figure 265. Cryo-ET 3D reconstruction of an individual Opp.-TEC particle.
(A) 3D reconstruction of the plasmid particle (index no. 265). The first column shows seven representative tilt images from +45° to -45° in step of 15°. The second, third, and fourth columns show 3D projections of the particle with spherical, ellipsoidal (thinner along the z-dimension), and particle-shaped masks, respectively. The fifth column displays the 3D projections of the enhanced and IsoNet missing-wedge-corrected particle. The sixth and seventh columns present the final 3D map and the flexibly fitted model, respectively. (B) Circular schematic representation of a plasmid particle. The outer rim is color-coded to match the corresponding 3D model. Arrowheads indicate the transcriptional direction of bound RNAPs, and circles denote apical sites. Inner circular sectors represent individual plectonemes, with colors indicating writhe density (blue to red scale, −0.3 to 0.1). (C) Resolution assessment of the final 3D map using Fourier shell correlation (FSC). Two criteria are shown: FSC between two half-maps reconstructed from even and odd frames (evaluated at 0.143) and FSC between the final 3D map and the fitted model (evaluated at 0.5). (D) Zoomed-in views of the final 3D density map from panel A, displayed at two contour levels. (E) Superimposition of the high-contour level map from panel D onto its fitted model.

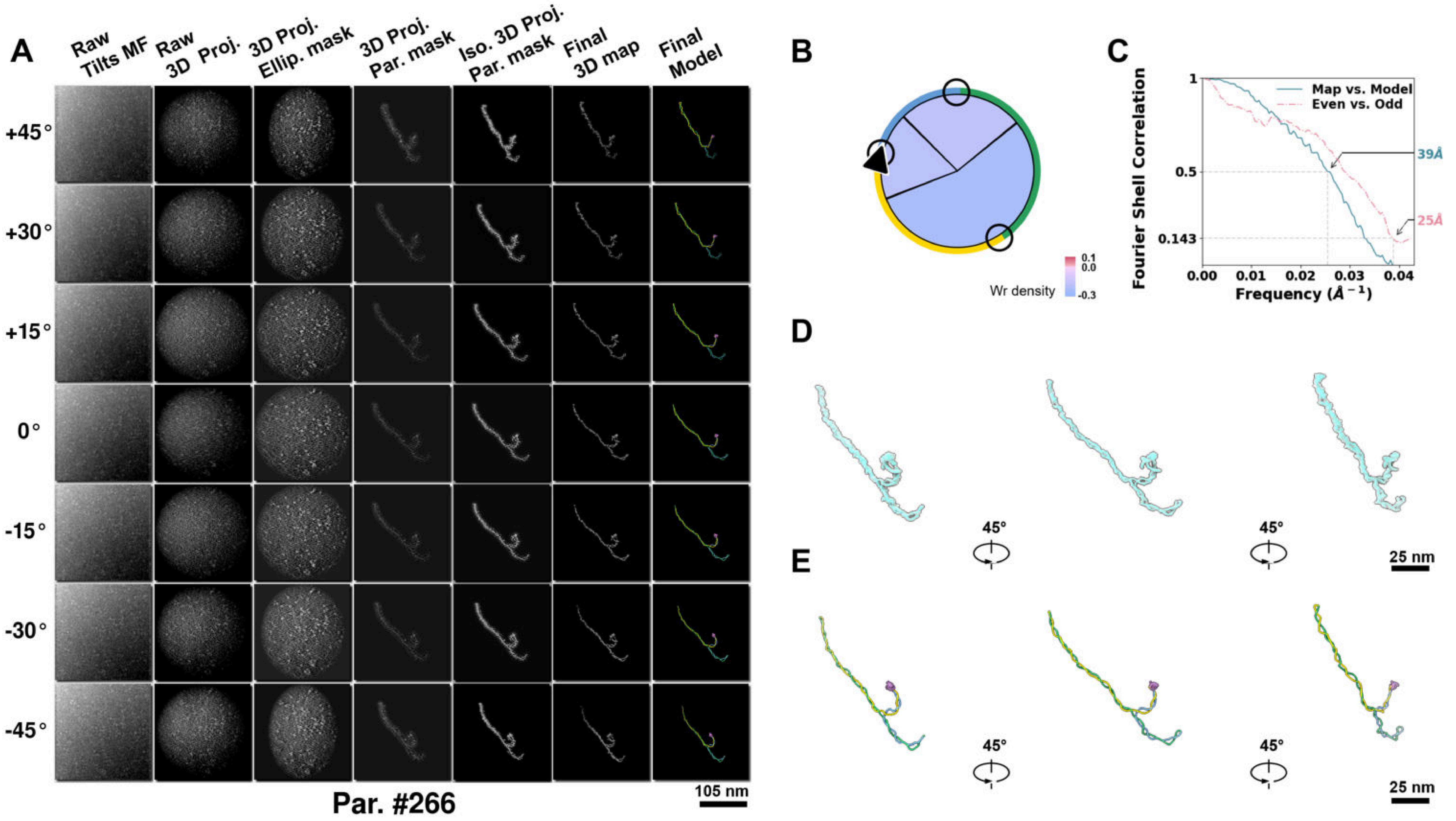

Supplementary Particle Figure 266. Cryo-ET 3D reconstruction of an individual Opp.-TEC particle.
(A) 3D reconstruction of the plasmid particle (index no. 266). The first column shows seven representative tilt images from +45° to -45° in step of 15°. The second, third, and fourth columns show 3D projections of the particle with spherical, ellipsoidal (thinner along the z-dimension), and particle-shaped masks, respectively. The fifth column displays the 3D projections of the enhanced and IsoNet missing-wedge-corrected particle. The sixth and seventh columns present the final 3D map and the flexibly fitted model, respectively. (B) Circular schematic representation of a plasmid particle. The outer rim is color-coded to match the corresponding 3D model. Arrowheads indicate the transcriptional direction of bound RNAPs, and circles denote apical sites. Inner circular sectors represent individual plectonemes, with colors indicating writhe density (blue to red scale, −0.3 to 0.1). (C) Resolution assessment of the final 3D map using Fourier shell correlation (FSC). Two criteria are shown: FSC between two half-maps reconstructed from even and odd frames (evaluated at 0.143) and FSC between the final 3D map and the fitted model (evaluated at 0.5). (D) Zoomed-in views of the final 3D density map from panel A, displayed at two contour levels. (E) Superimposition of the high-contour level map from panel D onto its fitted model.

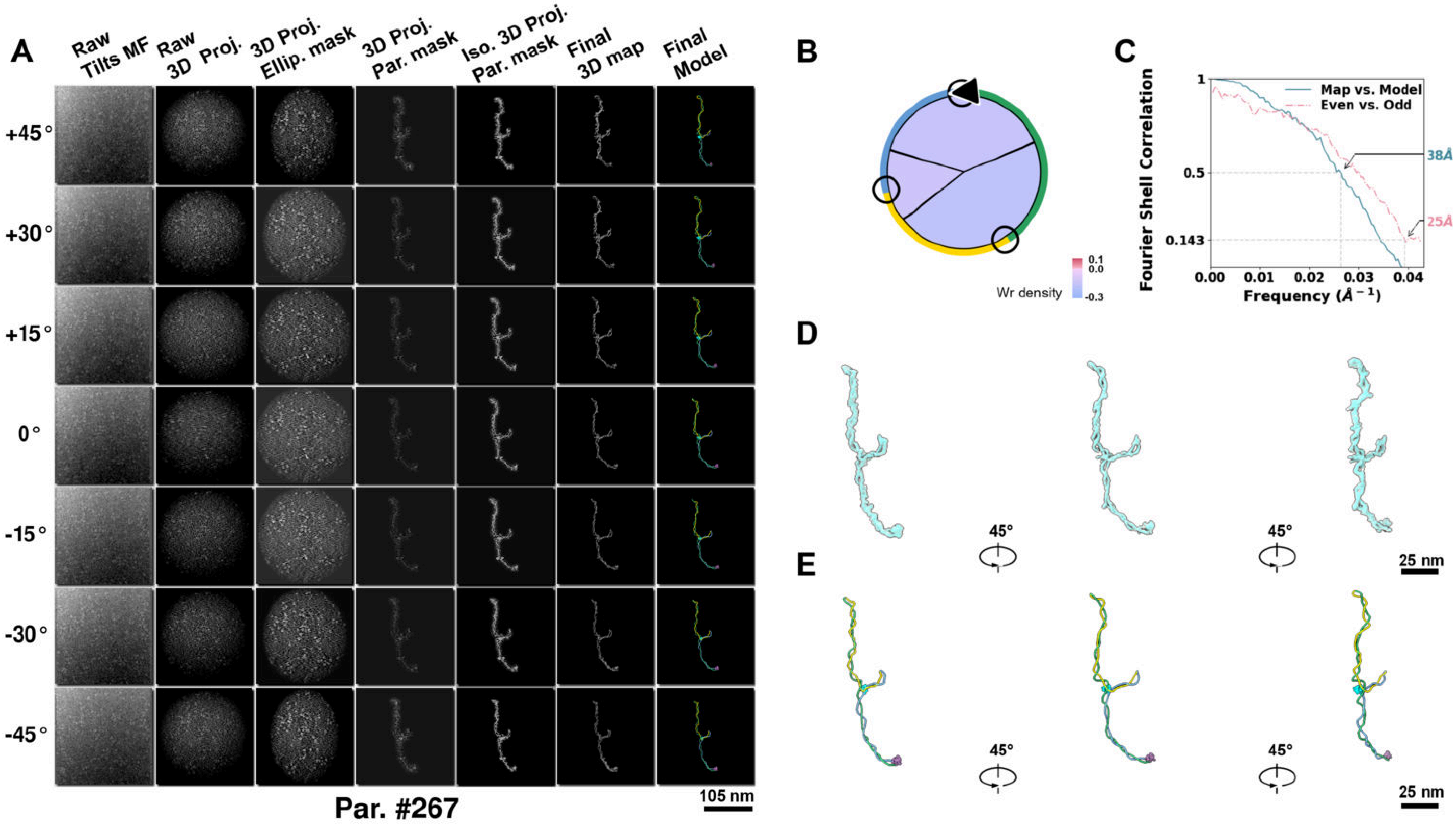

Supplementary Particle Figure 267. Cryo-ET 3D reconstruction of an individual Opp.-TEC particle.
(A) 3D reconstruction of the plasmid particle (index no. 267). The first column shows seven representative tilt images from +45° to -45° in step of 15°. The second, third, and fourth columns show 3D projections of the particle with spherical, ellipsoidal (thinner along the z-dimension), and particle-shaped masks, respectively. The fifth column displays the 3D projections of the enhanced and IsoNet missing-wedge-corrected particle. The sixth and seventh columns present the final 3D map and the flexibly fitted model, respectively. (B) Circular schematic representation of a plasmid particle. The outer rim is color-coded to match the corresponding 3D model. Arrowheads indicate the transcriptional direction of bound RNAPs, and circles denote apical sites. Inner circular sectors represent individual plectonemes, with colors indicating writhe density (blue to red scale, −0.3 to 0.1). (C) Resolution assessment of the final 3D map using Fourier shell correlation (FSC). Two criteria are shown: FSC between two half-maps reconstructed from even and odd frames (evaluated at 0.143) and FSC between the final 3D map and the fitted model (evaluated at 0.5). (D) Zoomed-in views of the final 3D density map from panel A, displayed at two contour levels. (E) Superimposition of the high-contour level map from panel D onto its fitted model.

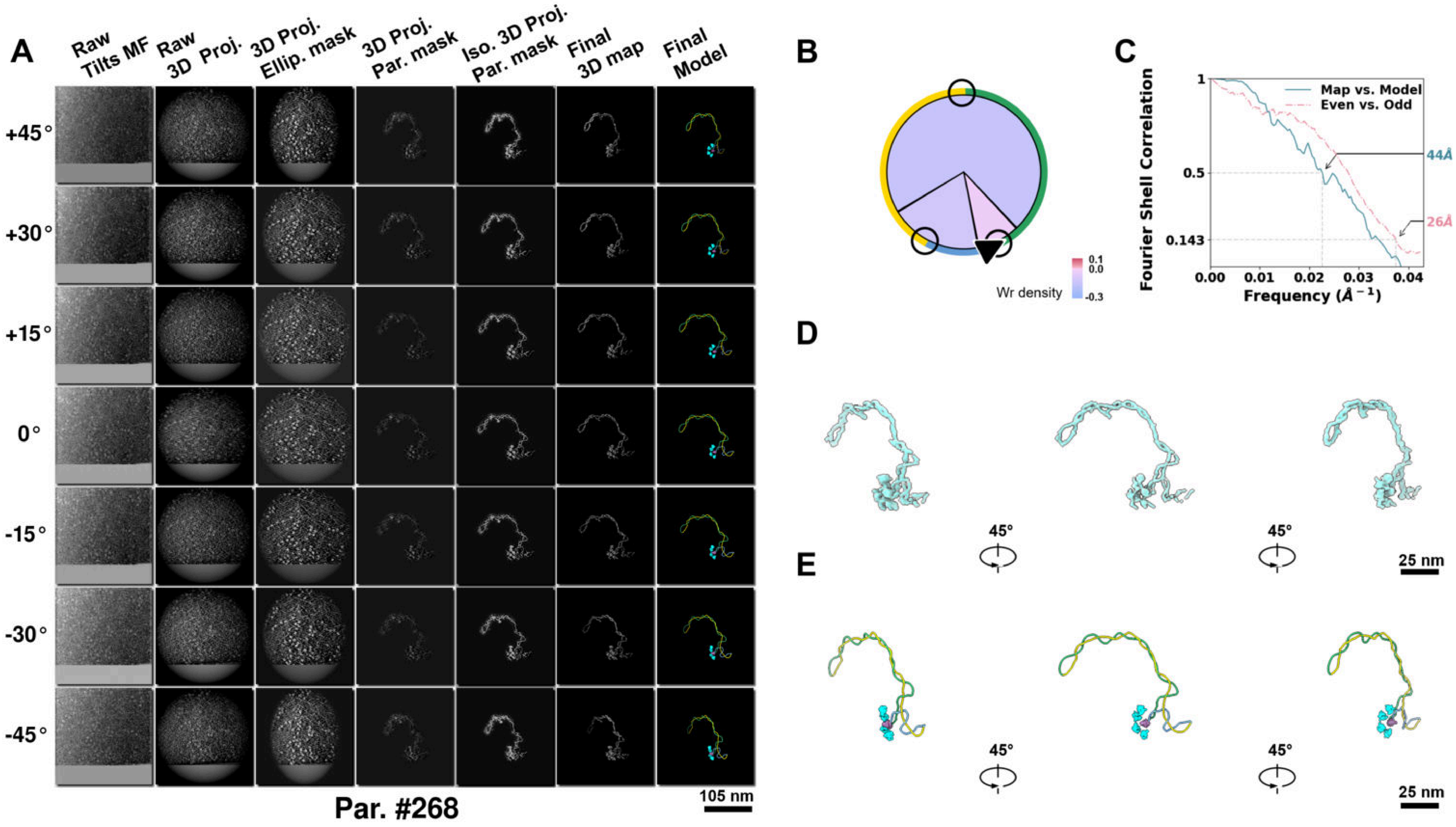

Supplementary Particle Figure 268. Cryo-ET 3D reconstruction of an individual Opp.-TEC particle.
(A) 3D reconstruction of the plasmid particle (index no. 268). The first column shows seven representative tilt images from +45° to -45° in step of 15°. The second, third, and fourth columns show 3D projections of the particle with spherical, ellipsoidal (thinner along the z-dimension), and particle-shaped masks, respectively. The fifth column displays the 3D projections of the enhanced and IsoNet missing-wedge-corrected particle. The sixth and seventh columns present the final 3D map and the flexibly fitted model, respectively. (B) Circular schematic representation of a plasmid particle. The outer rim is color-coded to match the corresponding 3D model. Arrowheads indicate the transcriptional direction of bound RNAPs, and circles denote apical sites. Inner circular sectors represent individual plectonemes, with colors indicating writhe density (blue to red scale, −0.3 to 0.1). (C) Resolution assessment of the final 3D map using Fourier shell correlation (FSC). Two criteria are shown: FSC between two half-maps reconstructed from even and odd frames (evaluated at 0.143) and FSC between the final 3D map and the fitted model (evaluated at 0.5). (D) Zoomed-in views of the final 3D density map from panel A, displayed at two contour levels. (E) Superimposition of the high-contour level map from panel D onto its fitted model.

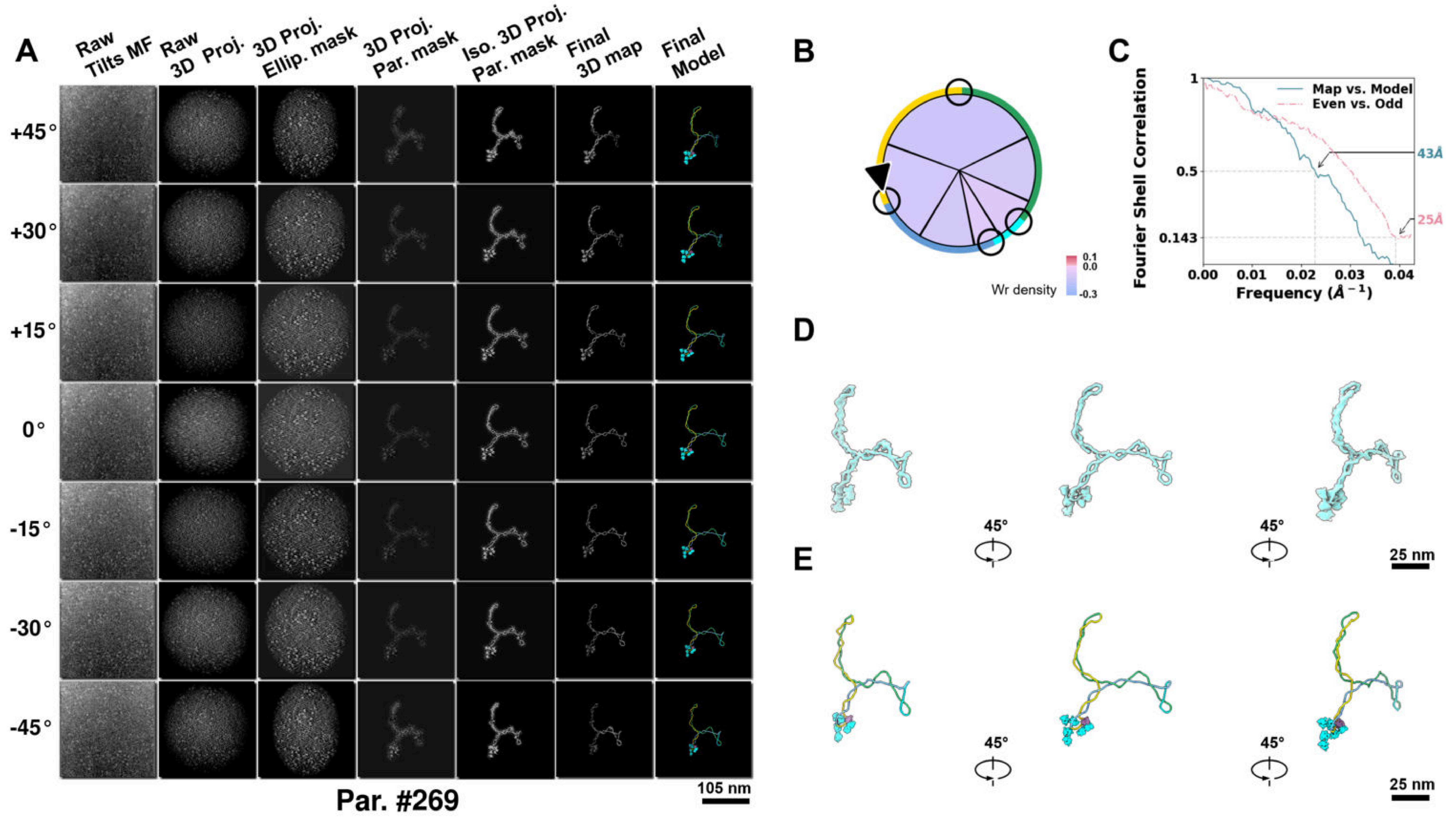

Supplementary Particle Figure 269. Cryo-ET 3D reconstruction of an individual Opp.-TEC particle.
(A) 3D reconstruction of the plasmid particle (index no. 269). The first column shows seven representative tilt images from +45° to -45° in step of 15°. The second, third, and fourth columns show 3D projections of the particle with spherical, ellipsoidal (thinner along the z-dimension), and particle-shaped masks, respectively. The fifth column displays the 3D projections of the enhanced and IsoNet missing-wedge-corrected particle. The sixth and seventh columns present the final 3D map and the flexibly fitted model, respectively. (B) Circular schematic representation of a plasmid particle. The outer rim is color-coded to match the corresponding 3D model. Arrowheads indicate the transcriptional direction of bound RNAPs, and circles denote apical sites. Inner circular sectors represent individual plectonemes, with colors indicating writhe density (blue to red scale, −0.3 to 0.1). (C) Resolution assessment of the final 3D map using Fourier shell correlation (FSC). Two criteria are shown: FSC between two half-maps reconstructed from even and odd frames (evaluated at 0.143) and FSC between the final 3D map and the fitted model (evaluated at 0.5). (D) Zoomed-in views of the final 3D density map from panel A, displayed at two contour levels. (E) Superimposition of the high-contour level map from panel D onto its fitted model.

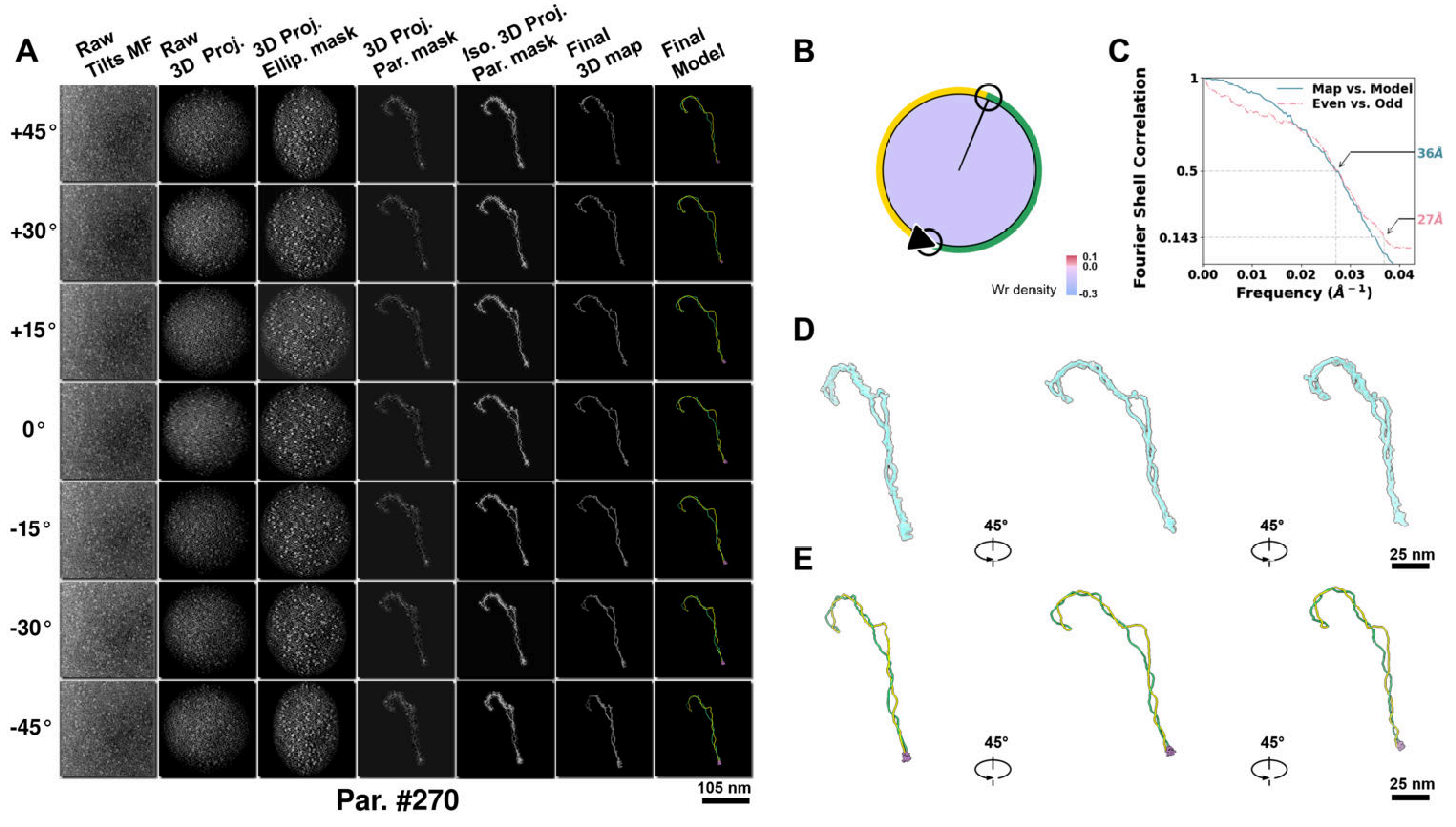

Supplementary Particle Figure 270. Cryo-ET 3D reconstruction of an individual Opp.-TEC particle.
(A) 3D reconstruction of the plasmid particle (index no. 270). The first column shows seven representative tilt images from +45° to -45° in step of 15°. The second, third, and fourth columns show 3D projections of the particle with spherical, ellipsoidal (thinner along the z-dimension), and particle-shaped masks, respectively. The fifth column displays the 3D projections of the enhanced and IsoNet missing-wedge-corrected particle. The sixth and seventh columns present the final 3D map and the flexibly fitted model, respectively. (B) Circular schematic representation of a plasmid particle. The outer rim is color-coded to match the corresponding 3D model. Arrowheads indicate the transcriptional direction of bound RNAPs, and circles denote apical sites. Inner circular sectors represent individual plectonemes, with colors indicating writhe density (blue to red scale, −0.3 to 0.1). (C) Resolution assessment of the final 3D map using Fourier shell correlation (FSC). Two criteria are shown: FSC between two half-maps reconstructed from even and odd frames (evaluated at 0.143) and FSC between the final 3D map and the fitted model (evaluated at 0.5). (D) Zoomed-in views of the final 3D density map from panel A, displayed at two contour levels. (E) Superimposition of the high-contour level map from panel D onto its fitted model.

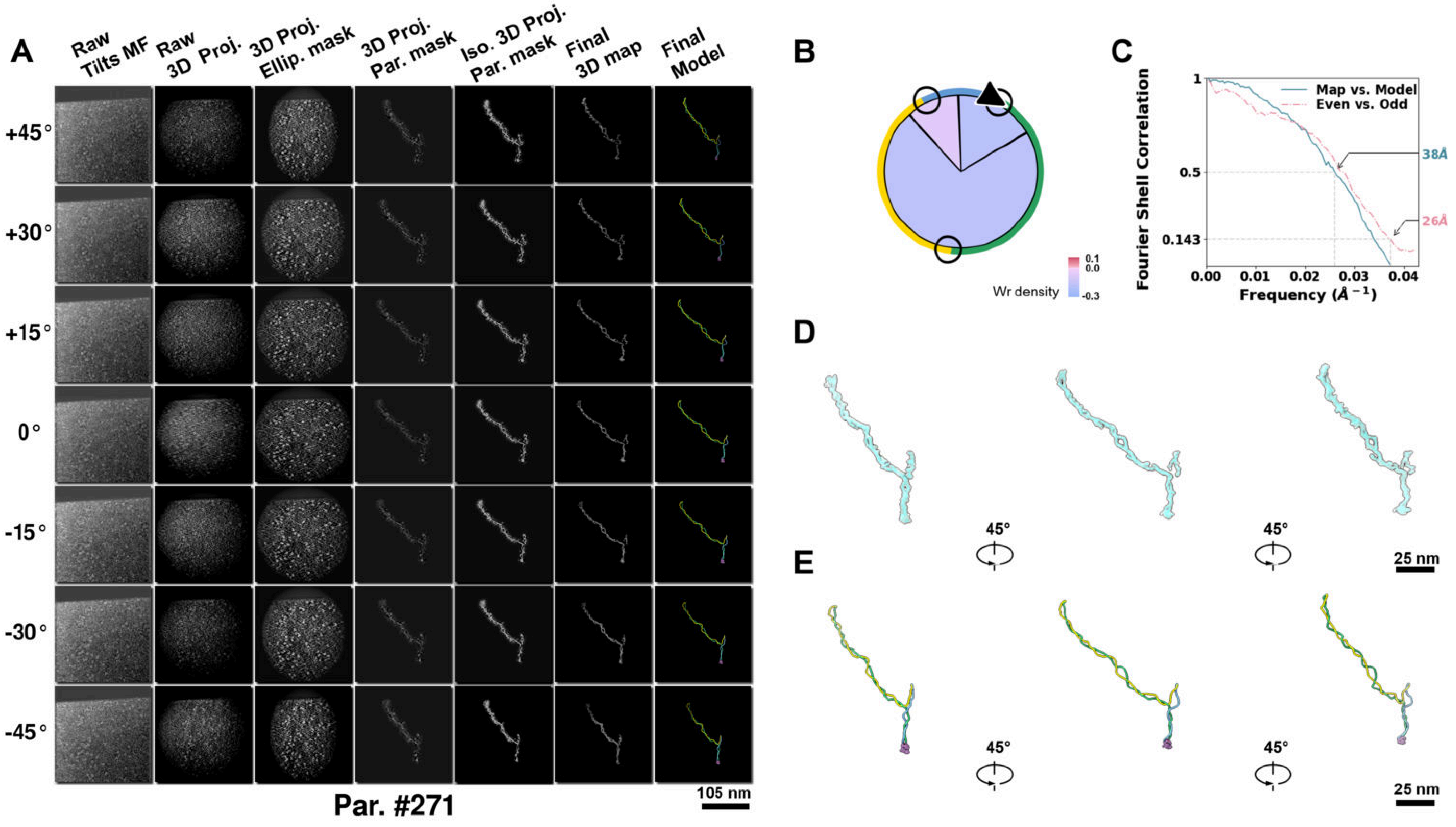

Supplementary Particle Figure 271. Cryo-ET 3D reconstruction of an individual Opp.-TEC particle.
(A) 3D reconstruction of the plasmid particle (index no. 271). The first column shows seven representative tilt images from +45° to -45° in step of 15°. The second, third, and fourth columns show 3D projections of the particle with spherical, ellipsoidal (thinner along the z-dimension), and particle-shaped masks, respectively. The fifth column displays the 3D projections of the enhanced and IsoNet missing-wedge-corrected particle. The sixth and seventh columns present the final 3D map and the flexibly fitted model, respectively. (B) Circular schematic representation of a plasmid particle. The outer rim is color-coded to match the corresponding 3D model. Arrowheads indicate the transcriptional direction of bound RNAPs, and circles denote apical sites. Inner circular sectors represent individual plectonemes, with colors indicating writhe density (blue to red scale, −0.3 to 0.1). (C) Resolution assessment of the final 3D map using Fourier shell correlation (FSC). Two criteria are shown: FSC between two half-maps reconstructed from even and odd frames (evaluated at 0.143) and FSC between the final 3D map and the fitted model (evaluated at 0.5). (D) Zoomed-in views of the final 3D density map from panel A, displayed at two contour levels. (E) Superimposition of the high-contour level map from panel D onto its fitted model.

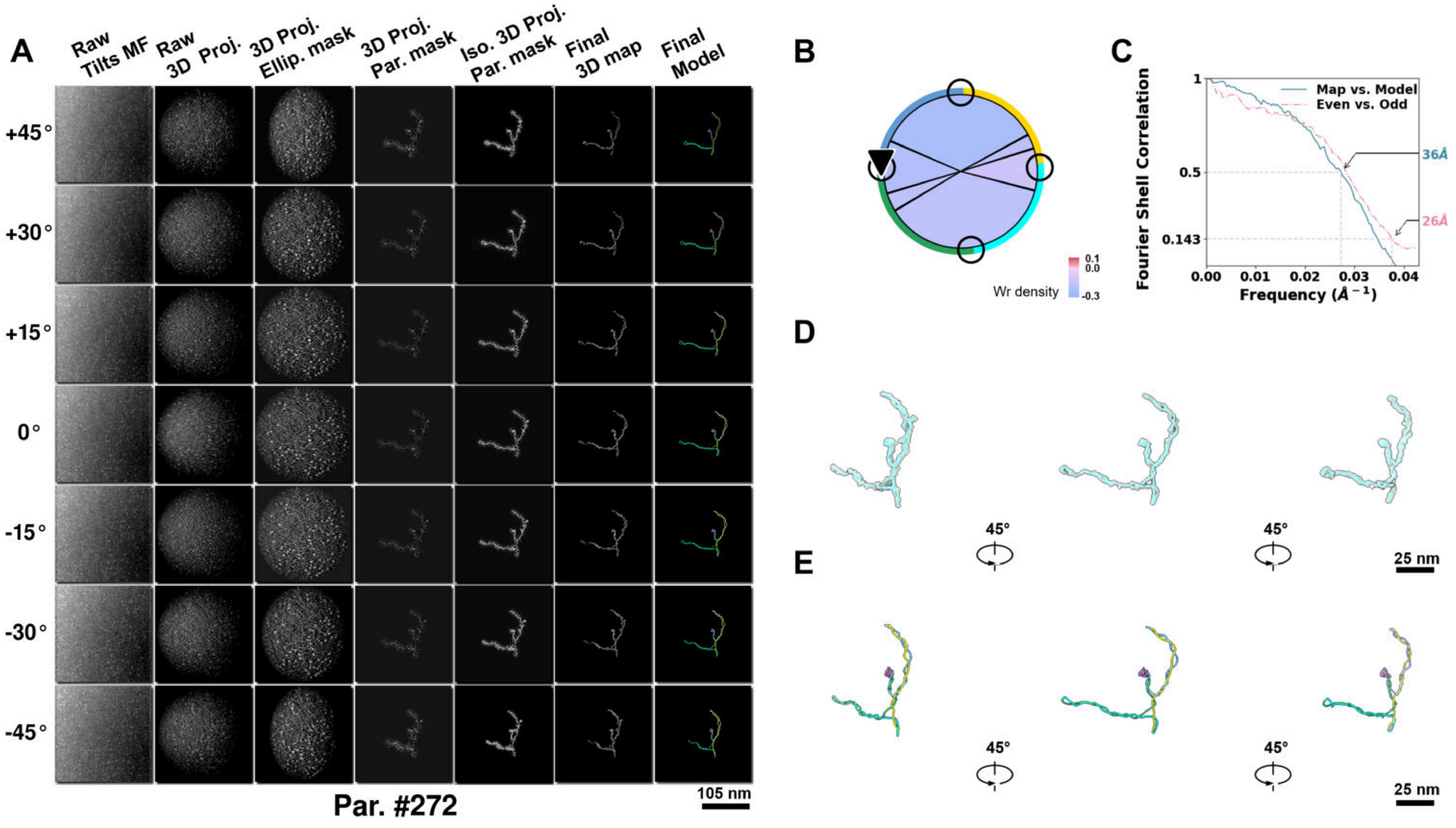

Supplementary Particle Figure 272. Cryo-ET 3D reconstruction of an individual Opp.-TEC particle.
(A) 3D reconstruction of the plasmid particle (index no. 272). The first column shows seven representative tilt images from +45° to -45° in step of 15°. The second, third, and fourth columns show 3D projections of the particle with spherical, ellipsoidal (thinner along the z-dimension), and particle-shaped masks, respectively. The fifth column displays the 3D projections of the enhanced and IsoNet missing-wedge-corrected particle. The sixth and seventh columns present the final 3D map and the flexibly fitted model, respectively. (B) Circular schematic representation of a plasmid particle. The outer rim is color-coded to match the corresponding 3D model. Arrowheads indicate the transcriptional direction of bound RNAPs, and circles denote apical sites. Inner circular sectors represent individual plectonemes, with colors indicating writhe density (blue to red scale, −0.3 to 0.1). (C) Resolution assessment of the final 3D map using Fourier shell correlation (FSC). Two criteria are shown: FSC between two half-maps reconstructed from even and odd frames (evaluated at 0.143) and FSC between the final 3D map and the fitted model (evaluated at 0.5). (D) Zoomed-in views of the final 3D density map from panel A, displayed at two contour levels. (E) Superimposition of the high-contour level map from panel D onto its fitted model.

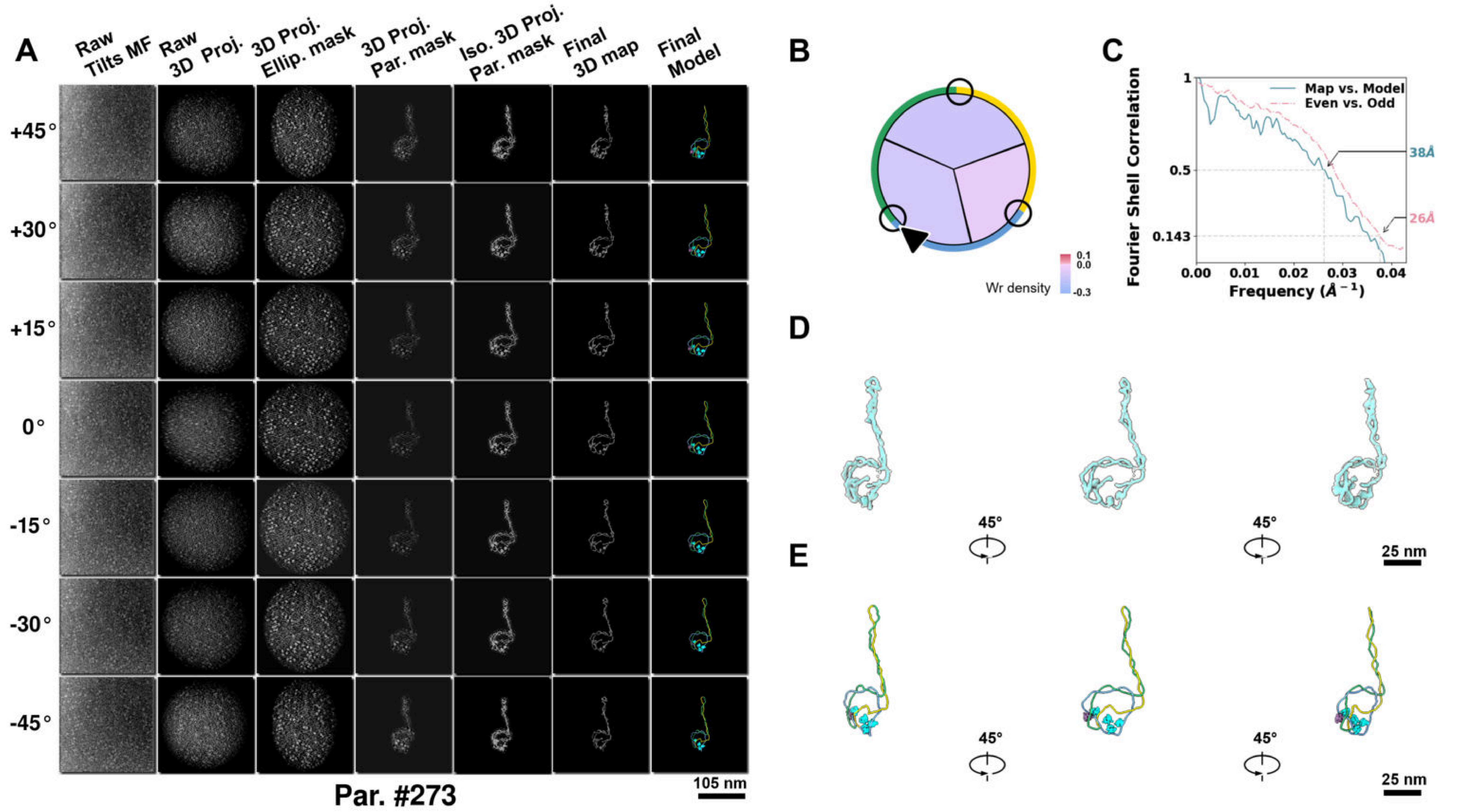

Supplementary Particle Figure 273. Cryo-ET 3D reconstruction of an individual Opp.-TEC particle.
(A) 3D reconstruction of the plasmid particle (index no. 273). The first column shows seven representative tilt images from +45° to -45° in step of 15°. The second, third, and fourth columns show 3D projections of the particle with spherical, ellipsoidal (thinner along the z-dimension), and particle-shaped masks, respectively. The fifth column displays the 3D projections of the enhanced and IsoNet missing-wedge-corrected particle. The sixth and seventh columns present the final 3D map and the flexibly fitted model, respectively. (B) Circular schematic representation of a plasmid particle. The outer rim is color-coded to match the corresponding 3D model. Arrowheads indicate the transcriptional direction of bound RNAPs, and circles denote apical sites. Inner circular sectors represent individual plectonemes, with colors indicating writhe density (blue to red scale, −0.3 to 0.1). (C) Resolution assessment of the final 3D map using Fourier shell correlation (FSC). Two criteria are shown: FSC between two half-maps reconstructed from even and odd frames (evaluated at 0.143) and FSC between the final 3D map and the fitted model (evaluated at 0.5). (D) Zoomed-in views of the final 3D density map from panel A, displayed at two contour levels. (E) Superimposition of the high-contour level map from panel D onto its fitted model.

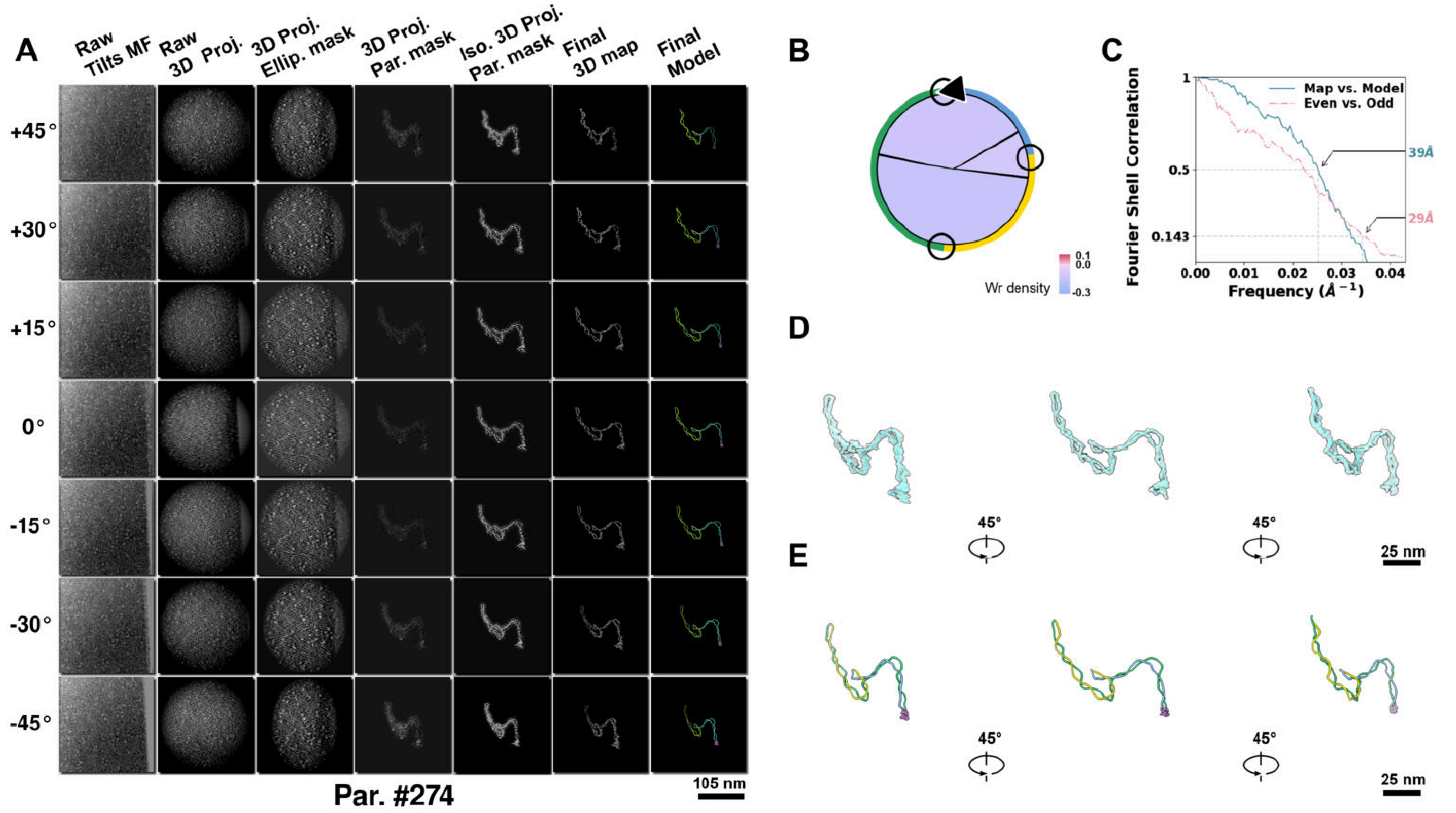

Supplementary Particle Figure 274. Cryo-ET 3D reconstruction of an individual Opp.-TEC particle.
(A) 3D reconstruction of the plasmid particle (index no. 274). The first column shows seven representative tilt images from +45° to -45° in step of 15°. The second, third, and fourth columns show 3D projections of the particle with spherical, ellipsoidal (thinner along the z-dimension), and particle-shaped masks, respectively. The fifth column displays the 3D projections of the enhanced and IsoNet missing-wedge-corrected particle. The sixth and seventh columns present the final 3D map and the flexibly fitted model, respectively. (B) Circular schematic representation of a plasmid particle. The outer rim is color-coded to match the corresponding 3D model. Arrowheads indicate the transcriptional direction of bound RNAPs, and circles denote apical sites. Inner circular sectors represent individual plectonemes, with colors indicating writhe density (blue to red scale, −0.3 to 0.1). (C) Resolution assessment of the final 3D map using Fourier shell correlation (FSC). Two criteria are shown: FSC between two half-maps reconstructed from even and odd frames (evaluated at 0.143) and FSC between the final 3D map and the fitted model (evaluated at 0.5). (D) Zoomed-in views of the final 3D density map from panel A, displayed at two contour levels. (E) Superimposition of the high-contour level map from panel D onto its fitted model.

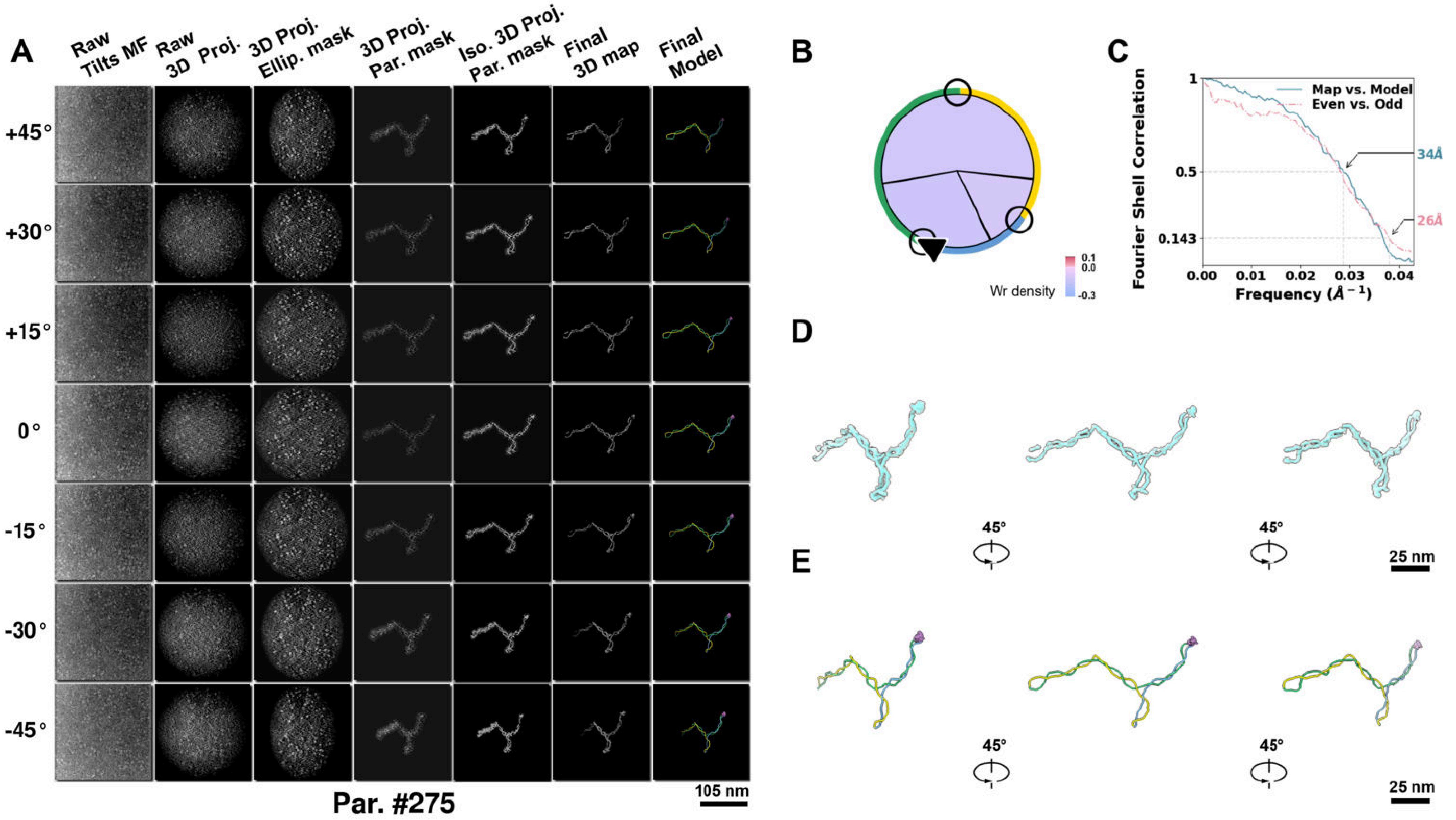

Supplementary Particle Figure 275. Cryo-ET 3D reconstruction of an individual Opp.-TEC particle.
(A) 3D reconstruction of the plasmid particle (index no. 275). The first column shows seven representative tilt images from +45° to -45° in step of 15°. The second, third, and fourth columns show 3D projections of the particle with spherical, ellipsoidal (thinner along the z-dimension), and particle-shaped masks, respectively. The fifth column displays the 3D projections of the enhanced and IsoNet missing-wedge-corrected particle. The sixth and seventh columns present the final 3D map and the flexibly fitted model, respectively. (B) Circular schematic representation of a plasmid particle. The outer rim is color-coded to match the corresponding 3D model. Arrowheads indicate the transcriptional direction of bound RNAPs, and circles denote apical sites. Inner circular sectors represent individual plectonemes, with colors indicating writhe density (blue to red scale, −0.3 to 0.1). (C) Resolution assessment of the final 3D map using Fourier shell correlation (FSC). Two criteria are shown: FSC between two half-maps reconstructed from even and odd frames (evaluated at 0.143) and FSC between the final 3D map and the fitted model (evaluated at 0.5). (D) Zoomed-in views of the final 3D density map from panel A, displayed at two contour levels. (E) Superimposition of the high-contour level map from panel D onto its fitted model.

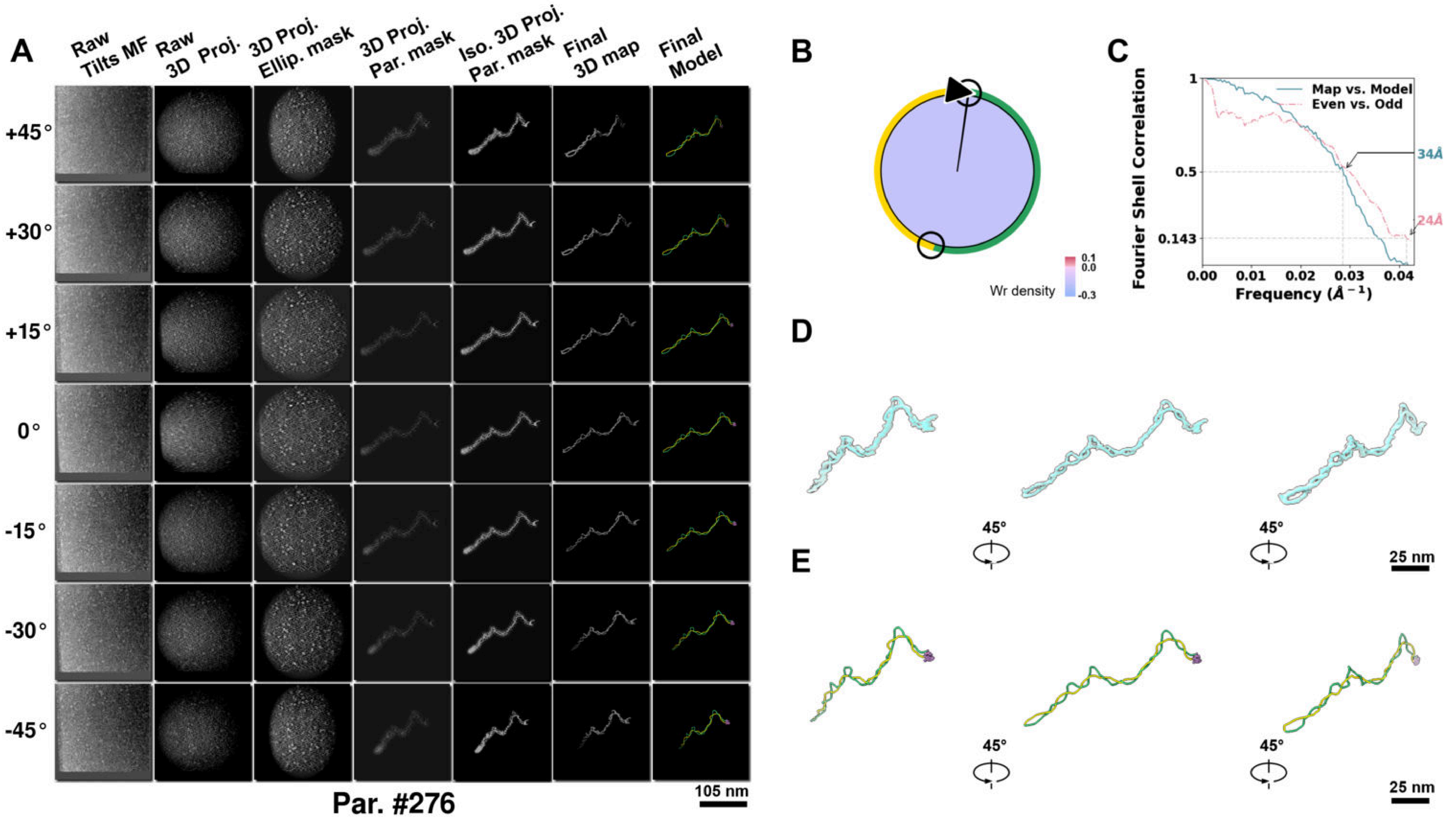

Supplementary Particle Figure 276. Cryo-ET 3D reconstruction of an individual Opp.-TEC particle.
(A) 3D reconstruction of the plasmid particle (index no. 276). The first column shows seven representative tilt images from +45° to -45° in step of 15°. The second, third, and fourth columns show 3D projections of the particle with spherical, ellipsoidal (thinner along the z-dimension), and particle-shaped masks, respectively. The fifth column displays the 3D projections of the enhanced and IsoNet missing-wedge-corrected particle. The sixth and seventh columns present the final 3D map and the flexibly fitted model, respectively. (B) Circular schematic representation of a plasmid particle. The outer rim is color-coded to match the corresponding 3D model. Arrowheads indicate the transcriptional direction of bound RNAPs, and circles denote apical sites. Inner circular sectors represent individual plectonemes, with colors indicating writhe density (blue to red scale, −0.3 to 0.1). (C) Resolution assessment of the final 3D map using Fourier shell correlation (FSC). Two criteria are shown: FSC between two half-maps reconstructed from even and odd frames (evaluated at 0.143) and FSC between the final 3D map and the fitted model (evaluated at 0.5). (D) Zoomed-in views of the final 3D density map from panel A, displayed at two contour levels. (E) Superimposition of the high-contour level map from panel D onto its fitted model.

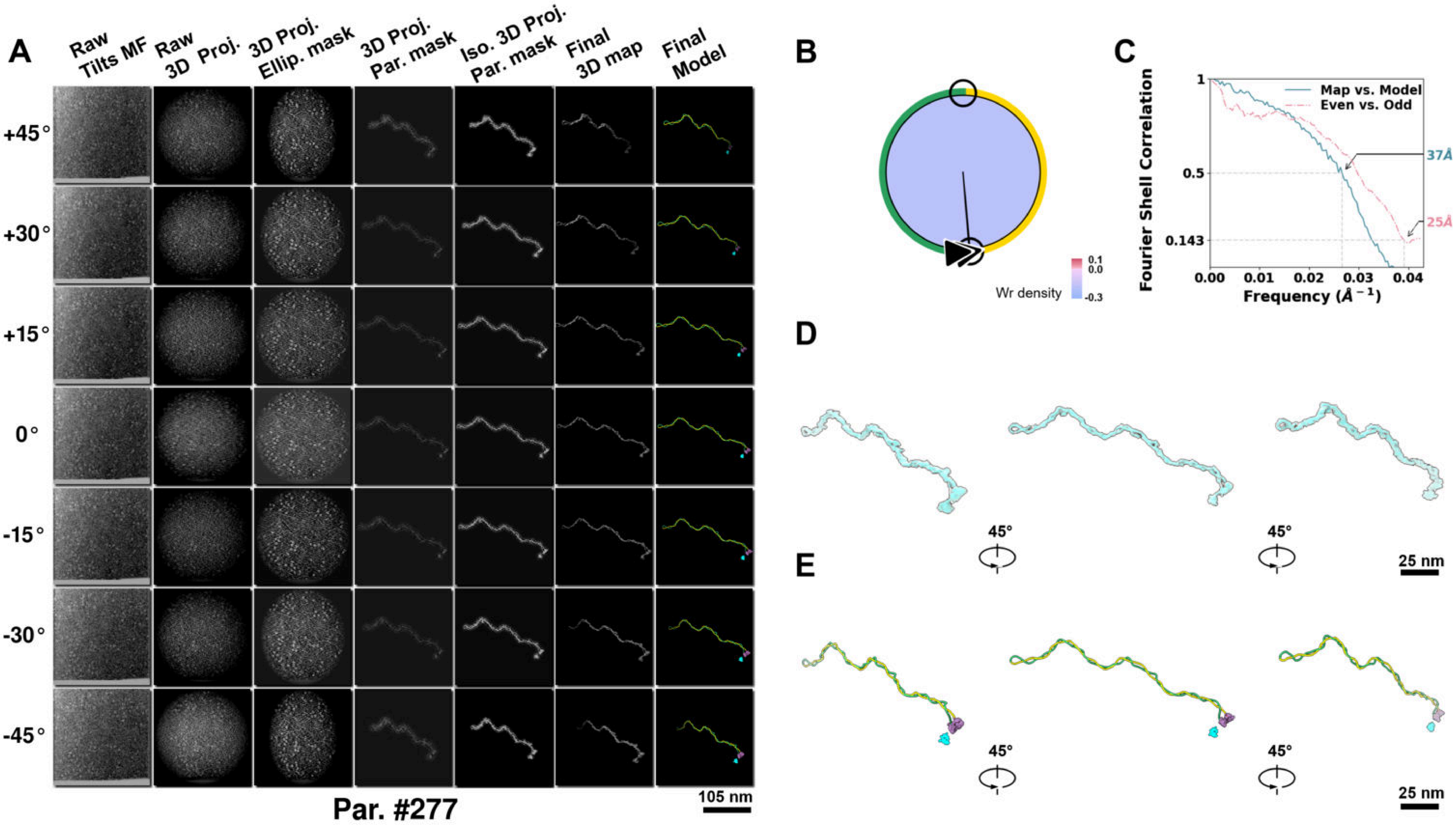

Supplementary Particle Figure 277. Cryo-ET 3D reconstruction of an individual Opp.-TEC particle.
(A) 3D reconstruction of the plasmid particle (index no. 277). The first column shows seven representative tilt images from +45° to -45° in step of 15°. The second, third, and fourth columns show 3D projections of the particle with spherical, ellipsoidal (thinner along the z-dimension), and particle-shaped masks, respectively. The fifth column displays the 3D projections of the enhanced and IsoNet missing-wedge-corrected particle. The sixth and seventh columns present the final 3D map and the flexibly fitted model, respectively. (B) Circular schematic representation of a plasmid particle. The outer rim is color-coded to match the corresponding 3D model. Arrowheads indicate the transcriptional direction of bound RNAPs, and circles denote apical sites. Inner circular sectors represent individual plectonemes, with colors indicating writhe density (blue to red scale, −0.3 to 0.1). (C) Resolution assessment of the final 3D map using Fourier shell correlation (FSC). Two criteria are shown: FSC between two half-maps reconstructed from even and odd frames (evaluated at 0.143) and FSC between the final 3D map and the fitted model (evaluated at 0.5). (D) Zoomed-in views of the final 3D density map from panel A, displayed at two contour levels. (E) Superimposition of the high-contour level map from panel D onto its fitted model.

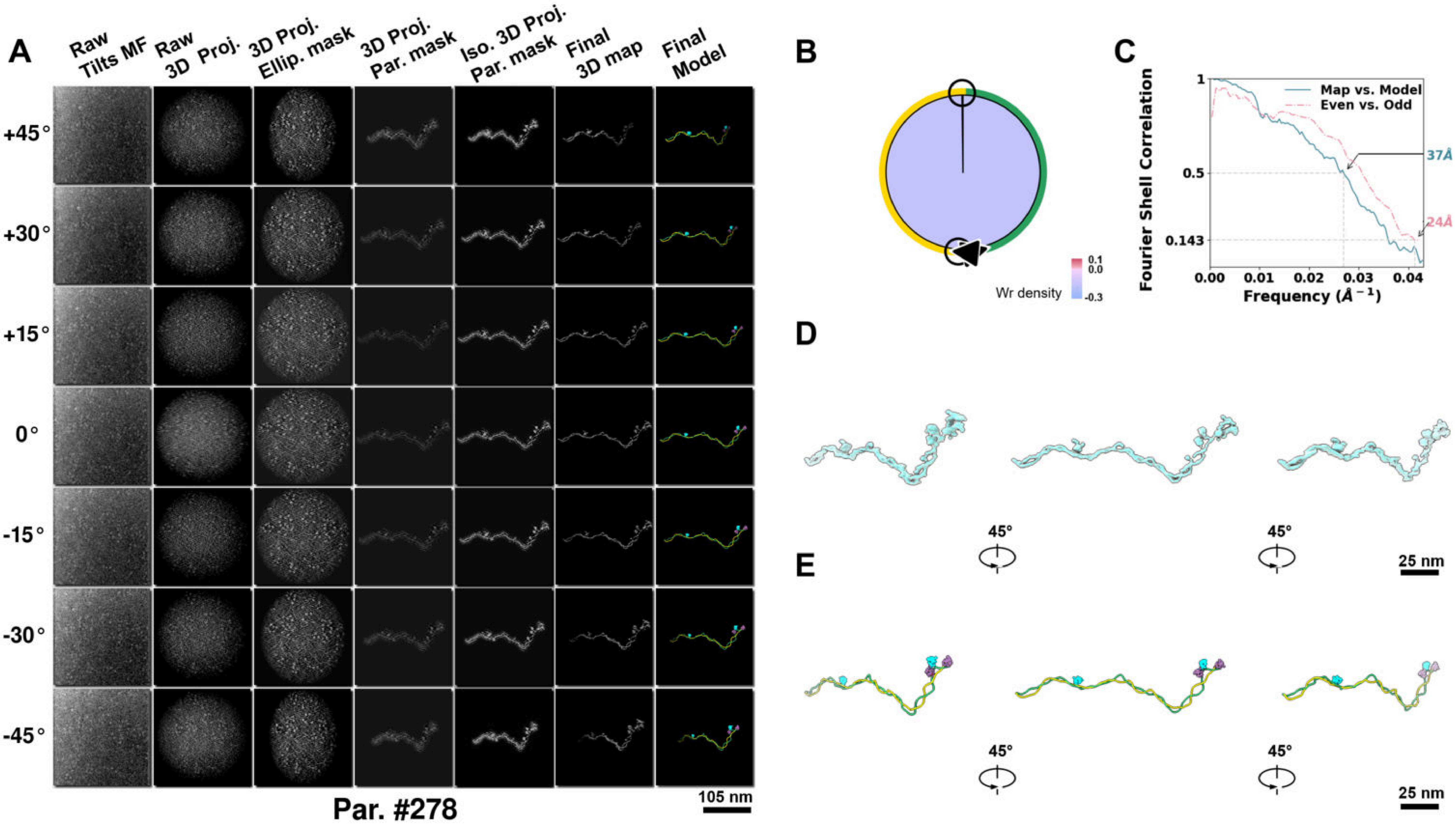

Supplementary Particle Figure 278. Cryo-ET 3D reconstruction of an individual Opp.-TEC particle.
(A) 3D reconstruction of the plasmid particle (index no. 278). The first column shows seven representative tilt images from +45° to -45° in step of 15°. The second, third, and fourth columns show 3D projections of the particle with spherical, ellipsoidal (thinner along the z-dimension), and particle-shaped masks, respectively. The fifth column displays the 3D projections of the enhanced and IsoNet missing-wedge-corrected particle. The sixth and seventh columns present the final 3D map and the flexibly fitted model, respectively. (B) Circular schematic representation of a plasmid particle. The outer rim is color-coded to match the corresponding 3D model. Arrowheads indicate the transcriptional direction of bound RNAPs, and circles denote apical sites. Inner circular sectors represent individual plectonemes, with colors indicating writhe density (blue to red scale, −0.3 to 0.1). (C) Resolution assessment of the final 3D map using Fourier shell correlation (FSC). Two criteria are shown: FSC between two half-maps reconstructed from even and odd frames (evaluated at 0.143) and FSC between the final 3D map and the fitted model (evaluated at 0.5). (D) Zoomed-in views of the final 3D density map from panel A, displayed at two contour levels. (E) Superimposition of the high-contour level map from panel D onto its fitted model.

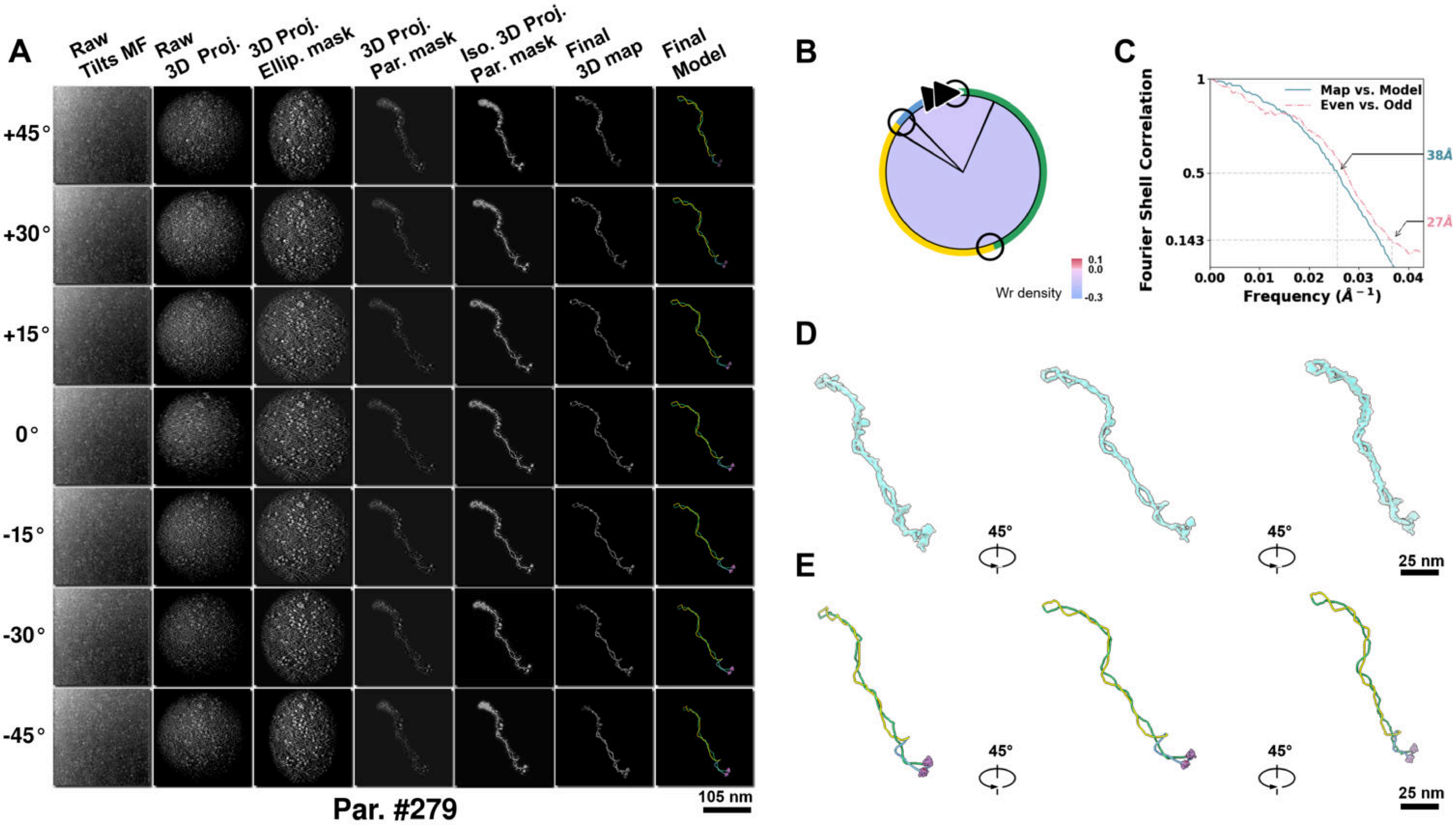

Supplementary Particle Figure 279. Cryo-ET 3D reconstruction of an individual Opp.-TEC particle.
(A) 3D reconstruction of the plasmid particle (index no. 279). The first column shows seven representative tilt images from +45° to -45° in step of 15°. The second, third, and fourth columns show 3D projections of the particle with spherical, ellipsoidal (thinner along the z-dimension), and particle-shaped masks, respectively. The fifth column displays the 3D projections of the enhanced and IsoNet missing-wedge-corrected particle. The sixth and seventh columns present the final 3D map and the flexibly fitted model, respectively. (B) Circular schematic representation of a plasmid particle. The outer rim is color-coded to match the corresponding 3D model. Arrowheads indicate the transcriptional direction of bound RNAPs, and circles denote apical sites. Inner circular sectors represent individual plectonemes, with colors indicating writhe density (blue to red scale, −0.3 to 0.1). (C) Resolution assessment of the final 3D map using Fourier shell correlation (FSC). Two criteria are shown: FSC between two half-maps reconstructed from even and odd frames (evaluated at 0.143) and FSC between the final 3D map and the fitted model (evaluated at 0.5). (D) Zoomed-in views of the final 3D density map from panel A, displayed at two contour levels. (E) Superimposition of the high-contour level map from panel D onto its fitted model.

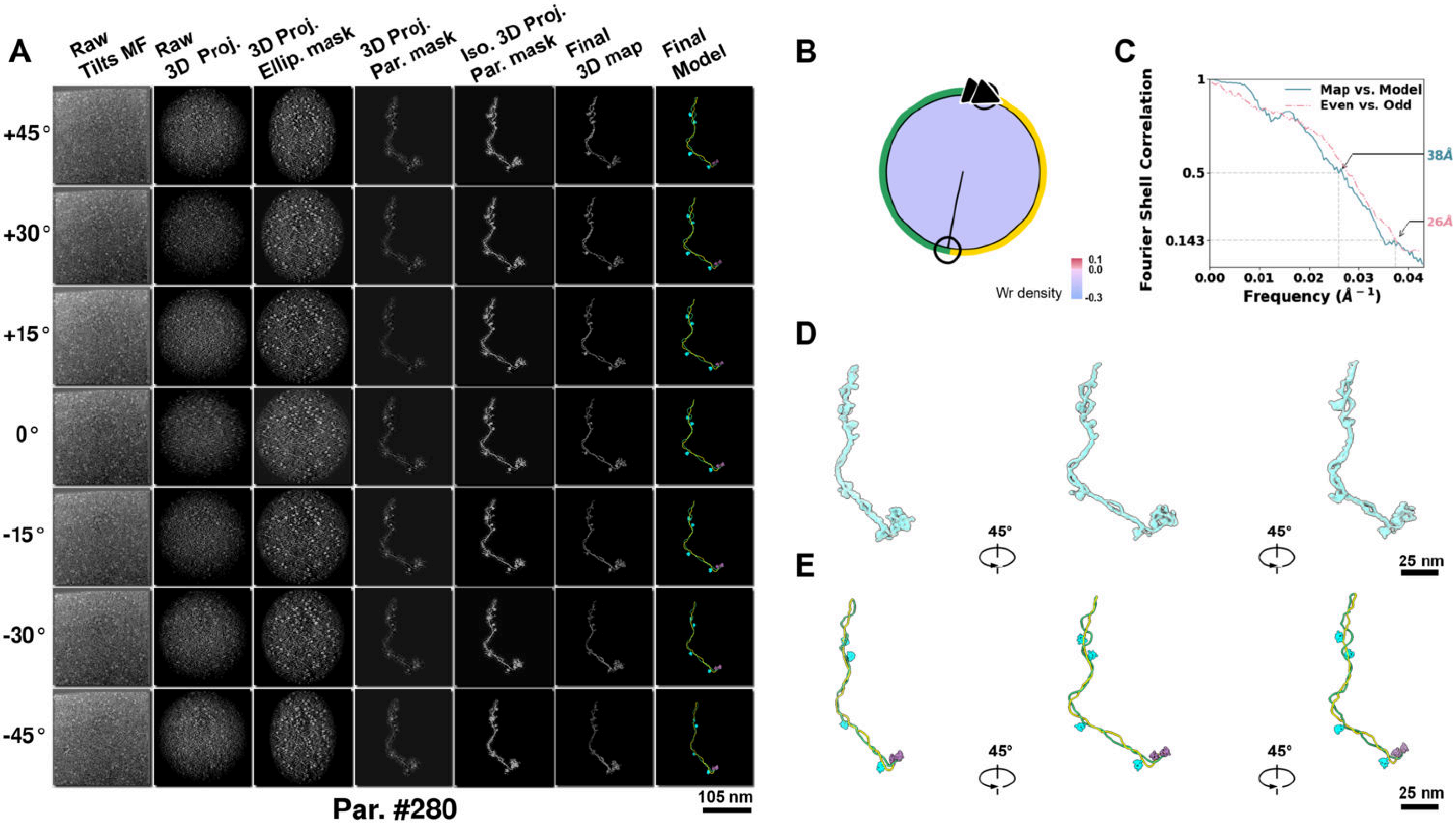

Supplementary Particle Figure 280. Cryo-ET 3D reconstruction of an individual Opp.-TEC particle.
(A) 3D reconstruction of the plasmid particle (index no. 280). The first column shows seven representative tilt images from +45° to -45° in step of 15°. The second, third, and fourth columns show 3D projections of the particle with spherical, ellipsoidal (thinner along the z-dimension), and particle-shaped masks, respectively. The fifth column displays the 3D projections of the enhanced and IsoNet missing-wedge-corrected particle. The sixth and seventh columns present the final 3D map and the flexibly fitted model, respectively. (B) Circular schematic representation of a plasmid particle. The outer rim is color-coded to match the corresponding 3D model. Arrowheads indicate the transcriptional direction of bound RNAPs, and circles denote apical sites. Inner circular sectors represent individual plectonemes, with colors indicating writhe density (blue to red scale, −0.3 to 0.1). (C) Resolution assessment of the final 3D map using Fourier shell correlation (FSC). Two criteria are shown: FSC between two half-maps reconstructed from even and odd frames (evaluated at 0.143) and FSC between the final 3D map and the fitted model (evaluated at 0.5). (D) Zoomed-in views of the final 3D density map from panel A, displayed at two contour levels. (E) Superimposition of the high-contour level map from panel D onto its fitted model.

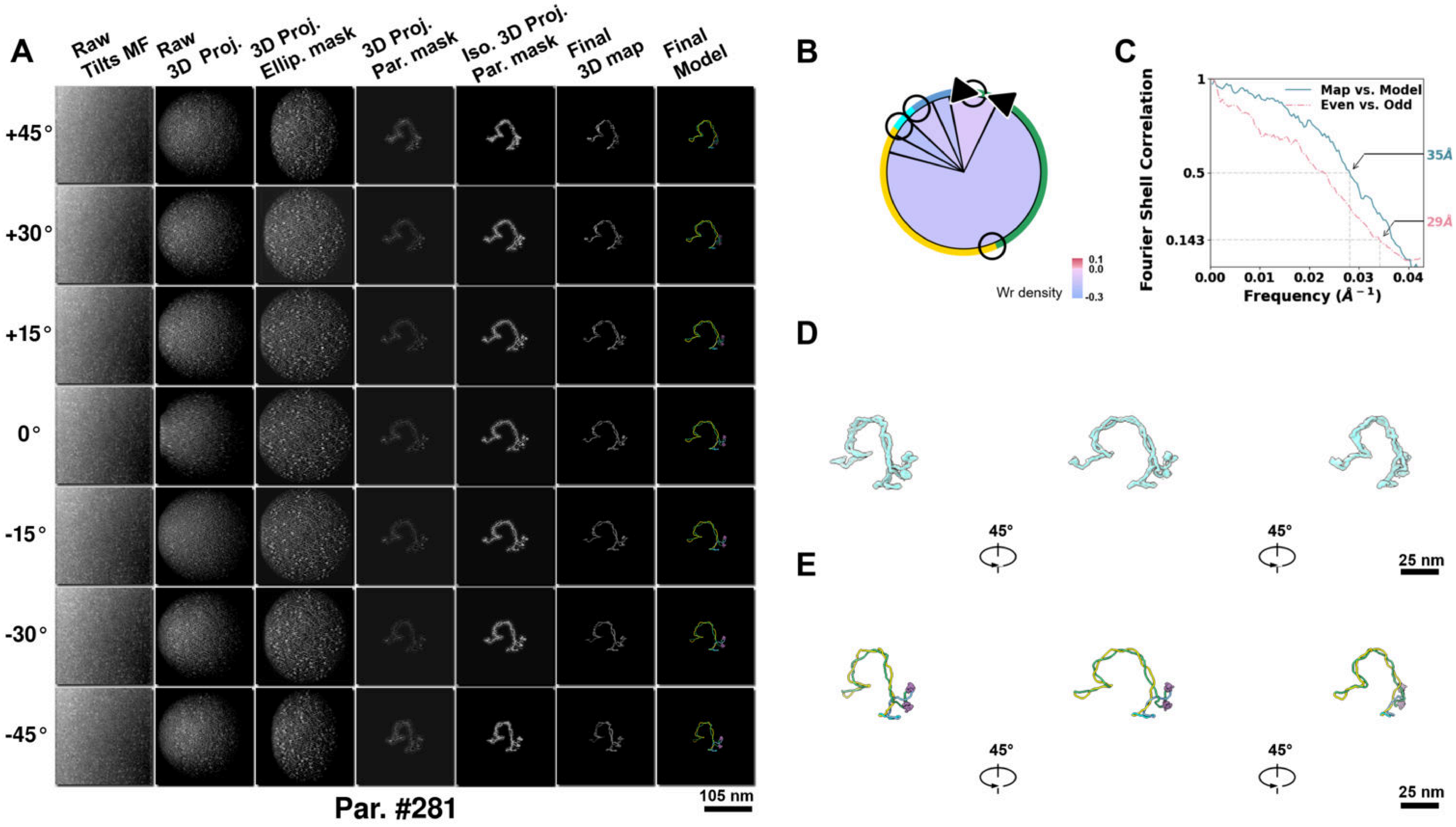

Supplementary Particle Figure 281. Cryo-ET 3D reconstruction of an individual Opp.-TEC particle.
(A) 3D reconstruction of the plasmid particle (index no. 281). The first column shows seven representative tilt images from +45° to -45° in step of 15°. The second, third, and fourth columns show 3D projections of the particle with spherical, ellipsoidal (thinner along the z-dimension), and particle-shaped masks, respectively. The fifth column displays the 3D projections of the enhanced and IsoNet missing-wedge-corrected particle. The sixth and seventh columns present the final 3D map and the flexibly fitted model, respectively. (B) Circular schematic representation of a plasmid particle. The outer rim is color-coded to match the corresponding 3D model. Arrowheads indicate the transcriptional direction of bound RNAPs, and circles denote apical sites. Inner circular sectors represent individual plectonemes, with colors indicating writhe density (blue to red scale, −0.3 to 0.1). (C) Resolution assessment of the final 3D map using Fourier shell correlation (FSC). Two criteria are shown: FSC between two half-maps reconstructed from even and odd frames (evaluated at 0.143) and FSC between the final 3D map and the fitted model (evaluated at 0.5). (D) Zoomed-in views of the final 3D density map from panel A, displayed at two contour levels. (E) Superimposition of the high-contour level map from panel D onto its fitted model.

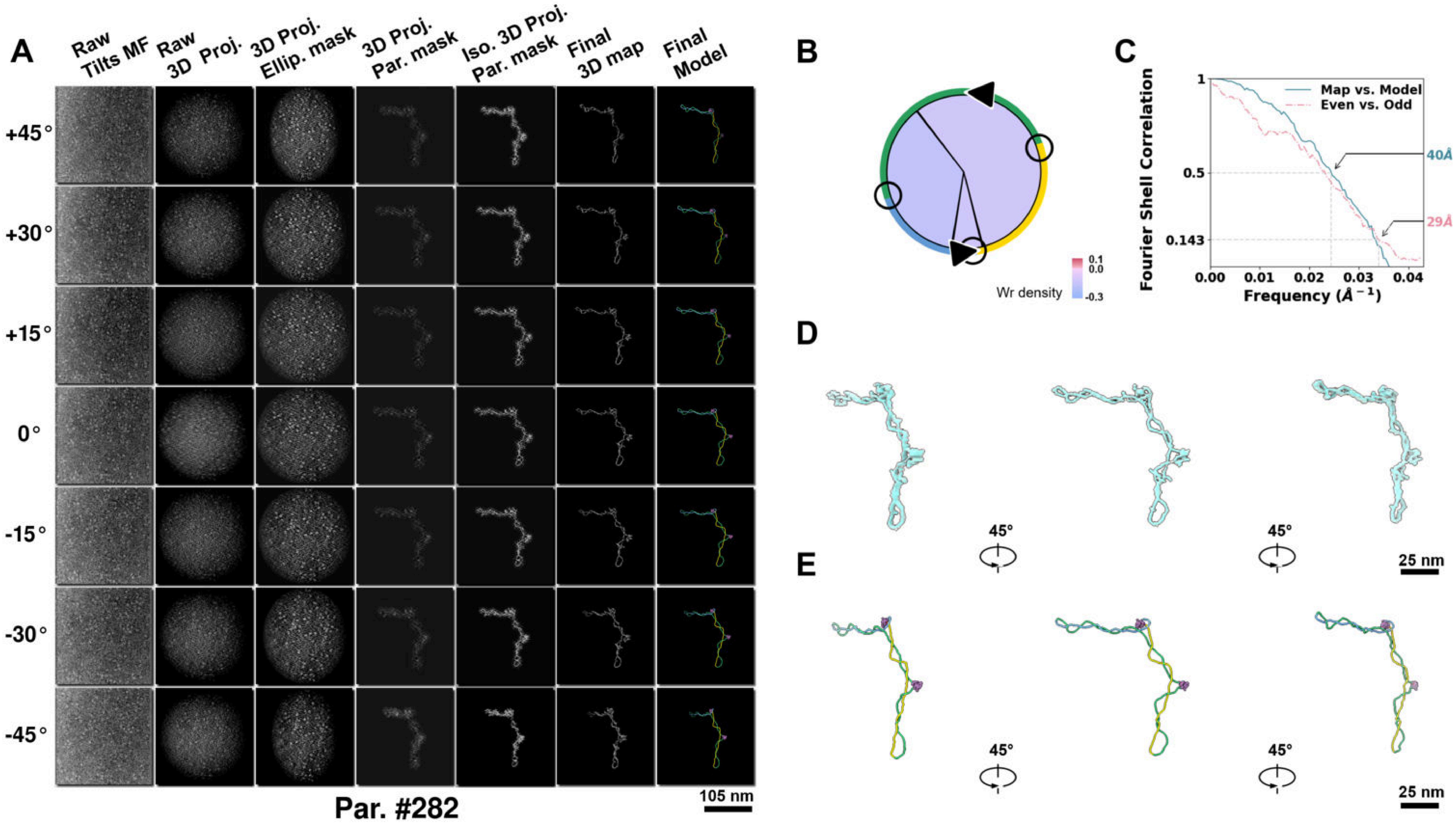

Supplementary Particle Figure 282. Cryo-ET 3D reconstruction of an individual Opp.-TEC particle.
(A) 3D reconstruction of the plasmid particle (index no. 282). The first column shows seven representative tilt images from +45° to -45° in step of 15°. The second, third, and fourth columns show 3D projections of the particle with spherical, ellipsoidal (thinner along the z-dimension), and particle-shaped masks, respectively. The fifth column displays the 3D projections of the enhanced and IsoNet missing-wedge-corrected particle. The sixth and seventh columns present the final 3D map and the flexibly fitted model, respectively. (B) Circular schematic representation of a plasmid particle. The outer rim is color-coded to match the corresponding 3D model. Arrowheads indicate the transcriptional direction of bound RNAPs, and circles denote apical sites. Inner circular sectors represent individual plectonemes, with colors indicating writhe density (blue to red scale, −0.3 to 0.1). (C) Resolution assessment of the final 3D map using Fourier shell correlation (FSC). Two criteria are shown: FSC between two half-maps reconstructed from even and odd frames (evaluated at 0.143) and FSC between the final 3D map and the fitted model (evaluated at 0.5). (D) Zoomed-in views of the final 3D density map from panel A, displayed at two contour levels. (E) Superimposition of the high-contour level map from panel D onto its fitted model.

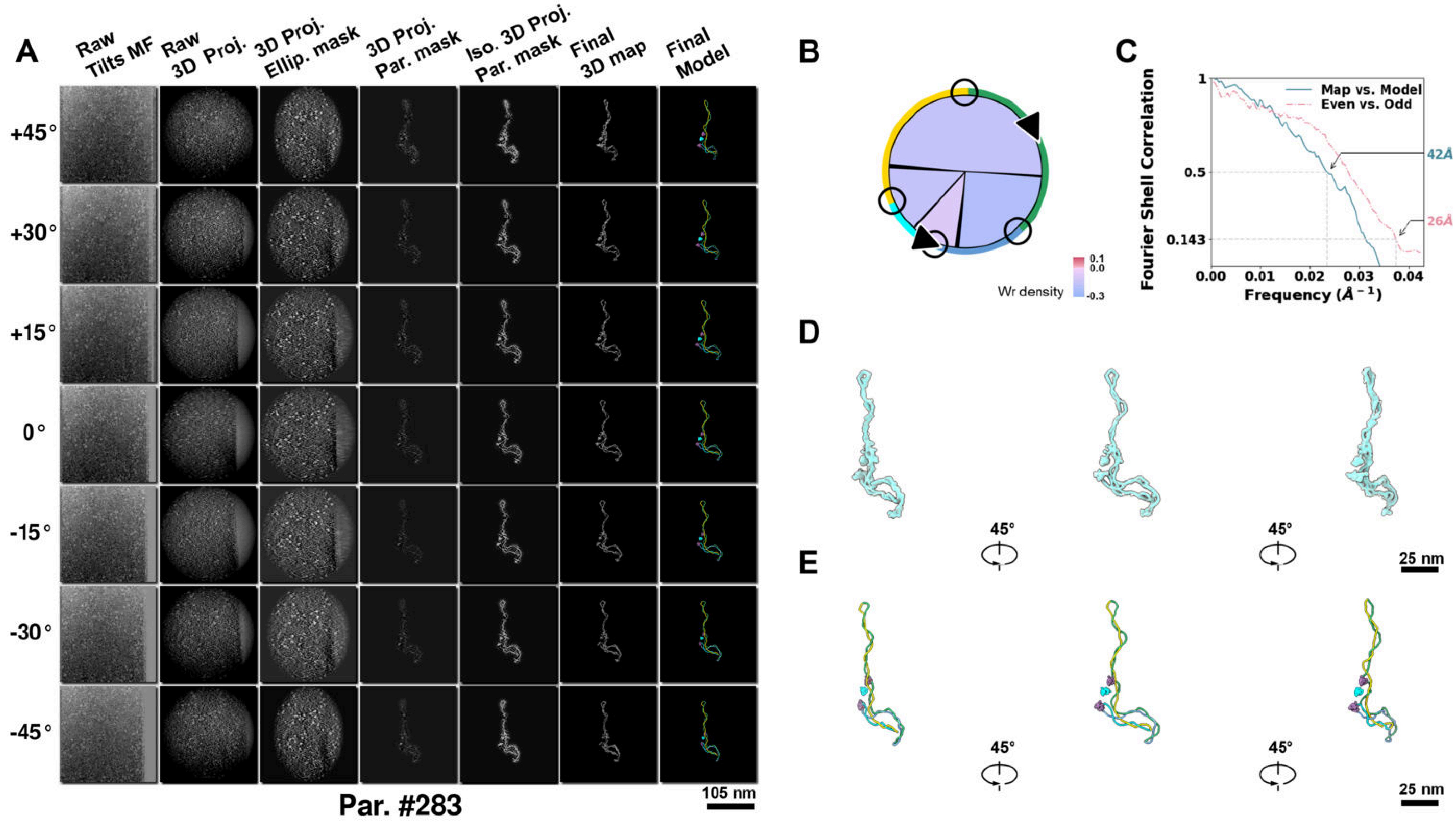

Supplementary Particle Figure 283. Cryo-ET 3D reconstruction of an individual Opp.-TEC particle.
(A) 3D reconstruction of the plasmid particle (index no. 283). The first column shows seven representative tilt images from +45° to -45° in step of 15°. The second, third, and fourth columns show 3D projections of the particle with spherical, ellipsoidal (thinner along the z-dimension), and particle-shaped masks, respectively. The fifth column displays the 3D projections of the enhanced and IsoNet missing-wedge-corrected particle. The sixth and seventh columns present the final 3D map and the flexibly fitted model, respectively. (B) Circular schematic representation of a plasmid particle. The outer rim is color-coded to match the corresponding 3D model. Arrowheads indicate the transcriptional direction of bound RNAPs, and circles denote apical sites. Inner circular sectors represent individual plectonemes, with colors indicating writhe density (blue to red scale, −0.3 to 0.1). (C) Resolution assessment of the final 3D map using Fourier shell correlation (FSC). Two criteria are shown: FSC between two half-maps reconstructed from even and odd frames (evaluated at 0.143) and FSC between the final 3D map and the fitted model (evaluated at 0.5). (D) Zoomed-in views of the final 3D density map from panel A, displayed at two contour levels. (E) Superimposition of the high-contour level map from panel D onto its fitted model.

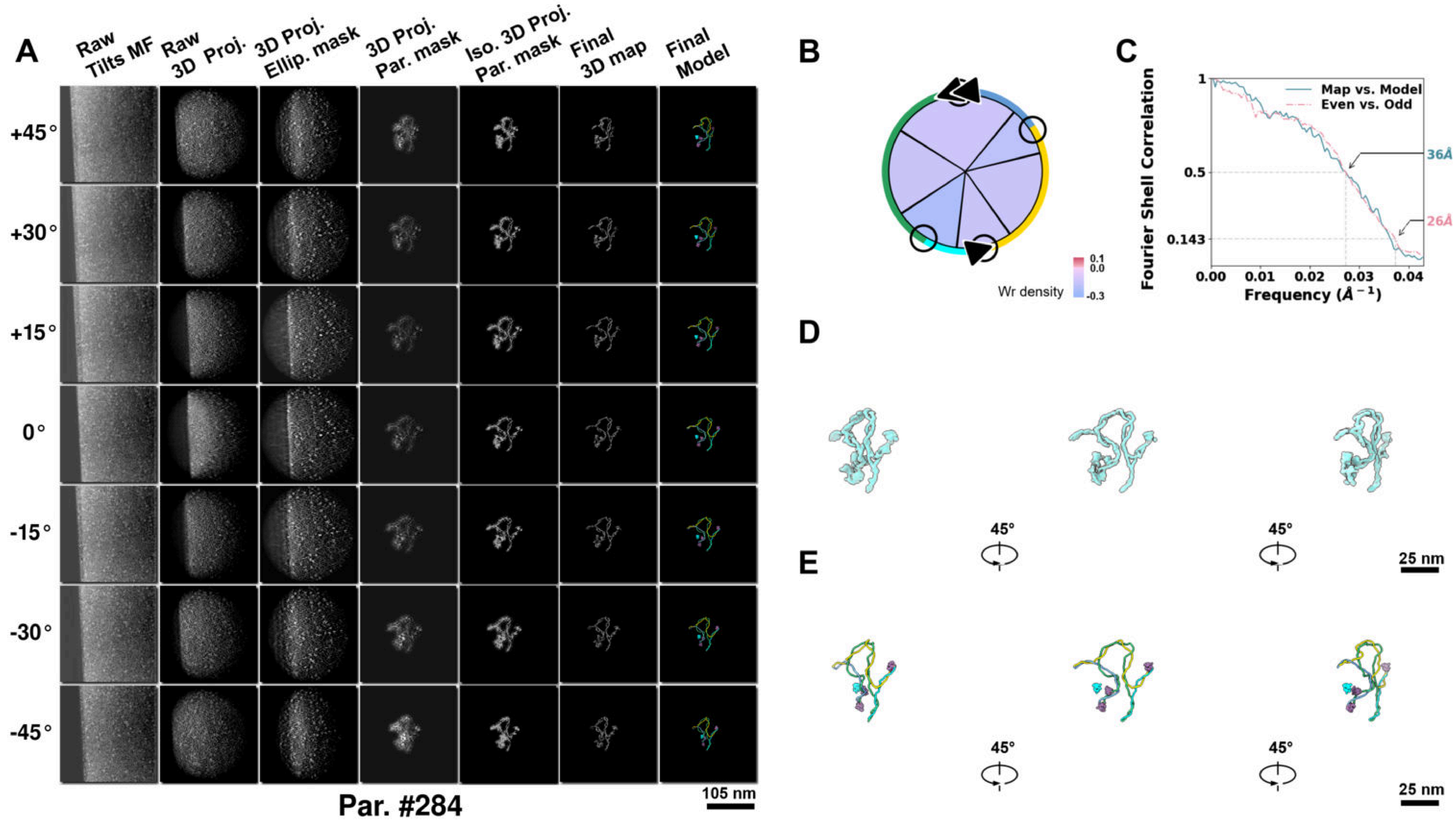

Supplementary Particle Figure 284. Cryo-ET 3D reconstruction of an individual Opp.-TEC particle.
(A) 3D reconstruction of the plasmid particle (index no. 284). The first column shows seven representative tilt images from +45° to -45° in step of 15°. The second, third, and fourth columns show 3D projections of the particle with spherical, ellipsoidal (thinner along the z-dimension), and particle-shaped masks, respectively. The fifth column displays the 3D projections of the enhanced and IsoNet missing-wedge-corrected particle. The sixth and seventh columns present the final 3D map and the flexibly fitted model, respectively. (B) Circular schematic representation of a plasmid particle. The outer rim is color-coded to match the corresponding 3D model. Arrowheads indicate the transcriptional direction of bound RNAPs, and circles denote apical sites. Inner circular sectors represent individual plectonemes, with colors indicating writhe density (blue to red scale, −0.3 to 0.1). (C) Resolution assessment of the final 3D map using Fourier shell correlation (FSC). Two criteria are shown: FSC between two half-maps reconstructed from even and odd frames (evaluated at 0.143) and FSC between the final 3D map and the fitted model (evaluated at 0.5). (D) Zoomed-in views of the final 3D density map from panel A, displayed at two contour levels. (E) Superimposition of the high-contour level map from panel D onto its fitted model.

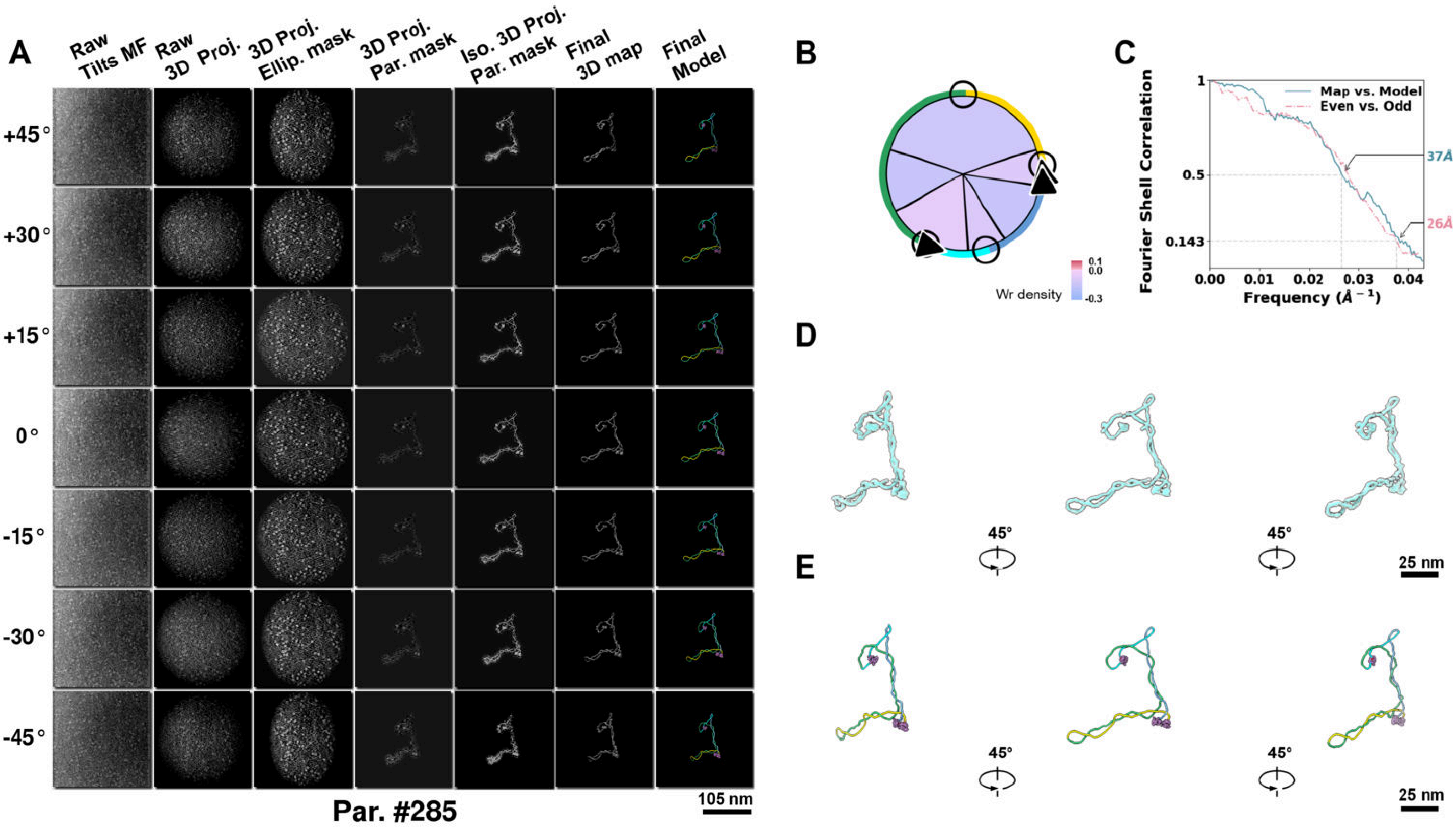

Supplementary Particle Figure 285. Cryo-ET 3D reconstruction of an individual Opp.-TEC particle.
(A) 3D reconstruction of the plasmid particle (index no. 285). The first column shows seven representative tilt images from +45° to -45° in step of 15°. The second, third, and fourth columns show 3D projections of the particle with spherical, ellipsoidal (thinner along the z-dimension), and particle-shaped masks, respectively. The fifth column displays the 3D projections of the enhanced and IsoNet missing-wedge-corrected particle. The sixth and seventh columns present the final 3D map and the flexibly fitted model, respectively. (B) Circular schematic representation of a plasmid particle. The outer rim is color-coded to match the corresponding 3D model. Arrowheads indicate the transcriptional direction of bound RNAPs, and circles denote apical sites. Inner circular sectors represent individual plectonemes, with colors indicating writhe density (blue to red scale, −0.3 to 0.1). (C) Resolution assessment of the final 3D map using Fourier shell correlation (FSC). Two criteria are shown: FSC between two half-maps reconstructed from even and odd frames (evaluated at 0.143) and FSC between the final 3D map and the fitted model (evaluated at 0.5). (D) Zoomed-in views of the final 3D density map from panel A, displayed at two contour levels. (E) Superimposition of the high-contour level map from panel D onto its fitted model.

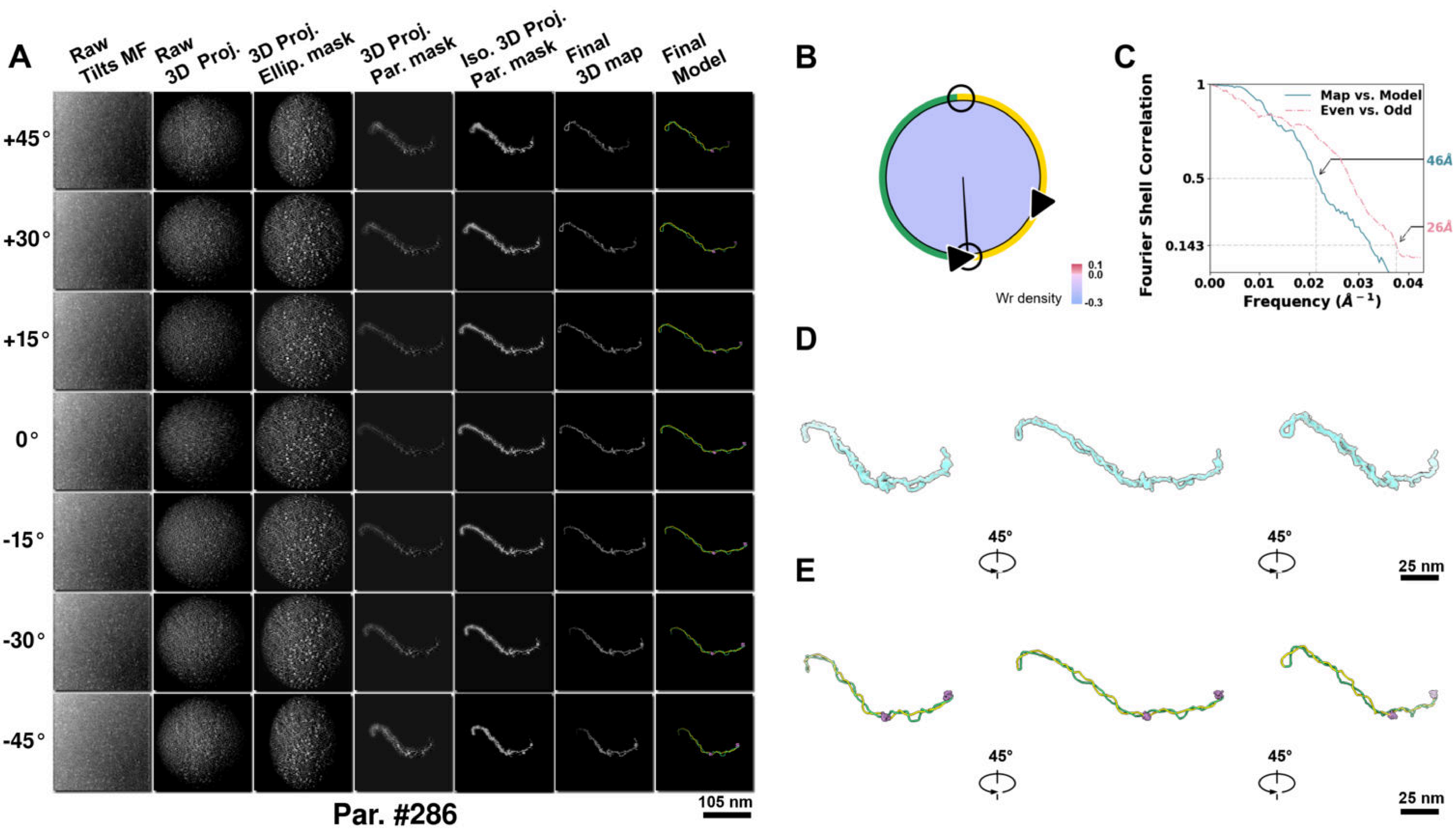

Supplementary Particle Figure 286. Cryo-ET 3D reconstruction of an individual Opp.-TEC particle.
(A) 3D reconstruction of the plasmid particle (index no. 286). The first column shows seven representative tilt images from +45° to -45° in step of 15°. The second, third, and fourth columns show 3D projections of the particle with spherical, ellipsoidal (thinner along the z-dimension), and particle-shaped masks, respectively. The fifth column displays the 3D projections of the enhanced and IsoNet missing-wedge-corrected particle. The sixth and seventh columns present the final 3D map and the flexibly fitted model, respectively. (B) Circular schematic representation of a plasmid particle. The outer rim is color-coded to match the corresponding 3D model. Arrowheads indicate the transcriptional direction of bound RNAPs, and circles denote apical sites. Inner circular sectors represent individual plectonemes, with colors indicating writhe density (blue to red scale, −0.3 to 0.1). (C) Resolution assessment of the final 3D map using Fourier shell correlation (FSC). Two criteria are shown: FSC between two half-maps reconstructed from even and odd frames (evaluated at 0.143) and FSC between the final 3D map and the fitted model (evaluated at 0.5). (D) Zoomed-in views of the final 3D density map from panel A, displayed at two contour levels. (E) Superimposition of the high-contour level map from panel D onto its fitted model.

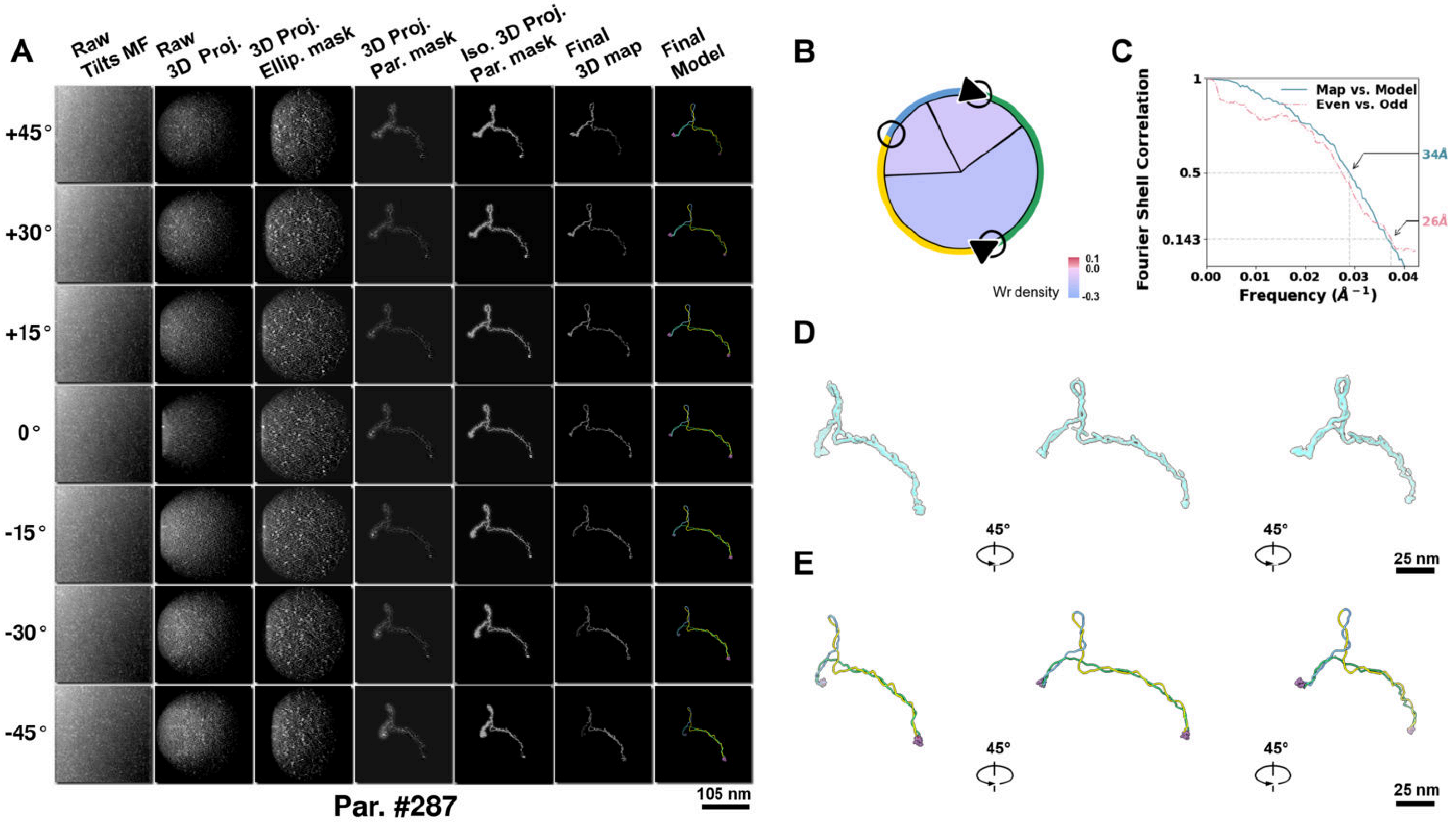

Supplementary Particle Figure 287. Cryo-ET 3D reconstruction of an individual Opp.-TEC particle.
(A) 3D reconstruction of the plasmid particle (index no. 287). The first column shows seven representative tilt images from +45° to -45° in step of 15°. The second, third, and fourth columns show 3D projections of the particle with spherical, ellipsoidal (thinner along the z-dimension), and particle-shaped masks, respectively. The fifth column displays the 3D projections of the enhanced and IsoNet missing-wedge-corrected particle. The sixth and seventh columns present the final 3D map and the flexibly fitted model, respectively. (B) Circular schematic representation of a plasmid particle. The outer rim is color-coded to match the corresponding 3D model. Arrowheads indicate the transcriptional direction of bound RNAPs, and circles denote apical sites. Inner circular sectors represent individual plectonemes, with colors indicating writhe density (blue to red scale, −0.3 to 0.1). (C) Resolution assessment of the final 3D map using Fourier shell correlation (FSC). Two criteria are shown: FSC between two half-maps reconstructed from even and odd frames (evaluated at 0.143) and FSC between the final 3D map and the fitted model (evaluated at 0.5). (D) Zoomed-in views of the final 3D density map from panel A, displayed at two contour levels. (E) Superimposition of the high-contour level map from panel D onto its fitted model.

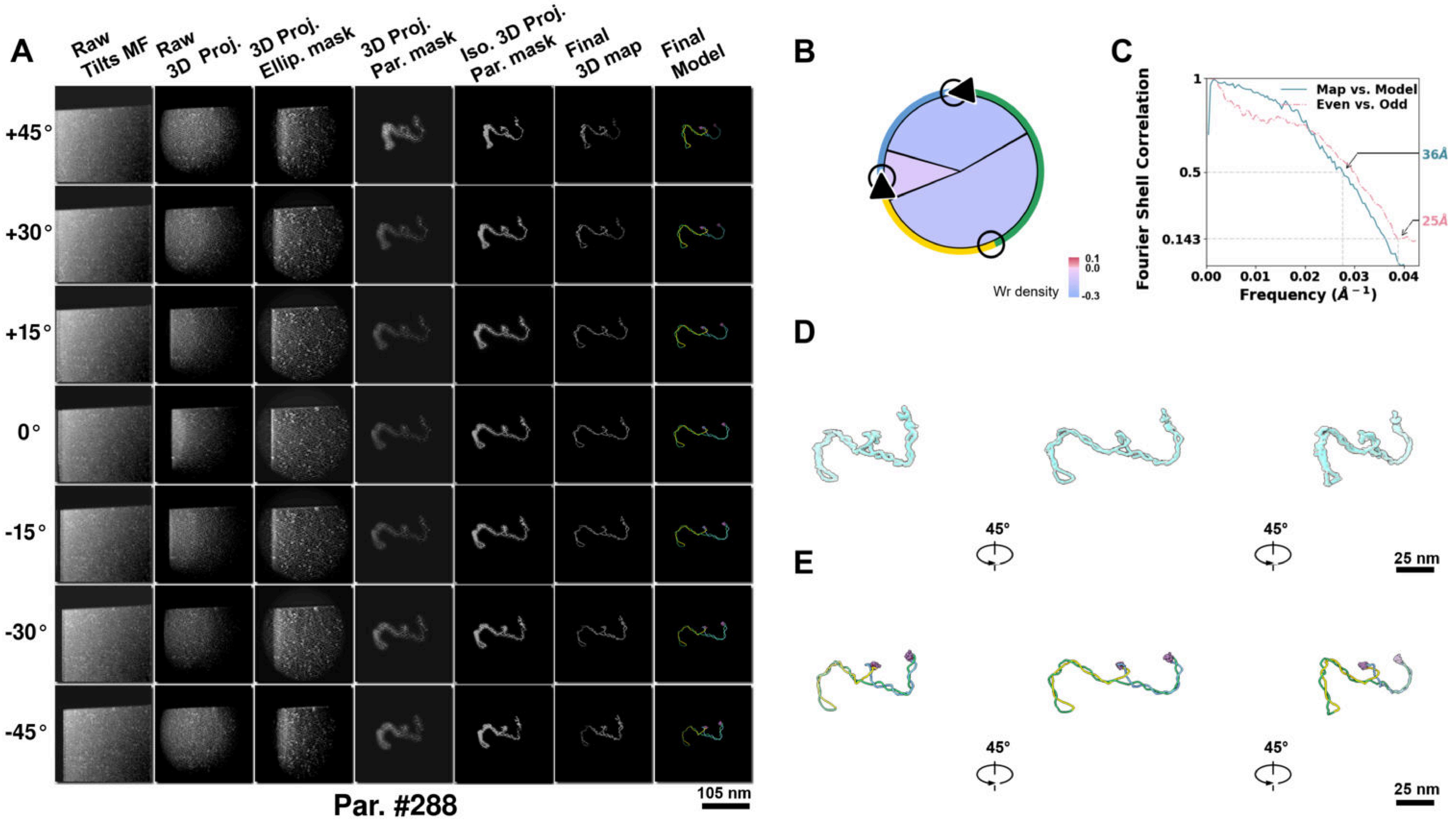

Supplementary Particle Figure 288. Cryo-ET 3D reconstruction of an individual Opp.-TEC particle.
(A) 3D reconstruction of the plasmid particle (index no. 288). The first column shows seven representative tilt images from +45° to -45° in step of 15°. The second, third, and fourth columns show 3D projections of the particle with spherical, ellipsoidal (thinner along the z-dimension), and particle-shaped masks, respectively. The fifth column displays the 3D projections of the enhanced and IsoNet missing-wedge-corrected particle. The sixth and seventh columns present the final 3D map and the flexibly fitted model, respectively. (B) Circular schematic representation of a plasmid particle. The outer rim is color-coded to match the corresponding 3D model. Arrowheads indicate the transcriptional direction of bound RNAPs, and circles denote apical sites. Inner circular sectors represent individual plectonemes, with colors indicating writhe density (blue to red scale, −0.3 to 0.1). (C) Resolution assessment of the final 3D map using Fourier shell correlation (FSC). Two criteria are shown: FSC between two half-maps reconstructed from even and odd frames (evaluated at 0.143) and FSC between the final 3D map and the fitted model (evaluated at 0.5). (D) Zoomed-in views of the final 3D density map from panel A, displayed at two contour levels. (E) Superimposition of the high-contour level map from panel D onto its fitted model.

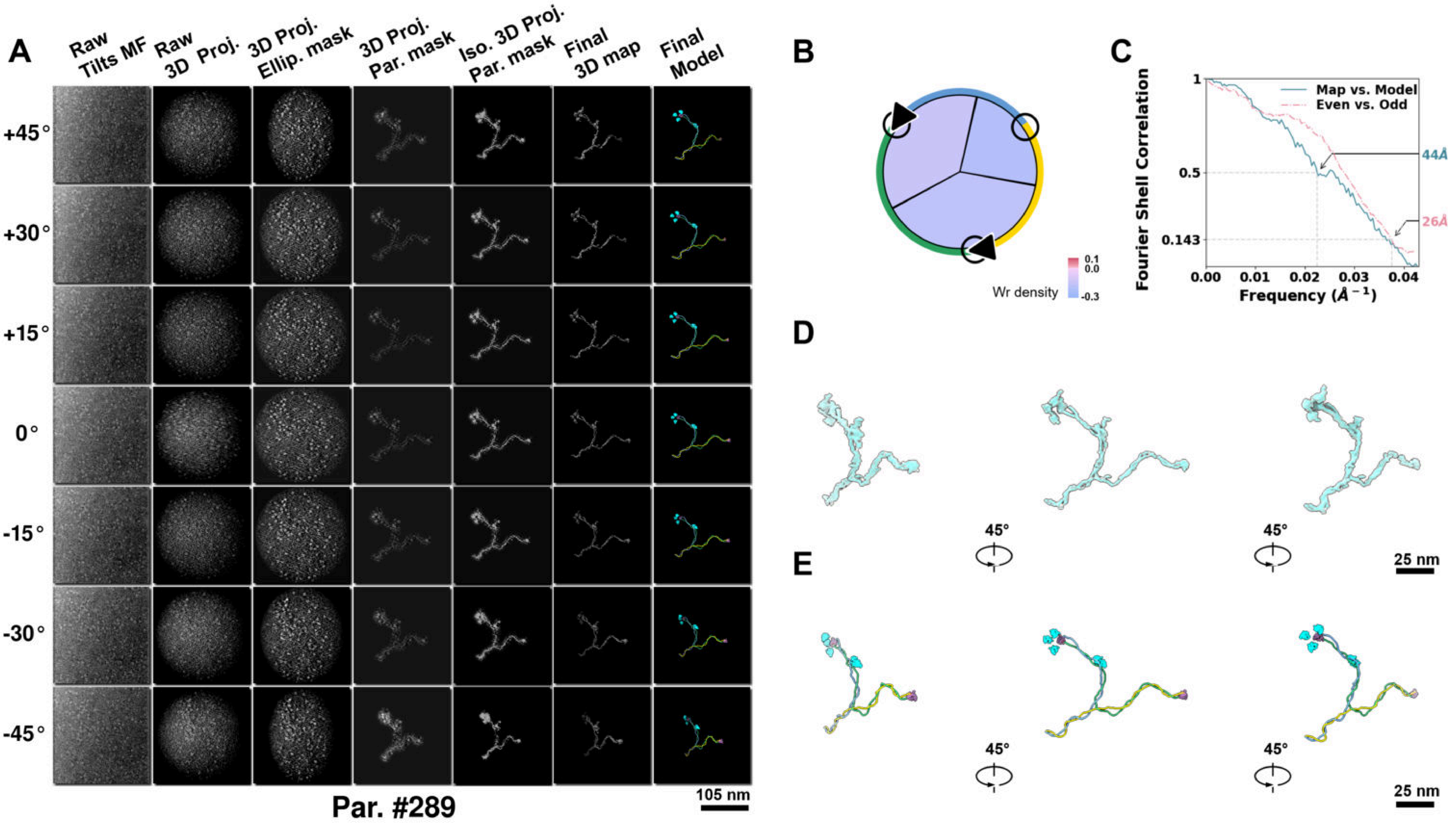

Supplementary Particle Figure 289. Cryo-ET 3D reconstruction of an individual Opp.-TEC particle.
(A) 3D reconstruction of the plasmid particle (index no. 289). The first column shows seven representative tilt images from +45° to -45° in step of 15°. The second, third, and fourth columns show 3D projections of the particle with spherical, ellipsoidal (thinner along the z-dimension), and particle-shaped masks, respectively. The fifth column displays the 3D projections of the enhanced and IsoNet missing-wedge-corrected particle. The sixth and seventh columns present the final 3D map and the flexibly fitted model, respectively. (B) Circular schematic representation of a plasmid particle. The outer rim is color-coded to match the corresponding 3D model. Arrowheads indicate the transcriptional direction of bound RNAPs, and circles denote apical sites. Inner circular sectors represent individual plectonemes, with colors indicating writhe density (blue to red scale, −0.3 to 0.1). (C) Resolution assessment of the final 3D map using Fourier shell correlation (FSC). Two criteria are shown: FSC between two half-maps reconstructed from even and odd frames (evaluated at 0.143) and FSC between the final 3D map and the fitted model (evaluated at 0.5). (D) Zoomed-in views of the final 3D density map from panel A, displayed at two contour levels. (E) Superimposition of the high-contour level map from panel D onto its fitted model.

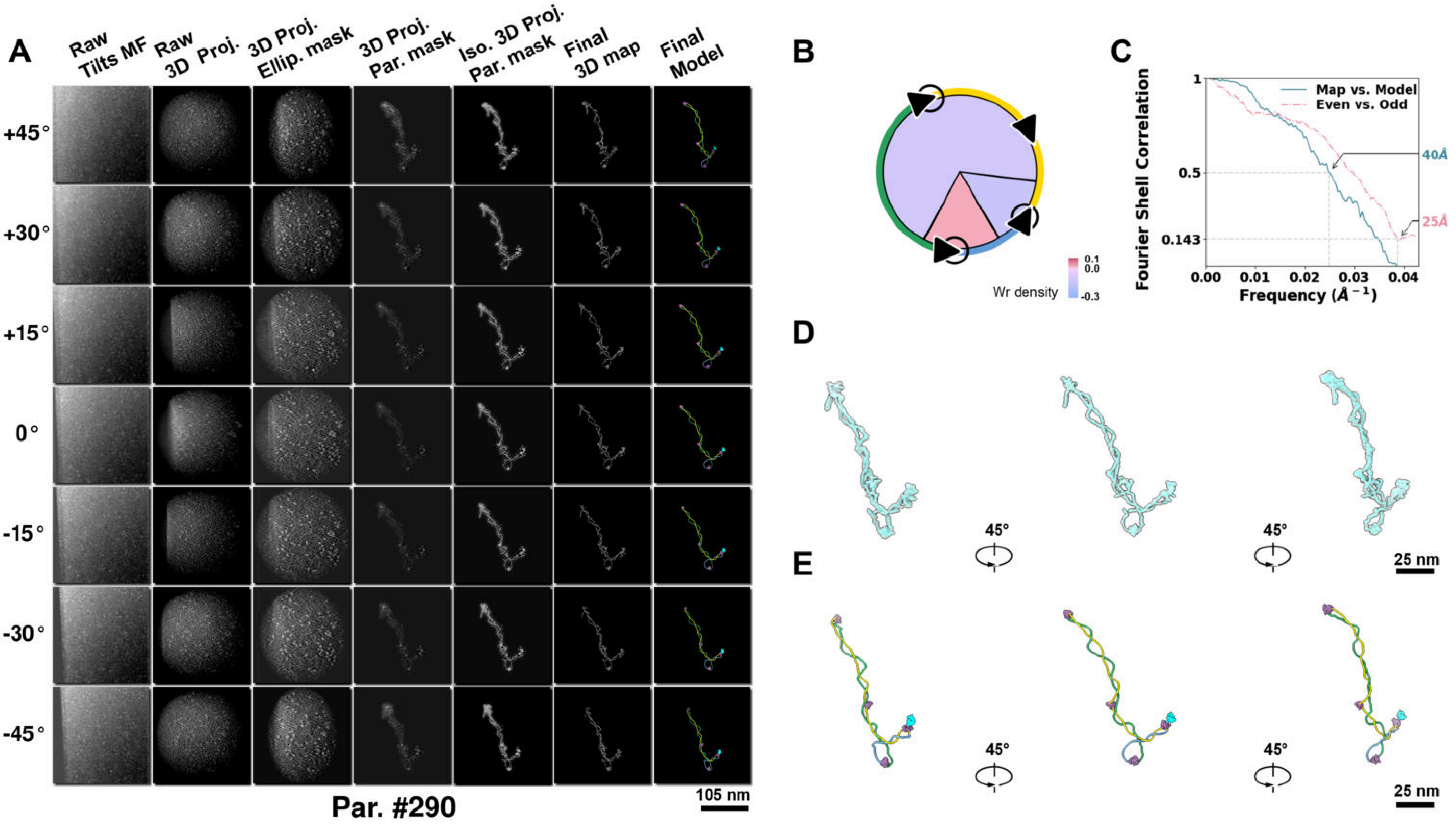

Supplementary Particle Figure 290. Cryo-ET 3D reconstruction of an individual Opp.-TEC particle.
(A) 3D reconstruction of the plasmid particle (index no. 290). The first column shows seven representative tilt images from +45° to -45° in step of 15°. The second, third, and fourth columns show 3D projections of the particle with spherical, ellipsoidal (thinner along the z-dimension), and particle-shaped masks, respectively. The fifth column displays the 3D projections of the enhanced and IsoNet missing-wedge-corrected particle. The sixth and seventh columns present the final 3D map and the flexibly fitted model, respectively. (B) Circular schematic representation of a plasmid particle. The outer rim is color-coded to match the corresponding 3D model. Arrowheads indicate the transcriptional direction of bound RNAPs, and circles denote apical sites. Inner circular sectors represent individual plectonemes, with colors indicating writhe density (blue to red scale, −0.3 to 0.1). (C) Resolution assessment of the final 3D map using Fourier shell correlation (FSC). Two criteria are shown: FSC between two half-maps reconstructed from even and odd frames (evaluated at 0.143) and FSC between the final 3D map and the fitted model (evaluated at 0.5). (D) Zoomed-in views of the final 3D density map from panel A, displayed at two contour levels. (E) Superimposition of the high-contour level map from panel D onto its fitted model.

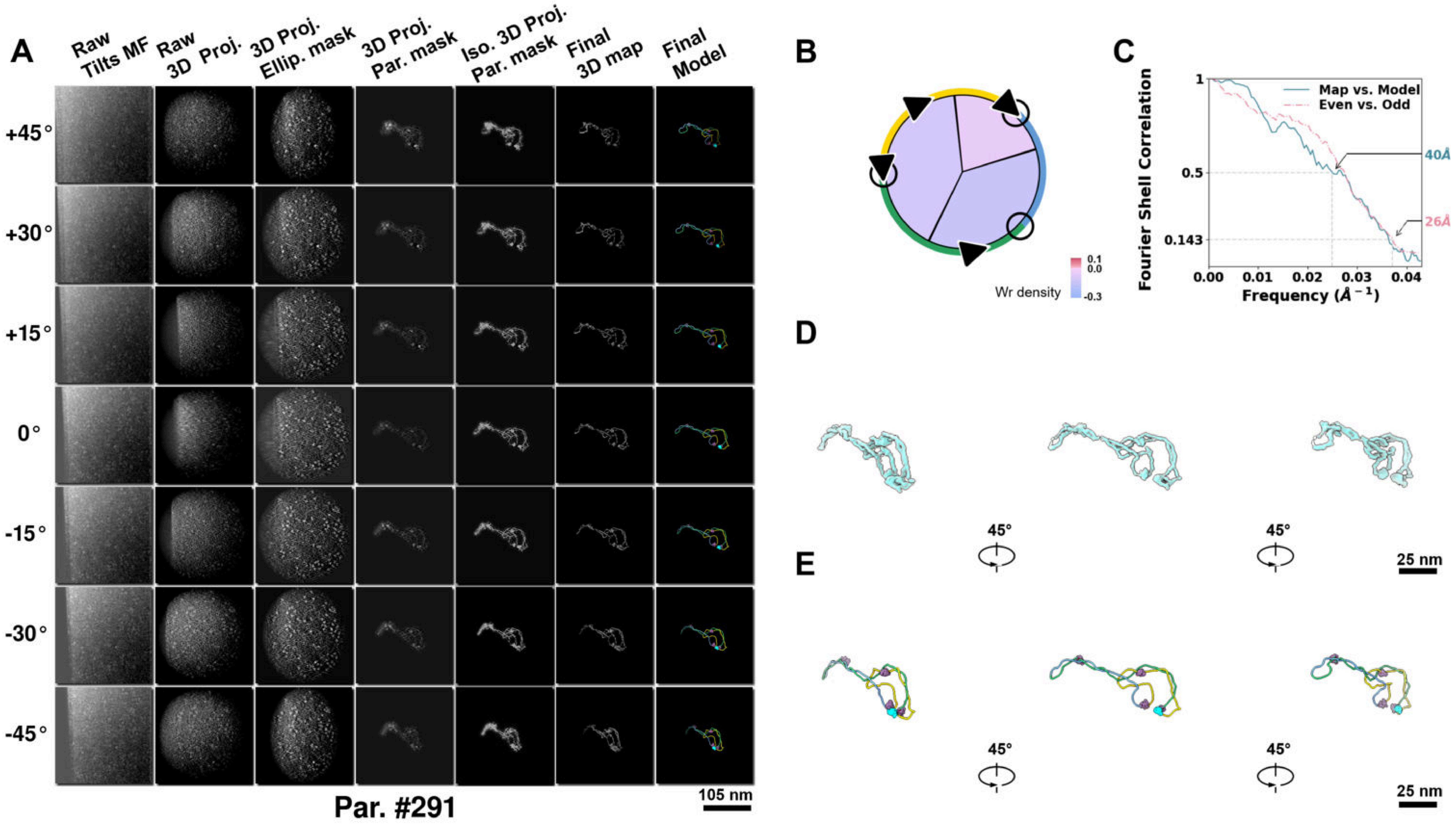

Supplementary Particle Figure 291. Cryo-ET 3D reconstruction of an individual Opp.-TEC particle.
(A) 3D reconstruction of the plasmid particle (index no. 291). The first column shows seven representative tilt images from +45° to -45° in step of 15°. The second, third, and fourth columns show 3D projections of the particle with spherical, ellipsoidal (thinner along the z-dimension), and particle-shaped masks, respectively. The fifth column displays the 3D projections of the enhanced and IsoNet missing-wedge-corrected particle. The sixth and seventh columns present the final 3D map and the flexibly fitted model, respectively. (B) Circular schematic representation of a plasmid particle. The outer rim is color-coded to match the corresponding 3D model. Arrowheads indicate the transcriptional direction of bound RNAPs, and circles denote apical sites. Inner circular sectors represent individual plectonemes, with colors indicating writhe density (blue to red scale, −0.3 to 0.1). (C) Resolution assessment of the final 3D map using Fourier shell correlation (FSC). Two criteria are shown: FSC between two half-maps reconstructed from even and odd frames (evaluated at 0.143) and FSC between the final 3D map and the fitted model (evaluated at 0.5). (D) Zoomed-in views of the final 3D density map from panel A, displayed at two contour levels. (E) Superimposition of the high-contour level map from panel D onto its fitted model.

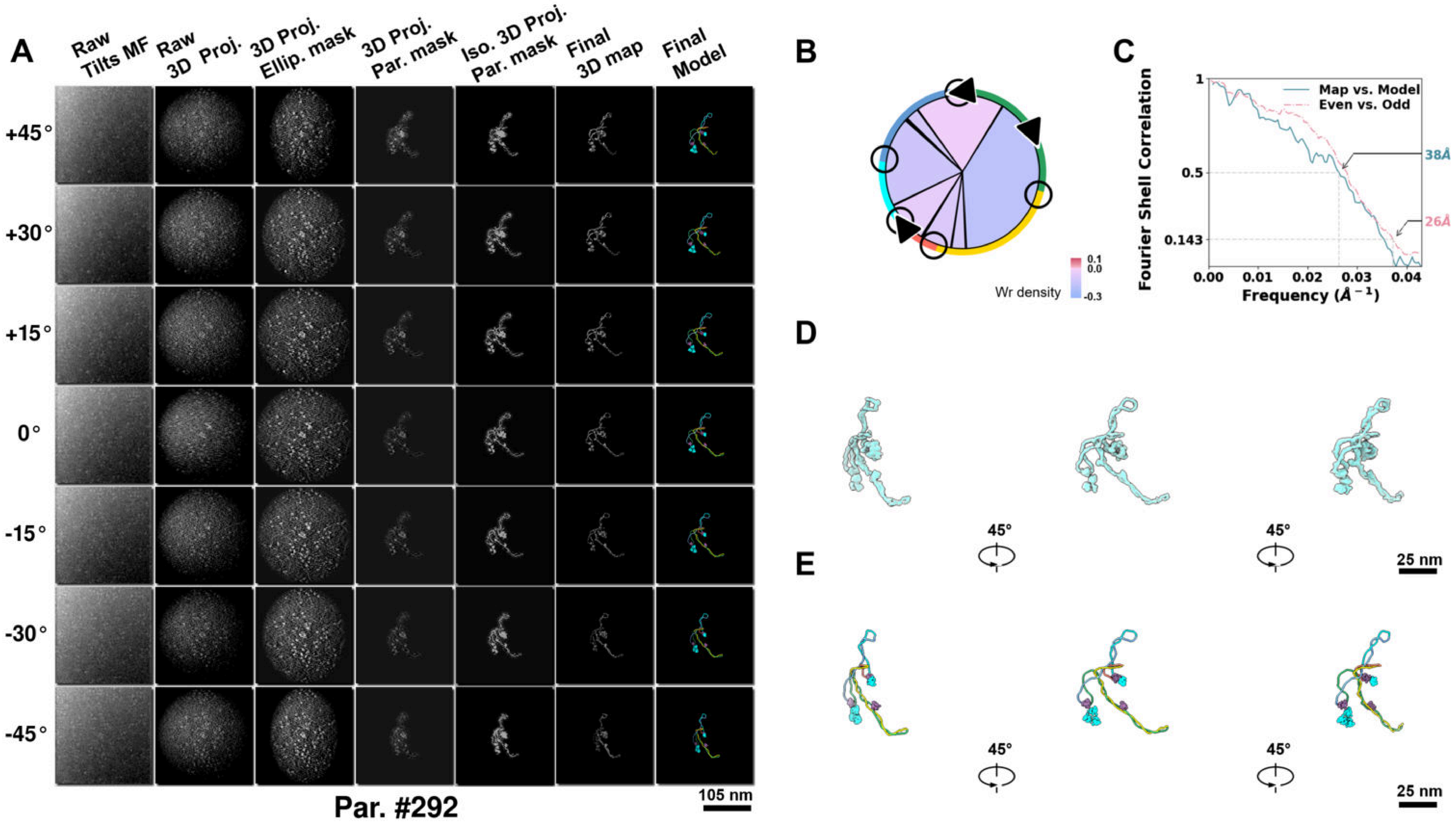

Supplementary Particle Figure 292. Cryo-ET 3D reconstruction of an individual Opp.-TEC particle.
(A) 3D reconstruction of the plasmid particle (index no. 292). The first column shows seven representative tilt images from +45° to -45° in step of 15°. The second, third, and fourth columns show 3D projections of the particle with spherical, ellipsoidal (thinner along the z-dimension), and particle-shaped masks, respectively. The fifth column displays the 3D projections of the enhanced and IsoNet missing-wedge-corrected particle. The sixth and seventh columns present the final 3D map and the flexibly fitted model, respectively. (B) Circular schematic representation of a plasmid particle. The outer rim is color-coded to match the corresponding 3D model. Arrowheads indicate the transcriptional direction of bound RNAPs, and circles denote apical sites. Inner circular sectors represent individual plectonemes, with colors indicating writhe density (blue to red scale, −0.3 to 0.1). (C) Resolution assessment of the final 3D map using Fourier shell correlation (FSC). Two criteria are shown: FSC between two half-maps reconstructed from even and odd frames (evaluated at 0.143) and FSC between the final 3D map and the fitted model (evaluated at 0.5). (D) Zoomed-in views of the final 3D density map from panel A, displayed at two contour levels. (E) Superimposition of the high-contour level map from panel D onto its fitted model.

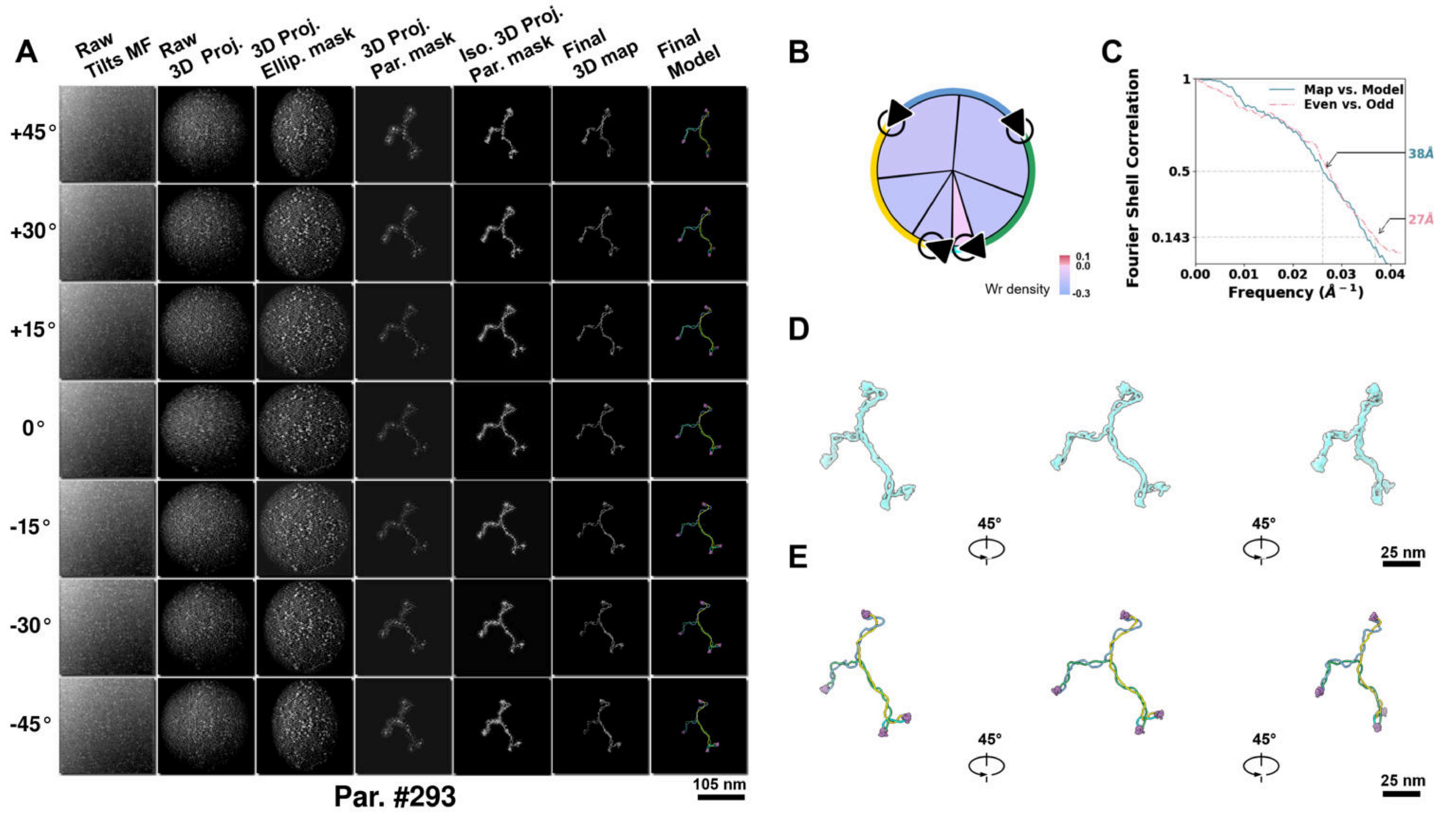

Supplementary Particle Figure 293. Cryo-ET 3D reconstruction of an individual Opp.-TEC particle.
(A) 3D reconstruction of the plasmid particle (index no. 293). The first column shows seven representative tilt images from +45° to -45° in step of 15°. The second, third, and fourth columns show 3D projections of the particle with spherical, ellipsoidal (thinner along the z-dimension), and particle-shaped masks, respectively. The fifth column displays the 3D projections of the enhanced and IsoNet missing-wedge-corrected particle. The sixth and seventh columns present the final 3D map and the flexibly fitted model, respectively. (B) Circular schematic representation of a plasmid particle. The outer rim is color-coded to match the corresponding 3D model. Arrowheads indicate the transcriptional direction of bound RNAPs, and circles denote apical sites. Inner circular sectors represent individual plectonemes, with colors indicating writhe density (blue to red scale, −0.3 to 0.1). (C) Resolution assessment of the final 3D map using Fourier shell correlation (FSC). Two criteria are shown: FSC between two half-maps reconstructed from even and odd frames (evaluated at 0.143) and FSC between the final 3D map and the fitted model (evaluated at 0.5). (D) Zoomed-in views of the final 3D density map from panel A, displayed at two contour levels. (E) Superimposition of the high-contour level map from panel D onto its fitted model.

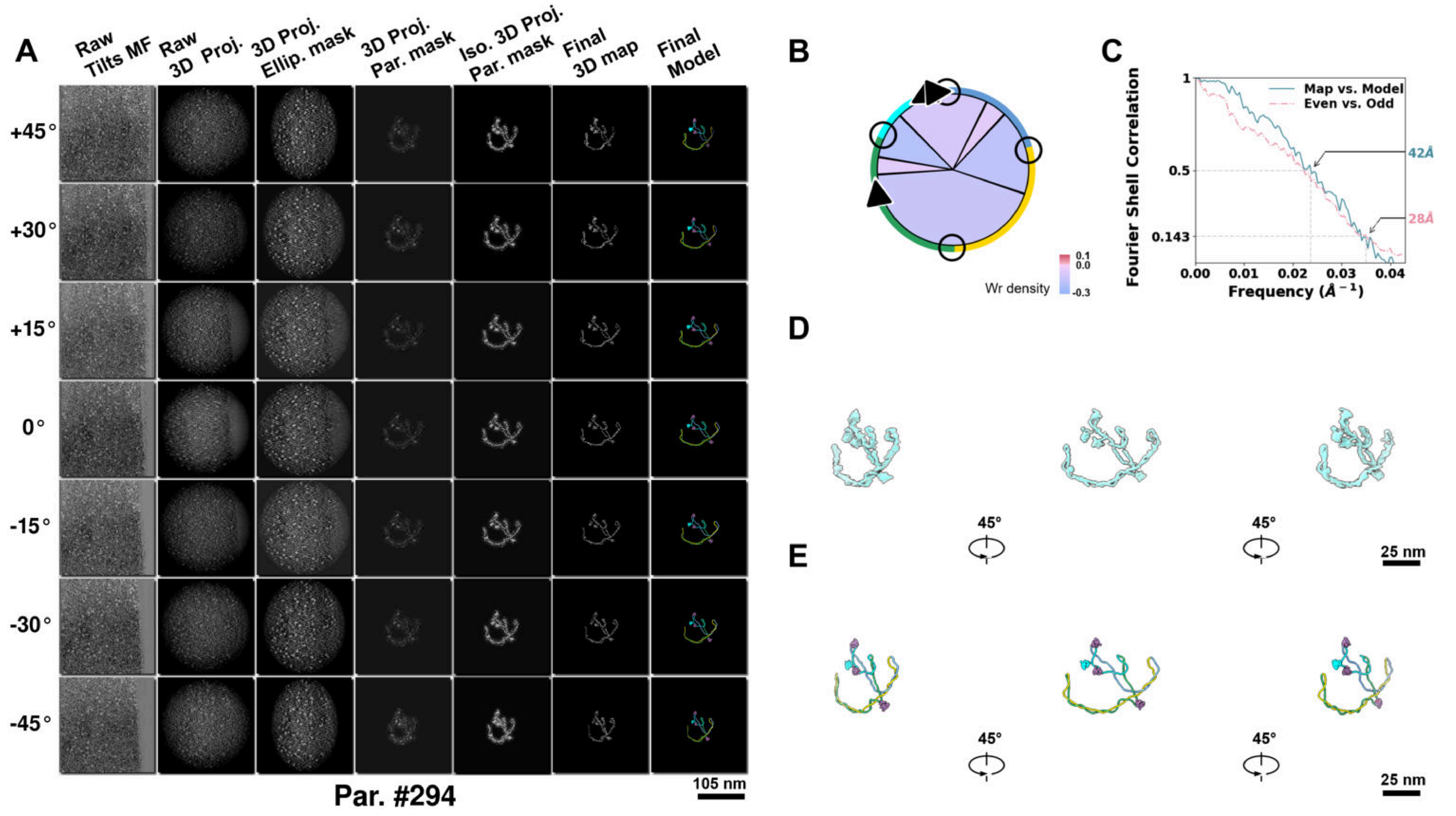

Supplementary Particle Figure 294. Cryo-ET 3D reconstruction of an individual Opp.-TEC particle.
(A) 3D reconstruction of the plasmid particle (index no. 294). The first column shows seven representative tilt images from +45° to -45° in step of 15°. The second, third, and fourth columns show 3D projections of the particle with spherical, ellipsoidal (thinner along the z-dimension), and particle-shaped masks, respectively. The fifth column displays the 3D projections of the enhanced and IsoNet missing-wedge-corrected particle. The sixth and seventh columns present the final 3D map and the flexibly fitted model, respectively. (B) Circular schematic representation of a plasmid particle. The outer rim is color-coded to match the corresponding 3D model. Arrowheads indicate the transcriptional direction of bound RNAPs, and circles denote apical sites. Inner circular sectors represent individual plectonemes, with colors indicating writhe density (blue to red scale, −0.3 to 0.1). (C) Resolution assessment of the final 3D map using Fourier shell correlation (FSC). Two criteria are shown: FSC between two half-maps reconstructed from even and odd frames (evaluated at 0.143) and FSC between the final 3D map and the fitted model (evaluated at 0.5). (D) Zoomed-in views of the final 3D density map from panel A, displayed at two contour levels. (E) Superimposition of the high-contour level map from panel D onto its fitted model.

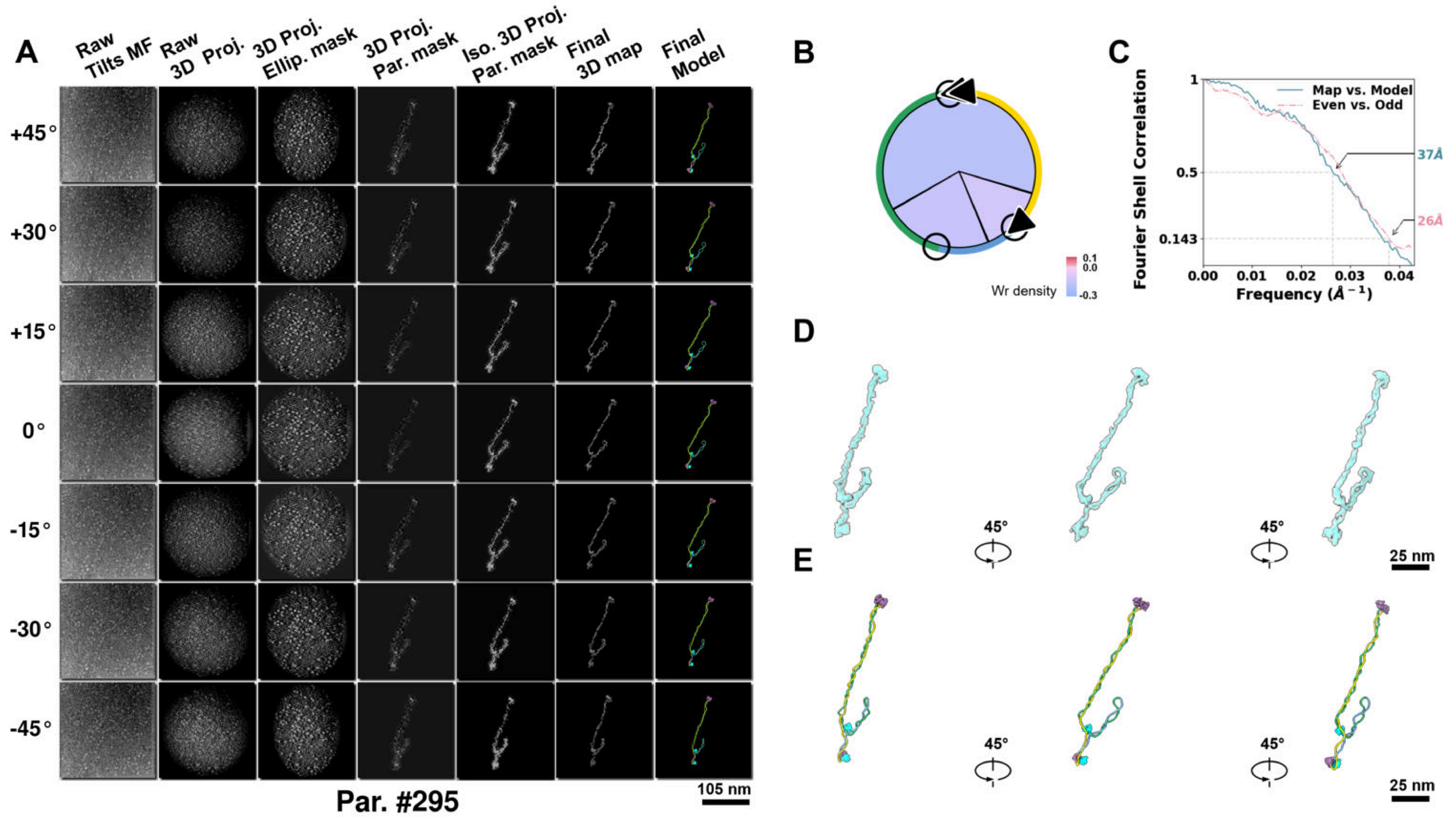

Supplementary Particle Figure 295. Cryo-ET 3D reconstruction of an individual Opp.-TEC particle.
(A) 3D reconstruction of the plasmid particle (index no. 295). The first column shows seven representative tilt images from +45° to -45° in step of 15°. The second, third, and fourth columns show 3D projections of the particle with spherical, ellipsoidal (thinner along the z-dimension), and particle-shaped masks, respectively. The fifth column displays the 3D projections of the enhanced and IsoNet missing-wedge-corrected particle. The sixth and seventh columns present the final 3D map and the flexibly fitted model, respectively. (B) Circular schematic representation of a plasmid particle. The outer rim is color-coded to match the corresponding 3D model. Arrowheads indicate the transcriptional direction of bound RNAPs, and circles denote apical sites. Inner circular sectors represent individual plectonemes, with colors indicating writhe density (blue to red scale, −0.3 to 0.1). (C) Resolution assessment of the final 3D map using Fourier shell correlation (FSC). Two criteria are shown: FSC between two half-maps reconstructed from even and odd frames (evaluated at 0.143) and FSC between the final 3D map and the fitted model (evaluated at 0.5). (D) Zoomed-in views of the final 3D density map from panel A, displayed at two contour levels. (E) Superimposition of the high-contour level map from panel D onto its fitted model.

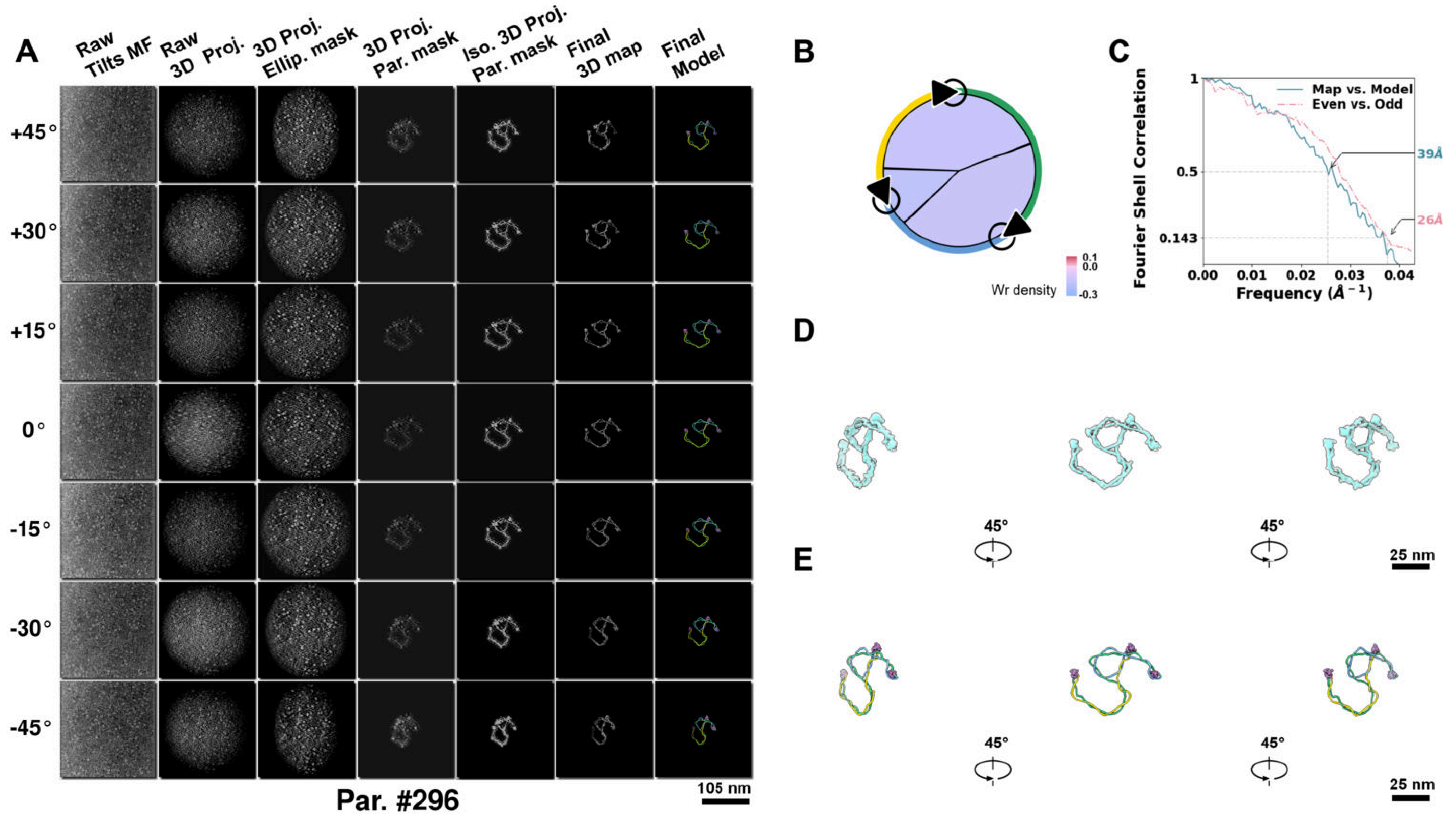

Supplementary Particle Figure 296. Cryo-ET 3D reconstruction of an individual Opp.-TEC particle.
(A) 3D reconstruction of the plasmid particle (index no. 296). The first column shows seven representative tilt images from +45° to -45° in step of 15°. The second, third, and fourth columns show 3D projections of the particle with spherical, ellipsoidal (thinner along the z-dimension), and particle-shaped masks, respectively. The fifth column displays the 3D projections of the enhanced and IsoNet missing-wedge-corrected particle. The sixth and seventh columns present the final 3D map and the flexibly fitted model, respectively. (B) Circular schematic representation of a plasmid particle. The outer rim is color-coded to match the corresponding 3D model. Arrowheads indicate the transcriptional direction of bound RNAPs, and circles denote apical sites. Inner circular sectors represent individual plectonemes, with colors indicating writhe density (blue to red scale, −0.3 to 0.1). (C) Resolution assessment of the final 3D map using Fourier shell correlation (FSC). Two criteria are shown: FSC between two half-maps reconstructed from even and odd frames (evaluated at 0.143) and FSC between the final 3D map and the fitted model (evaluated at 0.5). (D) Zoomed-in views of the final 3D density map from panel A, displayed at two contour levels. (E) Superimposition of the high-contour level map from panel D onto its fitted model.

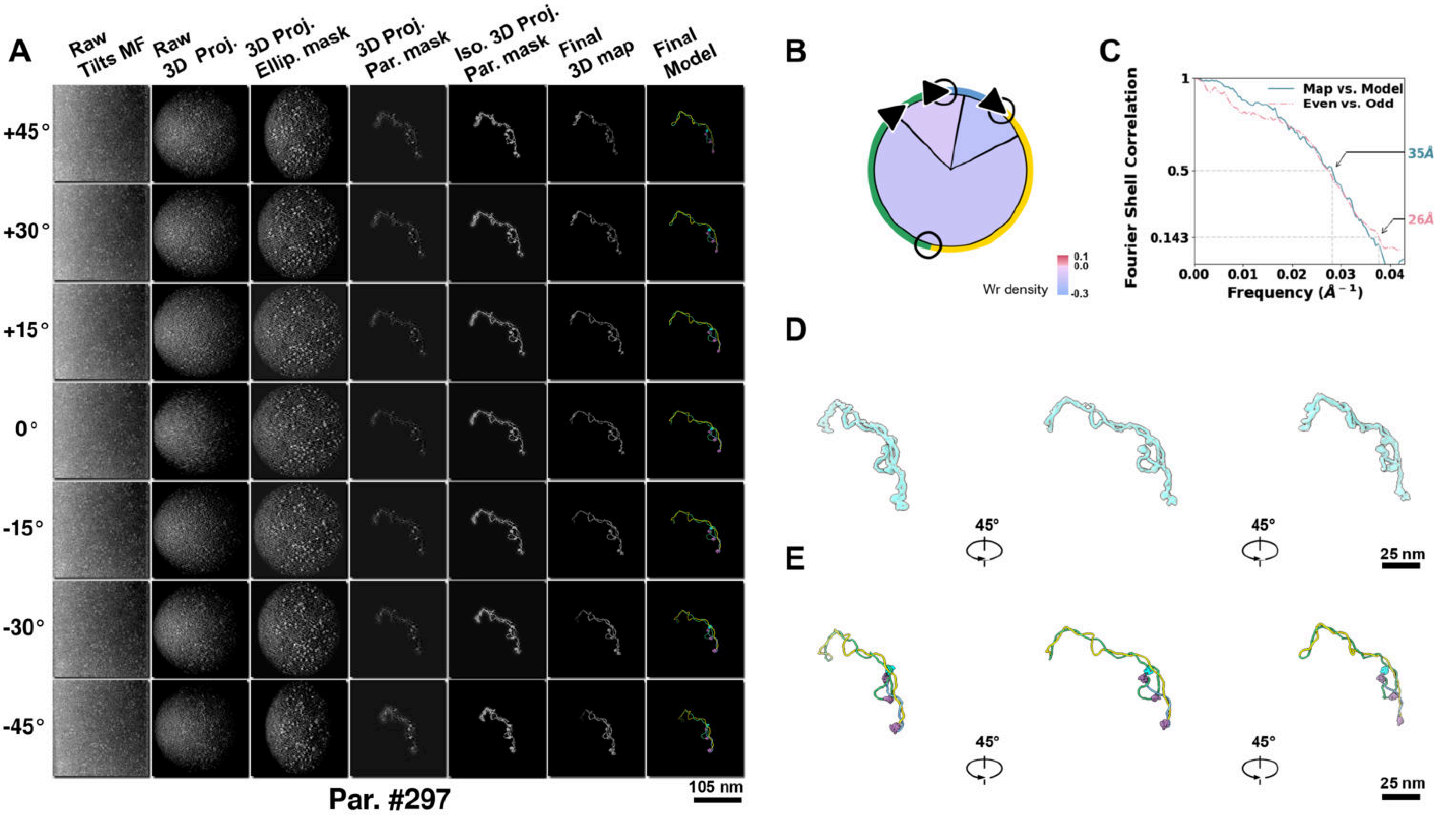

Supplementary Particle Figure 297. Cryo-ET 3D reconstruction of an individual Opp.-TEC particle.
(A) 3D reconstruction of the plasmid particle (index no. 297). The first column shows seven representative tilt images from +45° to -45° in step of 15°. The second, third, and fourth columns show 3D projections of the particle with spherical, ellipsoidal (thinner along the z-dimension), and particle-shaped masks, respectively. The fifth column displays the 3D projections of the enhanced and IsoNet missing-wedge-corrected particle. The sixth and seventh columns present the final 3D map and the flexibly fitted model, respectively. (B) Circular schematic representation of a plasmid particle. The outer rim is color-coded to match the corresponding 3D model. Arrowheads indicate the transcriptional direction of bound RNAPs, and circles denote apical sites. Inner circular sectors represent individual plectonemes, with colors indicating writhe density (blue to red scale, −0.3 to 0.1). (C) Resolution assessment of the final 3D map using Fourier shell correlation (FSC). Two criteria are shown: FSC between two half-maps reconstructed from even and odd frames (evaluated at 0.143) and FSC between the final 3D map and the fitted model (evaluated at 0.5). (D) Zoomed-in views of the final 3D density map from panel A, displayed at two contour levels. (E) Superimposition of the high-contour level map from panel D onto its fitted model.

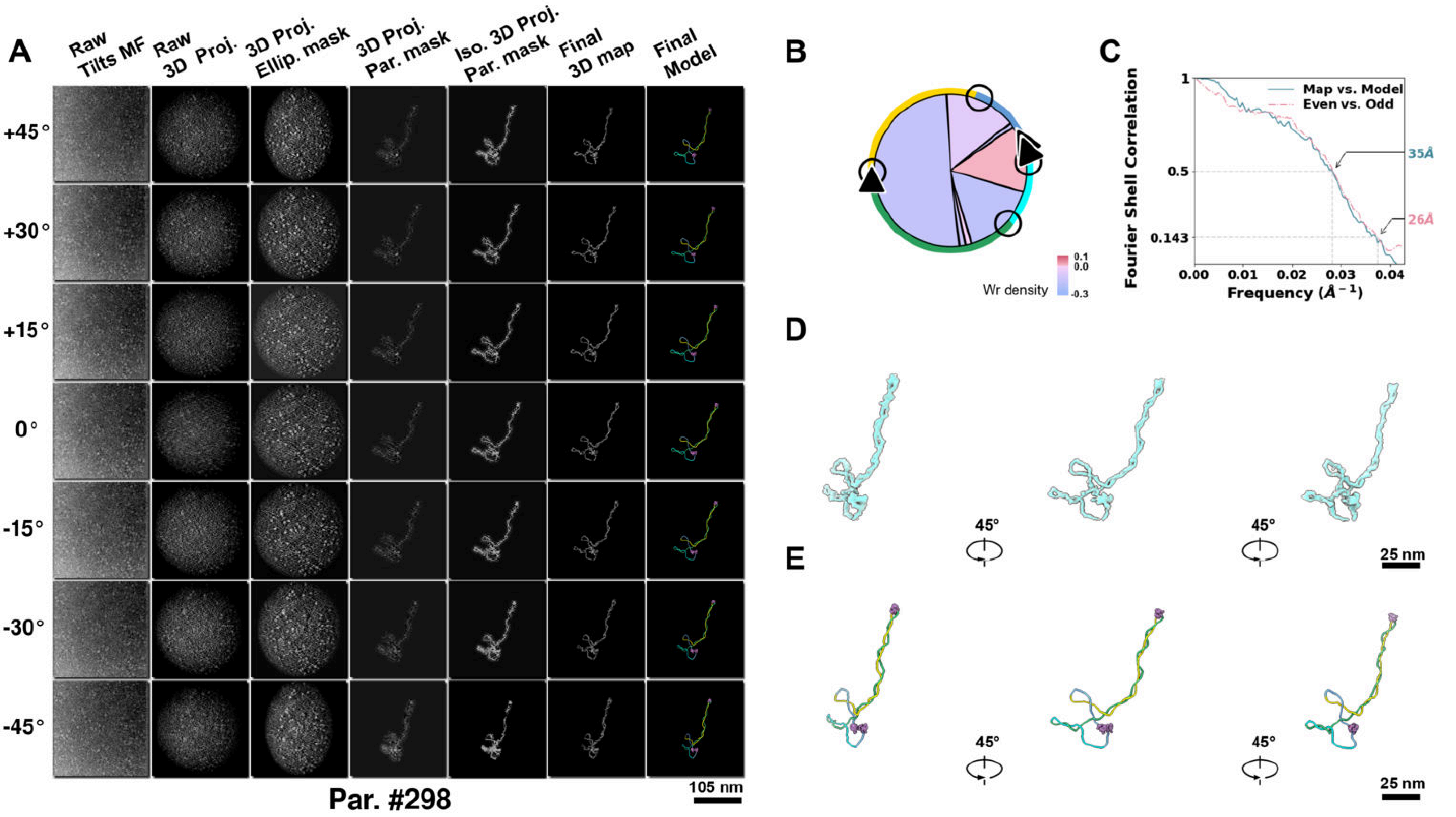

Supplementary Particle Figure 298. Cryo-ET 3D reconstruction of an individual Opp.-TEC particle.
(A) 3D reconstruction of the plasmid particle (index no. 298). The first column shows seven representative tilt images from +45° to -45° in step of 15°. The second, third, and fourth columns show 3D projections of the particle with spherical, ellipsoidal (thinner along the z-dimension), and particle-shaped masks, respectively. The fifth column displays the 3D projections of the enhanced and IsoNet missing-wedge-corrected particle. The sixth and seventh columns present the final 3D map and the flexibly fitted model, respectively. (B) Circular schematic representation of a plasmid particle. The outer rim is color-coded to match the corresponding 3D model. Arrowheads indicate the transcriptional direction of bound RNAPs, and circles denote apical sites. Inner circular sectors represent individual plectonemes, with colors indicating writhe density (blue to red scale, −0.3 to 0.1). (C) Resolution assessment of the final 3D map using Fourier shell correlation (FSC). Two criteria are shown: FSC between two half-maps reconstructed from even and odd frames (evaluated at 0.143) and FSC between the final 3D map and the fitted model (evaluated at 0.5). (D) Zoomed-in views of the final 3D density map from panel A, displayed at two contour levels. (E) Superimposition of the high-contour level map from panel D onto its fitted model.

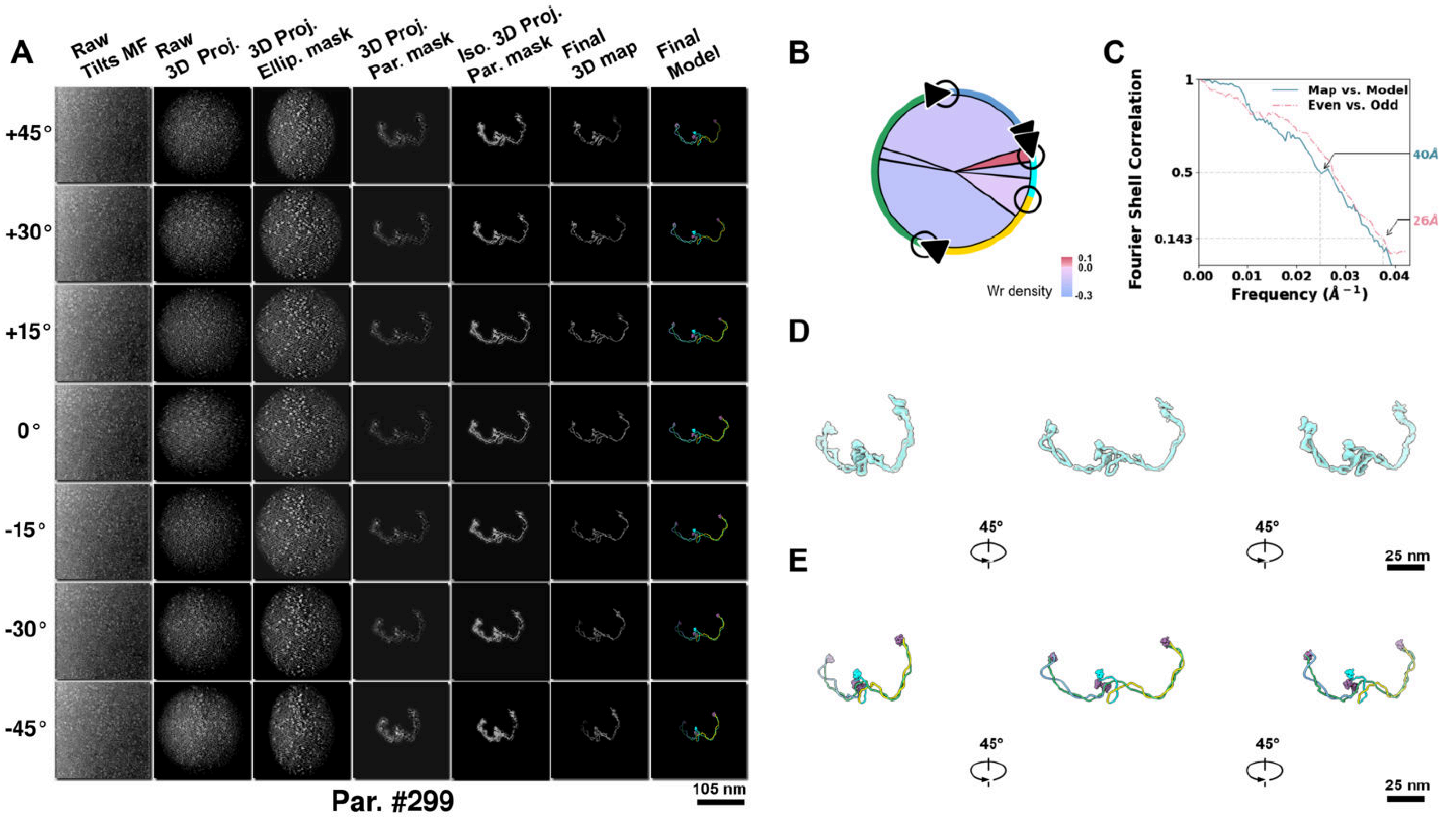

Supplementary Particle Figure 299. Cryo-ET 3D reconstruction of an individual Opp.-TEC particle.
(A) 3D reconstruction of the plasmid particle (index no. 299). The first column shows seven representative tilt images from +45° to -45° in step of 15°. The second, third, and fourth columns show 3D projections of the particle with spherical, ellipsoidal (thinner along the z-dimension), and particle-shaped masks, respectively. The fifth column displays the 3D projections of the enhanced and IsoNet missing-wedge-corrected particle. The sixth and seventh columns present the final 3D map and the flexibly fitted model, respectively. (B) Circular schematic representation of a plasmid particle. The outer rim is color-coded to match the corresponding 3D model. Arrowheads indicate the transcriptional direction of bound RNAPs, and circles denote apical sites. Inner circular sectors represent individual plectonemes, with colors indicating writhe density (blue to red scale, −0.3 to 0.1). (C) Resolution assessment of the final 3D map using Fourier shell correlation (FSC). Two criteria are shown: FSC between two half-maps reconstructed from even and odd frames (evaluated at 0.143) and FSC between the final 3D map and the fitted model (evaluated at 0.5). (D) Zoomed-in views of the final 3D density map from panel A, displayed at two contour levels. (E) Superimposition of the high-contour level map from panel D onto its fitted model.

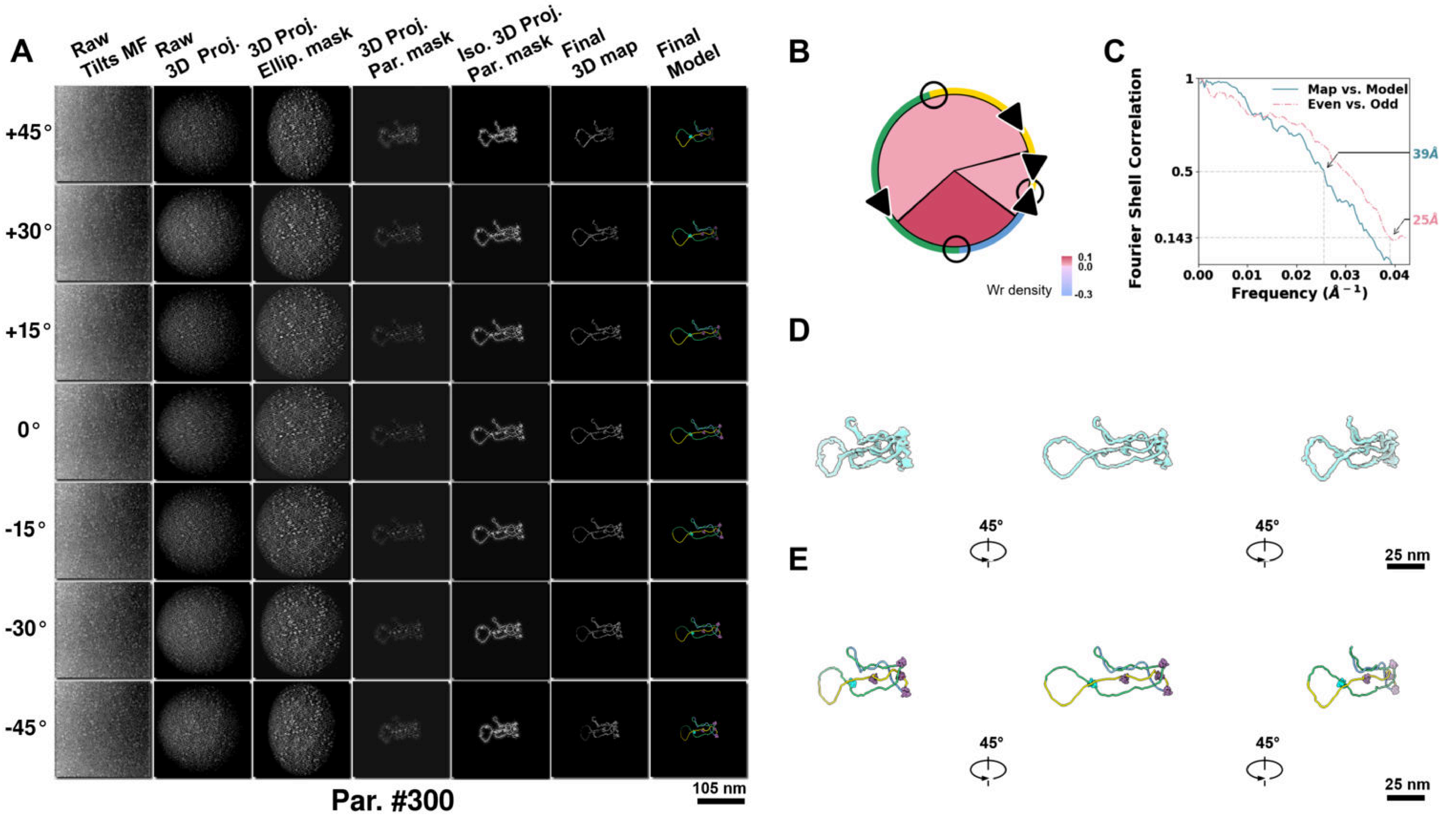

Supplementary Particle Figure 300. Cryo-ET 3D reconstruction of an individual Opp.-TEC particle.
(A) 3D reconstruction of the plasmid particle (index no. 300). The first column shows seven representative tilt images from +45° to -45° in step of 15°. The second, third, and fourth columns show 3D projections of the particle with spherical, ellipsoidal (thinner along the z-dimension), and particle-shaped masks, respectively. The fifth column displays the 3D projections of the enhanced and IsoNet missing-wedge-corrected particle. The sixth and seventh columns present the final 3D map and the flexibly fitted model, respectively. (B) Circular schematic representation of a plasmid particle. The outer rim is color-coded to match the corresponding 3D model. Arrowheads indicate the transcriptional direction of bound RNAPs, and circles denote apical sites. Inner circular sectors represent individual plectonemes, with colors indicating writhe density (blue to red scale, −0.3 to 0.1). (C) Resolution assessment of the final 3D map using Fourier shell correlation (FSC). Two criteria are shown: FSC between two half-maps reconstructed from even and odd frames (evaluated at 0.143) and FSC between the final 3D map and the fitted model (evaluated at 0.5). (D) Zoomed-in views of the final 3D density map from panel A, displayed at two contour levels. (E) Superimposition of the high-contour level map from panel D onto its fitted model.

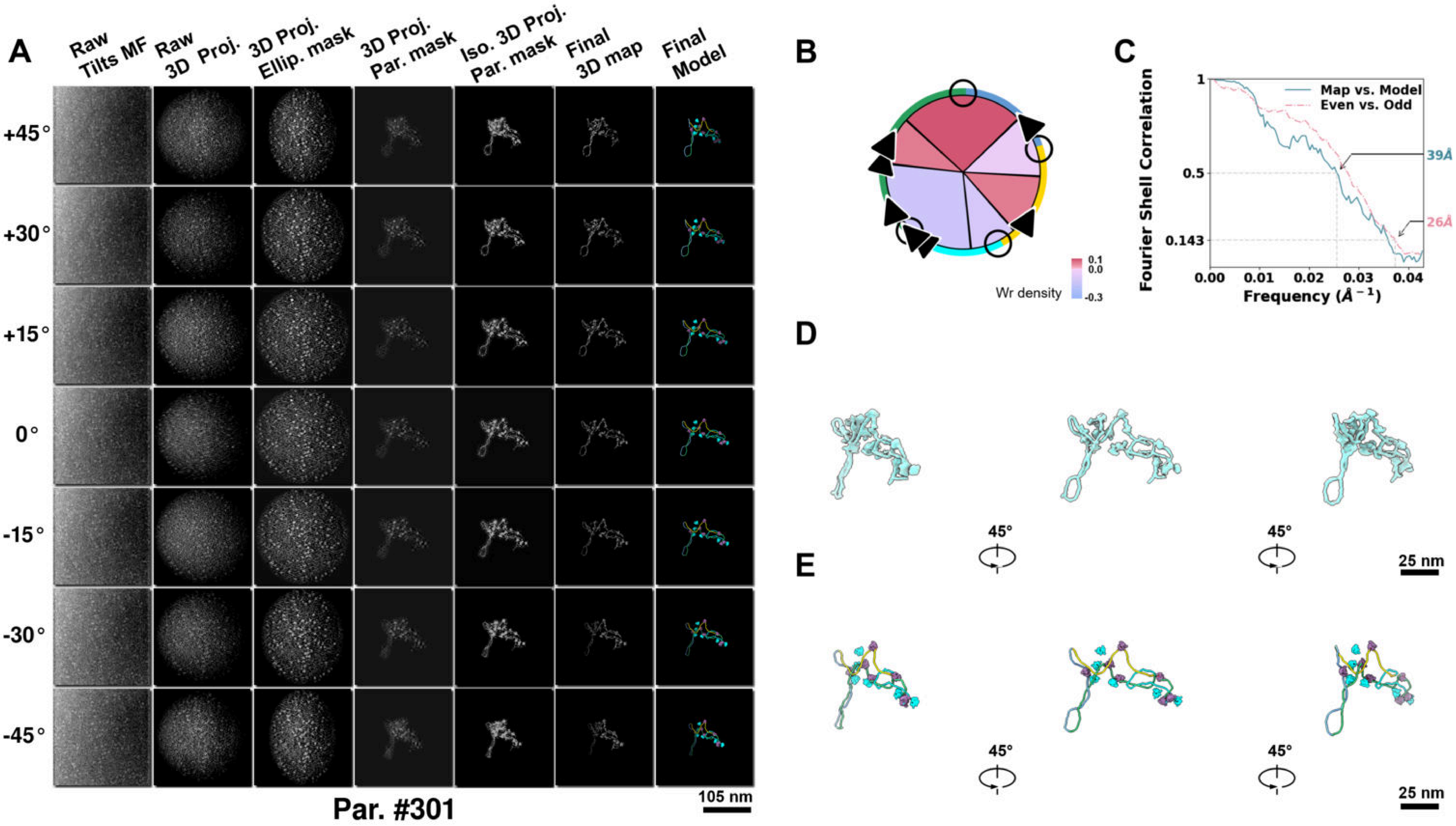

Supplementary Particle Figure 301. Cryo-ET 3D reconstruction of an individual Opp.-TEC particle.
(A) 3D reconstruction of the plasmid particle (index no. 301). The first column shows seven representative tilt images from +45° to -45° in step of 15°. The second, third, and fourth columns show 3D projections of the particle with spherical, ellipsoidal (thinner along the z-dimension), and particle-shaped masks, respectively. The fifth column displays the 3D projections of the enhanced and IsoNet missing-wedge-corrected particle. The sixth and seventh columns present the final 3D map and the flexibly fitted model, respectively. (B) Circular schematic representation of a plasmid particle. The outer rim is color-coded to match the corresponding 3D model. Arrowheads indicate the transcriptional direction of bound RNAPs, and circles denote apical sites. Inner circular sectors represent individual plectonemes, with colors indicating writhe density (blue to red scale, −0.3 to 0.1). (C) Resolution assessment of the final 3D map using Fourier shell correlation (FSC). Two criteria are shown: FSC between two half-maps reconstructed from even and odd frames (evaluated at 0.143) and FSC between the final 3D map and the fitted model (evaluated at 0.5). (D) Zoomed-in views of the final 3D density map from panel A, displayed at two contour levels. (E) Superimposition of the high-contour level map from panel D onto its fitted model.

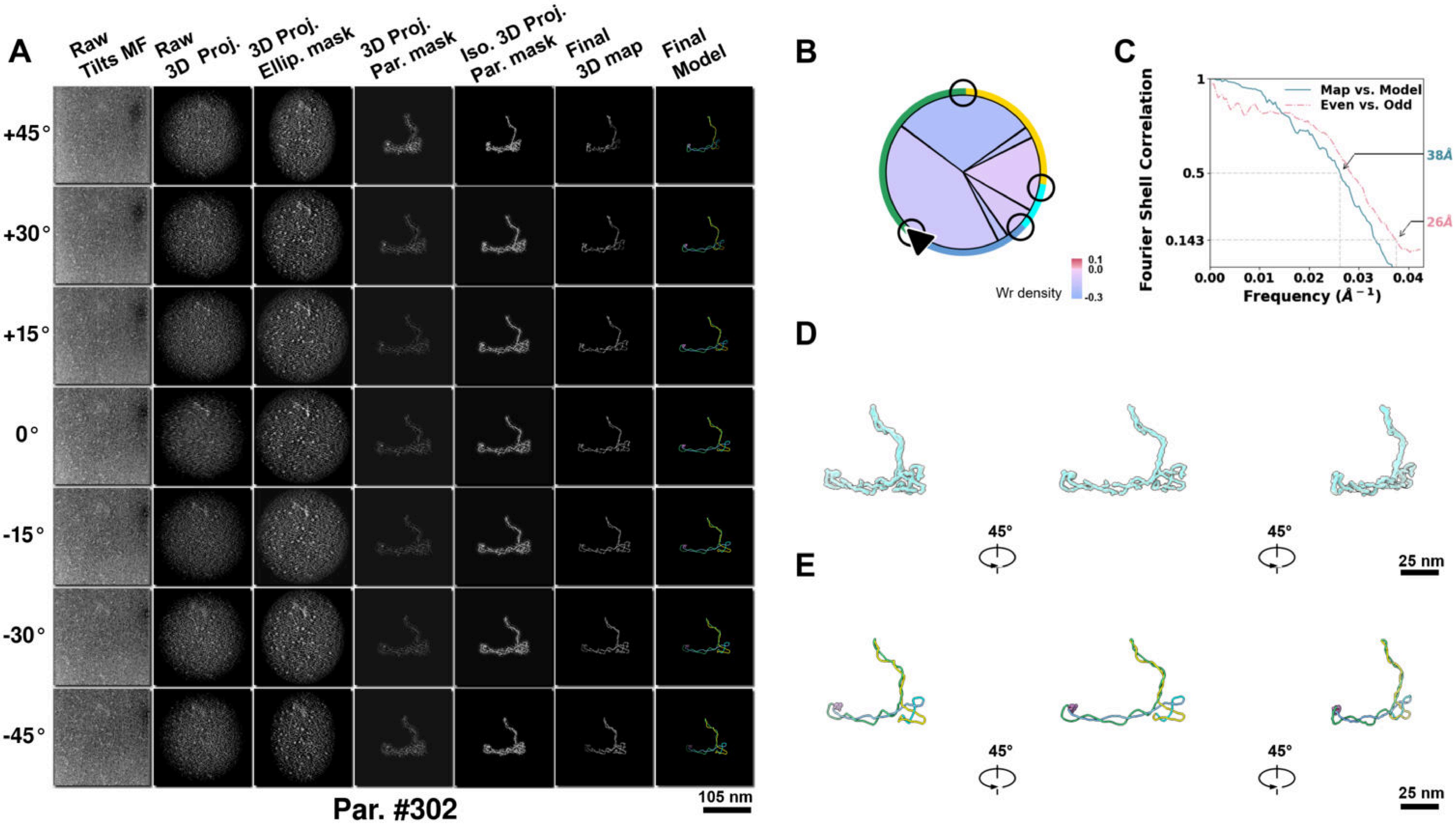

Supplementary Particle Figure 302. Cryo-ET 3D reconstruction of an individual Tan.-TEC particle.
(A) 3D reconstruction of the plasmid particle (index no. 302). The first column shows seven representative tilt images from +45° to -45° in step of 15°. The second, third, and fourth columns show 3D projections of the particle with spherical, ellipsoidal (thinner along the z-dimension), and particle-shaped masks, respectively. The fifth column displays the 3D projections of the enhanced and IsoNet missing-wedge-corrected particle. The sixth and seventh columns present the final 3D map and the flexibly fitted model, respectively. (B) Circular schematic representation of a plasmid particle. The outer rim is color-coded to match the corresponding 3D model. Arrowheads indicate the transcriptional direction of bound RNAPs, and circles denote apical sites. Inner circular sectors represent individual plectonemes, with colors indicating writhe density (blue to red scale, −0.3 to 0.1). (C) Resolution assessment of the final 3D map using Fourier shell correlation (FSC). Two criteria are shown: FSC between two half-maps reconstructed from even and odd frames (evaluated at 0.143) and FSC between the final 3D map and the fitted model (evaluated at 0.5). (D) Zoomed-in views of the final 3D density map from panel A, displayed at two contour levels. (E) Superimposition of the high-contour level map from panel D onto its fitted model.

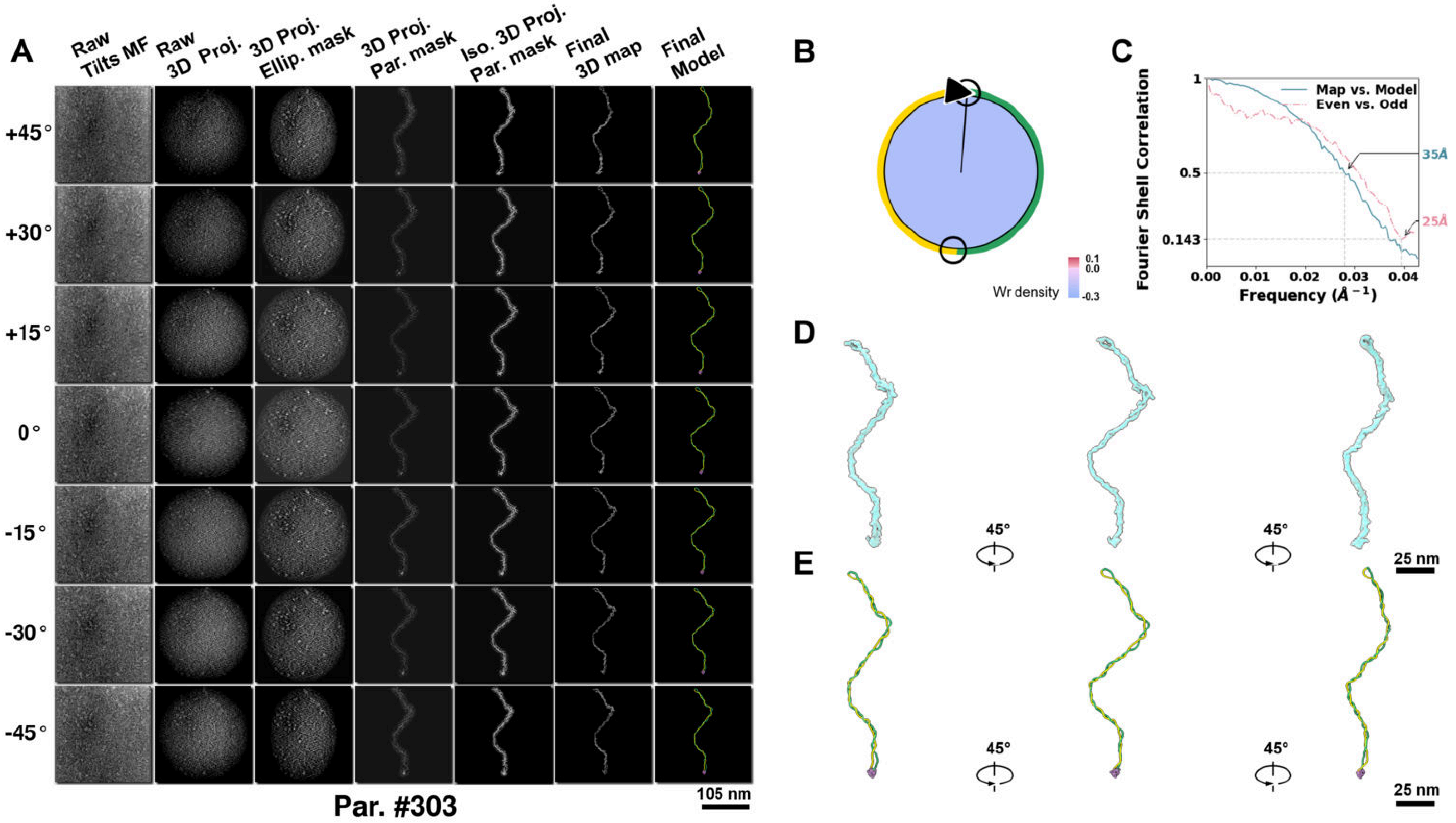

Supplementary Particle Figure 303. Cryo-ET 3D reconstruction of an individual Tan.-TEC particle.
(A) 3D reconstruction of the plasmid particle (index no. 303). The first column shows seven representative tilt images from +45° to -45° in step of 15°. The second, third, and fourth columns show 3D projections of the particle with spherical, ellipsoidal (thinner along the z-dimension), and particle-shaped masks, respectively. The fifth column displays the 3D projections of the enhanced and IsoNet missing-wedge-corrected particle. The sixth and seventh columns present the final 3D map and the flexibly fitted model, respectively. (B) Circular schematic representation of a plasmid particle. The outer rim is color-coded to match the corresponding 3D model. Arrowheads indicate the transcriptional direction of bound RNAPs, and circles denote apical sites. Inner circular sectors represent individual plectonemes, with colors indicating writhe density (blue to red scale, −0.3 to 0.1). (C) Resolution assessment of the final 3D map using Fourier shell correlation (FSC). Two criteria are shown: FSC between two half-maps reconstructed from even and odd frames (evaluated at 0.143) and FSC between the final 3D map and the fitted model (evaluated at 0.5). (D) Zoomed-in views of the final 3D density map from panel A, displayed at two contour levels. (E) Superimposition of the high-contour level map from panel D onto its fitted model.

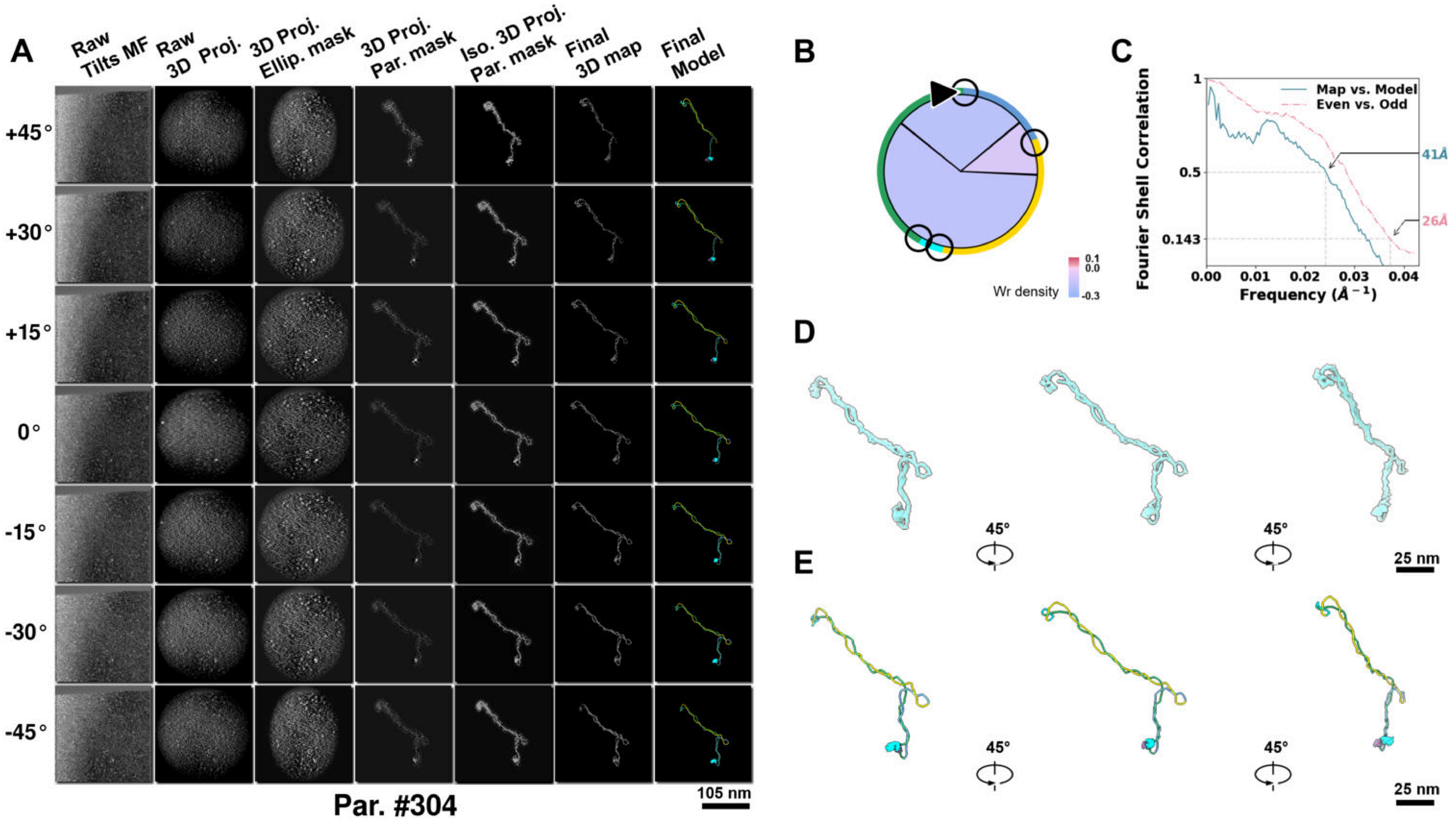

Supplementary Particle Figure 304. Cryo-ET 3D reconstruction of an individual Tan.-TEC particle.
(A) 3D reconstruction of the plasmid particle (index no. 304). The first column shows seven representative tilt images from +45° to -45° in step of 15°. The second, third, and fourth columns show 3D projections of the particle with spherical, ellipsoidal (thinner along the z-dimension), and particle-shaped masks, respectively. The fifth column displays the 3D projections of the enhanced and IsoNet missing-wedge-corrected particle. The sixth and seventh columns present the final 3D map and the flexibly fitted model, respectively. (B) Circular schematic representation of a plasmid particle. The outer rim is color-coded to match the corresponding 3D model. Arrowheads indicate the transcriptional direction of bound RNAPs, and circles denote apical sites. Inner circular sectors represent individual plectonemes, with colors indicating writhe density (blue to red scale, −0.3 to 0.1). (C) Resolution assessment of the final 3D map using Fourier shell correlation (FSC). Two criteria are shown: FSC between two half-maps reconstructed from even and odd frames (evaluated at 0.143) and FSC between the final 3D map and the fitted model (evaluated at 0.5). (D) Zoomed-in views of the final 3D density map from panel A, displayed at two contour levels. (E) Superimposition of the high-contour level map from panel D onto its fitted model.

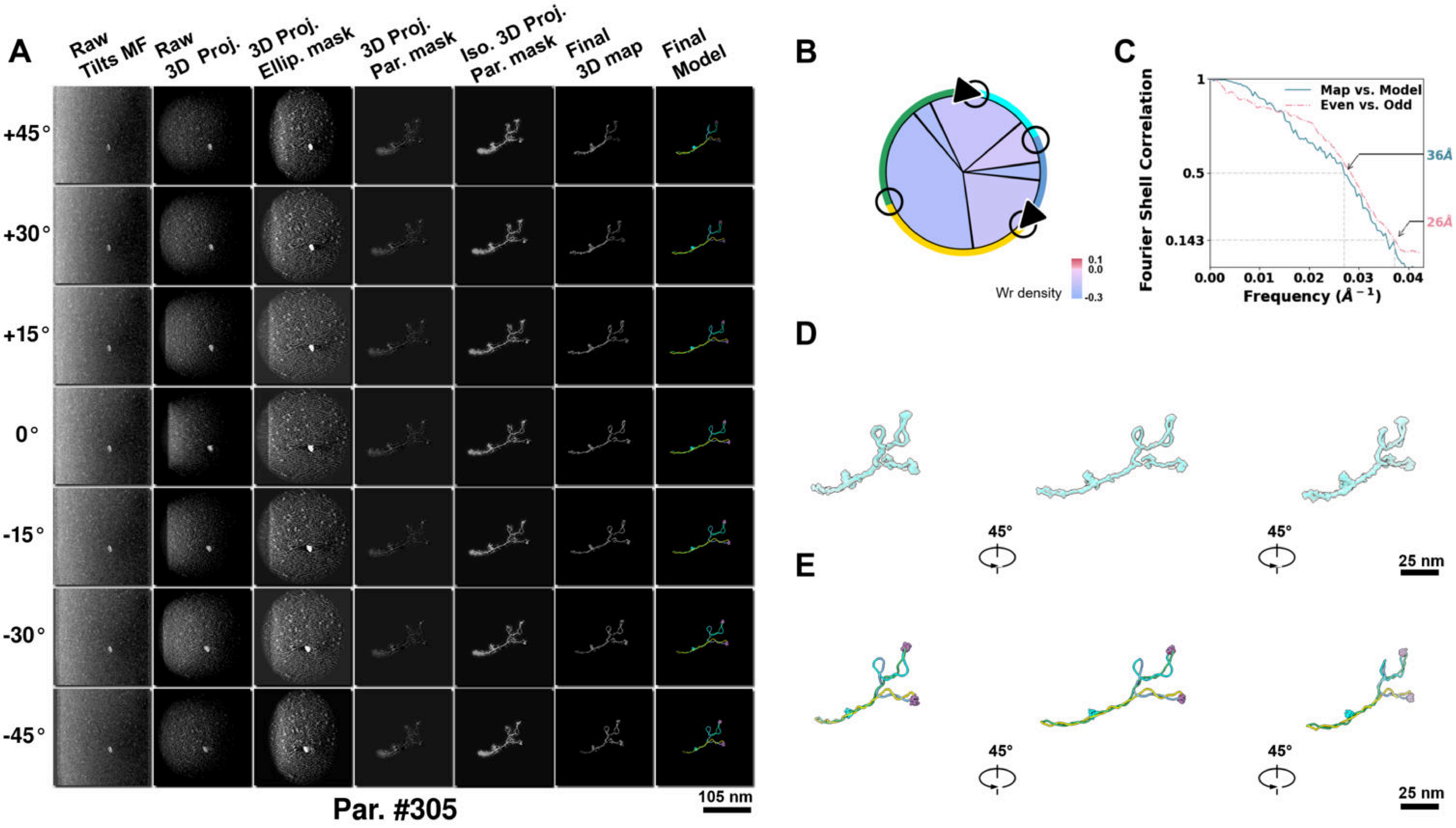

Supplementary Particle Figure 305. Cryo-ET 3D reconstruction of an individual Tan.-TEC particle.
(A) 3D reconstruction of the plasmid particle (index no. 305). The first column shows seven representative tilt images from +45° to -45° in step of 15°. The second, third, and fourth columns show 3D projections of the particle with spherical, ellipsoidal (thinner along the z-dimension), and particle-shaped masks, respectively. The fifth column displays the 3D projections of the enhanced and IsoNet missing-wedge-corrected particle. The sixth and seventh columns present the final 3D map and the flexibly fitted model, respectively. (B) Circular schematic representation of a plasmid particle. The outer rim is color-coded to match the corresponding 3D model. Arrowheads indicate the transcriptional direction of bound RNAPs, and circles denote apical sites. Inner circular sectors represent individual plectonemes, with colors indicating writhe density (blue to red scale, −0.3 to 0.1). (C) Resolution assessment of the final 3D map using Fourier shell correlation (FSC). Two criteria are shown: FSC between two half-maps reconstructed from even and odd frames (evaluated at 0.143) and FSC between the final 3D map and the fitted model (evaluated at 0.5). (D) Zoomed-in views of the final 3D density map from panel A, displayed at two contour levels. (E) Superimposition of the high-contour level map from panel D onto its fitted model.

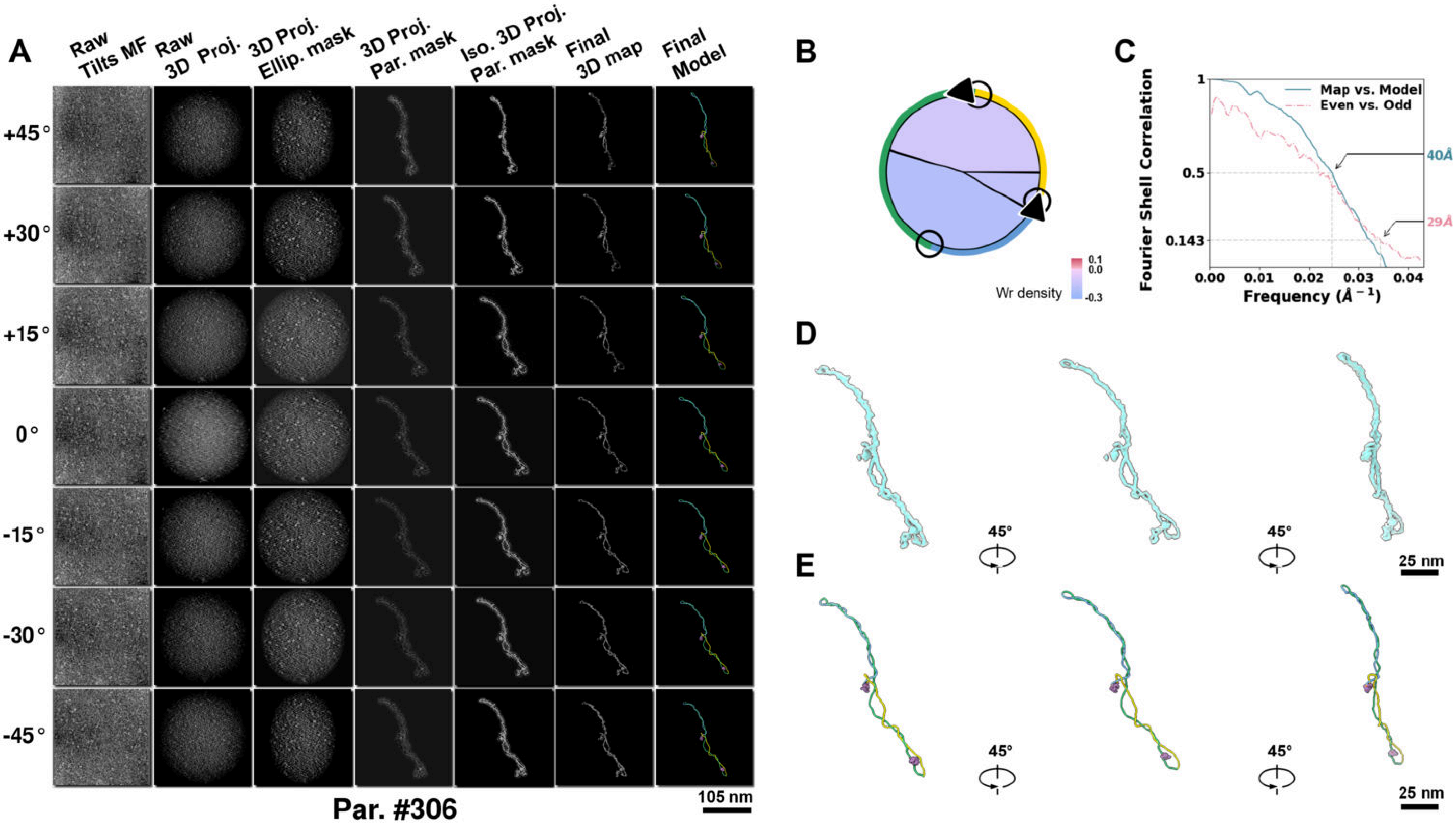

Supplementary Particle Figure 306. Cryo-ET 3D reconstruction of an individual Tan.-TEC particle.
(A) 3D reconstruction of the plasmid particle (index no. 306). The first column shows seven representative tilt images from +45° to -45° in step of 15°. The second, third, and fourth columns show 3D projections of the particle with spherical, ellipsoidal (thinner along the z-dimension), and particle-shaped masks, respectively. The fifth column displays the 3D projections of the enhanced and IsoNet missing-wedge-corrected particle. The sixth and seventh columns present the final 3D map and the flexibly fitted model, respectively. (B) Circular schematic representation of a plasmid particle. The outer rim is color-coded to match the corresponding 3D model. Arrowheads indicate the transcriptional direction of bound RNAPs, and circles denote apical sites. Inner circular sectors represent individual plectonemes, with colors indicating writhe density (blue to red scale, −0.3 to 0.1). (C) Resolution assessment of the final 3D map using Fourier shell correlation (FSC). Two criteria are shown: FSC between two half-maps reconstructed from even and odd frames (evaluated at 0.143) and FSC between the final 3D map and the fitted model (evaluated at 0.5). (D) Zoomed-in views of the final 3D density map from panel A, displayed at two contour levels. (E) Superimposition of the high-contour level map from panel D onto its fitted model.

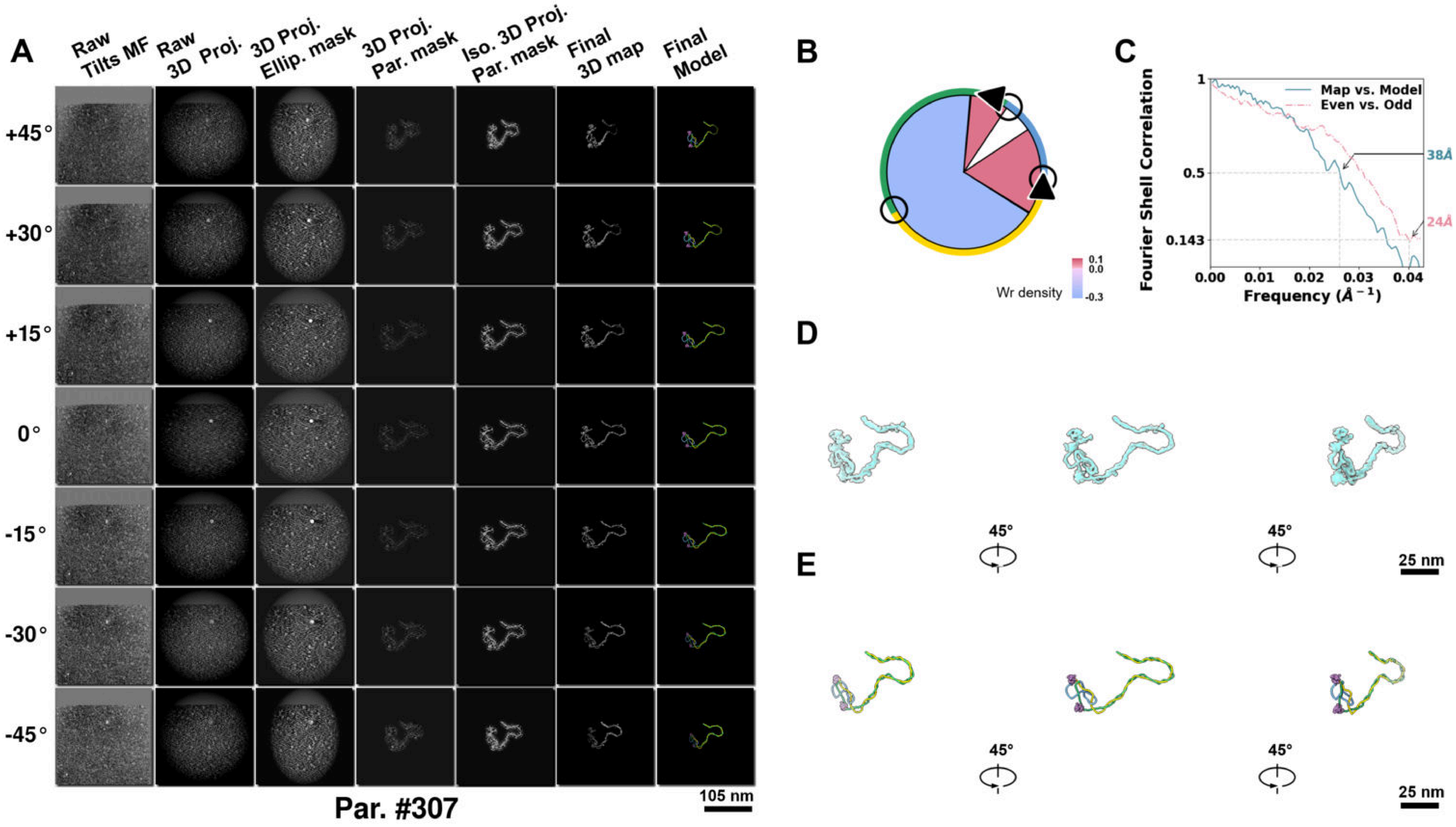

Supplementary Particle Figure 307. Cryo-ET 3D reconstruction of an individual Tan.-TEC particle.
(A) 3D reconstruction of the plasmid particle (index no. 307). The first column shows seven representative tilt images from +45° to -45° in step of 15°. The second, third, and fourth columns show 3D projections of the particle with spherical, ellipsoidal (thinner along the z-dimension), and particle-shaped masks, respectively. The fifth column displays the 3D projections of the enhanced and IsoNet missing-wedge-corrected particle. The sixth and seventh columns present the final 3D map and the flexibly fitted model, respectively. (B) Circular schematic representation of a plasmid particle. The outer rim is color-coded to match the corresponding 3D model. Arrowheads indicate the transcriptional direction of bound RNAPs, and circles denote apical sites. Inner circular sectors represent individual plectonemes, with colors indicating writhe density (blue to red scale, −0.3 to 0.1). (C) Resolution assessment of the final 3D map using Fourier shell correlation (FSC). Two criteria are shown: FSC between two half-maps reconstructed from even and odd frames (evaluated at 0.143) and FSC between the final 3D map and the fitted model (evaluated at 0.5). (D) Zoomed-in views of the final 3D density map from panel A, displayed at two contour levels. (E) Superimposition of the high-contour level map from panel D onto its fitted model.

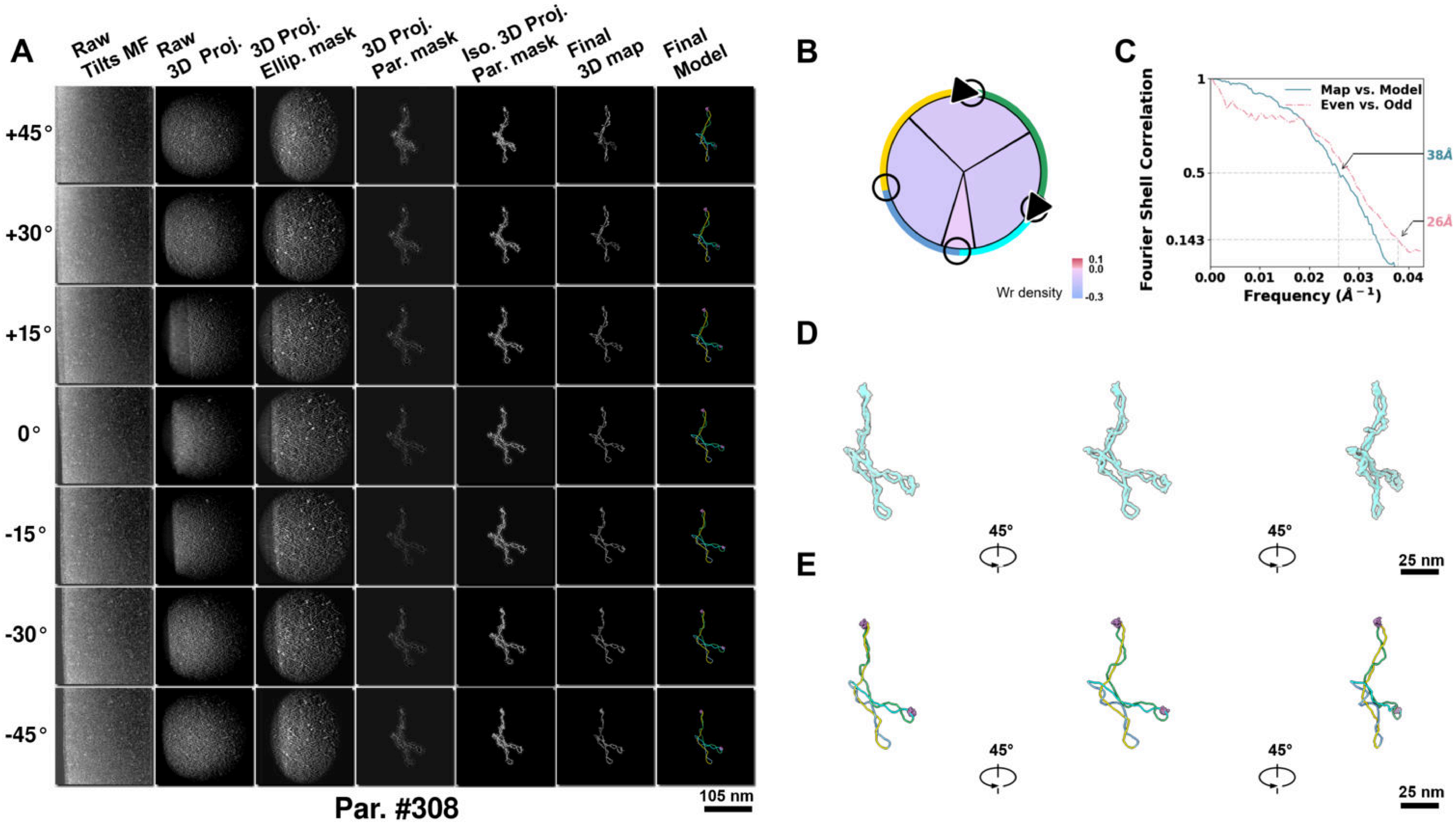

Supplementary Particle Figure 308. Cryo-ET 3D reconstruction of an individual Tan.-TEC particle.
(A) 3D reconstruction of the plasmid particle (index no. 308). The first column shows seven representative tilt images from +45° to -45° in step of 15°. The second, third, and fourth columns show 3D projections of the particle with spherical, ellipsoidal (thinner along the z-dimension), and particle-shaped masks, respectively. The fifth column displays the 3D projections of the enhanced and IsoNet missing-wedge-corrected particle. The sixth and seventh columns present the final 3D map and the flexibly fitted model, respectively. (B) Circular schematic representation of a plasmid particle. The outer rim is color-coded to match the corresponding 3D model. Arrowheads indicate the transcriptional direction of bound RNAPs, and circles denote apical sites. Inner circular sectors represent individual plectonemes, with colors indicating writhe density (blue to red scale, −0.3 to 0.1). (C) Resolution assessment of the final 3D map using Fourier shell correlation (FSC). Two criteria are shown: FSC between two half-maps reconstructed from even and odd frames (evaluated at 0.143) and FSC between the final 3D map and the fitted model (evaluated at 0.5). (D) Zoomed-in views of the final 3D density map from panel A, displayed at two contour levels. (E) Superimposition of the high-contour level map from panel D onto its fitted model.

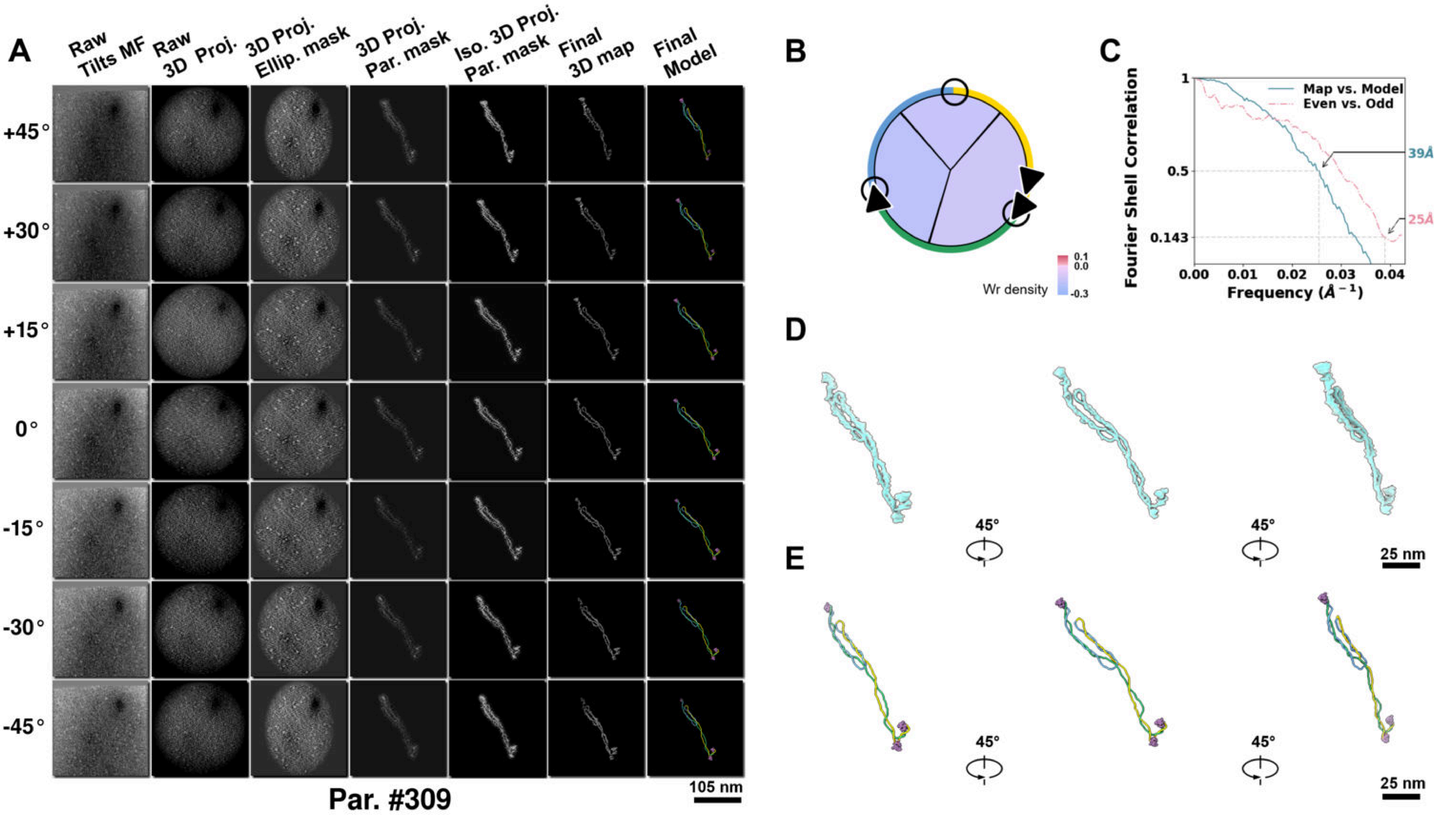

Supplementary Particle Figure 309. Cryo-ET 3D reconstruction of an individual Tan.-TEC particle.
(A) 3D reconstruction of the plasmid particle (index no. 309). The first column shows seven representative tilt images from +45° to -45° in step of 15°. The second, third, and fourth columns show 3D projections of the particle with spherical, ellipsoidal (thinner along the z-dimension), and particle-shaped masks, respectively. The fifth column displays the 3D projections of the enhanced and IsoNet missing-wedge-corrected particle. The sixth and seventh columns present the final 3D map and the flexibly fitted model, respectively. (B) Circular schematic representation of a plasmid particle. The outer rim is color-coded to match the corresponding 3D model. Arrowheads indicate the transcriptional direction of bound RNAPs, and circles denote apical sites. Inner circular sectors represent individual plectonemes, with colors indicating writhe density (blue to red scale, −0.3 to 0.1). (C) Resolution assessment of the final 3D map using Fourier shell correlation (FSC). Two criteria are shown: FSC between two half-maps reconstructed from even and odd frames (evaluated at 0.143) and FSC between the final 3D map and the fitted model (evaluated at 0.5). (D) Zoomed-in views of the final 3D density map from panel A, displayed at two contour levels. (E) Superimposition of the high-contour level map from panel D onto its fitted model.

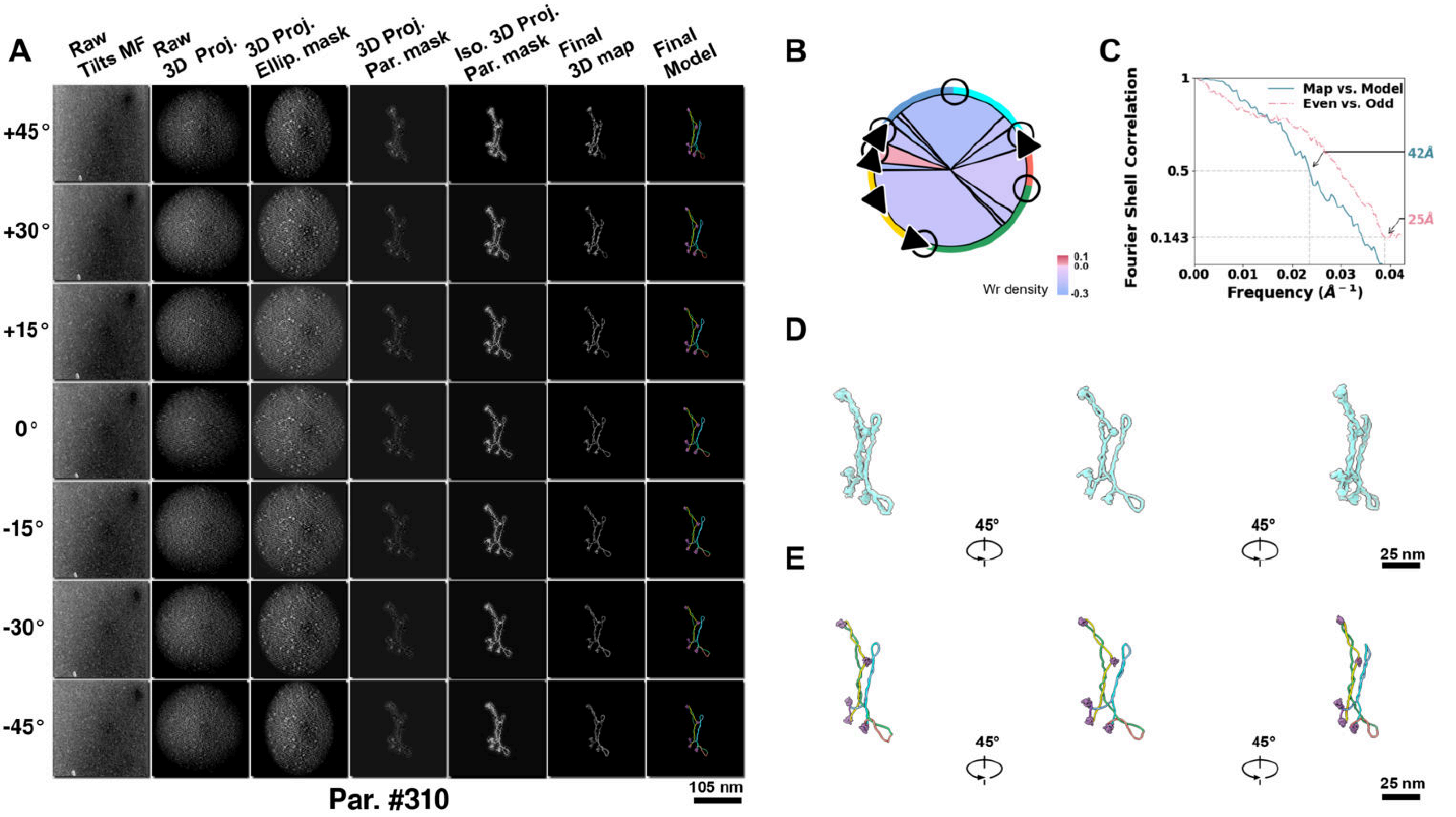

Supplementary Particle Figure 310. Cryo-ET 3D reconstruction of an individual Tan.-TEC particle.
(A) 3D reconstruction of the plasmid particle (index no. 310). The first column shows seven representative tilt images from +45° to -45° in step of 15°. The second, third, and fourth columns show 3D projections of the particle with spherical, ellipsoidal (thinner along the z-dimension), and particle-shaped masks, respectively. The fifth column displays the 3D projections of the enhanced and IsoNet missing-wedge-corrected particle. The sixth and seventh columns present the final 3D map and the flexibly fitted model, respectively. (B) Circular schematic representation of a plasmid particle. The outer rim is color-coded to match the corresponding 3D model. Arrowheads indicate the transcriptional direction of bound RNAPs, and circles denote apical sites. Inner circular sectors represent individual plectonemes, with colors indicating writhe density (blue to red scale, −0.3 to 0.1). (C) Resolution assessment of the final 3D map using Fourier shell correlation (FSC). Two criteria are shown: FSC between two half-maps reconstructed from even and odd frames (evaluated at 0.143) and FSC between the final 3D map and the fitted model (evaluated at 0.5). (D) Zoomed-in views of the final 3D density map from panel A, displayed at two contour levels. (E) Superimposition of the high-contour level map from panel D onto its fitted model.

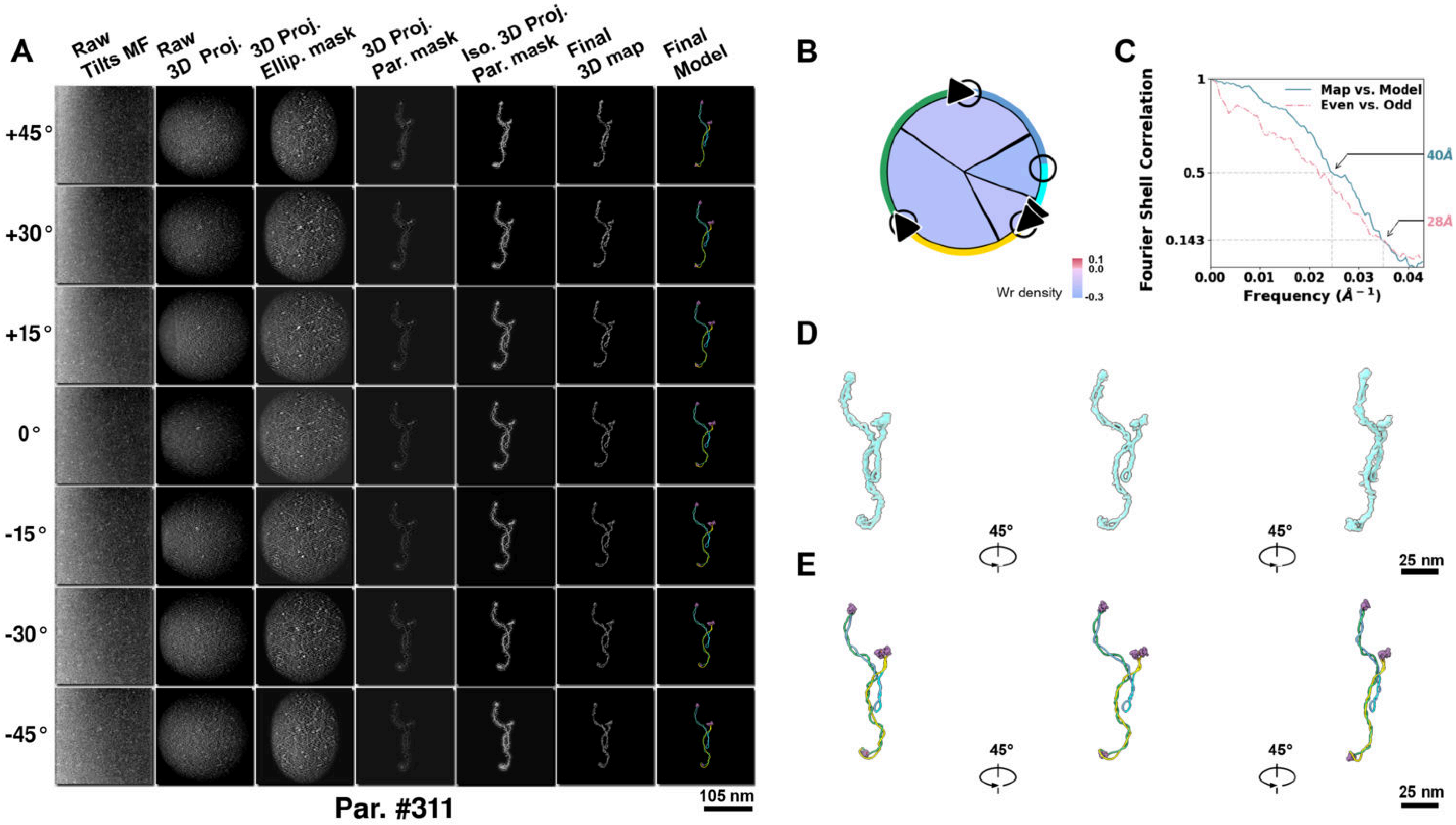

Supplementary Particle Figure 311. Cryo-ET 3D reconstruction of an individual Tan.-TEC particle.
(A) 3D reconstruction of the plasmid particle (index no. 311). The first column shows seven representative tilt images from +45° to -45° in step of 15°. The second, third, and fourth columns show 3D projections of the particle with spherical, ellipsoidal (thinner along the z-dimension), and particle-shaped masks, respectively. The fifth column displays the 3D projections of the enhanced and IsoNet missing-wedge-corrected particle. The sixth and seventh columns present the final 3D map and the flexibly fitted model, respectively. (B) Circular schematic representation of a plasmid particle. The outer rim is color-coded to match the corresponding 3D model. Arrowheads indicate the transcriptional direction of bound RNAPs, and circles denote apical sites. Inner circular sectors represent individual plectonemes, with colors indicating writhe density (blue to red scale, −0.3 to 0.1). (C) Resolution assessment of the final 3D map using Fourier shell correlation (FSC). Two criteria are shown: FSC between two half-maps reconstructed from even and odd frames (evaluated at 0.143) and FSC between the final 3D map and the fitted model (evaluated at 0.5). (D) Zoomed-in views of the final 3D density map from panel A, displayed at two contour levels. (E) Superimposition of the high-contour level map from panel D onto its fitted model.

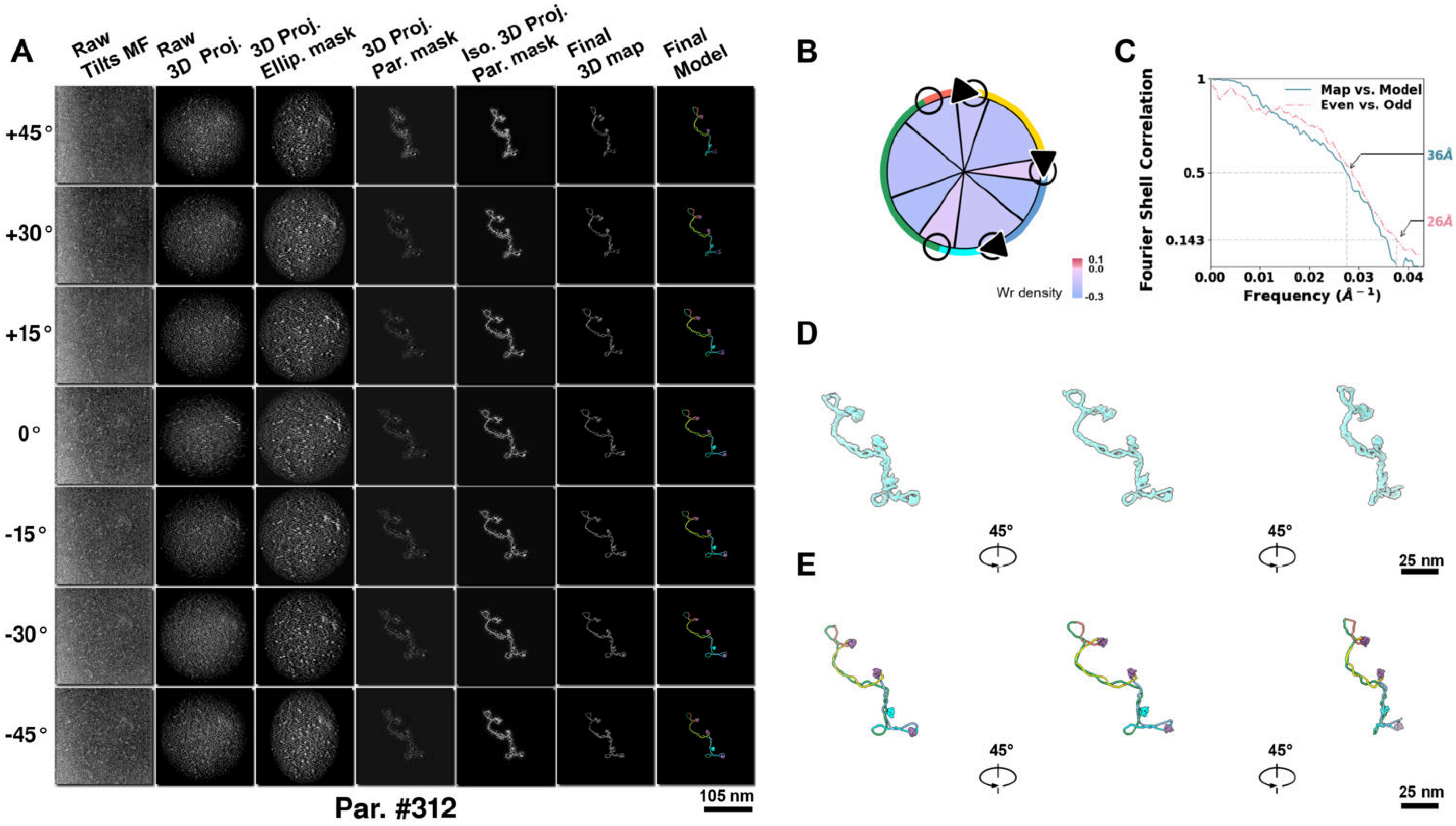

Supplementary Particle Figure 312. Cryo-ET 3D reconstruction of an individual Tan.-TEC particle.
(A) 3D reconstruction of the plasmid particle (index no. 312). The first column shows seven representative tilt images from +45° to -45° in step of 15°. The second, third, and fourth columns show 3D projections of the particle with spherical, ellipsoidal (thinner along the z-dimension), and particle-shaped masks, respectively. The fifth column displays the 3D projections of the enhanced and IsoNet missing-wedge-corrected particle. The sixth and seventh columns present the final 3D map and the flexibly fitted model, respectively. (B) Circular schematic representation of a plasmid particle. The outer rim is color-coded to match the corresponding 3D model. Arrowheads indicate the transcriptional direction of bound RNAPs, and circles denote apical sites. Inner circular sectors represent individual plectonemes, with colors indicating writhe density (blue to red scale, −0.3 to 0.1). (C) Resolution assessment of the final 3D map using Fourier shell correlation (FSC). Two criteria are shown: FSC between two half-maps reconstructed from even and odd frames (evaluated at 0.143) and FSC between the final 3D map and the fitted model (evaluated at 0.5). (D) Zoomed-in views of the final 3D density map from panel A, displayed at two contour levels. (E) Superimposition of the high-contour level map from panel D onto its fitted model.

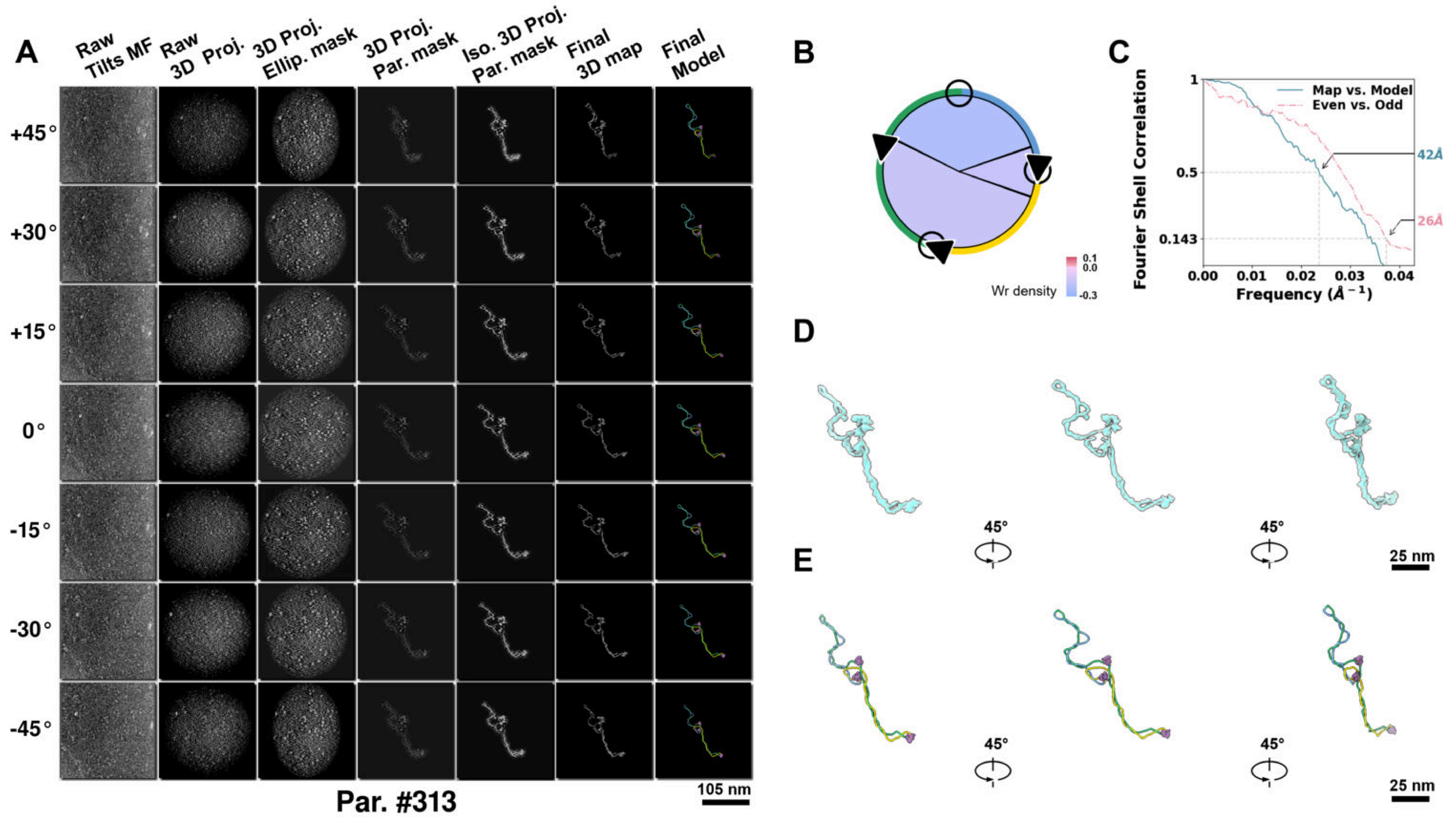

Supplementary Particle Figure 313. Cryo-ET 3D reconstruction of an individual Tan.-TEC particle.
(A) 3D reconstruction of the plasmid particle (index no. 313). The first column shows seven representative tilt images from +45° to -45° in step of 15°. The second, third, and fourth columns show 3D projections of the particle with spherical, ellipsoidal (thinner along the z-dimension), and particle-shaped masks, respectively. The fifth column displays the 3D projections of the enhanced and IsoNet missing-wedge-corrected particle. The sixth and seventh columns present the final 3D map and the flexibly fitted model, respectively. (B) Circular schematic representation of a plasmid particle. The outer rim is color-coded to match the corresponding 3D model. Arrowheads indicate the transcriptional direction of bound RNAPs, and circles denote apical sites. Inner circular sectors represent individual plectonemes, with colors indicating writhe density (blue to red scale, −0.3 to 0.1). (C) Resolution assessment of the final 3D map using Fourier shell correlation (FSC). Two criteria are shown: FSC between two half-maps reconstructed from even and odd frames (evaluated at 0.143) and FSC between the final 3D map and the fitted model (evaluated at 0.5). (D) Zoomed-in views of the final 3D density map from panel A, displayed at two contour levels. (E) Superimposition of the high-contour level map from panel D onto its fitted model.

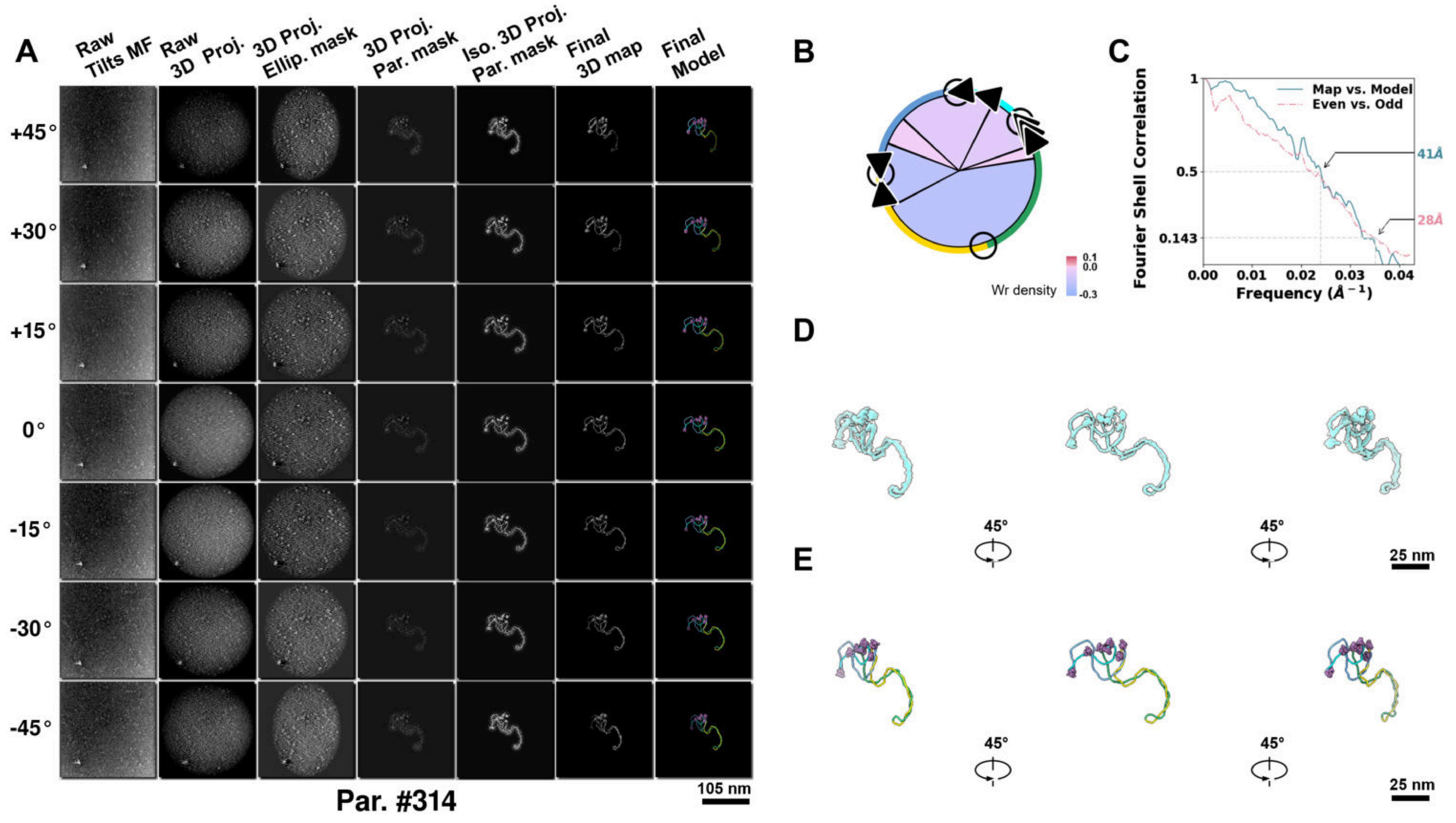

Supplementary Particle Figure 314. Cryo-ET 3D reconstruction of an individual Tan.-TEC particle.
(A) 3D reconstruction of the plasmid particle (index no. 314). The first column shows seven representative tilt images from +45° to -45° in step of 15°. The second, third, and fourth columns show 3D projections of the particle with spherical, ellipsoidal (thinner along the z-dimension), and particle-shaped masks, respectively. The fifth column displays the 3D projections of the enhanced and IsoNet missing-wedge-corrected particle. The sixth and seventh columns present the final 3D map and the flexibly fitted model, respectively. (B) Circular schematic representation of a plasmid particle. The outer rim is color-coded to match the corresponding 3D model. Arrowheads indicate the transcriptional direction of bound RNAPs, and circles denote apical sites. Inner circular sectors represent individual plectonemes, with colors indicating writhe density (blue to red scale, −0.3 to 0.1). (C) Resolution assessment of the final 3D map using Fourier shell correlation (FSC). Two criteria are shown: FSC between two half-maps reconstructed from even and odd frames (evaluated at 0.143) and FSC between the final 3D map and the fitted model (evaluated at 0.5). (D) Zoomed-in views of the final 3D density map from panel A, displayed at two contour levels. (E) Superimposition of the high-contour level map from panel D onto its fitted model.

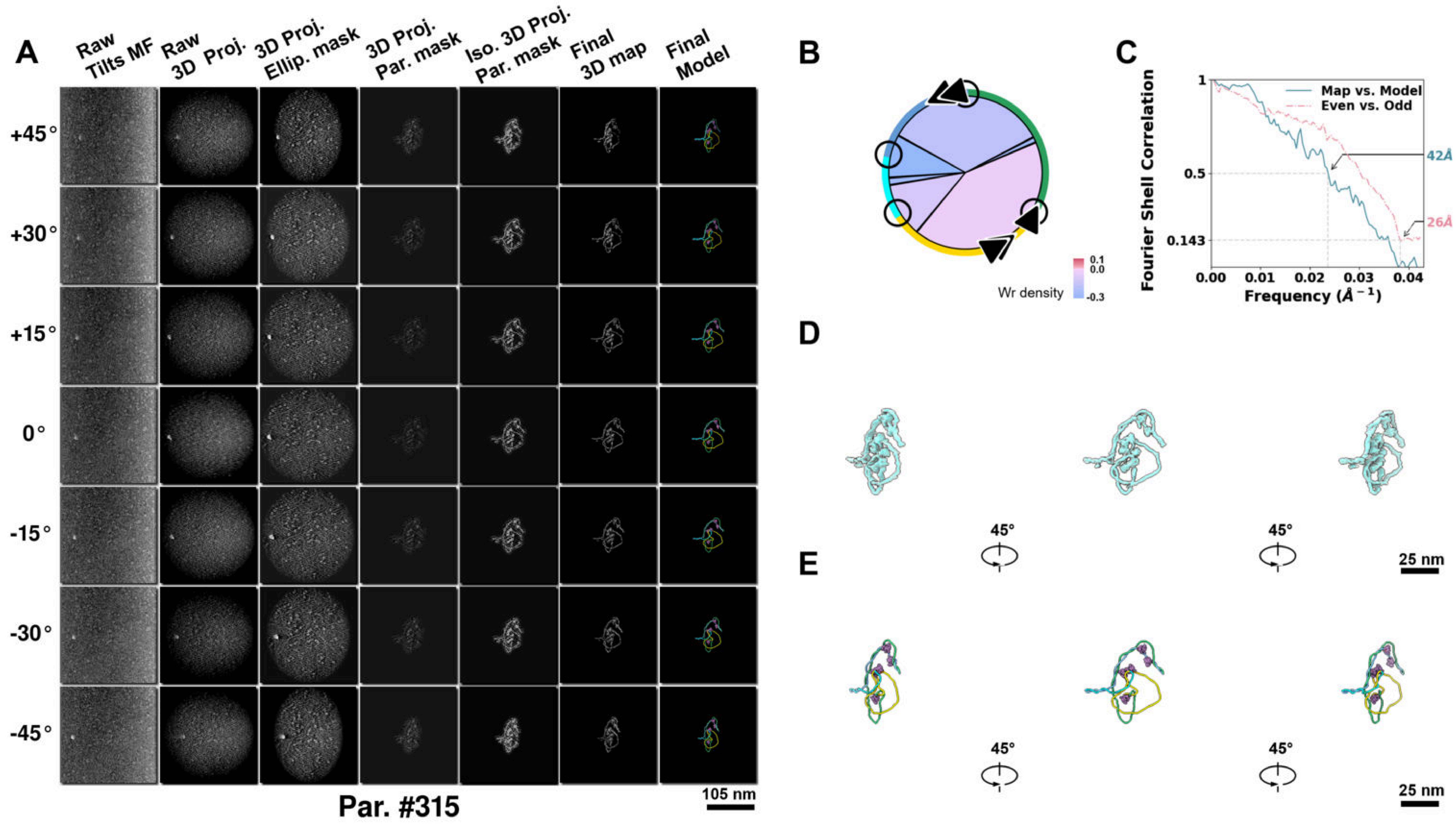

Supplementary Particle Figure 315. Cryo-ET 3D reconstruction of an individual Tan.-TEC particle.
(A) 3D reconstruction of the plasmid particle (index no. 315). The first column shows seven representative tilt images from +45° to -45° in step of 15°. The second, third, and fourth columns show 3D projections of the particle with spherical, ellipsoidal (thinner along the z-dimension), and particle-shaped masks, respectively. The fifth column displays the 3D projections of the enhanced and IsoNet missing-wedge-corrected particle. The sixth and seventh columns present the final 3D map and the flexibly fitted model, respectively. (B) Circular schematic representation of a plasmid particle. The outer rim is color-coded to match the corresponding 3D model. Arrowheads indicate the transcriptional direction of bound RNAPs, and circles denote apical sites. Inner circular sectors represent individual plectonemes, with colors indicating writhe density (blue to red scale, −0.3 to 0.1). (C) Resolution assessment of the final 3D map using Fourier shell correlation (FSC). Two criteria are shown: FSC between two half-maps reconstructed from even and odd frames (evaluated at 0.143) and FSC between the final 3D map and the fitted model (evaluated at 0.5). (D) Zoomed-in views of the final 3D density map from panel A, displayed at two contour levels. (E) Superimposition of the high-contour level map from panel D onto its fitted model.

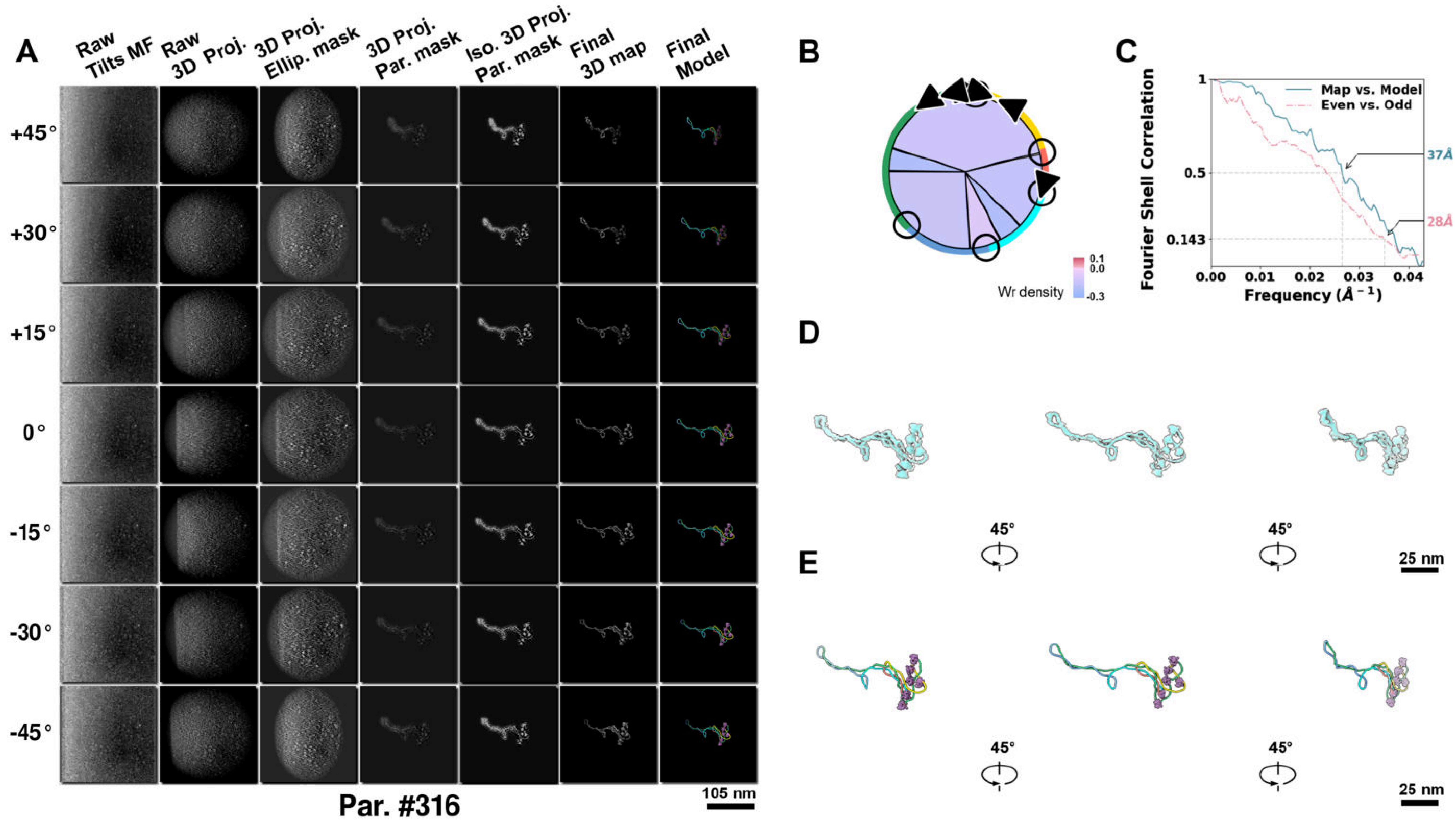

Supplementary Particle Figure 316. Cryo-ET 3D reconstruction of an individual Tan.-TEC particle.
(A) 3D reconstruction of the plasmid particle (index no. 316). The first column shows seven representative tilt images from +45° to -45° in step of 15°. The second, third, and fourth columns show 3D projections of the particle with spherical, ellipsoidal (thinner along the z-dimension), and particle-shaped masks, respectively. The fifth column displays the 3D projections of the enhanced and IsoNet missing-wedge-corrected particle. The sixth and seventh columns present the final 3D map and the flexibly fitted model, respectively. (B) Circular schematic representation of a plasmid particle. The outer rim is color-coded to match the corresponding 3D model. Arrowheads indicate the transcriptional direction of bound RNAPs, and circles denote apical sites. Inner circular sectors represent individual plectonemes, with colors indicating writhe density (blue to red scale, −0.3 to 0.1). (C) Resolution assessment of the final 3D map using Fourier shell correlation (FSC). Two criteria are shown: FSC between two half-maps reconstructed from even and odd frames (evaluated at 0.143) and FSC between the final 3D map and the fitted model (evaluated at 0.5). (D) Zoomed-in views of the final 3D density map from panel A, displayed at two contour levels. (E) Superimposition of the high-contour level map from panel D onto its fitted model.

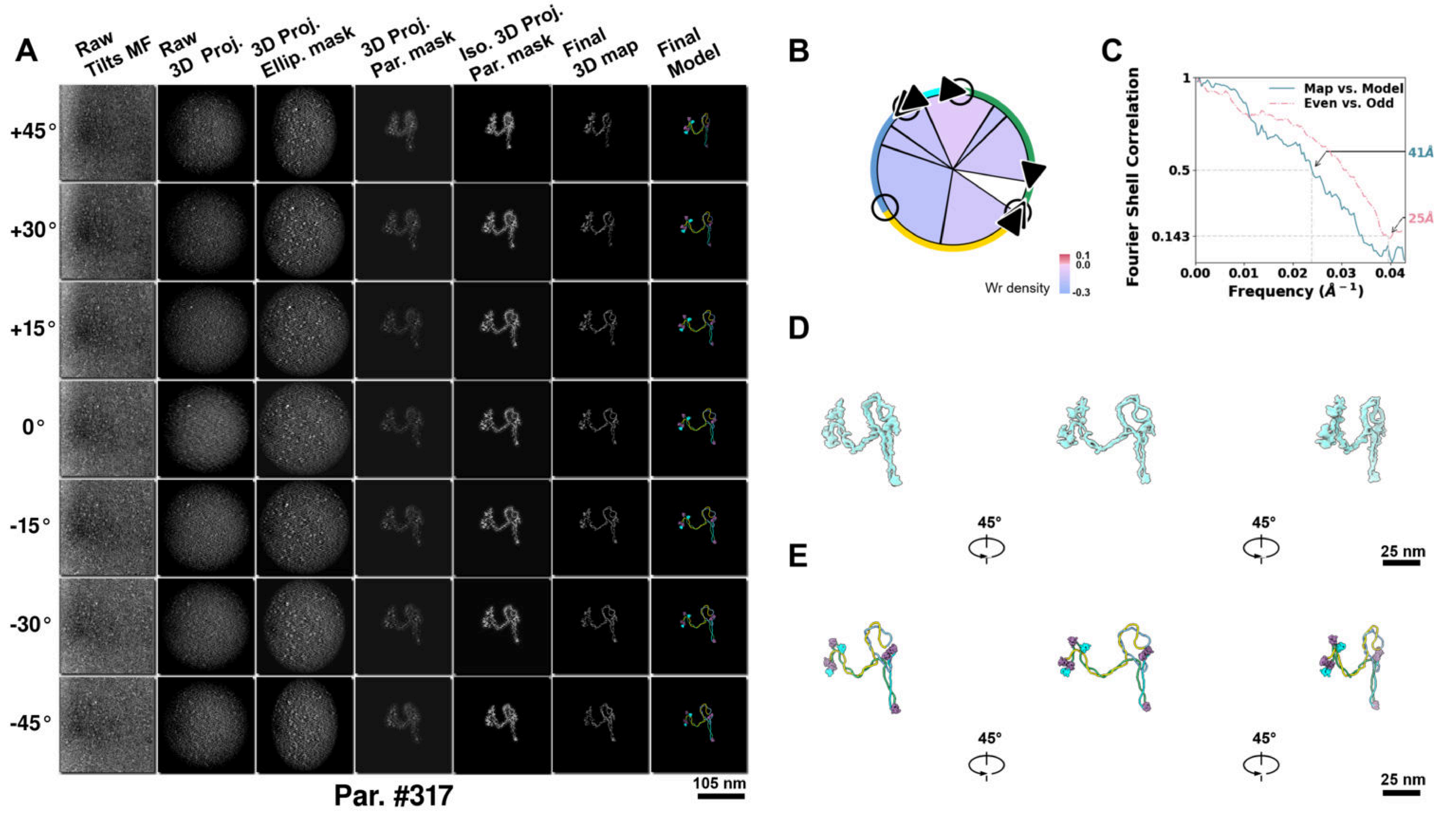

Supplementary Particle Figure 317. Cryo-ET 3D reconstruction of an individual Tan.-TEC particle.
(A) 3D reconstruction of the plasmid particle (index no. 317). The first column shows seven representative tilt images from +45° to -45° in step of 15°. The second, third, and fourth columns show 3D projections of the particle with spherical, ellipsoidal (thinner along the z-dimension), and particle-shaped masks, respectively. The fifth column displays the 3D projections of the enhanced and IsoNet missing-wedge-corrected particle. The sixth and seventh columns present the final 3D map and the flexibly fitted model, respectively. (B) Circular schematic representation of a plasmid particle. The outer rim is color-coded to match the corresponding 3D model. Arrowheads indicate the transcriptional direction of bound RNAPs, and circles denote apical sites. Inner circular sectors represent individual plectonemes, with colors indicating writhe density (blue to red scale, −0.3 to 0.1). (C) Resolution assessment of the final 3D map using Fourier shell correlation (FSC). Two criteria are shown: FSC between two half-maps reconstructed from even and odd frames (evaluated at 0.143) and FSC between the final 3D map and the fitted model (evaluated at 0.5). (D) Zoomed-in views of the final 3D density map from panel A, displayed at two contour levels. (E) Superimposition of the high-contour level map from panel D onto its fitted model.

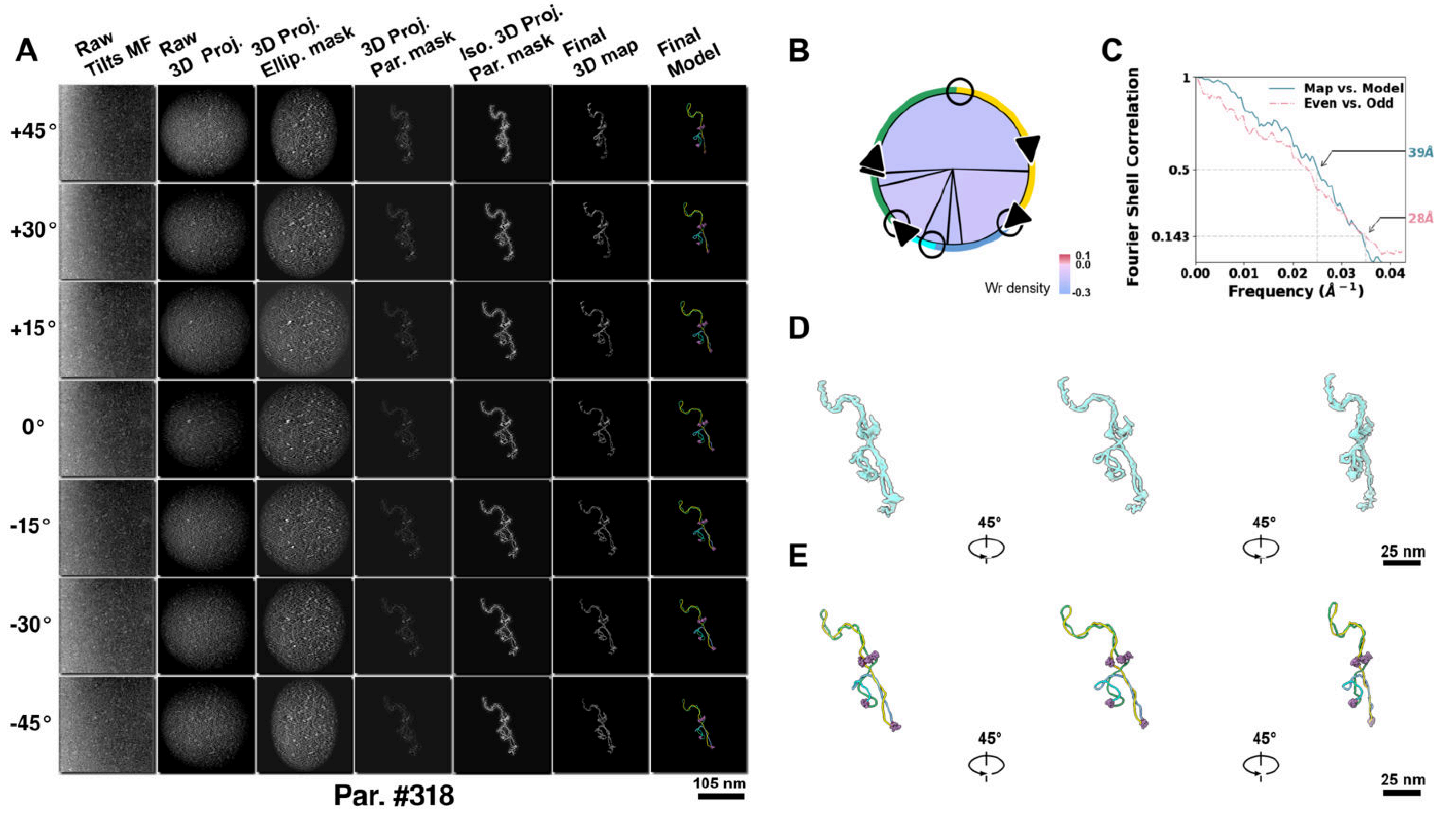

Supplementary Particle Figure 318. Cryo-ET 3D reconstruction of an individual Tan.-TEC particle.
(A) 3D reconstruction of the plasmid particle (index no. 318). The first column shows seven representative tilt images from +45° to -45° in step of 15°. The second, third, and fourth columns show 3D projections of the particle with spherical, ellipsoidal (thinner along the z-dimension), and particle-shaped masks, respectively. The fifth column displays the 3D projections of the enhanced and IsoNet missing-wedge-corrected particle. The sixth and seventh columns present the final 3D map and the flexibly fitted model, respectively. (B) Circular schematic representation of a plasmid particle. The outer rim is color-coded to match the corresponding 3D model. Arrowheads indicate the transcriptional direction of bound RNAPs, and circles denote apical sites. Inner circular sectors represent individual plectonemes, with colors indicating writhe density (blue to red scale, −0.3 to 0.1). (C) Resolution assessment of the final 3D map using Fourier shell correlation (FSC). Two criteria are shown: FSC between two half-maps reconstructed from even and odd frames (evaluated at 0.143) and FSC between the final 3D map and the fitted model (evaluated at 0.5). (D) Zoomed-in views of the final 3D density map from panel A, displayed at two contour levels. (E) Superimposition of the high-contour level map from panel D onto its fitted model.

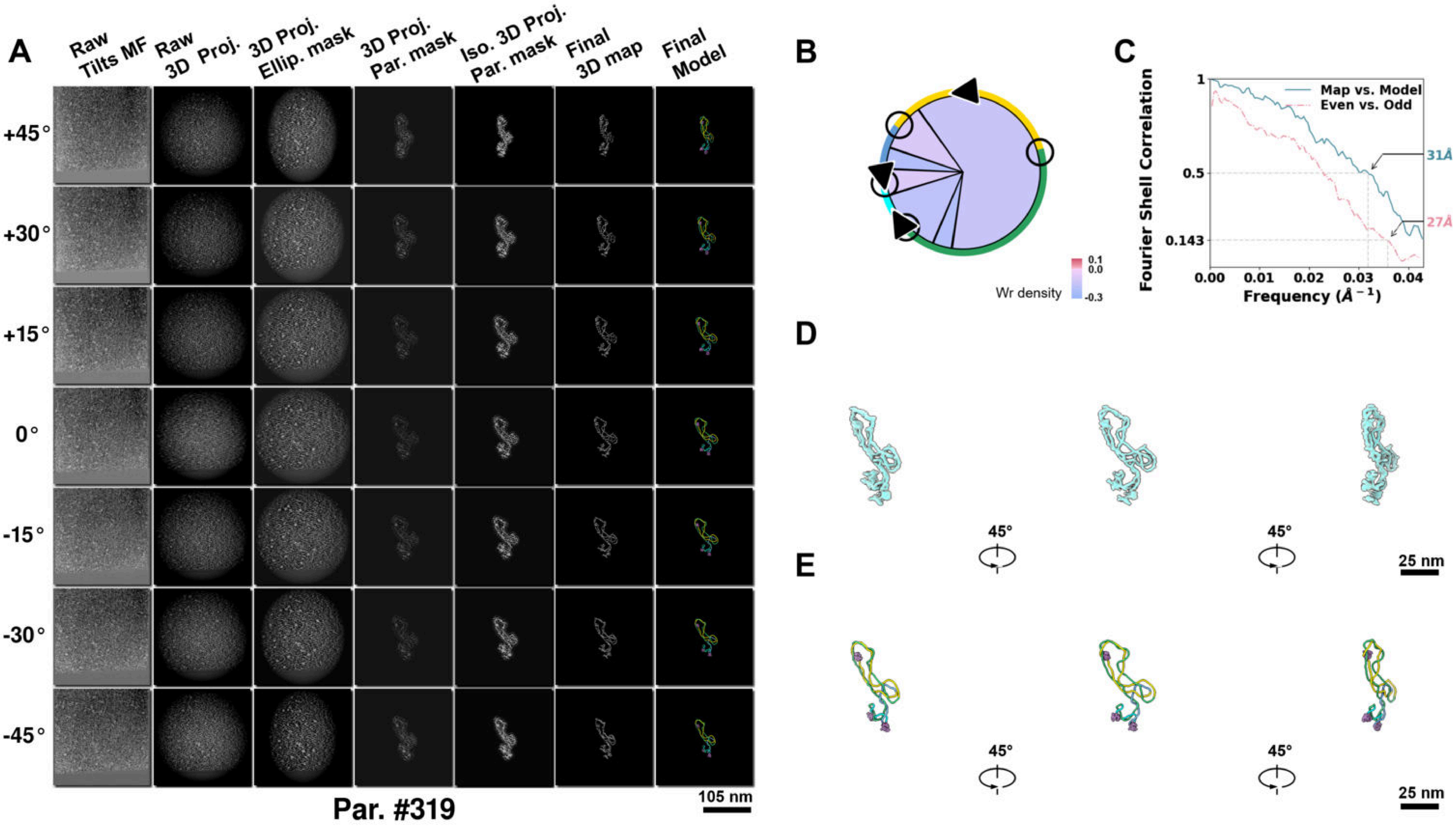

Supplementary Particle Figure 319. Cryo-ET 3D reconstruction of an individual Tan.-TEC particle.
(A) 3D reconstruction of the plasmid particle (index no. 319). The first column shows seven representative tilt images from +45° to -45° in step of 15°. The second, third, and fourth columns show 3D projections of the particle with spherical, ellipsoidal (thinner along the z-dimension), and particle-shaped masks, respectively. The fifth column displays the 3D projections of the enhanced and IsoNet missing-wedge-corrected particle. The sixth and seventh columns present the final 3D map and the flexibly fitted model, respectively. (B) Circular schematic representation of a plasmid particle. The outer rim is color-coded to match the corresponding 3D model. Arrowheads indicate the transcriptional direction of bound RNAPs, and circles denote apical sites. Inner circular sectors represent individual plectonemes, with colors indicating writhe density (blue to red scale, −0.3 to 0.1). (C) Resolution assessment of the final 3D map using Fourier shell correlation (FSC). Two criteria are shown: FSC between two half-maps reconstructed from even and odd frames (evaluated at 0.143) and FSC between the final 3D map and the fitted model (evaluated at 0.5). (D) Zoomed-in views of the final 3D density map from panel A, displayed at two contour levels. (E) Superimposition of the high-contour level map from panel D onto its fitted model.

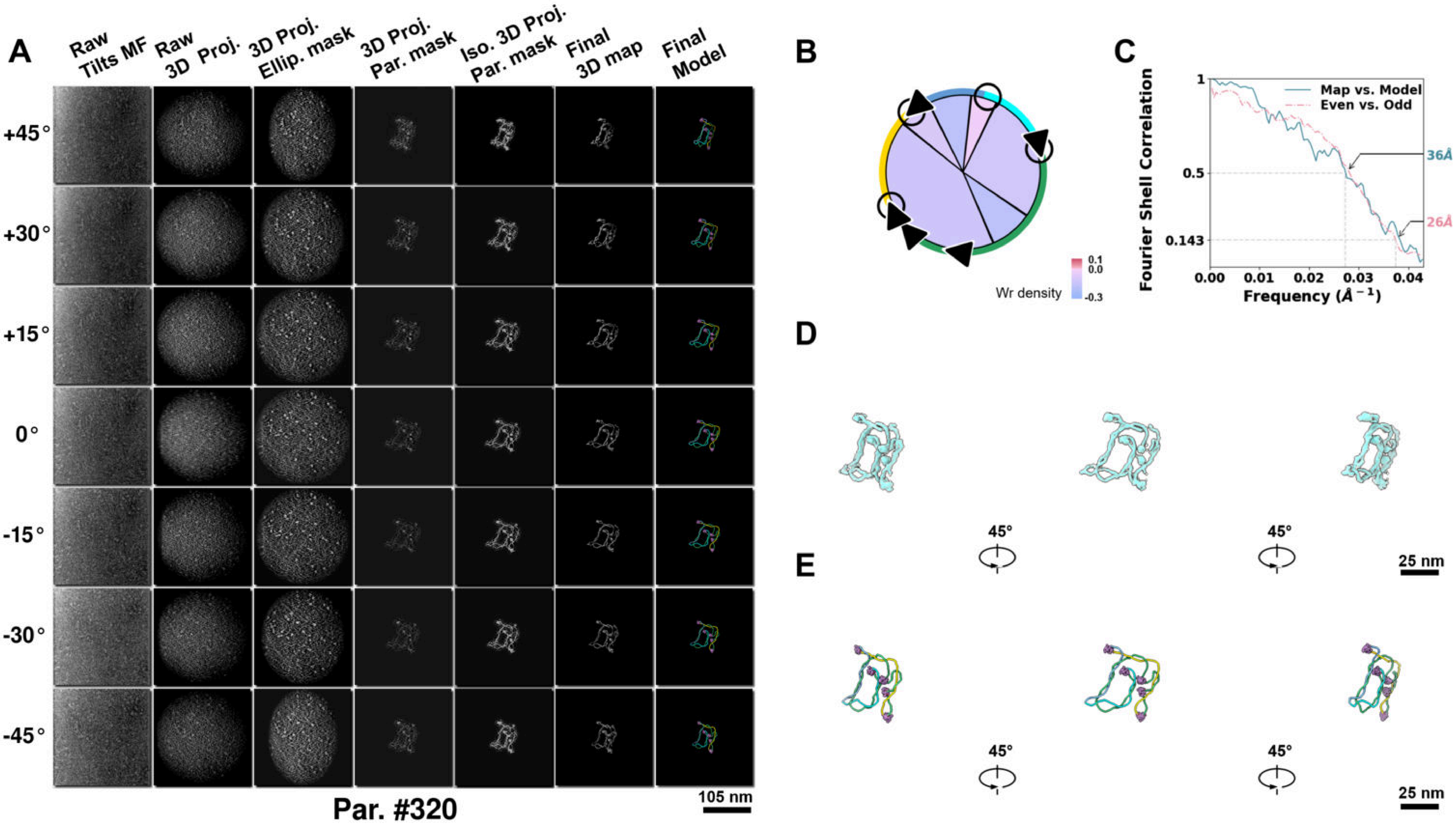

Supplementary Particle Figure 320. Cryo-ET 3D reconstruction of an individual Tan.-TEC particle.
(A) 3D reconstruction of the plasmid particle (index no. 320). The first column shows seven representative tilt images from +45° to -45° in step of 15°. The second, third, and fourth columns show 3D projections of the particle with spherical, ellipsoidal (thinner along the z-dimension), and particle-shaped masks, respectively. The fifth column displays the 3D projections of the enhanced and IsoNet missing-wedge-corrected particle. The sixth and seventh columns present the final 3D map and the flexibly fitted model, respectively. (B) Circular schematic representation of a plasmid particle. The outer rim is color-coded to match the corresponding 3D model. Arrowheads indicate the transcriptional direction of bound RNAPs, and circles denote apical sites. Inner circular sectors represent individual plectonemes, with colors indicating writhe density (blue to red scale, −0.3 to 0.1). (C) Resolution assessment of the final 3D map using Fourier shell correlation (FSC). Two criteria are shown: FSC between two half-maps reconstructed from even and odd frames (evaluated at 0.143) and FSC between the final 3D map and the fitted model (evaluated at 0.5). (D) Zoomed-in views of the final 3D density map from panel A, displayed at two contour levels. (E) Superimposition of the high-contour level map from panel D onto its fitted model.

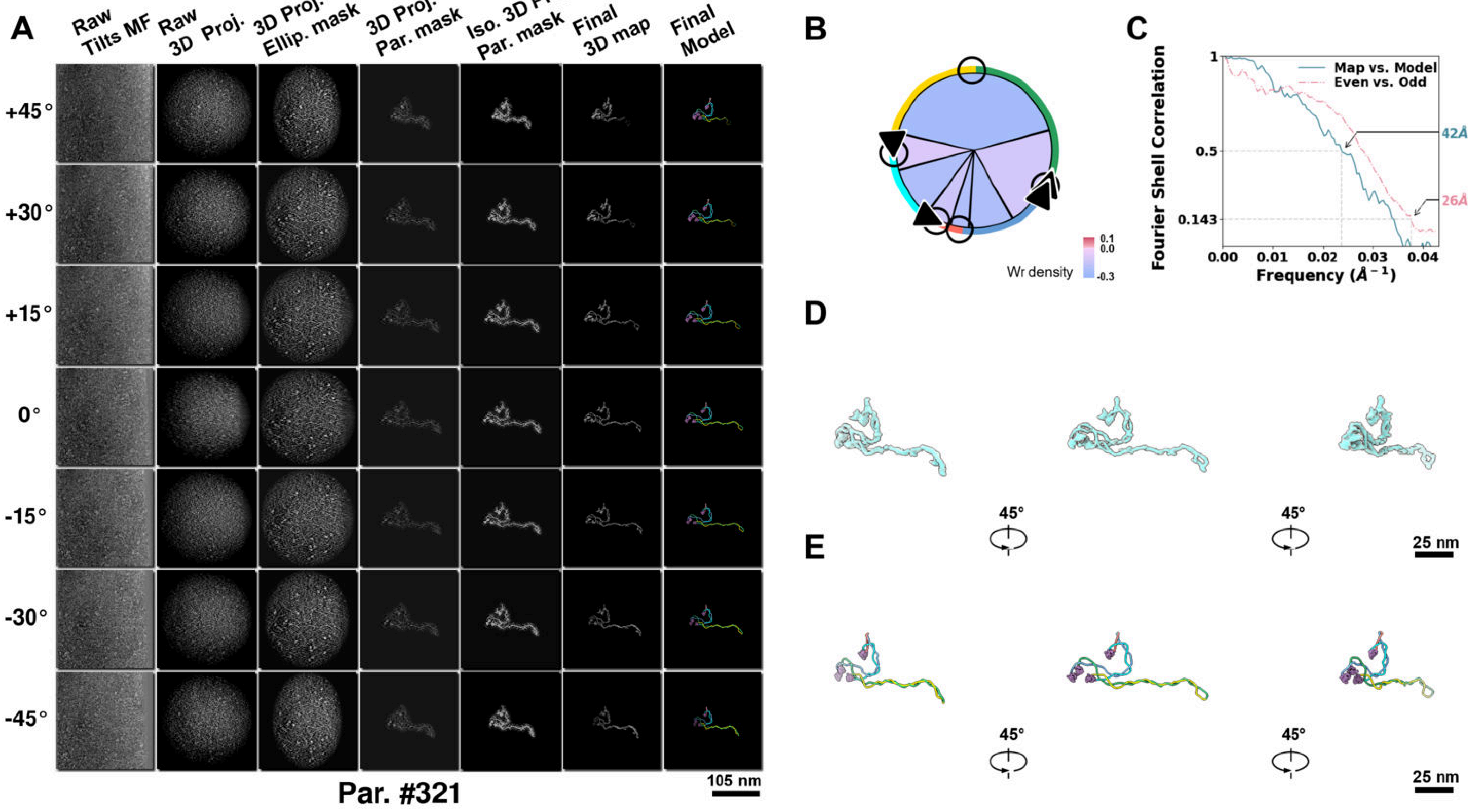

Supplementary Particle Figure 321. Cryo-ET 3D reconstruction of an individual Tan.-TEC particle.
(A) 3D reconstruction of the plasmid particle (index no. 321). The first column shows seven representative tilt images from +45° to -45° in step of 15°. The second, third, and fourth columns show 3D projections of the particle with spherical, ellipsoidal (thinner along the z-dimension), and particle-shaped masks, respectively. The fifth column displays the 3D projections of the enhanced and IsoNet missing-wedge-corrected particle. The sixth and seventh columns present the final 3D map and the flexibly fitted model, respectively. (B) Circular schematic representation of a plasmid particle. The outer rim is color-coded to match the corresponding 3D model. Arrowheads indicate the transcriptional direction of bound RNAPs, and circles denote apical sites. Inner circular sectors represent individual plectonemes, with colors indicating writhe density (blue to red scale, −0.3 to 0.1). (C) Resolution assessment of the final 3D map using Fourier shell correlation (FSC). Two criteria are shown: FSC between two half-maps reconstructed from even and odd frames (evaluated at 0.143) and FSC between the final 3D map and the fitted model (evaluated at 0.5). (D) Zoomed-in views of the final 3D density map from panel A, displayed at two contour levels. (E) Superimposition of the high-contour level map from panel D onto its fitted model.

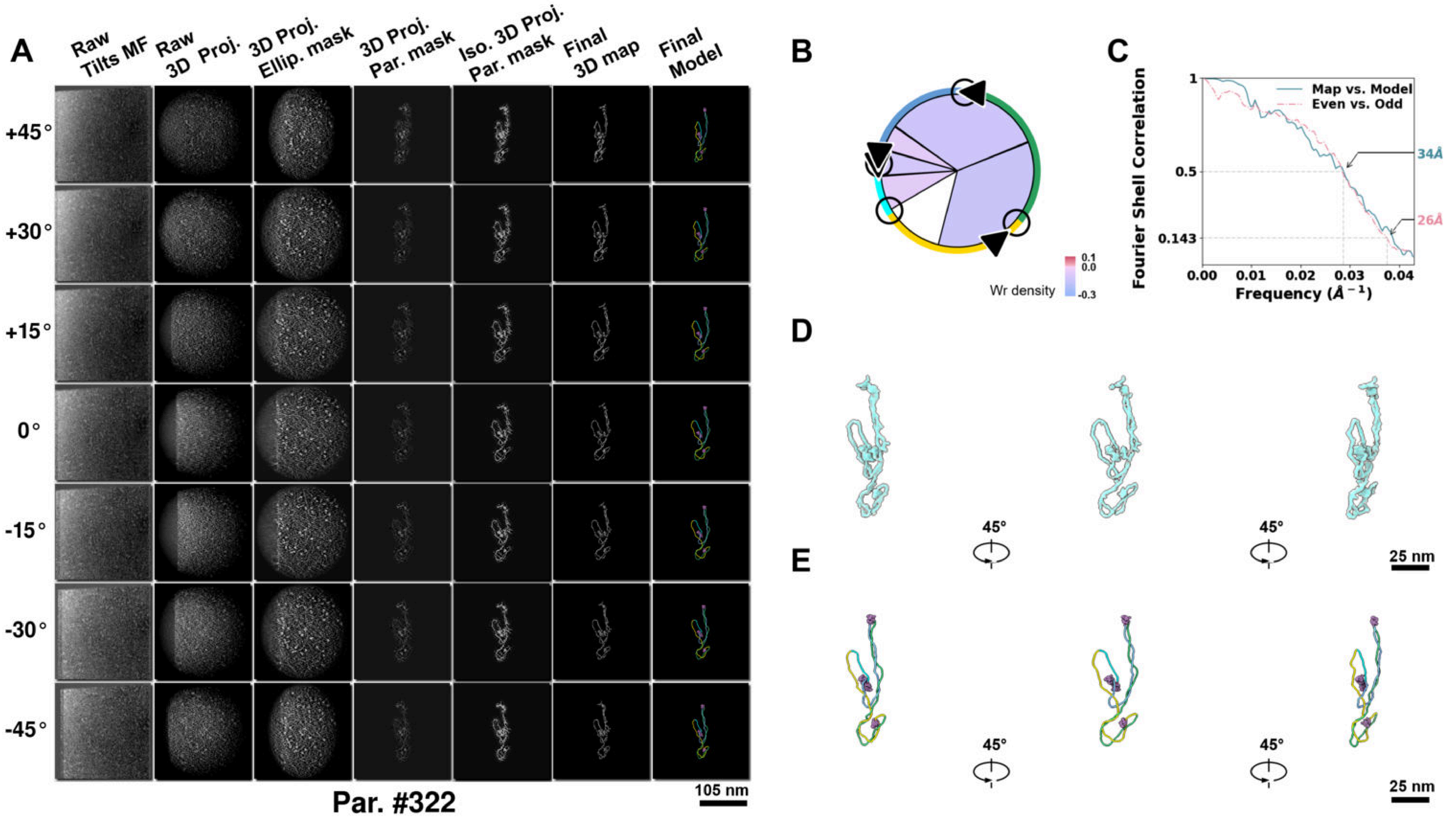

Supplementary Particle Figure 322. Cryo-ET 3D reconstruction of an individual Tan.-TEC particle.
(A) 3D reconstruction of the plasmid particle (index no. 322). The first column shows seven representative tilt images from +45° to -45° in step of 15°. The second, third, and fourth columns show 3D projections of the particle with spherical, ellipsoidal (thinner along the z-dimension), and particle-shaped masks, respectively. The fifth column displays the 3D projections of the enhanced and IsoNet missing-wedge-corrected particle. The sixth and seventh columns present the final 3D map and the flexibly fitted model, respectively. (B) Circular schematic representation of a plasmid particle. The outer rim is color-coded to match the corresponding 3D model. Arrowheads indicate the transcriptional direction of bound RNAPs, and circles denote apical sites. Inner circular sectors represent individual plectonemes, with colors indicating writhe density (blue to red scale, −0.3 to 0.1). (C) Resolution assessment of the final 3D map using Fourier shell correlation (FSC). Two criteria are shown: FSC between two half-maps reconstructed from even and odd frames (evaluated at 0.143) and FSC between the final 3D map and the fitted model (evaluated at 0.5). (D) Zoomed-in views of the final 3D density map from panel A, displayed at two contour levels. (E) Superimposition of the high-contour level map from panel D onto its fitted model.

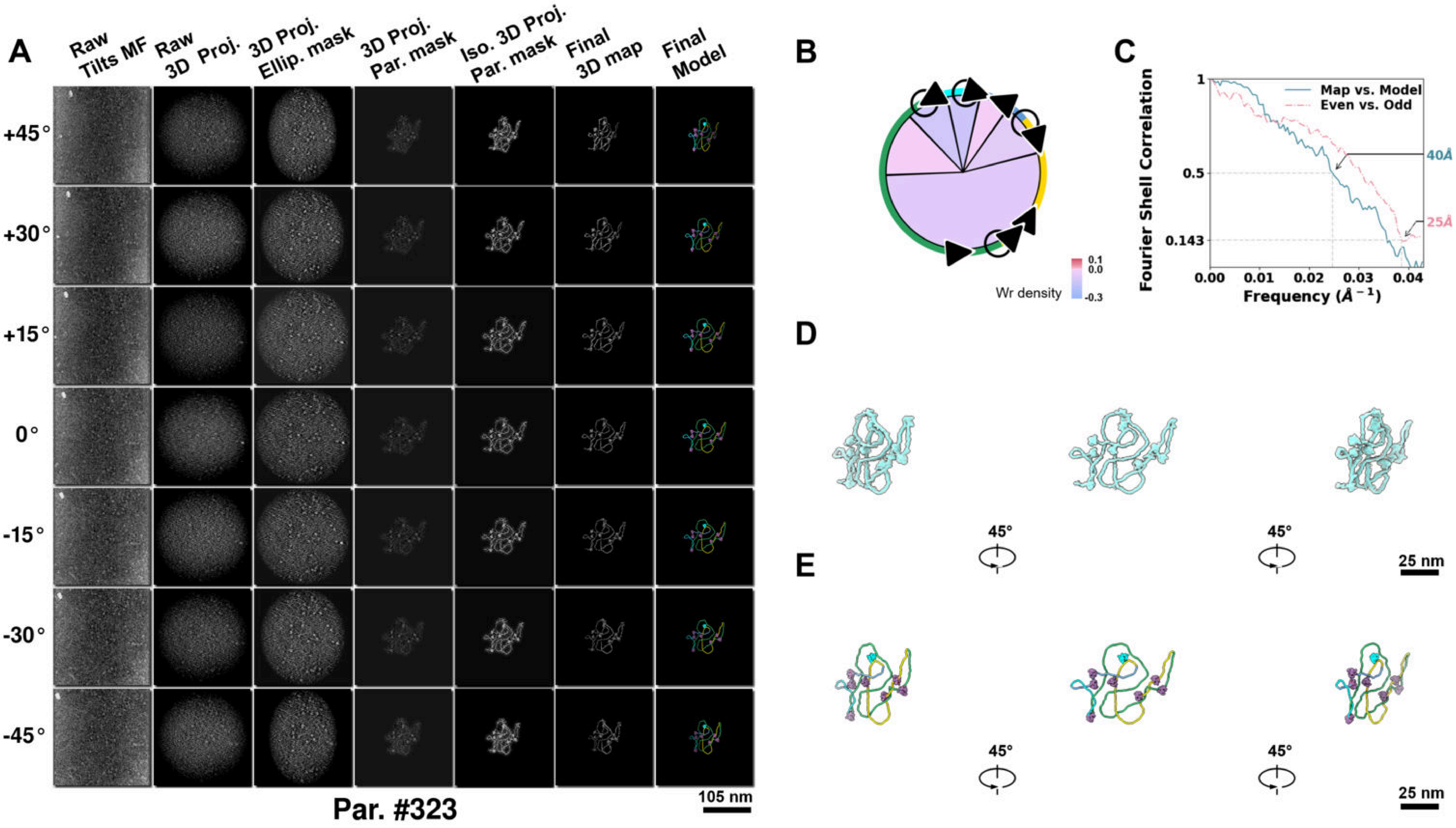

Supplementary Particle Figure 323. Cryo-ET 3D reconstruction of an individual Tan.-TEC particle.
(A) 3D reconstruction of the plasmid particle (index no. 323). The first column shows seven representative tilt images from +45° to -45° in step of 15°. The second, third, and fourth columns show 3D projections of the particle with spherical, ellipsoidal (thinner along the z-dimension), and particle-shaped masks, respectively. The fifth column displays the 3D projections of the enhanced and IsoNet missing-wedge-corrected particle. The sixth and seventh columns present the final 3D map and the flexibly fitted model, respectively. (B) Circular schematic representation of a plasmid particle. The outer rim is color-coded to match the corresponding 3D model. Arrowheads indicate the transcriptional direction of bound RNAPs, and circles denote apical sites. Inner circular sectors represent individual plectonemes, with colors indicating writhe density (blue to red scale, −0.3 to 0.1). (C) Resolution assessment of the final 3D map using Fourier shell correlation (FSC). Two criteria are shown: FSC between two half-maps reconstructed from even and odd frames (evaluated at 0.143) and FSC between the final 3D map and the fitted model (evaluated at 0.5). (D) Zoomed-in views of the final 3D density map from panel A, displayed at two contour levels. (E) Superimposition of the high-contour level map from panel D onto its fitted model.

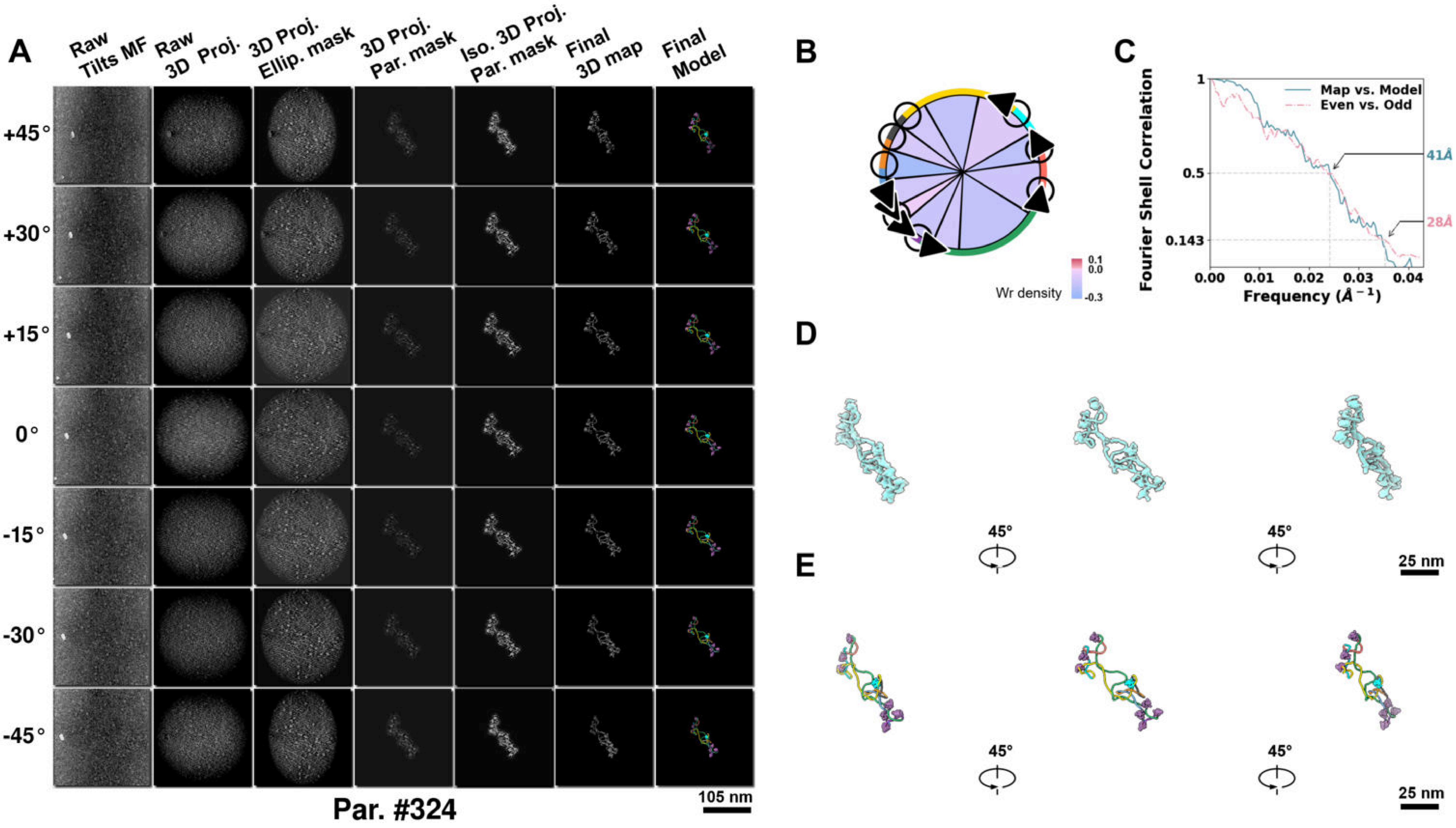

Supplementary Particle Figure 324. Cryo-ET 3D reconstruction of an individual Tan.-TEC particle.
(A) 3D reconstruction of the plasmid particle (index no. 324). The first column shows seven representative tilt images from +45° to -45° in step of 15°. The second, third, and fourth columns show 3D projections of the particle with spherical, ellipsoidal (thinner along the z-dimension), and particle-shaped masks, respectively. The fifth column displays the 3D projections of the enhanced and IsoNet missing-wedge-corrected particle. The sixth and seventh columns present the final 3D map and the flexibly fitted model, respectively. (B) Circular schematic representation of a plasmid particle. The outer rim is color-coded to match the corresponding 3D model. Arrowheads indicate the transcriptional direction of bound RNAPs, and circles denote apical sites. Inner circular sectors represent individual plectonemes, with colors indicating writhe density (blue to red scale, −0.3 to 0.1). (C) Resolution assessment of the final 3D map using Fourier shell correlation (FSC). Two criteria are shown: FSC between two half-maps reconstructed from even and odd frames (evaluated at 0.143) and FSC between the final 3D map and the fitted model (evaluated at 0.5). (D) Zoomed-in views of the final 3D density map from panel A, displayed at two contour levels. (E) Superimposition of the high-contour level map from panel D onto its fitted model.

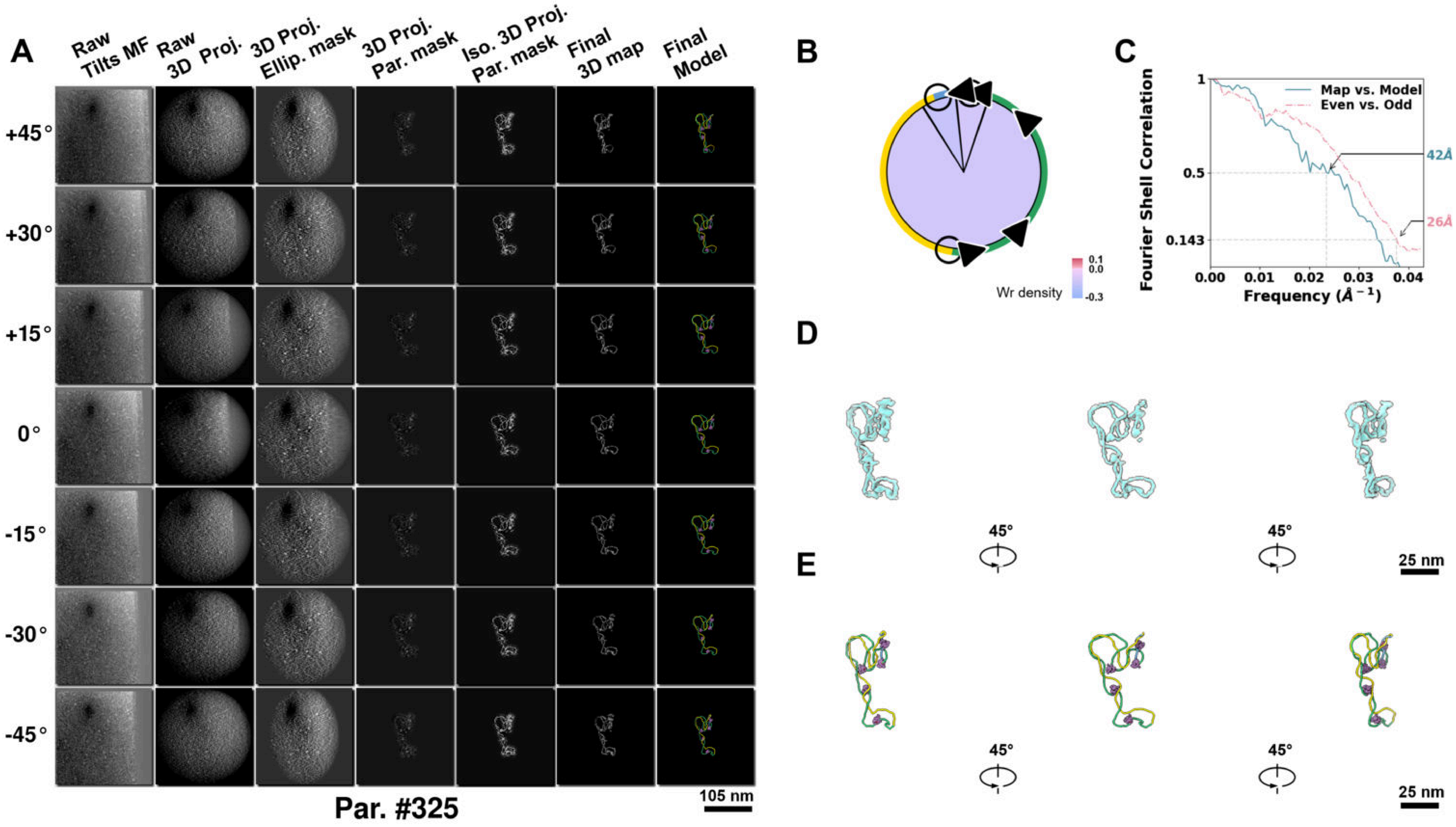

Supplementary Particle Figure 325. Cryo-ET 3D reconstruction of an individual Tan.-TEC particle.
(A) 3D reconstruction of the plasmid particle (index no. 325). The first column shows seven representative tilt images from +45° to -45° in step of 15°. The second, third, and fourth columns show 3D projections of the particle with spherical, ellipsoidal (thinner along the z-dimension), and particle-shaped masks, respectively. The fifth column displays the 3D projections of the enhanced and IsoNet missing-wedge-corrected particle. The sixth and seventh columns present the final 3D map and the flexibly fitted model, respectively. (B) Circular schematic representation of a plasmid particle. The outer rim is color-coded to match the corresponding 3D model. Arrowheads indicate the transcriptional direction of bound RNAPs, and circles denote apical sites. Inner circular sectors represent individual plectonemes, with colors indicating writhe density (blue to red scale, −0.3 to 0.1). (C) Resolution assessment of the final 3D map using Fourier shell correlation (FSC). Two criteria are shown: FSC between two half-maps reconstructed from even and odd frames (evaluated at 0.143) and FSC between the final 3D map and the fitted model (evaluated at 0.5). (D) Zoomed-in views of the final 3D density map from panel A, displayed at two contour levels. (E) Superimposition of the high-contour level map from panel D onto its fitted model.

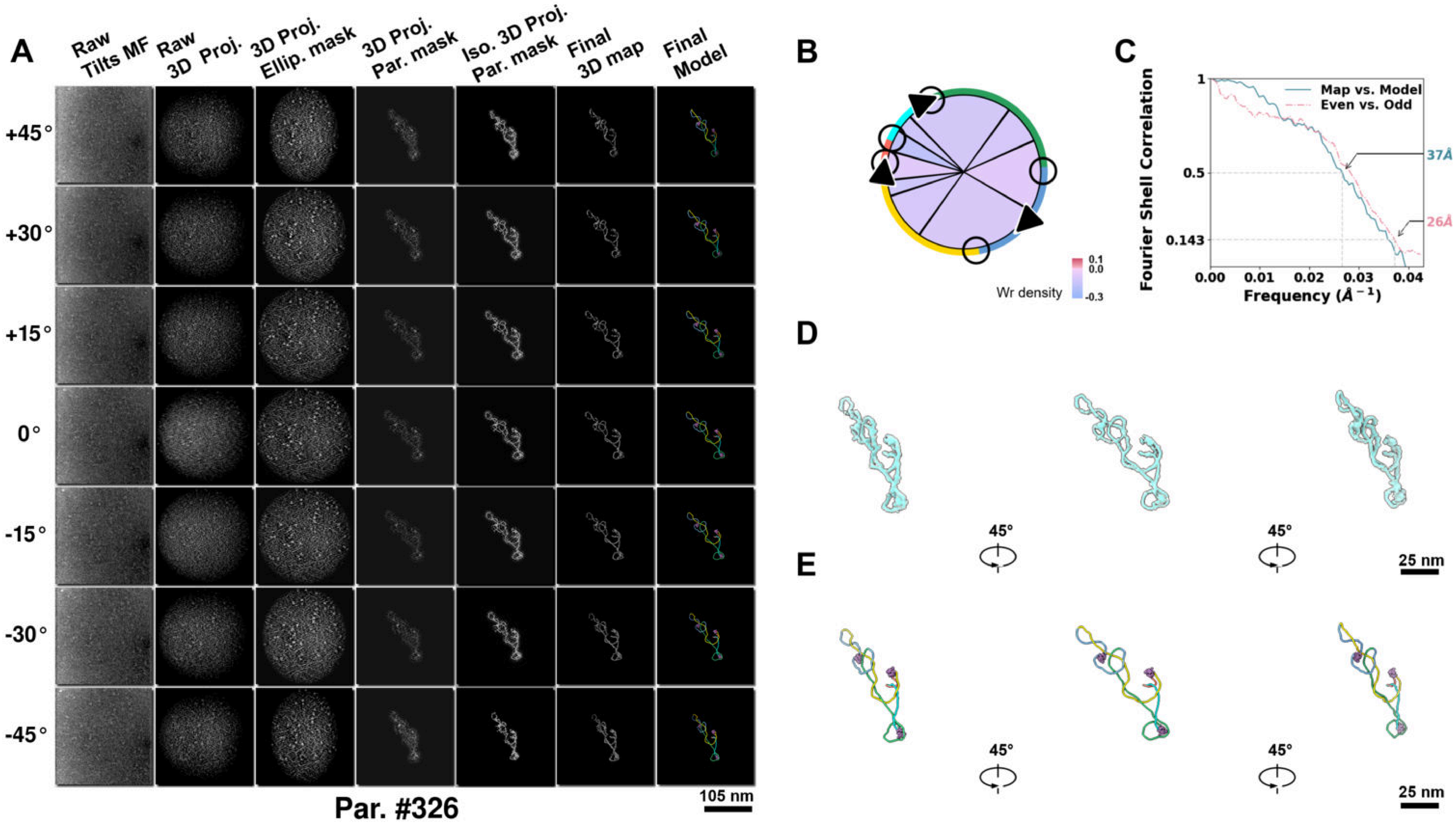

Supplementary Particle Figure 326. Cryo-ET 3D reconstruction of an individual Tan.-TEC particle.
(A) 3D reconstruction of the plasmid particle (index no. 326). The first column shows seven representative tilt images from +45° to -45° in step of 15°. The second, third, and fourth columns show 3D projections of the particle with spherical, ellipsoidal (thinner along the z-dimension), and particle-shaped masks, respectively. The fifth column displays the 3D projections of the enhanced and IsoNet missing-wedge-corrected particle. The sixth and seventh columns present the final 3D map and the flexibly fitted model, respectively. (B) Circular schematic representation of a plasmid particle. The outer rim is color-coded to match the corresponding 3D model. Arrowheads indicate the transcriptional direction of bound RNAPs, and circles denote apical sites. Inner circular sectors represent individual plectonemes, with colors indicating writhe density (blue to red scale, −0.3 to 0.1). (C) Resolution assessment of the final 3D map using Fourier shell correlation (FSC). Two criteria are shown: FSC between two half-maps reconstructed from even and odd frames (evaluated at 0.143) and FSC between the final 3D map and the fitted model (evaluated at 0.5). (D) Zoomed-in views of the final 3D density map from panel A, displayed at two contour levels. (E) Superimposition of the high-contour level map from panel D onto its fitted model.

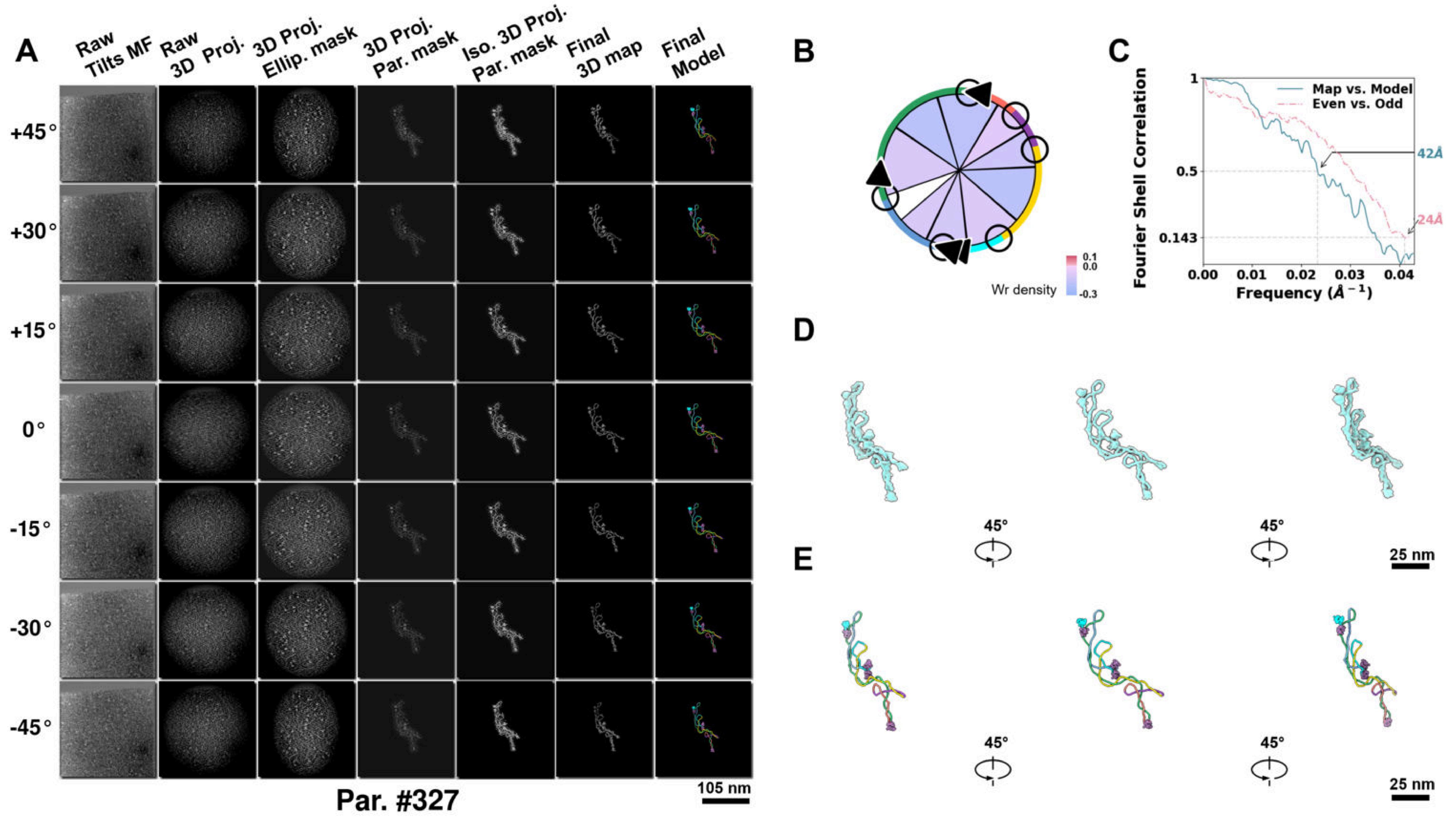

Supplementary Particle Figure 327. Cryo-ET 3D reconstruction of an individual Tan.-TEC particle.
(A) 3D reconstruction of the plasmid particle (index no. 327). The first column shows seven representative tilt images from +45° to -45° in step of 15°. The second, third, and fourth columns show 3D projections of the particle with spherical, ellipsoidal (thinner along the z-dimension), and particle-shaped masks, respectively. The fifth column displays the 3D projections of the enhanced and IsoNet missing-wedge-corrected particle. The sixth and seventh columns present the final 3D map and the flexibly fitted model, respectively. (B) Circular schematic representation of a plasmid particle. The outer rim is color-coded to match the corresponding 3D model. Arrowheads indicate the transcriptional direction of bound RNAPs, and circles denote apical sites. Inner circular sectors represent individual plectonemes, with colors indicating writhe density (blue to red scale, −0.3 to 0.1). (C) Resolution assessment of the final 3D map using Fourier shell correlation (FSC). Two criteria are shown: FSC between two half-maps reconstructed from even and odd frames (evaluated at 0.143) and FSC between the final 3D map and the fitted model (evaluated at 0.5). (D) Zoomed-in views of the final 3D density map from panel A, displayed at two contour levels. (E) Superimposition of the high-contour level map from panel D onto its fitted model.

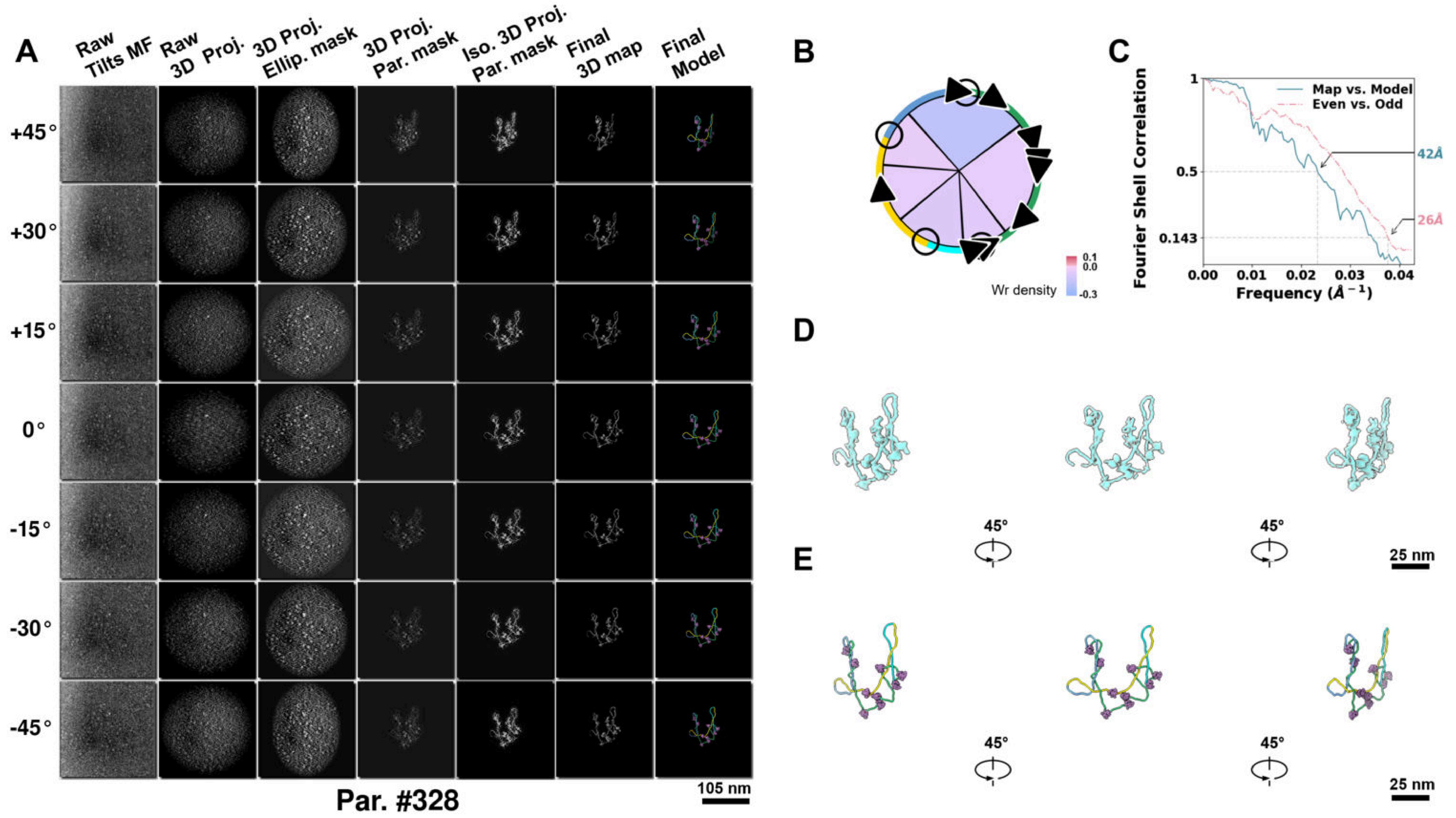

Supplementary Particle Figure 328. Cryo-ET 3D reconstruction of an individual Tan.-TEC particle.
(A) 3D reconstruction of the plasmid particle (index no. 328). The first column shows seven representative tilt images from +45° to -45° in step of 15°. The second, third, and fourth columns show 3D projections of the particle with spherical, ellipsoidal (thinner along the z-dimension), and particle-shaped masks, respectively. The fifth column displays the 3D projections of the enhanced and IsoNet missing-wedge-corrected particle. The sixth and seventh columns present the final 3D map and the flexibly fitted model, respectively. (B) Circular schematic representation of a plasmid particle. The outer rim is color-coded to match the corresponding 3D model. Arrowheads indicate the transcriptional direction of bound RNAPs, and circles denote apical sites. Inner circular sectors represent individual plectonemes, with colors indicating writhe density (blue to red scale, −0.3 to 0.1). (C) Resolution assessment of the final 3D map using Fourier shell correlation (FSC). Two criteria are shown: FSC between two half-maps reconstructed from even and odd frames (evaluated at 0.143) and FSC between the final 3D map and the fitted model (evaluated at 0.5). (D) Zoomed-in views of the final 3D density map from panel A, displayed at two contour levels. (E) Superimposition of the high-contour level map from panel D onto its fitted model.

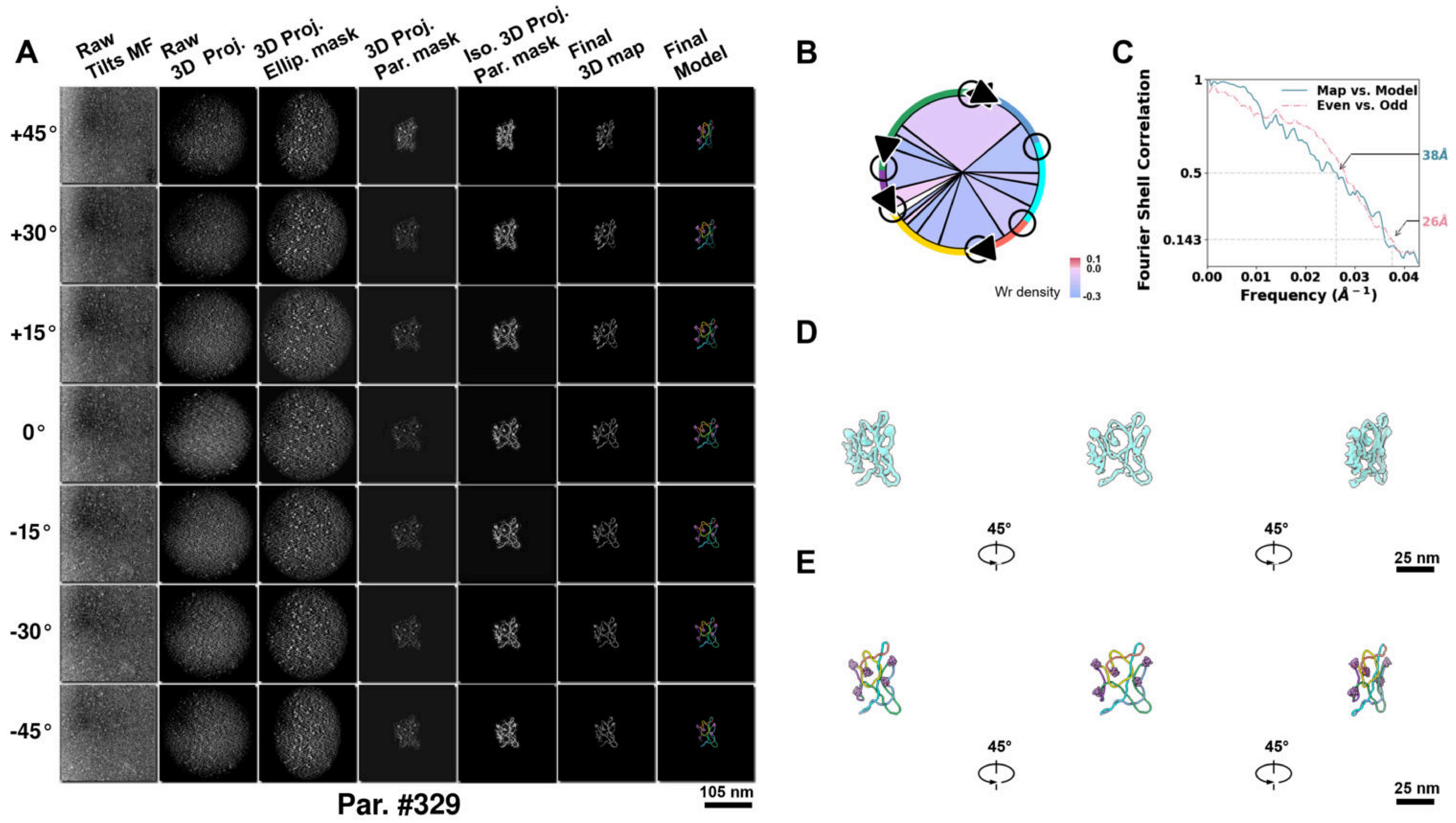

Supplementary Particle Figure 329. Cryo-ET 3D reconstruction of an individual Tan.-TEC particle.
(A) 3D reconstruction of the plasmid particle (index no. 329). The first column shows seven representative tilt images from +45° to -45° in step of 15°. The second, third, and fourth columns show 3D projections of the particle with spherical, ellipsoidal (thinner along the z-dimension), and particle-shaped masks, respectively. The fifth column displays the 3D projections of the enhanced and IsoNet missing-wedge-corrected particle. The sixth and seventh columns present the final 3D map and the flexibly fitted model, respectively. (B) Circular schematic representation of a plasmid particle. The outer rim is color-coded to match the corresponding 3D model. Arrowheads indicate the transcriptional direction of bound RNAPs, and circles denote apical sites. Inner circular sectors represent individual plectonemes, with colors indicating writhe density (blue to red scale, −0.3 to 0.1). (C) Resolution assessment of the final 3D map using Fourier shell correlation (FSC). Two criteria are shown: FSC between two half-maps reconstructed from even and odd frames (evaluated at 0.143) and FSC between the final 3D map and the fitted model (evaluated at 0.5). (D) Zoomed-in views of the final 3D density map from panel A, displayed at two contour levels. (E) Superimposition of the high-contour level map from panel D onto its fitted model.

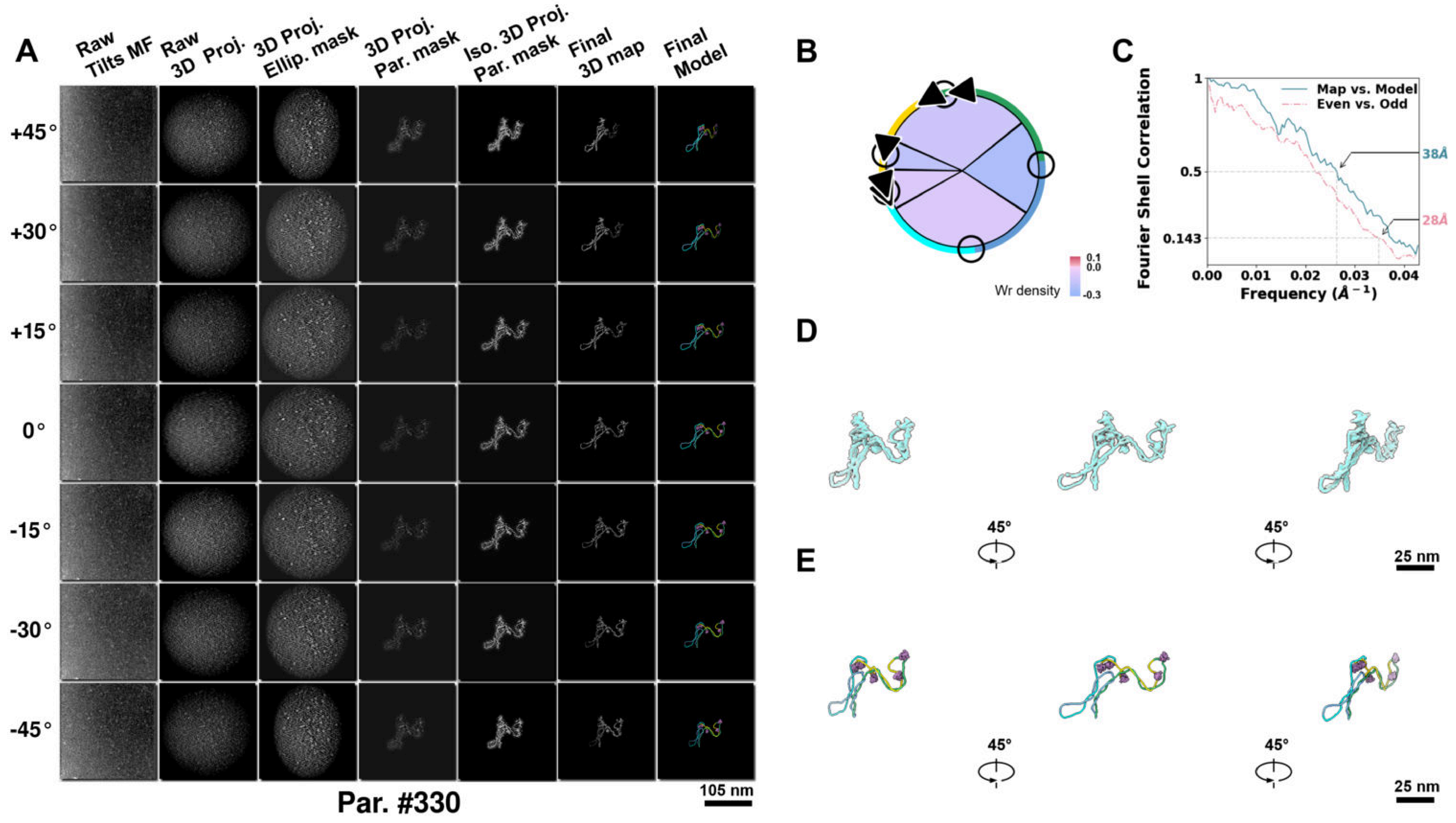

Supplementary Particle Figure 330. Cryo-ET 3D reconstruction of an individual Tan.-TEC particle.
(A) 3D reconstruction of the plasmid particle (index no. 330). The first column shows seven representative tilt images from +45° to -45° in step of 15°. The second, third, and fourth columns show 3D projections of the particle with spherical, ellipsoidal (thinner along the z-dimension), and particle-shaped masks, respectively. The fifth column displays the 3D projections of the enhanced and IsoNet missing-wedge-corrected particle. The sixth and seventh columns present the final 3D map and the flexibly fitted model, respectively. (B) Circular schematic representation of a plasmid particle. The outer rim is color-coded to match the corresponding 3D model. Arrowheads indicate the transcriptional direction of bound RNAPs, and circles denote apical sites. Inner circular sectors represent individual plectonemes, with colors indicating writhe density (blue to red scale, −0.3 to 0.1). (C) Resolution assessment of the final 3D map using Fourier shell correlation (FSC). Two criteria are shown: FSC between two half-maps reconstructed from even and odd frames (evaluated at 0.143) and FSC between the final 3D map and the fitted model (evaluated at 0.5). (D) Zoomed-in views of the final 3D density map from panel A, displayed at two contour levels. (E) Superimposition of the high-contour level map from panel D onto its fitted model.

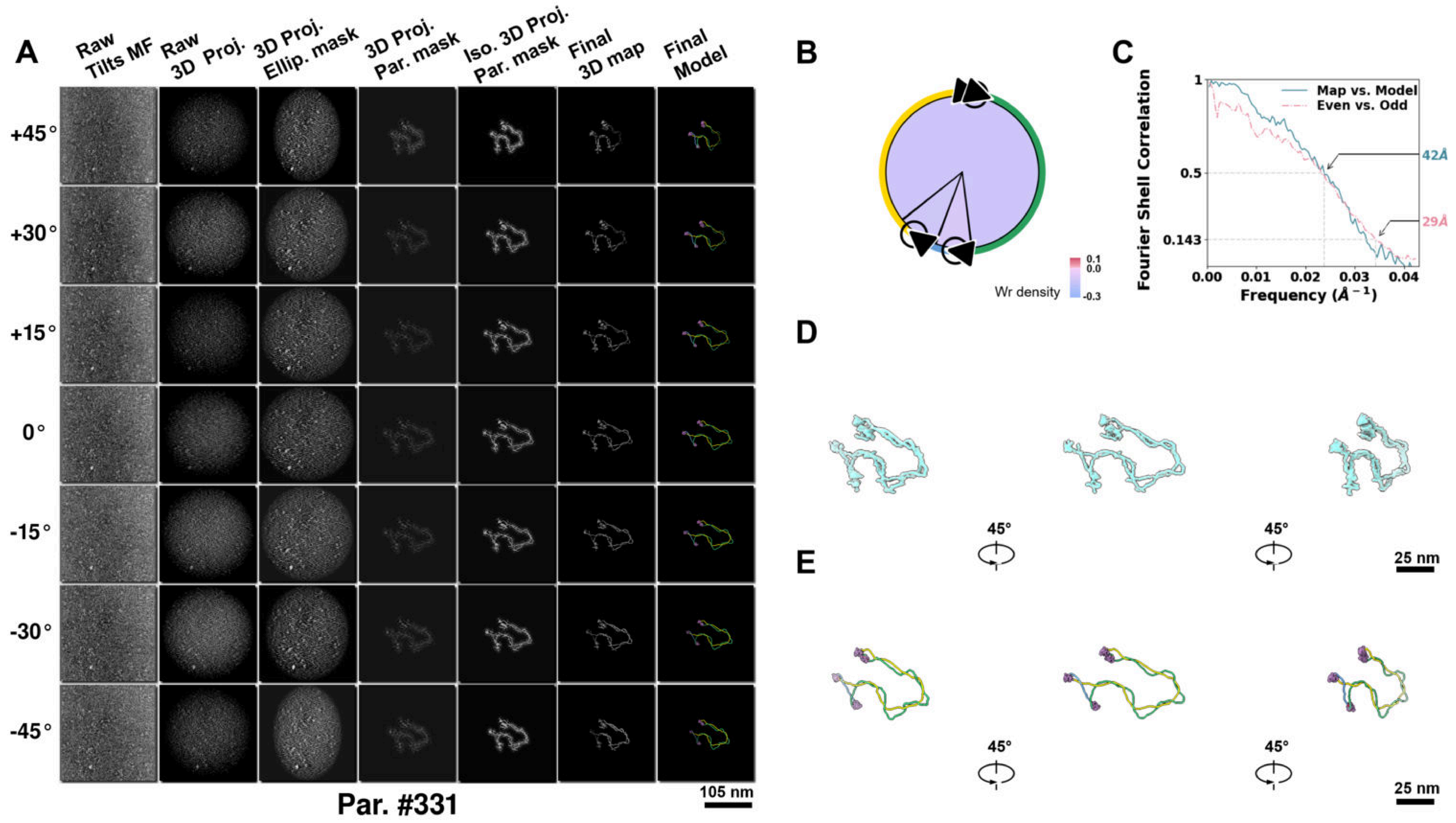

Supplementary Particle Figure 331. Cryo-ET 3D reconstruction of an individual Tan.-TEC particle.
(A) 3D reconstruction of the plasmid particle (index no. 331). The first column shows seven representative tilt images from +45° to -45° in step of 15°. The second, third, and fourth columns show 3D projections of the particle with spherical, ellipsoidal (thinner along the z-dimension), and particle-shaped masks, respectively. The fifth column displays the 3D projections of the enhanced and IsoNet missing-wedge-corrected particle. The sixth and seventh columns present the final 3D map and the flexibly fitted model, respectively. (B) Circular schematic representation of a plasmid particle. The outer rim is color-coded to match the corresponding 3D model. Arrowheads indicate the transcriptional direction of bound RNAPs, and circles denote apical sites. Inner circular sectors represent individual plectonemes, with colors indicating writhe density (blue to red scale, −0.3 to 0.1). (C) Resolution assessment of the final 3D map using Fourier shell correlation (FSC). Two criteria are shown: FSC between two half-maps reconstructed from even and odd frames (evaluated at 0.143) and FSC between the final 3D map and the fitted model (evaluated at 0.5). (D) Zoomed-in views of the final 3D density map from panel A, displayed at two contour levels. (E) Superimposition of the high-contour level map from panel D onto its fitted model.
